# Antiviral Profiling and Cellular Activation of Carbobicyclic Nucleoside Analogues

**DOI:** 10.1021/acs.jmedchem.5c02584

**Published:** 2026-02-20

**Authors:** Stephan Scheeff, Joan Marie Javillo Baguio, Benny Zhibin Liang, Josefina Xeque Amada, Kin Pong Tao, Steven De Jonghe, Leentje Persoons, Tiffany Hoi-Yee Chow, Carmen Ka Man Tse, Roy Yukang Wu, Xinzhou Xu, Zhong Zuo, Peter Pak-Hang Cheung, Renee Wan Yi Chan, Billy Wai-Lung Ng

**Affiliations:** † School of Pharmacy, Faculty of Medicine, 26451 The Chinese University of Hong Kong, Shatin, Hong Kong; ‡ Department of Paediatrics, Faculty of Medicine; S.H. Ho Research Centre for Infectious Diseases, 26451 The Chinese University of Hong Kong, Shatin, Hong Kong; § Department of Chemical Pathology, Faculty of Medicine, 26451 The Chinese University of Hong Kong, Shatin, Hong Kong; ∥ Li Ka Shing Institute of Health Sciences, Faculty of Medicine, 26451 The Chinese University of Hong Kong, Shatin, Hong Kong; ⊥ KU Leuven,Rega Institute for Medical Research, Department of Microbiology, Immunology and Transplantation, Molecular, Structural and Translational Virology Research Group, Herestraat 49, box 1049, 3000 Leuven, Belgium; # KU Leuven,Rega Institute for Medical Research, Department of Microbiology, Immunology and Transplantation, Molecular Genetics and Therapeutics in Virology and Oncology Research Group, Herestraat 49, box 1048, 3000 Leuven, Belgium; ∇ CUHK−Hub of Obstetric and Paediatric Excellence; 26451 The Chinese University of Hong Kong, Shatin, Hong Kong; ○ Gerald Choa Neuroscience Institute, 26451 The Chinese University of Hong Kong, Shatin, Hong Kong

## Abstract

Nucleoside analogues are important antiviral and anticancer agents. In this study, we investigated a new class of nucleoside analogues built on a synthetically accessible carbobicyclic scaffold designed as a conformational mimic of ribose. Antiviral screening of our library revealed pan-antiviral activity against a range of viruses, including HCV, HSV, and influenza. Structure–activity relationship (SAR) studies highlighted the critical role of the carbocyclic scaffold. The uracil analogue **2a** inhibited influenza A virus replication through direct disruption of the viral polymerase, as confirmed by a minigenome assay and further supported by *in silico* modeling. Importantly, metabolism studies demonstrated that congested C5′–OH is readily phosphorylated without the need for prodrug formulations. The resulting triphosphate metabolites are not substrates of human DNA/RNA polymerases, a primary mechanism of nucleoside drug toxicity. Supported by comprehensive synthetic schemes, we present a carbobicyclic scaffold with altered architecture as a promising chemotype for developing novel nucleoside therapeutics.

## Graphical abstract

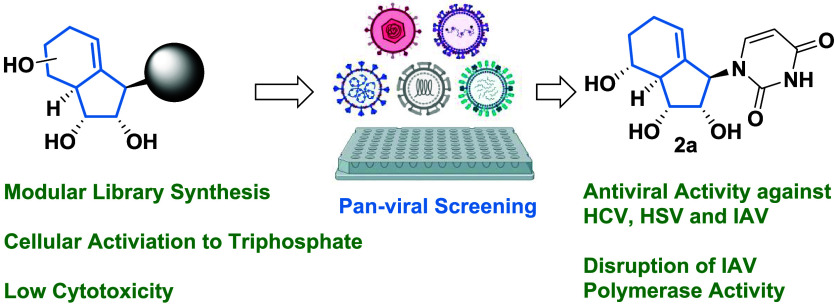

Nucleoside analogues (NA) are an established source of drugs, as demonstrated by the recent development of novel antivirals.^1–7^ While they have been highly successful in treating chronic diseases such as human immunodeficiency virus (HIV) and hepatitis B virus (HBV), their use against respiratory viruses such as influenza, severe acute respiratory syndrome coronavirus 2 (SARS-CoV-2), and respiratory syncytial virus (RSV) remains limited. Therapies against respiratory viral infections primarily rely on neuraminidase and protease inhibitors, such as oseltamivir (for influenza) and nirmatrelvir (for SARS-CoV-2). One factor contributing to the limited application of traditional NAs is the limited structural diversity. Typically, modification focuses on altering the nucleobase, as seen in ribavirin, molnupiravir,^2,7^ or obeldesivir (for more marketed NAs, Figure S1).^8,9^ Aside from truncated acyclic NAs, such as adefovir, NAs almost exclusively feature a ribose-type structure. Carbocyclic NA, designed by the replacement of the ring oxygen with methylene, are an important scaffold in NA research, as they promise higher metabolic stability and higher flexibility in the (synthetic) design of analogues.^10^

Although having these inherent structural advances, carbocyclic NAs are less common. This is attributed to the missing anomeric stabilization compared to the ribose conformation,^11^ as even subtle changes in conformation lead to inactive analogues. This was partially resolved by the development of locked sugars with similar conformation to ribose.^12^ In our approach, we have introduced a novel carbobicyclic NA scaffold (Figure 1B). The scaffold acts as conformational mimic of ribose as evident by the superimposition of their X-ray conformations.^13^ In our pilot study, we demonstrated that carbobicyclic ribavirin-type (**1**) and uridine-type (**2**) nucleoside analogues can efficiently reduce the RSV viral load in HEp-2 cells. Compounds **1a**–**c** exhibited greater activity than their parent compound ribavirin, while the cytotoxicity remained low.^13^

**Figure 1:**
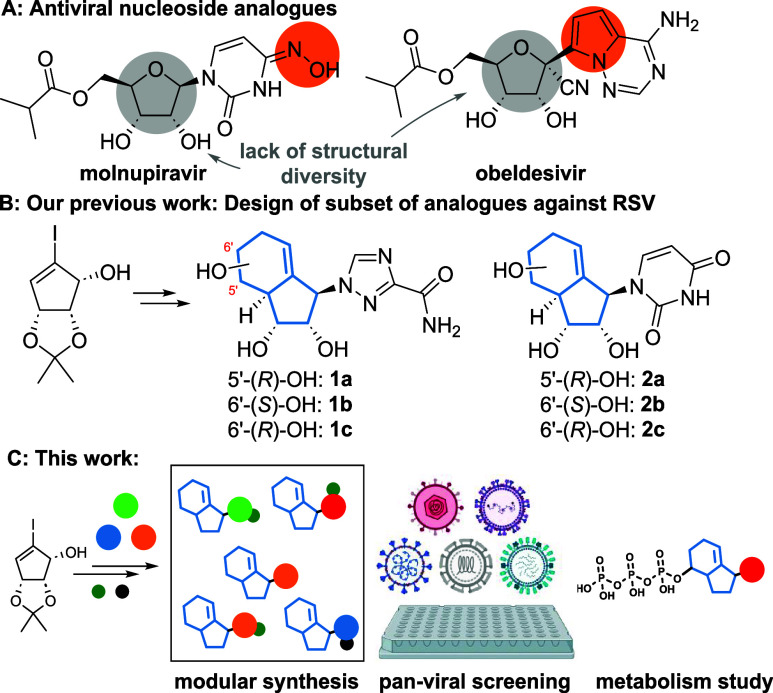
(A) Structures of selected marketed drugs. (B) Carbobicyclic NA scaffold synthesized in a pilot study. (C) Platform to investigate the antiviral potential of carbobicyclic NA.

In this study, we evaluate this novel, ribose mimicking, scaffold for its broad-spectrum antiviral efficacy, metabolic profile, and host cell interactions (Figure 1C). Conceptually, our study began with the synthesis of a library of NAs. Subsequently, biological evaluation informed further structure–activity relationship (SAR)-driven synthesis. In parallel, we conducted cellular metabolism studies to evaluate the triphosphate formation of these NAs.

## Results and Discussion

### Convergent Synthesis of Carbobicyclic Nucleoside Analogues Yielded an In-House Drug Library

In addition to ribavirin analogues **1** and uridine analogues **2** from our pilot study, we synthesized an expanded library of carbobicyclic NAs. As illustrated in Figure 2, this library includes both modified nucleobases (blue circle) and naturally occurring nucleobases (green circle). Several analogues were inspired by known antivirals, including molnupiravir, ribavirin, and 5-fluorouracil (5-FU) (red circle).

**Figure 2:**
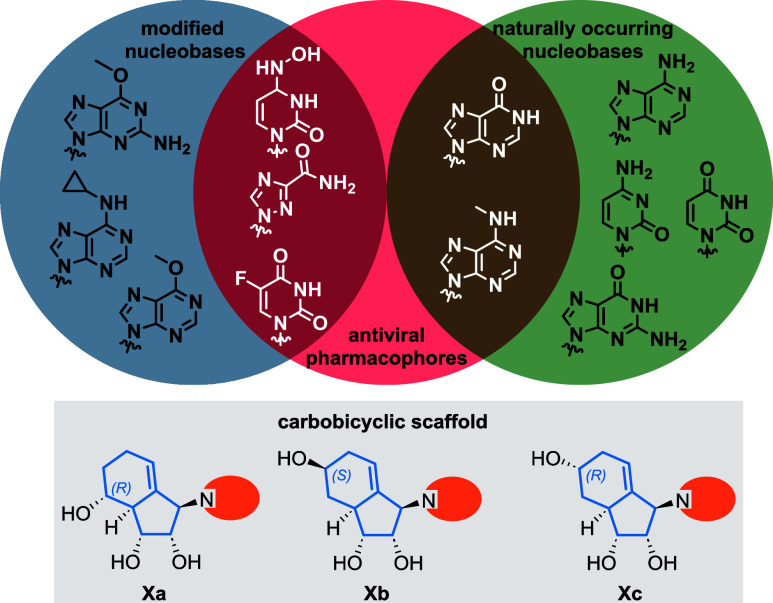
Schematic overview of structural diversity of carbobicyclic NAs in this study.

Following our previously published route, the analogues were typically synthesized in fewer than 10 steps from commercial substrates. For a full list of synthesized nucleoside analogues in this work, see Table S1 in the Supporting Information. Each unique nucleobase coupling was assigned a distinct compound number (e.g., *N*-hydroxy cytidine (NHC) **3**, adenine-type analogues as **5–8**, guanine-type analogues as **9–12**) with different isomers denoted by a subscript letter (e.g., **Xa**, **Xb**, **Xc**). The detailed synthesis of the compounds is outlined later in this work.

### Pan-viral Screening Identifies Promising Candidates

We benchmarked our carbobicyclic NAs against viruses from eight different families, concentrating on those with pandemic potential:

1. *Picornaviridae* (coxsackievirus A16, human rhinovirus 1B, enterovirus 71)
2. *Flaviviridae* (HCV GT1b, yellow fever virus 17D, Zika virus)
3. *Paramyxoviridae* (HPIV-3)
4. *Coronaviridae* (HCoV-229E, HCoV-OC43)
5. *Orthomyxoviridae* (influenza A/H1N1, A/H1N1pdm, A/H3N2, B)
6. *Pneumoviridae* (RSV A)
7. *Togaviridae* (Sindbis virus, Semliki Forest virus)
8. *Herpesviridae* (HSV-1)

The results revealed antiviral activity against HCV, HSV, HPIV-3, and IAV H1N1pdm (see Figure 3 and Tables S2–S4 for full results). Among the uridine analogues, **2a** exhibited antiviral activity against HCV, IAV (H1N1pdm), Zika virus, and HSV, but increased cytopathic effect (CPE) in HPIV-3-infected cells. Its isomers **2b** and **2c**, however, were not only inactive against HCV, but also enhanced viral replication, thus reversing the effect of **2a**. In contrast, for IAV all three isomers of **2** showed similar antiviral activity (*vide infra*). The closely related cytosine and NHC analogues **3** and **4** were inactive, which underlines the importance of the unmodified uracil moiety as a pharmacophore. Overall, despite their structural resemblance, none of the pyrimidine analogues except **2a** exhibited significant antiviral properties. For HCV, the strongest inhibition observed in this screening was 94% at 40 μM (24% at 2 μM) for **2a**.

**Figure 3:**
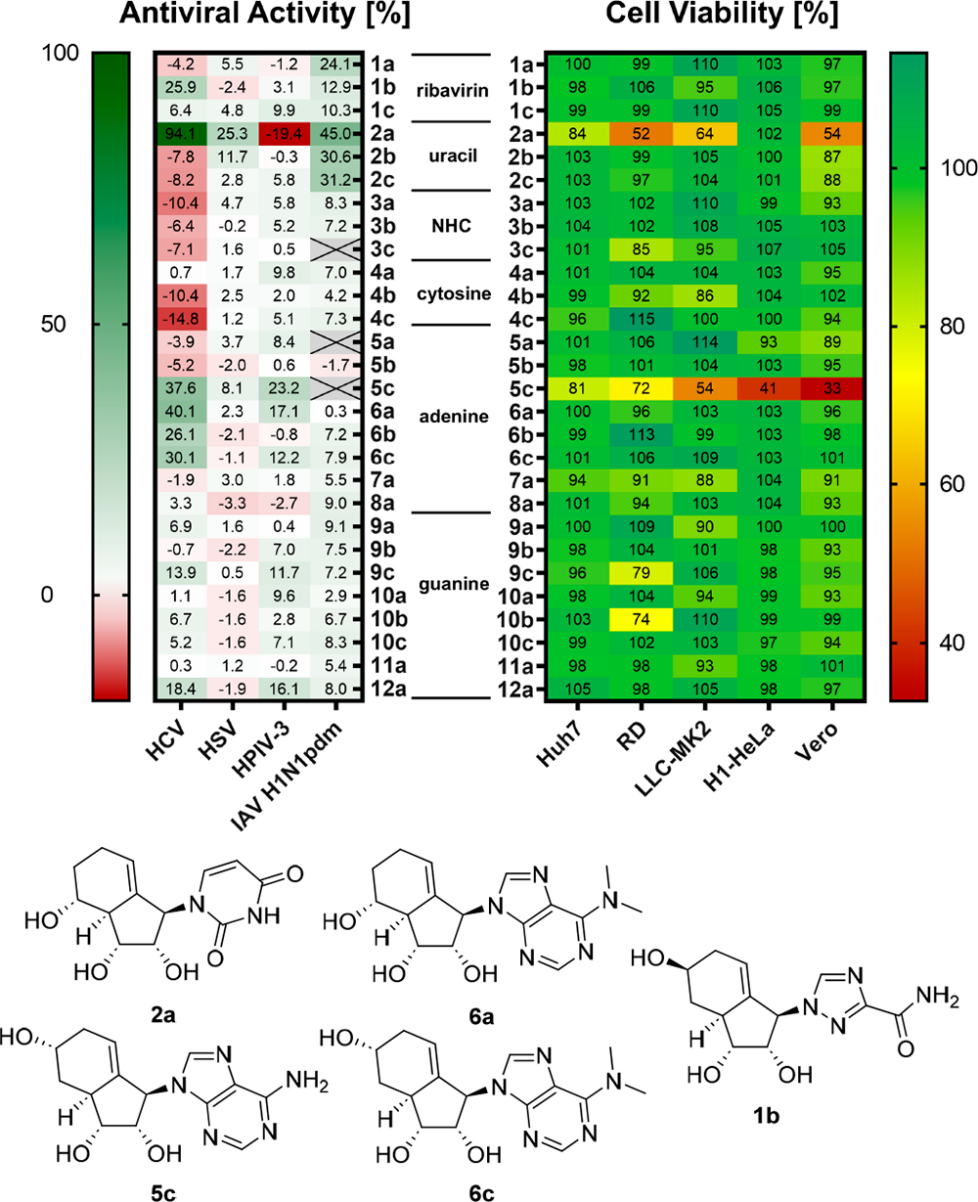
Biological assessment of NAs. Antiviral activity is expressed as % protection against virus-induced CPE, relative to uninfected cells, at a compound concentration of 40 μM. For HCV, a replicon assay was used. Cell viability is expressed as a percentage in comparison to the DMSO control at 40 μM. Cell lines used for CPE assay: HCV (Huh7), HSV (Vero), HPIV (LLC-MK2), IAV (MDCK). Values are mean for *n* ≥ 3.

Next, we evaluated the antiviral and cytotoxic profiles of adenine analogues **5–8**. Compound **5c** demonstrated activity against at least two viruses (HPIV-3, HSV). In the HCV replicon assay, **5c** reduced viral load by 38% (±7%, *n* = 3). However, it also exhibited significant cytotoxicity across multiple cell lines. This cytotoxicity was reduced in the *N*,*N*-dimethyl analogue **6c**; however, this modification also led to a concomitant decrease in antiviral activity. The antiviral activity was restored in compound **6a**, which is equipotent to compound **5c** and maintains sufficient cell viability in uninfected cells. It is important to note that the ribose-based *N*,*N*-dimethyl adenosine itself exhibited significant cytotoxicity (with 19% cell viability at 20 μM in the MDCK cell line) and did not display any antiviral activity in any of our assays. While **2a** was highly active against HCV but not HPIV-3, the adenosine analogues **5c** and **6a** displayed moderate activity against both viruses.

A similar pattern was observed among guanine analogues **9–12**. Analogue **9c**, the guanosine counterpart of **5c**, showed a similar activity profile, but with reduced potency. Within the guanosine series, only **12a** (with 2-amino-6-dimethylamine purine nucleobase), which is structurally related to **6a** (6-dimethylamine purine), demonstrated superior antiviral activity. In contrast, compound **11a** (2-amino-6-methylamine purine) was inactive.

Based on our previous findings, we anticipated that NAs bearing nucleobases known to confer antiviral activity would yield active carbobicyclic NA. To our surprise, most analogues in this group were completely inactive. Only compound **1b** exhibited limited activity against HCV, while compounds **1a**–**c** showed activity against IAV (H1N1pdm). Generally, NHC-type analogues **3a**–**c** showed low activity.

Interestingly, while some compounds induced an antiviral effect (e.g., **2a** or **6a**), others unexpectedly enhanced viral replication (e.g., the cytidine analogue **4c**). Importantly, this proviral effect was not correlated with compounds’ cytotoxicity. Most of the compounds lacked cytotoxicity across a broad panel of cell lines, with a few outliers such as the uracil-type analogue **2a** and the adenosine-type analogue **5c**. Cytotoxicity was cell line dependent: for example, **2a** was cytotoxic in Huh7, RD, LLC-MK2, and Vero cells, but not in H1-HeLa, MDCK, or A549 cells. Furthermore, despite their cytotoxicity, both **2a** and **5c** demonstrated antiviral activity.

In summary, our screening campaign identified several candidates, such as compounds **1b**, **2a**, **5c**, and **6a**, as potential scaffolds for further SAR optimization. While their overall activity needs to be optimized further to identify potential drug candidates, the screening indicated that carbobicyclic nucleoside analogues have the potential to act as antiviral scaffolds.

### Ribavirin Analogue **1b** Induced Viral Load Reduction in RSV without Cell-Protective Effect

All tested compounds, including analogue **1b**, were inactive in the CPE assay against RSV (see Table S3). This contrasts with our previous findings, where **1b** displayed greater antiviral activity than the parent compound ribavirin in the TCID_50_ assay. To investigate this discrepancy, we examined the RSV activity of **1b** in both CPE and viral load (TCID_50_) assays. In the CPE assay, the observed antiviral activity could not surpass 50%. Furthermore, the antiviral effect of **1b** decreased at higher multiplicities of infection (MOI), while results at lower MOIs were inconsistent and showed high standard deviations (Figure 4). These findings align with the overall negative results from CPE-based screening. However, when assessing the viral load reduction, as in our previous study, **1b** demonstrated a clear antiviral effect, reducing the viral load by more than 1000-fold (Figure 4).^13^

**Figure 4:**
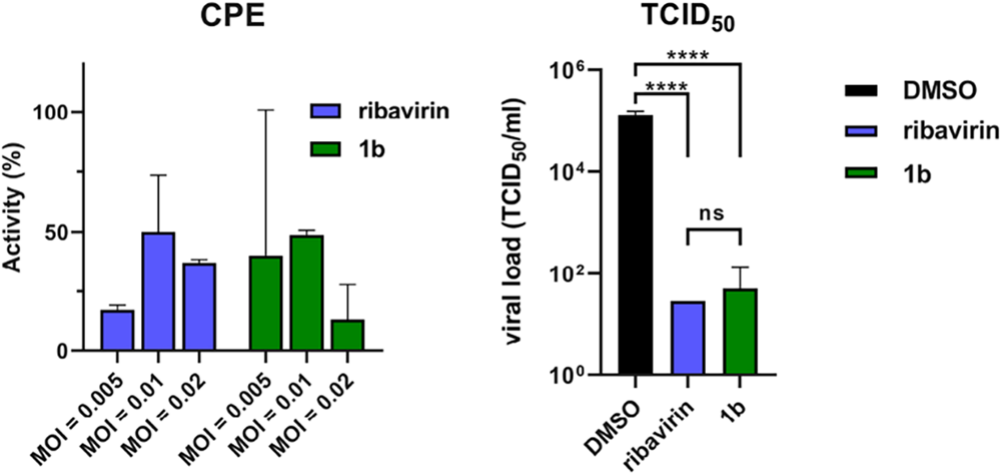
Antiviral activity of ribavirin and compound **1b** against RSV measured in CPE (CCK-8-based cell viability assay) and TCID_50_ (MOI = 0.01) assay. HEp-2 cells were treated with 40 μM of each compound. Values are mean for *n* = 3.

In other words, although **1b** reduced the viral load compared to the DMSO negative control, qualifying it as a positive hit in the TCID_50_ assay, it did not protect the cells from virus-induced cell death or cytopathic effects. This delivers a key message: a reduction in viral load does not necessarily translate into observable protection in a CPE assay. This distinction is important as early-stage screening campaigns often rely on CPE assay due to their cost-effectiveness and simplicity. In sum, CPE-based readouts may not correlate with actual reduction in viral load, and *vice versa*, underscoring the need for complementary assay strategies in antiviral drug discovery.

### TCID_50_-Based IAV Viral Load Reduction Does Not Guarantee Cell Protection in CPE Assays

Following the initial screening, we selected influenza A virus as the model for our follow-up SAR study. Based on previous findings with RSV, a reduction in TCID_50_ does not necessarily translate into an antiviral effect in the CPE assay. To investigate whether this discrepancy also applies to IAV, we compared both assay types in our IAV model. Potent antiviral activity against IAV in the CPE assay was observed only for ribavirin and oseltamivir (Figure 5A); compound **1a** exhibited moderate activity, while compound **1b** had minimal effects. At the same time, **1b** reduced viral load in the TCID_50_ assay, although this effect diminished at higher MOI (Figure 5B). Ribavirin maintained consistent viral load reduction across all MOIs. Next, we evaluated **1b** and ribavirin in an *ex vivo* model at 24 and 48 h postinfection (hpi) (Figure 5C). Both compounds achieved a 10-fold reduction in viral load at 24 hpi. Importantly, for seasonal H1N1, the reduction was sustained at 48 hpi; however, in an oseltamivir-resistant H1N1pdm strain, the antiviral effect of both compounds declined by 48 hpi.

**Figure 5:**
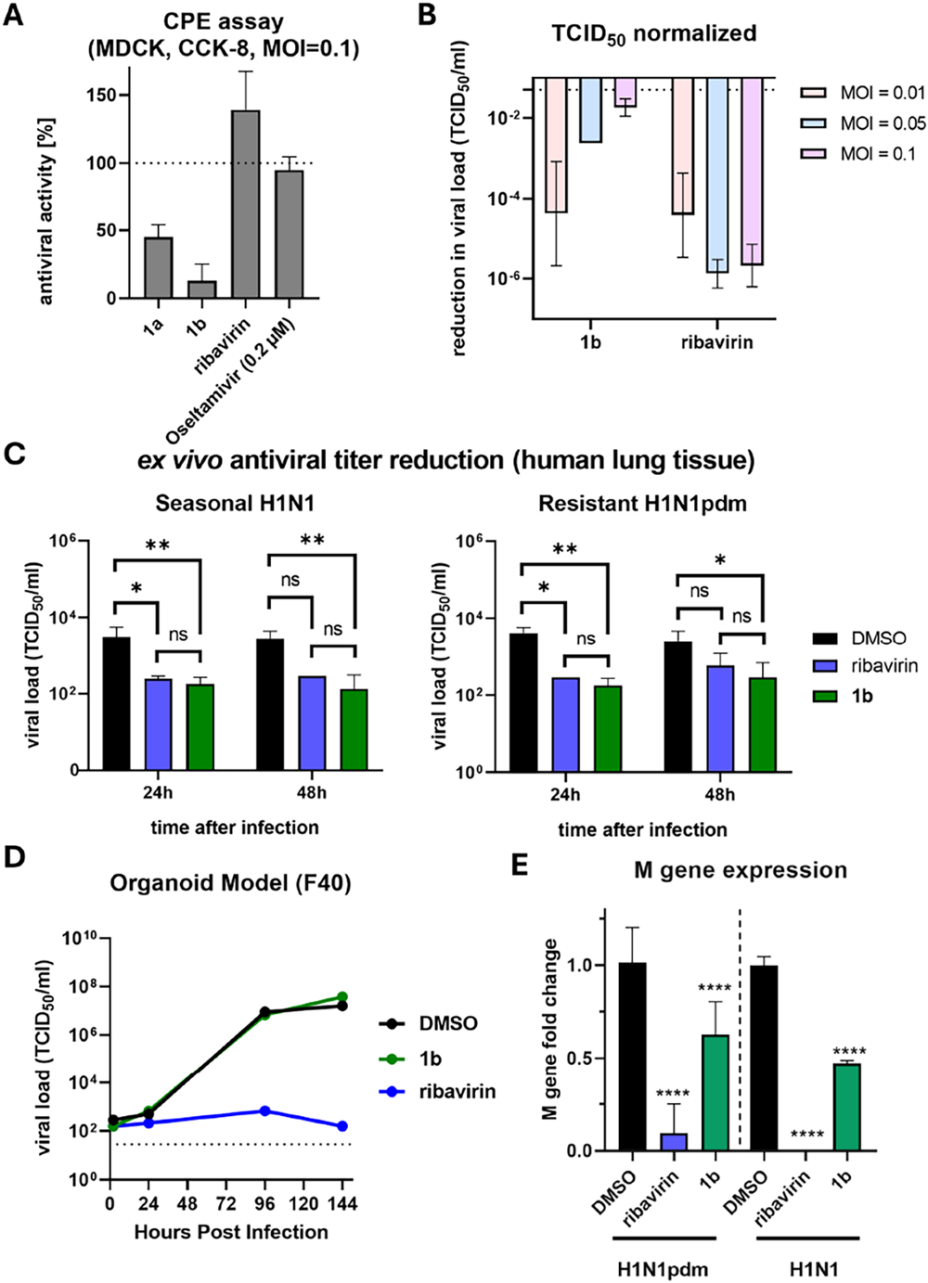
Comparative analysis of antiviral effects of test compounds against two influenza A virus strains: a clinical isolate (H1N1pdm) and A/Oklahoma/447/2008 (H1N1), using multiple assay systems. (A) CPE assay in MDCK cells (MOI = 0.1), with compounds (40 μM) added simultaneously with infection and CPE measured at 48 hpi. Antiviral activity is expressed as % protection relative to uninfected control. (B) TCID_50_ assay of MDCK supernatants collected at 48 hpi, normalized to DMSO control. (C) Time-dependent *ex vivo* human lung model and (D) nasal organoid infection model (MOI = 0.01), with supernatants collected at 2, 24, 96, and 144 hpi analyzed for viral titers. (E) qPCR assay in A549 cells (MOI = 2), quantifying viral M gene expression at 24 hpi from cDNA of infected cells. Values represent mean for *n* ≥ 3. Concentration of compounds, 40 μM.

To assess the translational potential of compound **1b**, the same experiment was repeated in a human nasal organoid model (Figure 5D). While ribavirin retained its efficacy in reducing infectious viruses, compound **1b** failed to suppress IAV replication. IAV matrix (M) gene expression was also assessed in A549 cells (MOI = 2) via qPCR; compound **1b** (40 μM) showed a significant, albeit less pronounced, reduction in viral RNA compared to ribavirin.

Compound **1b** exhibited potent antiviral activity in TCID_50_ assays using MDCK cells, as indicated by decreased infectious viral titers (Figure 5B), but this did not translate into a cell-protective effect (Figure 5A). Although a reduction in viral load indicates replication is suppressed, this may not be sufficient to rescue cells that already committed to programmed cell death. IAV is known to activate multiple cell death pathways, including apoptosis, pyroptosis, and necroptosis, independently of its replication efficiency.^14^ This narrows the therapeutic window during which antiviral agents can exert protective effects. Thus, although compound **1b** reduced viral replication, it did not prevent the activation of these cytopathic responses. Supporting this notion,^15^ previous work has shown that pentagalloylglucose (PGG) inhibits IAV replication not only by interfering with viral entry but also by suppressing viral budding and release. A similar mechanism may underlie the action of compound **1b**, resulting in partial inhibition of the viral life cycle without full protection from infection-induced cell death. Consequently, our subsequent studies focused on the CPE assay, as reductions in viral load observed in the TCID_50_ assay do not reliably predict cellular protection.

### SAR Study on the Antiviral Activity against Influenza A Virus

To improve the antiviral efficacy further pyrimidine and purine analogues were synthesized and benchmarked in their activity against IAV. First, we assessed the SAR data for pyrimidine analogues such as **2a** (see Table 1 for activity of selected compounds and Table S4 for full results), while later the SAR data for purine analogues is discussed (*vide infra*).

**Table 1:**
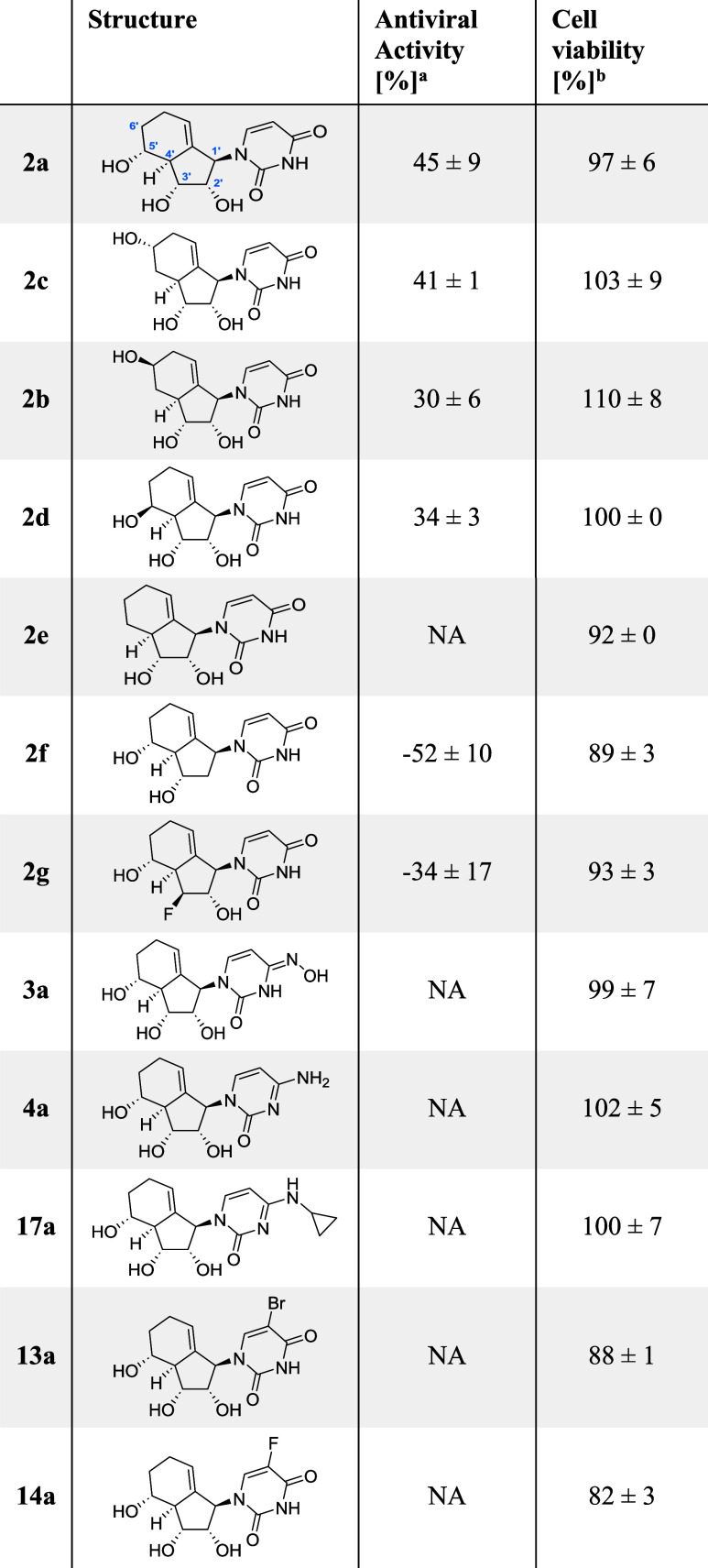
Antiviral Activity against IAV of Uridine Analogues

a CPE assay in IAV (H1N1pdm) infected MDCK cells. Activity compared to DMSO control and uninfected cells.

b Cell viability of uninfected cells after compound treatment (40 μM) in MDCK cells obtained by CCK-8 assay normalized to DMSO. Values are mean ± SD for *n* ≥ 3. Negative values indicate increased CPE relative to DMSO. NA stands for not active (CPE < 10% or inconclusive).

To begin with, we explored the role of the OH group at C5′. Isomers **2a**–**c** were obtained via the Diels–Alder reaction, whereas **2d** was synthesized by epimerization of **2a** (*vide infra*). These 5′/6′ analogues have been expected to display a fundamentally different antiviral activity, as this site is typically used for phosphorylation and RNA incorporation. To our surprise, all four analogues **2a**–**d** displayed similar antiviral profiles, with compound **2a** showing the highest activity (Table 1). Although the position and stereochemistry of the hydroxy group are to some extent flexible, the 5′-deoxy compound **2e** lost all antiviral activity. These observations align with the mode of action of NA in general as no activation of the 5′-deoxy compound to its triphosphate is possible. The flexibility in hydroxy substitution pattern at C5′/C6′ is further supported by our *in silico* model (*vide infra*).

We next explored structural modifications of the carbobicyclic core, focusing on substitutions at the C2′ and C3′ positions. Typically, C3′ analogues can act as chain terminator in antiviral drug development. If the analogue is a viable building block of RNA, the removal/substitution of C3′ should increase the activity, as such analogues cannot be prolonged leading to a chain termination. The observed results however were different: The fluoro-substituted C3′-analogue **2g** enhanced CPE and therefore promoted proviral activity. This effect was similarly observed for C2′-analogues. Not only was the pseudo-2′-deoxy analogue **2f** inactive but again promoted CPE effect (= proviral activity) by over 50%. This effect cannot be fully attributed to cytotoxicity, as cell viability in uninfected cells remained high (89%).

It is worth mentioning that the RNA-type molecule **2a** and the C2′-deoxy analogue **2f** exhibited opposing phenotypes: **2a** has the highest activity in our assay, whereas **2f** led to the strongest CPE among all tested compounds. These observations that changes in C2′/C3′ cannot increase the antiviral activity were later found to be validated by *in silico* modeling (*vide infra*). However, the proviral activity of C2′/C3′ is still under exploration.

Next, changes in the pyrimidine nucleobase have been investigated. Lately, uracil/cytidine analogues such as molnupiravir have been successfully introduced as novel antiviral drugs.^2,7^ Despite bearing the pharmacophore of molnupiravir, the NHC-type analogue **3a**, as well as the cytidine analogue **4a** and **17a**, failed to demonstrate antiviral activity. We also investigated 5-halogen modifications of the uracil base, including 5-bromo uracil (**13a**) and 5-fluorouracil (**14a**) as 5-halogen-uracils are known to be bioactive.^16^ However, neither compound **13**/**14a** showed significant antiviral activity.

In summary, while changes in the carbobicyclic scaffold at C5′ and C6′are tolerated, changes in C2′ and C3′ lead to a proviral activity. Furthermore, while the native uracil base displays antiviral activity when coupled with the novel carbobicyclic core structure, its close analogues with known antiviral activity are inactive. This hints at an activity-altering effect of the carbobicyclic nucleoside analogue scaffold. The results are summarized in Figure 6.

**Figure 6:**
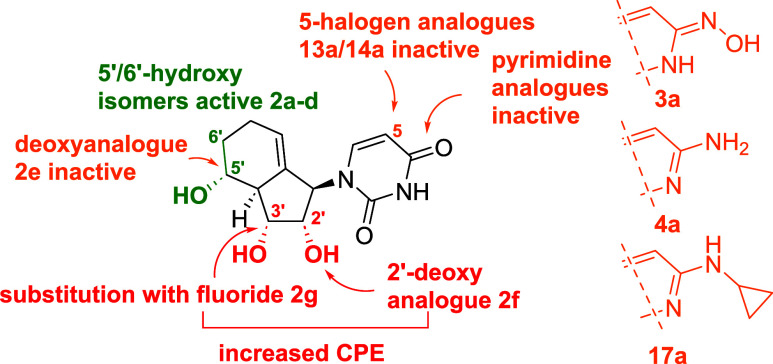
Schematic overview of SAR of analogue **2a**.

We next focused on purine-type analogues. Only a limited number of purine-based compounds displayed antiviral activity against IAV, as shown in Table 2 (CPE assay, 40 μM, ranked by activity). The four most active compounds were **15b**, **5d**, **1a**, and **16a**. Compound **15b** was of particular interest due to its thiopurine scaffold, which is known for immunosuppressive effects, as seen with azathioprine. Despite limited research on the antiviral potential of thiopurines, recent studies have shown that thioguanine and thioguanosine, but not mercaptopurine, can trigger the formation of stress granules in IAV-infected cells.^17^ Aside from **15b**, its isomers **15a** and **15c** did not demonstrate significant activity. Furthermore, other inosine analogues such as **8a** and **8b** were also inactive. Next, the adenosine analogue **5d** displayed activity comparable to that of **2a** and **15b**; however, its isomer **5b** lacked measurable activity.

**Table 2:**
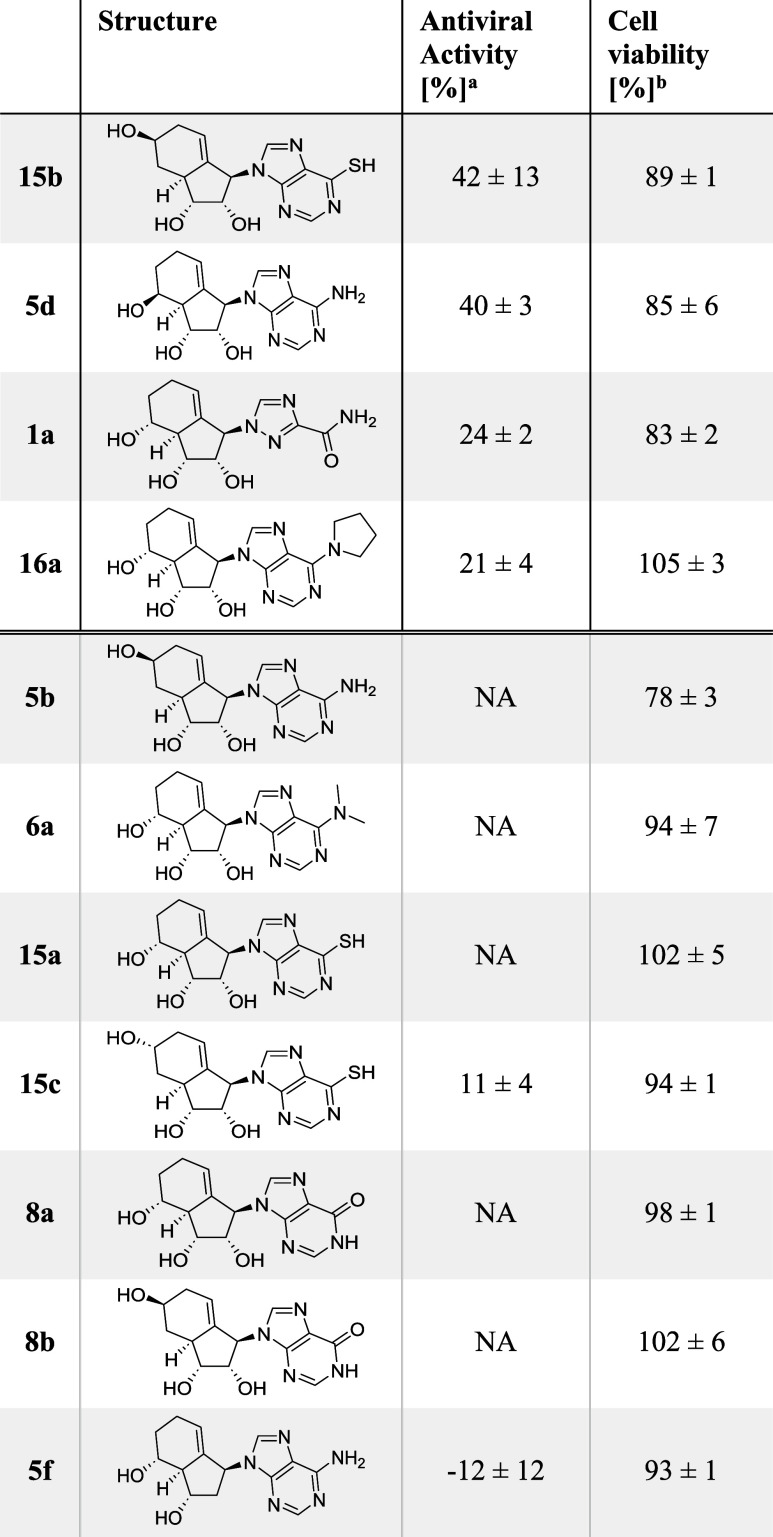
Antiviral Activity against IAV of Purine Analogues

a CPE assay in IAV (H1N1pdm) infected MDCK cells. Activity compared to DMSO control and uninfected cells.

b Cell viability of uninfected cells after compound treatment (40 μM) in MDCK cells obtained by CCK-8 assay normalized to DMSO. Values are mean ± SD for *n* ≥ 3. Negative values indicate increased CPE relative to DMSO. NA stands for not active (CPE < 10% or inconclusive).

While the ribavirin analogue **1b** could not reduce virus-induced CPE, its isomer **1a** showed moderate activity. Finally, the substituted adenosine analogue **16a** exhibited antiviral activity, in contrast to its structural analogue **6a**, which was inactive. Interestingly, the 2′-deoxy adenosine analogue **5f** promoted IAV-induced CPE, similar to the proviral effect observed with **2f**. In conclusion, purine analogues displayed lower activity against IAV compared to pyrimidine analogues such as **2a**.

### Carbobicyclic Analogue **2a** Disrupts IAV Polymerase Activity

Based on these findings, we investigated whether the antiviral activity of selected nucleoside analogues could be attributed to direct inhibition of the IAV RNA-dependent RNA polymerase (IAVpol). To this end, we employed a cell-based dual-luciferase minigenome assay as previously described (Figure 7).^18,19^

**Figure 7:**
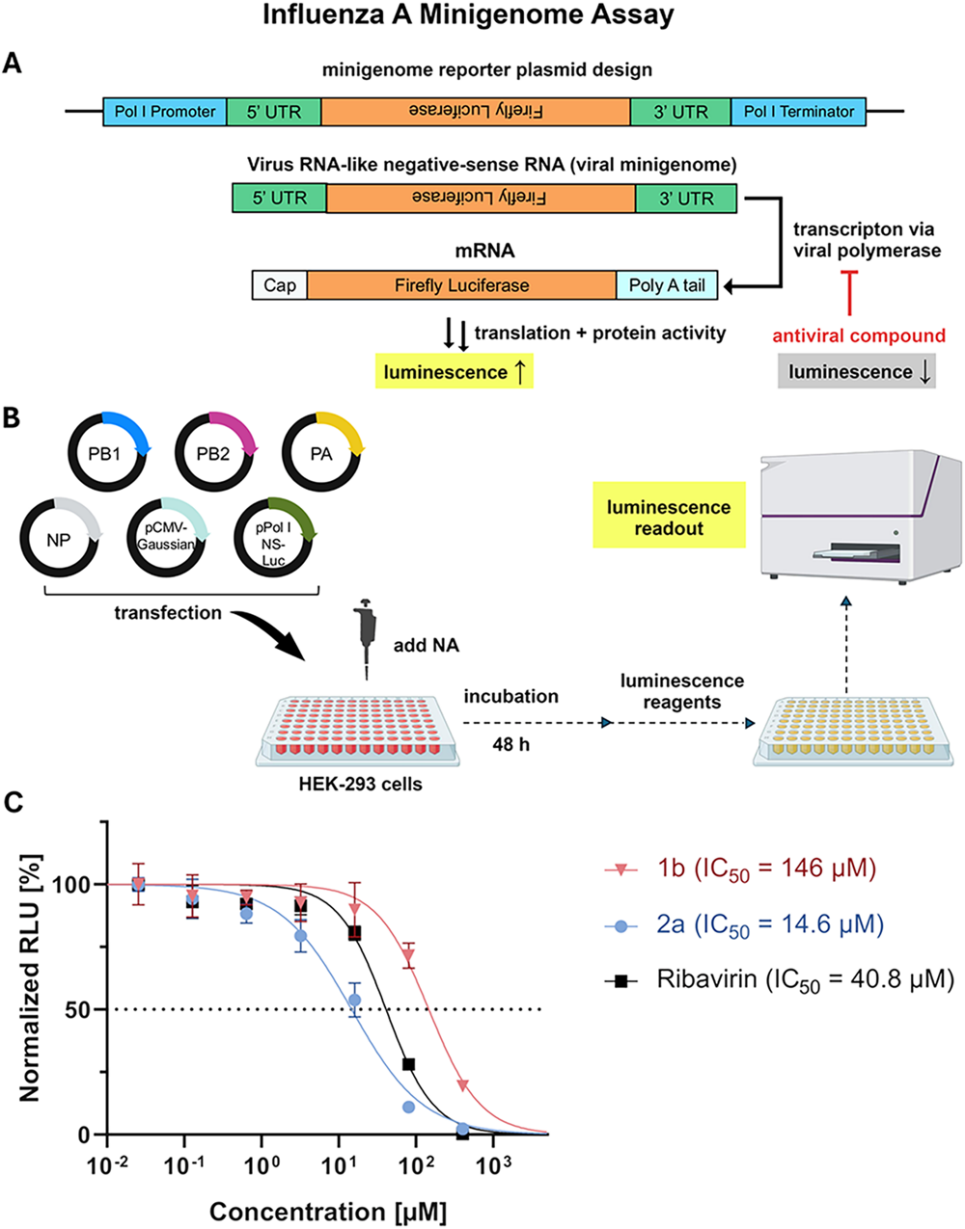
Minigenome assay: (A) Schematic of the firefly luciferase reporter construct. (B) Workflow of the IAV minigenome assay for NA screening. (C) Dose–response curves for compounds **1b** and **2a**, compared to ribavirin. A 5-fold serial dilution was performed (400 μM to 0 μM). Inhibitory activity is expressed as the ratio of firefly to Gaussia luciferase signal, normalized to the DMSO control (100%). The data points were then fitted to a Hill equation to determine the IC_50_ values. Each data point represents three replications.

The assay utilizes a construct containing the 5′ and 3′ untranslated regions (UTRs) of the viral genome flanking a negative-sense firefly luciferase gene. This minigenome is transcribed in cells under the control of the human Pol I promoter and terminator for RNA production. The viral minigenome is then recognized and captured by the viral polymerase complex, driving the transcription of the positive-sense mRNA for firefly luciferase expression. A reduction in luciferase signal indicates that the compound (or its cell metabolite) is likely to directly or indirectly inhibit/interfere with the viral polymerase complex activity (such as replication, or cap-snatching), or the ability to produce a correct RNA sequence. The assay is conducted by cotransfection of HEK-293T cells with plasmids coding viral polymerase subunits (PB1, PB2, PA, and NP), firefly luciferase reporter plasmid, and Gaussia luciferase control plasmid (Figure 7B). Test compounds were added post-transfection, and after 48 h, cells were lysed and analyzed using dual-luciferase reagents. Luminescence was measured for both firefly and Gaussia luciferase to normalize for transfection efficiency.

For this assay, compounds **2a**, **5d**, and **15b** have been assessed as they have shown the highest CPE activity (*vide supra*). Our results suggest that **2a** has a strong inhibitory effect on IAVpol activity, with an IC_50_ of 14.6 ± 1.3 μM (Figure 7C), surpassing ribavirin (40.8 ± 3.8 μM), while purine analogues **5d** and **15b** could not display relevant antiviral activity up to 400 μM (Figure S2 in the SI). Although uracil-type analogue **2a** and adenosine-type analogue **5d** displayed similar activity in the CPE assay, the results of this minigenome assay suggest a different mode of action as **5d** cannot inhibit viral replication, whereas **2a** shows an antiviral effect in the low micromolar range. Next, we also assessed ribavirin analogue **1b** to compare with previous TCID_50_ and CPE activity data (Figure 5). Again, only a weak effect (IC_50_ of 146 ± 16 μM) could be observed in accordance with its inability to provide cell-protective effect. In conclusion, despite sharing the same carbobicyclic scaffold, these analogues exhibit divergent antiviral profiles and mechanisms of action. This observation aligns with prior data showing that **2a** exhibits potent anti-HCV activity, a feature not observed for the other analogues in the series.

### Formation of Triphosphate of Carbobicyclic Analogues in Cells

Despite our extensive efforts in building a diverse library of carbobicyclic nucleoside analogues, none of the compounds displayed antiviral activity higher than 50% at 40 μM. This modest performance led us to hypothesize that inefficient cellular activation (i.e., limited formation of the active phosphorylated metabolites) might be constraining their efficacy. While some nucleoside analogues can act without being metabolized to phosphate forms (e.g., ribavirin can exert immunomodulatory effects^20^) most NAs function as their triphosphate (TP) metabolites.

We therefore examined whether carbobicyclic nucleoside analogues were metabolized into their corresponding monophosphate and triphosphate derivatives in cells (Figure 8A). While X-ray studies confirm that the carbobicyclic scaffold structurally mimics ribose,^13^ this does not guarantee efficient cellular phosphorylation. Unlike natural ribose-based nucleosides, carbobicyclic analogues bear a secondary alcohol at the pseudo C5′ position. There are only a few examples of C5′-modified analogues in the literature, all of which have reduced antiviral activity compared to primary C5′-analogues.^21,22^ The cellular phosphorylation of C5′-substituted analogues remains poorly understood, but steric hindrance at this position likely impairs monophosphate formation, thereby limiting the generation of the biologically active triphosphate species.

**Figure 8:**
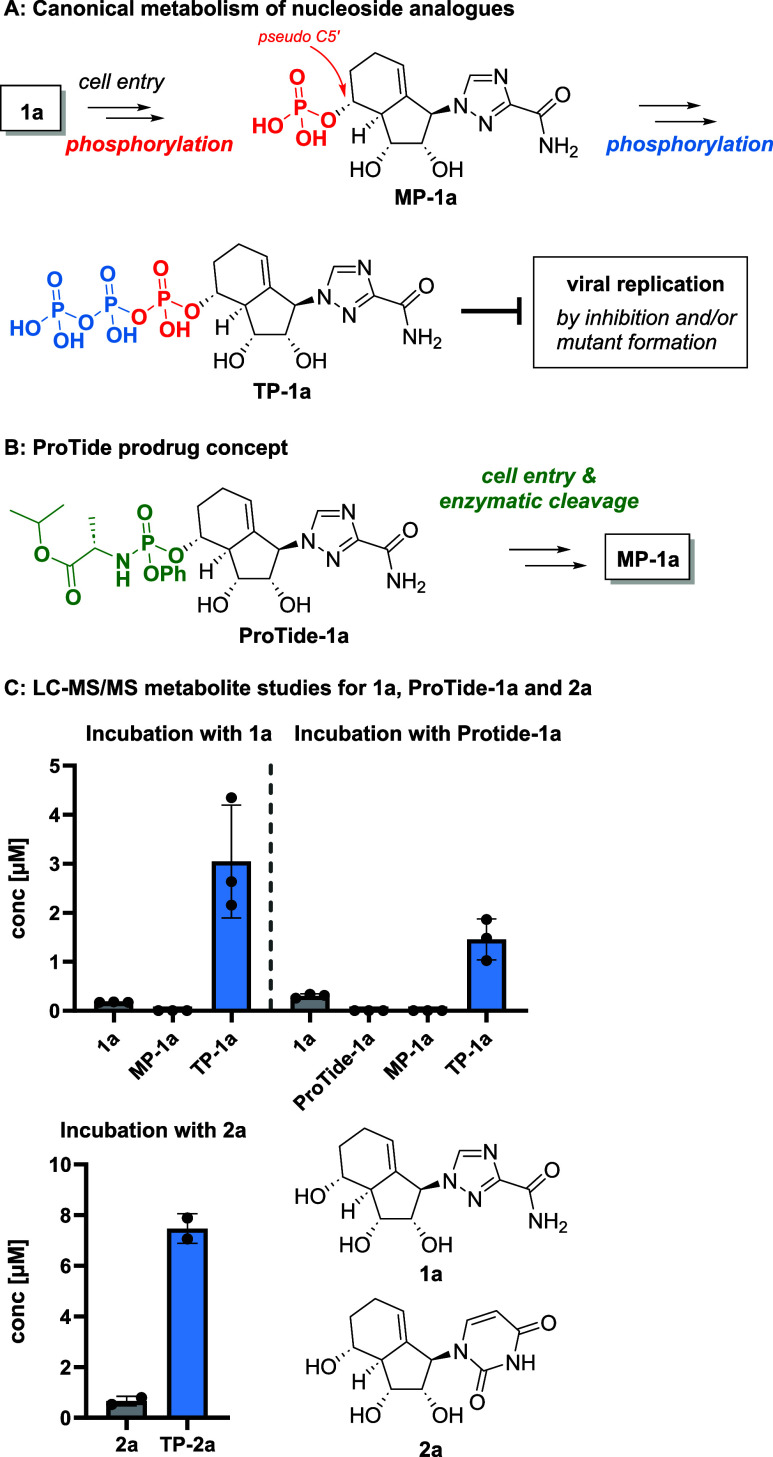
(A) Canonical metabolism of nucleoside analogues and (B) ProTide prodrug concept. (C) Formation of triphosphates **TP-1a** and **TP-2a** was observed after 24 h incubation of compound **1a**, **2a**, or **ProTide-1a** in HEp-2 cells. Bars represent the means of replicates (*n* ≥ 2).

Given the modest antiviral activity observed, we hypothesized that inefficient triphosphate formation might limit the efficacy of our carbobicyclic nucleoside analogues. To address this, we explored the use of prodrug strategies, specifically the ProTide approach, to enhance intracellular delivery of the nucleoside monophosphate form.^23^ For this study, we synthesized a ProTide prodrug of the ribavirin analogue **1a**.^24^ Enzymatic ester cleavage and subsequent hydrolysis of ProTide-**1a** should deliver the monophosphate **MP-1a** directly to the cell (Figure 8B). Next, we synthesized the MP and TP of **1a** and **2a** using methods described in the literature (see Figures S3 and S4 for details).^25,26^

With these substrates as references, we adopted LC-MS/MS to monitor the intracellular metabolism of our carbobicyclic nucleoside analogues. Briefly, HEp-2 cells were incubated with a drug-containing medium (either compound **1a**, **2a**, or prodrug **ProTide-1a** in two independent experiments 10 μM) for 24 h. Surprisingly, LC-MS/MS analysis revealed that TP was efficiently formed from **1a**, **2a**, and **ProTide-1a** (Figure 8C). Despite initial concerns about steric hindrance, **1a** and **2a** were efficiently phosphorylated to **TP** in HEp-2 cells, with only minimal accumulation of **MP**. For **2a**, no monophosphate product could be detected possibly due to their quick conversion to the triphosphate.^27^ Interestingly, minor amount of **1a** was also detected in cells treated with **ProTide-1a**, suggesting its partial hydrolysis back to the parent compound. The differences in the efficacy of the formation of **TP-1a** and **TP-2a** are influenced not only by their ability to be metabolized into triphosphates, but also by the cell permeability and degradation of their parent compounds. These other factors such as variability among different cell lines have not been studied here. It is noteworthy that the success of ProTides in general such as **ProTide-1a** depends on the cellular expression and activity of phosphoamidase in specific target cells, resulting in variable activity across different cell lines.^28^

To evaluate whether **ProTide-1a** could improve antiviral efficacy, we assessed it in a CPE assay against IAV (H1N1) at 40 μM. Only moderate activity was observed (10% ± 3, *n* = 3), which was significantly lower than that of **1a**. Such observation was in accordance with our cell metabolism study, which proved that TP formation was not the activity-limiting factor.

In conclusion, we demonstrated that pseudo C5′–OH carbobicyclic nucleoside analogues could be efficiently phosphorylated to their active triphosphate forms without the need of prodrug strategies. This suggests that host proteins in HEp-2 cells recognize the carbobicyclic scaffold as a suitable substrate, which underlines the usefulness of the ribose-mimic scaffold for antiviral drug development.

So far, the results for all three carbobicyclic NAs (**1a**, **ProTide-1a**, and **2a**) are consistent, and activation of the corresponding triphosphate was observed. Further proof of concept using C6′–OH analogues (e.g., **2b** or **2c**) could not be performed, as it was highly challenging to synthesize the MP and TP of these compounds, which is necessary for LC-MS/MS studies.

### *In Silico* Modeling Suggests Inhibition of Viral Polymerase by Locking the NTP Binding Site

To further elucidate the potential mechanism of action of the carbobicyclic nucleoside analogues, we conducted *in silico* modeling calculations for compounds **TP-1a** and **TP-2a**. To predict the binding mode of carbobicyclic nucleoside analogues in IAV RNA-dependent RNA polymerase (RdRp), we employed the state-of-the-art *in silico* model AlphaFold 3 (AF3),^29^ which allows prediction of protein–ligand complexes with high accuracy.

The feasibility of using AF3 model was supported by the high resemblance between predicted H1N1 IAV RdRp adenosine (A):uridine triphosphate (UTP) elongation complex and published cryo-EM resolved IAV polymerase complexes (Figure S6A–C). When compared with natural UTP, compound **TP-2a** bound to the active site in a precatalytic configuration (Figure 9A,B, Figure S6A,D). While maintaining base pairing interactions with the template nucleotide, the α-phosphate of the triphosphate group was displaced from the catalytic center, largely hindering the nucleophilic attack from C3′-oxygen of the nascent RNA. This suggested that **TP-2a** may noncatalytically inhibit RNA synthesis by locking the NTP binding site. Interestingly, an antiviral adenosine analogue (HNC-1664) was recently reported to inhibit viral RdRp through binding instead of catalysis, with support from cryo-EM studies.^30^ HNC-1664 demonstrated a noncatalytic binding mode in atypical base pairing and out-of-pocket triphosphate in the reported LASV polymerase complex (Figure S6E,F). The reported structural similarity between LASV and IAV RdRps,^31^ in addition to the conservation of viral RdRp catalytic motifs,^32^ further supports the potential analogous binding mode of **TP-2a**. Additionally, further analysis on **TP-2a** and **TP-1a** AF3 predicted complexes suggested that both carbobicyclic nucleoside analogues maintain intact interactions with active site residues of IAV polymerase, potentially forming competitive binding against natural NTP (see the Supporting Information for further details).

**Figure 9:**
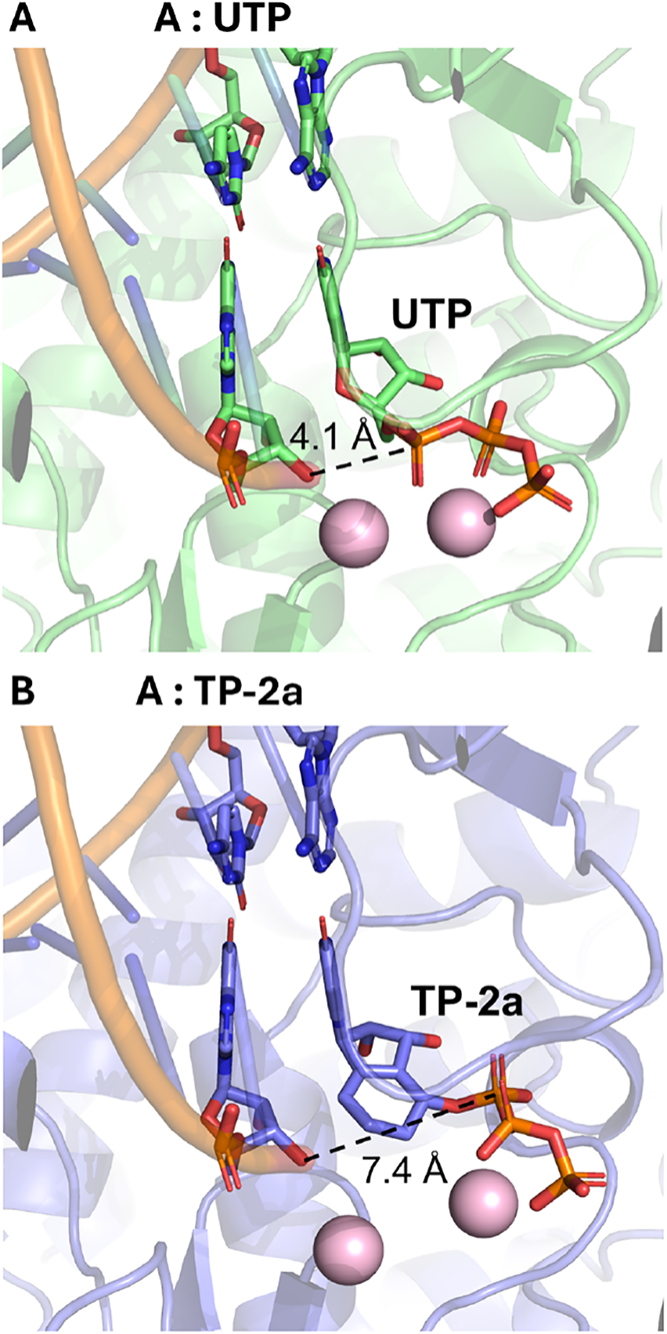
Binding mode of carbobicyclic nucleoside analogues predicted by AF3. (A) H1N1 IAV RdRp A: UTP complex (B) H1N1 IAV RdRp A:**TP-2a** complex. Distance between α-phosphate of the incoming NTP and 3′-oxygen of the nascent RNA was labeled in (A, B).

Our antiviral SAR studies (*vide supra*) validate this *in silico* modeling. As there is no nucleotide incorporation expected, the position of the hydroxy group is not as crucial. All C5′/C6′ stereoisomers **2a**–**2d** show comparable antiviral activity because the mode of action allows a certain flexibility. In contrast, compound **2e**, which lacks the hydroxy group, is inactive. This is because the hydroxy group is required to form the active triphosphate, which interacts with the enzyme’s catalytic center. Next, chain termination strategies, such as C3′ analogues, have failed in the SAR studies due to their inability to incorporate into viral RNA in the first place. Although the experimental data presented are consistent with the *in silico* model, our ongoing studies aim to elucidate this mechanism in depth at the biochemical level.

In summary, although the carbobicyclic scaffold is a substrate of viral polymerase, structural optimizations are necessary for further improvement as antiviral therapeutics.

### Triphosphorylated Analogue Does Not Inhibit Host DNA and RNA Polymerase

A major pitfall in the development of nucleoside analogues as antiviral drugs is their potential for off-target toxicity, particularly mitochondrial toxicity. BMS 986094, for example, was withdrawn from clinical trials for these reasons.^33^ Therefore, in an early stage of our studies, we opted to investigate the interaction of carbobicyclic NAs with human DNA and RNA polymerases, which could indicate potential toxicity. First, the inhibition of **TP-1a** of eukaryotic RNA polymerase II and human mitochondrial RNA polymerase was investigated. As shown in Figure S11, no inhibition of mitochondrial RNA polymerase activity was observed in a radioisotope-based assay at concentrations up to 50 μM. Similarly, **TP-1a** did not inhibit RNA polymerase II.

To further evaluate off-target effects, we also tested **TP-1a** and its parent compound **1a** against DNA polymerases α, β, and γ. As shown in Figure S12, no inhibition was observed at concentrations of up to 100 μM for both the triphosphate **TP-1a** and the parent compound **1a**.

In summary, parental nucleoside analogue **1a** as well as its triphosphate **TP-1a** did not inhibit host RNA and DNA polymerases. Furthermore, cell cytotoxicity screening (*vide supra*) indicated low cytotoxicity for most analogues. These results support the safety profile of this scaffold.

### A Divergent Route Allowed Synthesis in Less Than 10 Steps

The synthesis followed the established route from our pilot study (Figure 10). In short, after the Mitsunobu coupling of nucleobases to **A**, the following Stille coupling should provide diene **B**. This diene is the key intermediate for the following Diels–Alder reaction with vinylboronic acid pinacol ester as in intermediate **C**. Following the oxidation and cleavage of the borate, the carbobicyclic scaffold **D** is established. Final late-stage functionalization and deprotection will liberate the final products **E**. Note, many analogues share the same intermediate, which reduces the overall synthetic workload. For example, inosine and adenosine-type analogues all shared the same intermediate (**D**), and similarly, all pyrimidine analogues can be synthesized using a common intermediate.

**Figure 10:**
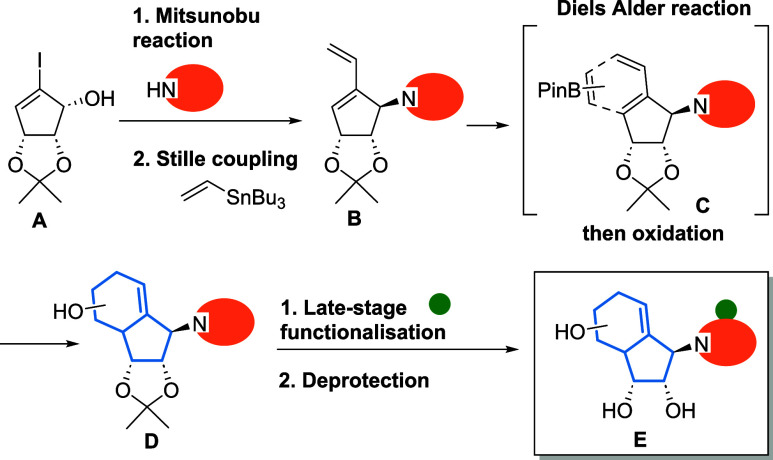
Schematic overview of the synthesis strategy.

The diastereoselective and regioselective outcomes of the Diels–Alder reaction were previously investigated using NOE studies, with further confirmation from X-ray analysis of compound **1c**.^34^ Each stereoisomer exhibits a distinctive fingerprint-like ^1^H NMR pattern in the high field region. Consequently, the stereoselectivity of the subsequent compounds was assigned based on comparisons to this earlier work.^13^

### Synthesis of Purine-Type Analogues

The route to the purine analogues starts from the alcohol **25** as shown in Scheme 1. This alcohol is available by iodination and reduction from commercially available **24** using established methods.^35^ To maintain flexibility during late-stage modifications, we have chosen 6-chloro-purine **26** and protected 2-amino-6-chloro amine **27** as the coupling partners, respectively.

**Scheme 1:**
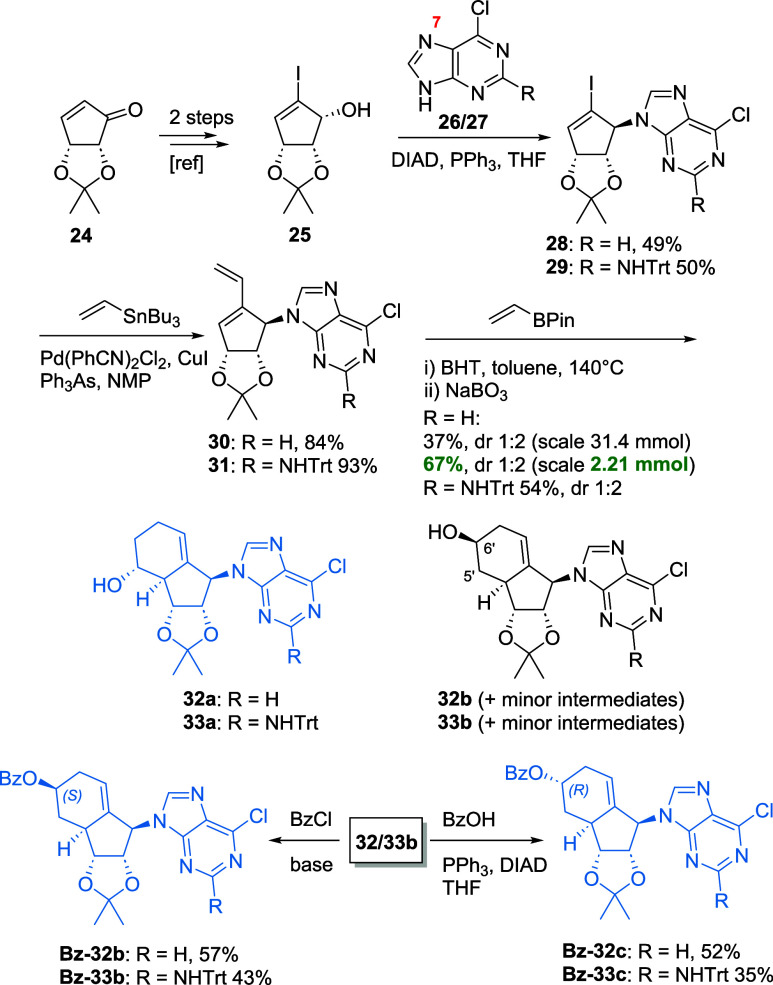
Synthesis of Key Intermediates **32** and **33** for Purine Analogue Synthesis

Both substrates proved to be fruitful partners under Mitsunobu coupling conditions with alcohol **25**. Of note, N7-coupling was also observed, leading to a much more polar side product. Furthermore, as the polarity of the product **28** (R = H) was identical to hydrazine dicarboxylate, an additional step to transform the urea into its less polar Boc analogue for purification purposes was necessary. Next, Stille coupling delivered the Diels–Alder precursors **30** and **31** in very good yields.^36^

During our efforts to scale up the following Diels–Alder reaction, yields dropped significantly from 67 to 37%, although the diastereoselectivity remained identical. Aside from that challenge, we isolated **32** and **33** in multigram scale in sufficient amounts. Of note, while **32a**/**33a** were isolated as single isomers, **32b** and **33b** could only be isolated in pure form after a tedious purification to separate it from the minor pseudo C5′ and C6′ analogues. To isolate the main isomer completely, we derivatized the mixture with BzCl, affording a separable mixture of analogues, with **Bz-32/33b** as the main isomers. Similarly, by employing Mitsunobu conditions, the epimers **Bz-32/33c** could be successfully isolated.

With these key components in hand, we first synthesized adenine-type analogues (Scheme 2A). Therefore, 6-Chloropurine was easily converted into the adenine-type nucleoside analogues **5a**–**c** by treatment with ammonia at elevated temperature, followed by deprotection. For all analogues, TFA was used for the deprotection of the acetonide. However, benzoyl deprotection was sluggish, but using a mixture of K_2_CO_3_, ammonia, and NaOMe allowed the benzoyl protecting group to hydrolyze. For substituted adenosine analogues, such as the *N*-methyl adenosine-type analogues, the synthesis was achieved by substitution of the chloride with a suitable primary or secondary amine (Scheme 2B). Deprotection then yielded analogues **6**,**7** and **19**(**a**–**c**) as well as **21a** and **22a**.

**Scheme 2:**
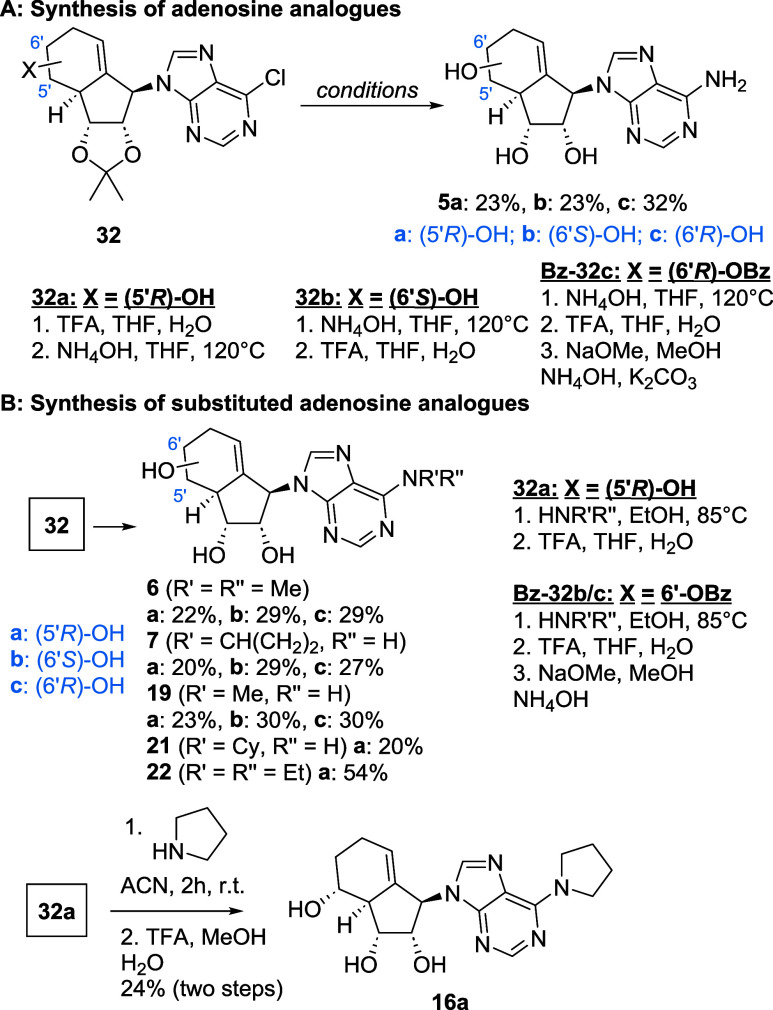
Synthesis of (A) Adenosine-Type and (B) Substituted Adenosine-Type Analogues

Inosine analogues **8a**–**c** were available from key intermediates **32a**–**c** under acidic conditions, followed by basic deprotection as shown in Scheme 3A following literature procedures.^37^ Similarly, the 6-methoxy analogue **23a** was synthesized from compound **32a**, by reaction with sodium methoxide, followed by cleavage of the acetonide. Finally, 6-mercaptopurine analogues **15a**–**c** were synthesized using potassium thioacetate as the nucleophile, followed by hydrolysis to the thiol (Scheme 3B).^38^

**Scheme 3:**
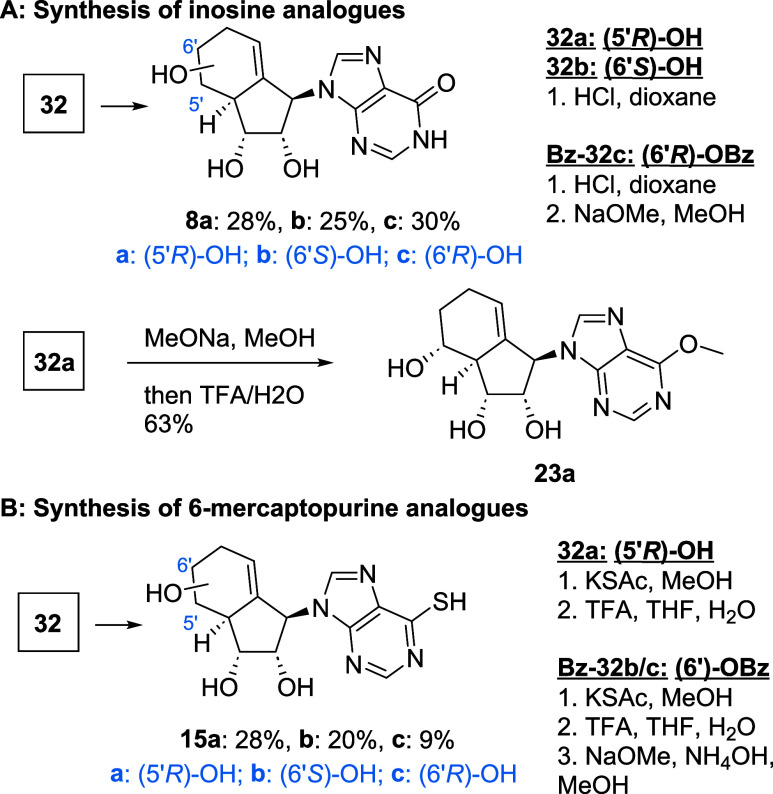
Synthesis of (A) Inosine and (B) 6-Mercaptopurine-Type Analogues

The synthesis of guanosine-type analogues is shown in Scheme 4. With key intermediates **33a** in hand, acidic treatment not only leads to hydrolysis of the 6-Cl purine but also deprotects the carbasugar diol as well as the 2-amino trityl group, furnishing compound **9a**. For the benzoylated substrates **Bz-33b**/**c** an additional alkaline deprotection step was required to access compounds **9b**–**c**.

**Scheme 4:**
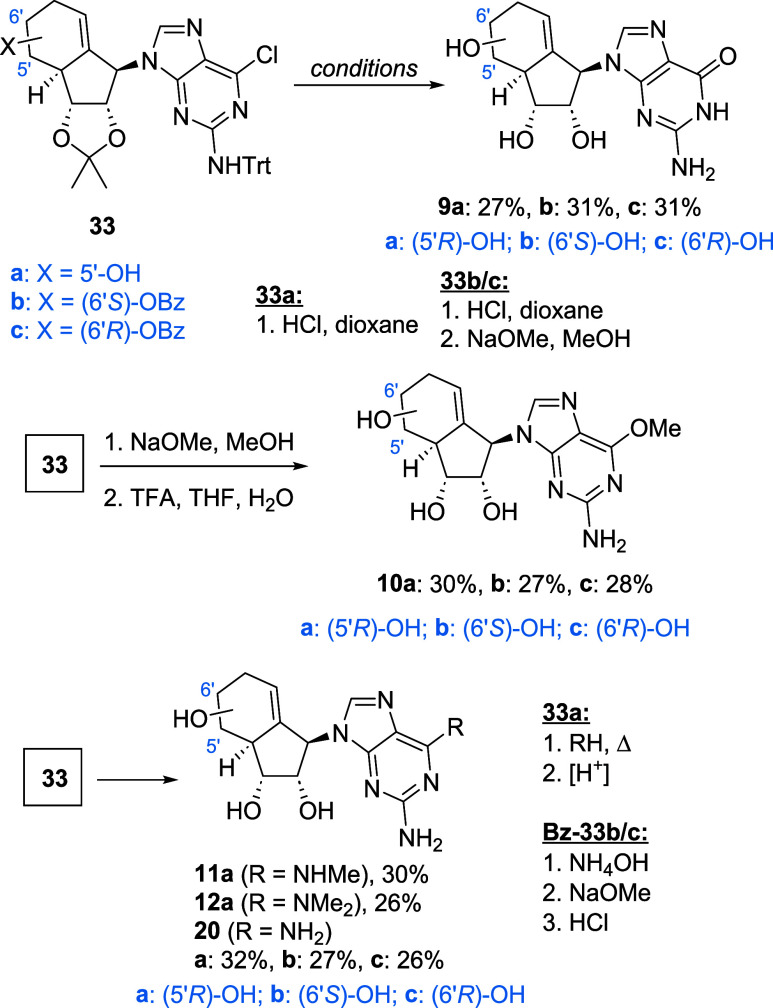
Synthesis of Guanosine-Type Analogues

Furthermore, synthesis of 2-amino-6-methoxypurine analogues **10a**–**c** was established by treatment of **33a**–**c** with sodium methoxide, followed by TFA-mediated deprotection. Like previously observed with adenosine analogues, treatment of compounds **33a**–**c** with various amines to replace the chlorine at position 6 yielded the 2,6-diamino purine analogues **11a**, **12a**, and **20a**–**c**, a divergent substitution pattern.

### Synthesis of Pyrimidine-Type Analogues

We expected that cytidine and NHC-type analogues could be synthesized from uracil intermediate in a short late-stage functionalization strategy (Scheme 5). We previously described the necessary uracil-type intermediates like **38** in our pilot study. Using this synthetic approach, key intermediate **38** was synthesized in gram scale.^13^ Transformation of uracil into cytosine required the protection of the free hydroxyl group in pseudo C5′/6′ position, so we use benzoyl (for **34b/c**) or silyl (for **34a**) protecting groups. For the transformation of uracil into cytidine, the enolized uracil was quenched with (iPr)_3_-benzenesulfonyl chloride **35**. The resulting sulfonate is a good leaving group, which can be substituted by amines. Therefore, treatment with various amines yielded cytidines **4a** and **17a** after acidic deprotection. For analogues **4b**–**c** and **17b**–**c**, an additional saponification was required to remove the benzoyl group.

**Scheme 5:**
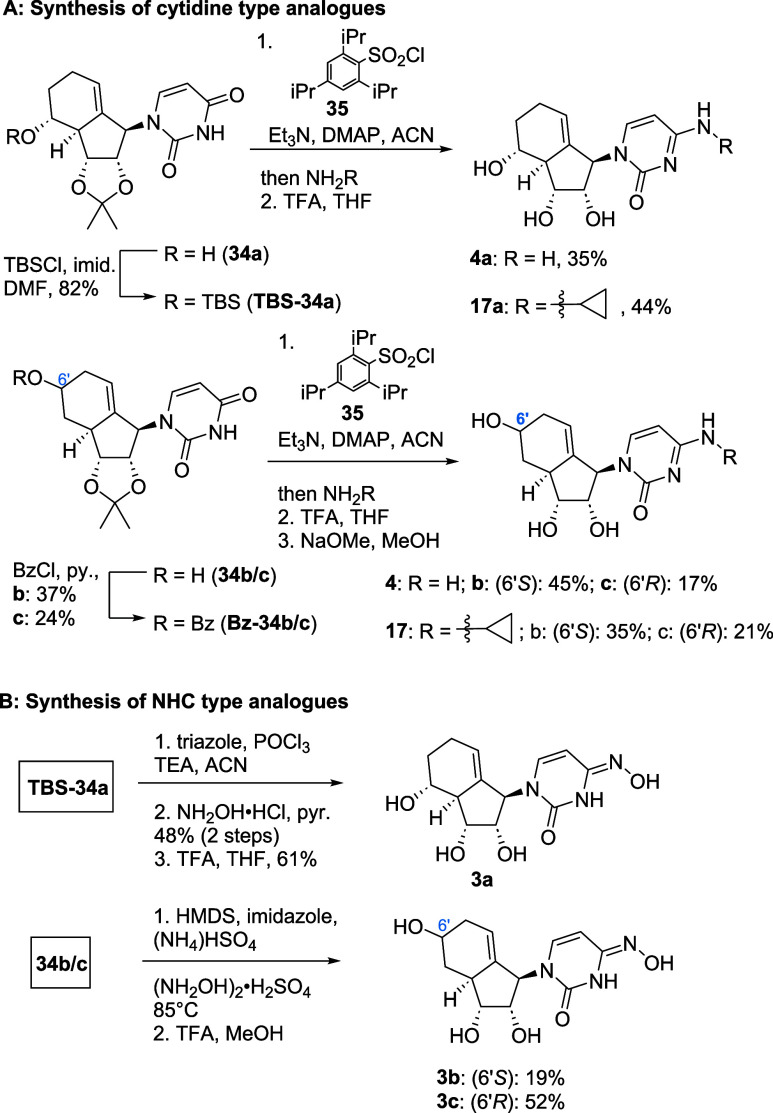
Synthesis of (A)-Cytidine-Type and (B) NHC-Type Analogues

For NHC-type analogue **3a** (Scheme 5B), a similar strategy as for the synthesis of cytidine **4a** was applied, using triazole as the leaving group, instead of triisopropylphenylsulfonate.^39^ To access compounds **3b**–**c**, C5′-unprotected **34b**–**c** were used as starting material, yielding, after acetonide deprotection, the NHC analogues directly by a recently developed method utilizing HMDS as a silyl source for *in situ* protection and enolization.^40^

Pyrimidine analogues carrying a halogen at position 5 were prepared as shown in Scheme 6. Early attempts to brominate the uracil moiety of compound **2a** using NBS yielded compound **13a** in only 2% yield, despite promising precedents in literature for ribose-type NA.^41^ We, therefore, did not examine the synthesis of other 5-halogen analogues such as 5-chloro uracil with NCS as chlorination source.

**Scheme 6:**
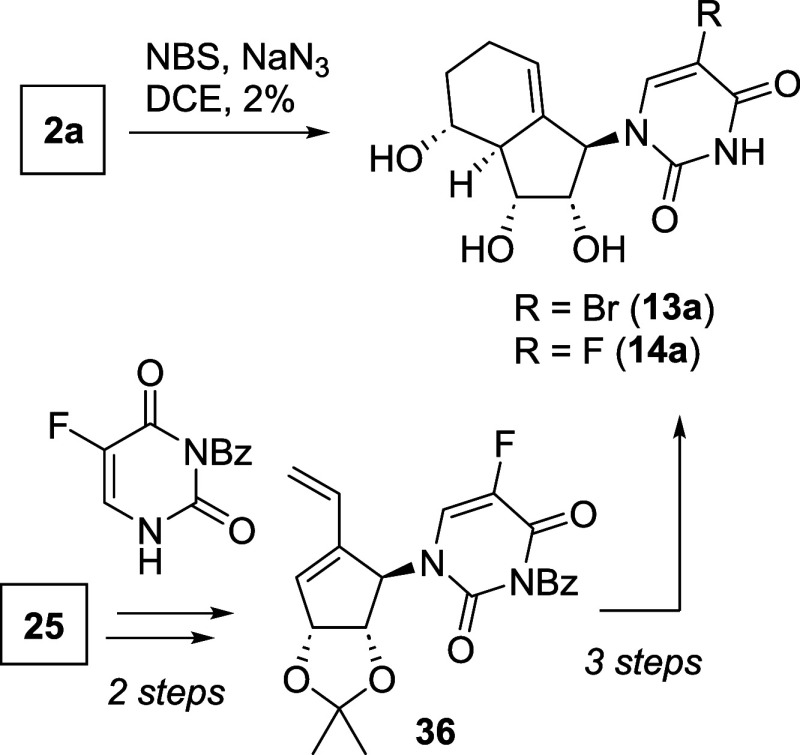
Synthesis of 5-Halogen Uracil Analogues

On the contrary, 5-fluorouracil-type analogues may show promising biological activity as the parent compound 5-FU is a marketed anticancer drug.^16^ As up to now, no method (except with the usage of F_2_) is known to insert fluoride in a late-stage modification, we decided to synthesize **14a** directly from **25** over **36** in 5 steps (see the Experimental Section for full synthesis route).

### Late-Stage Modification Led to the Diversification of the Carbobicyclic Scaffold

The Diels–Alder reaction yields two major isomers, namely the (5′*R*) and the (6′*S*) isomers, while from the minor isomers only (6′*R*) could be isolated in usable yields. To complete the set of four stereoisomers, we synthesized (5′*S*) by late-stage functionalization (Scheme 7). This was accomplished by coupling with benzoic acid (or its derivative) under Mitsunobu conditions with concomitant inversion of the 5′ stereocenter. For the purine analogues, the precursors **37–39** (for the synthesis, see the Experimental Section) were epimerized and deprotection delivered the (5′*S*)-purine analogues **5d**, **6d**, and **23d** (Scheme 7A). Furthermore, Barton-McCombie deoxygenation of **37–39** delivered analogues **5e, 6e, 8e**, and **23e** after deprotection in low, but usable, yields in a three-step procedure.

**Scheme 7:**
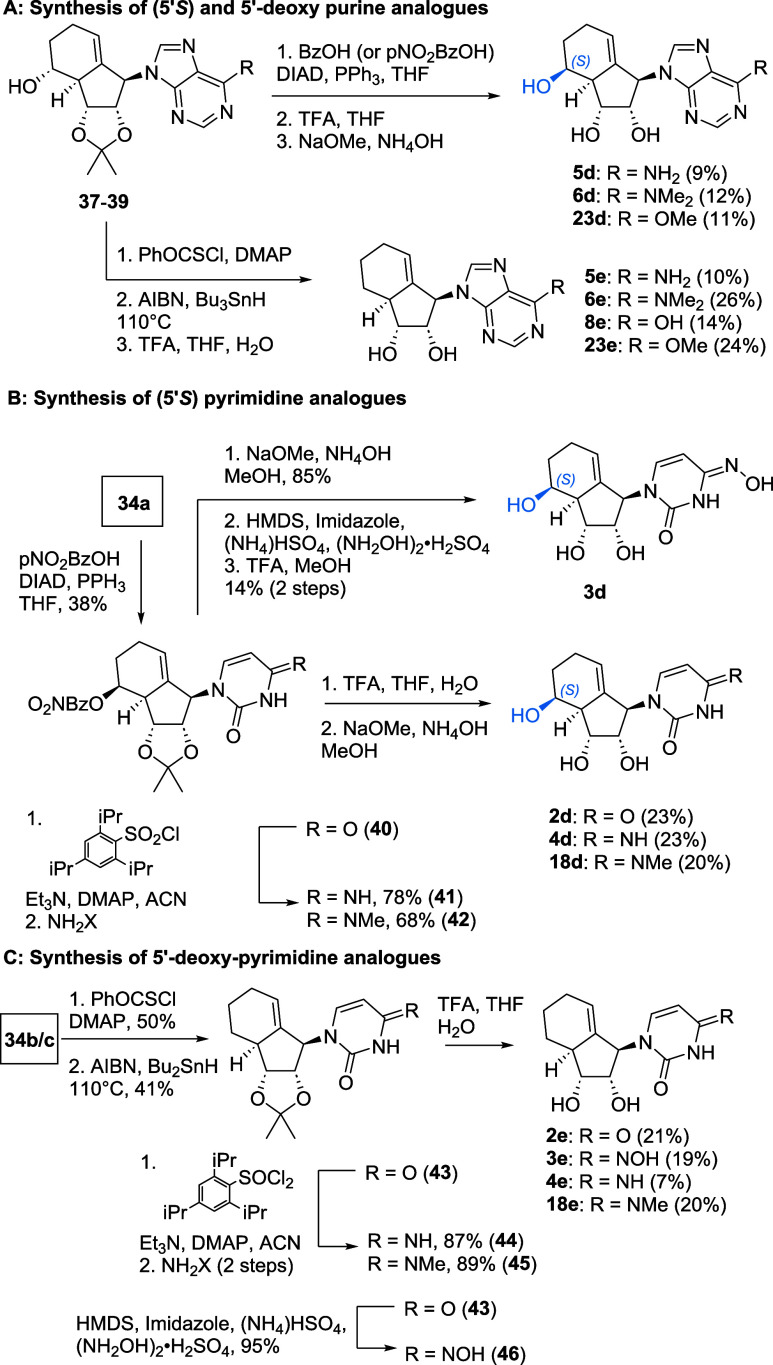
Late-Stage Modification for Epimerization and Dehydroxylation at C5′–OH^a^

a Synthesis of (A) 5′-modified purine analogues; (B) 5′S pyrimidine analogues; and (C) 5′-deoxy-pyrimidine analogues.

For the synthesis of the (5′*S*)-pyrimidine analogues, we used our established routes to synthesize uracil and cytidine analogues (Scheme 7B). In detail, (5′*R*) uracil intermediate **34a** was epimerized using Mitsunobu conditions yielding key intermediate **40** (5′*S*). Then, late-stage transformation of the nucleobase was conducted, furnishing the (5′*S*) analogues **2d**, **4d**, and **18d**. Finally, deoxygenation of **34a** yielded key intermediate **43**. With this intermediate in hand, previously established methods gave intermediates **44–46**, which were deprotected yielding the deoxygenated analogues **2–4e** and **18e** (Scheme 7C).

Aside from modifications at C5′, deoxygenation at C2′ provides 2′-deoxy analogues. Furthermore, manipulation at C3′ could potentially induce biological activity through blocking the elongation of viral RNA/DNA. However, common C2′/3′-modification strategies proved to be challenging. In comparison to reported ribose modifications, reactions using the carbobicyclic scaffold were often sluggish or did not proceed at all. Purine analogues were first selectively protected albeit in low yield (Scheme 8). Using TBSCl/pyridine afforded the C2′,5′–OH-protected silyl ethers **47** and **48**. Fluorination of the hydroxyl group at C-3′ with DAST^42,43^ gave, after acidic deprotection, the analogues **5g** and **6g**, but only in disappointing yields. Any other strategies to leverage the free hydroxyl at C3′ such as deoxygenation, oxidation, or alkylation failed presumably due to steric restraints.

**Scheme 8:**
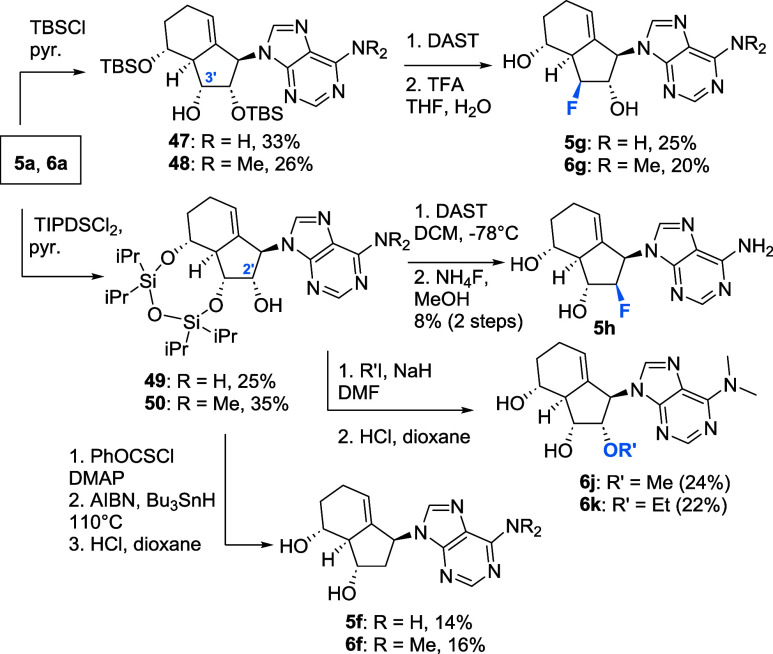
Late-Stage C2′/3 Modification Strategies for Purine-Type Analogues

When **5a** and **6a** were protected with bidental disiloxane affording **49** and **50**, the hydroxyl moiety at C2′ was subsequently modified. DAST-mediated fluorination followed by deprotection gave analogue **5h**. Alkylation of compound **50** yielded, after acidic deprotection, analogues **6j** and **6k**. The 2′-deoxy analogues **5f** and **5g** were obtained from **49** and **50** by deoxygenation followed by acidic cleavage of the TIPS protective group. Again, any other modification at C2′, such as oxidation, was not successful.

The pyrimidine analogues were prepared in an analogous way (Scheme 9). The uracil-type analogue **51** was protected to afford the C2′,5′-OTBS key intermediate **51**. In analogy to the previously described routes, the C3′-fluoro analogues **2g** and **4g** were accessible from **51** and **52**, respectively. With **51** in hand, we could successfully methylate C3′–OH; however, in an unselective fashion, yielding the C3′OH, N3-dimethyl analogue **2i**. For the synthesis of 2′-deoxy-carbobicyclic nucleoside analogues, C3′,5′–OH protection was conducted using a bidental silyl ether. Subsequent deoxygenation yielded the uracil intermediate **53**, which can be deprotected to 2′-deoxy analogue **2f**. In a similar fashion to previous attempts, 2′-deoxy analogue NHC analogue **3f**, cytidine analogue **4f** and **18f** were accessible as shown in Scheme 9.

**Scheme 9:**
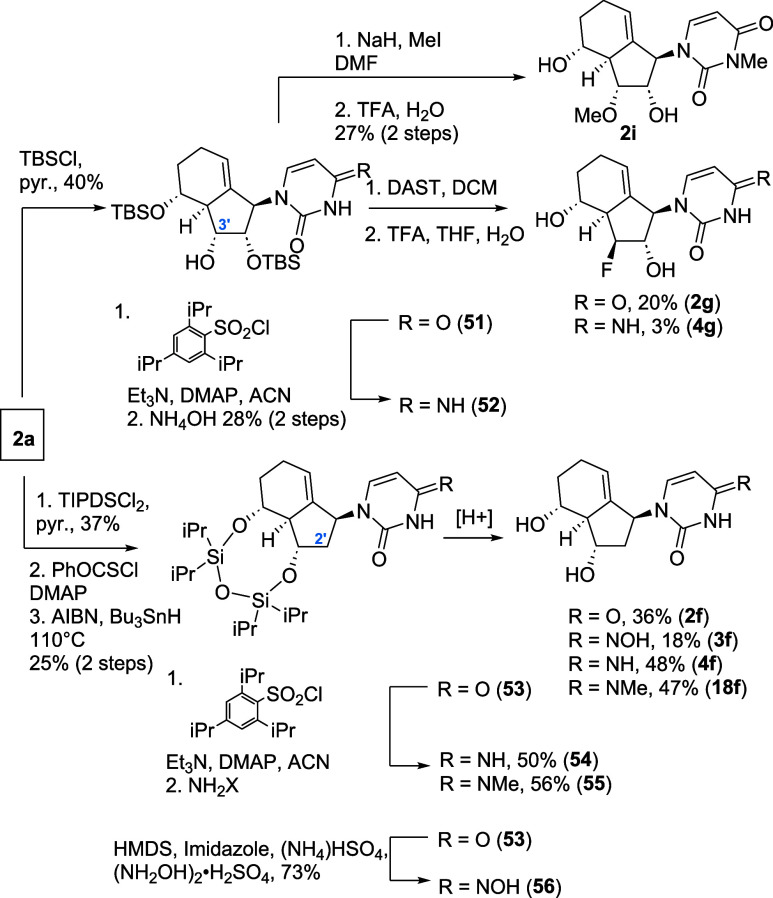
Late-Stage C2′/3 Modification Strategies for Pyrimidine-Type Analogues

In summary, carbobicyclic nucleoside analogues can typically be synthesized in fewer than ten steps using readily available compounds. The nucleobases attached to these structures can be modified similarly to those in known ribose-coupled compounds. However, substitutions and modifications at the pseudo C2′ and C3′ positions present significant challenges, resulting in only a limited number of analogues being produced through established methods. Importantly, despite these challenges, only two key intermediates—uracil and adenosine types—are required for the majority of analogues. Specifically, 28 pyrimidine analogues were synthesized from the advanced uracil-type intermediate **38**, while 35 purine analogues can be derived from the same precursor **32**. This highlights the efficacy of this synthetic strategy and demonstrates the potential of this synthetic route as a foundational platform for developing new nucleoside analogues.

## Conclusion

In summary, we have introduced a novel carbobicyclic nucleoside scaffold, a structural alternative to traditional ribose architecture as a promising antiviral chemotype. Our key findings are (1) a robust and divergent synthetic platform enables the rapid access to an NA drug library; (2) analogue **2a** exhibit significant activity against HCV, HSV, and IAV (including SAR data); (3) a minigenome assay confirms that the mechanism of **2a** involves direct or indirect disruption of viral IAV polymerase; (4) *in silico* modeling hinting toward an inhibition by locking of the catalytic center; (5) the scaffold is efficiently phosphorylated at pseudo C5′–OH at its congested secondary alcohol; (6) the resulting triphosphate demonstrates no off-target effects on human DNA/RNA polymerases.

Furthermore, the results demonstrate that the carbocyclic scaffold has the potential to significantly alter the biological profile of the attached nucleobase when compared to its ribose-type counterpart. While the uridine-based analogue **2a** exhibited antiviral activity, analogues featuring known active nucleobases, such as the NHC template from molnupiravir, were inactive. This divergence underlines the influence of the new scaffold on the antiviral activity. At this early stage, the moderate antiviral efficacy of our initial hits is anticipated, as future SAR optimization will be crucial for enhancing efficacy and advancing these compounds toward therapeutic relevance. *In silico* modeling suggests that the triphosphate group of the activated nucleoside analogue is pointing outward, not allowing for incorporation into viral RNA. Based on this model, hydroxylation at pseudo C7′ and/or manipulation of the C8′–C9′ double bond are promising entries for modification. Studies about the synthesis, antiviral activity, and metabolism of such analogues are underway and will be presented elsewhere.

Viral pathogens with pandemic potential pose a constant threat to public health. Therefore, a structurally diverse arsenal of compounds capable of acting as antivirals is essential for the development of drugs in emergency situations. The carbobicyclic scaffold, with its rapid synthetic access, can serve as a complementary strategy to ribose-type analogues, making it an ideal chemotype for new drug discovery projects.

## Experimental Section

### Antiviral Assessment

#### Antiviral Screening as in Figure 3 and Table S2

##### Virus Strains and Infection Protocols

The following virus strains (cell lines) were used in for the antiviral assay: Hepatitis C virus (HCV), replicon 1b (Huh7); Zika virus (ZIKV), PRVABC59 (Huh7); Herpes simplex virus type 1 (HSV-1), GHSV-UL46 (Vero); Human parainfluenza virus 3 (HPIV-3), C243 (LLC-MK2); EV71 (Shenzhen/120F1/09) (RD); Coxsackievirus (CV), A16 (RD); Human rhinovirus (HRV), 1B (H1 HeLa).

##### Cytopathic Effect (CPE) Assays

In microwell plates, cells were seeded and cultured at 37 °C and 5% CO_2_ overnight. The medium in each well was replenished with medium containing diluted compounds and virus. The resulting cultures were kept under 33–37 °C and 5% CO_2_ for an additional 2–5 days (depending on the protocol for the virus). Cytotoxicity of the compounds was assessed under the same conditions, but without virus infection, in parallel. Cell viability was measured by CCK-8 or CellTiter Glo following the manufacturer’s instructions.

##### HCV Replicon Assay

Diluted compounds were added into the 96-well plates. Then, HCV GT1b replicon cells were seeded at a density of 8000 cells per well and cultured at 37 °C and 5% CO_2_ for 3 days. The cell viability was determined with the CellTiter-Fluor, and the antiviral activity was determined by monitoring replicon reporter firefly luciferase using Bright-Glo following the manufacturer’s manual.

The antiviral activity of the test compounds was benchmarked against established reference drugs, namely BMS-790052, GS-7977, acyclovir, ribavirin, AG7088, and pleconaril.

#### Antiviral Screening as in Table S3

##### Host Cell Culture Conditions

The following host cell lines were employed for antiviral assays: HEL 299 (human embryonic lung fibroblasts, ATCC CCL-137), HEp-2 (epidermoid carcinoma of the larynx, ATCC CCL-23), U-87 (human glioblastoma, ATCC HTB-14), and MDCK (Madin-Darby canine kidney cells, kindly provided by M. Matrosovich, Marburg, Germany).

All cell lines were cultured in Dulbecco’s Modified Eagle Medium (DMEM) (Gibco Life Technologies, Merelbeke, Belgium), enriched with 8% heat-inactivated fetal bovine serum (HyClone, GE Healthcare Life Sciences, Diegem, Belgium), 0.075% sodium bicarbonate, and 1 mM sodium pyruvate (both from Gibco Life Technologies). Cultures were maintained at 37 °C in a humidified atmosphere containing 5% CO_2_.

##### Virus Strains and Infection Protocols

Antiviral efficacy was evaluated against a panel of viruses using cell-specific infection models:

On HEL 299: Herpes simplex virus type 1 (HSV-1 KOS), human coronaviruses HCoV-229E and HCoV-OC43, on HEp-2: Respiratory syncytial virus A (RSV-A), on U-87: Yellow fever virus, Zika virus, Sindbis virus, and Semliki Forest virus, and on MDCK: Influenza A/H1N1 (A/Ned/378/05), A/H3N2 (A/HK/7/87), and influenza B (B/Ned/537/05).

On the day of infection, the culture medium was removed and replaced with serial dilutions of the test compounds. For influenza viruses, OptiPRO serum-free medium was used, while DMEM supplemented with 4% FBS was applied for all other viruses. Viral inocula were adjusted to deliver 100 CCID_50_ (50% cell culture infectious dose) per well. Parallel mock-infected controls treated with the compounds alone were included to assess cytotoxicity. Following 3 to 7 days of incubation, virus-induced cytopathic effects were quantified using the MTS-based CellTiter 96 AQueous One Solution Cell Proliferation Assay (Promega, Leiden, The Netherlands). This colorimetric assay measures cell viability via formazan production. The 50% effective concentration (EC_50_) was calculated based on the inhibition of virus-induced CPE, while the 50% cytotoxic concentration (CC_50_) was determined from mock-infected cultures.

The antiviral activity of the test compounds was benchmarked against established reference drugs, namely remdesivir, chloroquine, ribavirin, zanamivir, rimantadine, and brivudin.

#### Antiviral Activity against IAV and RSV as in Figures 3–5 as well as Tables 1 and 2, and S4

##### Cells

A549 cells (ATCC, CCL-185) were maintained in Dulbecco’s modified Eagle’s medium (DMEM; Life Technologies, Gaithersburg, MD, USA) supplemented with 10% fetal bovine serum (FBS; Life Technologies) and 1% penicillin-streptomycin (P/S; Life Technologies).

MDCK cells (ATCC, CCL-34) and HEp-2 (ATCC, CCL-23) were maintained in minimum essential medium (MEM; Life Technologies) and Eagle’s minimum essential medium (EMEM), respectively, supplemented with 10% FBS and 1% P/S.

##### Virus Strains

Influenza A virus strains of a clinical isolate (H1N1pdm) and A/Oklahoma/447/2008 (H1N1) were used. Viral stocks were aliquoted and titrated to determine the 50% tissue culture infectious dose (TCID_50_) in MDCK cells. Clinical isolate of RSV-A2 long strain was a kind gift from Professor Paul Kay Sheung Chan, the Chinese University of Hong Kong.

##### Virus Propagation

MDCK cells were plated in T175 flasks. At 80–90% confluency, cells were washed with phosphate-buffered saline (PBS) and inoculated with influenza A virus for 1 h at 37 °C. Following adsorption, virus growth medium containing 2 μg/mL TPCK-treated trypsin was added. Cells were incubated for 48 h until >70% of cells displayed cytopathic effect (CPE). RSV was propagated similarly in HEp-2 cells, with virus propagation medium replaced as EMEM with 2% FBS. Supernatants were harvested, clarified by centrifugation, aliquoted, and stored at −80 °C.

##### Virus Titration

MDCK (for influenza) and HEp-2 (for RSV) cells were seeded into 96-well plates 1 day before titration. After washing with PBS, virus samples were serially diluted in half-log_10_ increments and added in quadruplicate to the plates. The highest dilution producing CPE was recorded. TCID_50_ values were calculated using the Spearman–Karber method.

##### Activity Assays

MDCK cells were seeded at 2 × 10^4^ cells/well in 96-well plates. Cells were infected at a multiplicity of infection (MOI) of 0.1 and simultaneously incubated at 37 °C with MEM containing 2 μg/mL TPCK-trypsin and test compounds at a final concentration of 40 μM. Compounds were initially dissolved in DMSO (Santa Cruz Biotechnology) at 16 mM and serially diluted in MEM containing 0.5% DMSO. A 0.5% DMSO vehicle control was included. Supernatants were collected at 48 h postinfection and stored at −80 °C for viral quantification.

##### Assessment of Activity/Viability with Cell Counting Kit-8

Cell viability was assessed using the Cell Counting Kit-8 (CCK-8; MedChemExpress, HY-K0301) after 48 h of incubation with virus and compounds. Absorbance was measured at 450 nm with a 690 nm reference using a Synergy HTX Multimode Microplate Reader (BioTek Instruments, Winooski, VT, USA).

Antiviral activity was calculated as the percentage protection relative to infected and uninfected controls. Cell viability was expressed as a percentage relative to uninfected controls.

#### *Ex Vivo* Studies as in Figure 5C

Fresh biopsies of human lung parenchyma from patients undergoing surgical resection of lung tissue as part of clinical care but surplus for routine diagnostic requirements were used in this study. The lung tissue was cut into multiple 2–3 mm snippets and were infected with the respective influenza A viruses within 2 h of collection. The lung snippets were inoculated with viruses at a titer of 1 × 10^6^ TCID_50_/mL at 37 °C. After 1 h, the unattached virus was removed by PBS washing. The mock- and virus-infected lung snippets were incubated in 500 μL of F12K with 100 units/mL penicillin and 100 μg/mL streptomycin at 37 °C. Viral yield in the cell-free supernatant was assessed at 24, 48, and 72 hpi by titration in quadruplicate in MDCK cells.

##### Isolation, Culture, and Differentiation of Human Nasal Epithelial Cells

Nasal epithelial cells were obtained from bilateral flocked nasal swabs of consenting donors. Swabs were placed in 5 mL of L15 medium and nasal cells were dislodged by flushing the swab ≥20 times. Cells were plated onto human collagen IV-coated 6-well plates and cultured in B/D expansion medium (see Table S7 in the SI) supplemented with 5 μM DAPT to promote monolayer growth. Medium was replaced every 2 days until confluence.

##### Air–Liquid Interface (ALI) Culture

At confluence, 2 × 10^5^ nasal epithelial cells were seeded onto the apical chamber of a 24-well Transwell insert (Corning 3470) coated with 30 μg/mL PureCol. Cells were first cultured under submerged conditions until a tight monolayer formed. The apical medium was then removed to initiate ALI culture. Cells were differentiated for 18 days using ALI-Diff medium (see Table S8 in the SI), refreshed twice weekly, prior to organoid differentiation.

##### Nasal Organoid Differentiation

After 18 days of ALI culture, cells were detached using TrypLE (Gibco), resuspended in Matrigel, and cultured in AO medium (see Table S9 in the SI) supplemented with 10% R-spondin-1 conditioned medium, 25 ng/mL FGF7, and 100 ng/mL FGF10 for 3D expansion. Organoids were passaged on day 5 into 12-well Transwell inserts and further differentiated for 4–6 weeks in AO medium containing 5 ng/mL FGF7 and 20 ng/mL FGF10. Organoids were considered fully differentiated once they reached ≥ 200 μm in diameter

#### Treatment of Organoids with Compounds to Treat Influenza A Infection as in Figure 5D

Fully differentiated organoids were dissociated into single cells and infected with influenza A virus at MOI = 0.01 for 2 h at 37 °C. DMEM was used for mock-infected controls. Following infection, organoids were washed with PBS and re-embedded in Matrigel containing AO medium. Supernatants were collected at 2, 24, 96, and 144 hpi and stored at −80 °C for viral titration.

##### qPCR Analysis

A549 cells were seeded at 1 × 10^5^ cells/well in 6-well plates and infected at MOI = 2 for 1 h. After infection, the inoculum was removed, cells were washed with PBS, and cultured in DMEM containing 0.2 μg/mL TPCK-trypsin and 40 μM of compound. Total RNA was extracted using the MiniBEST Universal RNA Extraction Kit (TaKaRa, 9767).

Reverse transcription was performed using PrimeScript RT Reagent Kit with the following conditions: 37 °C for 15 min, 85 °C for 5 s, then 4 °C hold. The Influenza M gene was quantified via real-time PCR using PowerUp SYBR Green Master Mix for qPCR on an ABI QuantStudio 12K system. Primer sequences are listed in Table S10 in the SI.

#### Minigenome IAV Assay

The pcDNA3-WSN-PB1, PB2, PA, and NP genes were used to express the recombinant IAV polymerase in HEK-293T cells. The reporter gene plasmid pPol-I-NS-Luc (pBZ81A36) contains the negative-sense influenza vRNA-like firefly luciferase gene flanked by human Pol I and IAV polymerase 3′promoter and 5′terminator. The pCMV-Gaussia luciferase plasmid, producing secreted Gaussia luciferase, was added as a transfection control. A total of 4 × 10^4^ cells in 88 μL culture medium were seeded in each well of a 96-well plate 1 day before transfection. For each well, 100 ng of the plasmid mix was prepared in a ratio of PB1: PB2: PA: NP: NS-Luc: Gaussia = 1:1:1:2:1:1 in 10 μL Opti-MEM Reduced Serum Medium (ThermoFisher, 31985062) and mixed with 400 ng PEI MAX (Polysciences, 24765) according to the transfection protocol. After 1 h of transfection, a series of 5-fold dilutions of the test compounds and control, ranging from 400 μM to 0 μM, were added to the medium in a total volume of 2 μL. After 48 h, 50 μL of the medium was removed for the Gaussia luciferase assay, and the remaining 50 μL of cells was lysed to measure luciferase activity, following the protocol from the Dual Luciferase Reporter Gene Assay Kit (Beyotime RG089). The relative inhibitory activity was calculated as the firefly luciferase intensity divided by the Gaussia luciferase intensity and normalized to the DMSO-only group (100%). Data points were then fitted to a Hill equation to determine the IC_50_ values.

### Metabolism Studies

#### Determination of Phosphorylated Metabolites in Hep-2 Cells

Hep-2 cells were seeded at 0.4 × 10^6^ cells/well in a 6-well plate. The next day, the cells were treated with either 10 μM **1a**, **ProTide-1a**, or **2a**. After 24 h of treatment, cells were collected, washed twice, and normalized to 0.5 × 106 cells in 500 mL of PBS. Cells were lysed with 3 cycles of freeze–thaw with liquid nitrogen and water bath. The lysate solution was centrifuged at 14,000 rpm for 15 min. Supernatant was collected and 10 μL was injected for LC-MS/MS analysis. Standard curves were prepared by spiking various concentrations into blank Hep-2 matrix (0.5 × 10^6^ cells per condition). Standard curves were prepared by spiking various concentrations of pure samples (**1a**, **ProTide-1a**, **2a**, **MP-1a**, **TP-1a**, **TP-2a**) into blank HEp-2 matrix (0.5 × 106 cells in 500 μL of PBS). For LC-MS parameters, see the Supporting Information.

### Polymerase Inhibition Assays

#### Human DNA Polymerases Inhibition Assays

Human DNA polymerases α, β, and γ assay kits were obtained from ProFoldin (Hudson, MA), and the assays were conducted as per the manufacturer’s protocol. In brief, 100× DNA template and 100× dNTP mix were diluted to working concentration with water, and 100× enzyme was diluted with 1× buffer to working concentration. The total volume of each reaction mixture was 30 mL, consisting of 17 mL of water, 3 mL of 10× DNA template, 3 mL of 10× buffer, 3 mL of 10× enzyme, 3 mL of dNTP mix, and 1 mL of test compound or control. The reaction was incubated in a 384-well plate (black, flat-bottom, medium-binding) at 37 °C for 30 min. At the end of the reaction, 30 mL of 1× fluorescence dye was added to the reaction mixture and was further incubated for 5 min. Fluorescence intensity was measured at 535 nm (ex. 485 nm) using CLARIOstrar PLUS microplate reader (BMG Labtech).

#### Human RNA Polymerases Radiolabeling Assay

The related sequences were ordered from IDT. The RNA9 oligo was labeled with [γ-^32^P]-ATP by T4 PNK at 37 °C for 2 h and heat-inactivated. A total of 7 pmol TDS was annealed with an equal molar amount of RNA9 from 42 to 20 °C over 30 min to form the transcription bubble, before 20 pmol polymerase was added to the mixture at room temperature and incubated for 10 min. Finally, 21 pmol NDS was added at 37 °C for 10 min to form the complete elongation complex. The reaction mixture was then aliquoted and preincubated with different concentrations of TP-1a, ranging from 0 μM, 2 μM, 10 μM, 25 μM, to 50 μM. After 30 min, each aliquot was supplemented with 50 μM rNTP to start transcription. One aliquot was mixed with 50 μM rNTP and 50 mM EDTA to serve as a negative control. The reactions were performed at 37 °C for 5 min and then quenched with an equal volume of 2× stop buffer (50 mM Tris-HCl pH 8, 100 mM EDTA, 8 M Urea, 20% Glycerol). The mixture was heated to 65 °C and subjected to a 20% denaturing PAGE with 8 M Urea. The gel was then exposed to a phosphor screen (Azure Biosystems) for 16 h and scanned by the Sapphire Biomolecular Imager (Azure Biosystems).

### Synthesis of Carbobicyclic Nucleoside Analogues

#### General Methods

All reagents and solvents (MeOH, DCM, THF, NMP, MeCN, pyridine) were purchased in the highest purity available from commercial suppliers (such as Sigma-Aldrich, Meryer Chemicals, Bidepharm, Energy, Dieckmann Chemical HK, and TCI). Solvents were dried over molecular sieve before usage, and reactions were performed under N_2_ or Ar atmosphere in oven-dried glassware using commonly described Schlenk techniques. Elevated reaction temperatures referring to the oil bath temperatures, whereas for cooling ice bath (0 °C) was used. The reaction progress can be monitored by TLC using silica gel precoated aluminum sheets (Machery Nagel “DC Fertigfolien ALUGRAM SIL G/UV 254; 0.20 mm Schichtdicke, Kieselgel 60 mit Fluoreszenz-Indikator UV 254”). The compounds could be visualized by UV light and staining with a solution of CAM with subsequent heating. All NMR spectra were recorded on a Bruker spectrometer at the Chinese University of Hong Kong or at the facilities of o2h Limited (India). All other spectral data were obtained in the facilities of o2h limited (India). All final products are >95% pure, analyzed by HPLC column chromatography.

See Schemes S1–S8 in the Supporting Information for full synthetic details.

### CAUTION! There is a risk of explosion when experimenting with sealed tubes under elevated temperatures. Only use suitable glassware and safety shielding

#### Synthesis of Adenosine-Type Nucleoside Analogues

##### Synthesis of 6-Chloro-9-((3aS,4S,6aR)-5-iodo-2,2-dimethyl-3a,6a-dihydro-4*H*-cyclopenta­[*d*]­[1,3]­dioxol-4-yl)-9*H*-purine (**28**)

To a stirred solution of alcohol **25** (65.0 g, 0.23 mol, 1.0 equiv), 6-chloro-7*H*-purine **26** (35.6 g, 0.23 mol, 1.0 equiv), and PPh_3_ (121 g, 0.46 mol, 2.0 equiv) in anhydrous THF (10 L) was dropwise added DIAD (89.8 mL, 0.46 mol, 2.0 equiv) at 0 °C under N_2_ atmosphere. The resulting reaction mixture was stirred at room temperature for 16 h. Due to difficulties in separating the final product **28** and the byproduct (hydrazine dicarboxylate), the byproduct was further derivatized. Therefore, the reaction mixture was enriched with TEA (96.2 mL, 0.69 mol, 3.0 equiv), DMAP (2.80 g, 23.0 mmol, 0.1 equiv), and Boc anhydride (318 mL, 1.38 mmol, 6.0 equiv) at 0 °C. The resulting reaction mixture was stirred at room temperature for another 16 h. After completion of the reaction, the reaction mixture was concentrated under reduced pressure, diluted with water (2 L), and extracted with ethyl acetate (3 × 200 mL). The combined organic phase was washed with brine (1 L), dried over Na_2_SO_4_, filtered, and concentrated to give the crude. Another reaction of the same batch size was performed simultaneously and was combined during workup. Purification by column chromatography on silica gel (20% EA/hex) afforded the title compound **28** as a yellow sticky solid (95.0 g, 227 mmol, 49%).

**R**_**f**_ = 0.3 (30% EA/hex); ^**1**^ **H NMR** (600 MHz, CDCl_3_) δ [ppm] = 8.73 (s, 1H), 8.07 (s, 1H), 6.68 (s, 1H), 5.55–5.48 (m, 2H), 4.93 (d, *J* = 6.4 Hz, 1H), 1.52 (s, 3H), 1.37 (s, 3H); ^**13**^ **C­{** ^**1**^ **H}-NMR** (150 MHz, CDCl_3_) δ [ppm] = 152.4, 151.7, 151.2, 146.5, 144.5, 132.4, 113.2, 95.5, 85.6, 82.2, 74.0, 27.4, 26.0; **HRMS** (ESI, 3.5 kV) *m*/*z*: [M + H]^+^ calc for C_13_H_12_ClIN_4_O_2_H^+^ 418.9766, found 418.9769.

##### Synthesis of 6-Chloro-9-((3aS,4R,6aR)-2,2-dimethyl-5-vinyl-3a,6a-dihydro-4*H*-cyclopenta­[*d*]­[1,3]­dioxol-4-yl)-9*H*-purine (**30**)

To a stirred solution of iodide **28** (95.0 g, 227 mmol, 1.0 equiv), Ph_3_As (6.92 g, 22.7 mmol, 0.1 equiv), Pd­(PhCN)_2_Cl_2_ (4.35 g, 11.3 mmol, 0.05 equiv), and CuI (4.18 g, 22.7 mmol, 0.1 equiv) in anhydrous NMP (1.90 L) was added dropwise vinyl tributyltin (108 g, 339 mmol, 1.5 equiv) at room temperature under N_2_ atmosphere. The resulting reaction mixture was stirred at room temperature for 2 h. After completion of the reaction, the reaction mixture was diluted with water (2 L) and extracted with ethyl acetate (3 × 300 mL). The combined organic phase was washed with brine (3 L), dried over Na_2_SO_4_, filtered, and concentrated to give the crude, which was purified by column chromatography on silica gel (23% EA/hex) to afford the title compound **30** as a yellowish solid (61.0 g, 191.3 mmol, 84%). **R**_**f**_ = 0.25 (30% EA/hex); ^**1**^ **H NMR** (600 MHz, CDCl_3_) δ [ppm] = 8.81 (s, 1H), 7.88 (s, 1H), 6.49 (dd, *J* = 11.6, 17.8 Hz, 1H), 6.28 (s, 1H), 5.90 (s, 1H), 5.50 (d, *J* = 5.2 Hz, 1H), 5.19 (d, *J* = 11.2 Hz, 1H), 4.98 (d, *J* = 17.3 Hz, 1H), 4.65 (d, *J* = 5.6 Hz, 1H), 1.48 (s, 3H), 1.35 (s, 3H). ^**13**^ **C­{** ^**1**^ **H}-NMR** (150 MHz, CDCl_3_) δ [ppm] = 152.3, 151.4, 151.3, 143.2, 139.0, 136.1, 131.9, 129.7, 119.9, 112.4, 84.5, 83.5, 63.6, 27.4, 25.8; **HRMS** (ESI, 3.5 kV) *m*/*z*: [M + H]^+^ calc for C_15_H_15_ClN_4_O_2_ 319.0956, found 319.0956.

##### Synthesis of (3aR,3bR,4R,8R,8aS)-8-(6-Chloro-9*H*-purin-9-yl)-2,2-dimethyl-3a,3b,5,6,8,8a-hexahydro-4*H*-indeno­[1,2-*d*]­[1,3]­dioxol-4-ol (**32a**)

To a 250 mL autoclave charged with diene **30** (10.0 g, 31.4 mol, 1.0 equiv) in toluene (100 mL) were added BHT (0.69 g, 3.13 mmol, 0.1 equiv) and 4,4,5,5-tetramethyl-2-vinyl-1,3,2-dioxaborolane (16 g, 93.9 mmol, 3.0 equiv) at room temperature. An autoclave was packed and heated to 140 °C for 3 days. After completion of the reaction, the reaction mixture was concentrated to give the crude. The obtained crude was dissolved in a mixture of THF (100 mL) and ∼7.0 pH buffer solution (100 mL) at room temperature. To this reaction mixture, NaBO_3_ × 4 H_2_O (13 g, 125 mmol, 4.0 eqiv) was added portion-wise at room temperature (exothermicity observed). The resulting reaction was stirred at room temperature for 1 h. After completion of the reaction, the reaction mixture was quenched with a saturated solution of Na_2_S_2_O_3_ (500 mL) and was stirred at room temperature until it was clear. The organic layer was separated, and the aqueous phase was extracted with ethyl acetate (3 × 300 mL). The combined organic phase was dried over Na_2_SO_4_, filtered, and concentrated to give the crude. Another 5 reactions of the same batch size were performed and were combined during workup. Purification by column chromatography on silica gel (2% MeOH/DCM) afforded **32a** as a pure compound (8.2 g, 22.6 mmol, 12%, **Rf**_**1**_ = 0.3 (5% MeOH/DCM)) and **32b/c** as a mixture of isomers (17 g, 46.9 mmol, 25%, **Rf**_**2**_ = 0.25 (5% MeOH/DCM)). **32a**: ^**1**^ **H NMR** (100 MHz, CDCl_3_) δ [ppm] = 8.75 (s, 1H), 8.15 (s, 1H), 5.50 (brs, 1H), 5.05 (dd, *J* = 5.2, 7.2 Hz, 1H), 4.93 (s, 1H), 4.81 (dd, *J* = 5.6, 7.2 Hz, 1H), 3.82–3.76 (m, 1H), 2.72 (s, 1H), 2.16–2.10 (m, 2H), 2.07–2.00 (m, 2H), 1.67 (m, 1H), 1.66 (s, 3H), 1.37 (s, 3H); ^**13**^ **C­{** ^**1**^ **H}-NMR** (400 MHz, CDCl_3_) δ [ppm] = 152.0, 151.6, 151.3, 144.6, 136.4, 131.8, 121.8, 114.3, 83.6, 82.5, 71.5, 63.5, 52.5, 31.1, 27.4, 25.1, 24.8; **HRMS** (ESI, 3.5 kV) *m*/*z*: [M + H]^+^ calc for C_17_H_19_ClN_4_O_3_H^+^ 363.1218, found 363.1214. **LCMS** *m*/*z* 363.3 (M + H) (ESI +ve), RT = 1.71 min. **HPLC** RT = 5.87 min, 100%.

##### Synthesis of (3aR,3bS,5S,8R,8aS)-8-(6-Chloro-9*H*-purin-9-yl)-2,2-dimethyl-3a,3b,5,6,8,8a-hexahydro-4*H*-indeno­[1,2-*d*]­[1,3]­dioxol-5-yl Benzoate (**Bz-32b**)

To a stirred solution of isomeric mixture **32b/c** (7.0 g, 19.3 mmol, 1.0 equiv) in DCM (70 mL), was added TEA (9.69 g, 96.5 mmol, 5.0 equiv) and benzoyl chloride (5.39 g, 38.6 mmol, 2.0 equiv) followed by the addition of DMAP (0.23 g, 1.92 mmol, 0.1 equiv) at 0 °C. The resulting reaction mixture was stirred at room temperature for 2 h. After completion of the reaction, the reaction mixture was diluted with water (500 mL) and extracted with ethyl acetate (3 × 150 mL). The combined organic phase was dried over Na_2_SO_4_, filtered, and concentrated to give the crude, which was purified by column chromatography on silica gel (38% EA/hex) to afford benzoate **Bz-32b** as a white solid (5.1 g, 10.9 mmol, 57%). **R**_**f**_ = 0.6 (50% EA/hex); ^**1**^ **H NMR** (400 MHz, CDCl_3_) δ [ppm] = 8.76 (s, 1H), 8.18 (s, 1H), 8.05 (d, *J* = 7.6 Hz, 2H), 7.59 (t, *J* = 7.2 Hz, 1H), 7.46 (dd, *J* = 7.6, 7.6 Hz, 2H), 5.51 (brs, 1H), 5.32–5.29 (m, 1H), 5.09 (dd, *J* = 6.4, 6.4 Hz, 1H), 4.98 (brs, 1H), 4.64 (dd, *J* = 6.0, 6.0 Hz, 1H), 2.98 (brs, 1H), 2.70–2.67 (m, 1H), 2.58–2.54 (m, 1H) 2.20–2.12 (m, 1H), 1.76 (dd, *J* = 11.6, 23.2 Hz, 1H), 1.62 (s, 3H), 1.35 (s, 3H); ^**13**^ **C­{** ^**1**^ **H}-NMR** (100 MHz, CDCl_3_) δ [ppm] = 165.9, 152.0, 151.5, 151.4, 144.7, 138.7, 133.09, 131.9, 130.1, 129.5, 128.3, 119.8, 114.0, 83.8, 83.1, 69.7, 62.9, 44.3, 32.4, 30.2, 27.5, 25.2. **HRMS** (ESI, 3.5 kV) *m*/*z*: [M + H]^+^ calc for C_24_H_23_ClN_4_O_4_H^+^ 467.1481, found 467.1475; **LCMS** *m*/*z* 467.2 (M + H) (ESI +ve), RT = 2.41 min; **HPLC** RT = 9.22 min, 100%.

##### Synthesis of (3aR,3bS,5R,8R,8aS)-8-(6-Chloro-9*H*-purin-9-yl)-2,2-dimethyl-3a,3b,5,6,8,8a-hexahydro-4*H*-indeno­[1,2-*d*]­[1,3]­dioxol-5-yl Benzoate (**Bz-32c**)

To a stirred solution of isomeric mixture **32b/c** (7.0 g, 19.3 mmol, 1.0 equiv) and benzoic acid (2.82 g, 23.1 mmol, 1.2 equiv) in THF (70 mL) was added PPh_3_ (10.1 g, 38.6 mmol, 2.0 equiv) followed by dropwise addition of DIAD (7.7 g, 38.6 mmol, 2.0 equiv) at 0 °C under N_2_ atmosphere. The resulting reaction mixture was stirred at room temperature for 2 h. After completion of the reaction, the reaction mixture was diluted with water (500 mL) and extracted with ethyl acetate (3 × 150 mL). The combined organic phase was dried over Na_2_SO_4_, filtered, and concentrated to give the crude, which was purified by column chromatography on silica gel (42% EA/hex) to benzoate **Bz-32c** as a white solid (4.7 g, 10.1 mmol, 52%). **R**_**f**_ = 0.5 (50% EA/hex); ^**1**^ **H NMR** (400 MHz, CDCl_3_) δ [ppm] = 8.77 (s, 1H), 8.19 (s, 1H), 8.04 (d, *J* = 7.6 Hz, 2H), 7.60 (t, *J* = 7.6 Hz, 1H), 7.48 (dd, *J* = 7.6, 7.6 Hz, 2H), 5.60 (brs, 1H), 5.54 (brs, 1H), 5.12 (dd, *J* = 6.8, 6.8 Hz, 1H), 4.96 (brs, 1H), 4.64 (dd, *J* = 6.8, 6.8 Hz, 1H), 3.00 (brs, 1H), 2.73–2.69 (m, 1H), 2.44–2.31 (m, 2H), 2.30 (d, *J* = 19.4 Hz, 1H), 1.69 (ddd, *J* = 2.3, 12.0, 12.0 Hz, 1H), 1.63 (s, 3H), 1.37 (s, 3H). ^**13**^ **C­{** ^**1**^ **H}-NMR** (100 MHz, CDCl_3_) δ [ppm] = 165.9, 152.0, 151.6, 151.4, 144.7, 138.5, 133.1, 131.9, 130.2, 129.6, 128.4, 118.6, 114.1, 83.6, 83.5, 67.5, 63.6, 39.4, 30.7, 29.8, 27.4, 25.1; **HRMS** (ESI, 3.5 kV) *m*/*z*: [M + H]^+^ calc for C_24_H_23_ClN_4_O_4_H^+^ 467.1481, found 467.1483; **LCMS** *m*/*z* 467.2 (M + H) (ESI +ve), RT = 2.34 min; **HPLC** RT = 8.82 min, 100%.

##### Synthesis of (1R,2S,3R,7R,7aR)-3-(6-Amino-9*H*-purin-9-yl)-2,3,5,6,7,7a-hexahydro-1*H*-indene-1,2,7-triol (**5a**)

To a stirred solution of acetonide **32a** (0.25 g, 0.68 mmol, 1.0 equiv) in a mixture of THF (20 mL) and water (20 mL) was added TFA (1.0 mL) at room temperature. The resulting reaction mixture was then stirred at room temperature for 16 h. After completion of the reaction, the reaction mixture was concentrated under reduced pressure to afford the crude as a brown oil (0.3 g). A 100 mL sealed tube was charged with the crude (0.3 g, 0.92 mmol, 1.0 equiv) in THF (2.0 mL) was added NH_4_OH (4.0 mL, 25% in H_2_O) at room temperature. The resulting reaction mixture was heated to 120 °C for 16 h. After completion of the reaction, the reaction mixture was concentrated under reduced pressure to give the crude, which was purified by reversed-phase chromatography (3% MeCN in water). The obtained pure fractions were lyophilized to afford the title compound **5a** as a white solid (57.0 mg, 0.18 mmol, 23% from **32a**). **R**_**f**_ = 0.2 (10% MeOH/DCM, 1% NH_4_OH); ^**1**^ **H NMR** (400 MHz, MeOD) δ [ppm] = 8.19 (s, 1H), 8.17 (s, 1H), 5.37–5.35 (m, 1H), 4.95–4.93 (m, 1H), 4.38 (dd, *J* = 6.0, 7.6 Hz, 1H), 4.24 (dd, *J* = 4.0, 6.0 Hz, 1H), 3.69–3.63 (m, 1H), 2.55–2.53 (m, 1H), 2.20–2.15 (m, 2H), 2.00–1.95 (m, 1H), 1.65–1.64 (m, 1H); ^**13**^ **C­{** ^**1**^ **H}-NMR** (100 MHz, MeOD) δ [ppm] = 155.9, 152.2, 149.7, 140.4, 134.7, 120.0, 118.7, 74.7, 71.9, 70.3, 62.3, 52.5, 31.0, 24.5; **HRMS** (ESI, 3.5 kV) *m*/*z*: [M + H]^+^ calc for C_14_H_17_N_5_O_3_H^+^ 304.1404, found 304.1403; **LCMS** *m*/*z* 304.2 (M + H) (ESI +ve), RT = 1.98 min; **HPLC** RT = 4.37 min, 98.5%.

##### Synthesis of (1R,2S,3R,6S,7aS)-3-(6-Amino-9*H*-purin-9-yl)-2,3,5,6,7,7a-hexahydro-1*H*-indene-1,2,6-triol (**5b**)

A 100 mL sealed tube was charged with **32b** (0.3 g, 0.82 mmol, 1.0 equiv) in THF (3.0 mL) was added NH_4_OH (3.0 mL, 25% in H_2_O) at room temperature. The resulting reaction mixture was heated to 120 °C for 16 h. After completion of the reaction, the reaction mixture was concentrated under reduced pressure to afford the crude as a yellow solid (0.3 g). To a stirred solution of the crude (0.3 g) in a mixture of THF (3 mL) and water (3 mL) was added TFA (0.8 mL) at room temperature. The resulting reaction mixture was then stirred at room temperature for 16 h. After completion of the reaction, the reaction mixture was concentrated, neutralized using NH_4_OH (pH ∼ 8.0), and concentrated to give the crude, which was purified by reversed-phase chromatography using 0.1% NH_4_OH in water (3% MeCN/Water). The obtained pure fractions were lyophilized to afford **5b** as a white solid (58 mg, 0.19 mmol, 23% from **32b**). **R**_**f**_ = 0.1 (10% MeOH/DCM, 1% NH_4_OH); ^**1**^ **H NMR** (400 MHz, MeOD) δ [ppm] = 8.20 (s, 1H), 8.14 (s, 1H), 5.30 (brs, 1H), 5.08–5.06 (m, 1H), 4.30 (dd, *J* = 5.6, 5.6 Hz, 1H), 4.06 (dd, *J* = 6.0, 7.6 Hz, 1H), 3.97–3.91 (m, 1H), 2.74 (m, 1H), 2.40–2.36 (m, 2H), 1.95–1.87 (m, 1H), 1.43 (dd, *J* = 11.6, 22.8 Hz, 1H); ^**13**^ **C­{** ^**1**^ **H}-NMR** (100 MHz, DMSO) δ [ppm] = 155.8, 152.2, 149.5, 140.5, 137.1, 120.0, 118.7, 75.9, 74.9, 66.8, 62.5, 44.0, 35.8, 33.9; **HRMS** (ESI, 3.5 kV) *m*/*z*: [M + H]^+^ calc for C_14_H_17_N_5_O_3_H^+^ 304.1404, found 304.1403; **LCMS** *m*/*z* 304.2 (M + H) (ESI +ve), RT = 1.87 min; **HPLC** RT = 3.97 min, 97.8%.

##### Synthesis of (1R,2S,3R,6R,7aS)-3-(6-Amino-9*H*-purin-9-yl)-2,3,5,6,7,7a-hexahydro-1*H*-indene-1,2,6-triol (**5c**)

A 100 mL sealed tube was charged with **Bz-32c** (0.30 g, 0.64 mmol, 1.0 equiv) in EtOH (3.0 mL) and NH_4_OH (3.0 mL, 25% in H_2_O) at room temperature. The resulting reaction mixture was heated to 120 °C for 16 h. After completion of the reaction, the reaction mixture was concentrated under reduced pressure to afford the crude as a yellow solid (0.30 g). To a stirred solution of the crude (0.30 g) in a mixture of THF (3 mL) and water (3 mL) was added TFA (0.8 mL) at room temperature. The resulting reaction mixture was then stirred at room temperature for 16 h. After completion of the reaction, the reaction mixture was concentrated under reduced pressure to afford the crude as a brown oil (0.30 g). To a stirred solution of the crude (0.30 g) in methanol (6 mL) was added NH_4_OH (2.0 mL, 25% in H_2_O), NaOMe (0.15 g, 2.94 mmol) and K_2_CO_3_ (0.2 g, 1.47 mmol) at room temperature. The resulting reaction mixture was stirred at room temperature for 16 h. After completion of the reaction, the reaction mixture was concentrated under reduced pressure to give the crude, which was purified by reversed-phase chromatography using 0.1% NH_4_OH in water (100% water). The enriched crude was further purified by RP Prep HPLC using 0.05% NH_4_OH in water. The obtained pure fractions were lyophilized to afford the title compound as a white solid (63 mg, 0.20 mmol, 32% from **Bz-32c**). **R**_**f**_ = 0.1 (10% MeOH/DCM, 1% NH_4_OH); ^**1**^ **H NMR** (400 MHz, MeOD) δ [ppm] = 8.19 (s, 1H), 8.14 (s, 1H), 5.36 (brs, 1H), 5.05–5.03 (m, 1H), 4.32 (dd, *J* = 4.8, 5.6 Hz,1H), 4.20 (brs, 1H), 4.06 (dd, *J* = 6.0, 7.6 Hz, 1H), 2.85 (brs, 1H), 2.34–2.27 (m, 2H), 2.10–2.05 (m, 1H), 1.54–1.47 (m, 1H); ^**13**^ **C­{** ^**1**^ **H}-NMR** (100 MHz, DMSO) δ [ppm] = 155.8, 152.2, 149.5, 140.6, 136.8, 118.7, 118.7, 75.5, 75.3, 63.7, 62.9, 38.1, 32.8, 32.5; **HRMS** (ESI, 3.5 kV) *m*/*z*: [M + H]^+^ calc for C_14_H_17_N_5_O_3_H^+^ 304.1404, found 304.1425; **LCMS** *m*/*z* 304.4 (M + H) (ESI +ve), RT = 4.13 min; **HPLC** RT = 3.87 min, 97.73%.

##### Synthesis of (3aR,3bR,4R,8R,8aS)-8-(6-(Dimethylamino)-9*H*-purin-9-yl)-2,2-dimethyl-3a,3b,5,6,8,8a-hexahydro-4*H*-indeno­[1,2-*d*]­[1,3]­dioxol-4-ol (**38**)

To a stirred solution of **32a** (0.30 g, 0.82 mmol, 1.0 equiv) in ethanol (3.0 mL) and water (1 mL) was added dimethylamine (2 M in THF, 3.0 mL) at room temperature. The resulting reaction mixture was heated to 90 °C for 16 h. After completion of the reaction, the reaction mixture was concentrated under reduced pressure to afford the crude as a brown solid (0.30 g, 99%). **Rf** = 0.4 (5% MeOH/DCM). **LCMS** *m*/*z* 372.4 (M + H) (ESI +ve), RT = 1.58 min. Note: The obtained material was directly used in the next step without purification.

##### Synthesis of (1R,2S,3R,7R,7aR)-3-(6-(Dimethylamino)-9*H*-purin-9-yl)-2,3,5,6,7,7a-hexahydro-1*H*-indene-1,2,7-triol (**6a**)

To a stirred solution of **38** (0.30 g, 0.81 mmol) in a mixture of THF (3 mL) and water (3 mL) was added TFA (0.8 mL) at room temperature. The resulting reaction mixture was then stirred at room temperature for 16 h. After completion of the reaction, the reaction mixture was concentrated under reduced pressure to give the crude, which was purified by reversed-phase chromatography using 0.1% NH_4_OH in water (6% MeCN/water). The obtained pure fractions were lyophilized to afford **6a** as a white solid (59 mg, 0.17 mmol, 22**%**). **R**_**f**_ = 0.35 (10% MeOH/DCM, 1% NH_4_OH); ^**1**^ **H NMR** (400 MHz, MeOD) δ [ppm] = 8.20 (s, 1H), 8.05 (s, 1H), 5.35 (d, *J* = 5.6 Hz, 1H), 4.89–4.85 (m, 1H), 4.36 (dd, *J* = 6.0, 7.6 Hz, 1H), 4.23–4.20 (dd, *J* = 4.4, 6.0 Hz, 1H), 3.67–3.62 (m, 1H), 3.53 (brs, 6H), 2.54 (brs, 1H), 2.19–2.17 (m, 2H), 1.97 (brs, 1H), 1.61 (brs, 1H); ^**13**^ **C­{** ^**1**^ **H}-NMR** (100 MHz, MeOD) δ [ppm] = 154.7, 151.5, 150.5, 138.4, 134.9, 119.8, 119.6, 74.7, 72.0, 70.3, 61.9, 52.5, 37.6, 31.0, 24.5; **HRMS** (ESI, 3.5 kV) *m*/*z*: [M + H]^+^ calc for C_16_H_21_N_5_O_3_H^+^ 332.1717, found 332.1716; **LCMS** *m*/*z* 332.3 (M + H) (ESI +ve) RT = 1.42 min; **HPLC** RT = 3.67 min, 98.80%.

##### Synthesis of (1R,2S,3R,6S,7aS)-3-(6-(Dimethylamino)-9*H*-purin-9-yl)-2,3,5,6,7,7a-hexahydro-1*H*-indene-1,2,6-triol (**6b**)

To a stirred solution of **Bz-32b** (0.30 g, 0.64 mmol, 1.0 equiv) in ethanol (3.0 mL) and water (2 mL) was added dimethylamine (2 M in THF, 3. 0 mL) at room temperature. The resulting reaction mixture was heated to 85 °C for 16 h. After completion of the reaction, the reaction mixture was concentrated under reduced pressure to afford the crude as a yellow solid (0.3 g). To a stirred solution of the crude (0.3 g) in a mixture of THF (3 mL) and water (3 mL) was added TFA (0.8 mL) at room temperature. The resulting reaction mixture was then stirred at room temperature for 16 h. After completion of the reaction, the reaction mixture was concentrated under reduced pressure to afford the crude as a brown oil (0.3 g). To a stirred solution of the crude (0.3 g) in methanol (6 mL) was added NaOMe (0.14 g, 2.75 mmol) and NH_4_OH (2 mL) at room temperature. The resulting reaction mixture was stirred at room temperature for 16 h. After completion of the reaction, the reaction mixture was concentrated under reduced pressure to give the crude, which was purified by reversed-phase chromatography using 0.1% NH_4_OH in water (12% MeCN/water). The enriched crude was further purified by RP Prep HPLC. The obtained pure fractions were lyophilized to afford **6b** as a white solid (62 mg, 0.18 mmol, 29% from **Bz-32b**). **R**_**f**_ = 0.3 (10% MeOH/DCM, 1% NH_4_OH); ^**1**^ **H NMR** (400 MHz, MeOD) δ [ppm] = 8.21 (s, 1H), 8.01 (s, 1H), 5.30 (brs, 1H), 5.06–5.04 (m, 1H), 4.27 (dd, *J* = 4.8, 6.0 Hz, 1H), 4.02 (dd, *J* = 6.0, 7.6 Hz, 1H), 3.97–3.90 (m, 1H), 3.53 (brs, 6H), 2.74 (brs, 1H), 2.40–2.35 (m, 2H), 1.94–1.87 (m, 1H), 1.43 (dd, *J* = 11.6, 22.8 Hz, 1H); ^**13**^ **C­{** ^**1**^ **H}-NMR** (100 MHz, DMSO) δ [ppm] = 154.7, 151.6, 150.2, 138.4, 137.2, 119.9, 119.6, 75.9, 75.0, 66.8, 62.1, 44.0, 37.6, 35.8, 33.9; **HRMS** (ESI, 3.5 kV) *m*/*z*: [M + H]^+^ calc for C_16_H_21_N_5_O_3_H^+^ 332.1717, found 332.1718; **LCMS** *m*/*z* 332.3 (M + H) (ESI +ve), RT = 2.05 min. **HPLC** RT = 6.09 min, 100%.

##### Synthesis of (1R,2S,3R,6R,7aS)-3-(6-(Dimethylamino)-9*H*-purin-9-yl)-2,3,5,6,7,7a-hexahydro-1*H*-indene-1,2,6-triol (**6c**)

To a stirred solution of **Bz-32c** (0.30 g, 0.64 mmol, 1.0 equiv) in ethanol (3.0 mL) and water (1.0 mL) was added dimethylamine (2 M in THF, 3.0 mL) at room temperature. The resulting reaction mixture was heated to 85 °C for 16 h. After completion of the reaction, the reaction mixture was concentrated under reduced pressure to afford the crude as a black solid (0.3 g). To a stirred solution of the crude (0.3 g) in a mixture of THF (3.0 mL) and water (3.0 mL) was added TFA (0.8 mL) at room temperature. The resulting reaction mixture was then stirred at room temperature for 16 h. After completion of the reaction, the reaction mixture was concentrated under reduced pressure to afford the crude as a brown oil. (0.3 g). To a stirred solution of the crude (0.3 g) in methanol (3.0 mL) was added NaOMe (0.14 g, 2.75 mmol) and NH_4_OH (2.0 mL) at room temperature. The resulting reaction mixture was stirred at room temperature for 16 h. After completion of the reaction, the reaction mixture was concentrated under reduced pressure to give the crude, which was purified by reversed-phase chromatography using 0.1% NH_4_OH in water (12% MeCN/water). The enriched crude was further purified by RP Prep HPLC. The obtained pure fractions were lyophilized to afford **6c** as a white solid (62 mg, 0.18 mmol, 29% from **Bz-32c**). **R**_**f**_ = 0.2 (10% MeOH/DCM, 1% NH_4_OH); ^**1**^ **H NMR** (400 MHz, MeOD) δ [ppm] = 8.20 (s, 1H), 8.01 (s, 1H), 5.36 (brs, 1H), 5.02 (m, 1H), 4.29 (dd, *J* = 5.6, 5.6 Hz, 1H), 4.20 (brs, 1H), 4.04 (dd, *J* = 6.0, 7.6 Hz, 1H), 3.53 (brs, 6H), 2.85 (brs, 1H), 2.34–2.27 (m, 2H), 2.10–2.05 (m, 1H), 1.53–1.46 (m, 1H); ^**13**^ **C­{** ^**1**^ **H}-NMR** (100 MHz, MeOD) δ [ppm] = 154.7, 151.5, 150.3, 138.5, 137.0, 119.6, 118.5, 75.5, 75.3, 63.7, 62.6, 38.1, 37.6, 32.8, 32.5; **HRMS** (ESI, 3.5 kV) *m*/*z*: [M + H]+ calc for C_16_H_21_N_5_O_3_H^+^ 332.1717, found 332.1718; **LCMS** *m*/*z* 332.3 (M + H) (ESI +ve), RT = 2.17 min; **HPLC** RT = 5.00 min, 100%.

##### Synthesis of (1R,2S,3R,7R,7aR)-3-(6-(Cyclopropylamino)-9*H*-purin-9-yl)-2,3,5,6,7,7a-hexahydro-1*H*-indene-1,2,7-triol (**7a**)

To a stirred solution of **32a** (0.30 g, 0.82 mmol, 1.0 equiv) in ethanol (3.0 mL) and water (2.0 mL) was added cyclopropylamine (3.0 mL) at room temperature. The resulting reaction mixture was heated to 90 °C for 16 h. After completion of the reaction, the reaction mixture was concentrated under reduced pressure to afford the crude as a yellow solid (0.3 g). To a stirred solution of the crude (0.3 g) in a mixture of THF (3 mL) and water (3.0 mL) was added TFA (0.8 mL) at room temperature. The resulting reaction mixture was then stirred at room temperature for 16 h. After completion of the reaction, the reaction mixture was concentrated, neutralized using NH_4_OH (pH ∼ 8.0), and concentrated to give the crude, which was purified by reversed-phase chromatography using 0.1% NH_4_OH in water (12% MeCN/water). The obtained pure fractions were lyophilized to afford **7a** as a white solid (56 mg, 0.16 mmol, 20% from **32a**). **R**_**f**_ = 0.35 (10% MeOH/DCM, 1% NH_4_OH); ^**1**^ **H NMR** (400 MHz, MeOD) δ [ppm] = 8.28 (brs, 1H), 8.12 (brs, 1H), 5.37–5.35 (m, 1H), 4.93–4.92 (m, 1H), 4.37 (dd, *J* = 6.4, 7.6 Hz, 1H), 4.24 (dd, *J* = 4.4, 6.0 Hz, 1H), 3.68–3.63 (m, 1H), 2.97 (brs, 1H), 2.55–2.53 (m, 1H), 2.19–1.17 (m, 2H), 2.00–1.97 (m, 1H), 1.61 (m, 1H), 0.94–0.89 (m, 2H), 0.68–0.64 (m, 2H); ^**13**^ **C­{** ^**1**^ **H}-NMR** (100 MHz, MeOD) δ [ppm] = 155.7, 152.1, 149.1, 140.1, 134.7, 119.9, 119.2, 74.7, 71.9, 70.3, 62.3, 52.5, 31.0, 24.5, 23.1, 6.2; **HRMS** (ESI, 3.5 kV) *m*/*z*: [M + H]^+^ calc for C_17_H_21_N_5_O_3_H^+^ 344.1717, found 344.1715; **LCMS** *m*/*z* 344.3 (M + H) (ESI +ve), RT = 1.38 min; **HPLC** RT = 3.41 min, 100%.

##### Synthesis of (1R,2S,3R,6S,7aS)-3-(6-(Cyclopropylamino)-9*H*-purin-9-yl)-2,3,5,6,7,7a-hexahydro-1*H*-indene-1,2,6-triol (**7b**)

To a stirred solution of **Bz-32b** (0.30 g, 0.64 mmol, 1.0 equiv) in ethanol (3.0 mL) and water (2.0 mL) was added cyclopropylamine (3.0 mL) at room temperature. The resulting reaction mixture was heated to 85 °C for 16 h. After completion of the reaction, the reaction mixture was concentrated under reduced pressure to afford the crude as a yellow solid (0.3 g). To a stirred solution of the crude (0.3 g) in a mixture of THF (3.0 mL) and water (3.0 mL) was added TFA (0.9 mL) at room temperature. The resulting reaction mixture was then stirred at room temperature for 16 h. After completion of the reaction, the reaction mixture was concentrated under reduced pressure to afford the crude as a brown oil (0.3 g). To a stirred solution of the crude (0.3 g) in methanol (6.0 mL) was added NaOMe (0.14 g, 2.68 mmol) and NH_4_OH (2.0 mL) at room temperature. The resulting reaction mixture was stirred at room temperature for 16 h. After completion of the reaction, the reaction mixture was concentrated under reduced pressure to give the crude, which was purified by reversed-phase chromatography using 0.1% NH_4_OH in water (13% MeCN/Water). The enriched crude was further purified by RP Prep HPLC. The obtained pure fractions were lyophilized to afford **7b** as a white solid (63 mg, 0.18 mmol, 29% from **Bz-32b**). **R**_**f**_ = 0.35 (10% MeOH/DCM, 1% NH_4_OH); ^**1**^ **H NMR** (400 MHz, MeOD) δ [ppm] = 8.29 (s, 1H), 8.10 (s, 1H), 5.30 (brs, 1H), 5.08 (brs, 1H), 4.30 (dd, *J* = 5.6, 5.6 Hz, 1H), 4.06 (dd, *J* = 6.4, 8.0 Hz, 1H), 3.98–3.90 (m, 1H), 2.97 (brs, 1H), 2.74 (brs, 1H), 2.40–2.36 (m, 2H), 1.93–1.87 (m, 1H), 1.43 (dd, *J* = 11.6, 22.8 Hz, 1H), 0.93–0.89 (m, 2H), 0.68–0.66 (m, 2H); ^**13**^ **C­{** ^**1**^ **H}-NMR** (100 MHz, MeOD) δ [ppm] = 155.7, 152.2, 148.7, 140.1, 137.1, 120.0, 119.2, 75.9, 74.9, 66.8, 62.4, 44.0, 35.8, 33.8, 23.1, 6.2; **HRMS** (ESI, 3.5 kV) *m*/*z*: [M + H]^+^ calc for C_17_H_21_N_5_O_3_H^+^ 344.1717, found 344.1713; **LCMS** *m*/*z* 344.3 (M + H) (ESI +ve), RT = 1.93 min; **HPLC** RT = 4.54 min, 100%.

##### Synthesis of (1R,2S,3R,6R,7aS)-3-(6-(Cyclopropylamino)-9*H*-purin-9-yl)-2,3,5,6,7,7a-hexahydro-1*H*-indene-1,2,6-triol (**7c**)

To a stirred solution of **Bz-32c** (0.30 g, 0.64 mmol, 1.0 equiv) in ethanol (3.0 mL) and water (1.0 mL) was added cyclopropylamine (3.0 mL) at room temperature. The resulting reaction mixture was heated to 85 °C for 16 h. After completion of the reaction, the reaction mixture was concentrated under reduced pressure to afford the crude as a yellow solid (0.3 g). To a stirred solution of the crude (0.3 g) in a mixture of THF (3.0 mL) and water (3.0 mL) was added TFA (0.8 mL) at room temperature. The resulting reaction mixture was then stirred at room temperature for 16 h. After completion of the reaction, the reaction mixture was concentrated under reduced pressure to afford the crude as a brown oil (0.3 g). To a stirred solution of the crude (0.3 g) in methanol (6.0 mL) was added NaOMe (0.14 g, 2.68 mmol) and NH_4_OH (2.0 mL) at room temperature. The resulting reaction mixture was stirred at room temperature for 16 h. After completion of the reaction, the reaction mixture was concentrated under reduced pressure to give the crude, which was purified by reversed-phase chromatography using 0.1% NH_4_OH in water (13% MeCN/Water). The enriched crude was further purified by RP Prep HPLC. The obtained pure fractions were lyophilized to afford the title compound **7c** as a white solid (60 mg, 0.17 mmol, 27% from **Bz-32c**). **R**_**f**_ = 0.23 (10% MeOH/DCM, 1% NH_4_OH); ^**1**^ **H NMR** (400 MHz, MeOD) δ [ppm] = 8.28 (s, 1H), 8.09 (s, 1H), 5.36 (brs, 1H), 5.02 (m, 1H), 4.32 (dd, *J* = 6.0, 6.0 Hz, 1H), 4.20 (brs, 1H), 4.07 (dd, *J* = 6.0, 7.6 Hz, 1H), 2.97 (brs, 1H), 2.85 (brs, 1H), 2.34–2.22 (m, 2H), 2.09–2.05 (m, 1H) 1.53–1.47 (m, 1H), 0.94–0.89 (m, 2H), 0.68–0.67 (m, 2H); ^**13**^ **C­{** ^**1**^ **H}-NMR** (100 MHz, MeOD) δ [ppm] = 155.7, 152.1, 148.8, 140.3, 136.9, 119.3, 118.5, 75.5, 75.3, 63.7, 62.9, 38.1, 32.8, 32.5, 23.1, 6.2; **HRMS** (ESI, 3.5 kV) *m*/*z*: [M + H]^+^ calc for C_17_H_21_N_5_O_3_H^+^ 344.1717, found 344.1738; **LCMS** *m*/*z* 344.3 (M + H) (ESI +ve), RT = 2.12 min; **HPLC** RT = 4.57 min, 100%.

##### Synthesis of (1R,2S,3R,7R,7aR)-3-(6-(Pyrrolidinyl)-9*H*-purin-9-yl)-2,3,5,6,7,7a-hexahydro-1*H*-indene-1,2,7-triol (**16a**)

To a stirred solution of **32a** (25 mg, 68.9 μmol, 1.0 equiv) in ACN (1.0 mL) was added pyrrolidine (24.5 mg, 0.34 mmol, 5.0 equiv) at room temperature. The resulting reaction mixture was then stirred at room temperature for 2 h. After completion of the reaction, NH4Cl sat. solution (1.0 mL) and EA (1.0 mL) were added. After phase separation, the aqueous phase was extracted with EA (3 × 2 mL). The combined organic phase was dried over MgSO_4_, and volatiles were removed *in vacuo*. The crude was redissolved in MeOH (1.0 mL) and 20% TFA in H_2_O was added (1.0 mL) at room temperature. The volatiles were removed *in vacuo* after 24 h. The crude was purified by reversed-phase chromatography using 0.1% NH_4_OH in water (5%–75% MeOH/Water). Analogue **16a** was isolated as a white solid (6.0 mg, 16.7 μmol, 24%). ^**1**^ **H NMR** (700 MHz, MeOD) δ [ppm] = 8.17 (s, 1H), 8.06 (s, 1H), 5.33–5.37 (m, 1H), 4.88–4.91 (m, 1H), 4.35 (dd, *J* = 6.0, 7.6 Hz, 1H), 4.21 (dd, *J* = 4.4, 6.0 Hz, 1H), 4.15 (brs, 2H), 3.72 (brs, 2H), 3.64 (ddd, *J* = 3.6, 9.3 11.8 Hz, 1H), 2.50–2.55 (m, 1H), 2.00–2.22 (m, 6H), 1.94–1.99 (m, 1H), 1.60 (dq, *J* = 7.8, 11.3 Hz, 1H); ^**13**^ **C­{** ^**1**^ **H}-NMR** (100 MHz, DMSO) δ [ppm] = 154.2, 153.3, 151.5, 140.4, 136.3, 121.2, 121.0, 76.1, 73.4, 71.7, 63.4, 54.0, 32.5, 26.0; **HRMS** (ESI, 3.5 kV) *m*/*z*: [M + Na]^+^ calc for C_18_H_23_N_5_O_3_Na^+^ 380.1693, found 380.1689; **HPLC** RT = 19.45 min, 96.61%. Note: Pyrrolidine signals could not be observed in ^13^C NMR presumably due to peak broadening.

##### Synthesis of (1R,2S,3R,7R,7aR)-3-(6-(Methylamino)-9*H*-purin-9-yl)-2,3,5,6,7,7a-hexahydro-1*H*-indene-1,2,7-triol (**19a**)

To a stirred solution of **32a** (0.30 g, 0.82 mmol, 1.0 equiv) in ethanol (3.0 mL) was added ∼40% MeNH_2_ in water (3.0 mL) at room temperature. The resulting reaction mixture was heated to 85 °C for 16 h. After completion of the reaction, the reaction mixture was concentrated under reduced pressure to afford the crude as a black gummy solid (0.3 g). To a stirred solution of the crude (0.3 g) in a mixture of THF (3.0 mL) and water (3.0 mL) was added TFA (0.6 mL) at room temperature. The resulting reaction mixture was then stirred at room temperature for 16 h. After completion of the reaction, the reaction mixture was concentrated, neutralized using NH_4_OH (pH ∼ 8.0), and concentrated to give the crude, which was purified by reversed-phase chromatography using 0.1% NH_4_OH in water (8% MeCN/Water). The obtained pure fractions were lyophilized to afford **19a** as a white solid (61 mg, 0.19 mmol, 23% from **32a**). **R**_**f**_ = 0.2 (10% MeOH/DCM, 1% NH_4_OH); ^**1**^ **H NMR** (400 MHz, MeOD) δ [ppm] = 8.28 (s, 1H), 8.19 (s, 1H), 5.38 (d, *J* = 4.0 Hz, 1H), 4.95 (s, 1H), 4.37 (dd, *J* = 7.2, 7.2 Hz, 1H), 4.24 (dd, *J* = 4.8, 4.8 Hz, 1H), 3.68–3.62 (m, 1H), 3.16 (brs, 3H), 2.54 (brs, 1H), 2.19 (brs, 2H), 2.00–1.97 (m, 1H), 1.64–1.59 (m, 1H); ^**13**^ **C­{** ^**1**^ **H}-NMR** (100 MHz, MeOD) δ [ppm] = 153.5, 149.5, 148.3, 140.8, 134.7, 120.1, 119.2, 74.9, 71.9, 70.2, 62.4, 52.5, 31.0, 26.7, 24.5; **HRMS** (ESI, 3.5 kV) *m*/*z*: [M + H]^+^ calc for C_15_H_19_N_5_O_3_H^+^ 318.1561, found 318.1560; **LCMS** *m*/*z* 318.2 (M + H) (ESI +ve), RT = 2.09 min; **HPLC** RT-4.09 min, 100%.

##### Synthesis of (1R,2S,3R,6S,7aS)-3-(6-(Methylamino)-9*H*-purin-9-yl)-2,3,5,6,7,7a-hexahydro-1*H*-indene-1,2,6-triol (**19b**)

To a stirred solution of **Bz-32b** (0.30 g, 0.64 mmol, 1.0 equiv) in ethanol (3.0 mL) was added ∼ 40% MeNH_2_ in water (3.0 mL) at room temperature. The resulting reaction mixture was heated to 90 °C for 16 h. After completion of the reaction, the reaction mixture was concentrated under reduced pressure to afford the crude as a yellow solid (0.3 g). To a stirred solution of the crude (0.3 g) in a mixture of THF (3.0 mL) and water (3.0 mL) was added TFA (0.8 mL) at room temperature. The resulting reaction mixture was then stirred at room temperature for 16 h. After completion of the reaction, the reaction mixture was concentrated under reduced pressure to afford the crude as a black solid (0.3 g). To a stirred solution of the crude (0.3 g) in methanol (6.0 mL) were added NaOMe (0.15 g, 2.84 mmol) and NH_4_OH (2.0 mL) at room temperature. The resulting reaction mixture was stirred at room temperature for 16 h. After completion of the reaction, the reaction mixture was concentrated under reduced pressure to give the crude, which was purified by reversed-phase chromatography using 0.1% NH_4_OH in water (15% MeCN/Water). The enriched crude was further purified by RP Prep HPLC. The obtained pure fractions were lyophilized to afford **20b** as a white solid (62 mg, 0.19 mmol, 30% from **Bz-32b**). **R**_**f**_ = 0.15 (10% MeOH/DCM, 1% NH_4_OH); ^**1**^ **H NMR** (400 MHz, MeOD) δ [ppm] = 8.25 (brs, 1H), 8.07 (s, 1H), 5.29 (brs, 1H), 5.07–5.05 (m, 1H), 4.29 (dd, *J* = 6.0, 6.0 Hz, 1H), 4.07–4.03 (dd, *J* = 6.4, 8.0 Hz, 1H), 3.96–3.91 (m, 1H), 3.13 (brs, 3H), 2.74 (m, 1H), 2.40–2.35 (m, 2H), 1.94–1.87 (m, 1H), 1.43 (dd, *J* = 11.6, 22.8 Hz, 1H); ^**13**^ **C­{** ^**1**^ **H}-NMR** (100 MHz, MeOD) δ [ppm] = 155.3, 152.3, 148.4 (broad), 139.8, 137.1, 120.0, 119.2, 75.9, 74.9, 66.8, 62.4, 44.0, 35.8, 33.9, 26.4 (broad); **HRMS** (ESI, 3.5 kV) *m*/*z*: [M + H]^+^ calc for C_15_H_19_N_5_O_3_H^+^ 318.1561, found 318.1566; **LCMS** *m*/*z* 318.2 (M + H) (ESI +ve), RT = 1.84 min; **HPLC** RT = 4.29 min, 100%.

##### Synthesis of (1R,2S,3R,6R,7aS)-3-(6-(Methylamino)-9*H*-purin-9-yl)-2,3,5,6,7,7a-hexahydro-1*H*-indene-1,2,6-triol (**19c**)

To a stirred solution of **Bz-32c** (0.30 g, 0.64 mmol, 1.0 equiv) in ethanol (3.0 mL) was added ∼ 40% MeNH_2_ in water (3.0 mL) at room temperature. The resulting reaction mixture was heated to 90 °C for 16 h. After completion of the reaction, the reaction mixture was concentrated under reduced pressure to afford the crude as a yellow solid (0.3 g). To a stirred solution of the crude (0.3 g) in a mixture of THF (3.0 mL) and water (3.0 mL) was added TFA (0.8 mL) at room temperature. The resulting reaction mixture was then stirred at room temperature for 16 h. After completion of the reaction, the reaction mixture was concentrated under reduced pressure to afford the crude as a brown oil (0.3 g). To a stirred solution of the crude (0.3 g) in methanol (3.0 mL) were added NaOMe (0.15 g, 2.84 mmol) and NH_4_OH (2 mL) at room temperature. The resulting reaction mixture was stirred at room temperature for 16 h. After completion of the reaction, the reaction mixture was concentrated under reduced pressure to give the crude, which was purified by reversed-phase chromatography using 0.1% NH_4_OH in water (12% MeCN/Water). The enriched crude was further purified by RP Prep HPLC. The obtained pure fractions were lyophilized to afford **20c** as a white solid (62 mg, 0.19 mmol, 30% from **Bz-32c**). **R**_**f**_ = 0.2 (10% MeOH/DCM, 1% NH_4_OH); ^**1**^ **H NMR** (400 MHz, MeOD) δ [ppm] = 8.25 (brs, 1H), 8.08 (s, 1H), 5.35 (brs, 1H), 5.02 (m, 1H), 4.32 (dd, *J* = 5.6, 5.6 Hz, 1H), 4.20 (brs, 1H), 4.05 (dd, *J* = 6.0, 7.6 Hz, 1H), 3.13 (brs, 3H), 2.85 (m, 1H), 2.34–2.27 (m, 2H), 2.10–2.05 (m, 1H), 1.53–1.47 (m, 1H); ^**13**^ **C­{** ^**1**^ **H}-NMR** (100 MHz, MeOD) δ [ppm] = 155.3, 152.2, 148.5 (br), 139.9, 136.9, 119.2, 118.5, 75.5, 75.2, 63.7, 62.9, 38.1, 32.8, 32.5, 26.3; **HRMS** (ESI, 3.5 kV) *m*/*z*: [M + H]^+^ calc for C_15_H_19_N_5_O_3_H^+^ 318.1561, found 318.1559; **LCMS** *m*/*z* 318.2 (M + H) (ESI +ve), RT = 2.22 min; **HPLC** RT = 4.31 min, 100%.

##### Synthesis of (1R,2S,3R,7R,7aR)-3-(6-(Cyclohexylamino)-9*H*-purin-9-yl)-2,3,5,6,7,7a-hexahydro-1*H*-indene-1,2,7-triol (**21a**)

To a stirred solution of **32a** (0.30 g, 0.82 mmol, 1.0 equiv) in ethanol (3.0 mL) and water (2.0 mL) was added cyclohexylamine (3.0 mL) at room temperature. The resulting reaction mixture was heated to 90 °C for 16 h. After completion of the reaction, the reaction mixture was concentrated under reduced pressure to afford the crude as a yellow solid (0.3 g). To a stirred solution of the crude (0.3 g) in a mixture of THF (3.0 mL) and water (2.0 mL) was added TFA (0.8 mL) at room temperature. The resulting reaction mixture was then stirred at room temperature for 16 h. After completion of the reaction, the reaction mixture was concentrated, neutralized using NH_4_OH (pH ∼ 8.0), and concentrated to give the crude, which was purified by reversed-phase chromatography using 0.1% NH_4_OH in water (12% MeCN/Water). The obtained pure fractions were lyophilized to afford **21a** as a white solid (63 mg, 0.16 mmol, 20% from **32a**). **R**_**f**_ = 0.35 (10% MeOH/DCM, 1% NH_4_OH); ^**1**^ **H NMR** (400 MHz, MeOD) δ [ppm] = 8.21 (s, 1H), 8.11 (s, 1H), 5.35–5.33 (m, 1H), 4.89 (m, 1H), 4.36 (dd, *J* = 6.4, 7.6 Hz, 1H), 4.23 (dd, *J* = 4.4, 5.6 Hz, 1H), 4.20–4.09 (brs, 1H), 3.68–3.63 (m, 1H), 2.55–2.53 (m, 1H), 2.18–2.00 (m, 4H), 1.99–1.96 (m,1H), 1.86–1.61 (m, 4H), 1.59–1.30 (m, 6H); ^**13**^ **C­{** ^**1**^ **H}-NMR** (100 MHz, MeOD) δ [ppm] = 153.9, 152.3, 148.9 (br), 139.7, 134.7, 120.0, 118.9, 74.7, 72.0, 70.3, 62.2, 52.5, 32.5, 31.0, 25.3, 24.9, 24.5, 24.4; **HRMS** (ESI, 3.5 kV) *m*/*z*: [M + H]^+^ calc for C_20_H_27_N_5_O_3_H^+^ 386.2187, found 386.2186; **LCMS** *m*/*z* 386.4 (M + H) (ESI +ve), RT = 1.61 min; **HPLC** RT = 4.65 min, 98.97%.

##### Synthesis of (1R,2S,3R,7R,7aR)-3-(6-(Diethylamino)-9*H*-purin-9-yl)-2,3,5,6,7,7a-hexahydro-1*H*-indene-1,2,7-triol (**22a**)

To a stirred solution of **32a** (25 mg, 69.8 μmol, 1.0 equiv) in ACN (1.0 mL) was added diethylamine (36 μL, 0.34 mmol, 5.0 equiv) at room temperature. The resulting reaction mixture was heated to 60 °C for 26 h. After completion of the reaction, the reaction mixture was concentrated under reduced pressure to afford the crude. To a stirred solution of the crude in MeOH (1 mL) was added TFA (20% in H_2_O, 1 mL) at room temperature. The resulting reaction mixture was then stirred at room temperature for 14 h. After completion of the reaction, the reaction mixture was concentrated. Purification by prep. HPLC yielded **22a** as a white solid (13.5 mg, 37.5 μmol, 54% from **32a**). ^**1**^ **H NMR** (700 MHz, MeOD) δ [ppm] = 8.17 (s, 1H), 8.01 (s, 1H), 5.34 (dd, *J* = 1.8, 5.8 Hz, 1H), 4.90 (m, 1H), 4.32 (dd, *J* = 5.8, 7.6 Hz, 1H), 4.20 (dd, *J* = 4.3, 5.6 Hz, 1H), 4.00 (brs, 4H), 3.64 (ddd, *J* = 4.0, 9.9, 12.6 Hz, 1H), 2.53 (brs, 1H), 2.11–2.21 (m, 2H), 1.97 (m,1H), 1.60 (m, 1H), 1.28 (t, *J* = 7.2 Hz, 6H); ^**13**^ **C­{** ^**1**^ **H}-NMR** (176 MHz, MeOD) δ [ppm] = 155.0, 153.1, 151.9, 139.8, 136.4, 121.2, 120.6, 76.1, 73.4, 71.7, 63.3, 54.0, 44.3, 32.5, 26.0, 13.9; **HRMS** (ESI, 3.5 kV) *m*/*z*: [M + Na]^+^ calc for C_18_H_25_N_5_O_3_Na^+^ 382.1850, found 382.1846; **HPLC** RT = 20.91 min, 97.79%.

#### Synthesis of Inosine-Type Analogues

##### Synthesis of 9-((1R,2S,3R,3aR,4R)-2,3,4-Trihydroxy-2,3,3a,4,5,6-hexahydro-1*H*-inden-1-yl)-1,9-dihydro-6*H*-purin-6-one (**8a**)

To a stirred solution of **32a** (0.30 g, 0.87 mmol) in 1,4-dioxane (3.0 mL) was added conc. HCl (3.0 mL) at room temperature. The resulting reaction mixture was then stirred at room temperature for 16 h. After completion of the reaction, the reaction mixture was concentrated, neutralized using NH_4_OH (pH ∼ 8.0), and concentrated *in vacuo*. The crude was purified by reversed-phase chromatography using 0.1% NH_4_OH in water (100% Water). The obtained pure fractions were lyophilized to afford **8a** as a white solid (71 mg, 0.23 mmol, 28%). **R**_**f**_ = 0.1 (10% MeOH/DCM, 1% NH_4_OH); ^**1**^ **H NMR** (400 MHz, MeOD) δ [ppm] = 8.11 (s, 1H), 8.03 (s, 1H), 5.37 (d, *J* = 4.0 Hz, 1H), 4.97 (brs, 1H), 4.35 (dd, *J* = 6.4, 7.2 Hz, 1H), 4.23 (dd, *J* = 4.8, 5.6 Hz, 1H), 3.66–3.60 (m, 1H), 2.53 (brs, 1H), 2.19–2.18 (m, 2H), 2.00–1.97 (m, 1H), 1.63–1.59 (m, 1H); ^**13**^ **C­{** ^**1**^ **H}-NMR** (100 MHz, MeOD) δ [ppm] = 157.6, 149.3, 145.0, 139.9, 134.8, 123.9, 120.2, 75.0, 72.1, 70.3, 62.6, 52.4, 30.9, 24.5; **HRMS** (ESI, 3.5 kV) *m*/*z*: [M + H]^+^ calc for C_14_H_16_N_4_O_4_H^+^ 305.1244, found 305.1240; **LCMS** *m*/*z* 305.1 (M + H) (ESI +ve), RT = 1.24 min; **HPLC** RT = 3.29 min, 100%.

##### Synthesis of 9-((1R,2S,3R,3aS,5S)-2,3,5-Trihydroxy-2,3,3a,4,5,6-hexahydro-1*H*-inden-1-yl)-1,9-dihydro-6*H*-purin-6-one (**8b**)

To a stirred solution of **32b** (0.30 g, 0.82 mmol) in 1,4-dioxane (3 mL) was added conc. HCl (3 mL) at room temperature. The resulting reaction mixture was then stirred at room temperature for 16 h. After completion of the reaction, the reaction mixture was concentrated under reduced to give the crude, which was neutralized using NH_4_OH (pH ∼ 8.0), and concentrated *in vacuo*. The crude was purified by reversed-phase chromatography using 0.1% NH_4_OH in water (100% water). The obtained pure fractions were lyophilized to afford **8b** as a white solid (64 mg, 0.21 mmol, 25%). **R**_**f**_ = 0.1 (10% MeOH/DCM, 1% NH_4_OH); ^**1**^ **H NMR** (400 MHz, MeOD) δ [ppm] = 8.11 (brs, 1H), 8.04 (s, 1H), 5.31 (brs, 1H), 5.08–5.07 (m, 1H), 4.31 (dd, *J* = 4.4, 5.6 Hz, 1H), 4.07 (dd, *J* = 6.0, 8.0 Hz, 1H), 3.96–3.91 (m, 1H), 2.73 (brs, 1H), 2.42–2.35 (m, 2H), 1.94–1.87 (m, 1H), 1.43 (dd, *J* = 11.6, 22.8 Hz, 1H); ^**13**^ **C­{** ^**1**^ **H}-NMR** (100 MHz, MeOD) δ [ppm] = 157.6, 152.9 (br), 149.0 (br), 145.0, 137.2, 124.1 (br), 120.0, 76.1, 75.0, 66.8, 62.9, 44.0, 35.8, 33.9; **HRMS** (ESI, 3.5 kV) *m*/*z*: [M + H]^+^ calc for C_14_H_16_N_4_O_4_H^+^ 305.1244, found 305.1238; **LCMS** *m*/*z* 305.2 (M + H) (ESI +ve), RT = 1.10 min; **HPLC** RT = 4.43 min, 98.09%.

##### Synthesis 9-((1R,2S,3R,3aS,5R)-2,3,5-Trihydroxy-2,3,3a,4,5,6-hexahydro-1*H*-inden-1-yl)-1,9-dihydro-6*H*-purin-6-one (**8c**)

To a stirred solution of **Bz-32c** (0.30 g, 0.64 mmol) in 1,4-dioxane (3.0 mL) was added conc. HCl (2.0 mL) at room temperature. The resulting reaction mixture was then stirred at room temperature for 16 h. After completion of the reaction, the reaction mixture was concentrated under reduced pressure to afford the crude as a brown sticky solid (0.3 g). To a stirred solution of the crude (0.3 g) in methanol (6.0 mL) was added NaOMe (0.12 g, 2.22 mmol) at room temperature. The resulting reaction mixture was stirred at room temperature for 16 h. After completion of the reaction, the reaction mixture was concentrated under reduced pressure to give the crude, which was purified by reversed-phase chromatography using 0.1% NH_4_OH in water (100% water). The enriched crude was further purified by RP Prep HPLC. The obtained pure fractions were lyophilized to afford **8c** as a white solid (59 mg, 0.19 mmol, 30% over 2 steps). **R**_**f**_ = 0.1 (10% MeOH/DCM, 1% NH_4_OH); ^**1**^ **H NMR** (400 MHz, MeOD) δ [ppm] = 8.09 (s, 1H), 8.03 (s, 1H), 5.37 (brs, 1H), 5.04 (brs, 1H), 4.31 (dd, *J* = 5.6, 5.6 Hz, 1H), 4.20 (brs, 1H), 4.06 (dd, *J* = 7.2, 7.2 Hz, 1H), 2.84 (brs, 1H), 2.34–2.26 (m, 2H), 2.10–2.05 (m, 1H), 1.45 (dd, *J* = 11.2, 11.2 Hz, 1H); ^**13**^ **C­{** ^**1**^ **H}-NMR** (100 MHz, MeOD) δ [ppm] = 157.6, 149.0, 145.0, 140.1, 137.0, 123.9, 118.5, 75.7, 75.3, 63.6, 63.2, 38.1, 32.8, 32.5; **HRMS** (ESI, 3.5 kV) *m*/*z*: [M + H]^+^ calc for C_14_H_16_N_4_O_4_H^+^ 305.1244, found 305.1246; **LCMS** *m*/*z* 305.2 (M + H) (ESI +ve), RT = 0.85 min; **HPLC** RT = 4.377 min, 100%.

##### Synthesis of (1R,2S,3R,7R,7aR)-3-(6-(Methoxy)-9*H*-purin-9-yl)-2,3,5,6,7,7a-hexahydro-1*H*-indene-1,2,7-triol (**23a**)

To a stirred solution of **32a** (25 mg, 68.9 μmol, 1.0 equiv) in MeOH (1.0 mL) was added MeONa (7.5 mL, 0.13 mmol, 2.0 equiv) at room temperature. The resulting reaction mixture was stirred for 26 h. Then, to the stirred solution was added TFA (20% in H_2_O, 1 mL) at room temperature. The resulting reaction mixture was then stirred at room temperature for 24 h. After completion of the reaction, the reaction mixture was concentrated. Purification by prep. HPLC yielded **23a** as a white solid (13.9 mg, 43.4 μmol, 63% from **32a**). ^**1**^ **H NMR** (700 MHz, MeOD) δ [ppm] = 8.49 (s, 1H), 8.35 (s, 1H), 5.44 (dd, *J* = 1.8, 5.3 Hz, 1H), 4.89 (m, 1H), 4.41 (dd, *J* = 6.0, 7.6 Hz, 1H), 4.25 (dd, *J* = 4.5, 5.8 Hz, 1H), 4.19 (s, 3H), 3.64 (ddd, *J* = 4.4, 9.9, 11.6 Hz, 1H), 2.53 (brs, 1H), 2.10–2.21 (m, 2H), 1.97 (m, 1H), 1.60 (m, 1H); ^**13**^ **C­{** ^**1**^ **H}-NMR** (176 MHz, MeOD) δ [ppm] = 162.3, 153.5, 153.1, 144.1, 136.1, 122.1, 121.4, 76.2, 73.4, 71.7, 64.1, 54.8, 54.0, 32.4, 26.0; **HRMS** (ESI, 3.5 kV) *m*/*z*: [M + Na]^+^ calc for C_15_H_18_N_4_O_4_Na^+^ 341.1220, found 341.1217; **HPLC** RT = 17.3 min, 95.74%.

#### Synthesis of 6-Mercaptopurine-Type Analogues

##### Synthesis of 9-((3aR,3bR,4R,8R,8aS)-4-Hydroxy-2,2-dimethyl-3a,3b,5,6,8,8a-hexahydro-4*H*-indeno­[1,2-*d*]­[1,3]­dioxol-8-yl)-1,9-dihydro-6*H*-purine-6-thione (**57**)

To a stirred solution of **32a** (0.25 g, 0.68 mmol, 1.0 equiv) in a methanol (5.0 mL) was added potassium thioacetate (0.24 g, 2.07 mmol, 3.0 equiv) at room temperature. The resulting reaction mixture was stirred at room temperature for 5 h. After completion of the reaction, the reaction mixture was concentrated under reduced pressure, diluted with ethyl acetate (100 mL), filtered through Celite bed, and the obtained filtrate was concentrated under reduced pressure to get the crude, which was purified by reversed-phase chromatography using (100% water). The obtained pure fractions were lyophilized to afford **57** as a white solid (0.16 g, 0.44 mmol, 64%). **R**_**f**_ = 0.2 (5% MeOH/DCM); **HRMS** (ESI, 3.5 kV) *m*/*z*: [M + H]^+^ calc for C_17_H_20_N_4_O_3_SH^+^ 361.1329, found 361.1340; **LCMS** *m*/*z* 361.1 (M + H) (ESI +ve), RT = 1.50 min.

##### Synthesis of 9-((1R,2S,3R,3aR,4R)-2,3,4-Trihydroxy-2,3,3a,4,5,6-hexahydro-1*H*-inden-1-yl)-1,9-dihydro-6*H*-purine-6-thione (**15a**)

To a stirred solution of **57** (0.13 g, 0.36 mmol, 1.0 equiv) in a mixture of THF (1.3 mL) and water (1.3 mL) was added TFA (0.52 mL) at room temperature. The resulting reaction mixture was stirred at room temperature for 5 h. After completion of the reaction, the reaction mixture was concentrated under reduced pressure to get the crude, which was purified by RP Prep HPLC. The obtained pure fractions were lyophilized to afford **15a** as a white solid (50 mg, 0.16 mmol, 43%). **R**_**f**_ = 0.2 (10% MeOH/DCM); ^**1**^ **H NMR** (400 MHz, DMSO) δ [ppm] = 13.5 (brs, 1H), 8.33 (s, 1H), 8.18 (s, 1H), 5.24–5.19 (m, 2H), 4.85–4.83 (m, 2H), 4.72 (dd, *J* = 2.4, 2.4 Hz, 1H), 4.24–4.18 (m, 1H), 4.03–4.00 (m, 1H), 3.43–3.37 (m, 1H), 2.30 (d, *J* = 8.8 Hz, 1H), 2.04–2.01 (m, 2H), 1.83–1.80 (m, 1H), 1.45–1.40 (m, 1H); ^**13**^ **C­{** ^**1**^ **H}-NMR** (100 MHz, DMSO) δ [ppm] = 176.2, 145.3, 145.2, 142.5, 136.3, 135.4, 119.0, 74.7, 71.1, 69.4, 62.2, 53.5, 31.9, 25.0; **HRMS** (ESI, 3.5 kV) *m*/*z*: [M + H]^+^ calc for C_14_H_16_N_4_O_3_SH^+^ 321.1016, found 321.1011; **LCMS** *m*/*z* 321.0 (M + H) (ESI +ve), RT = 1.34 min; **HPLC** RT = 4.43 min, 100%.

##### Synthesis of 9-((1R,2S,3R,3aS,5S)-2,3,5-Trihydroxy-2,3,3a,4,5,6-hexahydro-1*H*-inden-1-yl)-1,9-dihydro-6*H*-purine-6-thione (**15b**)

To a stirred solution of **Bz-32b** (0.40 g, 0.86 mmol, 1.0 equiv) in methanol (8.0 mL) was added potassium thioacetate (0.29 g, 2.57 mmol, 3.0 equiv) at room temperature. The resulting reaction mixture was stirred at room temperature for 16 h. After completion of the reaction, the reaction mixture was concentrated under reduced pressure, diluted with water (100 mL), and extracted with ethyl acetate (3 × 20 mL). The combined organic phase was dried over anhydrous Na_2_SO_4_, filtered, and concentrated to afford crude as a brown solid (0.38 g). To a stirred solution of the crude (0.38 g, 0.82 mmol, 1.0 equiv) in a mixture of THF (3.8 mL) and water (3.8 mL) was added TFA (1.1 mL) at room temperature. The resulting reaction mixture was stirred at room temperature for 16 h. After completion of the reaction, the reaction mixture was concentrated under reduced pressure to afford the crude as a brown solid (0.47 g). To a stirred solution of the crude (0.47 g, 1.12 mmol, 1.0 equiv) in methanol (4.8 mL) was added sodium methoxide (0.18 g, 3.36 mmol, 3.0 equiv) and NH_4_OH (9.5 mL) at room temperature. The resulting reaction mixture was stirred at room temperature for 48 h. After completion of the reaction, the reaction mixture was concentrated under reduced pressure to get the crude, which was purified by reversed-phase chromatography using (40% MeCN/water). The obtained pure fractions were lyophilized to afford **15b** as a white solid (57 mg, 0.17 mmol, 20% over 3 steps). **R**_**f**_ = 0.1­(10% MeOH/DCM); ^**1**^ **H NMR** (400 MHz, DMSO) δ [ppm] = 13.70 (brs, 1H), 8.29 (s, 1H), 8.18 (s, 1H), 5.23 (brs, 1H), 5.13 (s, 1H), 4.97 (brs, 1H), 4.97–4.88 (m, 1H), 4.77 (brs, 1H), 4.14 (s, 1H), 3.84 (dd, *J* = 6.4, 6.4 Hz, 1H), 3.72 (brs, 1H), 2.51–2.49 (m, 1H), 2.24–2.14 (m, 2H), 1.77–1.70 (m,1H), 1.22 (dd, *J* = 11.6, 22.8 Hz, 1H); ^**13**^ **C­{** ^**1**^ **H}-NMR** (100 MHz, DMSO) δ [ppm] = 176.4, 145.5, 144.7, 142.6, 138.1, 135.4, 119.9, 76.0, 75.0, 66.4, 62.6, 44.5, 36.9, 34.9; **HRMS** (ESI, 3.5 kV) *m*/*z*: [M + H]^+^ calc for C_14_H_16_N_4_O_3_SH^+^ 321.1016, found 321.1011; **LCMS** *m*/*z* 321.0 (M + H) (ESI +ve), RT = 1.57 min; **HPLC** RT= 5.01 min, 100%.

##### Synthesis of 9-((1R,2S,3R,3aS,5R)-2,3,5-Trihydroxy-2,3,3a,4,5,6-hexahydro-1*H*-inden-1-yl)-1,9-dihydro-6*H*-purine-6-thione (**15c**)

To a stirred solution of **Bz-32c** (0.40 g, 0.85 mmol, 1.0 equiv) in methanol (4.0 mL) was added potassium thioacetate (0.29 g, 2.57 mmol, 3.0 equiv) at room temperature. The resulting reaction mixture was stirred at room temperature for 16 h. After completion of the reaction, the reaction mixture was concentrated under reduced pressure, diluted with water (90 mL), and extracted with ethyl acetate (3 × 50 mL). The combined organic phase was dried over anhydrous Na_2_SO_4_, filtered, and concentrated under reduced pressure to afford the crude as a brown solid (0.40 g). To a stirred solution of the crude (0.40 g) in a mixture of THF (4.0 mL) and water (4.0 mL) was added TFA (0.8 mL) at room temperature. The resulting reaction mixture was stirred at room temperature for 16 h. After completion of the reaction, the reaction mixture was concentrated under reduced pressure to afford the crude as a light-yellow sticky solid (0.65 g). To a stirred solution of the crude (0.65 g) in methanol (6.5 mL) was added sodium methoxide (0.41 g, 7.66 mmol) and NH_4_OH (6.5 mL) at room temperature. The resulting reaction mixture was stirred at room temperature for 48 h. After completion of the reaction, the reaction mixture was concentrated under reduced pressure to get the crude, which was purified by RP Prep HPLC. The obtained pure fractions were lyophilized to afford **15c** as a white solid (32 mg, 0.17 mmol, 9% yield over 3 steps). **Rf** = 1.0 (10% MeOH/DCM); ^**1**^ **H NMR** (400 MHz, DMSO) δ [ppm] = 13.64–13.63 (brs, 1H), 8.29 (s, 1H), 8.18 (s, 1H), 5.19 (d, *J* = 5.2 Hz, 2H), 4.90 (d, *J* = 6.0 Hz, 1H), 4.82 (d, *J* = 2.4 Hz, 1H), 4.55 (d, *J* = 2.8 Hz, 1H), 4.15 (dd, *J* = 5.6, 11.2 Hz, 1H), 4.00 (brs, 1H), 3.83 (q, *J* = 6.0, 12.8 Hz, 1H), 2.66–2.62 (m, 1H), 2.13–2.04 (m, 2H), 1.87 (d, *J* = 18.0 Hz, 1H), 1.26 (dd, *J* = 11.2, 11.2 Hz, 1H); ^**13**^ **C­{** ^**1**^ **H}-NMR** (100 MHz, DMSO) δ [ppm] = 176.6, 145.6, 144.9, 142.5, 138.1, 135.4, 118.2, 75.4, 75.1, 62.9, 62.9, 38.8, 33.6, 33.5; **HRMS** (ESI, 3.5 kV) *m*/*z*: [M + H]^+^ calc for C_14_H_16_N_4_O_3_SH^+^ 321.1016, found 321.1031; **LCMS** *m*/*z* 321.0 (M + H) (ESI +ve); RT = 1.84 min; **HPLC** RT= 4.98 min, 99.07%.

#### Synthesis of Guanosine-Type Analogues

##### Synthesis of 6-Chloro-9-((3aS,4S,6aR)-5-iodo-2,2-dimethyl-3a,6a-dihydro-4*H*-cyclopenta­[*d*]­[1,3]­dioxol-4-yl)-*N*-trityl-9*H*-purin-2-amine (**29**)

To a stirred solution of alcohol **25** (10 g, 35.5 mmol, 1.0 equiv), 6-chloro-*N*-trityl-7*H*-purin-2-amine **27** (16.0 g, 39.0 mmol, 1.1 equiv), and PPh_3_ (12.1 g, 46.1 mmol, 1.3 equiv) in anhydrous THF (0.2 L) was dropwise added DIAD (9.05 mL, 46.1 mmol, 1.3 equiv) at 0 °C under N_2_ atmosphere. The resulting reaction mixture was stirred at room temperature for 2.5 h. After completion of the reaction, the reaction mixture was diluted with water (0.7 L) and extracted with ethyl acetate (3 × 500 mL). The combined organic phase was dried over anhydrous Na_2_SO_4_, filtered, and concentrated to give the crude, which was purified by column chromatography on silica gel (18% EA/Hex) to afford the title compound **29** as a white solid (12 g, 50%). **R**_**f**_ = 0.25 (30% EA/hex); ^**1**^ **H NMR** (400 MHz, CDCl_3_) δ [ppm] = 7.63 (s, 1H), 7.30–7.24 (m, 15H), 6.63 (s, 1H), 6.09 (s, 1H), 4.90 (s, 1H), 4.39 (d, *J* = 6.0 Hz, 1H), 4.18 (brs, 1H), 1.38 (s, 3H), 1.28 (s, 3H); ^**13**^ **C­{** ^**1**^ **H}-NMR** (100 MHz, CDCl_3_) δ [ppm] = 171.1, 157.7, 151.6, 151.0, 144.7, 144.6, 142.4, 128.8, 128.1, 127.9, 127.7, 127.2, 127.1, 125.6, 112.4, 96.6, 84.7, 81.0, 74.3, 71.4, 27.2, 26.2; **HRMS** (ESI, 3.5 kV) *m*/*z*: [M + H]^+^ calc for C_32_H_27_ClIN_5_O_2_H^+^ 676.0971, found 676.0954; **LCMS** *m*/*z* 676.1 (M + H) (ESI +ve); RT = 2.97 min; **HPLC** RT = 11.03 min 100%.

##### Synthesis of 6-Chloro-9-((3aS,4R,6aR)-2,2-dimethyl-5-vinyl-3a,6a-dihydro-4*H*-cyclopenta­[*d*]­[1,3]­dioxol-4-yl)-*N*-trityl-9*H*-purin-2-amine (**31**)

To a stirred solution of iodine **29** (7.0 g, 10.4 mmol, 1.0 equiv), Ph_3_As (0.32 g, 1.03 mmol, 0.1 equiv), [Pd­(PhCN)_2_Cl_2_] (0.19 g, 0.51 mmol, 0.05 equiv), and CuI (0.19 g, 1.03 mmol, 0.1 equiv) in anhydrous NMP (140 mL) was dropwise added vinyl tributyltin (4.92 g, 15.5 mmol, 1.5 equiv) at room temperature under N_2_ atmosphere. The resulting reaction mixture was stirred at room temperature for 8 h. After completion of the reaction, the reaction mixture was diluted with water (400 mL) and extracted with ethyl acetate (3 × 300 mL). The combined organic phase was washed with brine (3 L), dried over Na_2_SO_4_, filtered, and concentrated to give the crude, which was purified by column chromatography on silica gel (23% EA/hex) to afford the title compound **31** as a white solid. (5.6 g, 93%). **R**_**f**_ = 0.2 (40% EA/hex); ^**1**^ **H NMR** (400 MHz, CDCl_3_) δ [ppm] = 7.44 (s, 1H), 7.42–7.37 (m, 6H), 7.31–7.19 (m, 9H), 6.67 (s, 1H), 6.27 (dd, *J* = 10.8, 17.6 Hz, 1H), 5.91 (s, 1H), 5.14 (brs, 1H), 5.03 (d, *J* = 11.2 Hz, 1H), 4.93 (brs, 1H), 4.69 (d, *J* = 18.0 Hz, 1H), 4.29 (d, *J* = 5.6 Hz, 1H), 1.38 (s, 3H), 1.29 (s, 3H). ^**13**^ **C­{** ^**1**^ **H}-NMR** (100 MHz, CDCl_3_) δ [ppm] = 157.5, 152.1, 150.6, 144.8, 140.3, 139.2, 134.8, 129.7, 129.0, 127.7, 126.7, 125.0, 119.2, 111.7, 84.0, 83.1, 71.2, 63.3, 27.4, 26.0; **HRMS** (ESI, 3.5 kV) *m*/*z*: [M + H]^+^ calc for C_34_H_30_ClN_5_O_2_ 576.2161, found 576.2148; **LCMS** *m*/*z* 576.3 (M + H) (ESI +ve); RT = 2.91 min; **HPLC** RT = 10.78 min 100%.

##### Synthesis of (3aR,3bR,4R,8R,8aS)-8-(6-Chloro-2-(tritylamino)-9*H*-purin-9-yl)-2,2-dimethyl-3a,3b,5,6,8,8a-hexahydro-4*H*-indeno­[1,2-*d*]­[1,3]­dioxol-4-ol (**33a**)

A 100 mL sealed tube was charged with diene **31** (2.0 g, 3.47 mmol, 1.0 equiv) in toluene (44 mL) were added BHT (0.08 g, 0.34 mmol, 0.1 equiv) and vinyl pinacol borate (1.60 g, 10.4 mmol, 3.0 equiv) at room temperature. The sealed tube was packed, and the resulting reaction mixture was heated to 140 °C for 8 days. To the reaction mixture every 16 h was added another portion of vinyl pinacol borate (2.0 equiv) and BHT (0.15 equiv). After completion of the reaction, the reaction mixture was concentrated to give the crude. The obtained crude was dissolved in a mixture of THF (20 mL) and 7.0 pH buffer solution (20 mL). To this reaction mixture, NaBO_3_ 4H_2_O (2.13 g, 13.8 mmol, 4.0 equiv) was added portion-wise at room temperature *(exothermicity observed).* The resulting reaction was stirred at room temperature for 8 h. After completion of the reaction, the reaction mixture was diluted with saturated solution of Na_2_S_2_O_3_ (100 mL) and was stirred at room temperature until clear. The layer was separated, and aqueous phase was extracted with ethyl acetate (3 × 100 mL). The combined organic phase was dried over anhydrous Na_2_SO_4_, filtered, and concentrated to give the crude, which was purified by column chromatography on silica gel (1% MeOH/DCM) to afford **33b/c** (0.8 g, 1.29 mmol, 36%) as a mixture of isomers as well as **33a** (0.4 g, 0.64 mmol, 18%) as a pure compound. **33a**: **R**_**f**_ = 0.18 (5% MeOH/DCM); ^**1**^ **H NMR** (400 MHz, CDCl_3_) δ [ppm] = 7.62 (s, 1H) 7.28–7.22 (m, 15H), 6.60 (s, 1H), 4.76 (brs, 1H), 4.65 (brs, 1H), 4.42 (brs, 1H), 4.22 (brs, 1H), 3.30–3.27 (m, 1H), 2.42 (brs, 1H), 2.06–1.92 (m, 3H), 1.72 (brs, 1H), 1.52 (m, 1H), 1.46 (s, 3H), 1.31 (s, 3H). ^**13**^ **C­{** ^**1**^ **H}-NMR** (100 MHz, CDCl_3_) δ [ppm] = 157.4, 152.4, 150.8, 144.8, 142.0, 135.7, 128.8, 127.8, 126.7, 120.2, 113.7, 81.9, 81.3, 71.1, 70.9, 64.3, 51.6, 31.2, 27.8, 25.9, 24.6. **HRMS** (ESI, 3.5 kV) *m*/*z*: [M + H]^+^ calc for C_36_H_34_ClN_5_O_3_H^+^ 620.2423, found 620.2407; **LCMS** *m*/*z* 620.3 (M + H) (ESI +ve), RT = 2.52 min; **HPLC** RT = 9.39 min 97.23%.

##### Synthesis of (3aR,3bS,5S,8R,8aS)-8-(6-Chloro-2-(tritylamino)-9*H*-purin-9-yl)-2,2-dimethyl-3a,3b,5,6,8,8a-hexahydro-4*H*-indeno­[1,2-*d*]­[1,3]­dioxol-5-yl Benzoate (**Bz-33b**)

To a stirred solution of **33b/c** (1.50 g, 2.41 mmol, 1.0 equiv) in pyridine (15 mL) was added dropwise benzoyl chloride (0.51 g, 3.62 mmol, 1.5 equiv) at 0 °C. The resulting reaction mixture was stirred at room temperature for 2 h. After completion of the reaction, the reaction mixture was diluted with water (100 mL) and extracted with ethyl acetate (3 × 50 mL). The combined organic phase was dried over anhydrous Na_2_SO_4_, filtered, and concentrated to give the crude, which was purified by column chromatography on silica gel (30% EA/hex) to afford the title compound **Bz-33b** as a white solid (0.75 g, 1.03 mmol, 43%). **R**_**f**_ = 0.45 (50% EA/hex); ^**1**^ **H NMR** (400 MHz, CDCl_3_) δ [ppm] = 8.10 (d, *J* = 8.0 Hz, 2H), 7.64–7.60 (m, 2H), 7.51 (t, *J* = 7.6 Hz, 2H), 7.37–7.21 (m, 15H), 6.62 (s, 1H), 5.18 (brs, 1H), 4.80 (brs, 1H), 4.69 (s, 1H), 4.45­(brs, 1H), 4.01­(brs, 1H), 2.69 (brs, 1H), 2.44 (brs, 2H), 2.06–2.02­(m, 1H), 1.46 (s, 3H) 1.37–1.27 (m, 4H); ^**13**^ **C­{** ^**1**^ **H}-NMR** (100 MHz, CDCl_3_) δ [ppm] = 165.9, 157.5, 152.4, 150.9, 144.9, 142.0, 137.9, 133.1, 130.3, 129.5, 129.0, 128.8, 128.4, 127.8, 127.7, 127.6, 126.8, 125.3, 118.3, 113.5, 82.7, 81.5, 71.1, 70.0, 64.5, 43.2, 32.0, 27.9, 26.0; **HRMS** (ESI, 3.5 kV) *m*/*z*: [M + H]^+^ calc for C_43_H_38_ClN_5_O_4_, 724.2685, found 724.2684; **LCMS** *m*/*z* 724.3 (M + H) (ESI +ve), RT = 3.18 min; **HPLC** RT = 11.93 min 98.56%.

##### Synthesis of (3aR,3bS,5R,8R,8aS)-8-(6-Chloro-2-(tritylamino)-9*H*-purin-9-yl)-2,2-dimethyl-3a,3b,5,6,8,8a-hexahydro-4*H*-indeno­[1,2-*d*]­[1,3]­dioxol-5-yl Benzoate (**Bz-33c**)

To a stirred solution of **33b/c** (1.5 g, 2.41 mmol, 1.0 equiv) and benzoic acid (0.59 g, 4.83 mmol, 2.0 equiv) in THF (30 mL) were added PPh_3_ (1.26 g, 4.83 mmol, 2.0 equiv) followed by dropwise addition of DIAD (0.95 mL, 4.83 mmol, 2.0 equiv) at 0 °C under N_2_ atmosphere. The resulting reaction mixture was stirred at room temperature for 2 h. After completion of the reaction, the reaction mixture was diluted with water (100 mL) and extracted with ethyl acetate (3 × 150 mL). The combined organic phase was dried over anhydrous Na_2_SO_4_, filtered, and concentrated to give the crude, which was purified by column chromatography on silica gel (35% EA/hex) to afford the title compound **Bz-33c** as a white solid (0.72 g, 0.99 mmol, 35%). **R**_**f**_ = 0.5 (50% EA/hex); ^**1**^ **H NMR** (400 MHz, CDCl_3_) δ [ppm] = 8.03 (d, *J* = 7.2 Hz, 2H), 7.65–7.56 (m, 2H), 7.48–7.44 (m, 2H), 7.28–7.22 (m, 15H), 6.60 (brs, 1H), 5.40 (brs, 1H), 4.84 (brs, 1H), 4.69 (s, 1H), 4.46 (brs, 1H), 4.05­(brs, 1H), 2.74 (brs, 1H), 2.44 (brs, 1H), 2.25–2.20 (m, 2H), 1.46 (s, 3H) 1.30 (brs, 3H), 1.18 (brs, 1H); ^**13**^ **C­{** ^**1**^ **H}-NMR** (400 MHz, CDCl_3_) δ [ppm] = 165.9, 157.4, 152.4, 150.9, 144.8, 142.1, 137.6, 133.1, 130.3, 129.5, 128.8, 128.4, 127.8, 126.8, 117.0, 113.4, 83.1, 81.1, 71.0, 67.7, 64.6, 38.1, 30.3, 29.6, 27.8, 25.8; **HRMS** (ESI, 3.5 kV) *m*/*z*: [M + H]+ calc for C_43_H_38_ClN_5_O_4_, 724.2685, found 724.2687; **LCMS** *m*/*z* 724.3 (M + H) (ESI +ve), RT = 3.11 min; **HPLC** RT = 11.62 min. 99.13%.

##### Synthesis of 2-Amino-9-((1R,2S,3R,3aR,4R)-2,3,4-trihydroxy-2,3,3a,4,5,6-hexahydro-1*H*-inden-1-yl)-1,9-dihydro-6*H*-purin-6-one (**9a**)

To a stirred solution of **33a** (0.36 g, 0.58 mmol, 1.0 equiv) in 1,4-dioxane (3.6 mL) was added conc. HCl (3.6 mL) at room temperature. The resulting reaction mixture was heated to 100 °C for 4 h. After completion of the reaction, the reaction mixture was concentrated and neutralized using NH_4_OH (pH 8.0) and again concentrated to give the crude, which was purified by reversed-phase chromatography using 0.1% NH_4_OH in water (21% MeCN/Water). The pure fractions obtained were lyophilized to afford the title compound **9a** as a white solid (51 mg, 0.15 mmol, 27%). **R**_**f**_ = 0.1 (10% MeOH/DCM); ^**1**^ **H NMR** (400 MHz, MeOD) δ [ppm] = 7.73 (s, 1H), 5.18 (brs, 1H), 5.01 (s, 1H), 4.25 (dd, *J* = 6.4, 6.4 Hz, 1H), 4.19 (dd, *J* = 4.8, 4.8 Hz, 1H), 3.65–3.59 (m, 1H), 2.49–2.48 (m, 1H), 2.20 (brs, 2H), 1.99–1.96 (m, 1H), 1.62–1.57 (m, 1H); ^**13**^ **C­{** ^**1**^ **H}-NMR** (100 MHz, DMSO) δ [ppm] = 157.3, 153.9, 152.4, 137.0, 136.2, 118.3, 116.7, 74.5, 71.1, 69.4, 61.0, 53.3, 31.9, 25.0; **HRMS** (ESI, 3.5 kV) *m*/*z*: [M + H]^+^ calc for C_14_H_17_N_5_O_4_H^+^ 320.1353, found 320.1356; **LCMS** *m*/*z* 320.2 (M + H) (ESI +ve), RT = 2.02 min; **HPLC** RT = 8.06 min, 100%.

##### Synthesis of 2-Amino-9-((1R,2S,3R,3aS,5S)-2,3,5-trihydroxy-2,3,3a,4,5,6-hexahydro-1*H*-inden-1-yl)-1,9-dihydro-6*H*-purin-6-one (**9b**)

To a stirred solution of **Bz-33b** (0.38 g, 0.52 mmol, 1.0 equiv) in 1,4-dioxane (3.8 mL) was added conc. HCl (1.14 mL) at room temperature. The resulting reaction mixture was heated to 100 °C for 4h. After completion of the reaction, the reaction mixture was concentrated under reduced pressure to afford the crude as a yellow solid. To a stirred solution of the crude (0.38 g) in a mixture of MeOH (3.8 mL) and DCM (3.8 mL) was added NaOMe (0.29 g, 5.38 mmol) at room temperature. The resulting reaction mixture was then stirred at room temperature for 16 h. After completion of the reaction, the reaction mixture was concentrated under reduced pressure to give the crude, which was purified by reversed-phase chromatography using 0.1% NH_4_OH in water (31% MeCN/Water). The obtained pure fractions were lyophilized to afford **9b** as a white solid (53 mg, 0.17 mmol, 31% over 2 steps). **R**_**f**_ = 0.2 (15% MeOH/DCM); ^**1**^ **H NMR** (400 MHz, MeOD) δ [ppm] = 7.63 (s, 1H), 5.14–5.12 (m, 2H), 4.19 (dd, *J* = 5.6, 5.6 Hz, 1H), 3.99–3.89 (m, 2H), 2.70 (brs, 1H), 2.43–2.34 (m, 2H), 1.96–1.89 (m, 1H), 1.39 (dd, *J* = 11.6, 22.8 Hz, 1H); ^**13**^ **C­{** ^**1**^ **H}-NMR** (100 MHz, MeOD) δ [ppm] = 161.6 (br), 156.3 (br), 151.8, 137.4, 136.3, 120.0, 116.6, 76.1, 75.1, 66.8, 61.7, 43.9, 35.8, 33.9; **HRMS** (ESI, 3.5 kV) *m*/*z*: [M + H]^+^ calc for C_14_H_17_N_5_O_4_H^+^ 320.1353, found 320.1350; **LCMS** *m*/*z* 320.2 (M + H) (ESI +ve), RT = 1.94 min; **HPLC** RT = 7.73 min; 100%.

##### Synthesis of 2-Amino-9-((1R,2S,3R,3aS,5R)-2,3,5-trihydroxy-2,3,3a,4,5,6-hexahydro-1*H*-inden-1-yl)-1,9-dihydro-6*H*-purin-6-one (**9c**)

To a stirred solution of **Bz-33c** (0.37 g, 0.51 mmol, 1.0 equiv) in 1,4-dioxane (3.7 mL) was added conc. HCl (1.11 mL) at room temperature. The resulting reaction mixture was heated to 100 °C for 16 h. After completion of the reaction, the reaction mixture was concentrated under reduced pressure to afford the crude as a brown oil. To a stirred solution of the crude (0.37 g) in a mixture of MeOH (3.7 mL) and DCM (3.7 mL) was added NaOMe (0.28 g, 5.24 mmol) at room temperature. The resulting reaction mixture was stirred at room temperature for 16 h. After completion of the reaction, the reaction mixture was concentrated under reduced pressure to give the crude, which was purified by reversed-phase chromatography using 0.1% NH_4_OH in water (24% MeCN/water). The obtained pure fractions were lyophilized to afford **9c** as a white solid (51 mg, 0.16 mmol, 31% over 2 steps). **R**_**f**_ = 0.1 (10% MeOH/DCM); ^**1**^ **H NMR** (400 MHz, MeOD) δ [ppm] = 7.70 (s, 1H), 5.19 (s, 1H), 5.08 (s, 1H), 4.23–4.21 (m, 2H), 4.01 (dd, *J* = 7.6, 7.6 Hz, 1H), 2.80­(brs, 1H), 2.33–2.27­(m, 2H), 2.11–2.07 (m, 1H), 1.46 (dd, *J* = 11.2, 11.2 Hz, 1H); ^**13**^ **C­{** ^**1**^ **H}-NMR** (100 MHz, MeOD) δ [ppm] = 158.1, 153.7, 152.0, 137.3, 137.1, 118.5, 116.1, 75.7, 75.4, 63.7, 62.3, 38.0, 32.8, 32.5; HRMS (ESI, 3.5 kV) *m*/*z*: [M + H]^+^ calc for C_14_H_17_N_5_O_4_H^+^ 320.1353, found 320.1353; **LCMS** *m*/*z* 320.3 (M + H) (ESI +ve), RT = 1.90 min; **HPLC** RT = 7.70 min 100%.

##### Synthesis of (1R,2S,3R,7R,7aR)-3-(2-Amino-6-methoxy-9*H*-purin-9-yl)-2,3,5,6,7,7a-hexahydro-1*H*-indene-1,2,7-triol (**10a**)

To a stirred solution of **33a** (0.40 g, 0.64 mmol, 1.0 equiv) in MeOH (4 mL) was added NaOMe (0.1 g, 1.93 mmol, 3.0 equiv) at room temperature. The resulting reaction mixture was heated at 80 °C for 6 h. After completion of the reaction, the reaction mixture was diluted with cold water (70 mL) and extracted with ethyl acetate (2 × 100 mL). The combined organic phase was dried over Na_2_SO_4_, filtered, and concentrated to give the crude as a off-white solid (0.4 g), which was redissolved in THF (4.0 mL), water (4.0 mL), and added TFA (1.2 mL) at room temperature. The resulting reaction mixture was then stirred at room temperature for 16 h. After completion of the reaction, the reaction mixture was concentrated, neutralized using NH_4_OH (pH 8.0), and concentrated to give the crude, which was purified by reversed-phase chromatography using 0.1% NH_4_OH in water (43% MeCN/Water). The pure fractions obtained were lyophilized to afford the title compound **10a** as a white solid (66 mg, 0.19 mmol, 30% over 2 steps). **R**_**f**_ = 0.3 (5% MeOH/DCM); ^**1**^ **H NMR** (400 MHz, MeOD) δ [ppm] = 7.87 (s, 1H), 5.25–5.24 (m, 1H), 4.97 (brs, 1H), 4.30 (dd, *J* = 6.0, 6.0 Hz, 1H), 4.21 (dd, *J* = 4.8, 4.8 Hz, 1H), 4.07 (s, 3H), 3.66–3.60 (m, 1H), 2.51 (brs, 1H), 2.19–2.18 (m, 2H), 1.99–1.96 (m, 1H), 1.63–1.58 (m, 1H); ^**13**^ **C­{** ^**1**^ **H}-NMR** (100 MHz, MeOD) δ [ppm] = (161.8), (161.5), 161.2, 160.3, 154.0, 138.5, 134.9, 120.0, (118.3), (115.4), 113.7, 74.7, 72.1, 70.4, 61.7, 52.8, 52.3, 31.0, 24.5; **HRMS** (ESI, 3.5 kV) *m*/*z*: [M + H] calc for C_15_H_19_N_5_O_4_H^+^ 334.1510, found 334.1507; **LCMS** *m*/*z* 334.3 (M + H) (ESI +ve), RT = 2.15 min; **HPLC** RT = 8.55 min. 100%. Note: Although, in 1H-NMR, LCMS, and HPLC analysis only one product could be observed, four additional peaks in carbon NMR are visible (marked in brackets). This may be attributed to the salt of the nucleoside analogue.

##### Synthesis of (1R,2S,3R,6S,7aS)-3-(2-Amino-6-methoxy-9*H*-purin-9-yl)-2,3,5,6,7,7a-hexahydro-1*H*-indene-1,2,6-triol (**10b**)

To a stirred solution of **Bz-33b** (0.40 g, 0.55 mmol, 1.0 equiv) in MeOH (4 mL) was added NaOMe (0.17 g, 3.31 mmol, 6.0 equiv) at room temperature. The resulting reaction mixture was heated to 80 °C for 6 h. After completion of the reaction, the reaction mixture was diluted with cold water (70 mL) and extracted with ethyl acetate (2 × 70 mL). The combined organic phase was dried over Na_2_SO_4_, filtered, and concentrated to give the crude as an off-white solid (0.4 g). The combined organic phase was dried over Na_2_SO_4_, filtered, and concentrated to give the crude as an off-white solid (0.4 g), which was redissolved in THF (4.0 mL), water (4.0 mL), and added TFA (1.2 mL) at room temperature. The resulting reaction mixture was stirred at room temperature for 16 h. After completion of the reaction, the reaction mixture was concentrated, neutralized using NH_4_OH (pH 8.0), and concentrated to give the crude, which was purified by reversed-phase chromatography using 0.1% NH_4_OH in water (28% MeCN/Water). The obtained fractions were lyophilized to get crude. The enriched crude was further purified by SFC using 0.1% methanolic ammonia in methanol: acetonitrile (50:50), and the obtained pure fractions were concentrated to afford the title compound **10b** as a white solid (51 mg, 0.15 mmol, 27% over 2 steps). **R**_**f**_ = 0.1 (10% MeOH/DCM); ^**1**^ **H NMR** (400 MHz, MeOD) δ [ppm] = 7.83 (s, 1H), 5.18 (s, 1H), 5.09 (brs, 1H), 4.24 (dd, *J* = 5.6, 5.6 Hz, 1H), 4.07–4.01 (m, 4H), 3.94–3.89 (m, 1H), 2.72 (brs, 1H), 2.41–2.35 (m, 2H), 1.95–1.87 (m, 1H), 1.42 (dd, *J* = 11.6, 23.2 Hz, 1H); ^**13**^ **C­{** ^**1**^ **H}-NMR** (100 MHz, MeOD) δ [ppm] = 161.2, 160.3, 153.7, 138.6, 137.2, 119.9, 113.8, 75.9, 75.0, 66.8, 62.0, 52.7, 43.9, 35.8, 33.9; **HRMS** (ESI, 3.5 kV) *m*/*z*: [M + H]^+^ calc for C_15_H_19_N_5_O_4_H^+^ 334.1510 found 334.1508; **LCMS** *m*/*z* 334.2 (M + H) (ESI +ve), RT = 1.30 min; **HPLC** RT = 5.35 min 97.80%.

##### Synthesis of (1R,2S,3R,6R,7aS)-3-(2-Amino-6-methoxy-9*H*-purin-9-yl)-2,3,5,6,7,7a-hexahydro-1*H*-indene-1,2,6-triol (**10c**)

To a stirred solution of **Bz-33c** (0.4 g, 0.55 mmol, 1.0 equiv) in MeOH (4 mL) was added NaOMe (0.17 g, 3.31 mmol, 6.0 equiv) at room temperature. The resulting reaction mixture was heated to 80 °C for 6 h. After completion of the reaction, the reaction mixture was diluted with cold water (70 mL) and extracted with ethyl acetate (2 × 80 mL). The combined organic phase was dried over Na_2_SO_4_, filtered, and concentrated to give the crude as an off-white solid (0.4 g), which was redissolved in THF (4.0 mL), water (4.0 mL), and added TFA (1.2 mL) at room temperature. The resulting reaction mixture was stirred at room temperature for 16 h. After completion of the reaction, the reaction mixture was concentrated, neutralized using NH_4_OH (pH ∼ 8.0), and concentrated to give the crude, which was purified by reversed-phase chromatography using 0.1% NH_4_OH in water (40% MeCN/water). The obtained pure fractions were lyophilized to afford the title compound **10c** as a white solid (51 mg, 0.15 mmol, 28% over 2 steps). **R**_**f**_ = 0.2 (10% MeOH/DCM); ^**1**^ **H NMR** (400 MHz, MeOD) δ [ppm] = 7.83 (s, 1H), 5.24 (s, 1H), 5.05 (brs, 1H), 4.25 (dd, *J* = 5.6, 5.6 Hz, 1H), 4.20 (brs, 1H), 4.07–4.01 (m, 4H), 2.83 (brs, 1H), 2.33–2.28 (m, 2H), 2.10–2.05 (m, 1H), 1.48 (dd, *J* = 11.2, 11.2 Hz, 1H); ^**13**^ **C­{** ^**1**^ **H}-NMR** (100 MHz, MeOD) δ [ppm] = 161.2, 160.3, 153.8, 138.7, 137.0, 118.5, 113.8, 75.5, 75.4, 63.7, 62.3, 52.7, 38.0, 32.8, 32.5; **HRMS** (ESI, 3.5 kV) *m*/*z*: [M + H] calc for C_15_H_19_N_5_O_4_H^+^ 334.1510, found 334.1502; **LCMS** *m*/*z* 334.3 (M + H) (ESI +ve), RT = 5.41 min; **HPLC** RT = 5.45 min 100%.

##### Synthesis of (1R,2S,3R,7R,7aR)-3-(2-Amino-6-(methylamino)-9*H*-purin-9-yl)-2,3,5,6,7,7a-hexahydro-1*H*-indene-1,2,7-triol (**11a**)

A stirred solution of **33a** (0.4 g, 0.64 mmol, 1.0 equiv) in 1 M methylamine in THF (4 mL) was heated to 80 °C for 6 h. After completion of the reaction, the reaction mixture was concentrated under reduced pressure to give crude as a brown oil (0.40 g). The crude was dissolved in THF (4.0 mL) and TFA (4 mL) was added at room temperature. The resulting reaction mixture was stirred at room temperature for 16 h. After completion of the reaction, the reaction mixture was concentrated, neutralized using NH_4_OH (pH ∼ 8.0), and concentrated to give the crude, which was purified by reversed-phase chromatography using 0.1% NH_4_OH in water (37% MeCN/Water). The pure fractions obtained were lyophilized to afford the title compound **11a** as a white solid (65 mg, 0.19 mmol, 30% over 2 steps). **R**_**f**_ = 0.05 (10% MeOH/DCM); ^**1**^ **H NMR** (400 MHz, MeOD) δ [ppm] = 7.72 (s, 1H), 5.18–5.17 (m, 1H), 4.99–4.98­(m, 1H), 4.24 (dd, *J* = 6.0, 7.6 Hz, 1H), 4.18 (dd, *J* = 4.4, 6.0 Hz, 1H), 3.65–3.59 (m, 1H), 3.06 (s, 3H), 2.51 (brs, 1H), 2.18 (brs, 2H), 1.99–1.95 (m, 1H), 1.62–1.60 (m, 1H); ^**13**^ **C­{** ^**1**^ **H}-NMR** (100 MHz, MeOD) δ [ppm] = 160.5, 155.7, 150.9 (br), 136.2, 135.1, 119.9, 113.2, 74.8, 72.2, 70.4, 61.3, 52.3, 31.0, 26.2, 24.6. **HRMS** (ESI, 3.5 kV) *m*/*z*: [M + H] calc for C_15_H_20_N_6_O_3_H^+^ 333.1670 found 333.1685; **LCMS** *m*/*z* 333.3 (M + H) (ESI +ve), RT = 2.12 min; **HPLC** RT = 7.84 min 100%.

##### Synthesis of (1R,2S,3R,7R,7aR)-3-(2-Amino-6-(dimethylamino)-9*H*-purin-9-yl)-2,3,5,6,7,7a-hexahydro-1*H*-indene-1,2,7-triol (**12a**)

A stirred solution of **33a** (0.40 g, 0.64 mmol, 1.0 equiv) in 2 M dimethylamine in THF (4 mL) was heated to 80 °C for 6 h. After completion of the reaction, the reaction mixture was concentrated under reduced pressure to give the crude as an off-white solid (0.40 g). A solution of crude (0.40 g) in 4 M HCl in 1,4-dioxane (4.0 mL) was stirred at room temperature for 16 h. After completion of the reaction, the reaction mixture was concentrated, neutralized using NH_4_OH (pH ∼ 8.0), and concentrated to give the crude, which was purified by reversed-phase chromatography using 0.1% NH_4_OH in water (20% MeCN/Water). The obtained pure fractions were lyophilized to afford the title compound **12a** as a white solid (60 mg, 0.17 mmol, 26% over 2 steps). **R**_**f**_ = 0.1 (10% MeOH/DCM); ^**1**^ **H NMR** (400 MHz, MeOD) δ [ppm] = 7.69 (s, 1H), 5.20–5.19 (m, 1H), 4.97 (brs, 1H), 4.22 (t, *J* = 6.4 Hz, 1H), 4.17 (dd, *J* = 4.4, 4.4 Hz, 1H), 3.64–3.59 (m, 1H), 3.43 (s, 6H), 2.51 (brs, 1H), 2.19 (brs, 2H), 1.99–1.96 (m, 1H), 1.63–1.60 (m, 1H); ^**13**^ **C­{** ^**1**^ **H}-NMR** (100 MHz, MeOD) δ [ppm] = 159.7, 155.2, 152.8, 135.3, 135.2, 119.8, 113.8, 74.9, 72.1, 70.4, 61.0, 52.3, 37.4, 31.0, 24.6; **HRMS** (ESI, 3.5 kV) *m*/*z*: [M + H] calc for C_16_H_22_N_6_O_3_H^+^ 347.1826; found 347.1820; **LCMS** *m*/*z* 347.3 (M + H) (ESI +ve), RT = 1.37 min; **HPLC** RT = 4.78 min 100%.

##### Synthesis of (1R,2S,3R,7R,7aR)-3-(2,6-Diamino-9*H*-purin-9-yl)-2,3,5,6,7,7a-hexahydro-1*H*-indene-1,2,7-triol (**20a**)

A sealed tube was charged with **33a** (0.4 g, 0.64 mmol, 1.0 equiv) in THF (4.0 mL) and NH_4_OH in H_2_O (4.0 mL) was added at room temperature. The resulting reaction mixture was heated to 120 °C for 16 h. After completion of the reaction, the reaction mixture was concentrated under reduced pressure to give the crude as a brown oil. (0.40 g). To the crude in 1,4-dioxane (4.0 mL) was added conc. HCl (8.0 mL) at room temperature. The resulting reaction mixture was stirred at room temperature for 16 h. After completion of the reaction, the reaction mixture was concentrated under reduced to give the crude, which was neutralized using NH_4_OH (pH ∼ 8.0), and concentrated to give the crude, which was purified by reversed-phase chromatography using 0.1% NH_4_OH in water (39% MeCN/Water). The pure fractions obtained were lyophilized to afford the title compound **20a** as a white solid (67 mg, 2.10 mmol, 32% over 2 steps). **R**_**f**_ = 0.1 (10% MeOH/DCM); ^**1**^ **H NMR** (400 MHz, MeOD) δ [ppm] = 7.79 (s, 1H), 5.19–5.18 (m, 1H), 5.00 (d, *J* = 2.4, 2.4 Hz, 1H), 4.26 (dd, *J* = 6.0, 6.0 Hz, 1H), 4.19 (dd, *J* = 4.4, 4.4 Hz, 1H), 3.66–3.60 (m, 1H), 2.52–2.50 (m, 1H) 2.24–2.14 (m, 2H), 2.00–1.96 (m, 1H), 1.66–1.57 (m, 1H); ^**13**^ **C­{** ^**1**^ **H}-NMR** (100 MHz, MeOD) δ [ppm] = 160.1, 156.0, 152.0, 137.1, 135.0, 120.0, 112.7, 74.9, 72.1, 70.4, 61.4, 52.3, 31.0, 24.6; **HRMS** (ESI, 3.5 kV) *m*/*z*: [M + H]^+^ calc for C_14_H_18_N_6_O_3_H^+^ 319.1513, found 319.1505; **LCMS** *m*/*z* 319.2 (M + H) (ESI +ve), RT = 1.99 min; **HPLC** RT = 4.21 min 100%.

##### Synthesis (1R,2S,3R,6S,7aS)-3-(2,6-Diamino-9*H*-purin-9-yl)-2,3,5,6,7,7a-hexahydro-1*H*-indene-1,2,6-triol (**20b**)

To a sealed tube charged with **Bz-33b** (0.40 g, 0.55 mmol, 1.0 equiv) in ethanol (4.0 mL) was added NH_4_OH (4.0 mL) at room temperature. The resulting reaction mixture was heated to 100 °C for 48 h. After completion of the reaction, the reaction mixture was concentrated under reduced pressure to afford the crude as a brown oil (0.4 g). To a stirred solution of the crude in 1,4-dioxane (0.8 mL) was added 4 M HCl in dioxane (4.0 mL) at room temperature. The resulting reaction mixture was stirred at room temperature for 16 h. After completion of the reaction, the reaction mixture was concentrated under reduced to give the crude, which was neutralized using NH_4_OH (pH ∼ 8.0), and concentrated to give the crude, which was purified by reversed-phase chromatography using 0.1% NH_4_OH in water (23% MeCN/Water). The pure fractions obtained were lyophilized to afford the title compound **20b** as a white solid (48 mg, 0.15 mmol, 27% over 2 steps). **R**_**f**_ = 0.2 (20% MeOH/DCM); ^**1**^ **H NMR** (400 MHz, MeOD) δ [ppm] = 7.75 (s, 1H), 5.13 (s, 2H), 4.22–4.19 (m, 1H), 4.00–3.90 (m, 2H), 2.71 (brs, 1H), 2.42–2.34 (m, 2H), 1.97–1.89 (m, 1H), 1.41 (dd, *J* = 11.6, 22.8 Hz, 1H). ^**13**^ **C­{** ^**1**^ **H}-NMR (**400 MHz, MeOD) δ [ppm] = 160.3, 156.1, 151.7, 137.3, 137.1, 120.1, 112.7, 76.0, 75.0, 66.8, 61.6, 43.9, 35.8, 33.9; **HRMS** (ESI, 3.5 kV) *m*/*z*: [M + H]^+^ calc for C_14_H_18_N_6_O_3_H^+^ 319.1513, found 319.1516; **LCMS** *m*/*z* 319.2 (M + H) (ESI +ve), RT = 1.78 min; **HPLC** RT = 4.59 min 97.82%.

##### Synthesis of (1R,2S,3R,6R,7aS)-3-(2,6-Diamino-9*H*-purin-9-yl)-2,3,5,6,7,7a-hexahydro-1*H*-indene-1,2,6-triol (**20c**)

A 100 mL sealed tube was charged with **Bz-33c** (0.35 g, 0.48 mmol, 1.0 equiv) in THF (3.5 mL) and NH_4_OH (3.5 mL) was added at room temperature. The resulting reaction mixture was heated to 120 °C for 3 days. After completion of the reaction, the reaction mixture was concentrated under reduced pressure to give the intermediate as a brown oil (0.35 g), which was redissolved in MeOH (3.5 mL). Then, NH_4_OH (3.5 mL) and NaOMe (0.16 g, 2.96 mmol, 6.0 equiv) were added at room temperature. The resulting reaction mixture was stirred at room temperature for 48 h. After completion of the reaction, the reaction mixture was concentrated under reduced pressure to give the crude as a brown oil (0.35 g). To a stirred solution of the crude in 1,4-dioxane (0.7 mL) was added 4 M HCl in dioxane (3.5 mL) at room temperature. The resulting reaction mixture was stirred at room temperature for 16 h. After completion of the reaction, the reaction mixture was neutralized using NH_4_OH (pH ∼ 8.0) and concentrated to give the crude, which was purified by reversed-phase chromatography using 0.1% NH_4_OH in water (100% water). The obtained pure fraction was lyophilized to afford the title compound **20c** as a white solid (41 mg, 0.13 mmol, 26% over 3 steps). **R**_**f**_ = 0.2 (20% MeOH/DCM); ^**1**^ **H NMR** (400 MHz, MeOD) δ [ppm] = 7.75 (s, 1H), 5.19 (brs, 1H), 5.10 (s, 1H), 4.23–4.20 (m, 2H), 4.01–3.98 (m, 1H), 2.83 (brs, 1H), 2.34–2.29 (m, 2H), 2.11–2.07 (m, 1H), 1.50 (dd, *J* = 2.0, 2.0 Hz, 1H); ^**13**^ **C­{** ^**1**^ **H}-NMR** (100 MHz, MeOD) δ [ppm] = 160.3, 156.1, 151.8, 137.2, 137.0, 118.7, 112.7, 75.6, 75.4, 63.7, 62.0, 37.9, 32.8, 32.5; **HRMS** (ESI, 3.5 kV) *m*/*z*: [M + H]^+^ calc for C_14_H_18_N_6_O_3_H^+^ 319.1513, found 319.1516; **LCMS** *m*/*z* 319.3 (M + H) (ESI +ve), RT = 1.77 min; **HPLC** RT = 4.64 min. 95.26%.

#### Synthesis of Cytidine-Type Analogues

##### Synthesis of 1-((3aR,3bS,4R,8R,8aS)-4-((*tert*-Butyldimethylsilyl)­oxy)-2,2-dimethyl-3a,3b,5,6,8,8a-hexahydro-4*H*-indeno­[1,2-*d*]­[1,3]­dioxol-8-yl)­pyrimidine-2,4­(1*H*,3*H*)-dione (**TBS-34a**)

To a stirred solution of **34a** (0.80 g, 2.50 mmol, 1.0 equiv) in DMF (8.0 mL) were added imidazole (0.37 g, 5.50 mmol, 2.2 equiv), TBDMS-Cl (0.56 g, 3.75 mmol, 1.5 equiv) at 0 °C. The resulting reaction mixture was stirred at room temperature for 16 h. After completion of the reaction, the reaction mixture was diluted with water (30 mL) and extracted with ethyl acetate (2 × 20 mL). The combined organic phase was washed with brine (20 mL), dried over anhydrous Na_2_SO_4_, filtered, and concentrated to give the crude, which was purified by column chromatography using silica gel (2% MeOH/DCM) to afford the title compound as a white solid (0.90 g, 2.07 mmol, 82%). **R**_**f**_ = 0.4 (10% MeOH/DCM). **LCMS** *m*/*z* 435.3 (M + H) (ESI +ve), RT = 2.77 min.

##### Synthesis of 4-Amino-1-((3aR,3bS,4R,8R,8aS)-4-((*tert*-butyldimethylsilyl)­oxy)-2,2-dimethyl-3a,3b,5,6,8,8a-hexahydro-4*H*-indeno­[1,2-*d*]­[1,3]­dioxol-8-yl)­pyrimidin-2­(1*H*)-one (**58**)

To a stirred solution of **TBS-34a** (0.40 g, 0.92 mmol, 1.0 equiv) in acetonitrile (4 mL) were added DMAP (11 mg, 0.09 mmol, 0.1 equiv), TEA (0.25 mL, 1.84 mmol, 2.0 equiv), and 2,4,6-triisopropylbenzenesulfonyl chloride **35** (0.55 g, 1.84 mmol, 2.0 equiv) at 0 °C. The resulting reaction mixture was stirred at room temperature for 2 h. After completion of the reaction, the reaction mixture was charged with NH_4_OH (4.0 mL) at room temperature. The resulting reaction mixture was stirred at room temperature for 16 h. After completion of the reaction, the reaction mixture was concentrated to give the crude, which was purified by column chromatography using silica gel (6% MeOH/DCM) to afford the title compound **58** as a white solid (0.30 g, 0.69 mmol, 75%). **R**_**f**_ = 0.35 (10% MeOH/DCM); **LCMS** *m*/*z* 434.3 (M + H) (ESI +ve), RT = 2.27 min.

##### Synthesis of 4-Amino-1-((1R,2S,3R,3aR,4R)-2,3,4-trihydroxy-2,3,3a,4,5,6-hexahydro-1*H*-inden-1-yl)­pyrimidin-2­(1*H*)-one (**4a**)

To a stirred solution of **58** (0.30 g, 0.69 mmol, 1.0 equiv) in THF (6. 0 mL) was added 10% TFA in water (30 mL) at room temperature. The resulting reaction mixture was stirred at room temperature for 24 h. After completion of the reaction, the reaction mixture was concentrated under reduced pressure to give the crude, which was purified by reversed-phase chromatography using 0.1% NH_4_OH in water (100% water). The enriched crude was further purified by RP Prep HPLC. The obtained pure fractions were lyophilized to afford the title compound **4a** as a white solid (90 mg, 0.32 mmol, 46%). **R**_**f**_ = 0.05 (10% MeOH/DCM); ^**1**^ **H NMR** (400 MHz, MeOD) δ [ppm] = 7.61 (d, *J* = 7.2 Hz, 1H), 5.99 (d, *J* = 7.6 Hz, 1H), 5.42 (brs, 1H), 5.19 (s, 1H), 4.10 (brs, 1H), 3.99 (brs, 1H), 3.62–3.56 (m, 1H), 2.45, (brs, 1H), 2.23 (brs, 2H), 1.99–1.95 (m, 1H), 1.65–1.57 (m, 1H); ^**13**^ **C­{** ^**1**^ **H}-NMR** (100 MHz, MeOD) δ [ppm] = 163.4, 154.8, 144.5, 134.3, 119.8, 94.6, 74.5, 72.1, 70.4, 63.0, 52.1, 30.9, 24.6; **HRMS** (ESI, 3.5 kV) *m*/*z*: [M + H]^+^ calc for C_13_H_17_N_3_O_4_H^+^ 280.1292, found 280.1291; **LCMS** *m*/*z* 280.4 (M + H) (ESI +ve), RT = 4.04 min; **HPLC** RT = 4.77 min 100%.

##### Synthesis of (3aR,3bS,5S,8R,8aS)-8-(2,4-Dioxo-3,4-dihydropyrimidin-1­(2*H*)-yl)-2,2-dimethyl-3a,3b,5,6,8,8a-hexahydro-4*H*-indeno­[1,2-*d*]­[1,3]­dioxol-5-yl Benzoate (**Bz-34b**)

To a stirred solution of **34b** (7.0 g, 21.9 mmol, 1.0 equiv) in a a mixture of DCM (35 mL) and pyridine (35 mL) was added benzoyl chloride (5.06 mL, 43.8 mmol, 2.0 equiv) at 0 °C. The resulting reaction mixture was stirred at room temperature for 4h. After completion of the reaction, the reaction mixture was diluted with sat. NaHCO_3_ (3 L) and extracted with DCM (3 × 500 mL). The combined organic phase was washed with brine (300 mL), dried over anhydrous Na_2_SO_4_, filtered, and dried under reduced pressure to give the crude, which was purified by column chromatography using silica gel (3% MeOH/DCM). The enriched crude was further purified by reversed-phase chromatography using 0.1% NH_4_OH in water (40% MeCN/water). The obtained pure fractions were lyophilized to afford the title compound **Bz-34b** as a white solid (3.5 g, 8.25 mmol, 37%). **R**_**f**_ = 0.45 (10% MeOH/DCM). ^**1**^ **H NMR** (400 MHz, CDCl_3,_ 42 °C) δ [ppm] = 8.27 (s, 1H), 8.04 (dd, *J* = 5.2, 6.8 Hz, 2H), 7.60–7.56 (m, 1H), 7.46 (dd, *J* = 8.0, 8.0 Hz, 2H), 7.14 (brs, 1H), 5.76 (d, *J* = 7.6 Hz, 1H), 5.35–5.26 (m, 2H), 4.76 (brs, 1H), 4.46 (brs, 1H), 2.81 (brs, 1H), 2.68–2.60 (m, 2H), 2.28–2.23 (m, 1H), 1.66–1.60 (m, 1H), 1.58 (s, 3H), 1.35 (s, 3H); ^**13**^ **C­{** ^**1**^ **H}-NMR** (100 MHz, CD_3_CN) δ [ppm] = 165.6, 151.5, 142.4, 139.3, 133.1, 130.5, 129.2, 128.5, 119.5, 117.3, 113.2, 102.4, 83.9, 82.8, 70.2, 61.0, 44.1, 32.1, 30.0, 26.7, 24.5; **HRMS** (ESI, 3.5 kV) *m*/*z*: [M + H]^+^ calc for C_23_H_24_N_2_O_6_H^+^ 425.1707, found 425.1705; **LCMS** *m*/*z* 367.3 (M-57) (ESI +ve), RT = 2.08 min; **HPLC** RT = 7.56 min 100%.

##### Synthesis of (3aR,3bS,5S,8R,8aS)-8-(4-Amino-2-oxopyrimidin-1­(2*H*)-yl)-2,2-dimethyl-3a,3b,5,6,8,8a-hexahydro-4*H*-indeno­[1,2-*d*]­[1,3]­dioxol-5-yl Benzoate (**59**)

To a stirred solution of **Bz-34b** (0.30 g, 0.70 mmol, 1.0 equiv) in acetonitrile (3.0 mL) were added DMAP (8 mg, 0.07 mmol, 0.1 equiv), TEA (0.19 mL, 1.41 mmol, 2.0 equiv), and 2,4,6-triisopropylbenzenesulfonyl chloride **35** (0.42 g, 1.41 mmol, 2.0 equiv) at 0 °C. The resulting reaction mixture was stirred at room temperature for 2 h. After completion of the reaction, the reaction mixture was charged with NH_4_OH (3.0 mL) at room temperature. The resulting reaction mixture was stirred at room temperature for 16 h. After completion of the reaction mixture was concentrated to give the crude, which was purified by column chromatography using silica gel (3% MeOH/DCM) to afford the title compound **59** as a white solid (0.24 g, 0.56 mmol, 80%). **R**_**f**_ = 0.35 (10% MeOH/DCM); **LCMS** *m*/*z* 424.3 (M + H) (ESI +ve), RT = 2.01 min.

##### Synthesis of 4-Amino-1-((1R,2S,3R,3aS,5S)-2,3,5-trihydroxy-2,3,3a,4,5,6-hexahydro-1*H*-inden-1-yl)­pyrimidin-2­(1*H*)-one (**4b**)

To a stirred solution of **59** (0.24 g, 0.56 mmol, 1.0 equiv) in THF (4.8 mL) and 10% TFA in water (24 mL) was added at room temperature. The resulting reaction mixture was stirred at room temperature for 16 h. After completion of the reaction, the reaction mixture was concentrated to give the crude as a colorless oil (0.2 g). To a stirred solution of the crude in methanol (4.0 mL) was added NaOMe (0.14 g, 2.61 mmol) at 0 °C. The resulting reaction mixture was stirred at room temperature for 16 h. After completion of the reaction, the reaction mixture was concentrated to give the crude, which was purified by reversed-phase chromatography using 0.1% NH_4_OH in water (100% water). The enriched crude was further purified by RP Prep HPLC. The obtained pure fractions were lyophilized to afford the title compound **4b** as a white solid (90 mg, 0.32 mmol, 57% over 2 steps). **R**_**f**_ = 0.05 (10% MeOH/DCM); ^**1**^ **H NMR** (400 MHz, MeOD) δ [ppm] = 7.45 (brs, 1H), 5.90 (d, *J* = 7.2 Hz, 1H), 5.19 (brs, 1H), 5.00–4.95 (m, 1H), 4.46–3.71 (m, 3H), 2.63 (brs, 1H), 2.44–2.31 (m, 2H), 1.98–1.91 (m, 1H), 1.35 (dd, *J* = 11.2, 22.8 Hz, 1H); ^**13**^ **C­{** ^**1**^ **H}-NMR** (100 MHz, MeOD) δ [ppm] = 166.4, 157.9, 143.8, 137.3, 119.6, 95.0, 81.1, 75.8, 75.4, 66.8, 43.8, 35.8, 33.9. **HRMS** (ESI, 3.5 kV) *m*/*z*: [M + H]^+^ calc for C_13_H_17_N_3_O_4_H^+^ 280.1292, found 280.1292; **LCMS** *m*/*z* 278.3 (M – H) (ESI -ve), RT = 3.97 min; **HPLC** RT = 4.02 min. 100%. Note: Peak broadening observed in NMR at room temperature.

##### Synthesis of (3aR,3bS,5R,8R,8aS)-8-(2,4-Dioxo-3,4-dihydropyrimidin-1­(2*H*)-yl)-2,2-dimethyl-3a,3b,5,6,8,8a-hexahydro-4*H*-indeno­[1,2-*d*]­[1,3]­dioxol-5-yl Benzoate (**Bz-34c**)

To a stirred solution of **34c** (9.2 g, 28.8 mmol, 1.0 equiv) in a mixture of DCM (45.5 mL) and pyridine (45.5 mL) was added benzoyl chloride (6.70 mL, 57.5 mmol, 2 equiv) at 0 °C. The resulting reaction mixture was stirred at room temperature for 4h. After completion of the reaction, the reaction mixture was diluted with sat. NaHCO_3_ (300 mL) and extracted with DCM (3 × 50 mL). The combined organic phase was washed with brine (100 mL), dried over anhydrous Na_2_SO_4_, filtered, and concentrated under reduced pressure to give the crude, which was purified by column chromatography using silica gel (3% MeOH/DCM). The enriched crude was further purified by reversed-phase chromatography using 0.1% NH_4_OH in water (38% MeCN/water). The obtained pure fractions were lyophilized to afford the title compound **Bz-34c** as a white solid (3.0 g, 7.07 mmol, 24%). **R**_**f**_ = 0.35 (10% MeOH/DCM); ^**1**^ **H NMR** (400 MHz, CDCl_3_, 42 °C) δ [ppm] = 8.35 (s, 1H), 8.03 (d, *J* = 7.2 Hz, 2H), 7.58 (t, *J* = 7.6 Hz, 1H), 7.46 (t, *J* = 7.6 Hz, 1H), 7.13 (brs, 1H), 5.77 (d, *J* = 7.2 Hz, 1H), 5.54 (s, 1H), 5.32 (s, 1H), 4.45 (brs, 1H), 2.85 (brs, 1H), 2.65–2.61 (m, 1H), 2.53–2.36 (m, 2H), 1.58 (s, 4H), 1.36 (s, 3H); ^**13**^ **C­{** ^**1**^ **H}-NMR** (100 MHz, CD_3_CN) δ [ppm] = 165.6, 151.5, 142.5, 139.1, 133.1, 130.7, 129.3, 128.6, 118.5, 117.4, 113.4, 102.4, 83.7, 83.5, 68.0, 61.6, 39.5, 30.3, 29.6, 26.6, 24.3; **HRMS** (ESI, 3.5 kV) *m*/*z*: [M + H]^+^ calc for C_23_H_24_N_2_O_6_H^+^ 425.1707, found 425.1707; **LCMS** *m*/*z* 425.3 (M + H) (ESI +ve), RT = 2.01 min; **HPLC** RT = 7.19 min 97.2%.

##### Synthesis of (3aR,3bS,5R,8R,8aS)-8-(4-Amino-2-oxopyrimidin-1­(2*H*)-yl)-2,2-dimethyl-3a,3b,5,6,8,8a-hexahydro-4*H*-indeno­[1,2-*d*]­[1,3]­dioxol-5-yl Benzoate (**60**)

To a stirred solution of **Bz-34c** (0.30 g, 0.70̀mmol, 1.0 equiv) in acetonitrile (3.0 mL) were added DMAP (8 mg, 0.07 mmol, 0.1 equiv), TEA (0.19 mL, 1.41 mmol, 2.0 equiv), and 2,4,6-triisopropylbenzenesulfonyl chloride **35** (0.42 g, 1.41 mmol, 2.0 equiv) at 0 °C. The resulting reaction mixture was stirred at room temperature for 2 h. After completion of the reaction, the reaction mixture was charged with NH_4_OH (3.0 mL) at room temperature. The resulting reaction mixture was stirred at room temperature for 16 h. After completion of the reaction, the reaction mixture was concentrated to give the crude, which was purified by column chromatography using silica gel (3% MeOH/DCM) to afford the title compound **60** as a white solid (0.23 g, 0.54 mmol, 77%). **R**_**f**_ = 0.35 (10% MeOH/DCM). **HRMS** (ESI, 3.5 kV) *m*/*z*: [M + H]^+^ calc for C_23_H_25_N_3_O_5_H^+^ 424.1867, found 424.1862; **LCMS** *m*/*z* 424.3 (M + H)^+^ (ESI +ve), RT = 1.84 min.

##### Synthesis of 4-Amino-1-((1R,2S,3R,3aS,5R)-2,3,5-trihydroxy-2,3,3a,4,5,6-hexahydro-1*H*-inden-1-yl)­pyrimidin-2­(1*H*)-one (**4c**)

To a stirred solution of **60** (0.23 g, 0.54 mmol, 1.0 equiv) in THF (4.6 mL) and 10% TFA in water (23 mL) was added at room temperature. The resulting reaction mixture was stirred at room temperature for 16 h. After completion of the reaction, the reaction mixture was concentrated to give the crude as a colorless oil (0.18 g). To a stirred solution of the crude in methanol (3.6 mL) was added NaOMe (0.12 g, 2.34 mmol) at 0 °C. The resulting reaction mixture was stirred at room temperature for 16 h. After completion of the reaction, the reaction mixture was concentrated under reduced pressure to give the crude, which was purified by reversed-phase chromatography using 0.1% NH_4_OH in water (100% water). The enriched crude was further purified by RP Prep HPLC. The obtained pure fractions were lyophilized to afford the title compound **4c** as a white solid (35 mg, 0.12 mmol, 22% over 2 steps). **R**_**f**_ = 0.1 (20% MeOH/DCM); ^**1**^ **H NMR** (400 MHz, MeOD) δ [ppm] = 7.47 (brs, 1H), 5.89 (d, *J* = 7.2 Hz, 1H), 5.49 (brs, 1H), 5.16 (brs, 1H), 4.18 (s, 1H), 4.06 (brs, 1H), 3.88 (brs, 1H), 2.72 (brs, 1H), 2.35–2.24 (m, 2H), 2.13–2.08 (m, 1H), 1.42 (dd, *J* = 11.2, 11.2 Hz, 1H); ^**13**^ **C­{** ^**1**^ **H}-NMR** (100 MHz, MeOD) δ [ppm] = 166.0, 157.6, 144.1, 137.0, 94.6, 75.8, 75.3, 63.7, 37.8, 32.7, 32.5; **HRMS** (ESI, 3.5 kV) *m*/*z*: [M + Na]^+^ calc for C_13_H_17_N_3_O_4_Na^+^ 302.1111, found 302.1111; **LCMS** *m*/*z* 280.3 (M + H) (ESI +ve), RT = 4.22 min; **HPLC** RT = 4.38 min 100%. Note: Peak broadening observed in NMR at room temperature, and not all C signals could be identified.

##### Synthesis of 1-((3aR,3bS,4R,8R,8aS)-4-((*tert*-Butyldimethylsilyl)­oxy)-2,2-dimethyl-3a,3b,5,6,8,8a-hexahydro-4*H*-indeno­[1,2-*d*]­[1,3]­dioxol-8-yl)-4-(cyclopropylamino)­pyrimidin-2­(1*H*)-one (**61**)

To a stirred solution of **TBS-34a** (0.40 g, 0.92 mmol, 1.0 equiv) in acetonitrile (4.0 mL) were added DMAP (11 mg, 0.09 mmol, 0.1 equiv), TEA (0.25 mL, 1.84 mmol, 2.0 equiv), and 2,4,6-triisopropylbenzenesulfonyl chloride **35** (0.55 g, 1.84 mmol, 2.0 equiv) at 0 °C. The resulting reaction mixture was stirred at room temperature for 2 h. After completion of the reaction, the reaction mixture was charged with cyclopropylamine (4.0 mL) at room temperature. The resulting reaction mixture was stirred at room temperature for 16 h. After completion of the reaction, the reaction mixture was concentrated to give the crude, which was purified by column chromatography using silica gel (2% MeOH/DCM) to afford the title compound **61** as a white solid (0.30 g, 0.63 mmol 69%). **R**_**f**_ = 0.4 (10% MeOH/DCM); **LCMS** *m*/*z* 474.3 (M + H) (ESI +ve), RT = 2.45 min.

##### Synthesis of 4-(Cyclopropylamino)-1-((1R,2S,3R,3aR,4R)-2,3,4-trihydroxy-2,3,3a,4,5,6-hexahydro-1*H*-inden-1-yl)­pyrimidin-2­(1*H*)-one (**17a**)

To a stirred solution of **61** (0.30 g, 0.63 mmol, 1.0 equiv) in THF (6.0 mL) and was added 10% TFA in water (30 mL) at room temperature. The resulting reaction mixture was stirred at room temperature for 24 h. After completion of the reaction, the reaction mixture was concentrated to give the crude, which was purified by reversed-phase chromatography using 0.1% NH_4_OH in water (10% MeCN/water). The obtained pure fractions were lyophilized to afford the title compound **17a** as a white solid (0.13 g, 0.40 mmol, 64%). **R**_**f**_ = 0.1 (10% MeOH/DCM); ^**1**^ **H NMR** (400 MHz, DMSO, 75 °C) δ [ppm] = 7.45 (s, 1H), 7.33 (brs, 1H), 5.76 (brs, 1H) 5.23 (brs, 1H), 4.93 (dd, *J* = 2.8, 2.8 Hz, 1H), 4.62 (d, *J* = 5.6 Hz, 1H), 4.44 (d, *J* = 4.4 Hz, 1H), 4.37 (d, *J* = 4.8 Hz, 1H), 3.93 (dd, *J* = 5.2, 10.4 Hz, 1H), 3.82 (dd, *J* = 6.0, 12.4 Hz, 1H), 3.45–3.40 (m, 1H), 3.75 (brs, 1H), 2.65 (brs, 1H), 2.28–2.26 (m, 1H), 2.12–2.05 (m, 2H), 1.84–1.79 (m, 1H), 1.47–1.41 (m, 1H), 0.74–0.69 (m, 2H), 0.50–0.47 (m, 2H); ^**13**^ **C­{** ^**1**^ **H}-NMR** (100 MHz, DMSO) δ [ppm] = 164.6, 156.8, 142.1, 137.1, 117.8, 94.5, 91.3, 74.0, 71.4, 69.7, 62.4, 53.0, 31.9, 25.0, 23.8, 6.6; **HRMS** (ESI, 3.5 kV) *m*/*z*: [M + H]^+^ calc for C_16_H_21_N_3_O_4_H^+^ 320.1605, found 320.1603; **LCMS** *m*/*z* 320.4 (M + H) (ESI +ve), RT = 1.86 min; **HPLC** RT = 1.89 min 100%. Note: Rotamers observed at room temperature.

##### Synthesis of (3aR,3bS,5S,8R,8aS)-8-(4-(Cyclopropylamino)-2-oxopyrimidin-1­(2*H*)-yl)-2,2-dimethyl-3a,3b,5,6,8,8a-hexahydro-4*H*-indeno­[1,2-*d*]­[1,3]­dioxol-5-yl Benzoate (**62**)

To a stirred solution of **Bz-34b** (0.30 g, 0.70 mmol, 1.0 equiv) in acetonitrile (3.0 mL) were added DMAP (8 mg, 0.07 mmol, 0.1 equiv), TEA (0.19 mL, 1.41 mmol, 2.0 equiv), and 2,4,6-triisopropylbenzenesulfonyl chloride **35** (0.42 g, 1.41 mmol, 2.0 equiv) at 0 °C. The resulting reaction mixture was stirred at room temperature for 2 h. After completion of the reaction, the reaction mixture was charged with cyclopropylamine (3.0 mL) at room temperature. The resulting reaction mixture was stirred at room temperature for 16 h. After completion of the reaction, the reaction mixture was concentrated to give the crude, which was purified by column chromatography using silica gel (3% MeOH/DCM) to afford the title compound **62** as a white solid (0.30 g, 0.64 mmol, 91%). **R**_**f**_ = 0.30 (10% MeOH/DCM); **LCMS** *m*/*z* 464.2 (M + H) (ESI +ve), RT = 1.96 min.

##### Synthesis of 4-(Cyclopropylamino)-1-((1R,2S,3R,3aS,5S)-2,3,5-trihydroxy-2,3,3a,4,5,6-hexahydro-1*H*-inden-1-yl)­pyrimidin-2­(1*H*)-one (**17b**)

To a stirred solution of benzoate **62** (0.30 g, 0.64 mmol, 1.0 equiv) in THF (6 mL) was added 10% TFA in water (30 mL) at room temperature. The resulting reaction mixture was stirred at room temperature for 16 h. After completion of the reaction, the reaction mixture was concentrated to give the crude as a brown oil. To a stirred solution of the crude (0.20 g) in methanol (4 mL) was added NaOMe (0.13 g, 2.36 mmol) at 0 °C. The resulting reaction mixture was stirred at room temperature for 16 h. After completion of the reaction, the reaction mixture was concentrated to give the crude, which was purified by RP Prep HPLC using 0.05% NH_3_ in water. The enriched crude was further purified by chiral HPLC using 0.1% methanolic ammonia in methanol: acetonitrile (50:50). The obtained pure fractions were concentrated and lyophilized to afford the title compound **17b** as a white solid (80 mg, 0.25 mmol, 39% over 2 steps). **R**_**f**_ = 0.05 (10% MeOH/DCM); ^**1**^ **H NMR** (400 MHz, DMSO, 75 °C) δ [ppm] = 7.47 (brs, 1H), 7.34 (d, *J* = 15.6 Hz 1H), 5.74 (brs, 1H), 5.11 (brs, 1H), 5.00 (s, 1H), 4.63 (brs, 1H), 4.48 (s, 1H), 3.86 (brs, 1H), 3.73–3.68 (m, 2H), 2.76 (brs, 1H), 2.44 (brs, 1H), 2.30 (brs, 1H), 2.18–2.15 (m, 1H), 1.83–1.77 (m, 1H), 1.19–1.13 (m, 1H), 0.72 (d, *J* = 5.6 Hz, 2H), 0.49 (brs, 2H); ^**13**^ **C­{** ^**1**^ **H}-NMR** (100 MHz, MeOD) δ [ppm] = 137.4, 95.6, 75.9, 75.5, 66.8, 43.8, 35.8, 33.9, 23.1, 6.3, 5.6; **HRMS** (ESI, 3.5 kV) *m*/*z*: [M + H]^+^ calc for C_16_H_21_N_3_O_4_H^+^ 320.1605, found 320.1603; **LCMS** *m*/*z* 320.3 (M + H) (ESI +ve), RT = 4.96 min; **HPLC** RT = 5.26 min, 100%. Note: Rotamers observed at room temperature, leading to peak broadening in H/C NMR.

##### Synthesis of (3aR,3bS,5R,8R,8aS)-8-(4-(Cyclopropylamino)-2-oxopyrimidin-1­(2*H*)-yl)-2,2-dimethyl-3a,3b,5,6,8,8a-hexahydro-4*H*-indeno­[1,2-*d*]­[1,3]­dioxol-5-yl Benzoate (**63**)

To a stirred solution of **Bz-34c** (0.30 g, 0.70 mmol, 1.0 equiv) in acetonitrile (3.0 mL) were added DMAP (8 mg, 0.07 mmol, 0.1 equiv), TEA (0.19 mL, 1.41 mmol, 2.0 equiv), and 2,4,6-triisopropylbenzenesulfonyl chloride **35** (0.42 g, 1.41 mmol, 2.0 equiv) at 0 °C. The resulting reaction mixture was stirred at room temperature for 2 h. After completion of the reaction, the reaction mixture was charged with cyclopropylamine (3.0 mL) at room temperature. The resulting reaction mixture was stirred at room temperature for 16 h. After completion of the reaction, the reaction mixture was concentrated to give the crude, which was purified by column chromatography using silica gel (3% MeOH/DCM) to afford the title compound **63** as a white solid (0.31 g, 0.66 mmol, 94%). **R**_**f**_ = 0.3 (10% MeOH/DCM); **HRMS** (ESI, 3.5 kV) *m*/*z*: [M + H]^+^ calc for C_26_H_29_N_3_O_5_H^+^ 464.2180, found 464.2179; **LCMS** *m*/*z* 464.3 (M + H) (ESI +ve), RT = 1.90 min.

##### Synthesis of 4-(Cyclopropylamino)-1-((1R,2S,3R,3aS,5R)-2,3,5-trihydroxy-2,3,3a,4,5,6-hexahydro-1*H*-inden-1-yl)­pyrimidin-2­(1*H*)-one (**17c**)

To a stirred solution of **63** (0.30 g, 0.64 mmol, 1.0 equiv) in THF (6 mL) was added 10% TFA in water (30 mL) at room temperature. The resulting reaction mixture was stirred at room temperature for 16 h. After completion of the reaction, the reaction mixture was concentrated to give the crude as a yellowish oil. To a stirred solution of the crude (0.25 g) in methanol (5.0 mL) was added NaOMe (0.16 g, 2.95 mmol) at 0 °C. The resulting reaction mixture was stirred at room temperature for 16 h. After completion of the reaction, the reaction mixture was concentrated to give the crude, which was purified by reversed-phase chromatography using 0.1% NH_4_OH in water (100% water). The enriched crude was further purified by RP Prep HPLC. The obtained pure fractions were lyophilized to afford the title compound **17c** as a white solid (45 mg, 0.14 mmol, 21% over 2 steps). **R**_**f**_ = 0.05 (10% MeOH/DCM); ^**1**^ **H NMR** (400 MHz, DMSO, 75 °C) δ [ppm] = 7.45 (brs, 1H), 7.31 (brs, 1H), 5.74 (brs, 1H), 5.18 (brs, 1H), 4.96 (s, 1H), 4.62 (d, *J* = 4.4 Hz, 1H), 4.50 (d, *J* = 6.0 Hz, 1H), 4.27 (d, *J* = 3.2 Hz, 1H), 4.01 (brs, 1H), 3.86 (d, *J* = 4.8 Hz, 1H), 3.70 (dd, *J* = 6.0, 13.2 Hz, 1H), 2.76 (brs, 1H), 2.56 (brs, 1H), 2.20–2.16 (m, 1H), 2.08–2.04 (m, 1H), 1.98–1.93 (m, 1H), 1.25 (dd, *J* = 12.0, 12.0 Hz, 1H), 0.72 (dd, *J* = 6.4, 12.0 Hz, 2H), 0.48 (brs, 2H); ^**13**^ **C­{** ^**1**^ **H}-NMR** (100 MHz, MeOD) δ [ppm] = 167.0, 165.1, 137.1, 117.9, 95.7, 75.8, 75.5, 63.7, 37.8, 32.7, 32.5, 23.1, 23.0, 6.3, 5.7; **HRMS** (ESI, 3.5 kV) *m*/*z*: [M + H]^+^ calc for C_16_H_21_N_3_O_4_H^+^ 320.1605, found 320.1606; **LCMS** *m*/*z* 320.2 (M + H) (ESI +ve), RT = 1.85 min; **HPLC** RT = 5.51 min, 100%.

#### Synthesis of NHC-Type Analogues

##### Synthesis of 1-((3aR,3bS,4R,8R,8aS)-4-((*tert*-Butyldimethylsilyl)­oxy)-2,2-dimethyl-3a,3b,5,6,8,8a-hexahydro-4*H*-indeno­[1,2-*d*]­[1,3]­dioxol-8-yl)-4-(hydroxyimino)-3,4-dihydropyrimidin-2­(1*H*)-one (**64**)

To a stirred solution of **TBS-34a** (0.40 g, 0.92 mmol, 1.0 equiv) in MeCN (20.0 mL) were added 1,2,4-triazole (0.76 g, 11.1 mmol, 12 equiv), TEA (1.92 mL, 13.8 mmol, 15.0 equiv), and POCl_3_ (0.34 mL, 3.68 mmol, 4.0 equiv) at 0 °C. The resulting reaction mixture was stirred at room temperature for 2 h. After completion of the reaction, the reaction mixture was concentrated to give the crude product. The obtained crude was dissolved in pyridine (4.0 mL), and NH_2_OH HCl (0.25 g, 3.68 mmol, 4.0 equiv) was added at 0 °C. The resulting reaction mixture was then stirred at room temperature for 16 h. After completion of the reaction, the reaction mixture was diluted with water (30 mL) and extracted with ethyl acetate (2 × 20 mL). The combined organic phase was washed with brine (20 mL), dried over anhydrous Na_2_SO_4_, filtered, and concentrated to give the crude, which was purified by reversed-phase chromatography using 0.1% NH_4_OH in water (60% MeCN/Water). The obtained pure fractions were lyophilized to afford the title compound **64** as a white solid (0.20 g, 0.44 mmol, 48%). **R**_**f**_ = 0.2 (10% MeOH/DCM); ^**1**^ **H NMR** (400 MHz, CDCl_3_) δ [ppm] = 6.41 (brs, 1H), 5.64 (brs, 1H), 5.53 (brs, 1H), 5.28 (brs, 1H), 4.54–4.40 (brs, 2H), 3.59 (brs, 1H), 2.60 (brs, 1H), 2.19–2.11 (brs, 2H), 1.90 (m, 1H), 1.87 (brs, 1H), 1.62 (brs, 1H), 1.56 (s, 3H), 1.34 (s, 3H), 0.94 (s, 9H), 0.12 (s, 6H); **LCMS** *m*/*z* 450.3 (M + H) (ESI +ve), RT = 2.71 min. *Note: Peak broadening observed due to the formation of a conformer or rotamer.*

##### Synthesis of 4-(Hydroxyimino)-1-((1R,2S,3R,3aR,4R)-2,3,4-trihydroxy-2,3,3a,4,5,6-hexahydro-1*H*-inden-1-yl)-3,4-dihydropyrimidin-2­(1*H*)-one (**3a**)

To a stirred solution of **64** (0.20 g, 0.44 mmol, 1.0 equiv) in THF (4.0 mL) was added 10% TFA in water (20 mL) at room temperature. The resulting reaction mixture was stirred at room temperature for 24 h. After completion of the reaction, the reaction mixture was concentrated under reduced pressure to give the crude, which was purified by reversed-phase chromatography using 0.1% NH_4_OH in water. The enriched crude was further purified by RP Prep HPLC. The obtained pure fractions were lyophilized to afford the title compound **3a** as a white solid (80 mg, 0.27 mmol, 61%). **R**_**f**_ = 0.05 (10% MeOH/DCM); ^**1**^ **H NMR** (400 MHz, MeOD) δ [ppm] = 6.70 (d, *J* = 8.0 Hz, 1H), 5.60 (d, *J* = 8.0 Hz, 1H), 5.28 (brs, 2H), 4.03 (brs, 1H), 3.91 (brs, 1H), 3.59–3.53 (m, 1H), 2.40 (brs, 1H), 2.53 (brs, 2H), 1.98–1.95 (m, 1H), 1.61–1.56 (m, 1H); ^**13**^ **C­{** ^**1**^ **H}-NMR** (100 MHz, MeOH) δ [ppm] = 151.2, 145.5, 133.7, 132.3, 119.8, 97.6, 51.7, 73.8, 72.5, 70.6, 30.9, 24.5; **HRMS** (ESI, 3.5 kV) *m*/*z*: [M + H]^+^ calc for C_13_H_17_N_3_O_5_H^+^ 296.1241, found 296.1242; **LCMS** *m*/*z* 296.8 (M + H) (ESI +ve), RT = 5.05 min; **HPLC** RT = 4.81 min. 100%. *Note: One C signal could not be observed in 13C-NMR possibly due to peak broadening or overlapping.*

##### Synthesis of 4-(Hydroxyimino)-1-((1R,2S,3R,3aS,5S)-2,3,5-trihydroxy-2,3,3a,4,5,6-hexahydro-1*H*-inden-1-yl)-3,4-dihydropyrimidin-2­(1*H*)-one (**3b**)

To a stirred solution of **34b** (0.35 g, 1.09 mmol, 1.0 equiv) in HMDS (7. 0 mL) were added imidazole (37 mg, 0.54 mmol, 0.5 equiv), ammonium bisulfate (0.50 g, 4.37 mmol, 4.0 equiv), and hydroxylamine sulfate (0.35 g, 2.18 mmol, 2.0 equiv) at 0 °C. The resulting reaction mixture was heated to 85 °C for 16 h. After completion of the reaction, the reaction mixture was diluted with water (20 mL) and extracted with ethyl acetate (2 × 20 mL). The combined organic phase was washed with brine (20 mL), dried over anhydrous Na_2_SO_4_, filtered, and concentrated to give the crude. The obtained crude was dissolved in methanol (7 mL), and TFA (1.75 mL) was added at 0 °C. The resulting reaction mixture was stirred at room temperature for 24 h. After completion of the reaction, the reaction mixture was concentrated under reduced pressure to give the crude, which was purified by reversed-phase chromatography using 0.1% NH_4_OH in water (100% water). The enriched crude was further purified by RP Prep HPLC (see SI for details). The obtained pure fractions were lyophilized to afford the title compound **3b** as a white solid (60 mg, 0.20 mmol, 19%). **R**_**f**_ = 0.05 (10% MeOH/DCM); ^**1**^ **H NMR** (400 MHz, MeOD) δ [ppm] = 6.70 (brs, 1H), 5.61 (d, *J* = 8.0 Hz, 1H), 5.33 (brs, 1H), 5.16 (brs, 1H), 3.99 (brs, 1H), 3.95–3.87 (m, 1H), 3.76 (brs, 1H), 2.59, (brs, 1H), 2.46–2.42 (m, 1H), 2.35–2.30 (m, 1H), 2.00–1.93 (m, 1H) 1.31 (dd, *J* = 11.6, 22.8 Hz, 1H); ^**13**^ **C­{** ^**1**^ **H}-NMR** (100 MHz, MeOD) δ [ppm] = 150.8, 145.9, 136.3 119.3, 97.3, 75.4, 75.2, 66.8, 43.6, 35.8, 33.9; **HRMS** (ESI, 3.5 kV) *m*/*z*: [M + H]^+^ calc for C_13_H_17_N_3_O_5_H^+^ 296.1241, found 296.1261; **LCMS** *m*/*z* 296.3 (M + H) (ESI +ve), RT = 4.38 min; **HPLC** RT = 4.13 min, 100%. *Note: Two carbon signals could not be detected due to peak broadening.*

##### Synthesis of 1-((3aR,3bS,5R,8R,8aS)-5-Hydroxy-2,2-dimethyl-3a,3b,5,6,8,8a-hexahydro-4*H*-indeno­[1,2-*d*]­[1,3]­dioxol-8-yl)-4-(hydroxyimino)-3,4-dihydropyrimidin-2­(1*H*)-one (**65**)

To a stirred solution of **34c** (0.35 g, 1.09 mmol, 1.0 equiv) in HMDS (7.0 mL) were added imidazole (0.037 g, 0.54 mmol, 0.5 equiv), ammonium bisulfate (0.50 g, 4.37 mmol, 4.0 equiv), and hydroxylamine sulfate (0.35 g, 2.18 mmol, 2.0 equiv) at room temperature. The resulting reaction mixture was heated to 85 °C for 16 h. After completion of the reaction, the reaction mixture was diluted with water (30 mL) and extracted with ethyl acetate (2 × 20 mL). The combined organic phase was washed with brine (20 mL), dried over anhydrous Na_2_SO_4_, filtered, and concentrated to give the crude, which was purified by column chromatography using silica gel (6% MeOH/DCM) to afford the title compound **65** as a white solid (0.3 g, 0.89 mmol, 82%). **R**_**f**_ = 0.30 (10% MeOH/DCM); **HRMS** (ESI, 3.5 kV) *m*/*z*: [M + H]^+^ calc for C_16_H_21_N_3_O_5_H 336.1554, found 336.1553; **LCMS** *m*/*z* 336.3 (M + H) (ESI +ve), RT = 1.45 min.

##### Synthesis of 4-(Hydroxyimino)-1-((1R,2S,3R,3aS,5R)-2,3,5-trihydroxy-2,3,3a,4,5,6-hexahydro-1*H*-inden-1-yl)-3,4-dihydropyrimidin-2­(1*H*)-one (**3c**)

To a stirred solution of **65** (0.30 g, 0.89 mmol, 1.0 equiv) in methanol (3.0 mL) was added TFA (1.5 mL) at 0 °C. The resulting reaction mixture was then stirred at room temperature for 24 h. After completion of the reaction, the reaction mixture was concentrated to give the crude, which was purified by reversed-phase chromatography using 0.1% NH_4_OH in water (100% water). The enriched crude was further purified by RP Prep HPLC (see SI for details), and the obtained pure fractions were lyophilized to afford the title compound **3c** as a white solid (0.17 g, 0.57 mmol, 64%). **R**_**f**_ = 0.05 (10% MeOH/DCM); ^**1**^ **H NMR** (400 MHz, MeOD) δ [ppm] = 6.71 (d, *J* = 8.0 Hz, 1H), 5.60 (d, *J* = 8.4 Hz, 1H), 5.30 (s, 1H), 5.14 (brs, 1H), 4.18 (s, 1H), 4.00 (brs, 1H), 3.78 (brs, 1H), 2.69, (brs, 1H), 2.39–2.32 (m, 2H), 2.28–2.11 (m, 1H), 1.39 (dd, *J* = 11.2, 11.2 Hz, 1H); ^**13**^ **C­{** ^**1**^ **H}-NMR** (100 MHz, MeOD) δ [ppm] = 150.9, 145.8, 136.1 117.7, 97.2, 75.7, 74.7, 63.7, 37.7, 32.8, 32.5; **HRMS** (ESI, 3.5 kV) *m*/*z*: [M + H]^+^ calc for C_13_H_17_N_3_O_5_H^+^ 296.1241, found 296.1241; **LCMS** *m*/*z* 296.1 (M + H) (ESI +ve), RT = 1.58 min; **HPLC** RT = 4.12 min, 100%. *Note: Two carbon signals could not be detected due to peak broadening.*

#### Synthesis of 5-Bromo Uracil Analogue

##### Synthesis of 5-Bromo-1-((1R,2S,3R,3aR,4R)-2,3,4-trihydroxy-2,3,3a,4,5,6-hexahydro-1*H*-inden-1-yl)­pyrimidine-2,4­(1*H*,3*H*)-dione (**13a**)

To a stirred solution of **2a** (0.3 g, 1.07 mmol, 1.0 equiv) in 1,2-dimethoxyethane (195 mL) was dropwise added sodium azide solution (0.29 g, 4.43 mmol, 4.0 equiv) in water (1.35 mL) at room temperature. After 30 min, the reaction mixture was charged with N-bromosuccinimide (0.21 g, 1.18 mmol, 1.1 equiv) at room temperature. The resulting reaction mixture was stirred at room temperature for 16 h. After completion of the reaction, the reaction mixture was concentrated under reduced pressure to get the crude, which was purified by RP Prep HPLC. The obtained pure fractions were lyophilized to afford the title compound **13a** as a white solid (8 mg, 0.02 mmol, 2% Yield). **R**_**f**_ = 0.2 (10% MeOH/DCM); ^**1**^ **H NMR** (400 MHz, DMSO) δ [ppm] = 11.80 (s, 1H), 7.95 (s, 1H), 5.19–5.18 (m, 2H), 5.04 (brs, 1H), 4.75 (s, 1H), 4.65 (d, *J* = 3.2 Hz, 1H), 3.90 (s, 2H), 3.42–3.35 (m, 1H), 2.17–2.07 (m, 3H), 1.82–1.79 (m, 1H), 1.42–1.37 (m, 1H); **HRMS** (ESI, 3.5 kV) *m*/*z*: [M + Na]^+^ calc for C_13_H_15_BrN_2_O_5_Na^+^ 381.0057, found 381.0057. **LCMS** *m*/*z* 360.8 (M + H) (ESI +ve), RT = 3.30 min; **HPLC** RT = 4.09 min, 100%.

#### Synthesis of 5-Fluorouracil Analogue

##### Synthesis of 3-Benzoyl-5-fluoropyrimidine-2,4­(1*H*,3*H*)-dione (**66**)

To a stirred solution of 5-fluorouracil (5FU) (1.00 equiv, 10.0 mmol, 1.30 g) in dry ACN (4 mL) and pyridine (5 mL) under a nitrogen atmosphere, benzoyl chloride (2.50 equiv, 25.0 mmol, 2.90 mL) was added dropwise at 0 °C. The reaction mixture was then stirred at room temperature overnight for 18 h. The reaction was quenched by the addition of water (30 mL). After phase separation, the aqueous layer was extracted with DCM (3 × 30 mL), and the combined organic phases were dried over MgSO_4_ and filtered. The solvent was removed *in vacuo* to afford the crude product. The crude product was then dissolved in a mixture of dioxane and water (20 mL, 1:1), and a solution of K_2_CO_3_ (0.5 mmol in 10 mL water) was added. The mixture was stirred for 2 h at room temperature. Acetic acid (1.5 mL) was added to adjust the pH to 5, and the solvent was removed *in vacuo*. The white solid was collected and then washed with a saturated NaHCO3 solution (20 mL), purified water (20 mL), and ACN (20 mL), and dried *in vacuo* to yield N3-Bz-5FU **66** (1.78 g, 7.6 mmol, 76%). **R**_**f**_ = 0.51 (hexane/ethyl acetate 1:2); ^**1**^ **H NMR** (700 MHz, CDCl_3_) δ [ppm] = 7.95 (s, 2H), 7.71 (t, *J* = 7.5 Hz, 1H), 7.54 (dd, *J* = 8.0 8.0 Hz, 4H); ^**13**^ **C­{** ^**1**^ **H}-NMR** (176 MHz, CDCl_3_) δ [ppm] = 167.3, 156.5, 150.3, 140.1, 136.1, 131.0, 129.7, 124.8 (d, *J* = 31.8 Hz).

##### Synthesis of 3-Benzoyl-5-fluoro-1-((3aS,4S,6aR)-5-iodo-2,2-dimethyl-3a,6a-dihydro-4*H*-cyclopenta­[*d*]­[1,3]­dioxol-4-yl)­pyrimidine-2,4 (**67**)

To a stirred solution of alcohol **25** (1.00 equiv, 3.55 mmol, 1.00 g) and N3-Bz-5FU **66** (1.30 equiv, 4.61 mmol, 1.08 g) in dry THF (35 mL) under a nitrogen atmosphere, activated 3Å molecular sieves were added, and the mixture was stirred for 2 h to remove residual water. Then, at 0 °C under a nitrogen atmosphere, triphenylphosphine (PPh_3_, 1.20 equiv, 4.25 mmol, 1.12 g) and diisopropyl azodicarboxylate (DIAD, 1.20 equiv, 4.25 mmol, 0.86 g) were added. The reaction mixture was stirred at room temperature overnight. After 18 h, to remove the DIAD-derived side product, Boc_2_O (3.00 equiv, 10.6 mmol, 2.32 g) was added to the mixture at 0 °C, followed by the addition of DMAP (0.10 equiv, 0.35 mmol, 43.3 mg). The reaction was stirred at room temperature overnight. The reaction was quenched with a saturated NH_4_Cl solution (40 mL) and diluted with ethyl acetate (30 mL). After phase separation, the aqueous layer was extracted with ethyl acetate (3 × 30 mL) and washed with brine (30 mL). The combined organic phases were dried over MgSO4, filtered, and the solvent was removed *in vacuo*. The crude product was purified by silica gel column chromatography (hexane/EA 5:1 to 2:1). The solvent was removed *in vacuo* to yield compound **67** (1.02 g, 2.04 mmol, 58%). **R**_**f**_ = 0.21 (hexane/ethyl acetate 3:1).

##### Synthesis of 3-Benzoyl-1-((3aS,4R,6aR)-2,2-dimethyl-5-vinyl-3a,6a-dihydro-4*H*-cyclopenta­[*d*]­[1,3]­dioxol-4-yl)-5-fluoropyrimidine-2,4­(1*H*,3*H*)-dione (**36**)

To a stirred solution of compound **67** (1.00 equiv, 2.04 mmol, 1.02 g) in dry NMP (3.5 mL) under a nitrogen atmosphere, Pd­(PhCN)_2_Cl_2_ (0.03 equiv, 0.06 mmol, 23.5 mg), CuI (0.06 equiv, 0.12 mmol, 23.3 mg), triphenylarsine (Ph_3_As, 0.06 equiv, 0.12 mmol, 37.5 mg), and vinyl tributyltin (1.10 equiv, 2.25 mmol, 0.70 mL) were added at 0 °C. The reaction mixture was stirred at room temperature overnight. The reaction was quenched with a saturated NH_4_Cl solution (50 mL), diluted with ethyl acetate (30 mL). After phase separation, the aqueous layer was extracted with ethyl acetate (3 × 30 mL). The combined organic phases were washed with water (50 mL) and brine (50 mL), dried over MgSO_4_, and filtered. The solvent was removed in vacuo, and the crude product was purified by silica gel column chromatography (hexane/ethyl acetate, 2:1). The solvent was removed *in vacuo* to yield compound **36** (0.74 g, 1.86 mmol, 91%). **R**_**f**_ = 0.33 (hexane/ethyl acetate 2:1); ^**1**^ **H NMR** (700 MHz, CDCl_3_) δ [ppm] = 7.92–7.88 (d, *J* = 7.8 Hz, 2H), 7.69 (dt, *J* = 7.4, 1.3 Hz, 1H), 7.54–7.50 (m, 2H), 7.02–6.98 (brs, 1H), 6.48 (dd, *J* = 17.7, 11.0 Hz, 1H), 6.22 (s, 1H), 5.74–5.70 (brs, 1H), 5.39 (d, *J* = 11.0 Hz, 1H), 5.35 (d, *J* = 5.7 Hz, 1H), 5.25 (d, *J* = 17.7 Hz, 1H), 4.61 (s, 1H), 1.41 (s, 3H), 1.34 (s, 3H); ^**13**^ **C­{** ^**1**^ **H}-NMR** (176 MHz, CDCl_3_) δ [ppm] = 167.0, 155.9 (d, *J* = 26.4 Hz), 148.4, 140.4 (d, *J* = 240.2 Hz), 139.6, 137.8, 135.7, 131.0, 130.7, 129.8, 129.5, 124.2, 120.8, 112.5, 84.2, 83.1, 65.9, 27.3, 25.8.

##### Synthesis of 1-((3aS,4R,6aR)-2,2-Dimethyl-5-vinyl-3a,6a-dihydro-4*H*-cyclopenta­[*d*]­[1,3]­dioxol-4-yl)-5-fluoropyrimidine-2,4­(1*H*,3*H*)-dione (**68**)

To a stirred solution of **36** (1.00 equiv, 1.69 mmol, 0.67 g) in MeOH (5 mL) at 0 °C, NH_3_ in MeOH (7 M, 5 mL) was added. The reaction mixture was stirred at 0 °C for 2 h. The solvent was removed *in vacuo*, and the crude product was purified by silica gel column chromatography (hexane/ethyl acetate, 2:1). The solvent was removed in vacuo to yield compound **68** (0.48 g, 1.61 mmol, 96%). **R**_**f**_ = 0.24 (hexane/ethyl acetate 3:1).

##### Synthesis of 5-Fluoro-1-((3aR,3bR,4R,8R,8aS)-4-hydroxy-2,2-dimethyl-3a,3b,5,6,8,8a-hexahydro-4*H*-indeno­[1,2-*d*]­[1,3]­dioxol-8-yl)­pyrimidine-2,4­(1*H*,3*H*)-dione (**69**)

To a stirred solution of diene **68** (1.00 equiv, 1.68 mmol, 0.49 g) in toluene (15 mL), BHT (50 mg) and vinylboronic acid pinacol ester (5.00 equiv, 8.40 mmol, 1.40 mL) were added. The solution was stirred at 140 °C for 4 days in a sealed tube. The solvent was removed *in vacuo*, and the residue was dissolved in THF (10 mL) and pH 7 buffer (10 mL). NaBO_3_ 4H_2_O (4.0 equiv, 6.72 mmol, 1.03g) was added, and the mixture was stirred vigorously at room temperature for 6 h. The reaction was quenched by adding Na_2_S_2_O_3_ (1 g) and stirring for 10 min. Water (40 mL) was added, and after phase separation, the aqueous layer of the mixture was extracted with ethyl acetate (3 × 30 mL). The combined organic phases were dried over MgSO_4_, and the solvent was removed *in vacuo*. The crude product was purified by silica gel column chromatography (hexane/EA 1:2 to 1:8). The solvent was removed in vacuo to yield pure compound **69** (118.4 mg) and a mixture of other isomers (154.8 mg), corresponding to a total of 0.81 mmol (48% combined yield). **R**_**f**_ = 0.2 (hexane/ethyl acetate 1:1).

##### Synthesis of 5-Fluoro-1-((1R,2S,3R,3aR,4R)-2,3,4-trihydroxy-2,3,3a,4,5,6-hexahydro-1*H*-inden-1-yl)­pyrimidine-2,4­(1*H*,3*H*)-dione (**14a**)

To a stirred solution of **69** (1.00 equiv, 0.16 mmol, 53 mg) in MeOH (1.5 mL), TFA (20% in H_2_O, 1 mL) was added at room temperature. The reaction mixture was stirred at room temperature overnight. The solvent was removed *in vacuo*, and ACN (2 × 5 mL) was added for coevaporation to remove residual water. The crude product was purified by HPLC using a mobile phase of 20% H_2_O and 80% ACN. The solvent was removed *in vacuo* with ACN as the cosolvent for coevaporation to facilitate the removal of water, yielding compound **14a** (16.7 mg, 0.056 mmol, 38%). **R**_**f**_ = 0.22 (ethyl acetate/MeOH 9:1); ^**1**^ **H NMR** (700 MHz, methanol-d4) δ­[ppm] = 7.69 (s, 1H), 5.39 (brs, 1H), 5.25 (s, 1H), 4.07 (s, 1H), 3.94 (s, 1H), 3.57 (ddd, *J* = 11.4, 9.4, 3.7 Hz, 1H), 2.41–2.37 (m, 1H), 2.25 (brs, 2H), 2.00–1.93 (m, 1H), 1.62–1.52 (m, 1H), ^**13**^ **C­{** ^**1**^ **H}-NMR** (176 MHz, methanol-d4) δ­[ppm] = 159.4 (d, *J* = 27.1 Hz), 152.1, 141.7 (d, *J* = 233.4 Hz), 135.1, 128.1, 121.2, 75.3, 73.0, 71.5, 63.8, 53.7, 32.4, 26.0; **HRMS** (ESI, 3.5 kV) *m*/*z*: [M + Na]^+^ calc for C_13_H_15_FN_2_O_5_Na^+^ 321.0857, found 321.0852; **HPLC** RT = 13.67 min, 95.4%.

#### Synthesis of (5′S) and 5′-Deoxy Purine Analogues

##### Synthesis of (3aR,3bR,4R,8R,8aS)-8-(6-Amino-9*H*-purin-9-yl)-2,2-dimethyl-3a,3b,5,6,8,8a-hexahydro-4*H*-indeno­[1,2-*d*]­[1,3]­dioxol-4-ol (**37**)

To intermediate **32a** (2.0 g, 6.12 mmol, 1.0 equiv) in THF (20 mL) was added NH_4_OH (20 mL) at room temperature. The resulting reaction mixture was heated to 120 °C for 16 h. After completion of the reaction, the reaction mixture was concentrated under reduced pressure to afford the title compound **37** as a dark brown oil (1.9 g, 5.54 mmol, 90%). **R**_**f**_ = 0.4 (10% MeOH/DCM); ^**1**^ **H NMR** (400 MHz, DMSO) δ [ppm] = 8.23 (s, 1H), 8.11 (s, 1H), 7.29 (brs, 2H), 5.30 (brs, 1H), 5.10 (t, *J* = 6.4 Hz, 1H), 5.02­(d, *J* = 5.6 Hz, 1H), 4.65–4.61 (m, 2H), 3.47–3.44 (m, 1H), 2.51–2.50 (m, 1H), 2.01 (brs, 2H), 1.83–1.80 (m, 1H), 1.49 (s, 3H), 1.46–1.43 (m, 1H), 1.27 (s, 3H); ^**13**^ **C­{** ^**1**^ **H}-NMR** (176 MHz, DMSO) δ­[ppm] = 156.5, 153.0, 150.0, 140.5, 138.6, 119.9, 119.2, 113.0, 82.7, 82.0, 69.9, 62.8, 51.9, 31.7, 27.9, 25.6, 24.9; **LCMS** *m*/*z* 344.3 (M + H) (ESI +ve), RT = 1.42 min. The material obtained was directly used in the next step without purification.

##### Synthesis of (3aR,3bR,4S,8R,8aS)-8-(6-Amino-9*H*-purin-9-yl)-2,2-dimethyl-3a,3b,5,6,8,8a-hexahydro-4*H*-indeno­[1,2-*d*]­[1,3]­dioxol-4-yl 4-Nitrobenzoate (**70**)

To a stirred solution of **37** (0.80 g, 2.32 mmol, 1.0 equiv), 4-nitrobenzoic acid (1.94 g, 11.64 mmol, 5.0 equiv), and PPh_3_ (3.05 g, 11.64 mmol, 5.0 equiv) in anhydrous THF (12.0 mL, 15 vol) was added dropwise DIAD (2.35 g, 11.64 mmol, 5.0 equiv) at 0 °C. The resulting reaction mixture was stirred at room temperature for 2 h. After completion of the reaction, the reaction mixture was concentrated under reduced pressure to get the crude, which was purified by reversed-phase chromatography using (60% MeCN/water), to afford the title compound **70** as a light-yellow sticky oil (0.35 g, 0.71 mmol, 30% yield). **R**_**f**_ = 0.6 (5% MeOH/DCM); **LCMS** *m*/*z* 493.1 (M + H) (ESI +ve); RT = 2.01 min.

##### Synthesis of (1R,2S,3R,7S,7aR)-3-(6-Amino-9*H*-purin-9-yl)-2,3,5,6,7,7a-hexahydro-1*H*-indene-1,2,7-triol (**5d**)

To a stirred solution of **70** (0.35 g, 0.71 mmol, 1.0 equiv) in a mixture of THF (3.5 mL) and water (3.5 mL) was added TFA (0.35 mL, 1.0 vol) at room temperature. The resulting reaction mixture was stirred at room temperature for 7 h. After completion of the reaction, the reaction mixture was concentrated under reduced pressure to afford the crude as a light-yellow oil (0.42 g). To a stirred solution of the crude in methanol (4.3 mL) was added NaOMe (0.15 g, 2.85 mmol) and NH_4_OH (4.3 mL) at room temperature. The resulting reaction mixture was stirred at room temperature for 16 h. After completion of the reaction, the reaction mixture was concentrated under reduced pressure to get the crude, which was purified by reversed-phase chromatography using (60% MeCN/water). The enriched crude was further purified by RP Prep HPLC. The obtained pure fractions were lyophilized to afford the title compound **5d** as a white solid (65 mg, 0.21 mmol, 30% over 2 steps). **R**_**f**_ = 0.15 (10% MeOH/DCM); ^**1**^ **H NMR** (400 MHz, MeOD) δ [ppm] = 8.25 (s, 1H), 8.21 (s, 1H), 5.36–5.32 (m, 2H), 4.39–4.36 (m, 1H), 4.31 (dd, *J* = 6.0, 6.0 Hz, 1H), 4.09 (dd, *J* = 5.2, 5.2 Hz, 1H), 2.72 (brs, 1H), 2.25–2.20 (m, 1H), 2.16–2.14 (m, 1H), 2.01–1.95 (m, 1H), 1.81–1.76 (m, 1H); ^**13**^ **C­{** ^**1**^ **H}-NMR** (100 MHz, DMSO) δ [ppm] = 156.4, 152.7, 150.2, 139.9, 135.2, 121.6, 119.0, 75.9, 70.1, 62.9, 61.4, 49.1, 28.9, 20.8; **HRMS** (ESI, 3.5 kV) *m*/*z*: [M + H]^+^ calc for C_14_H_17_N_5_O_3_H^+^ 304.1404, found 304.1408; **LCMS** *m*/*z* 304.0 (M + H) (ESI +ve), RT = 2.01 min; **HPLC** RT = 3.98 min, 100%.

##### Synthesis of *O*-((3aR,3bR,4R,8R,8aS)-8-(6-Amino-9*H*-purin-9-yl)-2,2-dimethyl-3a,3b,5,6,8,8a-hexahydro-4*H*-indeno­[1,2-*d*]­[1,3]­dioxol-4-yl) *O*-Phenyl Carbonothioate (**71**)

To a stirred solution **37** (0.60 g, 1.74 mmol, 1.0 equiv) in acetonitrile (3.0 mL) was added DMAP (1.06 g, 8.73 mmol, 5.0 equiv), followed by the addition of phenyl thioxochloroformate (0.66 g, 3.84 mmol, 2.2 equiv) at room temperature. The resulting reaction mixture was stirred at room temperature for 2 h. After completion of the reaction, the reaction mixture was diluted with water (60 mL) and extracted with ethyl acetate (3 × 25 mL). The combined organic phase was dried over anhydrous Na_2_SO_4_, filtered, and concentrated under reduced pressure to get the crude, which was purified by column chromatography using silica gel (35% EA/hex). The obtained pure fractions were concentrated under reduced pressure to afford the title compound **71** as a light-yellow sticky oil (0.32 g, 0.66 mmol, 38%). **R**_**f**_ = 0.5 (50% EA/hex); **LCMS** *m*/*z* 480.1 (M + H) (ESI +ve), RT = 2.14 min.

##### Synthesis of 9-((3aR,3bS,8R,8aS)-2,2-Dimethyl-3a,3b,5,6,8,8a-hexahydro-4*H*-indeno­[1,2-*d*] [1,3]­dioxol-8-yl)-9*H*-purin-6-amine (**72**)

To a stirred solution of **71** (0.30 g, 0.62 mmol, 1.0 equiv) in toluene (6.0 mL) was purged with N_2_ gas for 15 min. To this reaction mixture, tributyltin hydride (0.36 g, 1.25 mmol, 2.0 equiv) and AIBN (0.03 g, 0.21 mmol, 0.35 equiv) were added at room temperature under N_2_ atmosphere. The resulting reaction mixture was heated to 110 °C for 16 h. After completion of the reaction, the reaction mixture was concentrated under reduced pressure to get the crude, which was purified by column chromatography using silica gel (35% EA/hex). The obtained pure fractions were concentrated under reduced pressure to afford the title compound **72** as a yellow sticky liquid (0.16 g, 0.48 mmol, 78%). **R**_**f**_ = 0.4 (50% EA/hex); ^**1**^ **H NMR** (400 MHz, DMSO) δ [ppm] = 8.34 (s, 2H), 8.22 (s, 1H), 7.83 (brs, 2H), 5.37 (s, 1H), 5.05 (dd, *J* = 5.2, 6.8 Hz, 1H), 4.81 (s, 1H), 4.44 (dd, *J* = 5.2, 7.2 Hz, 1H), 2.20–2.10 (m, 2H), 1.91–1.89 (m, 2H), 1.80–1.71 (m, 1H), 1.59–1.4 (m, 1H), 1.49 (s, 3H), 1.27 (s, 3H); **LCMS** *m*/*z* 328.2 (M + H) (ESI +ve), RT = 1.74 min.

##### Synthesis of (1R,2S,3R,7aS)-3-(6-Amino-9*H*-purin-9-yl)-2,3,5,6,7,7a-hexahydro-1*H*-indene-1,2-diol (**5e**)

To a stirred solution of **72** (0.16 g, 0.48 mmol, 1.0 equiv) in a mixture of THF (1.6 mL) and water (1.6 mL) was added TFA (0.16 mL) at room temperature. The resulting reaction mixture was stirred at room temperature for 16 h. After completion of the reaction, the reaction mixture was concentrated under reduced pressure to get the crude, which was purified by RP Prep. HPLC. The obtained pure fractions were lyophilized to afford title compound **5e** as a white solid (0.04 g, 0.13 mmol, 28%). **R**_**f**_ = 0.1 (10% MeOH/DCM); ^**1**^ **H NMR** (400 MHz, DMSO) δ [ppm] = 8.11 (s, 2H), 7.23 (brs, 2H), 5.16 (s, 1H), 5.12 (d, *J* = 5.2 Hz, 1H), 4.91–4.90 (m, 2H), 4.14 (dd, *J* = 5.2; 10.4 Hz, 1H), 3.83 (dd, *J* = 6.0, 13.2 Hz, 1H), 2.45 (brs, 1H), 2.11–2.07 (m, 1H), 1.97–1.88 (m, 2H), 1.80–1.76 (m, 1H), 1.44–1.39 (m, 1H), 1.23–1.10 (m, 1H); ^**13**^ **C­{** ^**1**^ **H}-NMR** (400 MHz, DMSO) δ [ppm] = 156.4, 152.8, 150.1, 140.5, 139.1, 120.5, 119.1, 75.6, 62.7, 44.0, 27.0, 24.6, 21.9; **HRMS** (ESI, 3.5 kV) *m*/*z*: [M + H]^+^ calc for C_14_H_17_N_5_O_2_H^+^ 288.1455, found 288.1451; **LCMS** *m*/*z* 288.1 (M + H) (ESI +ve) RT = 1.43 min; **HPLC** RT = 4.41 min, 100%.

##### Synthesis of (3aR,3bR,4S,8R,8aS)-8-(6-(Dimethylamino)-9*H*-purin-9-yl)-2,2-dimethyl-3a,3b,5,6,8,8a-hexahydro-4*H*-indeno­[1,2-*d*]­[1,3]­dioxol-4-yl Benzoate (**73**)

To a stirred solution of **38** (0.45 g, 1.21 mmol, 1.0 equiv), benzoic acid (0.22 g, 1.82 mmol, 1.5 equiv), and PPh_3_ (0.64 g, 2.42 mmol, 2.0 equiv) in THF (6.8 mL) was dropwise added DIAD (0.49 g, 2.42 mmol, 2.0 equiv) at 0 °C under N_2_ atmosphere. The resulting reaction mixture was stirred at room temperature for 3 h. After completion of the reaction, the reaction mixture was diluted with water (30 mL) and extracted with ethyl acetate (2 × 25 mL). The combined organic layer was dried over anhydrous Na_2_SO_4_, filtered, and concentrated under reduced pressure to get the crude, which was purified by reversed-phase chromatography using (55% MeCN/water). The pure fractions obtained were lyophilized to afford the title compound **73** as an off-white semisolid (0.2 g, 0.42 mmol, 35% yield). **R**_**f**_ = 0.5 (40% EA/Hex); ^**1**^ **H NMR** (400 MHz, DMSO) δ [ppm] = 8.29 (s, 1H), 8.21 (s, 1H), 8.08 (d, *J* = 6.8 Hz, 2H), 7.72–7.68 (m, 1H), 7.60–7.56 (m, 2H), 5.59 (dd, *J* = 3.6, 3.6 Hz, 1H), 5.47 (brs, 1H), 5.22 (dd, *J* = 5.6, 6.8 Hz, 1H), 4.88 (brs, 1H), 4.76 (d, *J* = 5.4, 6.8 Hz, 1H), 3.50 (brs, 6H), 3.05 (s, 1H), 2.09–2.06 (m, 1H), 1.99–1.89 (m, 2H), 1.84–1.79 (m, 1H), 1.53 (s, 3H), 1.24 (m, 3H); ^**13**^ **C­{** ^**1**^ **H}-NMR** (100 MHz, DMSO) δ [ppm] = 165.8, 154.8, 152.1, 150.6, 140.0, 136.8, 133.9, 130.3, 129.9, 129.3, 120.5, 120.2, 112.9, 82.7, 78.6, 67.8, 62.6, 46.9, 38.4, 28.1, 25.8, 25.4, 20.1; **HRMS** (ESI, 3.5 kV) *m*/*z*: [M + H]^+^ calc for C_26_H_29_N_5_O_4_H^+^ 476.2292, found 476.2291; **LCMS** *m*/*z* 476.4 (M + H) (ESI +ve), RT = 2.34 min; **HPLC** RT = 8.28 min, 99.56%.

##### (1R,2S,3R,7S,7aR)-3-(6-(Dimethylamino)-9*H*-purin-9-yl)-2,3,5,6,7,7a-hexahydro-1*H*-indene-1,2,7-triol (**6d**)

To a stirred solution of **73** (0.20 g, 0.42 mmol, 1.0 equiv) in a mixture of THF (2.0 mL) and water (2.0 mL) was added TFA (0.8 mL) at room temperature. The resulting reaction mixture was then stirred at room temperature for 16 h. After completion of the reaction, the reaction mixture was concentrated under reduced pressure to afford the crude as a light-brown semisolid (0.20 g, quantitative yield). To a stirred solution of the crude (0.2 g, 0.39 mmol, 1.0 equiv) in methanol (2.0 mL) was added sodium methoxide (0.17 g, 3.15 mmol, 8.0 equiv) and NH_4_OH (4.0 mL) at room temperature. The resulting reaction mixture was stirred at room temperature for 48 h. After completion of the reaction, the reaction mixture was concentrated under reduced pressure to get the crude, which was purified by RP Prep HPLC. The obtained pure fractions were lyophilized to afford the title compound **6d** as a white solid (0.050 g, 0.15 mmol, 36% over 2 steps). **R**_**f**_ = 0.2 (10% MeOH/DCM); ^**1**^ **H NMR** (400 MHz, DMSO) δ [ppm] = 8.20 (s, 1H), 8.07­(s, 1H), 5.19 (brs, 1H), 5.12 (d, *J* = 5.2 Hz, 1H), 5.06 (s, 1H), 4.80 (d, *J* = 5.6 Hz, 1H), 4.59 (d, *J* = 5.2 Hz, 1H), 4.16–4.15 (m, 1H), 4.09 (dd, *J* = 2.0, 3.6 Hz, 1H), 3.90 (dd, *J* = 5.6, 10.8 Hz, 1H), 3.45 (brs, 6H), 2.51–2.49 (m, 1H), 2.11–2.03 (m, 1H), 1.97–1.91 (m, 1H), 1.82–1.76 (m, 1H), 1.62–1.50 (m, 1H); ^**13**^ **C­{** ^**1**^ **H}-NMR** (100 MHz, DMSO) δ [ppm] = 154.6, 152.1, 151.0, 138.7, 135.2, 121.6, 119.5, 75.8, 70.1, 62.8, 61.3, 49.2, 38.4, 28.9, 20.8; **HRMS** (ESI, 3.5 kV) *m*/*z*: [M + H]^+^ calc for C_16_H_21_N_5_O_3_H^+^ 332.1717, found 332.1716; **LCMS** *m*/*z* 332.1 (M + H) (ESI +ve), RT = 1.41 min; **HPLC** RT = 6.49 min, 99.33%.

##### Synthesis of *O*-((3aR,3bR,4R,8R,8aS)-8-(6-(Dimethylamino)-9*H*-purin-9-yl)-2,2-dimethyl-3a,3b,5,6,8,8a-hexahydro-4*H*-indeno­[1,2-*d*]­[1,3]­dioxol-4-yl) *O*-Phenyl Carbonothioate (**74**)

To a stirred solution of **38** (0.45 g, 1.21 mmol, 1.0 equiv) and DMAP (0.74 g, 6.02 mmol, 5.0 equiv) in acetonitrile (2.25 mL) was added phenyl thioxochloroformate (0.42 g, 2.42 mmol, 2.0 equiv) at room temperature. The resulting reaction mixture was stirred at room temperature for 1 h. After completion of the reaction, the reaction mixture was diluted with water (50 mL) and extracted with ethyl acetate (2 × 40 mL). The combined organic phase was dried over anhydrous Na_2_SO_4_, filtered, and concentrated under reduced pressure to get the crude, which was purified by column chromatography using silica gel (30% EA/Hex). The obtained pure fractions were concentrated under reduced pressure to afford the title compound **74** as a light-brown solid (0.40 g, 0.78 mmol, 65% yield). **R**_**f**_ = 0.5 (50% EA/Hex); **LCMS** *m*/*z* = 508.2 (M + H) (ESI +ve), RT = 2.48 min.

##### Synthesis of 9-((3aR,3bS,8R,8aS)-2,2-Dimethyl-3a,3b,5,6,8,8a-hexahydro-4*H*-indeno­[1,2-*d*]­[1,3]­dioxol-8-yl)-*N*,*N*-dimethyl-9*H*-purin-6-amine (**75**)

A stirred solution of **74** (0.40 g, 0.79 mmol, 1.0 equiv) in toluene (8.0 mL) was purged with N_2_ for 15 min. To this reaction mixture tributyltin hydride (0.92 g, 3.15 mmol, 4.0 equiv) and AIBN (0.09 g, 0.55 mmol, 0.7 equiv) was added at room temperature. The resulting reaction mixture was heated to 110 °C for 5 h. After completion of the reaction, the reaction mixture was concentrated under reduced pressure to get the crude, which was purified by reversed-phase chromatography using (60% MeCN/water). The obtained pure fraction was lyophilized to afford the title compound **75** as a light-brown semisolid (0.14 g, 0.39 mmol, 50% yield). **R**_**f**_ = 0.45 (50% EA/Hex); ^**1**^ **H NMR** (400 MHz, DMSO) δ [ppm] = 8.25 (s, 1H), 8.20 (m, 1H), 5.77 (s, 1H), 5.39 (brs, 1H), 5.05 (dd, *J* = 5.2, 6.8 Hz, 1H), 4.74 (s, 1H), 4.44 (dd, *J* = 5.2, 7.2 Hz, 1H), 3.48 (brs, 6H), 2.55–2.51 (m, 1H), 2.16–2.12 (m, 1H), 1.91–1.83 (m, 2H), 1.78–1.74 (m, 1H), 1.40 (s, 3H), 1.26 (s, 3H), 1.33–1.22 (m, 2H); **HRMS** (ESI, 3.5 kV) *m*/*z*: [M + H]^+^ calc for C_19_H_25_N_5_O_2_H^+^ 356.2081, found 356.2075; **LCMS** *m*/*z* = 356.3 (M + H) (ESI +ve), RT = 2.00 min.

##### Synthesis of (1R,2S,3R,7aS)-3-(6-(Dimethylamino)-9*H*-purin-9-yl)-2,3,5,6,7,7a-hexahydro-1*H*-indene-1,2-diol (**6e**)

To a stirred solution of **75** (0.14 g, 0.39 mmol, 1.0 equiv) in a mixture of THF (1.4 mL) and water (1.4 mL) was added TFA (0.5 6 mL) at room temperature. The resulting reaction mixture was stirred at room temperature for 16 h. After completion of the reaction, the reaction mixture was concentrated under reduced pressure to get the crude, which was purified by RP prep. HPLC. The obtained pure fraction was lyophilized to afford title compound **6e** as a white solid (0.05 g, 0.16 mmol, 40%). **R**_**f**_ = 0.2 (10% MeOH/DCM); ^**1**^ **H NMR** (400 MHz, DMSO) δ [ppm] = 8.20 (s, 1H), 8.12 (s, 1H), 5.20 (s, 1H), 5.11 (d, *J* = 5.2 Hz, 1H), 4.91 (d, *J* = 6.0 Hz, 1H), 4.87 (s, 1H), 4.12 (dd, *J* = 5.2, 10.4 Hz, 1H), 3.83 (dd, *J* = 6.0, 13.6 Hz, 1H), 3.45 (brs, 6H), 2.41 (brs, 1H), 2.11–2.07 (m, 1H), 1.96–1.76 (m, 3H), 1.44–1.38 (m, 1H), 1.21–1.14 (m, 1H); ^**13**^ **C­{** ^**1**^ **H}-NMR** (100 MHz, DMSO) δ [ppm] = 154.7, 152.1, 150.9, 139.3, 139.1, 120.6, 119.6, 75.6, 70.1, 62.5, 44.0, 38.4, 27.0, 24.6, 21.9; **HRMS** (ESI, 3.5 kV) *m*/*z*: [M + H]^+^ calc. for C_16_H_21_N_5_O_2_H^+^ 316.1768, found 316.1766; **LCMS** *m*/*z* = 316.20 (M + H) (ESI +ve), RT = 1.55 min; **HPLC** RT = 4.33 min, 100%.

##### Synthesis of (3aR,3bS,8R,8aS)-8-(6-((4-Methoxybenzyl)­oxy)-9*H*-purin-9-yl)-2,2-dimethyl-3a,3b,5,6,8,8a-hexahydro-4*H*-indeno­[1,2-*d*]­[1,3]­dioxol-5-ol (**76**)

To a stirred solution of (4-methoxyphenyl)­methanol (1.14 g, 8.28 mmol, 1.5 equiv) in 1,4-dioxane (10 mL) was added Cs_2_CO_3_ (4.48 g, 13.81 mmol, 2.5 equiv) at room temperature. The resulting reaction mixture was stirred for 20 min at room temperature. To this reaction mixture **32b/c** (2.0 g, 5.52 mmol, 1.0 equiv) in 1,4-dioxane (10 mL) was added dropwise at room temperature. The resulting reaction mixture was heated to 80 °C for 3 h. After completion of the reaction, the reaction mixture was diluted with water (200 mL) and extracted with ethyl acetate (3 × 150 mL). The combined organic phase was dried over anhydrous Na_2_SO_4_, filtered, and concentrated under reduced pressure to get the crude, which was purified by reversed-phase chromatography using (40% MeCN/water). The pure fractions obtained were lyophilized to afford the title compound **76** as an off-white solid (1.0 g, 2.15 mmol, 39%). **R**_**f**_ = 0.4 (100% EA). ^**1**^ **H NMR** (400 MHz, DMSO) δ [ppm] = 8.53 (s, 1H), 8.49 (s, 1H), 7.46 (d, *J* = 8.8 Hz, 2H), 6.96 (d, *J* = 8.4 Hz, 2H), 5.54 (s, 2H), 5.43 (brs, 1H), 5.09–5.06 (m, 1H), 4.82 (d, *J* = 4.8 Hz, 1H), 4.69–4.68 (m, 1H), 4.49–4.46 (m, 1H), 3.74 (s, 3H), 2.69 (brs, 1H), 2.22–2.12 (m, 2H), 1.67–1.66 (m, 1H), 1.48 (s, 3H), 1.38–1.36 (m, 1H), 1.31–1.27 (m, 1H), 1.25 (s, 3H). **LCMS** *m*/*z* = 465.2 (M + H) (ESI +ve), RT = 2.01 min.

##### Synthesis of *O*-((3aR,3bS,8R,8aS)-8-(6-((4-Methoxybenzyl)­oxy)-9*H*-purin-9-yl)-2,2-dimethyl-3a,3b,5,6,8,8a-hexahydro-4*H*-indeno­[1,2-*d*]­[1,3]­dioxol-5-yl) *O*-Phenyl Carbonothioate (**77**)

To a stirred solution of **76** (1.0 g, 2.15 mmol, 1.0 equiv) and DMAP (1.05 g, 8.62 mmol, 5.0 equiv) in acetonitrile (5.0 mL) was added O-phenyl carbonochloridothioate (1.11 g, 6.46 mmol, 3.0 equiv) at room temperature. The resulting reaction mixture was stirred at room temperature for 2 h. After completion of the reaction, the reaction mixture was diluted with water (150 mL) and extracted with ethyl acetate (2 × 100 mL). The combined organic phase was dried over anhydrous Na_2_SO_4_, filtered, and concentrated under reduced pressure to get the crude, which was purified by reversed-phase chromatography using (80% MeCN/water). The obtained pure fractions were lyophilized to afford **77** as a light-brown solid. (0.7 g, 1.17 mmol, 54%). **R**_**f**_ = 0.4 (30% EA/Hex); **LCMS** *m*/*z* = 601.3 (M + H) (ESI +ve), RT = 2.80 min; **HPLC** RT = 10.2 min, 57.04%.

##### Synthesis of 9-((1R,2S,3R,3aS)-2,3-Dihydroxy-2,3,3a,4,5,6-hexahydro-1*H*-inden-1-yl)-1,9-dihydro-6*H*-purin-6-one (**8e**)

A stirred solution of **77** (0.5 g, 0.83 mmol, 1.0 equiv) in toluene (5.0 mL) was purged with N_2_ gas for 10 min. To this reaction mixture, tributyltin hydride (0.97 g, 3.33 mmol, 4.0 equiv) and AIBN (0.14 g, 0.83 mmol, 1.0 equiv) were added at room temperature. The resulting reaction mixture was heated to 110 °C for 2 h. After completion of the reaction, the reaction mixture was concentrated under reduced pressure to afford the crude as a yellowish oil (1.0 g). To a stirred solution of the crude in a mixture of THF (10 mL) and water (10 mL) was added TFA (5.0 mL) at room temperature. The resulting reaction mixture was stirred at room temperature for 5 h. After completion of the reaction, the reaction mixture was concentrated under reduced pressure to get the crude, which was purified by RP Prep. HPLC. The obtained pure fraction was lyophilized to afford the title compound **8e** as a white solid (0.06 g, 0.21 mmol, 25% over two steps). **R**_**f**_ = 0.2 (10% MeOH/DCM); ^**1**^ **H NMR** (400 MHz, DMSO) δ [ppm] = 12.30 (s, 1H), 8.07 (s, 1H), 8.03 (s, 1H), 5.17–5.16 (m, 2H), 4.92 (brs, 2H), 4.11 (dd, *J* = 4.8, 9.6 Hz, 1H), 3.79 (dd, *J* = 6.0, 12.0 Hz, 1H), 2.39 (brs, 1H), 2.10–2.07 (m, 1H), 1.98–1.83 (m, 2H), 1.80–1.76 (m, 1H), 1.44–1.39 (m, 1H), 1.20–1.17 (m, 1H); ^**13**^ **C­{** ^**1**^ **H}-NMR** (100 MHz, DMSO) δ [ppm] = 157.1, 149.0, 145.9, 139.9, 138.9, 124.3, 120.7, 75.7, 75.5, 63.0, 44.1, 27.0, 24.6, 21.8; **HRMS** (ESI, 3.5 kV) *m*/*z*: [M + H]^+^ calc. for C_14_H_16_N_4_O_3_ 289.1295, found 289.1299; **LCMS** *m*/*z* = 289.0 (M + H) (ESI +ve), RT = 1.44 min; **HPLC** RT = 4.33 min, 99.32%.

##### Synthesis of (3aR,3bR,4R,8R,8aS)-8-(6-Methoxy-9*H*-purin-9-yl)-2,2-dimethyl-3a,3b,5,6,8,8a-hexahydro-4*H*-indeno­[1,2-*d*]­[1,3]­dioxol-4-ol (**39**)

To a stirred solution of **32a** (0.60 g, 1.65 mmol, 1.0 equiv) in methanol (6.0 mL) was added K_2_CO_3_ (0.22 g, 1.65 mmol, 1.0 equiv) at room temperature. The resulting reaction mixture was stirred at room temperature for 1 h. After completion of the reaction, the reaction mixture was concentrated under reduced pressure, diluted with water (100 mL), and extracted with ethyl acetate (3 × 50 mL). The combined organic phase was dried over anhydrous Na_2_SO_4_, filtered, and concentrated under reduced pressure to afford the title compound **39** as a brown oil (0.67 g). **R**_**f**_ = 0.5 (10% MeOH/DCM); **LCMS** *m*/*z* 359.2 (M + H) (ESI +ve), RT = 1.65 min. Note: The obtained material was directly used in the next step without purification.

##### Synthesis of (1R,2S,3R,7S,7aR)-3-(6-Methoxy-9*H*-purin-9-yl)-2,3,5,6,7,7a-hexahydro-1*H*-indene-1,2,7-triol (**23d**)

To a stirred solution of **39** (0.67 g, 1.87 mmol, 1.0 equiv) and 4-nitrobenzoic acid (1.25 g, 7.48 mmol, 4.0 equiv) in THF (10 mL) were added triphenylphosphine (1.96 g, 7.48 mmol, 4.0 equiv), followed by dropwise addition of DIAD (1.51 g, 7.48 mmol, 4.0 equiv) at 0 °C under N_2_ atmosphere. The resulting reaction mixture was stirred at room temperature for 2 h. After completion of the reaction, the reaction mixture was diluted with water (200 mL) and extracted with ethyl acetate (3 × 50 mL). The combined organic phase was dried over anhydrous Na_2_SO_4_, filtered, and concentrated under reduced pressure to afford an orange sticky solid (5.50 g). The crude was dissolved in THF (9.5 mL), and water (9.5 mL) as well as TFA (4.5 mL) at room temperature was added. The resulting reaction mixture was stirred at room temperature for 48 h. After completion of the reaction, the reaction mixture was concentrated under reduced pressure to obtain the title compound as an orange solid (7.0 g). The crude was dissolved in methanol (6.4 mL), and sodium methoxide (0.44 g, 8.21 mmol) and NH_4_OH (12.8 mL) at room temperature were added. The resulting reaction mixture was stirred at room temperature for 48 h. After completion of the reaction, the reaction mixture was concentrated under reduced pressure to get the crude, which was purified by reversed-phase chromatography using (20% MeCN/water). The enriched crude was further purified by RP Prep HPLC. The obtained pure fractions were lyophilized to afford **23d** as a white solid (0.06 g, 0.19 mmol, 11% 3 steps). **R**_**f**_ = 0.3 (10% MeOH/DCM); ^**1**^ **H NMR** (400 MHz, DMSO) δ [ppm] = 8.53 (s, 1H), 8.33 (s, 1H), 5.26 (brs, 1H), 5.20 (d, *J* = 5.6 Hz, 1H), 5.07 (brs, 1H), 4.84 (d, *J* = 5.6 Hz, 1H), 4.58 (d, *J* = 5.2 Hz, 1H), 4.16 (s, 1H), 4.17–4.07 (m, 4H), 3.98 (dd, *J* = 5.6, 10.8 Hz, 1H), 2.07–2.03 (m, 1H), 1.96–1.92 (m, 1H), 1.82–1.76 (m, 1H), 1.63–1.61 (m, 1H); ^**13**^ **C­{** ^**1**^ **H}-NMR** (100 MHz, DMSO) δ [ppm] = 160.7, 152.8, 151.8, 142.9, 134.9, 121.7, 120.9, 75.7, 70.0, 62.9, 61.8, 54.3, 49.2, 28.8, 20.8; **HRMS** (ESI, 3.5 kV) *m*/*z*: [M + H]^+^ calc. for C_15_H_18_N_4_O_4_H^+^ 319.1401, found 319.1407; **LCMS** *m*/*z* 319.1 (M + H) (ESI +ve), RT = 1.44 min; **HPLC** RT = 4.40 min, 100%.

##### Synthesis of O-((3aR,3bR,4R,8R,8aS)-8-(6-Methoxy-9*H*-purin-9-yl)-2,2-dimethyl-3a,3b,5,6,8,8a-hexahydro-4*H*-indeno­[1,2-*d*]­[1,3]­dioxol-4-yl) *O*-Phenyl Carbonothioate (**78**)

To a stirred solution of **39** (0.30 g, 0.83 mmol, 1.0 equiv) and DMAP (0.51 g, 4.18 mmol, 5.0 equiv) in acetonitrile (1.5 mL) was added O-phenyl carbonochloridothioate (0.28 g, 1.67 mmol, 2.0 equiv) at room temperature. The resulting reaction mixture was stirred at room temperature for 2 h. After completion of the reaction, the reaction mixture was diluted with water (100 mL) and extracted with ethyl acetate (3 × 50 mL). The combined organic phase was dried over anhydrous Na_2_SO_4_, filtered, and concentrated under reduced pressure to get the crude, which was purified by column chromatography on silica gel (50% EA/Hex). The obtained pure fractions were concentrated under reduced pressure to afford the title compound **78** as a brown oil (0.37 g, 0.75 mmol, 89% Yield). **R**_**f**_ = 0.8 (5% MeOH/DCM); **LCMS** *m*/*z* 495.2 (M + H) (ESI +ve), RT = 2.49 min; **HPLC** RT = 9.24 min, 82.3%.

##### Synthesis of 9-((3aR,3bS,8R,8aS)-2,2-Dimethyl-3a,3b,5,6,8,8a-hexahydro-4*H*-indeno­[1,2-*d*]­[1,3]­dioxol-8-yl)-6-methoxy-9*H*-purine (**79**)

To a stirred solution of **78** (0.32 g, 0.64 mmol, 1.0 equiv) in toluene (6.4 mL) was purged with N_2_ for 15 min at room temperature. To this reaction mixture, tributyltin hydride (0.37 g, 1.29 mmol, 2.0 equiv) and AIBN (0.03 g, 0.22 mmol, 0.35 equiv) were added at room temperature under N_2_ atmosphere. The resulting reaction mixture was heated to 110 °C for 2 h. After completion of the reaction, the reaction mixture was concentrated under reduced pressure to get the crude, which was purified by reversed-phase chromatography (70% MeCN/water). The obtained pure fraction was lyophilized to afford the title compound **79** as a brown solid (0.15 g, 0.43 mmol, 68% Yield). **R**_**f**_ = 0.6 (50% EA/Hex); ^**1**^ **H NMR** (400 MHz, DMSO) δ [ppm] = 8.53 (s, 1H), 8.50 (s, 1H), 5.46 (s, 1H), 5.09 (dd, *J* = 5.2, 6.4 Hz, 1H), 4.76 (s, 1H), 4.47 (dd, *J* = 5.2, 6.8 Hz, 1H), 4.11 (s, 3H), 2.56 (brs, 1H), 2.19–2.11 (m, 1H), 1.93–1.81 (m, 2H), 1.79–1.71 (m, 1H), 1.50 (s, 3H), 1.35–1.21 (m, 2H), 1.25 (s, 3H); **HRMS** (ESI, 3.5 kV) *m*/*z*: [M + H]^+^ calc. for C_18_H_22_N_4_O_3_H^+^ 343.1765, found 343.1760; **LCMS** *m*/*z* 343.2 (M + H) (ESI +ve), RT = 2.11 min; **HPLC** RT = 7.95 min, 84.15%.

##### Synthesis of (1R,2S,3R,7aS)-3-(6-Methoxy-9*H*-purin-9-yl)-2,3,5,6,7,7a-hexahydro-1*H*-indene-1,2-diol (**23e**)

To a stirred solution of **79** (0.14 g, 0.4 mmol, 1.0 equiv) in a mixture of THF (1.4 mL) and water (1.4 mL) was added TFA (0.1 mL) at room temperature. The resulting reaction mixture was stirred at room temperature for 18 h. After completion of the reaction, the reaction mixture was concentrated under reduced pressure to get the crude, which was purified by RP Prep. HPLC. The pure fractions obtained were lyophilized to afford title compound **23e** as a white solid (0.05 g, 0.17 mmol, 40%). **R**_**f**_ = 0.4 (10% MeOH/DCM); ^**1**^ **H NMR** (400 MHz, DMSO) δ [ppm] = 8.52 (s, 1H), 8.40 (s, 1H), 5.27 (s, 1H), 5.18 (d, *J* = 5.2 Hz, 1H), 4.95 (d, *J* = 6.0 Hz, 1H), 4.87 (s, 1H), 4.18 (q, *J* = 5.2, 10.8 Hz, 1H), 4.10 (s, 3H), 3.86 (q, *J* = 6.0, 13.2 Hz, 1H), 2.42 (s, 1H), 2.12–2.07 (m, 1H), 1.91–1.84 (m, 2H), 1.80–1.76 (m, 1H), 1.45–1.42 (m, 1H), 1.23–1.16 (m, 1H); ^**13**^ **C­{** ^**1**^ **H}-NMR** (100 MHz, DMSO) δ [ppm] = 160.7, 152.6, 151.8, 143.6, 138.7, 121.1, 120.7, 75.5, 75.5, 63.1, 54.3, 44.1, 27.0, 24.6, 21.9; **HRMS** (ESI, 3.5 kV) *m*/*z*: [M + H]^+^ calc. for C_15_H_18_N_4_O_3_H^+^ 303.1452, found 303.1468; **LCMS** *m*/*z* 303.0 (M + H) (ESI +ve), RT = 1.60 min; **HPLC** RT = 5.12 min, 100%.

#### Synthesis of (5′S) and 5′-Deoxy-pyrimidine Analogues

##### Synthesis of (3aR,3bR,4S,8R,8aS)-8-(2,4-Dioxo-3,4-dihydropyrimidin-1­(2*H*)-yl)-2,2-dimethyl-3a,3b,5,6,8,8a-hexahydro-4*H*-indeno­[1,2-*d*]­[1,3]­dioxol-4-yl 4-Nitrobenzoate (**40**)

To a stirred solution of **34a** (2.0 g, 6.24 mmol, 1.0 equiv) and 4-nitrobenzoic acid (5.21 g, 31.21 mmol, 5.0 equiv) in anhydrous THF (20 mL) was added triphenylphosphine (8.19 g, 31.21 mmol, 5.0 equiv) followed by dropwise addition of DIAD (6.31 g, 31.21 mmol, 5.0 equiv) at 0 °C under N_2_ atmosphere. The resulting reaction mixture was stirred at room temperature for 16 h. After completion of the reaction, the reaction mixture was diluted with water (150 mL) and extracted with ethyl acetate (3 × 100 mL). The combined organic phase was dried over anhydrous Na_2_SO_4_, filtered, and concentrated to get the crude, which was purified by reversed-phase chromatography using (30% MeCN/water). The pure fractions obtained were lyophilized to afford the title compound **40** as a white solid (1.1 g, 2.34 mmol, 38%). **R**_**f**_ = 0.5 (10% MeOH/DCM); **LCMS** *m*/*z* 470.1 (M + H) (ESI +ve), RT = 2.12 min; **HPLC** RT = 7.76 min, 95.61%.

##### Synthesis of 1-((1R,2S,3R,3aR,4S)-2,3,4-Trihydroxy-2,3,3a,4,5,6-hexahydro-1*H*-inden-1-yl)­pyrimidine-2,4­(1*H*,3*H*)-dione (**2d**)

To a stirred solution of **40** (0.4 g, 0.85 mmol, 1.0 equiv) in a mixture of THF (4.0 mL) and water (4.0 mL) was added TFA (1.2 mL) at room temperature. The resulting reaction mixture was stirred at room temperature for 16 h. After completion of the reaction, the reaction mixture was concentrated under reduced pressure to afford the crude as a brown solid (0.4 g). To a stirred solution of the crude in methanol (4.0 mL) were added sodium methoxide (0.15 g, 2.79 mmol) and NH_4_OH (4.0 mL) at room temperature. The resulting reaction mixture was stirred at room temperature for 16 h. After completion of the reaction, the reaction mixture was concentrated under reduced pressure to get the crude, which was purified by reversed-phase chromatography (45% MeCN/water). The obtained pure fractions were lyophilized to afford the title compound **2d** as a white solid (0.055 g, 0.19 mmol, 23%). **R**_**f**_ = 0.2 (10% MeOH/DCM); ^**1**^ **H NMR** (400 MHz, DMSO) δ [ppm] = 11.29 (s, 1H), 7.39 (d, *J* = 8.0 Hz, 1H), 5.58 (d, *J* = 8.0 Hz, 1H), 5.24–5.23 (m, 1H), 5.10 (brs, 1H), 5.05 (d, *J* = 4.8 Hz, 1H), 4.74 (d, *J* = 4.8 Hz, 1H), 4.50 (d, *J* = 5.9 Hz, 1H), 4.08 (d, *J* = 1.2 Hz, 1H), 3.91 (d, *J* = 4.4 Hz, 1H), 3.64 (q, *J* = 5.2, 10.4 Hz, 1H), 2.34 (brs, 1H), 2.11–2.05 (m, 1H), 1.99–1.94 (m, 1H), 1.78–1.72 (m, 1H), 1.59–1.53 (m, 1H); ^**13**^ **C­{** ^**1**^ **H}-NMR** (100 MHz, DMSO) δ [ppm] = 163.6, 152.0, 143.0, 134.2, 121.1, 101.6, 75.0, 69.9, 62.9, 62.3, 49.1, 28.8, 20.8; **HRMS** (ESI, 3.5 kV) *m*/*z*: [M + H]^+^ calc. for C_13_H_16_N_2_O_5_H^+^ 281.1132, found 281.1131; **LCMS** *m*/*z* 281.0 (M + H) (ESI +ve), RT = 2.00 min; **HPLC** RT = 4.95 min, 100%.

##### Synthesis of 1-((3aR,3bR,4S,8R,8aS)-4-Hydroxy-2,2-dimethyl-3a,3b,5,6,8,8a-hexahydro-4*H*-indeno­[1,2-*d*]­[1,3]­dioxol-8-yl)­pyrimidine-2,4­(1*H*,3*H*)-dione (**80**)

To a stirred solution of **40** (0.6 g, 1.27 mmol, 1.0 equiv) in methanol (6.0 mL) were added sodium methoxide (0.20 g, 3.83 mmol, 3.0 equiv) and NH_4_OH (6.0 mL, 10 vol) at room temperature. The resulting reaction mixture was stirred at room temperature for 16 h. After completion of the reaction, the reaction mixture was concentrated under reduced pressure to get the crude, which was purified by reversed-phase chromatography using (35% MeCN/water). The obtained pure fractions were lyophilized to afford the title compound **80** as a white solid (0.35 g, 1.09 mmol, 85%). **R**_**f**_ = 0.3 (10% MeOH/DCM); ^**1**^ **H NMR** (400 MHz, DMSO) δ [ppm] = 11.37 (brs, 1H), 7.28 (d, *J* = 7.2 Hz, 1H), 5.66 (d, *J* = 7.2 Hz, 1H), 5.34 (s, 1H), 5.22 (s, 1H), 4.72 (d, *J* = 5.2 Hz, 2H), 4.51 (s, 1H), 4.10 (s, 1H), 2.51–2.50 (m, 1H), 2.07 (brs, 1H), 1.94–1.90 (m, 1H), 1.78–1.74 (m, 1H), 1.59–1.55 (m, 1H), 1.44 (s, 3H), 1.26 (s, 3H); **HRMS** (ESI, 3.5 kV) *m*/*z*: [M + H]^+^ calc. for C_16_H_20_N_2_O_5_H^+^ 321.1445, found 321.1442; **LCMS** *m*/*z* 321.0 (M + H) (ESI +ve), RT = 2.14 min; **HPLC** RT = 7.39 min, 91.65%.

##### Synthesis of 4-(Hydroxyimino)-1-((1R,2S,3R,3aR,4S)-2,3,4-trihydroxy-2,3,3a,4,5,6-hexahydro-1*H*-inden-1-yl)-3,4-dihydropyrimidin-2­(1*H*)-one (**3d**)

To a stirred solution of **80** (0.35 g, 1.09 mmol, 1.0 equiv) in HMDS (7.0 mL) were added imidazole (0.037 g, 0.54 mmol, 0.5 equiv), ammonium bisulfate (0.50 g, 4.37 mmol, 4.0 equiv), and hydroxylamine sulfate (0.35 g, 2.18 mmol, 2.0 equiv) at room temperature. The resulting reaction mixture was heated to 85 °C for 16 h. After completion of the reaction, the reaction mixture was concentrated under reduced pressure to afford crude as a brown oil. After redissolving in methanol (3.5 mL, 10 vol), TFA (3.5 mL) at room temperature was added. The resulting reaction mixture was stirred at room temperature for 16 h. After completion of the reaction, the reaction mixture was concentrated under reduced pressure to get the crude, which was purified by reversed-phase chromatography using (35% MeCN/water). The enriched crude was further purified by RP Prep HPLC. The obtained pure fractions were lyophilized to afford the title compound **3d** as a white solid (0.045 g, 0.15 mmol, 14%). **R**_**f**_ = 0.1 (10% MeOH/DCM); ^**1**^ **H NMR** (400 MHz, DMSO) δ [ppm] = 9.86 (s, 1H), 9.34 (s, 1H), 6.58 (d, *J* = 8.4 Hz, 1H), 5.51 (dd, *J* = 1.2, 8.0 Hz, 1H), 5.23 (s, 1H), 5.03 (brs, 1H), 4.92 (d, *J* = 5.2 Hz, 1H), 4.68 (d, *J* = 5.6 Hz, 1H), 4.46 (d, *J* = 4.8 Hz, 1H), 4.07 (s, 1H), 3.89 (dd, *J* = 5.6, 11.2 Hz, 1H), 3.58 (dd, *J* = 5.6, 11.2 Hz, 1H), 2.31 (s, 1H), 2.12–2.07 (m, 1H), 1.98–1.93 (m, 1H), 1.77–1.71 (m, 1H), 1.58–1.50 (m, 1H); ^**13**^ **C­{** ^**1**^ **H}-NMR** (100 MHz, DMSO) δ [ppm] 150.7, 144.4, 134.2, 132.2, 120.4, 98.1, 74.8, 70.0, 62.8, 61.8, 48.9, 28.9, 20.8; **HRMS** (ESI, 3.5 kV) *m*/*z*: [M + H]^+^ calc. for C_13_H_17_N_3_O_5_H^+^ 296.1241, found 296.1245; **LCMS** *m*/*z* 296.0 (M + H) (ESI +ve), RT = 1.90 min; **HPLC** RT = 5.07 min, 100%.

##### Synthesis of (3aR,3bR,4S,8R,8aS)-8-(4-Amino-2-oxopyrimidin-1­(2*H*)-yl)-2,2-dimethyl-3a,3b, 5,6,8,8a-hexahydro-4*H*-indeno­[1,2-*d*]­[1,3]­dioxol-4-yl 4-Nitrobenzoate (**41**)

To a stirred solution of **40** (0.45 g, 0.95 mmol, 1.0 equiv) in acetonitrile (4.5 mL) were added DMAP (0.011 g, 0.09 mmol, 0.1 equiv), TEA (0.4 mL, 2.87 mmol, 3.0 equiv), and 2,4,6-triisopropylbenzenesulfonyl chloride (0.29 g, 0.95 mmol, 1.0 equiv) at room temperature. The resulting reaction mixture was stirred at room temperature for 2 h. After completion of the reaction, the reaction mixture was charged with NH_4_OH (4.5 mL) at room temperature. The resulting reaction mixture was stirred at room temperature for 16 h. After completion of the reaction, the reaction mixture was concentrated under reduced pressure to get the crude, which was purified by reversed-phase chromatography using (55% MeCN/water). The obtained pure fractions were lyophilized to afford the title compound **41** as a white solid (0.35 g, 0.74 mmol, 78%). **R**_**f**_ = 0.4 (5% MeOH/DCM); **HRMS** (ESI, 3.5 kV) *m*/*z*: [M + H]^+^ calc. for C_23_H_24_N_4_O_7_H^+^ 469.1718, found 469.1727; **LCMS** *m*/*z* 469.1 (M + H) (ESI +ve), RT = 1.88 min.

##### Synthesis of 4-Amino-1-((1R,2S,3R,3aR,4S)-2,3,4-trihydroxy-2,3,3a,4,5,6-hexahydro-1*H*-inden-1-yl)­pyrimidin-2­(1*H*)-one (**4d**)

To a stirred solution of **41** (0.35 g, 0.74 mmol, 1.0 equiv) in a mixture of THF (3.5 mL) and water (3.5 mL) was added TFA (3.5 mL) at room temperature. The resulting reaction mixture was stirred at room temperature for 16 h. After completion of the reaction, the reaction mixture was concentrated under reduced pressure to afford the crude as a brown sticky solid (0.30 g). The crude was redissolved in methanol (2.9 mL), and sodium methoxide (0.10 g, 2.03 mmol, 3.0 equiv) and NH_4_OH (2.9 mL) were added at room temperature. The resulting reaction mixture was stirred at room temperature for 16 h. After completion of the reaction, the reaction mixture was concentrated under reduced pressure to get the crude, which was purified by RP Prep HPLC. The obtained pure fractions were lyophilized to afford the title compound **4d** as a white solid (0.050 g, 0.17 mmol, 23%). **R**_**f**_ = 0.1 (10% MeOH/DCM); ^**1**^ **H NMR** (400 MHz, DMSO) δ [ppm] = 7.35 (d, *J* = 7.2 Hz, 1H), 7.03–6.96 (m, 2H), 5.64 (d, *J* = 7.2 Hz, 1H), 5.17 (brs, 1H), 5.11 (s, 1H), 4.86 (brs, 1H), 4.66 (brs, 1H), 4.47 (brs, 1H), 4.08 (s, 1H), 3.90 (s, 1H), 3.58 (s, 1H), 2.34 (brs, 1H), 2.08–2.05 (m, 1H), 1.97–1.92 (m, 1H), 1.78–1.74 (m, 1H), 1.57–1.49 (m, 1H); ^**13**^ **C­{** ^**1**^ **H}-NMR** (100 MHz, DMSO) δ [ppm] = 165.6, 163.6, 156.6, 143.6, 135.6, 120.8, 93.8, 75.6, 70.0, 62.7, 49.0, 28.9, 20.8; **HRMS** (ESI, 3.5 kV) *m*/*z*: [M + H]^+^ calc. for C_13_H_17_N_3_O_4_H^+^ 280.1292, found 280.1282; **LCMS** *m*/*z* 280.0 (M + H) (ESI +ve), RT = 1.77 min; **HPLC** RT = 4.51 min, 100%.

##### Synthesis of (3aR,3bR,4S,8R,8aS)-2,2-Dimethyl-8-(4-(methylamino)-2-oxopyrimidin-1­(2*H*)-yl)-3a,3b,5,6,8,8a-hexahydro-4*H*-indeno­[1,2-*d*]­[1,3]­dioxol-4-yl 4-Nitrobenzoate (**42**)

To a stirred solution of **40** (0.5 g, 1.06 mmol, 1.0 equiv) in acetonitrile (5.0 mL) were added DMAP (0.013 g, 0.10 mmol, 0.1 equiv), TEA (0.44 mL, 3.19 mmol, 3.0 equiv), and 2,4,6-triisopropylbenzenesulfonyl chloride (0.32 g, 1.06 mmol, 1.0 equiv) at room temperature. The resulting reaction mixture was stirred at room temperature for 2 h. After completion of the reaction, the reaction mixture was charged with methylamine (2 M in THF, 5.0 mL) at room temperature. The resulting reaction mixture was stirred at room temperature for 16 h. After completion of the reaction, the reaction mixture was concentrated under reduced pressure to get the crude, which was purified by reversed-phase chromatography (45% MeCN/water). The obtained pure fractions were lyophilized to afford the title compound **42** as a white solid (0.35 g, 0.72 mmol, 68%). **R**_**f**_ = 0.4 (5% MeOH/DCM); **HRMS** (ESI, 3.5 kV) *m*/*z*: [M + H]^+^ calc. for C_24_H_26_N_4_O_7_H^+^ 483.1874, found 483.1902; **LCMS** *m*/*z* = 483.1 (M + H) (ESI +ve), RT = 1.88 min.

##### Synthesis of 4-(Methylamino)-1-((1R,2S,3R,3aR,4S)-2,3,4-trihydroxy-2,3,3a,4,5,6-hexahydro-1*H*-inden-1-yl)­pyrimidin-2­(1*H*)-one (**18d**)

To a stirred solution of **42** (0.35 g, 0.72 mmol, 1.0 equiv) in a mixture of THF (3.5 mL) and water (3.5 mL) was added TFA (3.5 mL) at room temperature. The resulting reaction mixture was stirred at room temperature for 16 h. After completion of the reaction, the reaction mixture was concentrated under reduced pressure to afford the crude as a brown solid, which was dissolved in methanol (3.5 mL), and sodium methoxide (0.12 g, 2.37 mmol, 3.0 equiv) and NH_4_OH (3.5 mL) at room temperature were added. The resulting reaction mixture was stirred at room temperature for 16 h. After completion of the reaction, the reaction mixture was concentrated under reduced pressure to get the crude, which was purified by reversed-phase chromatography using (30% MeCN/water). The enriched crude was further purified by RP Prep HPLC. The obtained pure fractions were lyophilized to afford the title compound **18d** as a white solid (0.043 g, 0.14 mmol, 20%). **Rf** = 0.1 (15% MeOH/DCM); ^**1**^ **H NMR** (400 MHz, DMSO) δ [ppm] = 7.54–7.53 (m, 1H), 7.30 (d, *J* = 7.2 Hz, 1H), 5.66 (d, *J* = 7.6 Hz, 1H), 5.18 (brs, 1H), 5.12 (s, 1H), 4.88 (d, *J* = 4.8 Hz, 1H), 4.67 (d, *J* = 5.2 Hz, 1H), 4.48 (brs, 1H), 4.10 (s, 1H), 3.91 (brs, 1H), 3.60 (d, *J* = 4.0 Hz, 1H), 2.74 (d, *J* = 4.8 Hz, 3H), 2.36 (brs, 1H), 2.10–2.05 (m, 1H), 1.98–1.93 (m, 1H), 1.79–1.74 (m, 1H), 1.58–1.54 (m, 1H); ^**13**^ **C­{** ^**1**^ **H}-NMR** (100 MHz, DMSO) δ [ppm] = 163.9, 156.6, 142.2, 135.6, 120.8, 94.5, 75.6, 70.0, 62.8 (br), 62.7, 49.0, 28.9, 27.3, 20.9; **HRMS** (ESI, 3.5 kV) *m*/*z*: [M + H]^+^ calc. for C_14_H_19_N_3_O_4_H^+^ 294.1448, found, 294.1435; **LCMS** *m*/*z* 294.0 (M + H) (ESI +ve), RT = 1.84 min; **HPLC** RT = 4.79 min, 100%.

#### Synthesis of 5′-Deoxy-pyrimidine Analogues

##### Synthesis of O-((3aR,3bS,8R,8aS)-8-(2,4-Dioxo-3,4-dihydropyrimidin-1­(2*H*)-yl)-2,2-dimethyl-3a,3b,5,6,8,8a-hexahydro-4*H*-indeno­[1,2-*d*]­[1,3]­dioxol-5-yl) *O*-Phenyl Carbonothioate (**81**)

To a stirred solution of **34b/c** (2.50 g, 7.80 mmol, 1.0 equiv) and DMAP (4.77 g, 39.0 mmol, 5.0 equiv) in acetonitrile (12.5 mL) was added O-phenyl carbonochloridothioate (2.69 g, 15.6 mmol, 2.0 equiv) at room temperature. The resulting reaction mixture was stirred at room temperature for 2 h. After completion of the reaction, the reaction mixture was diluted with water (200 mL) and extracted with ethyl acetate (2 × 100 mL). The combined organic phase was dried over anhydrous Na_2_SO_4_, filtered, and concentrated under reduced pressure to get the crude, which was purified by column chromatography on silica gel (1% MeOH/DCM). The obtained pure fractions were concentrated under reduced pressure to afford **81** as a light-brown solid (1.80 g, 3.94 mmol, 50%). **R**_**f**_ = 0.5 (10% MeOH/DCM); **HRMS** (ESI, 3.5 kV) *m*/*z*: [M-H]^−^ calc. for C_23_H_23_N_2_O_6_S^–^ 455.1282, found 455.1275; **LCMS** *m*/*z* = 303.1 (M-153) (ESI +ve), RT = 2.22 min.

##### Synthesis of 1-((3aR,3bS,8R,8aS)-2,2-Dimethyl-3a,3b,5,6,8,8a-hexahydro-4*H*-indeno­[1,2-*d*]­[1,3]­dioxol-8-yl)­pyrimidine-2,4­(1*H*,3*H*)-dione (**43**)

A solution of **81** (1.80 g, 3.94 mmol, 1.0 equiv) in toluene (36 mL) was purged with N_2_ gas for 15 min. To this reaction mixture, tributyltin hydride (2.29 g, 7.88 mmol, 2.0 equiv) and AIBN (0.22 g, 1.38 mmol, 0.35 equiv) were added at room temperature under N_2_ atmosphere. The resulting reaction mixture was heated to 110 °C for 2 h. After completion of the reaction, the reaction mixture was concentrated under reduced pressure to get the crude, which was purified by reversed-phase chromatography (40% MeCN/water). The obtained pure fractions were lyophilized to afford the title compound **43** as a white solid (0.50 g, 1.64 mmol, 41%). **R**_**f**_ = 0.6 (50% EA/Hex); **HRMS** (ESI, 3.5 kV) *m*/*z*: [M + Na]^+^ calc. for C_16_H_20_N_2_O_4_Na^+^ 327.1315, found 327.1315; **LCMS** *m*/*z* = 305.1 (M + H) (ESI +ve), RT = 1.82.

##### Synthesis of 1-((1R,2S,3R,3aS)-2,3-Dihydroxy-2,3,3a,4,5,6-hexahydro-1*H*-inden-1-yl)­pyrimidine-2,4­(1*H*,3*H*)-dione (**2e**)

To a stirred solution of **43** (0.30 g, 0.98 mmol, 1.0 equiv) in a mixture of THF (3.0 mL) and water (3.0 mL) was added TFA (0.3 mL) at room temperature. The resulting reaction mixture was stirred at room temperature for 16 h. After completion of the reaction, the reaction mixture was concentrated under reduced pressure to get the crude, which was purified by RP Prep. HPLC. The obtained pure fraction was lyophilized to afford title compound **2e** as a white solid (0.055 g, 0.21 mmol, 21%). **R**_**f**_ = 0.2 (10% MeOH/DCM); ^**1**^ **H NMR** (400 MHz, DMSO) δ [ppm] = 11.29 (s, 1H), 7.40 (s, 1H), 5.56 (dd, *J* = 1.2, 8.0 Hz, 1H), 5.16 (s, 1H), 5.00 (s, 2H), 4.82 (d, *J* = 6.0 Hz, 1H), 3.82 (s, 1H), 3.58 (s, 1H), 2.25 (s, 1H), 2.04–1.93 (m, 3H), 1.77–1.73 (m, 1H), 1.41–1.36 (m, 1H), 1.13–1.07 (m, 1H); ^**13**^ **C­{** ^**1**^ **H}-NMR** (100 MHz, DMSO) δ [ppm] = 163.7, 151.7, 143.8, 138.1, 120.1, 101.9, 75.5, 74.8, 43.8, 26.9, 24.7, 21.8; **HRMS** (ESI, 3.5 kV) *m*/*z*: [M + H]^+^ calc. for C_13_H_16_N_2_O_4_H^+^ 265.1183, found 265.1180; **LCMS** *m*/*z* = 265.1 (M + H) (ESI +ve), RT = 1.46 min; **HPLC** RT = 4.17 min, 100%. *Note: One C signal could not be observed in 13C-NMR possibly due to peak broadening or overlapping.*

##### Synthesis of 1-((3aR,3bS,8R,8aS)-2,2-Dimethyl-3a,3b,5,6,8,8a-hexahydro-4*H*-indeno­[1,2-*d*] [1,3]­dioxol-8-yl)-4-(hydroxyimino)-3,4-dihydropyrimidin-2­(1*H*)-one (**46**)

To a stirred solution of **43** (0.30 g, 0.98 mmol, 1.0 equiv) in HMDS (6.0 mL) were added imidazole (0.033 g, 0.49 mmol, 0.5 equiv), ammonium bisulfate (0.45 g, 3.94 mmol, 4.0 equiv), and hydroxylamine sulfate (0.32 g, 1.97 mmol, 2.0 equiv) at room temperature. The resulting reaction mixture was heated to 85 °C for 16 h. After completion of the reaction, the reaction mixture was diluted with water (20 mL) and extracted with ethyl acetate (2 × 20 mL). The combined organic phase was washed with brine (2 × 20 mL), dried over anhydrous Na_2_SO_4_, filtered, and concentrated under reduced pressure to get the crude, which was purified by reversed-phase chromatography using (40% MeCN/water). The pure fractions obtained were lyophilized to afford the title compound **46** as a white solid (0.30 g, 0.94 mmol, 95%). **R**_**f**_ = 0.25 (10% MeOH/DCM). **LCMS** *m*/*z* 320.1 (M + H) (ESI +ve), RT = 1.83 min.

##### Synthesis of 1-((1R,2S,3R,3aS)-2,3-Dihydroxy-2,3,3a,4,5,6-hexahydro-1*H*-inden-1-yl)-4-(hydroxyimino)-3,4-dihydropyrimidin-2­(1*H*)-one (**3e**)

To a stirred solution of **46** (0.29 g, 0.90 mmol, 1.0 equiv) in methanol (2.9 mL) was added TFA (1.45 mL) at 0 °C. The resulting reaction mixture was stirred at room temperature for 16 h. After completion of the reaction, the reaction mixture was concentrated to give the crude, which was purified by RP Prep HPLC. The obtained pure fractions were lyophilized to afford the title compound **3e** as a white solid (0.12 g, 0.43 mmol, 47%). **R**_**f**_ = 0.1 (10% MeOH/DCM); ^**1**^ **H NMR** (400 MHz, DMSO) δ [ppm] = 9.88 (s, 1H), 9.35 (s, 1H), 6.56 (brs, 1H), 5.49 (d, *J* = 8.0 Hz, 1H), 5.21 (s, 1H), 5.02 (brs, 1H), 4.92 (d, *J* = 4.4 Hz, 1H), 4.78 (d, *J* = 6.0 Hz, 1H), 3.77 (brs, 1H), 3.52 (brs, 1H), 2.25 (brs, 1H), 2.08–1.95 (m, 3H), 1.78–1.74 (m, 1H), 1.45–1.37 (m, 1H), 1.12–1.01 (m, 1H); ^**13**^ **C­{** ^**1**^ **H}-NMR** (100 MHz, DMSO) δ [ppm] = 150.4, 144.4, 138.1, 132.7, 119.8, 98.4, 75.9, 74.6, 62.7, 43.6, 27.0, 24.7, 21.9; **HRMS** (ESI, 3.5 kV) *m*/*z*: [M + H]^+^ calc for C_13_H_17_N_3_O_4_H^+^ 280.1292, found 280.1282; **LCMS** *m*/*z* 280.1 (M + H) (ESI +ve), RT = 1.44 min; **HPLC** RT = 4.35 min, 100%.

##### Synthesis of 4-Amino-1-((3aR,3bS,8R,8aS)-2,2-dimethyl-3a,3b,5,6,8,8a-hexahydro-4*H*-indeno­[1,2-*d*]­[1,3]­dioxol-8-yl)­pyrimidin-2­(1*H*)-one (**44**)

To a stirred solution of **43** (0.69 g, 2.23 mmol, 1.0 equiv) in acetonitrile (7.0 mL) were added TEA (1.14 g, 11.34 mmol, 5.0 equiv) and 2,4,6-triisopropylbenzenesulfonyl chloride (1.36 g, 4.53 mmol, 2.0 equiv) at room temperature. After 5 min, DMAP (0.028 g, 0.23 mmol, 0.1 equiv) was added, and the resulting reaction mixture was stirred at room temperature for 2 h. After completion of the reaction, the reaction mixture was charged with NH_4_OH (7.0 mL) and was stirred at room temperature for 16 h. After completion of the reaction, the reaction mixture was diluted with water (200 mL) and extracted with ethyl acetate (3 × 60 mL). The combined organic phase was dried over anhydrous Na_2_SO_4_, filtered, and concentrated under reduced pressure to get the crude, which was purified by reversed-phase chromatography (26% MeCN/water). The pure fractions obtained were lyophilized to afford the title compound **44** as a brown oil (0.60 g, 1.97 mmol, 87%). **R**_**f**_ = 0.5 (10% MeOH/DCM); **HRMS** (ESI, 3.5 kV) *m*/*z*: [M + H]^+^ calc. for C_16_H_21_N_3_O_3_H^+^ 304.1656, found 304.1652. **LCMS** *m*/*z* = 304.0 (M + H) (ESI +ve), RT = 1.63 min; **HPLC** RT = 5.54 min, 83.19%.

##### Synthesis of 4-Amino-1-((1R,2S,3R,3aS)-2,3-dihydroxy-2,3,3a,4,5,6-hexahydro-1*H*-inden-1-yl)­pyrimidin-2­(1*H*)-one (**4e**)

To a stirred solution of **44** (0.60 g, 1.97 mmol, 1.0 equiv) in a mixture of THF (6.0 mL) and water (6.0 mL) was added TFA (0.6 mL) at room temperature. The resulting reaction mixture was stirred at room temperature for 18 h. After completion of the reaction, the reaction mixture was concentrated under reduced pressure, neutralized using NH_4_OH (pH ∼ 7.0), and again concentrated under reduced pressure to get the crude, which was purified by reversed-phase chromatography using 0.1% NH_4_OH in water (30% MeCN/water). The enriched crude was further purified by RP Prep HPLC. The obtained pure fractions were lyophilized to afford the title compound **4e** as a white solid (0.04 g, 0.15 mmol, 7%). **R**_**f**_ = 0.3 (20% MeOH/DCM, 1% NH_4_OH); ^**1**^ **H NMR** (400 MHz, DMSO) δ [ppm] = 7.31 (s, 1H), 7.06 (s, 1H), 7.01 (s, 1H), 5.66 (d, *J* = 7.6 Hz, 1H), 5.25 (brs, 1H), 5.03 (s, 1H), 4.89 (s, 1H), 4.77 (d, *J* = 5.2 Hz, 1H), 3.76 (s, 1H), 3.57 (s, 1H), 2.28 (s, 1H), 2.08–1.90 (m, 3H), 1.78–1.75 (m, 1H), 1.41–1.38 (m, 1H), 1.15–1.02 (m, 1H); ^**13**^ **C­{** ^**1**^ **H}-NMR** (100 MHz, DMSO) δ [ppm] = 165.8, 139.6, 119.8, 94.1, 76.1, 75.5, 43.7, 27.1, 24.8, 21.9; **HRMS** (ESI, 3.5 kV) *m*/*z*: [M + H]^+^ calc. for C_13_H_17_N_3_O_3_H^+^ 264.1343, found 264.1351; **LCMS** *m*/*z* = 264.1 (M + H) (ESI +ve), RT = 1.33 min; **HPLC** RT = 5.36 min, 100%. *Note: Due to rotamers/conformers peak broadening was observed, leading to a lower carbon count.*

##### Synthesis of 1-((3aR,3bS,8R,8aS)-2,2-Dimethyl-3a,3b,5,6,8,8a-hexahydro-4*H*-indeno­[1,2-*d*] [1,3]­dioxol-8-yl)-4-(methylamino)­pyrimidin-2­(1*H*)-one (**45**)

To a stirred solution of **43** (0.30 g, 0.98 mmol, 1.0 equiv) in acetonitrile (3.0 mL) were added DMAP (0.012 g, 0.09 mmol, 0.1 equiv), TEA (0.68 mL, 4.93 mmol, 5.0 equiv), and 2,4,6-triisopropylbenzenesulfonyl chloride (0.35 g, 1.18 mmol, 1.2 equiv) at room temperature. The resulting reaction mixture was stirred at room temperature for 2 h. After completion of the reaction, the reaction mixture was charged with methylamine (2 M in THF, 3.0 mL) at room temperature. The resulting reaction mixture was stirred at room temperature for 16 h. After completion of the reaction, the reaction mixture was concentrated under reduced pressure to give the crude, which was purified by reversed-phase chromatography using 0.1% NH_4_OH in water (60% MeCN/water). The obtained pure fractions were lyophilized to afford the title **45** compound as a white solid (0.28 g, 0.88 mmol, 89%). **R**_**f**_ = 0.3 (10% MeOH/DCM); **LCMS** *m*/*z* 318.1 (M + H) (ESI +ve), RT = 1.65 min.

##### Synthesis of 1-((1R,2S,3R,3aS)-2,3-Dihydroxy-2,3,3a,4,5,6-hexahydro-1*H*-inden-1-yl)-4-(methylamino)­pyrimidin-2­(1*H*)-one (**18e**)

To a stirred solution of **45** (0.28 g, 0.88 mmol, 1.0 equiv) in a mixture of THF (2.8 mL) and water (2.8 mL) was added TFA (0.28 mL) at room temperature. The resulting reaction mixture was stirred at room temperature for 16 h. After completion of the reaction, the reaction mixture was concentrated under reduced pressure to get the crude, which was purified by RP Prep HPLC. The obtained pure fractions were lyophilized to afford the title compound **18e** as a white solid (0.048 g, 0.17 mmol, 20%). **R**_**f**_ = 0.1 (10% MeOH/DCM); ^**1**^ **H NMR** (400 MHz, DMSO) δ [ppm] = 7.55 (d, *J* = 4.8 Hz, 1H), 7.24 (brs, 1H), 5.67 (d, *J* = 7.2 Hz, 1H), 5.20 (brs, 1H), 5.03 (s, 1H), 4.87 (s, 1H), 4.77 (d, *J* = 6.0 Hz, 1H), 3.76 (brs, 1H) 3.57 (brs, 1H), 2.75 (d, *J* = 4.8 Hz, 3H), 2.28 (brs, 1H), 2.08–2.02 (m, 1H), 1.96–1.87 (m, 2H), 1.78–1.75 (m, 1H), 1.41–1.36 (m, 1H), 1.12–1.09 (m, 1H); ^**13**^ **C­{** ^**1**^ **H}-NMR** (100 MHz, DMSO) δ [ppm] = 164.0, 156.3, 142.6, 139.5, 119.6, 94.7, 76.0, 75.4, 64.1 (br), 43.7, 27.2, 27.0, 24.7, 21.9; **HRMS** (ESI, 3.5 kV) *m*/*z*: [M + H]^+^ calc for C_14_H_19_N_3_O_3_H^+^ 278.1499, found 278.1526; **LCMS** *m*/*z* 278.1 (M + H) (ESI +ve), RT = 2.06 min; **HPLC** RT = 5.69 min, 100%.

#### Synthesis of 3′-F Purine Analogues

##### Synthesis of (1R,2S,3R,7R,7aS)-3-(6-Amino-9*H*-purin-9-yl)-2,7-bis­((*tert*-butyldimethylsilyl) oxy)-2,3,5,6,7,7a-hexahydro-1*H*-inden-1-ol (**47**)

To a stirred solution of **5a** (1.20 g, 3.96 mmol, 1.0 equiv) in pyridine (18.0 mL) was added TBSCl (4.17 g, 27.7 mmol, 7.0 equiv) at room temperature. The resulting reaction mixture was heated at 50 °C for 5 h. After completion of the reaction, the reaction mixture was concentrated under reduced pressure to give the crude, which was purified by reversed-phase chromatography using (80% MeCN/water). The obtained pure fractions were lyophilized to afford the title compound **47** as a white solid (0.70 g, 1.31 mmol, 33%). **R**_**f**_ = 0.5 (50% EA/hex); ^**1**^ **H NMR** (400 MHz, DMSO) δ [ppm] = 8.22 (s, 1H), 8.10 (s, 1H), 7.20 (s, 2H), 5.37 (d, *J* = 8.0 Hz, 1H), 4.81 (s, 1H), 4.39–4.36 (m, 2H), 3.93–3.90 (m, 1H), 3.73–3.67 (m, 1H), 2.40 (d, *J* = 7.6 Hz, 1H), 2.11 (s, 2H), 1.85–1.82 (m, 1H), 1.54–1.48 (m, 1H), 0.91 (s, 9H), 0.65 (s, 9H), 0.12 (s, 3H), 0.11 (s, 3H), −0.11 (s, 3H), −0.32 (s, 3H); ^**13**^ **C­{** ^**1**^ **H}-NMR** (400 MHz, DMSO) δ [ppm] = 156.4, 152.6, 150.5, 140.5, 135.3, 119.3, 119.2, 76.9, 71.3, 71.0, 61.8, 54.0, 32.2, 26.2, 25.9, 24.9, 18.3, 18.1, −3.7, −4.2, −4.5, −5.0; **HRMS** (ESI, 3.5 kV) *m*/*z*: [M + H]^+^ calc for C_26_H_45_N_5_O_3_Si_2_H+ 532.3134, found 532.3126; **LCMS** *m*/*z* 532.4 (M + H) (ESI +ve), RT = 2.87 min; **HPLC** RT = 11.12 min, 96.1%.

##### Synthesis of (1R,2S,3S,3aR,4R)-1-(6-Amino-9*H*-purin-9-yl)-3-fluoro-2,3,3a,4,5,6-hexahydro-1*H*-indene-2,4-diol (**5g**)

To a stirred solution of **47** (0.35 g, 0.66 mmol, 1.0 equiv) in DCM (7.0 mL) was added DAST (0.14 g, 0.85 mmol, 1.3 equiv) at −78 °C. The resulting reaction mixture was stirred at −78 °C to room temperature for 30 min. After completion of the reaction, the reaction mixture was diluted with water (100 mL) and extracted with DCM (3 × 50 mL). The combined organic phase was dried over anhydrous Na_2_SO_4_, filtered, and concentrated under reduced pressure to afford the crude as a brown solid (0.36 g). The crude was redissolved in a mixture of THF (3.5 mL), water (3.5 mL) and TFA (0.4 mL) at room temperature. The resulting reaction mixture was then stirred at room temperature for 16 h. After completion of the reaction, the reaction mixture was concentrated under reduced pressure to get the crude, which was purified by RP Prep HPLC. The obtained pure fractions were lyophilized to afford the title compound **5g** as a white solid (0.050 g, 0.16 mmol, 25% over 2 steps). **R**_**f**_ = 0.1 (10% MeOH/DCM); ^**1**^ **H NMR** (400 MHz, DMSO-d6/H2O) δ [ppm] = 8.68 (brs, 2H), 8.42 (s, 1H), 8.01 (s, 1H), 6.02 (brs, 2H), 5.33–5.32 (m, 2H), 5.04 (dd, *J* = 4.0, 51.3 Hz, 1H), 4.32 (dd, *J* = 1.8, 16.7 Hz, 1H), 3.77 (ddd, *J* = 3.2, 8.9, 11.6 Hz, 1H), 2.84–2.68 (m, 1H), 2.14–2.06 (m, 1H), 1.95–1.85 (m, 1H), 1.56–1.43 (m, 1H); ^**13**^ **C­{** ^**1**^ **H}-NMR** (100 MHz, DMSO) δ [ppm] = 152.3, 149.2, 147.8, 141.1 (d, *J* = 6.4 Hz), 137.2, 123.4, 118.1, 96.1 (d, *J* = 178 Hz), 79.2 (d, *J* = 27.8 Hz), 63.9 (d, *J* = 7.3 Hz), 62.4, 50.0 (d, *J* = 20.4 Hz), 31.1, 24.8; **HRMS** (ESI, 3.5 kV) *m*/*z*: [M + H]^+^ calc for C_14_H_16_FN_5_O_2_H^+^ 306.1361, found 306.1357; **LCMS** *m*/*z* 306.1 (M + H) (ESI +ve), RT = 1.30 min; **HPLC** RT = 4.48 min, 100%.

##### Synthesis of (1R,2S,3R,7R,7aS)-2,7-Bis­((*tert*-butyldimethylsilyl)­oxy)-3-(6-(dimethylamino)-9*H*-purin-9-yl)-2,3,5,6,7,7a-hexahydro-1*H*-inden-1-ol (**48**)

To a stirred solution of **6a** (1.6 g, 4.83 mmol, 1.0 equiv) in pyridine (32 mL) was added TBSCl (6.54 g, 43.5 mmol, 9.0 equiv) at room temperature. The resulting reaction mixture was heated at 50 °C for 4 h. After completion of the reaction, the reaction mixture was concentrated under reduced pressure to get the crude, which was purified by reversed-phase chromatography using (90% MeCN/water). The obtained pure fractions were lyophilized to afford the title compound as a white solid (0.7 g, 1.25 mmol, 26% Yield). **R**_**f**_ = 0.3 (50% EA/hex); **LCMS** *m*/*z* 560.4 (M + H) (ESI +ve), RT = 1.96 min.

##### Synthesis of (1R,2S,3S,3aR,4R)-1-(6-(Dimethylamino)-9*H*-purin-9-yl)-3-fluoro-2,3,3a,4,5,6-hexahydro -1*H*-indene-2,4-diol (**6g**)

To a stirred solution of **48** (0.4 g, 1.20 mmol, 1.0 equiv) in DCM (8.0 mL, 20 vol) was added DAST (0.42 g, 2.64 mmol, 2.2 equiv) at 0 °C. The resulting reaction mixture was stirred at room temperature for 1 h. After completion of the reaction, the reaction mixture was diluted with DCM (20 mL) and washed with water (2 × 20 mL). The organic phase was dried over anhydrous Na_2_SO_4_, filtered, and concentrated under reduced pressure to afford the crude as a brown oil (0.4 g). The crude was redissolved in a mixture of THF (4.0 mL) and water (4.0 mL) was added TFA (0.8 mL, 2.0 vol) at room temperature. The resulting reaction mixture was stirred at room temperature for 16 h. After completion of the reaction, the reaction mixture was concentrated under reduced pressure to get the crude, which was purified by RP Prep HPLC. The obtained pure fractions were lyophilized to afford the title compound **6g** as a white solid (0.048 g, 0.14 mmol, 20% over 2 steps). **R**_**f**_ = 0.1 (10% MeOH/DCM); ^**1**^ **H NMR** (400 MHz, DMSO) δ [ppm] = 8.24 (s, 1H), 7.78 (s, 1H), 5.99 (brs, 1H), 5.29 (s, 1H), 5.15 (d, *J* = 2.4 Hz, 1H), 5.05 (dd, *J* = 4.4, 76.0 Hz, 1H), 5.02 (brs, 1H), 4.26 (dd, *J* = 2.0, 16.8 Hz, 1H), 3.78–3.72 (m, 1H) 3.45 (brs, 6H), 2.80–2.70 (m, 1H), 2.10–2.07 (m, 2H), 1.90–1.86 (m, 1H), 1.51–1.48 (m, 1H). ^**13**^ **C­{** ^**1**^ **H}-NMR** (100 MHz, DMSO) δ [ppm] = 154.6, 152.3, 151.1, 138.3 (d, *J* = 7.2 Hz), 138.2, 123.3, 119.2, 96.5 (d, *J* = 178 Hz), 79.6 (d, *J* = 26.9 Hz), 64.3 (d, *J* = 6.9 Hz), 62.3, 50.4 (d, *J* = 19.7 Hz), 38.3, 31.6, 25.1; **HRMS** (ESI, 3.5 kV) *m*/*z*: [M + H]^+^ calc for C_16_H_20_FN_5_O_2_H^+^ 334.1674, found 334.1682; **LCMS** *m*/*z* 334.2 (M + H) (ESI +ve), RT = 1.49 min; **HPLC** RT = 7.37 min, 100%.

#### Synthesis of 2′-Purine Analogues

##### Synthesis of (5aR,5a1R,6S,7R,10aR)-7-(6-Amino-9*H*-purin-9-yl)-2,2,4,4-tetraisopropyl-5a,5a1,7,9,10,10a-hexahydro-6*H*-indeno­[1,7-fg]­[1,3,5,2,4]­trioxadisilocin-6-ol (**49)**

To a stirred solution, **5a** (3.3 g, 10.9 mmol, 1.0 equiv) in pyridine (49.5 mL) was added 1,3-dichloro-1,1,3,3-tetraisopropyl disiloxane (4.46 g, 14.1 mmol, 1.3 equiv) at 0 °C. The resulting reaction mixture was stirred at 0 °C for 3 h. After completion of the reaction, the reaction mixture was concentrated under reduced pressure to get the crude, which was purified by reversed-phase chromatography using (83% MeCN/water). The obtained pure fractions were lyophilized to afford the title compound **49** as a white solid (1.50 g, 2.75 mmol, 25% Yield). **R**_**f**_ = 0.4 (50% EA/hex); **HRMS** (ESI, 3.5 kV) *m*/*z*: [M + H]^+^ calc for C_26_H_43_N_5_O_4_Si_2_H+ 546.2926, found 546.2926; **LCMS** *m*/*z* 546.4 (M + H) (ESI +ve), RT = 2.21 min.

##### Synthesis of *O*-((5aR,5a1R,6S,7R,10aR)-7-(6-Amino-9*H*-purin-9-yl)-2,2,4,4-tetraisopropyl-5a, 5a1,7,9,10,10a-hexahydro-6*H*-indeno­[1,7-fg]­[1,3,5,2,4]­trioxadisilocin-6-yl) *O*-Phenyl Carbonothioate (**82**)

To a stirred solution **49** (0.87 g, 1.59 mmol, 1.0 equiv) in acetonitrile (4.3 mL) was added DMAP (0.97 g, 7.96 mmol, 5.0 equiv), followed by the addition of O-phenyl carbonochloridothioate (0.55 g, 3.19 mmol, 2.0 equiv) at room temperature. The resulting reaction mixture was stirred at room temperature for 1 h. After completion of the reaction, the reaction mixture was concentrated under reduced pressure to get the crude, which was purified by reversed-phase chromatography using MeCN/water (100% MeCN). The obtained pure fractions were lyophilized to afford the title compound **82** as a white solid (0.33 g, 0.48 mmol, 30%). **R**_**f**_ = 0.5 (30% EA/hex); **HRMS** (ESI, 3.5 kV) *m*/*z*: [M + H]^+^ calc C_33_H_47_N_5_O_5_SSi_2_H^+^ 682.2909, found 682.2898; **LCMS** *m*/*z* 682.4 (M + H) (ESI +ve), RT = 2.68 min.

##### Synthesis of (1S,3S,7R,7aR)-3-(6-Amino-9*H*-purin-9-yl)-2,3,5,6,7,7a-hexahydro-1*H*-indene-1,7-diol (**5f**)

A solution of **82** (0.32 g, 0.46 mmol, 1.0 equiv) in toluene (6.4 mL, 20 vol) was purged with N_2_ for 15 min. To this reaction mixture, tributyltin hydride (0.27 g, 0.94 mmol, 2.0 equiv) and AIBN (0.027 g, 0.16 mmol, 0.35 equiv) were added at room temperature. The resulting reaction mixture was heated to 110 °C for 3 h. After completion of the reaction, the reaction mixture was concentrated under reduced pressure to afford the crude as a brown oil (0.30 g). The crude was redissolved in a mixture of 1,4-dioxane (3.0 mL) and water (3.0 mL) was added 4 M HCl in dioxane (3.0 mL) at room temperature. The resulting reaction mixture was stirred at room temperature for 16 h. After completion of the reaction, the reaction mixture was concentrated under reduced pressure to get the crude, which was purified by RP Prep HPLC. The obtained pure fractions were lyophilized to afford the title compound **5f** as a white solid (0.06 g, 0.21 mmol, 43%). **R**_**f**_ = 0.1 (5% MeOH/DCM); ^**1**^ **H NMR** (400 MHz, DMSO) δ [ppm] = 8.119 (s, 1H), 8.113 (s, 1H), 7.20 (brs, 2H), 5.49 (s, 1H), 4.89 (brs, 1H), 4.76 (s, 1H), 4.68 (s, 1H), 4.30 (q, *J* = 6.0, 12.4 Hz, 1H), 3.48 (dd, *J* = 8.4, 8.4 Hz, 1H), 2.39–2.27 (m, 1H), 2.21 (brs, 1H), 2.13–2.06 (m, 1H), 2.01–1.96 (m, 2H), 1.80–1.76 (m, 1H), 1.44–1.37 (m, 1H); ^**13**^ **C­{** ^**1**^ **H}-NMR** (100 MHz, DMSO) δ [ppm] = 156.4, 152.8, 149.9, 140.9, 140.2, 119.1, 119.1, 73.0, 70.7, 55.5, 53.9, 40.6, 31.5, 24.9; **HRMS** (ESI, 3.5 kV) *m*/*z*: [M + H]^+^ calc for C_14_H_17_N_5_O_2_H^+^ 288.1455, found 288.1449; **LCMS** *m*/*z* 288.3 (M + H) (ESI +ve), RT = 0.92 min; **HPLC** RT = 3.78 min, 99.67%.

##### Synthesis of (1R,2R,3R,7R,7aR)-3-(6-Amino-9*H*-purin-9-yl)-2-fluoro-2,3,5,6,7,7a-hexahydro-1*H*-indene-1,7-diol (**5h**)

To a stirred solution of **49** (0.5 g, 0.92 mmol, 1.0 equiv) in DCM (10 mL) was added DAST (0.32 g, 2.01 mmol, 2.2 equiv) at −70 °C. The resulting reaction mixture was allowed to reach room temperature after 30 min. After completion of the reaction, the reaction mixture was diluted with water (100 mL) and extracted with DCM (3 × 100 mL). The combined organic phase was dried over anhydrous Na_2_SO_4_, filtered, and concentrated under reduced pressure to afford the crude as a yellow solid (0.47 g). The crude was redissolved in methanol (9.0 mL, 20 vol) and ammonium fluoride (0.30 g, 8.21 mmol, 10 equiv) was added at room temperature. The resulting reaction mixture was heated to 50 °C for 16 h. After completion of the reaction, the reaction mixture was concentrated under reduced pressure to get the crude, which was purified by RP Prep HPLC. The obtained pure fractions were lyophilized to afford the title compound **5h** as a white solid (20 mg, 0.06 mmol, 8% over 2 steps). **R**_**f**_ = 0.1 (10% MeOH/DCM); ^**1**^ **H NMR** (400 MHz, DMSO) δ [ppm] = 8.15 (s, 1H), 7.94 (d, *J* = 2.4 Hz, 1H), 7.31 (s, 2H), 5.68 (d, *J* = 25.6 Hz, 1H), 5.52 (d, *J* = 5.2 Hz, 1H), 5.08 (s, 1H), 4.91­(d, *J* = 5.2 Hz, 1H), 4.79 (d, *J* = 2.0 Hz, 1H), 4.19 (d, *J* = 20.4 Hz, 1H), 3.51–3.45 (m, 1H), 2.25 (brs, 1H), 2.13–2.11 (m, 2H), 1.85–1.83 (m, 1H), 1.52–1.41 (m, 1H); **HRMS** (ESI, 3.5 kV) *m*/*z*: [M + H]^+^ calc for C_14_H_16_FN_5_O_2_H+ 306.1361, found 306.1357; **LCMS** *m*/*z* 306.0 (M + H) (ESI +ve), RT = 1.28 min; **HPLC** RT = 4.55 min, 100%.

##### Synthesis of (5aR,5a1R,6S,7R,10aR)-7-(6-(Dimethylamino)-9*H*-purin-9-yl)-2,2,4,4-tetraisopropyl-5a,5a1,7,9,10,10a-hexahydro-6*H*-indeno­[1,7-fg]­[1,3,5,2,4]­trioxadisilocin-6-ol (**50**)

To a stirred solution of **6a** (2.50 g, 7.54 mmol, 1.0 equiv) in pyridine (37.5 mL) was added 1,3-dichloro-1,1,3,3-tetraisopropyl disiloxane (3.33 g, 10.56 mmol, 1.4 equiv) at 0 °C. The resulting reaction mixture was stirred at 0 °C for 2 h. After completion of the reaction, the reaction mixture was concentrated under reduced pressure to get the crude, which was purified by reversed-phase chromatography using water/MeCN (100% MeCN). The pure fractions obtained were lyophilized to afford the title compound **50** as a brown sticky solid (1.50 g, 2.62 mmol, 35%). **R**_**f**_ = 0.5 (30% EA/hex); **LCMS** *m*/*z* 574.9 (M + H) (ESI +ve), RT = 2.33 min.

##### Synthesis of *O*-((5aR,5a1R,7R,10aR)-7-(6-(Dimethylamino)-9*H*-purin-9-yl)-2,2,4,4-tetra isopropyl-5a,5a1,7,9,10,10a-hexahydro-6*H*-indeno­[1,7-fg]­[1,3,5,2,4]­trioxadisilocin-6-yl) *O*-Phenyl Carbonothioate (**83**)

To a stirred solution of **50** (0.50 g, 0.87 mmol, 1.0 equiv) and DMAP (0.53 g, 4.36 mmol, 5.0 equiv) in acetonitrile (2.5 mL, 5.0 vol) was added O-phenyl carbonochloridothioate (0.30 g, 1.74 mmol, 2.0 equiv) at room temperature. The resulting reaction mixture was stirred at room temperature for 2 h. After completion of the reaction, the reaction mixture was diluted with water (100 mL) and extracted with ethyl acetate (2 × 70 mL). The combined organic phase was dried over anhydrous Na_2_SO_4_, filtered, and concentrated under reduced pressure to get the crude, which was purified by column chromatography using silica gel (40% EA/Hex) to afford the title compound **83** as a light-brown liquid (0.37 g, 0.52 mmol, 60% yield). **R**_**f**_ = 0.5 (30% EtOAc/Hexane); **HRMS** (ESI, 3.5 kV) *m*/*z*: [M + H]^+^ calc for C_35_H_51_N_5_O_5_SSi_2_H^+^ 710.3222, found 710.3231; **LCMS** *m*/*z* = 710.36 (M + H) (ESI +ve), RT = 7.78 min.

##### Synthesis of *N*,*N*-Dimethyl-9-((5aS,5a1R,7S,10aR)-2,2,4,4-tetraisopropyl-5a,5a1,7,9,10,10a-hexahydro-6*H*-indeno­[1,7-fg]­[1,3,5,2,4]­trioxadisilocin-7-yl)-9*H*-purin-6-amine (**84**)

A stirred solution of **83** (0.25 g, 0.35 mmol, 1.0 equiv) in toluene (10 mL) was purged with N_2_ gas for 30 min. To this reaction mixture, tributyltin hydride (0.41 g, 1.41 mmol, 4.0 equiv) and AIBN (0.05 g, 0.28 mmol, 0.8 equiv) were added at room temperature. The resulting reaction mixture was heated to 110 °C for 3 h. After completion of the reaction, the reaction mixture was concentrated under reduced pressure to get the crude, which was purified by column chromatography using silica gel (20% EA/Hex) to afford the title compound **84** as a light-brown liquid (0.2 g, quantitative yield). **R**_**f**_ = 0.5 (30% EA/Hex); **LCMS** *m*/*z* = 558.7 (M + H) (ESI +ve), RT = 2.68 min.

##### Synthesis of (1S,3S,7R,7aR)-3-(6-(Dimethylamino)-9*H*-purin-9-yl)-2,3,5,6,7,7a-hexahydro-1*H*-indene-1,7-diol (**6f**)

To a stirred solution of **84** (0.2 g, 0.36 mmol, 1.0 equiv) in a mixture of 1,4-dioxane (2.0 mL) and water (2.0 mL) was added 4 M HCl in dioxane (1.0 mL) at room temperature. The resulting reaction mixture was stirred at room temperature for 5 h. After completion of the reaction, the reaction mixture was concentrated under reduced pressure to get the crude, which was purified by RP Prep. HPLC. The obtained pure fraction was lyophilized to afford title compound **6f** as an off-white solid (0.03 g, 0.095 mmol, 27%). **R**_**f**_ = 0.2 (10% MeOH/DCM); ^**1**^ **H NMR** (400 MHz, DMSO) δ [ppm] = 8.19 (s, 1H), 8.12 (s, 1H), 5.52 (brs, 1H), 4.90 (d, *J* = 4.8 Hz, 1H), 4.74–4.69 (m, 2H), 4.30–4.27 (m, 1H), 3.51–3.44 (m, 7H), 2.32–2.25 (s, 1H), 2.21 (s, 1H), 2.13–2.06 (m, 1H), 2.00–1.96 (m, 2H), 1.79–1.76 (m, 1H), 1.44–1.38 (m, 1H); ^**13**^ **C­{** ^**1**^ **H}-NMR** (100 MHz, DMSO) δ [ppm] = 154.6, 152.1, 150.7, 140.9, 139.0, 119.6, 119.1, 73.0, 70.7, 55.5, 53.7, 38.4, 31.5, 24.9; **HRMS** (ESI, 3.5 kV) *m*/*z*: [M + H]^+^ calc. for C_16_H_21_N_5_O_2_H^+^ 316.1768, found 316.1772; **LCMS** *m*/*z* = 316.2 (M + H) (ESI +ve), RT = 1.43 min; **HPLC** RT = 3.78 min, 99.76%. *Note: One C signal could not be observed in ^13^C-NMR possibly due to peak broadening or overlapping.*

##### Synthesis of *N*,*N*-Dimethyl-9-((5aR,5a1R,6S,7R,10aR)-2,2,4,4-tetraisopropyl-6-methoxy-5a,5a1,7,9,10,10a-hexahydro-6*H*-indeno­[1,7-fg]­[1,3,5,2,4]­trioxadisilocin-7-yl)-9*H*-purin-6-amine (**85**)

To a stirred solution of **50** (0.46 g, 0.80 mmol, 1.0 equiv) in DMF (9.2 mL) was added sodium hydride (60% in mineral oil, 0.041 g, 1.04 mmol, 1.3 equiv), followed by the addition of methyl iodide (0.28 g, 0.10 mmol, 2.5 equiv) at 0 °C. The resulting reaction mixture was stirred at room temperature for 1 h. After completion of the reaction, the reaction mixture was diluted with cold water (30 mL) and extracted with ethyl acetate (2 × 25 mL). The combined organic layer was dried over anhydrous Na_2_SO_4_, filtered, and concentrated under reduced pressure to get the crude, which was purified by column chromatography using silica gel (15% EA/hex). The obtained pure fractions were concentrated under reduced pressure to afford the title compound **85** as a white solid (0.22 g, 0.37 mmol, 47%). **R**_**f**_ = 0.5 (50% EA/hex); **HRMS** (ESI, 3.5 kV) *m*/*z*: [M + H]^+^ calc for C_29_H_49_N_5_O_4_Si_2_H^+^ 588.3396, found 588.3390; **LCMS** *m*/*z* 588.4 (M + H) (ESI +ve), RT = 2.51 min.

##### Synthesis of (1R,2S,3R,7R,7aR)-3-(6-(Dimethylamino)-9*H*-purin-9-yl)-2-methoxy-2,3,5,6,7,7a-hexahydro-1*H*-indene-1,7-diol (**6j**)

To a stirred solution of **85** (0.22 g, 0.37 mmol, 1.0 equiv) in a mixture of 1,4-dioxane (2.2 mL) and water (2.2 mL) was added 4 M HCl in dioxane (2.2 mL) at room temperature. The resulting reaction mixture was stirred at room temperature for 16 h. After completion of the reaction, the reaction mixture was concentrated under reduced pressure to get the crude, which was purified by RP Prep HPLC. The obtained pure fractions were lyophilized to afford the title compound **6j** as a white solid (0.06 g, 0.17 mmol, 46%). **R**_**f**_ = 0.33 (50% EA/hex); ^**1**^ **H NMR** (400 MHz, DMSO) δ [ppm] = 8.21 (s, 1H), 8.19 (s, 1H), 5.39 (d, *J* = 1.6 Hz, 1H), 4.85 (d, *J* = 5.2 Hz, 2H), 4.68 (dd, *J* = 2.4, 2.4 Hz, 1H), 4.21 (q, *J* = 5.2, 8.8 Hz, 1H), 3.96 (dd, *J* = 5.6, 8.8 Hz, 1H), 3.60–3.30 (m, 7H), 3.25 (s, 3H), 2.33–2.31 (m, 1H), 2.04–2.00 (m, 2H), 1.83–1.80 (m, 1H), 1.45–1.40 (m, 1H); ^**13**^ **C­{** ^**1**^ **H}-NMR** (100 MHz, DMSO) δ [ppm] = 154.6, 152.2, 151.1, 138.9, 136.3, 119.6, 119.1, 83.6, 69.7, 69.3, 59.5, 57.2, 53.7, 38.3, 31.8, 25.0; **HRMS** (ESI, 3.5 kV) *m*/*z*: [M + H]^+^ calc for C_17_H_23_N_5_O_3_ 346.1874, found 346.1892; **LCMS** *m*/*z* 346.2 (M + H) (ESI +ve), RT = 1.46 min; **HPLC** RT = 3.94 min, 100%.

##### Synthesis of (1R,2S,3R,7R,7aR)-3-(6-(Dimethylamino)-9*H*-purin-9-yl)-2-ethoxy-2,3,5,6,7,7a-hexahydro-1*H*-indene-1,7-diol (**6k**)

To a stirred solution of **50** (0.40 g, 0.70 mmol, 1.0 equiv) in anhydrous DMF (8.0 mL) were added sodium hydride (0.041 g, 1.05 mmol, 1.5 equiv) and ethyl iodide (0.54 g, 3.48 mmol, 5.0 equiv) at 0 °C. The resulting reaction mixture was stirred at 0 °C for 1 h. After completion of the reaction, the reaction mixture was slowly quenched with ice-cold water (100 mL) and extracted with ethyl acetate (3 × 50 mL). The combined organic phase was dried over anhydrous Na_2_SO_4_, filtered, and concentrated under reduced pressure to afford the crude as a brown sticky solid (0.40g). The crude was redissolved in a mixture of 1,4-dioxane (2.0 mL) and water (2.0 mL) was added 4 M HCl in dioxane (2.0 mL) at room temperature. The resulting reaction mixture was stirred at room temperature for 3 h. After completion of the reaction, the reaction mixture was concentrated under reduced pressure to get the crude, which was purified by reversed-phase chromatography using (53% MeCN/water). The obtained pure fractions were lyophilized to afford the title compound **6k** as a white solid (0.06 g, 0.17 mmol, 22%). **R**_**f**_ = 0.5 (10% MeOH/DCM); ^**1**^ **H NMR** (400 MHz, DMSO) δ [ppm] = 8.21 (s, 1H), 8.19 (s, 1H), 5.36 (d, *J* = 8.8 Hz, 1H), 4.81 (d, *J* = 5.2 Hz, 1H), 4.72 (d, *J* = 4.8 Hz, 1H), 4.66 (dd, *J* = 2.4, 2.4 Hz, 1H), 4.17 (ddd, *J* = 3.5, 5.3, 5.5 Hz, 1H), 4.07 (dd, *J* = 5.6, 8.8 Hz, 1H), 3.60–3.45 (m, 9H), 2.33–2.31 (m, 1H), 2.03–2.00 (m, 2H), 1.83–1.80 (m, 1H), 1.48–1.35 (m, 1H), 1.01 (t, *J* = 6.8 Hz, 3H); ^**13**^ **C­{** ^**1**^ **H}-NMR** (100 MHz, DMSO) δ [ppm] = 154.6, 152.2, 151.1, 138.9, 136.4, 119.6, 119.0, 81.8, 69.7, 64.8, 59.7, 53.7, 38.4, 31.8, 25.0, 15.6; **HRMS** (ESI, 3.5 kV) *m*/*z*: [M + H]^+^ calc for C_18_H_25_N_5_O_3_H^+^ 360.2030, found 360.2042; **LCMS** *m*/*z* 360.2 (M + H)^+^ (ESI +ve), RT = 1.49 min; **HPLC** RT = 4.33 min, 95.56%. *Note: One C-signal could not be observed in* ^13^ *C NMR, possibly due to peak broadening or overlapping.*

#### Synthesis of 3′-Pyrimidine Analogues

##### Synthesis of 1-((1R,2S,3R,3aS,4R)-2,4-Bis­((*tert*-butyldimethylsilyl)­oxy)-3-hydroxy-2,3,3a,4,5,6-hexahydro-1*H*-inden-1-yl)­pyrimidine-2,4­(1*H*,3*H*)-dione (**51**)

To a stirred solution of **2a** (0.9 g, 3.21 mmol, 1.0 equiv) in pyridine (9.0 mL, 10.0 vol) was added TBSCl (2.17 g, 14.4 mmol, 4.5 equiv) at room temperature. The resulting reaction mixture was stirred at room temperature for 72 h. After completion of the reaction, the reaction mixture was concentrated under reduced pressure to get the crude, which was purified by reversed-phase chromatography using (90% MeCN/water). The obtained pure fractions were lyophilized to afford the title compound **51** as a white solid (0.66 g, 1.28 mmol, 40%). **R**_**f**_ = 0.7 (10% MeOH/DCM); ^**1**^ **H NMR** (400 MHz, DMSO) δ [ppm] = 11.30 (s, 1H), 7.59 (d, *J* = 8.0 Hz, 1H), 5.62 (d, *J* = 8.0 Hz, 1H), 5.38 (brs, 1H), 5.05 (s, 1H), 4.29 (d, *J* = 4.4 Hz, 1H), 3.99 (brs, 1H), 3.81–3.78 (m, 1H), 3.69–3.63 (m, 1H), 2.29 (d, *J* = 8.0 Hz, 1H), 2.15 (brs, 2H), 1.83–1.79 (m, 1H), 1.54–1.44 (m, 1H), 0.90 (s, 9H), 0.80 (s, 9H), 0.09 (d, *J* = 4.4 Hz, 6H), 0.02 (s, 3H), −0.04 (s, 3H); ^**13**^ **C­{** ^**1**^ **H}-NMR** (100 MHz, DMSO) δ [ppm] = 163.5, 152.1, 143.2, 134.6, 118.9, 101.7, 75.5, 71.0, 61.6, 54.0, 32.3, 26.4, 26.2, 26.1, 25.0, 18.3, 18.2, −3.8, −4.2, −4.2, −4.6; **HRMS** (ESI, 3.5 kV) *m*/*z*: [M + H]^+^ calc for C_25_H_44_N_2_O_5_Si_2_ 509.2862, found 509.2854; **LCMS** *m*/*z* 509.3 (M + H) (ESI +ve), RT = 2.97 min; **HPLC** RT = 11.08 min. 97.73%.

##### Synthesis of 1-((1R,2S,3S,3aR,4R)-3-Fluoro-2,4-dihydroxy-2,3,3a,4,5,6-hexahydro-1*H*-inden-1-yl)­pyrimidine-2,4­(1*H*,3*H*)-dione (**2g**)

To a stirred solution of **51** (0.40 g, 0.78 mmol, 1.0 equiv) in dichloromethane (20.0 mL) was added DAST (0.15 g, 0.94 mmol, 1.2 equiv) at −10 °C. The resulting reaction mixture was stirred at −10 °C to room temperature for 2 h. After completion of the reaction, the reaction mixture was diluted with water (70 mL) and extracted with dichloromethane (2 × 70 mL). The combined organic phase was dried over anhydrous Na_2_SO_4_, filtered, and concentrated under reduced pressure to afford crude as a brown solid (0.42 g). The crude was dissolved in a mixture of THF (4.2 mL), water (4.2 mL), and TFA (0.42 mL). The resulting reaction mixture was stirred at room temperature for 16 h. After completion of the reaction, the reaction mixture was concentrated under reduced pressure to get the crude, which was purified by RP Prep HPLC. The obtained pure fractions were lyophilized to afford the title compound **2g** as a white solid (0.044 g, 0.15 mmol, 20%). **R**_**f**_ = 0.1 (5% MeOH/DCM); ^**1**^ **H NMR** (400 MHz, DMSO) δ [ppm] = 11.36 (s, 1H), 7.03 (d, *J* = 8.0 Hz, 1H), 5.84 (d, *J* = 4.4 Hz, 1H), 5.64 (dd, *J* = 2.0, 8.0 Hz, 1H), 5.39 (s, 1H), 5.24 (s, 1H), 5.01 (m, 1H), 4.91 (dd, *J* = 3.6, 52.0 Hz, 1H), 4.03 (d, *J* = 17.2 Hz, 1H), 3.67 (dd, *J* = 8.8, 8.8 Hz, 1H), 2.67–2.57 (m, 1H), 2.12 (brs, 2H), 1.88–1.86 (m, 1H), 1.51–1.41 (m, 1H); ^**13**^ **C­{** ^**1**^ **H}-NMR** (100 MHz, DMSO) δ [ppm] = 163.6, 151.8, 142.7, 137.5, 123.1, 102.7, 97.4, 95.6, 79.4 (d, *J* = 28.6 Hz), 64.1, 50.3 (d, *J* = 19.6 Hz), 31.5, 25.1; **HRMS** (ESI, 3.5 kV) *m*/*z*: [M + H]^+^ calc for C_13_H_15_FN_2_O_4_H^+^ 283.1089, found 283.1087; **LCMS** *m*/*z* 283.1 (M + H) (ESI +ve), RT = 0.94 min; **HPLC** RT = 3.69 min. 99.22%.

##### Synthesis of 1-((1R,2S,3R,3aR,4R)-2,4-Dihydroxy-3-methoxy-2,3,3a,4,5,6-hexahydro-1*H*-inden-1-yl)-3-methylpyrimidine-2,4­(1*H*,3*H*)-dione (**2i**)

To a stirred solution of **51** (0.25 g, 0.49 mmol, 1.0 equiv) in anhydrous DMF (2.5 mL) was added sodium hydride (60% in mineral oil, 0.064 g, 1.47 mmol, 3.0 equiv), followed by dropwise addition of methyl iodide (0.14 g, 0.98 mmol, 2.0 equiv) at 0 °C. The resulting reaction mixture was stirred at room temperature for 16 h. After completion of the reaction, the reaction mixture was slowly quenched with ice-cold water (50 mL) and extracted with ethyl acetate (2 × 50 mL). The combined organic phase was dried over anhydrous Na_2_SO_4_, filtered, and concentrated under reduced pressure to afford the crude as a white solid (0.25 g). The crude was redissolved in a solution of THF (2.5 mL), water (2.5 mL), and TFA (2.5 mL, 10 vol) at room temperature. The resulting reaction mixture was stirred at room temperature for 16 h. After completion of the reaction, the reaction mixture was concentrated, neutralized using NH_4_OH (pH ∼ 8.0) and again concentrated to get the crude, which was purified by RP Prep HPLC. The obtained pure fractions were lyophilized to afford the title compound **2i** as a white solid (0.041 g, 0.13 mmol, 27%). **R**_**f**_ = 0.2 (10% MeOH/DCM, 0.1% NH_4_OH); ^**1**^ **H NMR** (400 MHz, DMSO) δ [ppm] = 7.54 (d, *J* = 6.8 Hz, 1H), 5.74 (d, *J* = 8.0 Hz, 1H), 5.35 (brs, 1H), 5.07 (d, *J* = 2.4 Hz, 1H), 4.73 (brs, 2H), 4.07 (s, 1H), 3.59 (brs, 1H), 3.40–3.35 (m, 1H), 3.32 (s, 3H), 3.28 (s, 3H), 2.22–2.20 (m, 1H), 2.09–2.06 (m, 2H), 1.81–1.78 (m, 1H), 1.45–1.38 (m, 1H); ^**13**^ **C­{** ^**1**^ **H}-NMR** (100 MHz, DMSO) δ [ppm] = 162.5, 152.2, 141.7, 135.1, 119.2, 101.0, 82.7, 69.5, 69.2, 61.2, 57.2, 53.3, 31.8, 28.0, 25.0; **HRMS** (ESI, 3.5 kV) *m*/*z*: [M + H]^+^ calc. for C_15_H_20_N_2_O_5_ 309.1445, found 309.1444; **LCMS** *m*/*z* 309.1 (M + H) (ESI +ve), RT = 1.45 min; **HPLC** RT = 4.11 min, 100%.

##### Synthesis of 4-Amino-1-((1R,2S,3R,3aS,4R)-2,4-bis­((*tert*-butyldimethylsilyl)­oxy)-3-hydroxy-2,3,3a,4,5,6-hexahydro-1*H*-inden-1-yl)­pyrimidin-2­(1*H*)-one (**52**)

To a stirred solution of **51** (1.0 g, 1.96 mmol, 1.0 equiv) in acetonitrile (10 mL, 10 vol) were added DMAP (0.024 g, 0.19 mmol, 0.1 equiv), TEA (0.59 g, 5.89 mmol, 3.0 equiv), and 2,4,6-triisopropylbenzenesulfonyl chloride (0.89 g, 2.94 mmol, 1.5 equiv) at room temperature. The resulting reaction mixture was stirred at room temperature for 3 h. After completion of the reaction, the reaction mixture was charged with NH_4_OH (10 mL, 10 vol) at room temperature. The resulting reaction mixture was stirred at room temperature for 16 h. After completion of the reaction, the reaction mixture was concentrated under reduced pressure to get the crude, which was purified by reversed-phase chromatography (85% MeCN/water). The pure fractions obtained were lyophilized to afford the title compound **52** as a white solid (0.28 g, 0.55 mmol, 28%). **R**_**f**_ = 0.4 (10% MeOH/DCM); ^**1**^ **H NMR** (400 MHz, DMSO) δ [ppm] = 7.47–7.42 (m, 1H), 7.04–6.98 (m, 2H), 5.68 (d, *J* = 7.2 Hz, 1H), 5.49 (brs, 1H), 4.88 (s, 1H), 4.13 (brs, 1H), 3.98–3.93 (m, 1H), 3.80 (brs, 1H), 3.65–3.60 (m, 1H), 2.27 (brs, 1H), 2.12 (brs, 2H), 1.81–1.78 (m, 1H), 1.54–1.43 (m, 1H), 0.89 (s, 9H), 0.79 (s, 9H), 0.09 (s, 3H), 0.08 (s, 3H), 0.00 (s, 3H), −0.06 (m, 3H); **LCMS** *m*/*z* = 508.4 (M + H) (ESI +ve), RT = 2.74 min.

##### Synthesis of 4-Amino-1-((1R,2S,3S,3aR,4R)-3-fluoro-2,4-dihydroxy-2,3,3a,4,5,6-hexahydro-1*H*-inden-1-yl)­pyrimidin-2­(1*H*)-one (**4g**)

To a stirred solution of **52** (0.28 g, 0.55 mmol, 1.0 equiv) in dichloromethane (5.6 mL) was added DAST (0.11 g, 0.66 mmol, 1.2 equiv) at −10 °C. The resulting reaction mixture was stirred at −10 °C to room temperature for 2 h. After completion of the reaction, the reaction mixture was diluted with water (70 mL) and extracted with dichloromethane (3 × 90 mL). The combined organic phase was dried over anhydrous Na_2_SO_4_, filtered, and concentrated under reduced pressure to afford crude as a brown solid (0.28 g). To a stirred solution of the crude in a mixture of THF (2.8 mL) and water (2.8 mL) was added TFA (0.8 mL) at room temperature. The resulting reaction mixture was then stirred at room temperature for 16 h. After completion of the reaction, the reaction mixture was concentrated under reduced pressure to get the crude, which was purified by RP Prep HPLC. The pure fractions obtained were lyophilized to afford the title compound **4g** as a white solid (5.0 mg, 0.01 mmol, 3% over 2 steps). **R**_**f**_ = 0.3 (15% MeOH/DCM); ^**1**^ **H NMR** (400 MHz, DMSO) δ [ppm] = 7.14 (brs, 1H), 7.05 (d, *J* = 7.6 Hz, 2H), 5.77 (brs, 1H), 5.71 (d, *J* = 7.2 Hz, 1H), 5.40 (s, 1H), 5.20 (s, 1H) 4.97 (brs, 1H), 4.89 (dd, *J* = 3.6, 52.0 Hz, 1H), 3.94 (d, *J* = 16.4 Hz, 1H), 3.66 (dd, *J* = 8.2, 8.4 Hz, 1H), 2.67–2.56 (m, 1H), 2.10 (brs, 2H), 1.86 (d, *J* = 12.0 Hz, 1H), 1.48–1.44 (m, 1H); **HRMS** (ESI, 3.5 kV) *m*/*z*: [M + H]^+^ calc for C_13_H_16_FN_3_O_3_ 282.1248, found 282.1244; **LCMS** *m*/*z* 282.0 (M + H) (ESI +ve), RT = 1.86 min; **HPLC** RT 5.07 min, 97.19%.

#### Synthesis of 2′-Pyrimidine Analogues

##### Synthesis of 1-((5aR,5a1R,6S,7R,10aR)-6-Hydroxy-2,2,4,4-tetraisopropyl-5a,5a1,7,9,10,10a-hexahydro-6*H*-indeno­[1,7-fg]­[1,3,5,2,4]­trioxadisilocin-7-yl)­pyrimidine-2,4­(1*H*,3*H*)-dione (**86**)

To a stirred solution **2a** (0.60 g, 2.14 mmol, 1.0 equiv) in pyridine (6.0 mL) was added 1,3-dichloro-1,1,3,3-tetraisopropyl disiloxane (2.02 g, 6.42 mmol, 3.0 equiv) at 0 °C. The resulting reaction mixture was stirred at room temperature for 2 h. After completion of the reaction, the reaction mixture was concentrated under reduced pressure to get the crude. Another reaction of the same batch size was performed and was combined during workup. The crude was purified by reversed-phase chromatography using (100% MeCN). The obtained pure fractions were lyophilized to afford the title compound **86** as a white solid (0.84 g, 1.60 mmol, 37%). **R**_**f**_ = 0.4 (50% EA/hex); ^**1**^ **H NMR** (400 MHz, DMSO) δ [ppm] = 11.30 (s, 1H), 7.57 (d, *J* = 4.0 Hz, 1H), 5.59 (d, *J* = 7.6 Hz, 1H), 5.20 (s, 1H), 4.63 (brs, 1H), 4.27 (brs, 1H), 3.98 (brs, 1H), 3.85 (brs, 1H), 3.49 (brs, 1H), 2.40 (brs, 1H), 2.12 (brs, 2H), 1.86–1.83 (m, 1H), 1.57–1.52 (m, 1H), 1.23–1.02 (m, 28H); ^**13**^ **C­{** ^**1**^ **H}-NMR** (400 MHz, DMSO) δ [ppm] = 163.7, 151.4, 134.8, 120.3, 101.8, 74.7, 73.1, 52.3, 50.6, 32.0, 25.0, 17.9, 17.7, 17.6, 17.5, 17.5, 13.4, 13.2, 12.9; **LCMS** *m*/*z* 523.3 (M + H) (ESI +ve), RT = 2.24 min; **HPLC** RT = 12.33 min, 100%.

##### Synthesis of 1-((5aS,5a1R,7S,10aR)-2,2,4,4-Tetraisopropyl-5a,5a1,7,9,10,10a-hexahydro-6*H*-indeno­[1,7-fg] [1,3,5,2,4]­trioxadisilocin-7-yl)­pyrimidine-2,4­(1*H*,3*H*)-dione (**53**)

To a stirred solution of **86** (0.53 g, 1.01 mmol, 1.0 equiv) in acetonitrile (2.65 mL) was added DMAP (0.61 g, 5.06 mmol, 5.0 equiv), followed by dropwise addition of O-phenyl carbonochloridothioate (0.34 g, 2.03 mmol, 2.0 equiv) at room temperature. The resulting reaction mixture was stirred at room temperature for 1 h. After completion of the reaction, the reaction mixture was concentrated under reduced pressure to get the crude, which was purified by normal-phase chromatography using (39% EA/Hex). The obtained pure fractions were concentrated under reduced pressure to afford the intermediate as a light-yellow sticky solid (0.40 g, 0.61 mmol). The intermediate was redissolved in toluene (4.8 mL) and was purged with N_2_ gas for 15 min. To this reaction mixture, tributyltin hydride (0.21 g, 0.72 mmol, 1.18 equiv) and AIBN (0.02 g, 0.12 mmol, 0.20 equiv) were added at room temperature under N_2_ atmosphere. The resulting reaction mixture was heated to 110 °C for 16 h. After completion of the reaction, the reaction mixture was concentrated under reduced pressure to get the crude, which was purified by normal-phase chromatography using (35% EA/Hex). The obtained pure fractions were concentrated under reduced pressure to afford the title compound **53** as a light-yellow sticky liquid (0.27 g, 0.53 mmol, 53%). **Rf** = 0.4 (40% EA/hex); **LCMS** *m*/*z* = 507.3 (M + H) (ESI +ve); RT = 3.51 min.

##### Synthesis of 1-((1S,3S,3aR,4R)-3,4-Dihydroxy-2,3,3a,4,5,6-hexahydro-1*H*-inden-1-yl)­pyrimidine-2,4­(1*H*,3*H*)-dione (**2f**)

To a stirred solution of **53** (0.25 g, 0.49 mmol, 1.0 equiv) in a mixture of 1,4-dioxane (2.5 mL) and water (2.5 mL) was added 4 M HCl in dioxane (2.5 mL) at room temperature. The resulting reaction mixture was stirred at room temperature for 16 h. After completion of the reaction, the reaction mixture was concentrated under reduced pressure to get the crude, which was purified by RP Prep HPLC. The obtained pure fractions were lyophilized to afford the title compound **2f** as a white solid (0.042 g, 0.15 mmol, 36%). **Rf** = 0.1 (10% MeOH/DCM); ^**1**^ **H NMR** (400 MHz, DMSO) δ [ppm] = 11.25 (brs, 1H), 7.38 (d, *J* = 8.0 Hz, 1H), 5.56 (d, *J* = 8.0 Hz, 1H), 5.41 (s, 1H), 5.08 (s, 1H), 4.82 (d, *J* = 4.0 Hz, 1H), 4.63 (d, *J* = 4.4 Hz, 1H), 4.11–4.08 (m, 1H), 3.43–3.41 (m, 1H), 2.09 (brs, 3H) 1.91 (dd, *J* = 6.4, 6.4 Hz, 2H), 1.77 (d, *J* = 12.4 Hz, 1H), 1.41–1.35 (m, 1H); ^**13**^ **C­{** ^**1**^ **H}-NMR** (100 MHz, DMSO) δ [ppm] = 163.6, 151.6, 143.5, 139.9, 119.2, 102.0, 73.1, 70.6, 55.1, 54.8, 31.4, 24.9; **HRMS** (ESI, 3.5 kV) *m*/*z*: [M + H]^+^ calc for C_13_H_16_N_2_O_4_H^+^ 265.1183, found 265.1181; **LCMS** *m*/*z* 265.2 (M + H) (ESI +ve), RT = 0.93 min; **HPLC** RT = 5.10 min, 100%. *Note: One C signal could not be observed in* ^13^ *C NMR possibly due to peak broadening or overlapping.*

##### Synthesis of 4-(Hydroxyimino)-1-((5aS,5a1R,7S,10aR)-2,2,4,4-tetraisopropyl-5a,5a1,7,9,10,10a -hexahydro-6*H*-indeno­[1,7-fg]­[1,3,5,2,4]­trioxadisilocin-7-yl)-3,4-dihydropyrimidin-2­(1*H*)-one (**56**)

To a stirred solution of **53** (0.25 g, 0.49 mmol, 1.0 equiv) in HMDS (5.0 mL) were added imidazole (0.016 g, 0.24 mmol, 0.5 equiv), ammonium bisulfate (0.22 g, 1.97 mmol, 4.0 equiv), and hydroxylamine sulfate (0.16 g, 0.98 mmol, 2.0 equiv) at room temperature. The resulting reaction mixture was heated to 85 °C for 16 h. After completion of the reaction, the reaction mixture was diluted with water (30 mL) and extracted with ethyl acetate (3 × 30 mL). The combined organic phase was washed with brine (20 mL), dried over anhydrous Na_2_SO_4_, filtered, and concentrated under reduced pressure to get the crude, which was purified by normal-phase chromatography using (6% MeOH/DCM). The pure fractions obtained were concentrated under reduced pressure to afford the title compound **56** as a white solid (0.19 g, 0.36 mmol, 73% Yield). **R**_**f**_ = 0.45 (5% MeOH/DCM); **LCMS** *m*/*z* 522.7 (M + H) (ESI +ve), RT = 3.49 min.

##### Synthesis of 1-((1S,3S,3aR,4R)-3,4-Dihydroxy-2,3,3a,4,5,6-hexahydro-1*H*-inden-1-yl)-4-(hydroxy imino)-3,4-dihydropyrimidin-2­(1*H*)-one (**3f**)

To a stirred solution of **56** (0.18 g, 0.34 mmol, 1.0 equiv) in methanol (1.8 mL) was added TFA (0.9 mL) at 0 °C. The resulting reaction mixture was stirred at room temperature for 16 h. After completion of the reaction, the reaction mixture was concentrated under reduced pressure to get the crude, which was purified by RP Prep HPLC. The obtained pure fractions were lyophilized to afford the title compound **3f** as a white solid (0.017 g, 0.060 mmol, 18%). **R**_**f**_ = 0.25 (15% MeOH/DCM); ^**1**^ **H NMR** (400 MHz, DMSO) δ [ppm] = 9.88 (s, 1H), 9.35 (s, 1H), 6.55 (d, *J* = 8.4 Hz, 1H), 5.49 (dd, *J* = 2.0, 8.0 Hz, 1H), 5.34 (brs, 1H), 5.10 (s, 1H), 4.79 (d, *J* = 4.4 Hz, 1H), 4.61 (d, *J* = 4.8 Hz, 1H), 4.05 (dd, *J* = 5.6, 5.6 Hz, 1H), 3.44–3.38 (m, 1H), 2.13–2.03 (m, 3H), 1.84 (dd, *J* = 6.8, 6.8 Hz, 2H), 1.78–1.75 (m, 1H), 1.44–1.38 (m, 1H); **HRMS** (ESI, 3.5 kV) *m*/*z*: [M + H]^+^ calc for C_13_H_17_N_3_O_4_H^+^ 280.1292, found 280.1297; **LCMS** *m*/*z* 280.0 (M + H) (ESI +ve), RT = 1.92 min; **HPLC** RT = 5.03 min, 99.39%.

##### Synthesis of 4-Amino-1-((5aS,5a1R,7S,10aR)-2,2,4,4-tetraisopropyl-5a,5a1,7,9,10,10a-hexahydro-6*H*-indeno­[1,7-fg]­[1,3,5,2,4]­trioxadisilocin-7-yl)­pyrimidin-2­(1*H*)-one (**54**)

To a stirred solution of **53** (0.32 g, 0.63 mmol, 1.0 equiv) in acetonitrile (3.2 mL) were added DMAP (0.007 g, 0.063 mmol, 0.1 equiv), TEA (0.17 mL, 1.26 mmol, 2.0 equiv), and 2,4,6-triisopropylbenzenesulfonyl chloride (0.38 g, 1.26 mmol, 2.0 equiv) at room temperature. The resulting reaction mixture was stirred at room temperature for 2 h. After completion of the reaction, the reaction mixture was charged with NH_4_OH (3.2 mL) at room temperature. The resulting reaction mixture was stirred at room temperature for 16 h. After completion of the reaction, the reaction mixture was concentrated under reduced pressure to get the crude, which was purified by column chromatography using silica gel (7% MeOH in DCM) to afford the title compound **54** as a light-yellow oil (0.16 g, 0.31 mmol, 50% Yield). **R**_**f**_ = 0.2 (5% MeOH/DCM); **LCMS** *m*/*z* 506.3 (M + H) (ESI +ve), RT = 2.06 min.

##### Synthesis of 4-Amino-1-((1S,3S,3aR,4R)-3,4-dihydroxy-2,3,3a,4,5,6-hexahydro-1*H*-inden-1-yl)­pyrimidine-2­(1*H*)-one (**4f**)

To a stirred solution of **54** (0.16 g, 0.31 mmol, 1.0 equiv) in a mixture of 1,4-dioxane (1.6 mL) and water (1.6 mL) was added 4 M HCl in dioxane (1.6 mL) at room temperature. The resulting reaction mixture was stirred at room temperature for 16 h. After completion of the reaction, the reaction mixture was concentrated under reduced pressure to get the crude, which was purified by RP Prep HPLC. The pure fractions obtained were lyophilized to afford the title compound **4f** as a white solid (0.040 g, 0.15 mmol, 48% yield). **R**_**f**_ = 0.1 (10% MeOH/DCM); ^**1**^ **H NMR** (400 MHz, DMSO) δ [ppm] = 7.31 (d, *J* = 7.2 Hz, 1H), 7.06–7.00 (m, 2H), 5.67 (d, *J* = 7.2 Hz, 1H), 5.52 (brs, 1H), 4.92 (s, 1H), 4.79 (brs, 1H), 4.61 (brs, 1H), 4.08 (d, *J* = 6.0 Hz, 1H), 3.41 (dd, *J* = 8.4, 8.4 Hz, 1H), 2.07–2.04 (m, 3H), 1.91–1.75 (m, 3H), 1.43–1.41 (m, 1H); ^**13**^ **C­{** ^**1**^ **H}-NMR** (400 MHz, DMSO) δ [ppm] = 165.6, 156.4, 143.8, 141.1, 118.5, 94.4, 73.3, 70.8, 55.3, 55.1, 31.5, 24.9; **HRMS** (ESI, 3.5 kV) *m*/*z*: [M + H]^+^ calc for C_13_H_17_N_3_O_3_H^+^ 264.1343, found 264.1343; **LCMS** *m*/*z* 264.0 (M + H) (ESI +ve), RT = 1.81 min; **HPLC** RT = 4.72 min, 100%. *Note: One C signal could not be observed in 13C-NMR possibly due to peak broadening or overlapping.*

##### Synthesis of 4-(Methylamino)-1-((5aS,5a1R,7S,10aR)-2,2,4,4-tetraisopropyl-5a,5a1,7,9,10,10a-hexahydro-6*H*-indeno­[1,7-fg]­[1,3,5,2,4]­trioxadisilocin-7-yl)­pyrimidin-2­(1*H*)-one (**55**)

To a stirred solution of **53** (0.28 g, 0.55 mmol, 1.0 equiv) in acetonitrile (2.8 mL) were added DMAP (0.006 g, 0.05 mmol, 0.1 equiv), TEA (0.38 mL, 2.76 mmol, 5.0 equiv), and 2,4,6-triisopropylbenzenesulfonyl chloride (0.33 g, 1.10 mmol, 2.0 equiv) at room temperature. The resulting reaction mixture was stirred at room temperature for 2 h. After completion of the reaction, the reaction mixture was charged with methylamine (2 M in THF, 2.8 mL) at room temperature. The resulting reaction mixture was stirred at room temperature for 16 h. After completion of the reaction, the reaction mixture was diluted with water (60 mL) and extracted with ethyl acetate (3 × 25 mL). The combined organic phase was dried over anhydrous Na_2_SO_4_, filtered, and concentrated under reduced pressure to afford the title compound **55** as a brown solid (0.16 g, 0.30 mmol, 56%). **R**_**f**_ = 0.4 (10% MeOH/DCM); **LCMS** *m*/*z* 1039.6 (2M+H) (ESI +ve), RT = 3.10 min. The obtained crude was directly used in the next step without purification.

##### Synthesis of 1-((1S,3S,3aR,4R)-3,4-Dihydroxy-2,3,3a,4,5,6-hexahydro-1*H*-inden-1-yl)-4-(methylamino) pyrimidin-2­(1*H*)-one (**18f**)

To a stirred solution of **55** (0.16 g, 0.30 mmol, 1.0 equiv) in 1,4-dioxane (1.6 mL) and water (1.6 mL) was added 4 M HCl in dioxane (1.6 mL) at room temperature. The resulting reaction mixture was stirred at room temperature for 16 h. After completion of the reaction, the reaction mixture was concentrated under reduced pressure to get the crude, which was purified by RP Prep HPLC. The obtained pure fractions were lyophilized to afford the title compound **18f** as a white solid (0.040 g, 0.14 mmol, 47%). **R**_**f**_ = 0.1 (10% MeOH/DCM); ^**1**^ **H NMR** (400 MHz, DMSO) δ [ppm] = 7.56 (d, *J* = 4.8 Hz, 1H), 7.24 (d, *J* = 7.2 Hz, 1H), 5.68 (d, *J* = 7.6 Hz, 1H), 5.52 (brs, 1H), 4.92 (s, 1H), 4.78 (d, *J* = 4.4 Hz, 1H), 4.61 (d, *J* = 4.8 Hz, 1H), 4.08 (t, *J* = 5.2 Hz, 1H) 3.43–3.37 (m, 1H), 2.74 (d, *J* = 4.8 Hz, 3H), 2.10–2.03 (m, 3H), 1.91–1.75 (m, 3H), 1.43–1.35 (m, 1H); ^**13**^ **C­{** ^**1**^ **H}-NMR** (400 MHz, DMSO) δ [ppm] = 168.1, 163.8, 156.4, 142.4, 141.1, 118.5, 95.0, 73.2, 70.8, 55.3, 31.5, 27.2, 24.9, 18.7; **HRMS** (ESI, 3.5 kV) *m*/*z*: [M + H]^+^ calc for C_14_H_19_N_3_O_3_ 278.1499, found 278.1494; **LCMS** *m*/*z* 278.0 (M + H) (ESI +ve), RT = 1.84 min; **HPLC** RT= 4.95 min, 99.59%.

## Supplementary Material





## Notes

Additional figures and full screening results; analytical details such as NMR spectra and HPLC traces (PDF)
Molecular formula strings (CSV)

## Abbreviations Used

5-FU: 5-Fluorouracil
AF3: AlphaFold 3
AIBN: 2,2’-Azobis­(2-methylpropionitrile)
ATP: Adenosine Triphosphate
BHT: Butylated Hydroxytoluene
Bz: Benzoyl
CAN: Ceric Ammonium Nitrate
CC_50_: Cytotoxic Concentration 50%
CPE: Cytopathic Effect
cryo-EM: Cryo-electron Microscopy
DAST: (Diethylamino)sulfur Trifluoride
DCE: 1,2-Dichloroethane
DCM: Dichloromethane
DIAD: Diisopropyl Azodicarboxylate
DMAP: 4-Dimethylaminopyridine
DMF: *N*,*N*-Dimethylformamide
DMSO: Dimethyl Sulfoxide
DNA: Deoxyribonucleic Acid
EA: Ethyl Acetate
EC_50_: Half Maximal Effective Concentration
HBV: Hepatitis B Virus
HCV: Hepatitis C Virus
HCoV: Human Coronavirus
HEK: Human Embryonic Kidney (cells)
HEL: Human Embryonic Lung (cells)
HeLa: Henrietta Lacks (cervical adenocarcinoma cells)
HEp-2: Human Epithelial type 2 (cells)
Hex: Hexane
HIV: Human Immunodeficiency Virus
HMDS: 1,1,1,3,3,3-Hexamethyldisilazane
hpi: Hours Post-Infection
HPIV: Human Parainfluenza Virus
HRMS: High-Resolution Mass Spectrometry
HSV: Herpes Simplex Virus
IAV: Influenza A Virus
IAVpol: Influenza A Virus Polymerase
IBV: Influenza B Virus
IC_50_: Half Maximal Inhibitory Concentration
LASV: Lassa Virus
LC-MS: Liquid Chromatography Mass Spectrometry
LLC-MK2: Rhesus Monkey Kidney (cells)
MDCK: Madin-Darby Canine Kidney (cells)
MOI: Multiplicity of Infection
MP: Monophosphate
NA: Nucleoside Analogue
NBS: *N*-Bromosuccinimide
NCS: *N*-Chlorosuccinimide
NHC: *N*-hydroxy cytidine
NMP: *N*-Methyl-2-pyrrolidone
NMR: Nuclear Magnetic Resonance
NOE: Nuclear Overhauser Effect
NP: Nucleoprotein
NTP: Nucleoside Triphosphate
PA: Polymerase Acidic (subunit of influenza polymerase)
PB1: Polymerase Basic 1 (subunit of influenza polymerase)
PB2: Polymerase Basic 2 (subunit of influenza polymerase)
PGG: Pentagalloylglucose
pyr: Pyridine
qPCR: Quantitative Polymerase Chain Reaction
RD: Rhabdomyosarcoma (cells)
RdRp: RNA-dependent RNA Polymerase
RNA: Ribonucleic Acid
RSV: Respiratory Syncytial Virus
SAR: Structure–Activity Relationship
SARS-CoV-2: Severe Acute Respiratory Syndrome Coronavirus 2
TBS: *tert*-Butyldimethylsilyl
TCID_50_: 50% Tissue Culture Infective Dose
TFA: Trifluoroacetic Acid
THF: Tetrahydrofuran
TIPS: Triisopropylsilyl
TP: Triphosphate
Trt: Trityl
UTP: Uridine Triphosphate
UTR: Untranslated Region
