# Supplementary material for: Antiviral Profiling and Cellular Activation of Carbobicyclic Nucleoside Analogues
Source: J Med Chem. 2026 Feb 20;69(5):5501–39. doi: 10.1021/acs.jmedchem.5c02584 (PMC12990120; doi:10.1021/acs.jmedchem.5c02584)

Supporting Information for

Antiviral Profiling and Cellular Activation of Carbobicyclic Nucleoside Analogues

Stephan Scheeff‡,[a], Joan Marie Javillo Baguio‡,[b,c], Benny Zhibin Liang,‡,[d,e] Josefina Xeque Amada[a], Kin Pong Tao[b,c], Steven De Jonghe[f], Leentje Persoons[g], Tiffany Hoi-Yee Chow[a], Carmen Ka Man Tse[d,e], Roy Yukang Wu[a], Xinzhou Xu[d,e], Zhong Zuo[a], Peter Pak-Hang Cheung[d,e], Renee Wan Yi Chan[b-d*], Billy Wai-Lung Ng[a,c,e,h*]

[a] School of Pharmacy, Faculty of Medicine, The Chinese University of Hong Kong, Hong Kong
[b] Department of Paediatrics, Faculty of Medicine; S.H. Ho Research Centre for Infectious Diseases, The Chinese University of Hong Kong, Hong Kong
[c] CUHK–Hub of Obstetric and Paediatric Excellence; The Chinese University of Hong Kong, Hong Kong
[d] Department of Chemical Pathology, Faculty of Medicine, The Chinese University of Hong Kong, Hong Kong
[e] Li Ka Shing Institute of Health Sciences, Faculty of Medicine, The Chinese University of Hong Kong, Hong Kong
[f] KU Leuven, Department of Microbiology, Immunology and Transplantation, Rega Institute for Medical Research, Molecular, Structural and Translational Virology Research Group, Herestraat 49, box 1049, 3000 Leuven, Belgium
[g] KU Leuven, Department of Microbiology, Immunology and Transplantation, Rega Institute for Medical Research, Molecular Genetics and Therapeutics in Virology and Oncology Research Group, Herestraat 49, box 1048, 3000 Leuven, Belgium
[h] Gerald Choa Neuroscience Institute, The Chinese University of Hong Kong, Hong Kong

*E-mail address: reneewy@cuhk.edu.hk; billyng@cuhk.edu.hk ‡These authors contributed equally.

Contents

Additional Materials....3
Nucleoside Analogues as Drugs....3
Synthesized Analogues in this Work....4
Full Synthetic Schemes....7
Additional Antiviral Screening....15
Synthesis of Monophosphate, Triphosphate and Protide Analogue....21
Additional Parameters for Metabolism Study....23
AlphaFold3 H1N1 IAV RNA-dependent RNA polymerase model....25
RNA Polymerase Inhibition Assay....33
DNA Polymerase Inhibition Assay....35
Additional Details for Cellular Assays....36
Additional Details for Chemical Synthesis....38
Parameters for HPLC separation....38
References....56
Copies of Analytical Spectra (HPLC, NMR)....57

Additional Materials

Nucleoside Analogues as Drugs

Nucleoside analogues are widely used for infections that lead to chronic diseases such as AIDS or hepatitis and are part of antiretroviral treatment. More recently, during the SARS-CoV-2 pandemic, novel nucleoside analogues such as remdesivir or molnupiravir were marketed for respiratory disease.

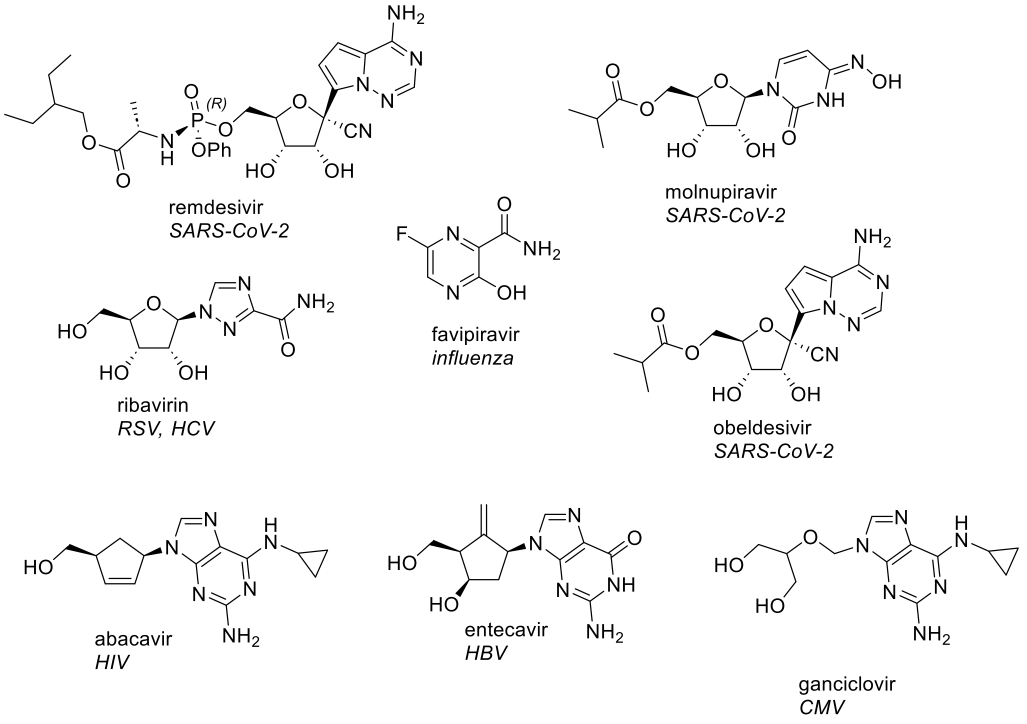

Figure S1: Examples for marketed nucleoside analogues.

Synthesized Analogues in this Work

In total 76 nucleoside analogues have been synthesized and tested for antiviral activity. The following list includes all their structures. Note that isomers **a**-**c** are direct products from the Diels-Alder reaction, while product **d** could be synthesized by epimerisation of **a**. Products e to k reflect late-stage modification, such as DNA-type product **f**.

Table S1: List of synthesized nucleoside analogues.

	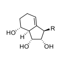	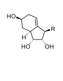	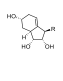	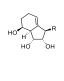	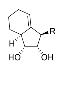	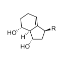	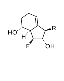	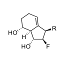	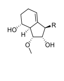	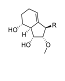	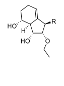
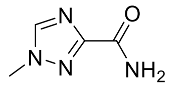	**1a**	**1b**	**1c**								
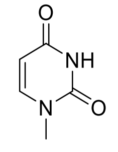	**2a**	**2b**	**2c**	**2d**	**2e**	**2f**	**2g**		**2i (N-Me)**		
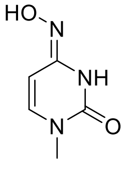	**3a**	**3b**	**3c**	**3d**	**3e**	**3f**					
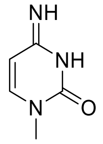	**4a**	**4b**	**4c**	**4d**	**4e**	**4f**	**4g**				
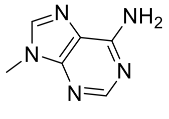	**5a**	**5b**	**5c**	**5d**	**5e**	**5f**	**5g**	**5h**			
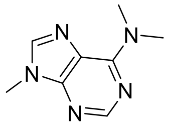	**6a**	**6b**	**6c**	**6d**	**6e**	**6f**	**6g**				
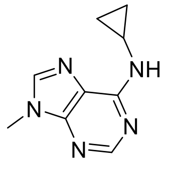	**7a**	**7b**	**7c**								
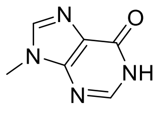	**8a**	**8b**	**8c**		**8d**						

	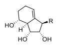	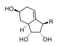	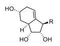	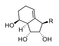	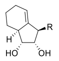	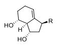	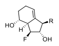	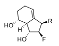	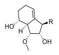	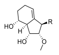	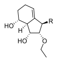
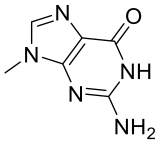	**9a**	**9b**	**9c**								
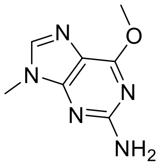	**10a**	**10b**	**10c**								
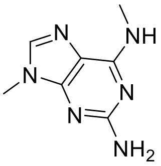	**11a**										
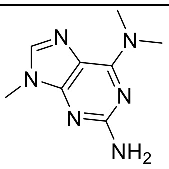	**12a**										
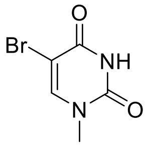	**13a**										
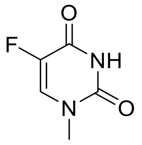	**14a**										
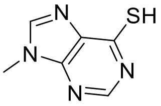	**15a**	**15b**	**15c**								
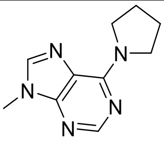	**16a**										
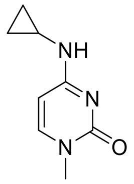	**17a**	**17b**	**17c**								

	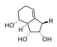	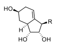	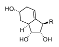	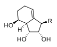	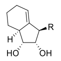	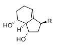	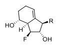	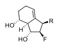	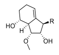	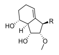	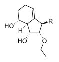
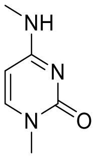				**18d**	**18e**	**18f**					
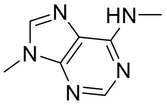	**19a**	**19b**	**19c**								
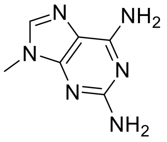	**20a**	**20b**	**20c**								
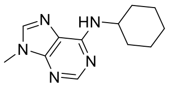	**21a**										
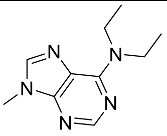	**22a**										
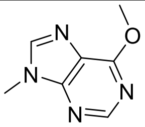	**23a**										

Full Synthetic Schemes

The compounds are in order in accordance with the experimental section of the manuscript.

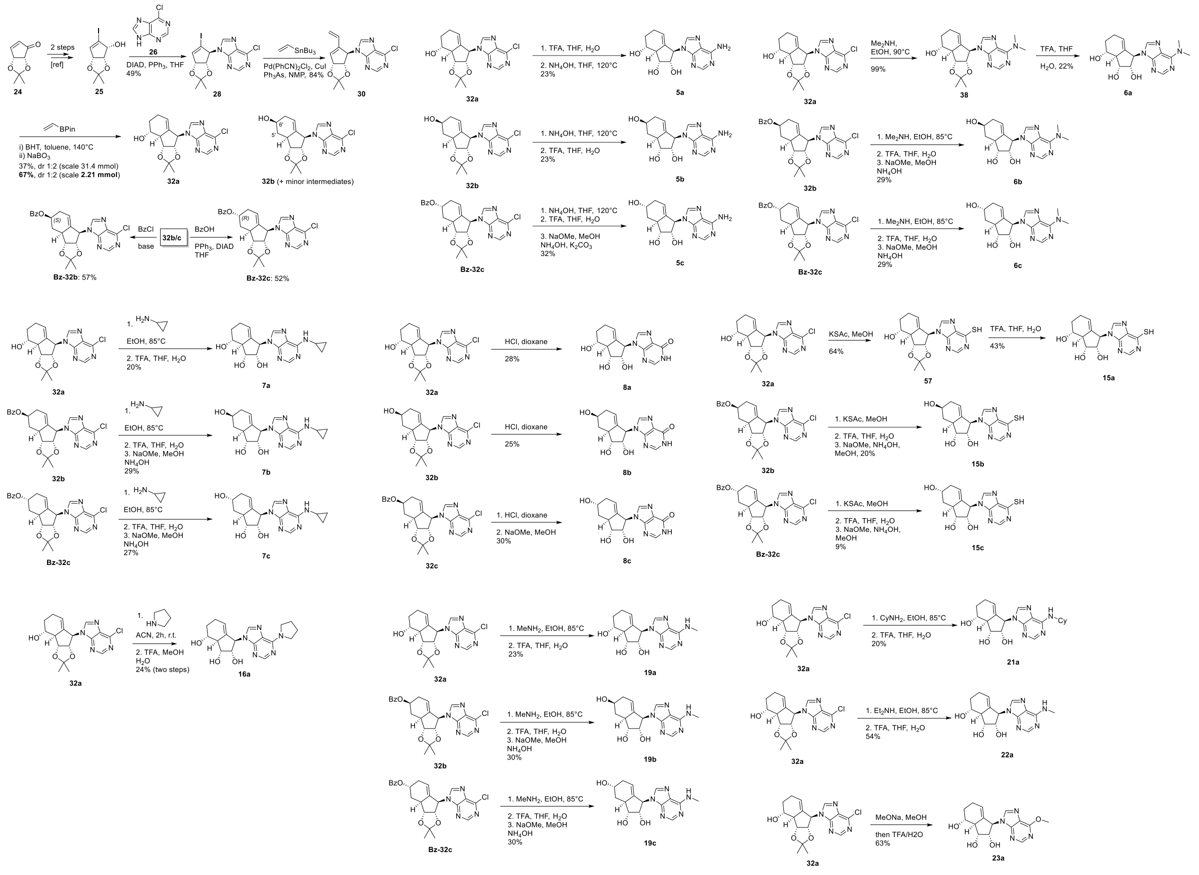

Scheme S1: Synthesis of adenosine, inosine and mercaptopurine analogues.

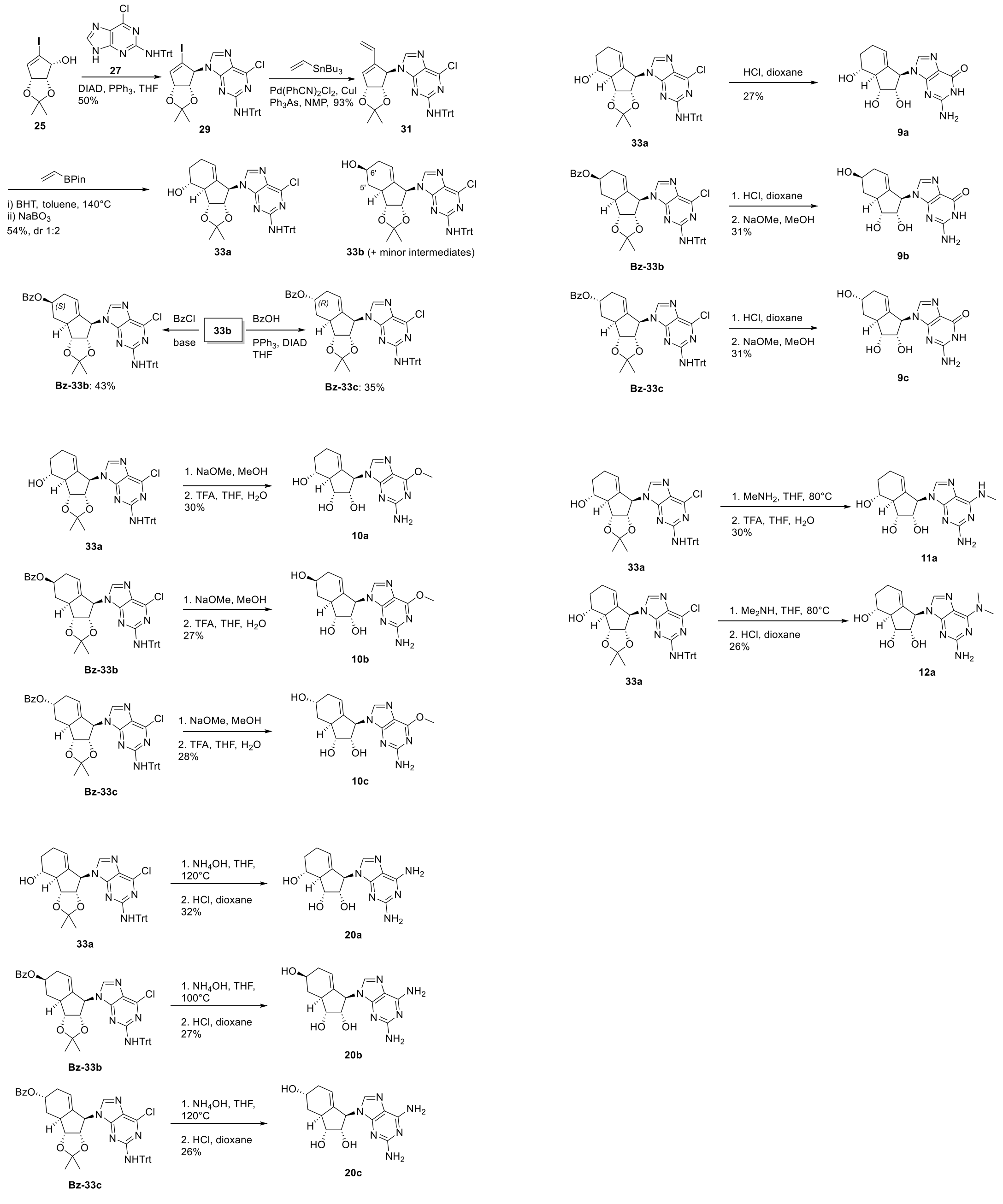

Scheme S2: Synthesis of guanosine analogues.

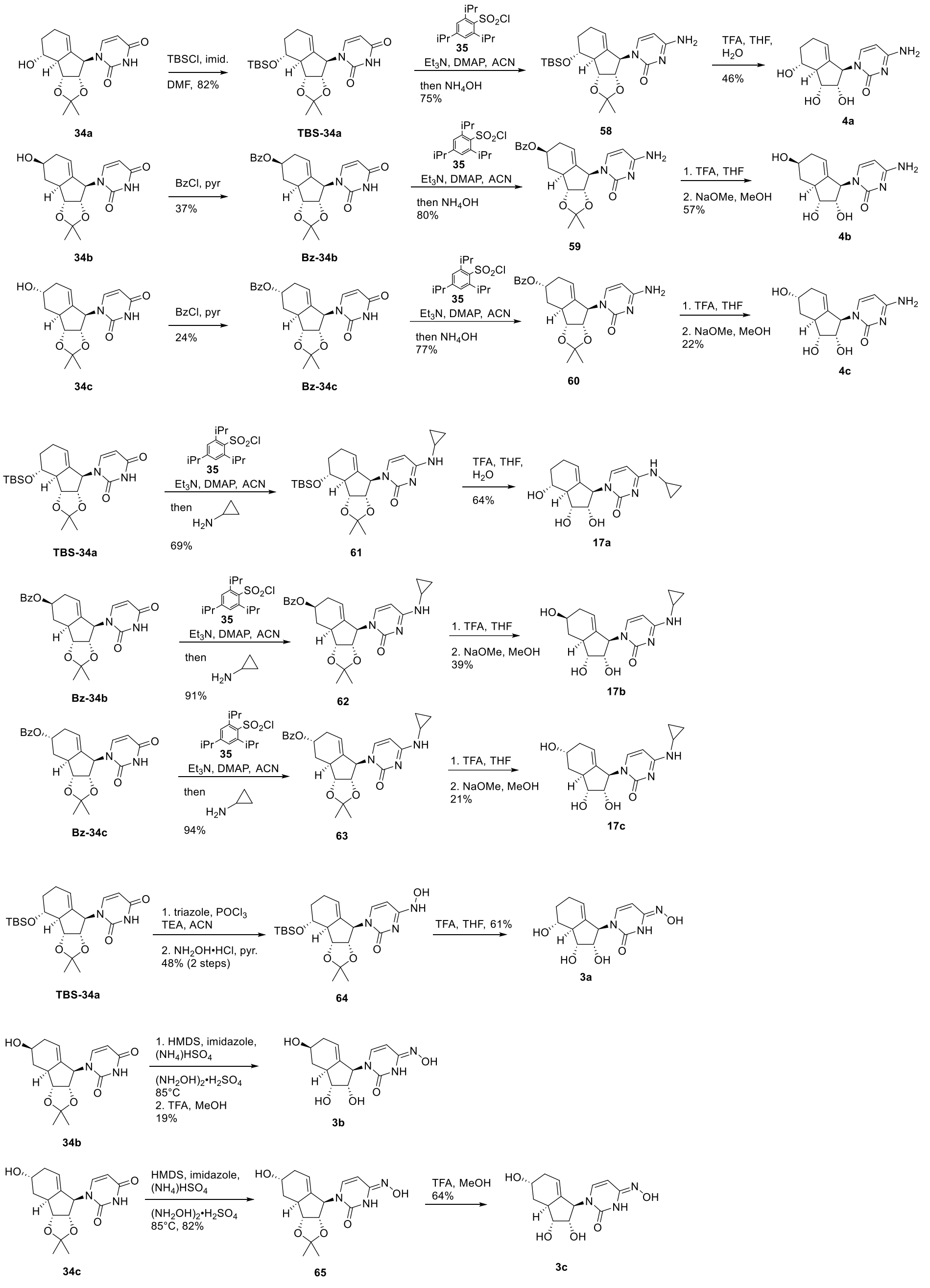

Scheme S3: Synthesis of cytidine and NHC type nucleoside analogues

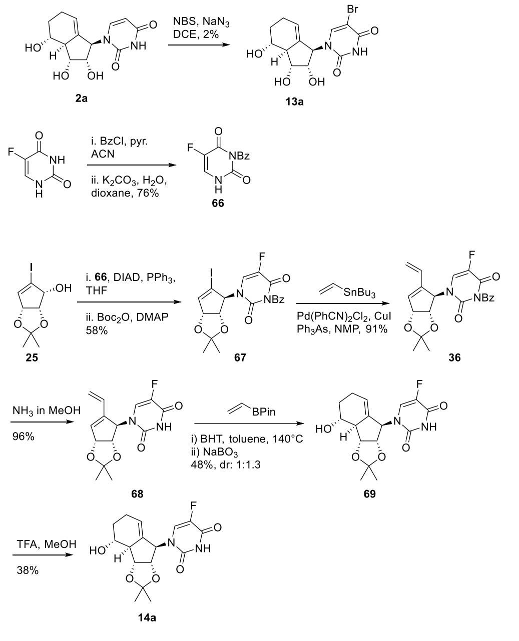

Scheme S4: Synthesis of 5-halogen uracil analogues.

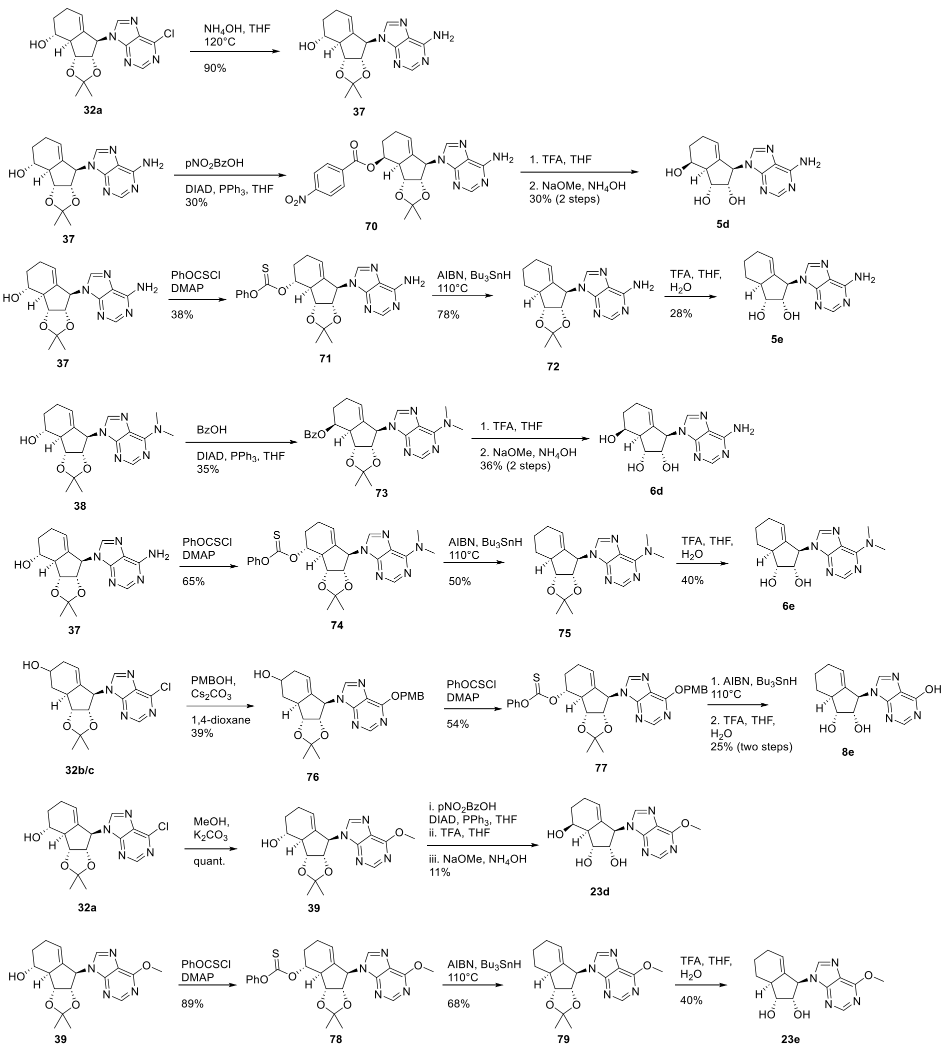

Scheme S5: Synthesis of (5'S) and 5'-deoxy purine analogues.

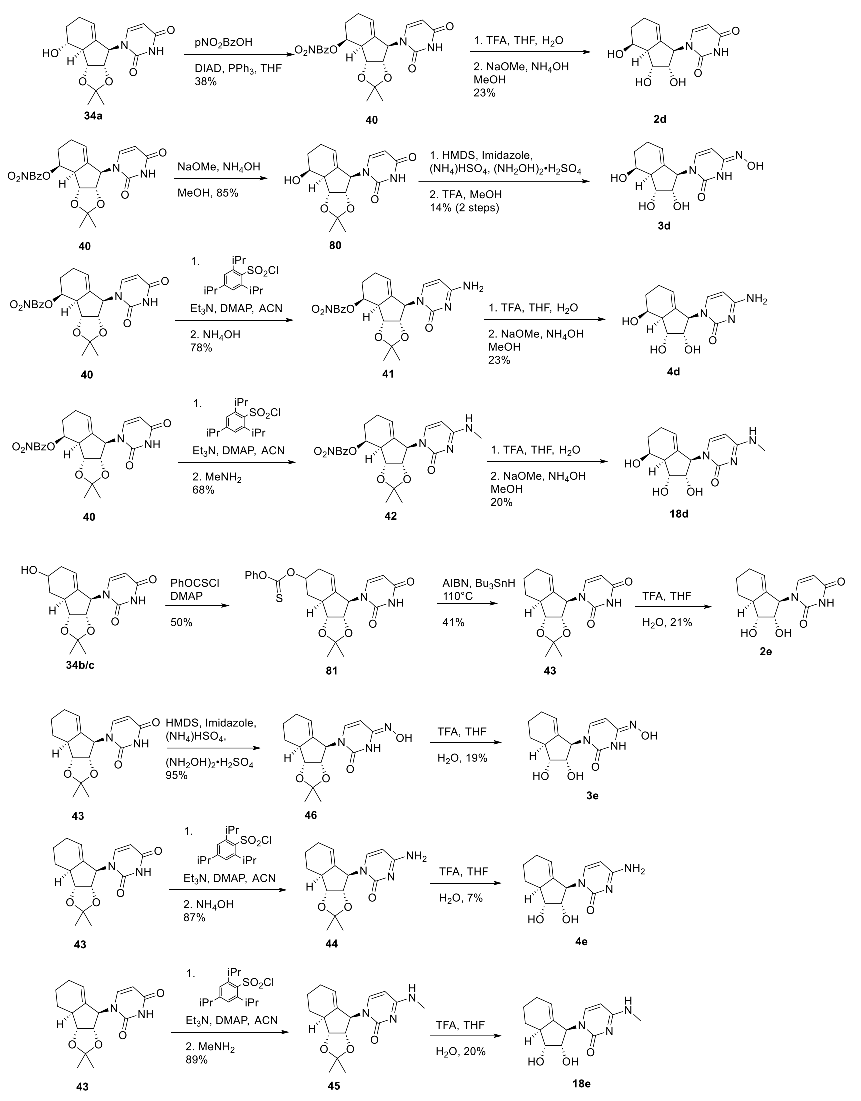

Scheme S6: Synthesis of (5’S) and 5’-deoxy pyrimidine analogues.

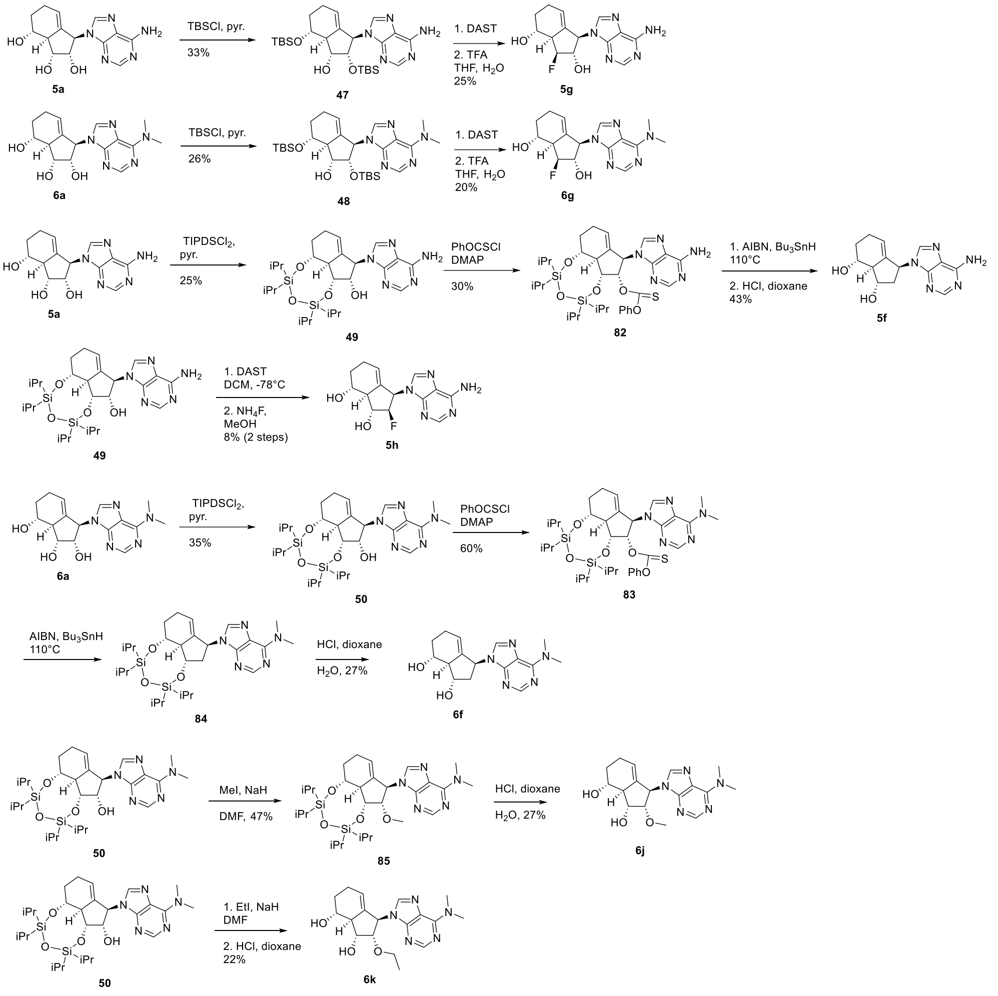

Scheme S7: Synthesis of 2’/3’ modified purine analogues.

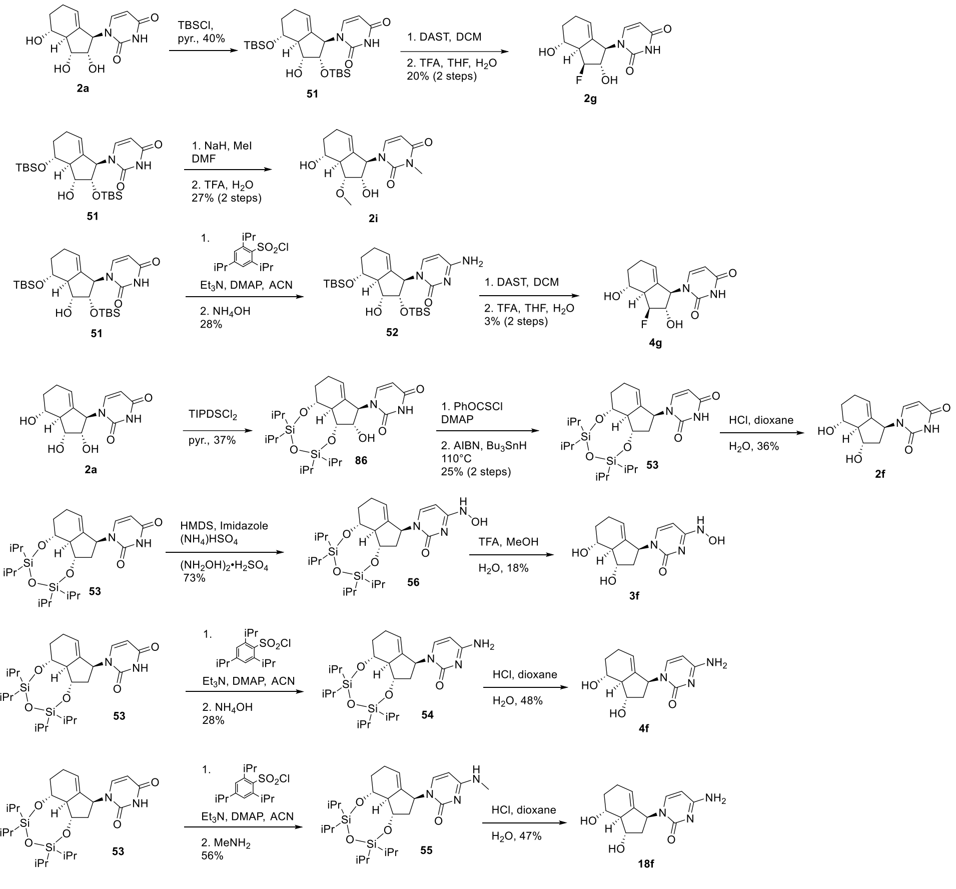

Scheme S8: Synthesis of 2'/3' modified purine analogues.

Additional Antiviral Screening

Table S2: Antiviral Screening and determination of cytotoxicity as described in the experimental part and as shown as in Figure 3. Antiviral activity is expressed as a percentage [%] indicating protection against CPE compared to uninfected cells compound concentration. For HCV, a replicon assay was used. Cell viability is expressed as a percentage in comparison to the DMSO control. Values are mean for n ≥ 3.

Antiviral Activity

	COX CPE				EV71 CPE				HCV Replicon Assay				HPIV CPE			
	40 µM		2 µM		40 µM		2 µM		40 µM		2 µM		40 µM		2 µM	
	AV	STD	AV	STD	AV	STD	AV	STD	AV	STD	AV	STD	AV	STD	AV	STD
**1a**	0.4	0.4	0.4	0.2	-0.1	0.1	-0.1	0.1	-4.2	4.4	-2.2	6.6	-1.2	7.8	0.2	19.7
**1b**	0.0	0.1	0.1	0.3	0.1	0.1	0.2	0.2	25.9	4.4	3.3	5.3	3.1	6.7	3.5	13.4
**1c**	0.2	0.2	0.0	0.1	0.0	0.3	-0.1	0.3	6.4	2.3	0.9	3.5	9.9	8.3	8.5	4.7
**2a**	0.1	0.1	0.2	0.1	0.0	0.2	-0.1	0.2	94.1	0.4	23.7	7.2	-19.4	6.8	10.2	18.1
**2b**	0.5	0.2	0.4	0.3	0.1	0.2	0.1	0.2	-7.8	0.9	-7.4	11.2	-0.3	14.6	-2.0	2.4
**2c**	0.5	0.1	0.5	0.2	-0.1	0.1	0.0	0.2	-8.2	2.9	2.6	8.2	5.8	15.6	7.7	12.7
**3a**	-0.1	0.4	0.0	0.2	-0.6	0.2	-0.1	0.1	-10.4	7.4	-8.4	5.6	5.8	7.9	15.7	20.0
**3b**	-0.2	0.3	0.2	0.3	-0.6	0.2	-0.1	0.2	-6.4	10.5	-7.0	9.5	5.2	11.5	-0.4	2.7
**3c**	-0.3	0.0	0.3	0.1	-0.5	0.2	-0.2	0.2	-7.1	4.7	2.8	4.9	0.5	6.1	-0.9	0.6
**4a**	0.1	0.2	-0.1	0.2	0.1	0.0	0.0	0.1	0.7	5.5	-2.0	8.5	9.8	18.5	-1.1	3.3
**4b**	-0.1	0.1	0.0	0.2	-0.1	0.2	-0.1	0.1	-10.4	9.5	-5.1	15.2	2.0	10.8	0.0	8.1
**4c**	0.1	0.3	0.0	0.2	0.3	0.1	0.2	0.1	-14.8	4.1	-1.6	1.8	5.1	5.4	8.9	12.5
**5a**	0.3	0.3	0.3	0.1	0.3	0.0	0.3	0.1	-3.9	5.0	-4.9	10.3	8.4	9.3	11.9	9.6
**5b**	0.4	0.5	0.1	0.3	0.3	0.1	0.3	0.1	-5.2	5.7	2.9	2.6	0.6	4.5	13.3	5.8
**5c**	0.1	0.4	0.2	0.4	0.1	0.2	0.3	0.2	37.6	6.5	-0.2	7.6	23.2	7.2	6.7	3.8
**6a**	0.0	0.4	0.0	0.3	0.1	0.2	0.1	0.0	40.1	5.3	4.0	3.8	17.1	10.7	7.0	9.5
**6b**	0.3	0.5	0.2	0.3	0.0	0.1	0.1	0.1	26.1	6.8	1.4	5.2	-0.8	0.7	-0.5	7.6
**6c**	0.0	0.3	0.1	0.3	0.0	0.1	0.1	0.1	30.1	6.1	-7.4	8.3	12.2	13.5	4.2	1.8
**7a**	0.0	0.1	0.0	0.1	0.0	0.0	0.1	0.1	-1.9	7.1	3.5	8.8	1.8	2.8	-2.9	12.7
**8a**	0.3	0.5	0.3	0.4	0.1	0.2	0.0	0.0	3.3	5.9	2.8	5.6	-2.7	11.7	-2.5	10.4
**9a**	0.4	0.6	0.0	0.3	0.2	0.1	0.1	0.1	6.9	8.4	2.2	1.5	0.4	4.9	-1.5	11.9
**9b**	0.0	0.2	0.1	0.3	0.2	0.1	0.2	0.1	-0.7	1.2	3.1	4.7	7.0	4.9	0.4	11.1
**9c**	-0.1	0.1	0.1	0.4	-0.1	0.2	0.1	0.2	13.9	5.3	10.6	6.4	11.7	4.8	0.5	3.6
**10a**	0.4	0.4	0.2	0.2	0.2	0.2	0.1	0.2	1.1	4.8	1.1	1.9	9.6	11.7	-2.8	3.0
**10b**	0.0	0.4	0.1	0.6	-0.1	0.1	0.1	0.1	6.7	6.8	3.1	4.4	2.8	7.7	12.5	10.0
**10c**	0.1	0.3	0.0	0.5	0.2	0.1	0.2	0.1	5.2	6.3	4.2	4.5	7.1	5.1	5.1	14.8
**11a**	-0.1	0.3	-0.1	0.3	0.1	0.2	0.0	0.2	0.3	3.1	8.2	3.2	-0.2	4.3	-5.0	10.7
**12a**	0.3	0.5	0.0	0.3	-0.1	0.1	0.0	0.2	18.4	4.2	8.6	5.1	16.1	2.6	15.3	14.5
**AG7088**		IC50 = 6.7 nM				IC50 = 7.6 nM										
**BMS790052**										IC50 = 6.2 nM						
**Ribavirin**														IC50 = 16.8 µM		

	HRV CPE				HSV CPE				ZIKA CPE			
	40 µM		2 µM		40 µM		2 µM		40 µM		2 µM	
	AV	STD	AV	STD	AV	STD	AV	STD	AV	STD	AV	STD
**1a**	-0.7	0.5	-0.6	0.6	5.5	3.7	5.6	4.5	-0.9	0.7	-0.3	0.7
**1b**	-0.2	0.4	-0.2	0.3	-2.4	5.5	-0.2	2.4	-0.8	0.8	-0.6	0.3
**1c**	-0.7	0.5	-0.5	0.1	4.8	7.1	3.6	6.1	-1.3	0.5	-0.4	1.0
**2a**	-0.2	0.1	0.3	0.3	25.3	2.9	3.6	6.3	10.9	1.2	-0.8	0.2
**2b**	0.6	0.3	0.1	0.3	11.7	7.9	4.6	0.8	0.8	1.0	0.5	1.4
**2c**	0.2	0.2	0.4	0.0	2.8	4.3	0.8	5.2	1.4	1.0	1.2	0.7
**3a**	0.3	0.3	0.2	0.4	4.7	6.0	1.8	4.1	0.6	1.1	0.0	0.8
**3b**	-0.7	0.6	-0.7	0.6	-0.2	3.2	4.8	4.7	-0.5	0.2	-0.4	0.8
**3c**	-0.5	0.6	-0.5	0.8	1.6	3.0	2.1	4.1	0.4	1.1	0.4	0.2
**4a**	0.0	0.2	0.0	0.2	1.7	7.0	1.6	2.6	-0.7	0.1	0.0	0.6
**4b**	-0.9	0.2	-0.9	0.0	2.5	2.2	1.2	1.0	0.3	1.4	-0.3	0.9
**4c**	-0.9	0.1	-0.9	0.1	1.2	5.6	1.1	3.5	-0.2	0.9	0.1	0.1
**5a**	0.2	0.3	-0.2	0.5	3.7	0.3	-0.9	3.8	0.2	0.3	-0.7	1.0
**5b**	-0.2	0.3	-0.2	0.3	-2.0	6.0	-3.3	1.6	0.8	1.2	0.2	0.6
**5c**	5.5	0.2	0.3	0.2	8.1	2.4	3.3	3.9	7.6	0.5	2.2	1.4
**6a**	0.4	0.3	0.0	0.1	2.3	8.8	-0.2	4.1	0.9	0.5	-1.1	0.6
**6b**	-0.6	0.3	-0.8	0.4	-2.1	1.8	3.8	5.5	0.9	0.6	-0.7	0.6
**6c**	-0.7	0.2	-0.8	0.2	-1.1	4.9	2.5	2.3	1.7	0.7	0.2	0.6
**7a**	-0.7	0.1	-0.8	0.2	3.0	2.1	-0.5	4.8	1.0	0.7	0.6	0.9
**8a**	0.1	0.3	-0.2	0.1	-3.3	4.4	-2.5	4.1	-1.5	0.3	-1.1	0.5
**9a**	-1.0	0.3	-0.9	0.3	1.6	2.0	5.9	4.2	0.6	0.9	-1.4	0.9
**9b**	-0.4	0.3	-0.4	0.2	-2.2	0.7	2.5	3.1	0.0	0.1	1.1	1.0
**9c**	-0.3	0.4	-0.5	0.1	0.5	3.2	3.3	4.3	0.3	0.5	0.0	0.6
**10a**	-0.2	0.4	-0.2	0.3	-1.6	1.3	-2.4	1.2	0.2	0.9	-0.4	0.8
**10b**	-0.2	0.1	0.0	0.1	-1.6	3.5	-2.9	1.2	1.2	0.9	-0.3	0.4
**10c**	-0.1	0.5	-0.2	0.4	-1.6	2.0	-3.5	1.1	0.4	0.8	0.2	0.4
**11a**	0.0	0.2	-0.1	0.3	1.2	1.2	0.1	2.3	1.0	0.8	0.8	0.8
**12a**	-0.1	0.1	0.1	0.3	-1.9	0.6	1.3	0.6	3.3	1.1	0.7	1.2
**Pleconaril**		IC50 = 0.11 µM										
**Acyclovir**						IC50 = 0.68 µM						
**GS-7977**										IC50 = 4.17 µM		

Cell Viability of Compounds

	HCV Stable/P37				RD cells				LLC-MK2			
	40 μM		2 μM		40 μM		2 μM		40 μM		2 μM	
	AV	STD	AV	STD	AV	STD	AV	STD	AV	STD	AV	STD
**1a**	84.0	5.5	84.3	6.0	98.8	17.9	94.2	21.5	110.3	2.9	106.4	6.6
**1b**	86.4	3.9	93.5	2.8	105.8	3.5	98.3	3.3	94.7	7.3	95.6	4.8
**1c**	87.8	1.8	89.4	1.9	99.1	18.9	100.2	17.0	110.0	1.5	102.2	2.5
**2a**	62.6	1.9	85.7	2.7	51.9	4.1	93.5	17.8	64.0	0.7	93.3	3.7
**2b**	84.0	4.3	88.2	0.4	99.0	19.1	94.3	22.5	104.8	5.5	104.8	6.8
**2c**	88.6	1.3	87.8	4.9	96.8	22.2	93.9	22.6	103.5	6.8	107.6	4.4
**3a**	85.3	4.4	91.4	5.6	101.9	14.1	99.1	18.5	109.7	2.6	105.9	5.6
**3b**	75.4	1.0	81.4	3.3	101.5	14.8	96.2	13.2	107.5	5.3	109.1	4.7
**3c**	82.6	5.0	85.2	3.1	84.9	4.0	96.2	9.0	95.1	7.7	96.5	3.9
**4a**	87.7	3.3	86.3	2.3	104.1	10.6	96.3	9.1	104.1	10.7	101.0	6.3
**4b**	101.0	4.9	110.7	1.3	91.8	5.9	99.0	20.2	86.4	2.9	88.8	3.3
**4c**	100.0	1.8	107.2	6.5	114.5	18.2	111.8	19.5	99.9	1.9	102.6	1.1
**5a**	82.4	2.1	88.3	5.6	105.6	5.5	101.4	10.8	114.0	2.3	100.2	2.7
**5b**	83.7	4.5	87.4	7.7	101.0	9.3	102.1	10.3	103.6	6.5	105.0	3.7
**5c**	46.9	1.4	70.1	2.0	71.7	2.2	103.0	8.1	53.8	8.9	103.6	7.1
**6a**	90.2	2.1	91.8	2.8	96.1	8.8	102.0	4.9	102.7	3.8	98.6	1.5
**6b**	84.4	4.7	93.2	2.4	112.9	20.6	116.3	10.9	98.9	4.3	103.8	4.1
**6c**	99.3	1.5	106.9	5.6	106.0	17.8	114.2	13.3	108.5	1.2	110.0	5.3
**7a**	99.3	2.5	103.2	0.1	90.6	1.9	101.4	10.2	88.0	1.0	94.5	4.8
**8a**	88.9	5.4	85.1	2.0	94.4	13.5	94.9	12.8	102.7	6.2	104.4	4.7
**9a**	95.4	1.5	98.9	2.7	109.1	13.9	107.6	12.6	90.0	5.4	89.7	7.2
**9b**	89.8	0.9	91.3	1.6	104.4	7.0	104.3	7.2	100.8	8.8	107.5	3.4
**9c**	89.5	1.0	91.2	2.3	78.9	4.3	105.5	5.1	106.0	9.9	110.6	5.3
**10a**	90.3	2.5	96.6	3.3	103.8	5.1	104.1	6.8	93.7	7.0	91.2	4.3
**10b**	92.6	4.1	88.3	2.5	73.9	8.4	99.5	11.2	110.2	1.1	108.2	6.6
**10c**	81.1	3.7	86.7	7.3	101.9	9.8	104.0	7.6	103.4	5.1	107.0	6.8
**11a**	85.7	2.3	90.0	4.3	98.3	16.9	98.4	17.7	93.4	6.2	85.0	2.3
**12a**	94.4	4.6	95.9	5.3	98.3	13.7	97.2	14.6	104.5	3.7	100.4	0.9

	h1hela				Vero				Huh7			
	40 μM		2 μM		40 μM		2 μM		40 μM		2 μM	
	AV	STD	AV	STD	AV	STD	AV	STD	AV	STD	AV	STD
**1a**	102.9	0.9	104.9	0.8	96.9	8.5	96.2	7.4	100.4	2.9	101.4	1.5
**1b**	105.8	0.9	103.1	2.8	96.5	1.1	93.0	1.0	97.8	1.0	98.8	1.6
**1c**	104.7	1.3	101.3	2.8	99.3	7.2	104.2	4.5	98.9	1.3	102.0	0.2
**2a**	102.2	1.7	99.5	2.7	54.3	3.0	88.5	3.0	83.5	1.2	95.7	2.4
**2b**	100.4	2.1	100.3	0.2	86.8	6.0	84.2	7.2	102.5	1.1	102.0	1.0
**2c**	101.4	1.7	99.7	2.4	87.7	4.3	87.8	4.1	102.5	1.9	102.6	0.3
**3a**	99.4	0.4	99.2	2.7	92.8	2.9	97.0	3.1	102.7	1.6	102.0	1.3
**3b**	105.1	0.4	104.5	2.6	102.5	6.2	92.7	8.4	103.6	0.9	103.8	3.3
**3c**	106.9	2.7	105.0	2.0	104.8	4.3	97.2	10.5	100.5	1.3	102.1	2.4
**4a**	103.4	2.7	103.2	0.3	94.9	8.1	93.2	8.9	101.0	1.1	101.3	2.8
**4b**	103.7	1.5	102.9	2.0	102.0	0.1	100.9	1.8	99.1	2.2	99.3	1.9
**4c**	100.4	1.2	101.6	4.1	94.0	1.5	94.5	1.2	95.6	3.2	99.3	2.0
**5a**	93.3	0.8	101.1	0.9	88.8	3.1	90.1	4.4	100.8	1.8	99.4	1.1
**5b**	102.5	1.6	102.7	1.4	95.4	0.4	95.8	2.5	97.5	0.7	99.6	1.4
**5c**	41.3	1.3	90.0	2.2	32.7	1.2	76.1	5.7	81.2	0.2	97.7	2.0
**6a**	103.4	2.0	102.1	1.2	96.2	6.1	100.6	4.4	99.9	3.2	100.1	2.5
**6b**	102.5	2.6	102.7	1.3	98.4	6.2	100.1	3.2	98.5	0.3	99.6	5.7
**6c**	102.8	1.0	102.8	0.4	100.8	2.0	100.3	2.9	100.6	1.9	98.6	0.6
**7a**	104.1	1.0	100.9	2.2	90.5	1.7	91.6	2.5	94.1	1.3	98.0	1.5
**8a**	103.7	0.8	102.6	1.5	93.4	9.1	96.8	6.0	101.3	4.6	101.2	3.3
**9a**	99.9	0.3	100.1	2.1	100.3	2.9	101.1	1.6	100.1	2.7	98.1	1.6
**9b**	97.7	3.8	96.0	1.5	93.0	3.4	93.2	0.5	97.5	1.1	97.4	0.7
**9c**	97.9	2.0	96.9	2.3	95.2	1.4	93.0	3.7	96.4	0.7	96.8	1.3
**10a**	98.7	2.4	97.8	2.5	93.0	2.8	94.6	3.9	98.4	2.6	98.3	1.8
**10b**	99.1	1.3	97.9	1.1	99.0	4.0	99.0	5.2	103.0	0.5	99.8	2.4
**10c**	97.1	1.6	99.6	2.5	93.8	2.6	93.2	5.5	98.8	1.3	99.4	1.5
**11a**	97.8	1.1	98.3	1.0	101.4	1.0	102.0	0.1	98.4	0.6	100.5	1.8
**12a**	98.2	1.5	97.8	1.2	96.8	5.0	99.9	3.7	104.6	1.8	99.2	2.6

The data was recorded at WuXi Apptec. Shanghai with the methods stated in the experimental section.

The following data was recorded at Rega Institute (Molecular Genetics and Therapeutics in Virology and Oncology Research Group, KU Leuven, Belgium) with the methods stated in the experimental section. Antiviral efficacy was evaluated against a panel of viruses using cell-specific infection models: On HEL 299: Herpes simplex virus type 1 (HSV-1 KOS), human coronaviruses HCoV-229E and HCoV-OC43, on HEp-2: Respiratory syncytial virus A (RSV-A), on U-87: Yellow fever virus, Zika virus, Sindbis virus, and Semliki Forest virus, and on MDCK: Influenza A/H1N1 (A/Ned/378/05), A/H3N2 (A/HK/7/87), and influenza B (B/Ned/537/05).

Table S3: Additional antiviral screening using CPE assay. The 50% effective concentration (EC_{50}) was calculated based on the inhibition of virus-induced CPE, while the 50% cytotoxic concentration (CC_{50}) was determined from mock-infected cultures.

compound code	start concentration	concentration unit	Cytotoxicity (CC50) HEL 299	MDCK	Hep2	U87	Antiviral Activity (EC50) HCoV 229E HEL 299	HCoV OC43 HEL 299	Influenza H1N1 MDCK	Influenza H3N2 MDCK	Influenza B MDCK	RSV A Long Hep2	HSV-1 KOS HEL 299	Yellow Fever Virus 17D U87	Zika Virus mr766 U88	Sindbis Ar-339 U89	Semliki Forest Virus Original U90
**1a**	50	µM	>50	>50	>50	>50	>50	>50	>50	>50	>50	>50	>50	>50	>50	>50	>50
**1b**	50	µM	>50	>50	>50	>50	>50	>50	>50	>50	>50	>50	>50	>50	>50	>50	>50
**1c**	50	µM	>50	>50	>50	>50	>50	>50	>50	>50	>50	>50	>50	>50	>50	>50	>50
**2a**	50	µM	>50	>50	26.3	10.1	>50	>50	>50	>50	>50	>50	>50	>50	>50	>50	>50
**2b**	50	µM	>50	>50	>50	>50	>50	>50	>50	>50	>50	>50	>50	>50	>50	>50	>50
**2c**	50	µM	>50	>50	>50	>50	>50	>50	>50	>50	>50	>50	>50	>50	>50	>50	>50
**3a**	50	µM	>50	>50	>50	>50	>50	>50	43.1	>50	>50	>50	>50	>50	>50	>50	>50
**3b**	50	µM	>50	>50	>50	>50	>50	>50	>50	>50	>50	>50	>50	>50	>50	>50	>50
**3c**	50	µM	>50	>50	>50	>50	>50	>50	>50	>50	>50	>50	>50	>50	>50	>50	>50
**4a**	50	µM	>50	>50	>50	>50	>50	>50	>50	>50	>50	>50	>50	>50	>50	>50	>50
**4b**	50	µM	>50	>50	>50	>50	>50	>50	>50	>50	>50	>50	>50	>50	>50	>50	>50
**4c**	50	µM	>50	>50	>50	>50	>50	>50	>50	>50	>50	>50	>50	>50	>50	>50	>50
**5a**	50	µM	>50	>50	>50	>50	>50	>50	>50	>50	>50	>50	>50	>50	>50	>50	>50
**5b**	50	µM	>50	>50	>50	>50	>50	>50	>50	>50	>50	>50	>50	>50	>50	>50	>50
**5c**	50	µM	>50	>50	>50	>50	>50	>50	>50	>50	>50	>50	>50	>50	>50	>50	>50
**6a**	50	µM	>50	>50	>50	>50	>50	>50	>50	>50	>50	>50	>50	>50	>50	>50	>50
**6b**	50	µM	>50	>50	>50	>50	>50	>50	>50	>50	>50	>50	>50	>50	>50	>50	>50
**6c**	50	µM	>50	>50	>50	>50	>50	>50	>50	>50	>50	>50	>50	>50	>50	>50	>50
**7a**	50	µM	>50	>50	>50	>50	>50	>50	>50	>50	>50	>50	>50	>50	>50	>50	>50
**7b**	50	µM	>50	>50	>50	>50	>50	>50	>50	>50	>50	>50	>50	>50	>50	>50	>50
**7c**	50	µM	>50	>50	>50	>50	>50	>50	>50	>50	>50	>50	>50	>50	>50	>50	>50
**8a**	50	µM	>50	>50	>50	>50	>50	>50	>50	>50	>50	>50	>50	>50	>50	>50	>50
**8b**	50	µM	>50	>50	>50	>50	>50	>50	>50	>50	>50	>50	>50	>50	>50	>50	>50
**8c**	50	µM	>50	>50	>50	>50	>50	>50	>50	>50	>50	>50	>50	>50	>50	>50	>50
**9a**	50	µM	>50	>50	>50	>50	>50	>50	48.8	>50	>50	>50	>50	>50	>50	>50	>50
**9b**	50	µM	>50	>50	>50	>50	>50	>50	>50	>50	>50	>50	>50	>50	>50	>50	>50
**9c**	50	µM	>50	>50	>50	>50	>50	>50	>50	>50	>50	>50	>50	>50	>50	>50	>50
**10a**	50	µM	>50	>50	>50	>50	>50	>50	>50	>50	>50	>50	>50	>50	>50	>50	>50
**10b**	50	µM	>50	>50	>50	>50	>50	>50	>50	>50	>50	>50	>50	>50	>50	>50	>50
**10c**	50	µM	>50	>50	>50	>50	>50	>50	>50	>50	>50	>50	>50	>50	>50	>50	>50
**11a**	50	µM	>50	>50	>50	>50	>50	>50	>50	>50	>50	>50	>50	>50	>50	>50	>50
**12a**	50	µM	>50	>50	>50	>50	>50	>50	>50	>50	>50	>50	>50	>50	>50	>50	>50
**17a**	50	µM	>50	>50	>50	>50	>50	>50	>50	>50	>50	>50	>50	>50	>50	>50	>50
**17b**	50	µM	>50	>50	>50	>50	>50	>50	>50	>50	>50	>50	>50	>50	>50	>50	>50
**17c**	50	µM	>50	>50	>50	>50	>50	>50	>50	>50	>50	>50	>50	>50	>50	>50	>50
**19a**	50	µM	>50	>50	>50	>50	>50	>50	>50	>50	>50	>50	>50	>50	>50	>50	>50
**19b**	50	µM	>50	>50	>50	>50	>50	>50	>50	>50	>50	>50	>50	>50	>50	>50	>50
**19c**	50	µM	>50	>50	>50	>50	>50	>50	>50	>50	>50	>50	>50	>50	>50	>50	>50
**20a**	50	µM	>50	>50	>50	>50	>50	>50	>50	>50	>50	>50	>50	>50	>50	>50	>50
**20b**	50	µM	>50	>50	>50	>50	>50	>50	>50	>50	>50	>50	>50	>50	>50	>50	>50
**20c**	50	µM	>50	>50	>50	>50	>50	>50	>50	>50	>50	>50	>50	>50	>50	>50	>50
**21a**	50	µM	>50	>50	>50	>50	>50	>50	>50	>50	>50	>50	>50	>50	>50	>50	>50
Remdesivir	10	µM	>10	-	>10	3	0.1	0.3	-	-	-	0.001	>10	0.8	0.3	>10	>10
Chloroquine	100	µM	-	-	-	53.7	-	-	-	-	-	-	-	5.9	7.7	11.8	16.4
Ribavirin	250	µM	>250	>250	124.4	133.1	34.2	38.9	14.7	10.6	4.3	16.1	88.9	-	-	-	-
Zanamivir	100	µM	-	>100	-	-	-	-	1.3	>100	0.4	-	-	-	-	-	-
Rimantadine	100	µM	-	69.7	-	-	-	-	0.4	0.3	>100	-	-	-	-	-	-

Table S4: Antiviral screening of drug library against IAV at 40 μM concentration. Values are mean ± SD (n ≥ 3). CPE assay in IAV (H1N1pdm) infected MDCK cells. Activity compared to DMSO control and uninfected cells; Cell viability of uninfected cells after compound treatment (40 μM) in MDCK cells obtained by CCK-8 assay normalized to DMSO. Negative values indicate increased CPE relative to DMSO.

	Antiviral Activity			Cell Viability			
	CPE			MDCK		A549	
	40 μM			40 μM		40 μM	
	AV	STD		AV	STD	AV	STD
**1a**	24.1%	2.4%		83.3%	2.1%	98.7%	1.3%
**1b**	12.9%	12.3%		92.5%	8.0%	97.3%	9.0%
**1c**	10.3%	22.2%		78.3%	2.6%	91.9%	3.5%
**Protide 1a**	10.4%	3.0%		100.7%	3.9%	100.3%	2.5%
**2a**	45.0%	9.0%		97.9%	6.1%	104.0%	1.8%
**2b**	30.6%	0.8%		109.8%	7.6%	111.7%	3.5%
**2c**	41.2%	1.4%		103.3%	8.8%	109.6%	2.5%
**2d**	33.9%	3.2%		99.8%	0.1%	109.9%	0.5%
**2e**	2.6%	5.0%		92.4%	0.2%	104.3%	1.0%
**2f**	-52.5%	9.8%		88.8%	3.3%	88.1%	1.5%
**2g**	-33.8%	17.4%		92.8%	2.7%	84.6%	0.3%
**2i**	-17.9%	18.0%		100.9%	3.1%	91.8%	1.0%
**3a**	8.3%	3.6%		99.0%	7.0%	96.3%	1.7%
**3b**	7.2%	4.7%		95.5%	1.9%	97.7%	5.8%
**3d**	12.6%	0.9%		95.6%	4.2%	86.4%	2.7%
**3e**	4.8%	3.3%		89.3%	1.8%	89.2%	0.2%
**3f**	19.4%	3.1%		95.1%	4.2%	94.0%	0.3%
**4a**	7.0%	5.1%		101.9%	5.0%	101.0%	6.2%
**4b**	4.2%	4.4%		103.5%	3.7%	103.3%	4.1%
**4c**	7.3%	3.8%		100.0%	6.4%	101.0%	5.3%
**4d**	16.1%	1.6%		94.4%	0.6%	92.3%	6.5%
**4e**	22.4%	8.9%		92.2%	4.7%	97.9%	2.8%
**4f**	11.8%	14.8%		90.5%	0.5%	106.8%	0.8%
**4g**	13.8%	7.8%		90.3%	0.2%	107.8%	1.7%
**5b**	-1.7%	0.6%		77.8%	2.8%	77.3%	1.5%
**5d**	39.6%	3.0%		84.5%	6.4%	95.9%	1.3%
**5e**	9.0%	0.3%		76.0%	1.6%	98.5%	1.2%
**5f**	-12.7%	12.5%		93.0%	1.3%	87.4%	1.0%
**5g**	-31.4%	19.3%		86.8%	1.9%	76.7%	1.4%
**5h**	17.7%	16.4%		72.7%	1.4%	99.0%	3.2%
**6a**	-0.3%	29.9%		94.5%	6.5%	98.5%	11.1%
**6b**	7.2%	3.1%		98.4%	4.0%	100.5%	3.2%
**6c**	7.9%	2.3%		96.2%	6.3%	100.3%	3.9%
**6d**	14.5%	18.9%		91.2%	0.9%	112.9%	2.9%
**6e**	2.0%	2.8%		87.2%	3.3%	109.3%	1.4%
**6f**	-3.3%	1.1%		89.9%	2.4%	106.4%	0.5%
**6g**	7.6%	20.9%		80.6%	0.4%	98.1%	3.6%
**6i**	1.4%	1.4%		96.3%	3.2%	105.4%	1.2%
**6k**	-5.7%	28.1%		83.1%	3.2%	98.3%	1.0%

**7a**	5.5%	5.8%		100.6%	4.8%	97.0%	2.8%
**7b**	10.1%	1.5%		98.1%	2.6%	99.3%	5.3%
**7c**	6.2%	4.8%		99.2%	5.7%	101.4%	4.0%
**8a**	9.0%	4.4%		98.0%	1.4%	99.5%	4.5%
**8b**	8.4%	2.8%		102.1%	5.7%	96.5%	4.6%
**8c**	8.5%	3.2%		100.4%	5.4%	100.3%	5.3%
**8e**	14.0%	4.2%		93.4%	2.6%	89.6%	0.6%
**9a**	9.1%	5.8%		97.0%	5.2%	98.8%	5.7%
**9b**	7.5%	4.7%		97.6%	3.5%	96.1%	2.5%
**9c**	7.2%	3.4%		98.4%	4.3%	95.3%	3.9%
**10a**	2.9%	2.7%		99.8%	5.1%	95.6%	2.0%
**10b**	6.7%	6.2%		101.3%	2.8%	96.1%	1.5%
**10c**	8.3%	0.8%		97.5%	5.2%	101.9%	4.1%
**11a**	5.4%	4.2%		98.8%	4.7%	100.2%	4.8%
**12a**	8.0%	4.9%		99.8%	3.0%	103.2%	6.2%
**13a**	3.9%	15.4%		87.7%	1.4%	93.5%	0.2%
**14a**	24.0%	42.2%		82.0%	2.8%	92.4%	4.3%
**15a**	-3.4%	21.6%		102.7%	5.3%	104.0%	1.2%
**15b**	42.4%	13.3%		89.1%	0.5%	98.1%	4.3%
**15c**	11.1%	4.3%		94.7%	1.0%	84.4%	4.6%
**16a**	21.6%	3.7%		105.9%	3.4%	100.8%	3.6%
**17a**	6.1%	5.3%		100.4%	6.9%	96.5%	4.3%
**17b**	6.2%	6.5%		98.5%	3.4%	96.7%	2.3%
**17c**	6.2%	2.4%		97.7%	6.7%	97.7%	3.1%
**18d**	16.4%	5.2%		98.6%	4.3%	84.0%	1.2%
**18e**	-3.9%	17.0%		93.5%	3.0%	101.9%	0.5%
**18f**	11.0%	1.8%		95.5%	0.1%	88.1%	3.7%
**19a**	5.3%	4.0%		96.7%	3.1%	101.4%	4.3%
**19b**	4.3%	4.3%		98.4%	4.4%	103.6%	3.1%
**19c**	3.5%	3.2%		96.2%	2.4%	100.2%	3.7%
**20a**	7.4%	3.6%		101.2%	2.0%	103.0%	2.0%
**20b**	4.9%	4.1%		102.2%	3.3%	102.2%	6.3%
**20c**	7.3%	5.9%		102.2%	1.9%	97.0%	1.2%
**21a**	5.9%	3.7%		99.1%	6.0%	100.7%	3.0%
**22a**	9.9%	1.1%		101.5%	2.6%	97.2%	3.7%
**23a**	9.0%	1.9%		96.0%	1.2%	101.1%	5.8%
**23d**	12.0%	3.3%		96.8%	0.5%	92.6%	2.9%
**23e**	4.0%	3.0%		96.6%	0.8%	110.0%	1.6%
**Ribavirin**	139.3%	28.2%		119.1%	9.7%	111.9%	4.5%
**Oseltamivir (0.2uM)**	95.0%	9.6%		95.8%	4.3%	95.6%	7.2%
**N6,N6-Dimethyladenosine**	-37.5%	4.9%		78.8%	0.4%	67.5%	7.9%

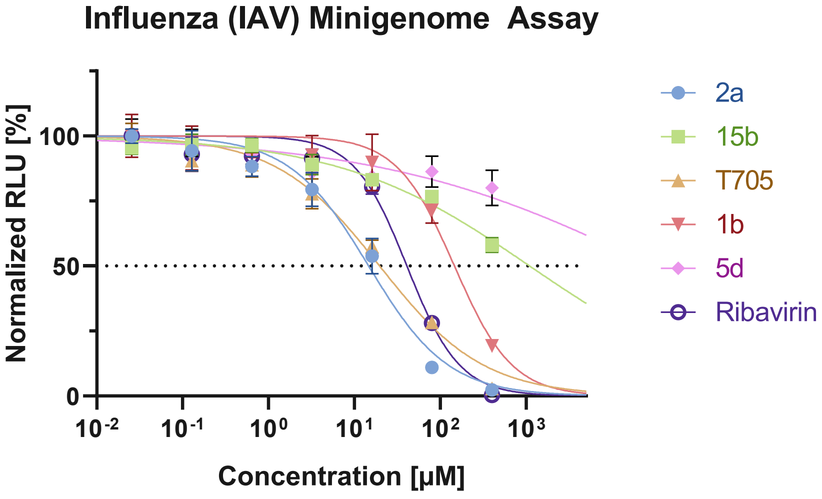

Figure S2: Cell-based dual-luciferase mini-genome assay for IAVpol. HEK-293T cells were co-transfected with PB1, PA, PB2, NP, NS-firefly luciferase, and Gaussia luciferase plasmids. A 5-fold series dilution of each tested compound, ranging from 400 μM to 0 μM, was added to the culture medium for 48 h. The relative inhibitory activity was represented as the firefly luciferase intensity divided by the Gaussia luciferase intensity and normalized to the DMSO-only group (100%). The data points were then fitted to a Hill equation to determine the IC_{50} values.

Synthesis of Monophosphate, Triphosphate and Protide Analogue

As alcohol in pseudo C5' position is secondary instead of primary such in natural ribose, it was expected that unprotected reagents will deliver unselective results. We therefore started with intermediate **A** which was coupled with *in situ* generated phosphorous acid anhydride.[1] Then after TFA mediated deprotection, the phosphonate was first oxidised and activated *in situ* with I_2/pyridine and then subjected to pyrophosphate to yield **TP-1a** or water to yield **MP-1a**.[2] Both products could be isolated by first Sephadex column (LH-20) and then prep. HPLC using a constant solvent mixture of 5% MeOH and 0.01M Et_3N/HCOOH buffer (pH = 8) yielding in the Et_3NH salt.

Protide analogue **Protide-1a** was synthesized following literature known procedures.[3] The coupling with chiral ligand **C** delivered **D** under inversion of the stereocenter at the phosphoamidate. Acidic deprotection yielded in **Protide-1a** in total of 30% yield from **A**.

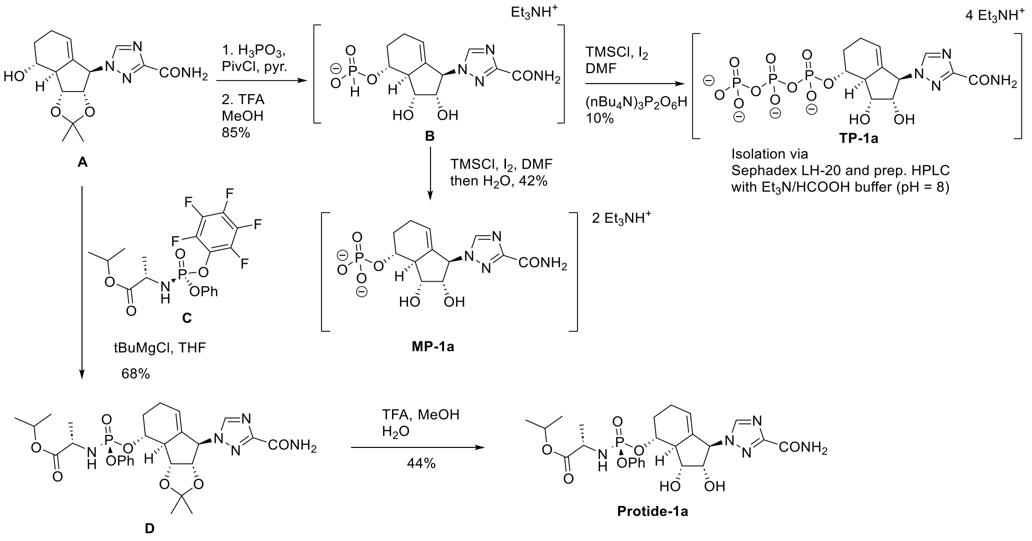

Figure S3: Synthesis of TP analogue (A) and protide analogue (B).

While **2a** displays the highest antiviral activity yet, we set out to synthesize its triphosphate as shown in the Figure S4. In short, while phosphite A was synthesized in usable yields, the synthesis of **TP-2a** failed using this synthetic route. While the formation of the oxidation product B could be confirmed by its quantitative hydrolysis to unwanted **MP-2a**, no TP could be detected in LC-MS studies. Several tries under strict exclusion of water (e.g. distillation of pyridine and TMSCl over CaH_2, drying of starting materials via azeotropic removal of water, addition of 3A MS to ensure anhydrous conditions) did not result in any TP.

Therefore, the synthesis using $POCl_3$ was probed. First direct phosphorylation followed by addition of pyrophosphate and TFA did not yield in any TP, while **MP-2a** could be isolated in usable yields. Out of this experience we concluded that intermediates B and C are too sensitive towards hydrolysis.

Therefore, we envisioned that intermediate D synthesized by activation with CDI would be less sensitive. With this method we could synthesize **TP-2a** in low yield.

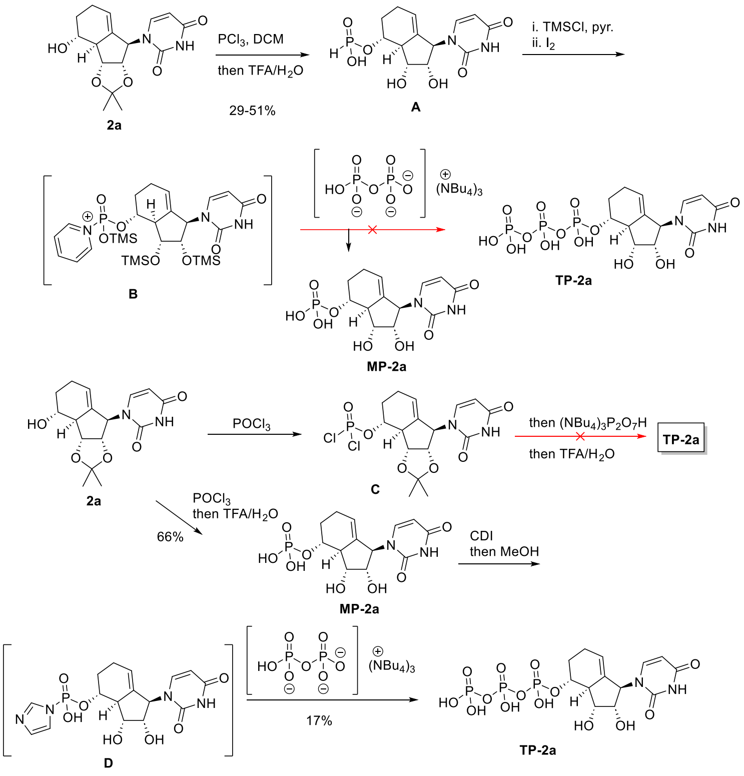

Figure S4: Synthesis of TP and MP for compound **2a**.

Unfortunately, further studies were hampered by our inability to synthesize the triphosphate of compound **2b** (Figure S5). While the above-mentioned approach delivered low but usable yields for **MP-2a** and **TP-2a**, we could not establish an analogues route for **2b**.

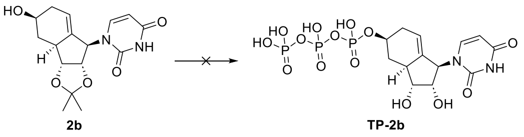

Figure S5: TP-2b could not be synthesized.

Additional Parameters for Metabolism Study

LC-MS/MS Method for the detection of 1a, Protide-1a, MP-1a and TP-1a

Column: Agilent 5HC-C18(2) 150 x 4.6mm (part no. 588915-902)

Mobile phase A: 5mM ammonium formate (pH 4.3)

Mobile phase B: acetonitrile

Flow rate: 0.3mL/min

Gradient:

Time (min)	Mobile Phase B (%)
0.00	5
2.00	5
10.00	10
12.00	95

MRM channels:

Analyte	Parent Mass (m/z)	Product Mass (m/z)	Fragmentor	Collision Energy (V)	Retention Time (min)
**1a**	281.1	96.0	50	41	6.45
**Protide-1a**	550.2	132.9	150	25	8.24
**MP-1a**	361.1	113.0	100	21	5.64
**TP-1a**	521.0	262.9	150	13	5.24

LC-MS/MS Method for the detection of 2a, MP-2a and TP-2a

Column: Waters Acquity UPLC BEH C18 1.7 µM 2.1x50mm

FOR COMPOUND 2a AND MP-2a (ESI positive)

Mobile phase A: 0.1%FA in H2O

Mobile phase B: methanol

Flow rate: 0.15mL/min

Gradient:

Time (min)	Mobile Phase B (%)
0.00	2
3.00	5
4.00	70
4.50	100
9.50	100

MRM channels:

Analyte	Parent Mass (m/z)	Product Mass (m/z)	Fragmentor	Collision Energy (V)	Retention Time (min)
**2a**	281.1	113.1	100	13	1.10
**MP-2a**	361.1	263.2	105	5	2.23

FOR TP-2a (ESI negative)

Mobile phase A: 5mM ammonium formate (pH 5.17)

Mobile phase B: methanol

Flow rate: 0.3mL/min

Gradient:

Time (min)	Mobile Phase B (%)
0.00	50
3.00	50
4.00	100
9.00	100

MRM channels:

Analyte	Parent Mass (m/z)	Product Mass (m/z)	Fragmentor	Collision Energy (V)	Retention Time (min)
**TP-2a**	519	158.7	138	41	1.47

AlphaFold3 H1N1 IAV RNA-dependent RNA polymerase model

The Human IAV (A/Brevig Mission/1/1918(H1N1)) RNA-dependent RNA polymerase (H1N1 IAV polymerase) complex was modelled using AlphaFold 3 (AF3)[4]. RNA sequence was taken from NS gene. The bold and underlined position is the complementary nucleotide for NTP binding. For simplicity and consistency, this position was mutated to other nucleotides to generate different template systems. Two magnesium ions were included.

vRNA 5'UTR: 5' – AGUAGAAACAAGG – 3'

vRNA template: 5' – CGCUCC<u>**A**</u>CUAUCUGCU – 3'

mRNA product: 5' – CGUGCUGGAAAGCAGAUAG – 3'

In total AF3 was performed with eight systems: (1) A:UTP, (2) A:**TP-2a**, (3) G:UTP, (4) G:**TP-2a**, (5) U:ATP, (6) U:**TP-1a**, (7) C:GTP, and (8) C:**TP-1a**.

Limitation of AF3 on correct chirality of carbobicyclic nucleoside analogues

One of the limitations of AF3 model is chirality as discussed in the original paper[4]. For our carbobicyclic nucleoside analogues AF3 predicted majorly incorrect stereoisomers. As a result, compared with natural NTPs, more trials were performed for **TP-2a** and **TP-1a** with multiple seeds, and all the predictions from top-5 output were examined to obtain samples with correct chirality. As a result, except only N=2 was obtained for A/G:**TP-2a** after respective 50 trials, N=5 was successfully obtained for **TP-1a** and natural NTPs with ranking scores in Table S5.

Template	A		G		U		C	
NTP	UTP	**TP-2a**	CTP	**TP-2a**	ATP	**TP-1a**	GTP	**TP-1a**
1	0.89	0.88	0.89	0.88	0.89	0.88	0.89	0.89
2	0.89	0.89	0.89	0.88	0.90	0.88	0.89	0.89
3	0.89		0.89		0.90	0.88	0.90	0.89
4	0.90		0.90		0.90	0.89	0.90	0.89
5	0.90		0.90		0.91	0.89	0.91	0.90

Table S5. Ranking scores of analyzed AF3 predicted structures.

Rescoring of AF3 predicted pose with free energy calculation

The protein and ligands were protonated at pH=7.4 using PDB2PQR v3.6.1[5] and Open Babel 2.4.1[6] respectively. The protein and ligands PDBQT files were generated using ADFRsuite v1.0 [7] and meeko 0.7.1[8] respectively. Binding energies were calculated with smina (version Oct 15 2019)[9] and

shown in Table S6. The structure with the lowest binding energy in each system was used as the corresponding representative structure.

Template	A		G		U		C	
NTP	UTP	**TP-2a**	CTP	**TP-2a**	ATP	**TP-1a**	GTP	**TP-1a**
1	-6.63	**-7.39**	-6.47	-7.06	**-7.12**	**-8.35**	-6.67	-7.35
2	-6.27	-7.38	-6.52	**-8.17**	-6.92	-7.15	**-6.91**	-6.60
3	**-7.06**		-6.46		-6.91	-6.77	-6.49	**-8.05**
4	-6.75		**-6.68**		-6.91	-6.87	-6.56	-7.15
5	-6.56		-5.51		-6.29	-6.66	-5.90	-6.28

Table S6. Binding energy of analyzed AF3 predicted structures. The one with the lowest binding energy was in bold. Units in kcal/mol.

Comparison of AF3 predicted binding pose of carbobicyclic nucleoside analogues with cryo-EM structures

To assess the quality of AF3 predicted structure for studying NTP binding in IAV polymerase, we compared the predicted structure with published IAV polymerase elongation complexes, including H7N9 IAV (PDBID: 7QTL[10]) and bat IAV (PDBID: 6SZV[11]). Since all the three subunits of H7N9 IAV polymerase was resolved, it was used as the reference structure for all structural alignment using PyMOL 2.5.0[12]. AF3 predicted H1N1 IAV polymerase superimposed well with the published structures (Fig. S6A-C) with intact catalytic motifs and catalytic ion binding, supporting the feasibility of applying AF3 in this study. Compared with UTP, **TP-2a** binds to catalytic site with the alpha-phosphate positioned away from the catalytic center. The nucleophilic attack from 3'-oxygen of the nascent RNA to triphosphate is largely hindered by the increased distance from ~4 Å in UTP to ~7 Å in **TP-2a** (Fig. S6A,D). As a result, carbobicyclic nucleoside analogue potentially inhibit IAV polymerase via non-catalytic binding instead of catalysis.

Such mechanism was demonstrated by cryo-EM structures in a recent study. An adenosine analogue 1664-TP was shown to inhibit both coronavirus and arenavirus through non-catalytic binding at the pre-catalytic stage[13]. Based on the higher structural homologies and similarities between its L protein and PA/PB1/PB2 complex of IAV, the analogue-containing cryo-EM structure of Lassa virus (LASV) was used for comparison. LASV polymerase elongation complex containing typical U_{PNPP} was also analyzed as reference. Structure alignment between LASV L protein (LASV polymerase) and IAV polymerase was facilitated by aligning conserved catalytic motifs of viral RNA-dependent RNA polymerase as the following:

7QTL-PB1: resid [222 to 229 239 to 243 (motif F)] [304 to 308 (motif A)] [439 to 448 (motif C)] [454 to 472 (motif D)] [491 to 497 (motif E)]

8XPO-L: resid [1120 to 1127 1134 to 1138 (motif F)] [1192 to 1196 (motif A)] [1328 to 1337 (motif C)] [1350 to 1369 (motif D)] [1385 to 1391 (motif E)]

Similar to **TP-2a** and UTP in IAV polymerase predicted by AF3, an increased attacking distance was also observed with LASV polymerase elongation complex, from ~3 Å with U_{PNPP} to ~7 Å with 1664-TP (Fig. S6E,F). Despite different interaction details, similar structural elements were involved in 1664-TP binding for its effective intervention[13], which was also observed for **TP-2a** and **TP-1a** (see next section).

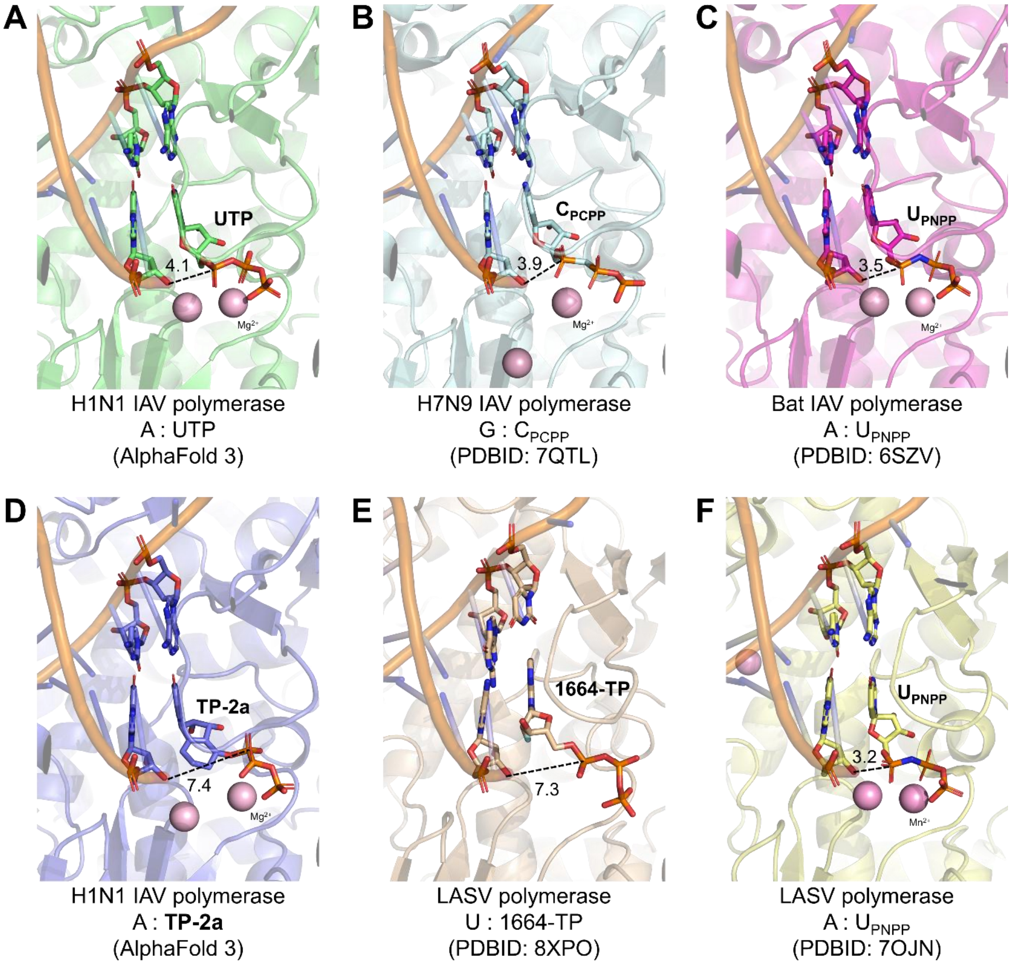

Figure S6. Predicted binding mode of carbobicyclic nucleoside analogues by AF3 and comparison with IAV and LASV polymerase cryo-EM structures. (A) H1N1 IAV polymerase A:UTP complex predicted by AF3. (B) H7N9 IAV polymerase G:C_{PCPP} complex (PDB ID: 7QTL[10]). (C) Bat IAV polymerase A:U_{PNPP} complex (PDB ID: 6SZV[11]). (D) H1N1 IAV polymerase A:**TP-2a** (uridine analogue) complex predicted by AF3. (E) LASV polymerase U:HNC-1644 complex (adenine analogue) (PDB ID: 8XPO[13]) (F) LASV polymerase A:U_{PNPP} complex (PDB ID: 7OJN[14]). Distance between alpha-phosphate of the incoming NTP and 3'-oxygen of the nascent RNA was labelled in unit of Å in (A-F).

Carbobicyclic nucleoside analogues maintain intact contact with active site residues via altered binding mode

Both **TP-1a** and **TP-2a** showed intact interactions with the critical active site residues despite different positioning of the triphosphate and ribose. For instance, in the A:NTP complex, **TP-2a**

maintained interactions with the positively charged residues R239 and K235 using different phosphate oxygens than UTP (Fig. S7A-B). In UTP, K235 interacted with alpha-phosphate, while R239 interacted with the bridging oxygen between alpha- and beta-phosphate. These interactions were mostly altered in **TP-2a** due to the non-catalytic binding of the alpha-phosphate. Firstly, **TP-2a** maintained the binding of alpha-phosphate in active site by interacting with R239 in replace of K235. Secondly, **TP-2a** interacted with both K235 and R239 via beta-phosphate to further stabilize the interactions. In addition, the interactions between K481 and gamma-phosphate were also preserved. Overall, **TP-2a** preserved intact contacts with these the critical positively charged residues in alternative triphosphate binding mode. The interactions between NTP and IAV polymerase PB1 loop 306-310, mainly through backbone interactions, were also largely maintained. Notably, N310 backbone interacted with alpha-phosphate of **TP-2a**, in contrast to ribose oxygen of UTP. The binding of ribose oxygen of **TP-2a** was instead maintained by interaction with N310 sidechain. Lastly, interaction between nucleobase and K229 was also preserved. Similar observations were also obtained in G:NTP complexes (Fig. S7C-D).

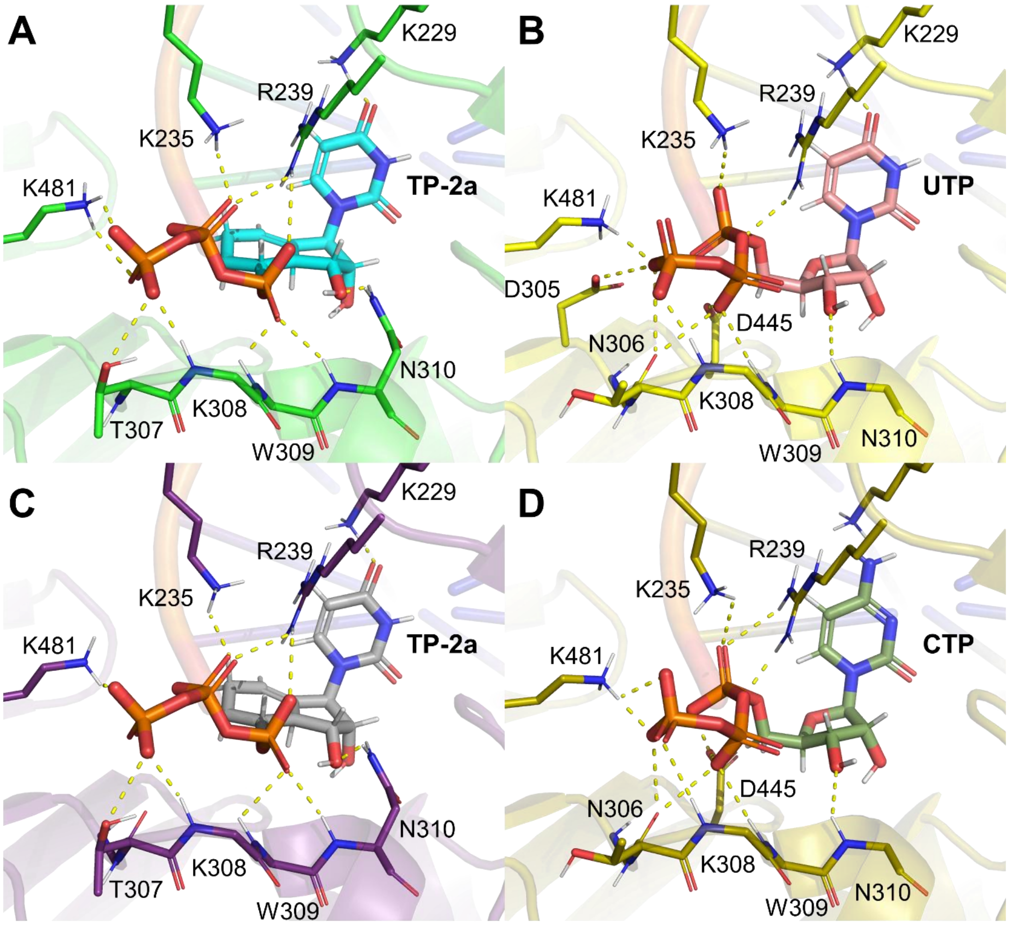

Figure S7. Comparison of protein-ligand contacts in active site predicted by AF3 between complexes containing **TP-2a** and natural NTPs with modelled hydrogens. (A) A:**TP-2a** (B) A:UTP (C) G:**TP-2a** (D) G:CTP.

TP-1a adopted a similar scheme to **TP-2a** in maintaining interactions between triphosphate and active site residues K235, R239 and K481 as shown in C:NTP complexes (Fig. S8A-B) and A:NTP complexes (Fig. S8C-D), with noticeable difference in ribose binding. In contrast to N310, **TP-1a** ribose interacted with S444 with a more tilted orientation. With this orientation, **TP-1a** as a guanine analogue, modified with six-member ring truncated, only maintained the interaction between K229 and the five-member ring of nucleobase as in GTP (Fig. S8C-D). Similar observations were also obtained in A:NTP complexes (Fig. S8C-D).

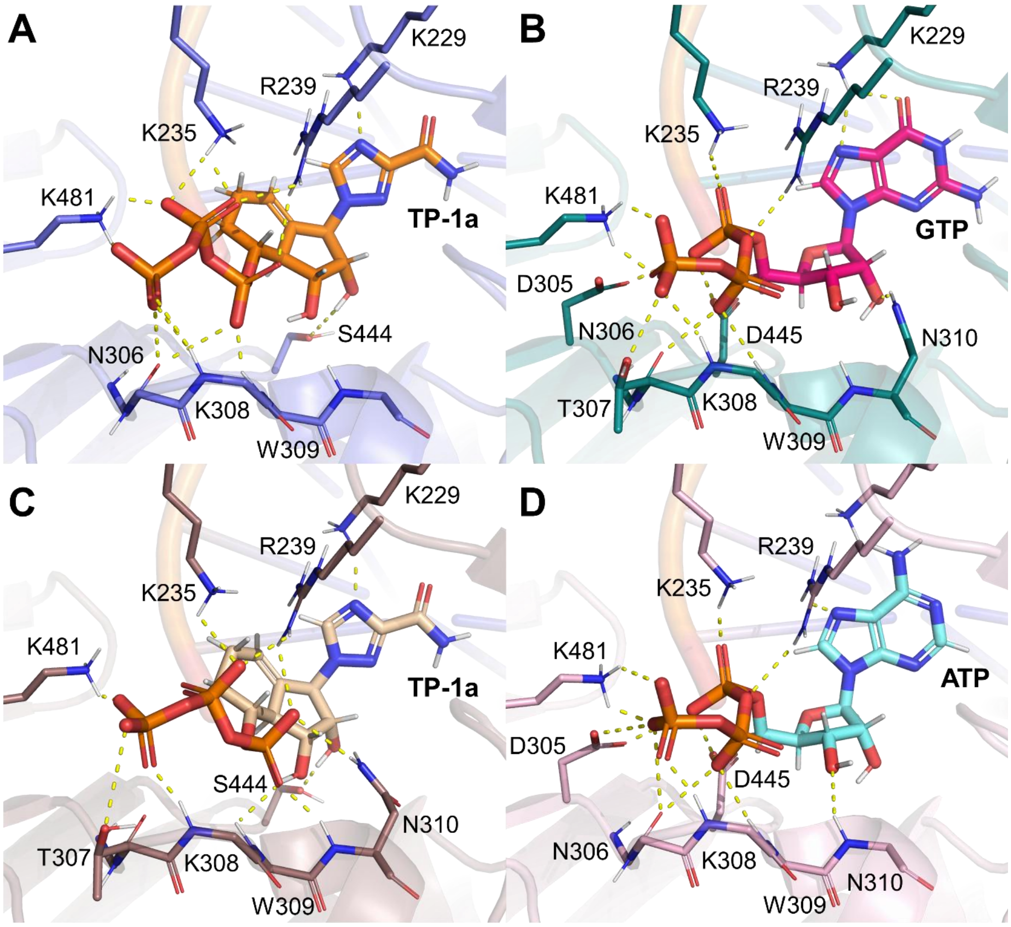

Figure S8. Comparison of protein-ligand contacts in active site predicted by AF3 between complexes containing **TP-1a** and natural NTPs with modelled hydrogens. (A) C:**TP-1a** (B) C:GTP (C) U:**TP-1a** (D) U:ATP.

Carbobicyclic nucleoside analogues forms base pairing with template nucleotide

Both **TP-1a** and **TP-2a** formed base pairing with the template nucleotide. **TP-2a** as a uridine analogue completely resembled base pairing mode of natural UTP with template A (Fig. S9A-B). It could also form Watson-Crick like base pair with tautomeric template G (Fig. S9C) in against to natural G:C base pair (Fig. S9D). **TP-1a** is a guanine analogue that lacks one amine group, causing a slightly different base pairing mode with template C compared with GTP (Fig. S10A-B). Compared with GTP, **TP-1a** adopted a slightly tilted orientation in forming base pair with template C. The $–NH_2$ group in **TP-1a**, originally the –NH– group at position 1 of G, formed hydrogen bond with the receptor at position 2 not 3 of template C. In addition, it also appeared to be able to form wobble base pair with U with a suboptimal angle. Overall, these suggested that carbobicyclic nucleoside analogues were able to form base pairing with template nucleotide as natural NTPs.

Totally, carbobicyclic nucleoside analogues potentially inhibit IAV polymerase through inhibitory binding at pre-catalytic stage, as supported by the high quality AF3 prediction and cryo-EM structures analyses.

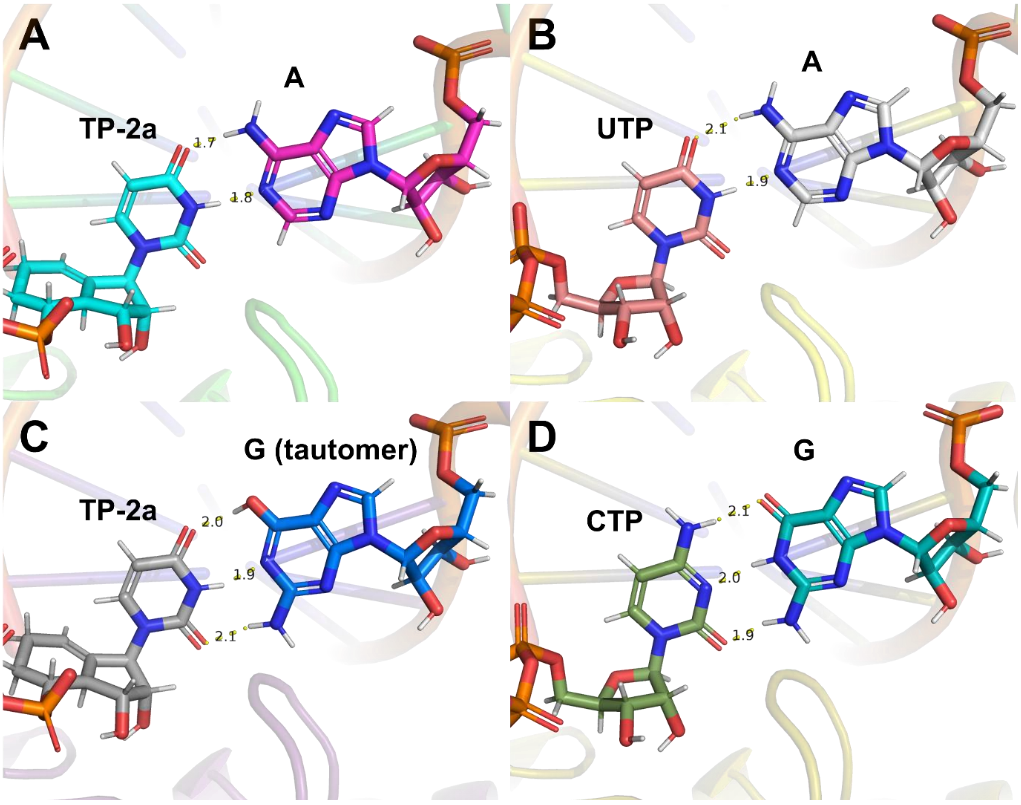

Figure S9. Base pairing interactions of **TP-2a** with template A/G compared with natural NTPs with modelled hydrogens. (A) A:**TP-2a** (B) A:UTP (C) G:**TP-2a** (D) G:CTP.

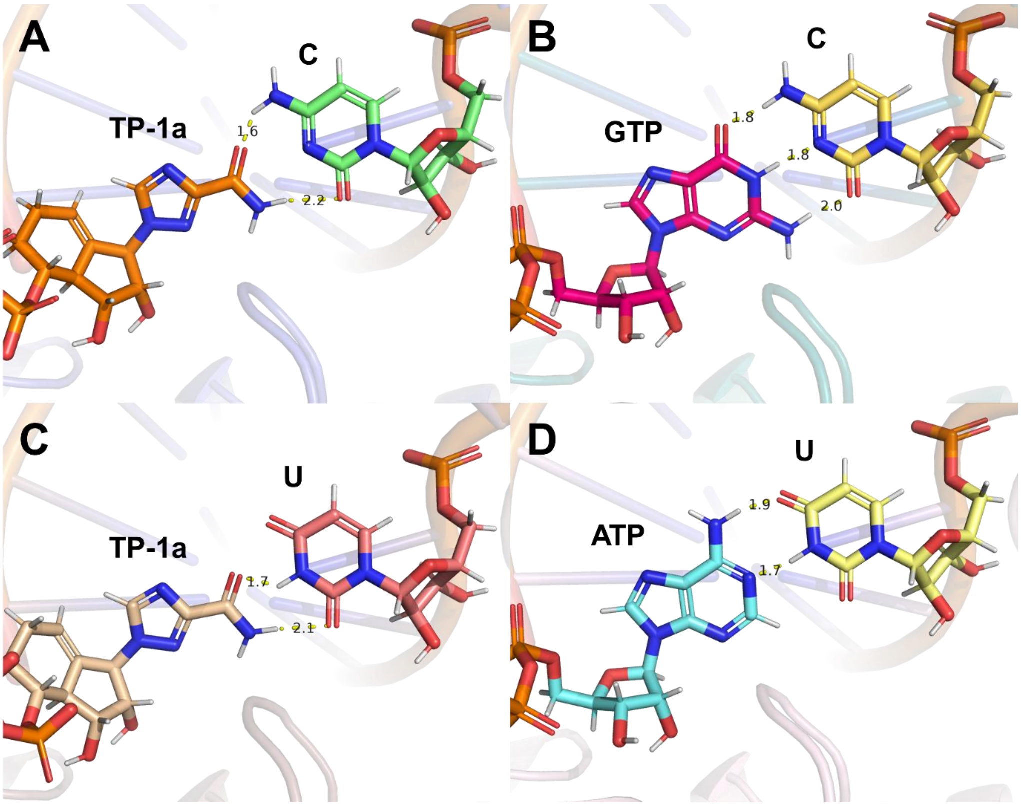

Figure S10. Base pairing interactions of **TP-1a** with template A/G compared with natural NTPs with modelled hydrogens. (A) C:**TP-1a** (B) C:ATP (C) U:**TP-1a** (D) U:ATP.

RNA Polymerase Inhibition Assay

Protein Purification

Yeast RNA Polymerase II (RNAP II)

The yeast cells were cultured in 20 L of YBD medium supplemented with Hygromycin B at 30°C. Cells were harvested when the optical density at 600 nm (OD600) reached 5-8. The harvested cells were resuspended in deionized water (ddH2O) for washing, then pelleted by centrifugation. The cell biomass was measured using an analytical balance, after which the cells were resuspended in an equal volume of lysis buffer. The cell suspension was aliquoted into liquid nitrogen to form pellets. Cells were lysed using a freezer mill for 20 cycles. The resulting cell powder was resuspended in an equal volume of lysis buffer and incubated with gentle stirring at 4°C for 2 hours. The lysate was centrifuged at 45,000 g for 1 hour at 4°C, and the supernatant was collected. The RNA Polymerase II (RNAPII) was purified using anti-Flag affinity beads. Eluted fractions were subsequently loaded onto a MonoQ anion exchange chromatography column and eluted with a linear salt gradient from low to high salt concentrations. Finally, the purified protein was subjected to size exclusion chromatography for further purification.

Mitochondrial Polymerase (RNAPMT) Purification

The RNAPMT gene sequence was synthesized by the company with codon optimization and fused with a His-tag at the N-terminus within the pET28a(+) plasmid. The plasmid was subsequently transformed into BL21 (DE3) cells. Bacterial cultures were incubated at 37°C until the optical density at 600 nm (OD600) reached 0.6. Protein expression was induced by the addition of 0.5 mM IPTG, followed by incubation at 16°C overnight. The bacterial cells were harvested and resuspended in lysis buffer. Cell disruption was performed through sonication, and the lysate was clarified by centrifugation to collect the supernatant. RNAPMT was purified using Ni-NTA affinity chromatography, followed by dialysis to remove excess salt. Final purification was achieved via heparin affinity chromatography using FPLC.

In vitro transcription elongation assay

The related sequences were procured from Integrated DNA Technologies (IDT). The RNA9 oligonucleotide was labeled with γ-^{32}P ATP by T4 PNK at 37°C for 2 hours, followed by heat inactivation of the enzyme. Subsequently, the transcription elongation complex was assembled. Specifically, 7 pmol of TDS was annealed with an equivalent amount of RNA9, gradually cooled from 42°C to 20°C. Then, 20 pmol of RNA polymerase was added to bind with the transcription bubble at

room temperature for 10 minutes. To complete the elongation complex, 21 pmol of NDS was introduced at 37°C for an additional 10 minutes. The mixture was aliquoted into six samples to evaluate the titration conditions of **TP-1a**. Each sample was combined with 50 μM rNTPs. Negative control samples were prepared by adding 50 mM EDTA to inhibit transcription in one group. The remaining five samples were incubated with TP-1a at concentrations of 0 μM, 2 μM, 10 μM, 25 μM, and 50 μM, respectively. All transcription reactions were conducted at 37°C for 5 minutes and subsequently quenched with 2x stop buffer. The samples were heated to 65°C and subjected to electrophoresis on an 8 M Urea, 20% polyacrylamide gel. The resolved gels were exposed to a phosphor screen (Azure Biosystems) and imaged using the Sapphire Biomolecular Imager (Azure Biosystems).

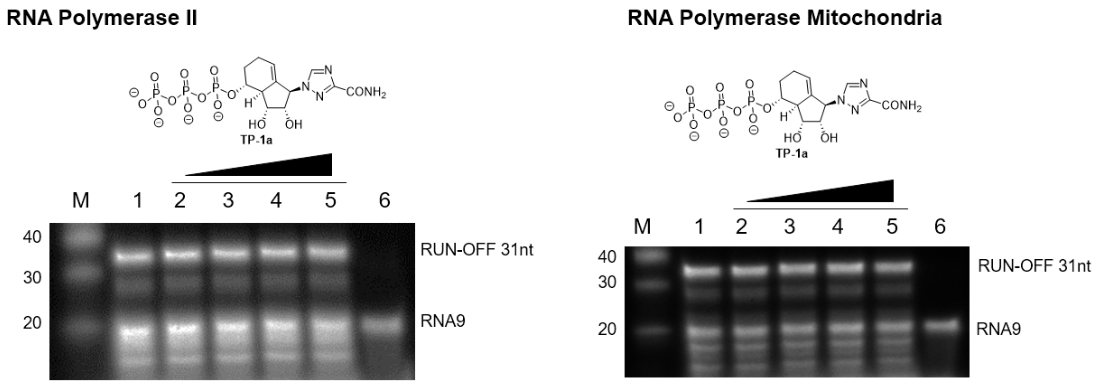

1. 50 μM rNTP
2. 50 μM rNTP + 2 μM **TP-1a**
3. 50 μM rNTP + 10 μM **TP-1a**
4. 50 μM rNTP + 25 μM **TP-1a**
5. 50 μM rNTP + 50 μM **TP-1a**
6. 50 μM rNTP + 100 mM EDTA

Figure S11: *In vitro* transcription elongation assay. Representative gel of two replicates.

DNA Polymerase Inhibition Assay

For compounds **1a** and **TP-1a** no inhibition of DNA polymerases was observed until 100 µM.

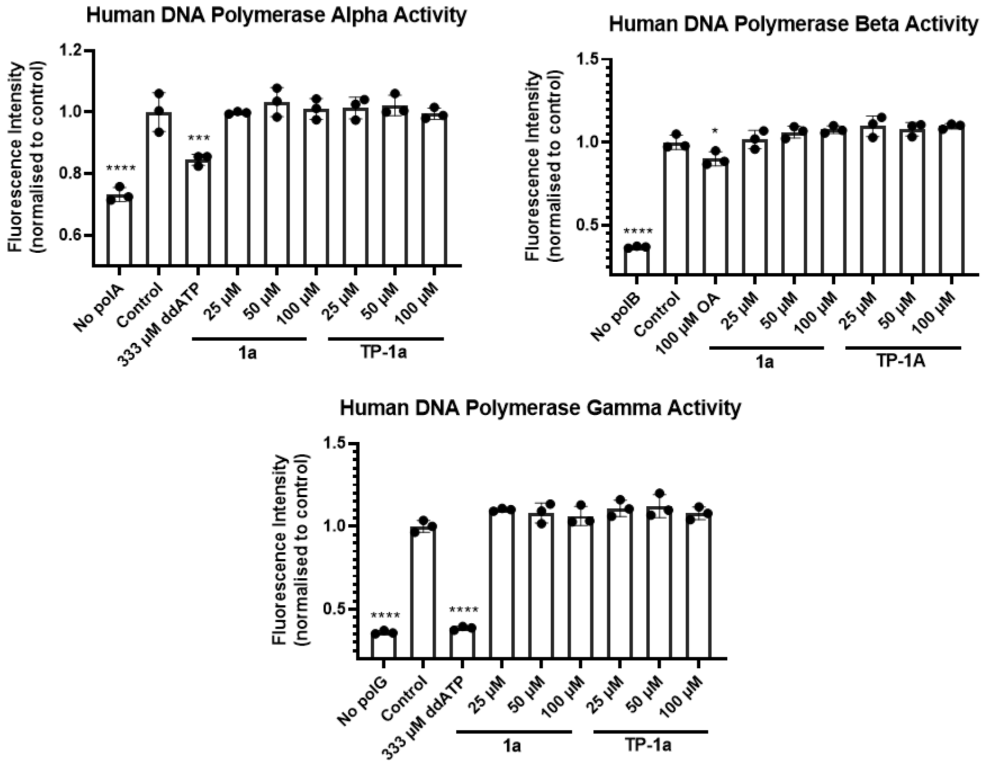

Figure S12: DNA polymerase inhibition assay. Polymerase activity is normalized to DMSO control and represents mean of 3 replicates. Positive controls are ddATP and OA (oleic acid).

Additional Details for Cellular Assays

Table S7: B/D expansion medium

Name of reagents or supplements	Brand	Final Concentration
Bronchial epithelial cell medium	Sciencell	50%
Advanced (ad) DMEM	Gibco	23.5%
R-spondin 1 conditioned medium	In-house preparation	20%
HEPES	Gibco	10 mM
Glutamax	Invitrogen	2.0 mM
P/S	Gibco	100 U/ml
Primocin	InvivoGen	100 U/ml
B27	Gibco	2%
Hydrocortisone	Sigma	0.5 µg/ml
3,3’,5-Triido-L-thyronine (T3)	Sigma	100 nM
Epinephrine	Sigma	0.5 µg/ml
N-acetyl-cysteine	Sigma	1.25 mM
Nicotinamide	Sigma	5 mM
TGFb inhibitor (A83-01)	Tocris	1 µM
BMPi (DMH-1)	Selleckem	1 µM
Rocki (Y-27632)	Abmole bioscience	5 µM
SB202190	Sigma	500 nM
Fibroblast growth factor 10 (FGF10)	Peprotech	100 ng/ml
Fibroblast growth factor 7 (FGF7)	Peprotech	25 ng/ml
Insulin-like growth factor-1 (IGF-1)	Peprotech	25 ng/ml

Table S8: ALI-Diff medium

Name of reagents or supplements	Brand	Final Concentration
Advanced DMEM/F12	Gibco	1x
Hydrocortisone	Sigma	0.5µg/ml
3,3’,5-Triido-L-thyronine (T3)	Sigma	100nM
Epinephrine	Sigma	0.5µg/ml
hEGF	Thermofisher	0.5ng/ml
TTNPB	Cayman	100nM
A83-01	Tocris	50nM
P/S	Gibco	100U/ml

Table S9: AO medium

Name of reagents or supplements	Brand	Final Concentration
R-spondin 1 conditioned medium	In-house preparation	
FGF7	Peprotech	25ng/ml or 5ng/ml
FGF10	Peprotech	100ng/ml or 25ng/ml
A83-01	Tocris	500nM
Y-27632	Abmole	5µM
SB202190	Sigma	500nM

B27	Gibco	1x
N-acetyl-cysteine	Sigma	1.25mM
Nicotinamide	Sigma	5mM
GlutaMax	Invitrogen	1x
HEPES	Invitrogen	10mM
P/S	Gibco	100U/ml
Primocin	Invivogen	50µg/ml
Advanced DMEM/F12	Invitrogen	1x

Table S10: Primer sequence for qPCR.

	**5'--> 3' sequence**
Influenza *M* F	GGCATTTTGGACAAAKCGTCTA
Influenza *M* R	CTTCTAACCGAGGTCGAAACG

Additional Details for Chemical Synthesis

Parameters for HPLC separation

Properties for Preparative HPLC Purification of 2e

PROJECT NAME	CIN2-C-818-CIN2-X-0059-053			
COLUMN NAME & ID	Sunfire Prep C18 (150 x 19 mm, 5um)			
COLUMN NO.	PCH-050			
MOBILE PHASE A	0.05% FORMIC ACID IN WATER			
MOBILE PHASE B	ACETONITRILE:THF (90:10)			
FLOW RATE(ML/MIN)	18			
INSTRUMENT ID	PHP-03-WATERS 2545 QUARTENERY SYSTEM WITH WATERS 2489 UV Detector.			
METHOD	TIME	FLOW	%A	%B
	Initial	18	100	0
	1.00	18	90	10
	17.00	18	83	17
	17.01	20	0	100
	19.00	20	0	100
	19.01	18	100	0
	23.00	18	100	0
LOADING (mg)	20			
NO. OF INJECTIONS	23			
SAMPLE PREPARATION	ACN-MEOH-WATER			

Properties for Preparative HPLC Purification of 2f

PROJECT NAME	CIN2-C-818-CIN2-X-0009-042-a			
COLUMN NAME & ID	Waters X-Bridge Prep C8 (250mm x 19mm x 5µm)			
COLUMN NO.	PCH-026			
MOBILE PHASE A	0.05 % AMMONIUM HYDROXIDE IN WATER			
MOBILE PHASE B	ACETONITRILE			
FLOW RATE(ML/MIN)	15			
INSTRUMENT ID	PHP-06-WATERS 2545 BINARY SYSTEM WITH WATERS 2489 UV Detector			
METHOD	TIME	FLOW	%A	%B
	Initial	15.00	100	0
	1.00	15.00	100	0
	17.00	15.00	90	10
	17.01	15.00	0	100
	19.00	15.00	0	100
	19.01	15.00	100	0
	21.00	15.00	100	0
LOADING (mg)	58			
NO. OF INJECTIONS	12			
SAMPLE PREPARATION	WATER			

Properties for Preparative HPLC Purification of 2g

PROJECT NAME	CIN2-C-816-CIN2-X-0001-022-A
COLUMN NAME & ID	Shim-Pack GIST C18 (250mm x 20mm x 5µm)
COLUMN NO.	PCH-053
MOBILE PHASE A	0.05 % TRIFLURO ACETIC ACID IN WATER
MOBILE PHASE B	ACETONITRILE
FLOW RATE(ML/MIN)	18
INSTRUMENT ID	PHP-06-WATERS 2545 BINARY SYSTEM WITH WATERS 2489 UV Detector

METHOD	TIME	FLOW	%A	%B
	Initial	18.00	100	0
	16.00	18.00	90	10
	27.00	18.00	90	10
	27.01	20.00	0	100
	30.00	20.00	0	100
	30.01	18.00	100	0
	32.00	18.00	100	0
LOADING (mg)	36			
NO. OF INJECTIONS	15			
SAMPLE PREPARATION	DMSO			

Properties for Preparative HPLC Purification of 2i

PROJECT NAME	CIN2-C-816-CIN2-X-0007-033-A			
COLUMN NAME & ID	Waters X-Bridge Prep C8 (250mm x 19mm x 5μm)			
COLUMN NO.	PCH-026			
MOBILE PHASE A	0.05% AMMONIUM HYDROXIDE IN WATER			
MOBILE PHASE B	ACETONITRILE:WATER[80:20]			
FLOW RATE(ML/MIN)	12			
INSTRUMENT ID	PHP-03-WATERS 2545 QUARTENERY SYSTEM WITH WATERS 2489 UV Detector.			
METHOD	TIME	FLOW	%A	%B
	Initial	12.00	100	0
	1.00	12.00	92	8
	17.00	12.00	77	23
	22.00	12.00	77	23
	22.01	12.00	0	100
	24.00	12.00	0	100
	24.01	12.00	100	0
	27.00	12.00	100	0
LOADING (mg)	27			
NO. OF INJECTIONS	20			
SAMPLE PREPARATION	DMSO:WATER			

Properties for Preparative HPLC Purification of 3a

PROJECT NAME	CIN-C-375-X-0004-023-a			
COLUMN NAME & ID	Sunfire Prep C18 (150 x 19 mm, 5um)			
COLUMN NO.	PCH-050			
MOBILE PHASE A	0.05% FORMIC ACID IN WATER			
MOBILE PHASE B	ACETONITRILE:METHANOL(50:50)			
FLOW RATE(ML/MIN)	14			
INSTRUMENT ID	PHP-03-WATERS 2545 QUARTENERY SYSTEM WITH WATERS 2489 UV Detector			
METHOD	TIME	FLOW	%A	%B
	Initial	14	95	5
	2	14	90	10
	19.00	14	90	10
	19.01	18	0	100
	22.00	18	0	100
	22.01	18	95	5
	24	14	95	5
AVERAGE LOADING(MG)	13.72			
LOADING (mg)	17			
NO. OF INJECTIONS	15			
SAMPLE PREPARATION	DMSO			

Properties for Preparative HPLC Purification of 3b

PROJECT NAME	CIN-C-375-CIN-X-0006-038-a
COLUMN NAME & ID	Sunfire Prep C18 (150 x 19 mm, 5um)

COLUMN NO.	PCH-050			
MOBILE PHASE A	0.05% FORMIC ACID IN WATER			
MOBILE PHASE B	METHANOL			
FLOW RATE(ML/MIN)	14			
INSTRUMENT ID	PHP-06-WATERS 2545 BINARY SYSTEM WITH WATERS 2489 UV Detector			
METHOD	TIME	FLOW	%A	%B
	Initial	14	100	0
	2.00	14	100	0
	16.00	14	95	5
	17.00	14	95	5
	17.01	18	0	100
	19.00	18	0	100
	19.01	14	100	0
	21.0	14	100	0
AVERAGE LOADING(MG)	13.54			
LOADING (mg)	15			
NO. OF INJECTIONS	22			
SAMPLE PREPARATION	DMSO, WATER			

Properties for Preparative HPLC Purification of 3c

PROJECT NAME	CIN-C-375-X-0005-050-a			
COLUMN NAME & ID	Sunfire Prep C18 (150 x 19 mm, 5um)			
COLUMN NO.	PCH-050			
MOBILE PHASE A	0.05% FORMIC ACID IN WATER			
MOBILE PHASE B	ACETONITRILE:METHANOL:IPA (65:25:10)			
FLOW RATE(ML/MIN)	15			
INSTRUMENT ID	PHP-06-WATERS 2545 BINARY SYSTEM WITH WATERS 2489 UV Detector			
METHOD	TIME	FLOW	%A	%B
	Initial	15	100	0
	2.00	15	100	0
	16.00	15	98	2
	16.01	20	0	100
	18.00	20	0	100
	18.01	15	100	0
	20.00	15	100	0
AVERAGE LOADING(MG)	49.59			
LOADING (mg)	65			
NO. OF INJECTIONS	26			
SAMPLE PREPARATION	WATER			

Properties for Preparative HPLC Purification of 3d

PROJECT NAME	CIN2-C-816-CIN2-X-0091-093			
COLUMN NAME & ID	Shim-Pack GIST C18 (250mm x 20mm x 5μm)			
COLUMN NO.	PCH-053			
MOBILE PHASE A	MERCK WATER			
MOBILE PHASE B	ACETONITRILE			
FLOW RATE(ML/MIN)	20			
INSTRUMENT ID	PHP-06-WATERS 2545 BINARY SYSTEM WITH WATERS 2489 UV Detector			
METHOD	TIME	FLOW	%A	%B
	Initial	20.00	95	5
	16.00	20.00	65	35
	16.01	20.00	0	100
	18.00	20.00	0	100
	18.01	20.00	95	5
	20.00	20.00	95	5
LOADING (mg)	18			
NO. OF INJECTIONS	8			
SAMPLE PREPARATION	ACETONITRILE:MEOH:WATER			

Properties for Preparative HPLC Purification of 3e

PROJECT NAME	CIN2-D-051-CIN2-X-0090-006-a			
COLUMN NAME & ID	Shim-Pack GIST C18 (250mm x 20mm x 5μm)			
COLUMN NO.	PCH-053			
MOBILE PHASE A	WATER			
MOBILE PHASE B	ACETONITRILE			
FLOW RATE(ML/MIN)	20			
INSTRUMENT ID	PHP-06-WATERS 2545 BINARY SYSTEM WITH WATERS 2489 UV Detector			
METHOD	TIME	FLOW	%A	%B
	Initial	20.00	100	0
	1.00	20.00	85	15
	16.00	20.00	85	15
	16.01	20.00	0	100
	18.00	20.00	0	100
	18.01	20.00	100	0
	20.00	20.00	100	0
LOADING (mg)	25			
NO. OF INJECTIONS	11			
SAMPLE PREPARATION	ACN:MEOH:WATER			

Properties for Preparative HPLC Purification of 3f

MOBILE PHASE A	WATER			
MOBILE PHASE B	ACETONITRILE:WATER (70:30)			
FLOW RATE(ML/MIN)	20			
INSTRUMENT ID	PHP-06-WATERS 2545 BINARY SYSTEM WITH WATERS 2489 UV Detector			
METHOD	TIME	FLOW	%A	%B
	TIME	FLOW	%A	%B
	Initial	20.00	100	0
	1.00	20.00	88	12
	16.00	20.00	88	12
	16.01	20.00	0	100
	18.00	20.00	0	100
	18.01	20.00	100	0
LOADING (mg)	34.00			
NO. OF INJECTIONS	9			
SAMPLE PREPARATION	DMSO			

Properties for Preparative HPLC Purification of 4a

PROJECT NAME	CIN-C-375-X-0007-022-A			
COLUMN NAME & ID	Sunfire Prep C18 (150 x 19 mm, 5um)			
COLUMN NO.	PCH-050			
MOBILE PHASE A	0.05% FORMIC ACID IN WATER			
MOBILE PHASE B	METHANOL			
FLOW RATE(ML/MIN)	15			
INSTRUMENT ID	PHP-03-WATERS 2545 QUARTENERY SYSTEM WITH WATERS 2489 UV Detector			
METHOD	TIME	FLOW	%A	%B
	Initial	15	100	0
	1	15	100	0
	15.00	15	92	8
	17.00	15	92	8
	17.01	15	0	100
	20.00	15	0	100

	20.01	15	100	0
	22.00	15	100	0
AVERAGE LOADING(MG)	23.08			
LOADING (mg)	30			
NO. OF INJECTIONS	12			
SAMPLE PREPARATION	WATER			

Properties for Preparative HPLC Purification of 4b

PROJECT NAME	CIN-C-375-CIN-X-0009-037-a			
COLUMN NAME & ID	Sunfire Prep C18 (150 x 19 mm, 5um)			
COLUMN NO.	PCH-050			
MOBILE PHASE A	WATER			
MOBILE PHASE B	METHANOL			
FLOW RATE(ML/MIN)	14			
INSTRUMENT ID	PHP-06-WATERS 2545 BINARY SYSTEM WITH WATERS 2489 UV Detector			
METHOD	TIME	FLOW	%A	%B
	Initial	14	100	0
	1	14	100	0
	16.00	14	97	3
	18.00	14	97	3
	18.01	18	0	100
	21.00	18	0	100
	21.01	18	100	0
	24.00	14	100	0
AVERAGE LOADING(MG)	40.60			
LOADING (mg)	60			
NO. OF INJECTIONS	15			
SAMPLE PREPARATION	WATER			

Properties for Preparative HPLC Purification of 4c

PROJECT NAME	CIN-C-375-X-0008-048-a			
COLUMN NAME & ID	Shim-Pack GIST C18 (250mm x 20mm x 5µm)			
COLUMN NO.	PCH-053			
MOBILE PHASE A	WATER			
MOBILE PHASE B	ACETONITRILE			
FLOW RATE(ML/MIN)	16			
INSTRUMENT ID	PHP-03-WATERS 2545 QUARTENERY SYSTEM WITH WATERS 2489 UV Detector			
METHOD	TIME	FLOW	%A	%B
	Initial	12	100	0
	3	12	100	0
	18.00	12	65	35
	18.01	15	0	100
	20.00	15	0	100
	20.01	15	100	0
	24.00	12	100	0
AVERAGE LOADING(MG)	6.00			
LOADING (mg)	7			
NO. OF INJECTIONS	14			
SAMPLE PREPARATION	DMSO + WATER			

Properties for Preparative HPLC Purification of 4d

PROJECT NAME	CIN2-C-816-CIN2-X-0058-072-C			
COLUMN NAME & ID	Waters Sunfire C18 OBD (250mm x 19mm x 5µm)			
COLUMN NO.	PCH-055			
MOBILE PHASE A	0.05% FA IN WATER			
MOBILE PHASE B	ACETONITRILE :WATER (80:20)			
FLOW RATE(ML/MIN)	8			
INSTRUMENT ID	PHP-08-WATERS 2545 QUATERNARY SYSTEM WITH WATERS 2489 UV Detector			
METHOD	TIME	FLOW	%A	%B
	0.01	8	100	0
	3	8	100	0

	15.00	8	70	30
	15.01	13	0	100
	17.00	13	0	100
	17.01	13	100	0
	22.00	13	100	0
LOADING (mg)	17			
NO. OF INJECTIONS	7			
SAMPLE PREPARATION	WATER			

Properties for Preparative HPLC Purification of 4e

PROJECT NAME	CIN2-D-051-CIN2-X-0060-003-F			
COLUMN NAME & ID	Shim-Pack GIST C18 (250mm x 20mm x 5μm)			
COLUMN NO.	PCH-053			
MOBILE PHASE A	WATER			
MOBILE PHASE B	ACETONITRILE			
FLOW RATE(ML/MIN)	17			
INSTRUMENT ID	PHP-06-WATERS 2545 BINARY SYSTEM WITH WATERS 2489 UV Detector			
METHOD	TIME	FLOW	%A	%B
	Initial	17	100	0
	1.00	17	87	13
	17.00	17	87	13
	17.01	20	0	100
	19.00	20	0	100
	19.01	17	100	0
	21.00	17	100	0
LOADING (mg)	15			
NO. OF INJECTIONS	6			
SAMPLE PREPARATION	DMSO:WATER			

Properties for Preparative HPLC Purification of 4f

PROJECT NAME	CIN2-C-818-CIN2-X-0024-044-e			
COLUMN NAME & ID	Waters X-Bridge Prep C8 (250mm x 19mm x 5μm)			
COLUMN NO.	PCH-026			
MOBILE PHASE A	0.05% NH3 IN WATER			
MOBILE PHASE B	METHANOL			
FLOW RATE(ML/MIN)	13			
INSTRUMENT ID	PHP-06-WATERS 2545 BINARY SYSTEM WITH WATERS 2489 UV Detector			
METHOD	TIME	FLOW	%A	%B
	Initial	18.00	100	0
	14.00	18.00	89	11
	14.01	18.00	89	11
	16.00	20.00	0	100
	16.01	20.00	0	100
	20.00	20.00	100	0
LOADING (mg)	11	18.00	100	0
NO. OF INJECTIONS	9			
SAMPLE PREPARATION	DMSO			

Properties for Preparative HPLC Purification of 4g

PROJECT NAME	CIN2-C-816-CIN2-X-0016-042-A
COLUMN NAME & ID	Sunfire Prep C18 (150 x 19 mm, 5um)
COLUMN NO.	PCH-050
MOBILE PHASE A	0.05% FORMIC ACID IN WATER
MOBILE PHASE B	METHANOL
FLOW RATE(ML/MIN)	10

INSTRUMENT ID	PHP-06-WATERS 2545 BINARY SYSTEM WITH WATERS 2489 UV Detector			
METHOD	TIME	FLOW	%A	%B
	Initial	10.00	100	0
	1.00	10.00	100	0
	15.00	10.00	93	7
	15.01	18.00	0	100
	18.00	18.00	0	100
	18.01	18.00	100	0
	21.00	10.00	100	0
LOADING (mg)	8			
NO. OF INJECTIONS	13			
SAMPLE PREPARATION	WATER			

Properties for Preparative HPLC Purification of 5c

PROJECT NAME	CIN-C-377-CIN-X-0026-063-a			
COLUMN NAME & ID	Gemini 5um NX-C18 110 A (250mm x 20mm x 5µm)			
COLUMN NO.	PCH-045			
MOBILE PHASE A	0.05% NH3 IN WATER			
MOBILE PHASE B	ACETONITRILE			
FLOW RATE(ML/MIN)	15			
INSTRUMENT ID	PHP-06-WATERS 2545 BINARY SYSTEM WITH WATERS 2489 UV Detector			
METHOD	TIME	FLOW	%A	%B
	Initial	Initial	Initial	Initial
	15	15	15	15
	100	100	100	100
	0	0	0	0
	3	3	3	3
	15	15	15	15
	100	100	100	100
	0	0	0	0
AVERAGE LOADING(MG)	20.67			
LOADING (mg)	25			
NO. OF INJECTIONS	12			
SAMPLE PREPARATION	WATER			

Properties for Preparative HPLC Purification of 5d

PROJECT NAME	CIN2-C-818-CIN2-X-0053-097-d			
COLUMN NAME & ID	Xbridge Prep C18 (150 x 19 mm, 5um)			
COLUMN NO.	PCH-051			
MOBILE PHASE A	0.05% AMMONIUM HYDROXIDE IN WATER			
MOBILE PHASE B	ACETONITRILE			
FLOW RATE(ML/MIN)	9			
INSTRUMENT ID	PHP-06-WATERS 2545 BINARY SYSTEM WITH WATERS 2489 UV Detector			
METHOD	TIME	FLOW	%A	%B
	Initial	9.00	95	5
	15.00	9.00	79	21
	15.01	12.00	0	100
	18.00	12.00	0	100
	18.01	12.00	95	5
	21.00	9.00	95	5
LOADING (mg)	13			
NO. OF INJECTIONS	14			
SAMPLE PREPARATION	DMSO:WATER			

Properties for Preparative HPLC Purification of 5e

PROJECT NAME	CIN2-C-818-CIN2-X-0055-078-b			
COLUMN NAME & ID	Shim-Pack GIST C18 (250mm x 20mm x 5µm)			
COLUMN NO.	PCH-053			
MOBILE PHASE A	0.05% FORMIC ACID IN WATER			
MOBILE PHASE B	ACETONITRILE			
FLOW RATE(ML/MIN)	18			
INSTRUMENT ID	PHP-03-WATERS 2545 QUARTENERY SYSTEM WITH WATERS 2489 UV Detector.			
METHOD	TIME	FLOW	%A	%B
	Initial	18.00	100	0
	1.00	18.00	90	10
	16.00	18.00	85	15
	22.00	18.00	85	15
	22.01	18.00	0	100
	25.00	18.00	0	100
	25.01	18.00	100	0
	28.00	18.00	100	0
LOADING (mg)	43			
NO. OF INJECTIONS	7			
SAMPLE PREPARATION	MEOH:WATER:DMSO			

Properties for Preparative HPLC Purification of 5f

PROJECT NAME	CIN2-C-817-CIN2-X-0039-057-G			
COLUMN NAME & ID	Waters X-Bridge Prep C8 (250mm x 19mm x 5µm)			
COLUMN NO.	PCH-026			
MOBILE PHASE A	0.05% AMMONIUM HYDROXIDE IN WATER			
MOBILE PHASE B	ACETONITRILE			
FLOW RATE(ML/MIN)	9			
INSTRUMENT ID	PHP-03-WATERS 2545 QUARTENERY SYSTEM WITH WATERS 2489 UV Detector.			
METHOD	TIME	FLOW	%A	%B
	Initial	9.00	100	0
	2.00	9.00	93	7
	17.00	9.00	88	12
	31.00	9.00	88	12
	31.01	14.00	0	100
	35.00	14.00	0	100
	35.01	14.00	100	0
	38.00	9.00	100	0
LOADING (mg)	10			
NO. OF INJECTIONS	13			
SAMPLE PREPARATION	DMSO+WATER			

Properties for Preparative HPLC Purification of 5g

PROJECT NAME	CIN2-C-817-CIN2-X-0031-039-e			
COLUMN NAME & ID	Shim-Pack GIST C18 (250mm x 20mm x 5µm)			
COLUMN NO.	PCH-053			
MOBILE PHASE A	0.05 % TRIFLURO ACETIC ACID IN WATER			
MOBILE PHASE B	ACETONITRILE			
FLOW RATE(ML/MIN)	18			
INSTRUMENT ID	PHP-06-WATERS 2545 BINARY SYSTEM WITH WATERS 2489 UV Detector			
METHOD	TIME	FLOW	%A	%B
	Initial	18.00	100	0
	16.00	18.00	90	10

	25.00	18.00	90	10
	25.01	22.00	0	100
	27.00	22.00	0	100
	27.01	22.00	100	0
	19.00	18.00	100	0
LOADING (mg)	35			
NO. OF INJECTIONS	24			
SAMPLE PREPARATION	DMSO:WATER			

Properties for Preparative HPLC Purification of 5h

PROJECT NAME	CIN2-C-817-CIN2-X-0038-067-e			
COLUMN NAME & ID	Sunfire Prep C18 (150 x 19 mm, 5um)			
COLUMN NO.	PCH-050			
MOBILE PHASE A	0.05% FORMIC ACID IN WATER			
MOBILE PHASE B	ACETONITRILE:MTBE:THF (85:10:05)			
FLOW RATE(ML/MIN)	12			
INSTRUMENT ID	PHP-06-WATERS 2545 BINARY SYSTEM WITH WATERS 2489 UV Detector			
METHOD	TIME	FLOW	%A	%B
	Initial	12.00	100	0
	1.00	12.00	97	3
	16.00	12.00	95	5
	16.01	18.00	0	100
	18.00	18.00	0	100
	18.01	12.00	100	0
	20.00	12.00	100	0
LOADING (mg)	45			
NO. OF INJECTIONS	13			
SAMPLE PREPARATION	WATER+ACN+MEOH			

Properties for Preparative HPLC Purification of 6b

PROJECT NAME	CIN-C-377-CIN-X-0036-075-a			
COLUMN NAME & ID	Sunfire Prep C18 (150 x 19 mm, 5um)			
COLUMN NO.	PCH-050			
MOBILE PHASE A	WATER			
MOBILE PHASE B	ACETONITRILE			
FLOW RATE(ML/MIN)	16			
INSTRUMENT ID	PHP-06-WATERS 2545 BINARY SYSTEM WITH WATERS 2489UV Detector			
METHOD	TIME	FLOW	%A	%B
	Initial	16	95	5
	2.00	16	89	11
	17.00	16	85	15
	17.01	19	0	100
	20.00	19	0	100
	20.01	19	95	5
	22.00	16	95	5
AVERAGE LOADING(MG)	16.42			
LOADING (mg)	18			
NO. OF INJECTIONS	12			
SAMPLE PREPARATION	WATER			

Properties for Preparative HPLC Purification of 6c

PROJECT NAME	CIN-C-377-CIN-X-0035-069-d			
COLUMN NAME & ID	Gemini 5um NX-C18 110 A (250mm x 20mm x 5µm)			
COLUMN NO.	PCH-045			
MOBILE PHASE A	0.05% NH3 IN WATER			
MOBILE PHASE B	ACETONITRILE			
FLOW RATE(ML/MIN)	16			
INSTRUMENT ID	PHP-06-WATERS 2545 BINARY SYSTEM WITH WATERS 2489UV Detector			
METHOD	TIME	FLOW	%A	%B
	Initial	16	100	0
	1.00	16	85	15

	16.00	16	85	15
	21.0	16	85	15
	21.01	20	0	100
	24.00	20	0	100
	24.01	16	100	0
	26.00	16	100	0
AVERAGE LOADING(MG)	19.95			
LOADING (mg)	22			
NO. OF INJECTIONS	13			
SAMPLE PREPARATION	WATER			

Properties for Preparative HPLC Purification of 6d

PROJECT NAME	CIN2-C-817-CIN2-X-0054-087-Crude			
COLUMN NAME & ID	Shim-Pack GIST C18 (250mm x 20mm x 5µm)			
COLUMN NO.	PCH-053			
MOBILE PHASE A	0.05% FORMIC ACID IN WATER			
MOBILE PHASE B	ACETONITRILE:THF[90:10]			
FLOW RATE(ML/MIN)	7			
INSTRUMENT ID	PHP-03-WATERS 2545 QUARTENERY SYSTEM WITH WATERS 2489 UV Detector.			
METHOD	TIME	FLOW	%A	%B
	Initial	7.00	90	10
	15.00	7.00	55	45
	20.00	7.00	55	45
	20.01	20.00	0	100
	23.00	20.00	0	100
	23.01	20.00	90	10
	26.00	7.00	90	10
LOADING (mg)	22			
NO. OF INJECTIONS	24			
SAMPLE PREPARATION	MEOH:ACN:WATER			

Properties for Preparative HPLC Purification of 6e

PROJECT NAME	CIN2-C-817-CIN2-X-0056-088-Crude			
COLUMN NAME & ID	Shim-Pack GIST C18 (250mm x 20mm x 5µm)			
COLUMN NO.	PCH-053			
MOBILE PHASE A	0.05% FORMIC ACID IN WATER			
MOBILE PHASE B	ACETONITRILE			
FLOW RATE(ML/MIN)	20			
INSTRUMENT ID	PHP-03-WATERS 2545 QUARTENERY SYSTEM WITH WATERS 2489 UV Detector.			
METHOD	TIME	FLOW	%A	%B
	Initial	20.00	100	0
	2.00	20.00	95	5
	16.00	20.00	75	25
	23.00	20.00	75	25
	23.01	20.00	0	100
	25.00	20.00	0	100
	25.01	20.00	100	0
	27.00	20.00	100	0
LOADING (mg)	35			
NO. OF INJECTIONS	6			
SAMPLE PREPARATION	MEOH:WATER			

Properties for Preparative HPLC Purification of 6f

PROJECT NAME	CIN2-D-044-CIN2-X-0063-002-Crude
COLUMN NAME & ID	Sunfire Prep C18 (150 x 19 mm, 5um)
COLUMN NO.	PCH-050
MOBILE PHASE A	0.05% FORMIC ACID IN WATER

MOBILE PHASE B	ACETONITRILE			
FLOW RATE(ML/MIN)	18			
INSTRUMENT ID	PHP-06-WATERS 2545 BINARY SYSTEM WITH WATERS 2489 UV Detector			
METHOD	TIME	FLOW	%A	%B
	Initial	18.00	100	0
	1.00	18.00	93	7
	16.00	18.00	90	10
	23.00	18.00	90	10
	23.01	18.00	0	100
	25.00	18.00	0	100
	25.01	18.00	100	0
	27.00	18.00	100	0
LOADING (mg)	22			
NO. OF INJECTIONS	12			
SAMPLE PREPARATION	MEOH:WATER:ACN:THF			

Properties for Preparative HPLC Purification of 6g

PROJECT NAME	CIN2-C-818-CIN2-X-0061-085-a			
COLUMN NAME & ID	Shim-Pack GIST C18 (250mm x 20mm x 5µm)			
COLUMN NO.	PCH-053			
MOBILE PHASE A	0.05%TRIFLURO ACETIC ACID IN WATER			
MOBILE PHASE B	ACETONITRILE			
FLOW RATE(ML/MIN)	18			
INSTRUMENT ID	PHP-06-WATERS 2545 BINARY SYSTEM WITH WATERS 2489 UV Detector			
METHOD	TIME	FLOW	%A	%B
	Initial	18.00	90	10
	16.00	18.00	84	16
	19.00	18.00	84	16
	19.01	20.00	0	100
	21.00	20.00	0	100
	21.01	20.00	90	10
	24.00	18.00	90	10
LOADING (mg)	55			
NO. OF INJECTIONS	16			
SAMPLE PREPARATION	DMSO:MEOH:WATER			

Properties for Preparative HPLC Purification of 6j

PROJECT NAME	CIN2-D-044-CIN2-X-0064-005-A			
COLUMN NAME & ID	YMC-Actus Triart C18 (250mm x 20mm x 5µm)			
COLUMN NO.	PCH-057			
MOBILE PHASE A	0.05% FORMIC ACID IN WATER			
MOBILE PHASE B	ACETONITRILE			
FLOW RATE(ML/MIN)	17			
INSTRUMENT ID	PHP-11-SHIMADZU LC20AP WITH UV DETECTOR			
METHOD	TIME	FLOW	%A	%B
	0	17	100	0
	23	17	70	30
	23.01	17	0	100
	26.00	20	0	100
	26.01	20	100	0
	29.00	17	100	0

LOADING (mg)	40
NO. OF INJECTIONS	8
SAMPLE PREPARATION	ACETONITRILE:MEOH:WATER

Properties for Preparative HPLC Purification of 7b

PROJECT NAME	CIN-C-377-CIN-X-0039-081-a			
COLUMN NAME & ID	Shim-Pack GIST C18 (250mm x 20mm x 5µm)			
COLUMN NO.	PCH-053			
MOBILE PHASE A	WATER			
MOBILE PHASE B	ACETONITRILE			
FLOW RATE(ML/MIN)	15			
INSTRUMENT ID	PHP-03-WATERS 2545 QUARTENERY SYSTEM WITH WATERS, 2489 UV Detector			
METHOD	TIME	FLOW	%A	%B
	Initial	15	98	2
	2.00	15	87	13
	23.00	15	87	13
	23.01	20	0	100
	26.00	20	0	100
	26.01	20	98	2
	28.00	15	98	2
AVERAGE LOADING(MG)	17.13			
LOADING (mg)	27			
NO. OF INJECTIONS	12			
SAMPLE PREPARATION	DMSO			

Properties for Preparative HPLC Purification of 7c

PROJECT NAME	CIN-C-377-CIN-X-0038-062-c			
COLUMN NAME & ID	Sunfire Prep C18 (150 x 19 mm, 5um)			
COLUMN NO.	PCH-050			
MOBILE PHASE A	MILIQ- WATER			
MOBILE PHASE B	ACETONITRILE			
FLOW RATE(ML/MIN)	17			
INSTRUMENT ID	PHP-03-WATERS 2545 QUARTENERY SYSTEM WITH WATERS, 2489 UV Detector			
METHOD	TIME	FLOW	%A	%B
	Initial	17	95	5
	1.00	17	88	12
	19.00	17	88	12
	19.01	17	0	100
	21.00	17	0	100
	21.01	17	95	5
	23.00	17	95	5
AVERAGE LOADING(MG)	26.00			
LOADING (mg)	16			
NO. OF INJECTIONS	16			
SAMPLE PREPARATION	WATER			

Properties for Preparative HPLC Purification of 8c

PROJECT NAME	CIN-C-377-CIN-X-0029-044-a			
COLUMN NAME & ID	Sunfire Prep C18 (150 x 19 mm, 5um)			
COLUMN NO.	PCH-050			
MOBILE PHASE A	0.05% FORMIC ACID IN WATER			
MOBILE PHASE B	METHANOL			
FLOW RATE(ML/MIN)	15			
INSTRUMENT ID	PHP-03-WATERS 2545 QUARTENERY SYSTEM WITH WATERS 2489 UV Detector			
METHOD	TIME	FLOW	%A	%B
	Initial	15	95	5
	14.00	15	90	10
	14.01	15	0	100
	17.00	15	0	100
	17.01	15	95	5
	21.00	15	95	5
AVERAGE LOADING(MG)	40.90			
LOADING (mg)	60			
NO. OF INJECTIONS	10			
SAMPLE PREPARATION	WATER			

Properties for Preparative HPLC Purification of 8e

PROJECT NAME	CIN2-D-044-CIN2-X-0087-031-d			
COLUMN NAME & ID	Shim-Pack GIST C18 (250mm x 20mm x 5μm)			
COLUMN NO.	PCH-053			
MOBILE PHASE A	WATER			
MOBILE PHASE B	ACETONITRILE			
FLOW RATE(ML/MIN)	20			
INSTRUMENT ID	PHP-06-WATERS 2545 BINARY SYSTEM WITH WATERS 2489 UV Detector			
METHOD	TIME	FLOW	%A	%B
	Initial	20.00	100	0
	1.00	20.00	88	12
	26.00	20.00	88	12
	26.01	25.00	0	100
	28.00	25.00	0	100
	28.01	23.00	100	0
	31.00	20.00	100	0
LOADING (mg)	30			
NO. OF INJECTIONS	11			
SAMPLE PREPARATION	DMSO			

Properties for Preparative HPLC Purification of 10b

PROJECT NAME	CIN-C-376-CIN-X-0057-063-A			
COLUMN NAME & ID	Chiralpak IG (250mm x 50mm x 5μm)			
COLUMN NO.	PCH-049			
MOBILE PHASE A	LIQUID. CO2			
MOBILE PHASE B	*0.1% METHANOLIC AMMONIA IN METHANOL:ACETONITRILE(50:50*			
FLOW RATE(ML/MIN)	150			
INSTRUMENT ID	WATERS SFC 350 WITH 2489 UV Detector			
METHOD	TIME	FLOW	%A	%B
	0.01	*150*	*55*	*45*
	45	*150*	*55*	*45*
AVERAGE LOADING(MG)	*10*			
LOADING (mg)	*12*			
NO. OF INJECTIONS	20			
SAMPLE PREPARATION	*METHANOL:DICHLOROMETHANE(30-70)*			

Properties for Preparative HPLC Purification of 13a

PROJECT NAME	CIN2-D-044-CIN2-X-0076-009-c			
COLUMN NAME & ID	Sunfire Prep C18 (150 x 19 mm, 5um)			
COLUMN NO.	PCH-050			
MOBILE PHASE A	0.05 % FORMIC ACID IN WATER			
MOBILE PHASE B	ACETONITRILE			
FLOW RATE(ML/MIN)	18			
INSTRUMENT ID	PHP-06-WATERS 2545 BINARY SYSTEM WITH WATERS 2489 UV Detector			
METHOD	TIME	FLOW	%A	%B
	Initial	18.00	100	0
	15.00	18.00	90	10
	21.00	18.00	90	10
	21.01	18.00	0	100
	23.00	18.00	0	100
	23.01	18.00	100	0
	25.00	18.00	100	0
LOADING (mg)	21			
NO. OF INJECTIONS	6			
SAMPLE PREPARATION	ACN:MEOH:H2O			

Properties for Preparative HPLC Purification of 15a

PROJECT NAME	CIN2-D-044-CIN-X-0099-011-b			
COLUMN NAME & ID	Sunfire Prep C18 (150 x 19 mm, 5um)			
COLUMN NO.	PCH-050			
MOBILE PHASE A	0.05 % FORMIC ACID IN MERCK WATER			
MOBILE PHASE B	ACETONITRILE			
FLOW RATE(ML/MIN)	18			
INSTRUMENT ID	PHP-06-WATERS 2545 BINARY SYSTEM WITH WATERS 2489 UV Detector			
METHOD	TIME	FLOW	%A	%B
	Initial	18.00	100	0
	15.00	18.00	82	18
	15.10	18.00	0	100
	18.00	18.00	0	100
	18.10	18.00	100	0
	19.00	18.00	100	0
LOADING (mg)	42			
NO. OF INJECTIONS	13			
SAMPLE PREPARATION	ACN:MEOH:H2O			

Properties for Preparative HPLC Purification of 15c

PROJECT NAME	CIN2-D-051-CIN2-X-0100-032-I			
COLUMN NAME & ID	Sunfire Prep C18 (150 x 19 mm, 5um)			
COLUMN NO.	PCH-050			
MOBILE PHASE A	0.05% FORMIC ACID IN WATER			
MOBILE PHASE B	ACETONITRILE			
FLOW RATE(ML/MIN)	18			
INSTRUMENT ID	PHP-06-WATERS 2545 BINARY SYSTEM WITH WATERS 2489 UV Detector			
METHOD	TIME	FLOW	%A	%B
	Initial	18.00	100	0
	1.00	18.00	100	0
	16.00	18.00	93	7
	16.01	18.00	0	100
	18.00	18.00	0	100
	18.01	18.00	100	0
	20.00	18.00	100	0
LOADING (mg)	13			
NO. OF INJECTIONS	10			
SAMPLE PREPARATION	ACN:MEOH:WATER:DMSO			

Properties for Preparative HPLC Purification of 17b

PROJECT NAME	CIN-C-375-CIN-X-0072-036-a			
COLUMN NAME & ID	Waters X-Bridge Prep C8 (250mm x 19mm x 5µm)			
COLUMN NO.	PCH-026			
MOBILE PHASE A	0.05% NH3 IN WATER			
MOBILE PHASE B	ACETONITRILE			
FLOW RATE(ML/MIN)	9			
INSTRUMENT ID	PHP-03-WATERS 2545 QUARTENERY SYSTEM WITH WATERS 2489 UV Detector.			
METHOD	TIME	FLOW	%A	%B
	Initial	9	100	0
	2	9	95	5
	20.00	9	85	15
	20.01	15	0	100
	22.00	15	0	100
	22.01	15	100	0

	24.00	12	100	0
AVERAGE LOADING(MG)	32.32			
LOADING (mg)	42			
NO. OF INJECTIONS	20			
SAMPLE PREPARATION	WATER			

PROJECT NAME	CIN-C-375-CIN-X-0072-036-b			
COLUMN NAME & ID	Chiralpak IG (250mm x 50mm x 5µm)			
COLUMN NO.	PCH-023			
MOBILE PHASE A	LIQ. CO2			
MOBILE PHASE B	0.1% METHANOLIC AMMONIA IN METHANOL:ACETONITRILE(50:50)			
FLOW RATE(ML/MIN)	150			
INSTRUMENT ID	WATERS SFC 350 WITH 2489 UV Detector			
METHOD	TIME	FLOW	%A	%B
	0.01	150	55	45
	20	150	55	45
AVERAGE LOADING(MG)	10			
LOADING (mg)	15			
NO. OF INJECTIONS	20			
SAMPLE PREPARATION	METHANOL:DICHLOROMETHANE			

Properties for Preparative HPLC Purification of 17c

PROJECT NAME	CIN-C-375-X-0071-049-B			
COLUMN NAME & ID	Shim-Pack GIST C18 (250mm x 20mm x 5µm)			
COLUMN NO.	PCH-053			
MOBILE PHASE A	WATER			
MOBILE PHASE B	ACETONITRILE			
FLOW RATE(ML/MIN)	20			
INSTRUMENT ID	PHP-03-WATERS 2545 QUARTENERY SYSTEM WITH WATERS 2489 UV Detector			
METHOD	TIME	FLOW	%A	%B
	Initial	20	100	0
	2	20	95	5
	16.00	20	83	17
	18.00	20	83	17
	18.01	21	0	100
	21.00	21	0	100
	21.01	21	100	0
	24.00	20	100	0
AVERAGE LOADING(MG)	17.67			
LOADING (mg)	27			
NO. OF INJECTIONS	6			
SAMPLE PREPARATION	DMSO			

Properties for Preparative HPLC Purification of 18d

PROJECT NAME	CIN2-C-816-CIN2-X-0093-080-A			
COLUMN NAME & ID	Sunfire Prep C18 (150 x 19 mm, 5um)			
COLUMN NO.	PCH-050			
MOBILE PHASE A	0.05% FORMIC ACID IN WATER			
MOBILE PHASE B	ACETONITRILE			
FLOW RATE(ML/MIN)	14			
INSTRUMENT ID	PHP-06-WATERS 2545 BINARY SYSTEM WITH WATERS 2489 UV Detector			
METHOD	TIME	FLOW	%A	%B
	Initial	14.00	100	0
	2.00	14.00	100	0
	14.00	14.00	93	7
	14.01	20.00	0	100
	17.00	20.00	0	100
	17.01	20.00	100	0

	20.00	14.00	100	0
LOADING (mg)	25			
NO. OF INJECTIONS	10			
SAMPLE PREPARATION	MEOH:WATER			

Properties for Preparative HPLC Purification of 18e

PROJECT NAME	CIN2-D-051-CIN2-X-0092-004-b			
COLUMN NAME & ID	Shim-Pack GIST C18 (250mm x 20mm x 5µm)			
COLUMN NO.	PCH-053			
MOBILE PHASE A	MERCK WATER			
MOBILE PHASE B	ACETONITRILE			
FLOW RATE(ML/MIN)	18			
INSTRUMENT ID	PHP-06-WATERS 2545 BINARY SYSTEM WITH WATERS 2489 UV Detector			
METHOD	TIME	FLOW	%A	%B
	Initial	18.00	100	0
	1.00	18.00	89	11
	35.00	18.00	89	11
	35.01	20.00	0	100
	39.00	20.00	0	100
	39.01	20.00	100	0
	42.00	18.00	100	0
LOADING (mg)	30			
NO. OF INJECTIONS	12			
SAMPLE PREPARATION	ACN:MEOH:WATER			

Properties for Preparative HPLC Purification of 18f

PROJECT NAME	CIN2-D-051-CIN2-X-0097-013-b			
COLUMN NAME & ID	Xbridge C8 (250 x 19 mm x 5um)			
COLUMN NO.	PCH-058			
MOBILE PHASE A	0.05% AMMONIUM HYDROXIDE IN WATER			
MOBILE PHASE B	METHANOL			
FLOW RATE(ML/MIN)	11			
INSTRUMENT ID	PHP-11-SHIMADZU LC20AP WITH UV DETECTOR			
METHOD	TIME	FLOW	%A	%B
	Initial	11	100	0
	19.00	11	60	40
	19.01	11	0	100
	21.00	11	0	100
	21.01	11	100	0
	25.00	11	100	0
LOADING (mg)	30			
NO. OF INJECTIONS	14			
SAMPLE PREPARATION	MEOH:ACN:WATER			

Properties for Preparative HPLC Purification of 19b

PROJECT NAME	CIN-C-377-CIN-X-0033-074-b
COLUMN NAME & ID	Sunfire Prep C18 (150 x 19 mm, 5um)
COLUMN NO.	PCH-050
MOBILE PHASE A	WATER
MOBILE PHASE B	ACETONITRILE
FLOW RATE(ML/MIN)	16
INSTRUMENT ID	PHP-06-WATERS 2545 BINARY SYSTEM WITH WATERS 2489UV Detector

METHOD	TIME	FLOW	%A	%B
	Initial	16	100	0
	2.00	16	100	0
	25.00	16	80	20
	25.01	19	0	100
	28.00	19	0	100
	28.01	19	100	0
	30.00	17	100	0
AVERAGE LOADING(MG)	14:57			
LOADING (mg)	22			
NO. OF INJECTIONS	7			
SAMPLE PREPARATION	MEOH:WATER			

Properties for Preparative HPLC Purification of 19c

PROJECT NAME	CIN-C-377-CIN-X-0032-068-a			
COLUMN NAME & ID	Sunfire Prep C18 (150 x 19 mm, 5um)			
COLUMN NO.	PCH-050			
MOBILE PHASE A	WATER			
MOBILE PHASE B	ACETONITRILE			
FLOW RATE(ML/MIN)	17			
INSTRUMENT ID	PHP-06-WATERS 2545 BINARY SYSTEM WITH WATERS 2489UV Detector			
METHOD	TIME	FLOW	%A	%B
	Initial	17	100	0
	1.00	17	92	8
	15.00	17	92	8
	15.01	17	0	100
	18.00	17	0	100
	18.01	17	100	0
	21.00	17	100	0
AVERAGE LOADING(MG)	9.89			
LOADING (mg)	17			
NO. OF INJECTIONS	9			
SAMPLE PREPARATION	MEOH:WATER			

Properties for Preparative HPLC Purification of 23d

PROJECT NAME	CIN2-D-051-CIN2-X-0072-017-2			
COLUMN NAME & ID	Shim-Pack GIST C18 (250mm x 20mm x 5μm)			
COLUMN NO.	PCH-053			
MOBILE PHASE A	0.05% FORMIC ACID IN WATER			
MOBILE PHASE B	ACETONITRILE			
FLOW RATE(ML/MIN)	18			
INSTRUMENT ID	PHP-06-WATERS 2545 BINARY SYSTEM WITH WATERS 2489 UV Detector			
METHOD	TIME	FLOW	%A	%B
	Initial	18	90	10
	16.00	18	80	20
	18.00	18	80	20
	18.01	20	0	100
	20.00	20	0	100
	20.01	18	90	10
	22.00	18	90.10	20
LOADING (mg)	17			
NO. OF INJECTIONS	10			
SAMPLE PREPARATION	DMSO			

Properties for Preparative HPLC Purification of 23e

PROJECT NAME	CIN2-C-818-CIN2-X-0073-090			
COLUMN NAME & ID	Shim-Pack GIST C18 (250mm x 20mm x 5μm)			
COLUMN NO.	PCH-053			
MOBILE PHASE A	0.05% FORMIC ACID IN WATER			
MOBILE PHASE B	ACETONITRILE			
FLOW RATE(ML/MIN)	18			
INSTRUMENT ID	PHP-06-WATERS 2545 BINARY SYSTEM WITH WATERS 2489 UV Detector			
METHOD	TIME	FLOW	%A	%B

	Initial	18	80	20
	16.00	18	75	25
	17.00	18	75	25
	17.01	20	0	100
	19.00	20	0	100
	19.01	18	80	20
	21.00	18	80	20
LOADING (mg)	22			
NO. OF INJECTIONS	14			
SAMPLE PREPARATION	DMSO			

References

(1) Faurel-Paul, E.; Yoshida, K.; Sépulcre, M.; Dhimane, H.; Le Merrer, Y. Synthesis of Three Regioisomeric (7-Methoxychromenonyl)methyl Guanosine 5'-Phosphates. *Synth. Commun.* **2009**, *39* (3), 459-474. DOI: 10.1080/00397910802379975.
(2) Sun, Q.; Edathil, J. P.; Wu, R.; Smidansky, E. D.; Cameron, C. E.; Peterson, B. R. One-Pot Synthesis of Nucleoside 5'-Triphosphates from Nucleoside 5'-*H*-Phosphonates. *Org. Lett.* **2008**, *10* (9), 1703-1706. DOI: 10.1021/ol8003029.
(3) Ross, B. S.; Ganapati Reddy, P.; Zhang, H.-R.; Rachakonda, S.; Sofia, M. J. Synthesis of Diastereomerically Pure Nucleotide Phosphoramidates. *J. Org. Chem.* **2011**, *76* (20), 8311-8319. DOI: 10.1021/jo201492m.
(4) Abramson, J.; Adler, J.; Dunger, J.; Evans, R.; Green, T.; Pritzel, A.; Ronneberger, O.; Willmore, L.; Ballard, A. J.; Bambrick, J. Accurate structure prediction of biomolecular interactions with AlphaFold 3. *Nature* **2024**, *630* (8016), 493-500.
(5) Jurrus, E.; Engel, D.; Star, K.; Monson, K.; Brandi, J.; Felberg, L. E.; Brookes, D. H.; Wilson, L.; Chen, J.; Liles, K. Improvements to the APBS biomolecular solvation software suite. *Protein science* **2018**, *27* (1), 112-128.
(6) O'Boyle, N. M.; Banck, M.; James, C. A.; Morley, C.; Vandermeersch, T.; Hutchison, G. R. Open Babel: An open chemical toolbox. *Journal of cheminformatics* **2011**, *3* (1), 33.
(7) Zhang, Y.; Sanner, M. F. AutoDock CrankPep: combining folding and docking to predict protein–peptide complexes. *Bioinformatics* **2019**, *35* (24), 5121-5127.
(8) Forli, S.; Huey, R.; Pique, M. E.; Sanner, M. F.; Goodsell, D. S.; Olson, A. J. Computational protein–ligand docking and virtual drug screening with the AutoDock suite. *Nature protocols* **2016**, *11* (5), 905-919.
(9) Koes, D. R.; Baumgartner, M. P.; Camacho, C. J. Lessons learned in empirical scoring with smina from the CSAR 2011 benchmarking exercise. *Journal of chemical information and modeling* **2013**, *53* (8), 1893-1904.
(10) Kouba, T.; Dubankova, A.; Drncova, P.; Donati, E.; Vidossich, P.; Speranzini, V.; Pflug, A.; Huchting, J.; Meier, C.; De Vivo, M. Direct observation of backtracking by influenza A and B polymerases upon consecutive incorporation of the nucleoside analog T1106. *Cell reports* **2023**, *42* (1).
(11) Wandzik, J. M.; Kouba, T.; Karuppasamy, M.; Pflug, A.; Drncova, P.; Provaznik, J.; Azevedo, N.; Cusack, S. A structure-based model for the complete transcription cycle of influenza polymerase. *Cell* **2020**, *181* (4), 877-893. e821.
(12) Schrodinger, LLC. The PyMOL Molecular Graphics System, Version 1.8. 2015.
(13) Jia, X.; Jing, X.; Li, M.; Gao, M.; Zhong, Y.; Li, E.; Liu, Y.; Li, R.; Yao, G.; Liu, Q. An adenosine analog shows high antiviral potency against coronavirus and arenavirus mainly through an unusual base pairing mode. *Nature Communications* **2024**, *15* (1), 10750.
(14) Kouba, T.; Vogel, D.; Thorkelsson, S. R.; Quemin, E. R.; Williams, H. M.; Milewski, M.; Busch, C.; Günther, S.; Grünewald, K.; Rosenthal, M. Conformational changes in Lassa virus L protein associated with promoter binding and RNA synthesis activity. *Nature Communications* **2021**, *12* (1), 7018.

Copies of Analytical Spectra (HPLC, NMR)

For analytical data, please refer to the following pages. First, the LCMS and HPLC traces of the carbobicyclic nucleoside analogues are presented, followed by the NMR spectra. The next section displays the NMR spectra for key intermediates.

HPLC and LCMS Traces for Carbobicyclic Nucleoside Analogues

HPLC and LCMS Traces for Compound 2d

o2h discovery
Ahmedabad, Gujarat
India

LCMS Analysis Report

Sample Name:	CIN2-C-816-CIN2-X-0057-064-A		Injection Id 66260
Sample Type:	Unknown	Acquired By:	LCMS-05
Vial:	2:F,2	Sample Set Name:	29112024_UCH_090_FD
Injection #:	1	Acq. Method Set:	o2h_LCMS_Method_A_SLOW
Injection Volume:	2.00 ul	Processing Method	O2H_LCMS_03_00,
Run Time:	4.0 Minutes	Channel Name:	MS TIC, 254.0nm, 210.0nm
Project Name:	2024\LCMS-05_NOV-2024	Proc. Chnl. Descr.:	PDA 254.0 nm Blank Subtracted
Date Acquired:	29-11-2024 10:30:06 IST		
Date Processed:	29-11-2024 10:40:42 IST, 29-11-2024 10:40:50 IST, 29-11-2024 10:44:07 IST		
Column:	X-BRIDGE C18 2.1X50mm 2.5um		

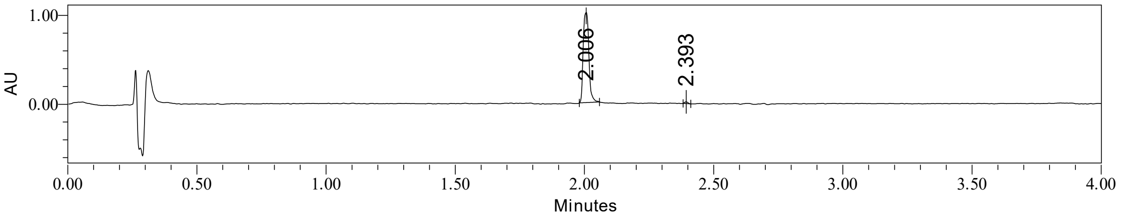

Channel Name 210.0nm; Channel PDA Spectrum

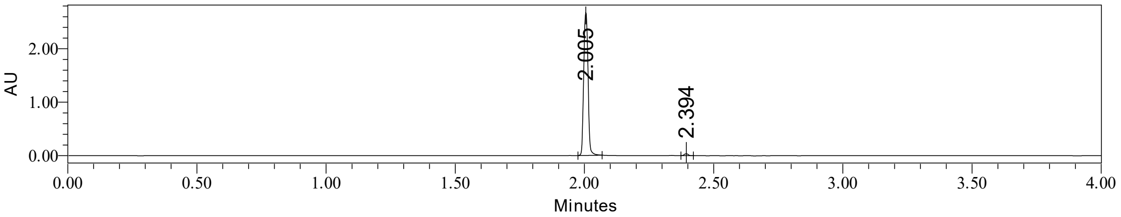

Channel Name 254.0nm; Channel PDA Spectrum

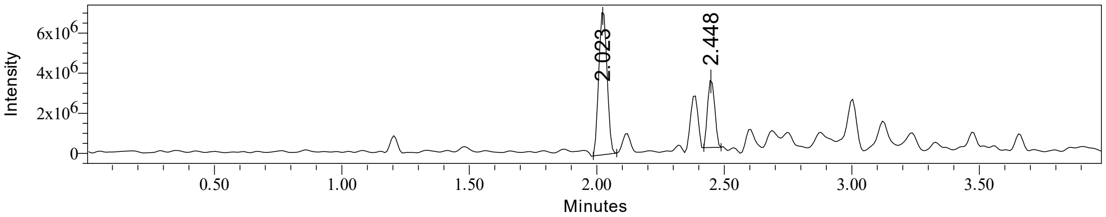

Channel Name MS TIC; Channel QDa 1: MS Scan

Peak Results
Channel: PDA Spectrum

	RT	Base Peak (m/z)	Height	Area	% Area	Channel	Channel Name
1	2.005		2678494	3107891	98.76	PDA Spectrum	254.0nm
2	2.006		1016732	1542092	98.90	PDA Spectrum	210.0nm
3	2.393		19118	17135	1.10	PDA Spectrum	210.0nm
4	2.394		35444	39055	1.24	PDA Spectrum	254.0nm

HPLC and LCMS Traces for Compound 2d

Peak Results
Channel: QDa 1: MS Scan

	RT	Base Peak (m/z)	Height	Area	% Area	Channel	Channel Name
1	2.023	280.96	7139362	16149009	71.00	QDa 1: MS Scan	MS TIC
2	2.448	325.05	3431981	6594746	29.00	QDa 1: MS Scan	MS TIC

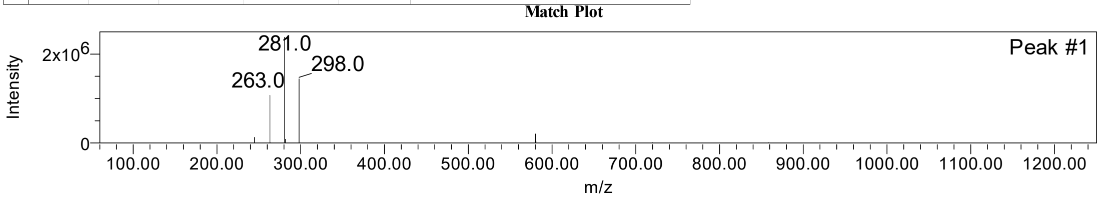

Base Peak 280.96 Channel Description 1: QDa Positive(+) Scan (60.00-1240.00)Da, Centroid, CV=10 - AVG (2.6:4.0;2.1:2.3;0.1:1.7) x 20.000 Th: 0.001 - AVG (2.1:2.2;2.4:2.4;2.5:4.0) x 20.000 Th: 0.001 Retention Time 2.023

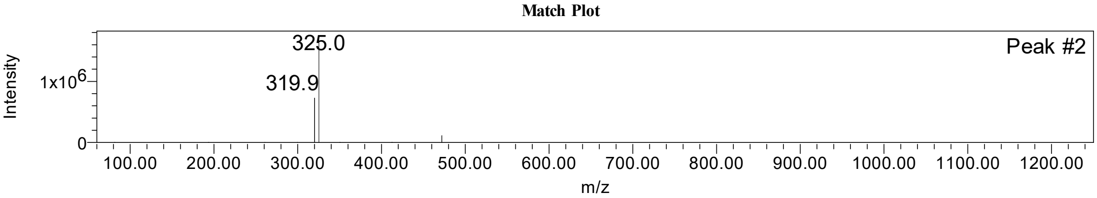

Base Peak 325.05 Channel Description 1: QDa Positive(+) Scan (60.00-1240.00)Da, Centroid, CV=10 - AVG (2.6:4.0;2.1:2.3;0.1:1.7) x 20.000 Th: 0.001 - AVG (2.1:2.2;2.4:2.4;2.5:4.0) x 20.000 Th: 0.001 Retention Time 2.448

HPLC and LCMS Traces for Compound 2d

o2h discovery
Ahmedabad, Gujarat
India

HPLC Analysis Report

Sample name: CIN2-C-816-CIN2-X-0057-064-A
Location: P2-E9
Injection: 1 of 1
Injection volume: 10.000
Project Name HPLC_07_NOV-2024
Date Acquired: 2024-11-29 14:16:31+05:30
Date Processed: 2024-11-29 14:54:30+05:30

Instrument Name HPLC-07
Acq. method: o2h_HPLC_Method-D.amx
Processing method: *3D UV Quantitative_DefaultMethod.pmx
Column: XBridge C18 150x4.6mm, 3.5um

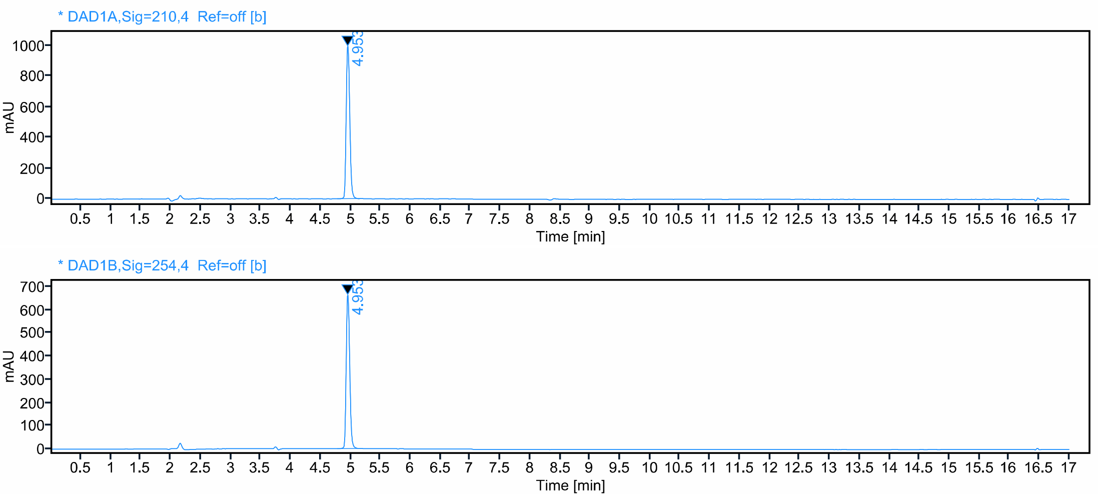

Signal: * DAD1A,Sig=210,4 Ref=off [b]

RT [min]	Height	Area	Area%
4.953	991	4017	100.00

Signal: * DAD1B,Sig=254,4 Ref=off [b]

RT [min]	Height	Area	Area%
4.953	661	2633	100.00

HPLC and LCMS Traces for Compound 2e

o2h discovery
Ahmedabad, Gujarat
India

LCMS Analysis Report

Sample Name:	CIN2-C-818-CIN2-X-0059-053		Injection Id 34058
Sample Type:	Unknown	Acquired By:	LCMS-05
Vial:	1:A,3	Sample Set Name:	15112024_UCH_090_FD_02
Injection #:	1	Acq. Method Set:	o2h_LCMS_Method_A
Injection Volume:	2.00 ul	Processing Method	MASS_000, O2H_LCMS_02_00,
Run Time:	4.0 Minutes	Channel Name:	MS TIC, 254.0nm, 210.0nm
Project Name:	2024\LCMS-05_NOV-2024	Proc. Chnl. Descr.:	QDa 3: MS Scan MS TIC,
Date Acquired:	16-11-2024 10:17:20 IST		
Date Processed:	16-11-2024 10:22:34 IST, 16-11-2024 10:22:38 IST, 16-11-2024 10:23:02 IST, 16-11-2024		
Column:	X-BRIDGE C18 2.1X50mm 2.5um		

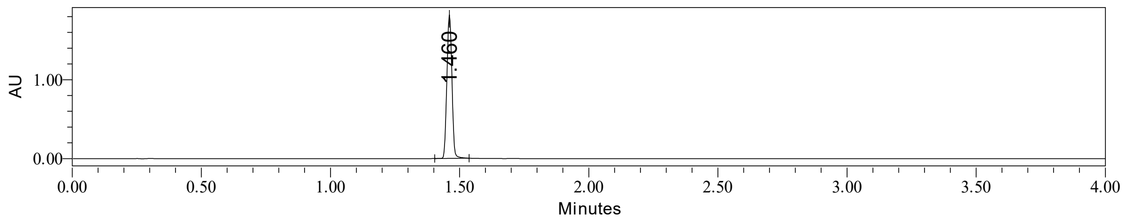

Channel Name 254.0nm; Channel PDA Spectrum

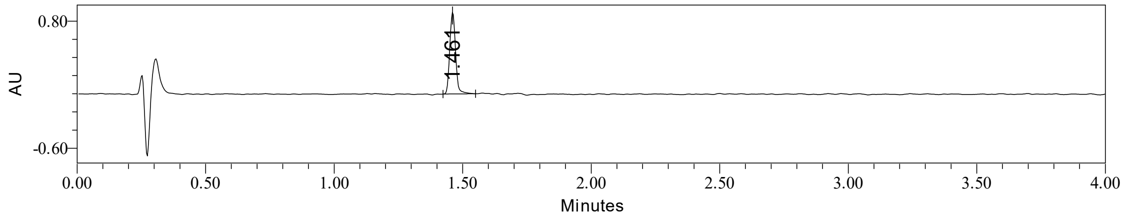

Channel Name 210.0nm; Channel PDA Spectrum

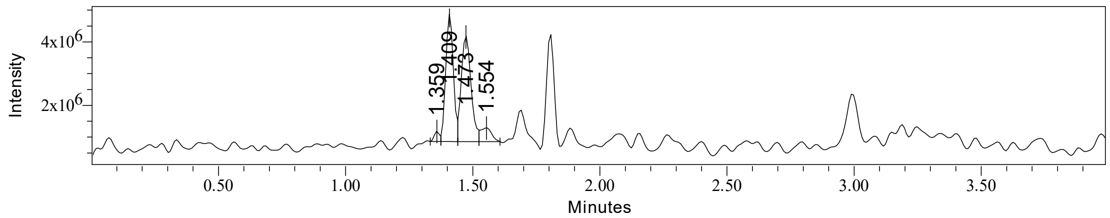

Channel Name MS TIC; Channel QDa 1: MS Scan

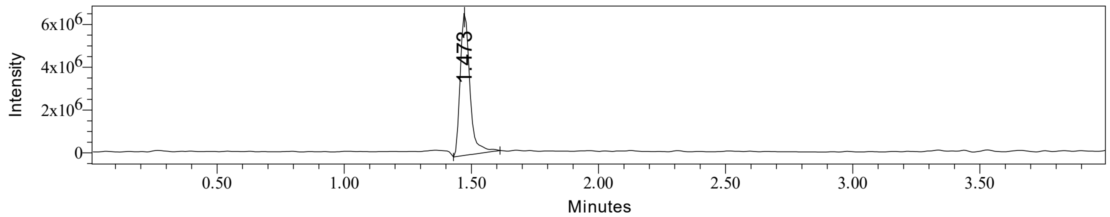

Channel Name MS TIC; Channel QDa 3: MS Scan

HPLC and LCMS Traces for Compound 2e

Peak Results
Channel: PDA Spectrum

	RT	Base Peak (m/z)	Height	Area	% Area	Channel	Channel Name
1	1.460		1820065	2460734	100.00	PDA Spectrum	254.0nm
2	1.461		899054	1413082	100.00	PDA Spectrum	210.0nm

Peak Results
Channel: QDa 1: MS Scan

	RT	Base Peak (m/z)	Height	Area	% Area	Channel	Channel Name
1	1.359	300.19	327937	454755	2.39	QDa 1: MS Scan	MS TIC
2	1.409	332.11	4033368	8389909	44.04	QDa 1: MS Scan	MS TIC
3	1.473	265.06	3303238	8875886	46.59	QDa 1: MS Scan	MS TIC
4	1.554	276.98	434998	1329959	6.98	QDa 1: MS Scan	MS TIC

Peak Results
Channel: QDa 3: MS Scan

	RT	Base Peak (m/z)	Height	Area	% Area	Channel	Channel Name
1	1.473	309.13	6594556	17633214	100.00	QDa 3: MS Scan	MS TIC

Match Plot

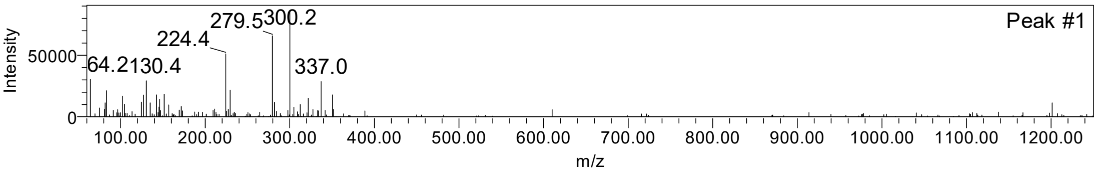

Base Peak 300.19 Channel Description 1: QDa Positive(+) Scan (60.00-1250.00)Da, Centroid, CV=10 - AVG (0.0:1.2;1.6:4.0) x 30.000 Th: 0.010 Retention Time 1.359

Match Plot

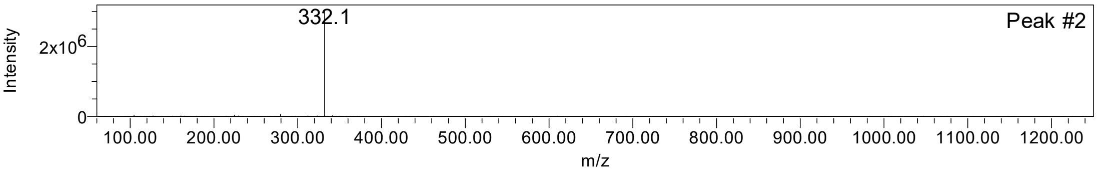

Base Peak 332.11 Channel Description 1: QDa Positive(+) Scan (60.00-1250.00)Da, Centroid, CV=10 - AVG (0.0:1.2;1.6:4.0) x 30.000 Th: 0.010 Retention Time 1.409

Match Plot

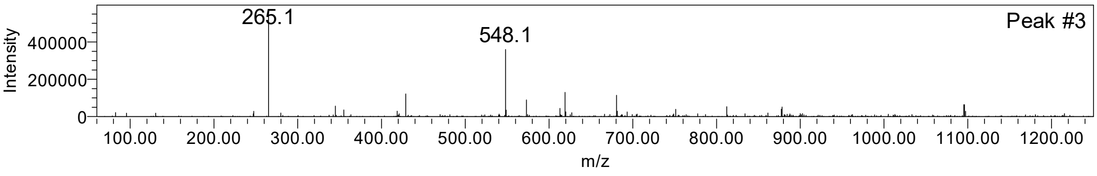

Base Peak 265.06 Channel Description 1: QDa Positive(+) Scan (60.00-1250.00)Da, Centroid, CV=10 - AVG (0.0:1.2;1.6:4.0) x 30.000 Th: 0.010 Retention Time 1.473

HPLC and LCMS Traces for Compound 2e

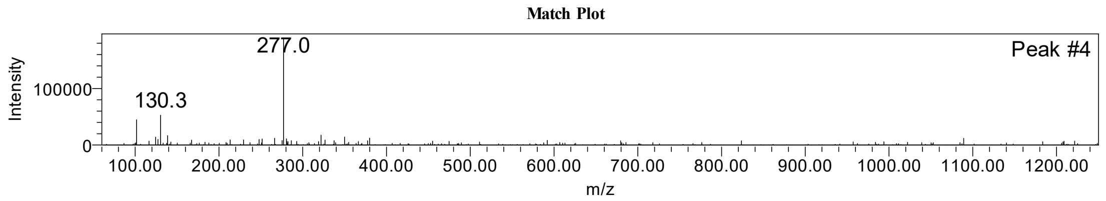

Base Peak 276.98 Channel Description 1: QDa Positive(+) Scan (60.00-1250.00)Da, Centroid, CV=10 - AVG (0.0:1.2;1.6:4.0) x 30.000 Th: 0.010 Retention Time 1.554

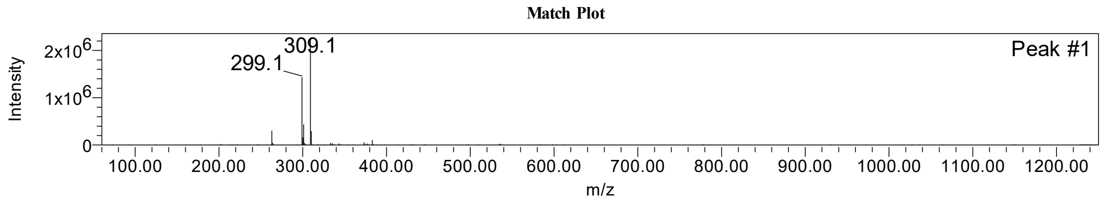

Base Peak 309.13 Channel Description 3: QDa Negative(-) Scan (60.00-1250.00)Da, Centroid, CV=10 - AVG (0.0:1.3;1.7:4.0) x 30.000 Th: 0.010 Retention Time 1.473

HPLC and LCMS Traces for Compound 2e

o2h discovery
Ahmedabad, Gujarat
India

HPLC Analysis Report

Sample name: CIN2-C-818-CIN2-X-0059-053
Location: P2-A1
Injection: 1 of 1
Injection volume: 10.000
Project Name HPLC-10-NOV-2024
Date Acquired: 2024-11-16 10:52:18+05:30
Date Processed: 2024-11-16 12:13:57+05:30

Instrument Name HPLC-10
Acq. method: o2h_HPLC_Method-A.amx
Processing method: *3D UV Quantitative_DefaultMethod.pmx
Column: SUNFIRE C18 150x4.6mm,3.5um

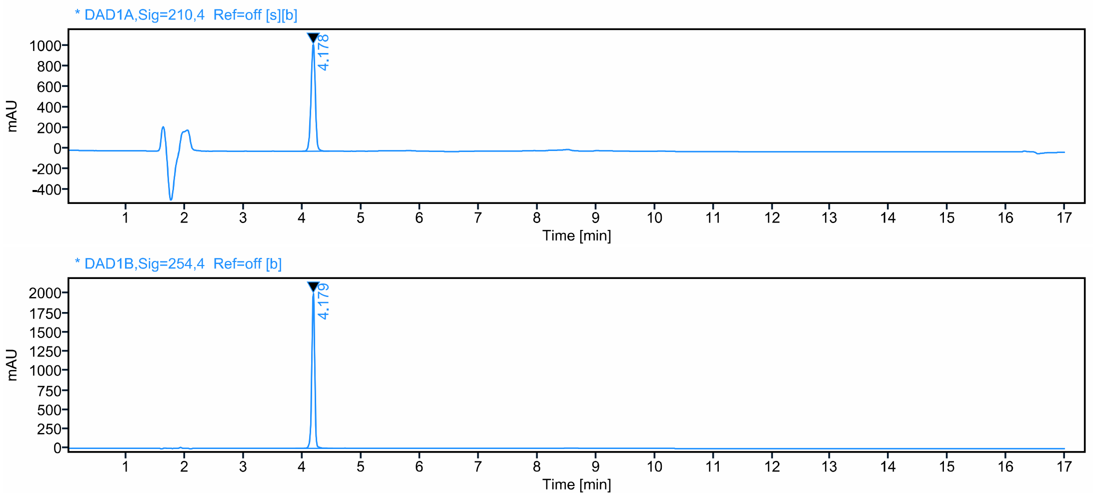

Signal: * DAD1A,Sig=210,4 Ref=off [s][b]

RT [min]	Height	Area	Area%
4.178	1030	5171	100.00

Signal: * DAD1B,Sig=254,4 Ref=off [b]

RT [min]	Height	Area	Area%
4.179	2005	6870	100.00

HPLC and LCMS Traces for Compound 2f

o2h discovery
Ahmedabad, Gujarat
India

LCMS Analysis Report

Sample Name:	CIN2-C-818-CIN2-X-0009-042-b		
Sample Type:	Unknown	Acquired By:	LCMS-03
Vial:	2:B,1	Sample Set Name	21102024_LCMS_087_01
Injection #:		Acq. Method Set:	o2h_LCMS_Method_A
Injection Volume:	2.00 ul	Processing Method	Processing method_03,
Run Time:	4.0 Minutes	Channel Name:	220.0nm, 254.0nm, MS TIC
Injection Id	42365	Proc. Chnl. Descr.:	PDA 220.0 nm Blank Subtracted
Date Acquired:	21-10-2024 14:56:56 IST		
Date Processed:	21-10-2024 15:28:36 IST, 21-10-2024 15:31:24 IST, 21-10-2024 15:31:54 IST		
Column	XBRIDGE BEH C18 2.1*50mm,2.5um		

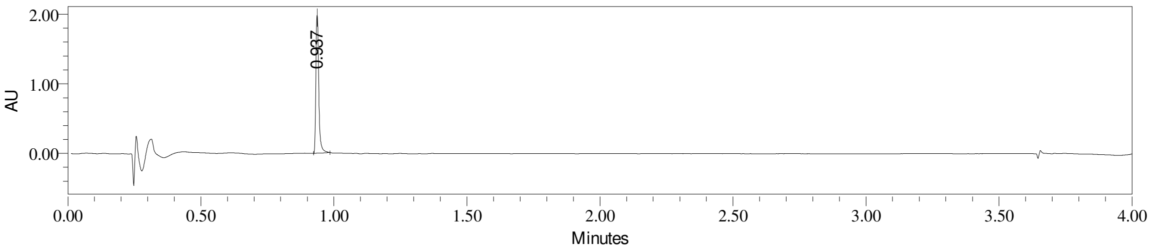

Channel: PDA Spectrum; Channel Name 220.0nm

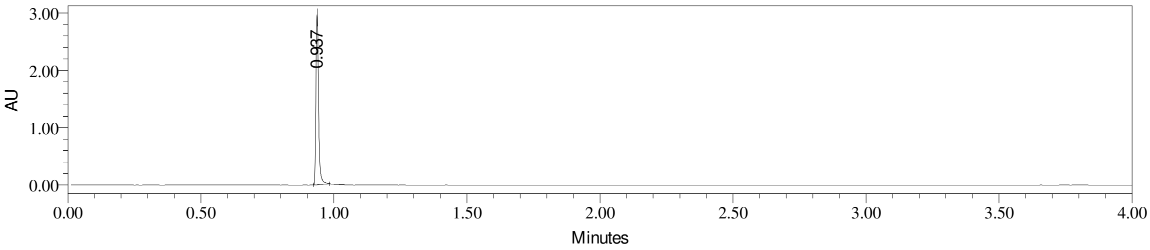

Channel: PDA Spectrum; Channel Name 254.0nm

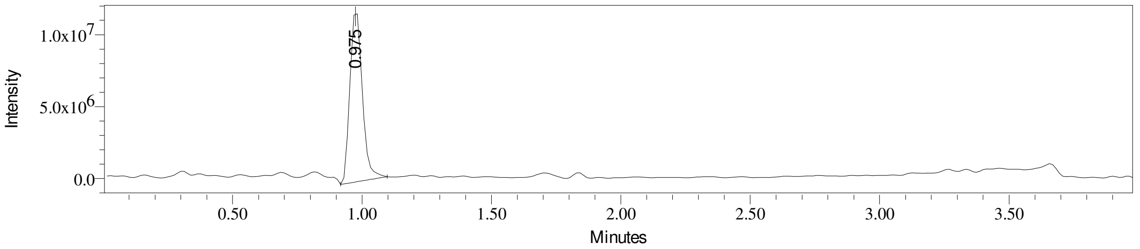

Channel: SQ 1: MS Scan; Channel Name MS TIC

Peak Results
Channel: PDA Spectrum

	Retention Time (min)	Base Peak (m/z)	Height (µV)	Area (µV*sec)	% Area	Channel	Channel Name
1	0.937		2970051	2058923	100.00	PDA Spectrum	254.0nm
2	0.937		1985063	1504617	100.00	PDA Spectrum	220.0nm

Peak Results
Channel: SQ 1: MS Scan

	Retention Time (min)	Base Peak (m/z)	Height (µV)	Area (µV*sec)	% Area	Channel	Channel Name
1	0.975	265.17	12068600	39548740	100.00	SQ 1: MS Scan	MS TIC

Page: 1 of 2

HPLC and LCMS Traces for Compound 2f

o2h discovery<br>Ahmedabad, Gujarat<br>India	LCMS Analysis Report

Match Plot

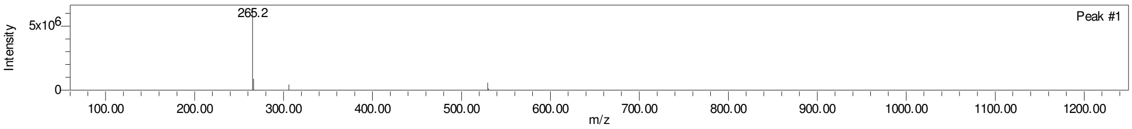

Base Peak 265.17 Channel Description 1: 50.00-1250.00 ES+, Centroid, CV=10 - AVG (0.0:0.6;1.5:3.9) x 22.000 Th: 0.010 - AVG (0.0:0.6;1.5:3.9;1.5:3.8) x 22.000 Th: 0.010 Retention Time 0.975

HPLC and LCMS Traces for Compound 2f

o2h discovery
Ahmedabad, Gujarat
India

HPLC Analysis Report

Sample name: CIN2-C-818-CIN2-X-0009-042-b
Location: P2-A3
Injection: 1 of 1
Injection volume: 10.000
Project Name HPLC-07_OCT-2024
Date Acquired: 2024-10-21 12:46:37+05:30
Date Processed: 2024-10-21 13:34:16+05:30

Instrument Name HPLC-07
Acq. method: o2h_HPLC_Method-D.amx
Processing method: *3D UV Quantitative_DefaultMethod.pmx
Column: XBridge C18 150x4.6mm, 3.5um

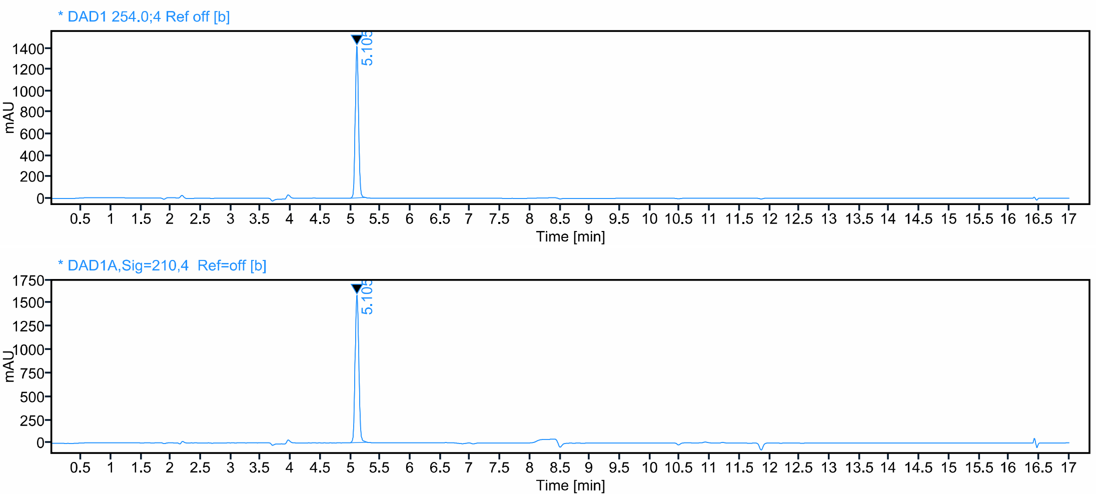

Signal: * DAD1 254.0;4 Ref off [b]

RT [min]	Height	Area	Area%
5.105	1421	5345	100.00

Signal: * DAD1A,Sig=210,4 Ref=off [b]

RT [min]	Height	Area	Area%
5.105	1587	6775	100.00

HPLC and LCMS Traces for Compound 2g

o2h discovery
Ahmedabad, Gujarat
India

LCMS Analysis Report

Sample Name:	CIN2-C-816-CIN2-X-0001-022-A		
Sample Type:	Unknown	Acquired By:	LCMS-03
Vial:	1:C,3	Sample Set Name	11102024_UCH_081_FD_01
Injection #:		Acq. Method Set:	o2h_LCMS_Method_A
Injection Volume:	3.00 ul	Processing Method	Processing method_06,
Run Time:	4.0 Minutes	Channel Name:	266.0nm, 220.0nm, MS TIC
Injection Id	29089	Proc. Chnl. Descr.:	PDA 266.0 nm Blank Subtracted
Date Acquired:	11-10-2024 12:55:30 IST		
Date Processed:	11-10-2024 13:03:27 IST, 11-10-2024 13:03:34 IST, 11-10-2024 13:04:52 IST		
Column	XBRIDGE BEH C18 2.1*50mm,2.5um		

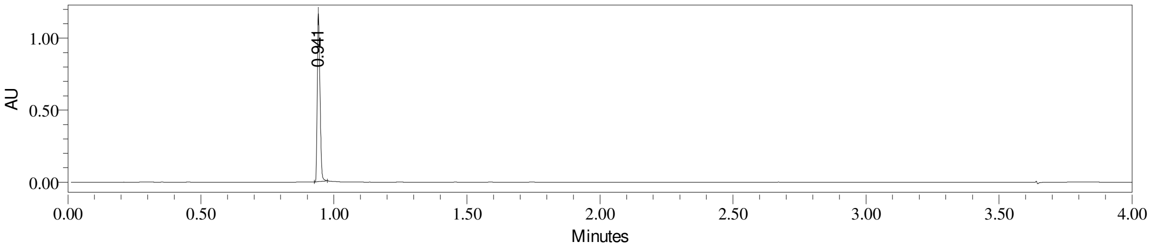

Channel: PDA Spectrum; Channel Name 266.0nm

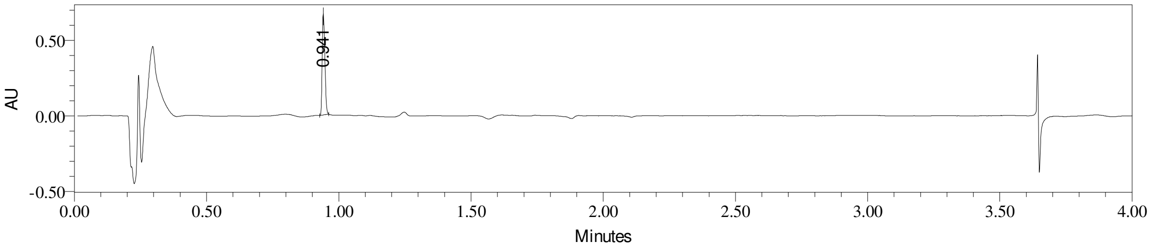

Channel: PDA Spectrum; Channel Name 220.0nm

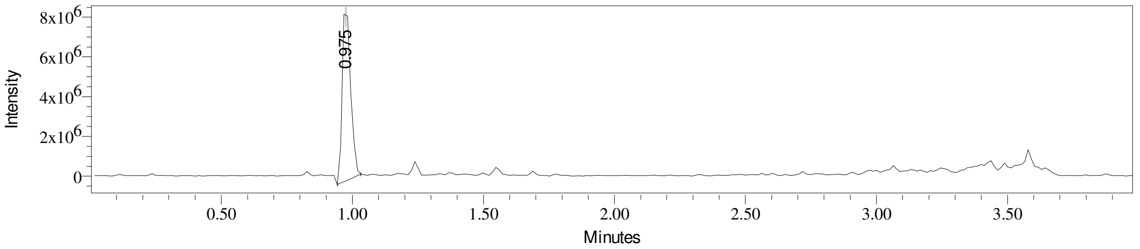

Channel: SQ 1: MS Scan; Channel Name MS TIC

Peak Results
Channel: PDA Spectrum

	Retention Time (min)	Base Peak (m/z)	Height (µV)	Area (µV*sec)	% Area	Channel	Channel Name
1	0.941		1169492	837592	100.00	PDA Spectrum	266.0nm
2	0.941		672419	474915	100.00	PDA Spectrum	220.0nm

Peak Results
Channel: SQ 1: MS Scan

	Retention Time (min)	Base Peak (m/z)	Height (µV)	Area (µV*sec)	% Area	Channel	Channel Name
1	0.975	283.12	9180392	19061003	100.00	SQ 1: MS Scan	MS TIC

Page: 1 of 2

HPLC and LCMS Traces for Compound 2g

o2h discovery
Ahmedabad, Gujarat
India

LCMS Analysis Report

Match Plot

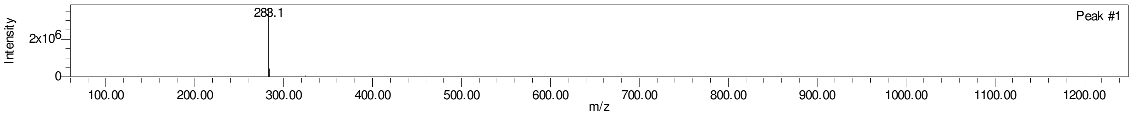

Base Peak 283.12 Channel Description 1: 50.00-1250.00 ES+, Centroid, CV=10 - AVG (1.6:4.0;0.0:0.2) x 30.000 Th: 0.010 - AVG (1.6:4.0;0.0:0.2;2.5:4.0;0.0:0.9;1.2:1.5) x 50.000 Th: 0.010 Retention Time 0.975

Page: 2 of 2

HPLC and LCMS Traces for Compound 2g

o2h discovery
Ahmedabad, Gujarat
India

HPLC Analysis Report

**Sample name:**	CIN2-C-816-CIN2-X-0001-022-A		
**Location:**	P2-B11	**Instrument Name**	HPLC-10
**Injection:**	1 of 1	**Acq. method:**	o2h_HPLC_Method-A.amx
**Injection volume:**	10.000	**Processing method:**	3D UV Quantitative_DefaultMethod.pmx
**Project Name**	HPLC-10_OCT-2024		
**Date Acquired:**	2024-10-10 17:03:42+05:30	**Column:**	SUNFIRE C18 150x4.6mm,3.5um
**Date Processed:**	2024-10-10 17:42:02+05:30		

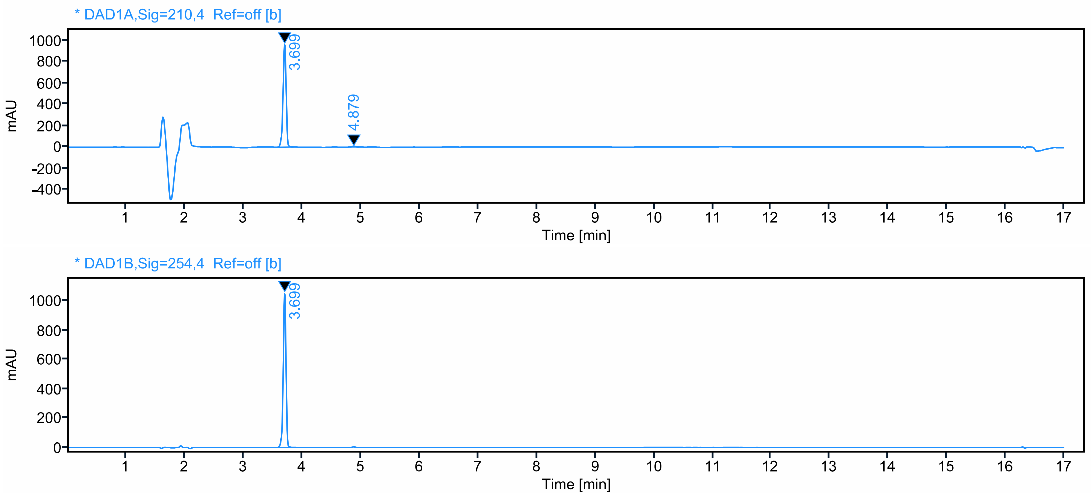

Signal: * DAD1A,Sig=210,4 Ref=off [b]

RT [min]	Height	Area	Area%
3.699	965	3577	99.22
4.879	8	28	0.78

Signal: * DAD1B,Sig=254,4 Ref=off [b]

RT [min]	Height	Area	Area%
3.699	1059	3509	100.00

HPLC and LCMS Traces for Compound 2i

o2h discovery
Ahmedabad, Gujarat
India

LCMS Analysis Report

Sample Name:	CIN2-C-816-CIN2-X-0074-033-B		Injection Id 3163
Sample Type:	Unknown	Acquired By:	LCMS-05
Vial:	1:A,2	Sample Set Name:	05112024_UCH_090_FD
Injection #:	1	Acq. Method Set:	o2h_LCMS_Method_A
Injection Volume:	2.00 ul	Processing Method	O2H_LCMS_02_0, MASS_000
Run Time:	4.0 Minutes	Channel Name:	MS TIC, 254.0nm, 210.0nm
Project Name:	2024\LCMS-05_NOV-2024	Proc. Chnl. Descr.:	PDA 254.0 nm Blank Subtracted
Date Acquired:	05-11-2024 08:33:23 IST		
Date Processed:	05-11-2024 14:10:22 IST, 05-11-2024 14:10:27 IST, 05-11-2024 14:10:34 IST		
Column:	X-BRIDGE C18 2.1X50mm 2.5um		

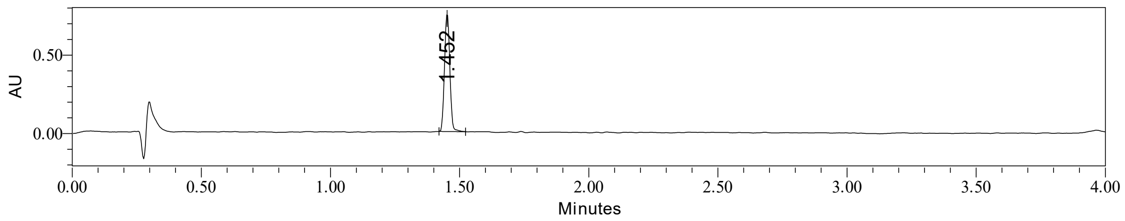

Channel Name 210.0nm; Channel PDA Spectrum

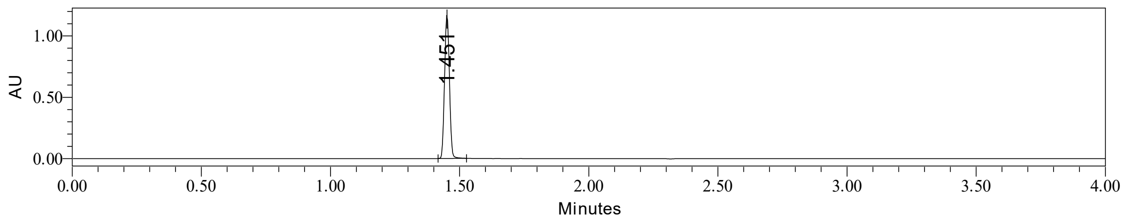

Channel Name 254.0nm; Channel PDA Spectrum

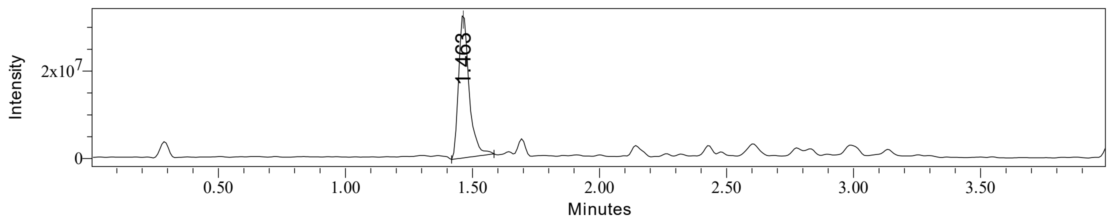

Channel Name MS TIC; Channel QDa 1: MS Scan

Peak Results
Channel: PDA Spectrum

	RT	Base Peak (m/z)	Height	Area	% Area	Channel	Channel Name
1	1.451		1164671	1528570	100.00	PDA Spectrum	254.0nm
2	1.452		750952	1074077	100.00	PDA Spectrum	210.0nm

Peak Results
Channel: QDa 1: MS Scan

	RT	Base Peak (m/z)	Height	Area	% Area	Channel	Channel Name
1	1.463	309.13	32677352	94480484	100.00	QDa 1: MS Scan	MS TIC

HPLC and LCMS Traces for Compound 2i

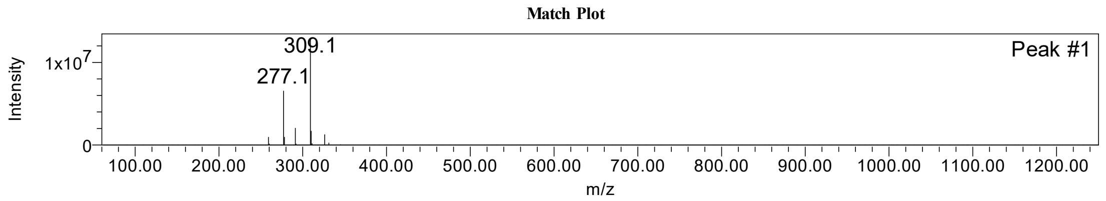

Base Peak 309.13 Channel Description 1: QDa Positive(+) Scan (60.00-1250.00)Da, Centroid, CV=10 - AVG (1.7:4.0;0.1:1.3) x 10.000 Th: 0.010 Retention Time 1.463

HPLC and LCMS Traces for Compound 2i

o2h discovery
Ahmedabad, Gujarat
India

HPLC Analysis Report

Sample name: CIN2-C-816-CIN2-X-0074-033-B
Location: P2-A1
Injection: 1 of 1
Injection volume: 10.000
Project Name HPLC-10-NOV-2024
Date Acquired: 2024-11-05 09:05:37+05:30
Date Processed: 2024-11-05 14:10:38+05:30

Instrument Name HPLC-10
Acq. method: o2h_HPLC_Method-A.amx
Processing method: *3D UV Quantitative_DefaultMethod.pmx
Column: SUNFIRE C18 150x4.6mm,3.5um

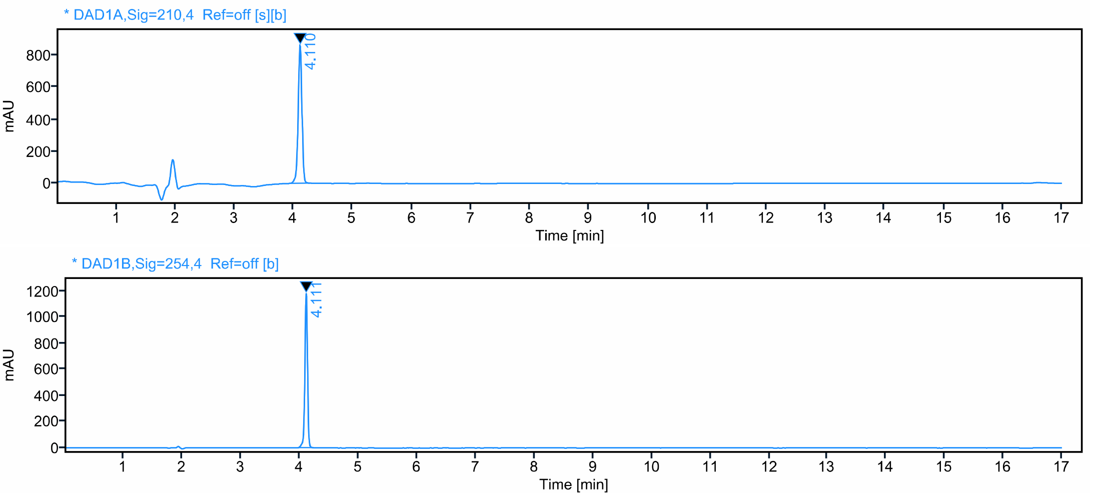

Signal: * DAD1A,Sig=210,4 Ref=off [s][b]

RT [min]	Height	Area	Area%
4.110	859	3788	100.00

Signal: * DAD1B,Sig=254,4 Ref=off [b]

RT [min]	Height	Area	Area%
4.111	1185	3770	100.00

HPLC and LCMS Traces for Compound 3a

o2h discovery
Ahmedabad, Gujarat
India

LCMS Analysis Report

**Sample name:**	CIN-C-375-X-0004-023-b		
**Location:**	P2-A5	**Instrument Name**	LCMS-09
**Injection:**	1 of 1	**Acq. method:**	o2h_LCMS_Method_D.amx
**Injection volume:**	10.000	**Processing method:**	*LC_MS Sample Purity_DefaultMethod_NEW.pmx
**Project Name**	LCMS-09_MAY-2024	**Description:**	
**Date Acquired:**	2024-05-27 13:59:16+05:30	**Column:**	SUNFIRE C18 150x4.6mm, 3.5um
**Date Processed:**	2024-05-27 14:22:09+05:30		

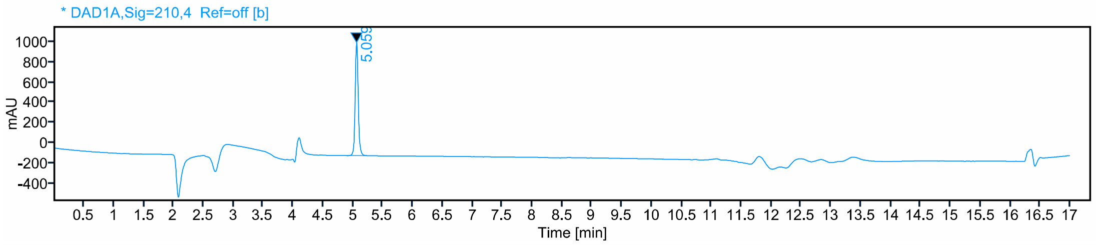

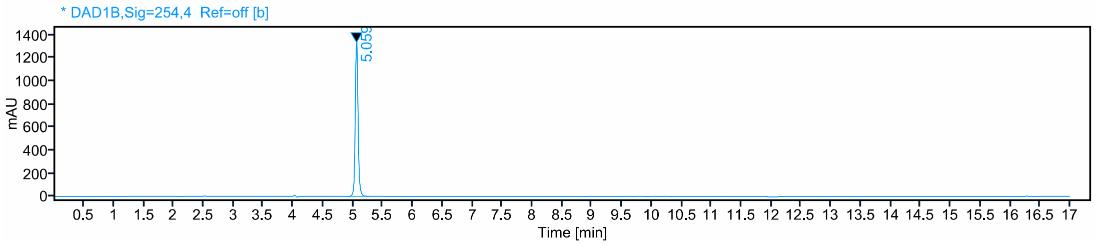

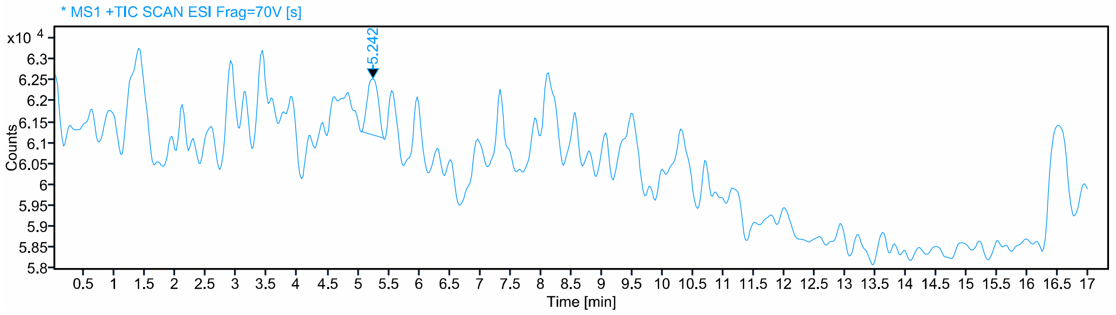

Printed: 2024-05-27 14:22:32+05:30 **Page 1 of 3**

HPLC and LCMS Traces for Compound 3a

o2h discovery
Ahmedabad, Gujarat
India

LCMS Analysis Report

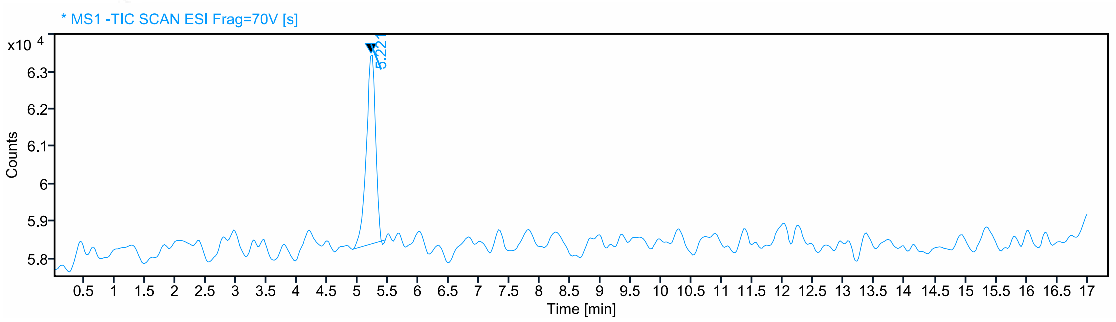

Signal: * DAD1A,Sig=210,4 Ref=off [b]

RT [min]	Peak MS Base Peak m/z	Height	Area	Area%
5.059		1106	3944	100.00

Signal: * DAD1B,Sig=254,4 Ref=off [b]

RT [min]	Peak MS Base Peak m/z	Height	Area	Area%
5.059		1330	4425	100.00

Signal: * MS1 +TIC SCAN ESI Frag=70V [s]

RT [min]	Peak MS Base Peak m/z	Height	Area	Area%
5.242	296.800	1339	16941	100.00

Signal: * MS1 -TIC SCAN ESI Frag=70V [s]

RT [min]	Peak MS Base Peak m/z	Height	Area	Area%
5.221	295.000	5101	53929	100.00

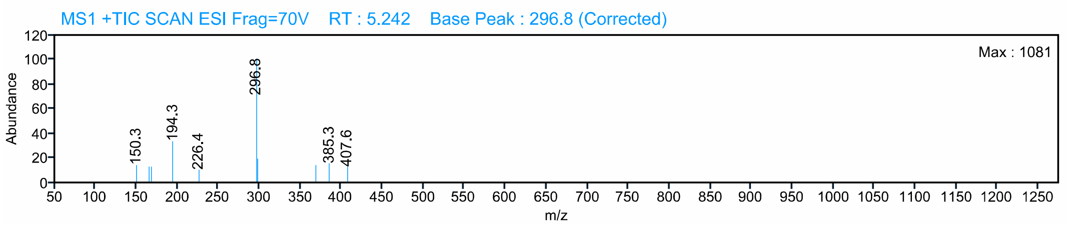

HPLC and LCMS Traces for Compound 3a

o2h discovery
Ahmedabad, Gujarat
India

LCMS Analysis Report

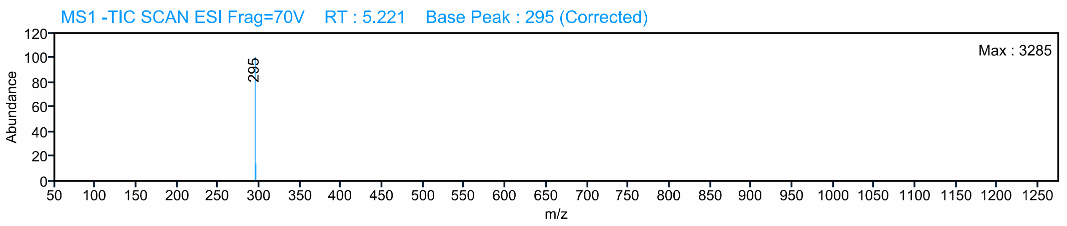

Printed: 2024-05-27 14:22:32+05:30 **Page 3 of 3**

HPLC and LCMS Traces for Compound 3a

o2h discovery<br>Ahmedabad, Gujarat<br>India	**HPLC Analysis Report**

Sample name: CIN-C-375-X-0004-023-b
Location: P1-A8
Injection: 1 of 1
Injection volume: 10.000
Project Name HPLC-10_MAY-2024
Date Acquired: 2024-05-27 12:59:14+05:30
Date Processed: 2024-05-27 13:19:02+05:30

Instrument Name HPLC-10
Acq. method: o2h_HPLC_Method-C.amx
Processing method: 3D UV Quantitative_DefaultMethod.pmx
Column: SUNFIRE C18 150x4.6mm,3.5um

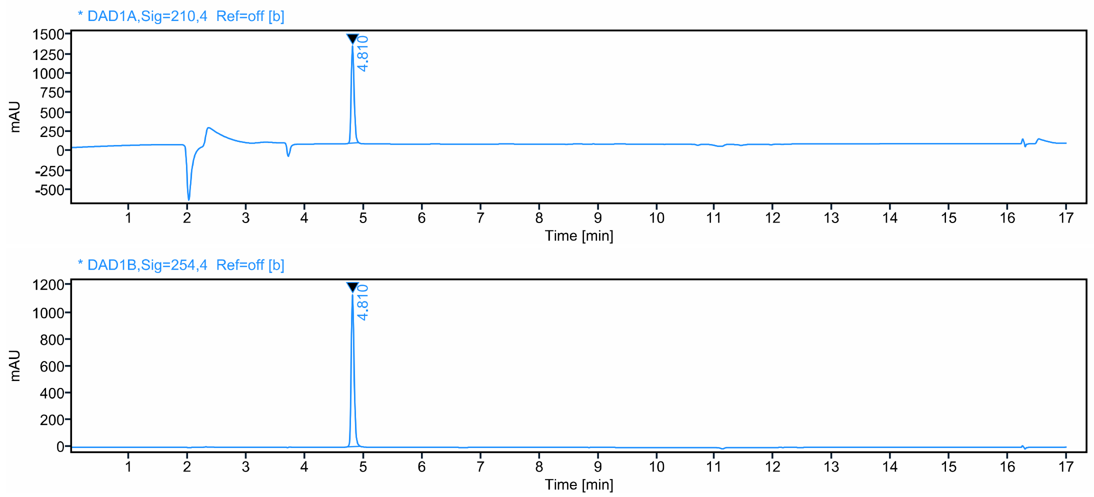

Signal: * DAD1A,Sig=210,4 Ref=off [b]

RT [min]	Height	Area	Area%
4.810	1257	4305	100.00

Signal: * DAD1B,Sig=254,4 Ref=off [b]

RT [min]	Height	Area	Area%
4.810	1136	3887	100.00

HPLC and LCMS Traces for Compound 3b

o2h discovery
Ahmedabad, Gujarat
India

LCMS Analysis Report

**Sample name:**	CIN-C-375-CIN-X-0006-038-b		
**Location:**	P2-D4	**Instrument Name**	LCMS-09
**Injection:**	1 of 1	**Acq. method:**	o2h_LCMS_Method_D.amx
**Injection volume:**	10.000	**Processing method:**	*LC_MS Sample Purity_DefaultMethod_NEW.pmx
**Project Name**	LCMS-09_JUNE-2024	**Description:**	
**Date Acquired:**	2024-06-27 14:10:05+05:30	**Column:**	SUNFIRE C18 150x4.6mm, 3.5um
**Date Processed:**	2024-06-27 15:03:36+05:30		

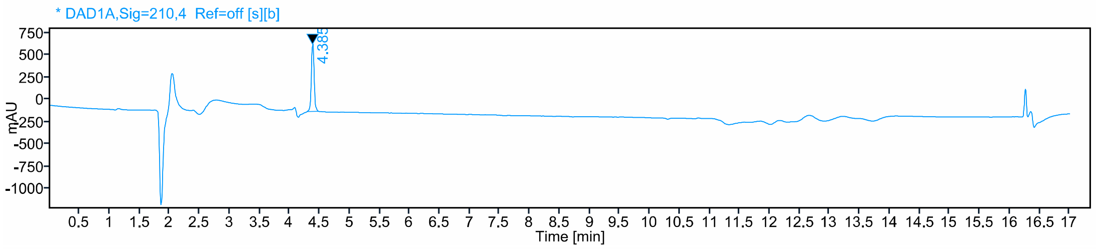

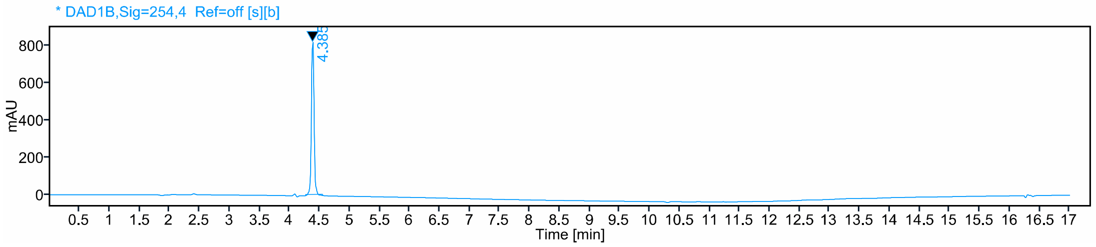

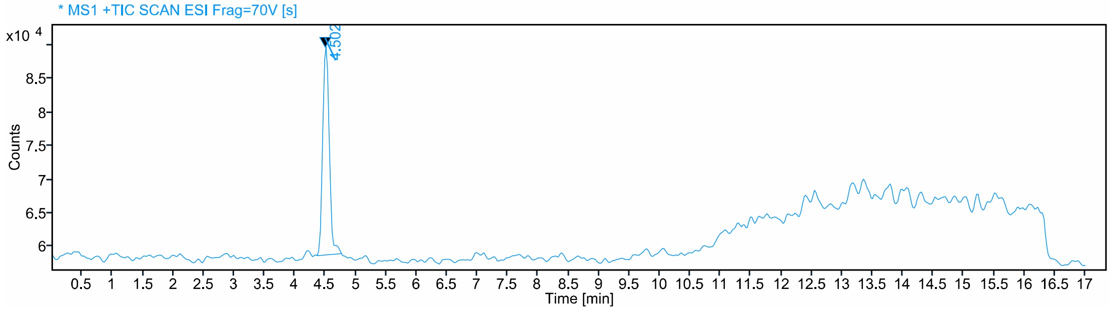

Printed: 2024-06-27 15:06:00+05:30 **Page 1 of 3**

HPLC and LCMS Traces for Compound 3b

o2h discovery
Ahmedabad, Gujarat
India

LCMS Analysis Report

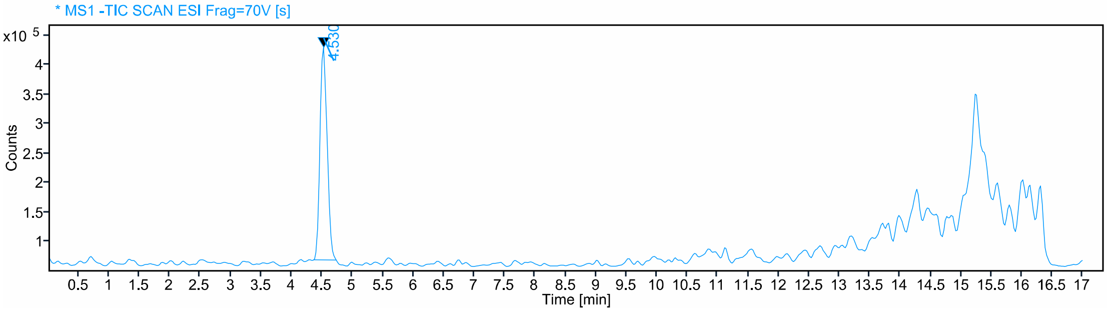

Signal: * DAD1A,Sig=210,4 Ref=off [s][b]

RT [min]	Base Peak (m/z)	Height	Area	Area%
4.385		751	2432	100.00

Signal: * DAD1B,Sig=254,4 Ref=off [s][b]

RT [min]	Base Peak (m/z)	Height	Area	Area%
4.385		814	2461	100.00

Signal: * MS1 +TIC SCAN ESI Frag=70V [s]

RT [min]	Base Peak (m/z)	Height	Area	Area%
4.502	296.300	30779	237624	100.00

Signal: * MS1 -TIC SCAN ESI Frag=70V [s]

RT [min]	Base Peak (m/z)	Height	Area	Area%
4.530	309.100	360482	2968130	100.00

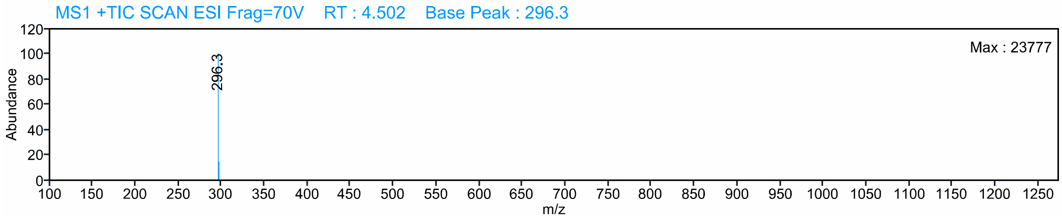

Printed: 2024-06-27 15:06:00+05:30 **Page 2 of 3**

HPLC and LCMS Traces for Compound 3b

o2h discovery
Ahmedabad, Gujarat
India

LCMS Analysis Report

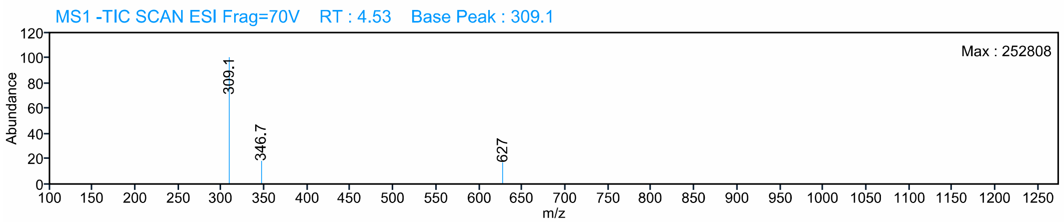

Printed: 2024-06-27 15:06:00+05:30

HPLC and LCMS Traces for Compound 3b

o2h discovery
Ahmedabad, Gujarat
India

HPLC Analysis Report

Sample name: CIN-C-375-CIN-X-0006-038-b
Location: P2-A3
Injection: 1 of 1
Injection volume: 10.000
Project Name HPLC-10_JUNE-2024
Date Acquired: 2024-06-27 11:48:53+05:30
Date Processed: 2024-06-27 12:22:04+05:30

Instrument Name HPLC-10
Acq. method: o2h_HPLC_Method-C.amx
Processing method: *3D UV Quantitative_DefaultMethod.pmx
Column: SUNFIRE C18 150x4.6mm,3.5um

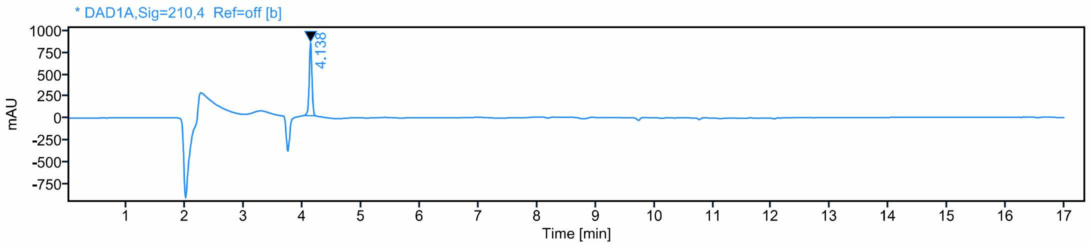

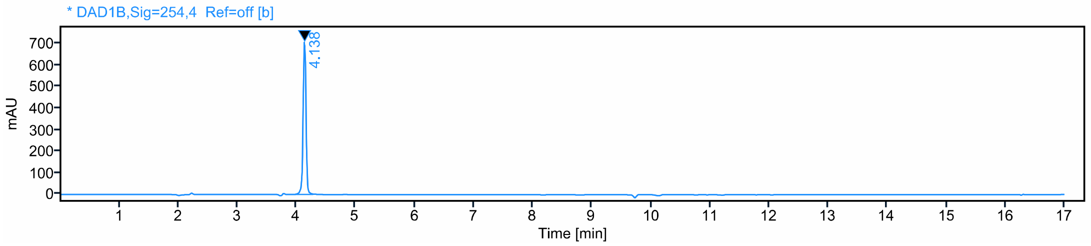

Signal: * DAD1A,Sig=210,4 Ref=off [b]

RT [min]	Height	Area	Area%
4.138	841	2754	100.00

Signal: * DAD1B,Sig=254,4 Ref=off [b]

RT [min]	Height	Area	Area%
4.138	707	2349	100.00

HPLC and LCMS Traces for Compound 3c

o2h discovery
Ahmedabad, Gujarat
India

LCMS Analysis Report

Sample Name: CIN-C-375-CIN-X-0005-050-a
Injection Id 28555
Sample Type: Unknown
Acquired By: LCMS-05
Vial: 2:B,4
Sample Set Name: 11072024_UCH_082_RD
Injection #: 1
Acq. Method Set: o2h_LCMS_Method_A_SLOW_01
Injection Volume: 6.00 ul
Processing Method O2H_LCMS_02,
Run Time: 4.0 Minutes
Channel Name: 220.0nm, MS TIC, 254.0nm
Project Name: 2024\LCMS-05_JUL-2024
Proc. Chnl. Descr.: QDa 1: MS Scan MS TIC,
Date Acquired: 11-07-2024 13:23:59 IST
Date Processed: 11-07-2024 13:40:47 IST, 11-07-2024 13:41:04 IST, 11-07-2024 13:42:13 IST
Column: X-BRIDGE C18 2.1X50mm 2.5um

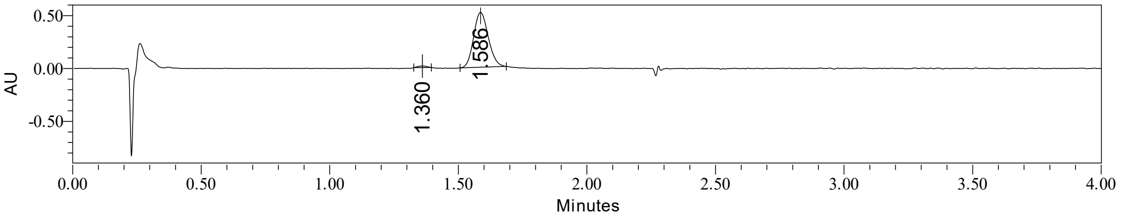

Channel PDA Spectrum; Channel Name 220.0nm

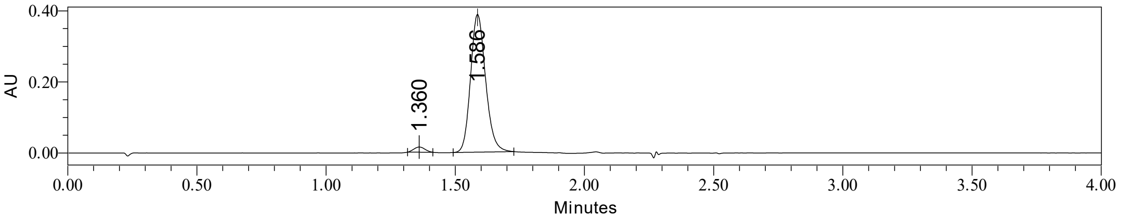

Channel PDA Spectrum; Channel Name 254.0nm

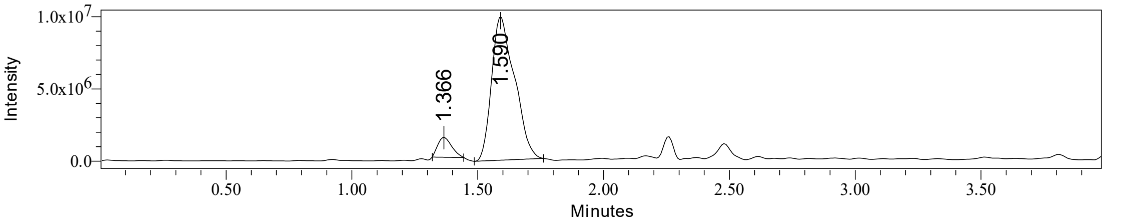

Channel QDa 1: MS Scan; Channel Name MS TIC

Peak Results
Channel: PDA Spectrum

	RT	Base Peak (m/z)	Height	Area	% Area	Channel	Channel Name
1	1.360		14791	35982	1.72	PDA Spectrum	220.0nm
2	1.360		14615	43499	2.70	PDA Spectrum	254.0nm
3	1.586		520041	2053286	98.28	PDA Spectrum	220.0nm
4	1.586		387843	1566811	97.30	PDA Spectrum	254.0nm

HPLC and LCMS Traces for Compound 3c

Peak Results
Channel: QDa 1: MS Scan

	RT	Base Peak (m/z)	Height	Area	% Area	Channel	Channel Name
1	1.366	318.10	1369310	5292688	7.76	QDa 1: MS Scan	MS TIC
2	1.590	296.09	9955119	62924743	92.24	QDa 1: MS Scan	MS TIC

Match Plot

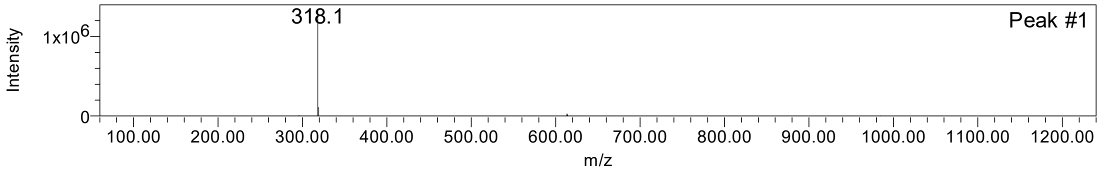

Base Peak 318.10 Channel Description 1: QDa Positive(+) Scan (60.00-1240.00)Da, Centroid, CV=10 - AVG (2.2:3.4)
20.000 Th: 0.010 - AVG (2.2:2.9;0.3:0.9;0.6:1.3) x 20.000 Th: 0.010 Retention Time 1.366

Match Plot

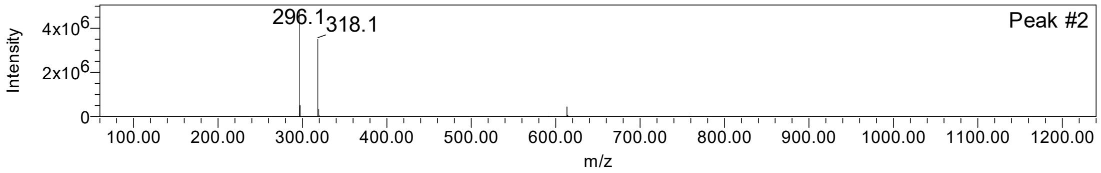

Base Peak 296.09 Channel Description 1: QDa Positive(+) Scan (60.00-1240.00)Da, Centroid, CV=10 - AVG (2.2:3.4)
20.000 Th: 0.010 - AVG (2.2:2.9;0.3:0.9;0.6:1.3) x 20.000 Th: 0.010 Retention Time 1.590

HPLC and LCMS Traces for Compound 3c

o2h discovery
Ahmedabad, Gujarat
India

HPLC Analysis Report

**Sample name:**	CIN-C-375-CIN-X-0005-050-a		
**Location:**	P1-A5	**Instrument Name**	HPLC-10
**Injection:**	1 of 1	**Acq. method:**	o2h_HPLC_Method-C.amx
**Injection volume:**	10.000	**Processing method:**	*3D UV Quantitative_DefaultMethod.pmx
**Project Name**	HPLC-10_JULY-2024		
**Date Acquired:**	2024-07-11 11:45:56+05:30	**Column:**	SUNFIRE C18 150x4.6mm,3.5um
**Date Processed:**	2024-07-11 12:44:48+05:30		

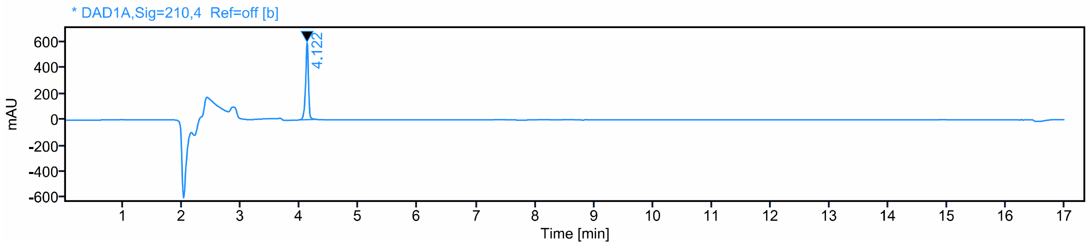

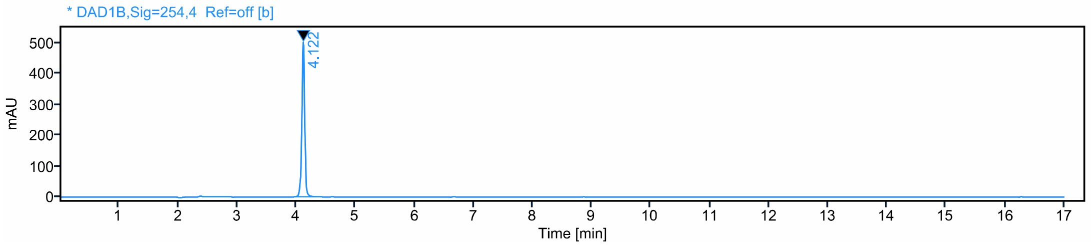

Signal: * DAD1A,Sig=210,4 Ref=off [b]

RT [min]	Height	Area	Area%
4.122	596	2008	100.00

Signal: * DAD1B,Sig=254,4 Ref=off [b]

RT [min]	Height	Area	Area%
4.122	503	1687	100.00

HPLC and LCMS Traces for Compound 3d

o2h discovery<br>Ahmedabad, Gujarat<br>India	**LCMS Analysis Report**

Sample Name:	CIN2-C-816-CIN2-X-0091-097-b		Injection Id 53574
Sample Type:	Unknown	Acquired By:	LCMS-05
Vial:	1:B,1	Sample Set Name:	26122024_UCH_NEW_FD_
Injection #:	1	Acq. Method Set:	o2h_LCMS_Method_A_SLOW_01
Injection Volume:	3.00 ul	Processing Method	O2H_LCMS_02_0,
Run Time:	4.0 Minutes	Channel Name:	MS TIC, 254.0nm, 210.0nm
Project Name:	2024\LCMS-05_DEC-2024_	Proc. Chnl. Descr.:	PDA 210.0 nm Blank Subtracted
Date Acquired:	26-12-2024 10:43:15 IST		
Date Processed:	26-12-2024 10:46:17 IST, 26-12-2024 10:46:52 IST, 26-12-2024 10:47:52 IST		
Column:	X-BRIDGE C18 2.1X50mm 2.5um		

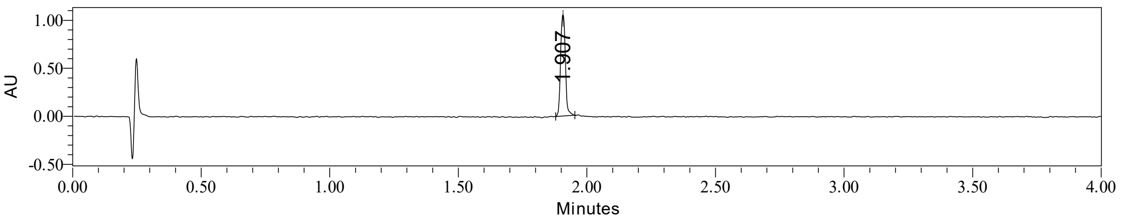

Channel Name 210.0nm; Channel PDA Spectrum

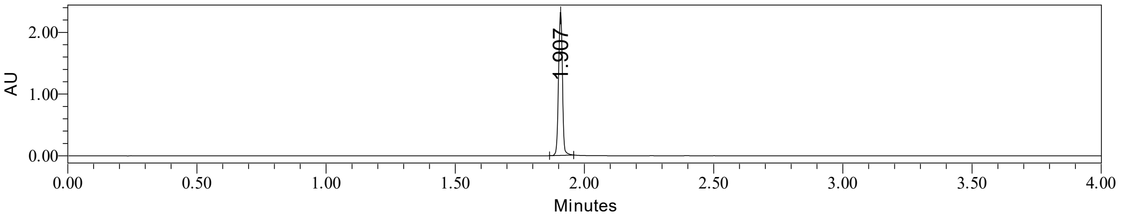

Channel Name 254.0nm; Channel PDA Spectrum

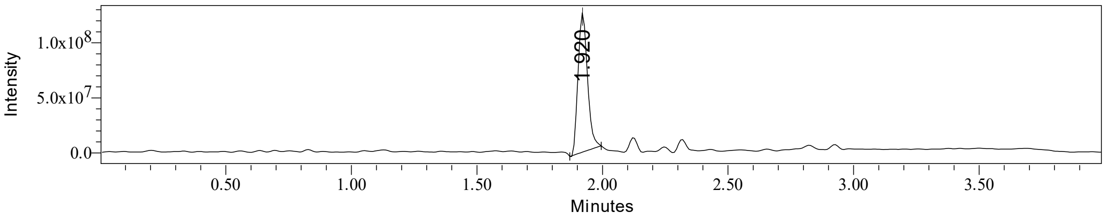

Channel Name MS TIC; Channel QDa 1: MS Scan

Peak Results
Channel: PDA Spectrum

	RT	Base Peak (m/z)	Height	Area	% Area	Channel	Channel Name
1	1.907		1055768	1368104	100.00	PDA Spectrum	210.0nm
2	1.907		2328218	2402113	100.00	PDA Spectrum	254.0nm

Peak Results
Channel: QDa 1: MS Scan

	RT	Base Peak (m/z)	Height	Area	% Area	Channel	Channel Name
1	1.920	296.00	125278319	330017333	100.00	QDa 1: MS Scan	MS TIC

HPLC and LCMS Traces for Compound 3d

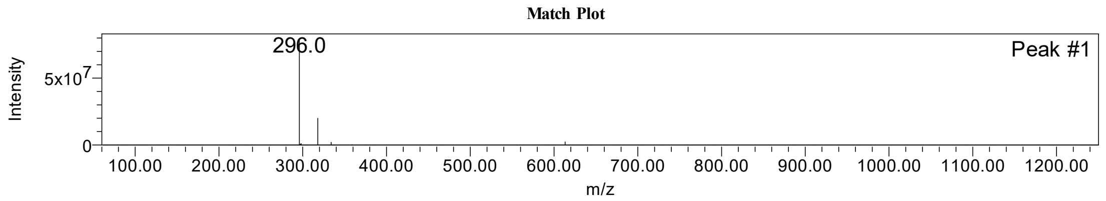

Base Peak 296.00 Channel Description 1: QDa Positive(+) Scan (60.00-1240.00)Da, Centroid, CV=10 - AVG (2.1:3.9;0.1:0.7) x 20.000 Th: 0.010 Retention Time 1.920

HPLC and LCMS Traces for Compound 3d

o2h discovery
Ahmedabad, Gujarat
India

HPLC Analysis Report

Sample name: CIN2-C-816-CIN2-X-0091-097-b

Location: P2-A3

Injection: 1 of 1

Injection volume: 10.000

Project Name HPLC-07_DEC-2024

Date Acquired: 2024-12-26 14:28:16+05:30

Date Processed: 2024-12-26 15:16:06+05:30

Instrument Name HPLC-07

Acq. method: o2h_HPLC_Method-C.amx

Processing method: *3D UV Quantitative_DefaultMethod.pmx

Column: SUNFIRE C18 150x4.6mm 3.5um

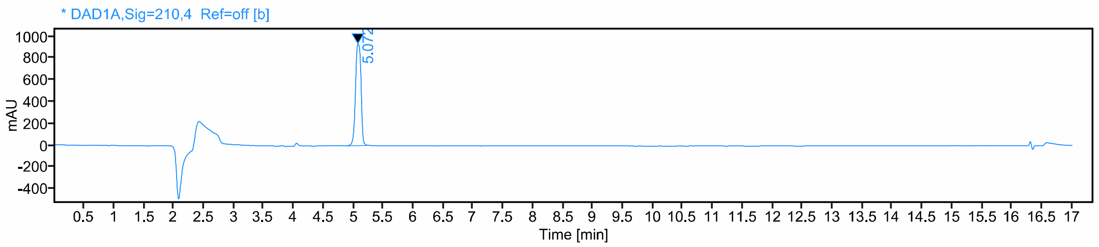

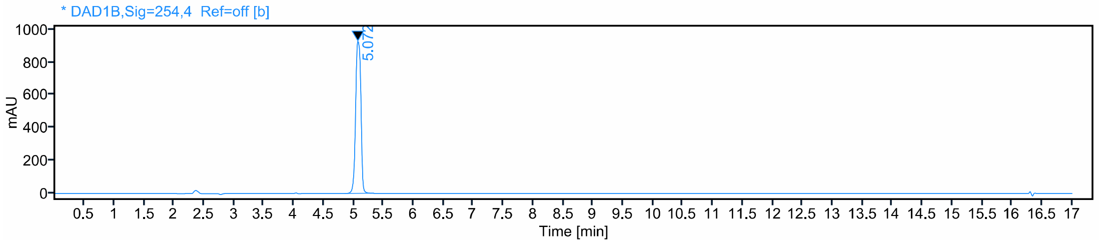

Signal: * DAD1A,Sig=210,4 Ref=off [b]

RT [min]	Height	Area	Area%
5.072	933	5954	100.00

Signal: * DAD1B,Sig=254,4 Ref=off [b]

RT [min]	Height	Area	Area%
5.072	927	5633	100.00

HPLC and LCMS Traces for Compound 3e

o2h discovery
Ahmedabad, Gujarat
India

LCMS Analysis Report

Sample Name:	CIN2-D-051-CIN2-X-0090-006-b		Injection Id 26783
Sample Type:	Unknown	Acquired By:	LCMS-05
Vial:	2:C,8	Sample Set Name:	12122024_UCH_NEW_FD
Injection #:	1	Acq. Method Set:	o2h_LCMS_Method_A
Injection Volume:	2.00 ul	Processing Method	O2H_LCMS_02_0,
Run Time:	4.0 Minutes	Channel Name:	MS TIC, 254.0nm, 210.0nm
Project Name:	2024\LCMS-05_DEC-2024_	Proc. Chnl. Descr.:	QDa 1: MS Scan MS TIC,
Date Acquired:	12-12-2024 13:56:58 IST		
Date Processed:	12-12-2024 14:00:01 IST, 12-12-2024 14:00:07 IST, 12-12-2024 14:01:57 IST		
Column:	X-BRIDGE C18 2.1X50mm 2.5um		

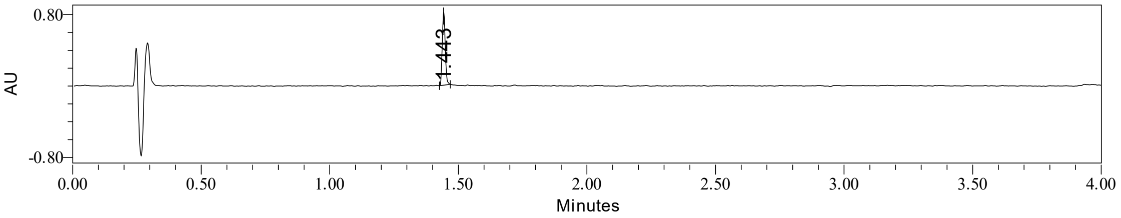

Channel Name 210.0nm; Channel PDA Spectrum

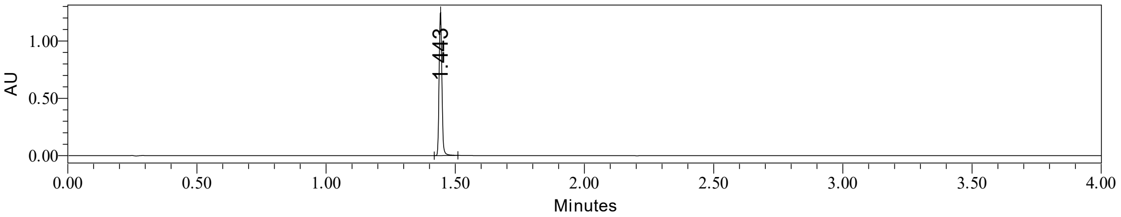

Channel Name 254.0nm; Channel PDA Spectrum

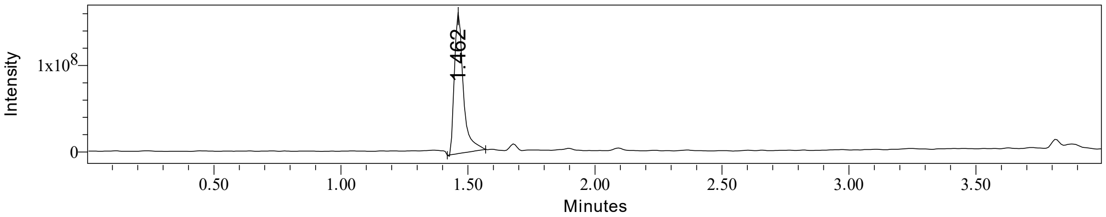

Channel Name MS TIC; Channel QDa 1: MS Scan

Peak Results
Channel: PDA Spectrum

	RT	Base Peak (m/z)	Height	Area	% Area	Channel	Channel Name
1	1.443		1254886	936902	100.00	PDA Spectrum	254.0nm
2	1.443		816790	664826	100.00	PDA Spectrum	210.0nm

Peak Results
Channel: QDa 1: MS Scan

	RT	Base Peak (m/z)	Height	Area	% Area	Channel	Channel Name
1	1.462	280.08	163499518	398303055	100.00	QDa 1: MS Scan	MS TIC

HPLC and LCMS Traces for Compound 3e

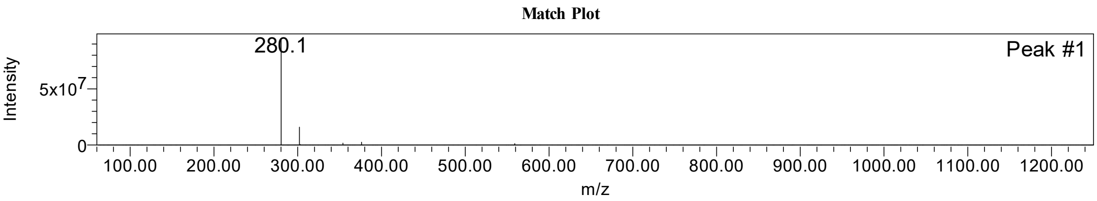

Base Peak 280.08 Channel Description 1: QDa Positive(+) Scan (60.00-1250.00)Da, Centroid, CV=10 - AVG (1.7:3.8;0.0:1.2) x 20.000 Th: 0.010 Retention Time 1.462

HPLC and LCMS Traces for Compound 3e

o2h discovery
Ahmedabad, Gujarat
India

HPLC Analysis Report

Sample name: CIN2-D-051-CIN2-X-0090-006-b

Location: P2-B4

Injection: 1 of 1

Injection volume: 10.000

Project Name HPLC-07_DEC-2024

Date Acquired: 2024-12-12 16:20:16+05:30

Date Processed: 2024-12-12 16:43:32+05:30

Instrument Name HPLC-07

Acq. method: o2h_HPLC_Method-A.amx

Processing method: 3D UV Quantitative_DefaultMethod.pmx

Column: SUNFIRE C18 150x4.6mm 3.5um

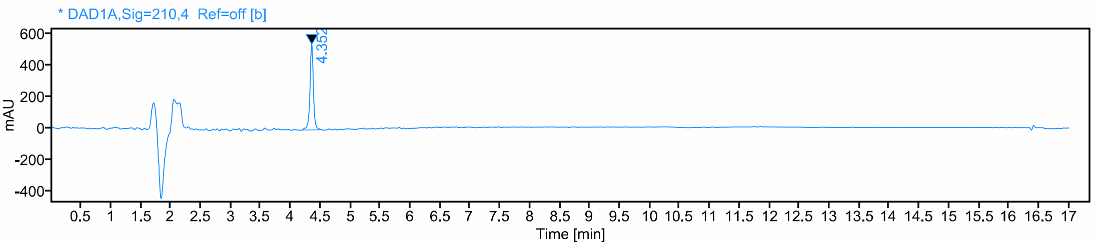

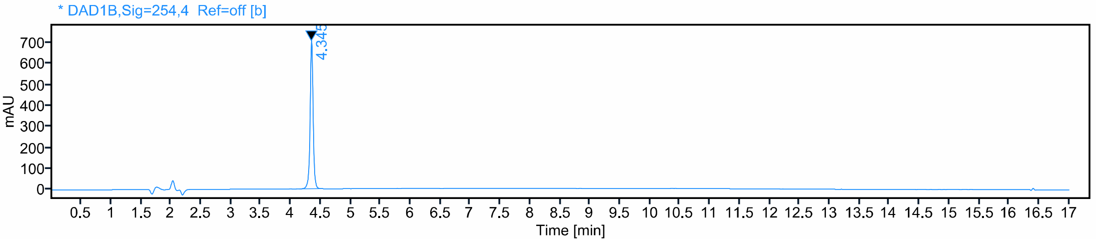

Signal: * DAD1A,Sig=210,4 Ref=off [b]

RT [min]	Height	Area	Area%
4.352	545	2223	100.00

Signal: * DAD1B,Sig=254,4 Ref=off [b]

RT [min]	Height	Area	Area%
4.345	701	2537	100.00

HPLC and LCMS Traces for Compound 3f

o2h discovery
Ahmedabad, Gujarat
India

LCMS Analysis Report

Sample Name:	CIN2-D-051-CIN2-X-0096-026-d		Injection Id 46438
Sample Type:	Unknown	Acquired By:	LCMS-05
Vial:	1:F,2	Sample Set Name:	20122024_UCH_NEW_FD
Injection #:	1	Acq. Method Set:	o2h_LCMS_Method_A_SLOW_01
Injection Volume:	8.00 ul	Processing Method	O2H_LCMS_02,
Run Time:	4.0 Minutes	Channel Name:	220.0nm, 254.0nm, MS TIC
Project Name:	2024\LCMS-05_DEC-2024_	Proc. Chnl. Descr.:	QDa 1: MS Scan MS TIC,
Date Acquired:	20-12-2024 09:38:16 IST		
Date Processed:	20-12-2024 10:02:43 IST, 20-12-2024 10:02:56 IST, 20-12-2024 10:03:47 IST		
Column:	X-BRIDGE C18 2.1X50mm 2.5um		

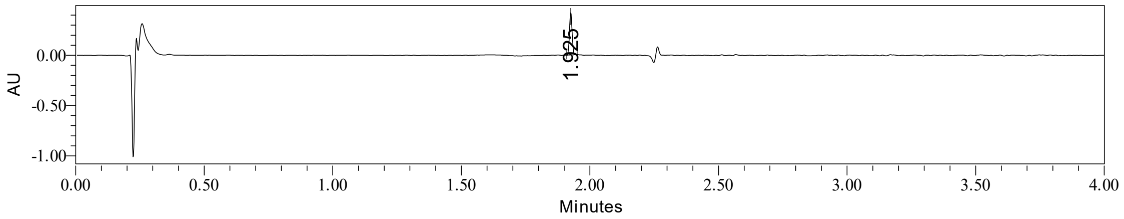

Channel Name 220.0nm; Channel PDA Spectrum

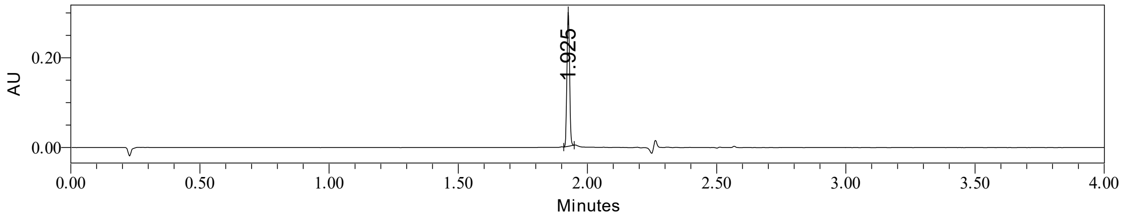

Channel Name 254.0nm; Channel PDA Spectrum

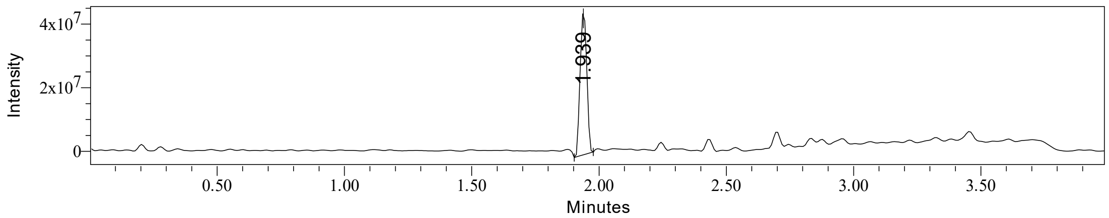

Channel Name MS TIC; Channel QDa 1: MS Scan

Peak Results
Channel: PDA Spectrum

	RT	Base Peak (m/z)	Height	Area	% Area	Channel	Channel Name
1	1.925		298748	212247	100.00	PDA Spectrum	254.0nm
2	1.925		419048	297112	100.00	PDA Spectrum	220.0nm

Peak Results
Channel: QDa 1: MS Scan

	RT	Base Peak (m/z)	Height	Area	% Area	Channel	Channel Name
1	1.939	280.02	45454281	81000894	100.00	QDa 1: MS Scan	MS TIC

O2h Discovery Page: 1 of 2

HPLC and LCMS Traces for Compound 3f

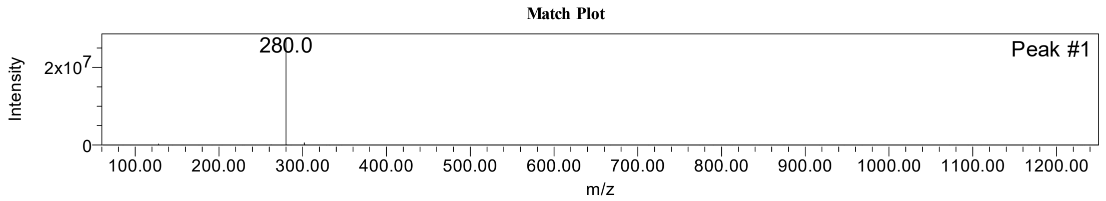

Base Peak 280.02 Channel Description 1: QDa Positive(+) Scan (60.00-1240.00)Da, Centroid, CV=10 - AVG (0.1:1.3;2.2:3.9) x 20.000 Th: 0.010 - AVG (2.2:2.6) x 20.000 Th: 0.010 Retention Time 1.939

HPLC and LCMS Traces for Compound 3f

o2h discovery
Ahmedabad, Gujarat
India

HPLC Analysis Report

**Sample name:**	CIN2-D-051-CIN2-X-0096-026-d		
**Location:**	P1-A3	**Instrument Name**	HPLC-10
**Injection:**	1 of 1	**Acq. method:**	o2h_HPLC_Method-D.amx
**Injection volume:**	100.000	**Processing method:**	*3D UV Quantitative_DefaultMethod.pmx
**Project Name**	HPLC-10_DEC-2024	**Column:**	XBridge C18 150x4.6mm, 3.5um
**Date Acquired:**	2024-12-20 09:57:35+05:30		
**Date Processed:**	2024-12-20 10:23:24+05:30		

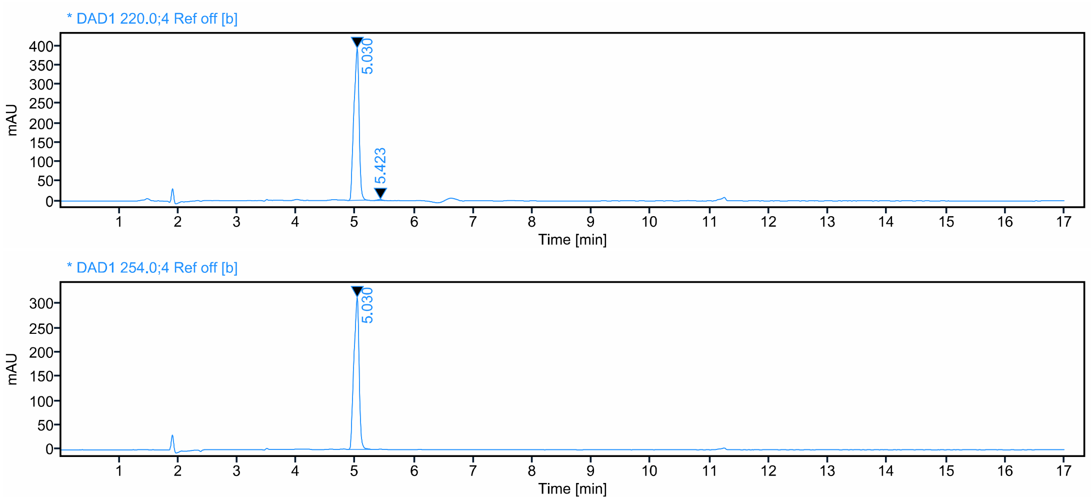

Signal: * DAD1 220.0;4 Ref off [b]

RT [min]	Height	Area	Area%
5.030	391	2390	99.39
5.423	3	15	0.61

Signal: * DAD1 254.0;4 Ref off [b]

RT [min]	Height	Area	Area%
5.030	313	1922	100.00

Printed: 2024-12-20 10:23:42+05:30 **Page 1 of 1**

HPLC and LCMS Traces for Compound 4a

o2h discovery
Ahmedabad, Gujarat
India

LCMS Analysis Report

**Sample name:**	CIN-C-375-X-0007-022-b		
**Location:**	P2-C1	**Instrument Name**	LCMS-09
**Injection:**	1 of 1	**Acq. method:**	o2h_LCMS_Method_D.amx
**Injection volume:**	10.000	**Processing method:**	*LC_MS Sample Purity_DefaultMethod_NEW.pmx
**Project Name**	LCMS-09_JUNE-2024	**Description:**	
**Date Acquired:**	2024-06-01 11:04:27+05:30	**Column:**	SUNFIRE C18 150x4.6mm, 3.5um
**Date Processed:**	2024-06-01 12:20:58+05:30		

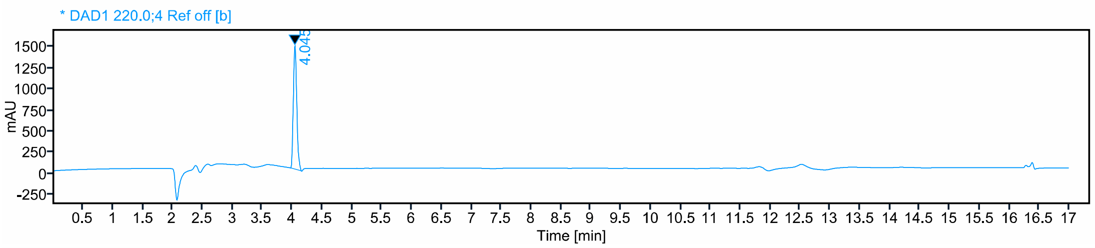

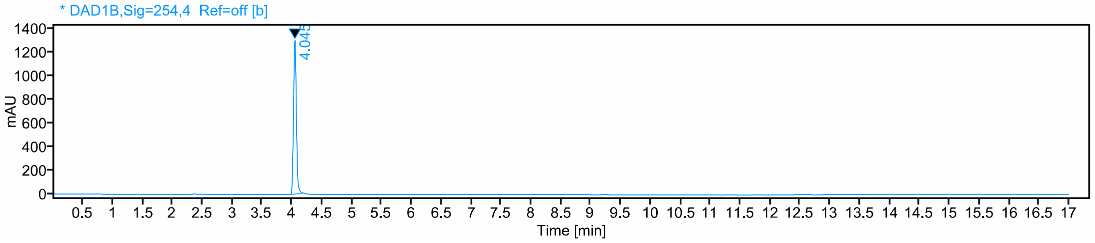

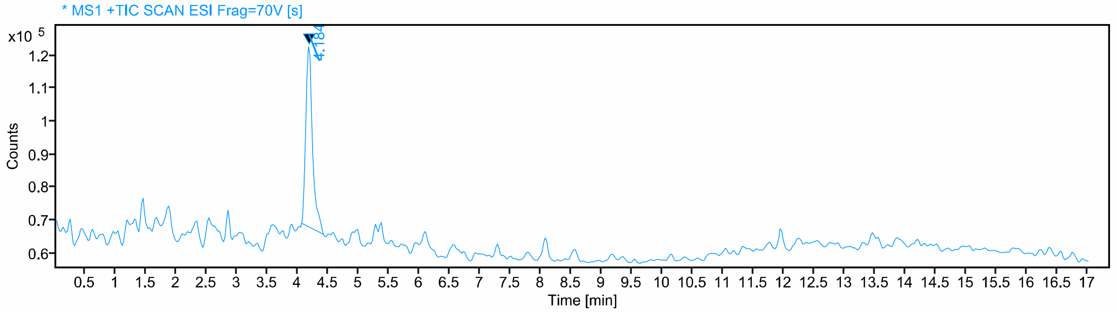

Signal: * DAD1 220.0;4 Ref off [b]

RT [min]	Base Peak (m/z)	Height	Area	Area%
4.045		1465	5835	100.00

Printed: 2024-06-01 12:21:23+05:30 **Page 1 of 2**

HPLC and LCMS Traces for Compound 4a

o2h discovery
Ahmedabad, Gujarat
India

LCMS Analysis Report

Signal: * DAD1B,Sig=254,4 Ref=off [b]

RT [min]	Base Peak (m/z)	Height	Area	Area%
4.045		1307	4238	100.00

Signal: * MS1 +TIC SCAN ESI Frag=70V [s]

RT [min]	Base Peak (m/z)	Height	Area	Area%
4.184	280.400	55272	427803	100.00

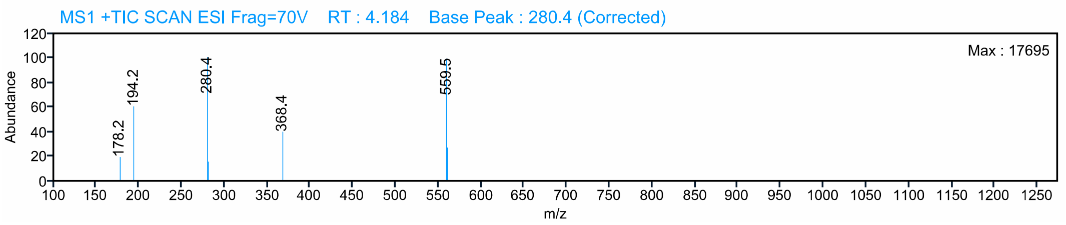

HPLC and LCMS Traces for Compound 4a

o2h discovery
Ahmedabad, Gujarat
India

HPLC Analysis Report

Sample name: CIN-C-375-X-0007-022-b
Location: P2-D1
Injection: 1 of 1
Injection volume: 10.000
Project Name HPLC-07_JUNE-2024
Date Acquired: 2024-06-01 13:16:26+05:30
Date Processed: 2024-06-01 14:05:44+05:30

Instrument Name HPLC-07
Acq. method: o2h_HPLC_Method-D.amx
Processing method: *3D UV Quantitative_DefaultMethod.pmx
Column: XBridge C18 150x4.6mm, 3.5um

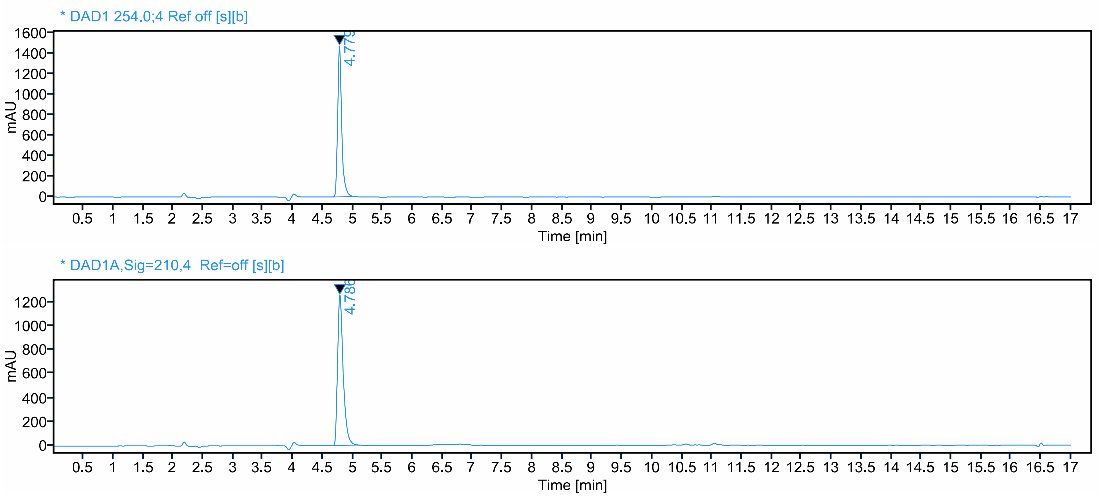

Signal: * DAD1 254.0;4 Ref off [s][b]

RT [min]	Height	Area	Area%
4.779	1470	6915	100.00

Signal: * DAD1A,Sig=210,4 Ref=off [s][b]

RT [min]	Height	Area	Area%
4.786	1254	8152	100.00

HPLC and LCMS Traces for Compound 4b

o2h discovery
Ahmedabad, Gujarat
India

LCMS Analysis Report

**Sample name:**	CIN-C-375-CIN-X-0009-037-b		
**Location:**	P2-B5	**Instrument Name**	LCMS-09
**Injection:**	1 of 1	**Acq. method:**	o2h_LCMS_Method_J.amx
**Injection volume:**	10.000	**Processing method:**	*LC_MS Sample Purity_DefaultMethod_NEW.pmx
**Project Name**	LCMS-09_JUNE-2024	**Description:**	
**Date Acquired:**	2024-06-22 20:55:07+05:30	**Column:**	XBridge C18 150x4.6mm,3.5um
**Date Processed:**	2024-06-22 22:03:52+05:30		

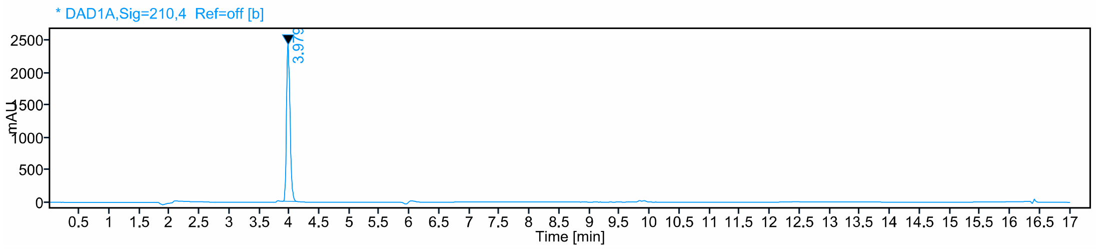

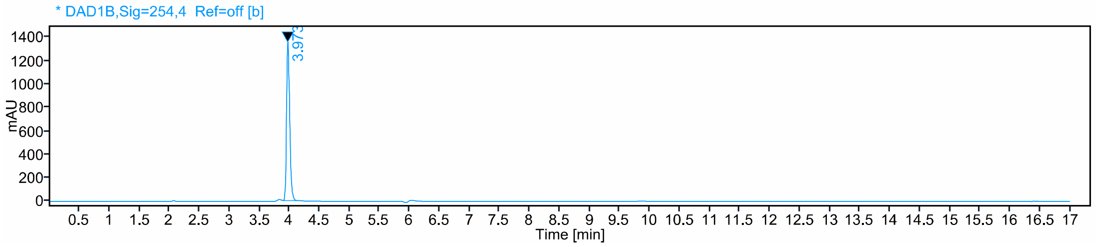

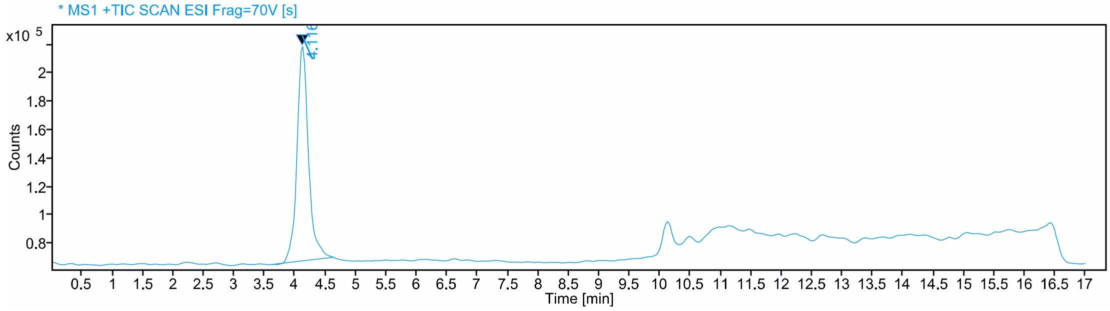

HPLC and LCMS Traces for Compound 4b

o2h discovery
Ahmedabad, Gujarat
India

LCMS Analysis Report

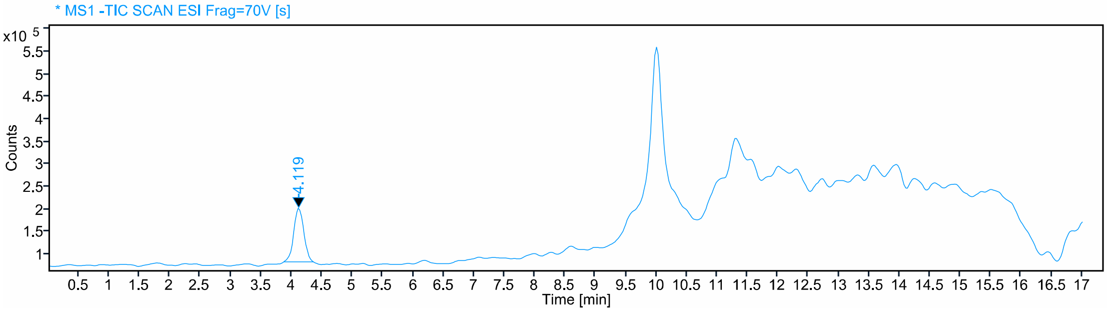

Signal: * DAD1A,Sig=210,4 Ref=off [b]

RT [min]	Base Peak (m/z)	Height	Area	Area%
3.979		2414	9966	100.00

Signal: * DAD1B,Sig=254,4 Ref=off [b]

RT [min]	Base Peak (m/z)	Height	Area	Area%
3.973		1349	4940	100.00

Signal: * MS1 +TIC SCAN ESI Frag=70V [s]

RT [min]	Base Peak (m/z)	Height	Area	Area%
4.116	559.500	151601	1987290	100.00

Signal: * MS1 -TIC SCAN ESI Frag=70V [s]

RT [min]	Base Peak (m/z)	Height	Area	Area%
4.119	278.300	119252	1370313	100.00

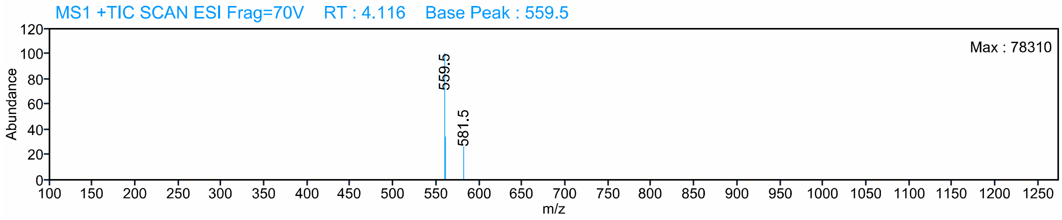

Printed: 2024-06-22 22:04:17+05:30 **Page 2 of 3**

HPLC and LCMS Traces for Compound 4b

o2h discovery
Ahmedabad, Gujarat
India

LCMS Analysis Report

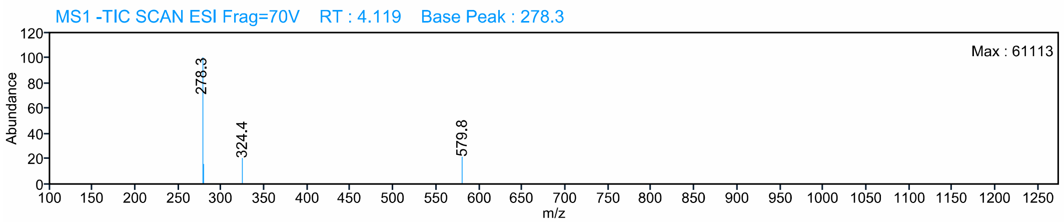

HPLC and LCMS Traces for Compound 4b

o2h discovery
Ahmedabad, Gujarat
India

HPLC Analysis Report

Sample name: CIN-C-375-CIN-X-0009-037-b
Location: P2-A3
Injection: 1 of 1
Injection volume: 10.000
Project Name HPLC-07_JUNE-2024
Date Acquired: 2024-06-22 18:47:00+05:30
Date Processed: 2024-06-22 19:21:54+05:30

Instrument Name HPLC-07
Acq. method: o2h_HPLC_Method-D.amx
Processing method: 3D UV Quantitative_DefaultMethod.pmx
Column: XBridge C18 150x4.6mm, 3.5um

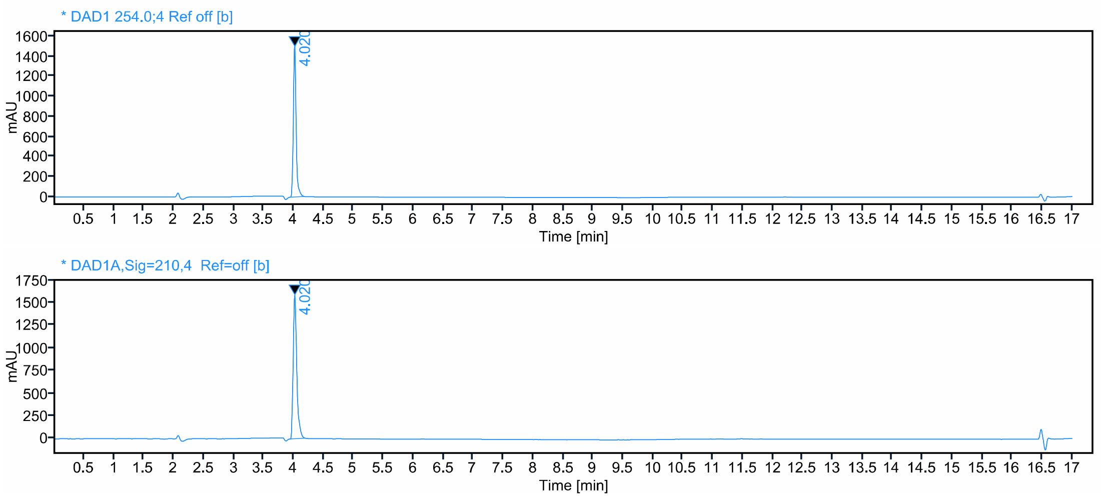

Signal: * DAD1 254.0;4 Ref off [b]

RT [min]	Height	Area	Area%
4.020	1494	4911	100.00

Signal: * DAD1A,Sig=210,4 Ref=off [b]

RT [min]	Height	Area	Area%
4.020	1578	6647	100.00

HPLC and LCMS Traces for Compound 4c

o2h discovery
Ahmedabad, Gujarat
India

LCMS Analysis Report

**Sample name:**	CIN-C-375-CIN-X-0008-048-a		
**Location:**	P2-A9	**Instrument Name**	LCMS-09
**Injection:**	1 of 1	**Acq. method:**	o2h_LCMS_Method_J.amx
**Injection volume:**	20.000	**Processing method:**	*LC_MS Sample Purity_DefaultMethod_NEW.pmx
**Project Name**	LCMS-09_JULY-2024	**Description:**	
**Date Acquired:**	2024-07-06 00:26:39+05:30	**Column:**	XBridge C18 150x4.6mm,3.5um
**Date Processed:**	2024-07-10 12:44:20+05:30		

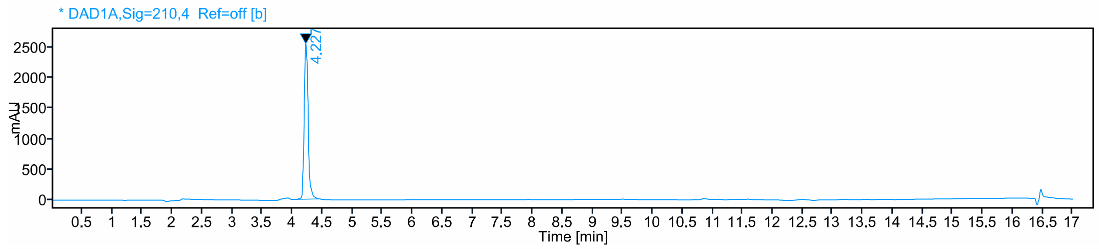

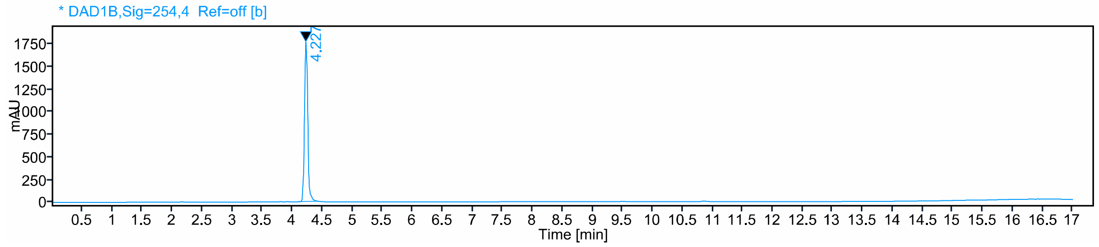

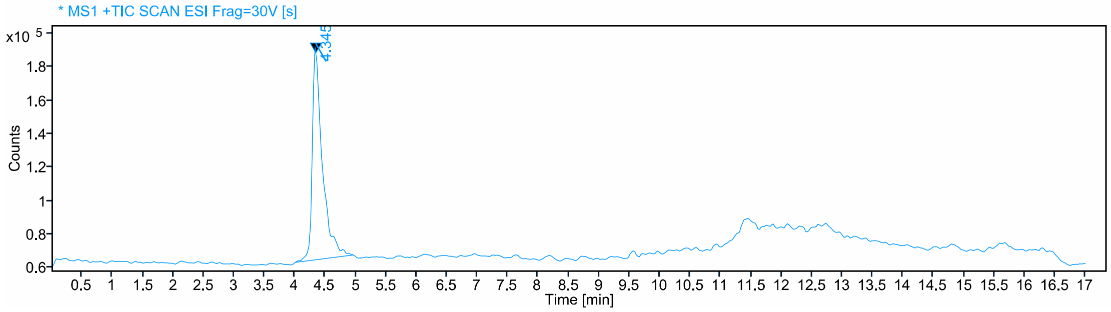

Signal: * DAD1A,Sig=210,4 Ref=off [b]

RT [min]	Base Peak (m/z)	Height	Area	Area%
4.227		2538	11647	100.00

HPLC and LCMS Traces for Compound 4c

o2h discovery
Ahmedabad, Gujarat
India

LCMS Analysis Report

Signal: * DAD1B,Sig=254,4 Ref=off [b]

RT [min]	Base Peak (m/z)	Height	Area	Area%
4.227		1757	6618	100.00

Signal: * MS1 +TIC SCAN ESI Frag=30V [s]

RT [min]	Base Peak (m/z)	Height	Area	Area%
4.345	559.600	123436	1460578	100.00

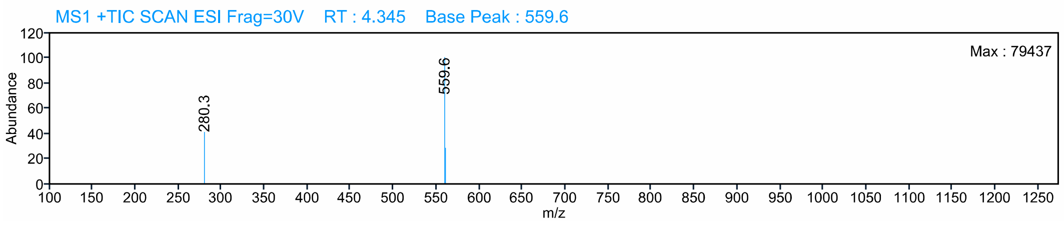

HPLC and LCMS Traces for Compound 4c

o2h discovery
Ahmedabad, Gujarat
India

HPLC Analysis Report

Sample name: CIN-C-375-CIN-X-0008-048-a
Location: P2-B2
Injection: 1 of 1
Injection volume: 20.000
Project Name HPLC-07_JUL-2024
Date Acquired: 2024-07-05 13:05:24+05:30
Date Processed: 2024-07-10 12:40:43+05:30

Instrument Name HPLC-07
Acq. method: o2h_HPLC_Method-D.amx
Processing method: *3D UV Quantitative_DefaultMethod.pmx
Column: XBridge C18 150x4.6mm, 3.5um

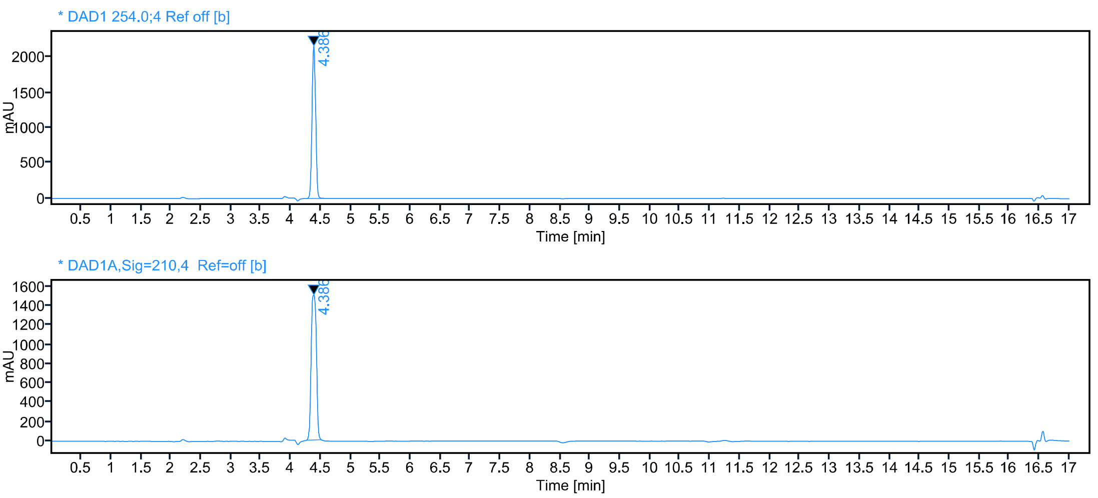

Signal: * DAD1 254.0;4 Ref off [b]

RT [min]	Height	Area	Area%
4.386	2153	9248	100.00

Signal: * DAD1A,Sig=210,4 Ref=off [b]

RT [min]	Height	Area	Area%
4.386	1497	9215	100.00

HPLC and LCMS Traces for Compound 4d

o2h discovery
Ahmedabad, Gujarat
India

LCMS Analysis Report

Sample Name:	CIN2-C-816-CIN2-X-0058-072-E		Injection Id 46162
Sample Type:	Unknown	Acquired By:	LCMS-05
Vial:	1:F,4	Sample Set Name:	19122024_UCH_NEW_FD
Injection #:	1	Acq. Method Set:	o2h_LCMS_Method_A_SLOW_01
Injection Volume:	3.00 ul	Processing Method	O2H_LCMS_02,
Run Time:	4.0 Minutes	Channel Name:	MS TIC, 254.0nm, 210.0nm
Project Name:	2024\LCMS-05_DEC-2024_	Proc. Chnl. Descr.:	QDa 1: MS Scan MS TIC,
Date Acquired:	20-12-2024 02:12:36 IST		
Date Processed:	20-12-2024 02:19:42 IST, 20-12-2024 02:19:46 IST, 20-12-2024 02:20:06 IST		
Column:	X-BRIDGE C18 2.1X50mm 2.5um		

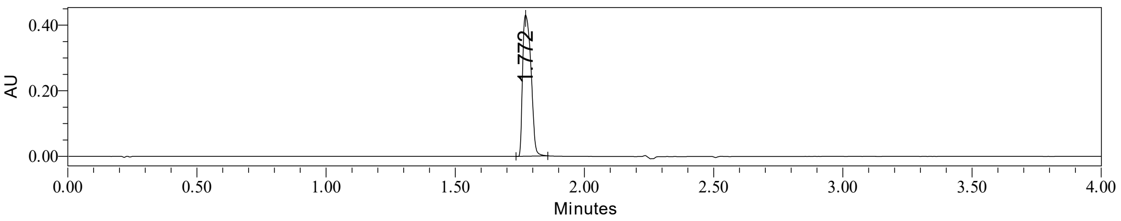

Channel Name 254.0nm; Channel PDA Spectrum

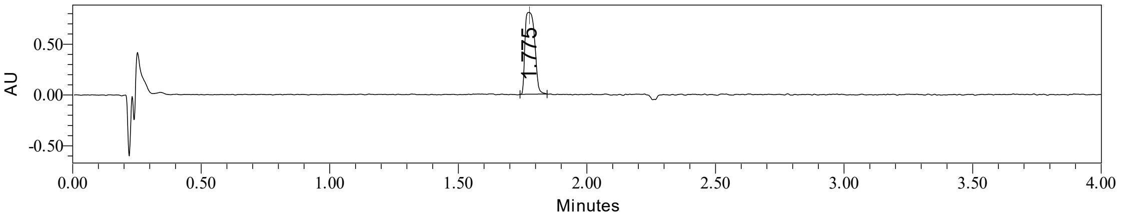

Channel Name 210.0nm; Channel PDA Spectrum

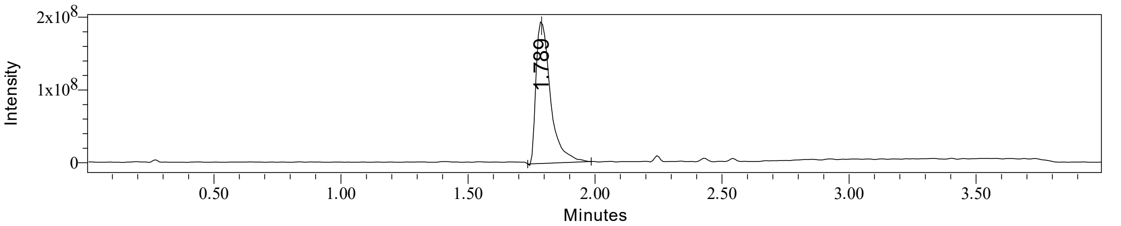

Channel Name MS TIC; Channel QDa 1: MS Scan

Peak Results
Channel: PDA Spectrum

	RT	Base Peak (m/z)	Height	Area	% Area	Channel	Channel Name
1	1.772		431266	967872	100.00	PDA Spectrum	254.0nm
2	1.775		805847	2102721	100.00	PDA Spectrum	210.0nm

Peak Results
Channel: QDa 1: MS Scan

	RT	Base Peak (m/z)	Height	Area	% Area	Channel	Channel Name
1	1.789	559.23	197819237	772051122	100.00	QDa 1: MS Scan	MS TIC

HPLC and LCMS Traces for Compound 4d

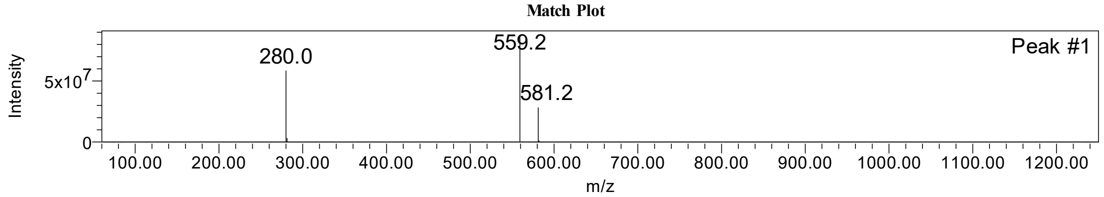

Base Peak 559.23 Channel Description 1: QDa Positive(+) Scan (60.00-1240.00)Da, Centroid, CV=10 - AVG (0.2:1.5;2.2:4.0) x 30.000 Th: 0.010 Retention Time 1.789

HPLC and LCMS Traces for Compound 4d

o2h discovery
Ahmedabad, Gujarat
India

HPLC Analysis Report

**Sample name:**	CIN2-C-816-CIN2-X-0058-072-E		
**Location:**	P2-A6	**Instrument Name**	HPLC-10
**Injection:**	1 of 1	**Acq. method:**	o2h_HPLC_Method-D.amx
**Injection volume:**	20.000	**Processing method:**	*3D UV Quantitative_DefaultMethod.pmx
**Project Name**	HPLC-10_DEC-2024	**Column:**	XBridge C18 150x4.6mm, 3.5um
**Date Acquired:**	2024-12-19 18:13:40+05:30		
**Date Processed:**	2024-12-19 20:16:17+05:30		

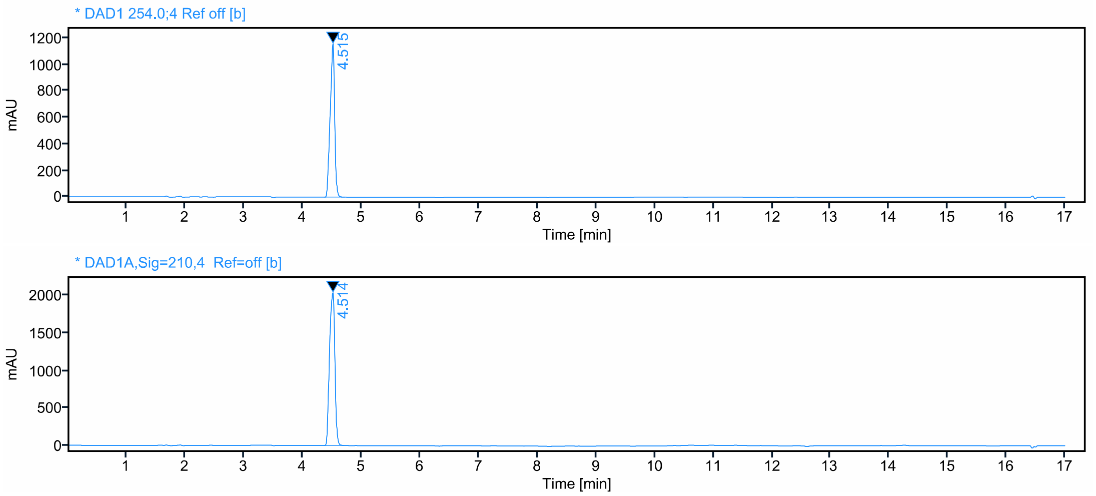

Signal: * DAD1 254.0;4 Ref off [b]

RT [min]	Height	Area	Area%
4.515	1160	6214	100.00

Signal: * DAD1A,Sig=210,4 Ref=off [b]

RT [min]	Height	Area	Area%
4.514	2046	13126	100.00

Printed: 2024-12-19 20:16:32+05:30 **Page 1 of 1**

HPLC and LCMS Traces for Compound 4e

o2h discovery
Ahmedabad, Gujarat
India

LCMS Analysis Report

Sample Name:	CIN2-D-051-CIN2-X-0060-003-F		Injection Id 17658
Sample Type:	Unknown	Acquired By:	LCMS-05
Vial:	1:A,8	Sample Set Name:	09122024_UCH_083_RD
Injection #:	1	Acq. Method Set:	o2h_LCMS_Method_A
Injection Volume:	2.00 ul	Processing Method	O2H_LCMS_02,
Run Time:	4.0 Minutes	Channel Name:	MS TIC, 254.0nm, 210.0nm
Project Name:	2024\LCMS-05_DEC-2024_	Proc. Chnl. Descr.:	QDa 1: MS Scan MS TIC,
Date Acquired:	09-12-2024 10:06:41 IST		
Date Processed:	09-12-2024 10:31:39 IST, 09-12-2024 10:31:45 IST, 09-12-2024 10:32:11 IST		
Column:	X-BRIDGE C18 2.1X50mm 2.5um		

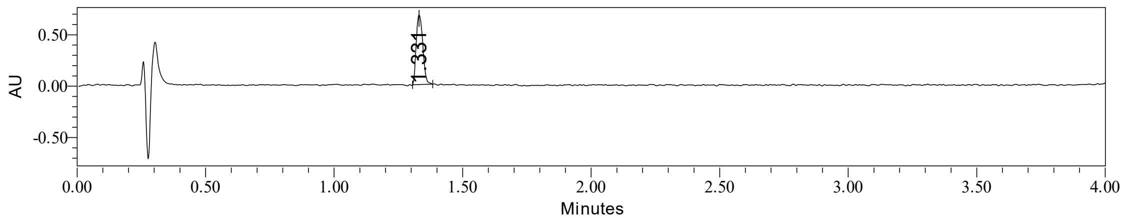

Channel Name 210.0nm; Channel PDA Spectrum

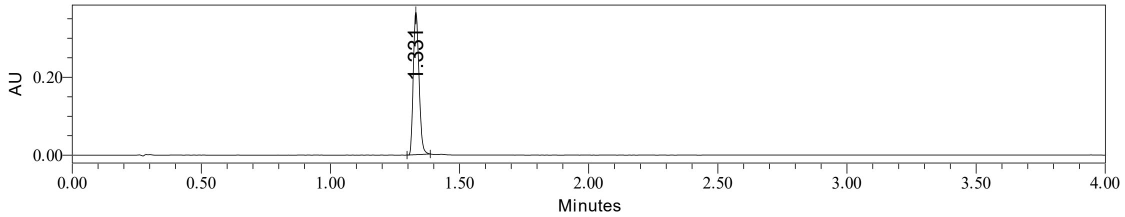

Channel Name 254.0nm; Channel PDA Spectrum

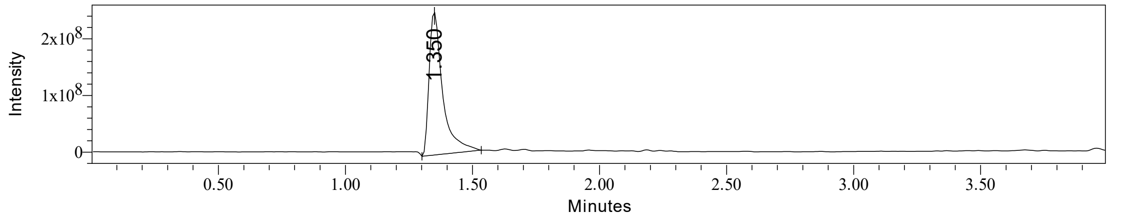

Channel Name MS TIC; Channel QDa 1: MS Scan

Peak Results
Channel: PDA Spectrum

	RT	Base Peak (m/z)	Height	Area	% Area	Channel	Channel Name
1	1.331		679109	1203722	100.00	PDA Spectrum	210.0nm
2	1.331		366122	562263	100.00	PDA Spectrum	254.0nm

Peak Results
Channel: QDa 1: MS Scan

	RT	Base Peak (m/z)	Height	Area	% Area	Channel	Channel Name
1	1.350	527.28	251126682	936230367	100.00	QDa 1: MS Scan	MS TIC

HPLC and LCMS Traces for Compound 4e

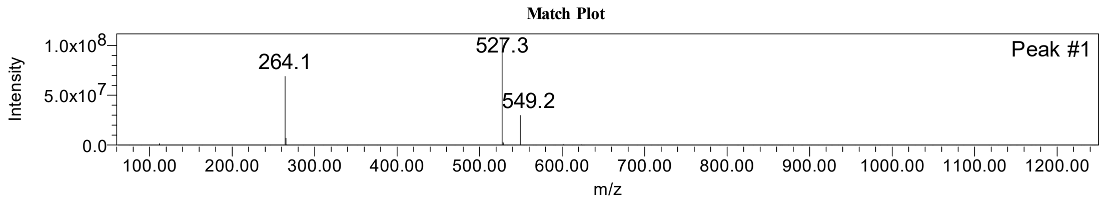

Base Peak 527.28 Channel Description 1: QDa Positive(+) Scan (60.00-1250.00)Da, Centroid, CV=10 - AVG (2.6:3.4;0.2:0.5) x 20.000 Retention Time 1.350

HPLC and LCMS Traces for Compound 4e

o2h discovery<br>Ahmedabad, Gujarat<br>India

HPLC Analysis Report

**Sample name:**	CIN2-D-051-CIN2-X-0060-003-F		
**Location:**	P2-B1	**Instrument Name**	HPLC-10
**Injection:**	1 of 1	**Acq. method:**	o2h_HPLC_Method-D.amx
**Injection volume:**	10.000	**Processing method:**	*3D UV Quantitative_DefaultMethod.pmx
**Project Name**	HPLC-10_DEC-2024	**Column:**	XBridge C18 150x4.6mm, 3.5um
**Date Acquired:**	2024-12-09 13:26:21+05:30		
**Date Processed:**	2024-12-09 14:12:10+05:30		

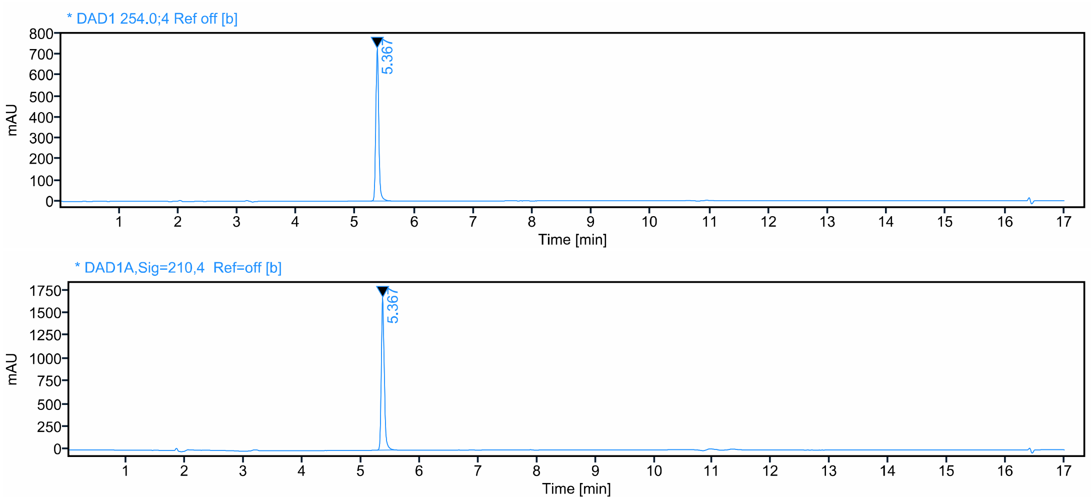

Signal: * DAD1 254.0;4 Ref off [b]

RT [min]	Height	Area	Area%
5.367	728	2453	100.00

Signal: * DAD1A,Sig=210,4 Ref=off [b]

RT [min]	Height	Area	Area%
5.367	1675	5931	100.00

Printed: 2024-12-09 14:16:00+05:30 **Page 1 of 1**

HPLC and LCMS Traces for Compound 4f

o2h discovery<br>Ahmedabad, Gujarat<br>India	**LCMS Analysis Report**

Sample Name:	CIN2-C-818-CIN2-X-0024-044-f		Injection Id 57261
Sample Type:	Unknown	Acquired By:	LCMS-05
Vial:	2:F,3	Sample Set Name:	26112024_UCH_090_FD_01
Injection #:	1	Acq. Method Set:	o2h_LCMS_Method_A_SLOW_01
Injection Volume:	2.00 ul	Processing Method	O2H_LCMS_02_0,
Run Time:	4.0 Minutes	Channel Name:	MS TIC, 254.0nm, 210.0nm
Project Name:	2024\LCMS-05_NOV-2024	Proc. Chnl. Descr.:	QDa 1: MS Scan MS TIC,
Date Acquired:	26-11-2024 15:30:40 IST		
Date Processed:	26-11-2024 15:42:54 IST, 26-11-2024 15:43:13 IST, 26-11-2024 15:43:49 IST		
Column:	X-BRIDGE C18 2.1X50mm 2.5um		

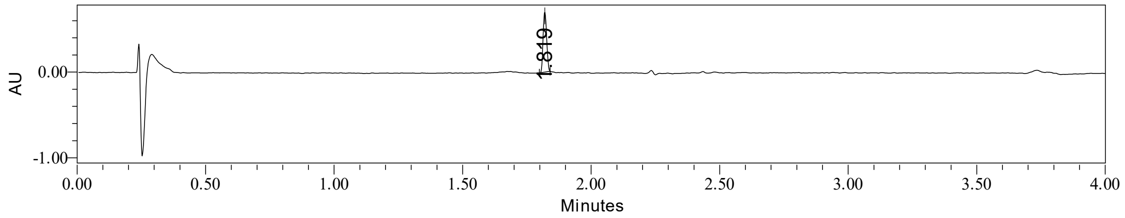

Channel PDA Spectrum; Channel Name 210.0nm

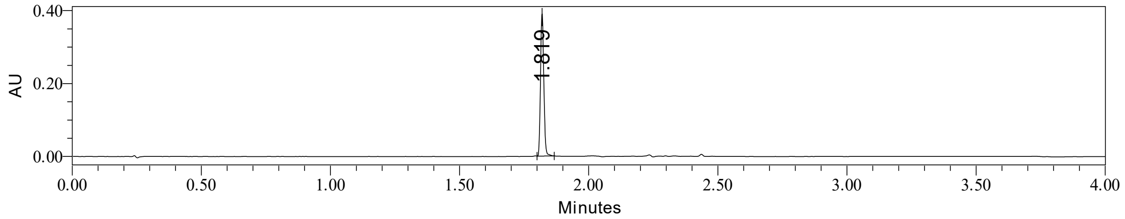

Channel PDA Spectrum; Channel Name 254.0nm

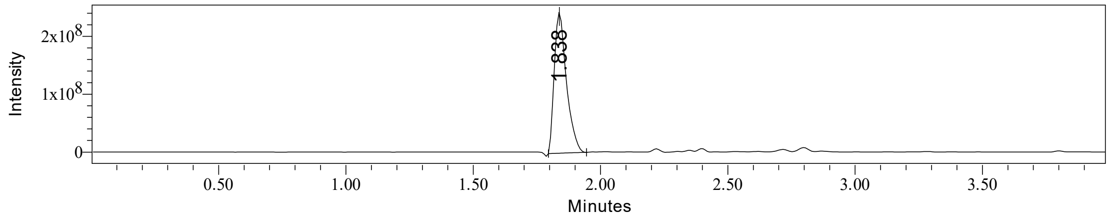

Channel QDa 1: MS Scan; Channel Name MS TIC

Peak Results
Channel: PDA Spectrum

	RT	Base Peak (m/z)	Height	Area	% Area	Channel	Channel Name
1	1.819		391480	345605	100.00	PDA Spectrum	254.0nm
2	1.819		699238	672666	100.00	PDA Spectrum	210.0nm

Peak Results
Channel: QDa 1: MS Scan

	RT	Base Peak (m/z)	Height	Area	% Area	Channel	Channel Name
1	1.838	527.29	242353998	806602319	100.00	QDa 1: MS Scan	MS TIC

HPLC and LCMS Traces for Compound 4f

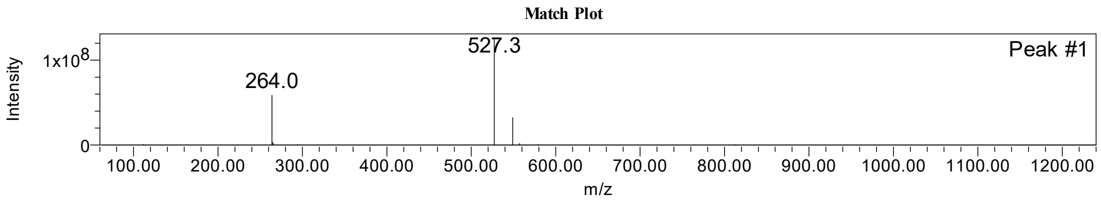

Base Peak 527.29 Channel Description 1: QDa Positive(+) Scan (60.00-1240.00)Da, Centroid, CV=10 - AVG (0.1:1.5;1.9:3.8) x 30.000 Th: 0.010 - AVG (0.1:1.5;1.9:3.8;2.1:3.7) x 30.000 Th: 0.010 Retention Time 1.838

HPLC and LCMS Traces for Compound 4f

o2h discovery<br>Ahmedabad, Gujarat<br>India	**HPLC Analysis Report**

Sample name: CIN2-C-818-CIN2-X-0024-044-f
Location: P2-B3
Injection: 1 of 1
Injection volume: 20.000
Project Name HPLC_07_NOV-2024
Date Acquired: 2024-11-26 15:43:32+05:30
Date Processed: 2024-11-26 16:02:16+05:30

Instrument Name HPLC-07
Acq. method: o2h_HPLC_Method-D.amx
Processing method: *3D UV Quantitative_DefaultMethod.pmx
Column: XBridge C18 150x4.6mm, 3.5um

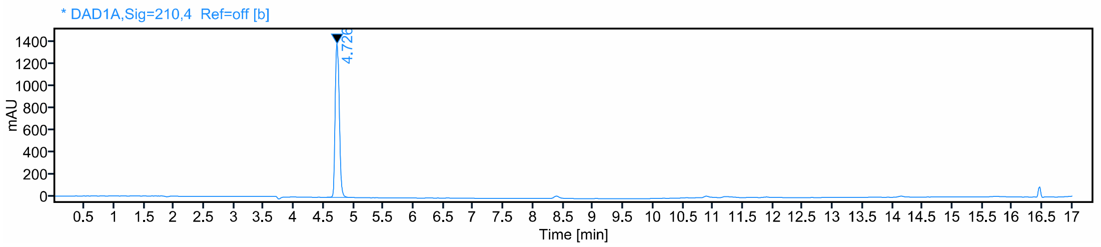

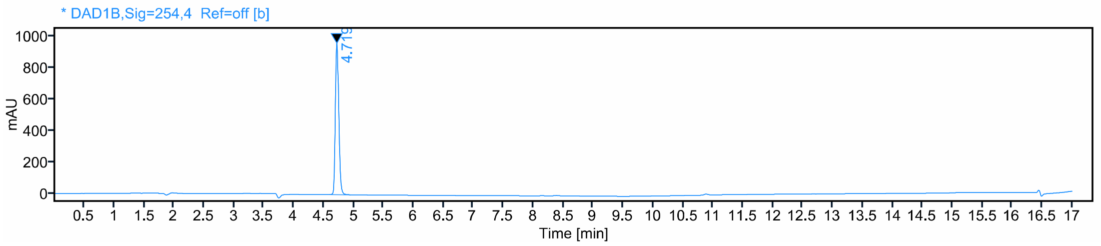

Signal: * DAD1A,Sig=210,4 Ref=off [b]

RT [min]	Height	Area	Area%
4.726	1393	6649	100.00

Signal: * DAD1B,Sig=254,4 Ref=off [b]

RT [min]	Height	Area	Area%
4.719	955	3795	100.00

HPLC and LCMS Traces for Compound 4g

o2h discovery
Ahmedabad, Gujarat
India

LCMS Analysis Report

Sample Name:	CIN2-C-816-CIN2-X-0016-042-A		Injection Id 35236
Sample Type:	Unknown	Acquired By:	LCMS-05
Vial:	2:F,5	Sample Set Name:	18112024_UCH_090_FD
Injection #:	1	Acq. Method Set:	o2h_LCMS_Method_A_SLOW_01
Injection Volume:	4.00 ul	Processing Method	O2H_LCMS_02_0,
Run Time:	4.0 Minutes	Channel Name:	MS TIC, 254.0nm, 210.0nm
Project Name:	2024\LCMS-05_NOV-2024	Proc. Chnl. Descr.:	QDa 1: MS Scan MS TIC,
Date Acquired:	18-11-2024 12:56:58 IST		
Date Processed:	18-11-2024 13:20:04 IST, 18-11-2024 13:20:50 IST, 18-11-2024 13:22:51 IST		
Column:	X-BRIDGE C18 2.1X50mm 2.5um		

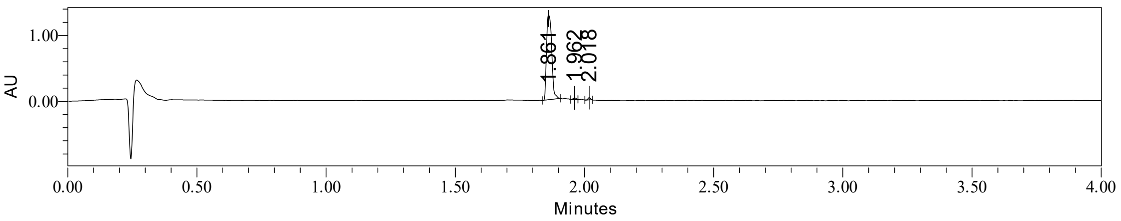

Channel PDA Spectrum; Channel Name 210.0nm

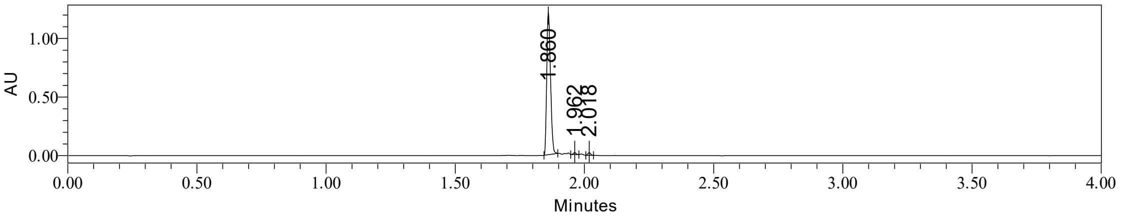

Channel PDA Spectrum; Channel Name 254.0nm

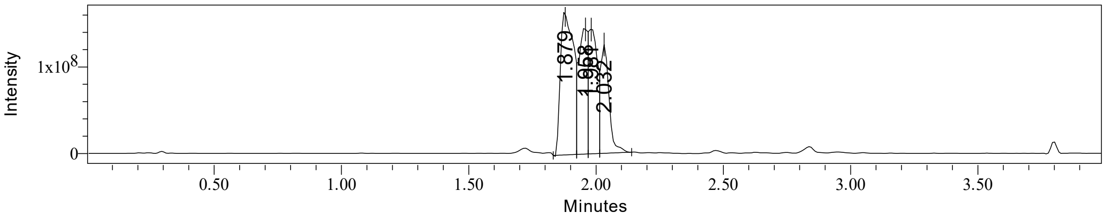

Channel QDa 1: MS Scan; Channel Name MS TIC

Peak Results
Channel: PDA Spectrum

	RT	Base Peak (m/z)	Height	Area	% Area	Channel	Channel Name
1	1.860		1213550	1229892	97.67	PDA Spectrum	254.0nm
2	1.861		1295010	1802210	97.72	PDA Spectrum	210.0nm
3	1.962		23167	18538	1.01	PDA Spectrum	210.0nm
4	1.962		16909	12249	0.97	PDA Spectrum	254.0nm
5	2.018		22782	17048	1.35	PDA Spectrum	254.0nm
6	2.018		33920	23420	1.27	PDA Spectrum	210.0nm

HPLC and LCMS Traces for Compound 4g

Peak Results
Channel: QDa 1: MS Scan

	RT	Base Peak (m/z)	Height	Area	% Area	Channel	Channel Name
1	1.879	563.20	168088075	581623861	37.11	QDa 1: MS Scan	MS TIC
2	1.958	290.00	146627781	352241306	22.48	QDa 1: MS Scan	MS TIC
3	1.981	263.98	143855107	345269647	22.03	QDa 1: MS Scan	MS TIC
4	2.032	264.01	125681795	288063687	18.38	QDa 1: MS Scan	MS TIC

Match Plot

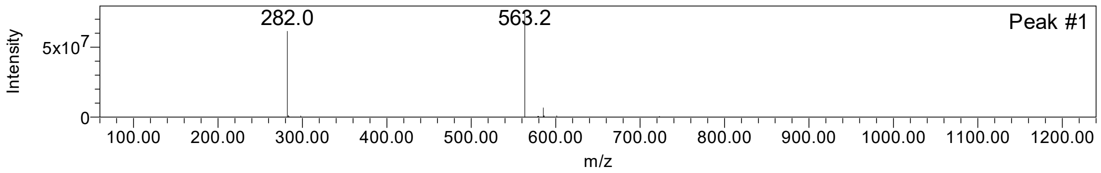

Base Peak 563.20 Channel Description 1: QDa Positive(+) Scan (60.00-1240.00)Da, Centroid, CV=10 - AVG (0.2:1.1;3.1:3.8) x 20.000 Th: 0.010 - AVG (2.3:2.4;2.3:2.4) x 20.000 Th: 0.010 Retention Time 1.879

Match Plot

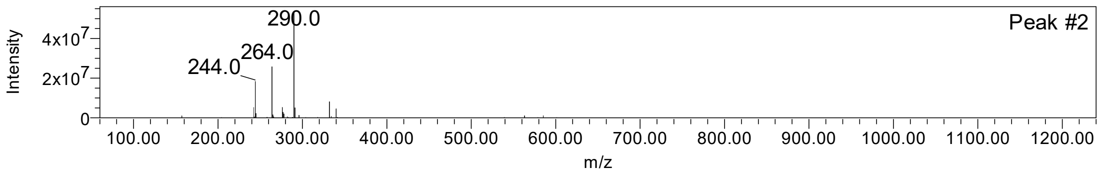

Base Peak 290.00 Channel Description 1: QDa Positive(+) Scan (60.00-1240.00)Da, Centroid, CV=10 - AVG (0.2:1.1;3.1:3.8) x 20.000 Th: 0.010 - AVG (2.3:2.4;2.3:2.4) x 20.000 Th: 0.010 Retention Time 1.958

Match Plot

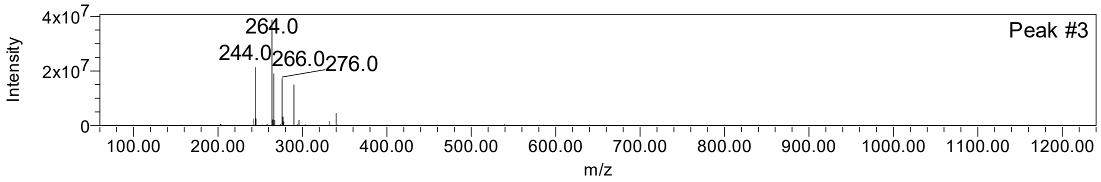

Base Peak 263.98 Channel Description 1: QDa Positive(+) Scan (60.00-1240.00)Da, Centroid, CV=10 - AVG (0.2:1.1;3.1:3.8) x 20.000 Th: 0.010 - AVG (2.3:2.4;2.3:2.4) x 20.000 Th: 0.010 Retention Time 1.981

Match Plot

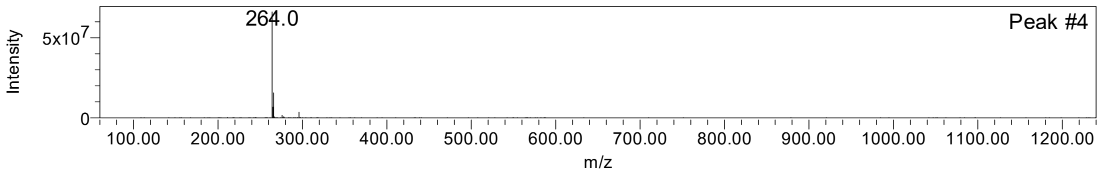

Base Peak 264.01 Channel Description 1: QDa Positive(+) Scan (60.00-1240.00)Da, Centroid, CV=10 - AVG (0.2:1.1;3.1:3.8) x 20.000 Th: 0.010 - AVG (2.3:2.4;2.3:2.4) x 20.000 Th: 0.010 Retention Time 2.032

HPLC and LCMS Traces for Compound 4g

o2h discovery
Ahmedabad, Gujarat
India

HPLC Analysis Report

Sample name: CIN2-C-816-CIN2-X-0016-042-A
Location: P2-A6
Injection: 1 of 1
Injection volume: 15.000
Project Name HPLC_07_NOV-2024
Date Acquired: 2024-11-18 17:21:57+05:30
Date Processed: 2024-11-18 19:32:21+05:30

Instrument Name HPLC-07
Acq. method: o2h_HPLC_Method-D.amx
Processing method: *3D UV Quantitative_DefaultMethod.pmx
Column: XBridge C18 150x4.6mm, 3.5um

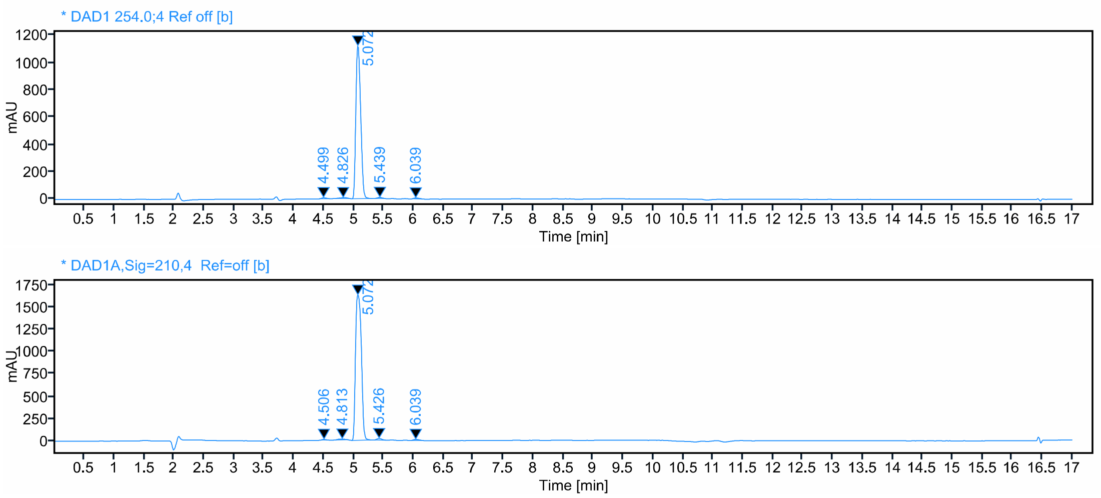

Signal: * DAD1 254.0;4 Ref off [b]

RT [min]	Height	Area	Area%
4.499	8	39	0.61
4.826	7	59	0.92
5.072	1116	6184	97.19
5.439	8	42	0.67
6.039	7	39	0.61

Signal: * DAD1A,Sig=210,4 Ref=off [b]

RT [min]	Height	Area	Area%
4.506	5	20	0.18
4.813	8	64	0.58
5.072	1618	10879	97.68
5.426	17	93	0.84
6.039	12	80	0.72

HPLC and LCMS Traces for Compound 5a

o2h discovery
Ahmedabad, Gujarat
India

LCMS Analysis Report

Sample Name:	CIN-C-377-CIN-X-0025-021-c		Injection Id 19852
Sample Type:	Unknown	Acquired By:	LCMS-05
Vial:	1:D,5	Sample Set Name:	13052024_UCH_077_FD
Injection #:	1	Acq. Method Set:	o2h_LCMS_Method_A_SLOW
Injection Volume:	2.00 ul	Processing Method:	O2H_LCMS_02_000,
Run Time:	4.0 Minutes	Channel Name:	220.0nm, MS TIC, 254.0nm
Project Name:	2024\LCMS-05_MAY-2024	Proc. Chnl. Descr.:	PDA 220.0 nm Blank Subtracted
Date Acquired:	13-05-2024 11:51:06 IST		
Date Processed:	13-05-2024 11:55:43 IST, 13-05-2024 11:55:56 IST, 13-05-2024 11:57:38 IST		
Column:	X-BRIDGE C18 2.1X50mm 2.5um		

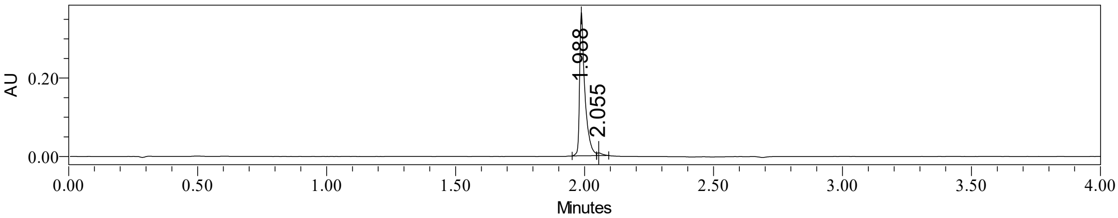

Channel Name 254.0nm; Channel PDA Spectrum

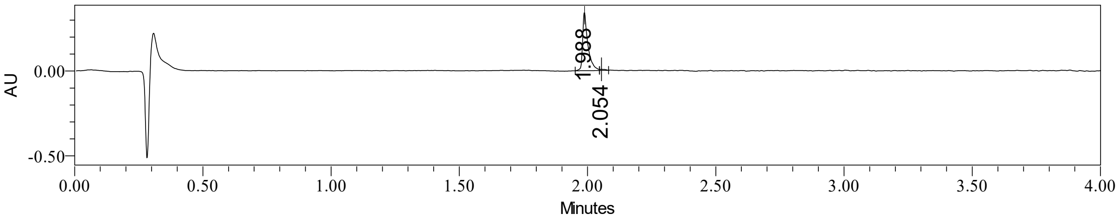

Channel Name 220.0nm; Channel PDA Spectrum

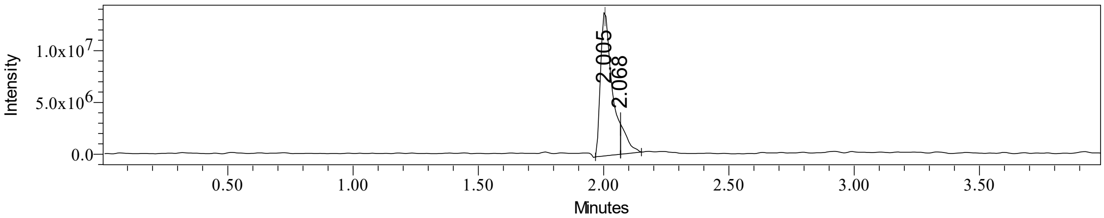

Channel Name MS TIC; Channel QDa 1: MS Scan

Peak Results
Channel: PDA Spectrum

	RT	Base Peak (m/z)	Height	Area	% Area	Channel	Channel Name
1	1.988		343168	489596	98.17	PDA Spectrum	220.0nm
2	1.988		366327	516652	98.07	PDA Spectrum	254.0nm
3	2.054		6433	9152	1.83	PDA Spectrum	220.0nm
4	2.055		7102	10175	1.93	PDA Spectrum	254.0nm

HPLC and LCMS Traces for Compound 5a

Peak Results
Channel: QDa 1: MS Scan

	RT	Base Peak (m/z)	Height	Area	% Area	Channel	Channel Name
1	2.005	304.15	13942223	42627697	88.75	QDa 1: MS Scan	MS TIC
2	2.068	304.17	2902917	5404223	11.25	QDa 1: MS Scan	MS TIC

Match Plot

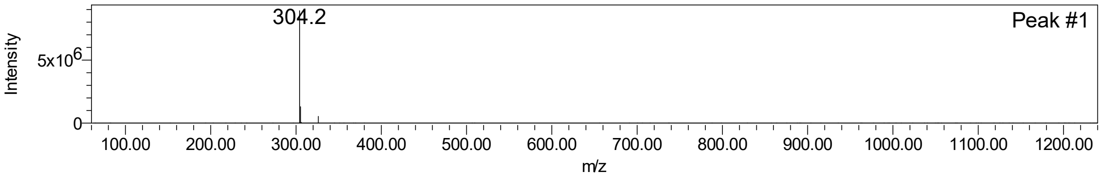

Base Peak 304.15 Channel Description 1: QDa Positive(+) Scan (60.00-1240.00)Da, Centroid, CV=10 - AVG (0.1:1.3;2.9:3.0;2.2:3.9) x 20.000 Th: 0.010 - AVG (2.3:2.6) x 20.000 Th: 0.010 Retention Time 2.005

Match Plot

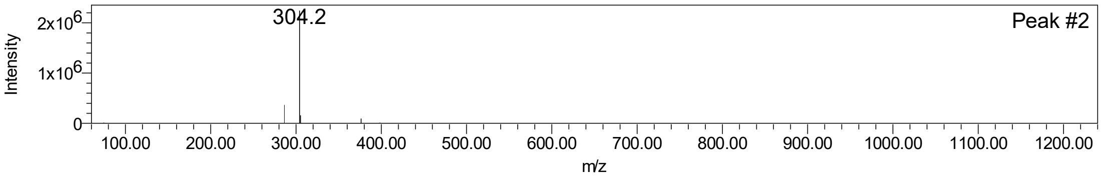

Base Peak 304.17 Channel Description 1: QDa Positive(+) Scan (60.00-1240.00)Da, Centroid, CV=10 - AVG (0.1:1.3;2.9:3.0;2.2:3.9) x 20.000 Th: 0.010 - AVG (2.3:2.6) x 20.000 Th: 0.010 Retention Time 2.068

HPLC and LCMS Traces for Compound 5a

o2h discovery
Ahmedabad, Gujarat
India

HPLC Analysis Report

**Sample name:**	CIN-C-377-CIN-X-0025-021-c		
**Location:**	P2-A7	**Instrument Name**	HPLC-10
**Injection:**	1 of 1	**Acq. method:**	o2h_HPLC_Method-C.amx
**Injection volume:**	10.000	**Processing method:**	*3D UV Quantitative_DefaultMethod.pmx
**Project Name**	HPLC-10_MAY-2024		
**Date Acquired:**	2024-05-13 14:46:32+05:30	**Column:**	SUNFIRE C18 150x4.6mm,3.5um
**Date Processed:**	2024-05-13 16:05:29+05:30		

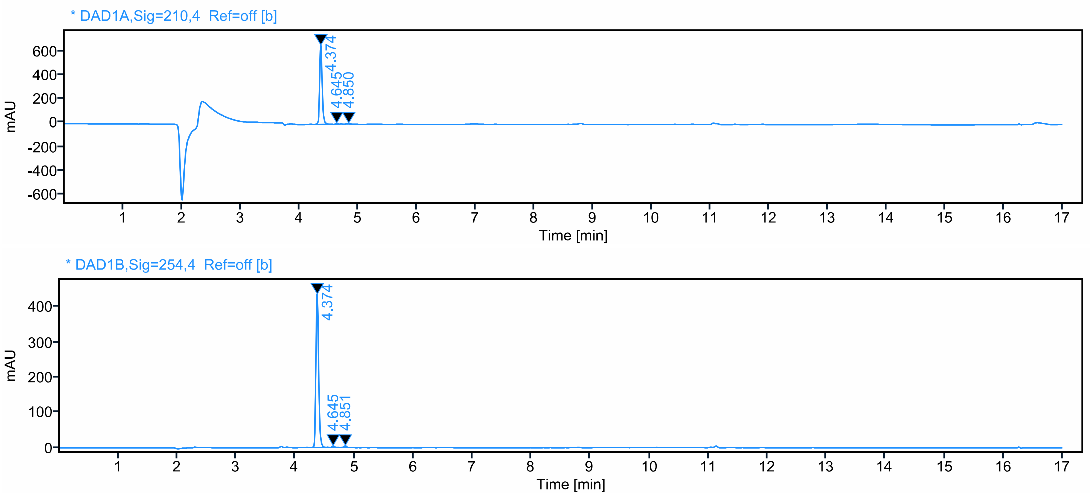

Signal: * DAD1A,Sig=210,4 Ref=off [b]

RT [min]	Height	Area	Area%
4.374	654	2055	98.72
4.645	6	16	0.79
4.850	4	10	0.49

Signal: * DAD1B,Sig=254,4 Ref=off [b]

RT [min]	Height	Area	Area%
4.374	433	1356	98.47
4.645	4	9	0.69
4.851	4	12	0.85

HPLC and LCMS Traces for Compound 5b

o2h discovery
Ahmedabad, Gujarat
India

LCMS Analysis Report

Sample Name:	CIN-C-377-CIN-X-0027-046-a		Injection Id 39184
Sample Type:	Unknown	Acquired By:	LCMS-05
Vial:	1:B,4	Sample Set Name:	23052024_UCH_082_FD
Injection #:	1	Acq. Method Set:	o2h_LCMS_Method_A_SLOW_01
Injection Volume:	2.00 ul	Processing Method:	O2H_LCMS_03_00,
Run Time:	4.0 Minutes	Channel Name:	MS TIC, 254.0nm, 210.0nm
Project Name:	2024\LCMS-05_MAY-2024	Proc. Chnl. Descr.:	PDA 254.0 nm Blank Subtracted
Date Acquired:	23-05-2024 11:36:38 IST		
Date Processed:	23-05-2024 11:44:47 IST, 23-05-2024 11:44:57 IST, 23-05-2024 11:45:34 IST		
Column:	X-BRIDGE C18 2.1X50mm 2.5um		

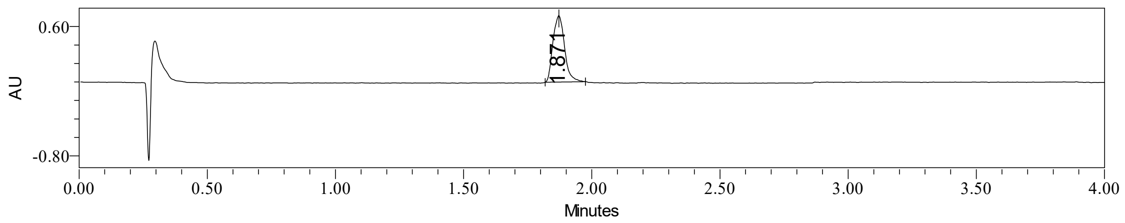

Channel PDA Spectrum; Channel Name 210.0nm

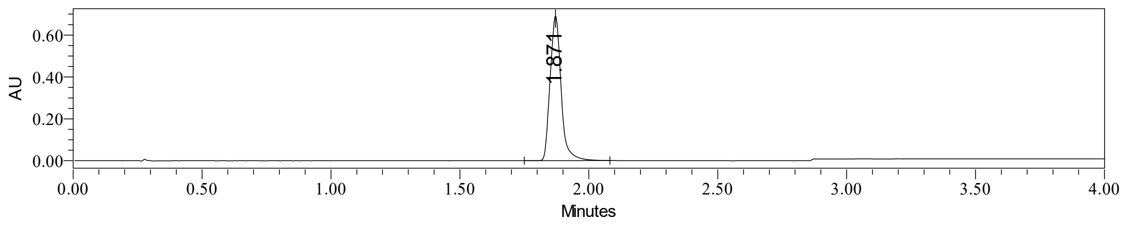

Channel PDA Spectrum; Channel Name 254.0nm

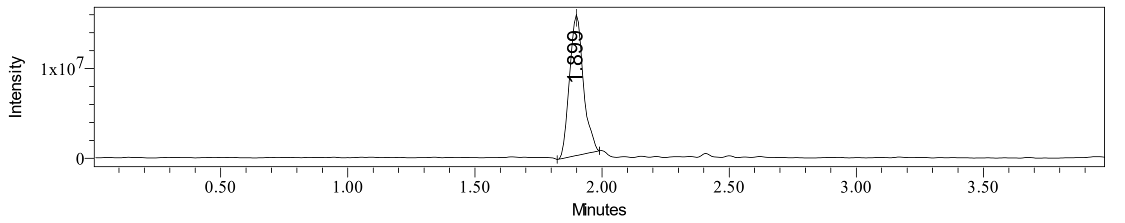

Channel QDa 1: MS Scan; Channel Name MS TIC

Peak Results
Channel: PDA Spectrum

	RT	Base Peak (m/z)	Height	Area	% Area	Channel	Channel Name
1	1.871		691451	2052245	100.00	PDA Spectrum	254.0nm
2	1.871		720069	2258322	100.00	PDA Spectrum	210.0nm

Peak Results
Channel: QDa 1: MS Scan

	RT	Base Peak (m/z)	Height	Area	% Area	Channel	Channel Name
1	1.899	304.17	15657420	59279369	100.00	QDa 1: MS Scan	MS TIC

HPLC and LCMS Traces for Compound 5b

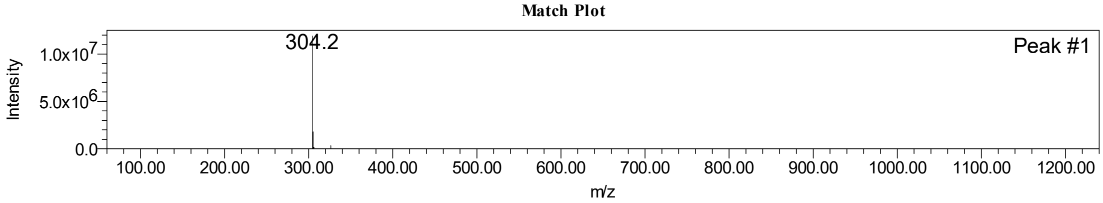

Base Peak 304.17 Channel Description 1: QDa Positive(+) Scan (60.00-1240.00)Da, Centroid, CV=10 - AVG (2.4:4.0;0.0:0.7) x 20.000 Th: 0.010 - AVG (2.1:3.1) x 20.000 Th: 0.010 Retention Time 1.899

HPLC and LCMS Traces for Compound 5b

o2h discovery
Ahmedabad, Gujarat
India

HPLC Analysis Report

**Sample name:**	CIN-C-377-CIN-X-0027-046-a		
**Location:**	P1-B6	**Instrument Name**	HPLC-10
**Injection:**	1 of 1	**Acq. method:**	o2h_HPLC_Method-C.amx
**Injection volume:**	40.000	**Processing method:**	*3D UV Quantitative_DefaultMethod.pmx
**Project Name**	HPLC-10_MAY-2024		
**Date Acquired:**	2024-05-22 19:21:20+05:30	**Column:**	SUNFIRE C18 150x4.6mm,3.5um
**Date Processed:**	2024-05-22 20:21:15+05:30		

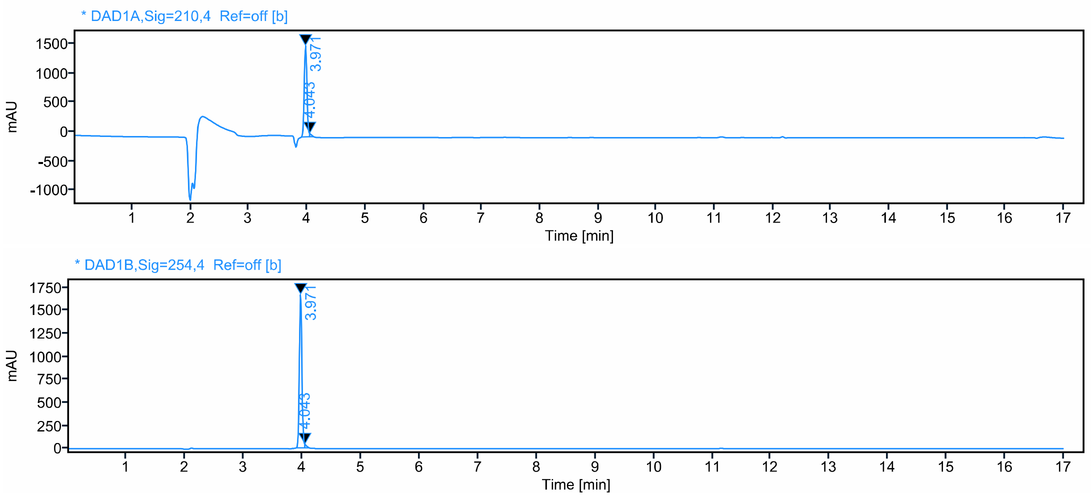

Signal: * DAD1A,Sig=210,4 Ref=off [b]

RT [min]	Height	Area	Area%
3.971	1553	5166	97.84
4.043	58	114	2.16

Signal: * DAD1B,Sig=254,4 Ref=off [b]

RT [min]	Height	Area	Area%
3.971	1662	5009	98.51
4.043	38	76	1.49

HPLC and LCMS Traces for Compound 5c

o2h discovery
Ahmedabad, Gujarat
India

LCMS Analysis Report

**Sample name:**	CIN-C-377-CIN-X-0026-063-a		
**Location:**	P2-B3	**Instrument Name**	LCMS-09
**Injection:**	1 of 1	**Acq. method:**	o2h_LCMS_Method_D.amx
**Injection volume:**	20.000	**Processing method:**	*LC_MS Sample Purity_DefaultMethod_NEW.pmx
**Project Name**	LCMS-09_JUNE-2024	**Description:**	
**Date Acquired:**	2024-06-11 17:21:02+05:30	**Column:**	SUNFIRE C18 150x4.6mm, 3.5um
**Date Processed:**	2024-06-11 17:42:18+05:30		

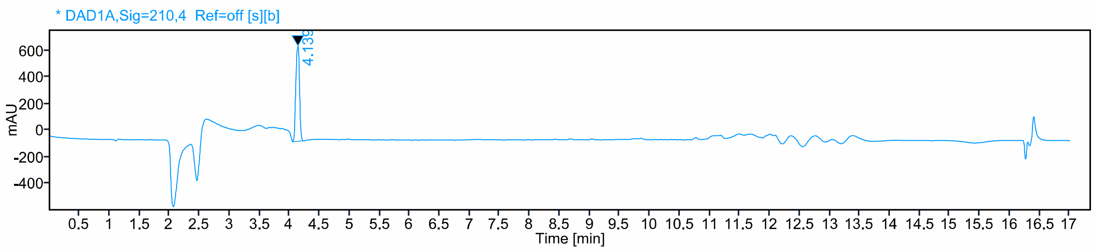

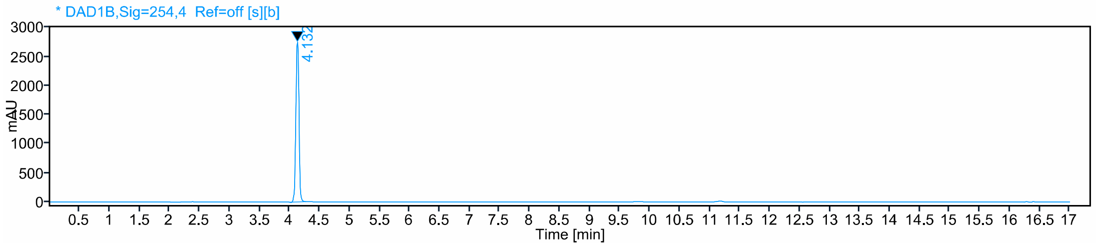

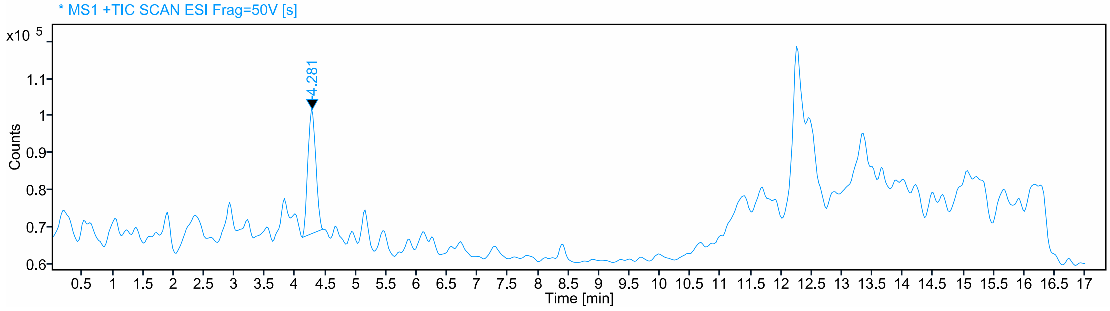

Signal: * DAD1A,Sig=210,4 Ref=off [s][b]

RT [min]	Base Peak (m/z)	Height	Area	Area%
4.139		714	3041	100.00

Printed: 2024-06-11 17:42:35+05:30 **Page 1 of 2**

HPLC and LCMS Traces for Compound 5c

o2h discovery
Ahmedabad, Gujarat
India

LCMS Analysis Report

Signal: * DAD1B,Sig=254,4 Ref=off [s][b]

RT [min]	Base Peak (m/z)	Height	Area	Area%
4.132		2750	9756	100.00

Signal: * MS1 +TIC SCAN ESI Frag=50V [s]

RT [min]	Base Peak (m/z)	Height	Area	Area%
4.281	304.400	33325	302047	100.00

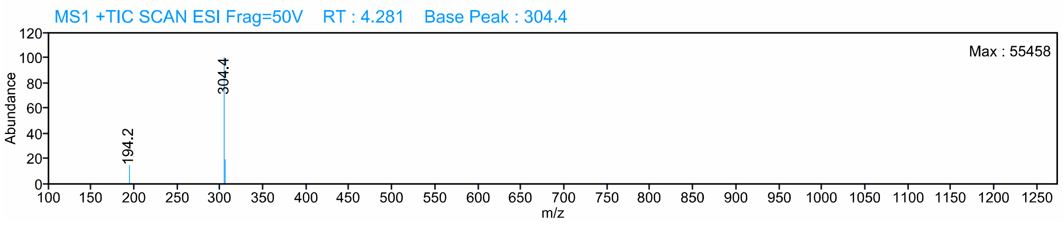

Printed: 2024-06-11 17:42:35+05:30 **Page 2 of 2**

HPLC and LCMS Traces for Compound 5c

o2h discovery
Ahmedabad, Gujarat
India

HPLC Analysis Report

Sample name: CIN-C-377-CIN-X-0026-063-a
Location: P1-B10
Injection: 1 of 1
Injection volume: 10.000
Project Name HPLC-10_JUNE-2024
Date Acquired: 2024-06-08 21:57:21+05:30
Date Processed: 2024-06-08 22:36:16+05:30

Instrument Name HPLC-10
Acq. method: o2h_HPLC_Method-C.amx
Processing method: *3D UV Quantitative_DefaultMethod.pmx
Column: SUNFIRE C18 150x4.6mm,3.5um

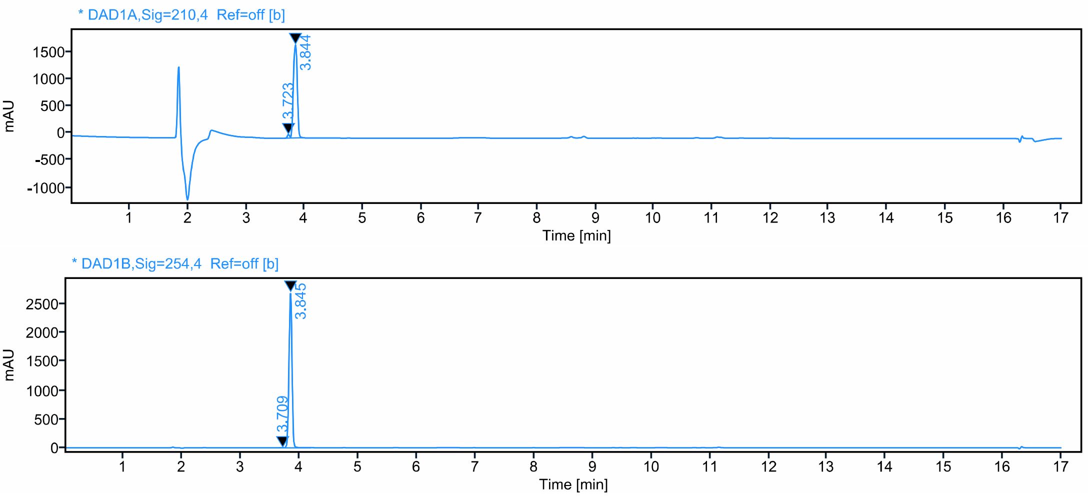

Signal: * DAD1A,Sig=210,4 Ref=off [b]

RT [min]	Height	Area	Area%
3.723	65	179	2.27
3.844	1715	7727	97.73

Signal: * DAD1B,Sig=254,4 Ref=off [b]

RT [min]	Height	Area	Area%
3.709	1	3	0.03
3.845	2709	9817	99.97

HPLC and LCMS Traces for Compound 5d

o2h discovery
Ahmedabad, Gujarat
India

LCMS Analysis Report

Sample Name:	CIN2-C-818-CIN2-X-0053-097-e		Injection Id 10483
Sample Type:	Unknown	Acquired By:	LCMS-05
Vial:	2:F,3	Sample Set Name:	04122024_UCH_090_FD_02
Injection #:	1	Acq. Method Set:	o2h_LCMS_Method_A_SLOW
Injection Volume:	2.00 ul	Processing Method	O2H_LCMS_02_0,
Run Time:	4.0 Minutes	Channel Name:	MS TIC, 254.0nm, 210.0nm
Project Name:	2024\LCMS-05_DEC-2024_	Proc. Chnl. Descr.:	PDA 254.0 nm Blank Subtracted
Date Acquired:	05-12-2024 05:03:54 IST		
Date Processed:	05-12-2024 08:24:33 IST, 05-12-2024 08:24:41 IST, 05-12-2024 08:25:12 IST		
Column:	X-BRIDGE C18 2.1X50mm 2.5um		

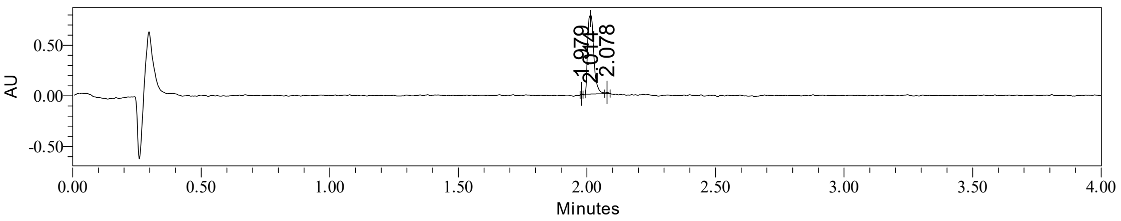

Channel Name 210.0nm; Channel PDA Spectrum

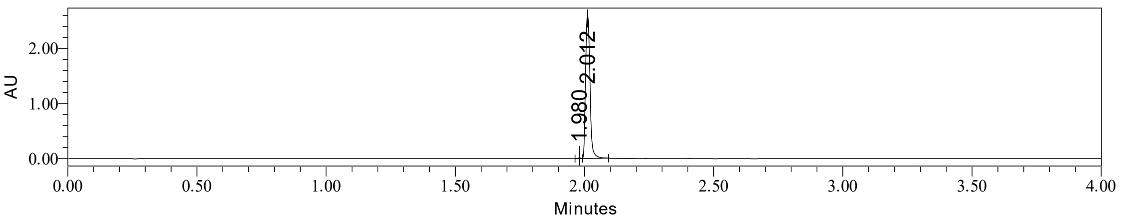

Channel Name 254.0nm; Channel PDA Spectrum

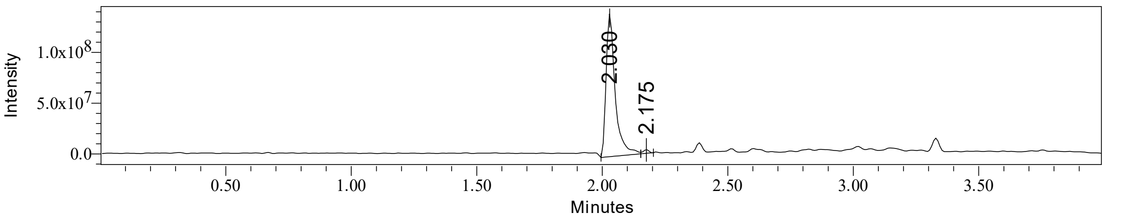

Channel Name MS TIC; Channel QDa 1: MS Scan

Peak Results
Channel: PDA Spectrum

	RT	Base Peak (m/z)	Height	Area	% Area	Channel	Channel Name
1	1.979		9206	3936	0.30	PDA Spectrum	210.0nm
2	1.980		11884	8703	0.28	PDA Spectrum	254.0nm
3	2.012		2601072	3109331	99.72	PDA Spectrum	254.0nm
4	2.014		783244	1288440	98.94	PDA Spectrum	210.0nm
5	2.078		11953	9850	0.76	PDA Spectrum	210.0nm

HPLC and LCMS Traces for Compound 5d

Peak Results
Channel: QDa 1: MS Scan

	RT	Base Peak (m/z)	Height	Area	% Area	Channel	Channel Name
1	2.030	304.05	140974104	353237644	98.41	QDa 1: MS Scan	MS TIC
2	2.175	304.25	3581208	5713337	1.59	QDa 1: MS Scan	MS TIC

Match Plot

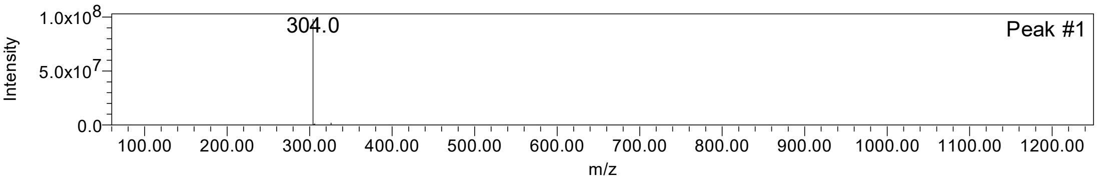

Base Peak 304.05 Channel Description 1: QDa Positive(+) Scan (60.00-1240.00)Da, Centroid, CV=10 - AVG (2.3:3.5;0.0:0.8) x 10.000 Th: 0.010 Retention Time 2.030

Match Plot

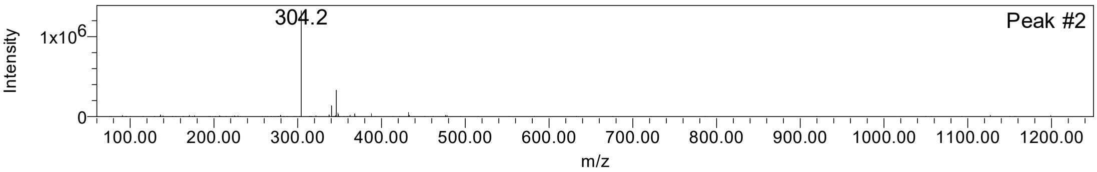

Base Peak 304.25 Channel Description 1: QDa Positive(+) Scan (60.00-1240.00)Da, Centroid, CV=10 - AVG (2.3:3.5;0.0:0.8) x 10.000 Th: 0.010 Retention Time 2.175

HPLC and LCMS Traces for Compound 5d

o2h discovery
Ahmedabad, Gujarat
India

HPLC Analysis Report

Sample Name: CIN2-C-818-CIN2-X-0053-097-f
Sample Type: Unknown
Vial: 1:F,6
Injection #:
Injection Volume: 3.00 ul
Run Time: 17.0 Minutes
Injection Id 6426
Date Acquired: 06-12-2024 21:11:10 IST
Date Processed: 06-12-2024 21:33:38 IST, 06-12-2024 21:33:45 IST
Column XTIMATE 4.6*150mm, 5um

Acquired By: LCMS-03
Sample Set Name 06122024_02
Acq. Method Set: O2H_LCMS_Method_F
Processing Method Processing method_02
Channel Name: 254.0nm, 210.0nm
Proc. Chnl. Descr.: PDA 210.0 nm Blank Subtracted

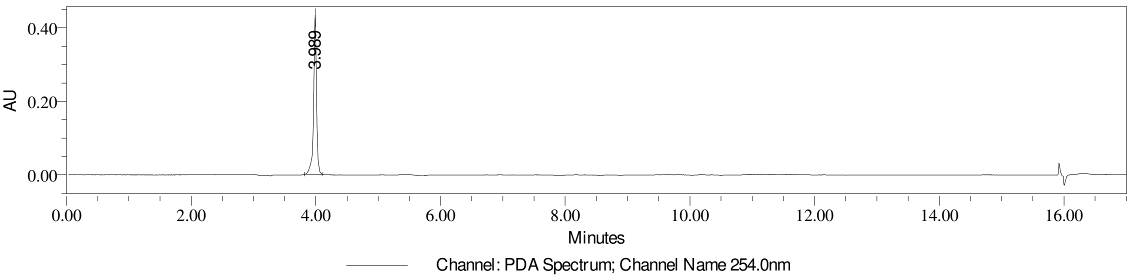

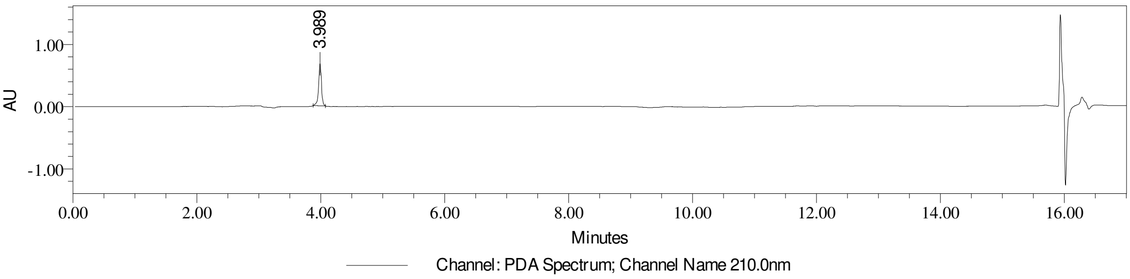

Peak Results
Channel: PDA Spectrum

	Retention Time (min)	Base Peak (m/z)	Height (µV)	Area (µV*sec)	% Area	Channel	Channel Name
1	3.989		433390	1447159	100.00	PDA Spectrum	254.0nm
2	3.989		672616	2140453	100.00	PDA Spectrum	210.0nm

Page: 1 of 1

HPLC and LCMS Traces for Compound 5e

o2h discovery
Ahmedabad, Gujarat
India

LCMS Analysis Report

Sample Name:	CIN2-C-818-CIN2-X-0055-078-c		Injection Id 53283
Sample Type:	Unknown	Acquired By:	LCMS-05
Vial:	1:F,3	Sample Set Name:	25112024_UCH_090_FD
Injection #:	1	Acq. Method Set:	o2h_LCMS_Method_A
Injection Volume:	2.00 ul	Processing Method	O2H_LCMS_02_0, MASS_000
Run Time:	4.0 Minutes	Channel Name:	254.0nm, MS TIC, 210.0nm
Project Name:	2024\LCMS-05_NOV-2024	Proc. Chnl. Descr.:	PDA 254.0 nm Blank Subtracted
Date Acquired:	25-11-2024 10:09:25 IST		
Date Processed:	25-11-2024 10:42:59 IST, 25-11-2024 10:43:41 IST, 25-11-2024 10:44:48 IST		
Column:	X-BRIDGE C18 2.1X50mm 2.5um		

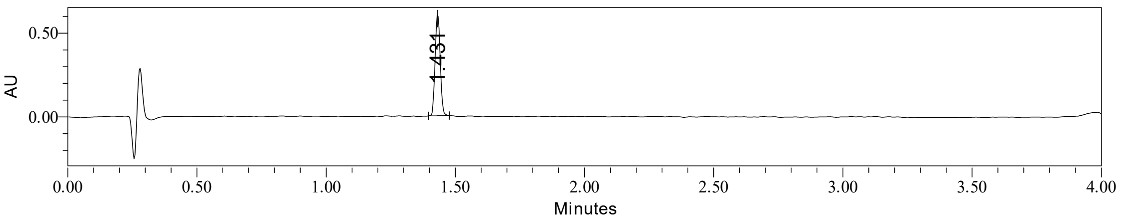

Channel Name 210.0nm; Channel PDA Spectrum

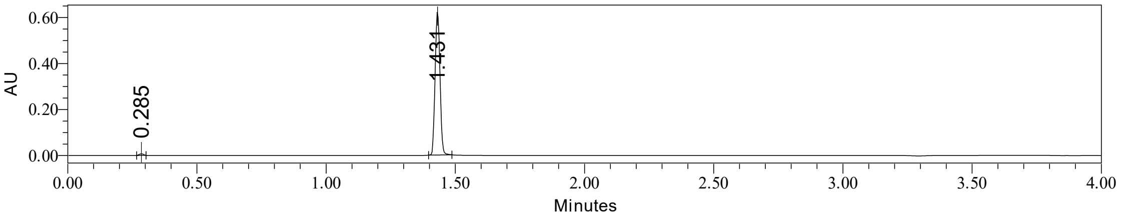

Channel Name 254.0nm; Channel PDA Spectrum

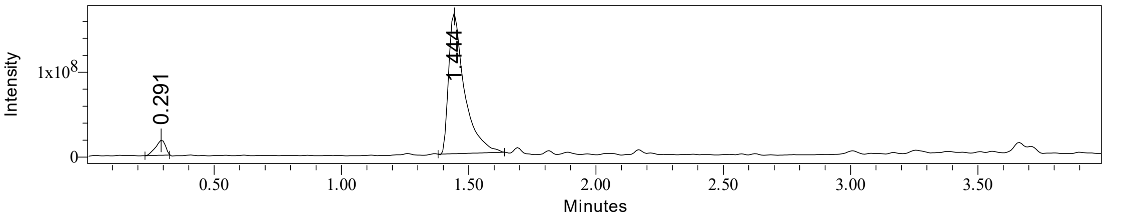

Channel Name MS TIC; Channel QDa 1: MS Scan

Peak Results
Channel: PDA Spectrum

	RT	Base Peak (m/z)	Height	Area	% Area	Channel	Channel Name
1	0.285		6853	7952	0.96	PDA Spectrum	254.0nm
2	1.431		619936	818048	99.04	PDA Spectrum	254.0nm
3	1.431		606558	831425	100.00	PDA Spectrum	210.0nm

HPLC and LCMS Traces for Compound 5e

Peak Results
Channel: QDa 1: MS Scan

	RT	Base Peak (m/z)	Height	Area	% Area	Channel	Channel Name
1	0.291	301.94	17452456	48132918	6.31	QDa 1: MS Scan	MS TIC
2	1.444	288.09	165251872	714220246	93.69	QDa 1: MS Scan	MS TIC

Match Plot

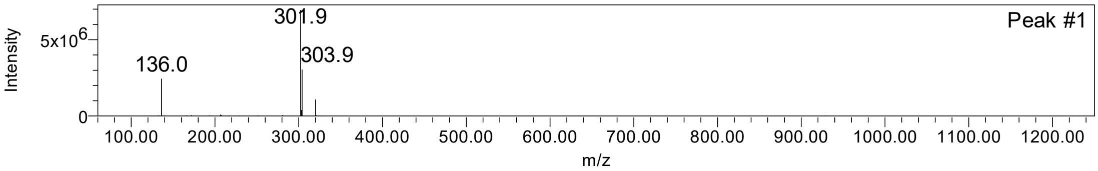

Base Peak 301.94 Channel Description 1: QDa Positive(+) Scan (60.00-1250.00)Da, Centroid, CV=10 - AVG (1.7:3.9;0.0:0.8) x 10.000 Th: 0.010 Retention Time 0.291

Match Plot

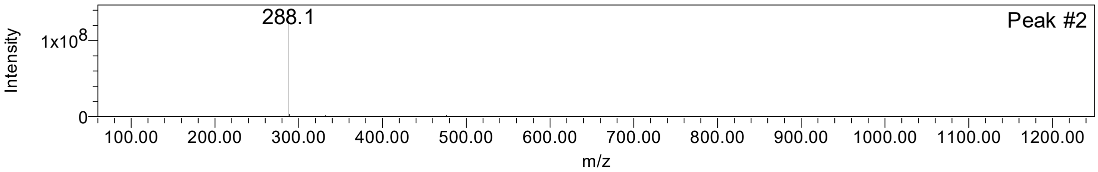

Base Peak 288.09 Channel Description 1: QDa Positive(+) Scan (60.00-1250.00)Da, Centroid, CV=10 - AVG (1.7:3.9;0.0:0.8) x 10.000 Th: 0.010 Retention Time 1.444

HPLC and LCMS Traces for Compound 5e

o2h discovery
Ahmedabad, Gujarat
India

HPLC Analysis Report

Sample name: CIN2-C-818-CIN2-X-0055-078-c
Location: P2-A5
Injection: 1 of 1
Injection volume: 15.000
Project Name HPLC_07_NOV-2024
Date Acquired: 2024-11-25 16:00:09+05:30
Date Processed: 2024-11-25 18:33:15+05:30

Instrument Name HPLC-07
Acq. method: o2h_HPLC_Method-B.amx
Processing method: 3D UV Quantitative_DefaultMethod.pmx
Column: XBridge C18 150x4.6mm, 3.5um

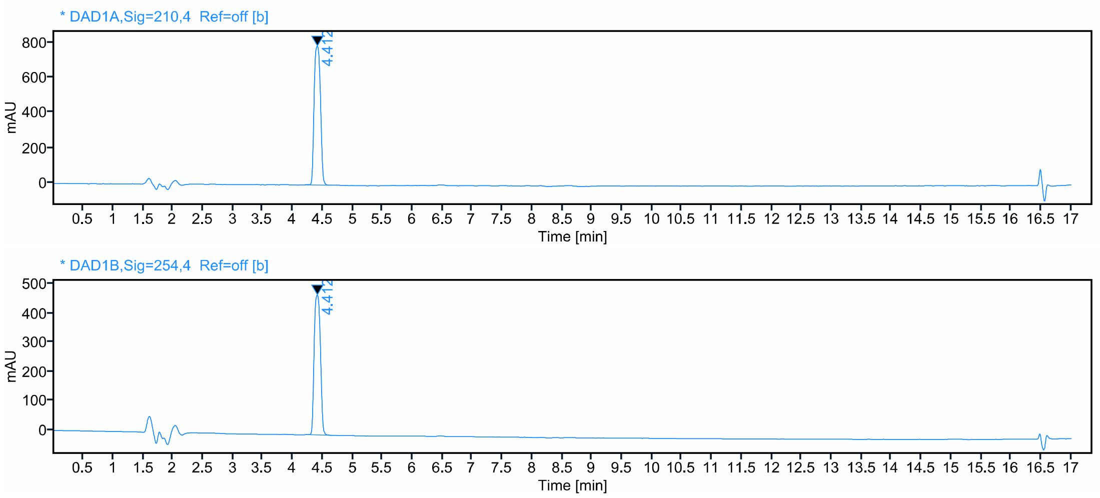

Signal: * DAD1A,Sig=210,4 Ref=off [b]

RT [min]	Height	Area	Area%
4.412	785	5752	100.00

Signal: * DAD1B,Sig=254,4 Ref=off [b]

RT [min]	Height	Area	Area%
4.412	479	3469	100.00

HPLC and LCMS Traces for Compound 5f

o2h discovery<br>Ahmedabad, Gujarat<br>India	**LCMS Analysis Report**

Sample Name:	CIN2-C-817-CIN2-X-0039-057-h		Injection Id 2427
Sample Type:	Unknown	Acquired By:	LCMS-04
Vial:	1:C,4	Sample Set Name:	05112024_UCH_088_FD
Injection #:	1	Acq. Method Set:	o2h_LCMS_Method_A
Injection Volume:	2.00 ul	Processing Method	O2H_LCMS_06, O2H_LCMS_0
Run Time:	4.0 Minutes	Channel Name:	220.0nm, MS TIC, 254.0nm
Project Name:	2024\LCMS-04_NOV-2024	Proc. Chnl. Descr.:	PDA 254.0 nm Blank Subtracted
Date Acquired:	05-11-2024 10:58:17 IST		
Date Processed:	05-11-2024 11:08:42 IST, 05-11-2024 11:08:50 IST, 05-11-2024 11:09:49 IST		
Column:	X-BRIDGE C18 2.1X50mm 2.5um		

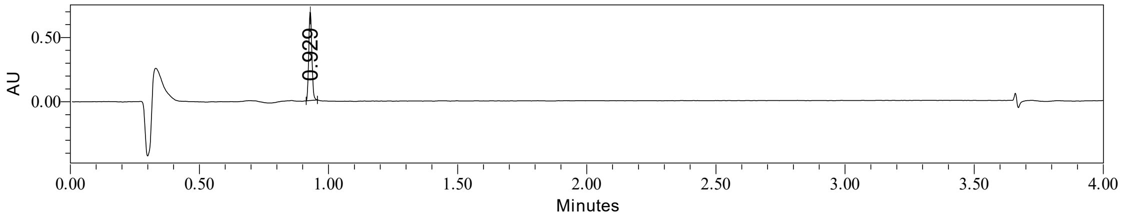

Channel PDA Spectrum; Channel Name 220.0nm

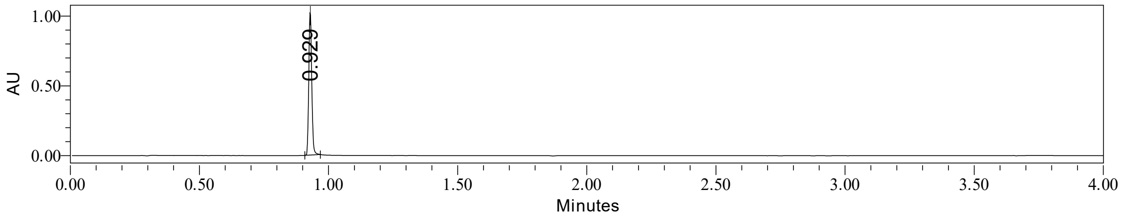

Channel PDA Spectrum; Channel Name 254.0nm

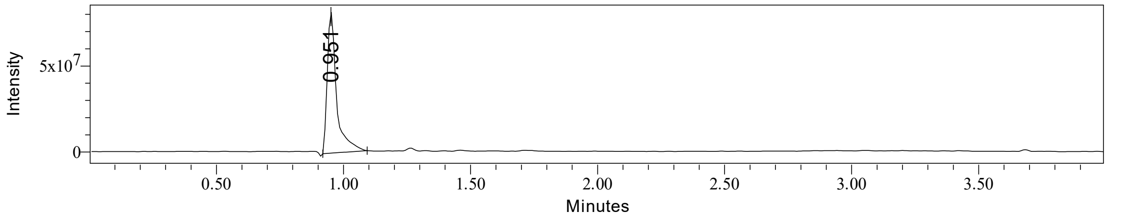

Channel QDa 1: MS Scan; Channel Name MS TIC

Peak Results
Channel: PDA Spectrum

	RT	Base Peak (m/z)	Height	Area	% Area	Channel	Channel Name
1	0.929		1024762	817082	100.00	PDA Spectrum	254.0nm
2	0.929		691584	575993	100.00	PDA Spectrum	220.0nm

Peak Results
Channel: QDa 1: MS Scan

	RT	Base Peak (m/z)	Height	Area	% Area	Channel	Channel Name
1	0.951	288.26	81962608	207532749	100.00	QDa 1: MS Scan	MS TIC

HPLC and LCMS Traces for Compound 5f

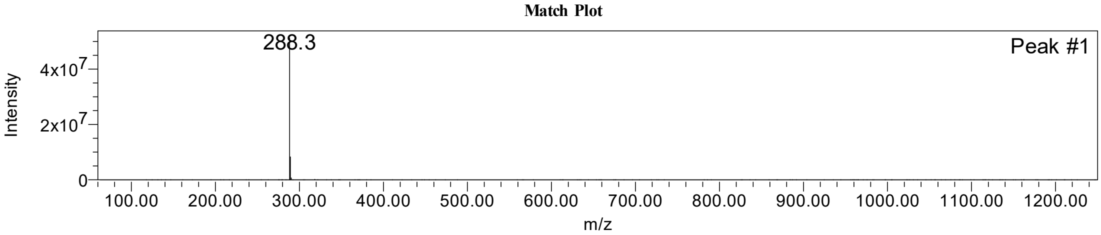

Base Peak 288.26 Channel Description 1: QDa Positive(+) Scan (60.00-1250.00)Da, Centroid, CV=10 - AVG (0.0:0.7;1.1:4.0) x 20.000 Th: 0.010 Retention Time 0.951

HPLC and LCMS Traces for Compound 5f

o2h discovery
Ahmedabad, Gujarat
India

HPLC Analysis Report

Sample Name: **CIN2-C-817-CIN2-X-0039-057-h**

Vial:	29	System Name:	HPLC-11
Injection Volume:	10.00 ul	Sample Set Name:	05112024_HPLC_01
Run Time:	17.0 Minutes	Acq. Method Set:	o2h_HPLC_Method_D
Date Acquired:	05-11-2024 14:37:09 IST	Processing Method:	o2h_HPLC_Method_B
Date Processed:	05-11-2024 16:08:02 IST, 05-11-2024	Channel Name:	220.0nm, 262.0nm
Project Name:	HPLC-11\2024\Nov-2024		
Column:	ACE Excel 3 Super C18 (150 X 4.6mm)		

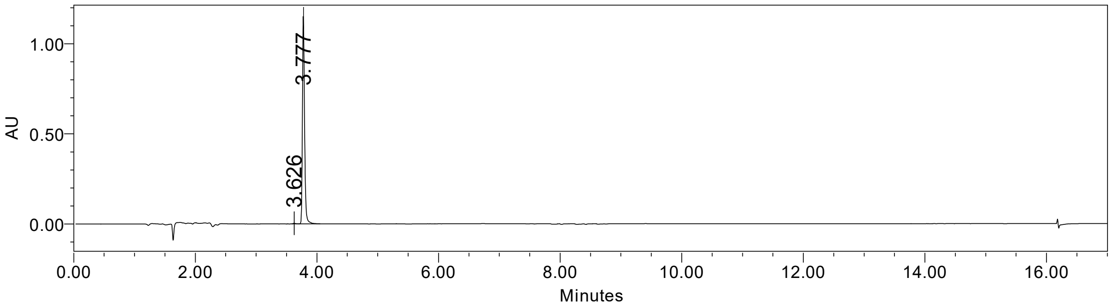

SampleName CIN2-C-817-CIN2-X-0039-057-h; Channel Name 220.0nm

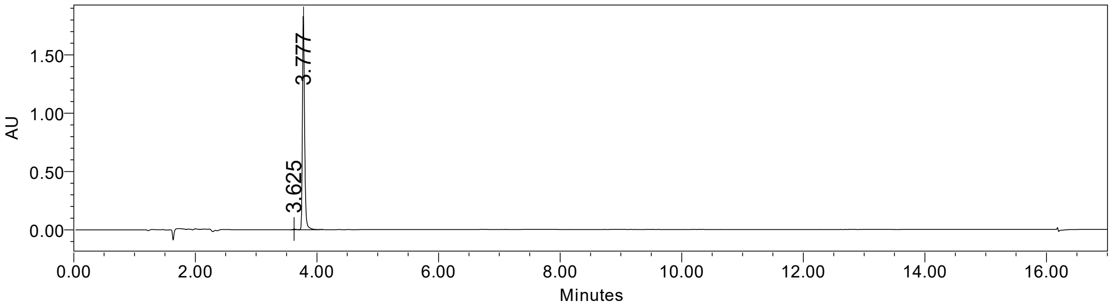

SampleName CIN2-C-817-CIN2-X-0039-057-h; Channel Name 262.0nm

Channel Name: 220.0nm

	Retention Time (min)	Height (µV)	Area (µV*sec)	% Area	Channel Name
1	3.63	3502	9401	0.33	220.0nm
2	3.78	1150901	2804421	99.67	220.0nm

HPLC and LCMS Traces for Compound 5f

Channel Name: 262.0nm

	Retention Time (min)	Height (µV)	Area (µV*sec)	% Area	Channel Name
1	3.63	5941	16069	0.36	262.0nm
2	3.78	1830347	4496577	99.64	262.0nm

Date Printed: 05-11-2024 16:08:38 Asia/Kolkata

HPLC and LCMS Traces for Compound 5g

o2h discovery
Ahmedabad, Gujarat
India

LCMS Analysis Report

Sample Name:	CIN2-C-817-CIN2-X-0031-039-F		Injection Id 13916
Sample Type:	Unknown	Acquired By:	LCMS-05
Vial:	1:C,8	Sample Set Name:	07102024_UCH_083_FD
Injection #:	1	Acq. Method Set:	o2h_LCMS_Method_A
Injection Volume:	4.00 ul	Processing Method	O2H_LCMS_07_0, MASS_000
Run Time:	4.0 Minutes	Channel Name:	MS TIC, 254.0nm, 210.0nm
Project Name:	2024\LCMS-05_OCT-2024	Proc. Chnl. Descr.:	PDA 254.0 nm Blank Subtracted
Date Acquired:	07-10-2024 11:52:16 IST		
Date Processed:	07-10-2024 11:55:36 IST, 07-10-2024 11:57:01 IST, 07-10-2024 11:58:02 IST		
Column:	X-BRIDGE C18 2.1X50mm 2.5um		

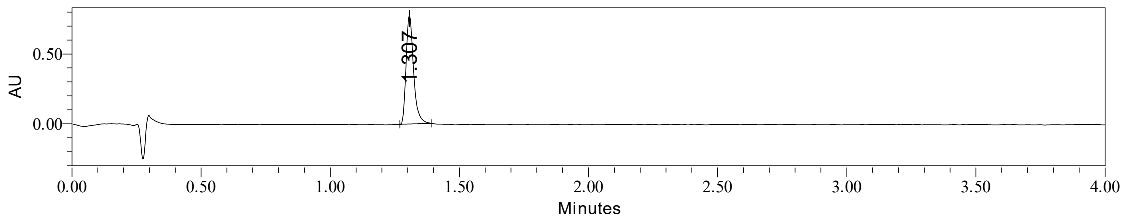

Channel Name 210.0nm; Channel PDA Spectrum

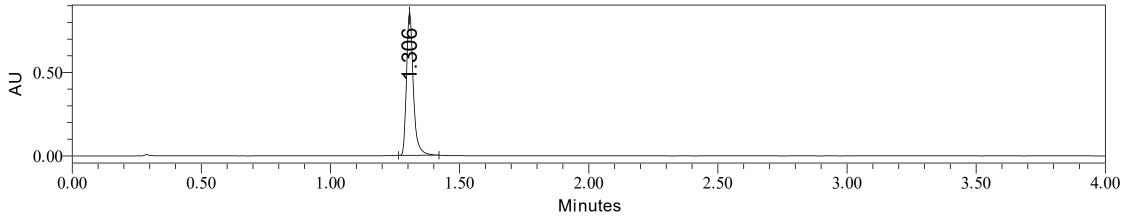

Channel Name 254.0nm; Channel PDA Spectrum

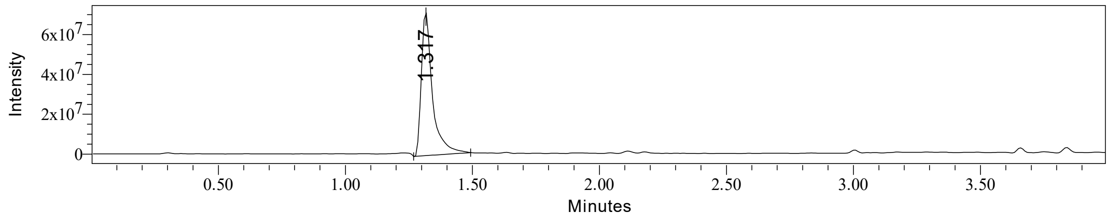

Channel Name MS TIC; Channel QDa 1: MS Scan

Peak Results
Channel: PDA Spectrum

	RT	Base Peak (m/z)	Height	Area	% Area	Channel	Channel Name
1	1.306		855934	1564020	100.00	PDA Spectrum	254.0nm
2	1.307		778494	1559806	100.00	PDA Spectrum	210.0nm

Peak Results
Channel: QDa 1: MS Scan

	RT	Base Peak (m/z)	Height	Area	% Area	Channel	Channel Name
1	1.317	306.09	71096216	217907599	100.00	QDa 1: MS Scan	MS TIC

HPLC and LCMS Traces for Compound 5g

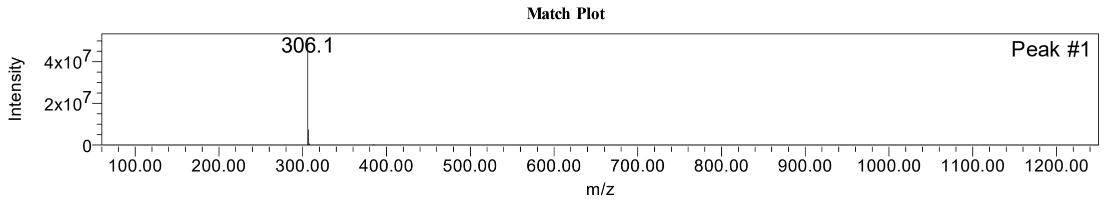

Base Peak 306.09 Channel Description 1: QDa Positive(+) Scan (60.00-1250.00)Da, Centroid, CV=10 - AVG (2.2:3.7;0.1:0.9) x 20.000 Th: 0.010 Retention Time 1.317

HPLC and LCMS Traces for Compound 5g

o2h discovery
Ahmedabad, Gujarat
India

HPLC Analysis Report

**Sample name:**	CIN2-C-817-CIN2-X-0031-039-F		
**Location:**	P1-B1	**Instrument Name**	HPLC-10
**Injection:**	1 of 1	**Acq. method:**	o2h_HPLC_Method-C.amx
**Injection volume:**	15.000	**Processing method:**	*3D UV Quantitative_DefaultMethod.pmx
**Project Name**	HPLC-10_OCT-2024		
**Date Acquired:**	2024-10-07 14:44:09+05:30	**Column:**	SUNFIRE C18 150x4.6mm,3.5um
**Date Processed:**	2024-10-07 15:03:05+05:30		

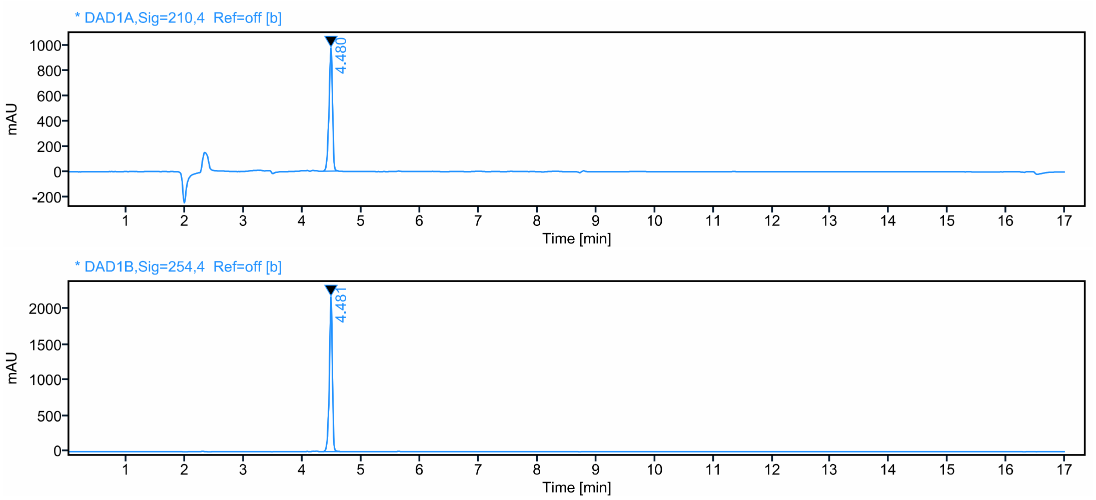

Signal: * DAD1A,Sig=210,4 Ref=off [b]

RT [min]	Height	Area	Area%
4.480	973	4101	100.00

Signal: * DAD1B,Sig=254,4 Ref=off [b]

RT [min]	Height	Area	Area%
4.481	2177	7949	100.00

HPLC and LCMS Traces for Compound 5h

o2h discovery<br>Ahmedabad, Gujarat<br>India	**LCMS Analysis Report**

Sample Name:	CIN2-C-817-CIN2-X-0038-067-g		Injection Id 35518
Sample Type:	Unknown	Acquired By:	LCMS-05
Vial:	1:E,4	Sample Set Name:	18112024_UCH_090_FD
Injection #:	1	Acq. Method Set:	o2h_LCMS_Method_A
Injection Volume:	2.00 ul	Processing Method	O2H_LCMS_01,
Run Time:	4.0 Minutes	Channel Name:	MS TIC, 254.0nm, 210.0nm
Project Name:	2024\LCMS-05_NOV-2024	Proc. Chnl. Descr.:	QDa 1: MS Scan MS TIC,
Date Acquired:	18-11-2024 14:44:14 IST		
Date Processed:	18-11-2024 14:49:11 IST, 18-11-2024 14:49:17 IST, 18-11-2024 14:49:36 IST, 18-11-2024		
Column:	X-BRIDGE C18 2.1X50mm 2.5um		

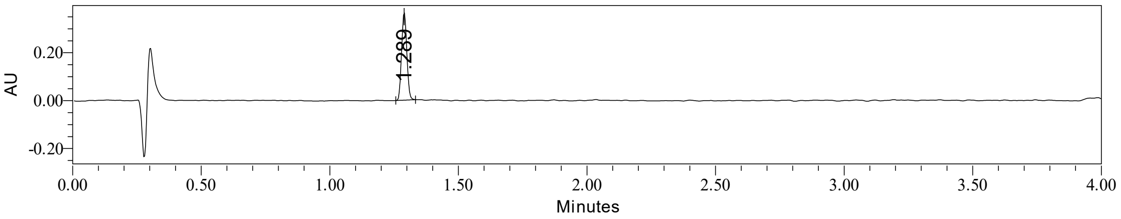

Channel Name 210.0nm; Channel PDA Spectrum

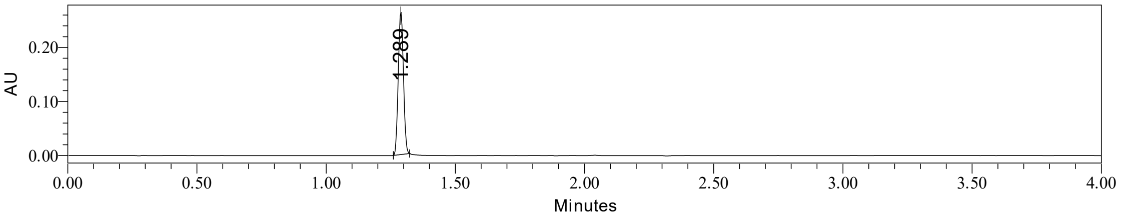

Channel Name 254.0nm; Channel PDA Spectrum

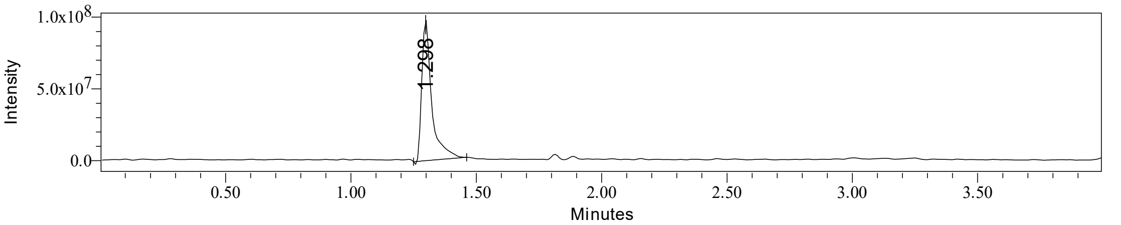

Channel Name MS TIC; Channel QDa 1: MS Scan

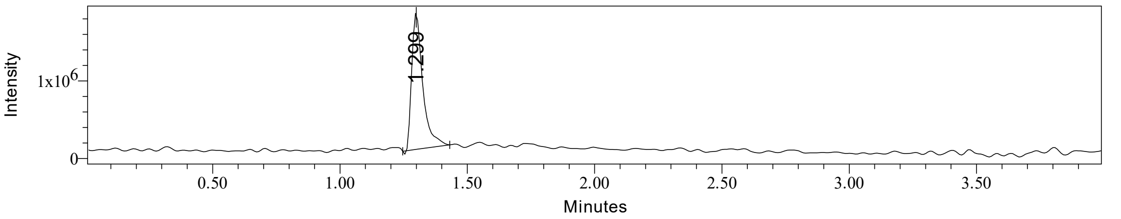

Channel Name MS TIC; Channel QDa 3: MS Scan

HPLC and LCMS Traces for Compound 5h

Peak Results
Channel: PDA Spectrum

	RT	Base Peak (m/z)	Height	Area	% Area	Channel	Channel Name
1	1.289		263125	365876	100.00	PDA Spectrum	254.0nm
2	1.289		364744	516196	100.00	PDA Spectrum	210.0nm

Peak Results
Channel: QDa 1: MS Scan

	RT	Base Peak (m/z)	Height	Area	% Area	Channel	Channel Name
1	1.298	306.00	98107978	270898702	100.00	QDa 1: MS Scan	MS TIC

Peak Results
Channel: QDa 3: MS Scan

	RT	Base Peak (m/z)	Height	Area	% Area	Channel	Channel Name
1	1.299	350.19	1750356	5334055	100.00	QDa 3: MS Scan	MS TIC

Match Plot

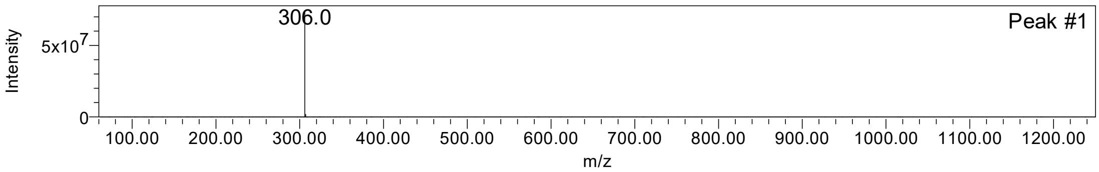

Base Peak 306.00 Channel Description 1: QDa Positive(+) Scan (60.00-1250.00)Da, Centroid, CV=10 - AVG (0.0:1.0;1.6:3.9) x 20.000 Th: 0.010 Retention Time 1.298

Match Plot

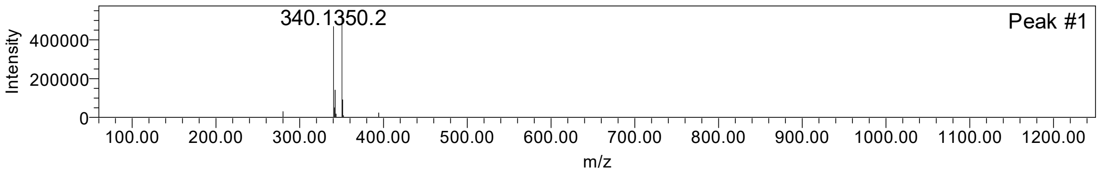

Base Peak 350.19 Channel Description 3: QDa Negative(-) Scan (60.00-1250.00)Da, Centroid, CV=10 - AVG (0.0:1.0;1.8:4.0) x 20.000 Th: 0.010 Retention Time 1.299

HPLC and LCMS Traces for Compound 5h

o2h discovery
Ahmedabad, Gujarat
India

HPLC Analysis Report

Sample name: CIN2-C-817-CIN2-X-0038-067-g
Location: P2-B3
Injection: 1 of 1
Injection volume: 30.000
Project Name HPLC-10-NOV-2024
Date Acquired: 2024-11-18 16:18:59+05:30
Date Processed: 2024-11-18 16:39:01+05:30

Instrument Name HPLC-10
Acq. method: o2h_HPLC_Method-C.amx
Processing method: *3D UV Quantitative_DefaultMethod.pmx
Column: SUNFIRE C18 150x4.6mm,3.5um

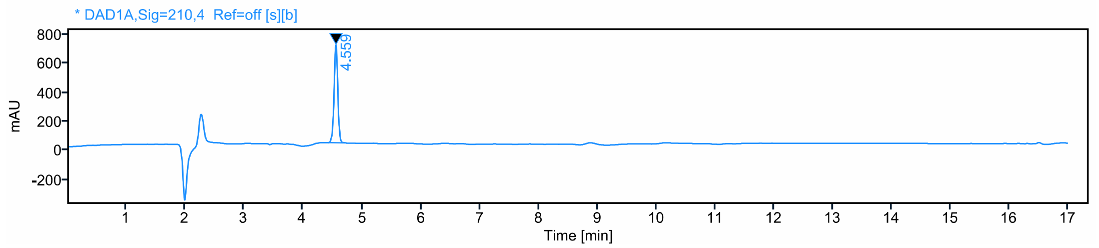

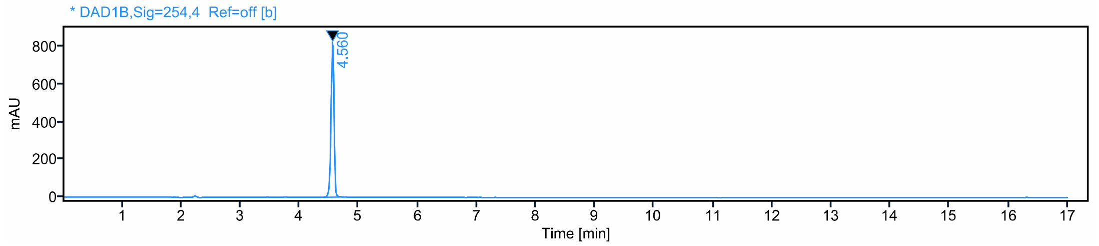

Signal: * DAD1A,Sig=210,4 Ref=off [s][b]

RT [min]	Height	Area	Area%
4.559	671	3030	100.00

Signal: * DAD1B,Sig=254,4 Ref=off [b]

RT [min]	Height	Area	Area%
4.560	825	2860	100.00

HPLC and LCMS Traces for Compound 6a

o2h discovery
Ahmedabad, Gujarat
India

LCMS Analysis Report

Sample Name:	CIN-C-377-CIN-X-0034-038-a		Injection Id 21830
Sample Type:	Unknown	Acquired By:	LCMS-05
Vial:	1:C,5	Sample Set Name:	14052024_UCH_077_FD
Injection #:	1	Acq. Method Set:	o2h_LCMS_Method_A
Injection Volume:	2.00 ul	Processing Method:	O2H_LCMS_02_0,
Run Time:	4.0 Minutes	Channel Name:	MS TIC, 254.0nm, 210.0nm
Project Name:	2024\LCMS-05_MAY-2024	Proc. Chnl. Descr.:	PDA 254.0 nm Blank Subtracted
Date Acquired:	14-05-2024 11:06:34 IST		
Date Processed:	14-05-2024 11:25:15 IST, 14-05-2024 11:25:25 IST, 14-05-2024 11:25:57 IST		
Column:	X-BRIDGE C18 2.1X50mm 2.5um		

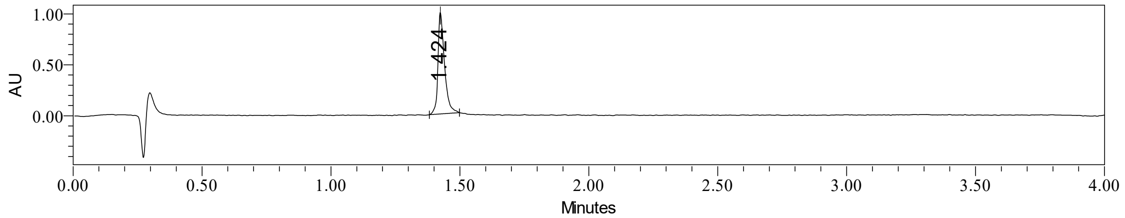

Channel Name 210.0nm; Channel PDA Spectrum

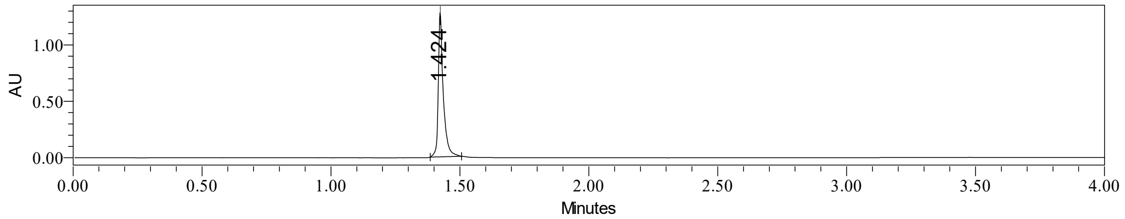

Channel Name 254.0nm; Channel PDA Spectrum

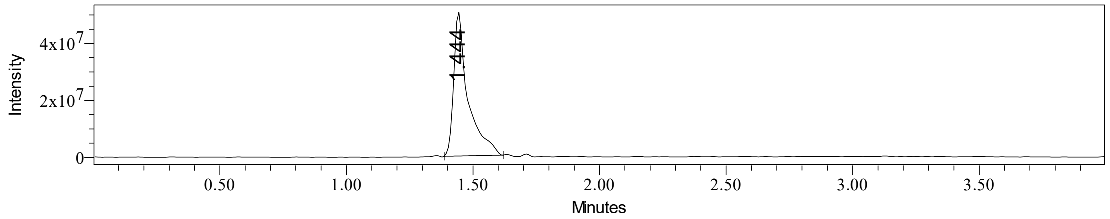

Channel Name MS TIC; Channel QDa 1: MS Scan

Peak Results
Channel: PDA Spectrum

	RT	Base Peak (m/z)	Height	Area	% Area	Channel	Channel Name
1	1.424		1277709	1713679	100.00	PDA Spectrum	254.0nm
2	1.424		998511	1762597	100.00	PDA Spectrum	210.0nm

Peak Results
Channel: QDa 1: MS Scan

	RT	Base Peak (m/z)	Height	Area	% Area	Channel	Channel Name
1	1.444	332.25	49903777	198034498	100.00	QDa 1: MS Scan	MS TIC

HPLC and LCMS Traces for Compound 6a

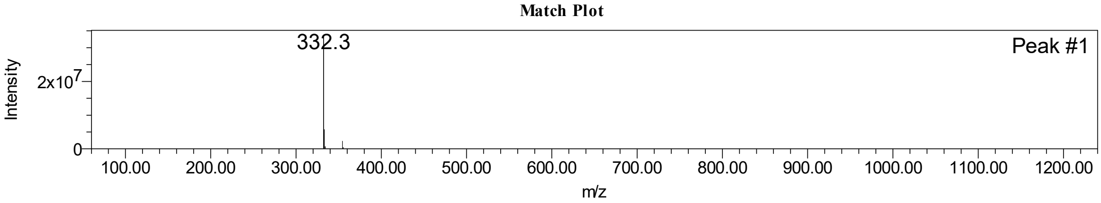

Base Peak 332.25 Channel Description 1: QDa Positive(+) Scan (60.00-1250.00)Da, Centroid, CV=10 - AVG (0.1:1.1;1.7:3.8) x 20.000 Th: 0.010 Retention Time 1.444

HPLC and LCMS Traces for Compound 6a

o2h discovery
Ahmedabad, Gujarat
India

HPLC Analysis Report

Sample name: CIN-C-377-CIN-X-0034-038-a
Location: P1-B7
Injection: 1 of 1
Injection volume: 10.000
Project Name HPLC-10_MAY-2024
Date Acquired: 2024-05-14 17:27:46+05:30
Date Processed: 2024-05-14 18:10:14+05:30

Instrument Name HPLC-10
Acq. method: o2h_HPLC_Method-A.amx
Processing method: *3D UV Quantitative_DefaultMethod.pmx
Column: SUNFIRE C18 150x4.6mm,3.5um

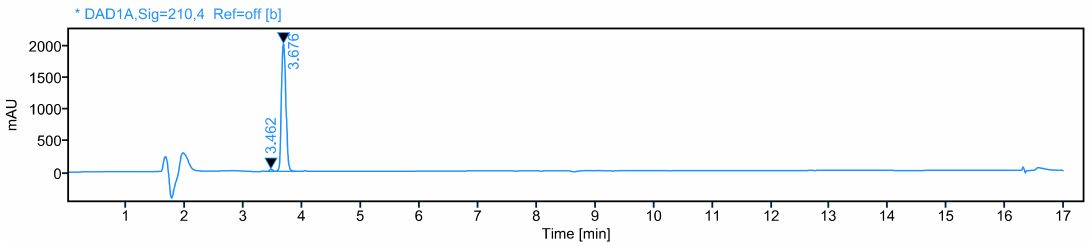

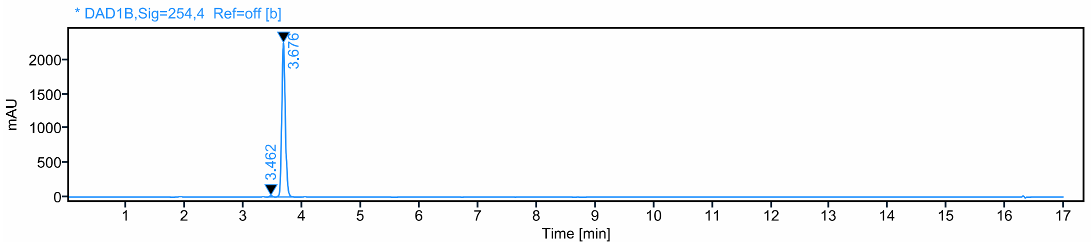

Signal: * DAD1A,Sig=210,4 Ref=off [b]

RT [min]	Height	Area	Area%
3.462	34	130	1.20
3.676	2002	10705	98.80

Signal: * DAD1B,Sig=254,4 Ref=off [b]

RT [min]	Height	Area	Area%
3.462	19	68	0.71
3.676	2250	9546	99.29

HPLC and LCMS Traces for Compound 6b

o2h discovery
Ahmedabad, Gujarat
India

LCMS Analysis Report

Sample Name:	CIN-C-377-CIN-X-0036-075-b		Injection Id 44213
Sample Type:	Unknown	Acquired By:	LCMS-05
Vial:	2:A,1	Sample Set Name:	22062024_UCH_082_RD
Injection #:	1	Acq. Method Set:	o2h_LCMS_Method_A_SLOW
Injection Volume:	2.00 ul	Processing Method	O2H_LCMS_02_00, MASS_01
Run Time:	4.0 Minutes	Channel Name:	MS TIC, 254.0nm, 210.0nm
Project Name:	2024\LCMS-05_JUN-2024	Proc. Chnl. Descr.:	PDA 254.0 nm Blank Subtracted
Date Acquired:	22-06-2024 12:46:25 IST		
Date Processed:	22-06-2024 13:17:20 IST, 22-06-2024 13:17:25 IST, 22-06-2024 13:18:23 IST		
Column:	X-BRIDGE C18 2.1X50mm 2.5um		

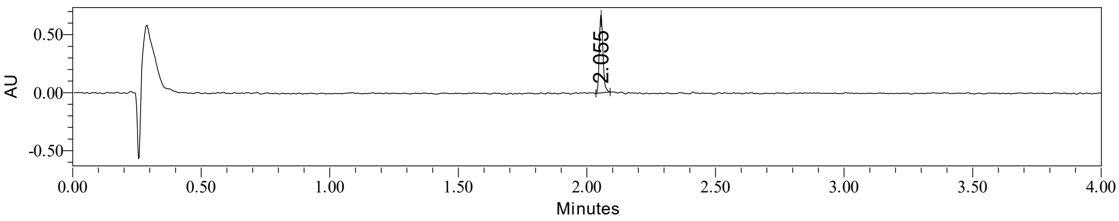

Channel Name 210.0nm; Channel PDA Spectrum

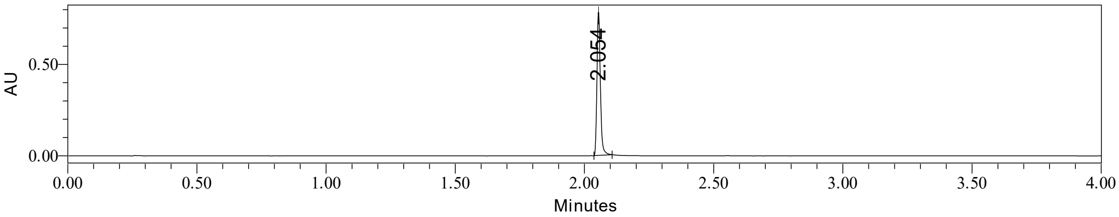

Channel Name 254.0nm; Channel PDA Spectrum

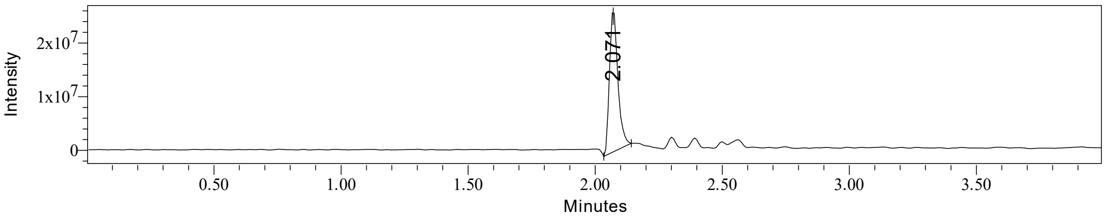

Channel Name MS TIC; Channel QDa 1: MS Scan

Peak Results
Channel: PDA Spectrum

	RT	Base Peak (m/z)	Height	Area	% Area	Channel	Channel Name
1	2.054		789143	721972	100.00	PDA Spectrum	254.0nm
2	2.055		674271	669689	100.00	PDA Spectrum	210.0nm

Peak Results
Channel: QDa 1: MS Scan

	RT	Base Peak (m/z)	Height	Area	% Area	Channel	Channel Name
1	2.071	332.28	26864973	60517995	100.00	QDa 1: MS Scan	MS TIC

HPLC and LCMS Traces for Compound 6b

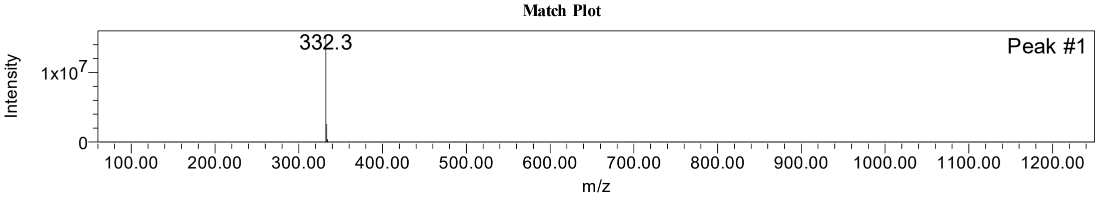

Base Peak 332.28 Channel Description 1: QDa Positive(+) Scan (60.00-1240.00)Da, Centroid, CV=10 - AVG (0.0:1.5;2.7:3.8) x 30.000 Th: 0.010 Retention Time 2.071

HPLC and LCMS Traces for Compound 6b

o2h discovery
Ahmedabad, Gujarat
India

HPLC Analysis Report

Sample name: CIN-C-377-CIN-X-0036-075-b
Location: P2-B1
Injection: 1 of 1
Injection volume: 10.000
Project Name HPLC-07_JUNE-2024
Date Acquired: 2024-06-22 14:09:00+05:30
Date Processed: 2024-06-22 14:28:46+05:30

Instrument Name HPLC-07
Acq. method: o2h_HPLC_Method-D.amx
Processing method: *3D UV Quantitative_DefaultMethod.pmx
Column: XBridge C18 150x4.6mm, 3.5um

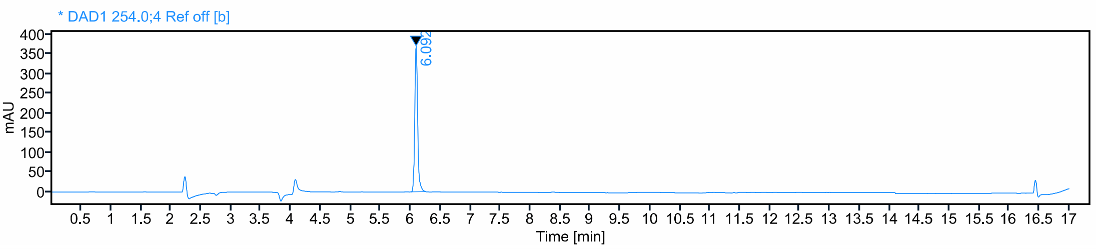

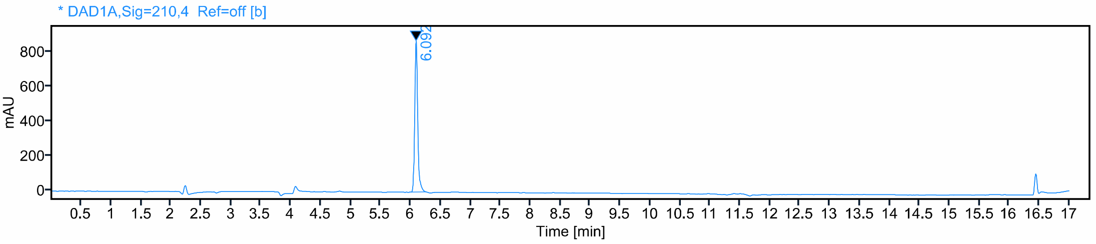

Signal: * DAD1 254.0;4 Ref off [b]

RT [min]	Height	Area	Area%
6.092	370	1272	100.00

Signal: * DAD1A,Sig=210,4 Ref=off [b]

RT [min]	Height	Area	Area%
6.092	870	3076	100.00

HPLC and LCMS Traces for Compound 6c

o2h discovery
Ahmedabad, Gujarat
India

LCMS Analysis Report

Sample Name:	CIN-C-377-CIN-X-0035-069-a		Injection Id 32193
Sample Type:	Unknown	Acquired By:	LCMS-05
Vial:	2:C,3	Sample Set Name:	17062024_UCH_083_RD
Injection #:	1	Acq. Method Set:	o2h_LCMS_Method_A_SLOW
Injection Volume:	2.00 ul	Processing Method	O2H_LCMS_02_00,
Run Time:	4.0 Minutes	Channel Name:	220.0nm, MS TIC, 254.0nm
Project Name:	2024\LCMS-05_JUN-2024	Proc. Chnl. Descr.:	PDA 254.0 nm Blank Subtracted
Date Acquired:	17-06-2024 15:02:07 IST		
Date Processed:	17-06-2024 15:08:38 IST, 17-06-2024 15:08:42 IST, 17-06-2024 15:09:05 IST		
Column:	X-BRIDGE C18 2.1X50mm 2.5um		

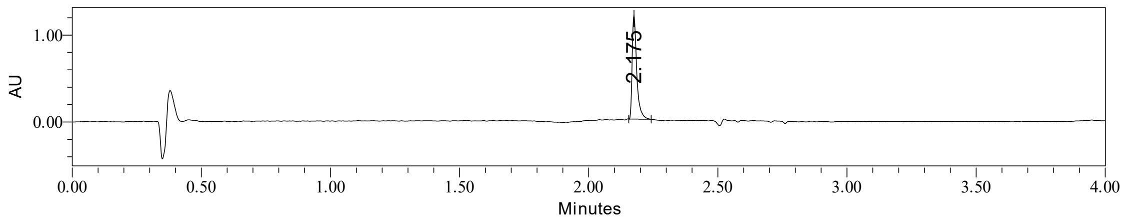

Channel Name 220.0nm; Channel PDA Spectrum

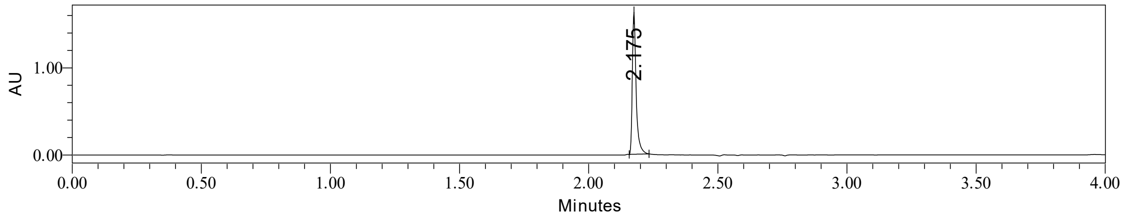

Channel Name 254.0nm; Channel PDA Spectrum

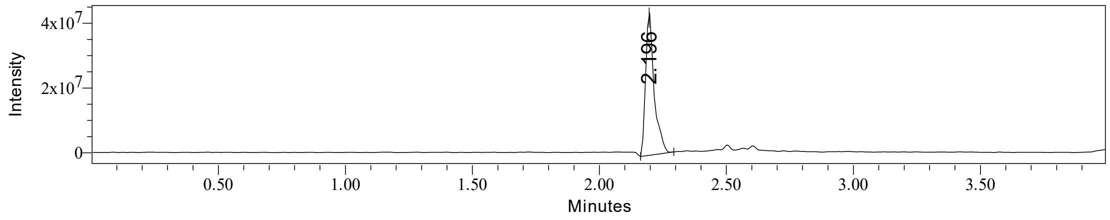

Channel Name MS TIC; Channel QDa 1: MS Scan

Peak Results
Channel: PDA Spectrum

	RT	Base Peak (m/z)	Height	Area	% Area	Channel	Channel Name
1	2.175		1626910	1669037	100.00	PDA Spectrum	254.0nm
2	2.175		119842C	1463386	100.00	PDA Spectrum	220.0nm

Peak Results
Channel: QDa 1: MS Scan

	RT	Base Peak (m/z)	Height	Area	% Area	Channel	Channel Name
1	2.196	332.27	44375921	103684187	100.00	QDa 1: MS Scan	MS TIC

HPLC and LCMS Traces for Compound 6c

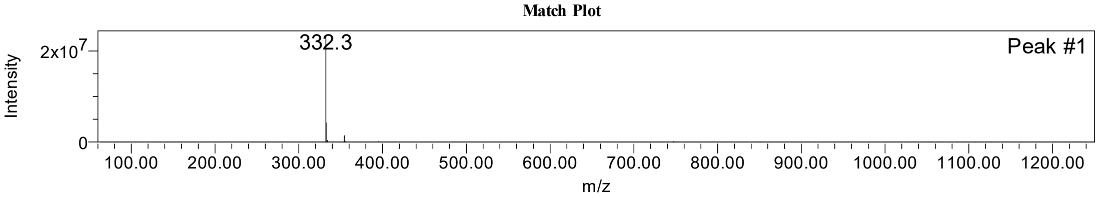

Base Peak 332.27 Channel Description 1: QDa Positive(+) Scan (60.00-1240.00)Da, Centroid, CV=10 - AVG (0.2:1.7;2.5:4.0) x 30.000 Th: 0.010 Retention Time 2.196

HPLC and LCMS Traces for Compound 6c

o2h discovery
Ahmedabad, Gujarat
India

HPLC Analysis Report

**Sample name:**	CIN-C-377-CIN-X-0035-069-a		
**Location:**	P1-A11	**Instrument Name**	HPLC-10
**Injection:**	1 of 1	**Acq. method:**	o2h_HPLC_Method-C.amx
**Injection volume:**	10.000	**Processing method:**	*3D UV Quantitative_DefaultMethod.pmx
**Project Name**	HPLC-10_JUNE-2024		
**Date Acquired:**	2024-06-17 17:18:57+05:30	**Column:**	SUNFIRE C18 150x4.6mm,3.5um
**Date Processed:**	2024-06-17 20:37:46+05:30		

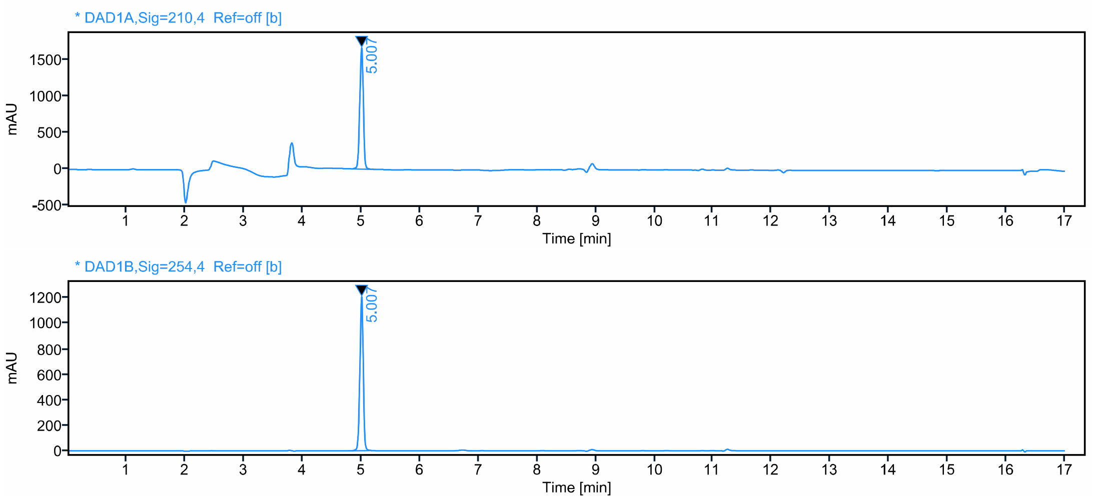

Signal: * DAD1A,Sig=210,4 Ref=off [b]

RT [min]	Height	Area	Area%
5.007	1666	6915	100.00

Signal: * DAD1B,Sig=254,4 Ref=off [b]

RT [min]	Height	Area	Area%
5.007	1213	4719	100.00

HPLC and LCMS Traces for Compound 6d

o2h discovery
Ahmedabad, Gujarat
India

LCMS Analysis Report

Sample Name:	CIN2-C-817-CIN2-X-0054-087		Injection Id 24886
Sample Type:	Unknown	Acquired By:	LCMS-05
Vial:	1:D,8	Sample Set Name:	13112024_UCH_090_FD
Injection #:	1	Acq. Method Set:	o2h_LCMS_Method_A
Injection Volume:	2.00 ul	Processing Method	MASS_000, O2H_LCMS_02_00
Run Time:	4.0 Minutes	Channel Name:	MS TIC, 254.0nm, 210.0nm
Project Name:	2024\LCMS-05_NOV-2024	Proc. Chnl. Descr.:	QDa 1: MS Scan MS TIC,
Date Acquired:	13-11-2024 11:35:54 IST		
Date Processed:	13-11-2024 11:51:42 IST, 13-11-2024 11:51:47 IST, 13-11-2024 11:52:12 IST		
Column:	X-BRIDGE C18 2.1X50mm 2.5um		

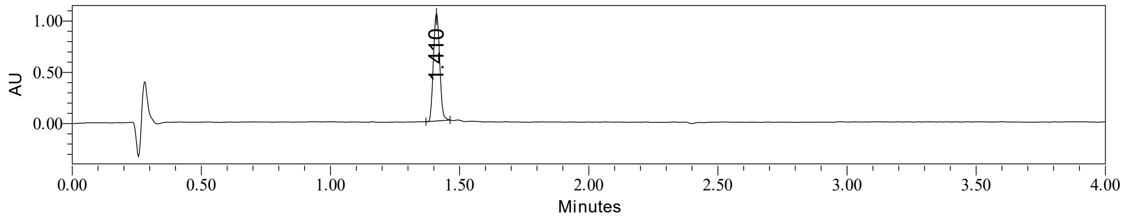

Channel Name 210.0nm; Channel PDA Spectrum

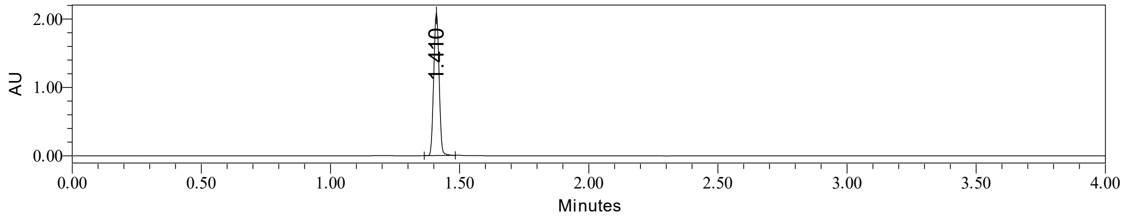

Channel Name 254.0nm; Channel PDA Spectrum

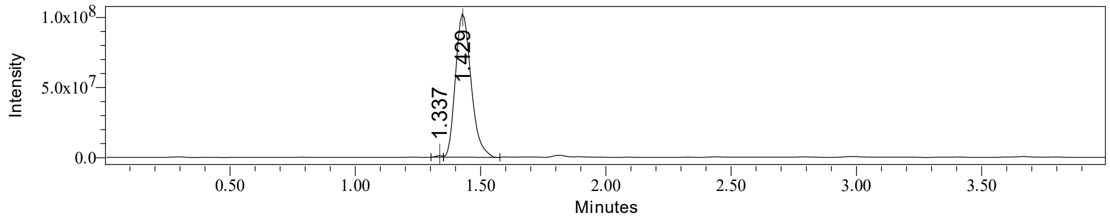

Channel Name MS TIC; Channel QDa 1: MS Scan

Peak Results
Channel: PDA Spectrum

	RT	Base Peak (m/z)	Height	Area	% Area	Channel	Channel Name
1	1.410		2091670	3001479	100.00	PDA Spectrum	254.0nm
2	1.410		1055529	1769761	100.00	PDA Spectrum	210.0nm

Peak Results
Channel: QDa 1: MS Scan

	RT	Base Peak (m/z)	Height	Area	% Area	Channel	Channel Name
1	1.337	318.13	1229193	2120174	0.48	QDa 1: MS Scan	MS TIC

HPLC and LCMS Traces for Compound 6d

Peak Results
Channel: QDa 1: MS Scan

	RT	Base Peak (m/z)	Height	Area	% Area	Channel	Channel Name
2	1.429	332.19	102205895	443462977	99.52	QDa 1: MS Scan	MS TIC

Match Plot

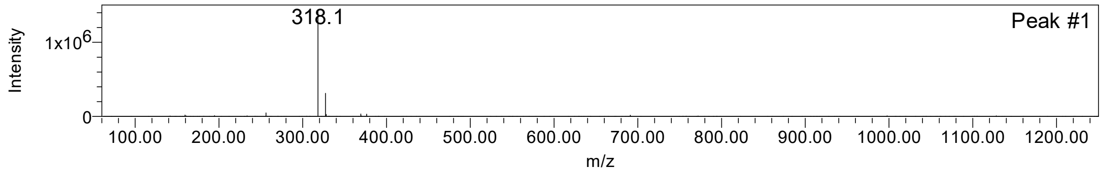

Base Peak 318.13 Channel Description 1: QDa Positive(+) Scan (60.00-1250.00)Da, Centroid, CV=10 - AVG (2.0:2.4;0.2:0.5) x 20.000 Th: 0.010 Retention Time 1.337

Match Plot

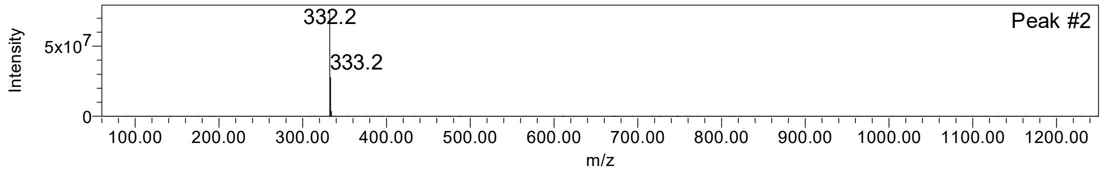

Base Peak 332.19 Channel Description 1: QDa Positive(+) Scan (60.00-1250.00)Da, Centroid, CV=10 - AVG (2.0:2.4;0.2:0.5) x 20.000 Th: 0.010 Retention Time 1.429

HPLC and LCMS Traces for Compound 6d

o2h discovery
Ahmedabad, Gujarat
India

HPLC Analysis Report

Sample name: CIN2-C-817-CIN2-X-0054-087
Location: P2-A3
Injection: 1 of 1
Injection volume: 10.000
Project Name HPLC_07_NOV-2024
Date Acquired: 2024-11-13 16:54:31+05:30
Date Processed: 2024-11-13 17:52:21+05:30

Instrument Name HPLC-07
Acq. method: o2h_HPLC_Method-D.amx
Processing method: 3D UV Quantitative_DefaultMethod.pmx
Column: XBridge C18 150x4.6mm, 3.5um

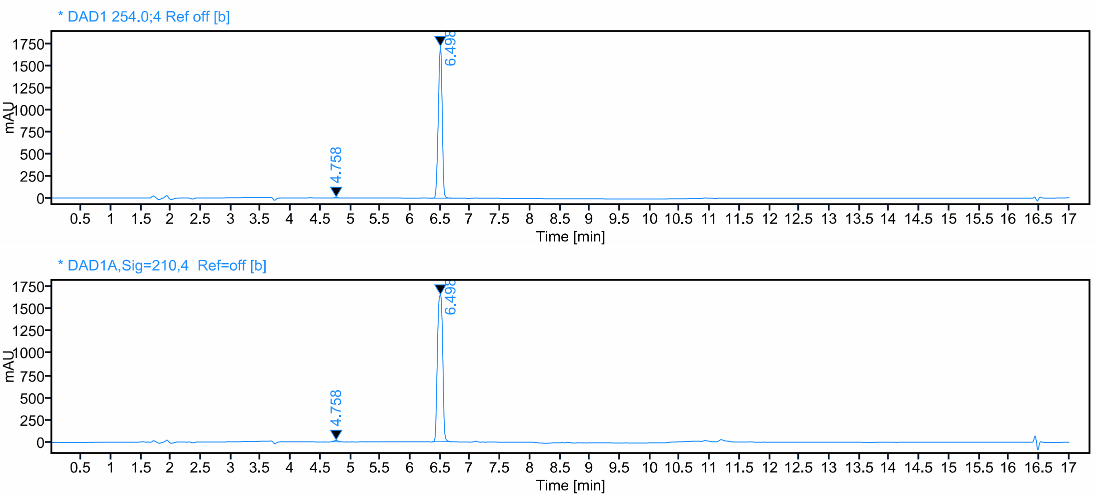

Signal: * DAD1 254.0;4 Ref off [b]

RT [min]	Height	Area	Area%
4.758	6	26	0.34
6.498	1719	7613	99.66

Signal: * DAD1A,Sig=210,4 Ref=off [b]

RT [min]	Height	Area	Area%
4.758	16	67	0.67
6.498	1655	9931	99.33

HPLC and LCMS Traces for Compound 6e

o2h discovery Ahmedabad, Gujarat India	**LCMS Analysis Report**

Sample Name:	CIN2-C-817-CIN2-X-0056-088		Injection Id 24896
Sample Type:	Unknown	Acquired By:	LCMS-05
Vial:	1:E,1	Sample Set Name:	13112024_UCH_090_FD
Injection #:	1	Acq. Method Set:	o2h_LCMS_Method_A
Injection Volume:	2.00 ul	Processing Method	O2H_LCMS_02_0, MASS_000
Run Time:	4.0 Minutes	Channel Name:	MS TIC, 254.0nm, 210.0nm
Project Name:	2024\LCMS-05_NOV-2024	Proc. Chnl. Descr.:	QDa 1: MS Scan MS TIC,
Date Acquired:	13-11-2024 11:40:31 IST		
Date Processed:	13-11-2024 11:53:19 IST, 13-11-2024 11:53:26 IST, 13-11-2024 11:53:45 IST		
Column:	X-BRIDGE C18 2.1X50mm 2.5um		

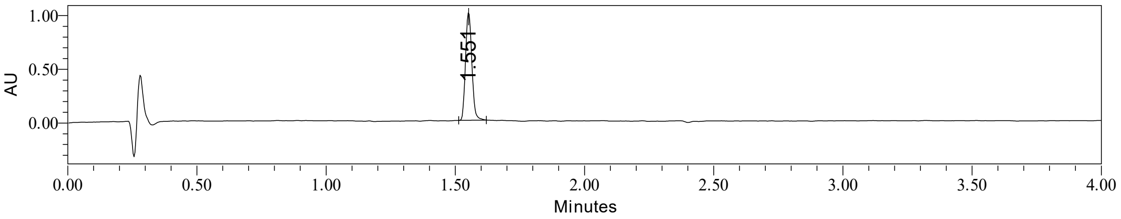

Channel Name 210.0nm; Channel PDA Spectrum

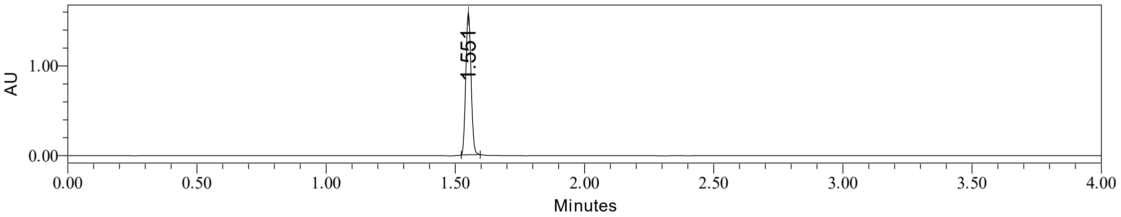

Channel Name 254.0nm; Channel PDA Spectrum

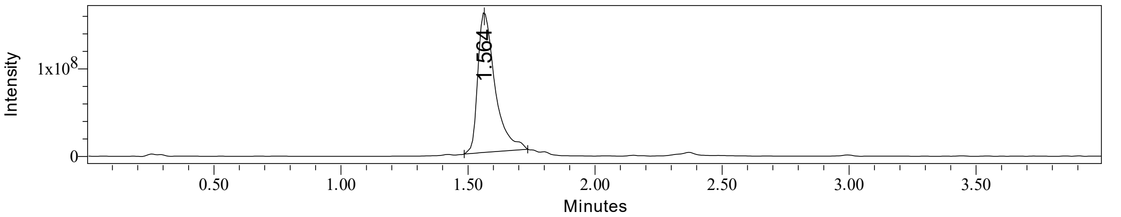

Channel Name MS TIC; Channel QDa 1: MS Scan

Peak Results
Channel: PDA Spectrum

	RT	Base Peak (m/z)	Height	Area	% Area	Channel	Channel Name
1	1.551		1583667	2240564	100.00	PDA Spectrum	254.0nm
2	1.551		1003385	1703890	100.00	PDA Spectrum	210.0nm

Peak Results
Channel: QDa 1: MS Scan

	RT	Base Peak (m/z)	Height	Area	% Area	Channel	Channel Name
1	1.564	316.20	161106558	751235845	100.00	QDa 1: MS Scan	MS TIC

HPLC and LCMS Traces for Compound 6e

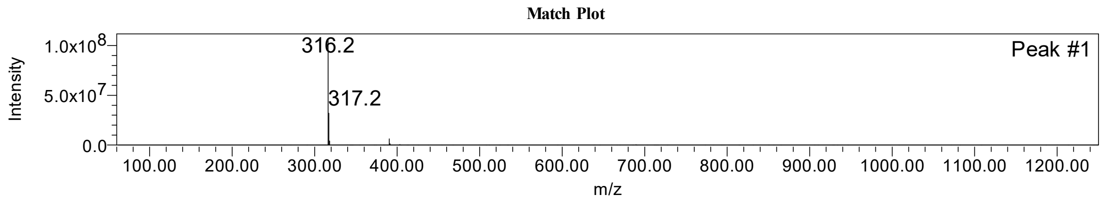

Base Peak 316.20 Channel Description 1: QDa Positive(+) Scan (60.00-1250.00)Da, Centroid, CV=10 - AVG (2.6:2.9;0.8:1.2) x 20.000 Th: 0.010 Retention Time 1.564

HPLC and LCMS Traces for Compound 6e

o2h discovery
Ahmedabad, Gujarat
India

HPLC Analysis Report

**Sample name:**	CIN2-C-817-CIN2-X-0056-088		
**Location:**	P1-B2	**Instrument Name**	HPLC-10
**Injection:**	1 of 1	**Acq. method:**	o2h_HPLC_Method-A.amx
**Injection volume:**	10.000	**Processing method:**	*3D UV Quantitative_DefaultMethod.pmx
**Project Name**	HPLC-10-NOV-2024		
**Date Acquired:**	2024-11-13 15:00:35+05:30	**Column:**	SUNFIRE C18 150x4.6mm,3.5um
**Date Processed:**	2024-11-13 15:26:37+05:30		

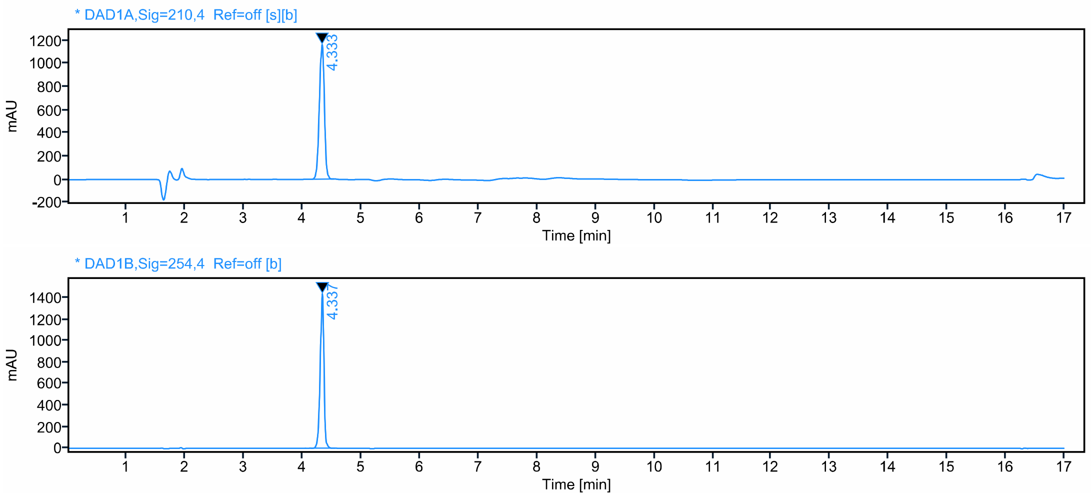

Signal: * DAD1A,Sig=210,4 Ref=off [s][b]

RT [min]	Height	Area	Area%
4.333	1161	6937	100.00

Signal: * DAD1B,Sig=254,4 Ref=off [b]

RT [min]	Height	Area	Area%
4.337	1439	6402	100.00

HPLC and LCMS Traces for Compound 6f

o2h discovery
Ahmedabad, Gujarat
India

LCMS Analysis Report

Sample Name:	CIN2-D-044-CIN2-X-0063-002		Injection Id 56266
Sample Type:	Unknown	Acquired By:	LCMS-05
Vial:	1:B,2	Sample Set Name:	26112024_UCH_090_FD_01
Injection #:	1	Acq. Method Set:	o2h_LCMS_Method_A
Injection Volume:	2.00 ul	Processing Method	O2H_LCMS_02_0, MASS_000
Run Time:	4.0 Minutes	Channel Name:	MS TIC, 254.0nm, 210.0nm
Project Name:	2024\LCMS-05_NOV-2024	Proc. Chnl. Descr.:	QDa 1: MS Scan MS TIC,
Date Acquired:	26-11-2024 09:45:15 IST		
Date Processed:	26-11-2024 09:58:53 IST, 26-11-2024 09:59:02 IST, 26-11-2024 09:59:32 IST		
Column:	X-BRIDGE C18 2.1X50mm 2.5um		

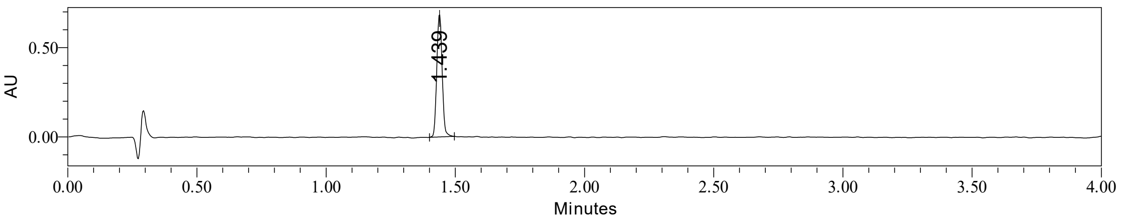

Channel Name 210.0nm; Channel PDA Spectrum

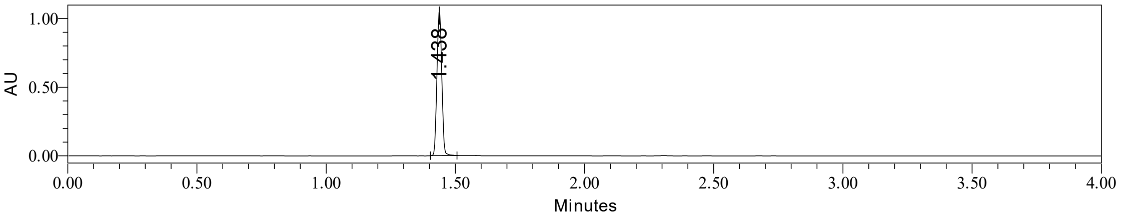

Channel Name 254.0nm; Channel PDA Spectrum

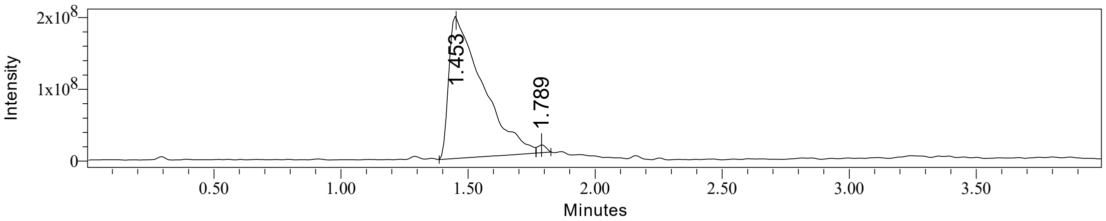

Channel Name MS TIC; Channel QDa 1: MS Scan

Peak Results
Channel: PDA Spectrum

	RT	Base Peak (m/z)	Height	Area	% Area	Channel	Channel Name
1	1.438		1049327	1359995	100.00	PDA Spectrum	254.0nm
2	1.439		689736	991359	100.00	PDA Spectrum	210.0nm

Peak Results
Channel: QDa 1: MS Scan

	RT	Base Peak (m/z)	Height	Area	% Area	Channel	Channel Name
1	1.453	316.24	198508121	1827704659	98.62	QDa 1: MS Scan	MS TIC

HPLC and LCMS Traces for Compound 6f

Peak Results
Channel: QDa 1: MS Scan

	RT	Base Peak (m/z)	Height	Area	% Area	Channel	Channel Name
2	1.789	316.05	10893741	25664954	1.38	QDa 1: MS Scan	MS TIC

Match Plot

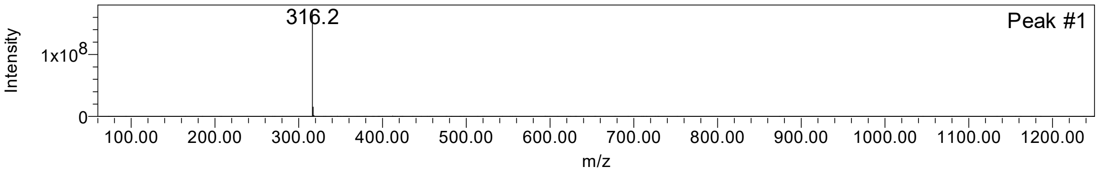

Base Peak 316.24 Channel Description 1: QDa Positive(+) Scan (60.00-1250.00)Da, Centroid, CV=10 - AVG (2.4:4.0;0.0:1.1) x 10.000 Th: 0.010 Retention Time 1.453

Match Plot

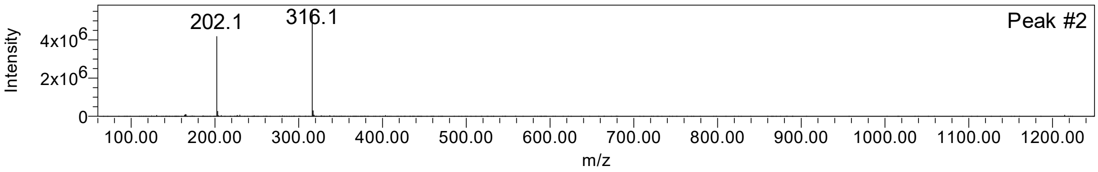

Base Peak 316.05 Channel Description 1: QDa Positive(+) Scan (60.00-1250.00)Da, Centroid, CV=10 - AVG (2.4:4.0;0.0:1.1) x 10.000 Th: 0.010 Retention Time 1.789

HPLC and LCMS Traces for Compound 6f

o2h discovery
Ahmedabad, Gujarat
India

HPLC Analysis Report

Sample name: CIN2-D-044-CIN2-X-0063-002
Location: P1-A3
Injection: 1 of 1
Injection volume: 10.000
Project Name HPLC-10-NOV-2024
Date Acquired: 2024-11-26 09:58:29+05:30
Date Processed: 2024-11-26 10:18:40+05:30

Instrument Name HPLC-10
Acq. method: o2h_HPLC_Method-A.amx
Processing method: 3D UV Quantitative_DefaultMethod.pmx
Column: SUNFIRE C18 150x4.6mm,3.5um

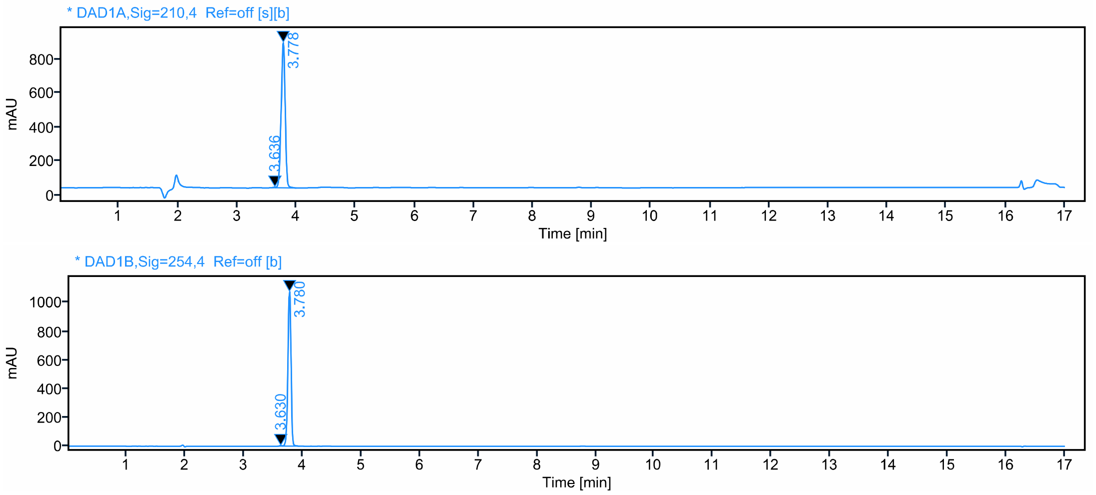

Signal: * DAD1A,Sig=210,4 Ref=off [s][b]

RT [min]	Height	Area	Area%
3.636	3	8	0.19
3.778	859	4124	99.81

Signal: * DAD1B,Sig=254,4 Ref=off [b]

RT [min]	Height	Area	Area%
3.630	3	9	0.24
3.780	1078	3869	99.76

HPLC and LCMS Traces for Compound 6g

o2h discovery
Ahmedabad, Gujarat
India

LCMS Analysis Report

Sample Name:	CIN2-C-818-CIN2-X-0061-085-C		Injection Id 4258
Sample Type:	Unknown	Acquired By:	LCMS-05
Vial:	1:A,6	Sample Set Name:	03122024_UCH_090_FD
Injection #:	1	Acq. Method Set:	o2h_LCMS_Method_A
Injection Volume:	2.00 ul	Processing Method	O2H_LCMS_02_0, MASS_000
Run Time:	4.0 Minutes	Channel Name:	MS TIC, 254.0nm, 210.0nm
Project Name:	2024\LCMS-05_DEC-2024_	Proc. Chnl. Descr.:	PDA 254.0 nm Blank Subtracted
Date Acquired:	03-12-2024 09:34:50 IST		
Date Processed:	03-12-2024 10:11:05 IST, 03-12-2024 10:11:14 IST, 03-12-2024 10:11:48 IST		
Column:	X-BRIDGE C18 2.1X50mm 2.5um		

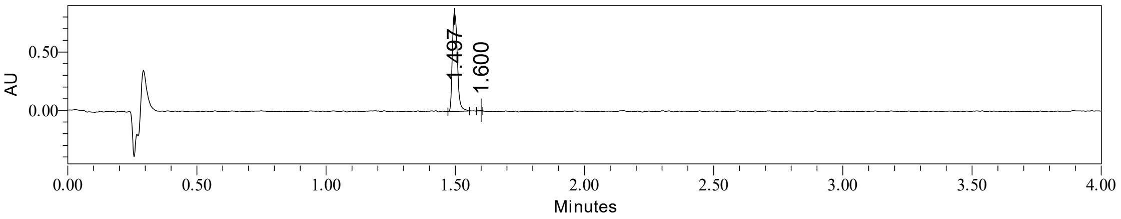

Channel Name 210.0nm; Channel PDA Spectrum

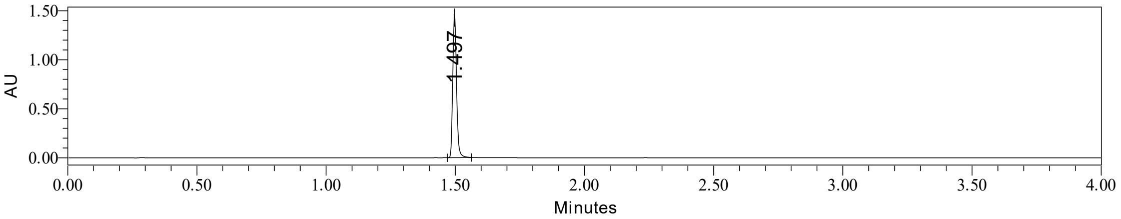

Channel Name 254.0nm; Channel PDA Spectrum

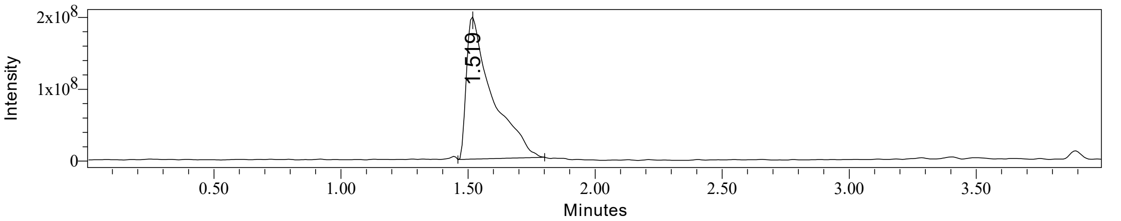

Channel Name MS TIC; Channel QDa 1: MS Scan

Peak Results
Channel: PDA Spectrum

	RT	Base Peak (m/z)	Height	Area	% Area	Channel	Channel Name
1	1.497		846446	1140409	99.63	PDA Spectrum	210.0nm
2	1.497		1461127	1481496	100.00	PDA Spectrum	254.0nm
3	1.600		5607	4212	0.37	PDA Spectrum	210.0nm

HPLC and LCMS Traces for Compound 6g

Peak Results
Channel: QDa 1: MS Scan

	RT	Base Peak (m/z)	Height	Area	% Area	Channel	Channel Name
1	1.519	334.19	197964089	1391668530	100.00	QDa 1: MS Scan	MS TIC

Match Plot

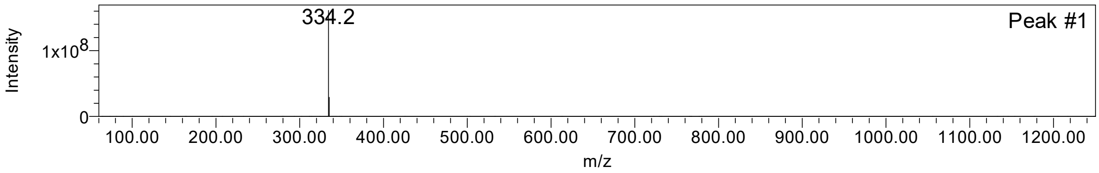

Base Peak 334.19 Channel Description 1: QDa Positive(+) Scan (60.00-1250.00)Da, Centroid, CV=10 - AVG (2.3:3.9;0.0:1.0) x 20.000 Th: 0.010 Retention Time 1.519

HPLC and LCMS Traces for Compound 6g

o2h discovery<br>Ahmedabad, Gujarat<br>India	**HPLC Analysis Report**

Sample name: CIN2-C-818-CIN2-X-0061-085-C
Location: P2-D6
Injection: 1 of 1
Injection volume: 10.000
Project Name HPLC-07_DEC-2024
Date Acquired: 2024-12-03 11:51:06+05:30
Date Processed: 2024-12-03 12:20:48+05:30

Instrument Name HPLC-07
Acq. method: o2h_HPLC_Method-D.amx
Processing method: *3D UV Quantitative_DefaultMethod.pmx
Column: XBridge C18 150x4.6mm, 3.5um

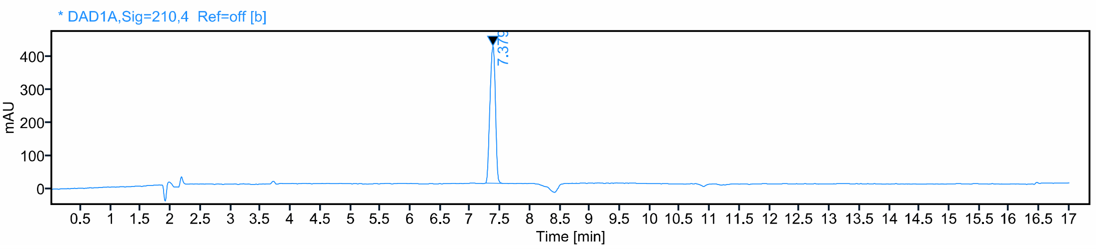

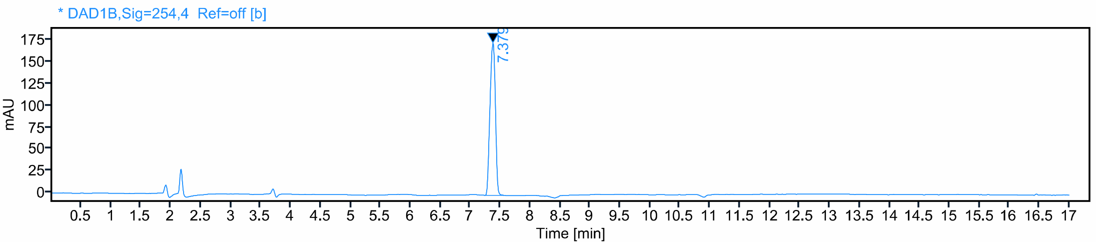

Signal: * DAD1A,Sig=210,4 Ref=off [b]

RT [min]	Height	Area	Area%
7.379	414	2602	100.00

Signal: * DAD1B,Sig=254,4 Ref=off [b]

RT [min]	Height	Area	Area%
7.379	174	1090	100.00

HPLC and LCMS Traces for Compound 6j

o2h discovery
Ahmedabad, Gujarat
India

LCMS Analysis Report

Sample Name:	CIN2-D-044-CIN2-X-0064-005-a		Injection Id 56324
Sample Type:	Unknown	Acquired By:	LCMS-05
Vial:	1:B,4	Sample Set Name:	26112024_UCH_090_FD_01
Injection #:	1	Acq. Method Set:	o2h_LCMS_Method_A
Injection Volume:	2.00 ul	Processing Method	O2H_LCMS_02, MASS_000
Run Time:	4.0 Minutes	Channel Name:	MS TIC, 254.0nm, 210.0nm
Project Name:	2024\LCMS-05_NOV-2024	Proc. Chnl. Descr.:	QDa 2: MS Scan MS TIC,
Date Acquired:	26-11-2024 10:03:55 IST		
Date Processed:	26-11-2024 10:26:26 IST, 26-11-2024 10:26:32 IST, 26-11-2024 10:27:48 IST		
Column:	X-BRIDGE C18 2.1X50mm 2.5um		

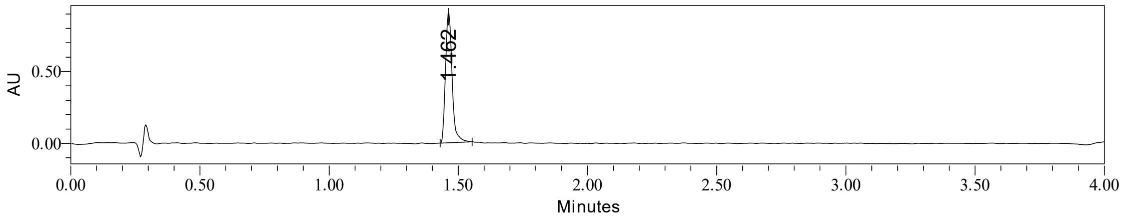

Channel Name 210.0nm; Channel PDA Spectrum

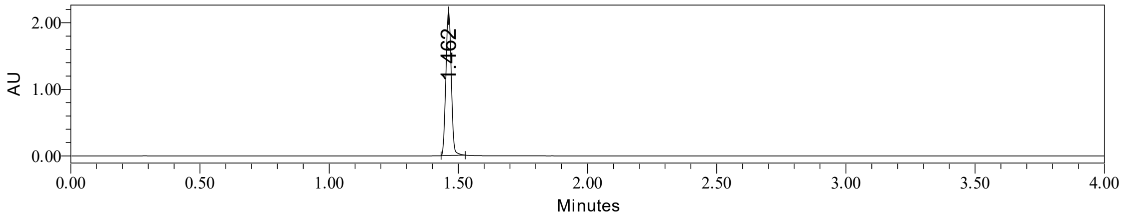

Channel Name 254.0nm; Channel PDA Spectrum

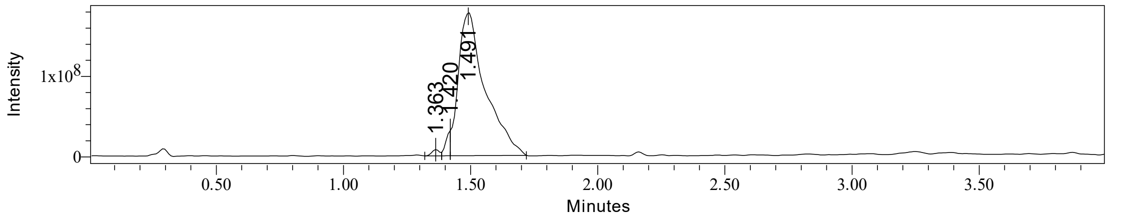

Channel Name MS TIC; Channel QDa 2: MS Scan

Peak Results
Channel: PDA Spectrum

	RT	Base Peak (m/z)	Height	Area	% Area	Channel	Channel Name
1	1.462		2161105	3122509	100.00	PDA Spectrum	254.0nm
2	1.462		905623	1602336	100.00	PDA Spectrum	210.0nm

Peak Results
Channel: QDa 2: MS Scan

	RT	Base Peak (m/z)	Height	Area	% Area	Channel	Channel Name
1	1.363	332.09	7828381	16363148	1.21	QDa 2: MS Scan	MS TIC

O2h Discovery Page: 1 of 2

HPLC and LCMS Traces for Compound 6j

Peak Results
Channel: QDa 2: MS Scan

	RT	Base Peak (m/z)	Height	Area	% Area	Channel	Channel Name
2	1.420	332.10	31457575	36226769	2.67	QDa 2: MS Scan	MS TIC
3	1.491	346.22	177976572	1301735125	96.12	QDa 2: MS Scan	MS TIC

Match Plot

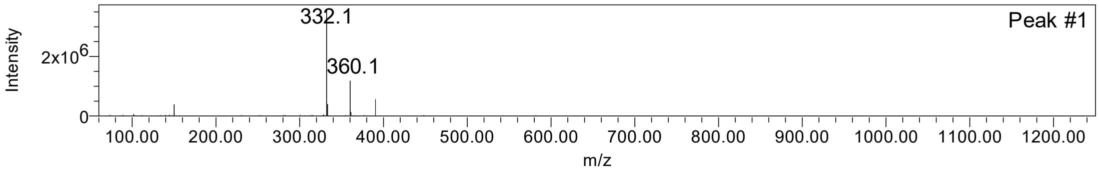

Base Peak 332.09 Channel Description 2: QDa Positive(+) Scan (60.00-1250.00)Da, Centroid, CV=30 - AVG (1.8:4.0;0.3:1.2) x 10.000 Th: 0.010 Retention Time 1.363

Match Plot

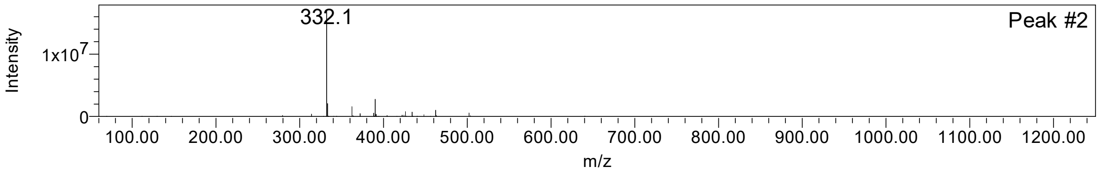

Base Peak 332.10 Channel Description 2: QDa Positive(+) Scan (60.00-1250.00)Da, Centroid, CV=30 - AVG (1.8:4.0;0.3:1.2) x 10.000 Th: 0.010 Retention Time 1.420

Match Plot

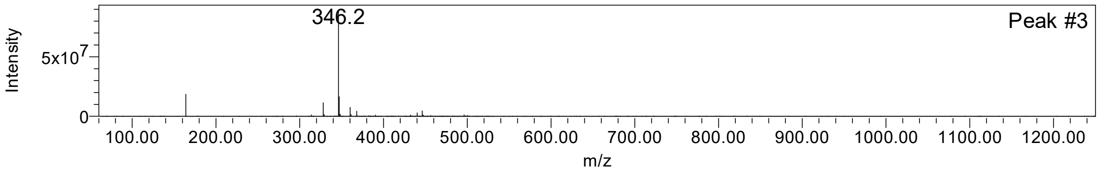

Base Peak 346.22 Channel Description 2: QDa Positive(+) Scan (60.00-1250.00)Da, Centroid, CV=30 - AVG (1.8:4.0;0.3:1.2) x 10.000 Th: 0.010 Retention Time 1.491

HPLC and LCMS Traces for Compound 6j

o2h discovery
Ahmedabad, Gujarat
India

HPLC Analysis Report

**Sample name:**	CIN2-D-044-CIN2-X-0064-005-a		
**Location:**	P1-A5	**Instrument Name**	HPLC-10
**Injection:**	1 of 1	**Acq. method:**	o2h_HPLC_Method-A.amx
**Injection volume:**	10.000	**Processing method:**	*3D UV Quantitative_DefaultMethod.pmx
**Project Name**	HPLC-10-NOV-2024		
**Date Acquired:**	2024-11-26 10:56:19+05:30	**Column:**	SUNFIRE C18 150x4.6mm,3.5um
**Date Processed:**	2024-11-26 11:20:50+05:30		

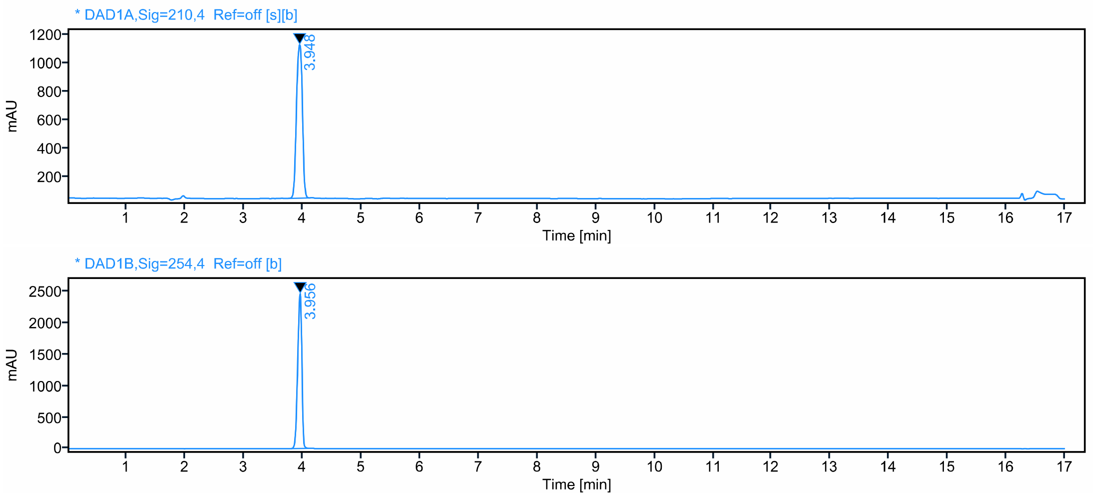

Signal: * DAD1A,Sig=210,4 Ref=off [s][b]

RT [min]	Height	Area	Area%
3.948	1082	7536	100.00

Signal: * DAD1B,Sig=254,4 Ref=off [b]

RT [min]	Height	Area	Area%
3.956	2471	12115	100.00

HPLC and LCMS Traces for Compound 6k

o2h discovery
Ahmedabad, Gujarat
India

LCMS Analysis Report

Sample Name:	CIN2-C-816-CIN2-X-0066-086		Injection Id 14137
Sample Type:	Unknown	Acquired By:	LCMS-05
Vial:	2:F,4	Sample Set Name:	06122024_UCH_090_RD
Injection #:	1	Acq. Method Set:	o2h_LCMS_Method_A
Injection Volume:	2.00 ul	Processing Method	MASS_000, O2H_LCMS_02_00
Run Time:	4.0 Minutes	Channel Name:	220.0nm, MS TIC, 254.0nm
Project Name:	2024\LCMS-05_DEC-2024_	Proc. Chnl. Descr.:	PDA 254.0 nm Blank Subtracted
Date Acquired:	06-12-2024 10:31:06 IST		
Date Processed:	06-12-2024 11:07:20 IST, 06-12-2024 11:07:30 IST, 06-12-2024 11:08:28 IST		
Column:	X-BRIDGE C18 2.1X50mm 2.5um		

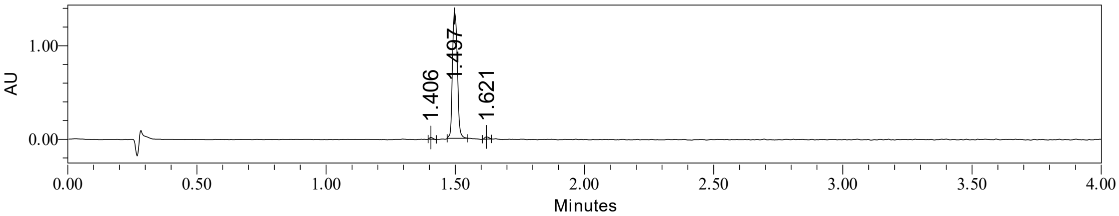

Channel Name 220.0nm; Channel PDA Spectrum

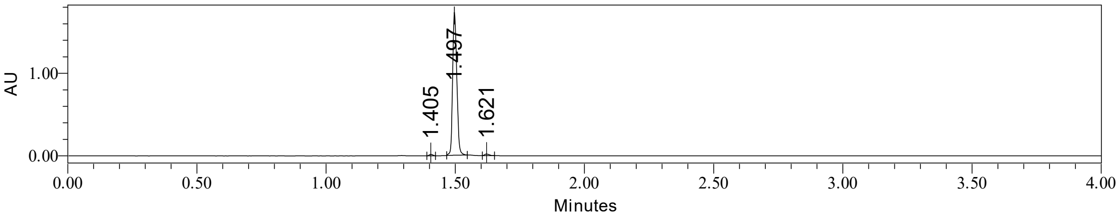

Channel Name 254.0nm; Channel PDA Spectrum

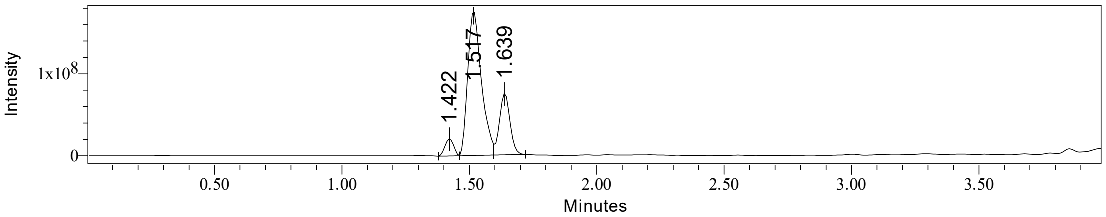

Channel Name MS TIC; Channel QDa 1: MS Scan

Peak Results
Channel: PDA Spectrum

	RT	Base Peak (m/z)	Height	Area	% Area	Channel	Channel Name
1	1.405		17380	16638	0.85	PDA Spectrum	254.0nm
2	1.406		18494	16960	0.93	PDA Spectrum	220.0nm
3	1.497		1729581	1929773	98.03	PDA Spectrum	254.0nm
4	1.497		1349751	1774235	97.59	PDA Spectrum	220.0nm
5	1.621		19132	22129	1.12	PDA Spectrum	254.0nm
6	1.621		23418	26882	1.48	PDA Spectrum	220.0nm

HPLC and LCMS Traces for Compound 6k

Peak Results
Channel: QDa 1: MS Scan

	RT	Base Peak (m/z)	Height	Area	% Area	Channel	Channel Name
1	1.422	332.07	20657854	49565128	5.38	QDa 1: MS Scan	MS TIC
2	1.517	360.22	174933357	655474853	71.17	QDa 1: MS Scan	MS TIC
3	1.639	388.20	74710446	215990723	23.45	QDa 1: MS Scan	MS TIC

Match Plot

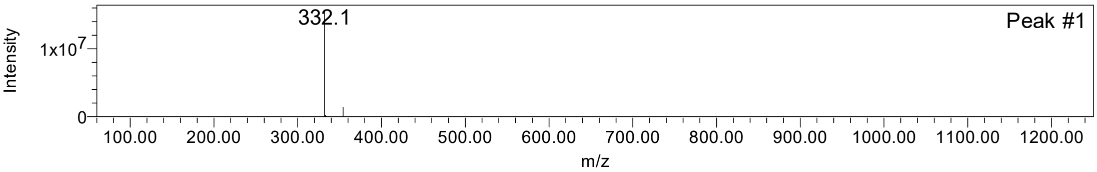

Base Peak 332.07 Channel Description 1: QDa Positive(+) Scan (60.00-1250.00)Da, Centroid, CV=10 - AVG (0.1:1.4;1.9:3.8) x 20.000 Th: 0.010 - AVG (1.0:1.4;1.8:1.9) x 20.000 Th: 0.010 Retention Time 1.422

Match Plot

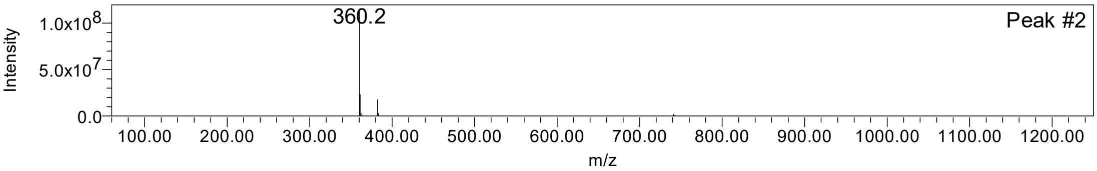

Base Peak 360.22 Channel Description 1: QDa Positive(+) Scan (60.00-1250.00)Da, Centroid, CV=10 - AVG (0.1:1.4;1.9:3.8) x 20.000 Th: 0.010 - AVG (1.0:1.4;1.8:1.9) x 20.000 Th: 0.010 Retention Time 1.517

Match Plot

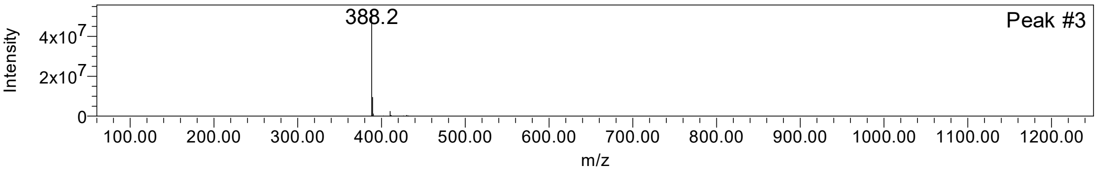

Base Peak 388.20 Channel Description 1: QDa Positive(+) Scan (60.00-1250.00)Da, Centroid, CV=10 - AVG (0.1:1.4;1.9:3.8) x 20.000 Th: 0.010 - AVG (1.0:1.4;1.8:1.9) x 20.000 Th: 0.010 Retention Time 1.639

HPLC and LCMS Traces for Compound 6k

o2h discovery
Ahmedabad, Gujarat
India

HPLC Analysis Report

Sample name: CIN2-C-816-CIN2-X-0066-086
Location: P2-B4
Injection: 1 of 1
Injection volume: 10.000
Project Name HPLC-07_DEC-2024
Date Acquired: 2024-12-06 14:55:12+05:30
Date Processed: 2024-12-06 15:30:33+05:30

Instrument Name HPLC-07
Acq. method: o2h_HPLC_Method-A.amx
Processing method: *3D UV Quantitative_DefaultMethod.pmx
Column: SUNFIRE C18 150x4.6mm 3.5um

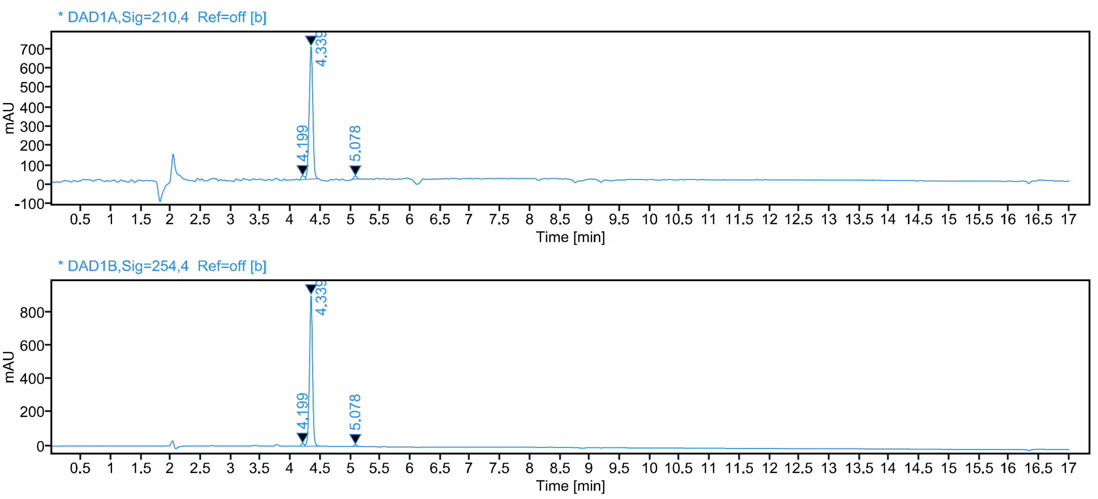

Signal: * DAD1A,Sig=210,4 Ref=off [b]

RT [min]	Height	Area	Area%
4.199	20	78	2.58
4.339	686	2887	95.70
5.078	16	52	1.72

Signal: * DAD1B,Sig=254,4 Ref=off [b]

RT [min]	Height	Area	Area%
4.199	16	49	1.45
4.339	908	3294	97.56
5.078	10	33	0.99

HPLC and LCMS Traces for Compound 7a

o2h discovery<br>Ahmedabad, Gujarat<br>India	**LCMS Analysis Report**

Sample Name:	CIN-C-377-CIN-X-0037-042-a		Injection Id 37332
Sample Type:	Unknown	Acquired By:	LCMS-05
Vial:	2:A,2	Sample Set Name:	22052024_UCH_083_FD
Injection #:	1	Acq. Method Set:	o2h_LCMS_Method_A
Injection Volume:	2.00 ul	Processing Method:	O2H_LCMS_03_0,
Run Time:	4.0 Minutes	Channel Name:	MS TIC, 254.0nm, 210.0nm
Project Name:	2024\LCMS-05_MAY-2024	Proc. Chnl. Descr.:	QDa 1: MS Scan MS TIC,
Date Acquired:	22-05-2024 12:08:08 IST		
Date Processed:	22-05-2024 12:09:53 IST, 22-05-2024 12:10:02 IST, 22-05-2024 12:12:50 IST		
Column:	X-BRIDGE C18 2.1X50mm 2.5um		

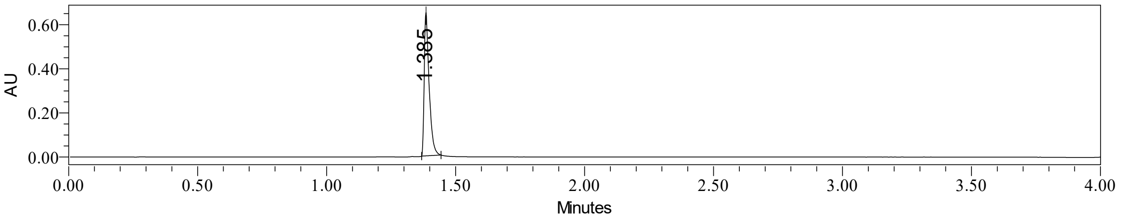

Channel Name 254.0nm; Channel PDA Spectrum

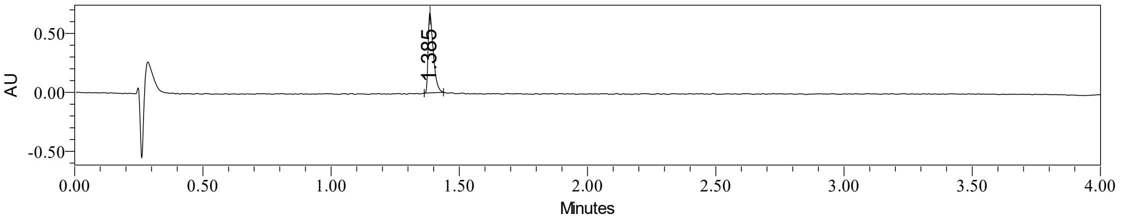

Channel Name 210.0nm; Channel PDA Spectrum

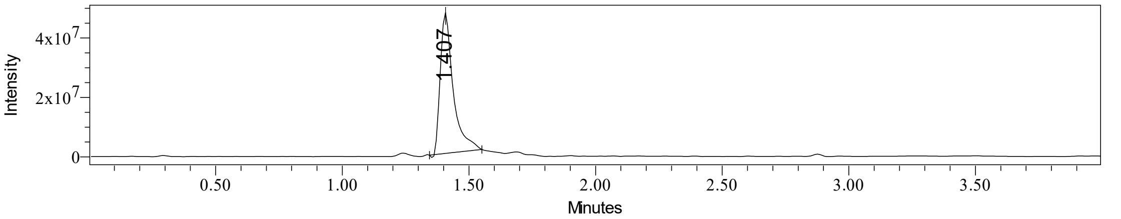

Channel Name MS TIC; Channel QDa 1: MS Scan

Peak Results
Channel: PDA Spectrum

	RT	Base Peak (m/z)	Height	Area	% Area	Channel	Channel Name
1	1.385		649919	855554	100.00	PDA Spectrum	254.0nm
2	1.385		681277	945935	100.00	PDA Spectrum	210.0nm

Peak Results
Channel: QDa 1: MS Scan

	RT	Base Peak (m/z)	Height	Area	% Area	Channel	Channel Name
1	1.407	344.27	46969330	169513007	100.00	QDa 1: MS Scan	MS TIC

HPLC and LCMS Traces for Compound 7a

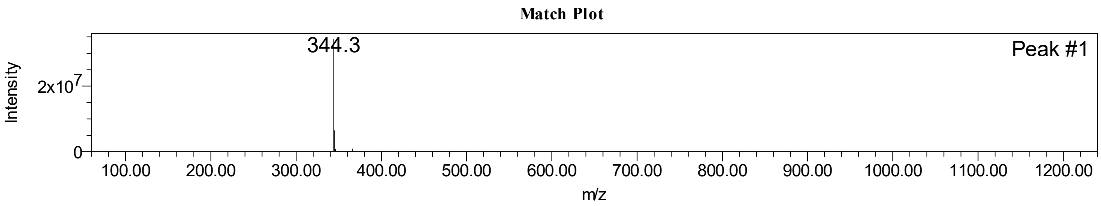

Base Peak 344.27 Channel Description 1: QDa Positive(+) Scan (60.00-1250.00)Da, Centroid, CV=10 - AVG (2.4:3.4;0.1:0.5) x 20.010 Th: 0.010 Retention Time 1.407

HPLC and LCMS Traces for Compound 7a

o2h discovery<br>Ahmedabad, Gujarat<br>India	**HPLC Analysis Report**

Sample name: CIN-C-377-CIN-X-0037-042-a
Location: P1-B3
Injection: 1 of 1
Injection volume: 10.000
Project Name HPLC-10_MAY-2024
Date Acquired: 2024-05-22 13:22:38+05:30
Date Processed: 2024-05-22 13:42:57+05:30

Instrument Name HPLC-10
Acq. method: o2h_HPLC_Method-A.amx
Processing method: *3D UV Quantitative_DefaultMethod.pmx
Column: SUNFIRE C18 150x4.6mm,3.5um

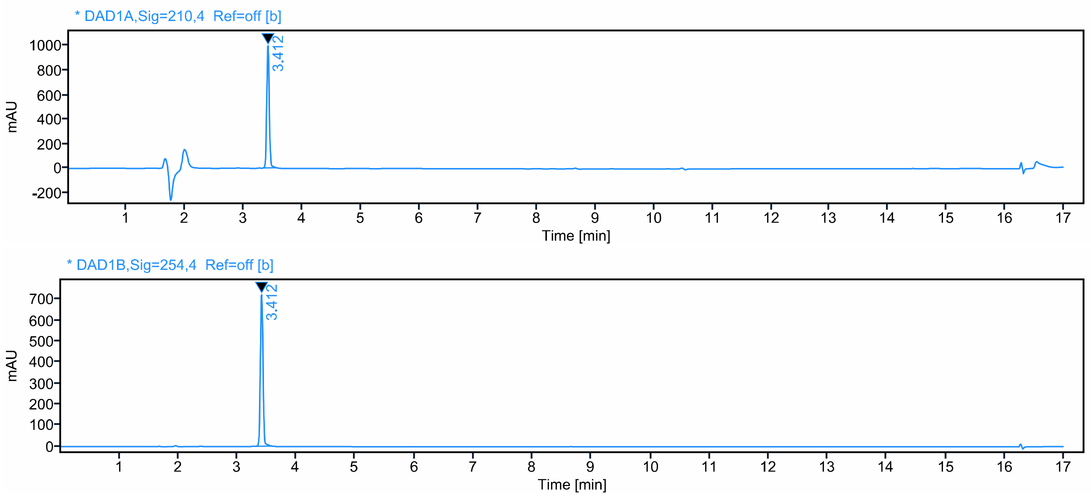

Signal: * DAD1A,Sig=210,4 Ref=off [b]

RT [min]	Height	Area	Area%
3.412	1006	3014	100.00

Signal: * DAD1B,Sig=254,4 Ref=off [b]

RT [min]	Height	Area	Area%
3.412	728	2177	100.00

HPLC and LCMS Traces for Compound 7b

o2h discovery<br>Ahmedabad, Gujarat<br>India	**LCMS Analysis Report**

Sample Name: CIN-C-377-CIN-X-0039-081-a | Injection Id 60016
Sample Type: Unknown | Acquired By: LCMS-05
Vial: 2:A,1 | Sample Set Name: 29062024_UCH_082_RD_01
Injection #: 1 | Acq. Method Set: o2h_LCMS_Method_A_SLOW_01
Injection Volume: 2.00 ul | Processing Method O2H_LCMS_02_0,
Run Time: 4.0 Minutes | Channel Name: MS TIC, 254.0nm, 210.0nm
Project Name: 2024\LCMS-05_JUN-2024 | Proc. Chnl. Descr.: PDA 254.0 nm Blank Subtracted
Date Acquired: 29-06-2024 13:24:49 IST
Date Processed: 29-06-2024 14:04:41 IST, 29-06-2024 14:04:47 IST, 29-06-2024 14:05:28 IST
Column: X-BRIDGE C18 2.1X50mm 2.5um

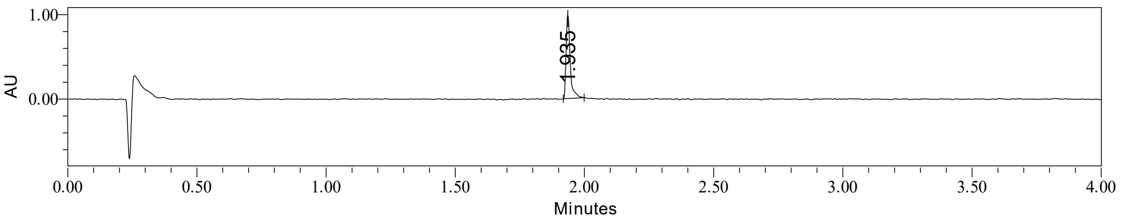

——— Channel PDA Spectrum; Channel Name 210.0nm

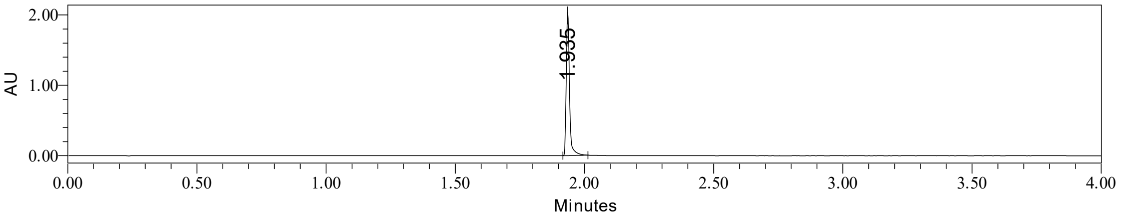

——— Channel PDA Spectrum; Channel Name 254.0nm

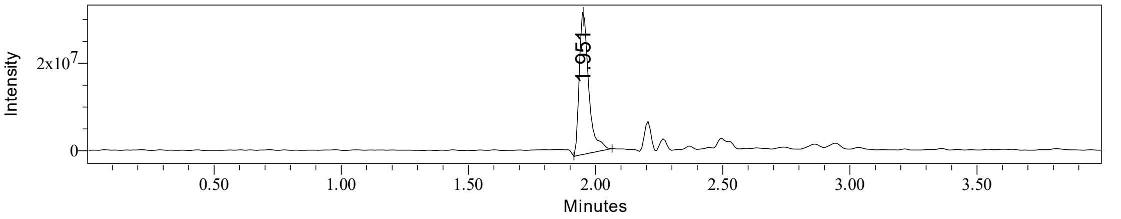

——— Channel QDa 1: MS Scan; Channel Name MS TIC

Peak Results
Channel: PDA Spectrum

	RT	Base Peak (m/z)	Height	Area	% Area	Channel	Channel Name
1	1.935		2033792	1776675	100.00	PDA Spectrum	254.0nm
2	1.935		990688	1099689	100.00	PDA Spectrum	210.0nm

Peak Results
Channel: QDa 1: MS Scan

	RT	Base Peak (m/z)	Height	Area	% Area	Channel	Channel Name
1	1.951	344.32	32987093	83662392	100.00	QDa 1: MS Scan	MS TIC

HPLC and LCMS Traces for Compound 7b

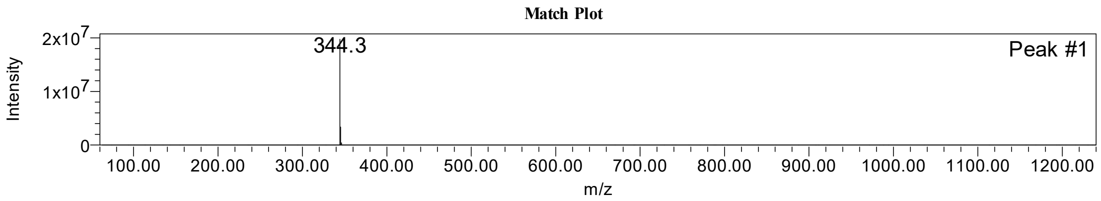

Base Peak 344.32 Channel Description 1: QDa Positive(+) Scan (60.00-1240.00)Da, Centroid, CV=10 - AVG (2.1:4.0;0.0:1.6;2.1:2.1) x 20.000 Th: 0.010 Retention Time 1.951

HPLC and LCMS Traces for Compound 7b

o2h discovery
Ahmedabad, Gujarat
India

HPLC Analysis Report

Sample name: CIN-C-377-CIN-X-0039-081-a
Location: P2-A5
Injection: 1 of 1
Injection volume: 10.000
Project Name HPLC-10_JUNE-2024
Date Acquired: 2024-06-29 12:16:36+05:30
Date Processed: 2024-06-29 12:36:54+05:30

Instrument Name HPLC-10
Acq. method: o2h_HPLC_Method-C.amx
Processing method: *3D UV Quantitative_DefaultMethod.pmx
Column: SUNFIRE C18 150x4.6mm,3.5um

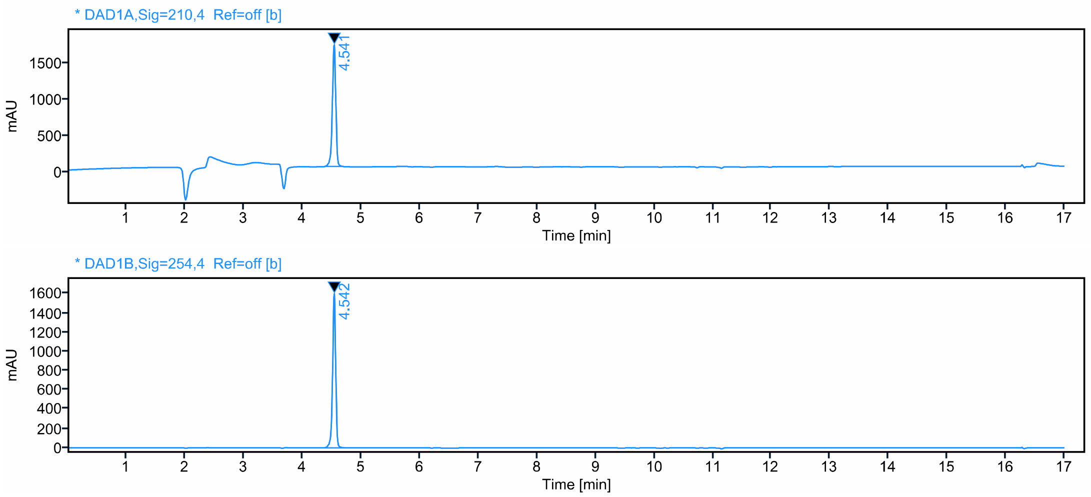

Signal: * DAD1A,Sig=210,4 Ref=off [b]

RT [min]	Height	Area	Area%
4.541	1678	6799	100.00

Signal: * DAD1B,Sig=254,4 Ref=off [b]

RT [min]	Height	Area	Area%
4.542	1604	5863	100.00

HPLC and LCMS Traces for Compound 7c

o2h discovery
Ahmedabad, Gujarat
India

LCMS Analysis Report

Sample Name:	CIN-C-377-CIN-X-0038-062-a		Injection Id 32210
Sample Type:	Unknown	Acquired By:	LCMS-05
Vial:	2:C,4	Sample Set Name:	17062024_UCH_083_RD
Injection #:	1	Acq. Method Set:	o2h_LCMS_Method_A_SLOW
Injection Volume:	2.00 ul	Processing Method	O2H_LCMS_02_00,
Run Time:	4.0 Minutes	Channel Name:	220.0nm, MS TIC, 254.0nm
Project Name:	2024\LCMS-05_JUN-2024	Proc. Chnl. Descr.:	PDA 254.0 nm Blank Subtracted
Date Acquired:	17-06-2024 15:06:54 IST		
Date Processed:	17-06-2024 15:10:58 IST, 17-06-2024 15:11:05 IST, 17-06-2024 15:11:40 IST		
Column:	X-BRIDGE C18 2.1X50mm 2.5um		

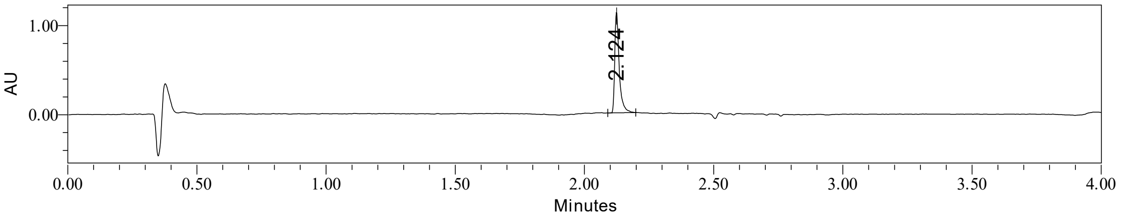

Channel Name 220.0nm; Channel PDA Spectrum

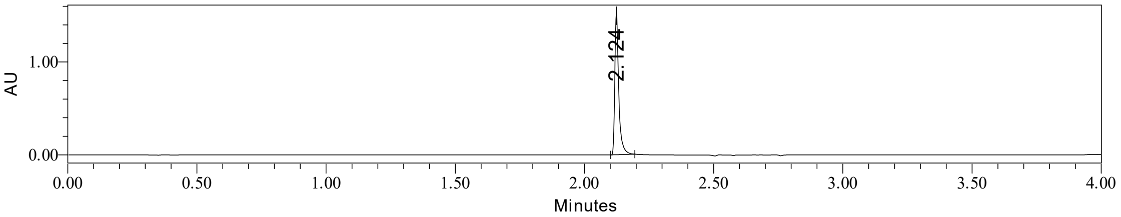

Channel Name 254.0nm; Channel PDA Spectrum

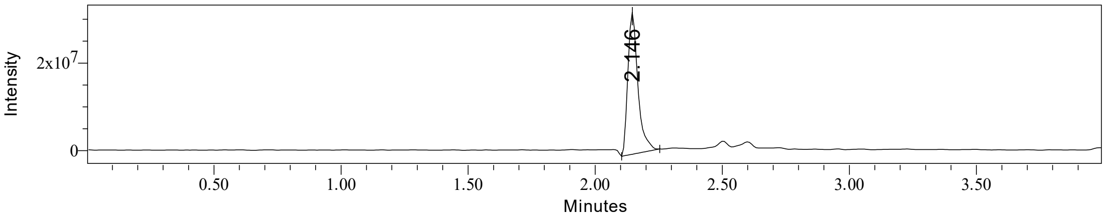

Channel Name MS TIC; Channel QDa 1: MS Scan

Peak Results
Channel: PDA Spectrum

	RT	Base Peak (m/z)	Height	Area	% Area	Channel	Channel Name
1	2.124		1531614	1608632	100.00	PDA Spectrum	254.0nm
2	2.124		1129848	1354863	100.00	PDA Spectrum	220.0nm

Peak Results
Channel: QDa 1: MS Scan

	RT	Base Peak (m/z)	Height	Area	% Area	Channel	Channel Name
1	2.146	344.28	32061739	88811217	100.00	QDa 1: MS Scan	MS TIC

HPLC and LCMS Traces for Compound 7c

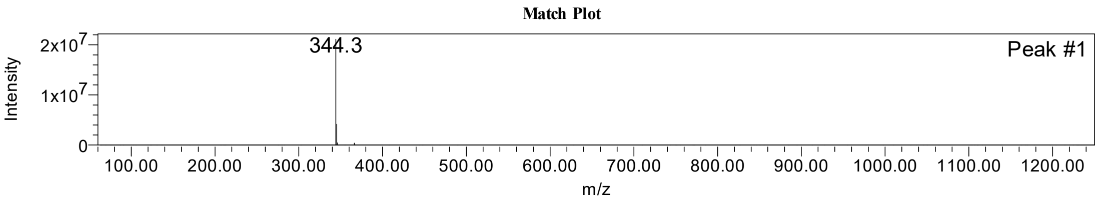

Base Peak 344.28 Channel Description 1: QDa Positive(+) Scan (60.00-1240.00)Da, Centroid, CV=10 - AVG (0.1:1.7;2.4:4.0) x 30.000 Th: 0.010 Retention Time 2.146

HPLC and LCMS Traces for Compound 7c

o2h discovery<br>Ahmedabad, Gujarat<br>India	**HPLC Analysis Report**

**Sample name:**	CIN-C-377-CIN-X-0038-062-a		
**Location:**	P1-B1	**Instrument Name**	HPLC-10
**Injection:**	1 of 1	**Acq. method:**	o2h_HPLC_Method-C.amx
**Injection volume:**	10.000	**Processing method:**	*3D UV Quantitative_DefaultMethod.pmx
**Project Name**	HPLC-10_JUNE-2024		
**Date Acquired:**	2024-06-17 17:38:00+05:30	**Column:**	SUNFIRE C18 150x4.6mm,3.5um
**Date Processed:**	2024-06-17 20:32:11+05:30		

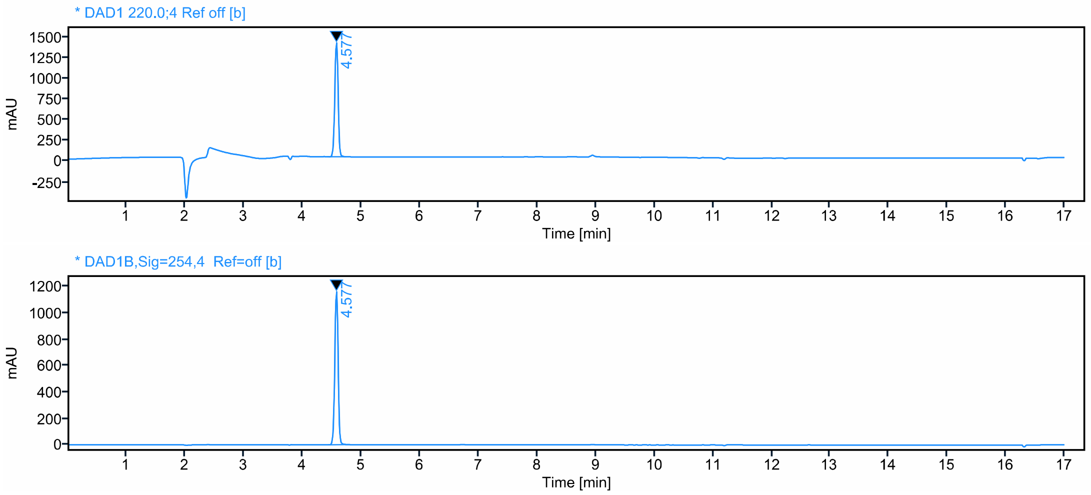

Signal: * DAD1 220.0;4 Ref off [b]

RT [min]	Height	Area	Area%
4.577	1373	5265	100.00

Signal: * DAD1B,Sig=254,4 Ref=off [b]

RT [min]	Height	Area	Area%
4.577	1166	4479	100.00

HPLC and LCMS Traces for Compound 8a

o2h discovery
Ahmedabad, Gujarat
India

LCMS Analysis Report

Sample Name:	CIN-C-377-CIN-X-0028-020-e		Injection Id 15477
Sample Type:	Unknown	Acquired By:	LCMS-05
Vial:	1:E,7	Sample Set Name:	10052024_UCH_077_RD
Injection #:	1	Acq. Method Set:	o2h_LCMS_Method_A
Injection Volume:	2.00 ul	Processing Method:	O2H_LCMS_02_00,
Run Time:	4.0 Minutes	Channel Name:	MS TIC, 254.0nm, 210.0nm
Project Name:	2024\LCMS-05_MAY-2024	Proc. Chnl. Descr.:	PDA 254.0 nm Blank Subtracted
Date Acquired:	10-05-2024 12:35:09 IST		
Date Processed:	10-05-2024 12:49:28 IST, 10-05-2024 12:49:35 IST, 10-05-2024 12:49:56 IST		
Column:	X-BRIDGE C18 2.1X50mm 2.5um		

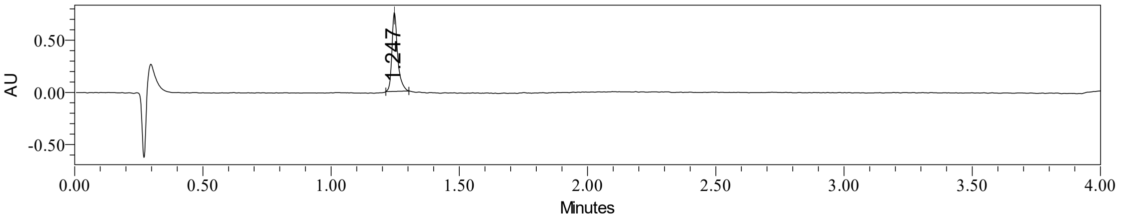

Channel Name 210.0nm; Channel PDA Spectrum

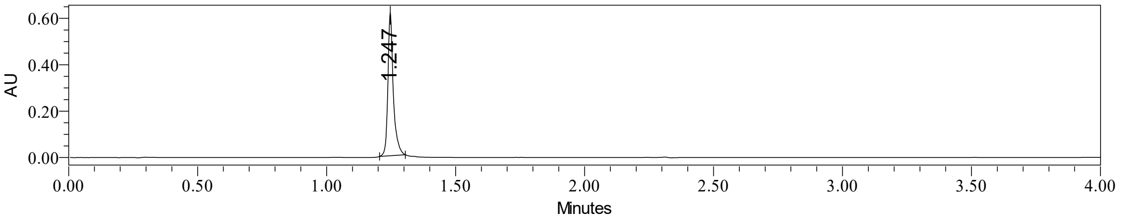

Channel Name 254.0nm; Channel PDA Spectrum

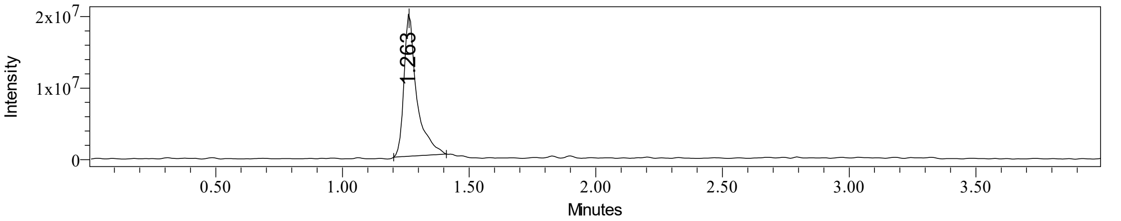

Channel Name MS TIC; Channel QDa 1: MS Scan

Peak Results
Channel: PDA Spectrum

	RT	Base Peak (m/z)	Height	Area	% Area	Channel	Channel Name
1	1.247		754829	1167886	100.00	PDA Spectrum	210.0nm
2	1.247		616656	894402	100.00	PDA Spectrum	254.0nm

Peak Results
Channel: QDa 1: MS Scan

	RT	Base Peak (m/z)	Height	Area	% Area	Channel	Channel Name
1	1.263	305.12	19762312	69426505	100.00	QDa 1: MS Scan	MS TIC

HPLC and LCMS Traces for Compound 8a

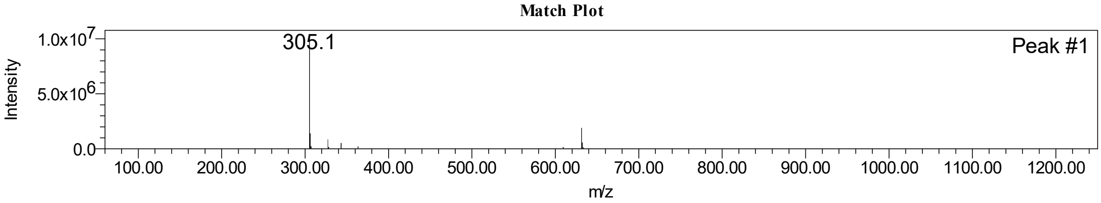

Base Peak 305.12 Channel Description 1: QDa Positive(+) Scan (60.00-1250.00)Da, Centroid, CV=10 - AVG (0.1:0.9;1.5:3.9) x 20.000 Th: 0.010 Retention Time 1.263

HPLC and LCMS Traces for Compound 8a

o2h discovery
Ahmedabad, Gujarat
India

HPLC Analysis Report

**Sample name:**	CIN-C-377-CIN-X-0028-020-e		
**Location:**	P2-B2	**Instrument Name**	HPLC-10
**Injection:**	1 of 1	**Acq. method:**	o2h_HPLC_Method-A.amx
**Injection volume:**	10.000	**Processing method:**	*3D UV Quantitative_DefaultMethod.pmx
**Project Name**	HPLC-10_MAY-2024		
**Date Acquired:**	2024-05-10 14:48:50+05:30	**Column:**	SUNFIRE C18 150x4.6mm,3.5um
**Date Processed:**	2024-05-10 15:30:42+05:30		

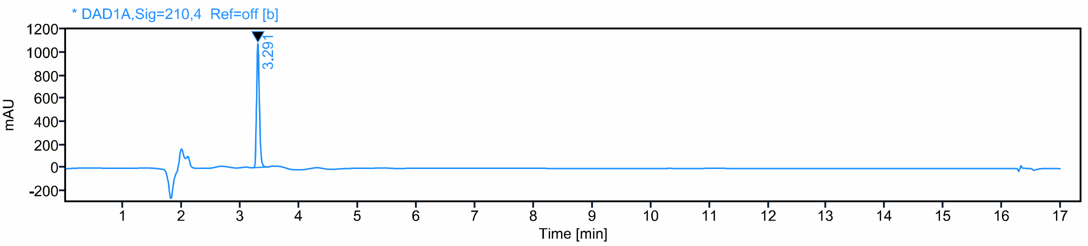

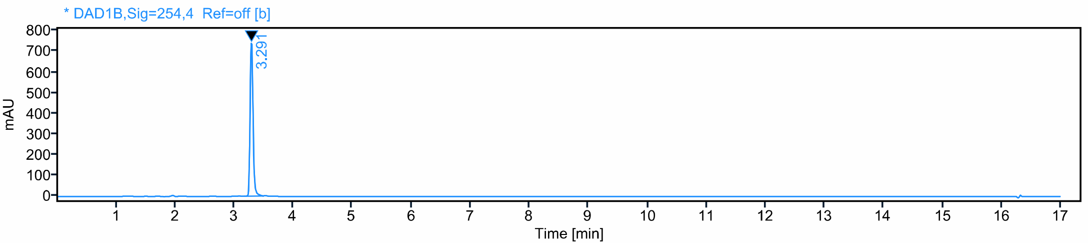

Signal: * DAD1A,Sig=210,4 Ref=off [b]

RT [min]	Height	Area	Area%
3.291	1085	3627	100.00

Signal: * DAD1B,Sig=254,4 Ref=off [b]

RT [min]	Height	Area	Area%
3.291	744	2490	100.00

HPLC and LCMS Traces for Compound 8b

o2h discovery
Ahmedabad, Gujarat
India

LCMS Analysis Report

Sample Name:	CIN-C-377-CIN-X-0030-037- a		Injection Id 37503
Sample Type:	Unknown	Acquired By:	LCMS-05
Vial:	2:A,1	Sample Set Name:	22052024_UCH_083_FD
Injection #:	1	Acq. Method Set:	o2h_LCMS_Method_A_SLOW
Injection Volume:	4.00 ul	Processing Method:	O2H_LCMS_02_00,
Run Time:	4.0 Minutes	Channel Name:	215.0nm, MS TIC, 254.0nm
Project Name:	2024\LCMS-05_MAY-2024	Proc. Chnl. Descr.:	PDA 254.0 nm Blank Subtracted
Date Acquired:	22-05-2024 13:47:00 IST		
Date Processed:	22-05-2024 13:53:17 IST, 22-05-2024 13:53:22 IST, 22-05-2024 13:53:39 IST		
Column:	X-BRIDGE C18 2.1X50mm 2.5um		

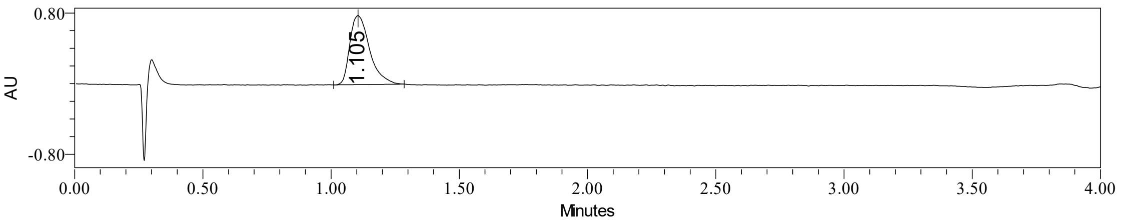

Channel Name 215.0nm; Channel PDA Spectrum

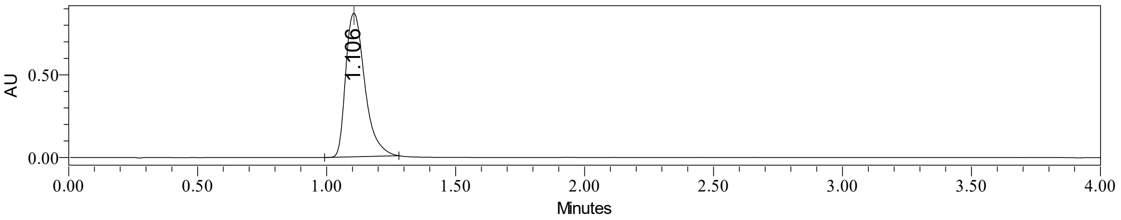

Channel Name 254.0nm; Channel PDA Spectrum

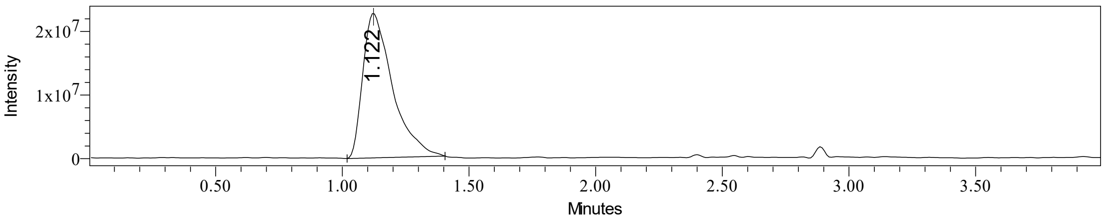

Channel Name MS TIC; Channel QDa 1: MS Scan

Peak Results
Channel: PDA Spectrum

	RT	Base Peak (m/z)	Height	Area	% Area	Channel	Channel Name
1	1.105		782862	4190031	100.00	PDA Spectrum	215.0nm
2	1.106		869839	4472148	100.00	PDA Spectrum	254.0nm

Peak Results
Channel: QDa 1: MS Scan

	RT	Base Peak (m/z)	Height	Area	% Area	Channel	Channel Name
1	1.122	305.17	22778755	185052190	100.00	QDa 1: MS Scan	MS TIC

HPLC and LCMS Traces for Compound 8b

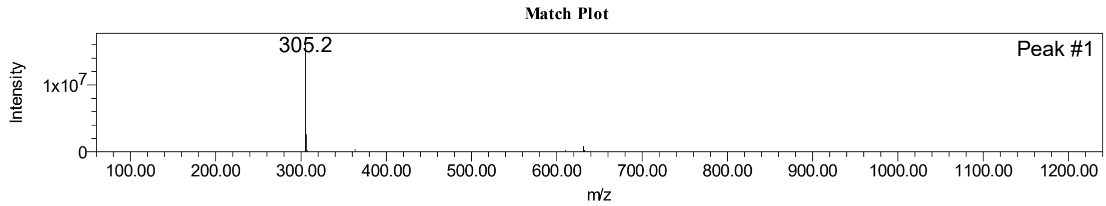

Base Peak 305.17 Channel Description 1: QDa Positive(+) Scan (60.00-1240.00)Da, Centroid, CV=10 - AVG (2.1:3.7) x 20.000 Th: 0.010 Retention Time 1.122

HPLC and LCMS Traces for Compound 8b

o2h discovery
Ahmedabad, Gujarat
India

HPLC Analysis Report

Sample name: CIN-C-377-CIN-X-0030-037- a
Location: P1-B5
Injection: 1 of 1
Injection volume: 10.000
Project Name HPLC-10_MAY-2024
Date Acquired: 2024-05-22 14:30:56+05:30
Date Processed: 2024-05-22 14:55:57+05:30

Instrument Name HPLC-10
Acq. method: o2h_HPLC_Method-C.amx
Processing method: *3D UV Quantitative_DefaultMethod.pmx
Column: SUNFIRE C18 150x4.6mm,3.5um

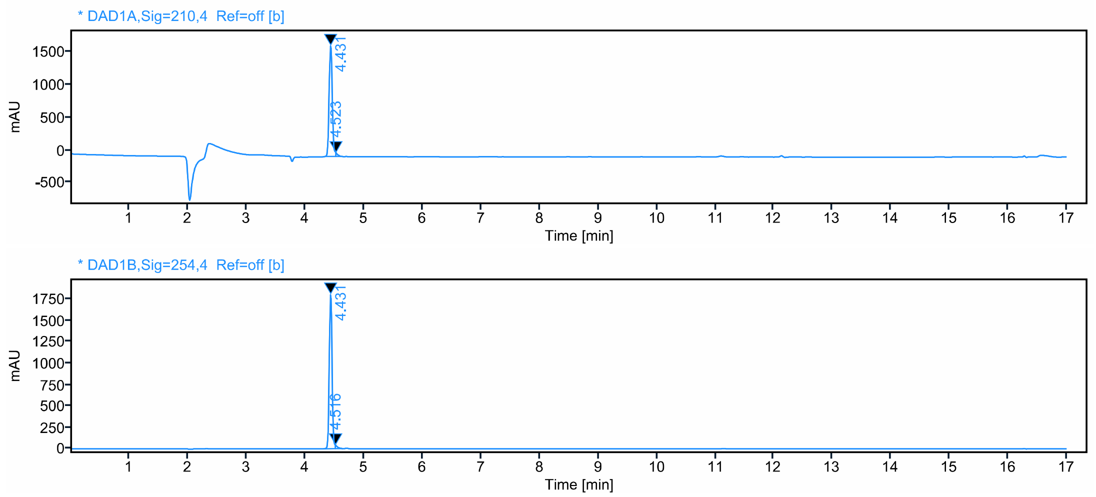

Signal: * DAD1A,Sig=210,4 Ref=off [b]

RT [min]	Height	Area	Area%
4.431	1699	6068	98.09
4.523	57	118	1.91

Signal: * DAD1B,Sig=254,4 Ref=off [b]

RT [min]	Height	Area	Area%
4.431	1802	5701	98.19
4.516	42	105	1.81

HPLC and LCMS Traces for Compound 8c

o2h discovery
Ahmedabad, Gujarat
India

LCMS Analysis Report

Sample Name:	CIN-C-377-CIN-X-0029-044-a		Injection Id 54325
Sample Type:	Unknown	Acquired By:	LCMS-05
Vial:	1:A,2	Sample Set Name:	30052024_UCH_082_FD
Injection #:	1	Acq. Method Set:	o2h_LCMS_Method_A_SLOW
Injection Volume:	3.00 ul	Processing Method:	O2H_LCMS_02_000,
Run Time:	4.0 Minutes	Channel Name:	220.0nm, MS TIC, 254.0nm
Project Name:	2024\LCMS-05_MAY-2024	Proc. Chnl. Descr.:	PDA 254.0 nm Blank Subtracted
Date Acquired:	30-05-2024 16:05:26 IST		
Date Processed:	30-05-2024 16:10:26 IST, 30-05-2024 16:10:29 IST, 30-05-2024 16:10:55 IST		
Column:	X-BRIDGE C18 2.1X50mm 2.5um		

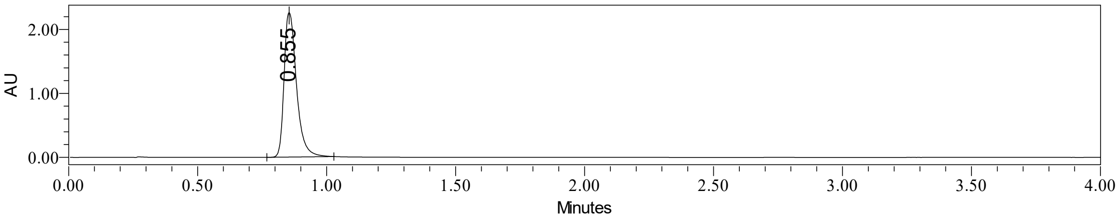

Channel Name 254.0nm; Channel PDA Spectrum

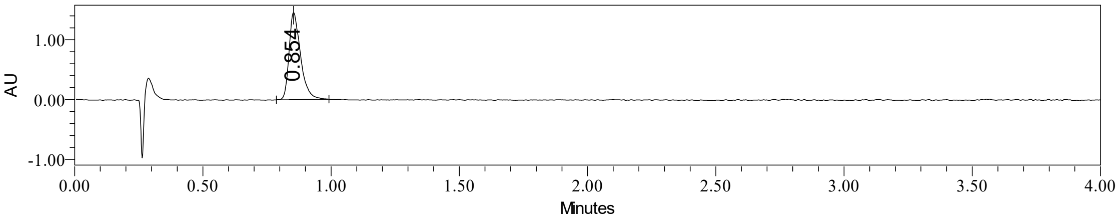

Channel Name 220.0nm; Channel PDA Spectrum

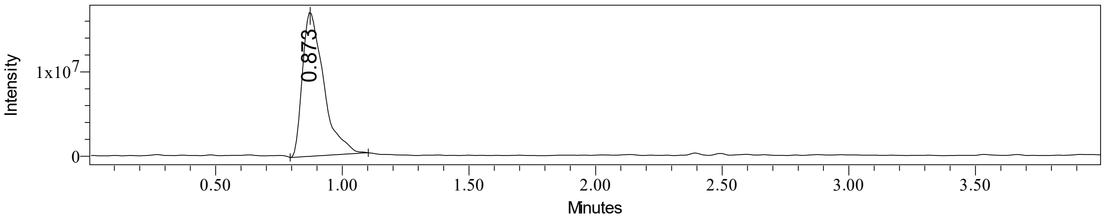

Channel Name MS TIC; Channel QDa 1: MS Scan

Peak Results
Channel: PDA Spectrum

	RT	Base Peak (m/z)	Height	Area	% Area	Channel	Channel Name
1	0.854		1456426	4535954	100.00	PDA Spectrum	220.0nm
2	0.855		2260257	7521391	100.00	PDA Spectrum	254.0nm

Peak Results
Channel: QDa 1: MS Scan

	RT	Base Peak (m/z)	Height	Area	% Area	Channel	Channel Name
1	0.873	305.17	17135526	99288126	100.00	QDa 1: MS Scan	MS TIC

HPLC and LCMS Traces for Compound 8c

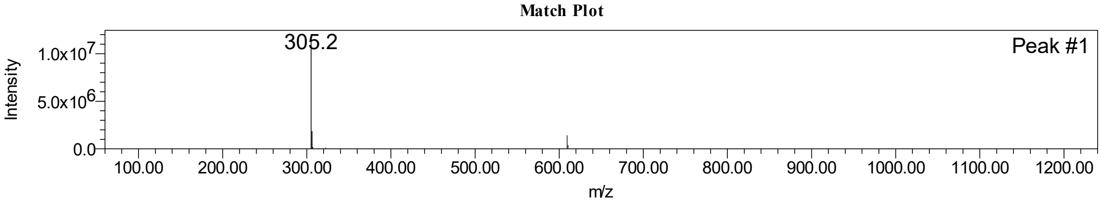

Base Peak 305.17 Channel Description 1: QDa Positive(+) Scan (60.00-1240.00)Da, Centroid, CV=10 - AVG (1.5:4.0;0.0:0.4) x 30.000 Th: 0.010 Retention Time 0.873

HPLC and LCMS Traces for Compound 8c

o2h discovery
Ahmedabad, Gujarat
India

HPLC Analysis Report

Sample name: CIN-C-377-CIN-X-0029-044-a
Location: P2-A5
Injection: 1 of 1
Injection volume: 10.000
Project Name HPLC-10_MAY-2024
Date Acquired: 2024-05-30 12:26:52+05:30
Date Processed: 2024-05-30 13:41:48+05:30

Instrument Name HPLC-10
Acq. method: o2h_HPLC_Method-C.amx
Processing method: *3D UV Quantitative_DefaultMethod.pmx
Column: SUNFIRE C18 150x4.6mm,3.5um

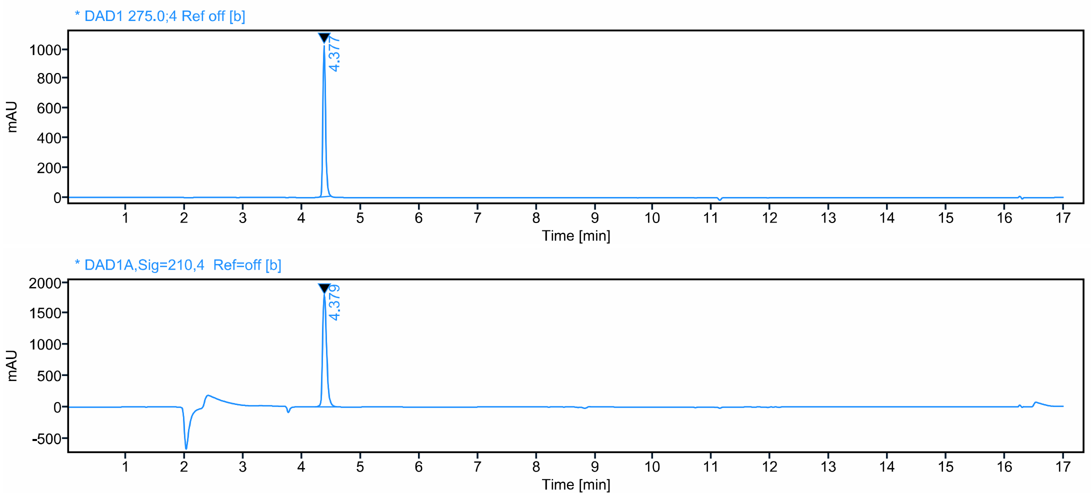

Signal: * DAD1 275.0;4 Ref off [b]

RT [min]	Height	Area	Area%
4.377	1030	3351	100.00

Signal: * DAD1A,Sig=210,4 Ref=off [b]

RT [min]	Height	Area	Area%
4.379	1803	8679	100.00

HPLC and LCMS Traces for Compound 8e

o2h discovery
Ahmedabad, Gujarat
India

LCMS Analysis Report

Sample Name:	CIN2-D-044-CIN2-X-0087-031-b		Injection Id 2005
Sample Type:	Unknown	Acquired By:	LCMS-05
Vial:	2:A,8	Sample Set Name:	01012025_UCH_NEW_RD
Injection #:	1	Acq. Method Set:	o2h_LCMS_Method_A
Injection Volume:	2.00 ul	Processing Method	O2H_LCMS_01, O2H_LCMS_02
Run Time:	4.0 Minutes	Channel Name:	220.0nm, 254.0nm, MS TIC
Project Name:	2025\LCMS-05_JAN-2025	Proc. Chnl. Descr.:	PDA 220.0 nm Blank Subtracted
Date Acquired:	01-01-2025 17:52:41 IST		
Date Processed:	01-01-2025 18:00:37 IST, 01-01-2025 18:00:43 IST, 01-01-2025 18:00:57 IST		
Column:	X-BRIDGE C18 2.1X50mm 2.5um		

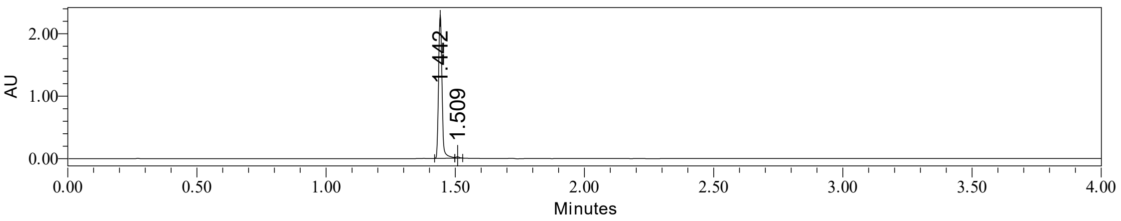

Channel Name 254.0nm; Channel PDA Spectrum

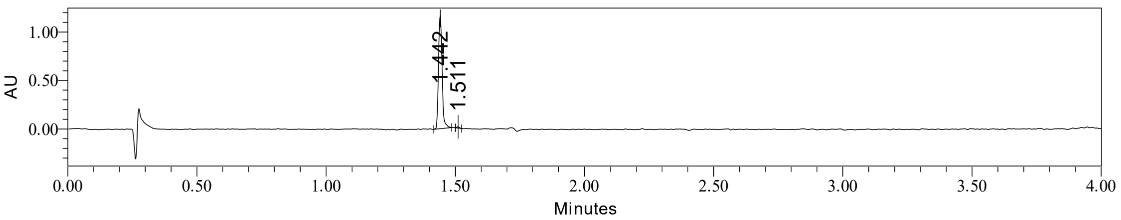

Channel Name 220.0nm; Channel PDA Spectrum

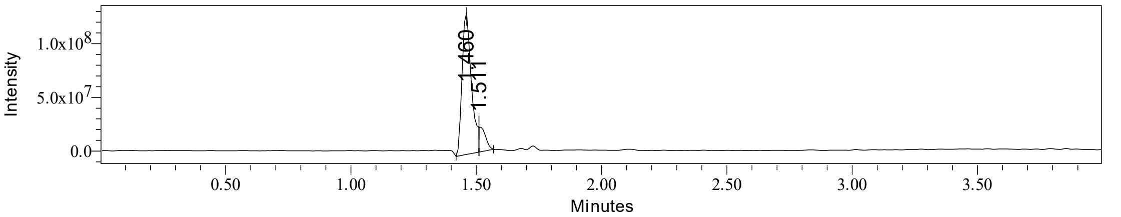

Channel Name MS TIC; Channel QDa 1: MS Scan

Peak Results
Channel: PDA Spectrum

	RT	Base Peak (m/z)	Height	Area	% Area	Channel	Channel Name
1	1.442		1167742	1124875	99.23	PDA Spectrum	220.0nm
2	1.442		2296260	2136222	99.05	PDA Spectrum	254.0nm
3	1.509		22134	20528	0.95	PDA Spectrum	254.0nm
4	1.511		11949	8743	0.77	PDA Spectrum	220.0nm

HPLC and LCMS Traces for Compound 8e

Peak Results
Channel: QDa 1: MS Scan

	RT	Base Peak (m/z)	Height	Area	% Area	Channel	Channel Name
1	1.460	289.03	130109029	338472956	89.26	QDa 1: MS Scan	MS TIC
2	1.511	289.01	23019065	40707019	10.74	QDa 1: MS Scan	MS TIC

Match Plot

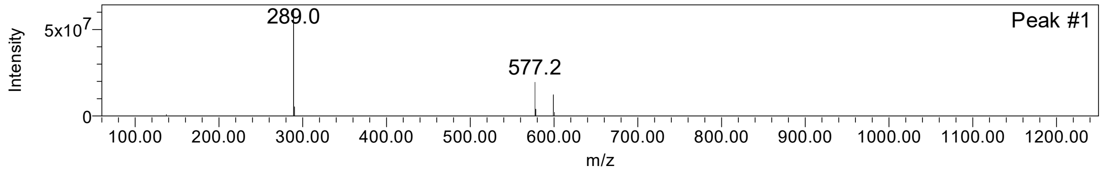

Base Peak 289.03 Channel Description 1: QDa Positive(+) Scan (60.00-1250.00)Da, Centroid, CV=10 - AVG (0.1:1.4;1.7:4.0) x 40.000 Th: 0.010 Retention Time 1.460

Match Plot

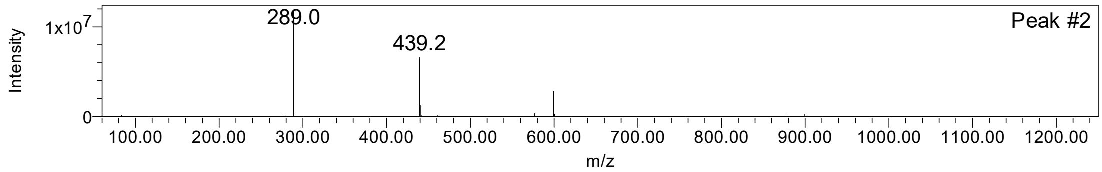

Base Peak 289.01 Channel Description 1: QDa Positive(+) Scan (60.00-1250.00)Da, Centroid, CV=10 - AVG (0.1:1.4;1.7:4.0) x 40.000 Th: 0.010 Retention Time 1.511

HPLC and LCMS Traces for Compound 8e

o2h discovery
Ahmedabad, Gujarat
India

HPLC Analysis Report

Sample name: CIN2-D-044-CIN2-X-0087-031-b

Location: P2-B1

Injection: 1 of 1

Injection volume: 10.000

Project Name HPLC-07_JAN-2025

Date Acquired: 2025-01-01 18:25:07+05:30

Date Processed: 2025-01-01 18:58:37+05:30

Instrument Name HPLC-07

Acq. method: o2h_HPLC_Method-A.amx

Processing method: 3D UV Quantitative_DefaultMethod.pmx

Column: SUNFIRE C18 150x4.6mm 3.5um

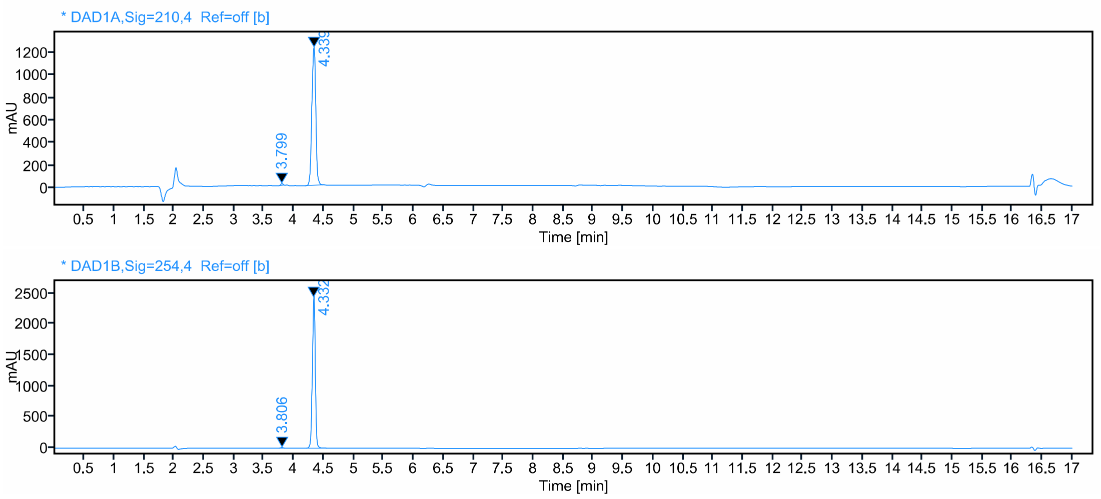

Signal: * DAD1A,Sig=210,4 Ref=off [b]

RT [min]	Height	Area	Area%
3.799	16	39	0.68
4.339	1217	5624	99.32

Signal: * DAD1B,Sig=254,4 Ref=off [b]

RT [min]	Height	Area	Area%
3.806	11	29	0.34
4.332	2426	8409	99.66

HPLC and LCMS Traces for Compound 9a

o2h discovery
Ahmedabad, Gujarat
India

LCMS Analysis Report

Sample Name: CIN-C-376-CIN-X-0049-027-C
Injection Id 8067
Sample Type: Unknown
Acquired By: LCMS-05
Vial: 2:F,4
Sample Set Name: 05062024_UCH_082_FD
Injection #: 1
Acq. Method Set: o2h_LCMS_Method_A_SLOW
Injection Volume: 5.00 ul
Processing Method O2H_LCMS_02,
Run Time: 4.0 Minutes
Channel Name: 220.0nm, MS TIC, 254.0nm
Project Name: 2024\LCMS-05_JUN-2024
Proc. Chnl. Descr.: PDA 254.0 nm Blank Subtracted
Date Acquired: 05-06-2024 21:20:52 IST
Date Processed: 05-06-2024 21:26:25 IST, 05-06-2024 21:26:29 IST, 05-06-2024 21:26:53 IST
Column: X-BRIDGE C18 2.1X50mm 2.5um

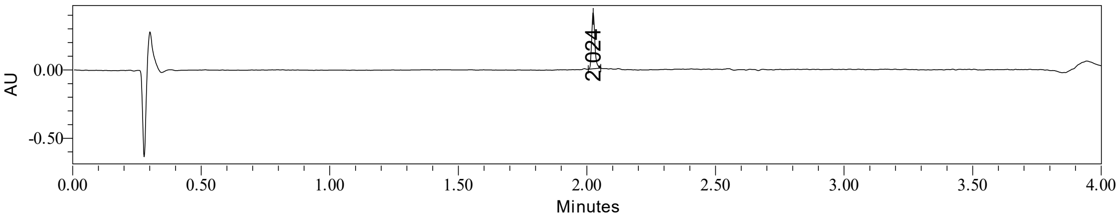

Channel Name 220.0nm; Channel PDA Spectrum

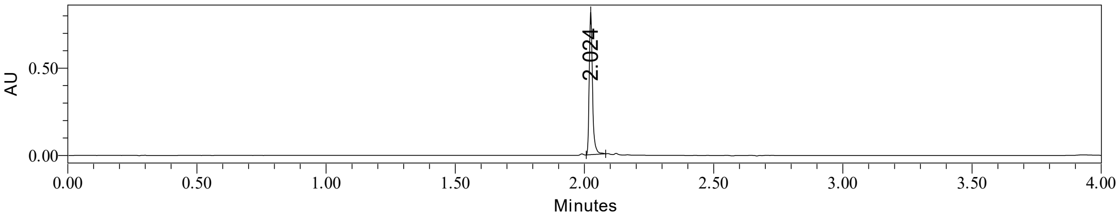

Channel Name 254.0nm; Channel PDA Spectrum

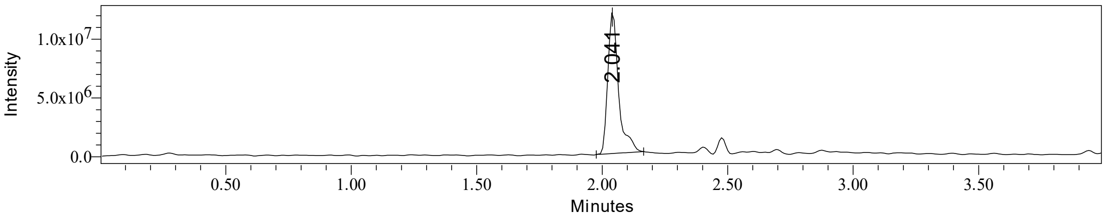

Channel Name MS TIC; Channel QDa 1: MS Scan

Peak Results
Channel: PDA Spectrum

	RT	Base Peak (m/z)	Height	Area	% Area	Channel	Channel Name
1	2.024		412197	346974	100.00	PDA Spectrum	220.0nm
2	2.024		821731	714617	100.00	PDA Spectrum	254.0nm

Peak Results
Channel: QDa 1: MS Scan

	RT	Base Peak (m/z)	Height	Area	% Area	Channel	Channel Name
1	2.041	320.25	11902325	36049834	100.00	QDa 1: MS Scan	MS TIC

O2h Discovery Page: 1 of 2

HPLC and LCMS Traces for Compound 9a

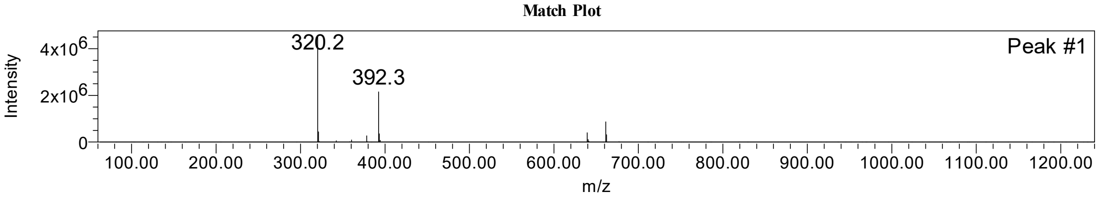

Base Peak 320.25 Channel Description 1: QDa Positive(+) Scan (60.00-1240.00)Da, Centroid, CV=10 - AVG (0.1:1.7;2.2:3.9) x 30.000 Th: 0.010 Retention Time 2.041

HPLC and LCMS Traces for Compound 9a

o2h discovery
Ahmedabad, Gujarat
India

HPLC Analysis Report

**Sample name:**	CIN-C-376-CIN-X-0049-027-C
**Location:**	P2-C1
**Injection:**	1 of 1
**Injection volume:**	50.000
**Project Name**	HPLC-10_JUNE-2024
**Date Acquired:**	2024-06-05 17:21:53+05:30
**Date Processed:**	2024-06-05 21:13:34+05:30
**Instrument Name**	HPLC-10
**Acq. method:**	HP10_FAS2.amx
**Processing method:**	*3D UV Quantitative_DefaultMethod.pmx
**Column:**	SUNFIRE C18 150x4.6mm,3.5um

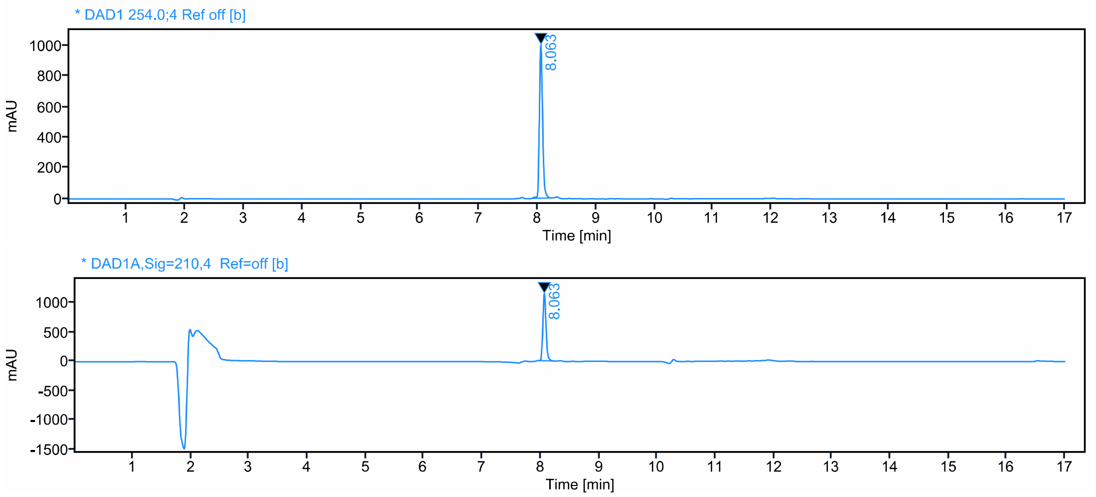

Signal: * DAD1 254.0;4 Ref off [b]

RT [min]	Height	Area	Area%
8.063	1001	3779	100.00

Signal: * DAD1A,Sig=210,4 Ref=off [b]

RT [min]	Height	Area	Area%
8.063	1148	4346	100.00

HPLC and LCMS Traces for Compound 9b

o2h discovery
Ahmedabad, Gujarat
India

LCMS Analysis Report

Sample Name: CIN-C-376-CIN-X-0051-040-B
Injection Id 11262
Sample Type: Unknown
Acquired By: LCMS-05
Vial: 1:F,1
Sample Set Name: 06062024_UCH_082_FD
Injection #: 1
Acq. Method Set: o2h_LCMS_Method_A_SLOW_01
Injection Volume: 6.00 ul
Processing Method O2H_LCMS_02_0,
Run Time: 4.0 Minutes
Channel Name: 254.0nm, MS TIC, 210.0nm
Project Name: 2024\LCMS-05_JUN-2024
Proc. Chnl. Descr.: QDa 1: MS Scan MS TIC,
Date Acquired: 07-06-2024 02:01:43 IST
Date Processed: 07-06-2024 02:16:02 IST, 07-06-2024 02:16:16 IST, 07-06-2024 02:17:57 IST
Column: X-BRIDGE C18 2.1X50mm 2.5um

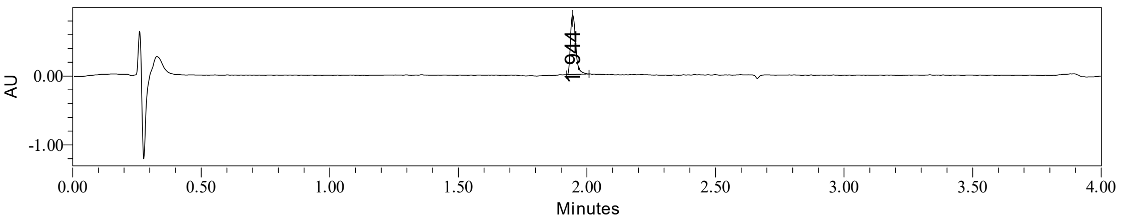

Channel PDA Spectrum; Channel Name 210.0nm

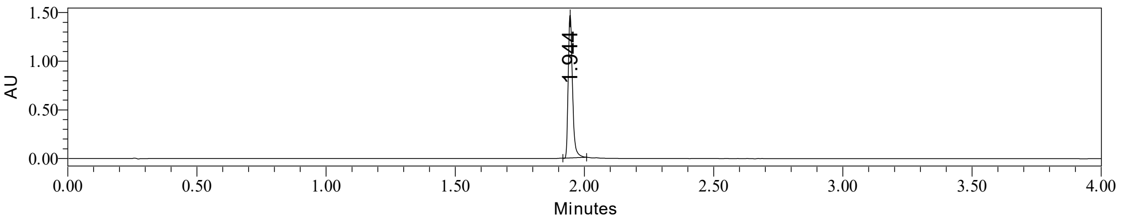

Channel PDA Spectrum; Channel Name 254.0nm

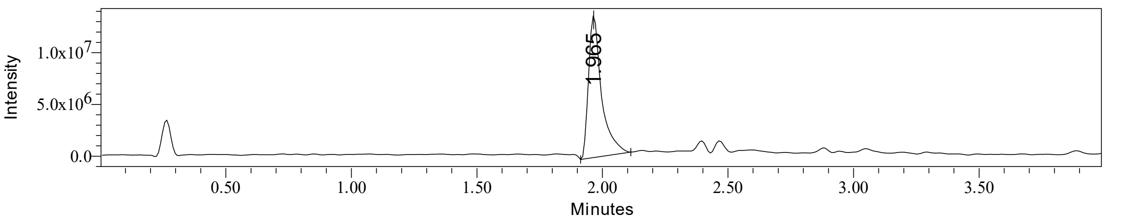

Channel QDa 1: MS Scan; Channel Name MS TIC

Peak Results
Channel: PDA Spectrum

	RT	Base Peak (m/z)	Height	Area	% Area	Channel	Channel Name
1	1.944		1470832	1692661	100.00	PDA Spectrum	254.0nm
2	1.944		865985	1210300	100.00	PDA Spectrum	210.0nm

Peak Results
Channel: QDa 1: MS Scan

	RT	Base Peak (m/z)	Height	Area	% Area	Channel	Channel Name
1	1.965	320.25	13667259	49245184	100.00	QDa 1: MS Scan	MS TIC

HPLC and LCMS Traces for Compound 9b

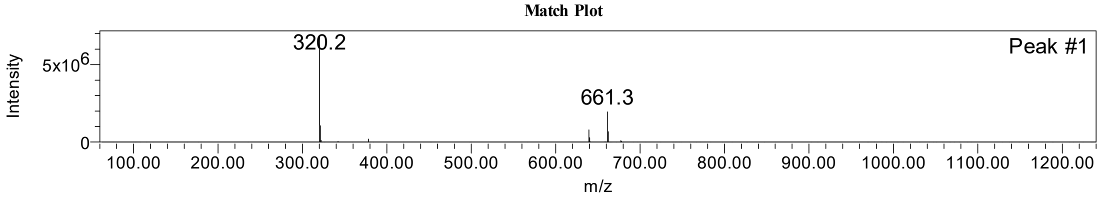

Base Peak 320.25 Channel Description 1: QDa Positive(+) Scan (60.00-1240.00)Da, Centroid, CV=10 - AVG (2.1:4.0;0.1:1.8;0.0:0.4;2.8:3.0) x 20.000 Th: 0.010 Retention Time 1.965

HPLC and LCMS Traces for Compound 9b

o2h discovery<br>Ahmedabad, Gujarat<br>India	**HPLC Analysis Report**

Sample name: CIN-C-376-CIN-X-0051-040-B
Location: P1-A8
Injection: 1 of 1
Injection volume: 30.000
Project Name HPLC-10_JUNE-2024
Date Acquired: 2024-06-06 14:55:56+05:30
Date Processed: 2024-06-06 16:01:06+05:30

Instrument Name HPLC-10
Acq. method: HP10_FAS2.amx
Processing method: *3D UV Quantitative_DefaultMethod.pmx
Column: SUNFIRE C18 150x4.6mm,3.5um

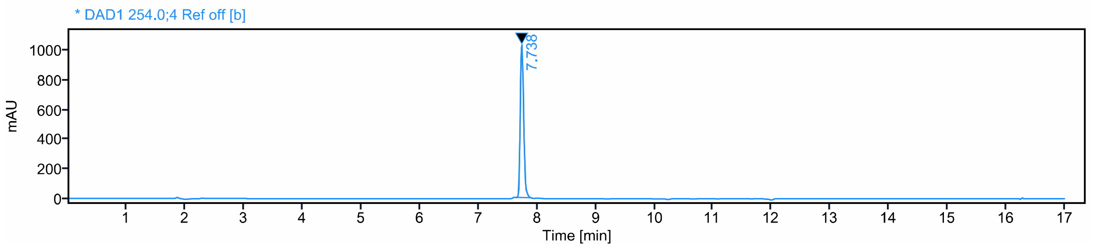

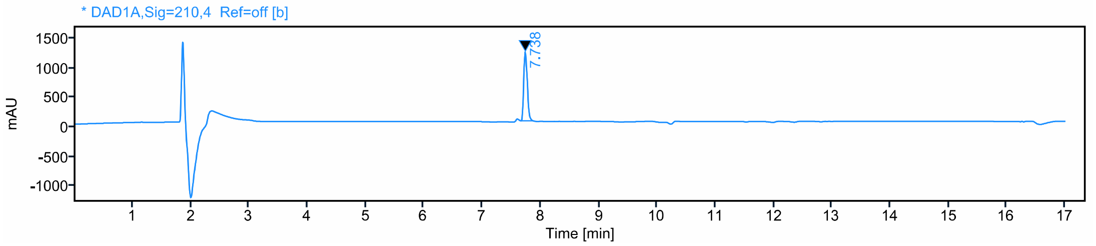

Signal: * DAD1 254.0;4 Ref off [b]

RT [min]	Height	Area	Area%
7.738	1041	4269	100.00

Signal: * DAD1A,Sig=210,4 Ref=off [b]

RT [min]	Height	Area	Area%
7.738	1165	4888	100.00

O2h Discovery Page 1 of 1

HPLC and LCMS Traces for Compound 9c

o2h discovery<br>Ahmedabad, Gujarat<br>India	**LCMS Analysis Report**

Sample Name:	CIN-C-376-CIN-X-0050-0038-C		Injection Id 11268
Sample Type:	Unknown	Acquired By:	LCMS-05
Vial:	1:F,2	Sample Set Name:	06062024_UCH_082_FD
Injection #:	1	Acq. Method Set:	o2h_LCMS_Method_A_SLOW_01
Injection Volume:	3.00 ul	Processing Method	O2H_LCMS_02, MASS_000
Run Time:	4.0 Minutes	Channel Name:	MS TIC, 254.0nm, 210.0nm
Project Name:	2024\LCMS-05_JUN-2024	Proc. Chnl. Descr.:	QDa 1: MS Scan MS TIC,
Date Acquired:	07-06-2024 02:06:25 IST		
Date Processed:	07-06-2024 02:19:31 IST, 07-06-2024 02:19:49 IST, 07-06-2024 02:20:30 IST		
Column:	X-BRIDGE C18 2.1X50mm 2.5um		

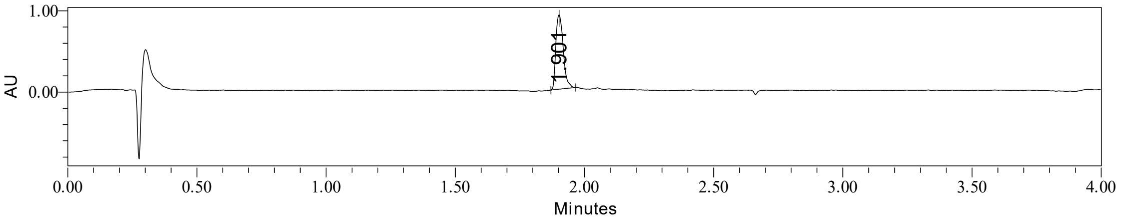

Channel PDA Spectrum; Channel Name 210.0nm

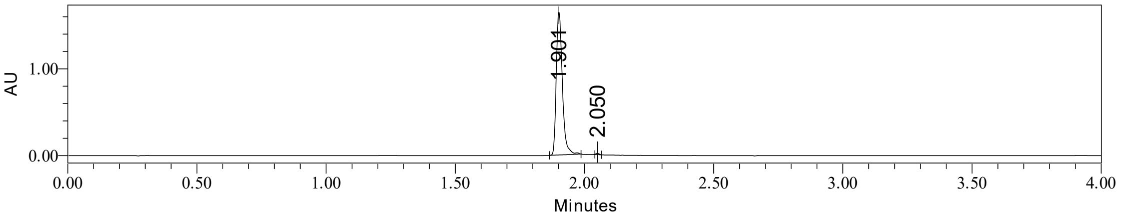

Channel PDA Spectrum; Channel Name 254.0nm

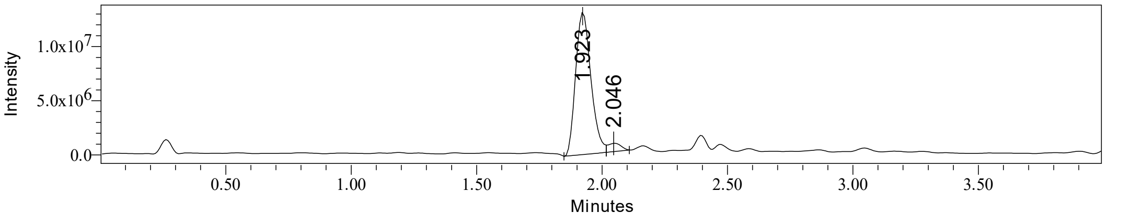

Channel QDa 1: MS Scan; Channel Name MS TIC

Peak Results
Channel: PDA Spectrum

	RT	Base Peak (m/z)	Height	Area	% Area	Channel	Channel Name
1	1.901		1645492	2677953	99.55	PDA Spectrum	254.0nm
2	1.901		913478	1800355	100.00	PDA Spectrum	210.0nm
3	2.050		15574	12135	0.45	PDA Spectrum	254.0nm

HPLC and LCMS Traces for Compound 9c

Peak Results
Channel: QDa 1: MS Scan

	RT	Base Peak (m/z)	Height	Area	% Area	Channel	Channel Name
1	1.923	320.25	13125696	55456221	95.22	QDa 1: MS Scan	MS TIC
2	2.046	661.23	766971	2780825	4.78	QDa 1: MS Scan	MS TIC

Match Plot

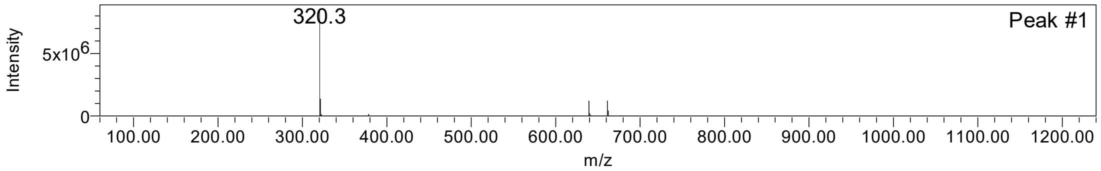

Base Peak 320.25 Channel Description 1: QDa Positive(+) Scan (60.00-1240.00)Da, Centroid, CV=10 - AVG (0.1:1.8;2.0:4.0;2.7:3.0) x 20.000 Th: 0.010 Retention Time 1.923

Match Plot

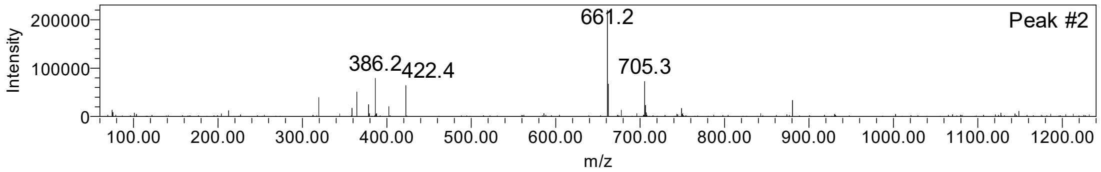

Base Peak 661.23 Channel Description 1: QDa Positive(+) Scan (60.00-1240.00)Da, Centroid, CV=10 - AVG (0.1:1.8;2.0:4.0;2.7:3.0) x 20.000 Th: 0.010 Retention Time 2.046

HPLC and LCMS Traces for Compound 9c

o2h discovery
Ahmedabad, Gujarat
India

HPLC Analysis Report

Sample name: CIN-C-376-CIN-X-0050-0038-C
Location: P1-A9
Injection: 1 of 1
Injection volume: 10.000
Project Name HPLC-10_JUNE-2024
Date Acquired: 2024-06-06 15:15:02+05:30
Date Processed: 2024-06-06 15:55:19+05:30

Instrument Name HPLC-10
Acq. method: HP10_FAS2.amx
Processing method: *3D UV Quantitative_DefaultMethod.pmx
Column: SUNFIRE C18 150x4.6mm,3.5um

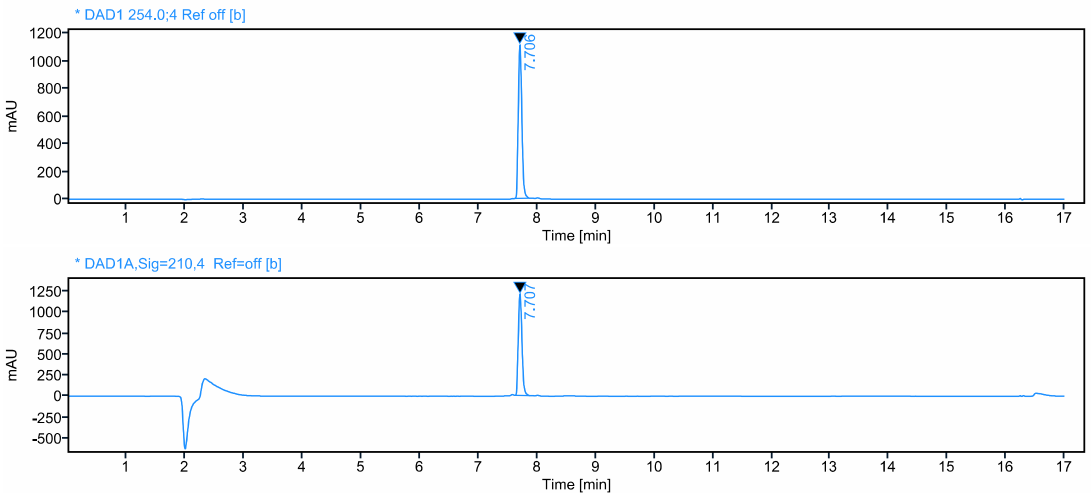

Signal: * DAD1 254.0;4 Ref off [b]

RT [min]	Height	Area	Area%
7.706	1116	4600	100.00

Signal: * DAD1A,Sig=210,4 Ref=off [b]

RT [min]	Height	Area	Area%
7.707	1221	5201	100.00

HPLC and LCMS Traces for Compound 10a

o2h discovery
Ahmedabad, Gujarat
India

LCMS Analysis Report

Sample Name: CIN-C-376-CIN-X-0055-036-C
Injection Id 9434
Sample Type: Unknown
Acquired By: LCMS-05
Vial: 2:C,6
Sample Set Name: 06062024_UCH_082_FD
Injection #: 1
Acq. Method Set: o2h_LCMS_Method_A_SLOW
Injection Volume: 2.00 ul
Processing Method O2H_LCMS_09_0,
Run Time: 4.0 Minutes
Channel Name: MS TIC, 254.0nm, 210.0nm
Project Name: 2024\LCMS-05_JUN-2024
Proc. Chnl. Descr.: PDA 254.0 nm Blank Subtracted
Date Acquired: 06-06-2024 13:34:51 IST
Date Processed: 06-06-2024 14:08:11 IST, 06-06-2024 14:08:18 IST, 06-06-2024 14:09:03 IST
Column: X-BRIDGE C18 2.1X50mm 2.5um

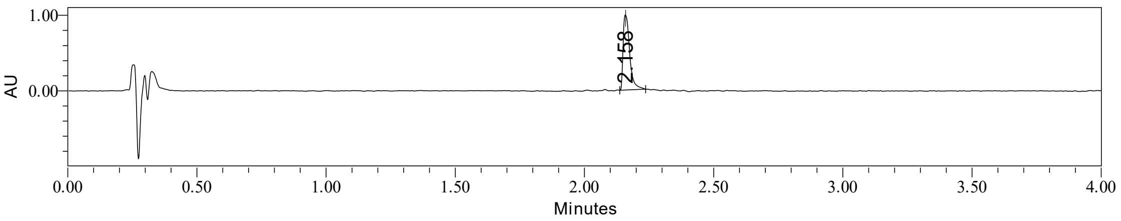

Channel Name 210.0nm; Channel PDA Spectrum

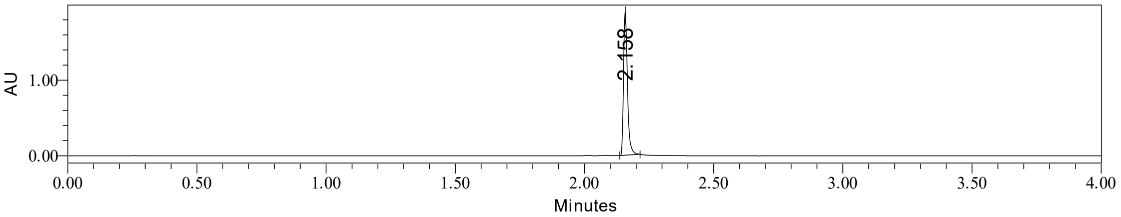

Channel Name 254.0nm; Channel PDA Spectrum

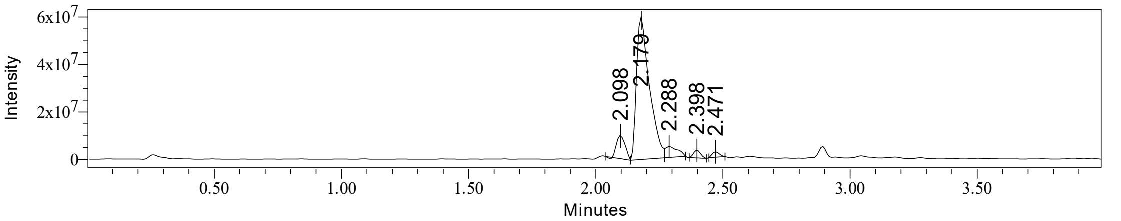

Channel Name MS TIC; Channel QDa 1: MS Scan

Peak Results
Channel: PDA Spectrum

	RT	Base Peak (m/z)	Height	Area	% Area	Channel	Channel Name
1	2.158		1895871	2005025	100.00	PDA Spectrum	254.0nm
2	2.158		996211	1752104	100.00	PDA Spectrum	210.0nm

Peak Results
Channel: QDa 1: MS Scan

	RT	Base Peak (m/z)	Height	Area	% Area	Channel	Channel Name
1	2.098	348.31	9724753	23954992	9.14	QDa 1: MS Scan	MS TIC

HPLC and LCMS Traces for Compound 10a

Peak Results
Channel: QDa 1: MS Scan

	RT	Base Peak (m/z)	Height	Area	% Area	Channel	Channel Name
2	2.179	334.27	59800090	21196879C	80.90	QDa 1: MS Scan	MS TIC
3	2.288	334.29	4618147	15482605	5.91	QDa 1: MS Scan	MS TIC
4	2.398	183.15	3256902	6206256	2.37	QDa 1: MS Scan	MS TIC
5	2.471	320.29	2285437	4415185	1.69	QDa 1: MS Scan	MS TIC

Match Plot

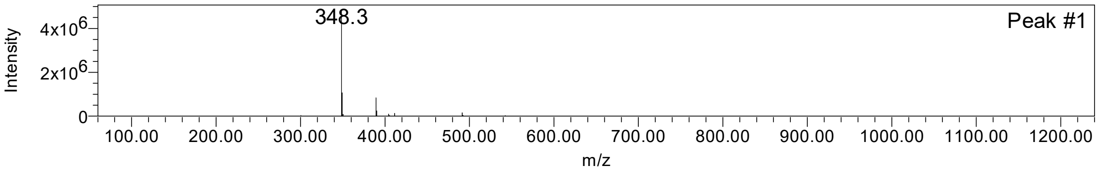

Base Peak 348.31 Channel Description 1: QDa Positive(+) Scan (60.00-1240.00)Da, Centroid, CV=10 - AVG (2.3:4.0;0.4:2.1) x 10.000 Th: 0.010 Retention Time 2.098

Match Plot

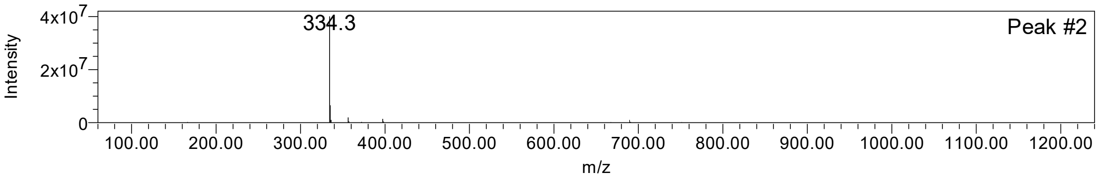

Base Peak 334.27 Channel Description 1: QDa Positive(+) Scan (60.00-1240.00)Da, Centroid, CV=10 - AVG (2.3:4.0;0.4:2.1) x 10.000 Th: 0.010 Retention Time 2.179

Match Plot

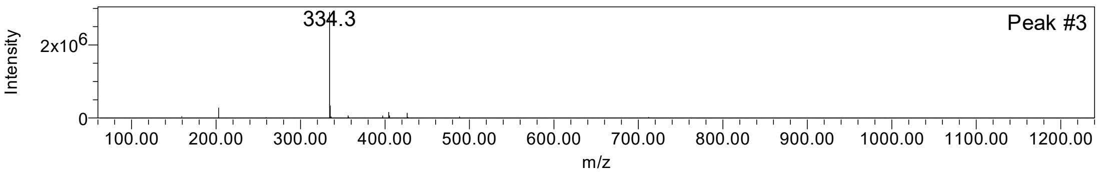

Base Peak 334.29 Channel Description 1: QDa Positive(+) Scan (60.00-1240.00)Da, Centroid, CV=10 - AVG (2.3:4.0;0.4:2.1) x 10.000 Th: 0.010 Retention Time 2.288

Match Plot

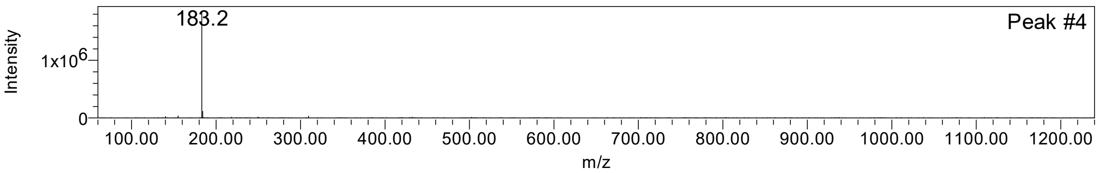

Base Peak 183.15 Channel Description 1: QDa Positive(+) Scan (60.00-1240.00)Da, Centroid, CV=10 - AVG (2.3:4.0;0.4:2.1) x 10.000 Th: 0.010 Retention Time 2.398

Match Plot

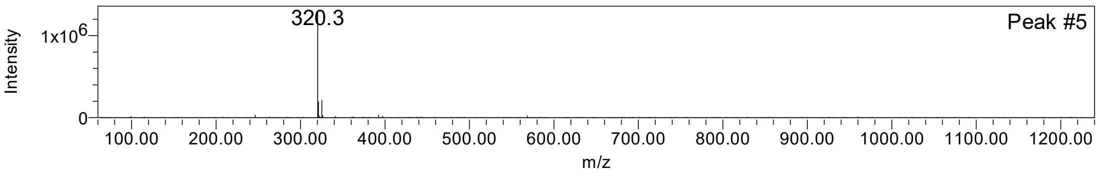

Base Peak 320.29 Channel Description 1: QDa Positive(+) Scan (60.00-1240.00)Da, Centroid, CV=10 - AVG (2.3:4.0;0.4:2.1) x 10.000 Th: 0.010 Retention Time 2.471

HPLC and LCMS Traces for Compound 10a

o2h discovery<br>Ahmedabad, Gujarat<br>India	**HPLC Analysis Report**

**Sample name:**	CIN-C-376-CIN-X-0055-036-C		
**Location:**	P1-A6	**Instrument Name**	HPLC-10
**Injection:**	1 of 1	**Acq. method:**	HP10_FAS2.amx
**Injection volume:**	10.000	**Processing method:**	*3D UV Quantitative_DefaultMethod.pmx
**Project Name**	HPLC-10_JUNE-2024		
**Date Acquired:**	2024-06-06 14:17:54+05:30	**Column:**	SUNFIRE C18 150x4.6mm,3.5um
**Date Processed:**	2024-06-06 14:37:48+05:30		

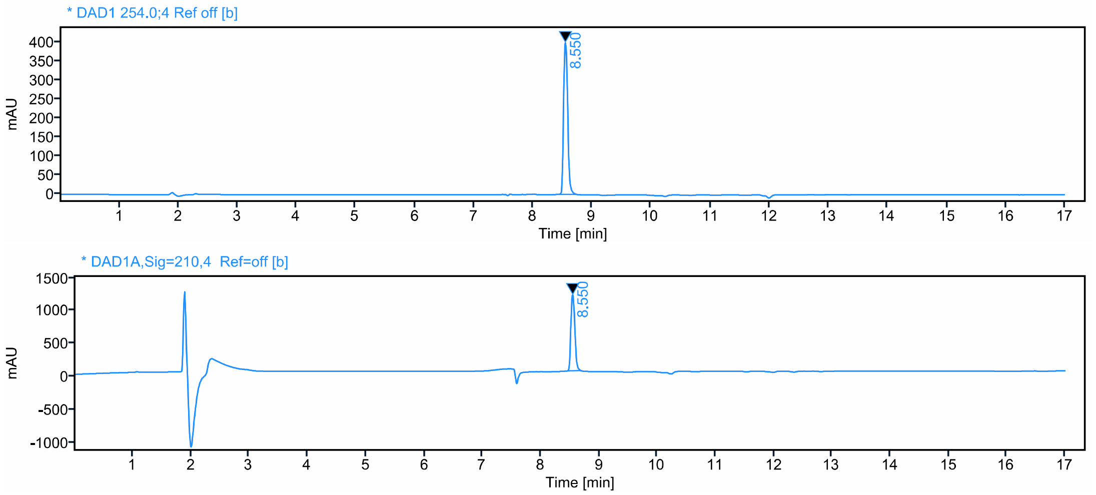

Signal: * DAD1 254.0;4 Ref off [b]

RT [min]	Height	Area	Area%
8.550	398	1875	100.00

Signal: * DAD1A,Sig=210,4 Ref=off [b]

RT [min]	Height	Area	Area%
8.550	1150	5416	100.00

HPLC and LCMS Traces for Compound 10b

o2h discovery
Ahmedabad, Gujarat
India

LCMS Analysis Report

Sample Name:	CIN-C-376-CIN-X-0057-063-A		Injection Id 19947
Sample Type:	Unknown	Acquired By:	LCMS-05
Vial:	2:F,2	Sample Set Name:	08072024_UCH_082_RD_
Injection #:	1	Acq. Method Set:	o2h_LCMS_Method_A
Injection Volume:	2.00 ul	Processing Method	O2H_LCMS_02_0, MASS_000
Run Time:	4.0 Minutes	Channel Name:	MS TIC, 254.0nm, 210.0nm
Project Name:	2024\LCMS-05_JUL-2024	Proc. Chnl. Descr.:	QDa 1: MS Scan MS TIC,
Date Acquired:	08-07-2024 11:47:45 IST		
Date Processed:	08-07-2024 11:58:04 IST, 08-07-2024 11:58:12 IST, 08-07-2024 11:58:33 IST		
Column:	X-BRIDGE C18 2.1X50mm 2.5um		

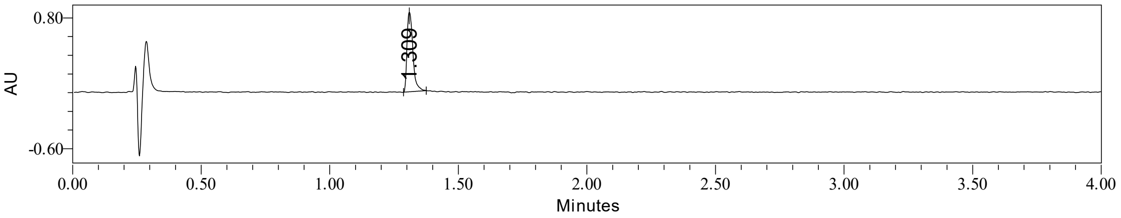

Channel Name 210.0nm; Channel PDA Spectrum

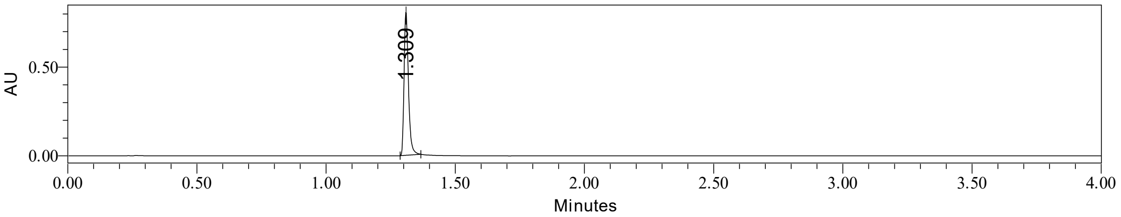

Channel Name 254.0nm; Channel PDA Spectrum

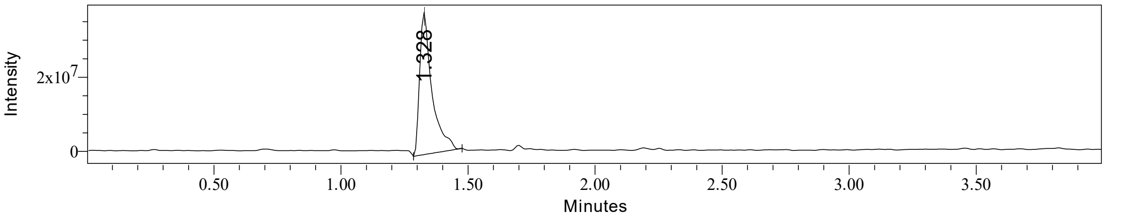

Channel Name MS TIC; Channel QDa 1: MS Scan

Peak Results
Channel: PDA Spectrum

	RT	Base Peak (m/z)	Height	Area	% Area	Channel	Channel Name
1	1.309		808885	951267	100.00	PDA Spectrum	254.0nm
2	1.309		852337	1351514	100.00	PDA Spectrum	210.0nm

Peak Results
Channel: QDa 1: MS Scan

	RT	Base Peak (m/z)	Height	Area	% Area	Channel	Channel Name
1	1.328	334.23	37988553	129088102	100.00	QDa 1: MS Scan	MS TIC

HPLC and LCMS Traces for Compound 10b

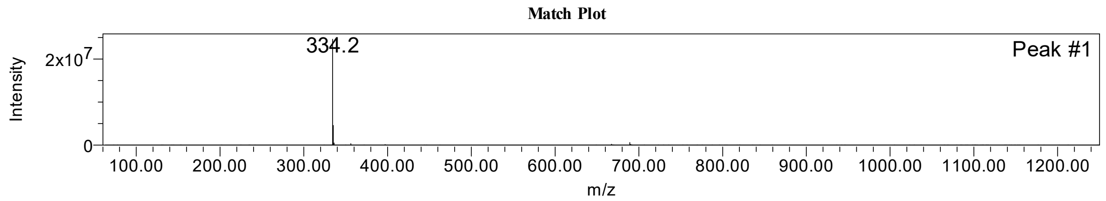

Base Peak 334.23 Channel Description 1: QDa Positive(+) Scan (60.00-1250.00)Da, Centroid, CV=10 - AVG (0.1:1.1;1.5:3.9) x 20.000 Th: 0.010 Retention Time 1.328

HPLC and LCMS Traces for Compound 10b

o2h discovery
Ahmedabad, Gujarat
India

HPLC Analysis Report

Sample name: CIN-C-376-CIN-X-0057-063-A
Location: P2-A5
Injection: 1 of 1
Injection volume: 10.000
Project Name HPLC-07_JUL-2024
Date Acquired: 2024-07-08 13:41:36+05:30
Date Processed: 2024-07-08 14:05:33+05:30

Instrument Name HPLC-07
Acq. method: o2h_HPLC_Method-D.amx
Processing method: 3D UV Quantitative_DefaultMethod.pmx
Column: XBridge C18 150x4.6mm, 3.5um

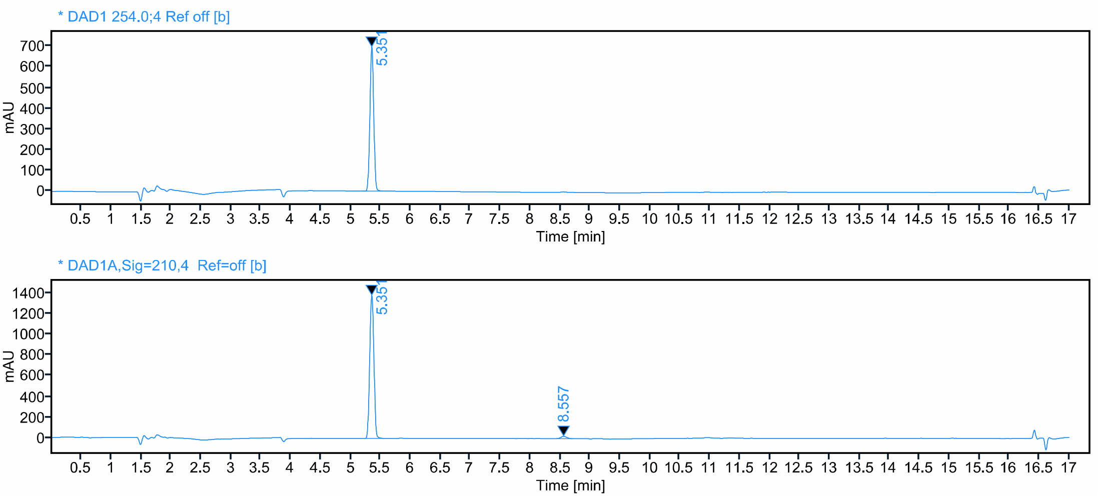

Signal: * DAD1 254.0;4 Ref off [b]

RT [min]	Height	Area	Area%
5.351	694	3086	100.00

Signal: * DAD1A,Sig=210,4 Ref=off [b]

RT [min]	Height	Area	Area%
5.351	1387	6794	97.80
8.557	23	153	2.20

HPLC and LCMS Traces for Compound 10c

o2h discovery
Ahmedabad, Gujarat
India

LCMS Analysis Report

Sample name: CIN-C-376-CIN-X-0056-062-A
Location: P2-C7
Injection: 1 of 1
Injection volume: 10.000

Instrument Name LCMS-09
Acq. method: o2h_LCMS_Method_J.amx
Processing method: *LC_MS Sample Purity_DefaultMethod_NEW.pmx

Project Name LCMS-09_JULY-2024
Date Acquired: 2024-07-05 08:41:29+05:30
Date Processed: 2024-07-05 16:37:37+05:30

Description:
Column: XBridge C18 150x4.6mm,3.5um

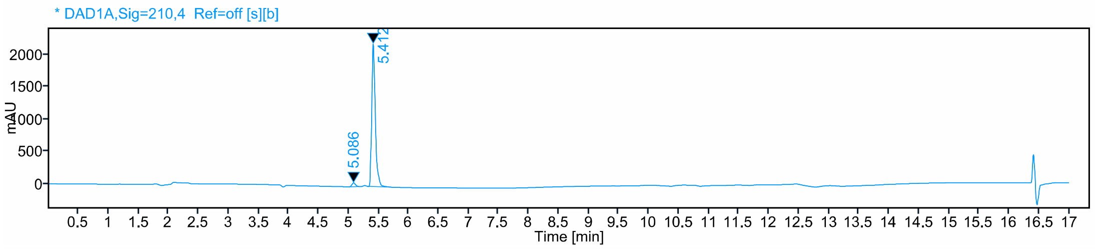

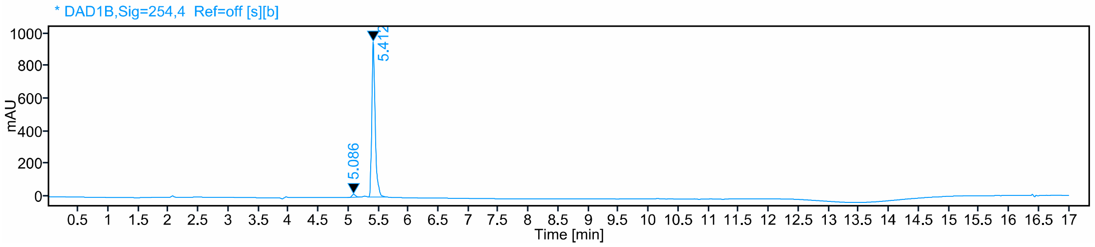

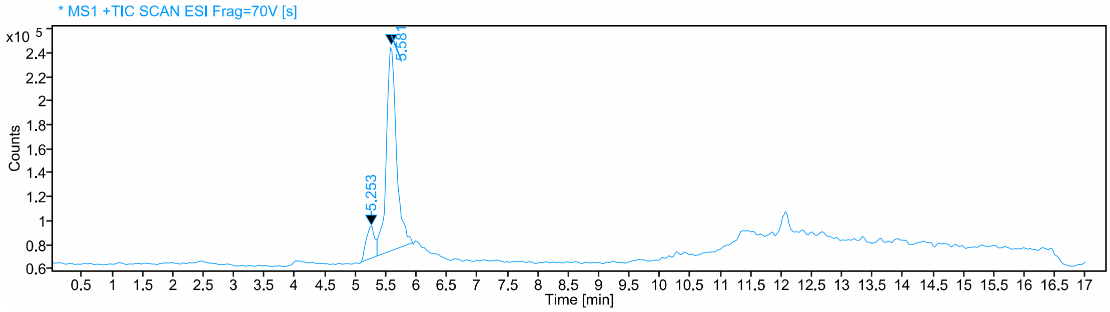

Signal: * DAD1A,Sig=210,4 Ref=off [s][b]

RT [min]	Base Peak (m/z)	Height	Area	Area%
5.086		60	232	2.46
5.412		2216	9187	97.54

Printed: 2024-07-05 16:38:20+05:30 **Page 1 of 2**

HPLC and LCMS Traces for Compound 10c

o2h discovery
Ahmedabad, Gujarat
India

LCMS Analysis Report

Signal: * DAD1B,Sig=254,4 Ref=off [s][b]

RT [min]	Base Peak (m/z)	Height	Area	Area%
5.086		20	79	2.05
5.412		958	3759	97.95

Signal: * MS1 +TIC SCAN ESI Frag=70V [s]

RT [min]	Base Peak (m/z)	Height	Area	Area%
5.253	331.300	27144	265396	11.79
5.581	334.300	171518	1986546	88.21

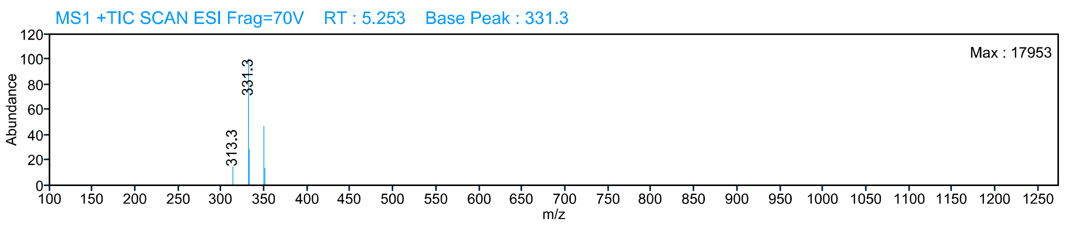

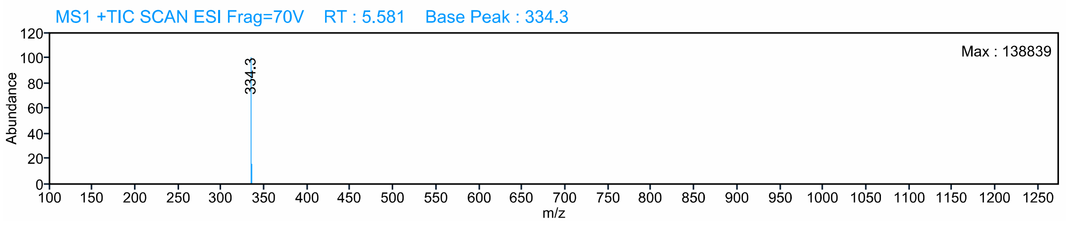

Printed: 2024-07-05 16:38:20+05:30 **Page 2 of 2**

HPLC and LCMS Traces for Compound 10c

o2h discovery
Ahmedabad, Gujarat
India

HPLC Analysis Report

Sample name: CIN-C-376-CIN-X-0056-062-A
Location: P2-A6
Injection: 1 of 1
Injection volume: 10.000
Project Name HPLC-07_JUL-2024
Date Acquired: 2024-07-08 15:16:15+05:30
Date Processed: 2024-07-08 15:36:19+05:30

Instrument Name HPLC-07
Acq. method: o2h_HPLC_Method-D.amx
Processing method: 3D UV Quantitative_DefaultMethod.pmx
Column: XBridge C18 150x4.6mm, 3.5um

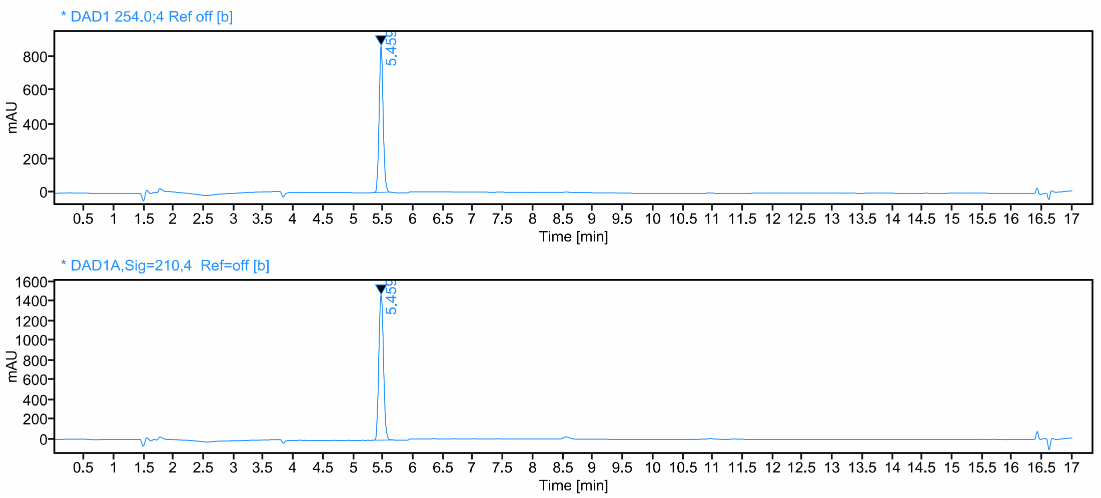

Signal: * DAD1 254.0;4 Ref off [b]

RT [min]	Height	Area	Area%
5.459	856	4028	100.00

Signal: * DAD1A,Sig=210,4 Ref=off [b]

RT [min]	Height	Area	Area%
5.459	1474	8084	100.00

HPLC and LCMS Traces for Compound 11a

o2h discovery
Ahmedabad, Gujarat
India

LCMS Analysis Report

Sample Name:	CIN-C-376-CIN-X-0058-047-A		Injection Id 31116
Sample Type:	Unknown	Acquired By:	LCMS-05
Vial:	1:B,3	Sample Set Name:	15062024_UCH_083_RD_1
Injection #:	1	Acq. Method Set:	o2h_LCMS_Method_A_SLOW
Injection Volume:	2.00 ul	Processing Method	O2H_LCMS_02,
Run Time:	4.0 Minutes	Channel Name:	220.0nm, MS TIC, 254.0nm
Project Name:	2024\LCMS-05_JUN-2024	Proc. Chnl. Descr.:	PDA 254.0 nm Blank Subtracted
Date Acquired:	15-06-2024 11:56:10 IST		
Date Processed:	15-06-2024 12:07:02 IST, 15-06-2024 12:07:10 IST, 15-06-2024 12:07:42 IST		
Column:	X-BRIDGE C18 2.1X50mm 2.5um		

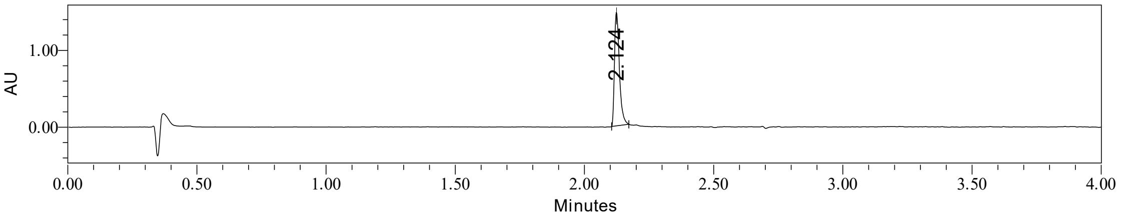

Channel Name 220.0nm; Channel PDA Spectrum

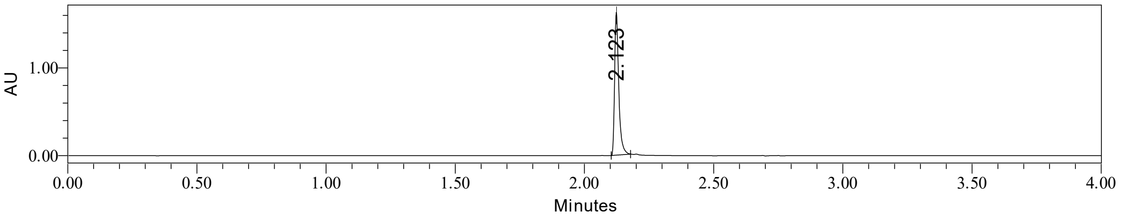

Channel Name 254.0nm; Channel PDA Spectrum

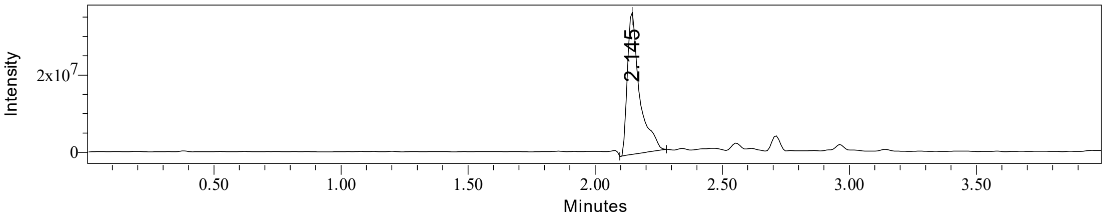

Channel Name MS TIC; Channel QDa 1: MS Scan

Peak Results
Channel: PDA Spectrum

	RT	Base Peak (m/z)	Height	Area	% Area	Channel	Channel Name
1	2.123		1627152	1805550	100.00	PDA Spectrum	254.0nm
2	2.124		1477901	1947618	100.00	PDA Spectrum	220.0nm

Peak Results
Channel: QDa 1: MS Scan

	RT	Base Peak (m/z)	Height	Area	% Area	Channel	Channel Name
1	2.145	333.29	36521635	122273481	100.00	QDa 1: MS Scan	MS TIC

HPLC and LCMS Traces for Compound 11a

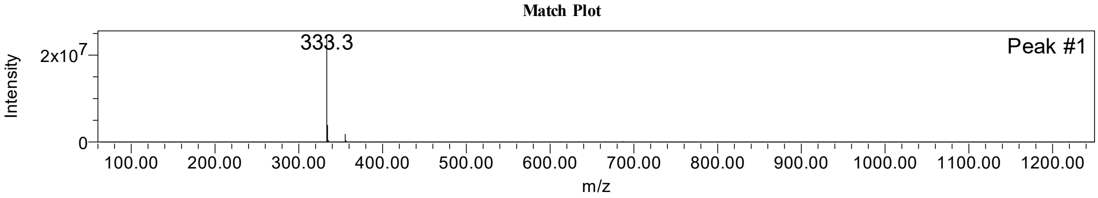

Base Peak 333.29 Channel Description 1: QDa Positive(+) Scan (60.00-1240.00)Da, Centroid, CV=10 - AVG (0.1:1.8;2.4:3.9) x 30.000 Th: 0.010 Retention Time 2.145

HPLC and LCMS Traces for Compound 11a

**o2h discovery**<br>**Ahmedabad, Gujarat**<br>**India**	**HPLC Analysis Report**

Sample name: CIN-C-376-CIN-X-0058-047-A
Location: P1-A2
Injection: 1 of 1
Injection volume: 10.000
Project Name HPLC-10_JUNE-2024
Date Acquired: 2024-06-15 12:56:36+05:30
Date Processed: 2024-06-15 14:05:54+05:30

Instrument Name HPLC-10
Acq. method: HP10_FAS2.amx
Processing method: *3D UV Quantitative_DefaultMethod.pmx
Column: SUNFIRE C18 150x4.6mm,3.5um

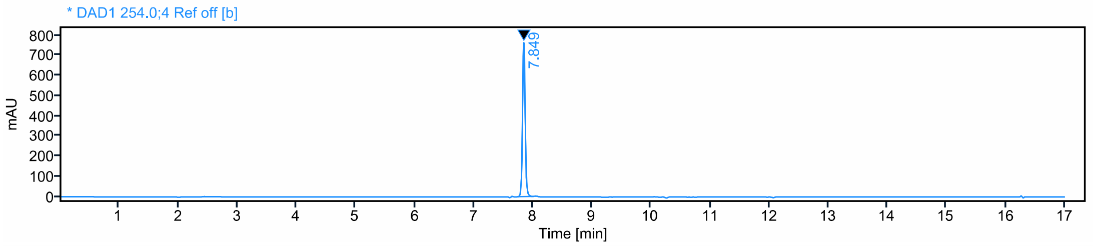

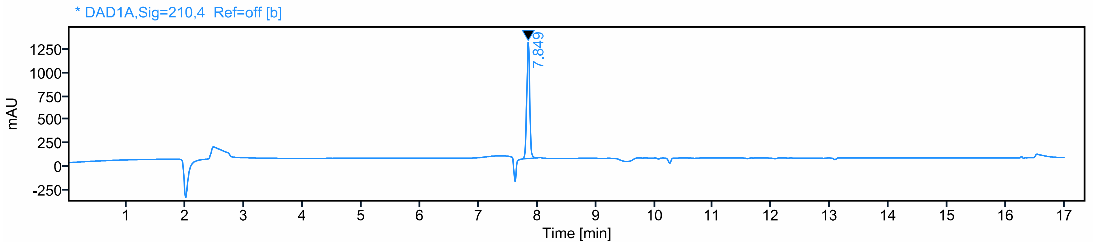

Signal: * DAD1 254.0;4 Ref off [b]

RT [min]	Height	Area	Area%
7.849	771	2522	100.00

Signal: * DAD1A,Sig=210,4 Ref=off [b]

RT [min]	Height	Area	Area%
7.849	1243	4216	100.00

HPLC and LCMS Traces for Compound 12a

o2h discovery
Ahmedabad, Gujarat
India

LCMS Analysis Report

Sample Name:	CIN-C-376-CIN-X-0061-059-A		Injection Id 58253
Sample Type:	Unknown	Acquired By:	LCMS-05
Vial:	1:F,4	Sample Set Name:	28062024_UCH_082_RD
Injection #:	1	Acq. Method Set:	o2h_LCMS_Method_A
Injection Volume:	2.00 ul	Processing Method	O2H_LCMS_02_000, MASS_01
Run Time:	4.0 Minutes	Channel Name:	220.0nm, 254.0nm, MS TIC
Project Name:	2024\LCMS-05_JUN-2024	Proc. Chnl. Descr.:	PDA 220.0 nm Blank Subtracted
Date Acquired:	28-06-2024 19:18:24 IST		
Date Processed:	28-06-2024 19:42:40 IST, 28-06-2024 19:42:48 IST, 28-06-2024 19:43:01 IST		
Column:	X-BRIDGE C18 2.1X50mm 2.5um		

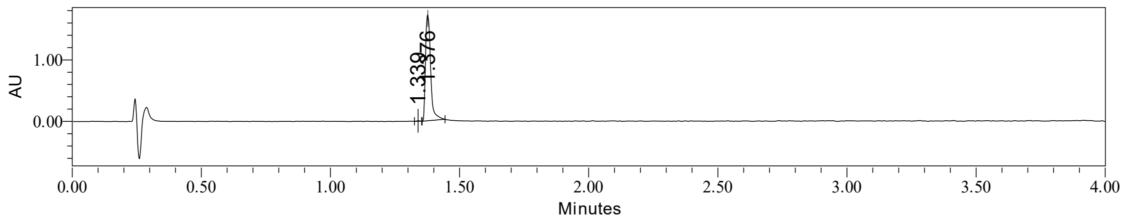

Channel Name 220.0nm; Channel PDA Spectrum

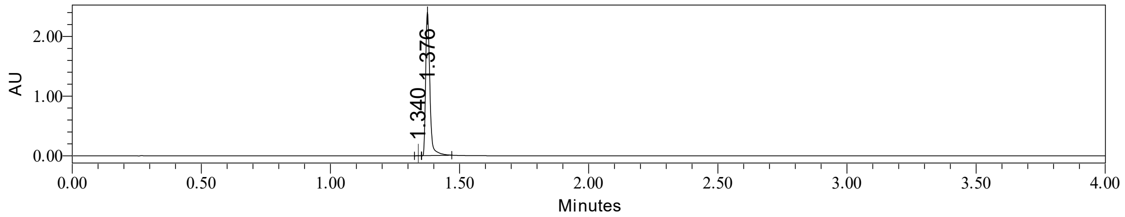

Channel Name 254.0nm; Channel PDA Spectrum

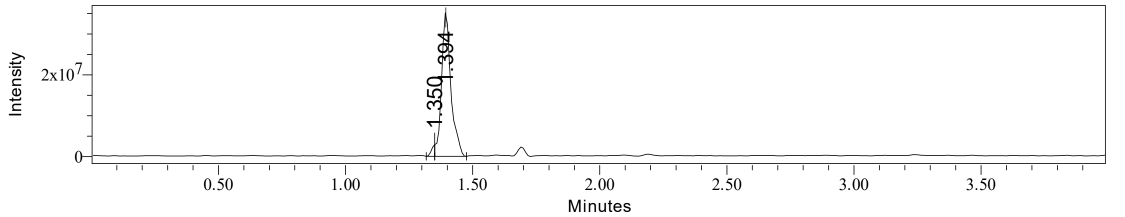

Channel Name MS TIC; Channel QDa 1: MS Scan

Peak Results
Channel: PDA Spectrum

	RT	Base Peak (m/z)	Height	Area	% Area	Channel	Channel Name
1	1.339		8141	6689	0.28	PDA Spectrum	220.0nm
2	1.340		2582	2167	0.08	PDA Spectrum	254.0nm
3	1.376		2405578	2720806	99.92	PDA Spectrum	254.0nm
4	1.376		1718302	2371722	99.72	PDA Spectrum	220.0nm

HPLC and LCMS Traces for Compound 12a

Peak Results
Channel: QDa 1: MS Scan

	RT	Base Peak (m/z)	Height	Area	% Area	Channel	Channel Name
1	1.350	303.37	2884847	2336656	2.52	QDa 1: MS Scan	MS TIC
2	1.394	347.32	34601476	90320228	97.48	QDa 1: MS Scan	MS TIC

Match Plot

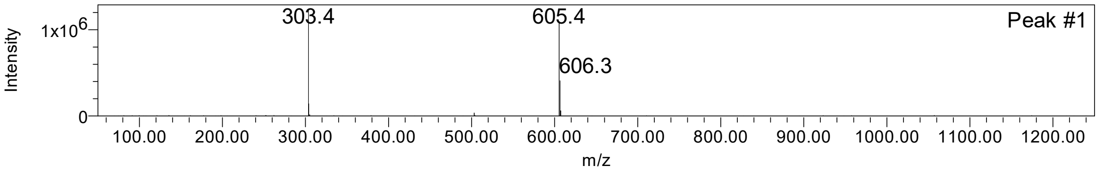

Base Peak 303.37 Channel Description 1: QDa Positive(+) Scan (60.00-1250.00)Da, Centroid, CV=10 - AVG (0.2:1.2;1.6:4.0) x 30.000 Th: 0.010 Retention Time 1.350

Match Plot

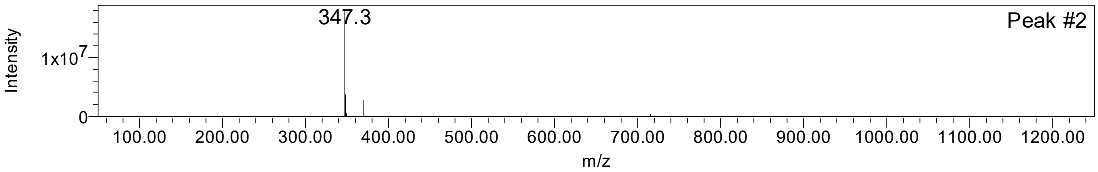

Base Peak 347.32 Channel Description 1: QDa Positive(+) Scan (60.00-1250.00)Da, Centroid, CV=10 - AVG (0.2:1.2;1.6:4.0) x 30.000 Th: 0.010 Retention Time 1.394

HPLC and LCMS Traces for Compound 12a

o2h discovery
Ahmedabad, Gujarat
India

HPLC Analysis Report

Sample name: CIN-C-376-CIN-X-0061-059-A
Location: P1-B7
Injection: 1 of 1
Injection volume: 10.000
Project Name HPLC-10_JUNE-2024
Date Acquired: 2024-06-28 22:28:32+05:30
Date Processed: 2024-06-29 00:01:04+05:30

Instrument Name HPLC-10
Acq. method: o2h_HPLC_Method-C.amx
Processing method: *3D UV Quantitative_DefaultMethod.pmx
Column: SUNFIRE C18 150x4.6mm,3.5um

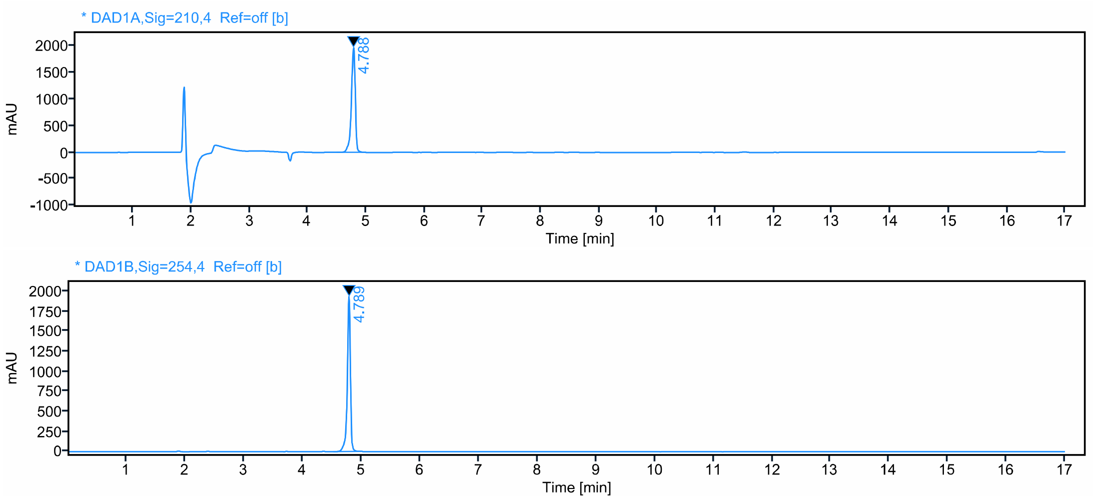

Signal: * DAD1A,Sig=210,4 Ref=off [b]

RT [min]	Height	Area	Area%
4.788	1966	9110	100.00

Signal: * DAD1B,Sig=254,4 Ref=off [b]

RT [min]	Height	Area	Area%
4.789	1935	7370	100.00

HPLC and LCMS Traces for Compound 13a

o2h discovery
Ahmedabad, Gujarat
India

LCMS Analysis Report

Sample Name:	CIN2-D-044-CIN2-X-0076-009-d		Injection Id 20160
Sample Type:	Unknown	Acquired By:	LCMS-05
Vial:	1:F,3	Sample Set Name:	09122024_UCH_083_RD
Injection #:	1	Acq. Method Set:	O2H_LCMS_DEV_96
Injection Volume:	4.00 ul	Processing Method	O2H_LCMS_01, O2H_LCMS_02,
Run Time:	9.0 Minutes	Channel Name:	220.0nm, 254.0nm@1, MS TIC
Project Name:	2024\LCMS-05_DEC-2024_	Proc. Chnl. Descr.:	PDA 254.0 nm Blank Subtracted
Date Acquired:	10-12-2024 02:14:25 IST		
Date Processed:	10-12-2024 02:34:42 IST, 10-12-2024 02:34:55 IST, 10-12-2024 02:35:33 IST, 10-12-2024		
Column:	X-BRIDGE C18 2.1X50mm 2.5um		

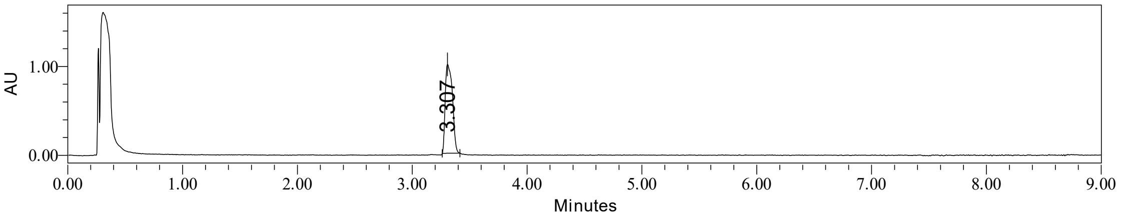

Channel Name 220.0nm; Channel PDA Spectrum

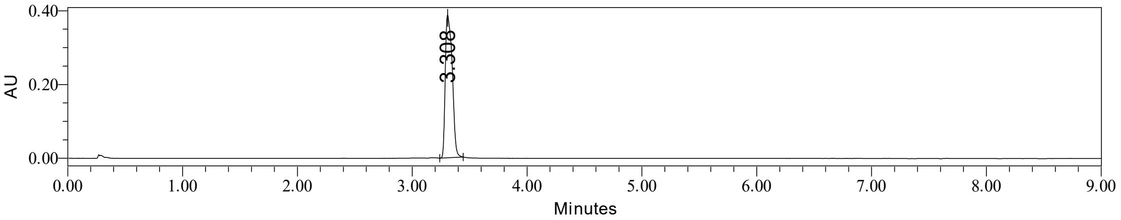

Channel Name 254.0nm@1; Channel PDA Spectrum

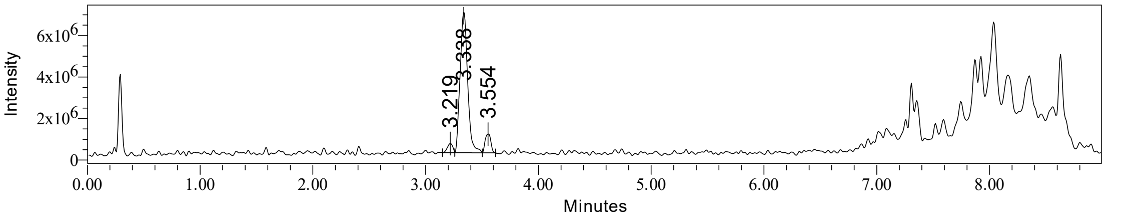

Channel Name MS TIC; Channel QDa 1: MS Scan

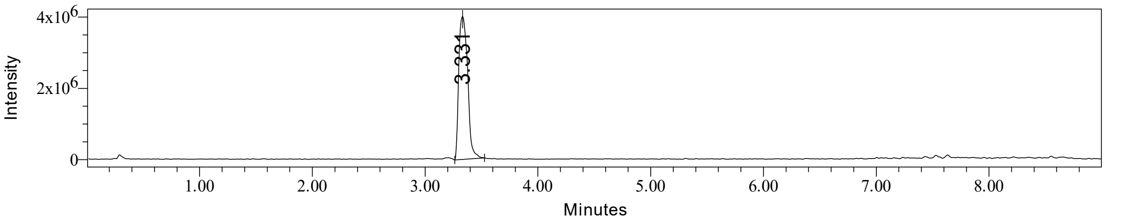

Channel Name MS TIC; Channel QDa 3: MS Scan

HPLC and LCMS Traces for Compound 13a

Peak Results
Channel: PDA Spectrum

	RT	Base Peak (m/z)	Height	Area	% Area	Channel	Channel Name
1	3.307		1001183	4345157	100.00	PDA Spectrum	220.0nm
2	3.308		387799	1626360	100.00	PDA Spectrum	254.0nm@1

Peak Results
Channel: QDa 1: MS Scan

	RT	Base Peak (m/z)	Height	Area	% Area	Channel	Channel Name
1	3.219	460.87	440888	1454211	4.01	QDa 1: MS Scan	MS TIC
2	3.338	358.94	6790607	31623703	87.28	QDa 1: MS Scan	MS TIC
3	3.554	308.96	912270	3155857	8.71	QDa 1: MS Scan	MS TIC

Peak Results
Channel: QDa 3: MS Scan

	RT	Base Peak (m/z)	Height	Area	% Area	Channel	Channel Name
1	3.331	357.03	4016148	21902911	100.00	QDa 3: MS Scan	MS TIC

Match Plot

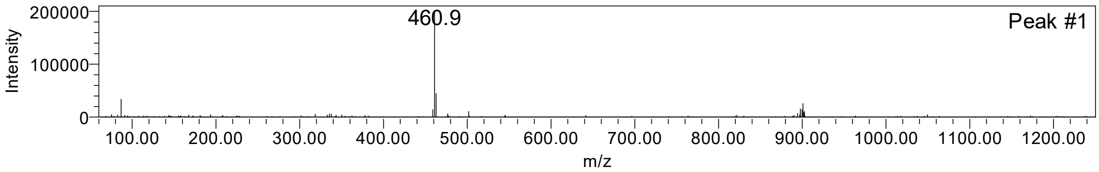

Base Peak 460.87 Channel Description 1: QDa Positive(+) Scan (50.00-1250.00)Da, Centroid, CV=10 - AVG (0.1:2.5;4.6:8.8) x 30.000 Th: 0.010 Retention Time 3.219

Match Plot

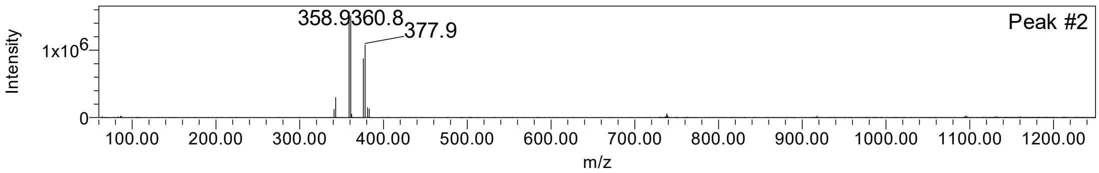

Base Peak 358.94 Channel Description 1: QDa Positive(+) Scan (50.00-1250.00)Da, Centroid, CV=10 - AVG (0.1:2.5;4.6:8.8) x 30.000 Th: 0.010 Retention Time 3.338

Match Plot

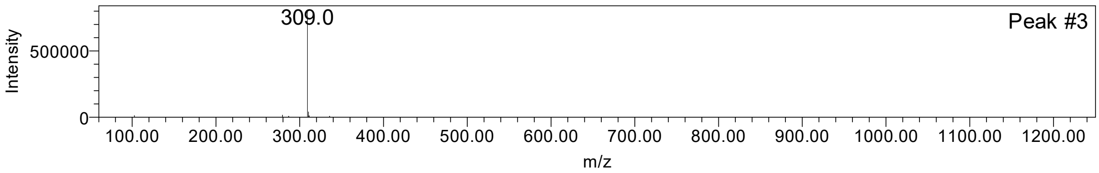

Base Peak 308.96 Channel Description 1: QDa Positive(+) Scan (50.00-1250.00)Da, Centroid, CV=10 - AVG (0.1:2.5;4.6:8.8) x 30.000 Th: 0.010 Retention Time 3.554

HPLC and LCMS Traces for Compound 13a

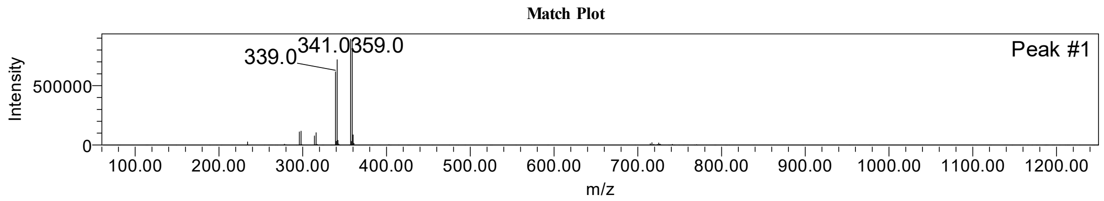

Base Peak 357.03 Channel Description 3: QDa Negative(-) Scan (50.00-1250.00)Da, Centroid, CV=30 - AVG (0.2:3.0;3.7:8.4) x 30.000 Th: 0.010 Retention Time 3.331

HPLC and LCMS Traces for Compound 13a

o2h discovery
Ahmedabad, Gujarat
India

HPLC Analysis Report

**Sample name:**	CIN2-D-044-CIN2-X-0076-009-d		
**Location:**	P2-C10	**Instrument Name**	HPLC-07
**Injection:**	1 of 1	**Acq. method:**	o2h_HPLC_Method-A.amx
**Injection volume:**	15.000	**Processing method:**	3D UV Quantitative_DefaultMethod.pmx
**Project Name**	HPLC-07_DEC-2024		
**Date Acquired:**	2024-12-09 23:41:49+05:30	**Column:**	SUNFIRE C18 150x4.6mm 3.5um
**Date Processed:**	2024-12-10 00:12:19+05:30		

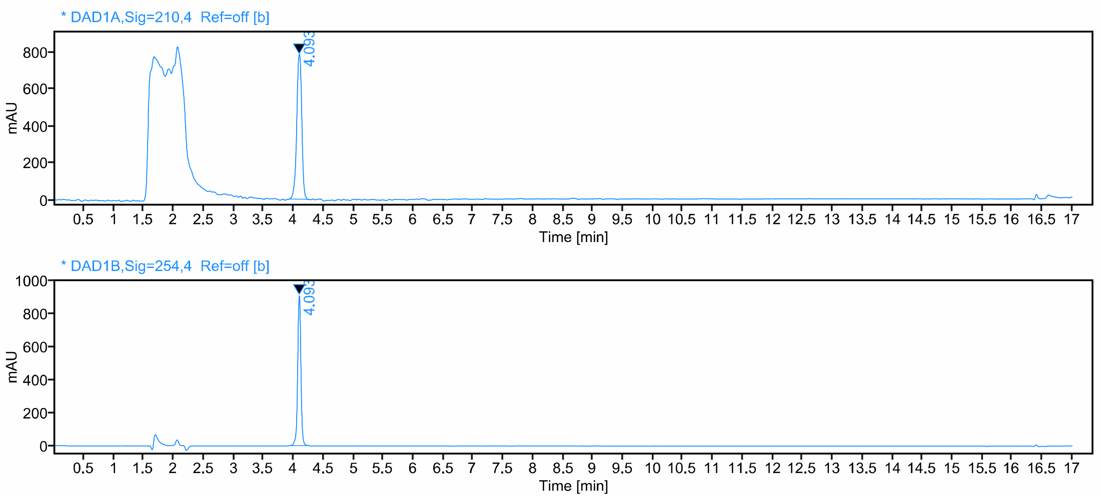

Signal: * DAD1A,Sig=210,4 Ref=off [b]

RT [min]	Height	Area	Area%
4.093	784	4538	100.00

Signal: * DAD1B,Sig=254,4 Ref=off [b]

RT [min]	Height	Area	Area%
4.093	915	3321	100.00

HPLC and LCMS Traces for Compound 14a

Method1

SAMPLE INFORMATION

Sample Name:	CNA-108	Acquired By:	BillyGroup
Sample Type:	Unknown	Sample Set Name:	NA_28112025
Vial:	1:F,4	Acq. Method Set:	HPLC_NA
Injection #:	1	Processing Method:	gj
Injection Volume:	3.00 ul	Channel Name:	220.0nm
Run Time:	25.0 Minutes	Proc. Chnl. Descr.:	2998 PDA 220.0 nm (2998
Date Acquired:	28/11/2025 3:49:51 PM HKT		
Date Processed:	4/12/2025 11:16:26 AM HKT		

Auto-Scaled Chromatogram

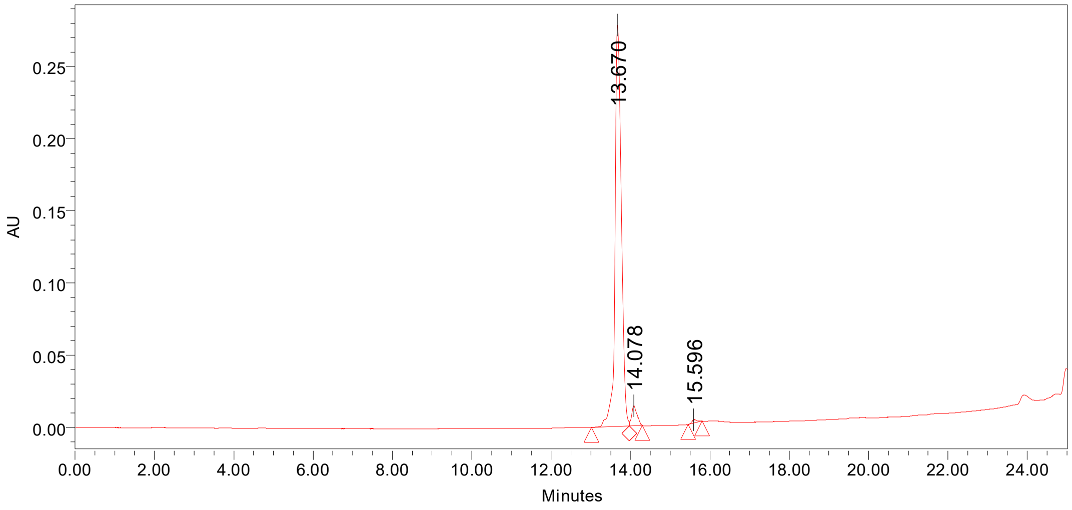

Peak Results

	Retention Time (min)	% Area	Sample Set Name	Channel Name	% Height
1	13.670	95.25	NA_28112025	220.0nm	94.45
2	14.078	4.12	NA_28112025	220.0nm	4.77
3	15.596	0.63	NA_28112025	220.0nm	0.78

Reported by User: BillyGroup (BillyGroup)
Report Method: Method1
Report Method ID: 2308
Page: 1 of 1

Project Name: Nucleoside Analogues
Date Printed:
4/12/2025
11:16:56 am Asia/Hong_Kong

HPLC and LCMS Traces for Compound 14a

Method1

SAMPLE INFORMATION

Sample Name:	CNA-108	Acquired By:	BillyGroup
Sample Type:	Unknown	Sample Set Name:	NA_28112025
Vial:	1:F,4	Acq. Method Set:	HPLC_NA
Injection #:	1	Processing Method:	Huso
Injection Volume:	3.00 ul	Channel Name:	280.0nm@2
Run Time:	25.0 Minutes	Proc. Chnl. Descr.:	2998 PDA 280.0 nm (2998
Date Acquired:	28/11/2025 3:49:51 PM HKT		
Date Processed:	4/12/2025 11:19:26 AM HKT		

Auto-Scaled Chromatogram

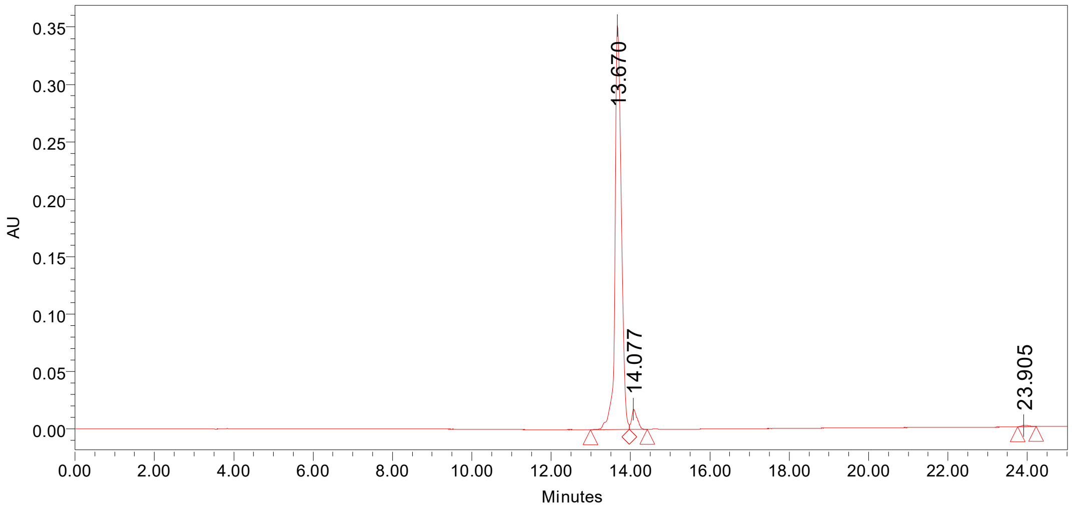

Peak Results

	Retention Time (min)	% Area	Sample Set Name	Channel Name	% Height
1	13.670	95.36	NA_28112025	280.0nm@2	94.94
2	14.077	4.22	NA_28112025	280.0nm@2	4.74
3	23.905	0.41	NA_28112025	280.0nm@2	0.31

Reported by User: BillyGroup (BillyGroup)
Report Method: Method1
Report Method ID: 2308
Page: 1 of 1

Project Name: Nucleoside Analogues
Date Printed:
4/12/2025
11:19:34 am Asia/Hong_Kong

HPLC and LCMS Traces for Compound 15a

o2h discovery
Ahmedabad, Gujarat
India

LCMS Analysis Report

Sample Name:	CIN2-D-044-CIN-X-0099-011-c		Injection Id 17795
Sample Type:	Unknown	Acquired By:	LCMS-05
Vial:	1:C,3	Sample Set Name:	09122024_UCH_083_RD
Injection #:	1	Acq. Method Set:	o2h_LCMS_Method_A
Injection Volume:	2.00 ul	Processing Method	O2H_LCMS_01, O2H_LCMS_02
Run Time:	4.0 Minutes	Channel Name:	310.0nm, MS TIC, 210.0nm
Project Name:	2024\LCMS-05_DEC-2024_	Proc. Chnl. Descr.:	PDA 310.0 nm Blank Subtracted
Date Acquired:	09-12-2024 11:05:33 IST		
Date Processed:	09-12-2024 11:16:43 IST, 09-12-2024 11:16:53 IST, 09-12-2024 11:17:24 IST		
Column:	X-BRIDGE C18 2.1X50mm 2.5um		

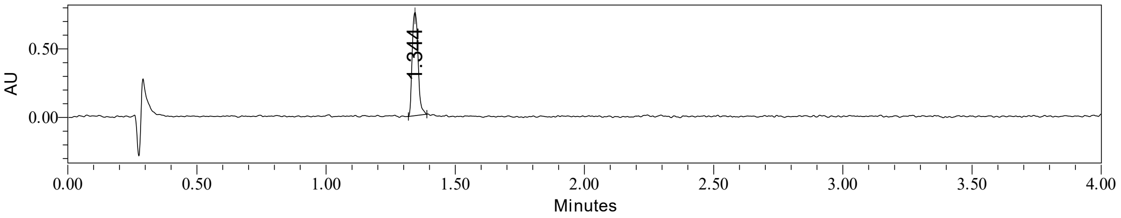

Channel Name 210.0nm; Channel PDA Spectrum

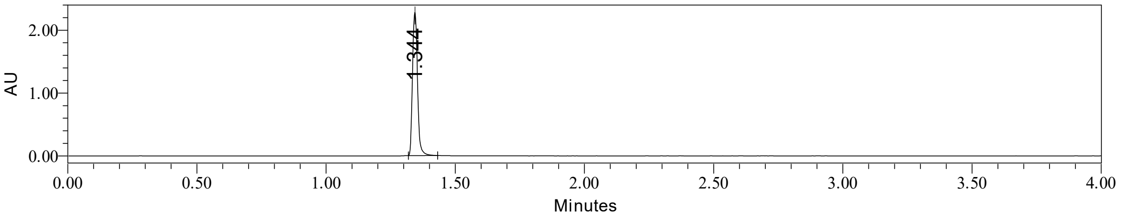

Channel Name 310.0nm; Channel PDA Spectrum

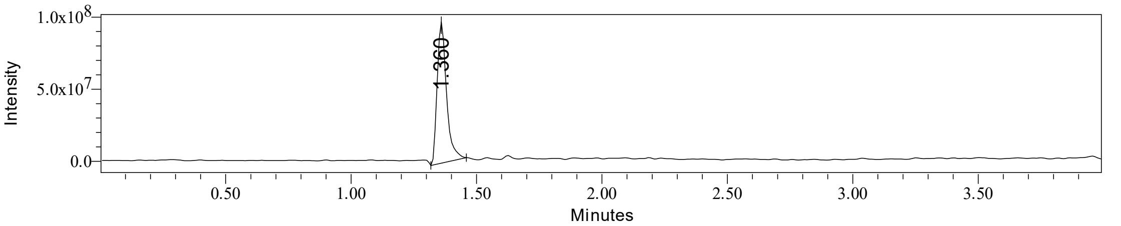

Channel Name MS TIC; Channel QDa 1: MS Scan

Peak Results
Channel: PDA Spectrum

	RT	Base Peak (m/z)	Height	Area	% Area	Channel	Channel Name
1	1.344		2281061	3073545	100.00	PDA Spectrum	310.0nm
2	1.344		755268	119068C	100.00	PDA Spectrum	210.0nm

Peak Results
Channel: QDa 1: MS Scan

	RT	Base Peak (m/z)	Height	Area	% Area	Channel	Channel Name
1	1.360	320.98	96684810	253342324	100.00	QDa 1: MS Scan	MS TIC

HPLC and LCMS Traces for Compound 15a

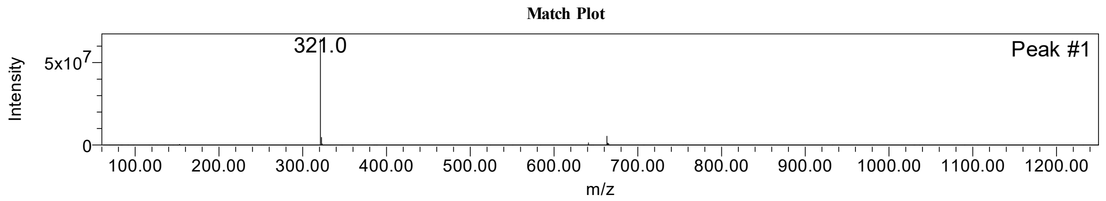

Base Peak 320.98 Channel Description 1: QDa Positive(+) Scan (60.00-1250.00)Da, Centroid, CV=10 - AVG (1.6:3.8;0.3:0.5) x 20.000 Th: 0.010 Retention Time 1.360

HPLC and LCMS Traces for Compound 15a

o2h discovery
Ahmedabad, Gujarat
India

HPLC Analysis Report

**Sample name:**	CIN2-D-044-CIN-X-0099-011-c		
**Location:**	P2-B2	**Instrument Name**	HPLC-10
**Injection:**	1 of 1	**Acq. method:**	o2h_HPLC_Method-D.amx
**Injection volume:**	40.000	**Processing method:**	*3D UV Quantitative_DefaultMethod.pmx
**Project Name**	HPLC-10_DEC-2024	**Column:**	XBridge C18 150x4.6mm, 3.5um
**Date Acquired:**	2024-12-10 18:21:10+05:30		
**Date Processed:**	2024-12-10 18:56:05+05:30		

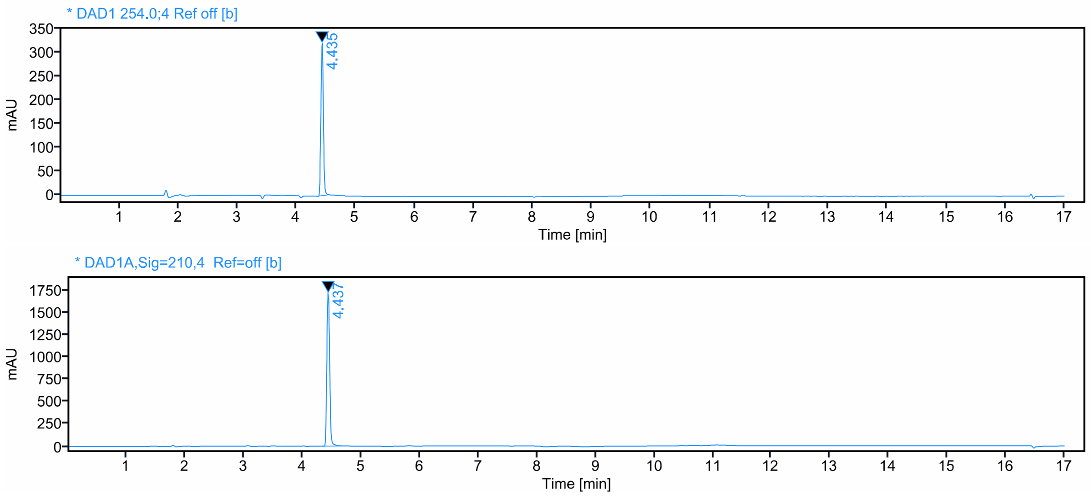

Signal: * DAD1 254.0;4 Ref off [b]

RT [min]	Height	Area	Area%
4.435	320	908	100.00

Signal: * DAD1A,Sig=210,4 Ref=off [b]

RT [min]	Height	Area	Area%
4.437	1723	5504	100.00

Printed: 2024-12-10 18:56:17+05:30 **Page 1 of 1**

HPLC and LCMS Traces for Compound 15b

o2h discovery
Ahmedabad, Gujarat
India

LCMS Analysis Report

Sample Name:	CIN2-C-816-CIN2-X-0101-088-c		Injection Id 23256
Sample Type:	Unknown	Acquired By:	LCMS-05
Vial:	1:B,5	Sample Set Name:	11122024_UCH_NEW_FD
Injection #:	1	Acq. Method Set:	o2h_LCMS_Method_A_SLOW
Injection Volume:	2.00 ul	Processing Method	O2H_LCMS_02_00,
Run Time:	4.0 Minutes	Channel Name:	320.0nm, MS TIC, 210.0nm
Project Name:	2024\LCMS-05_DEC-2024_	Proc. Chnl. Descr.:	PDA 320.0 nm Blank Subtracted
Date Acquired:	11-12-2024 11:10:55 IST		
Date Processed:	11-12-2024 11:13:03 IST, 11-12-2024 11:13:09 IST, 11-12-2024 11:15:45 IST		
Column:	X-BRIDGE C18 2.1X50mm 2.5um		

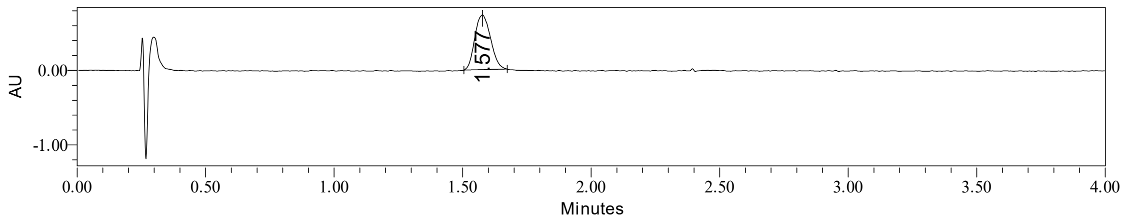

Channel Name 210.0nm; Channel PDA Spectrum

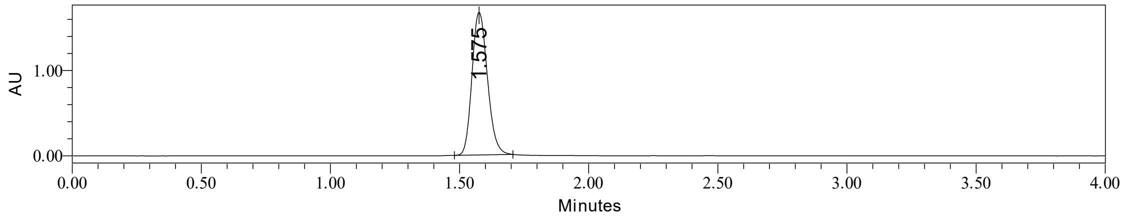

Channel Name 320.0nm; Channel PDA Spectrum

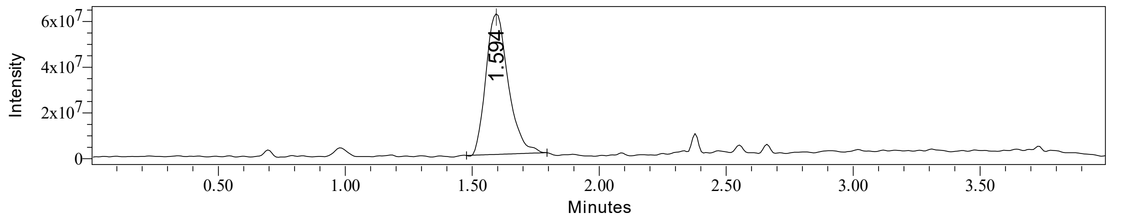

Channel Name MS TIC; Channel QDa 1: MS Scan

Peak Results
Channel: PDA Spectrum

	RT	Base Peak (m/z)	Height	Area	% Area	Channel	Channel Name
1	1.575		1672316	6953918	100.00	PDA Spectrum	320.0nm
2	1.577		734870	3121970	100.00	PDA Spectrum	210.0nm

Peak Results
Channel: QDa 1: MS Scan

	RT	Base Peak (m/z)	Height	Area	% Area	Channel	Channel Name
1	1.594	320.97	61539881	376392453	100.00	QDa 1: MS Scan	MS TIC

HPLC and LCMS Traces for Compound 15b

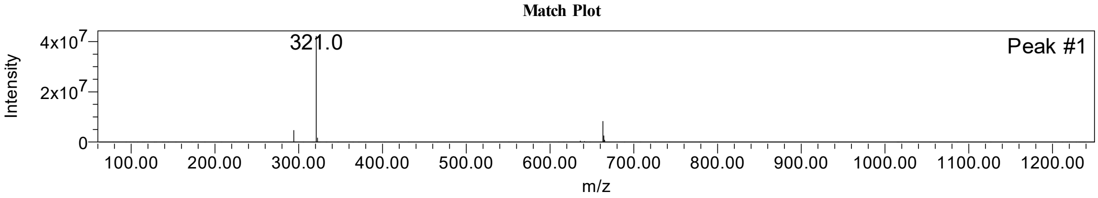

Base Peak 320.97 Channel Description 1: QDa Positive(+) Scan (60.00-1240.00)Da, Centroid, CV=10 - AVG (2.1:4.0;0.1:0.8) x 20.000 Th: 0.010 Retention Time 1.594

HPLC and LCMS Traces for Compound 15b

o2h discovery
Ahmedabad, Gujarat
India

HPLC Analysis Report

Sample name: CIN2-C-816-CIN2-X-0101-088-c

Location: P2-A9

Injection: 1 of 1

Injection volume: 20.000

Project Name HPLC-07_DEC-2024

Date Acquired: 2024-12-12 02:59:43+05:30

Date Processed: 2024-12-12 03:23:40+05:30

Instrument Name HPLC-07

Acq. method: o2h_HPLC_Method-C.amx

Processing method: *3D UV Quantitative_DefaultMethod.pmx

Column: SUNFIRE C18 150x4.6mm 3.5um

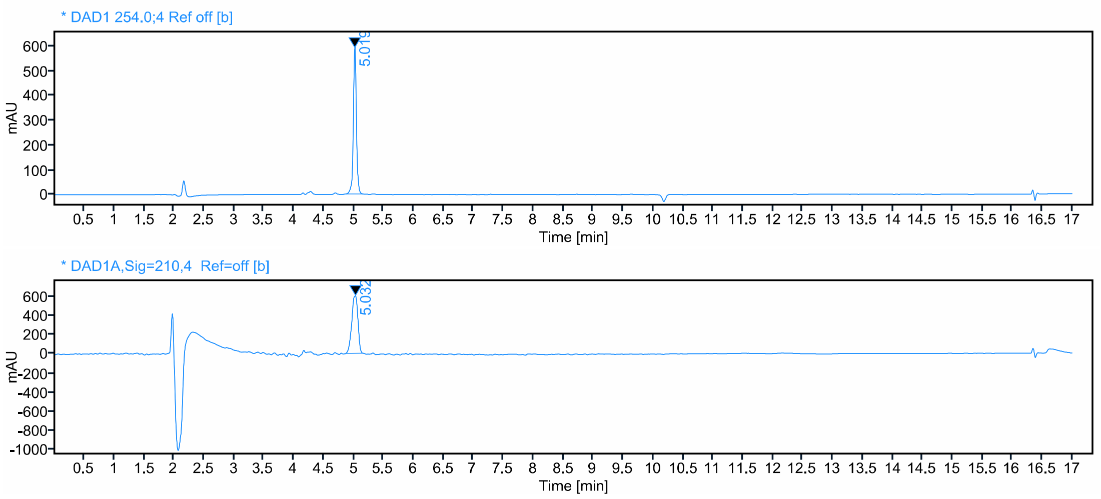

Signal: * DAD1 254.0;4 Ref off [b]

RT [min]	Height	Area	Area%
5.019	591	2160	100.00

Signal: * DAD1A,Sig=210,4 Ref=off [b]

RT [min]	Height	Area	Area%
5.032	604	4318	100.00

HPLC and LCMS Traces for Compound 15c

o2h discovery
Ahmedabad, Gujarat
India

LCMS Analysis Report

Sample Name:	CIN2-D-051-CIN2-X-0100-032-J		Injection Id 53568
Sample Type:	Unknown	Acquired By:	LCMS-05
Vial:	1:A,8	Sample Set Name:	26122024_UCH_NEW_FD_
Injection #:	1	Acq. Method Set:	o2h_LCMS_Method_A_SLOW_01
Injection Volume:	8.00 ul	Processing Method	O2H_LCMS_02,
Run Time:	4.0 Minutes	Channel Name:	325.0nm, MS TIC, 210.0nm
Project Name:	2024\LCMS-05_DEC-2024_	Proc. Chnl. Descr.:	QDa 1: MS Scan MS TIC,
Date Acquired:	26-12-2024 10:38:30 IST		
Date Processed:	26-12-2024 10:42:10 IST, 26-12-2024 10:42:20 IST, 26-12-2024 10:45:13 IST		
Column:	X-BRIDGE C18 2.1X50mm 2.5um		

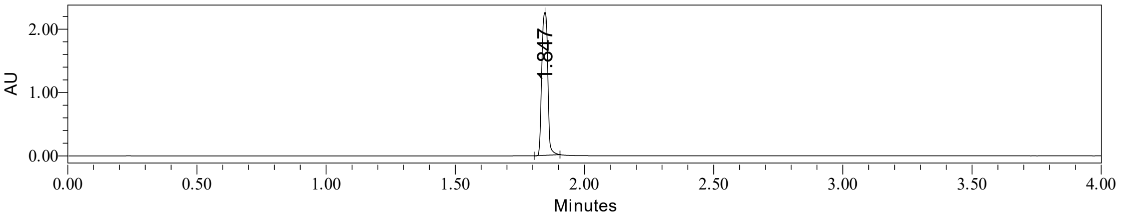

Channel Name 325.0nm; Channel PDA Spectrum

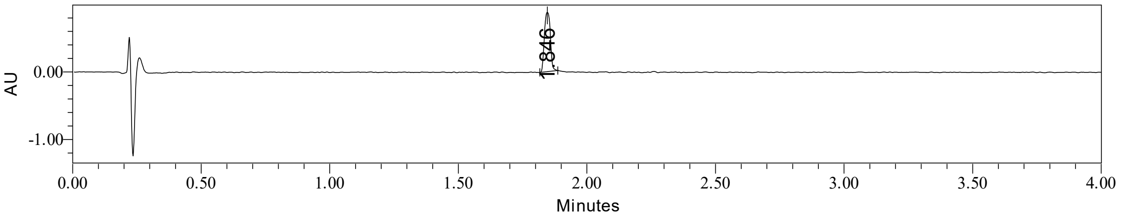

Channel Name 210.0nm; Channel PDA Spectrum

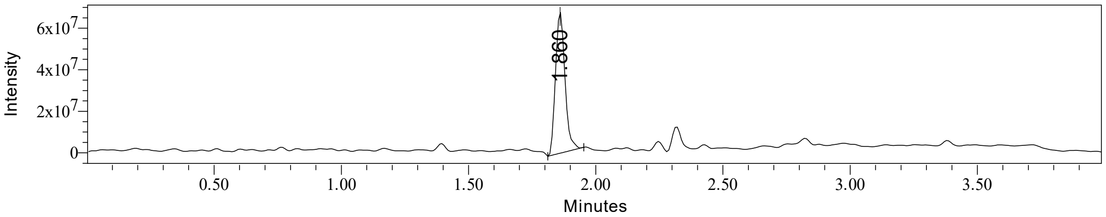

Channel Name MS TIC; Channel QDa 1: MS Scan

Peak Results
Channel: PDA Spectrum

	RT	Base Peak (m/z)	Height	Area	% Area	Channel	Channel Name
1	1.846		878998	1409855	100.00	PDA Spectrum	210.0nm
2	1.847		2258564	3606835	100.00	PDA Spectrum	325.0nm

Peak Results
Channel: QDa 1: MS Scan

	RT	Base Peak (m/z)	Height	Area	% Area	Channel	Channel Name
1	1.860	320.96	67328796	175478424	100.00	QDa 1: MS Scan	MS TIC

HPLC and LCMS Traces for Compound 15c

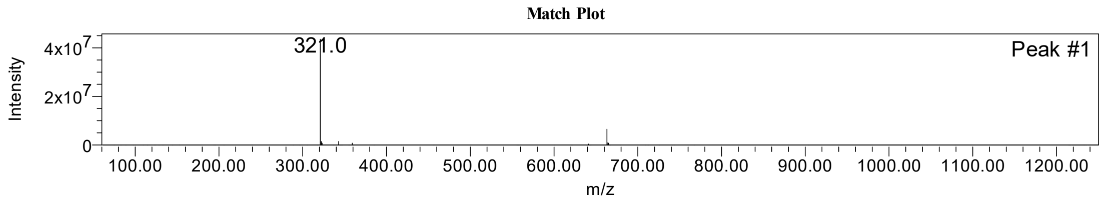

Base Peak 320.96 Channel Description 1: QDa Positive(+) Scan (60.00-1240.00)Da, Centroid, CV=10 - AVG (2.1:4.0;0.3:0.9) x 20.000 Th: 0.010 Retention Time 1.860

HPLC and LCMS Traces for Compound 15c

o2h discovery
Ahmedabad, Gujarat
India

HPLC Analysis Report

Sample name: CIN2-D-051-CIN2-X-0100-032-J
Location: P2-A2
Injection: 1 of 1
Injection volume: 30.000
Project Name HPLC-07_DEC-2024
Date Acquired: 2024-12-26 15:11:50+05:30
Date Processed: 2024-12-26 15:50:16+05:30

Instrument Name HPLC-07
Acq. method: o2h_HPLC_Method-C.amx
Processing method: *3D UV Quantitative_DefaultMethod.pmx
Column: SUNFIRE C18 150x4.6mm 3.5um

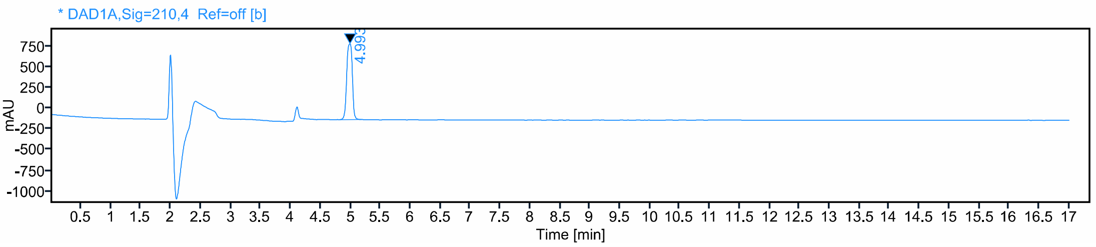

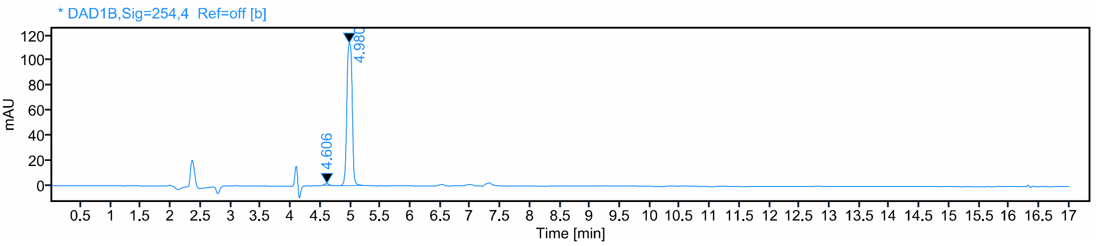

Signal: * DAD1A,Sig=210,4 Ref=off [b]

RT [min]	Height	Area	Area%
4.993	911	5893	100.00

Signal: * DAD1B,Sig=254,4 Ref=off [b]

RT [min]	Height	Area	Area%
4.606	1	7	0.93
4.980	114	698	99.07

HPLC and LCMS Traces for Compound 16a

Method1

SAMPLE INFORMATION

Sample Name:	CNA-80	Acquired By:	BillyGroup
Sample Type:	Unknown	Sample Set Name:	NA_28112025
Vial:	1:F,2	Acq. Method Set:	HPLC_NA
Injection #:	1	Processing Method:	Huso
Injection Volume:	3.00 ul	Channel Name:	220.0nm@3
Run Time:	25.0 Minutes	Proc. Chnl. Descr.:	2998 PDA 220.0 nm (2998
Date Acquired:	28/11/2025 2:26:31 PM HKT		
Date Processed:	4/12/2025 11:08:55 AM HKT		

Auto-Scaled Chromatogram

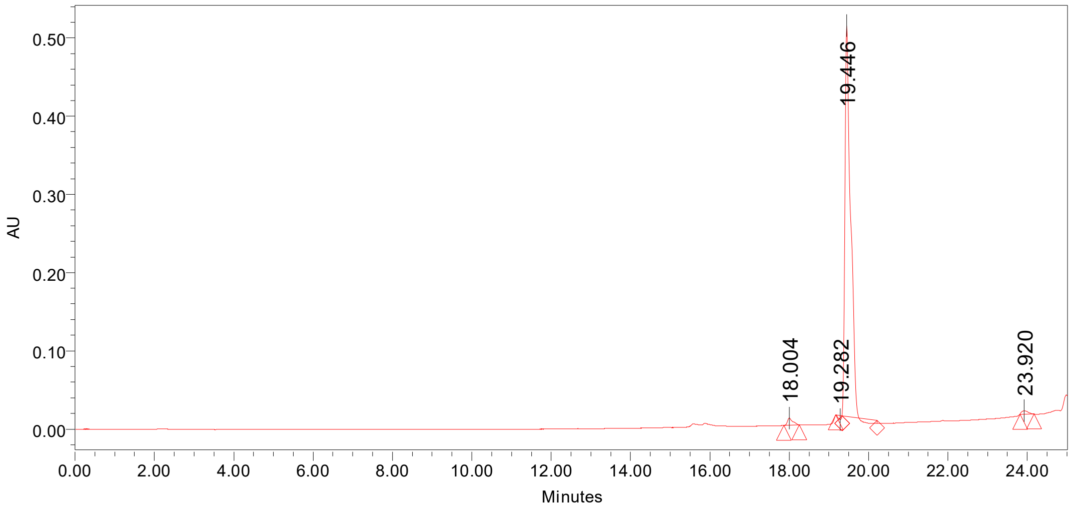

Peak Results

	Retention Time (min)	% Area	Sample Set Name	Channel Name	% Height
1	18.004	1.64	NA_28112025	220.0nm@3	1.79
2	19.282	0.56	NA_28112025	220.0nm@3	0.99
3	19.446	96.89	NA_28112025	220.0nm@3	96.41
4	23.920	0.90	NA_28112025	220.0nm@3	0.81

Reported by User: BillyGroup (BillyGroup)
Report Method: Method1
Report Method ID: 2308
Page: 1 of 1

Project Name: Nucleoside Analogues
Date Printed:
4/12/2025
11:09:04 am Asia/Hong_Kong

HPLC and LCMS Traces for Compound 16a

Method1

SAMPLE INFORMATION

Sample Name:	CNA-80	Acquired By:	BillyGroup
Sample Type:	Unknown	Sample Set Name:	NA_28112025
Vial:	1:F,2	Acq. Method Set:	HPLC_NA
Injection #:	1	Processing Method:	Huso
Injection Volume:	3.00 ul	Channel Name:	280.0nm@1
Run Time:	25.0 Minutes	Proc. Chnl. Descr.:	2998 PDA 280.0 nm (2998
Date Acquired:	28/11/2025 2:26:31 PM HKT		
Date Processed:	4/12/2025 11:07:58 AM HKT		

Auto-Scaled Chromatogram

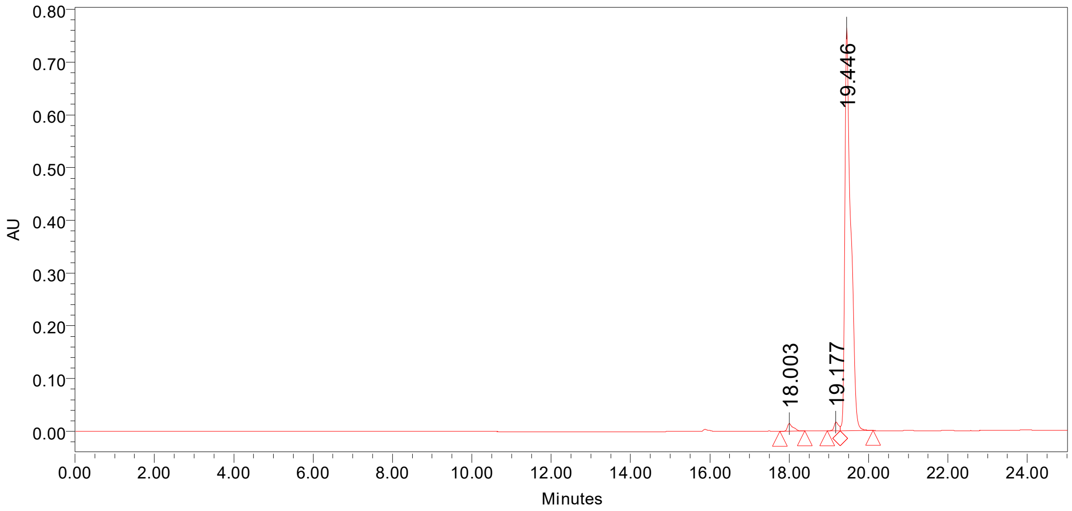

Peak Results

	Retention Time (min)	% Area	Sample Set Name	Channel Name	% Height
1	18.003	1.73	NA_28112025	280.0nm@1	1.81
2	19.177	1.66	NA_28112025	280.0nm@1	2.12
3	19.446	96.61	NA_28112025	280.0nm@1	96.08

Reported by User: BillyGroup (BillyGroup)
Report Method: Method1
Report Method ID: 2308
Page: 1 of 1

Project Name: Nucleoside Analogues
Date Printed:
4/12/2025
11:09:39 am Asia/Hong_Kong

HPLC and LCMS Traces for Compound 17a

o2h discovery
Ahmedabad, Gujarat
India

LCMS Analysis Report

**Sample name:**	CIN-C-375-X-0070-027-a		
**Location:**	P2-B2	**Instrument Name**	LCMS-09
**Injection:**	1 of 1	**Acq. method:**	o2h_LCMS_DEV_27.amx
**Injection volume:**	20.000	**Processing method:**	*LC_MS Sample Purity_DefaultMethod_NEW.pmx
**Project Name**	LCMS-09_JUNE-2024	**Description:**	
**Date Acquired:**	2024-06-03 19:53:51+05:30	**Column:**	SUNFIRE C18 150x4.6mm, 3.5um
**Date Processed:**	2024-06-03 20:41:36+05:30		

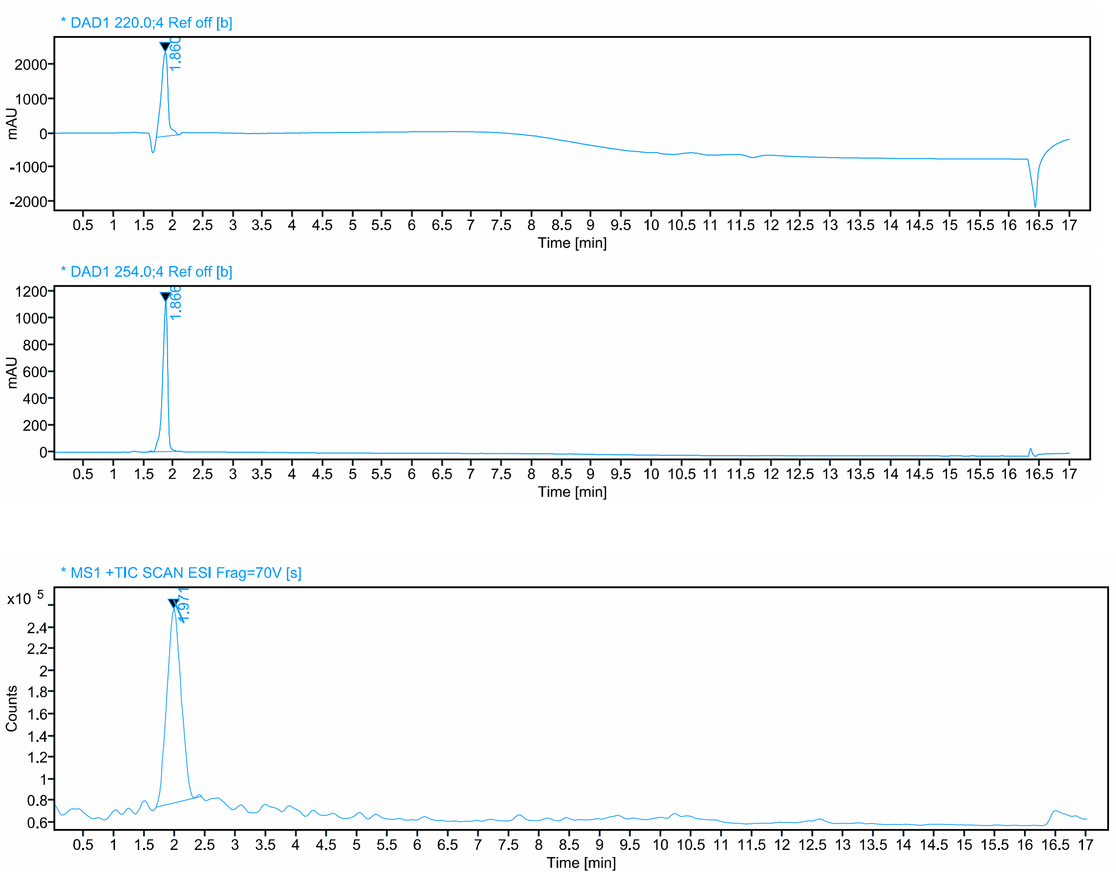

Signal: * DAD1 220.0;4 Ref off [b]

RT [min]	Base Peak (m/z)	Height	Area	Area%
1.860		2462	19334	100.00

Printed: 2024-06-03 20:42:06+05:30 **Page 1 of 2**

HPLC and LCMS Traces for Compound 17a

o2h discovery
Ahmedabad, Gujarat
India

LCMS Analysis Report

Signal: * DAD1 254.0;4 Ref off [b]

RT [min]	Base Peak (m/z)	Height	Area	Area%
1.866		1108	6577	100.00

Signal: * MS1 +TIC SCAN ESI Frag=70V [s]

RT [min]	Base Peak (m/z)	Height	Area	Area%
1.971	639.700	178751	2925213	100.00

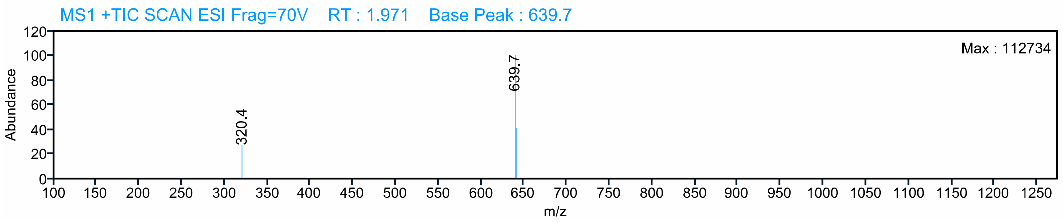

HPLC and LCMS Traces for Compound 17a

o2h discovery
Ahmedabad, Gujarat
India

HPLC Analysis Report

Sample name: CIN-C-375-X-0070-027-a
Location: P2-C2
Injection: 1 of 1
Injection volume: 20.000
Project Name HPLC-10_JUNE-2024
Date Acquired: 2024-06-03 20:58:40+05:30
Date Processed: 2024-06-03 21:25:50+05:30

Instrument Name HPLC-10
Acq. method: o2h_HPLC_Method-A.amx
Processing method: 3D UV Quantitative_DefaultMethod.pmx
Column: SUNFIRE C18 150x4.6mm,3.5um

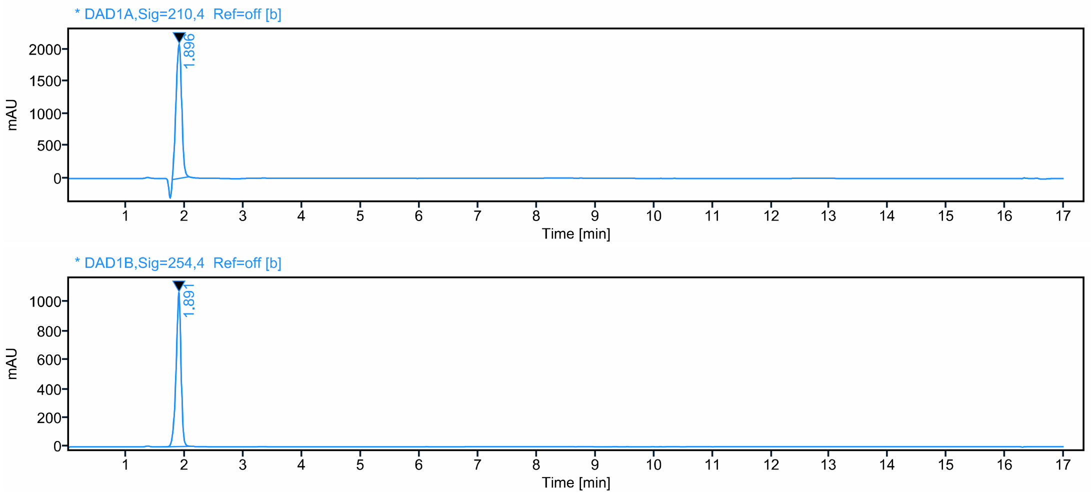

Signal: * DAD1A,Sig=210,4 Ref=off [b]

RT [min]	Height	Area	Area%
1.896	2079	14108	100.00

Signal: * DAD1B,Sig=254,4 Ref=off [b]

RT [min]	Height	Area	Area%
1.891	1066	6315	100.00

HPLC and LCMS Traces for Compound 17b

o2h discovery
Ahmedabad, Gujarat
India

LCMS Analysis Report

Sample name: CIN-C-375-CIN-X-0072-036-b
Location: P2-B4
Injection: 1 of 1
Injection volume: 10.000

Instrument Name LCMS-09
Acq. method: LC05_MSS3.amx
Processing method: *LC_MS Sample Purity_DefaultMethod_NEW.pmx

Project Name LCMS-09_JULY-2024
Date Acquired: 2024-07-08 12:12:47+05:30
Date Processed: 2024-07-08 12:34:25+05:30

Description:
Column: SUNFIRE C18 150x4.6mm, 3.5um

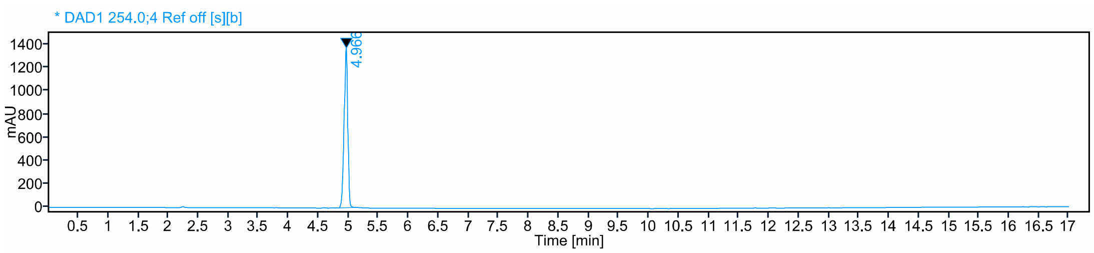

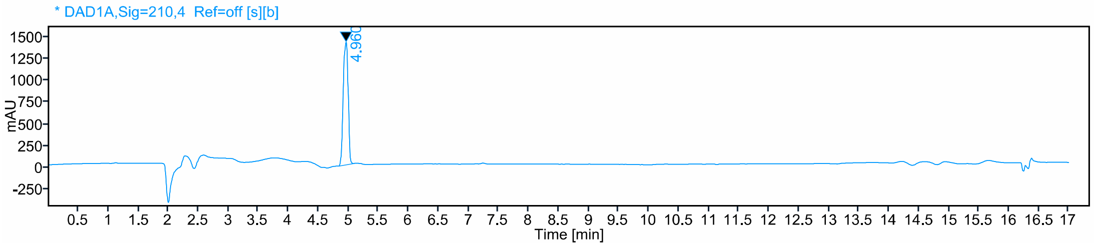

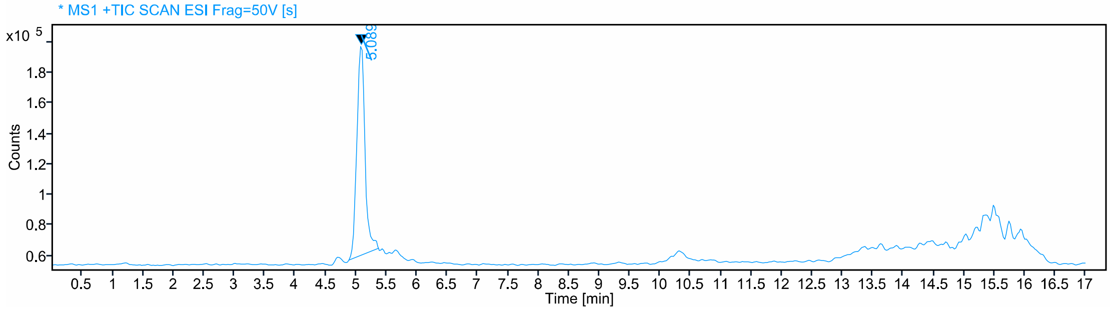

Signal: * DAD1 254.0;4 Ref off [s][b]

RT [min]	Base Peak (m/z)	Height	Area	Area%
4.966		1366	5833	100.00

Printed: 2024-07-08 12:34:36+05:30 **Page 1 of 2**

HPLC and LCMS Traces for Compound 17b

o2h discovery
Ahmedabad, Gujarat
India

LCMS Analysis Report

Signal: * DAD1A,Sig=210,4 Ref=off [s][b]

RT [min]	Base Peak (m/z)	Height	Area	Area%
4.960		1403	7948	100.00

Signal: * MS1 +TIC SCAN ESI Frag=50V [s]

RT [min]	Base Peak (m/z)	Height	Area	Area%
5.089	639.700	137686	1281516	100.00

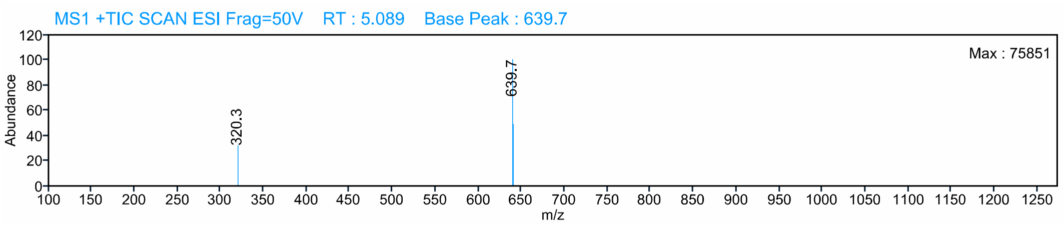

Printed: 2024-07-08 12:34:36+05:30

HPLC and LCMS Traces for Compound 17b

o2h discovery
Ahmedabad, Gujarat
India

HPLC Analysis Report

Sample name: CIN-C-375-CIN-X-0072-036-b
Location: P2-A3
Injection: 1 of 1
Injection volume: 10.000
Project Name HPLC-07_JUL-2024
Date Acquired: 2024-07-08 14:19:29+05:30
Date Processed: 2024-07-08 14:39:35+05:30

Instrument Name HPLC-07
Acq. method: o2h_HPLC_Method-D.amx
Processing method: 3D UV Quantitative_DefaultMethod.pmx
Column: XBridge C18 150x4.6mm, 3.5um

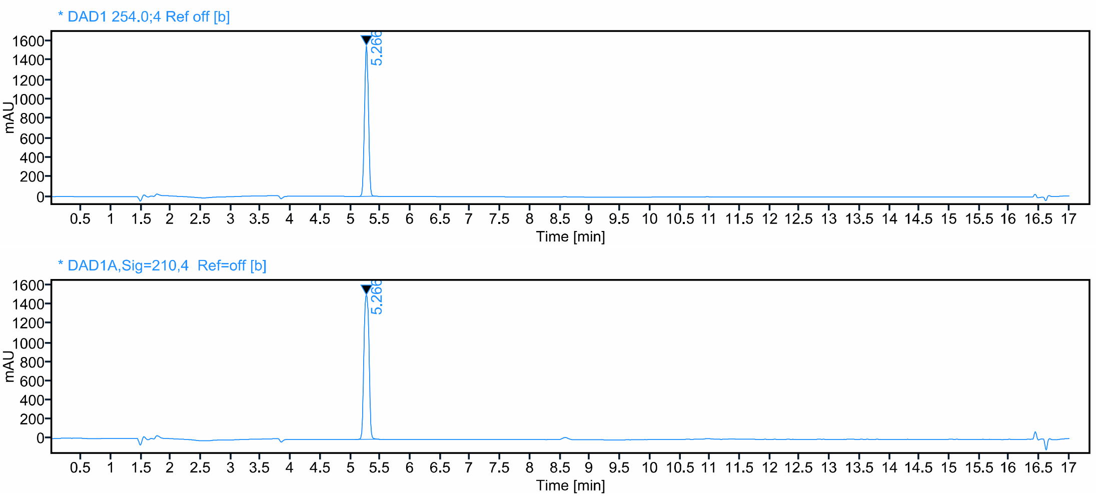

Signal: * DAD1 254.0;4 Ref off [b]

RT [min]	Height	Area	Area%
5.266	1546	6985	100.00

Signal: * DAD1A,Sig=210,4 Ref=off [b]

RT [min]	Height	Area	Area%
5.266	1506	8863	100.00

HPLC and LCMS Traces for Compound 17c

o2h discovery
Ahmedabad, Gujarat
India

LCMS Analysis Report

Sample Name:	CIN-C-375-CIN-X-0071-049-d		Injection Id 20535
Sample Type:	Unknown	Acquired By:	LCMS-05
Vial:	2:F,8	Sample Set Name:	08072024_UCH_082_RD_
Injection #:	1	Acq. Method Set:	o2h_LCMS_Method_A_SLOW_01
Injection Volume:	2.00 ul	Processing Method	O2H_LCMS_02_00,
Run Time:	4.0 Minutes	Channel Name:	MS TIC, 254.0nm, 210.0nm
Project Name:	2024\LCMS-05_JUL-2024	Proc. Chnl. Descr.:	PDA 254.0 nm Blank Subtracted
Date Acquired:	08-07-2024 15:16:12 IST		
Date Processed:	08-07-2024 16:00:45 IST, 08-07-2024 16:01:04 IST, 08-07-2024 16:01:47 IST		
Column:	X-BRIDGE C18 2.1X50mm 2.5um		

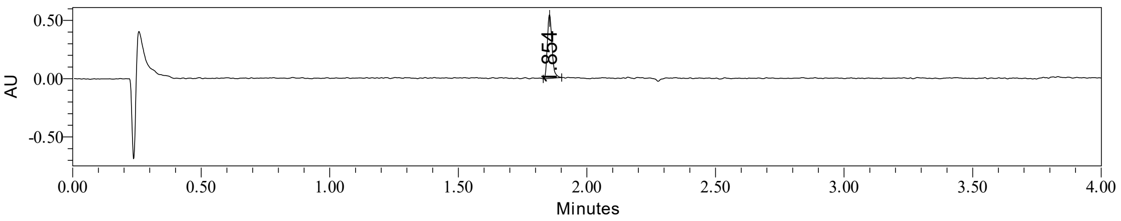

Channel PDA Spectrum; Channel Name 210.0nm

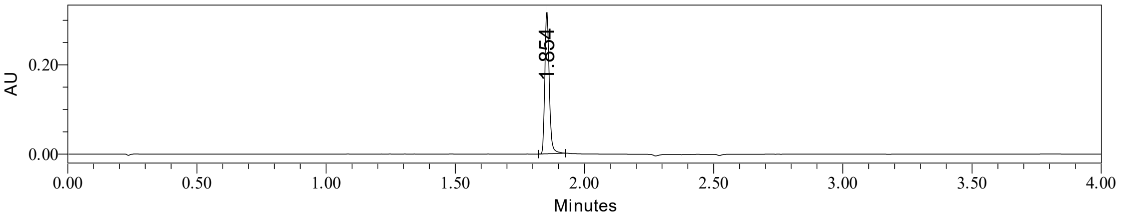

Channel PDA Spectrum; Channel Name 254.0nm

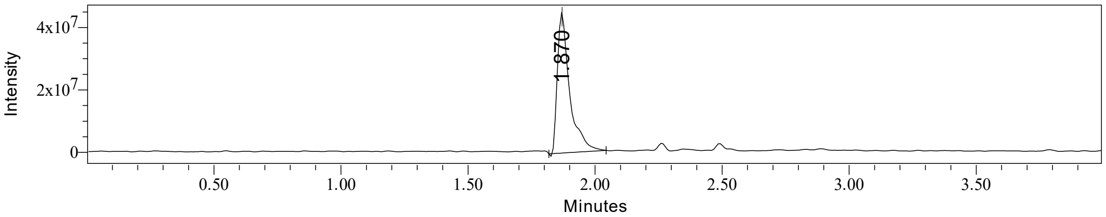

Channel QDa 1: MS Scan; Channel Name MS TIC

Peak Results
Channel: PDA Spectrum

	RT	Base Peak (m/z)	Height	Area	% Area	Channel	Channel Name
1	1.854		546763	682816	100.00	PDA Spectrum	210.0nm
2	1.854		317847	376913	100.00	PDA Spectrum	254.0nm

Peak Results
Channel: QDa 1: MS Scan

	RT	Base Peak (m/z)	Height	Area	% Area	Channel	Channel Name
1	1.870	320.23	44762597	151927735	100.00	QDa 1: MS Scan	MS TIC

HPLC and LCMS Traces for Compound 17c

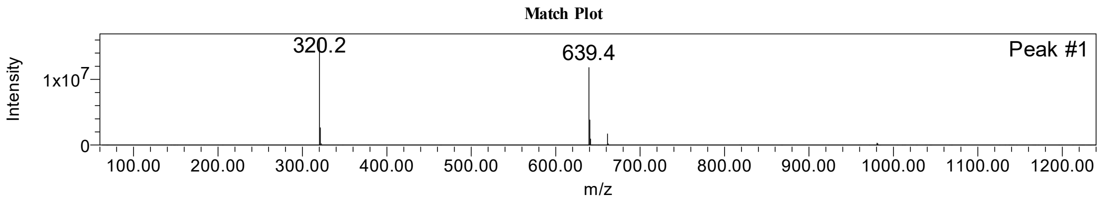

Base Peak 320.23 Channel Description 1: QDa Positive(+) Scan (60.00-1240.00)Da, Centroid, CV=10 - AVG (2.5:3.9;0.1:1.0) x 20.000 Th: 0.010 Retention Time 1.870

HPLC and LCMS Traces for Compound 17c

o2h discovery<br>Ahmedabad, Gujarat<br>India

HPLC Analysis Report

Sample name: CIN-C-375-CIN-X-0071-049-d
Location: P2-A4
Injection: 1 of 1
Injection volume: 10.000
Project Name HPLC-07_JUL-2024
Date Acquired: 2024-07-08 14:57:21+05:30
Date Processed: 2024-07-08 15:15:55+05:30

Instrument Name HPLC-07
Acq. method: o2h_HPLC_Method-D.amx
Processing method: 3D UV Quantitative_DefaultMethod.pmx
Column: XBridge C18 150x4.6mm, 3.5um

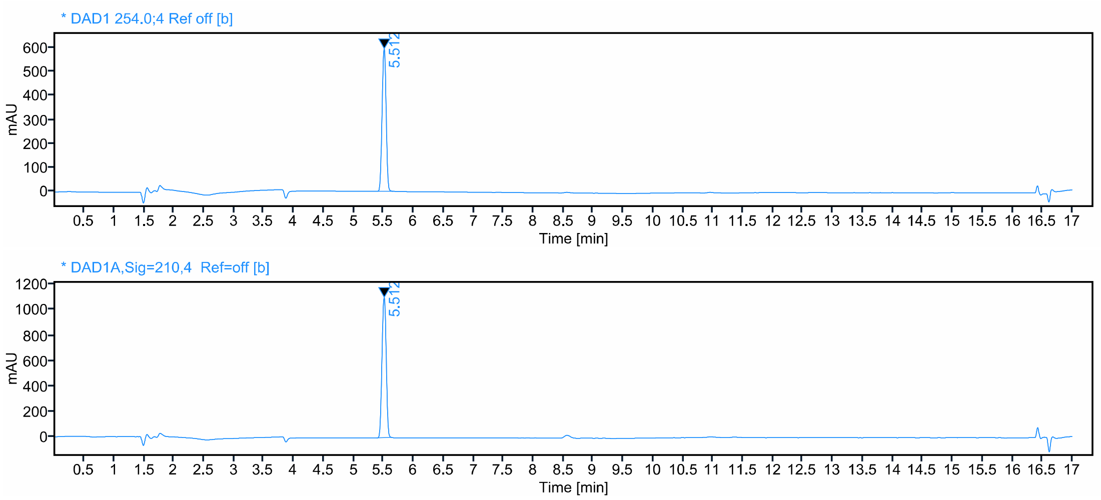

Signal: * DAD1 254.0;4 Ref off [b]

RT [min]	Height	Area	Area%
5.512	591	2718	100.00

Signal: * DAD1A,Sig=210,4 Ref=off [b]

RT [min]	Height	Area	Area%
5.512	1103	5336	100.00

HPLC and LCMS Traces for Compound 18d

o2h discovery
Ahmedabad, Gujarat
India

LCMS Analysis Report

Sample Name:	CIN2-C-816-CIN2-X-0093-080-A		Injection Id 32476
Sample Type:	Unknown	Acquired By:	LCMS-05
Vial:	2:A,4	Sample Set Name:	14122024_UCH_NEW_FD
Injection #:	1	Acq. Method Set:	o2h_LCMS_Method_A_SLOW_01
Injection Volume:	2.00 ul	Processing Method	O2H_LCMS_02_00,
Run Time:	4.0 Minutes	Channel Name:	MS TIC, 254.0nm, 210.0nm
Project Name:	2024\LCMS-05_DEC-2024_	Proc. Chnl. Descr.:	QDa 1: MS Scan MS TIC,
Date Acquired:	14-12-2024 13:08:49 IST		
Date Processed:	14-12-2024 13:23:21 IST, 14-12-2024 13:23:27 IST, 14-12-2024 13:23:52 IST		
Column:	X-BRIDGE C18 2.1X50mm 2.5um		

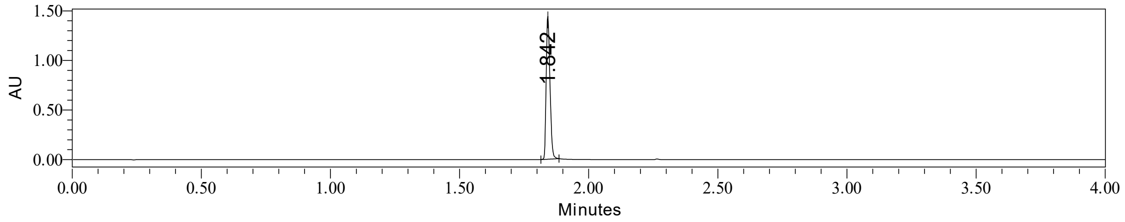

Channel PDA Spectrum; Channel Name 254.0nm

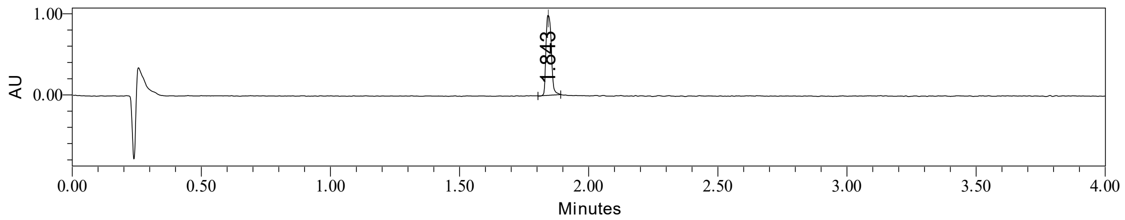

Channel PDA Spectrum; Channel Name 210.0nm

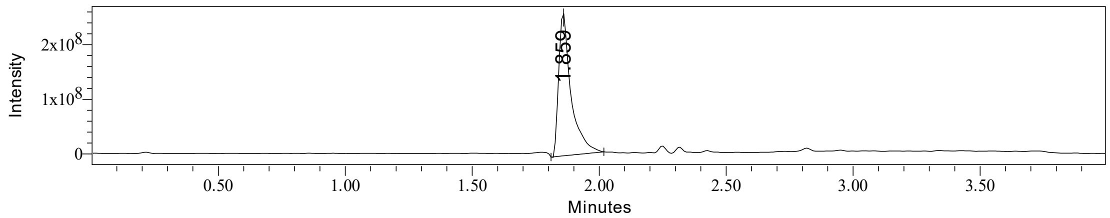

Channel QDa 1: MS Scan; Channel Name MS TIC

Peak Results
Channel: PDA Spectrum

	RT	Base Peak (m/z)	Height	Area	% Area	Channel	Channel Name
1	1.842		1439700	1469404	100.00	PDA Spectrum	254.0nm
2	1.843		992178	1421272	100.00	PDA Spectrum	210.0nm

Peak Results
Channel: QDa 1: MS Scan

	RT	Base Peak (m/z)	Height	Area	% Area	Channel	Channel Name
1	1.859	587.29	258438504	881475135	100.00	QDa 1: MS Scan	MS TIC

HPLC and LCMS Traces for Compound 18d

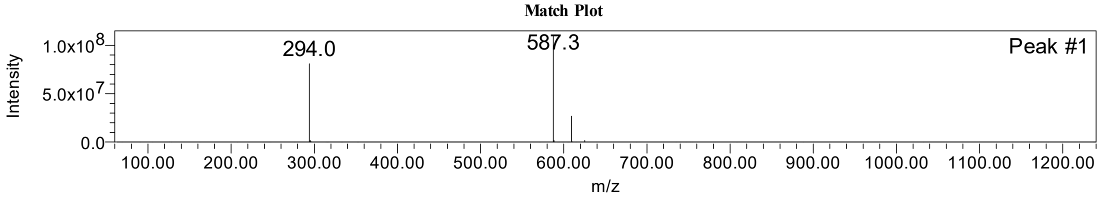

Base Peak 587.29 Channel Description 1: QDa Positive(+) Scan (60.00-1240.00)Da, Centroid, CV=10 - AVG (2.3:3.8;0.6:0.8) x 20.000 Th: 0.010 Retention Time 1.859

HPLC and LCMS Traces for Compound 18d

o2h discovery
Ahmedabad, Gujarat
India

HPLC Analysis Report

**Sample name:**	CIN2-C-816-CIN2-X-0093-080-A		
**Location:**	P1-A5	**Instrument Name**	HPLC-10
**Injection:**	1 of 1	**Acq. method:**	o2h_HPLC_Method-D.amx
**Injection volume:**	20.000	**Processing method:**	*3D UV Quantitative_DefaultMethod.pmx
**Project Name**	HPLC-10_DEC-2024	**Column:**	XBridge C18 150x4.6mm, 3.5um
**Date Acquired:**	2024-12-16 08:36:04+05:30		
**Date Processed:**	2024-12-16 09:16:39+05:30		

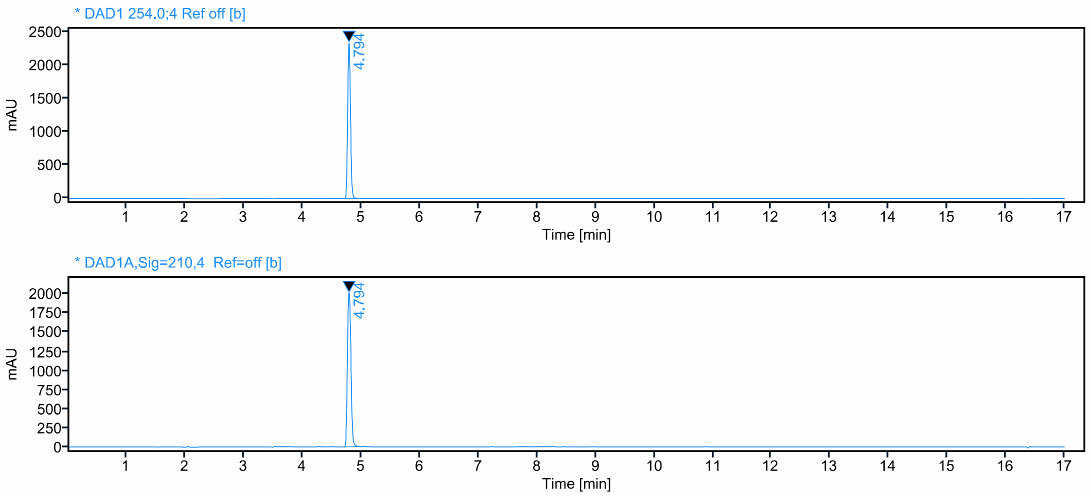

Signal: * DAD1 254.0;4 Ref off [b]

RT [min]	Height	Area	Area%
4.794	2335	8045	100.00

Signal: * DAD1A,Sig=210,4 Ref=off [b]

RT [min]	Height	Area	Area%
4.794	2022	8625	100.00

Printed: 2024-12-16 09:16:50+05:30 **Page 1 of 1**

HPLC and LCMS Traces for Compound 18e

o2h discovery
Ahmedabad, Gujarat
India

LCMS Analysis Report

Sample Name:	CIN2-D-051-CIN2-X-0092-004-c		Injection Id 26621
Sample Type:	Unknown	Acquired By:	LCMS-05
Vial:	2:C,7	Sample Set Name:	12122024_UCH_NEW_FD
Injection #:	1	Acq. Method Set:	o2h_LCMS_Method_A_SLOW
Injection Volume:	3.00 ul	Processing Method	O2H_LCMS_03_0,
Run Time:	4.0 Minutes	Channel Name:	285.0nm, MS TIC, 210.0nm
Project Name:	2024\LCMS-05_DEC-2024_	Proc. Chnl. Descr.:	QDa 1: MS Scan MS TIC,
Date Acquired:	12-12-2024 13:04:37 IST		
Date Processed:	12-12-2024 13:22:33 IST, 12-12-2024 13:22:42 IST, 12-12-2024 13:23:07 IST		
Column:	X-BRIDGE C18 2.1X50mm 2.5um		

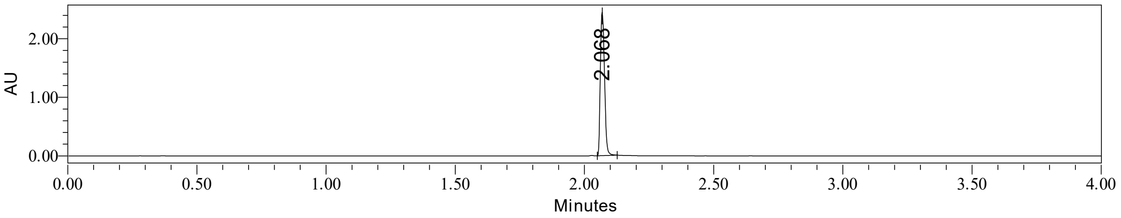

Channel Name 285.0nm; Channel PDA Spectrum

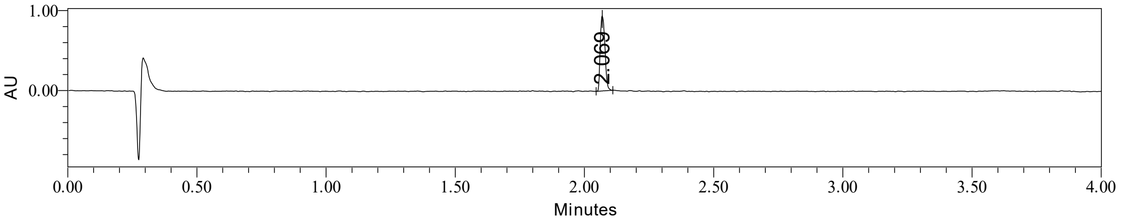

Channel Name 210.0nm; Channel PDA Spectrum

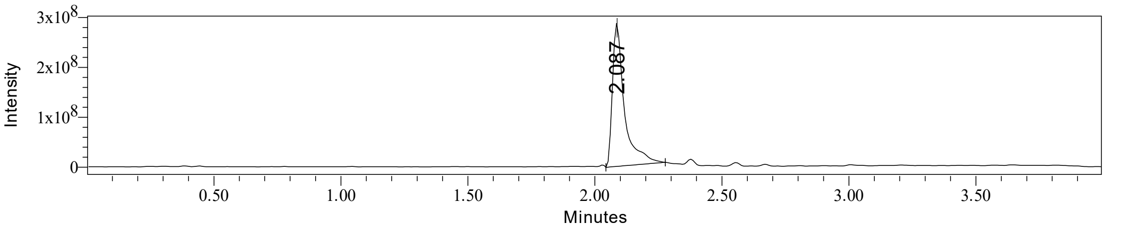

Channel Name MS TIC; Channel QDa 1: MS Scan

Peak Results
Channel: PDA Spectrum

	RT	Base Peak (m/z)	Height	Area	% Area	Channel	Channel Name
1	2.068		2444329	2755667	100.00	PDA Spectrum	285.0nm
2	2.069		939693	118215[illegible]	100.00	PDA Spectrum	210.0nm

Peak Results
Channel: QDa 1: MS Scan

	RT	Base Peak (m/z)	Height	Area	% Area	Channel	Channel Name
1	2.087	555.38	284131629	939985953	100.00	QDa 1: MS Scan	MS TIC

HPLC and LCMS Traces for Compound 18e

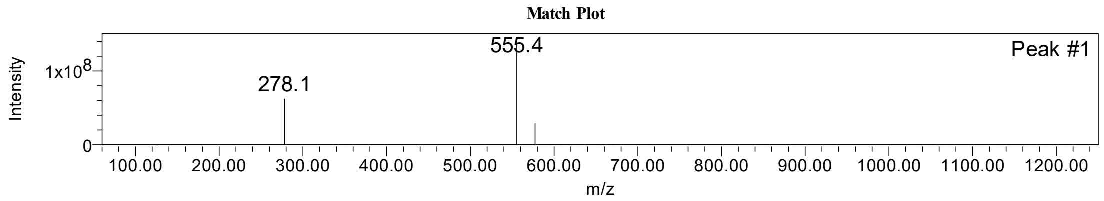

Base Peak 555.38 Channel Description 1: QDa Positive(+) Scan (60.00-1240.00)Da, Centroid, CV=10 - AVG (2.7:3.8;0.5:1.3) x 20.000 Th: 0.010 Retention Time 2.087

HPLC and LCMS Traces for Compound 18e

o2h discovery
Ahmedabad, Gujarat
India

HPLC Analysis Report

**Sample name:**	CIN2-D-051-CIN2-X-0092-004-c		
**Location:**	P1-A5	**Instrument Name**	HPLC-10
**Injection:**	1 of 1	**Acq. method:**	o2h_HPLC_Method-D.amx
**Injection volume:**	10.000	**Processing method:**	*3D UV Quantitative_DefaultMethod.pmx
**Project Name**	HPLC-10_DEC-2024	**Column:**	XBridge C18 150x4.6mm, 3.5um
**Date Acquired:**	2024-12-12 13:29:56+05:30		
**Date Processed:**	2024-12-12 13:57:02+05:30		

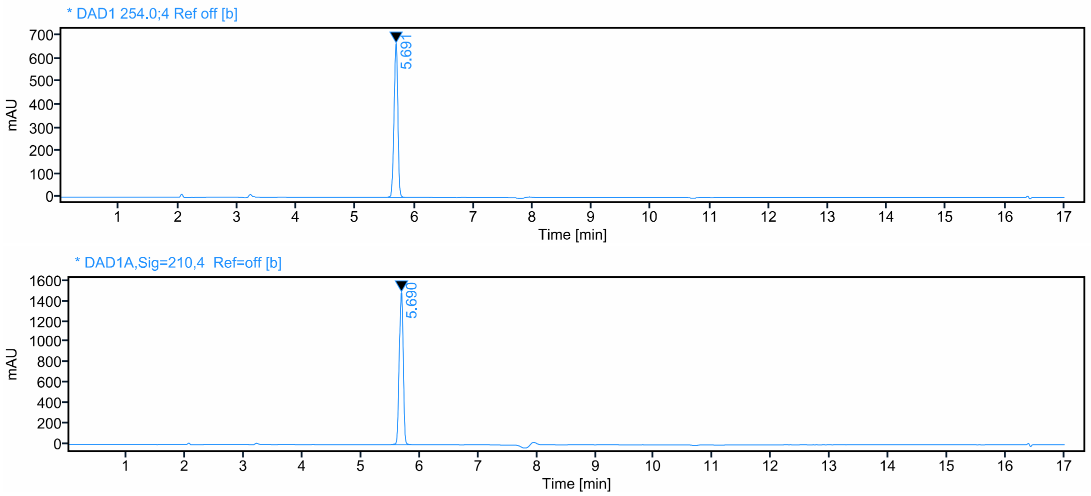

Signal: * DAD1 254.0;4 Ref off [b]

RT [min]	Height	Area	Area%
5.691	668	3035	100.00

Signal: * DAD1A,Sig=210,4 Ref=off [b]

RT [min]	Height	Area	Area%
5.690	1488	6914	100.00

Printed: 2024-12-12 13:57:12+05:30 **Page 1 of 1**

HPLC and LCMS Traces for Compound 18f

o2h discovery
Ahmedabad, Gujarat
India

LCMS Analysis Report

Sample Name: CIN2-D-051-CIN2-X-0097-013-c
Injection Id 34625
Sample Type: Unknown
Acquired By: LCMS-05
Vial: 1:F,2
Sample Set Name: 16122024_UCH_NEW_FD
Injection #: 1
Acq. Method Set: o2h_LCMS_Method_A_SLOW_01
Injection Volume: 2.00 ul
Processing Method O2H_LCMS_02_0,
Run Time: 4.0 Minutes
Channel Name: MS TIC, 254.0nm, 210.0nm
Project Name: 2024\LCMS-05_DEC-2024_
Proc. Chnl. Descr.: PDA 254.0 nm Blank Subtracted
Date Acquired: 16-12-2024 12:12:57 IST
Date Processed: 16-12-2024 12:25:28 IST, 16-12-2024 12:25:41 IST, 16-12-2024 12:26:06 IST
Column: X-BRIDGE C18 2.1X50mm 2.5um

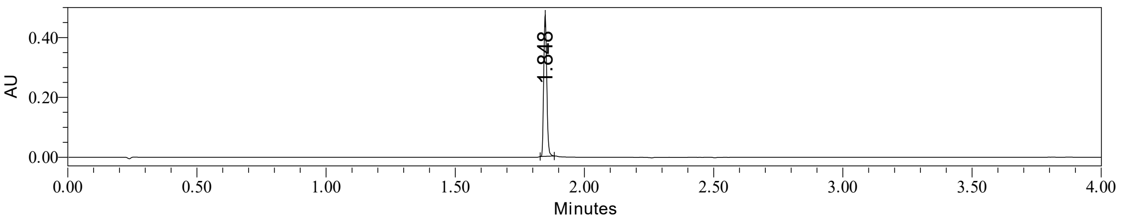

Channel Name 254.0nm; Channel PDA Spectrum

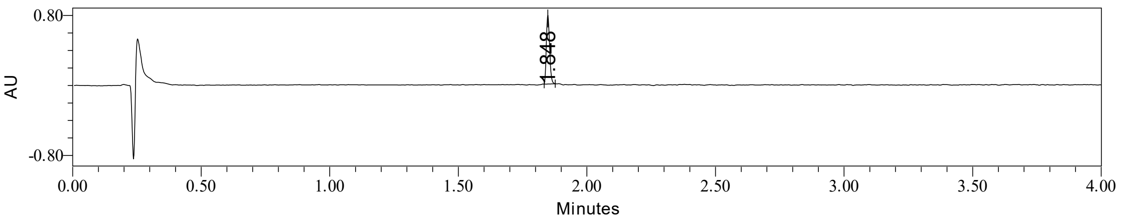

Channel Name 210.0nm; Channel PDA Spectrum

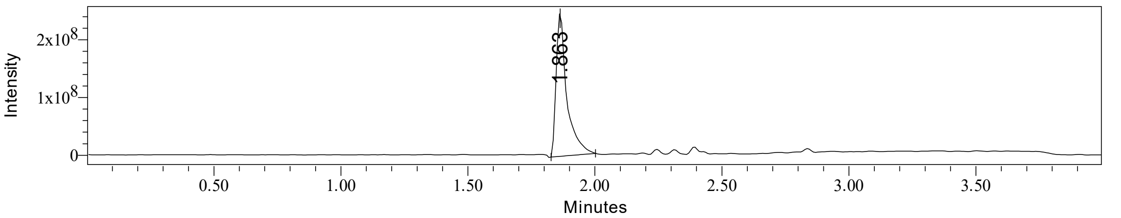

Channel Name MS TIC; Channel QDa 1: MS Scan

Peak Results
Channel: PDA Spectrum

	RT	Base Peak (m/z)	Height	Area	% Area	Channel	Channel Name
1	1.848		473627	386358	100.00	PDA Spectrum	254.0nm
2	1.848		784983	692150	100.00	PDA Spectrum	210.0nm

Peak Results
Channel: QDa 1: MS Scan

	RT	Base Peak (m/z)	Height	Area	% Area	Channel	Channel Name
1	1.863	278.04	249286900	724038675	100.00	QDa 1: MS Scan	MS TIC

HPLC and LCMS Traces for Compound 18f

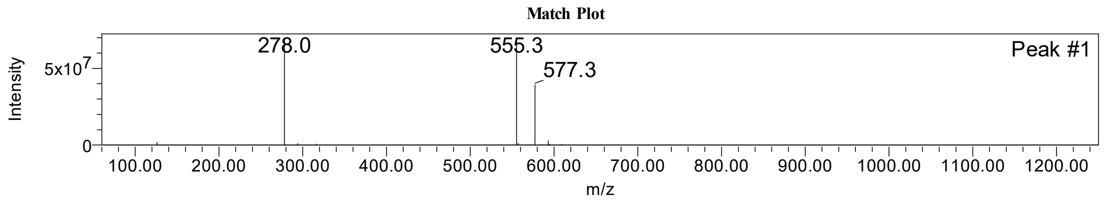

Base Peak 278.04 Channel Description 1: QDa Positive(+) Scan (60.00-1240.00)Da, Centroid, CV=10 - AVG (0.4:1.6;2.1:4.0) x 20.000 Th: 0.010 Retention Time 1.863

HPLC and LCMS Traces for Compound 18f

o2h discovery
Ahmedabad, Gujarat
India

HPLC Analysis Report

Sample name: CIN2-D-051-CIN2-X-0097-013-c
Location: P1-A6
Injection: 1 of 1
Injection volume: 10.000
Instrument Name HPLC-10
Acq. method: o2h_HPLC_Method-D.amx
Processing method: *3D UV Quantitative_DefaultMethod.pmx

Project Name HPLC-10_DEC-2024
Date Acquired: 2024-12-16 11:16:36+05:30
Date Processed: 2024-12-16 11:37:36+05:30
Column: XBridge C18 150x4.6mm, 3.5um

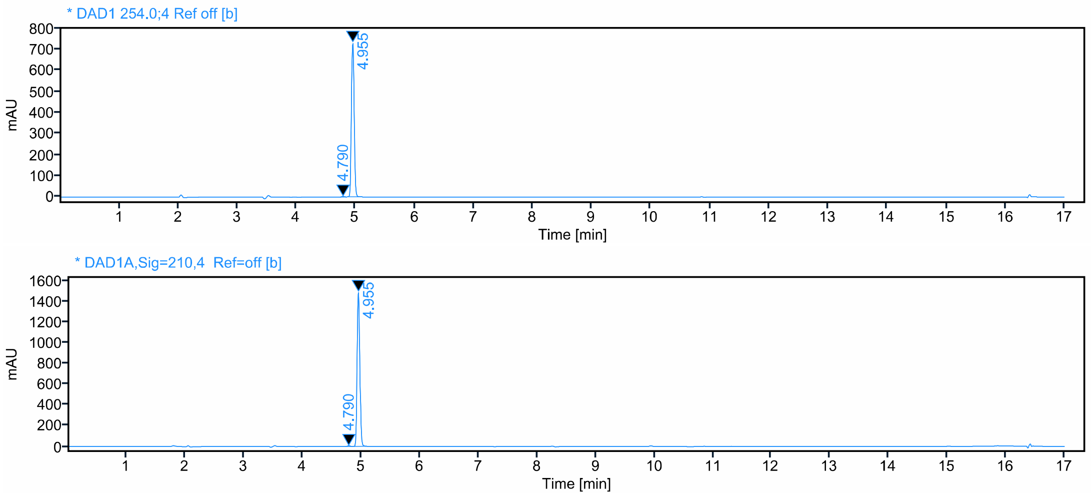

Signal: * DAD1 254.0;4 Ref off [b]

RT [min]	Height	Area	Area%
4.790	3	9	0.39
4.955	732	2285	99.61

Signal: * DAD1A,Sig=210,4 Ref=off [b]

RT [min]	Height	Area	Area%
4.790	7	20	0.41
4.955	1488	4782	99.59

Printed: 2024-12-16 11:39:22+05:30 **Page 1 of 1**

HPLC and LCMS Traces for Compound 19a

o2h discovery
Ahmedabad, Gujarat
India

LCMS Analysis Report

Sample Name:	CIN-C-377-CIN-X-0031-034-b		Injection Id 3651
Sample Type:	Unknown	Acquired By:	LCMS-05
Vial:	1:E,1	Sample Set Name:	03062024_UCH_082_FD_01
Injection #:	1	Acq. Method Set:	o2h_LCMS_Method_A_SLOW
Injection Volume:	2.00 ul	Processing Method:	O2H_LCMS_02, O2H_LCMS_01_0
Run Time:	4.0 Minutes	Channel Name:	MS TIC, 254.0nm, 210.0nm
Project Name:	2024\LCMS-05_JUN-2024	Proc. Chnl. Descr.:	PDA 254.0 nm Blank Subtracted
Date Acquired:	03-06-2024 22:58:59 IST		
Date Processed:	04-06-2024 08:45:16 IST, 04-06-2024 08:45:22 IST, 04-06-2024 08:45:50 IST		
Column:	X-BRIDGE C18 2.1X50mm 2.5um		

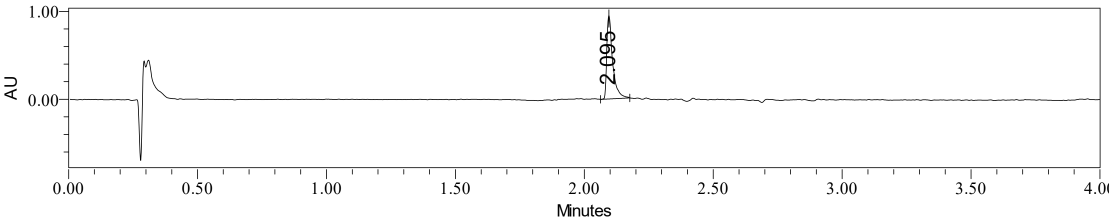

Channel Name 210.0nm; Channel PDA Spectrum

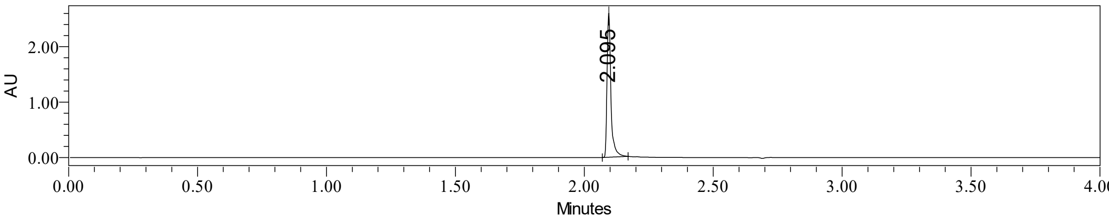

Channel Name 254.0nm; Channel PDA Spectrum

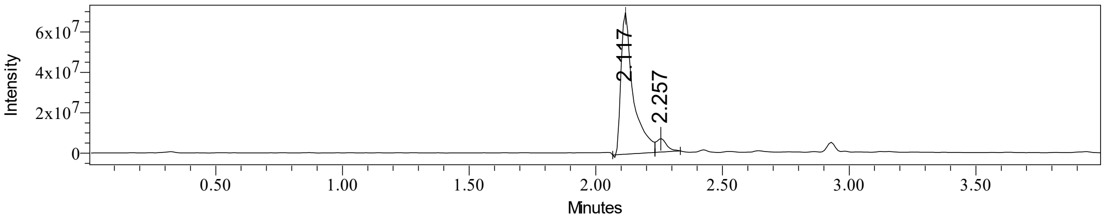

Channel Name MS TIC; Channel QDa 1: MS Scan

Peak Results
Channel: PDA Spectrum

	RT	Base Peak (m/z)	Height	Area	% Area	Channel	Channel Name
1	2.095		2601523	2711322	100.00	PDA Spectrum	254.0nm
2	2.095		949994	1497266	100.00	PDA Spectrum	210.0nm

Peak Results
Channel: QDa 1: MS Scan

	RT	Base Peak (m/z)	Height	Area	% Area	Channel	Channel Name
1	2.117	318.23	69329529	236846303	92.60	QDa 1: MS Scan	MS TIC

HPLC and LCMS Traces for Compound 19a

Peak Results
Channel: QDa 1: MS Scan

	RT	Base Peak (m/z)	Height	Area	% Area	Channel	Channel Name
2	2.257	136.08	6698300	18921477	7.40	QDa 1: MS Scan	MS TIC

Match Plot

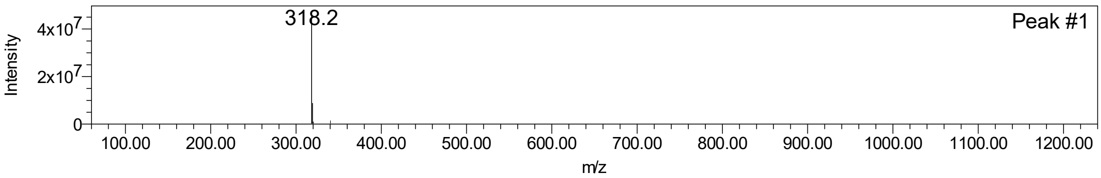

Base Peak 318.23 Channel Description 1: QDa Positive(+) Scan (60.00-1240.00)Da, Centroid, CV=10 - AVG (2.4:2.7;2.9:4.0;0.0:1.0) x 10.000 Th: 0.010 Retention Time 2.117

Match Plot

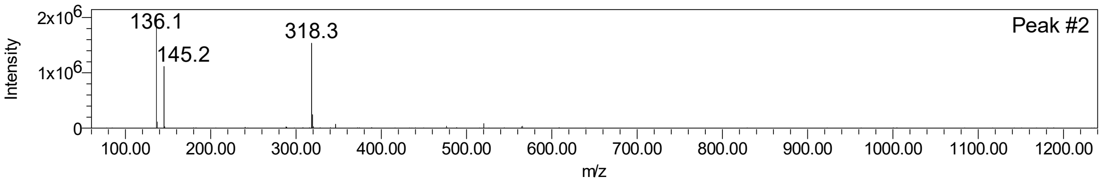

Base Peak 136.08 Channel Description 1: QDa Positive(+) Scan (60.00-1240.00)Da, Centroid, CV=10 - AVG (2.4:2.7;2.9:4.0;0.0:1.0) x 10.000 Th: 0.010 Retention Time 2.257

HPLC and LCMS Traces for Compound 19a

o2h discovery
Ahmedabad, Gujarat
India

HPLC Analysis Report

Sample name: CIN-C-377-CIN-X-0031-034-b
Location: P2-D3
Injection: 1 of 1
Injection volume: 10.000
Project Name HPLC-07_JUNE-2024
Date Acquired: 2024-06-04 12:01:15+05:30
Date Processed: 2024-06-04 12:24:27+05:30

Instrument Name HPLC-07
Acq. method: o2h_HPLC_Method-B.amx
Processing method: *3D UV Quantitative_DefaultMethod.pmx
Column: XBridge C18 150x4.6mm, 3.5um

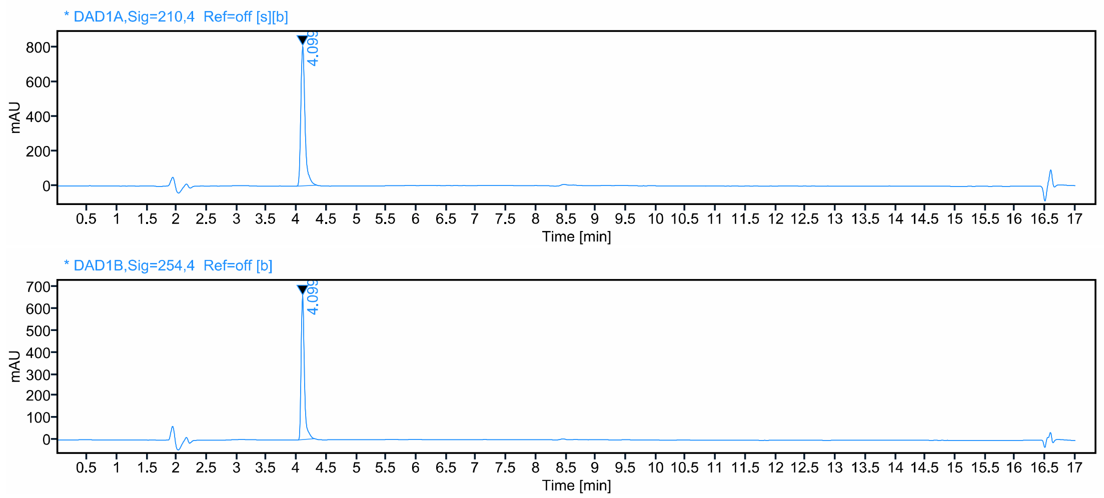

Signal: * DAD1A,Sig=210,4 Ref=off [s][b]

RT [min]	Height	Area	Area%
4.099	808	3870	100.00

Signal: * DAD1B,Sig=254,4 Ref=off [b]

RT [min]	Height	Area	Area%
4.099	658	2444	100.00

HPLC and LCMS Traces for Compound 19b

o2h discovery
Ahmedabad, Gujarat
India

LCMS Analysis Report

Sample Name:	CIN-C-377-CIN-X-0033-074-a		Injection Id 43491
Sample Type:	Unknown	Acquired By:	LCMS-05
Vial:	2:F,8	Sample Set Name:	21062024_UCH_082_RD
Injection #:	1	Acq. Method Set:	o2h_LCMS_Method_A_SLOW
Injection Volume:	2.00 ul	Processing Method	O2H_LCMS_08_0,
Run Time:	4.0 Minutes	Channel Name:	MS TIC, 254.0nm, 210.0nm
Project Name:	2024\LCMS-05_JUN-2024	Proc. Chnl. Descr.:	PDA 254.0 nm Blank Subtracted
Date Acquired:	21-06-2024 23:15:34 IST		
Date Processed:	21-06-2024 23:40:34 IST, 21-06-2024 23:40:47 IST, 21-06-2024 23:41:12 IST		
Column:	X-BRIDGE C18 2.1X50mm 2.5um		

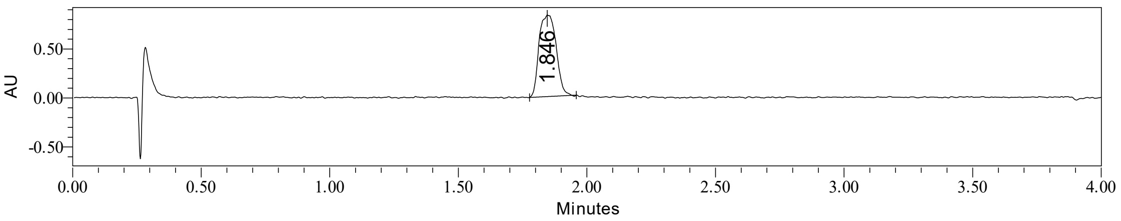

——— Channel Name 210.0nm; Channel PDA Spectrum

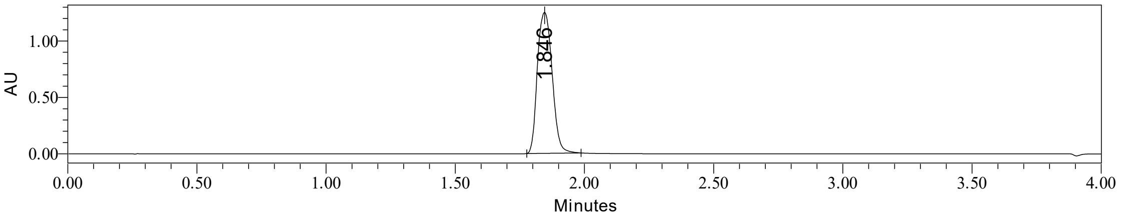

——— Channel Name 254.0nm; Channel PDA Spectrum

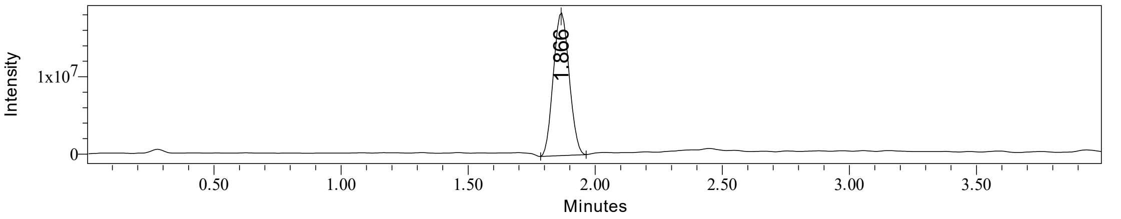

——— Channel Name MS TIC; Channel QDa 1: MS Scan

Peak Results
Channel: PDA Spectrum

	RT	Base Peak (m/z)	Height	Area	% Area	Channel	Channel Name
1	1.846		1250342	4956951	100.00	PDA Spectrum	254.0nm
2	1.846		832848	3837292	100.00	PDA Spectrum	210.0nm

Peak Results
Channel: QDa 1: MS Scan

	RT	Base Peak (m/z)	Height	Area	% Area	Channel	Channel Name
1	1.866	318.25	18467249	79652408	100.00	QDa 1: MS Scan	MS TIC

HPLC and LCMS Traces for Compound 19b

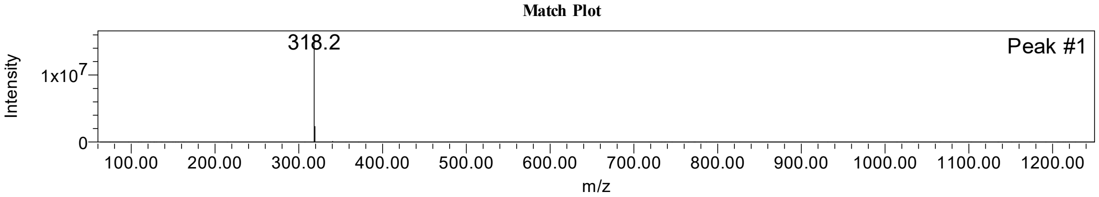

Base Peak 318.25 Channel Description 1: QDa Positive(+) Scan (60.00-1240.00)Da, Centroid, CV=10 - AVG (2.1:3.9;0.2:1.7) x 40.000 Th: 0.010 Retention Time 1.866

HPLC and LCMS Traces for Compound 19b

o2h discovery
Ahmedabad, Gujarat
India

HPLC Analysis Report

Sample name: CIN-C-377-CIN-X-0033-074-a
Location: P1-B10
Injection: 1 of 1
Injection volume: 10.000
Project Name HPLC-10_JUNE-2024
Date Acquired: 2024-06-21 21:13:16+05:30
Date Processed: 2024-06-21 21:53:28+05:30

Instrument Name HPLC-10
Acq. method: o2h_HPLC_Method-C.amx
Processing method: *3D UV Quantitative_DefaultMethod.pmx
Column: SUNFIRE C18 150x4.6mm,3.5um

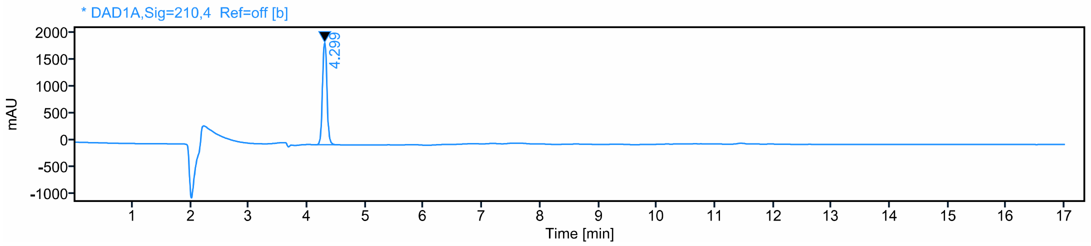

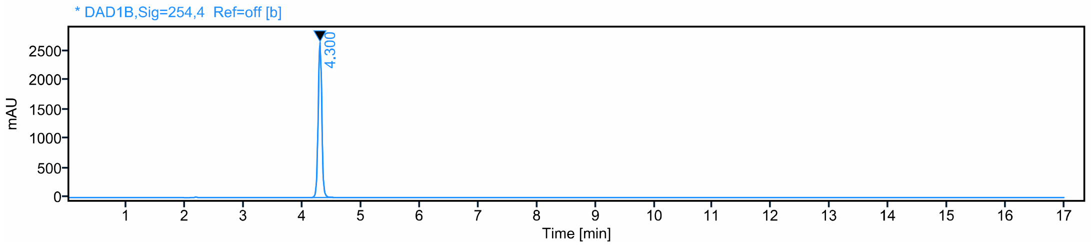

Signal: * DAD1A,Sig=210,4 Ref=off [b]

RT [min]	Height	Area	Area%
4.299	1898	10309	100.00

Signal: * DAD1B,Sig=254,4 Ref=off [b]

RT [min]	Height	Area	Area%
4.300	2648	11189	100.00

HPLC and LCMS Traces for Compound 19c

o2h discovery
Ahmedabad, Gujarat
India

LCMS Analysis Report

Sample Name: CIN-C-377-CIN-X-0032-068-a
Injection Id 29265
Sample Type: Unknown
Acquired By: LCMS-05
Vial: 2:A,7
Sample Set Name: 14062024_UCH_083_RD
Injection #: 1
Acq. Method Set: o2h_LCMS_Method_A_SLOW
Injection Volume: 2.00 ul
Processing Method O2H_LCMS_02_0,
Run Time: 4.0 Minutes
Channel Name: MS TIC, 254.0nm, 210.0nm
Project Name: 2024\LCMS-05_JUN-2024
Proc. Chnl. Descr.: QDa 1: MS Scan MS TIC,
Date Acquired: 14-06-2024 13:24:29 IST
Date Processed: 14-06-2024 13:46:08 IST, 14-06-2024 13:46:18 IST, 14-06-2024 13:46:46 IST
Column: X-BRIDGE C18 2.1X50mm 2.5um

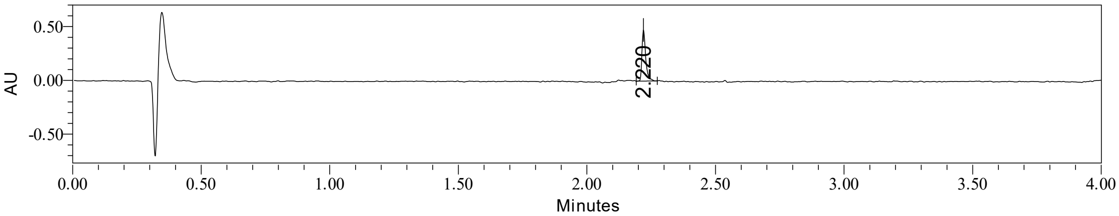

Channel Name 210.0nm; Channel PDA Spectrum

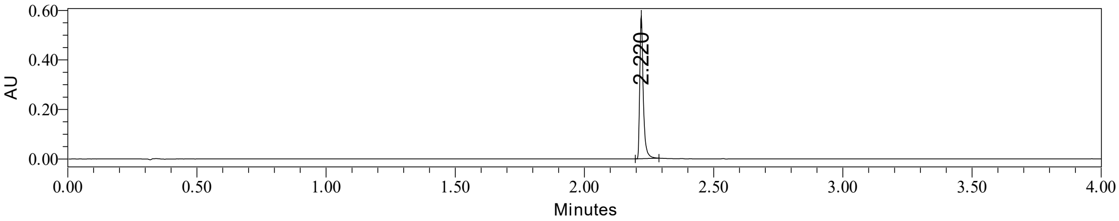

Channel Name 254.0nm; Channel PDA Spectrum

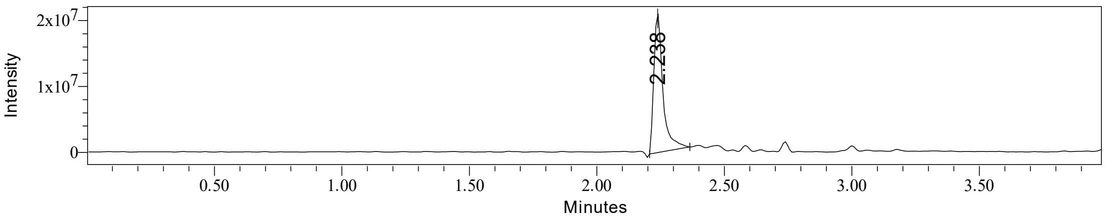

Channel Name MS TIC; Channel QDa 1: MS Scan

Peak Results
Channel: PDA Spectrum

	RT	Base Peak (m/z)	Height	Area	% Area	Channel	Channel Name
1	2.220		576558	541931	100.00	PDA Spectrum	254.0nm
2	2.220		474916	482888	100.00	PDA Spectrum	210.0nm

Peak Results
Channel: QDa 1: MS Scan

	RT	Base Peak (m/z)	Height	Area	% Area	Channel	Channel Name
1	2.238	318.25	21169408	51924499	100.00	QDa 1: MS Scan	MS TIC

HPLC and LCMS Traces for Compound 19c

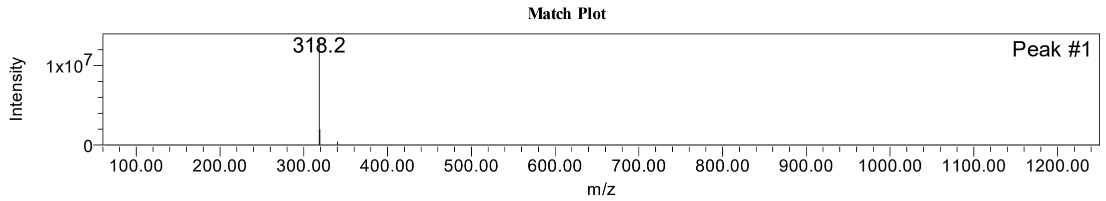

Base Peak 318.25 Channel Description 1: QDa Positive(+) Scan (60.00-1240.00)Da, Centroid, CV=10 - AVG (2.6:3.8;0.5:1.3) x 20.000 Th: 0.010 - AVG (2.5:3.0) x 20.000 Th: 0.010 Retention Time 2.238

HPLC and LCMS Traces for Compound 19c

o2h discovery
Ahmedabad, Gujarat
India

HPLC Analysis Report

**Sample name:**	CIN-C-377-CIN-X-0032-068-a		
**Location:**	P2-A11	**Instrument Name**	HPLC-10
**Injection:**	1 of 1	**Acq. method:**	o2h_HPLC_Method-C.amx
**Injection volume:**	10.000	**Processing method:**	*3D UV Quantitative_DefaultMethod.pmx
**Project Name**	HPLC-10_JUNE-2024		
**Date Acquired:**	2024-06-14 13:46:00+05:30	**Column:**	SUNFIRE C18 150x4.6mm,3.5um
**Date Processed:**	2024-06-14 14:32:26+05:30		

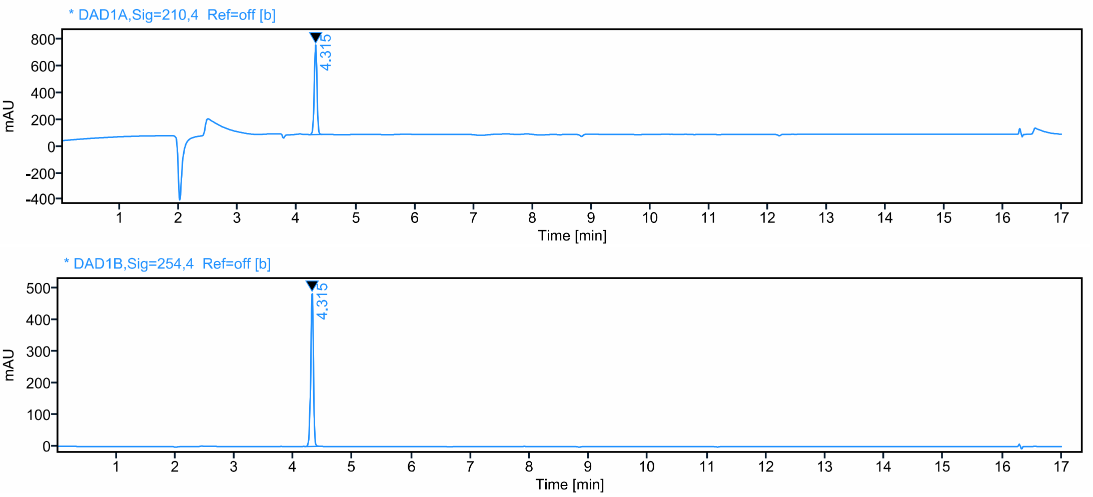

Signal: * DAD1A,Sig=210,4 Ref=off [b]

RT [min]	Height	Area	Area%
4.315	677	2179	100.00

Signal: * DAD1B,Sig=254,4 Ref=off [b]

RT [min]	Height	Area	Area%
4.315	487	1564	100.00

HPLC and LCMS Traces for Compound 20a

o2h discovery<br>Ahmedabad, Gujarat<br>India	**LCMS Analysis Report**

Sample Name:	CIN-C-376-CIN-X-0052-032		Injection Id 49169
Sample Type:	Unknown	Acquired By:	LCMS-05
Vial:	1:C,6	Sample Set Name:	28052024_UCH_082_FD
Injection #:	1	Acq. Method Set:	o2h_LCMS_Method_A_SLOW
Injection Volume:	2.00 ul	Processing Method:	O2H_LCMS_02_00,
Run Time:	4.0 Minutes	Channel Name:	MS TIC, 254.0nm, 210.0nm
Project Name:	2024\LCMS-05_MAY-2024	Proc. Chnl. Descr.:	PDA 254.0 nm Blank Subtracted
Date Acquired:	28-05-2024 09:49:03 IST		
Date Processed:	28-05-2024 09:53:04 IST, 28-05-2024 09:53:21 IST, 28-05-2024 09:54:10 IST, 28-05-2024		
Column:	X-BRIDGE C18 2.1X50mm 2.5um		

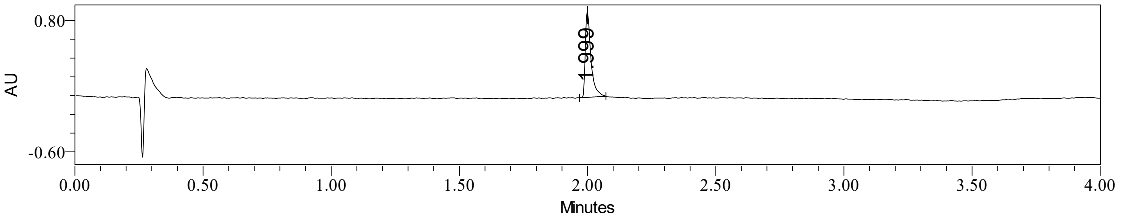

Channel Name 210.0nm; Channel PDA Spectrum

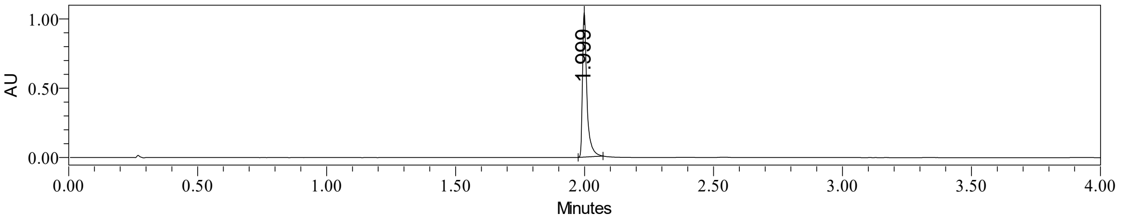

Channel Name 254.0nm; Channel PDA Spectrum

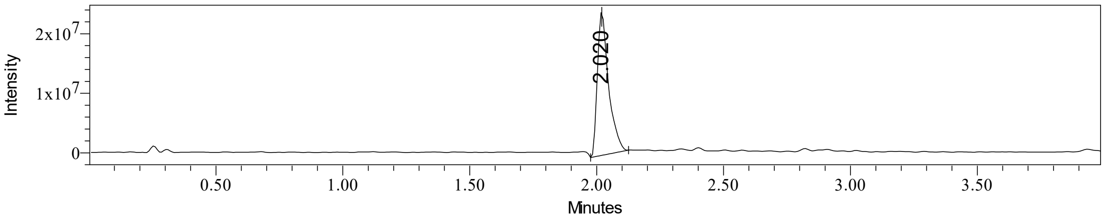

Channel Name MS TIC; Channel QDa 1: MS Scan

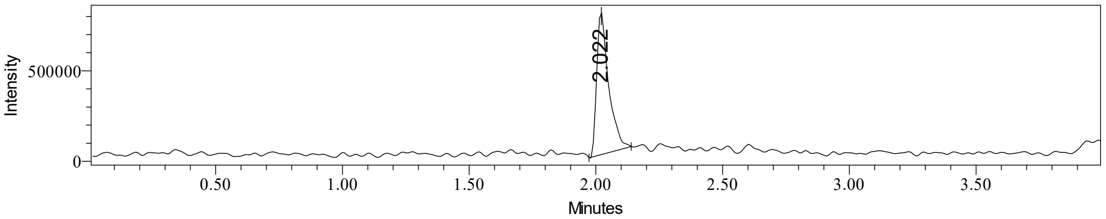

Channel Name MS TIC; Channel QDa 3: MS Scan

HPLC and LCMS Traces for Compound 20a

Peak Results
Channel: PDA Spectrum

	RT	Base Peak (m/z)	Height	Area	% Area	Channel	Channel Name
1	1.999		1043730	1240673	100.00	PDA Spectrum	254.0nm
2	1.999		910200	1390973	100.00	PDA Spectrum	210.0nm

Peak Results
Channel: QDa 1: MS Scan

	RT	Base Peak (m/z)	Height	Area	% Area	Channel	Channel Name
1	2.020	319.22	23984578	73843581	100.00	QDa 1: MS Scan	MS TIC

Peak Results
Channel: QDa 3: MS Scan

	RT	Base Peak (m/z)	Height	Area	% Area	Channel	Channel Name
1	2.022	363.18	779333	2574967	100.00	QDa 3: MS Scan	MS TIC

Match Plot

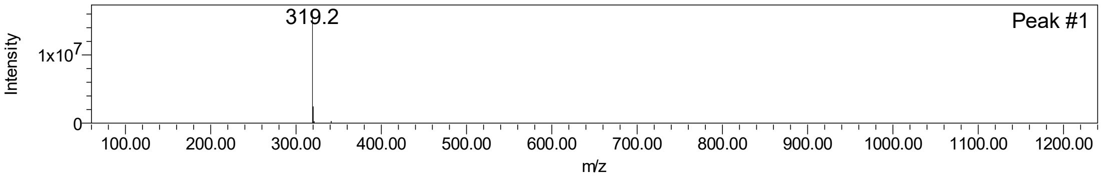

Base Peak 319.22 Channel Description 1: QDa Positive(+) Scan (60.00-1240.00)Da, Centroid, CV=10 - AVG (0.1:2.0;2.1:3.9) x 30.000 Th: 0.010 Retention Time 2.020

Match Plot

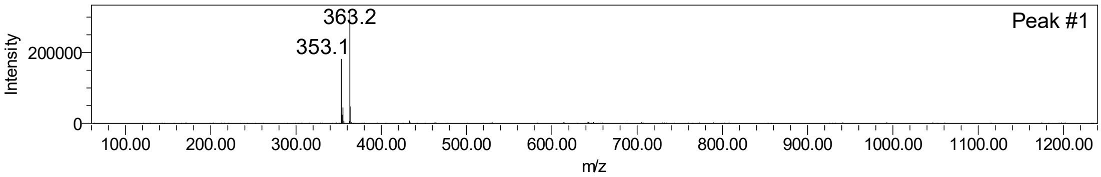

Base Peak 363.18 Channel Description 3: QDa Negative(-) Scan (60.00-1240.00)Da, Centroid, CV=10 - AVG (0.1:1.6;2.3:3.8) x 20.000 Th: 0.010 Retention Time 2.022

HPLC and LCMS Traces for Compound 20a

o2h discovery
Ahmedabad, Gujarat
India

HPLC Analysis Report

**Sample name:**	CIN-C-376-CIN-X-0052-032		
**Location:**	P2-A1	**Instrument Name**	HPLC-10
**Injection:**	1 of 1	**Acq. method:**	o2h_HPLC_Method-C.amx
**Injection volume:**	10.000	**Processing method:**	*3D UV Quantitative_DefaultMethod.pmx
**Project Name**	HPLC-10_MAY-2024		
**Date Acquired:**	2024-05-28 10:00:27+05:30	**Column:**	SUNFIRE C18 150x4.6mm,3.5um
**Date Processed:**	2024-05-28 10:27:56+05:30		

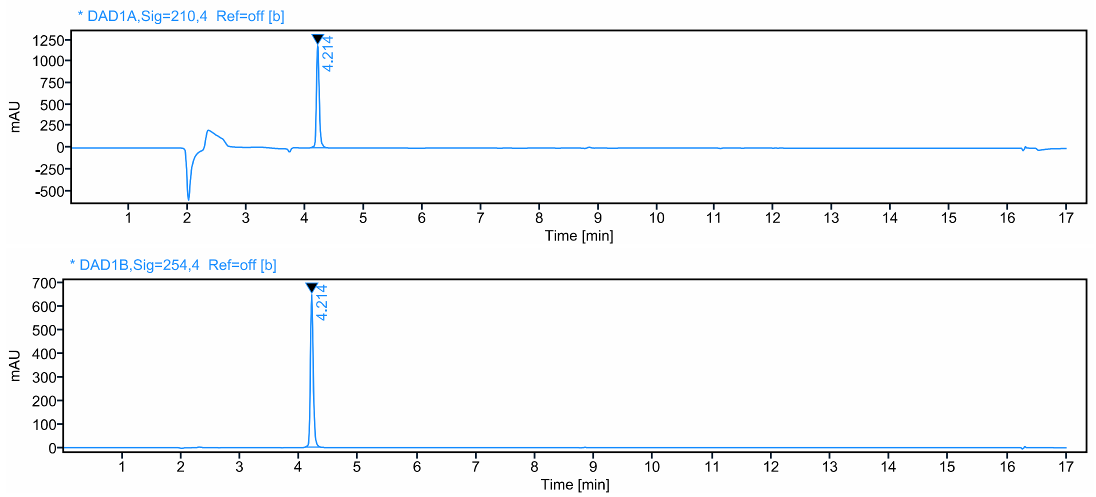

Signal: * DAD1A,Sig=210,4 Ref=off [b]

RT [min]	Height	Area	Area%
4.214	1187	4084	100.00

Signal: * DAD1B,Sig=254,4 Ref=off [b]

RT [min]	Height	Area	Area%
4.214	649	2159	100.00

HPLC and LCMS Traces for Compound 20b

o2h discovery<br>Ahmedabad, Gujarat<br>India	**LCMS Analysis Report**

Sample Name:	CIN-C-376-CIN-X-0054-050-A		Injection Id 46300
Sample Type:	Unknown	Acquired By:	LCMS-05
Vial:	1:C,6	Sample Set Name:	24062024_UCH_082_RD
Injection #:	1	Acq. Method Set:	o2h_LCMS_Method_A_SLOW_01
Injection Volume:	2.00 ul	Processing Method	O2H_LCMS_08_0,
Run Time:	4.0 Minutes	Channel Name:	MS TIC, 254.0nm, 210.0nm
Project Name:	2024\LCMS-05_JUN-2024	Proc. Chnl. Descr.:	PDA 254.0 nm Blank Subtracted
Date Acquired:	24-06-2024 12:18:25 IST		
Date Processed:	24-06-2024 12:22:28 IST, 24-06-2024 12:22:32 IST, 24-06-2024 12:23:00 IST		
Column:	X-BRIDGE C18 2.1X50mm 2.5um		

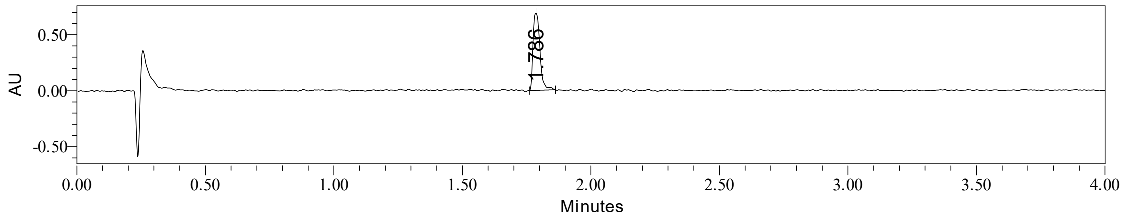

——— Channel PDA Spectrum; Channel Name 210.0nm

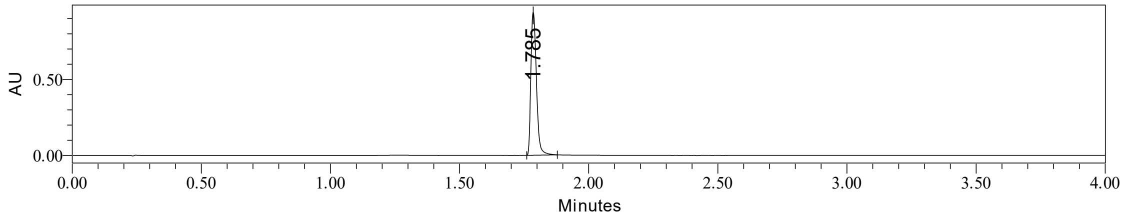

——— Channel PDA Spectrum; Channel Name 254.0nm

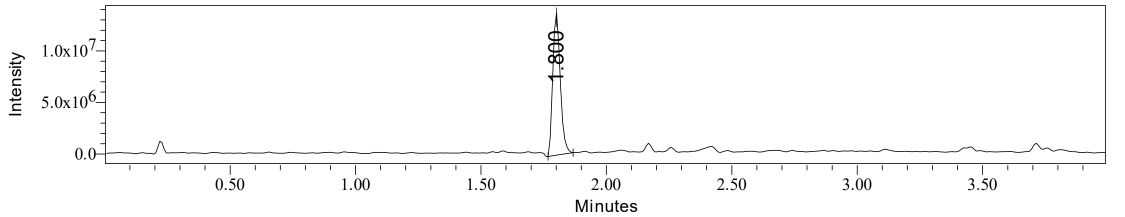

——— Channel QDa 1: MS Scan; Channel Name MS TIC

Peak Results
Channel: PDA Spectrum

	RT	Base Peak (m/z)	Height	Area	% Area	Channel	Channel Name
1	1.785		939512	1405689	100.00	PDA Spectrum	254.0nm
2	1.786		691247	1285601	100.00	PDA Spectrum	210.0nm

Peak Results
Channel: QDa 1: MS Scan

	RT	Base Peak (m/z)	Height	Area	% Area	Channel	Channel Name
1	1.800	319.24	13827592	28558012	100.00	QDa 1: MS Scan	MS TIC

HPLC and LCMS Traces for Compound 20b

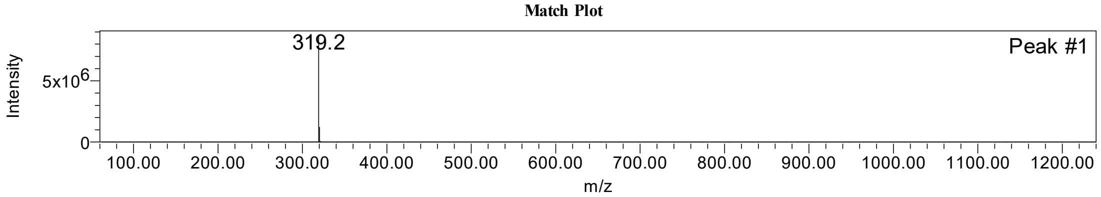

Base Peak 319.24 Channel Description 1: QDa Positive(+) Scan (60.00-1240.00)Da, Centroid, CV=10 - AVG (2.0:4.0;0.0:1.5) x 20.000 Th: 0.010 Retention Time 1.800

HPLC and LCMS Traces for Compound 20b

o2h discovery
Ahmedabad, Gujarat
India

HPLC Analysis Report

Sample name: CIN-C-376-CIN-X-0054-050-A
Location: P2-A3
Injection: 1 of 1
Injection volume: 10.000
Project Name HPLC-07_JUNE-2024
Date Acquired: 2024-06-24 12:21:49+05:30
Date Processed: 2024-06-24 12:43:37+05:30

Instrument Name HPLC-07
Acq. method: o2h_HPLC_Method-D.amx
Processing method: *3D UV Quantitative_DefaultMethod.pmx
Column: XBridge C18 150x4.6mm, 3.5um

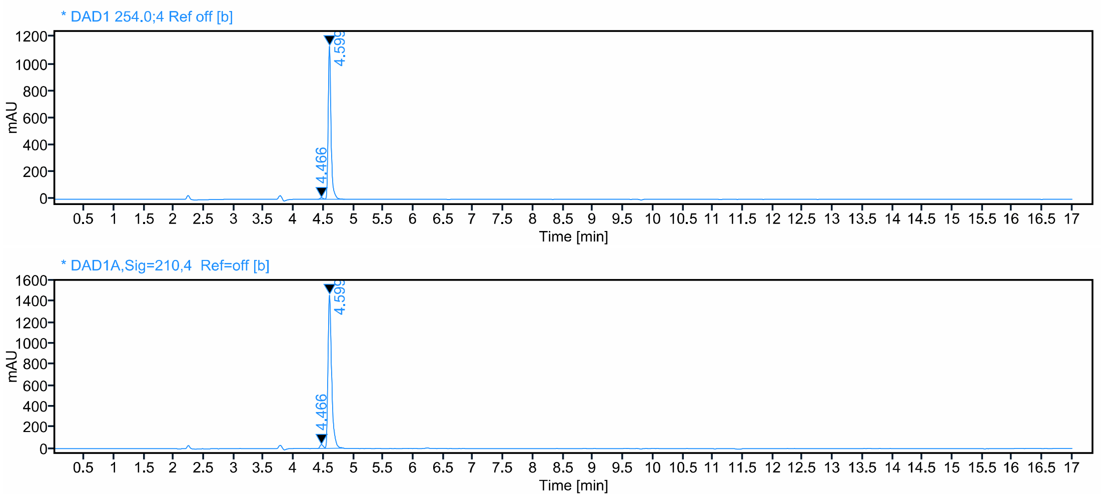

Signal: * DAD1 254.0;4 Ref off [b]

RT [min]	Height	Area	Area%
4.466	11	42	1.09
4.599	1135	3802	98.91

Signal: * DAD1A,Sig=210,4 Ref=off [b]

RT [min]	Height	Area	Area%
4.466	36	138	2.18
4.599	1461	6197	97.82

HPLC and LCMS Traces for Compound 20c

o2h discovery<br>Ahmedabad, Gujarat<br>India	**LCMS Analysis Report**

Sample Name:	CIN-C-376-CIN-X-0053-053-A		Injection Id 46310
Sample Type:	Unknown	Acquired By:	LCMS-05
Vial:	1:C,5	Sample Set Name:	24062024_UCH_082_RD
Injection #:	1	Acq. Method Set:	o2h_LCMS_Method_A_SLOW_01
Injection Volume:	2.00 ul	Processing Method	O2H_LCMS_02_0,
Run Time:	4.0 Minutes	Channel Name:	MS TIC, 254.0nm, 210.0nm
Project Name:	2024\LCMS-05_JUN-2024	Proc. Chnl. Descr.:	PDA 254.0 nm Blank Subtracted
Date Acquired:	24-06-2024 12:23:08 IST		
Date Processed:	24-06-2024 12:27:29 IST, 24-06-2024 12:27:33 IST, 24-06-2024 12:28:06 IST		
Column:	X-BRIDGE C18 2.1X50mm 2.5um		

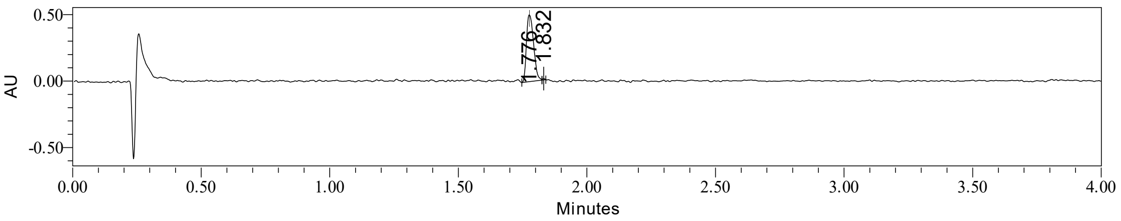

Channel PDA Spectrum; Channel Name 210.0nm

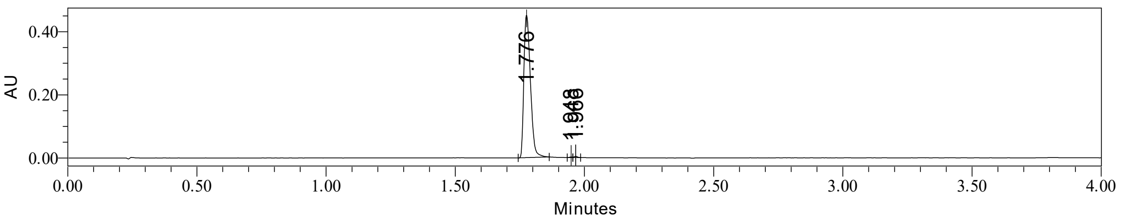

Channel PDA Spectrum; Channel Name 254.0nm

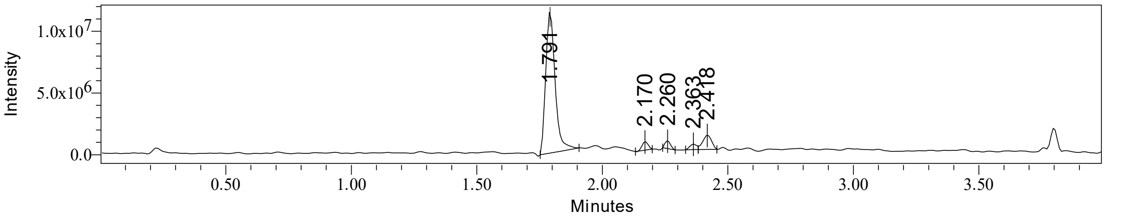

Channel QDa 1: MS Scan; Channel Name MS TIC

Peak Results
Channel: PDA Spectrum

	RT	Base Peak (m/z)	Height	Area	% Area	Channel	Channel Name
1	1.776		451315	796480	99.38	PDA Spectrum	254.0nm
2	1.776		502953	956846	99.37	PDA Spectrum	210.0nm
3	1.832		9960	6083	0.63	PDA Spectrum	210.0nm
4	1.948		1903	1404	0.18	PDA Spectrum	254.0nm
5	1.966		4122	3590	0.45	PDA Spectrum	254.0nm

HPLC and LCMS Traces for Compound 20c

Peak Results
Channel: QDa 1: MS Scan

	RT	Base Peak (m/z)	Height	Area	% Area	Channel	Channel Name
1	1.791	319.27	11290885	29759315	83.63	QDa 1: MS Scan	MS TIC
2	2.170	212.21	686026	1184649	3.33	QDa 1: MS Scan	MS TIC
3	2.260	283.11	631816	961322	2.70	QDa 1: MS Scan	MS TIC
4	2.363	212.18	441173	839522	2.36	QDa 1: MS Scan	MS TIC
5	2.418	331.35	1137724	2841788	7.99	QDa 1: MS Scan	MS TIC

Match Plot

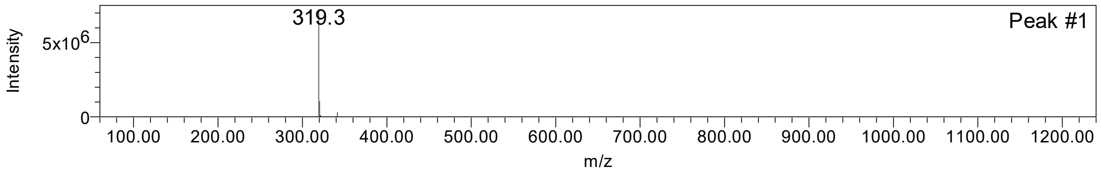

Base Peak 319.27 Channel Description 1: QDa Positive(+) Scan (60.00-1240.00)Da, Centroid, CV=10 - AVG (2.6:3.9;0.1:1.0) x 10.000 Th: 0.010 Retention Time 1.791

Match Plot

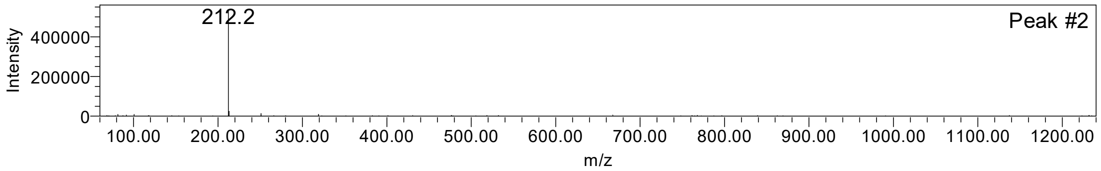

Base Peak 212.21 Channel Description 1: QDa Positive(+) Scan (60.00-1240.00)Da, Centroid, CV=10 - AVG (2.6:3.9;0.1:1.0) x 10.000 Th: 0.010 Retention Time 2.170

Match Plot

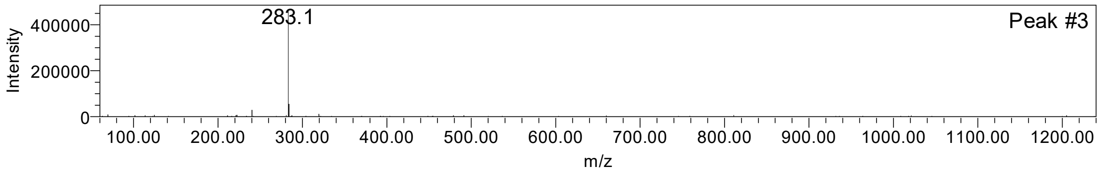

Base Peak 283.11 Channel Description 1: QDa Positive(+) Scan (60.00-1240.00)Da, Centroid, CV=10 - AVG (2.6:3.9;0.1:1.0) x 10.000 Th: 0.010 Retention Time 2.260

Match Plot

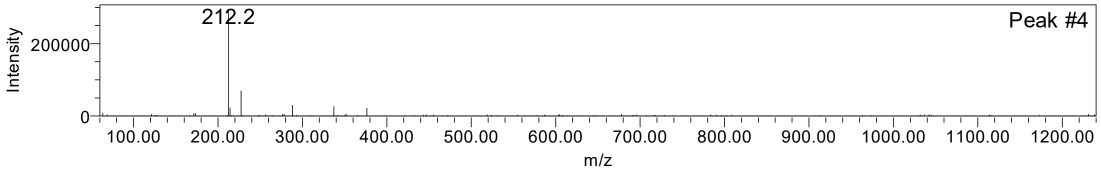

Base Peak 212.18 Channel Description 1: QDa Positive(+) Scan (60.00-1240.00)Da, Centroid, CV=10 - AVG (2.6:3.9;0.1:1.0) x 10.000 Th: 0.010 Retention Time 2.363

HPLC and LCMS Traces for Compound 20c

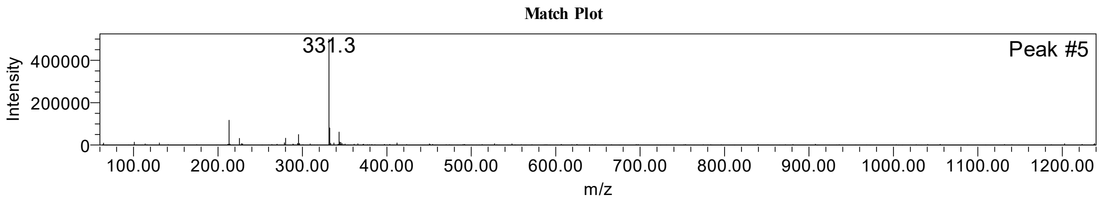

Base Peak 331.35 Channel Description 1: QDa Positive(+) Scan (60.00-1240.00)Da, Centroid, CV=10 - AVG (2.6:3.9;0.1:1.0) x 10.000 Th: 0.010 Retention Time 2.418

HPLC and LCMS Traces for Compound 20c

o2h discovery<br>Ahmedabad, Gujarat<br>India	**HPLC Analysis Report**

Sample name: CIN-C-376-CIN-X-0053-053-A
Location: P2-A4
Injection: 1 of 1
Injection volume: 10.000
Project Name HPLC-07_JUNE-2024
Date Acquired: 2024-06-24 12:40:47+05:30
Date Processed: 2024-06-24 13:06:42+05:30

Instrument Name HPLC-07
Acq. method: o2h_HPLC_Method-D.amx
Processing method: *3D UV Quantitative_DefaultMethod.pmx
Column: XBridge C18 150x4.6mm, 3.5um

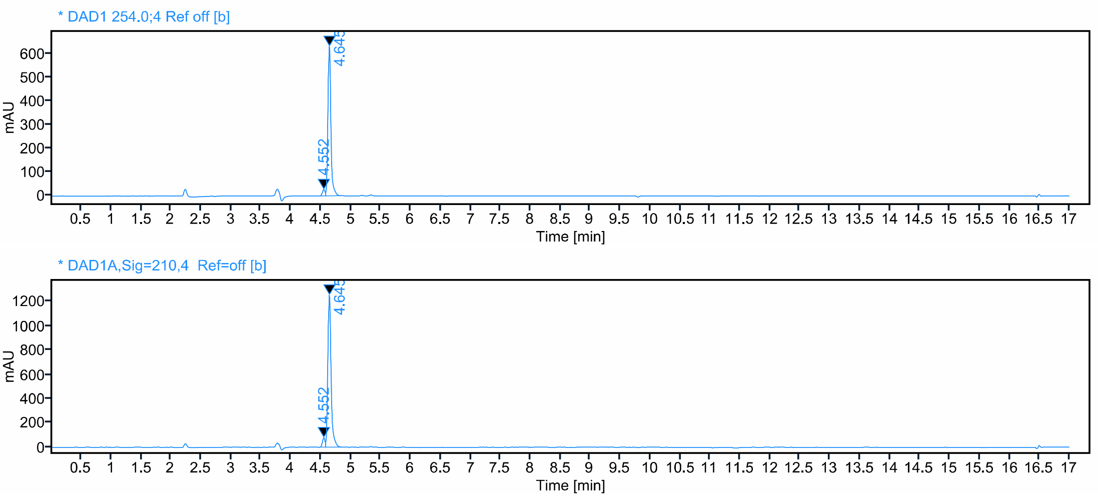

Signal: * DAD1 254.0;4 Ref off [b]

RT [min]	Height	Area	Area%
4.552	27	75	3.39
4.645	629	2128	96.61

Signal: * DAD1A,Sig=210,4 Ref=off [b]

RT [min]	Height	Area	Area%
4.552	77	231	4.74
4.645	1250	4641	95.26

HPLC and LCMS Traces for Compound 21a

o2h discovery<br>Ahmedabad, Gujarat<br>India	**LCMS Analysis Report**

Sample Name:	CIN-C-377-CIN-X-0040-043-a		Injection Id 46975
Sample Type:	Unknown	Acquired By:	LCMS-05
Vial:	1:B,6	Sample Set Name:	27052024_UCH_082_FD
Injection #:	1	Acq. Method Set:	o2h_LCMS_Method_A
Injection Volume:	2.00 ul	Processing Method:	O2H_LCMS_01,
Run Time:	4.0 Minutes	Channel Name:	220.0nm, MS TIC, 254.0nm
Project Name:	2024\LCMS-05_MAY-2024	Proc. Chnl. Descr.:	PDA 220.0 nm Blank Subtracted
Date Acquired:	27-05-2024 11:00:14 IST		
Date Processed:	27-05-2024 11:06:39 IST, 27-05-2024 11:06:46 IST, 27-05-2024 11:07:31 IST, 27-05-2024		
Column:	X-BRIDGE C18 2.1X50mm 2.5um		

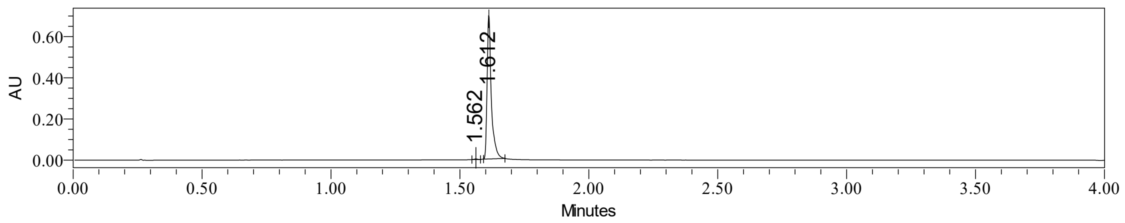

Channel Name 254.0nm; Channel PDA Spectrum

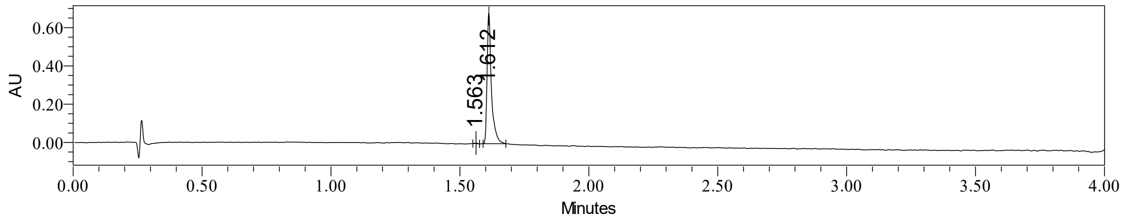

Channel Name 220.0nm; Channel PDA Spectrum

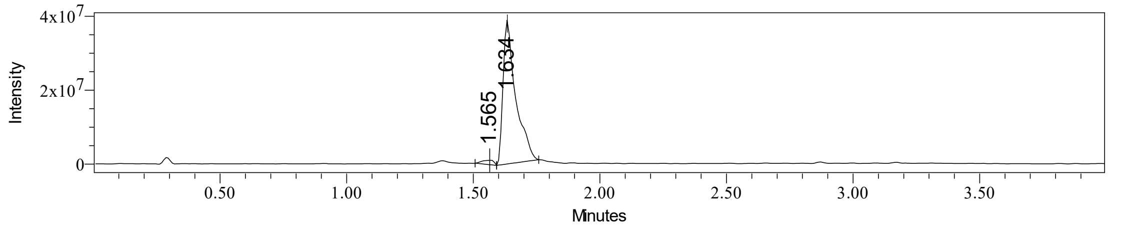

Channel Name MS TIC; Channel QDa 1: MS Scan

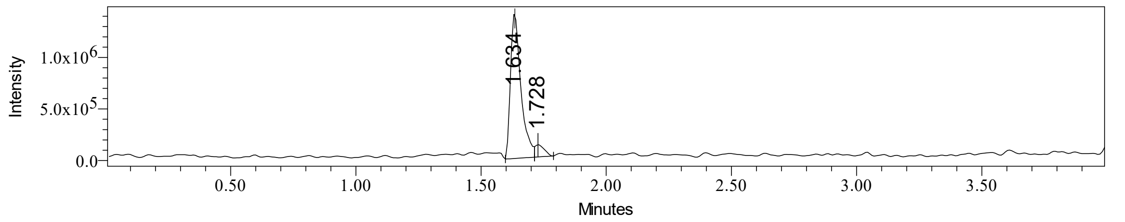

Channel Name MS TIC; Channel QDa 3: MS Scan

HPLC and LCMS Traces for Compound 21a

Peak Results
Channel: PDA Spectrum

	RT	Base Peak (m/z)	Height	Area	% Area	Channel	Channel Name
1	1.562		2768	2374	0.30	PDA Spectrum	254.0nm
2	1.563		2900	2351	0.29	PDA Spectrum	220.0nm
3	1.612		698472	789360	99.70	PDA Spectrum	254.0nm
4	1.612		684051	797565	99.71	PDA Spectrum	220.0nm

Peak Results
Channel: QDa 1: MS Scan

	RT	Base Peak (m/z)	Height	Area	% Area	Channel	Channel Name
1	1.565	218.23	1248312	3824054	2.75	QDa 1: MS Scan	MS TIC
2	1.634	386.39	38966988	135083194	97.25	QDa 1: MS Scan	MS TIC

Peak Results
Channel: QDa 3: MS Scan

	RT	Base Peak (m/z)	Height	Area	% Area	Channel	Channel Name
1	1.634	430.25	1405411	4075882	93.26	QDa 3: MS Scan	MS TIC
2	1.728	430.15	116808	294647	6.74	QDa 3: MS Scan	MS TIC

Match Plot

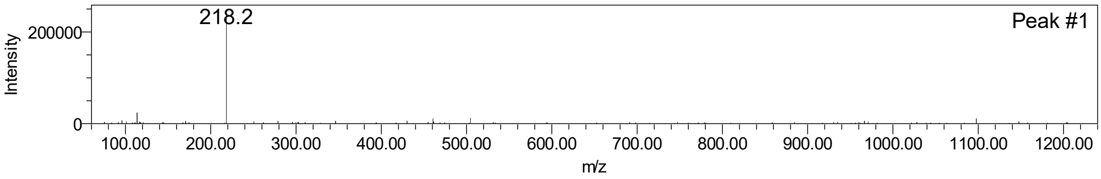

Base Peak 218.23 Channel Description 1: QDa Positive(+) Scan (60.00-1250.00)Da, Centroid, CV=10 - AVG (0.0:1.3;2.0:4.0) x 30.000 Th: 0.010 Retention Time 1.565

Match Plot

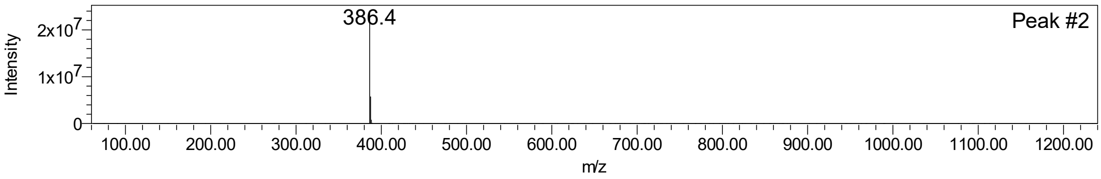

Base Peak 386.39 Channel Description 1: QDa Positive(+) Scan (60.00-1250.00)Da, Centroid, CV=10 - AVG (0.0:1.3;2.0:4.0) x 30.000 Th: 0.010 Retention Time 1.634

Match Plot

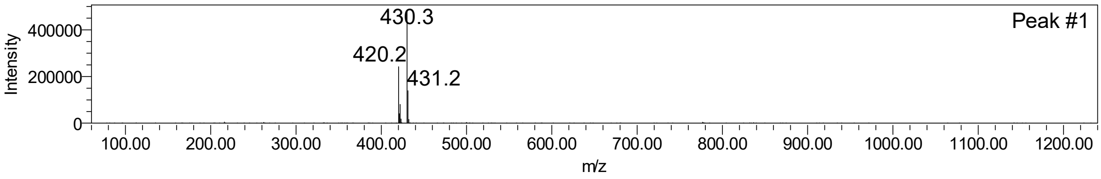

Base Peak 430.25 Channel Description 3: QDa Negative(-) Scan (60.00-1250.00)Da, Centroid, CV=10 - AVG (0.1:1.2;1.8:3.4) x 20.000 Th: 0.010 Retention Time 1.634

HPLC and LCMS Traces for Compound 21a

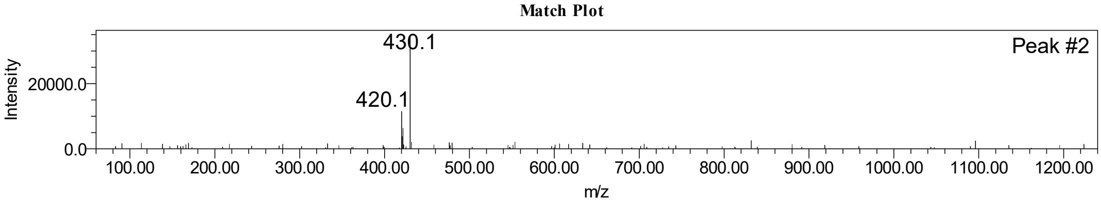

Base Peak 430.15 Channel Description 3: QDa Negative(-) Scan (60.00-1250.00)Da, Centroid, CV=10 - AVG (0.1:1.2;1.8:3.4) x 20.000 Th: 0.010 Retention Time 1.728

HPLC and LCMS Traces for Compound 21a

o2h discovery
Ahmedabad, Gujarat
India

HPLC Analysis Report

Sample name: CIN-C-377-CIN-X-0040-043-a
Location: P1-A9
Injection: 1 of 1
Injection volume: 10.000
Project Name HPLC-10_MAY-2024
Date Acquired: 2024-05-27 11:32:20+05:30
Date Processed: 2024-05-27 11:53:05+05:30

Instrument Name HPLC-10
Acq. method: o2h_HPLC_Method-A.amx
Processing method: 3D UV Quantitative_DefaultMethod.pmx
Column: SUNFIRE C18 150x4.6mm,3.5um

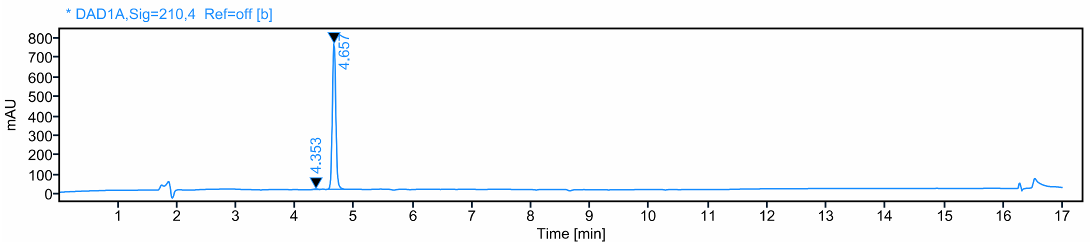

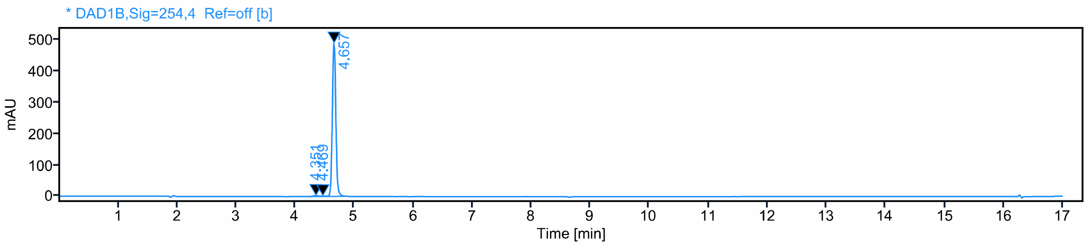

Signal: * DAD1A,Sig=210,4 Ref=off [b]

RT [min]	Height	Area	Area%
4.353	3	14	0.46
4.657	745	3046	99.54

Signal: * DAD1B,Sig=254,4 Ref=off [b]

RT [min]	Height	Area	Area%
4.351	2	13	0.66
4.469	2	7	0.37
4.657	491	2006	98.97

HPLC and LCMS Traces for Compound 22a

Method1

SAMPLE INFORMATION			
Sample Name:	CNA-79	Acquired By:	BillyGroup
Sample Type:	Unknown	Sample Set Name:	NA_28112025
Vial:	1:F,1	Acq. Method Set:	HPLC_NA
Injection #:	1	Processing Method:	Huso
Injection Volume:	10.00 ul	Channel Name:	220.0nm@1
Run Time:	25.0 Minutes	Proc. Chnl. Descr.:	2998 PDA 220.0 nm (2998
Date Acquired:	28/11/2025 1:04:44 PM HKT		
Date Processed:	4/12/2025 10:50:01 AM HKT		

Auto-Scaled Chromatogram

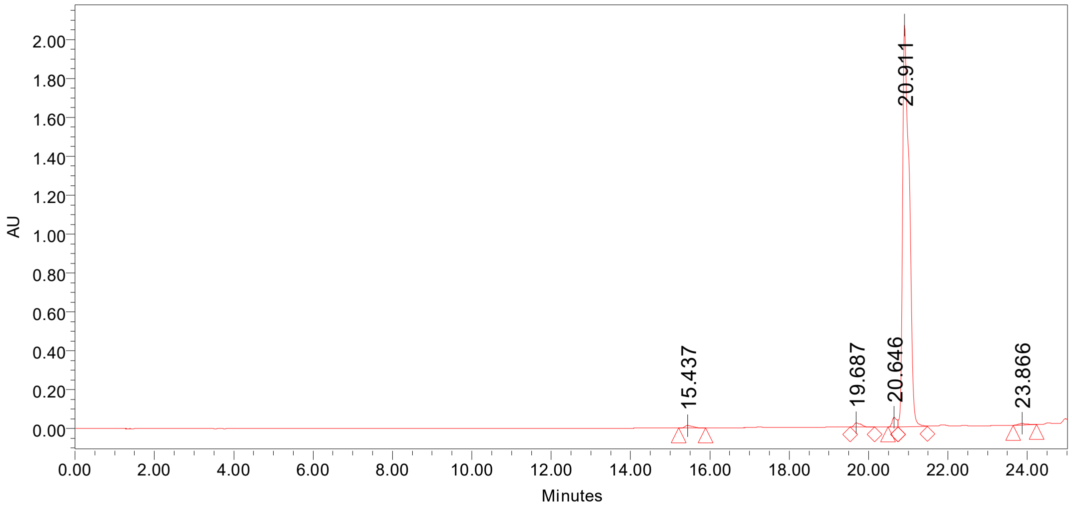

Peak Results

	Retention Time (min)	% Area	Sample Set Name	Channel Name	% Height
1	15.437	0.69	NA_28112025	220.0nm@1	0.59
2	19.687	1.13	NA_28112025	220.0nm@1	1.00
3	20.646	1.61	NA_28112025	220.0nm@1	2.28
4	20.911	96.10	NA_28112025	220.0nm@1	95.77
5	23.866	0.46	NA_28112025	220.0nm@1	0.36

Reported by User: BillyGroup (BillyGroup)
Report Method: Method1
Report Method ID: 2308
Page: 1 of 1

Project Name: Nucleoside Analogues
Date Printed:
4/12/2025
11:00:23 am Asia/Hong_Kong

HPLC and LCMS Traces for Compound 22a

Method1

SAMPLE INFORMATION

Sample Name:	CNA-79	Acquired By:	BillyGroup
Sample Type:	Unknown	Sample Set Name:	NA_28112025
Vial:	1:F,1	Acq. Method Set:	HPLC_NA
Injection #:	1	Processing Method:	Huso
Injection Volume:	10.00 ul	Channel Name:	254.0nm@1
Run Time:	25.0 Minutes	Proc. Chnl. Descr.:	2998 PDA 254.0 nm (2998
Date Acquired:	28/11/2025 1:04:44 PM HKT		
Date Processed:	4/12/2025 10:48:56 AM HKT		

Auto-Scaled Chromatogram

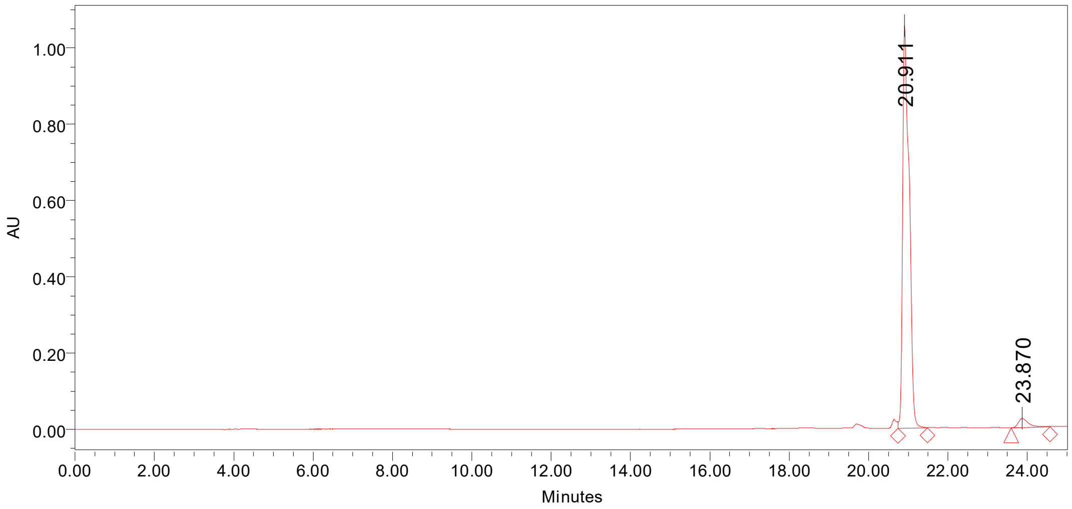

Peak Results

	Retention Time (min)	% Area	Sample Set Name	Channel Name	% Height
1	20.911	96.60	NA_28112025	254.0nm@1	97.79
2	23.870	3.40	NA_28112025	254.0nm@1	2.21

Reported by User: BillyGroup (BillyGroup)
Report Method: Method1
Report Method ID: 2308
Page: 1 of 1

Project Name: Nucleoside Analogues
Date Printed:
4/12/2025
11:00:54 am Asia/Hong_Kong

HPLC and LCMS Traces for Compound 23a

Method1

SAMPLE INFORMATION

Sample Name:	CNA-82	Acquired By:	BillyGroup
Sample Type:	Unknown	Sample Set Name:	NA_28112025
Vial:	1:F,3	Acq. Method Set:	HPLC_NA
Injection #:	1	Processing Method:	Huso
Injection Volume:	3.00 ul	Channel Name:	210.0nm
Run Time:	25.0 Minutes	Proc. Chnl. Descr.:	2998 PDA 210.0 nm (2998
Date Acquired:	28/11/2025 3:08:11 PM HKT		
Date Processed:	4/12/2025 11:12:24 AM HKT		

Auto-Scaled Chromatogram

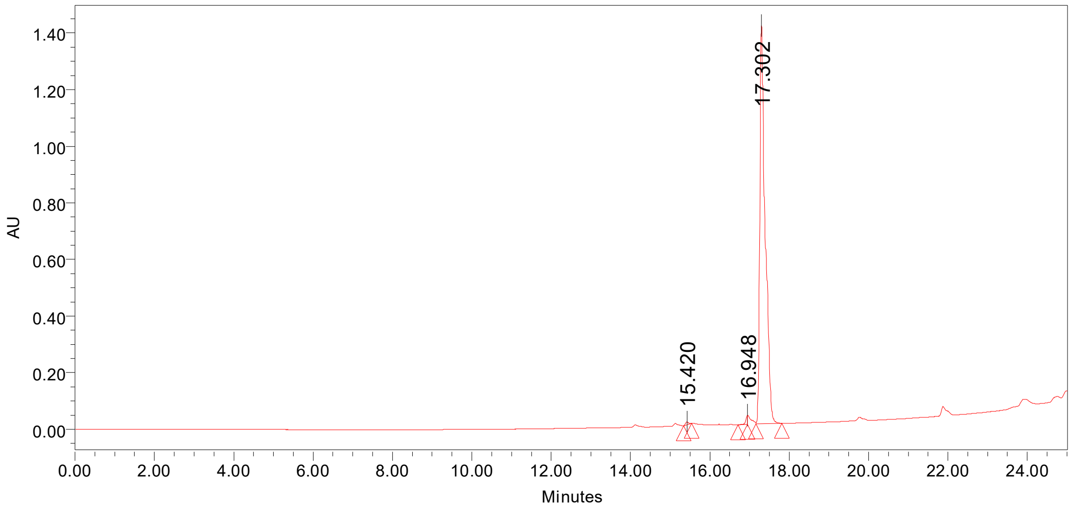

Peak Results

	Retention Time (min)	% Area	Sample Set Name	Channel Name	% Height
1	15.420	0.36	NA_28112025	210.0nm	0.66
2	16.948	0.68	NA_28112025	210.0nm	2.21
3	17.302	98.96	NA_28112025	210.0nm	97.13

Reported by User: BillyGroup (BillyGroup)
Report Method: Method1
Report Method ID: 2308
Page: 1 of 1

Project Name: Nucleoside Analogues
Date Printed:
4/12/2025
11:13:16 am Asia/Hong_Kong

HPLC and LCMS Traces for Compound 23a

Method1

SAMPLE INFORMATION

Sample Name:	CNA-82	Acquired By:	BillyGroup
Sample Type:	Unknown	Sample Set Name:	NA_28112025
Vial:	1:F,3	Acq. Method Set:	HPLC_NA
Injection #:	1	Processing Method:	Huso
Injection Volume:	3.00 ul	Channel Name:	254.0nm@2
Run Time:	25.0 Minutes	Proc. Chnl. Descr.:	2998 PDA 254.0 nm (2998
Date Acquired:	28/11/2025 3:08:11 PM HKT		
Date Processed:	4/12/2025 11:13:07 AM HKT		

Auto-Scaled Chromatogram

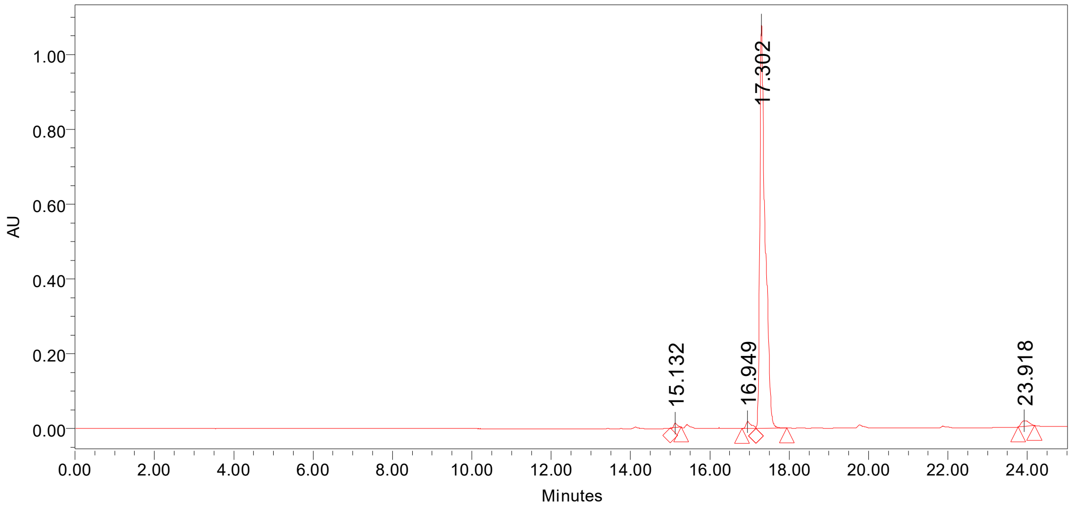

Peak Results

	Retention Time (min)	% Area	Sample Set Name	Channel Name	% Height
1	15.132	0.71	NA_28112025	254.0nm@2	1.05
2	16.949	1.59	NA_28112025	254.0nm@2	1.62
3	17.302	95.74	NA_28112025	254.0nm@2	95.99
4	23.918	1.96	NA_28112025	254.0nm@2	1.34

Reported by User: BillyGroup (BillyGroup)
Report Method: Method1
Report Method ID: 2308
Page: 1 of 1

Project Name: Nucleoside Analogues
Date Printed:
4/12/2025
11:15:25 am Asia/Hong_Kong

HPLC and LCMS Traces for Compound 23d

o2h discovery
Ahmedabad, Gujarat
India

LCMS Analysis Report

Sample Name:	CIN2-D-051-CIN2-X-0072-017-A		Injection Id 43419
Sample Type:	Unknown	Acquired By:	LCMS-05
Vial:	1:C,5	Sample Set Name:	19122024_UCH_NEW_FD
Injection #:	1	Acq. Method Set:	o2h_LCMS_Method_A
Injection Volume:	2.00 ul	Processing Method	O2H_LCMS_02,
Run Time:	4.0 Minutes	Channel Name:	MS TIC, 254.0nm, 210.0nm
Project Name:	2024\LCMS-05_DEC-2024_	Proc. Chnl. Descr.:	PDA 254.0 nm Blank Subtracted
Date Acquired:	19-12-2024 10:23:44 IST		
Date Processed:	19-12-2024 10:32:14 IST, 19-12-2024 10:33:05 IST, 19-12-2024 10:33:48 IST, 19-12-2024		
Column:	X-BRIDGE C18 2.1X50mm 2.5um		

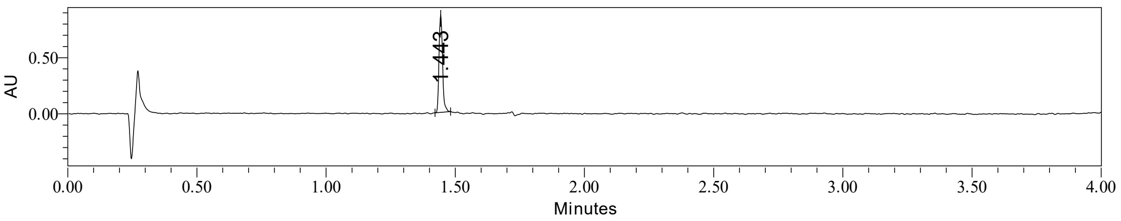

Channel Name 210.0nm; Channel PDA Spectrum

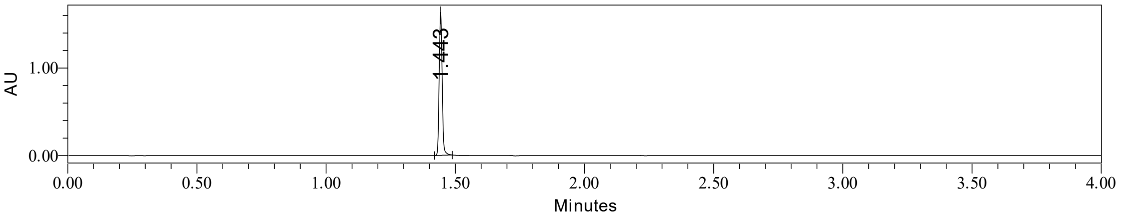

Channel Name 254.0nm; Channel PDA Spectrum

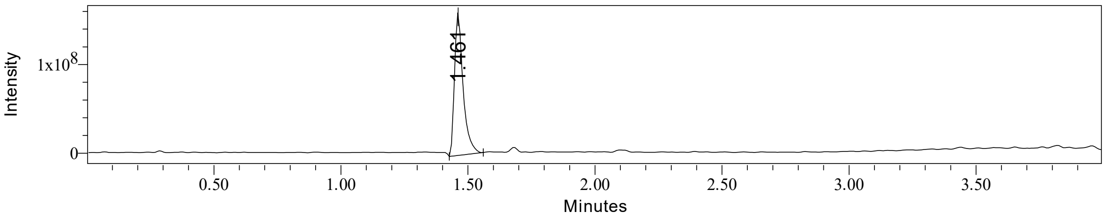

Channel Name MS TIC; Channel QDa 1: MS Scan

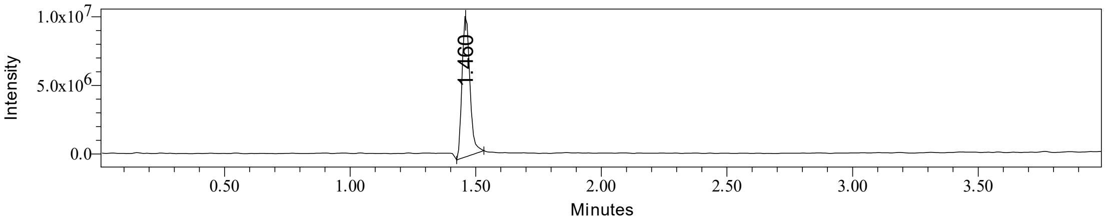

Channel Name MS TIC; Channel QDa 3: MS Scan

HPLC and LCMS Traces for Compound 23d

Peak Results
Channel: PDA Spectrum

	RT	Base Peak (m/z)	Height	Area	% Area	Channel	Channel Name
1	1.443		1628017	1299133	100.00	PDA Spectrum	254.0nm
2	1.443		867096	821478	100.00	PDA Spectrum	210.0nm

Peak Results
Channel: QDa 1: MS Scan

	RT	Base Peak (m/z)	Height	Area	% Area	Channel	Channel Name
1	1.461	319.06	161151557	376945697	100.00	QDa 1: MS Scan	MS TIC

Peak Results
Channel: QDa 3: MS Scan

	RT	Base Peak (m/z)	Height	Area	% Area	Channel	Channel Name
1	1.460	363.13	10421355	22200834	100.00	QDa 3: MS Scan	MS TIC

Match Plot

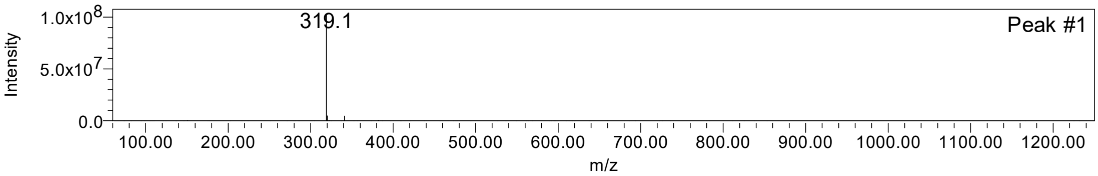

Base Peak 319.06 Channel Description 1: QDa Positive(+) Scan (60.00-1250.00)Da, Centroid, CV=10 - AVG (0.2:1.1;1.7:4.0) x 20.000 Th: 0.010 Retention Time 1.461

Match Plot

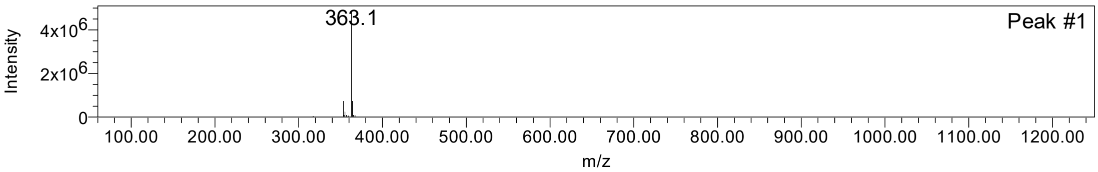

Base Peak 363.13 Channel Description 3: QDa Negative(-) Scan (60.00-1250.00)Da, Centroid, CV=10 - AVG (0.1:0.8;2.1:3.9) x 20.000 Th: 0.010 Retention Time 1.460

HPLC and LCMS Traces for Compound 23d

o2h discovery
Ahmedabad, Gujarat
India

HPLC Analysis Report

Sample name: CIN2-D-051-CIN2-X-0072-017-A

Location: P2-A6

Injection: 1 of 1

Injection volume: 10.000

Project Name HPLC-07_DEC-2024

Date Acquired: 2024-12-19 11:20:00+05:30

Date Processed: 2024-12-19 11:40:20+05:30

Instrument Name HPLC-07

Acq. method: o2h_HPLC_Method-A.amx

Processing method: *3D UV Quantitative_DefaultMethod.pmx

Column: SUNFIRE C18 150x4.6mm 3.5um

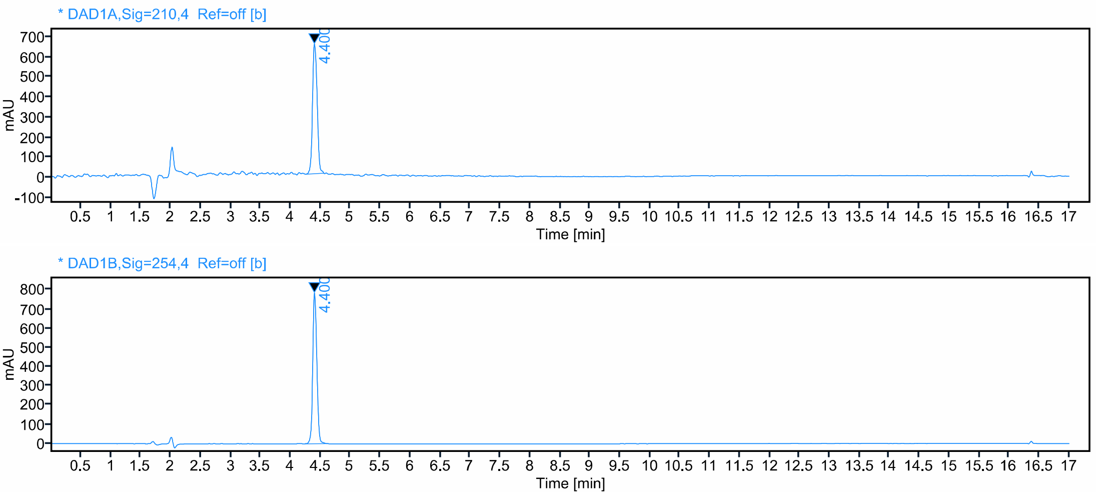

Signal: * DAD1A,Sig=210,4 Ref=off [b]

RT [min]	Height	Area	Area%
4.400	647	3474	100.00

Signal: * DAD1B,Sig=254,4 Ref=off [b]

RT [min]	Height	Area	Area%
4.400	786	3715	100.00

HPLC and LCMS Traces for Compound 23e

o2h discovery<br>Ahmedabad, Gujarat<br>India	LCMS Analysis Report

Sample Name:	CIN2-C-818-CIN2-X-0073-090		Injection Id 62914
Sample Type:	Unknown	Acquired By:	LCMS-05
Vial:	1:C,7	Sample Set Name:	28112024_UCH_090_FD
Injection #:	1	Acq. Method Set:	o2h_LCMS_Method_A
Injection Volume:	2.00 ul	Processing Method	MASS_000, O2H_LCMS_02_00
Run Time:	4.0 Minutes	Channel Name:	MS TIC, 254.0nm, 210.0nm
Project Name:	2024\LCMS-05_NOV-2024	Proc. Chnl. Descr.:	PDA 254.0 nm Blank Subtracted
Date Acquired:	28-11-2024 10:04:00 IST		
Date Processed:	28-11-2024 10:46:51 IST, 28-11-2024 10:46:57 IST, 28-11-2024 10:47:41 IST		
Column:	X-BRIDGE C18 2.1X50mm 2.5um		

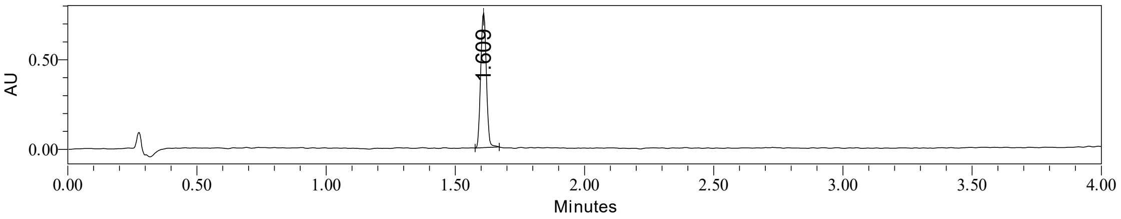

Channel Name 210.0nm; Channel PDA Spectrum

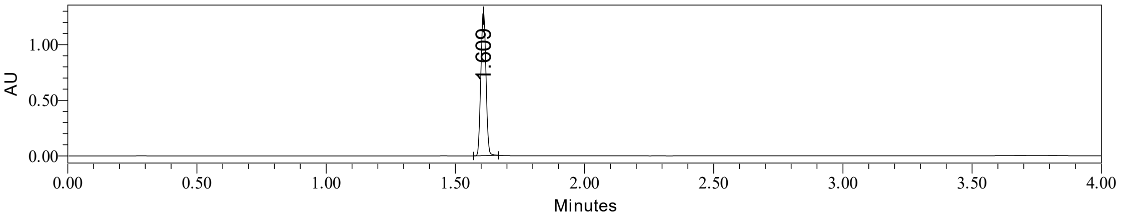

Channel Name 254.0nm; Channel PDA Spectrum

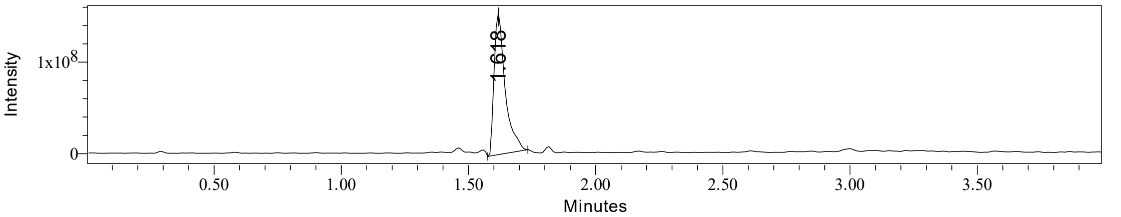

Channel Name MS TIC; Channel QDa 1: MS Scan

Peak Results
Channel: PDA Spectrum

	RT	Base Peak (m/z)	Height	Area	% Area	Channel	Channel Name
1	1.609		1295070	1768588	100.00	PDA Spectrum	254.0nm
2	1.609		753792	1134666	100.00	PDA Spectrum	210.0nm

Peak Results
Channel: QDa 1: MS Scan

	RT	Base Peak (m/z)	Height	Area	% Area	Channel	Channel Name
1	1.618	303.04	153904767	466578681	100.00	QDa 1: MS Scan	MS TIC

HPLC and LCMS Traces for Compound 23e

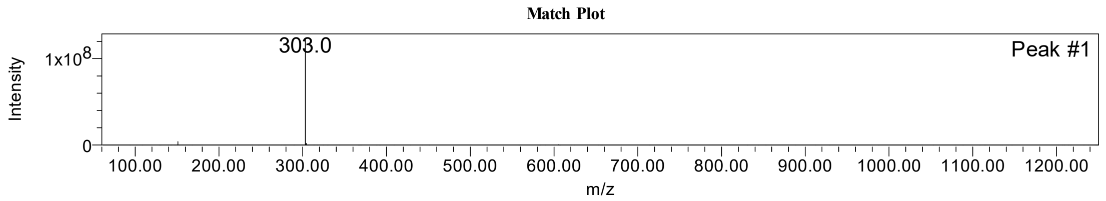

Base Peak 303.04 Channel Description 1: QDa Positive(+) Scan (90.00-1250.00)Da, Centroid, CV=10 - AVG (1.9:4.0;0.1:1.2) x 10.000 Th: 0.010 Retention Time 1.618

HPLC and LCMS Traces for Compound 23e

o2h discovery
Ahmedabad, Gujarat
India

HPLC Analysis Report

**Sample name:**	CIN2-C-818-CIN2-X-0073-090		
**Location:**	P1-A5	**Instrument Name**	HPLC-10
**Injection:**	1 of 1	**Acq. method:**	o2h_HPLC_Method-A.amx
**Injection volume:**	10.000	**Processing method:**	3D UV Quantitative_DefaultMethod.pmx
**Project Name**	HPLC-10-NOV-2024		
**Date Acquired:**	2024-11-28 10:53:05+05:30	**Column:**	SUNFIRE C18 150x4.6mm,3.5um
**Date Processed:**	2024-11-28 11:41:26+05:30		

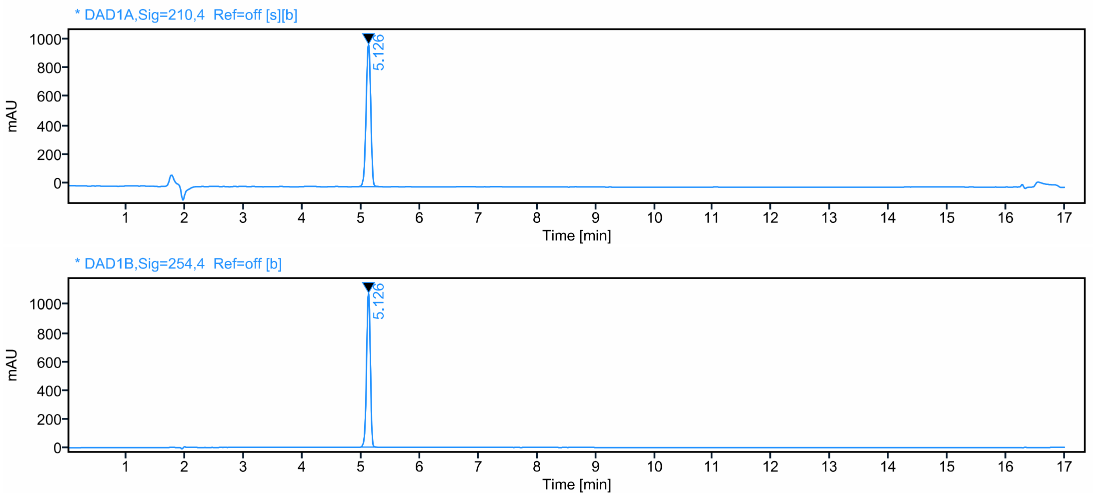

Signal: * DAD1A,Sig=210,4 Ref=off [s][b]

RT [min]	Height	Area	Area%
5.126	980	5207	100.00

Signal: * DAD1B,Sig=254,4 Ref=off [b]

RT [min]	Height	Area	Area%
5.126	1074	4658	100.00

NMR Spectra For Carbobicyclic Nucleoside Analogues

NMR-Spectra for Compound 2d

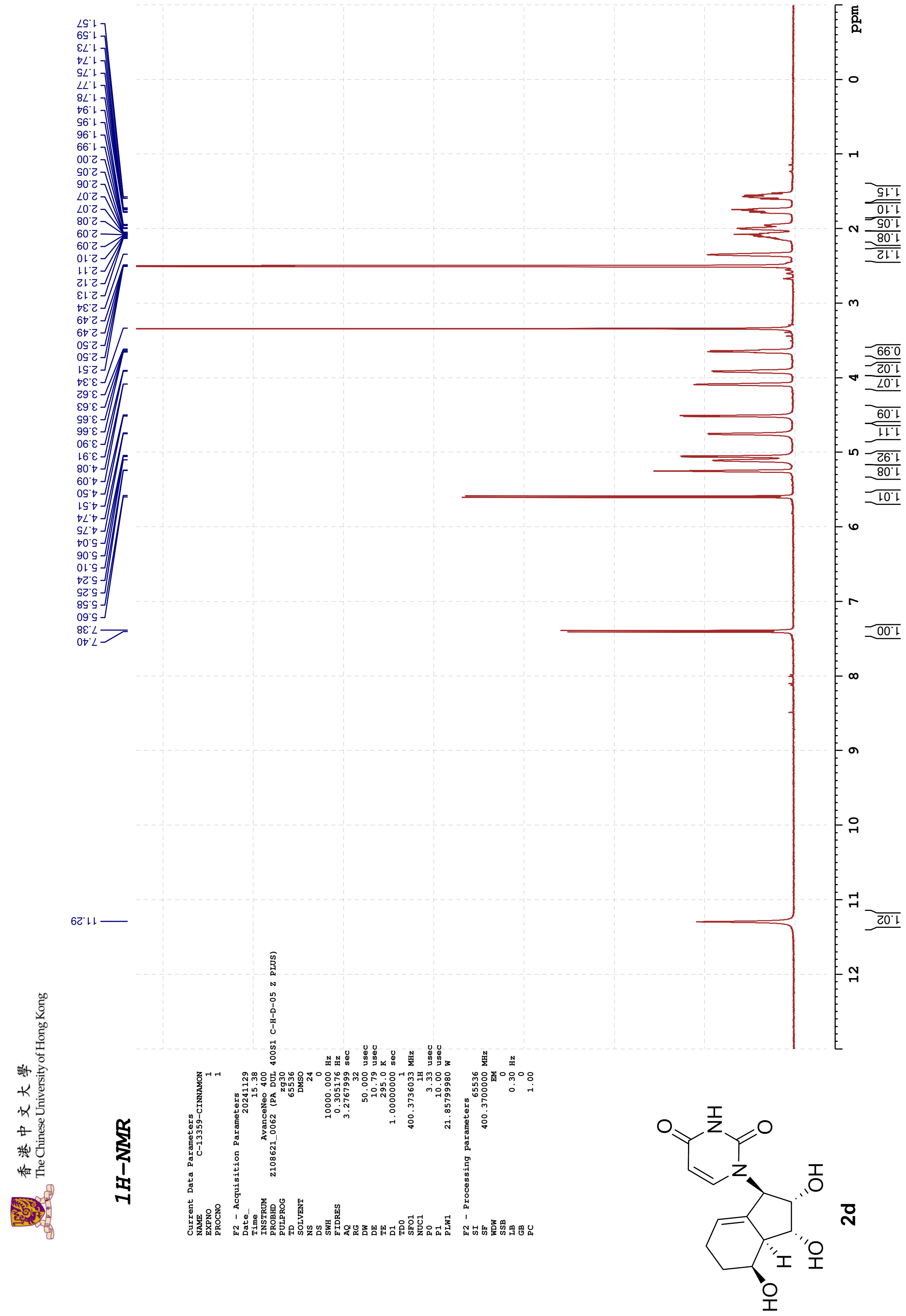

NMR-Spectra for Compound 2d

The Chinese University of Hong Kong 香港中文大學

13C{1H}-NMR

```
Current Data Parameters
NAME          C-13500-CINNAMON
EXPNO                        1
PROCNO                       1

F2 - Acquisition Parameters
Date_                 20241130
Time                      8.12
INSTRUM                 Avance
PROBHD      Z163739_1043 (PI HR-BBO400S1-BBF/H/D-5.0-Z SP)
PULPROG                zgpg30
TD                       65536
SOLVENT                   DMSO
NS                        1024
DS                           0
SWH                  32679.739 Hz
FIDRES                0.997306 Hz
AQ                   1.0027008 sec
RG                        12.7
DW                      15.300 usec
DE                        6.50 usec
TE                       294.2 K
D1                  2.00000000 sec
D11                 0.03000000 sec
TD0                          1
SFO1               100.7490748 MHz
NUC1                       13C
P0                        2.67 usec
P1                        8.00 usec
PLW1               96.23899841 W
SFO2               400.6216025 MHz
NUC2                        1H
CPDPRG2                waltz65
PCPD2                    90.00 usec
PLW2               21.54100037 W
PLW12               0.17020001 W
PLW13               0.08560800 W

F2 - Processing parameters
SI                       32768
SF                 100.7359791 MHz
WDW                         EM
SSB                          0
LB                        1.00 Hz
GB                           0
PC                        1.40
```

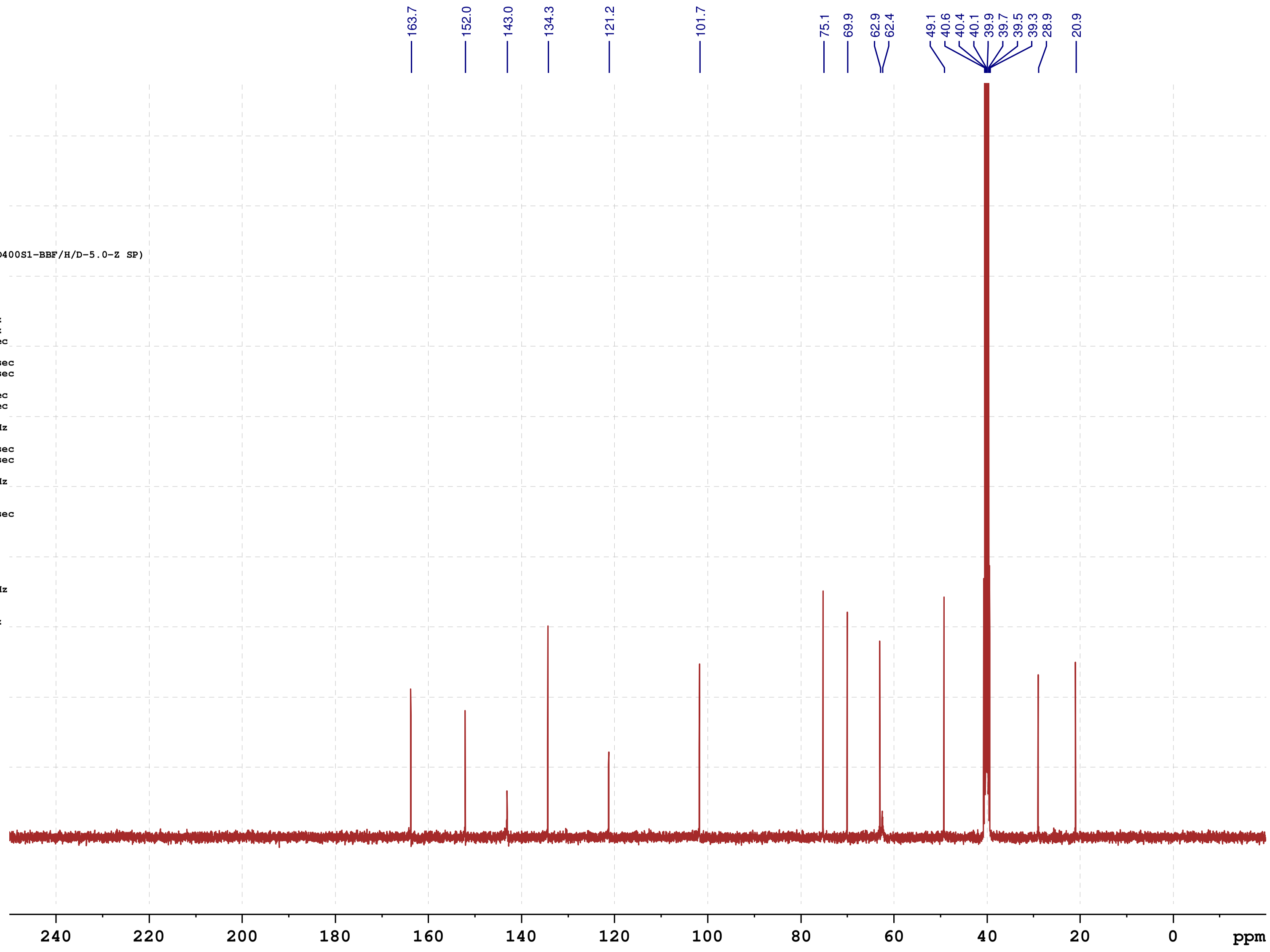

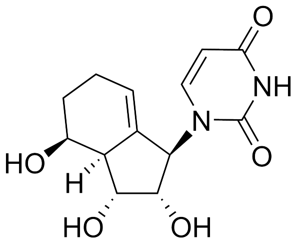

2d

NMR-Spectra for Compound 2e

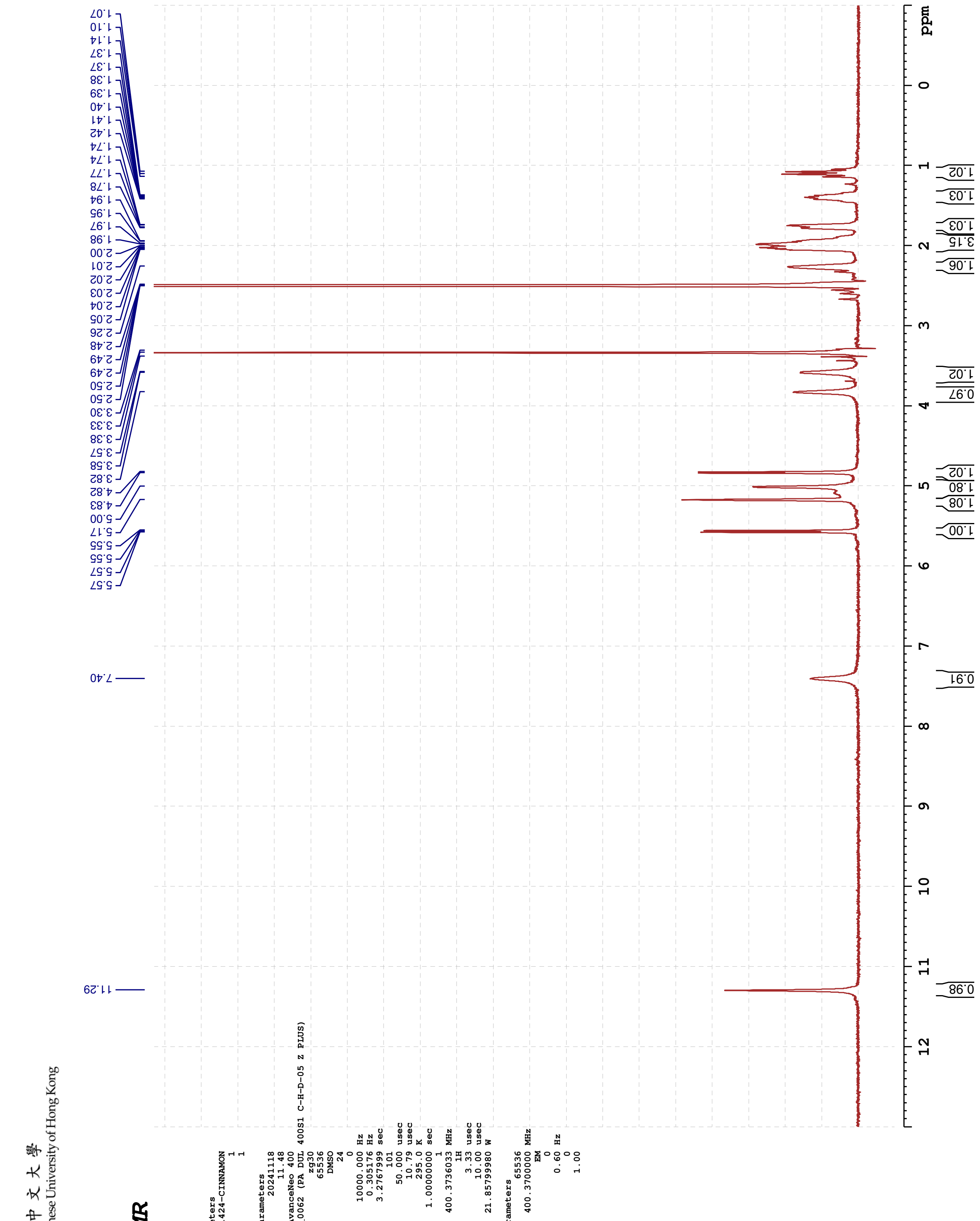

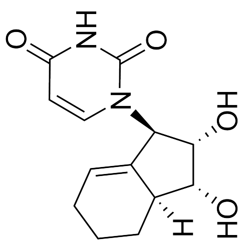

2e

NMR-Spectra for Compound 2e

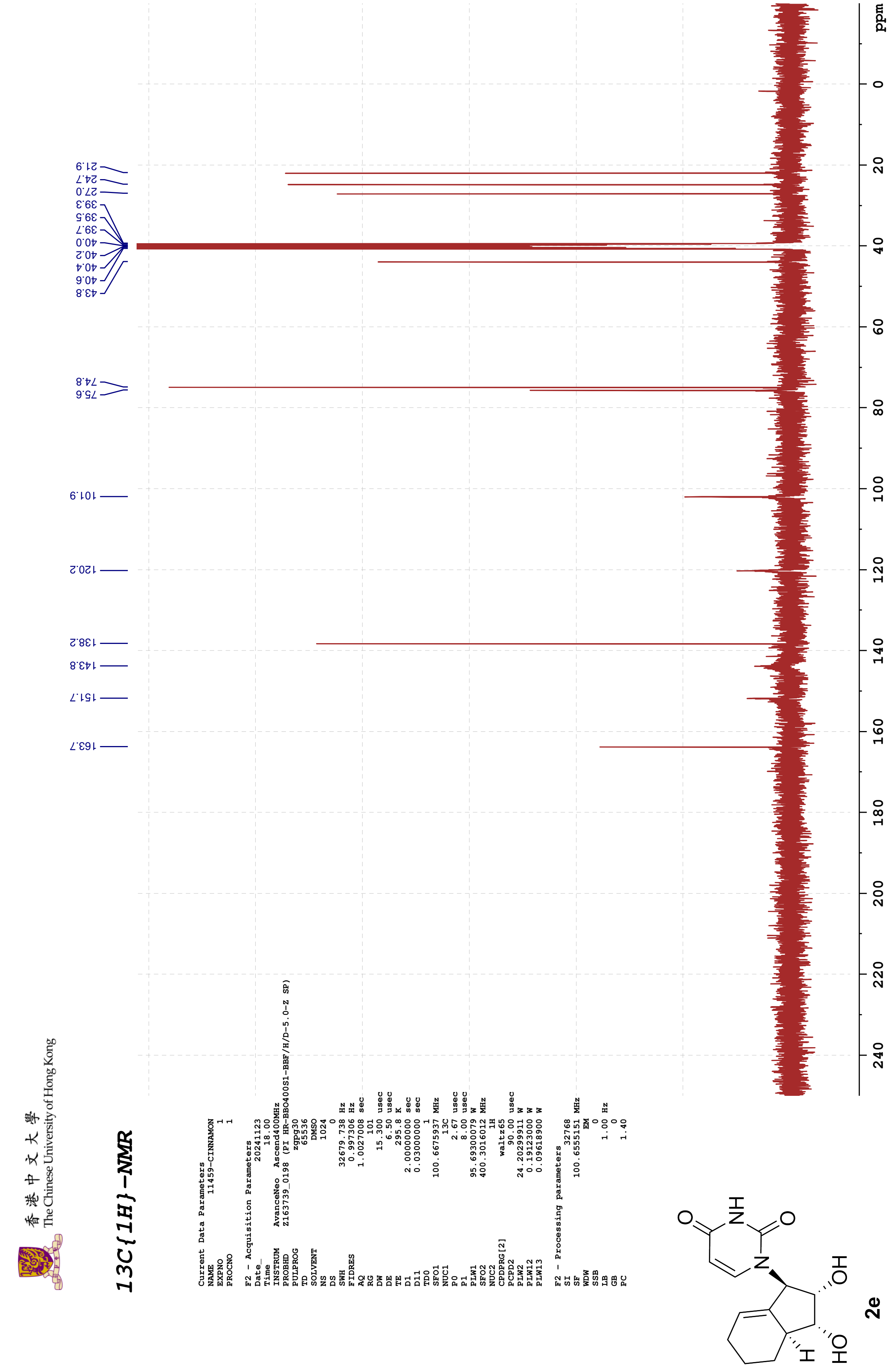

2e

NMR-Spectra for Compound 2f

香港中文大學
The Chinese University of Hong Kong

1H-NMR

```
Current Data Parameters
NAME          C-8567-CINNAMON
EXPNO                       1
PROCNO                      1

F2 - Acquisition Parameters
Date_                20241021
Time                    18.11
INSTRUM                Avance
PROBHD      Z163739_0989 (PI HR-BBO400S1-BBF/H/D-5.0-Z SP)
PULPROG                  zg30
TD                      65536
SOLVENT                  DMSO
NS                         24
DS                          0
SWH                 10000.000 Hz
FIDRES               0.305176 Hz
AQ                  3.2767999 sec
RG                        101
DW                     50.000 usec
DE                      11.14 usec
TE                      294.2 K
D1                 1.00000000 sec
TD0                         1
SFO1              400.6236056 MHz
NUC1                       1H
P0                       2.67 usec
P1                       8.00 usec
PLW1              21.67200089 W

F2 - Processing parameters
SI                      65536
SF                400.6200000 MHz
WDW                        EM
SSB                         0
LB                       0.30 Hz
GB                          0
PC                       1.00
```

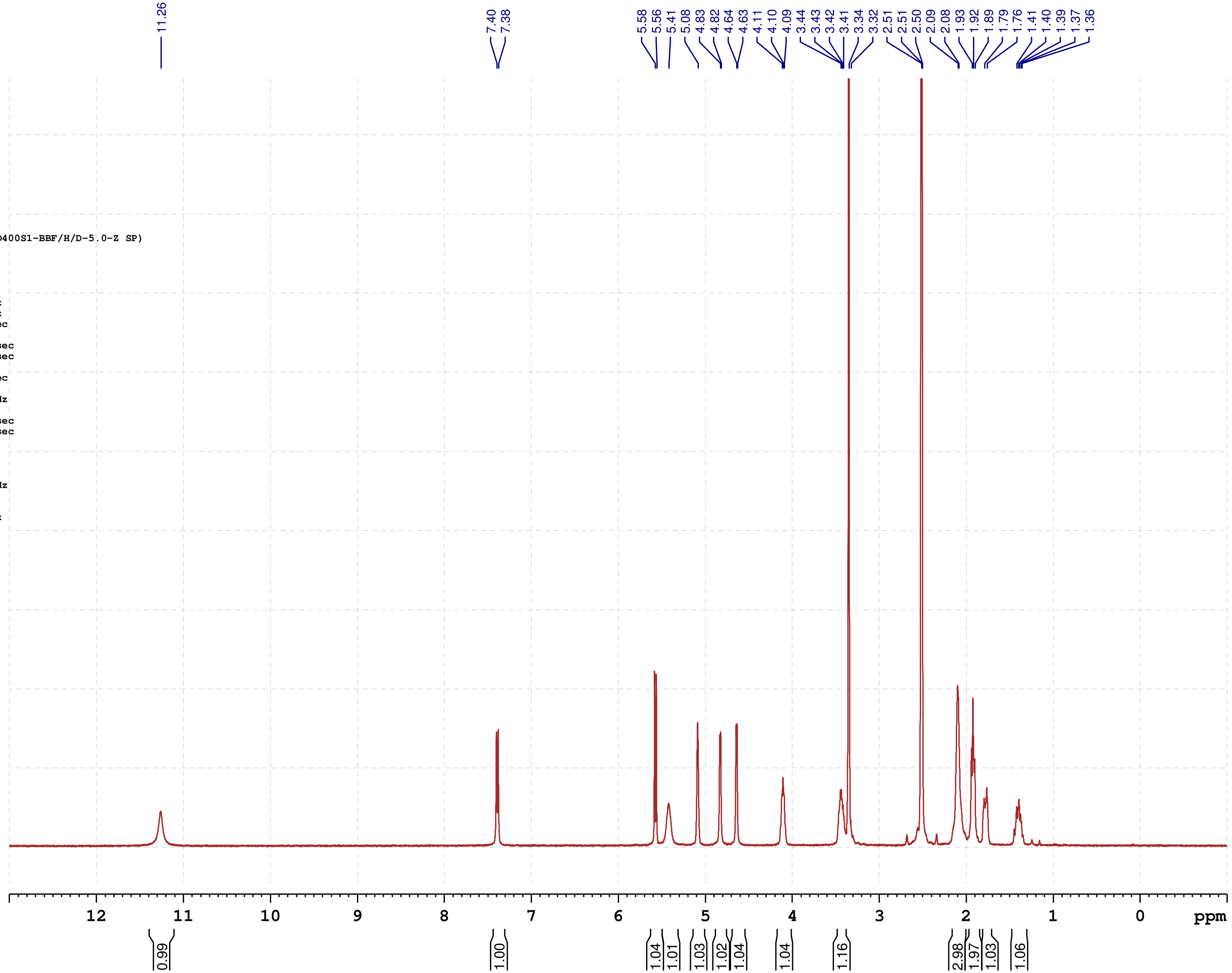

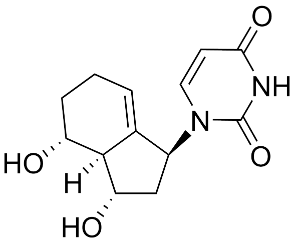

2f

NMR-Spectra for Compound 2f

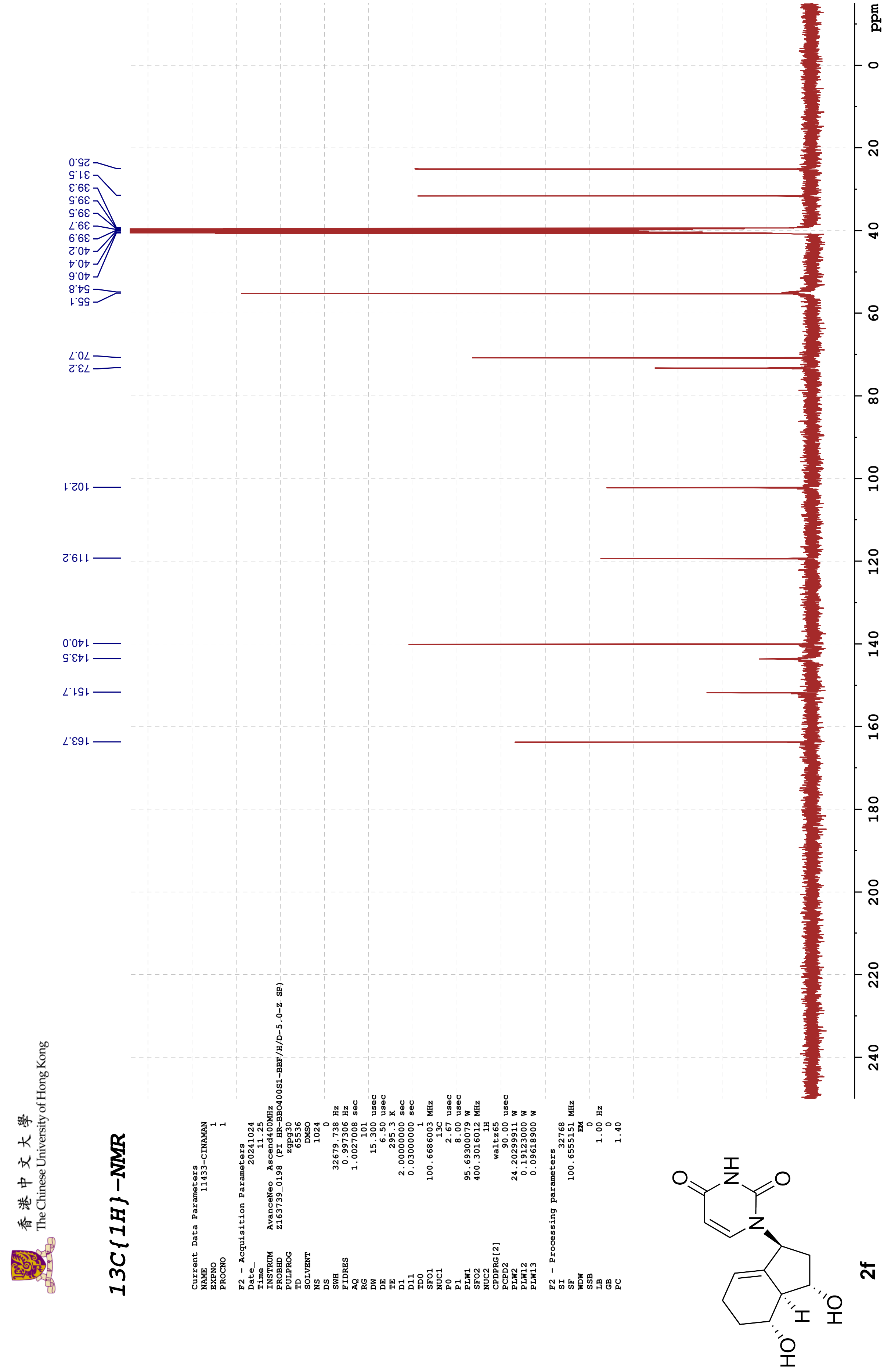

NMR-Spectra for Compound 2g

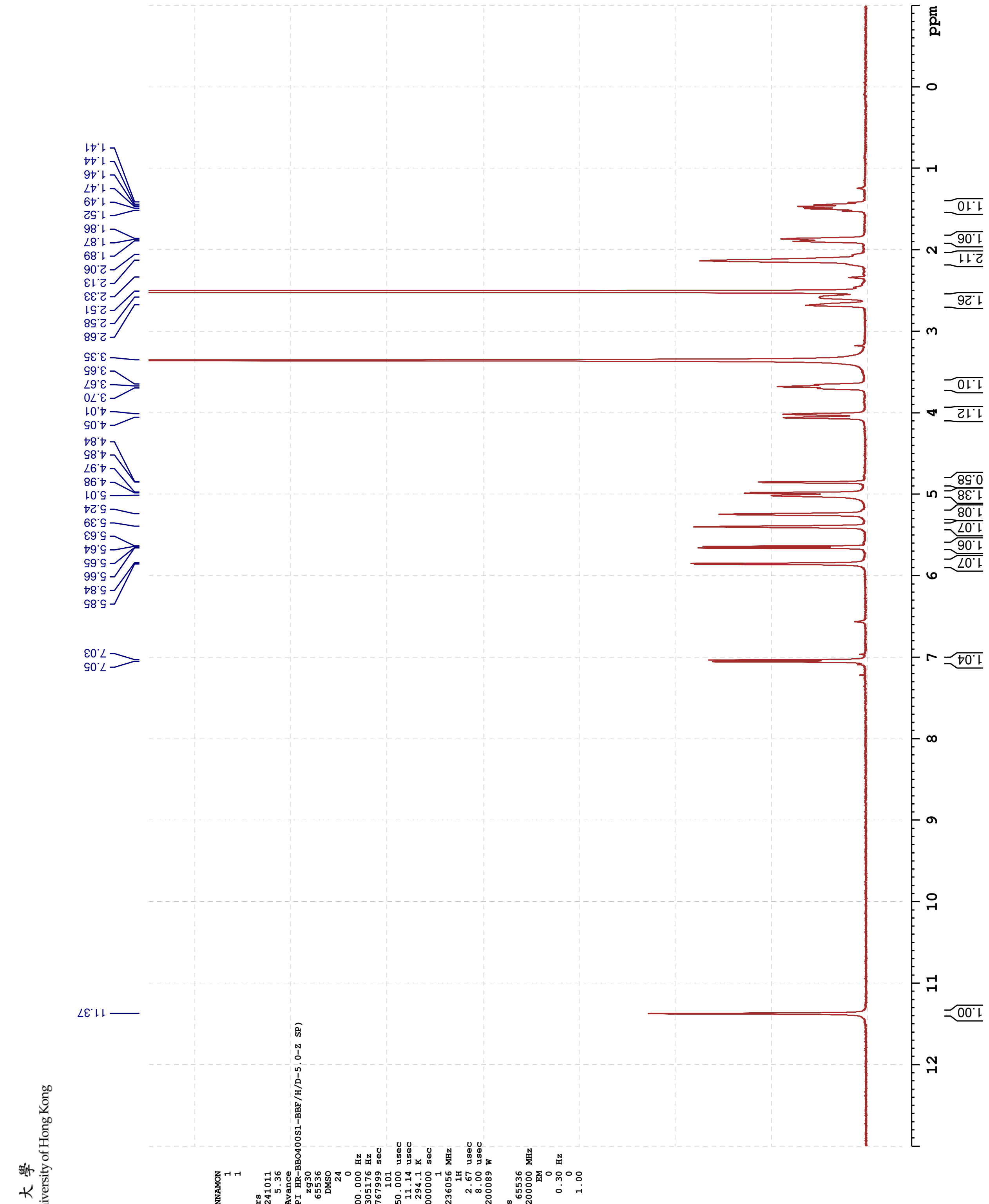

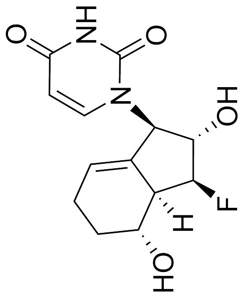

2g

NMR-Spectra for Compound 2g

香港中文大學
The Chinese University of Hong Kong

13C{1H}-NMR

```
Current Data Parameters
NAME            B-24519-CINNAMON
EXPNO                          1
PROCNO                         1

F2 - Acquisition Parameters
Date_                   20241018
Time                       14.36
INSTRUM             AvanceNeo 400
PROBHD      Z163739_0214 (PI HR-BBO400S1-BBF/H/D-5.0-Z SP)
PULPROG                  zgpg30
TD                        65536
SOLVENT                    DMSO
NS                         1067
DS                            0
SWH                   32679.738 Hz
FIDRES                 0.997306 Hz
AQ                    1.0027008 sec
RG                          101
DW                       15.300 usec
DE                         6.50 usec
TE                        295.8 K
D1                   2.00000000 sec
D11                  0.03000000 sec
TD0                           1
SFO1                100.6862041 MHz
NUC1                        13C
P0                         2.63 usec
P1                         7.90 usec
PLW1               100.30000305 W
SFO2                400.3716015 MHz
NUC2                         1H
CPDPRG[2]               waltz65
PCPD2                     90.00 usec
PLW2                24.89999962 W
PLW12                0.18703000 W
PLW13                0.09407300 W

F2 - Processing parameters
SI                        32768
SF                  100.6731166 MHz
WDW                          EM
SSB                           0
LB                         3.00 Hz
GB                            0
PC                         1.40
```

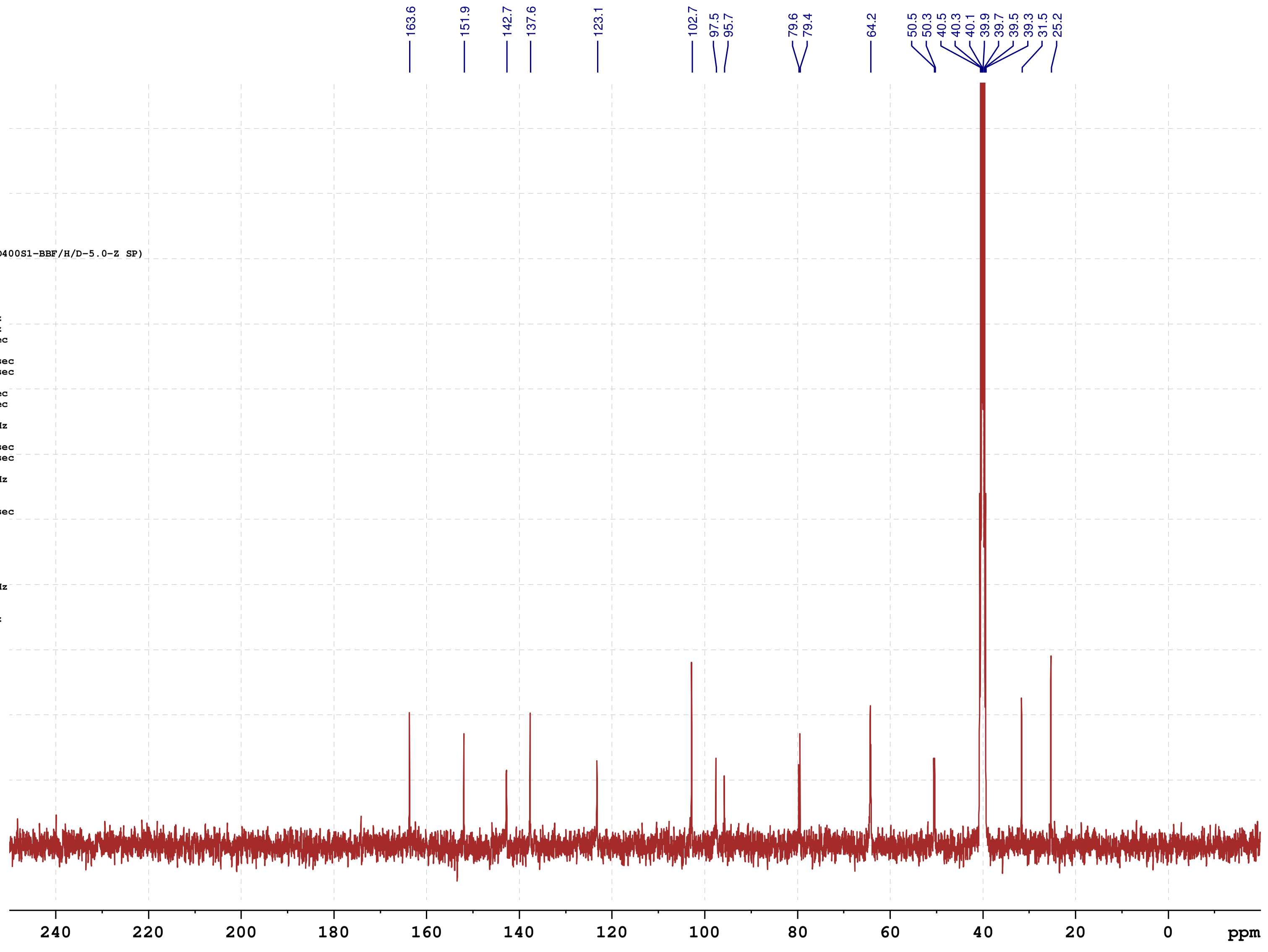

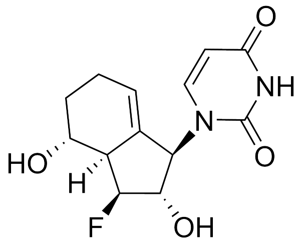

2g

NMR-Spectra for Compound 2i

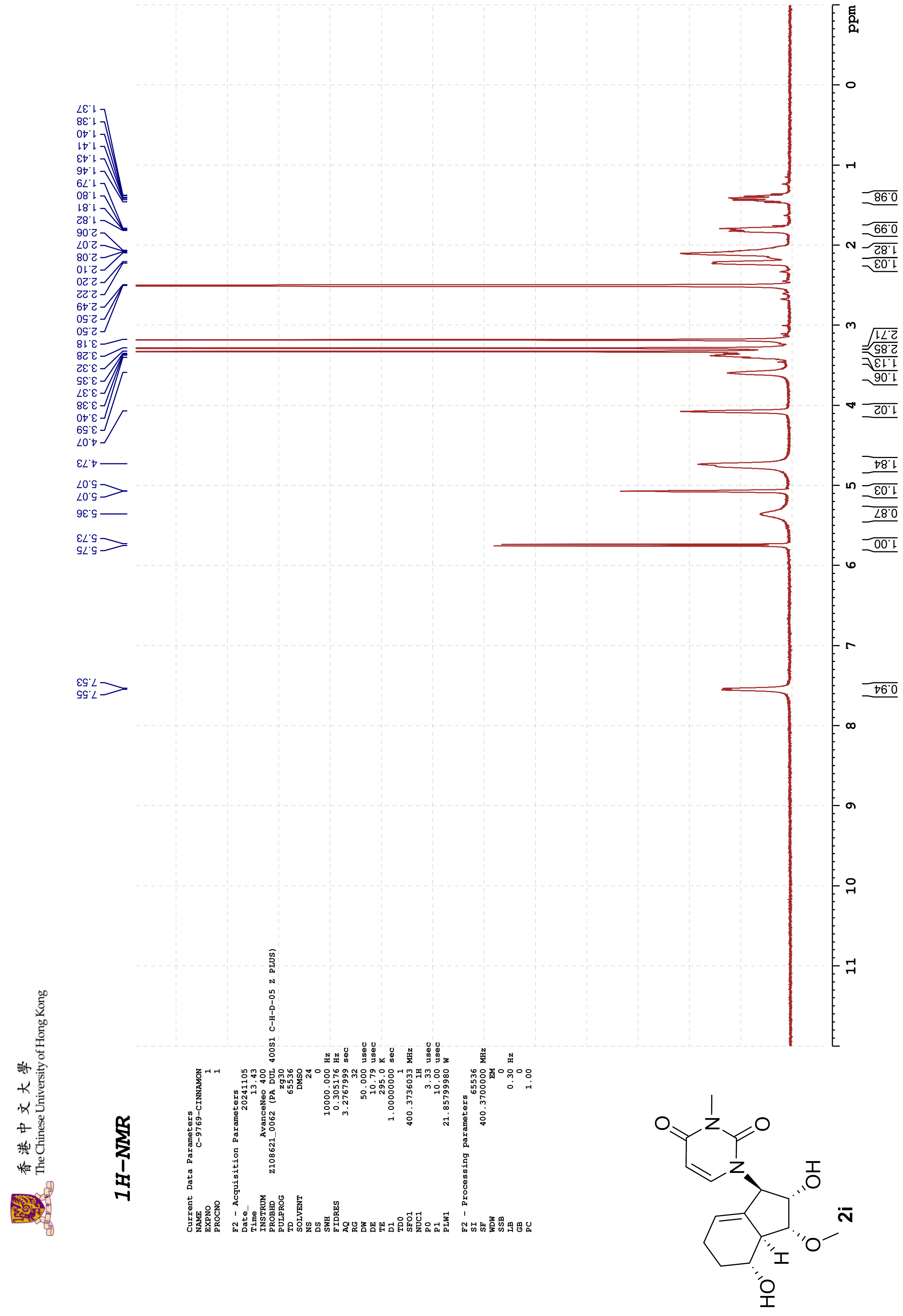

NMR-Spectra for Compound 2i

香港中文大學
The Chinese University of Hong Kong

13C{1H}-NMR

```
Current Data Parameters
NAME          C-10346-CINNAMON
EXPNO                        1
PROCNO                       1

F2 - Acquisition Parameters
Date_                 20241109
Time                      4.37
INSTRUM                 Avance
PROBHD     Z163739_1043 (PI HR-BBO400S1-BBF/H/D-5.0-Z SP)
PULPROG                zgpg30
TD                      65536
SOLVENT                  DMSO
NS                       1024
DS                          0
SWH                 32679.739 Hz
FIDRES               0.997306 Hz
AQ                  1.0027008 sec
RG                       12.7
DW                     15.300 usec
DE                       6.50 usec
TE                      294.4 K
D1                 2.00000000 sec
D11                0.03000000 sec
TD0                         1
SFO1              100.7490748 MHz
NUC1                      13C
P0                       2.67 usec
P1                       8.00 usec
PLW1              96.23899841 W
SFO2              400.6216025 MHz
NUC2                       1H
CPDPRG2               waltz65
PCPD2                   90.00 usec
PLW2              21.54100037 W
PLW12              0.17020001 W
PLW13              0.08560800 W

F2 - Processing parameters
SI                      32768
SF                100.7359791 MHz
WDW                        EM
SSB                         0
LB                       2.00 Hz
GB                          0
PC                       1.40
```

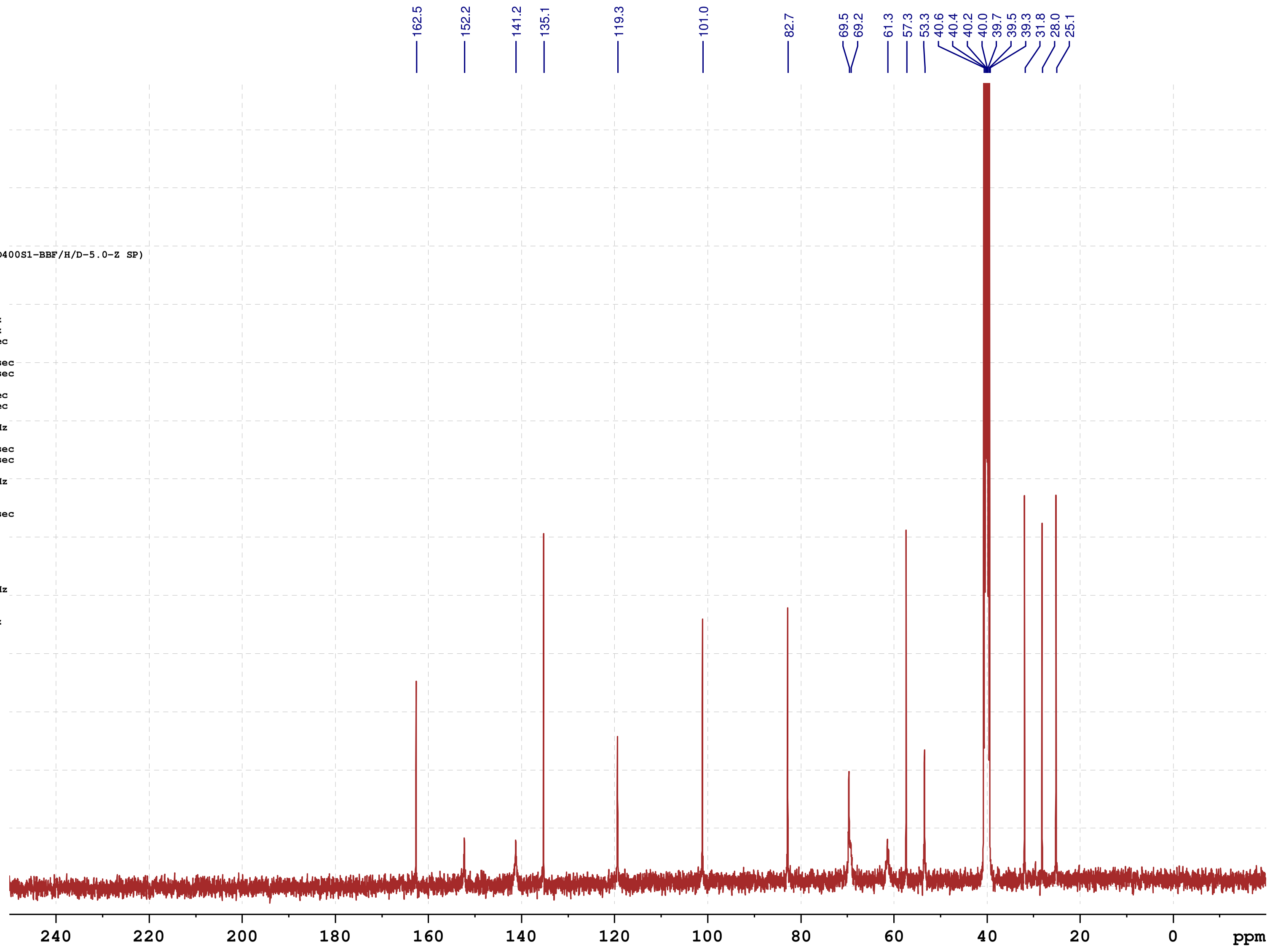

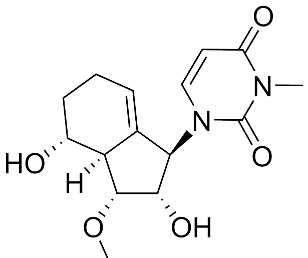

2i

NMR-Spectra for Compound 3a

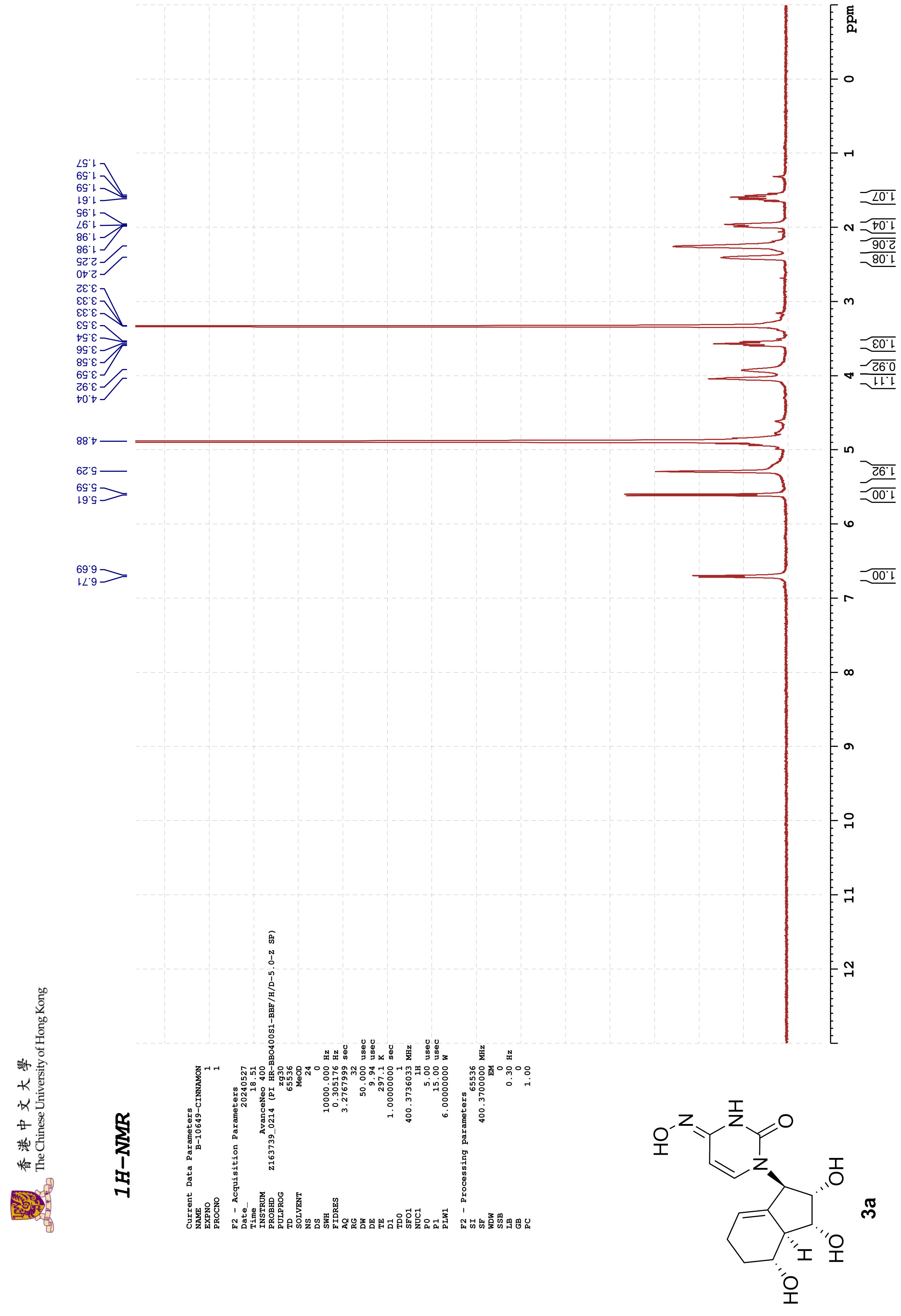

NMR-Spectra for Compound 3a

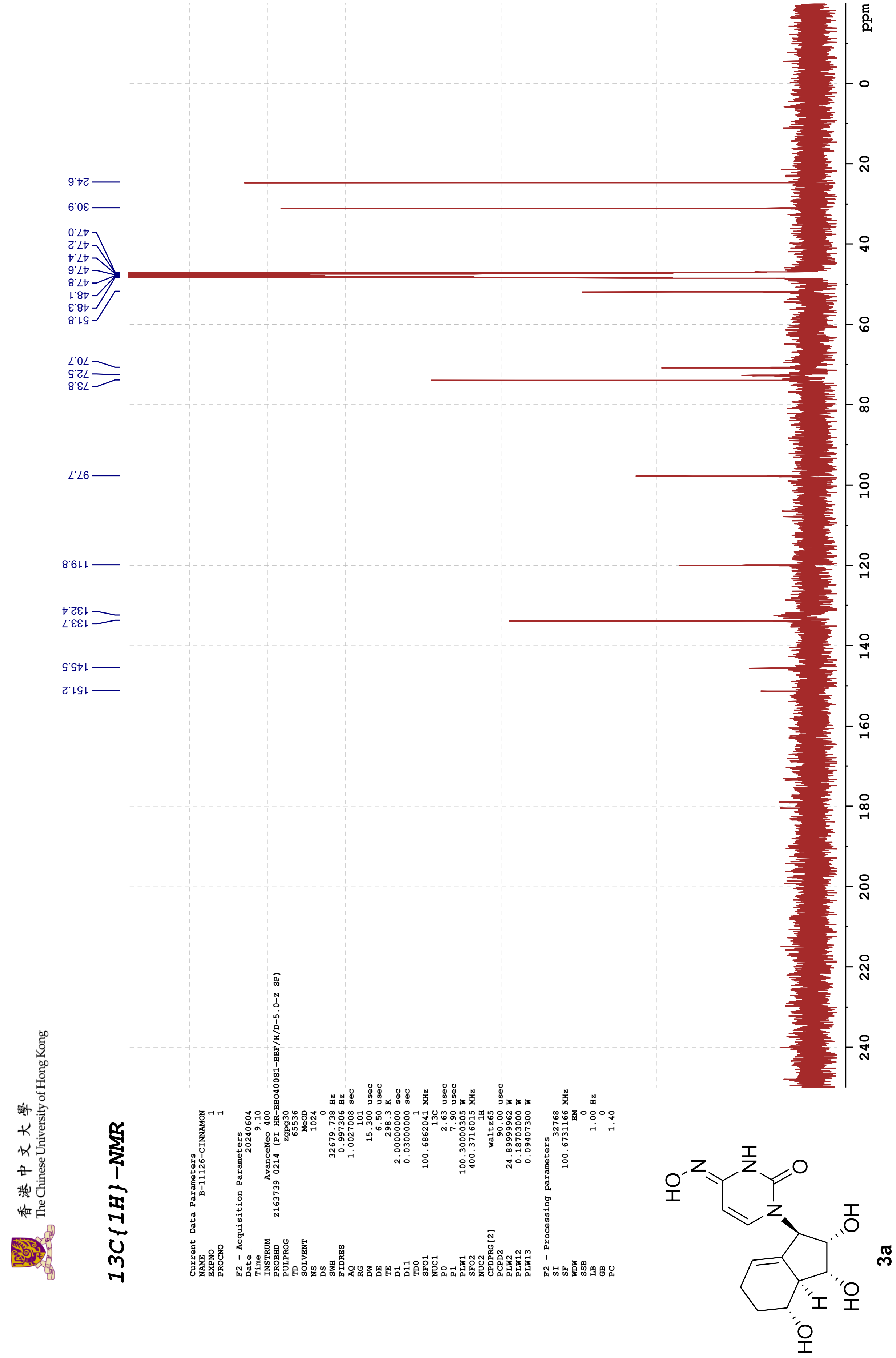

NMR-Spectra for Compound 3b

香港中文大學 The Chinese University of Hong Kong

1H-NMR

```
Current Data Parameters
NAME          B-13056-CINNAMON
EXPNO                        1
PROCNO                       1

F2 - Acquisition Parameters
Date_                 20240627
Time                     20.53
INSTRUM          AvanceNeo 400
PROBHD     Z163739_0214 (PI HR-BBO400S1-BBF/H/D-5.0-Z SP)
PULPROG                   zg30
TD                       65536
SOLVENT                   MeOD
NS                          24
DS                           0
SWH              10000.000 Hz
FIDRES            0.305176 Hz
AQ               3.2767999 sec
RG                          32
DW                50.000 usec
DE                  9.94 usec
TE                   295.3 K
D1              1.00000000 sec
TD0                          1
SFO1           400.3736033 MHz
NUC1                        1H
P0                  5.00 usec
P1                 15.00 usec
PLW1            6.00000000 W

F2 - Processing parameters
SI                       65536
SF             400.3700000 MHz
WDW                         EM
SSB                          0
LB                     0.30 Hz
GB                           0
PC                        1.00
```

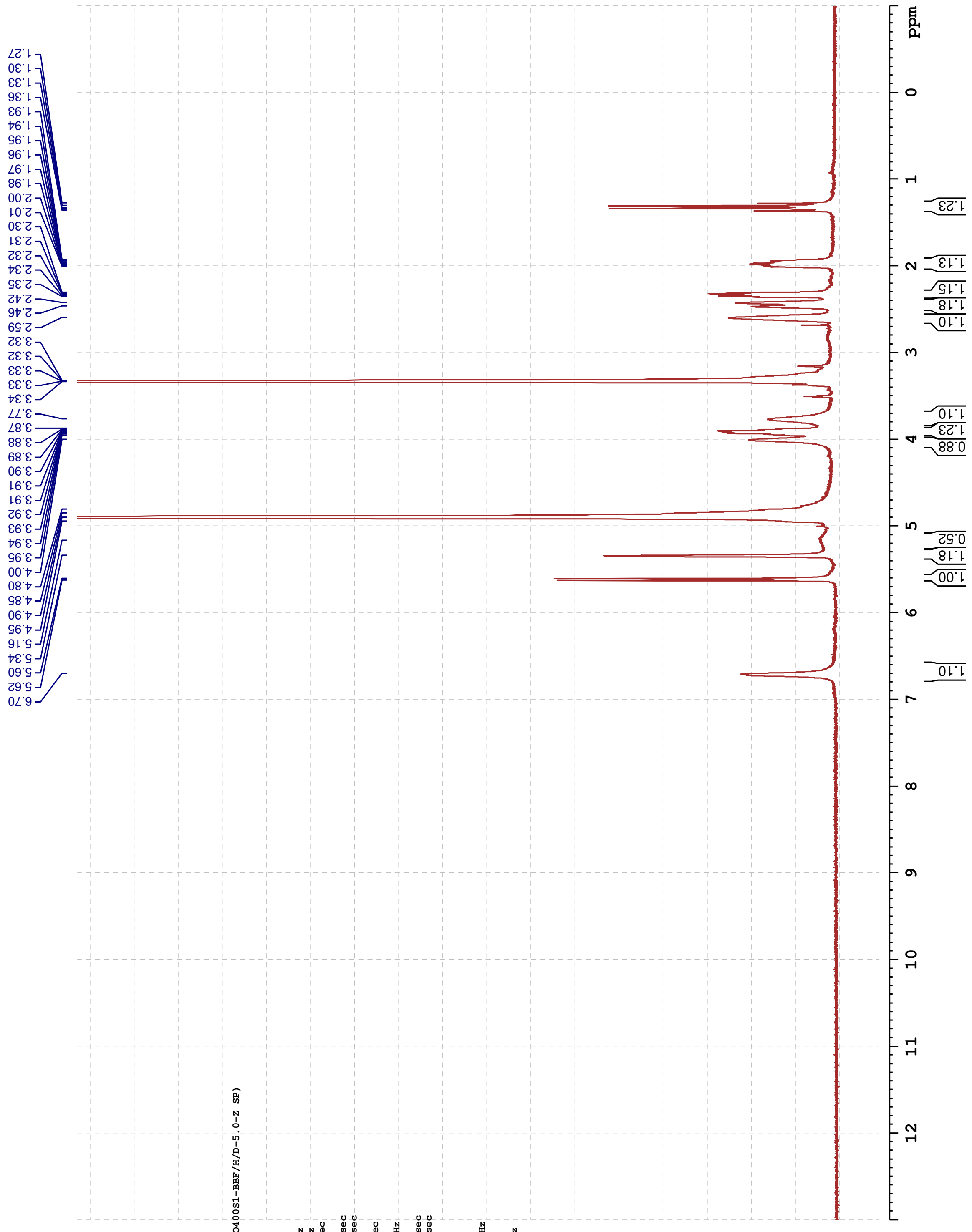

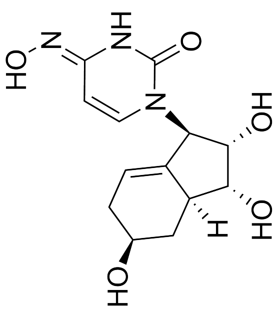

3c

NMR-Spectra for Compound 3b

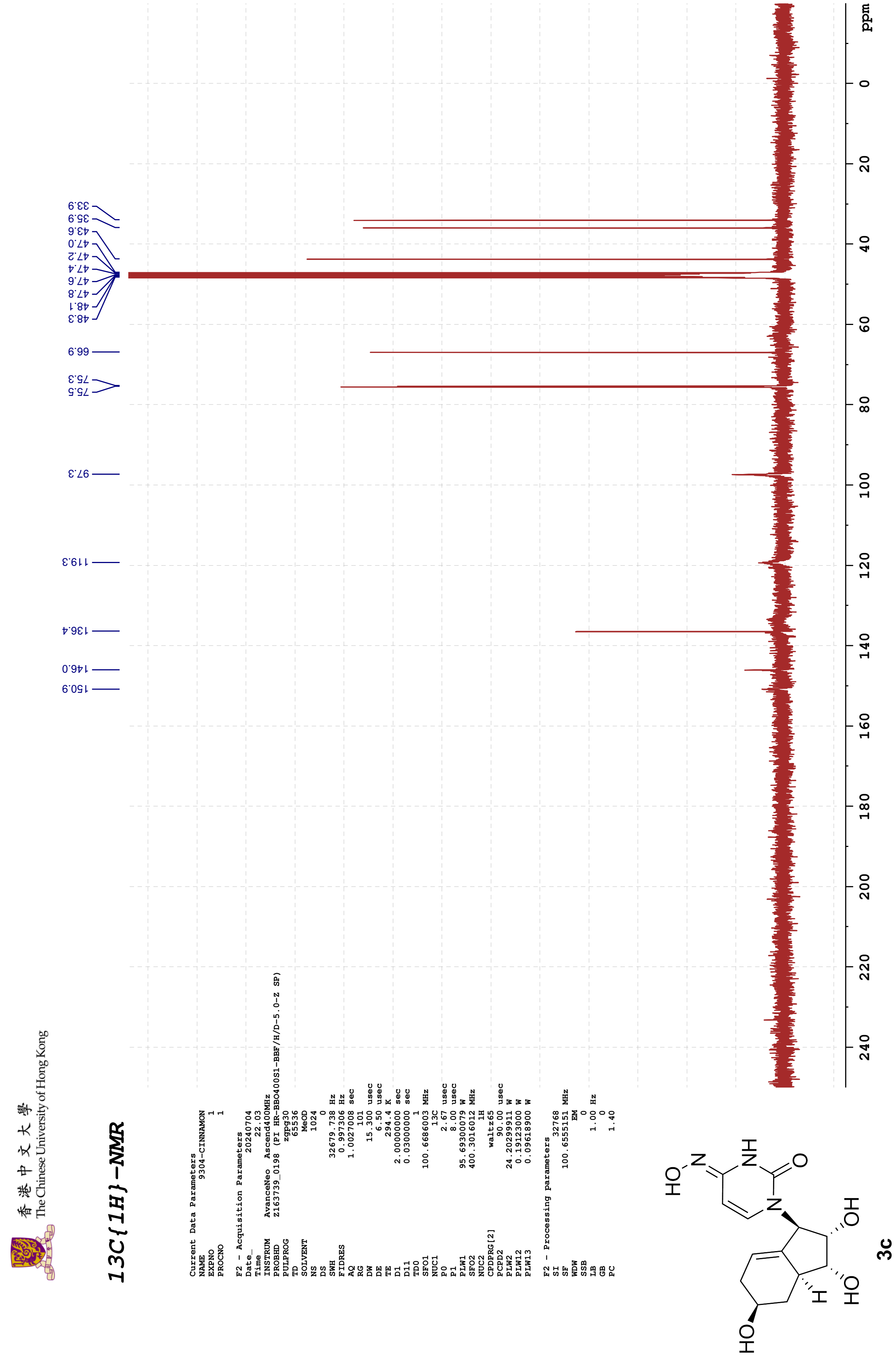

NMR-Spectra for Compound 3c

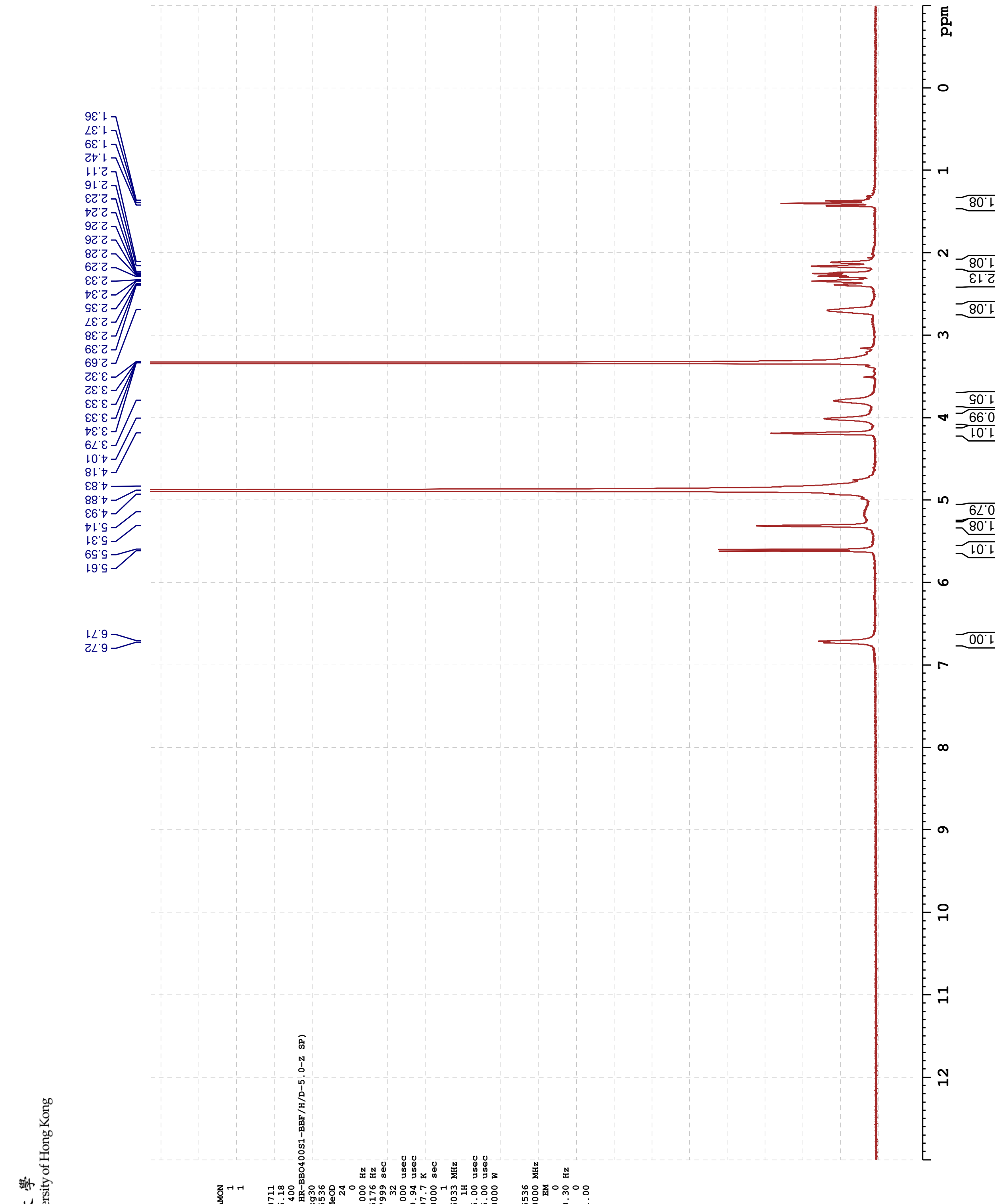
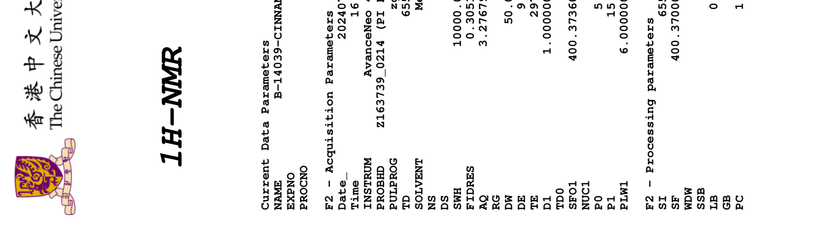

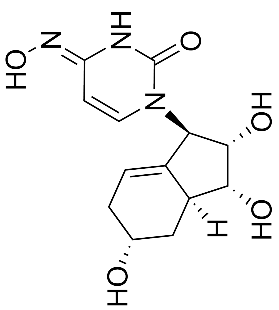

3c

NMR-Spectra for Compound 3c

香港中文大學
The Chinese University of Hong Kong

13C{1H}-NMR

```
Current Data Parameters
NAME               9832-CINNAMON
EXPNO                          1
PROCNO                         1

F2 - Acquisition Parameters
Date_                   20240714
Time                       10.32
INSTRUM    AvanceNeo  Ascend400MHz
PROBHD     Z163739_0198 (PI HR-BBO400S1-BBF/H/D-5.0-Z SP)
PULPROG                  zgpg30
TD                        65536
SOLVENT                    MeOD
NS                         1024
DS                            0
SWH                   32679.738 Hz
FIDRES                 0.997306 Hz
AQ                    1.0027008 sec
RG                          101
DW                       15.300 usec
DE                         6.50 usec
TE                        295.2 K
D1                   2.00000000 sec
D11                  0.03000000 sec
TD0                           1
SFO1                100.6686003 MHz
NUC1                        13C
P0                         2.67 usec
P1                         8.00 usec
PLW1                95.69300079 W
SFO2                400.3016012 MHz
NUC2                         1H
CPDPRG[2]               waltz65
PCPD2                     90.00 usec
PLW2                24.20299911 W
PLW12                0.19123000 W
PLW13                0.09618900 W

F2 - Processing parameters
SI                        32768
SF                  100.6555151 MHz
WDW                          EM
SSB                           0
LB                         1.00 Hz
GB                            0
PC                         1.40
```

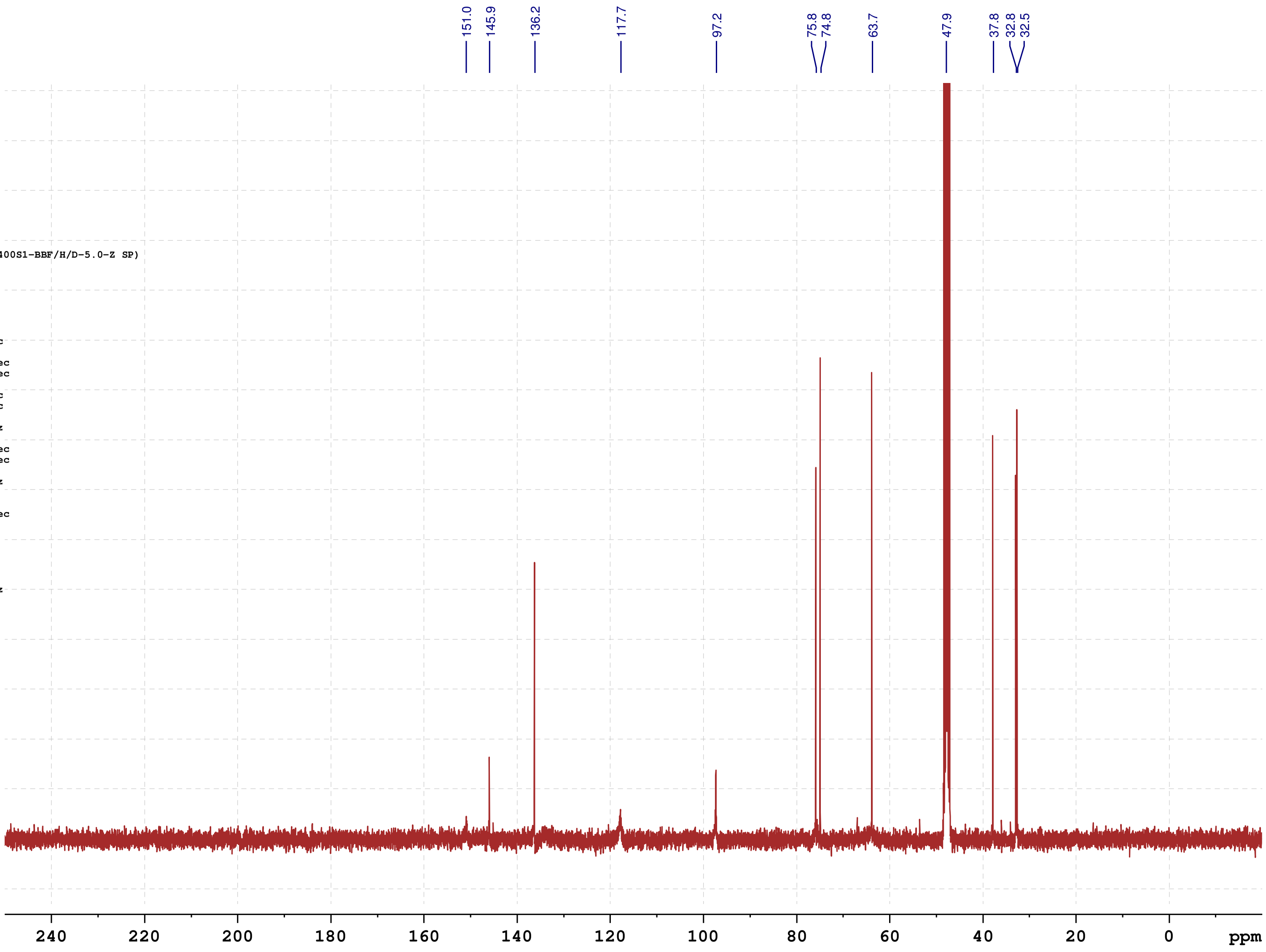

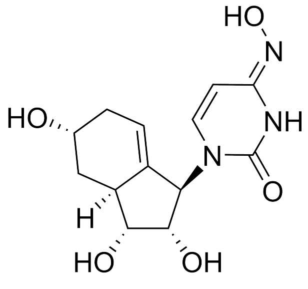

3c

NMR-Spectra for Compound 3d

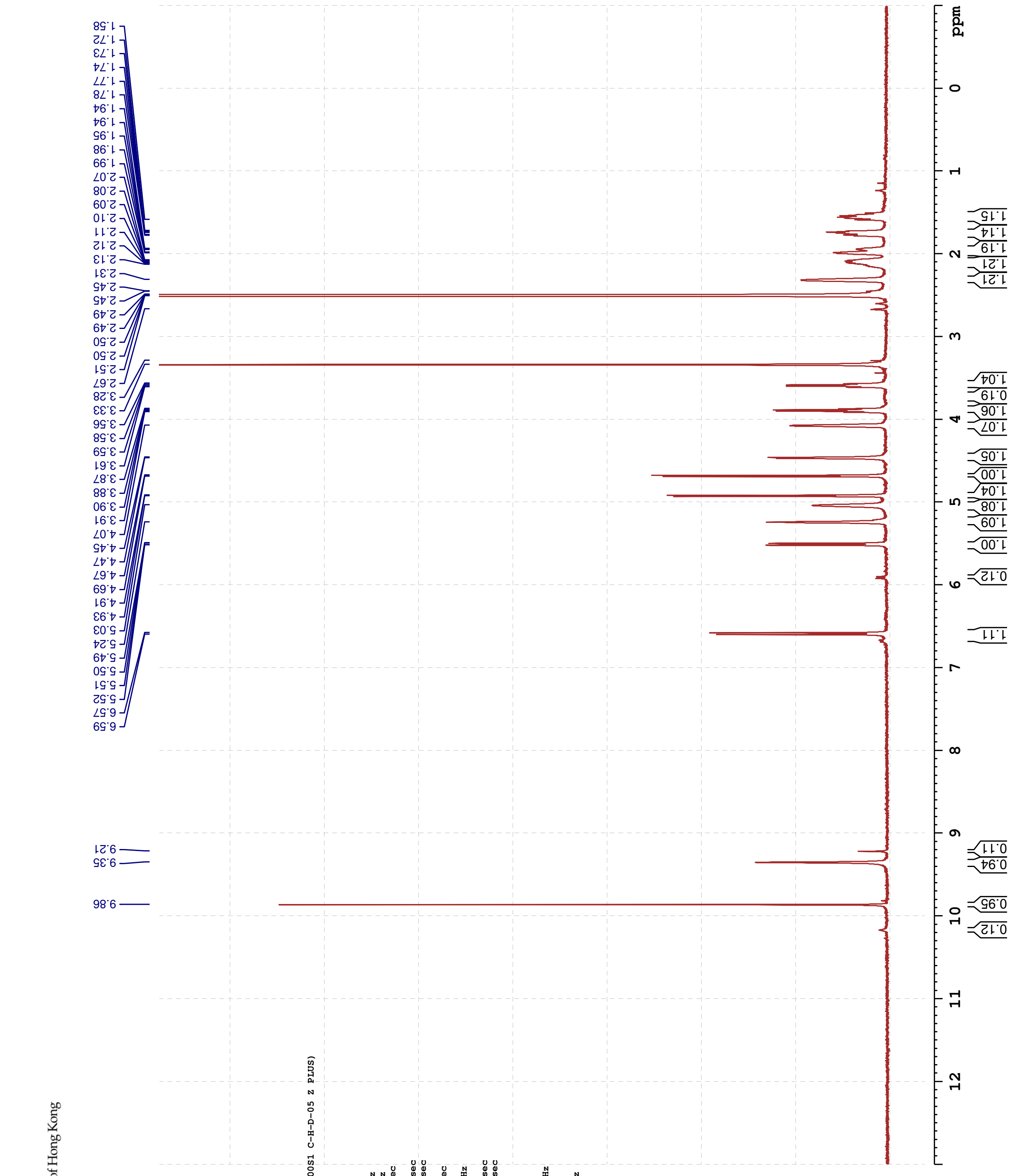

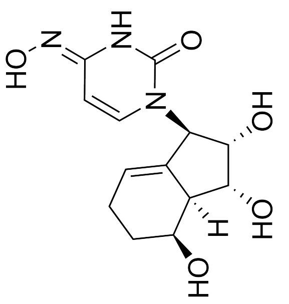

3d

NMR-Spectra for Compound 3d

香港中文大學
The Chinese University of Hong Kong

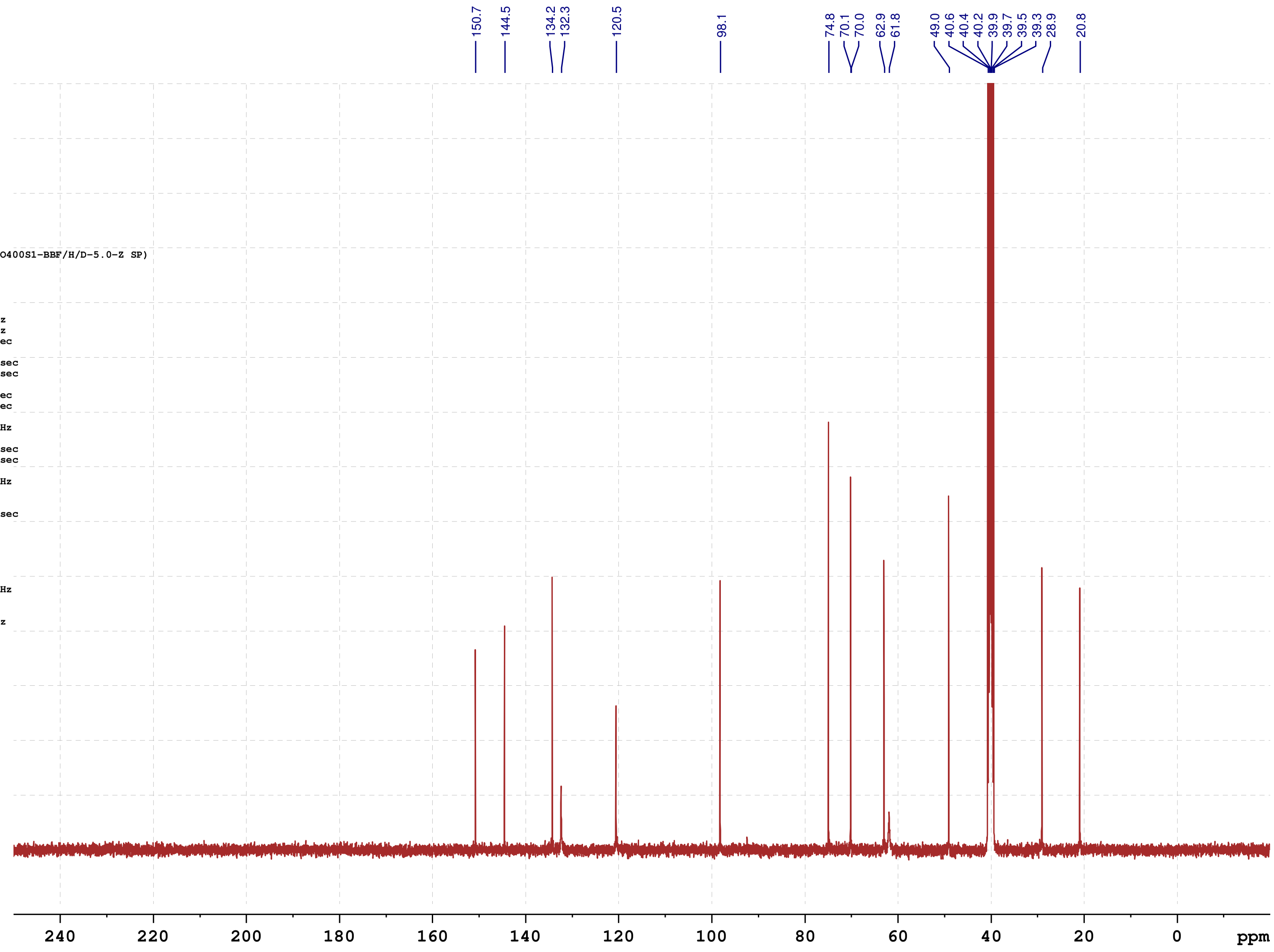

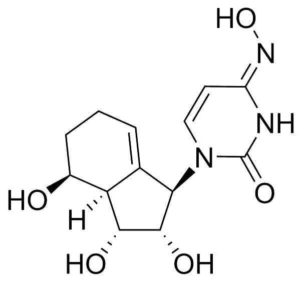

3d

NMR-Spectra for Compound 3e

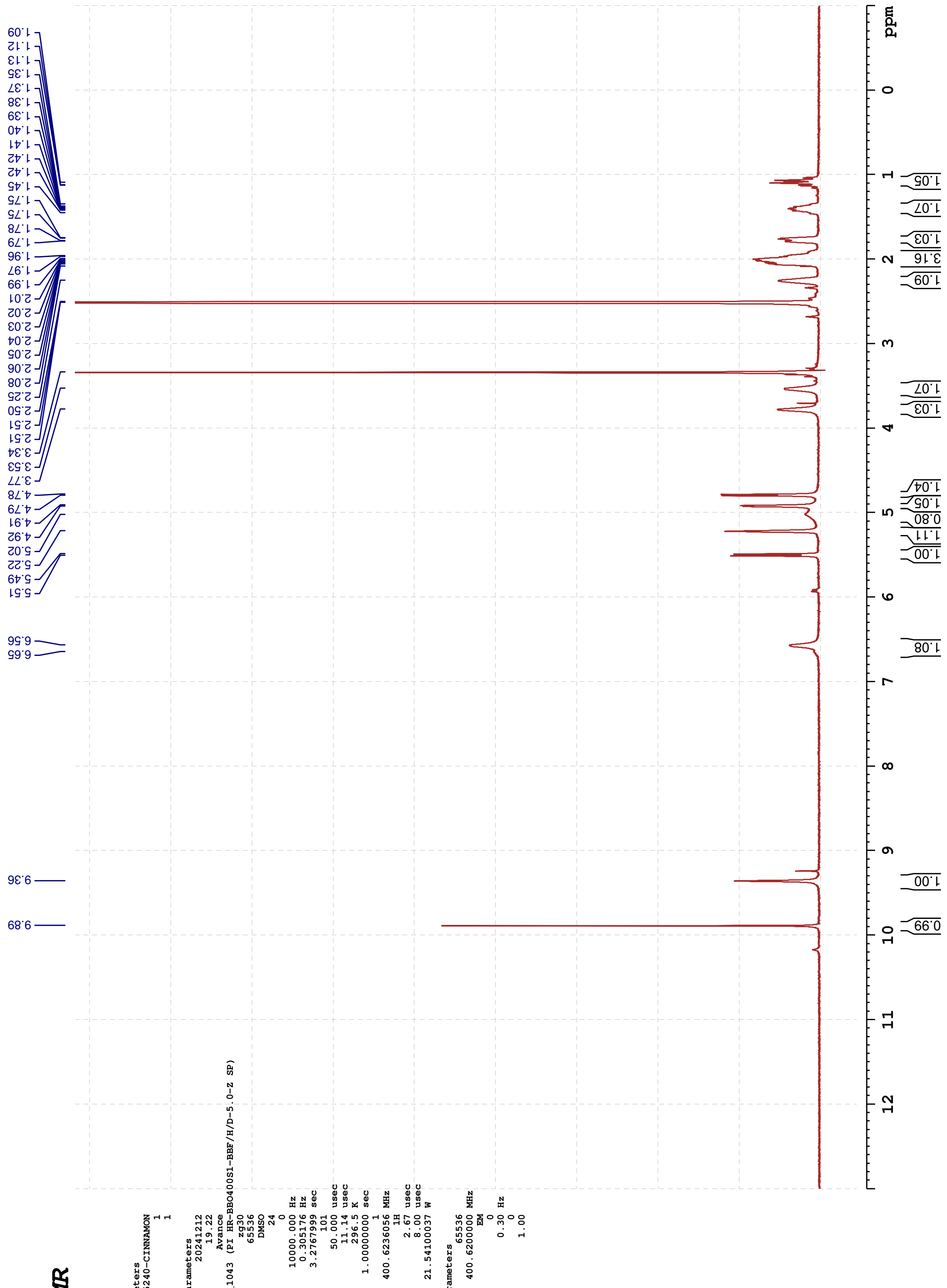

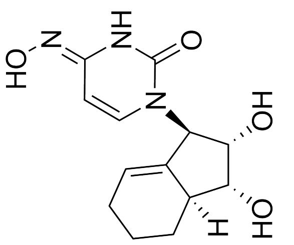

3e

NMR-Spectra for Compound 3e

香港中文大學 The Chinese University of Hong Kong

13C{1H}-NMR

```
Current Data Parameters
NAME       C-16519-CINNAMON-cmr
EXPNO                         1
PROCNO                        1

F2 - Acquisition Parameters
Date_                  20241220
Time                       9.33
INSTRUM                  Avance
PROBHD     Z163739_1043 (PI HR-BBO400S1-BBF/H/D-5.0-Z SP)
PULPROG                 zgpg30
TD                        65536
SOLVENT                    DMSO
NS                         1024
DS                            0
SWH                   32679.739 Hz
FIDRES                 0.997306 Hz
AQ                    1.0027008 sec
RG                         12.7
DW                       15.300 usec
DE                         6.50 usec
TE                        294.1 K
D1                   2.00000000 sec
D11                  0.03000000 sec
TD0                           1
SFO1                100.7490748 MHz
NUC1                        13C
P0                         2.67 usec
P1                         8.00 usec
PLW1                96.23899841 W
SFO2                400.6216025 MHz
NUC2                         1H
CPDPRG2                 waltz65
PCPD2                     90.00 usec
PLW2                21.54100037 W
PLW12                0.17020001 W
PLW13                0.08560800 W

F2 - Processing parameters
SI                        32768
SF                  100.7359791 MHz
WDW                          EM
SSB                           0
LB                         2.00 Hz
GB                            0
PC                         1.40
```

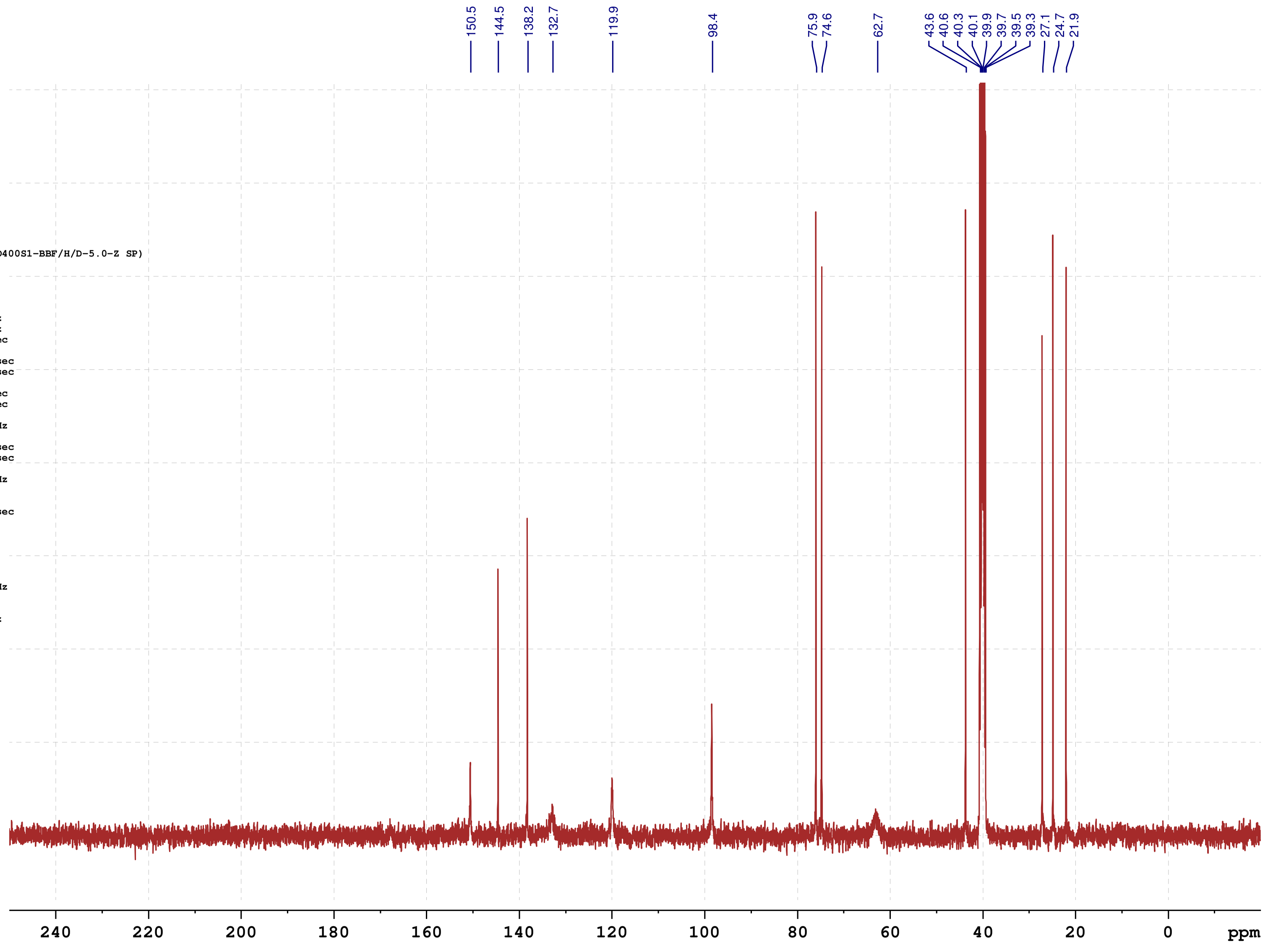

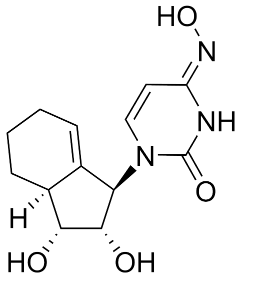

3e

NMR-Spectra for Compound 3f

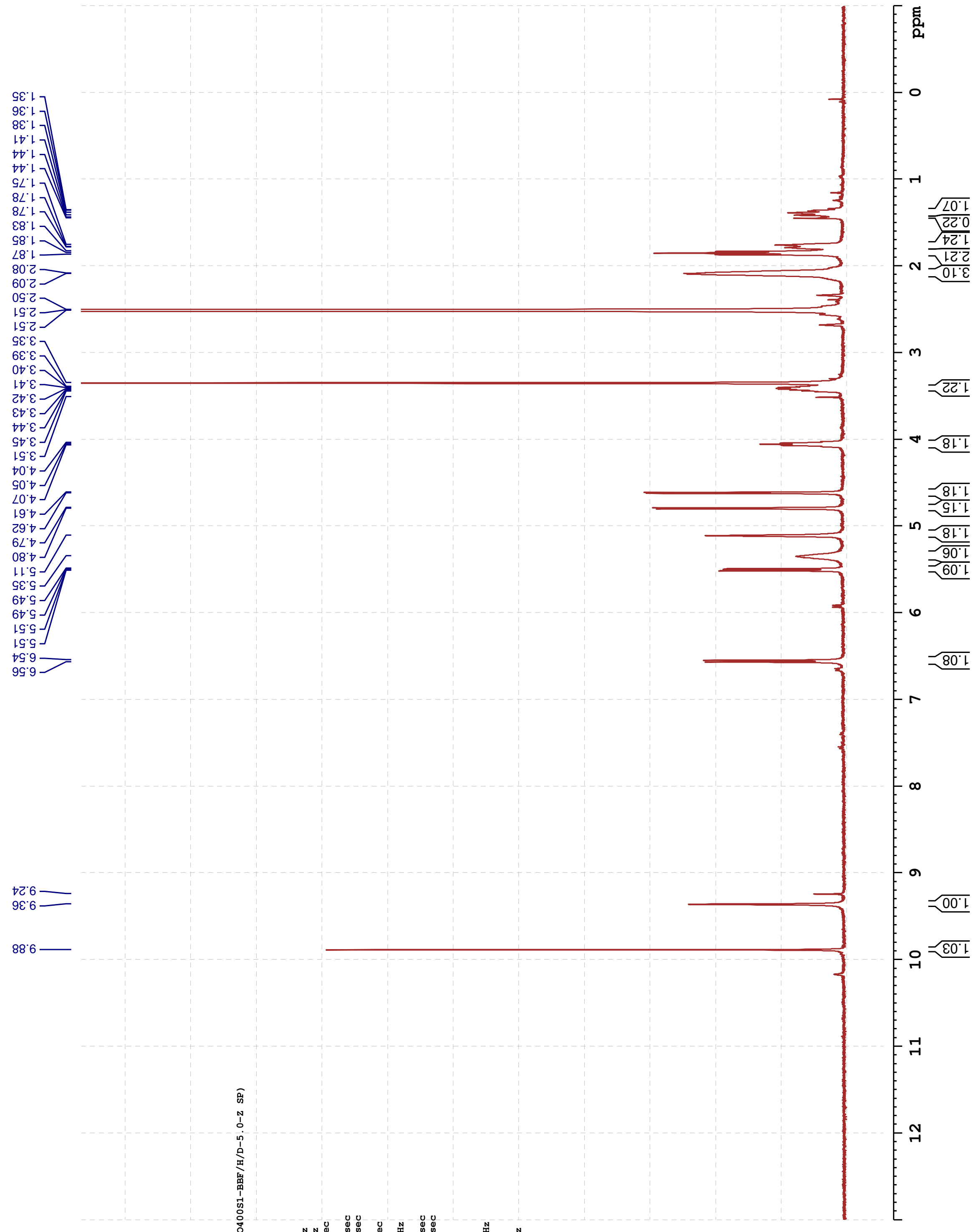

香港中文大學 The Chinese University of Hong Kong

1H-NMR

Current Data Parameters
NAME C-16359-CINNAMON
EXPNO 1
PROCNO 1

F2 - Acquisition Parameters
Date_ 20241219
Time 14.28
INSTRUM Avance
PROBHD Z163739_1043 (PI HR-BBO400S1-BBF/H/D-5.0-Z SP)
PULPROG zg30
TD 65536
SOLVENT DMSO
NS 24
DS 0
SWH 10000.000 Hz
FIDRES 0.305176 Hz
AQ 3.2767999 sec
RG 101
DW 50.000 usec
DE 11.14 usec
TE 293.6 K
D1 1.00000000 sec
TD0 1
SFO1 400.6236056 MHz
NUC1 1H
P0 2.67 usec
P1 8.00 usec
PLW1 21.54100037 W

F2 - Processing parameters
SI 65536
SF 400.6200000 MHz
WDW EM
SSB 0
LB 0.30 Hz
GB 0
PC 1.00

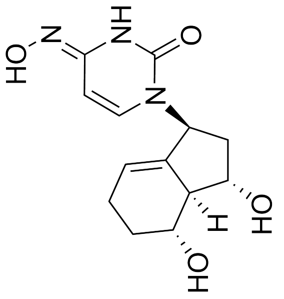

3f

NMR-Spectra for Compound 4a

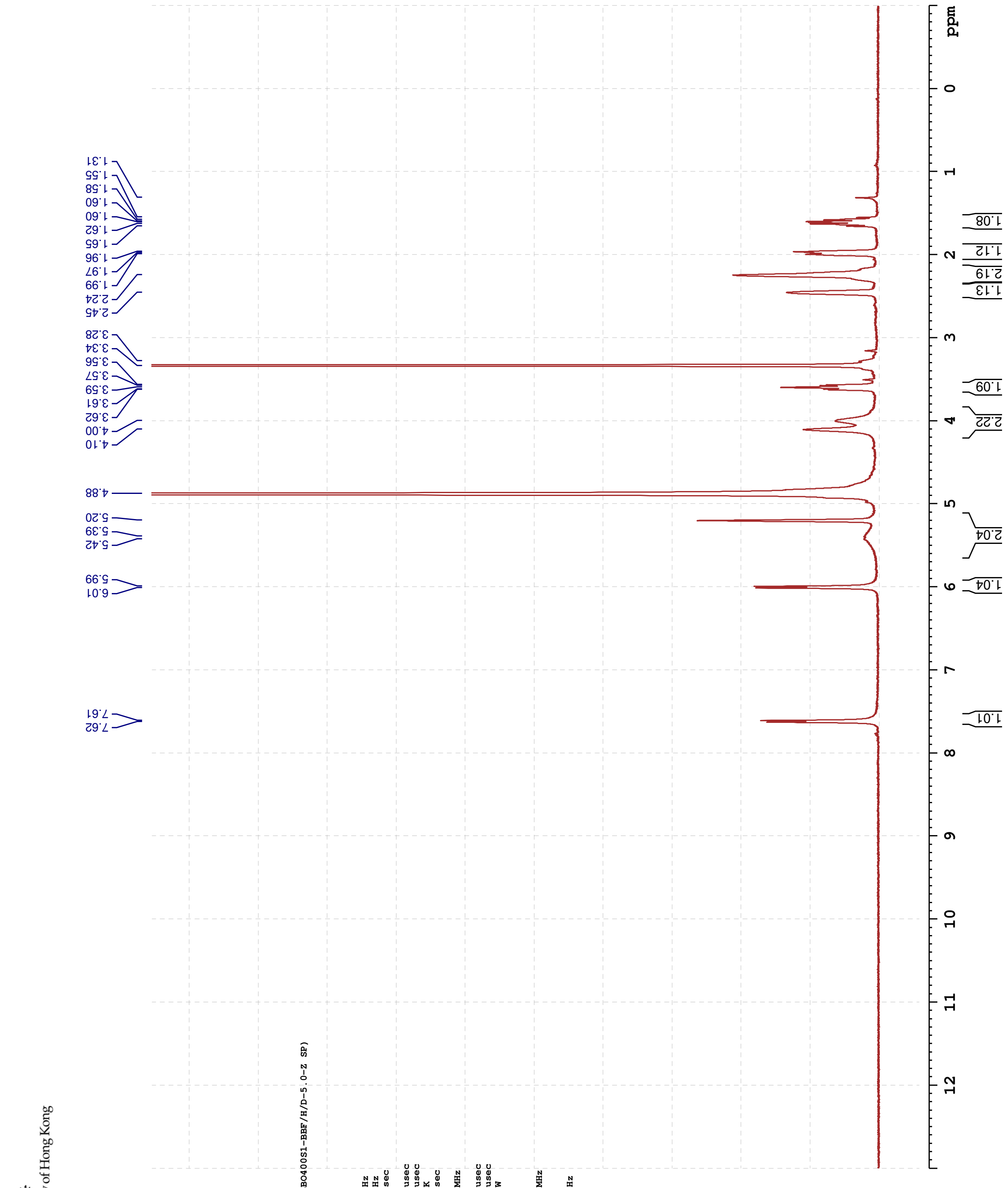

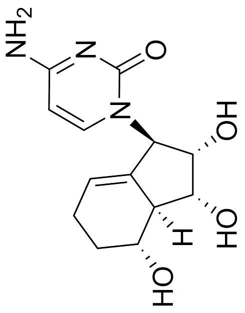

4a

NMR-Spectra for Compound 4a

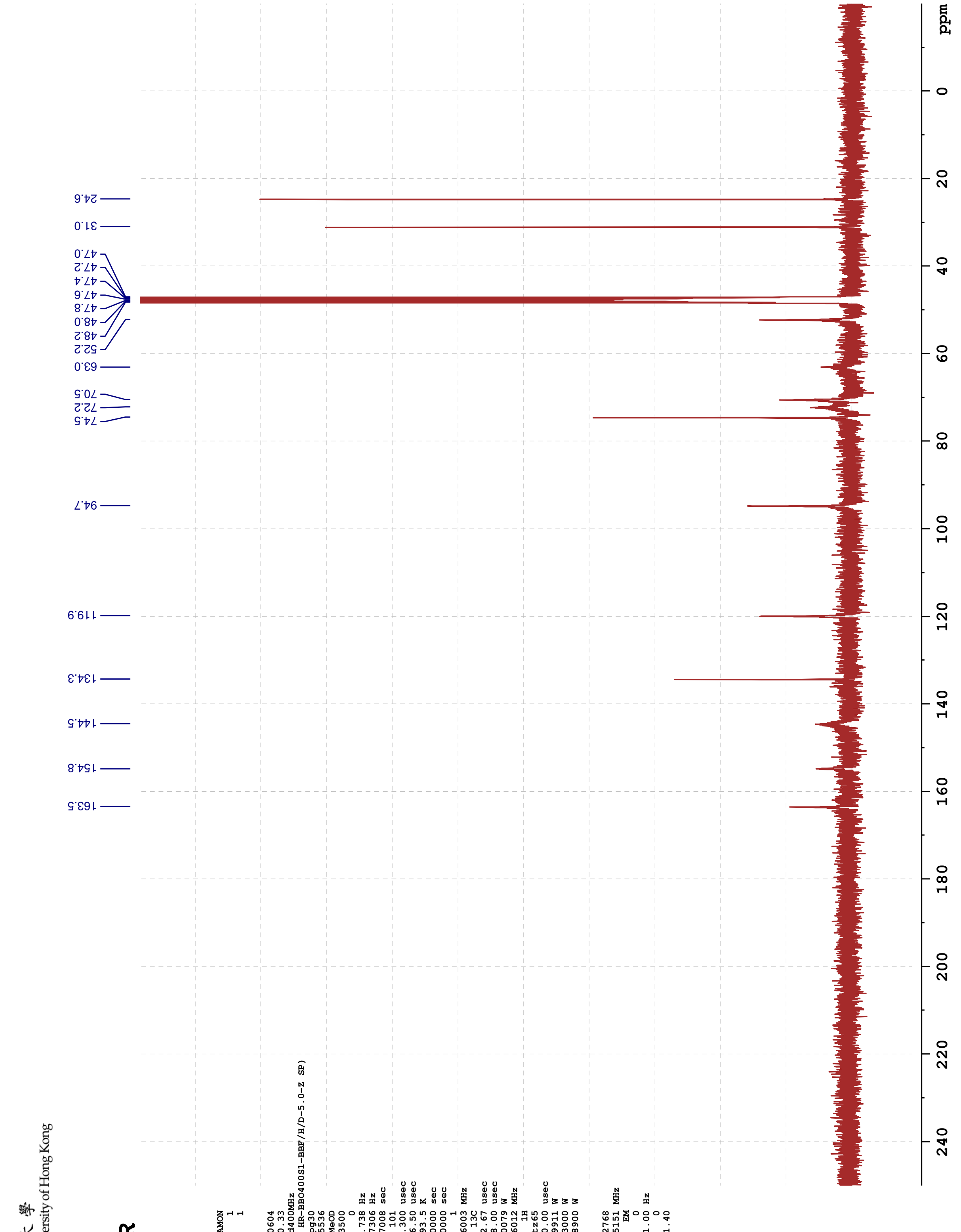

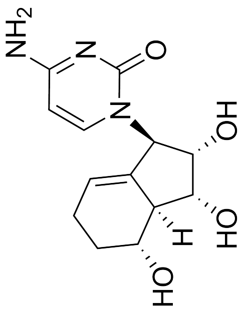

4a

NMR-Spectra for Compound 4b

香港中文大學 The Chinese University of Hong Kong

1H-NMR

```
Current Data Parameters
NAME              B-12693-CINNAMON
EXPNO                            1
PROCNO                           1

F2 - Acquisition Parameters
Date_                     20240623
Time                          8.53
INSTRUM             AvanceNeo 400
PROBHD      Z163739_0214 (PI HR-BBO400S1-BBF/H/D-5.0-Z SP)
PULPROG                       zg30
TD                           65536
SOLVENT                       MeOD
NS                              24
DS                               0
SWH                      10000.000 Hz
FIDRES                    0.305176 Hz
AQ                       3.2767999 sec
RG                              32
DW                          50.000 usec
DE                            9.94 usec
TE                           295.5 K
D1                      1.00000000 sec
TD0                              1
SFO1                   400.3736033 MHz
NUC1                            1H
P0                            5.00 usec
P1                           15.00 usec
PLW1                    6.00000000 W

F2 - Processing parameters
SI                           65536
SF                     400.3700000 MHz
WDW                             EM
SSB                              0
LB                            0.30 Hz
GB                               0
PC                            1.00
```

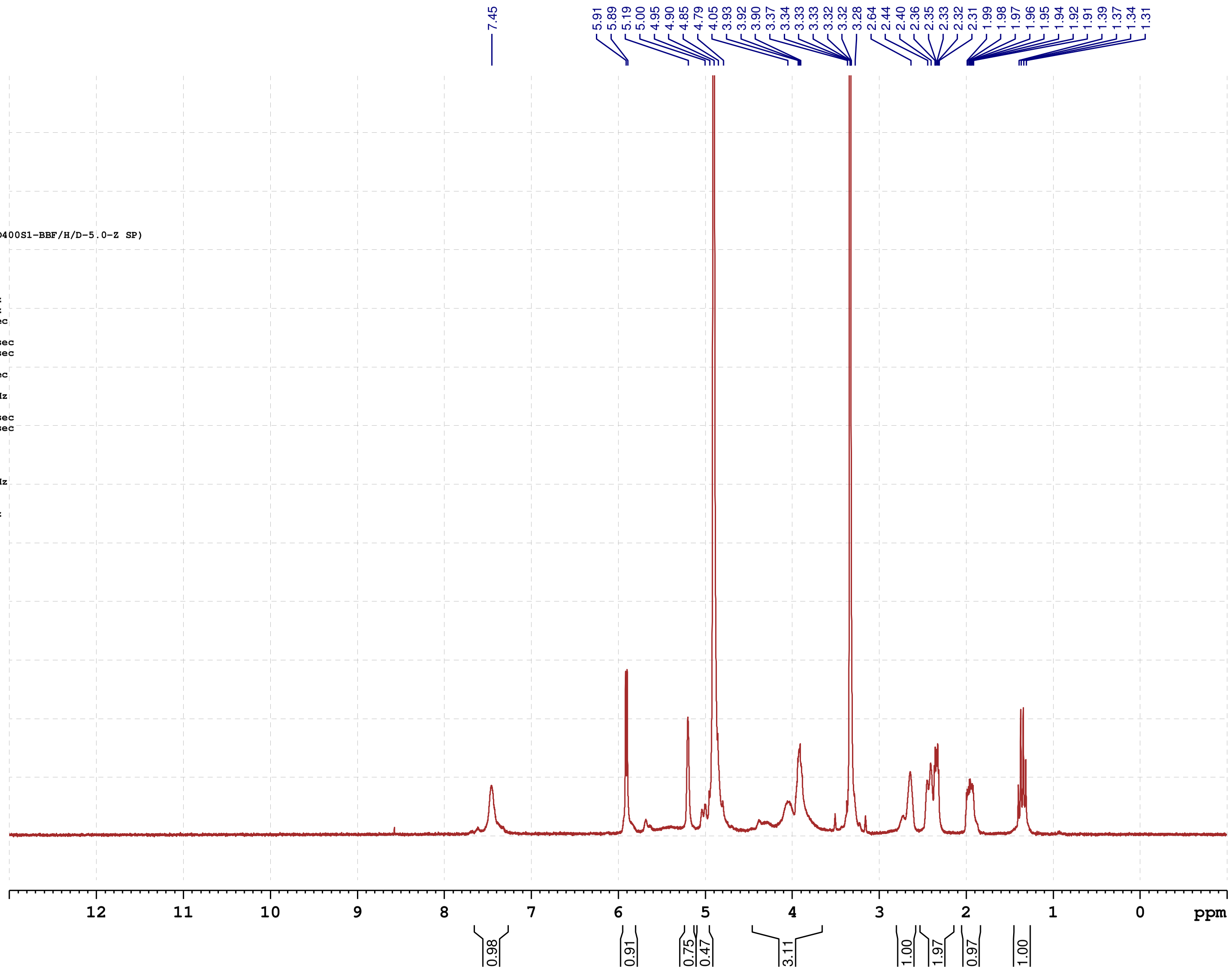

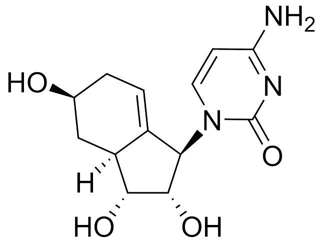

4b

NMR-Spectra for Compound 4b

香港中文大學
The Chinese University of Hong Kong

13C{1H}-NMR

```
Current Data Parameters
NAME        B-13753-CINNAMON cmr
EXPNO                          1
PROCNO                         1

F2 - Acquisition Parameters
Date_                   20240706
Time                       17.50
INSTRUM           AvanceNeo 400
PROBHD      Z163739_0214 (PI HR-BBO400S1-BBF/H/D-5.0-Z SP)
PULPROG                  zgpg30
TD                         65536
SOLVENT                     MeOD
NS                         10000
DS                             0
SWH                    32679.738 Hz
FIDRES                  0.997306 Hz
AQ                     1.0027008 sec
RG                           101
DW                        15.300 usec
DE                          6.50 usec
TE                         295.5 K
D1                    2.00000000 sec
D11                   0.03000000 sec
TD0                            1
SFO1                 100.6862041 MHz
NUC1                         13C
P0                          2.63 usec
P1                          7.90 usec
PLW1                100.30000305 W
SFO2                 400.3716015 MHz
NUC2                          1H
CPDPRG[2]                waltz65
PCPD2                      90.00 usec
PLW2                 24.89999962 W
PLW12                 0.18703000 W
PLW13                 0.09407300 W

F2 - Processing parameters
SI                         32768
SF                   100.6731166 MHz
WDW                           EM
SSB                            0
LB                          2.00 Hz
GB                             0
PC                          1.40
```

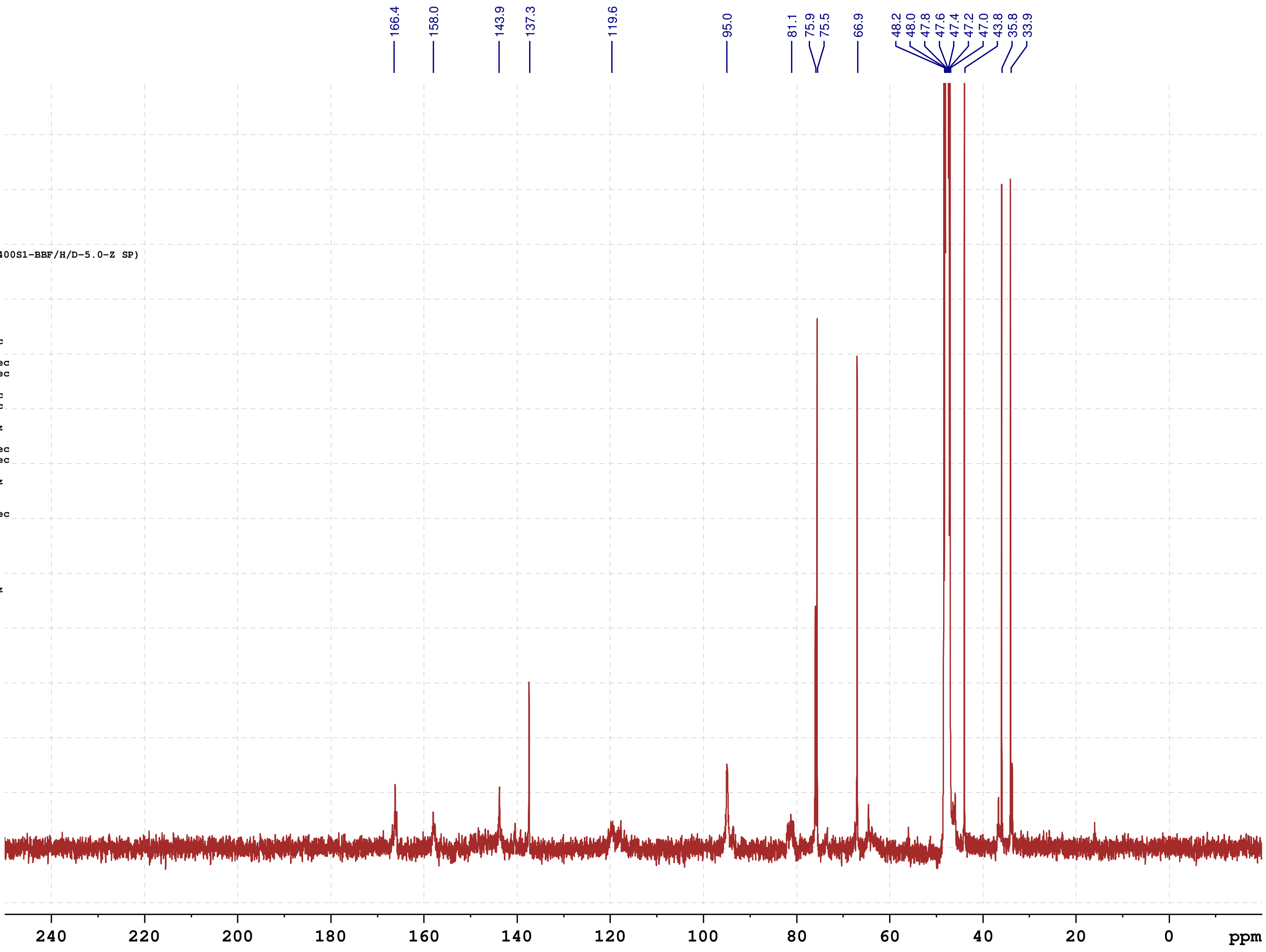

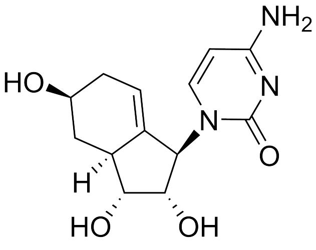

4b

NMR-Spectra for Compound 4c

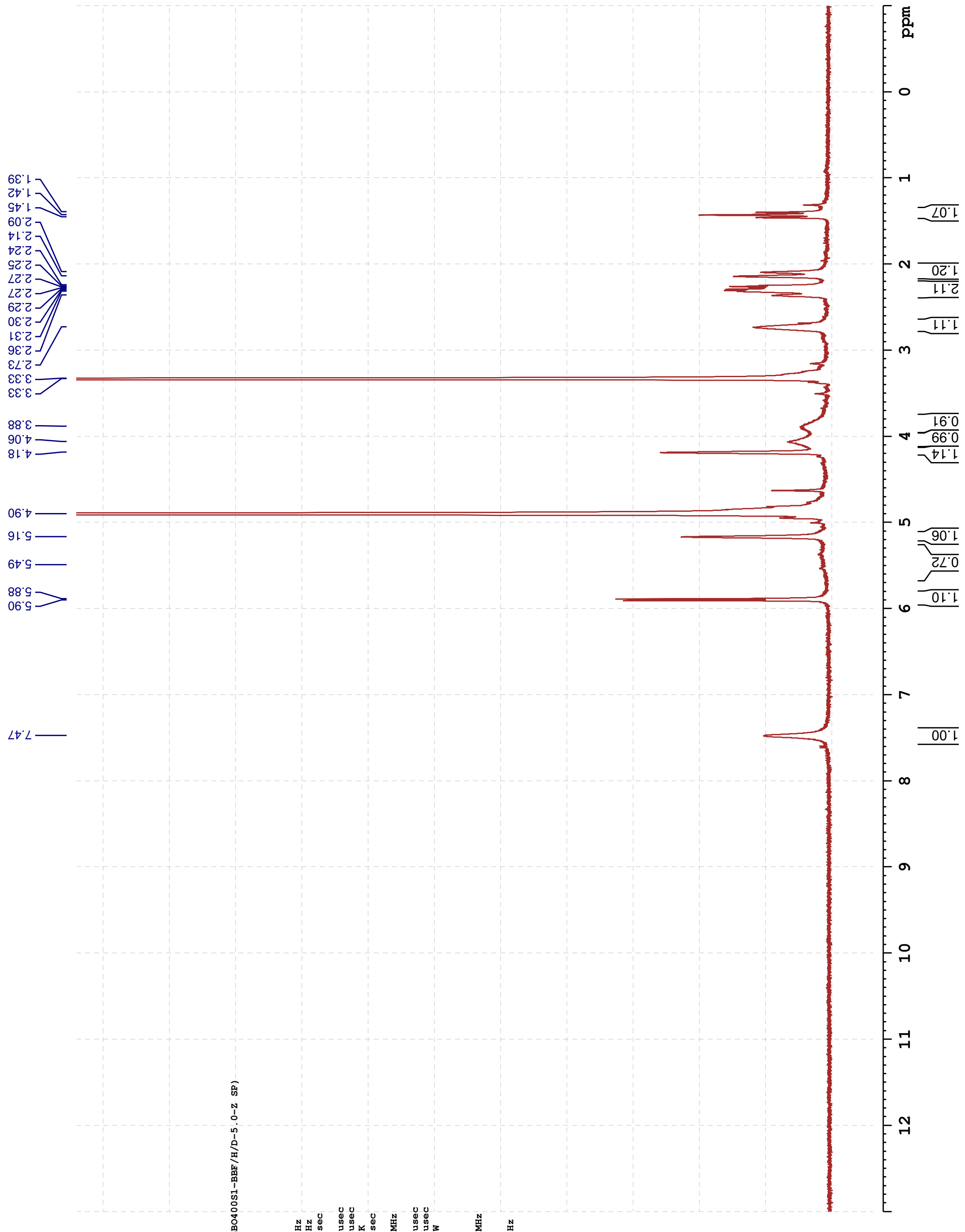

香港中文大學
The Chinese University of Hong Kong

1H-NMR

```
Current Data Parameters
NAME      B-13816-CINNAMON
EXPNO                    1
PROCNO                   1

F2 - Acquisition Parameters
Date_             20240709
Time                  3.09
INSTRUM      AvanceNeo 400
PROBHD    Z163739_0214 (PI HR-BBO400S1-BBF/H/D-5.0-Z SP)
PULPROG               zg30
TD                   65536
SOLVENT               MeOD
NS                      24
DS                       0
SWH          10000.000 Hz
FIDRES        0.305176 Hz
AQ           3.2767999 sec
RG                      32
DW              50.000 usec
DE                9.94 usec
TE               295.6 K
D1          1.00000000 sec
TD0                      1
SFO1      400.3736033 MHz
NUC1                    1H
P0                5.00 usec
P1               15.00 usec
PLW1        6.00000000 W

F2 - Processing parameters
SI                   65536
SF        400.3700000 MHz
WDW                     EM
SSB                      0
LB                0.30 Hz
GB                       0
PC                    1.00
```

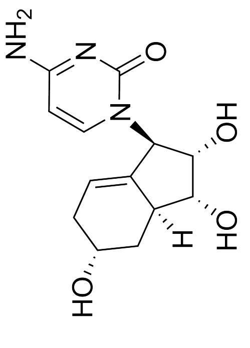

4c

NMR-Spectra for Compound 4c

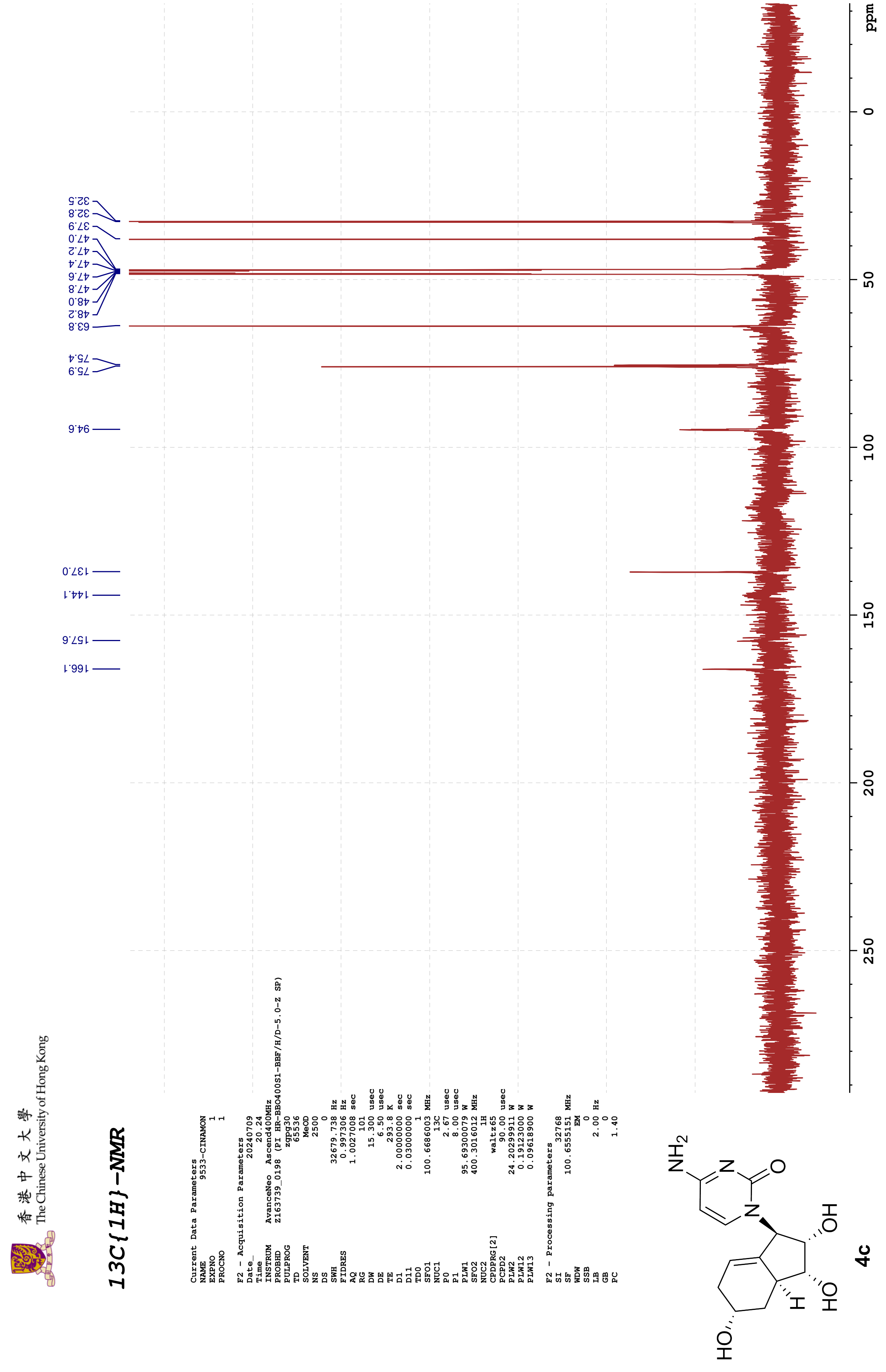

NMR-Spectra for Compound 4d

香港中文大學
The Chinese University of Hong Kong

1H-NMR

```
Current Data Parameters
NAME          C-16494-CINNAMON
EXPNO                        1
PROCNO                       1

F2 - Acquisition Parameters
Date_                 20241220
Time                      7.59
INSTRUM         AvanceNeo 400
PROBHD     Z108621_0062 (PA DUL 400S1 C-H-D-05 Z PLUS)
PULPROG                   zg30
TD                       65536
SOLVENT                   DMSO
NS                          24
DS                           0
SWH                  10000.000 Hz
FIDRES                0.305176 Hz
AQ                   3.2767999 sec
RG                         101
DW                      50.000 usec
DE                       10.79 usec
TE                       295.0 K
D1                  1.00000000 sec
TD0                          1
SFO1               400.3736033 MHz
NUC1                        1H
P0                        3.33 usec
P1                       10.00 usec
PLW1               21.85799980 W

F2 - Processing parameters
SI                       65536
SF                 400.3700000 MHz
WDW                         EM
SSB                          0
LB                        0.30 Hz
GB                           0
PC                        1.00
```

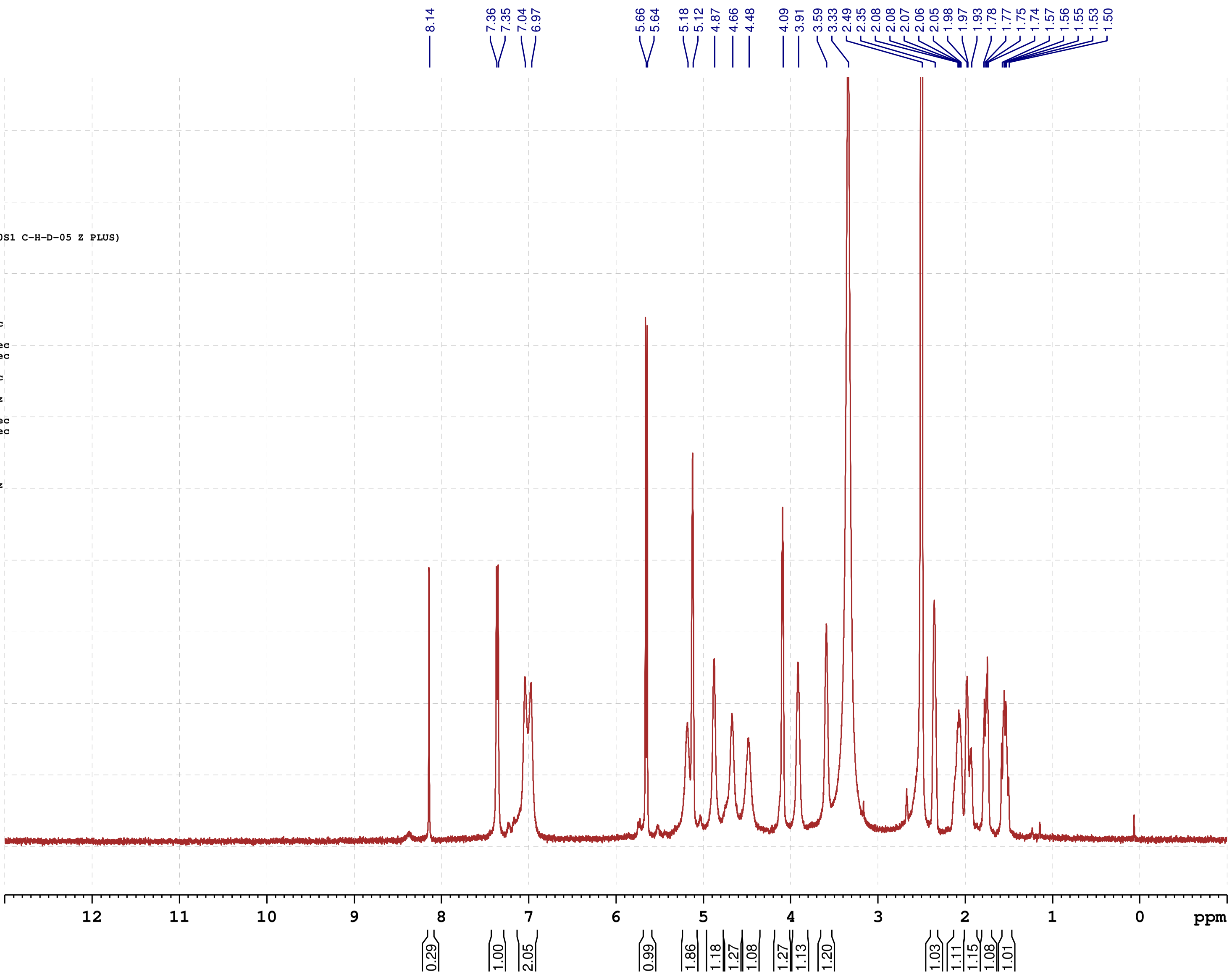

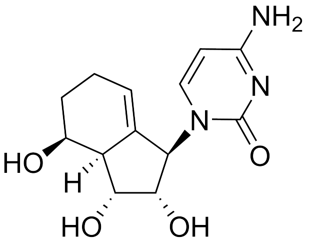

4d

NMR-Spectra for Compound 4d

香港中文大學
The Chinese University of Hong Kong

13C{1H}-NMR

```
Current Data Parameters
NAME          C-17114-CINNAMON-cmr
EXPNO                            1
PROCNO                           1

F2 - Acquisition Parameters
Date_                     20241226
Time                          5.11
INSTRUM                     Avance
PROBHD      Z163739_1043 (PI HR-BBO400S1-BBF/H/D-5.0-Z SP)
PULPROG                    zgpg30
TD                           65536
SOLVENT                       DMSO
NS                            1700
DS                               0
SWH                      32679.739 Hz
FIDRES                    0.997306 Hz
AQ                       1.0027008 sec
RG                            12.7
DW                          15.300 usec
DE                            6.50 usec
TE                           295.4 K
D1                      2.00000000 sec
D11                     0.03000000 sec
TD0                              1
SFO1                   100.7490748 MHz
NUC1                           13C
P0                            2.67 usec
P1                            8.00 usec
PLW1                   96.23899841 W
SFO2                   400.6216025 MHz
NUC2                            1H
CPDPRG2                    waltz65
PCPD2                        90.00 usec
PLW2                   21.54100037 W
PLW12                   0.17020001 W
PLW13                   0.08560800 W

F2 - Processing parameters
SI                           32768
SF                     100.7359791 MHz
WDW                             EM
SSB                              0
LB                            3.00 Hz
GB                               0
PC                            1.40
```

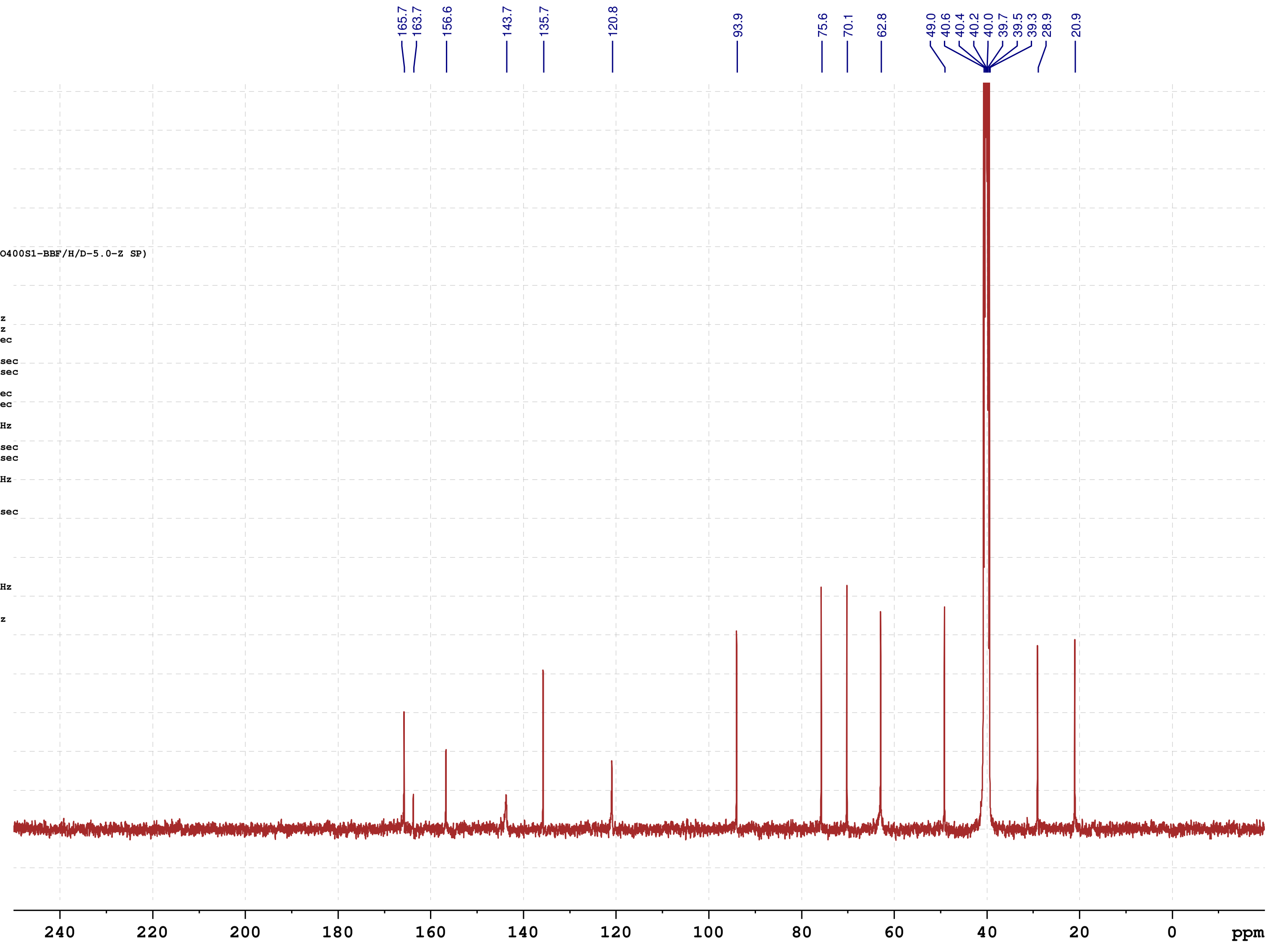

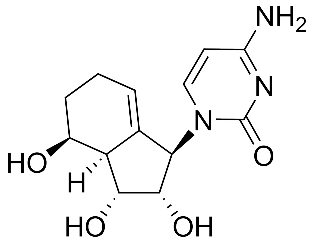

4d

NMR-Spectra for Compound 4e

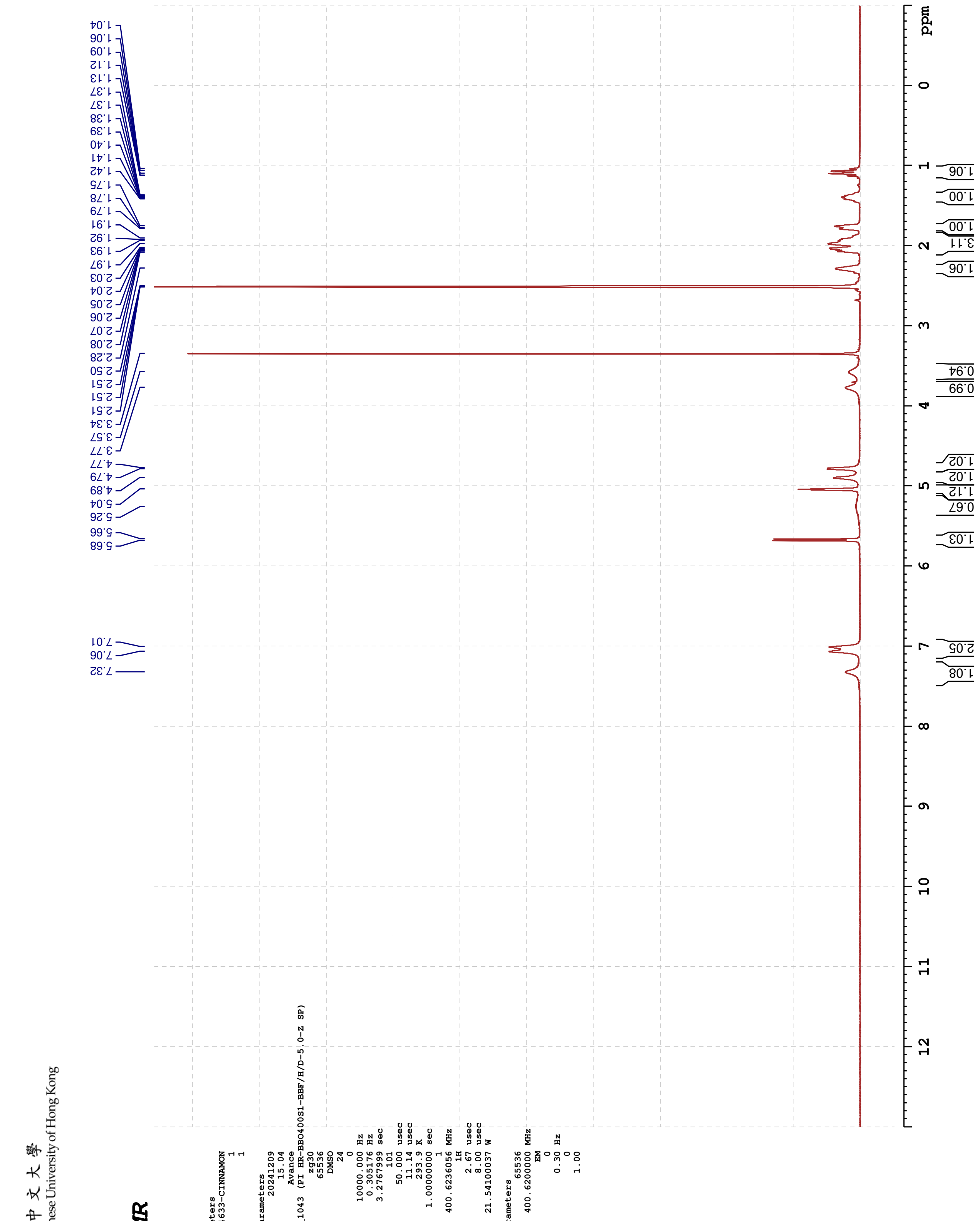

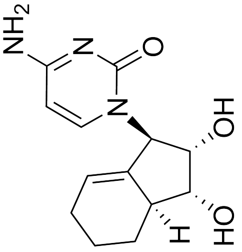

4e

NMR-Spectra for Compound 4e

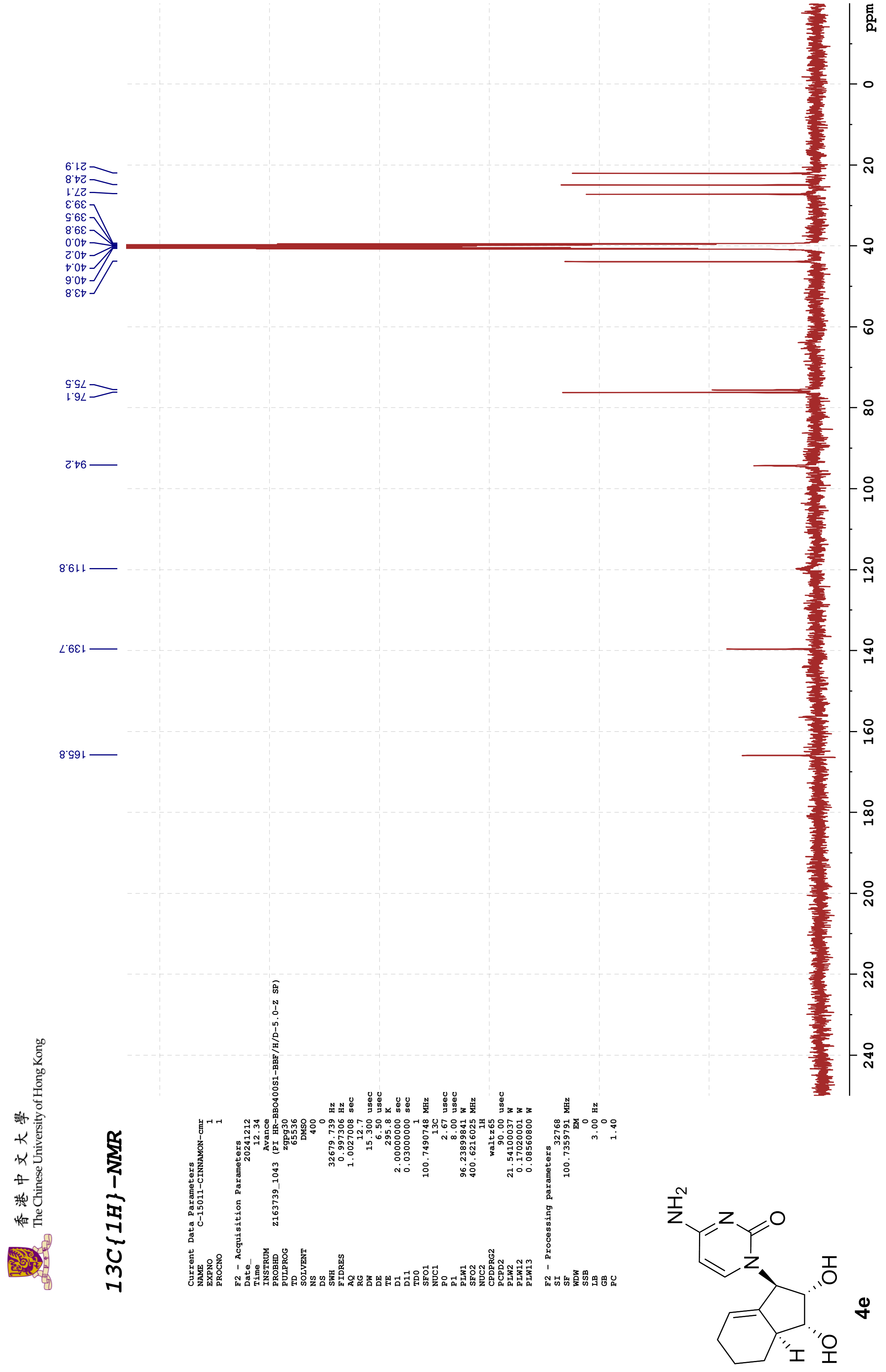

NMR-Spectra for Compound 4f

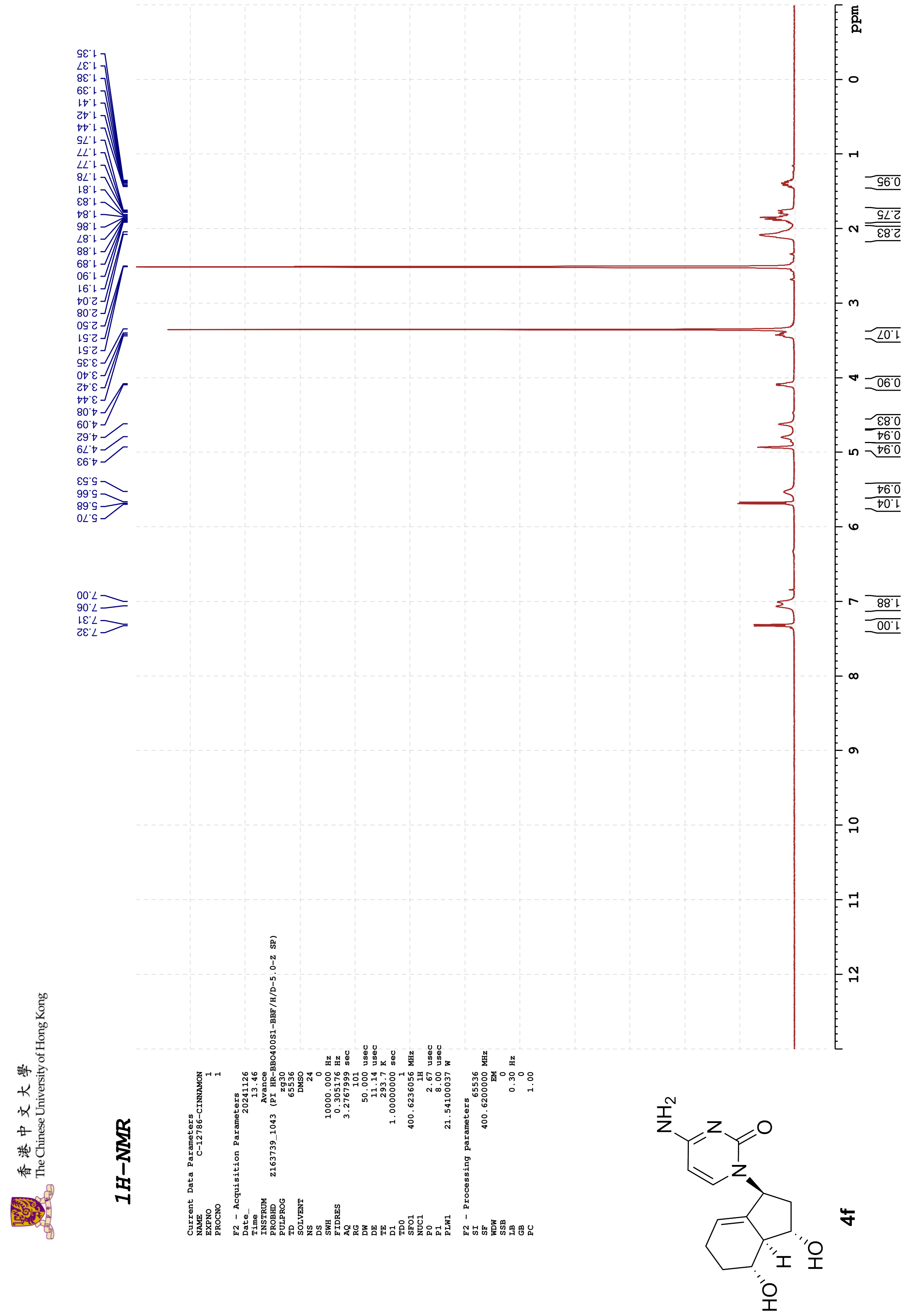

NMR-Spectra for Compound 4f

香港中文大學
The Chinese University of Hong Kong

13C{1H}-NMR

```
Current Data Parameters
NAME          C-12948-CINNAMON
EXPNO                        1
PROCNO                       1

F2 - Acquisition Parameters
Date_                 20241127
Time                     12.14
INSTRUM                 Avance
PROBHD      Z163739_1043 (PI HR-BBO400S1-BBF/H/D-5.0-Z SP)
PULPROG                zgpg30
TD                       65536
SOLVENT                   DMSO
NS                        2048
DS                           0
SWH                  32679.739 Hz
FIDRES                0.997306 Hz
AQ                   1.0027008 sec
RG                        12.7
DW                      15.300 usec
DE                        6.50 usec
TE                       294.8 K
D1                  2.00000000 sec
D11                 0.03000000 sec
TD0                          1
SFO1               100.7490748 MHz
NUC1                       13C
P0                        2.67 usec
P1                        8.00 usec
PLW1               96.23899841 W
SFO2               400.6216025 MHz
NUC2                        1H
CPDPRG2                waltz65
PCPD2                    90.00 usec
PLW2               21.54100037 W
PLW12               0.17020001 W
PLW13               0.08560800 W

F2 - Processing parameters
SI                       32768
SF                 100.7359791 MHz
WDW                         EM
SSB                          0
LB                        3.00 Hz
GB                           0
PC                        1.40
```

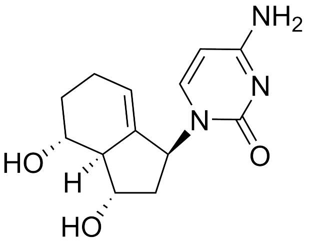

4f

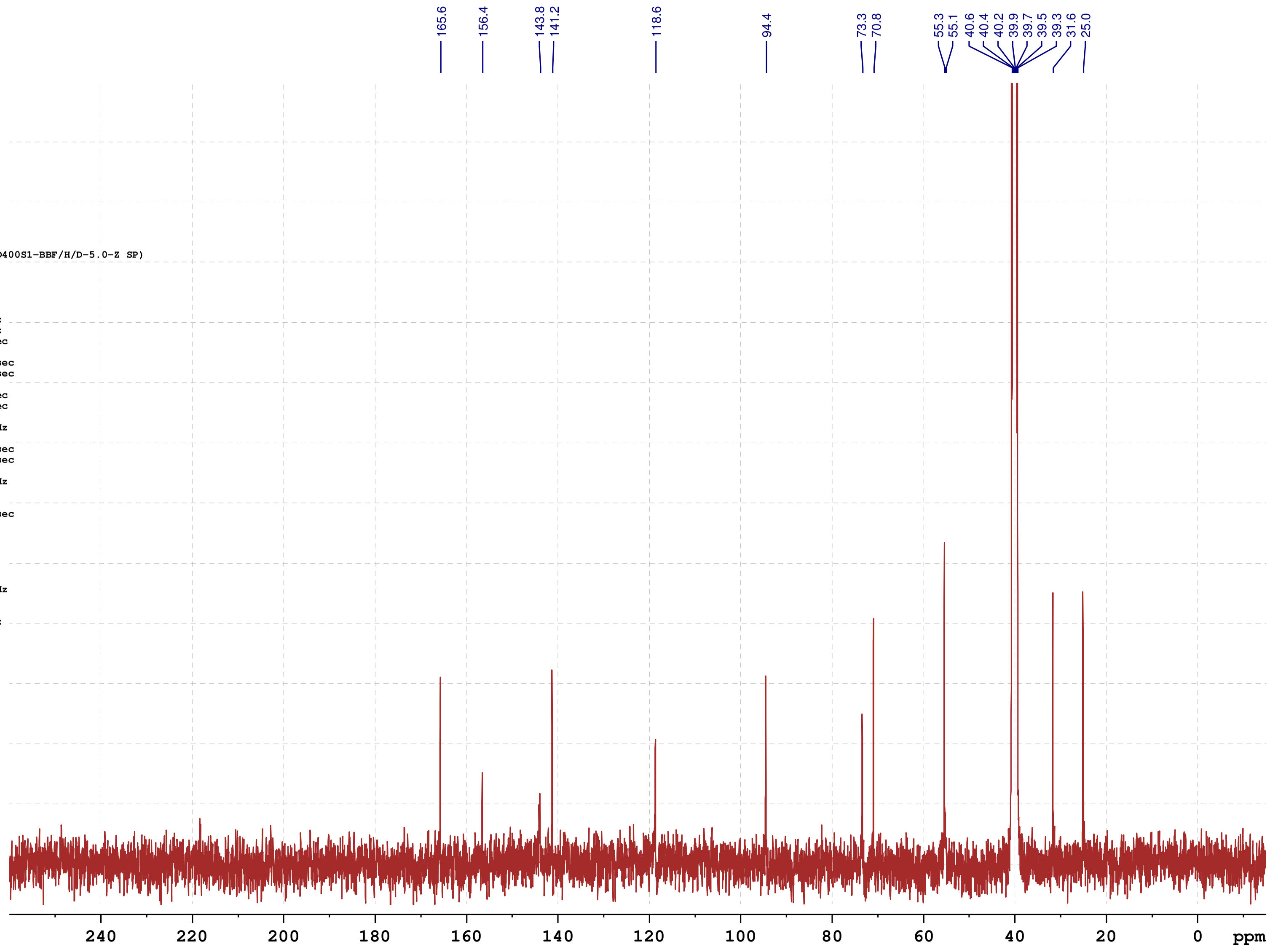

NMR-Spectra for Compound 4g

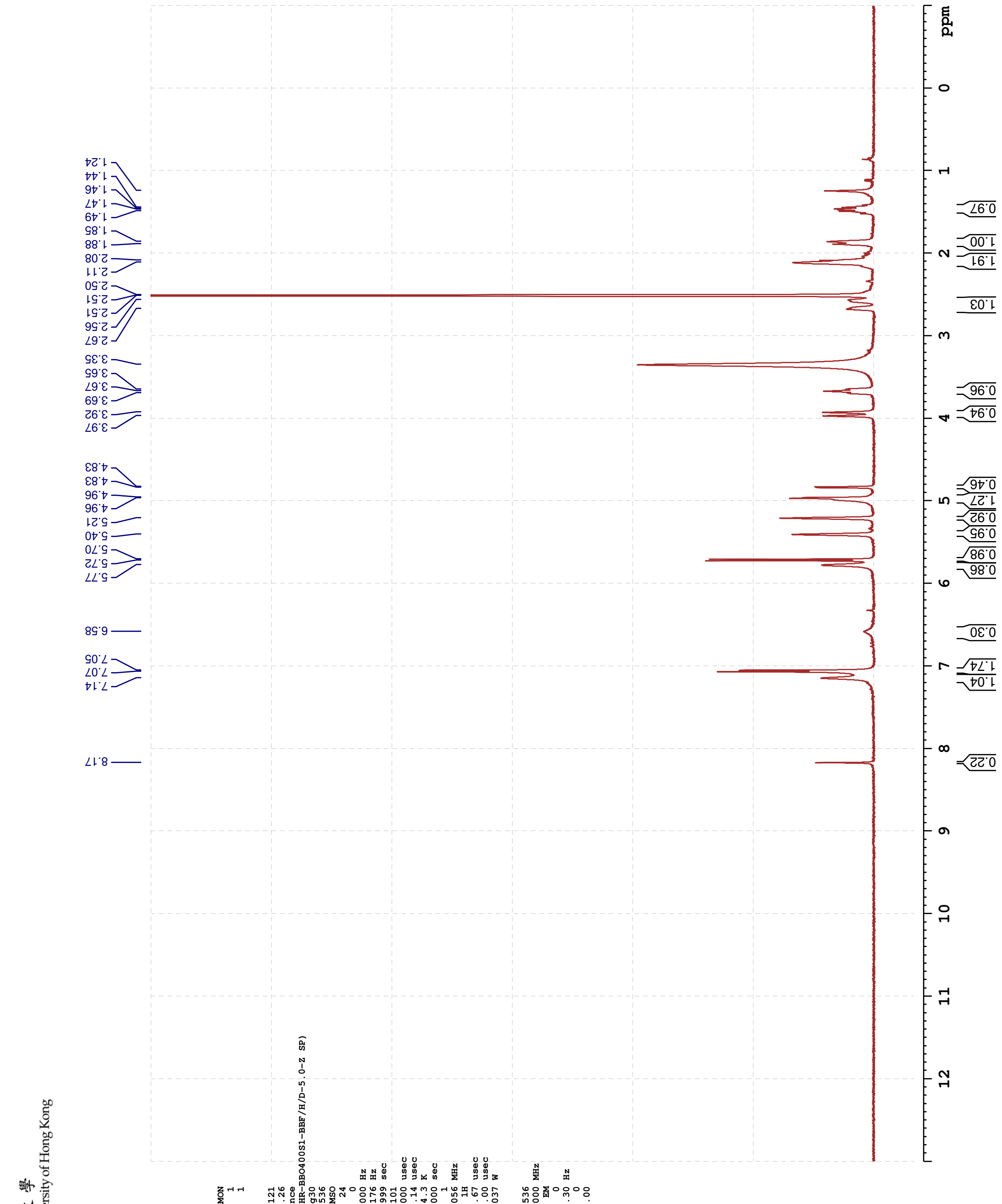

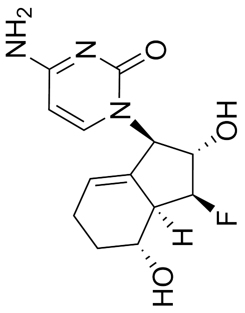

4g

NMR-Spectra for Compound 5a

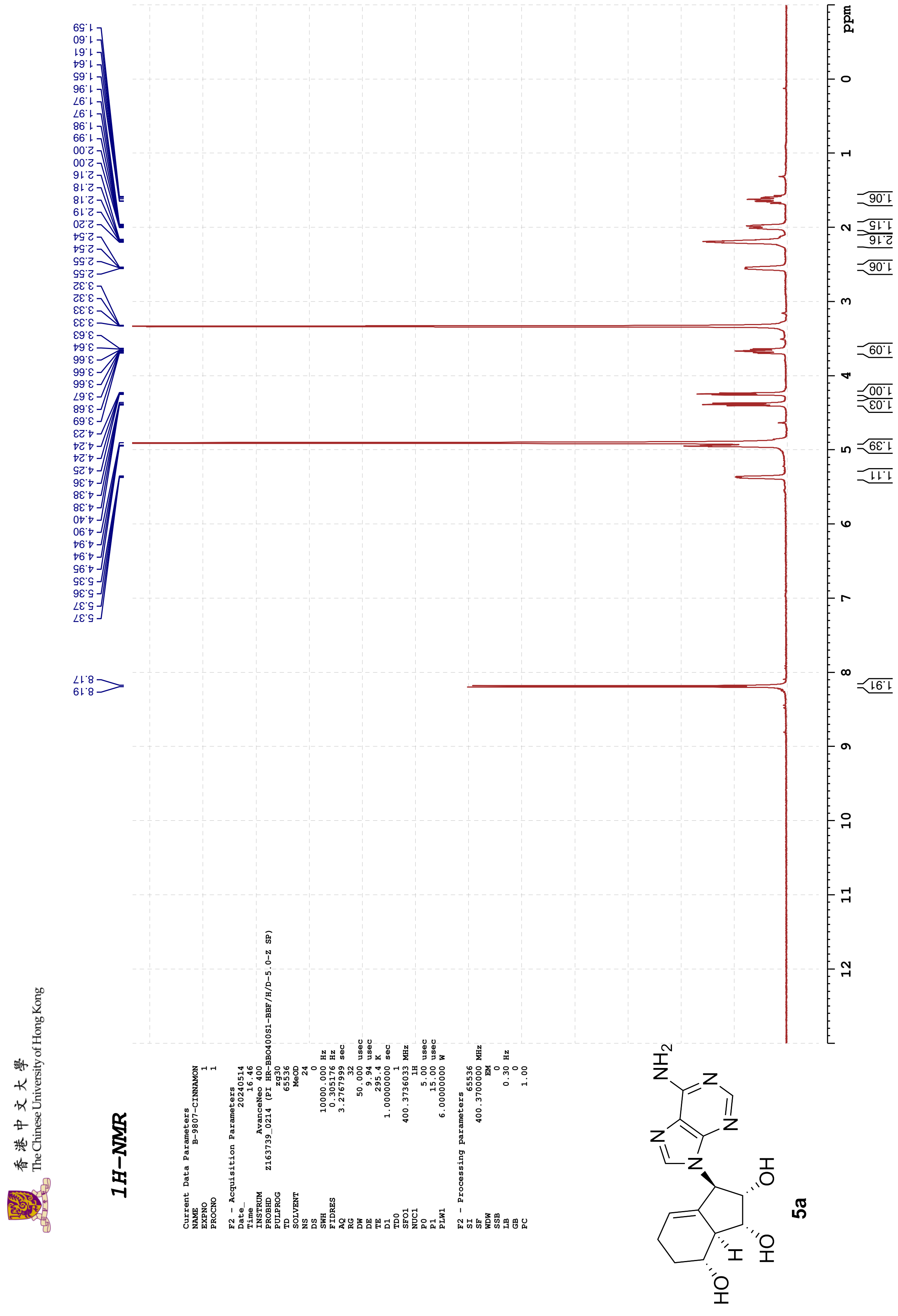

NMR-Spectra for Compound 5a

The Chinese University of Hong Kong

13C{1H}-NMR

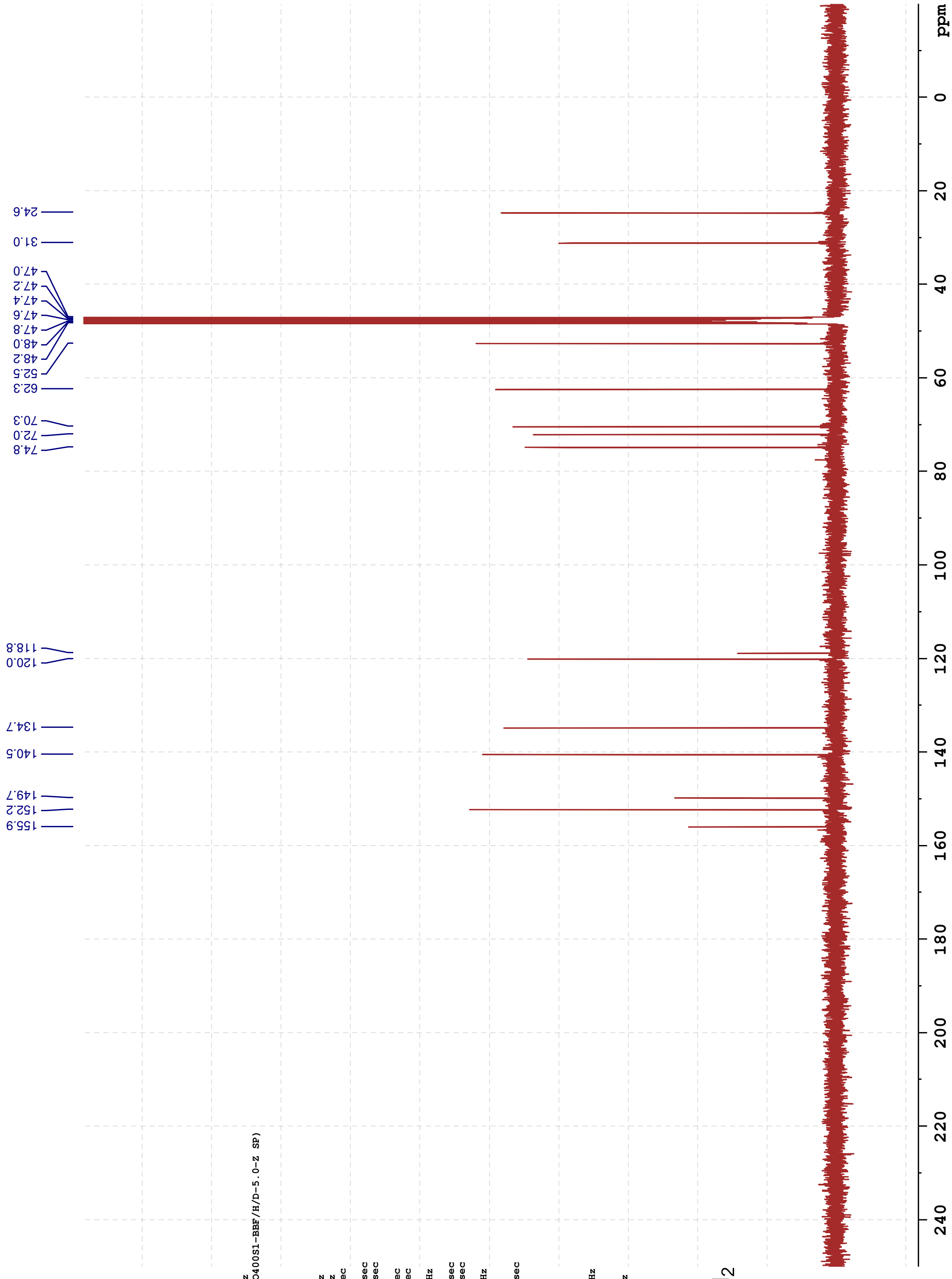


```
Current Data Parameters
NAME              7032-CINNAMON
EXPNO                         1
PROCNO                        1

F2 - Acquisition Parameters
Date_                  20240516
Time                      17.50
INSTRUM    AvanceNeo  Ascend400MHz
PROBHD     Z163739_0198 (PI HR-BBO400S1-BBF/H/D-5.0-Z SP)
PULPROG                 zgpg30
TD                       65536
SOLVENT                   MeOD
NS                        1024
DS                           4
SWH                  32679.738 Hz
FIDRES                0.997306 Hz
AQ                   1.0027008 sec
RG                         101
DW                      15.300 usec
DE                        6.50 usec
TE                       293.6 K
D1                  2.00000000 sec
D11                 0.03000000 sec
TD0                          1
SFO1               100.6686003 MHz
NUC1                       13C
P0                        2.67 usec
P1                        8.00 usec
PLW1               95.69300079 W
SFO2               400.3016012 MHz
NUC2                        1H
CPDPRG[2]              waltz65
PCPD2                    90.00 usec
PLW2               24.20299911 W
PLW12               0.19123000 W
PLW13               0.09618900 W

F2 - Processing parameters
SI                       32768
SF                 100.6555151 MHz
WDW                         EM
SSB                          0
LB                        1.00 Hz
GB                           0
PC                        1.40
```

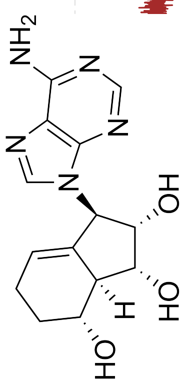

5a

NMR-Spectra for Compound 5b

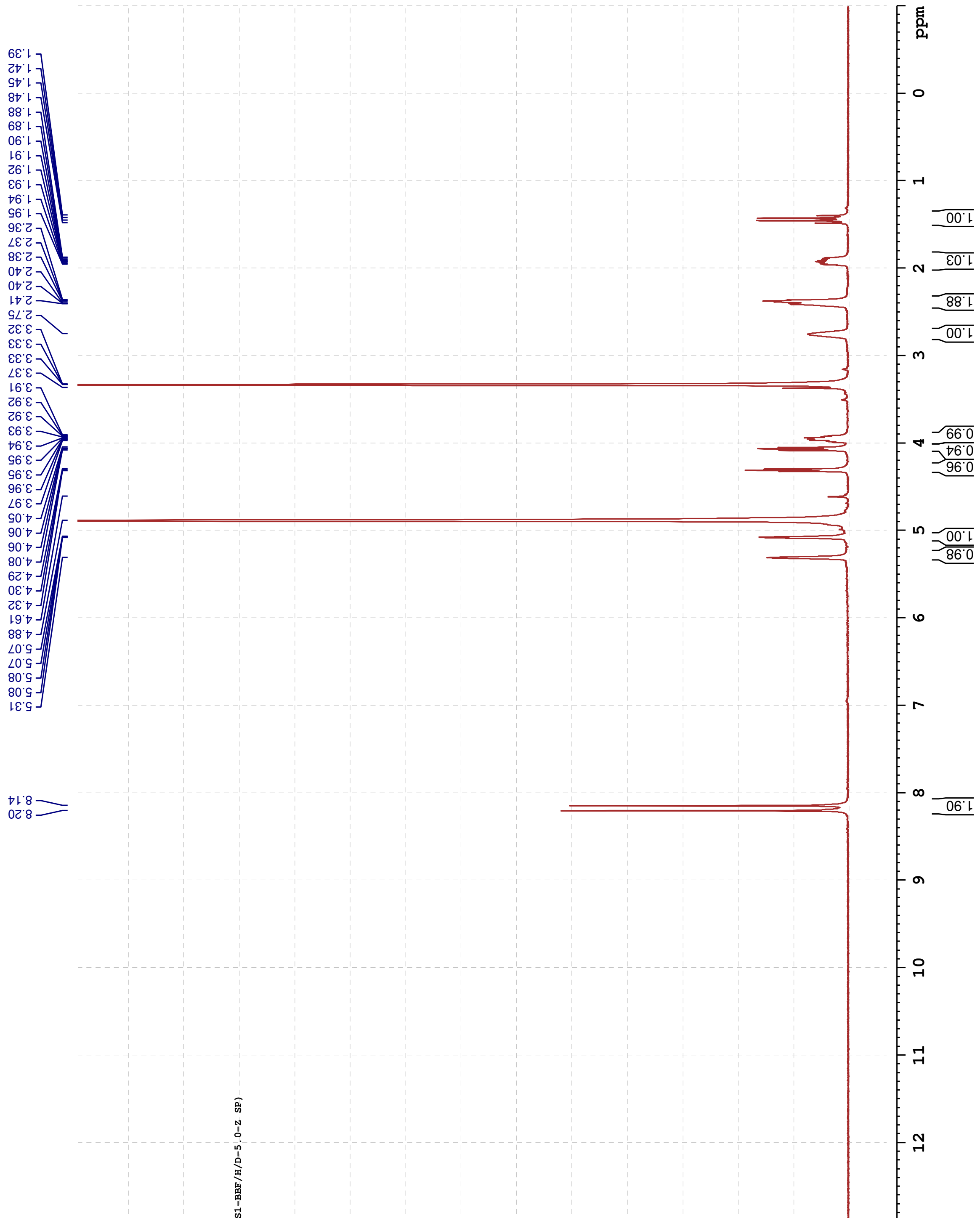

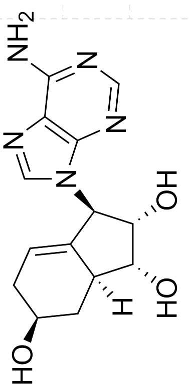

5b

NMR-Spectra for Compound 5b

香港中文大學 The Chinese University of Hong Kong

13C{1H}-NMR

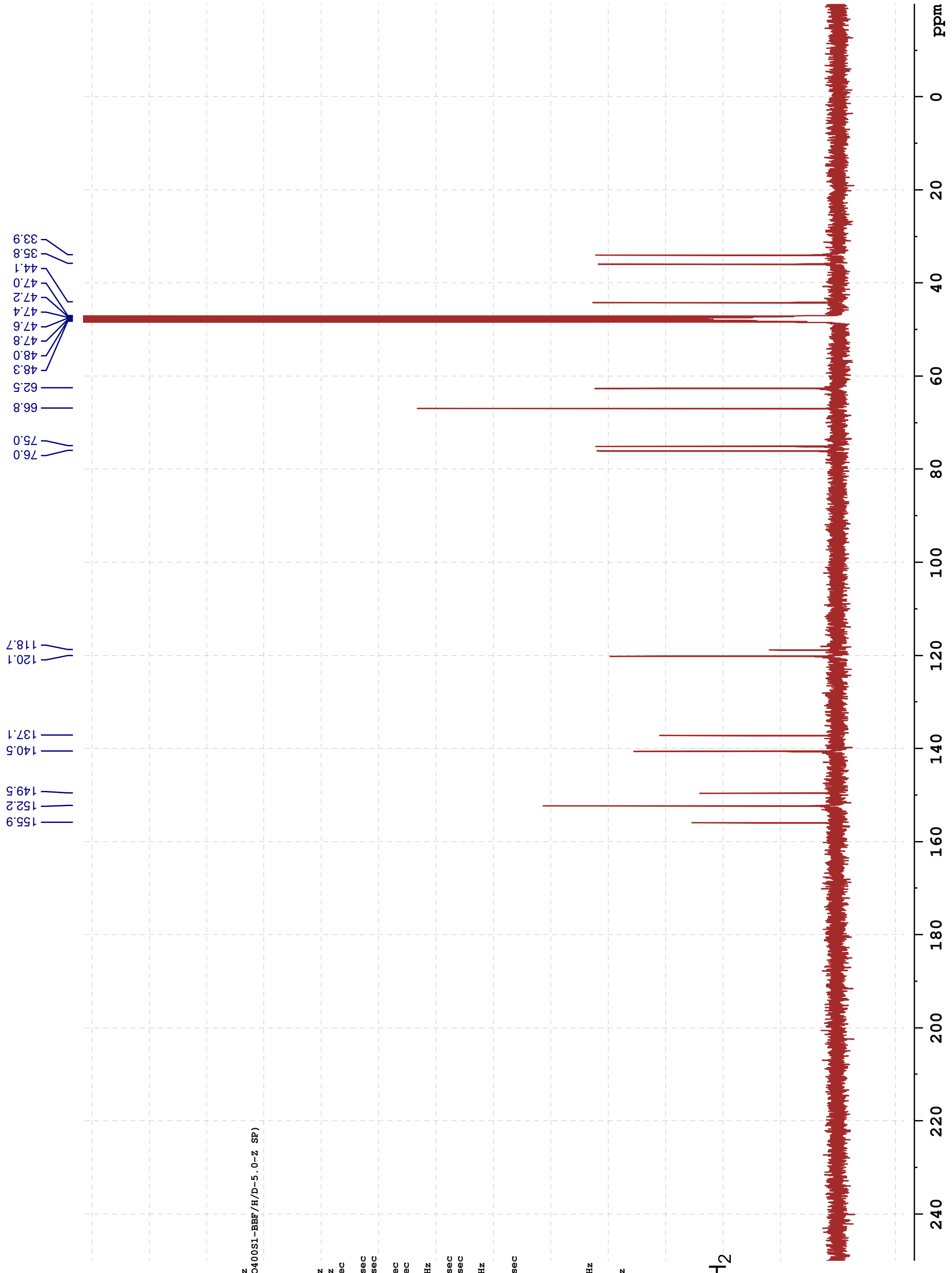


```
Current Data Parameters
NAME          7563-CINNAMON
EXPNO                     1
PROCNO                    1

F2 - Acquisition Parameters
Date_              20240528
Time                  22.00
INSTRUM    AvanceNeo  Ascend400MHz
PROBHD     Z163739_0198 (PI HR-BBO400S1-BBF/H/D-5.0-Z SP)
PULPROG             zgpg30
TD                   65536
SOLVENT               MeOD
NS                    1024
DS                       0
SWH              32679.738 Hz
FIDRES            0.997306 Hz
AQ               1.0027008 sec
RG                     101
DW                  15.300 usec
DE                    6.50 usec
TE                   294.3 K
D1              2.00000000 sec
D11             0.03000000 sec
TD0                      1
SFO1           100.6686003 MHz
NUC1                   13C
P0                    2.67 usec
P1                    8.00 usec
PLW1           95.69300079 W
SFO2           400.3016012 MHz
NUC2                    1H
CPDPRG[2]          waltz65
PCPD2                90.00 usec
PLW2           24.20299911 W
PLW12           0.19123000 W
PLW13           0.09618900 W

F2 - Processing parameters
SI                   32768
SF             100.6555151 MHz
WDW                     EM
SSB                      0
LB                    1.00 Hz
GB                       0
PC                    1.40
```

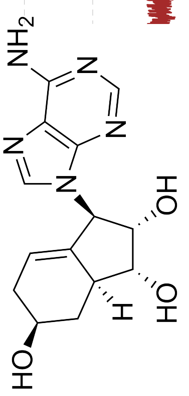

5b

NMR-Spectra for Compound 5c

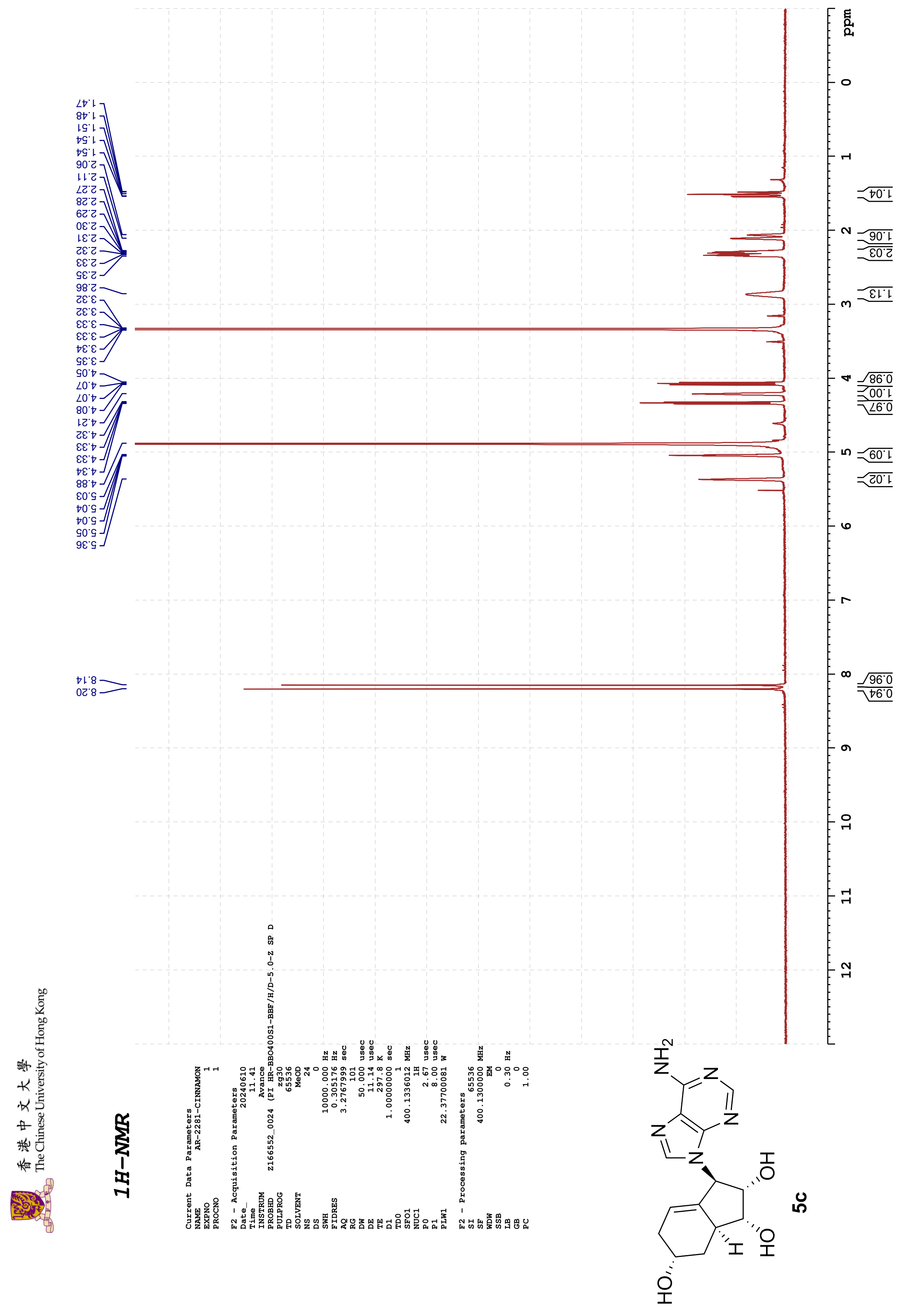

NMR-Spectra for Compound 5c

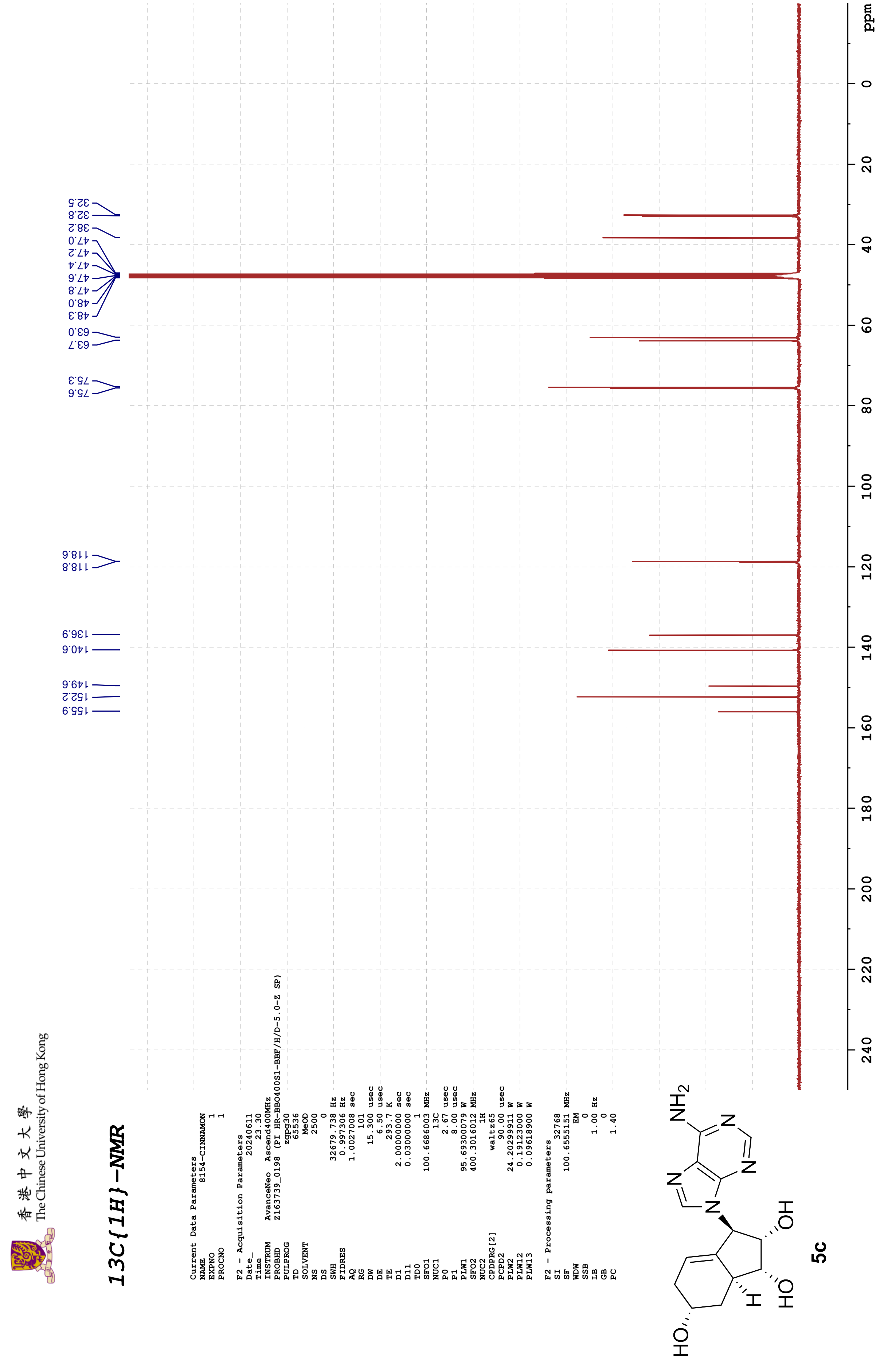

NMR-Spectra for Compound 5d

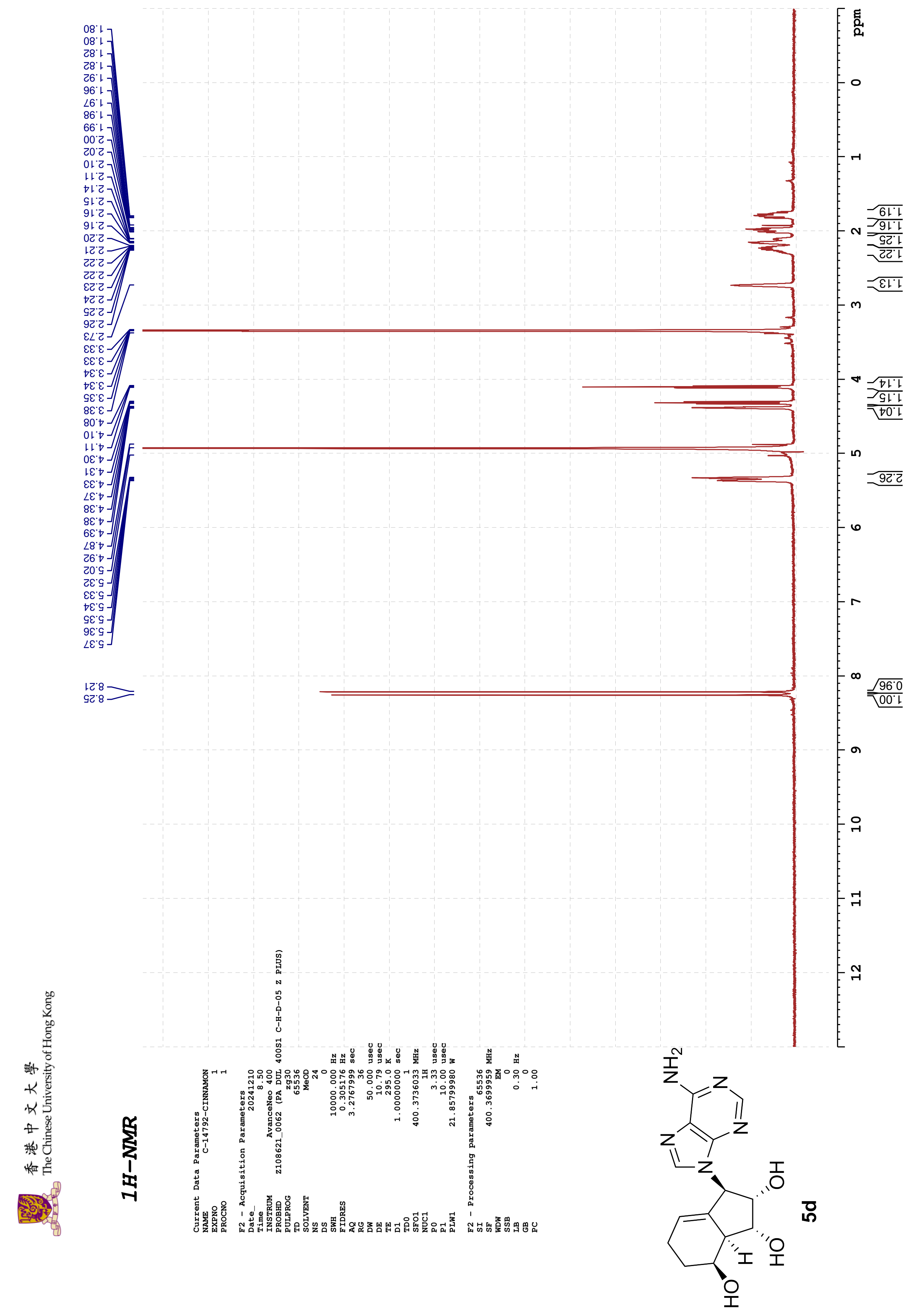

NMR-Spectra for Compound 5d

香港中文大學
The Chinese University of Hong Kong

13C{1H}-NMR

```
Current Data Parameters
NAME        C-15012-CINNAMON-cmr
EXPNO                          1
PROCNO                         1

F2 - Acquisition Parameters
Date_                   20241214
Time                        6.49
INSTRUM                   Avance
PROBHD      Z163739_1043 (PI HR-BBO400S1-BBF/H/D-5.0-Z SP)
PULPROG                  zgpg30
TD                         65536
SOLVENT                     DMSO
NS                          2000
DS                             0
SWH                    32679.739 Hz
FIDRES                  0.997306 Hz
AQ                     1.0027008 sec
RG                          12.7
DW                        15.300 usec
DE                          6.50 usec
TE                         295.6 K
D1                    2.00000000 sec
D11                   0.03000000 sec
TD0                            1
SFO1                 100.7460527 MHz
NUC1                         13C
P0                          2.67 usec
P1                          8.00 usec
PLW1                 96.23899841 W
SFO2                 400.6216025 MHz
NUC2                          1H
CPDPRG2                  waltz65
PCPD2                      90.00 usec
PLW2                 21.54100037 W
PLW12                 0.17020001 W
PLW13                 0.08560800 W

F2 - Processing parameters
SI                         32768
SF                   100.7359791 MHz
WDW                           EM
SSB                            0
LB                          1.00 Hz
GB                             0
PC                          1.40
```

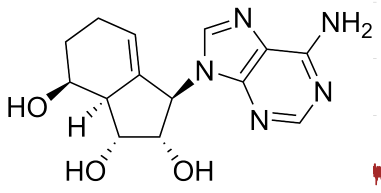

5d

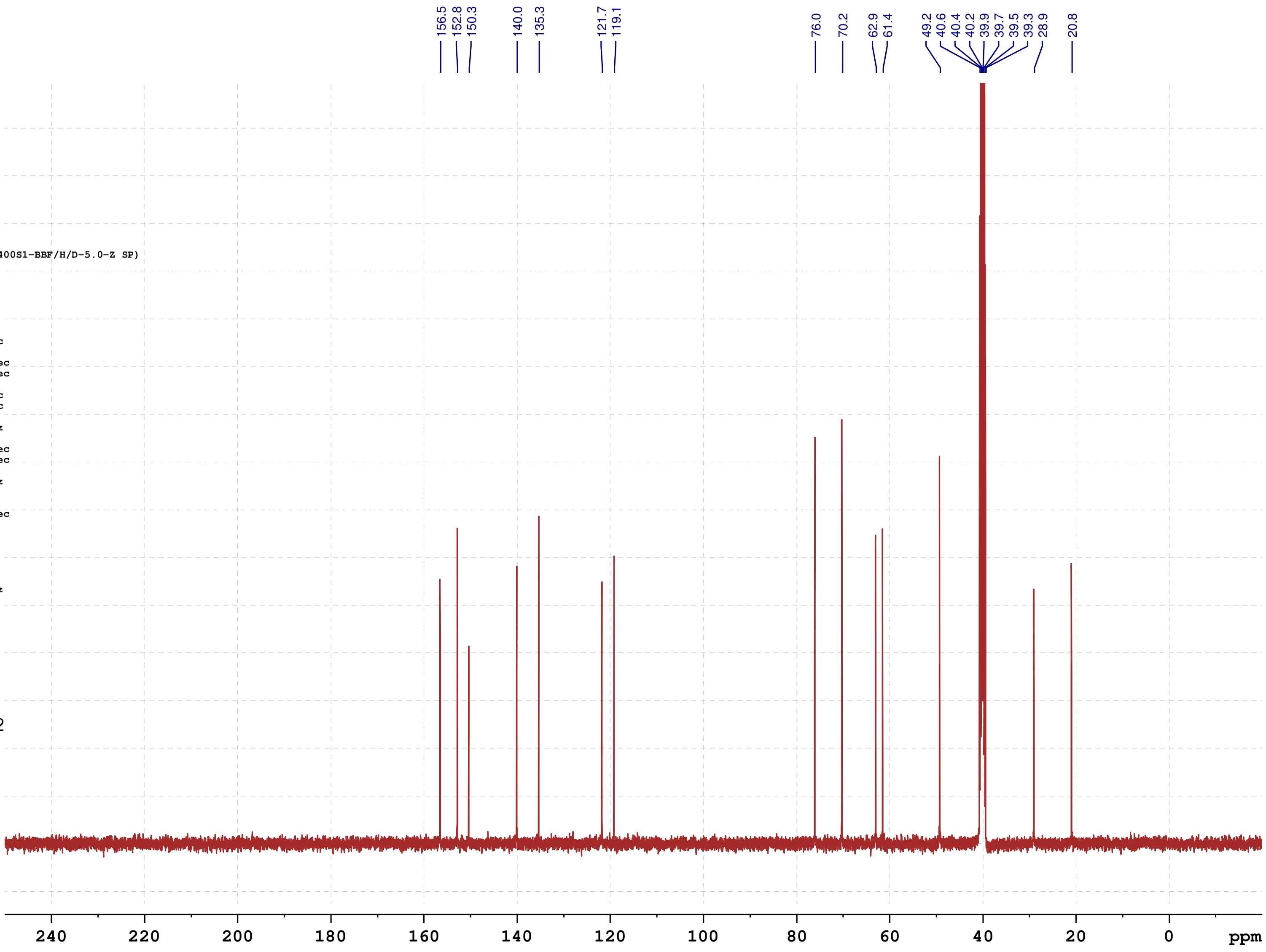

NMR-Spectra for Compound 5e

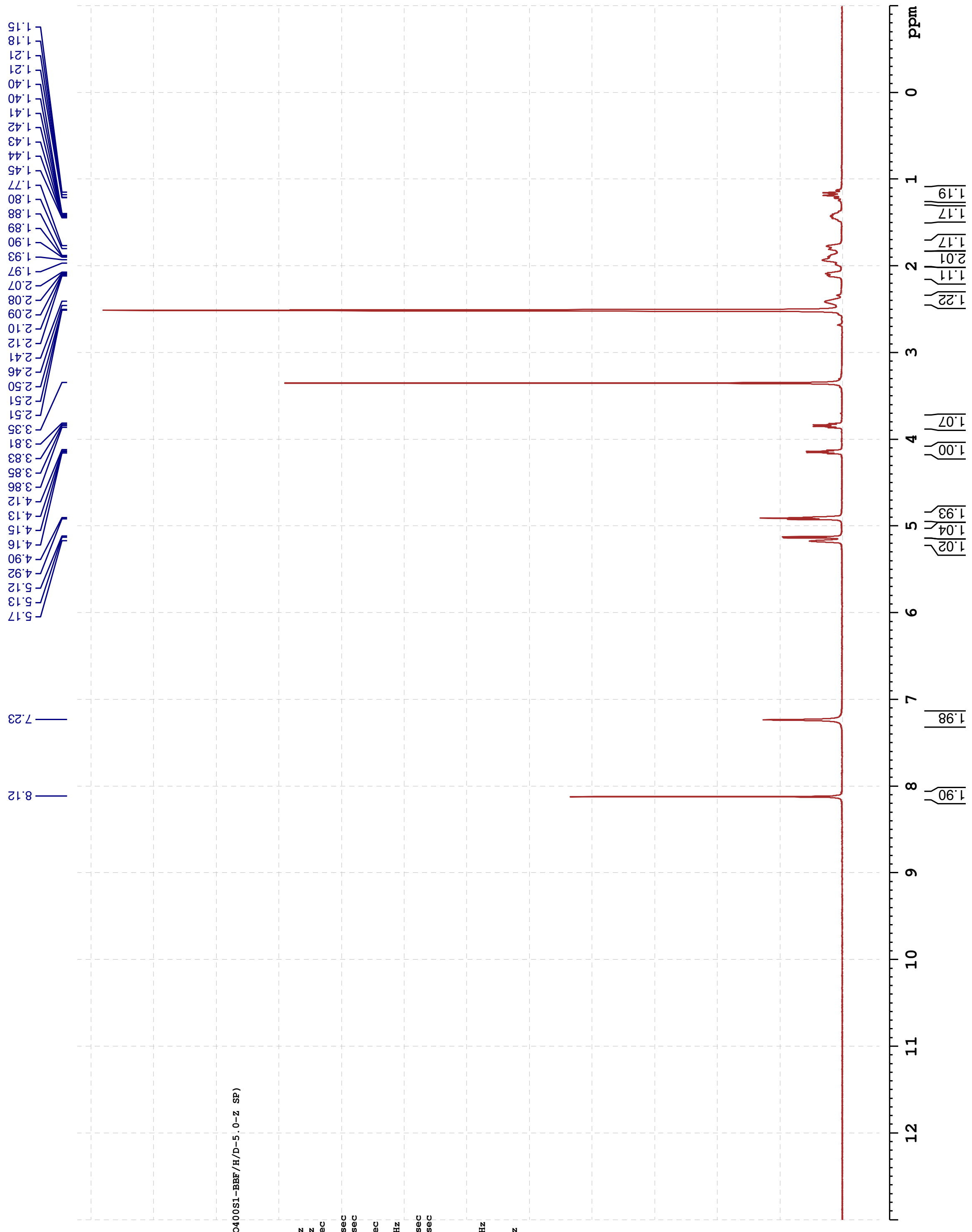

The Chinese University of Hong Kong

1H-NMR

```
Current Data Parameters
NAME          C-12785-CINNAMON
EXPNO                    1
PROCNO                   1

F2 - Acquisition Parameters
Date_             20241126
Time                 13.42
INSTRUM             Avance
PROBHD   Z163739_1043 (PI HR-BBO400S1-BBF/H/D-5.0-Z SP)
PULPROG               zg30
TD                   65536
SOLVENT               DMSO
NS                      24
DS                       0
SWH              10000.000 Hz
FIDRES            0.305176 Hz
AQ               3.2767999 sec
RG                     101
DW                  50.000 usec
DE                   11.14 usec
TE                   293.8 K
D1              1.00000000 sec
TD0                      1
SFO1           400.6236056 MHz
NUC1                    1H
P0                    2.67 usec
P1                    8.00 usec
PLW1           21.54100037 W

F2 - Processing parameters
SI                   65536
SF             400.6200000 MHz
WDW                     EM
SSB                      0
LB                    0.30 Hz
GB                       0
PC                    1.00
```

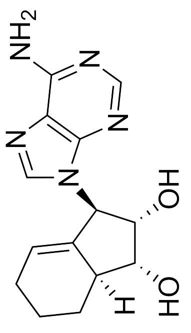

5e

NMR-Spectra for Compound 5e

The Chinese University of Hong Kong

13C{1H}-NMR

```
Current Data Parameters
NAME          C-13136-CINNAMON
EXPNO                        1
PROCNO                       1

F2 - Acquisition Parameters
Date_                 20241128
Time                      4.37
INSTRUM                 Avance
PROBHD     Z163739_1043 (PI HR-BBO400S1-BBF/H/D-5.0-Z SP)
PULPROG                zgpg30
TD                       65536
SOLVENT                   DMSO
NS                        2048
DS                           0
SWH                  32679.739 Hz
FIDRES                0.997306 Hz
AQ                   1.0027008 sec
RG                        12.7
DW                      15.300 usec
DE                        6.50 usec
TE                       294.4 K
D1                  2.00000000 sec
D11                 0.03000000 sec
TD0                          1
SFO1               100.7490748 MHz
NUC1                       13C
P0                        2.67 usec
P1                        8.00 usec
PLW1               96.23899841 W
SFO2               400.6216025 MHz
NUC2                        1H
CPDPRG2                waltz65
PCPD2                    90.00 usec
PLW2               21.54100037 W
PLW12               0.17020001 W
PLW13               0.08560800 W

F2 - Processing parameters
SI                       32768
SF                 100.7359791 MHz
WDW                         EM
SSB                          0
LB                        1.00 Hz
GB                           0
PC                        1.40
```

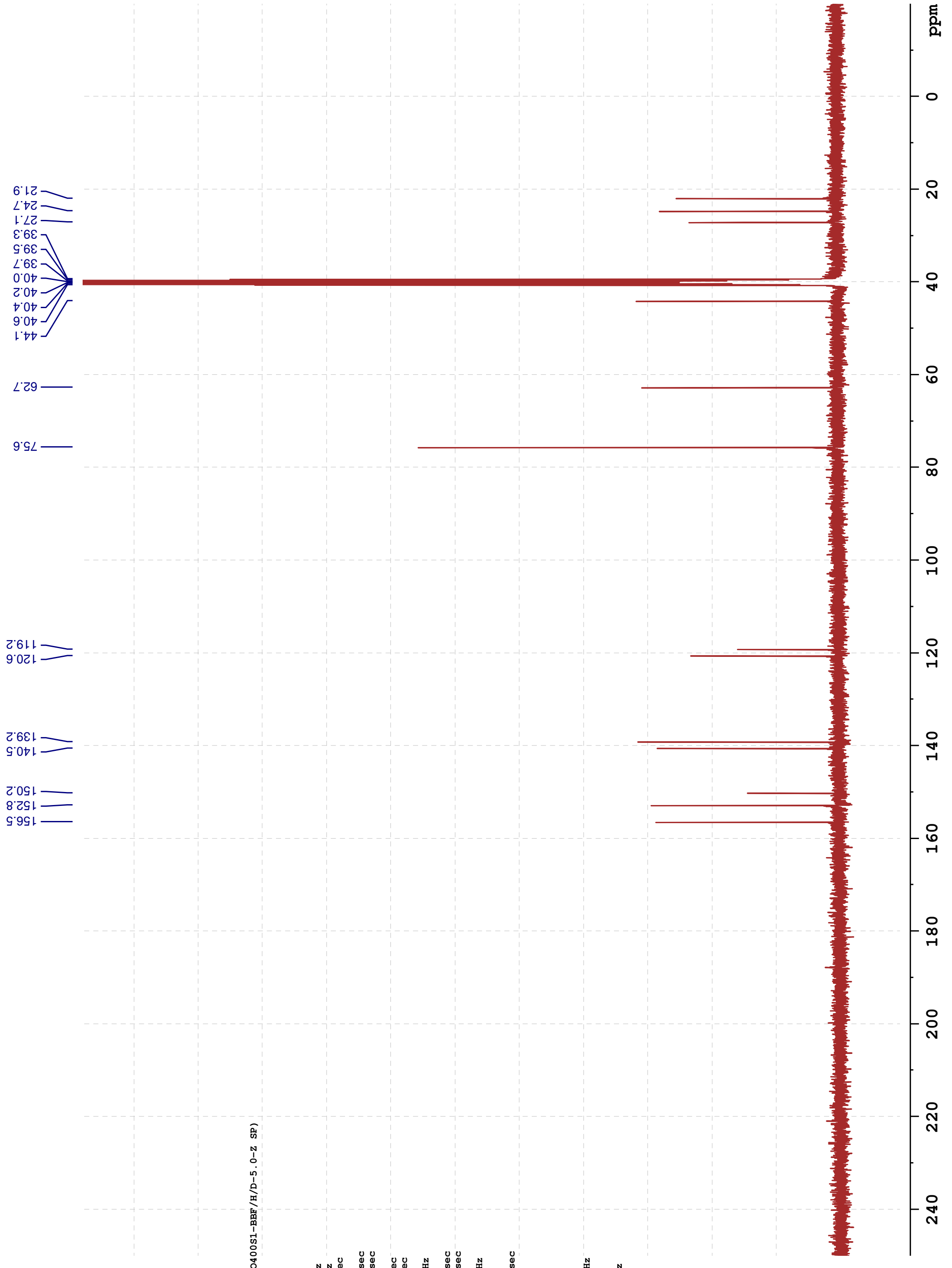

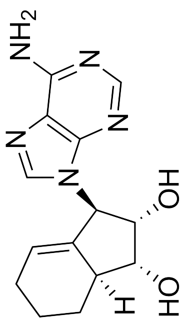

5e

NMR-Spectra for Compound 5f

The Chinese University of Hong Kong

1H-NMR

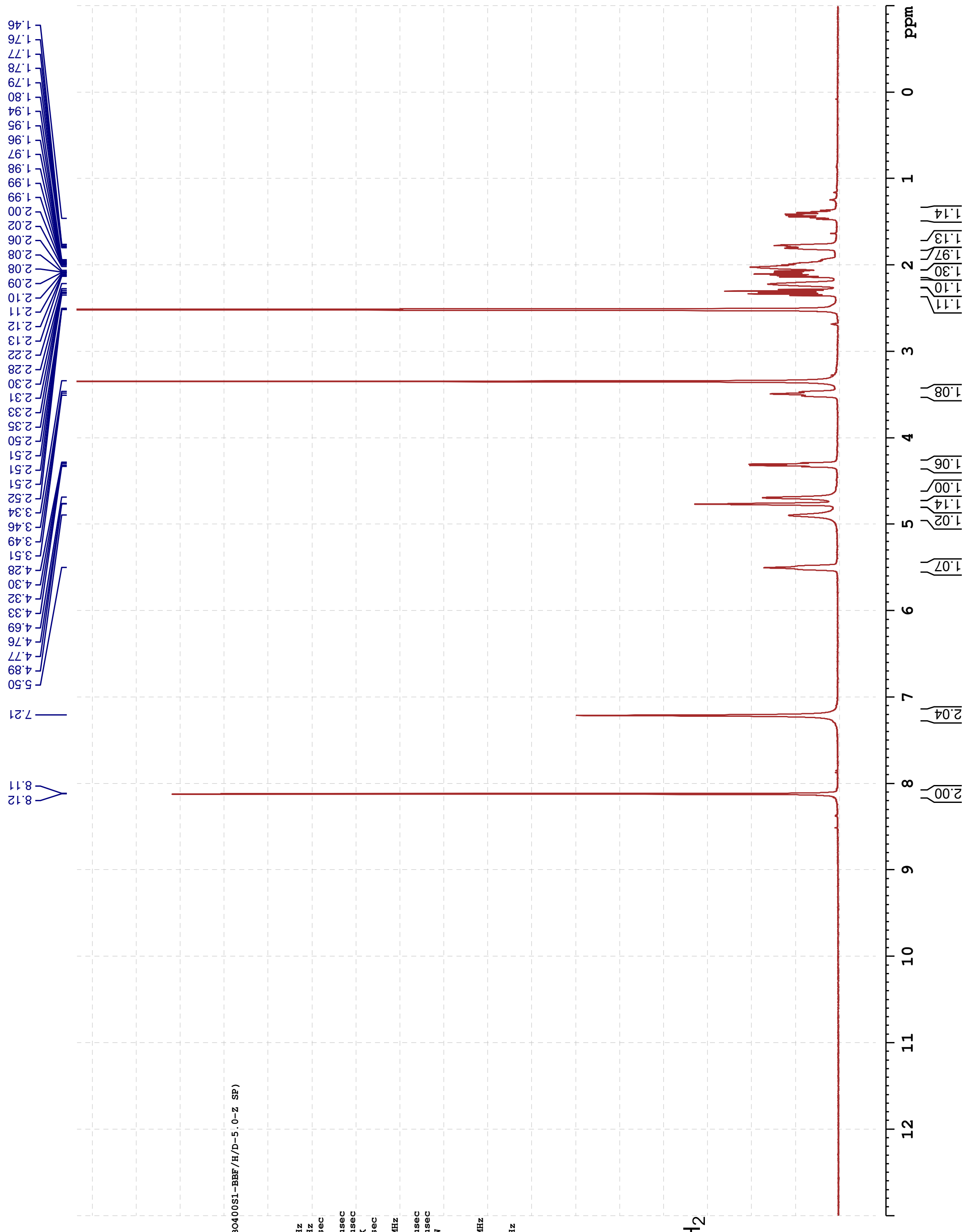


```
Current Data Parameters
NAME          C-9759-CINNAMON
EXPNO                       1
PROCNO                      1

F2 - Acquisition Parameters
Date_                20241105
Time                    13.51
INSTRUM                Avance
PROBHD     Z163739_1043 (PI HR-BBO400S1-BBF/H/D-5.0-Z SP)
PULPROG                  zg30
TD                      65536
SOLVENT                  DMSO
NS                         24
DS                          0
SWH             10000.000 Hz
FIDRES           0.305176 Hz
AQ              3.2767999 sec
RG                        101
DW                 50.000 usec
DE                  11.14 usec
TE                  297.0 K
D1             1.00000000 sec
TD0                         1
SFO1          400.6236056 MHz
NUC1                       1H
P0                   2.67 usec
P1                   8.00 usec
PLW1          25.00000000 W

F2 - Processing parameters
SI                      65536
SF            400.6200000 MHz
WDW                        EM
SSB                         0
LB                      0.30 Hz
GB                          0
PC                       1.00
```

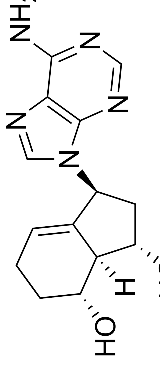

5f

NMR-Spectra for Compound 5f

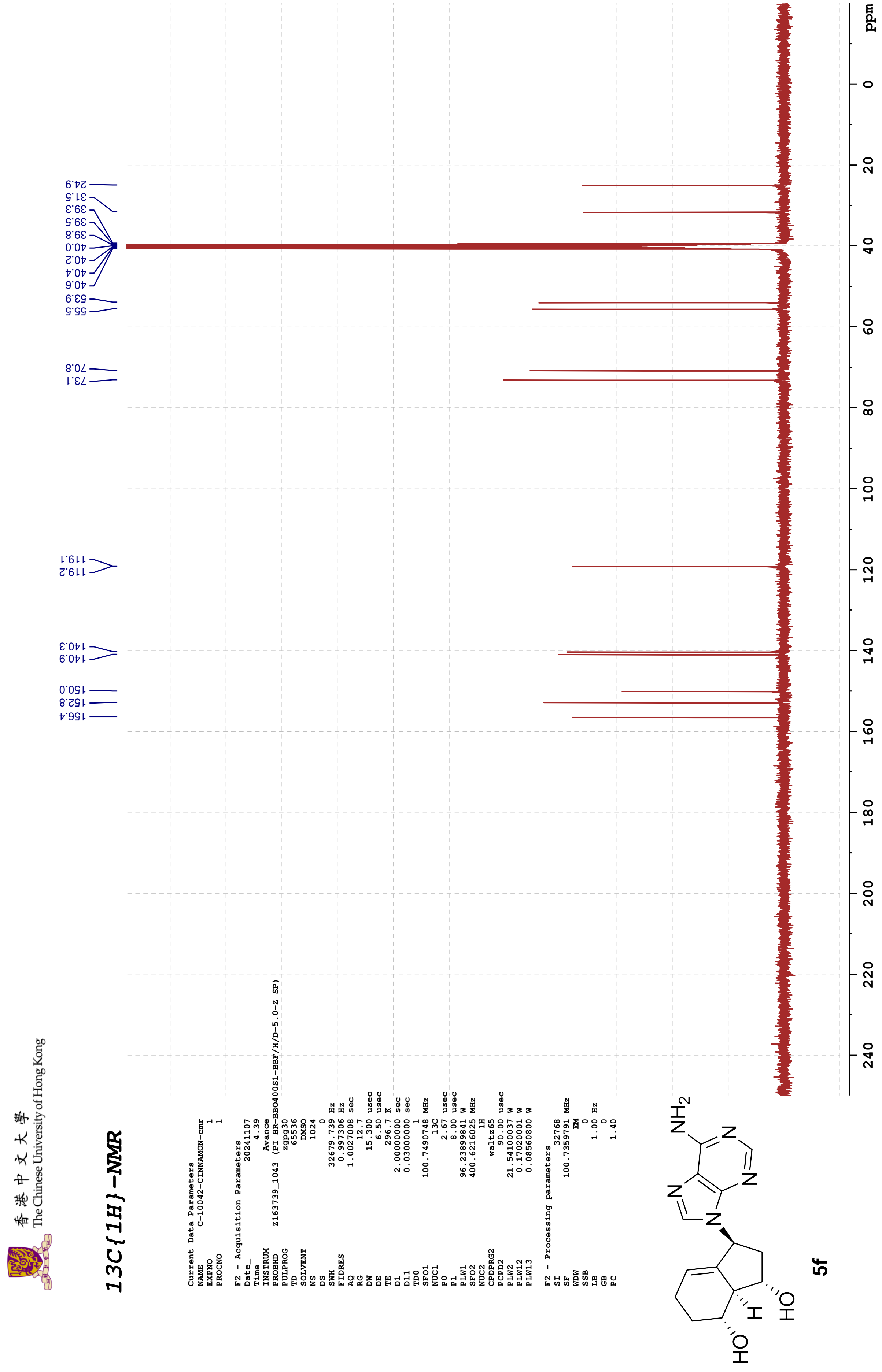

NMR-Spectra for Compound 5g

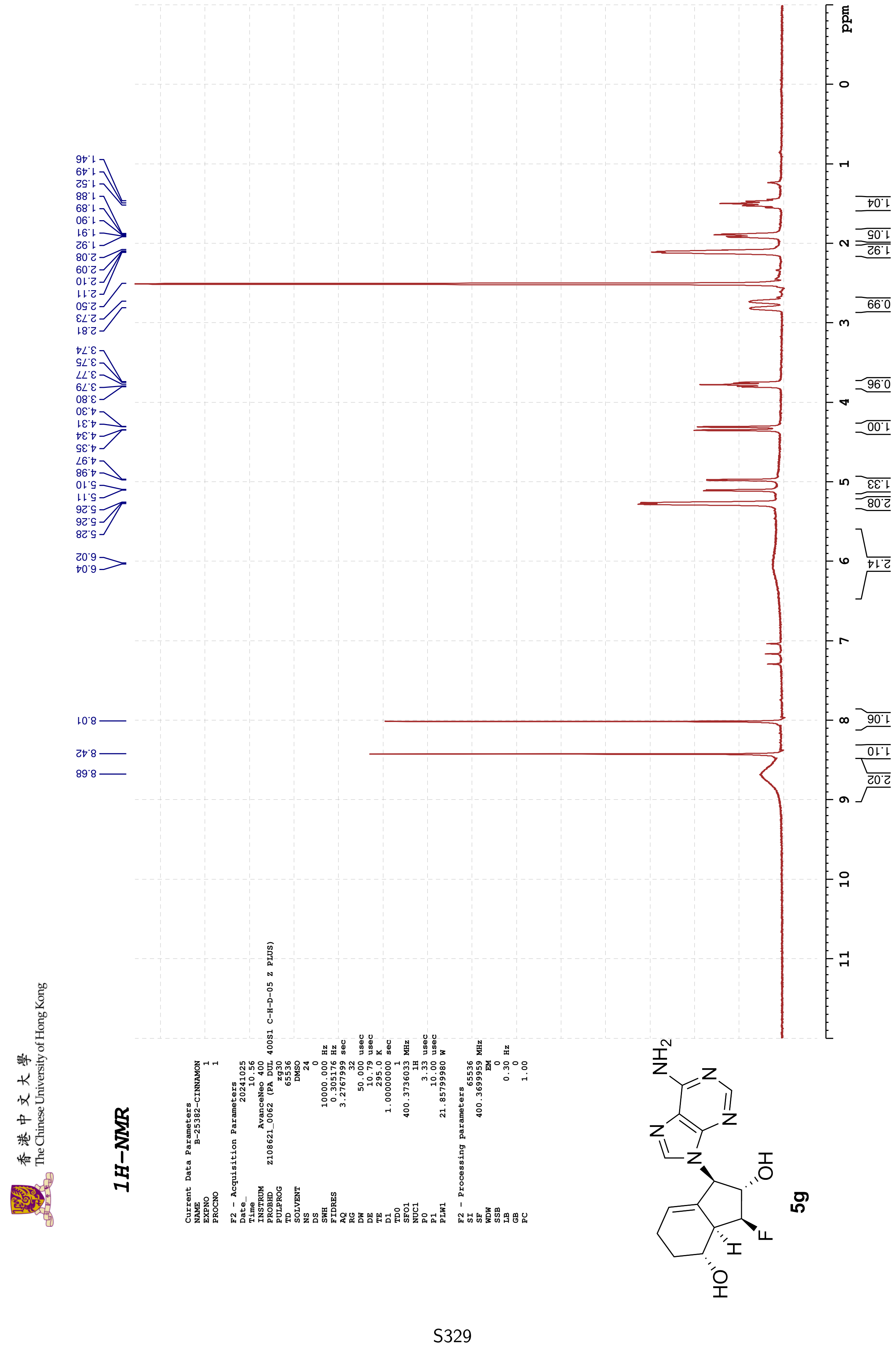

NMR-Spectra for Compound 5g

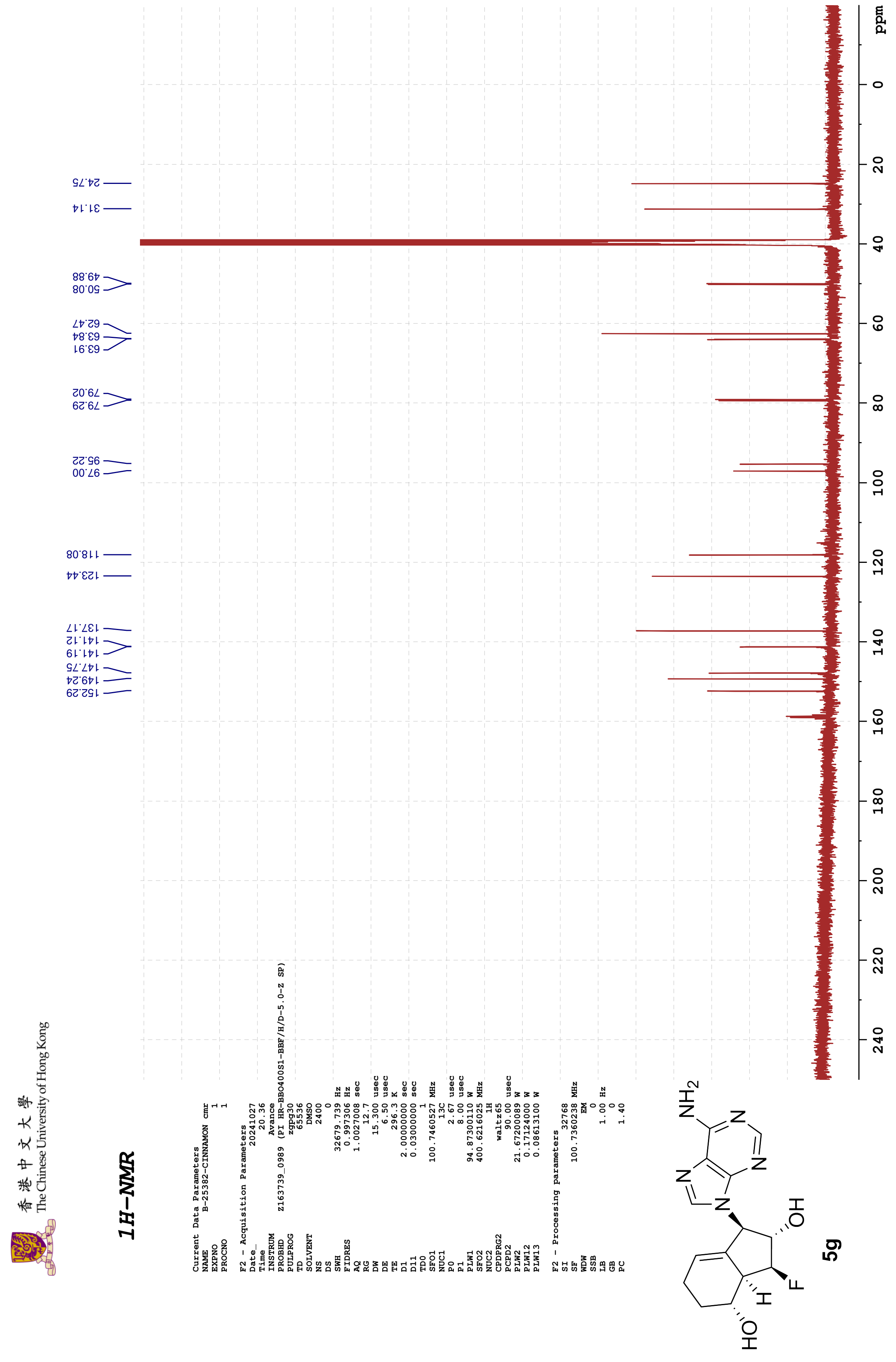

NMR-Spectra for Compound 5h

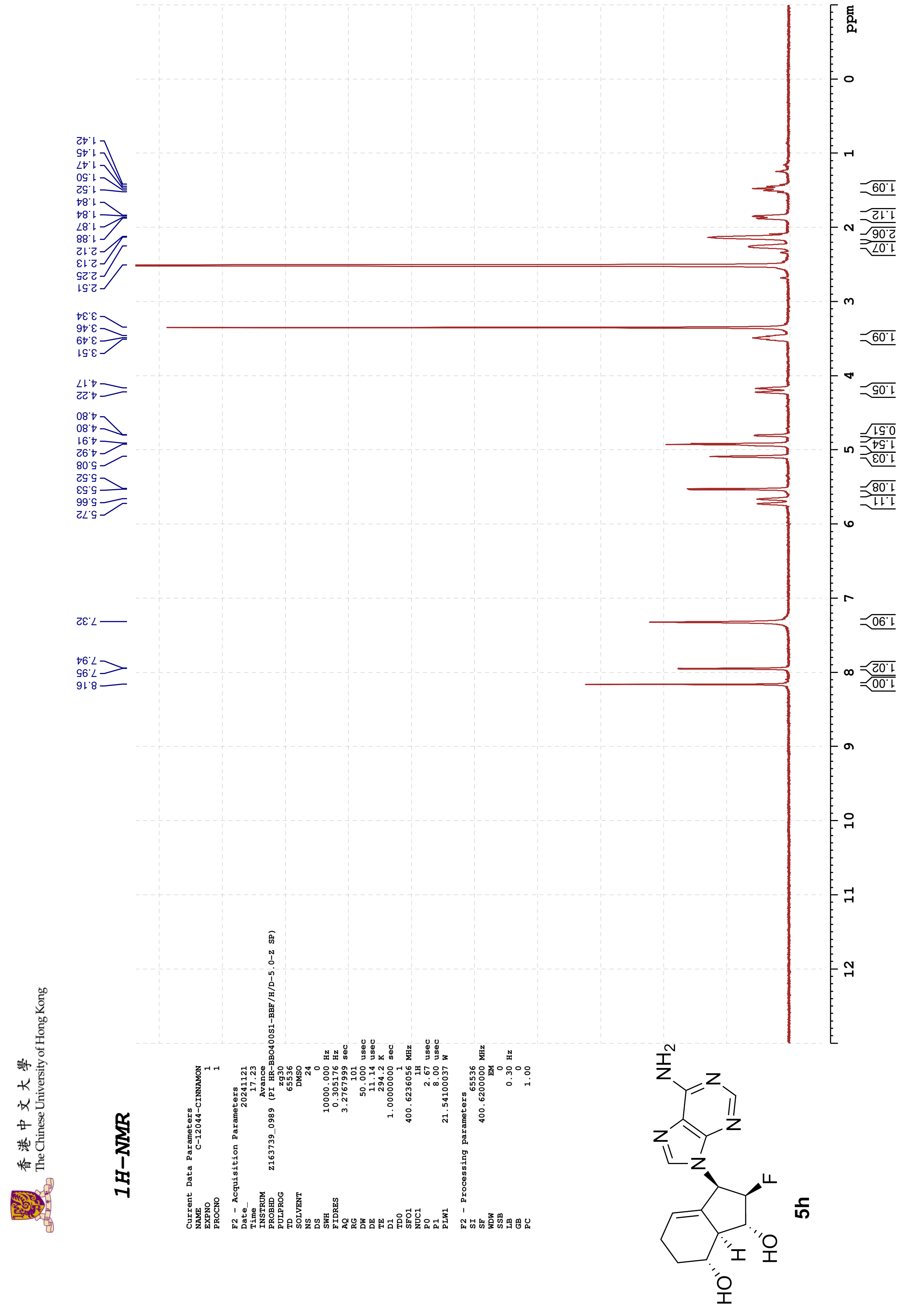

NMR-Spectra for Compound 6a

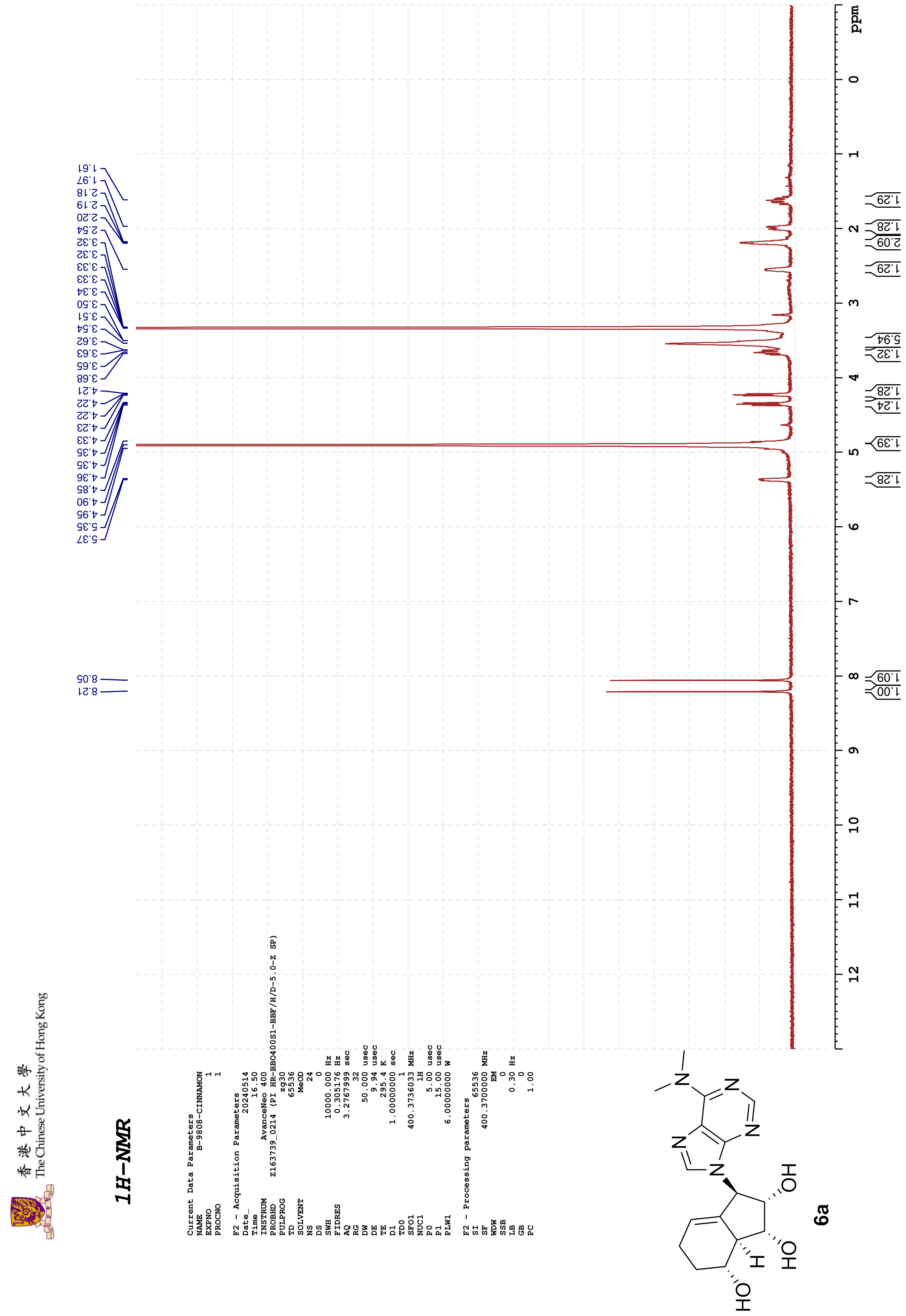

6a

NMR-Spectra for Compound 6a

香 港 中 文 大 學
The Chinese University of Hong Kong

13C{1H}-NMR

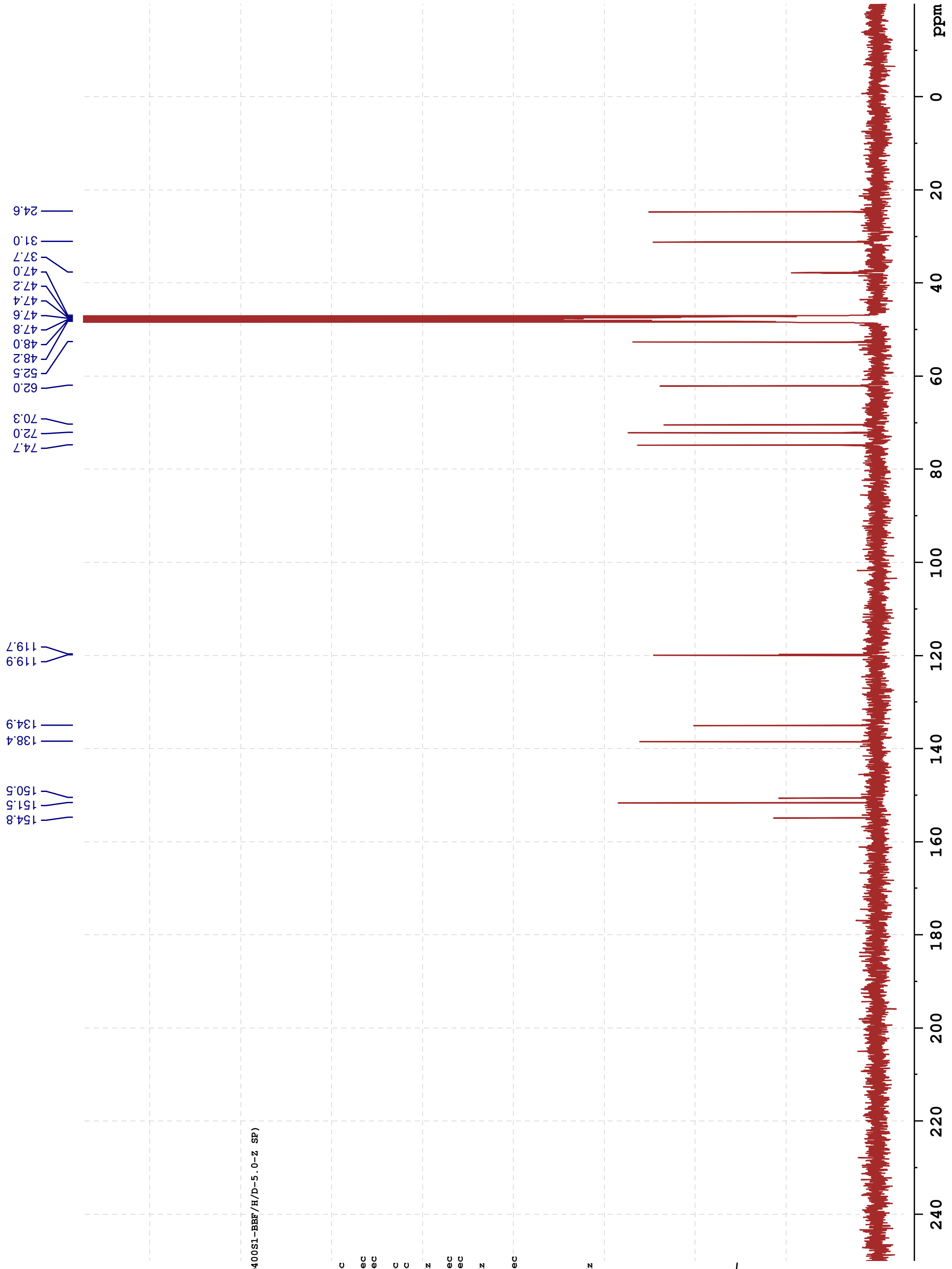


```
Current Data Parameters
NAME              7034-CINNAMON
EXPNO                         1
PROCNO                        1

F2 - Acquisition Parameters
Date_                  20240516
Time                      17.14
INSTRUM    AvanceNeo  Ascend400MHz
PROBHD     Z163739_0198 (PI HR-BBO400S1-BBF/H/D-5.0-Z SP)
PULPROG                 zgpg30
TD                       65536
SOLVENT                   MeOD
NS                        1024
DS                           4
SWH                  32679.738 Hz
FIDRES                0.997306 Hz
AQ                   1.0027008 sec
RG                         101
DW                      15.300 usec
DE                        6.50 usec
TE                       293.7 K
D1                  2.00000000 sec
D11                 0.03000000 sec
TD0                          1
SFO1               100.6686003 MHz
NUC1                       13C
P0                        2.67 usec
P1                        8.00 usec
PLW1               95.69300079 W
SFO2               400.3016012 MHz
NUC2                        1H
CPDPRG[2]              waltz65
PCPD2                    90.00 usec
PLW2               24.20299911 W
PLW12               0.19123000 W
PLW13               0.09618900 W

F2 - Processing parameters
SI                       32768
SF                 100.6555151 MHz
WDW                         EM
SSB                          0
LB                        2.00 Hz
GB                           0
PC                        1.40
```

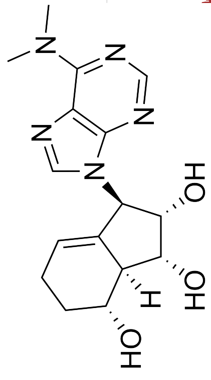

6a

NMR-Spectra for Compound 6b

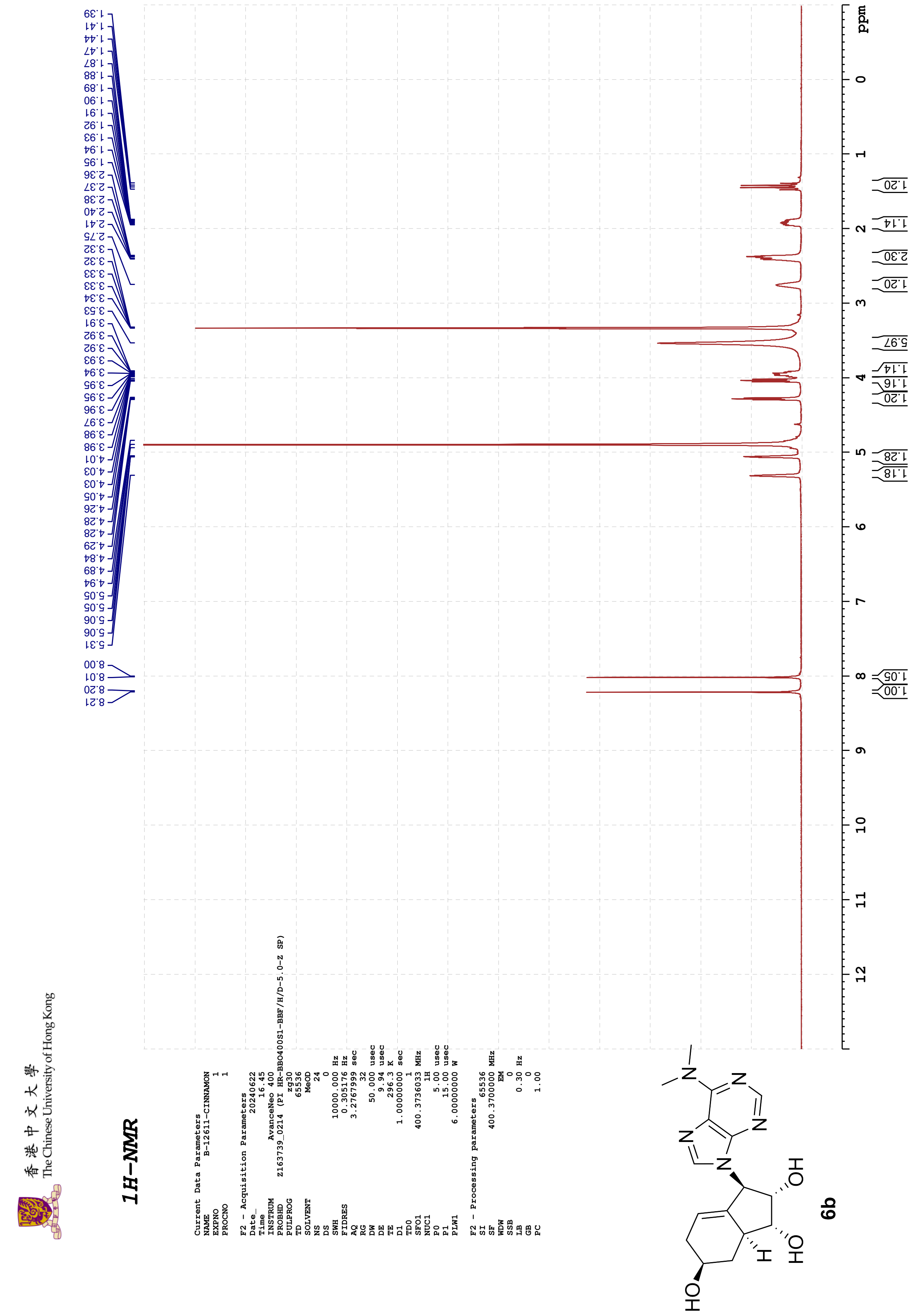

NMR-Spectra for Compound 6b

香港中文大學
The Chinese University of Hong Kong

13C{1H}-NMR

**Current Data Parameters**	
NAME	AR-2827-CINNAMON cmr
EXPNO	1
PROCNO	1
**F2 - Acquisition Parameters**	
Date_	20240622
Time	23.11
INSTRUM	Avance
PROBHD	Z166552_0024 (PI HR-BBO400S1-BBF/H/D-5.0-Z SP DI
PULPROG	zgpg30
TD	65536
SOLVENT	MeOD
NS	1024
DS	0
SWH	32679.739 Hz
FIDRES	0.997306 Hz
AQ	1.0027008 sec
RG	101
DW	15.300 usec
DE	6.50 usec
TE	297.8 K
D1	2.00000000 sec
D11	0.03000000 sec
TD0	1
SFO1	100.6258482 MHz
NUC1	13C
P0	2.67 usec
P1	8.00 usec
PLW1	104.50000000 W
SFO2	400.1316005 MHz
NUC2	1H
CPDPRG2	waltz65
PCPD2	90.00 usec
PLW2	22.37700081 W
PLW12	0.17681000 W
PLW13	0.08893200 W
**F2 - Processing parameters**	
SI	32768
SF	100.6127685 MHz
WDW	EM
SSB	0
LB	1.00 Hz
GB	0
PC	1.40

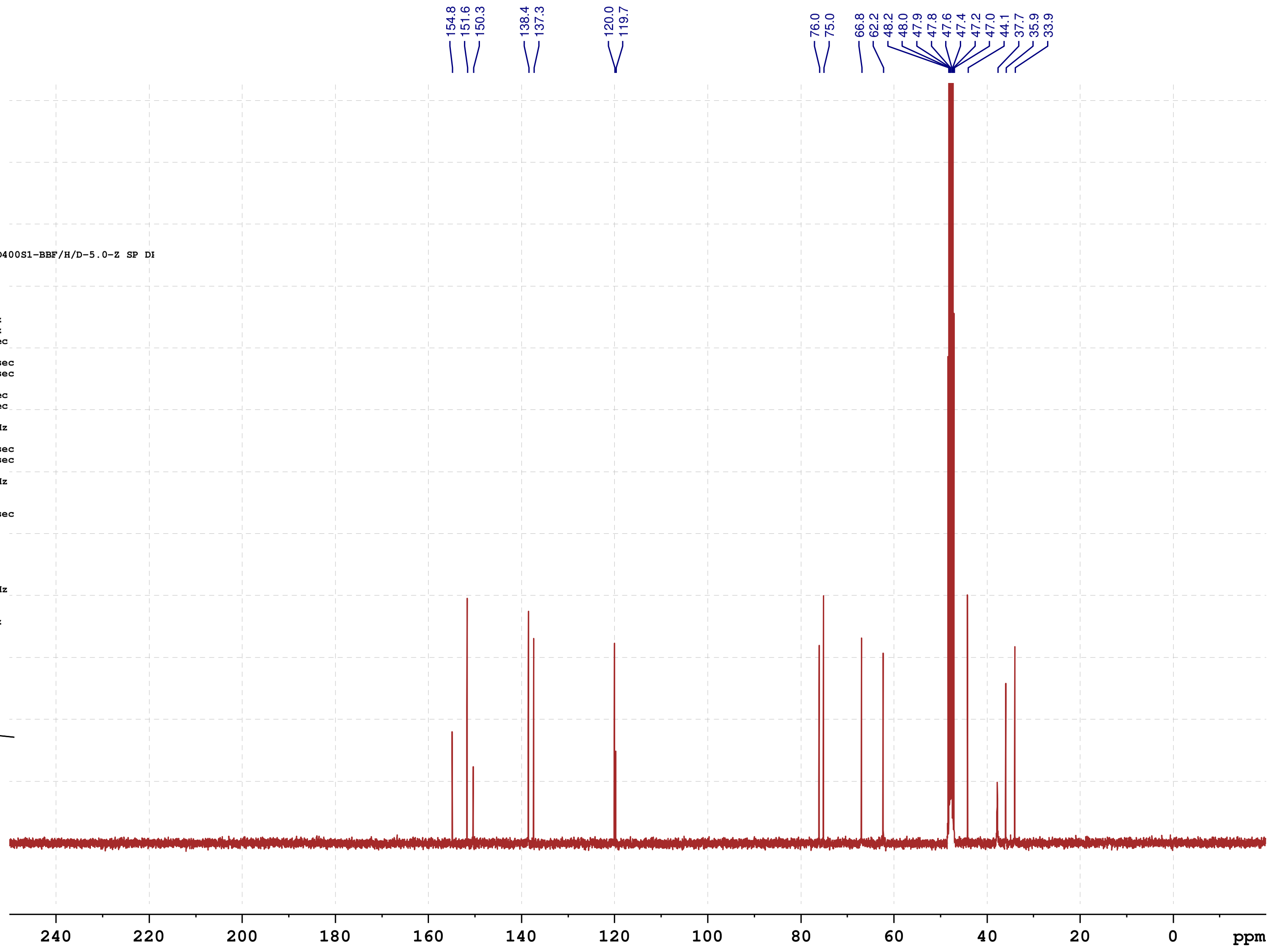

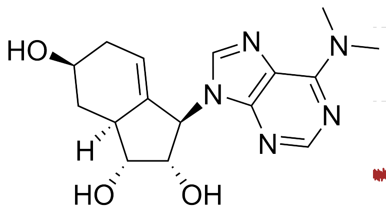

6b

NMR-Spectra for Compound 6c

1H-NMR

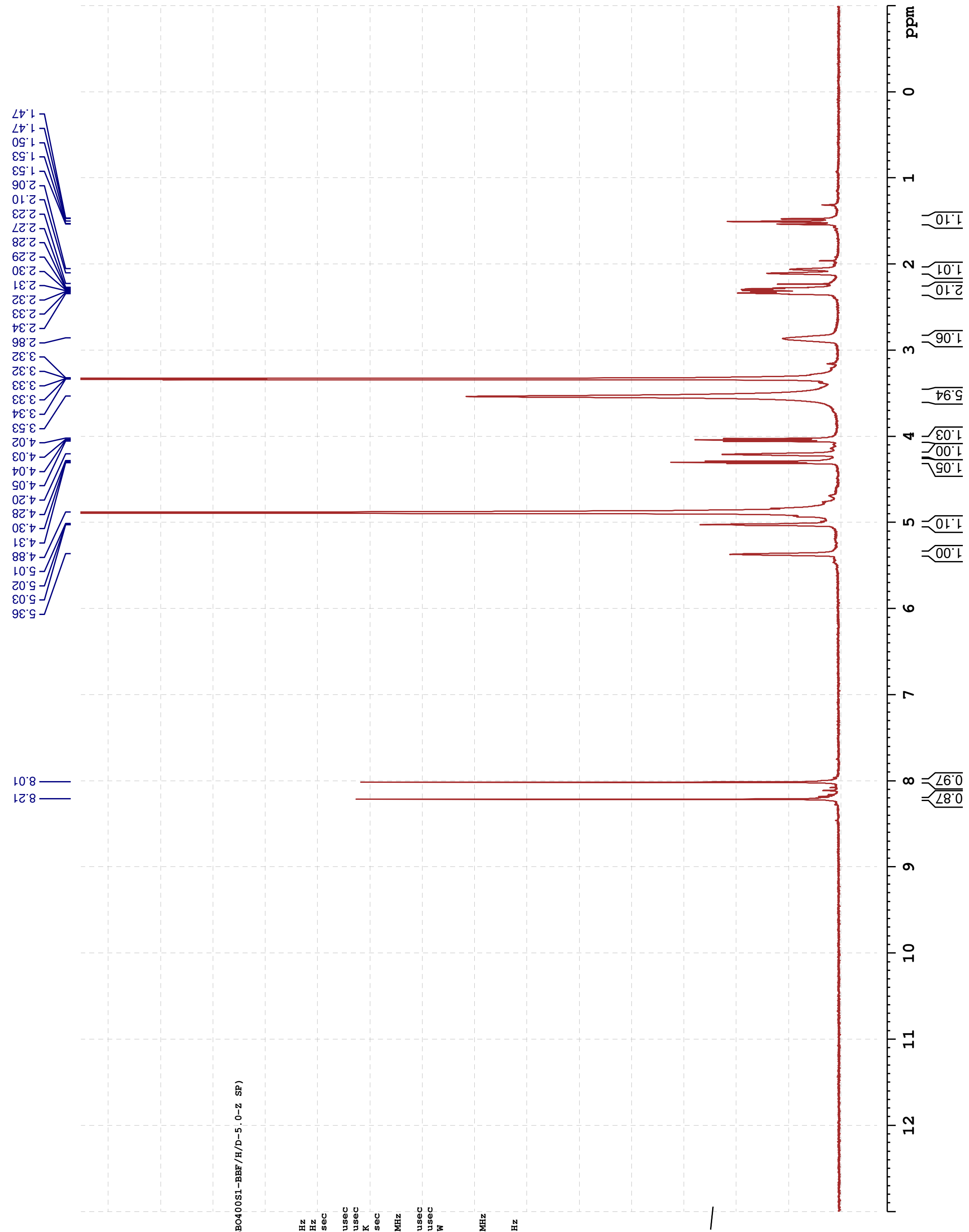

Current Data Parameters
NAME B-12189-CINNAMON
EXPNO 1
PROCNO 1

F2 - Acquisition Parameters
Date_ 20240618
Time 6.40
INSTRUM AvanceNeo 400
PROBHD Z163739_0214 (PI HR-BBO400S1-BBF/H/D-5.0-Z SP)
PULPROG zg30
TD 65536
SOLVENT MeOD
NS 24
DS 0
SWH 10000.000 Hz
FIDRES 0.305176 Hz
AQ 3.2767999 sec
RG 32
DW 50.000 usec
DE 9.94 usec
TE 297.5 K
D1 1.00000000 sec
TD0 1
SFO1 400.3736033 MHz
NUC1 1H
P0 5.00 usec
P1 15.00 usec
PLW1 6.00000000 W

F2 - Processing parameters
SI 65536
SF 400.3700000 MHz
WDW EM
SSB 0
LB 0.30 Hz
GB 0
PC 1.00

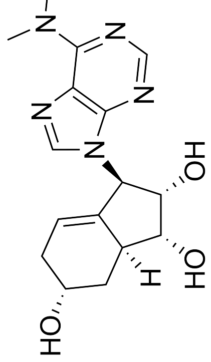

6c

NMR-Spectra for Compound 6c

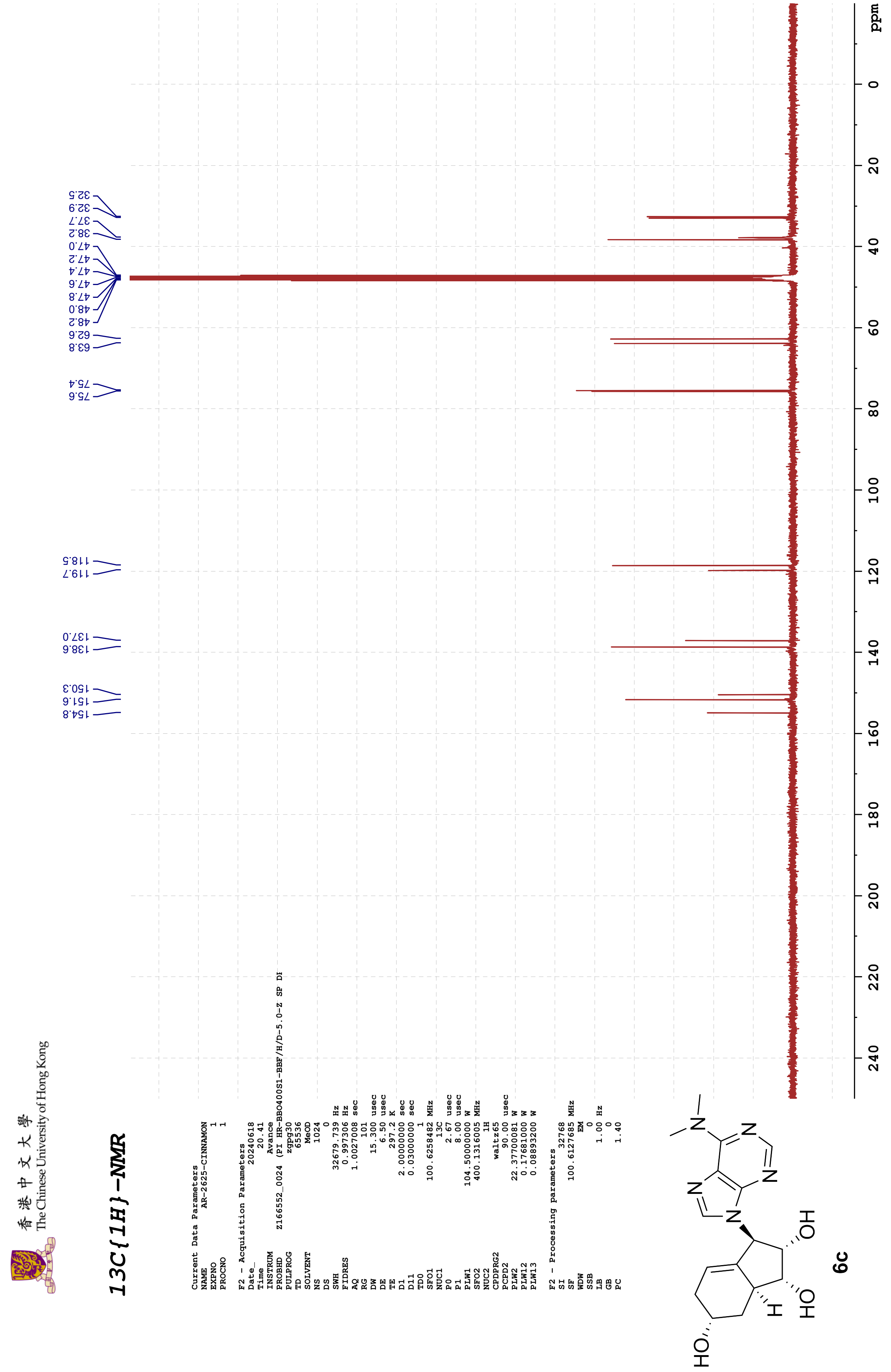

NMR-Spectra for Compound 6d

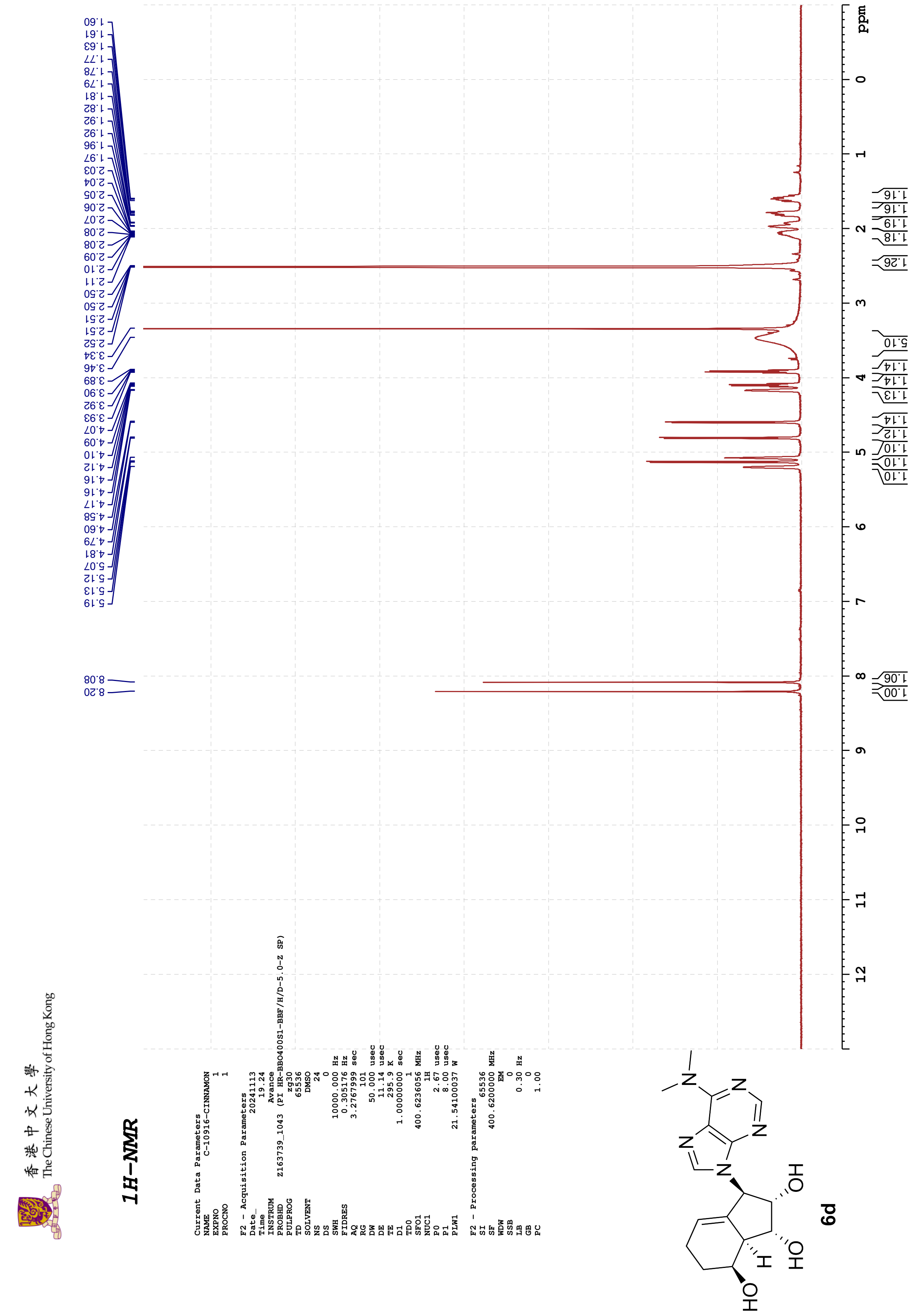

NMR-Spectra for Compound 6d

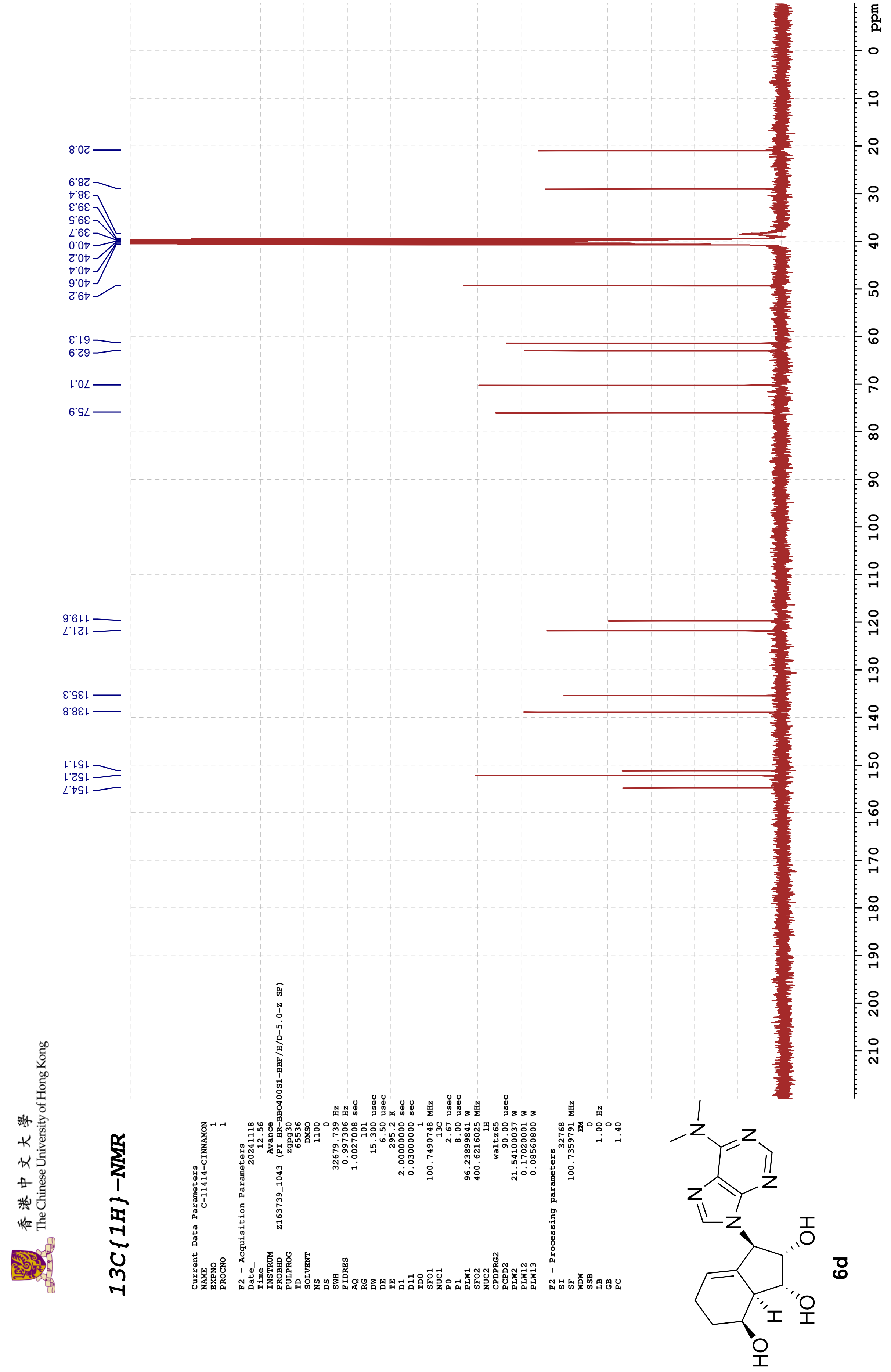

NMR-Spectra for Compound 6e

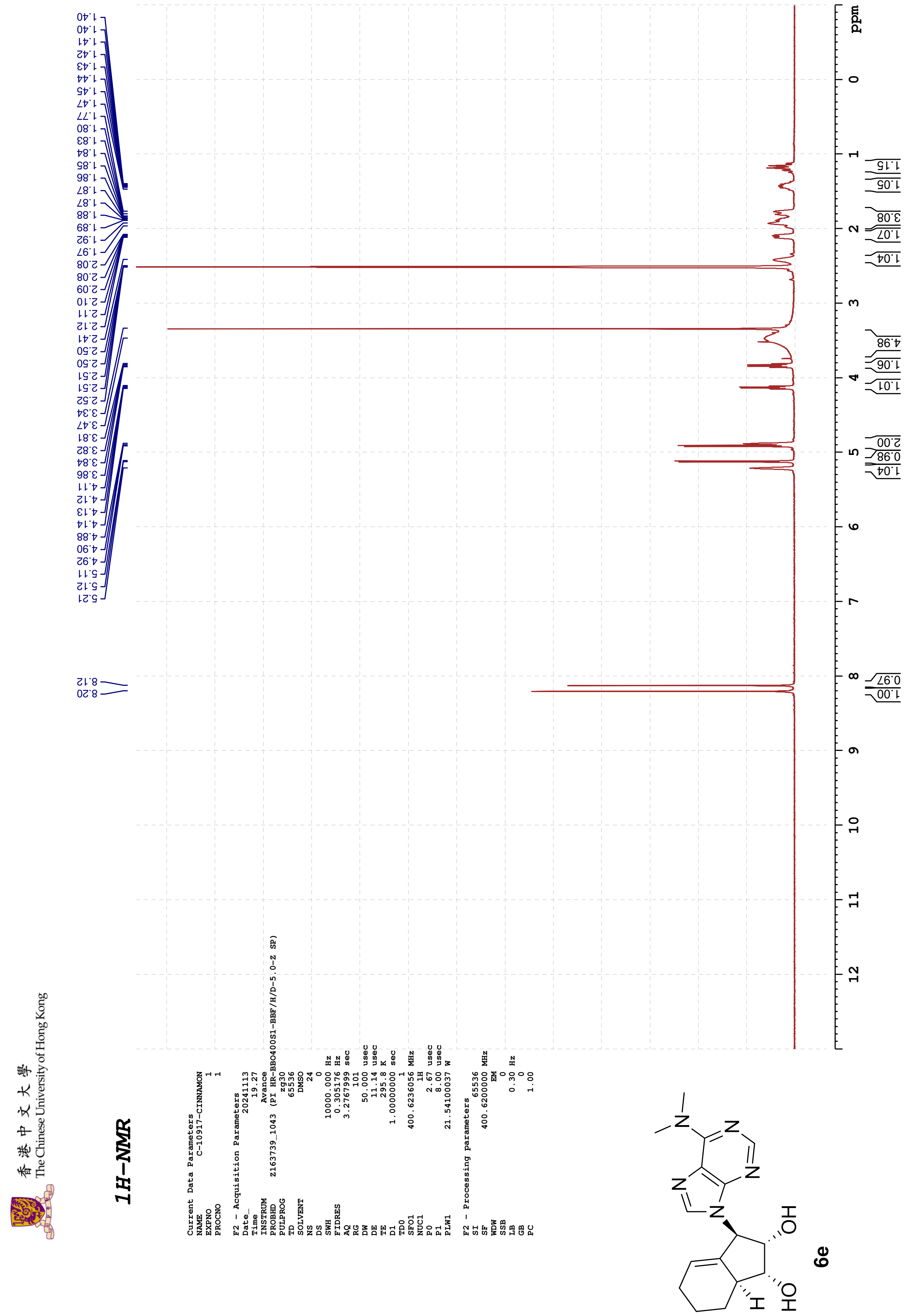

6e

NMR-Spectra for Compound 6e

香港中文大學 The Chinese University of Hong Kong

13C{1H}-NMR

```
Current Data Parameters
NAME          C-11415-CINNAMON
EXPNO                        1
PROCNO                       1

F2 - Acquisition Parameters
Date_                 20241119
Time                      8.40
INSTRUM                 Avance
PROBHD      Z163739_1043 (PI HR-BBO400S1-BBF/H/D-5.0-Z SP)
PULPROG                zgpg30
TD                       65536
SOLVENT                   DMSO
NS                        1024
DS                           0
SWH                  32679.739 Hz
FIDRES                0.997306 Hz
AQ                   1.0027008 sec
RG                        12.7
DW                      15.300 usec
DE                        6.50 usec
TE                       295.1 K
D1                  2.00000000 sec
D11                 0.03000000 sec
TD0                          1
SFO1               100.7490748 MHz
NUC1                       13C
P0                        2.67 usec
P1                        8.00 usec
PLW1               96.23899841 W
SFO2               400.6216025 MHz
NUC2                        1H
CPDPRG2                waltz65
PCPD2                    90.00 usec
PLW2               21.54100037 W
PLW12               0.17020001 W
PLW13               0.08560800 W

F2 - Processing parameters
SI                       32768
SF                 100.7359791 MHz
WDW                         EM
SSB                          0
LB                        3.00 Hz
GB                           0
PC                        1.40
```

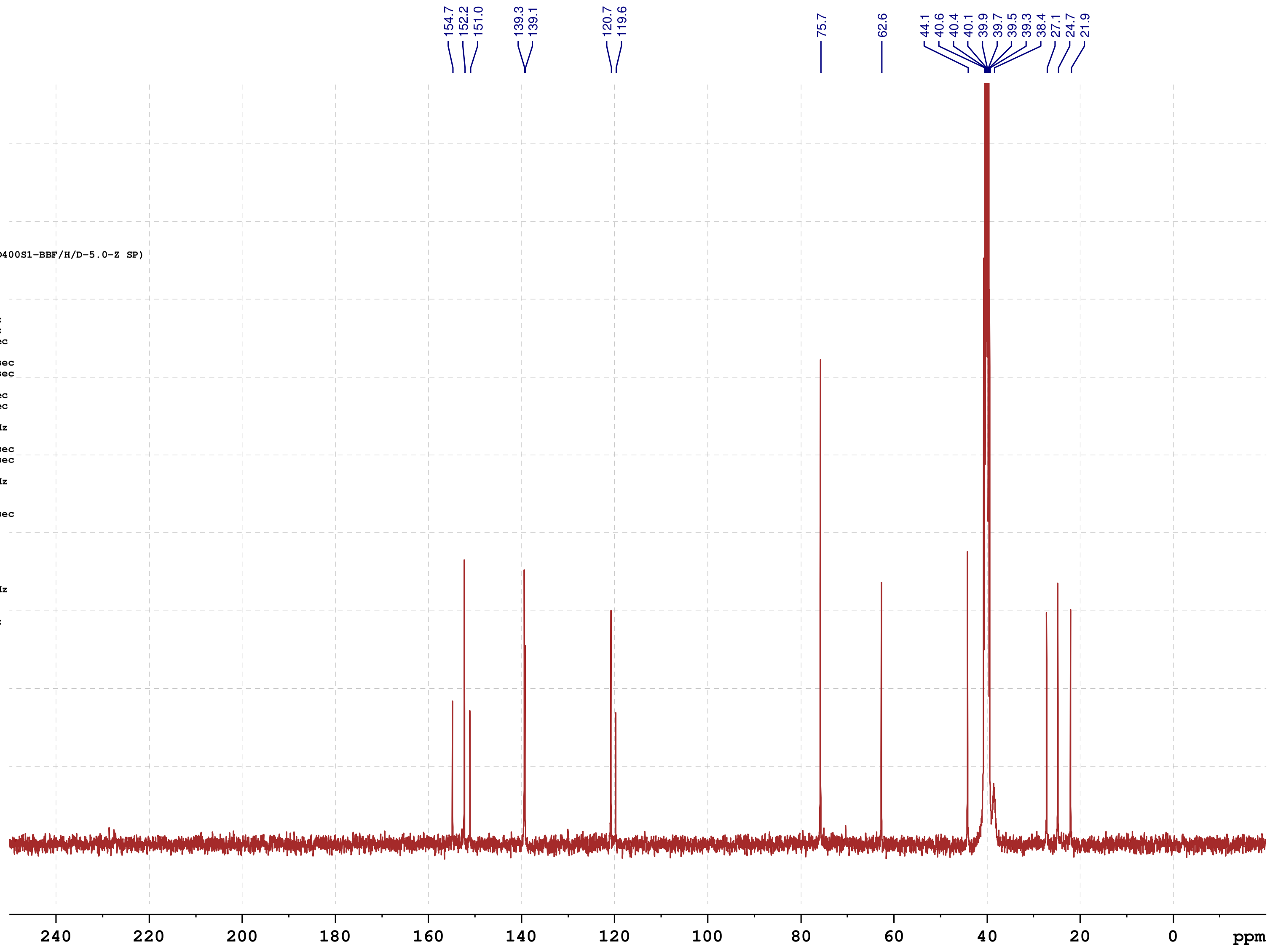

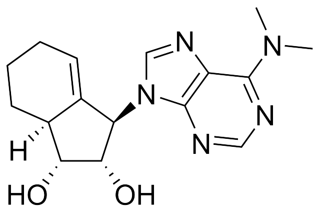

6e

NMR-Spectra for Compound 6f

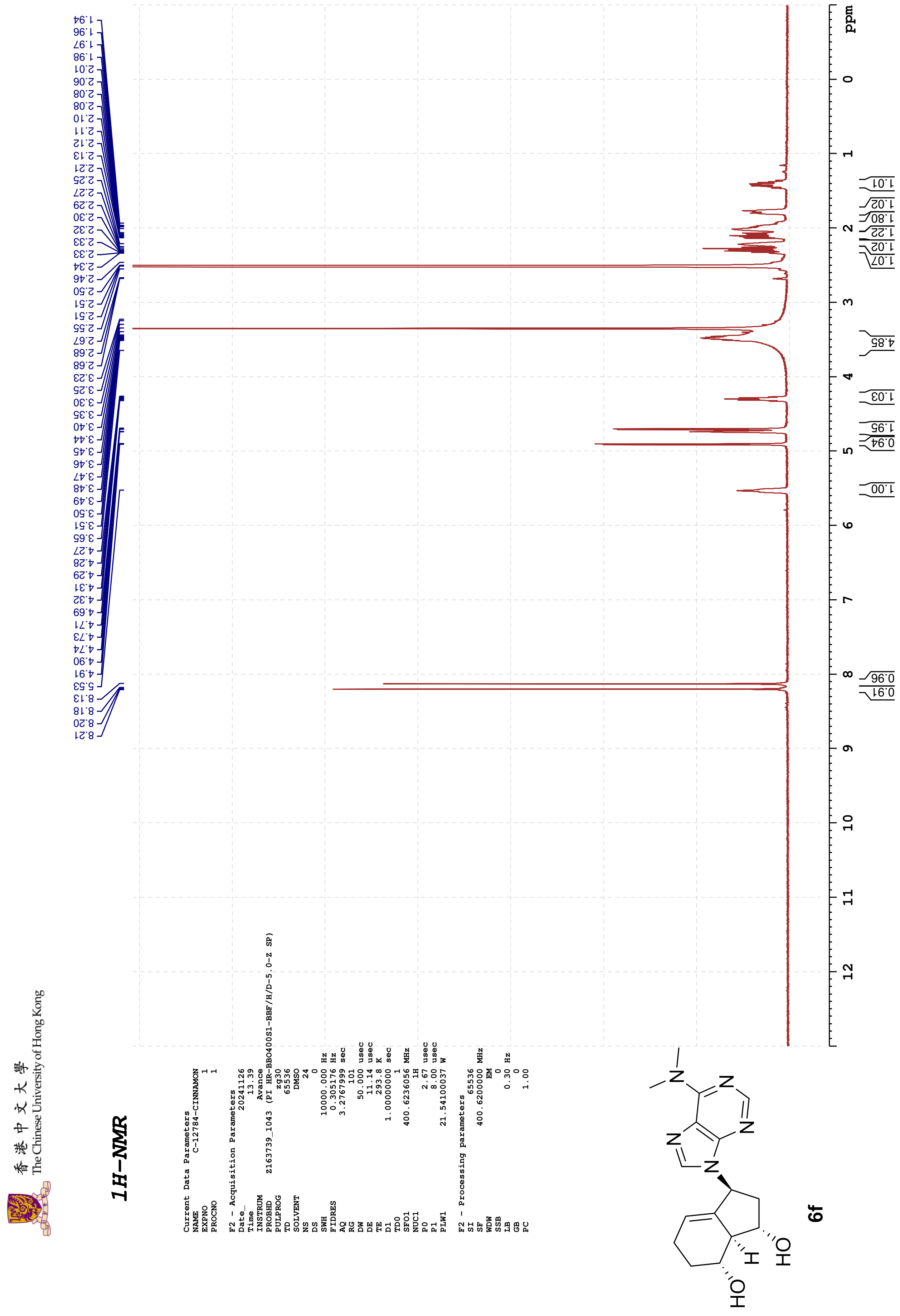

6f

NMR-Spectra for Compound 6f

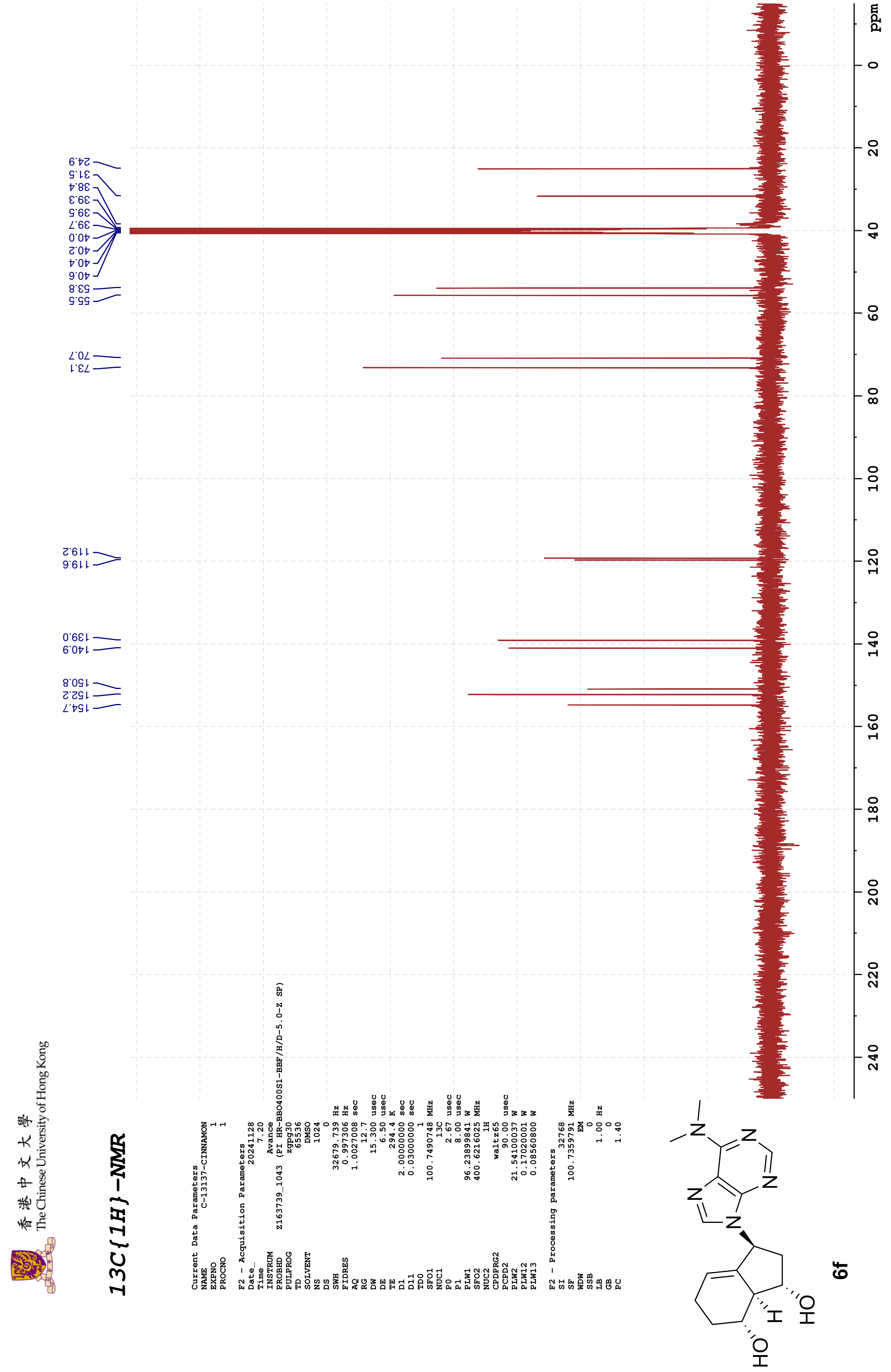

NMR-Spectra for Compound 6g

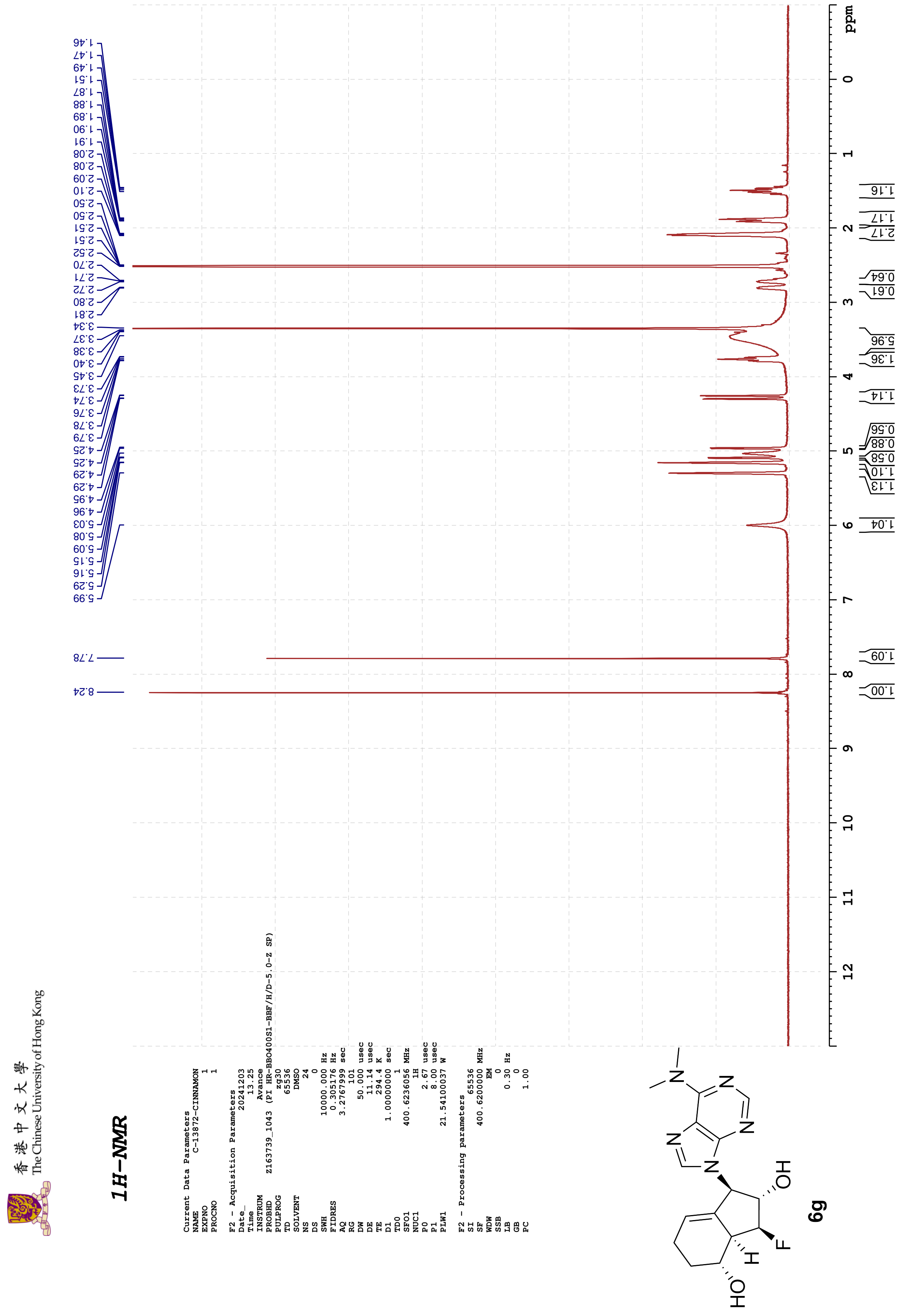

NMR-Spectra for Compound 6g

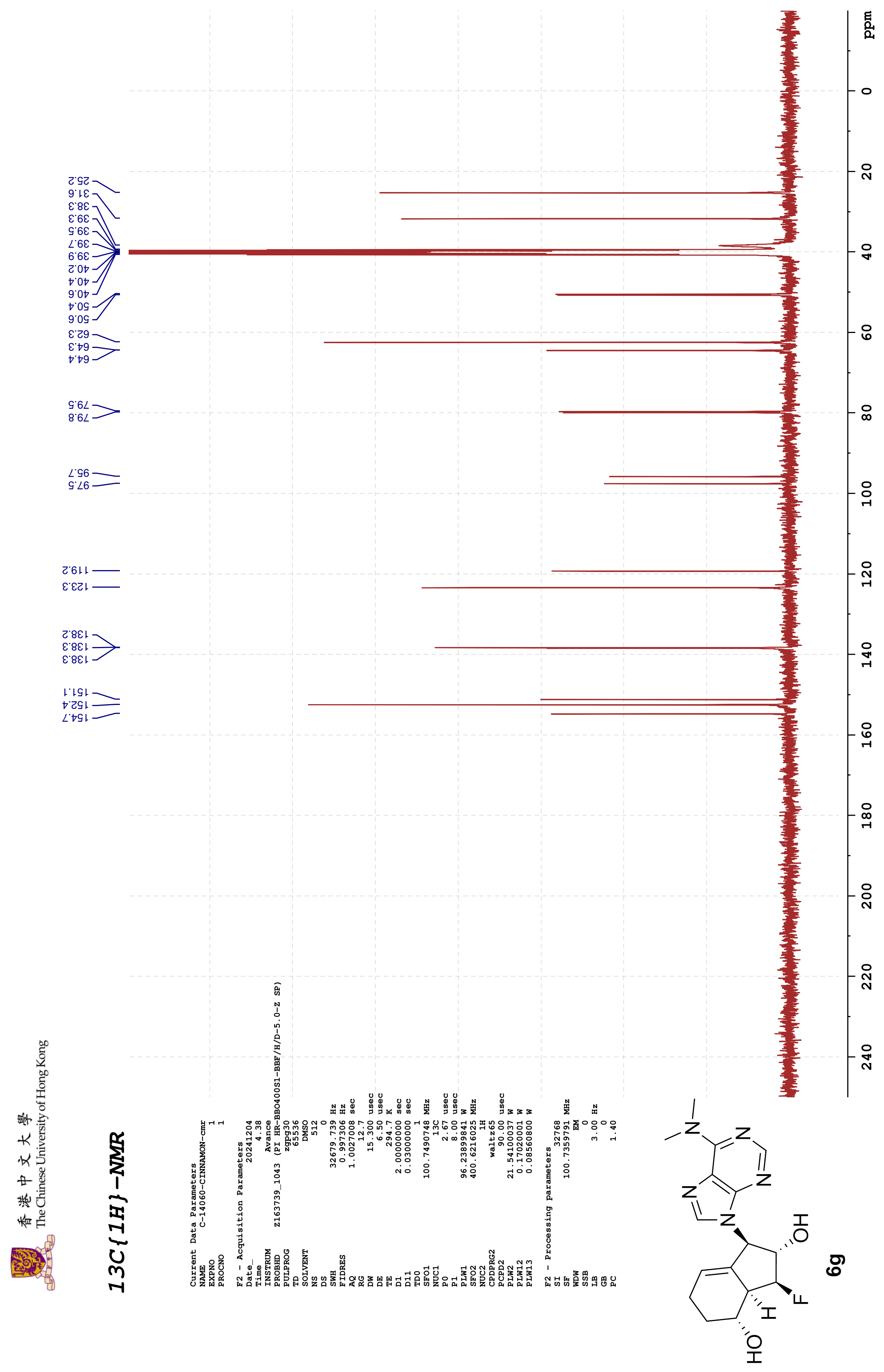

NMR-Spectra for Compound 6j

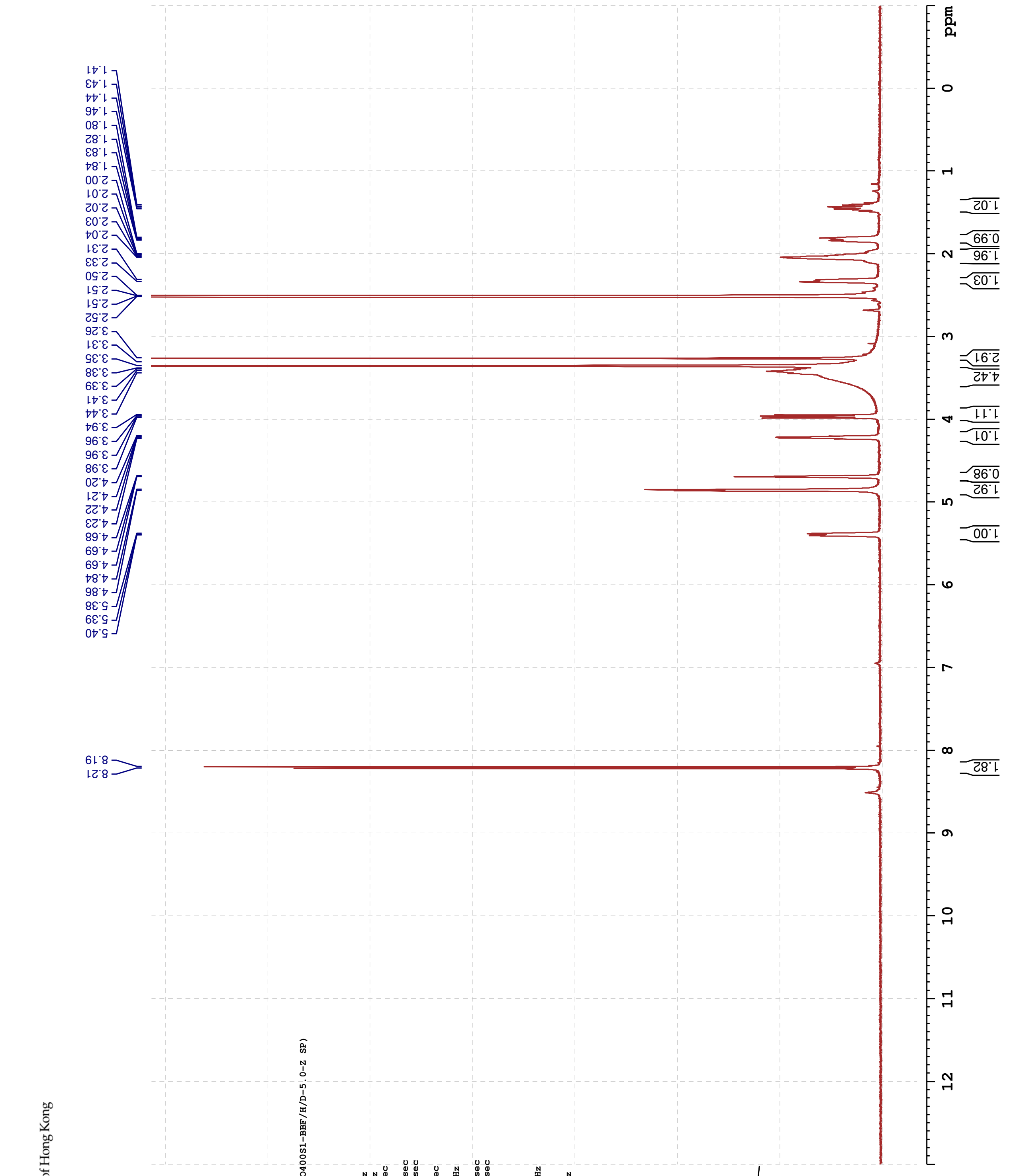

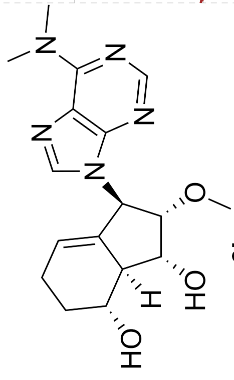

6j

NMR-Spectra for Compound 6j

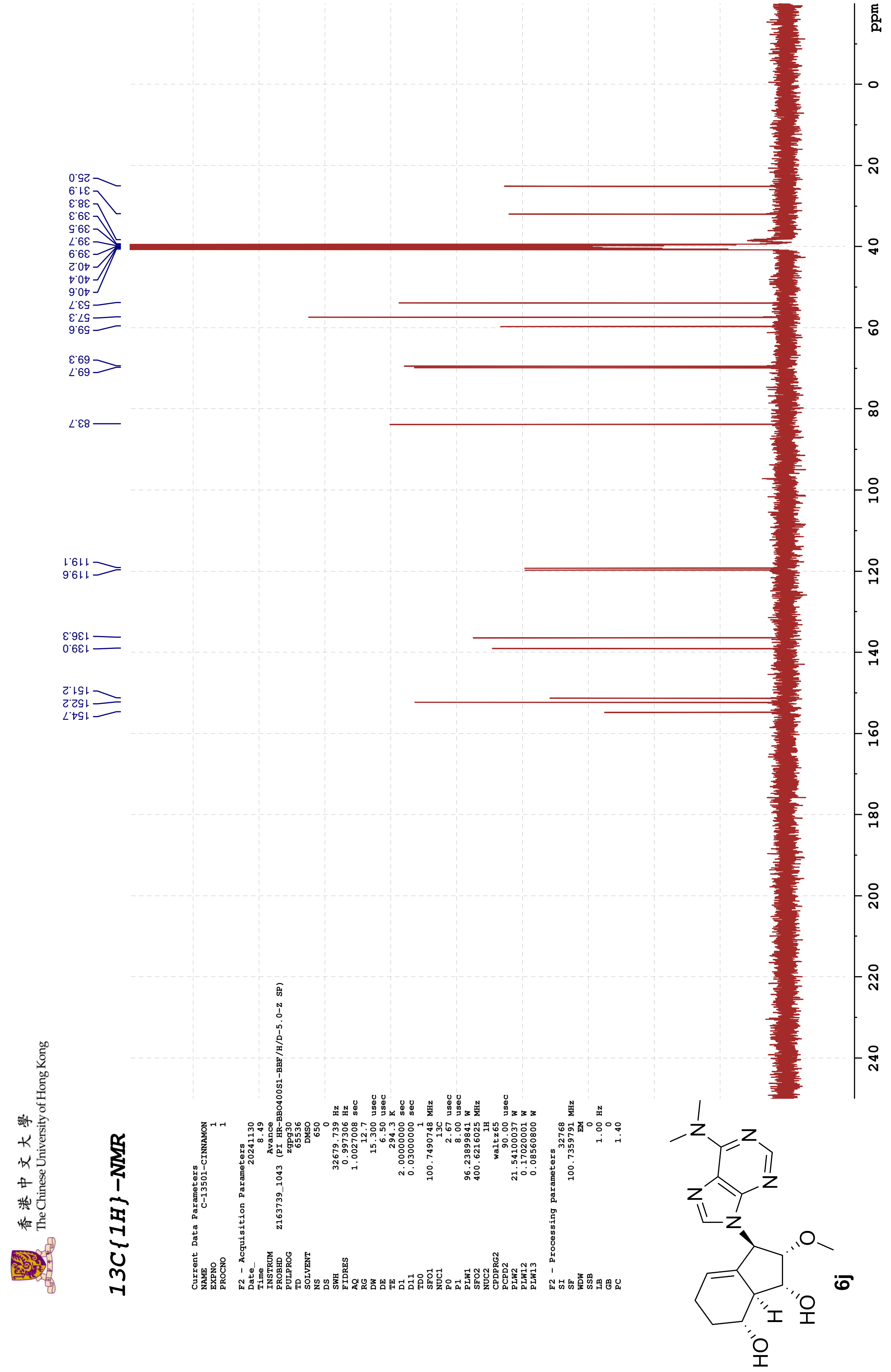

NMR-Spectra for Compound 6k

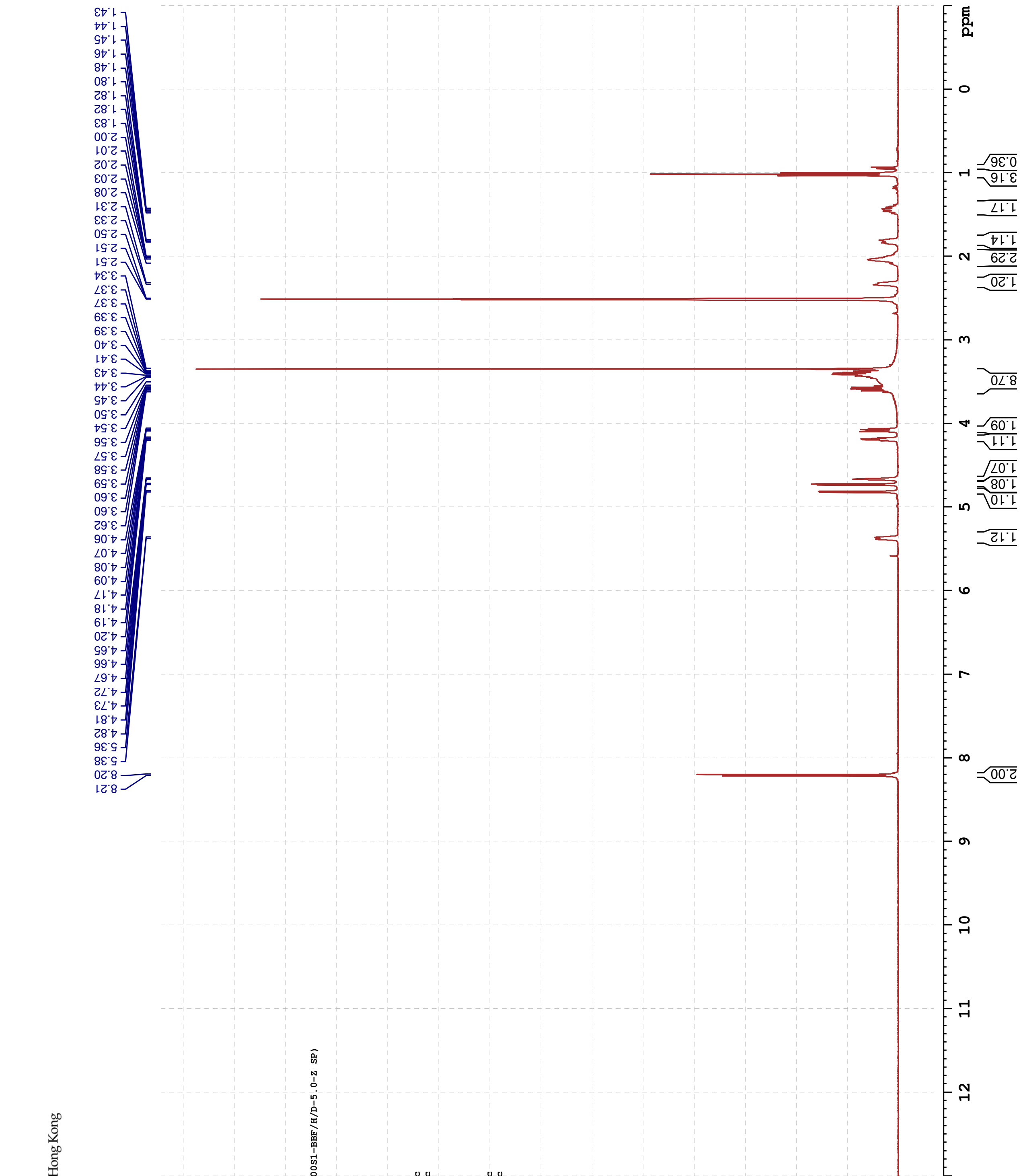

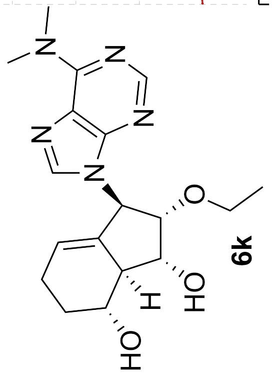

6k

NMR-Spectra for Compound 6k

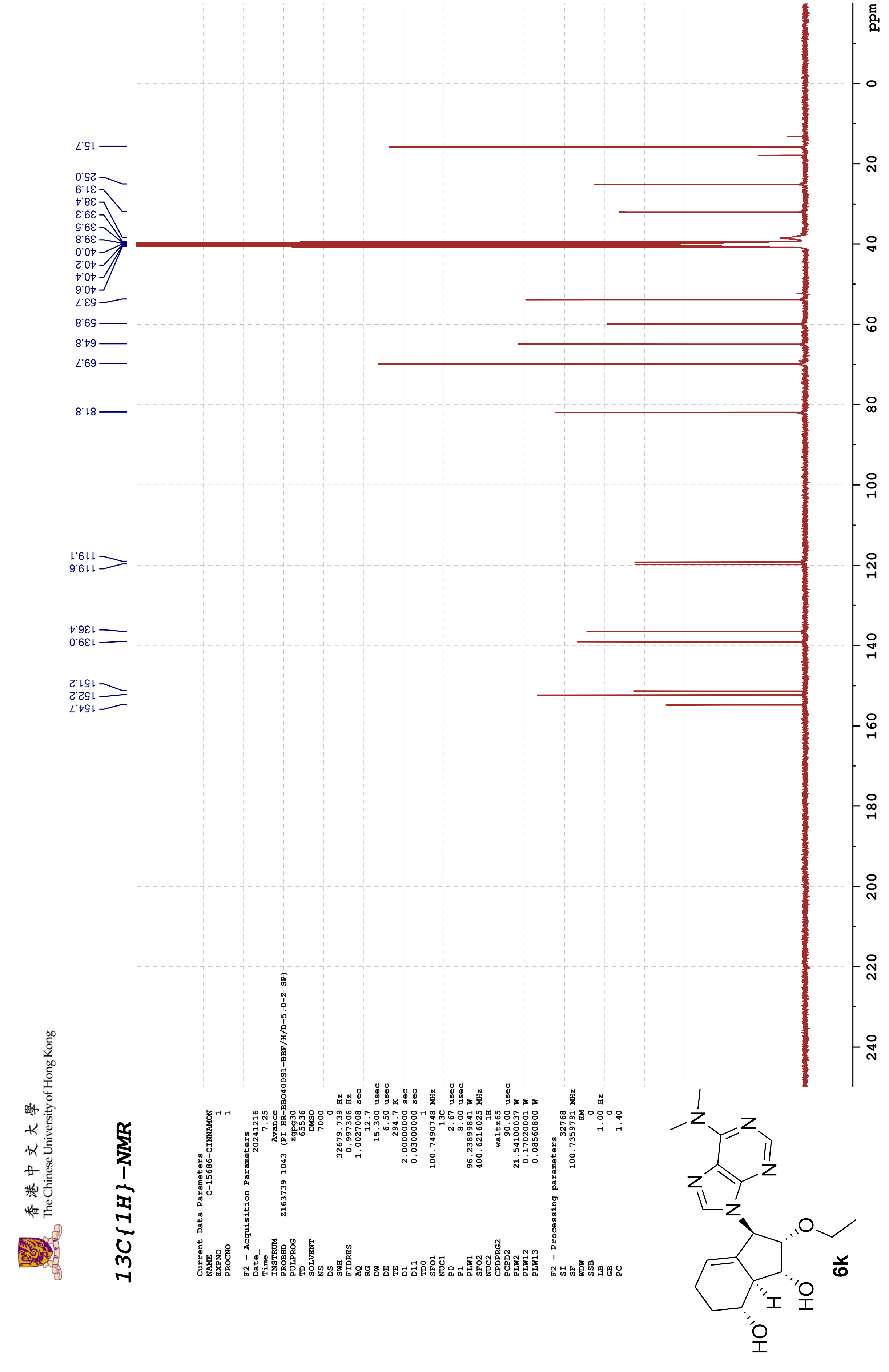

NMR-Spectra for Compound 7a

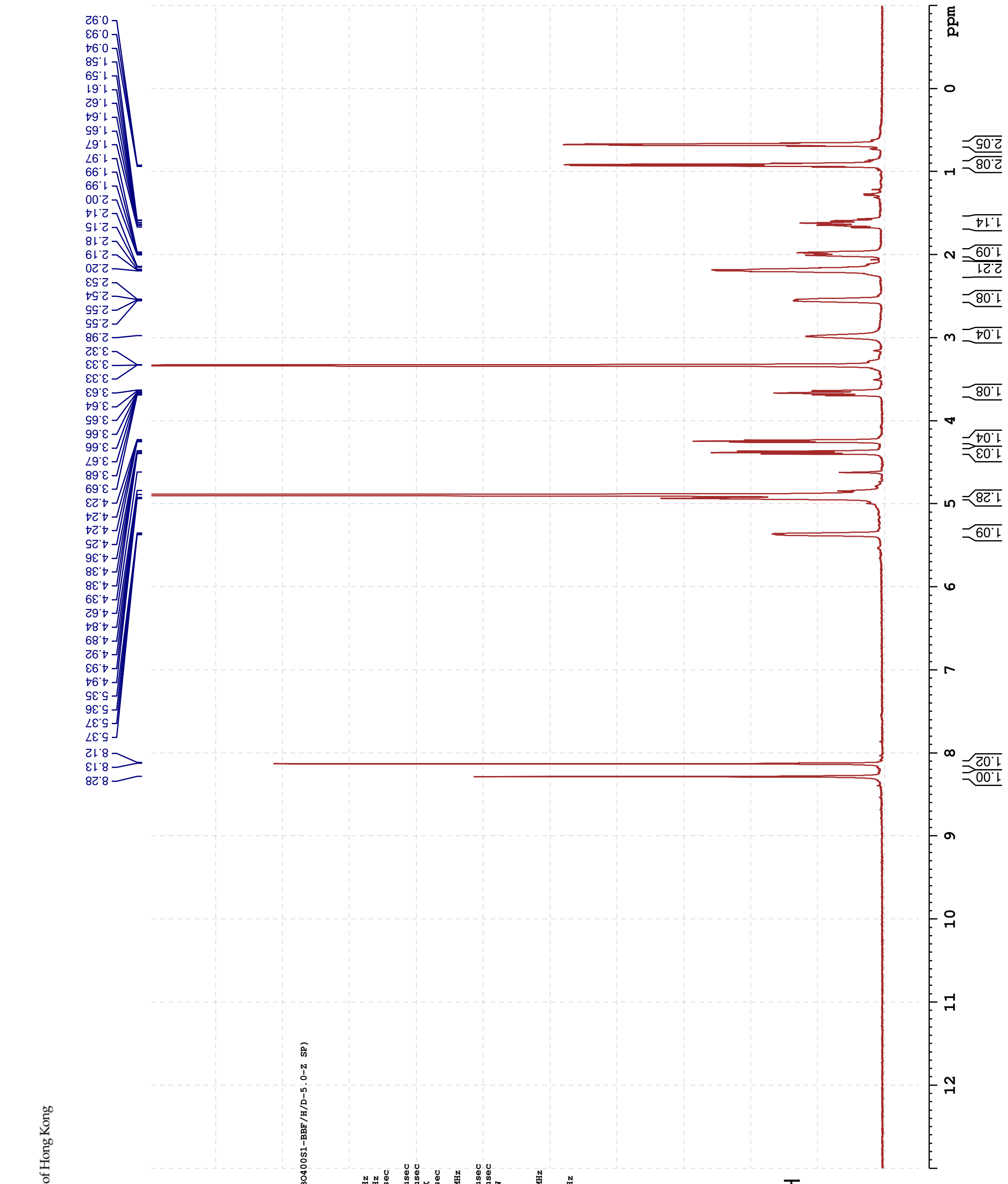

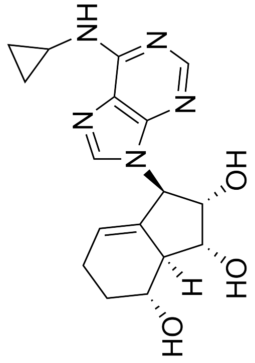

7a

NMR-Spectra for Compound 7a

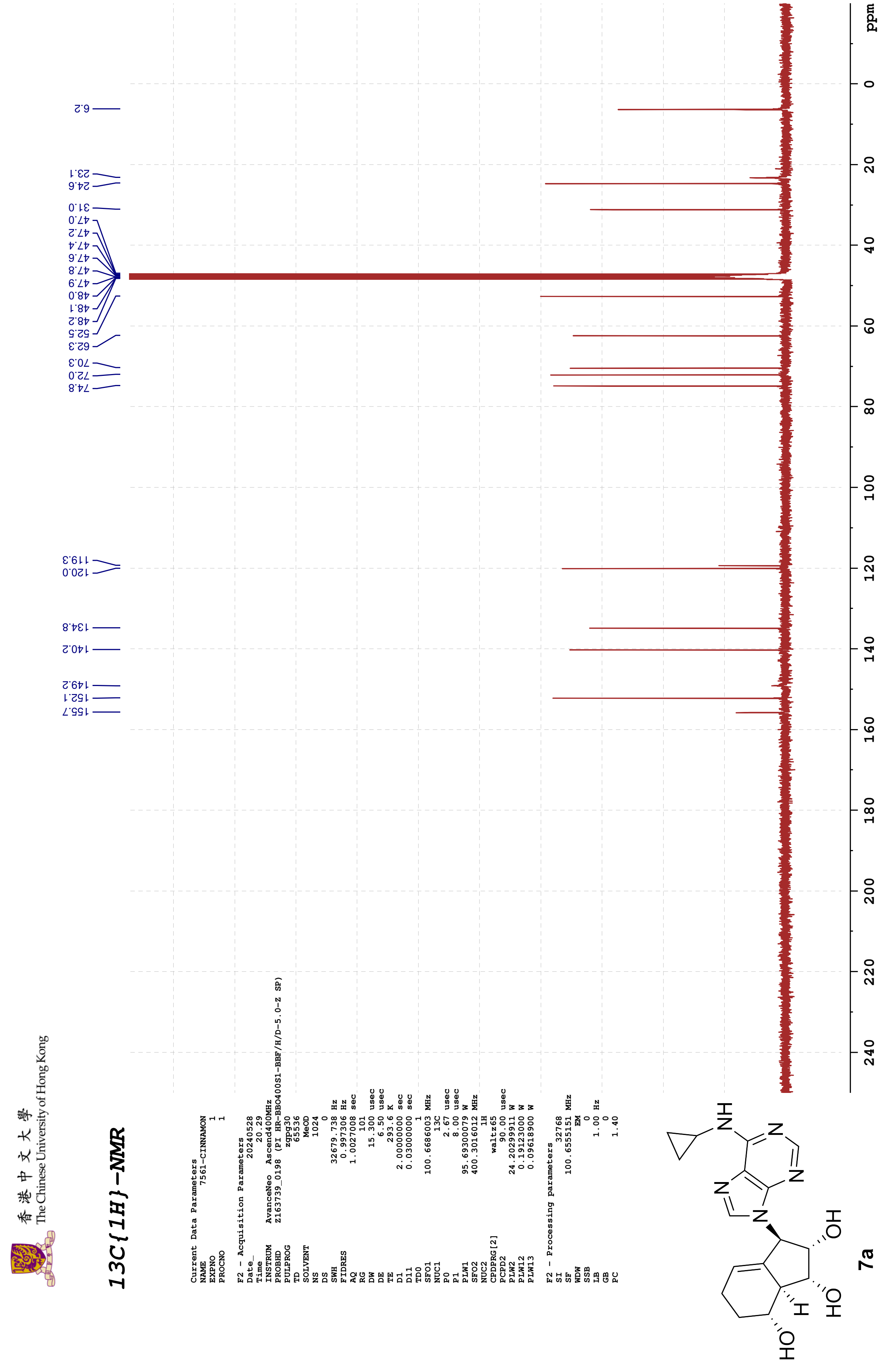

NMR-Spectra for Compound 7b

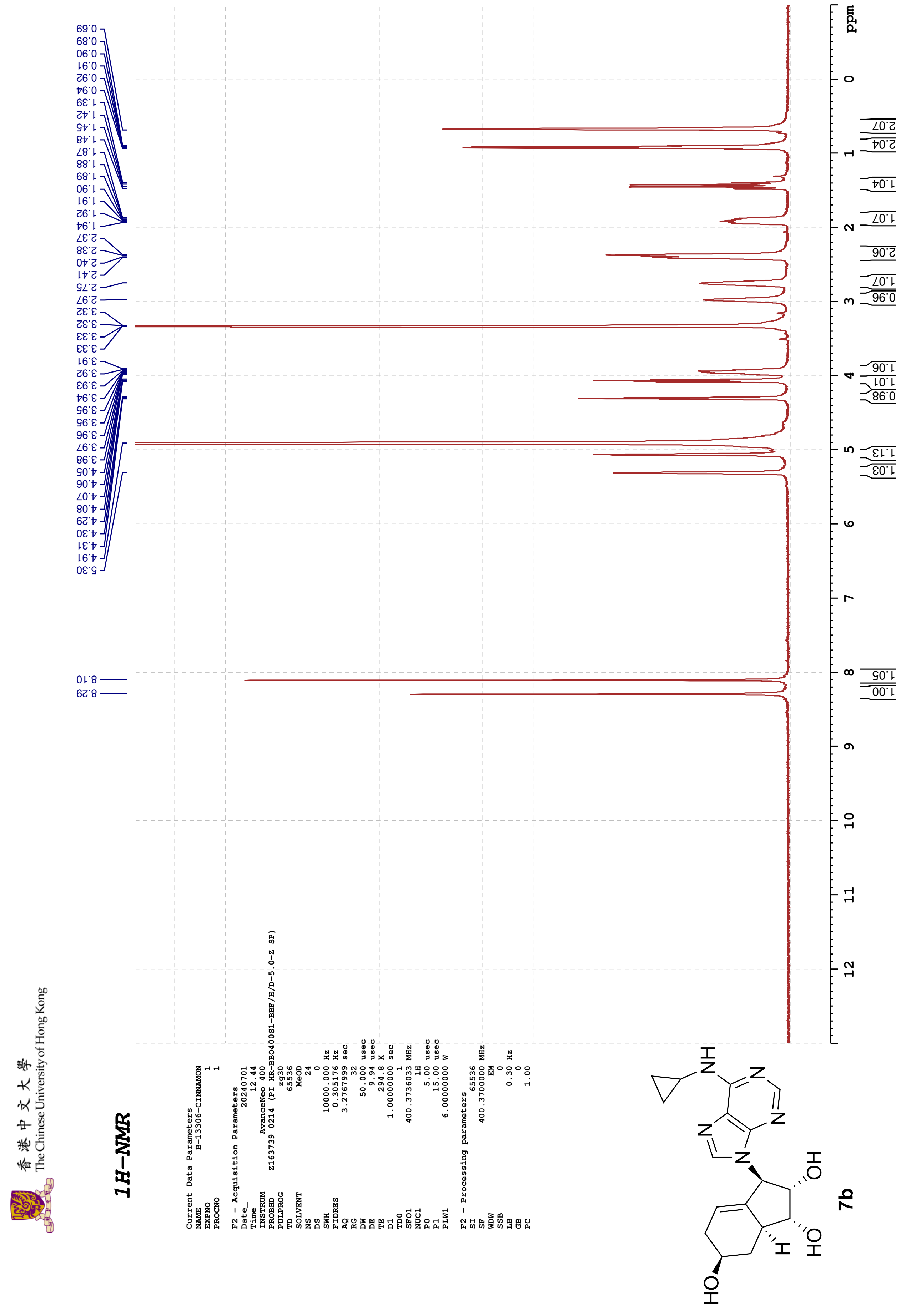

NMR-Spectra for Compound 7b

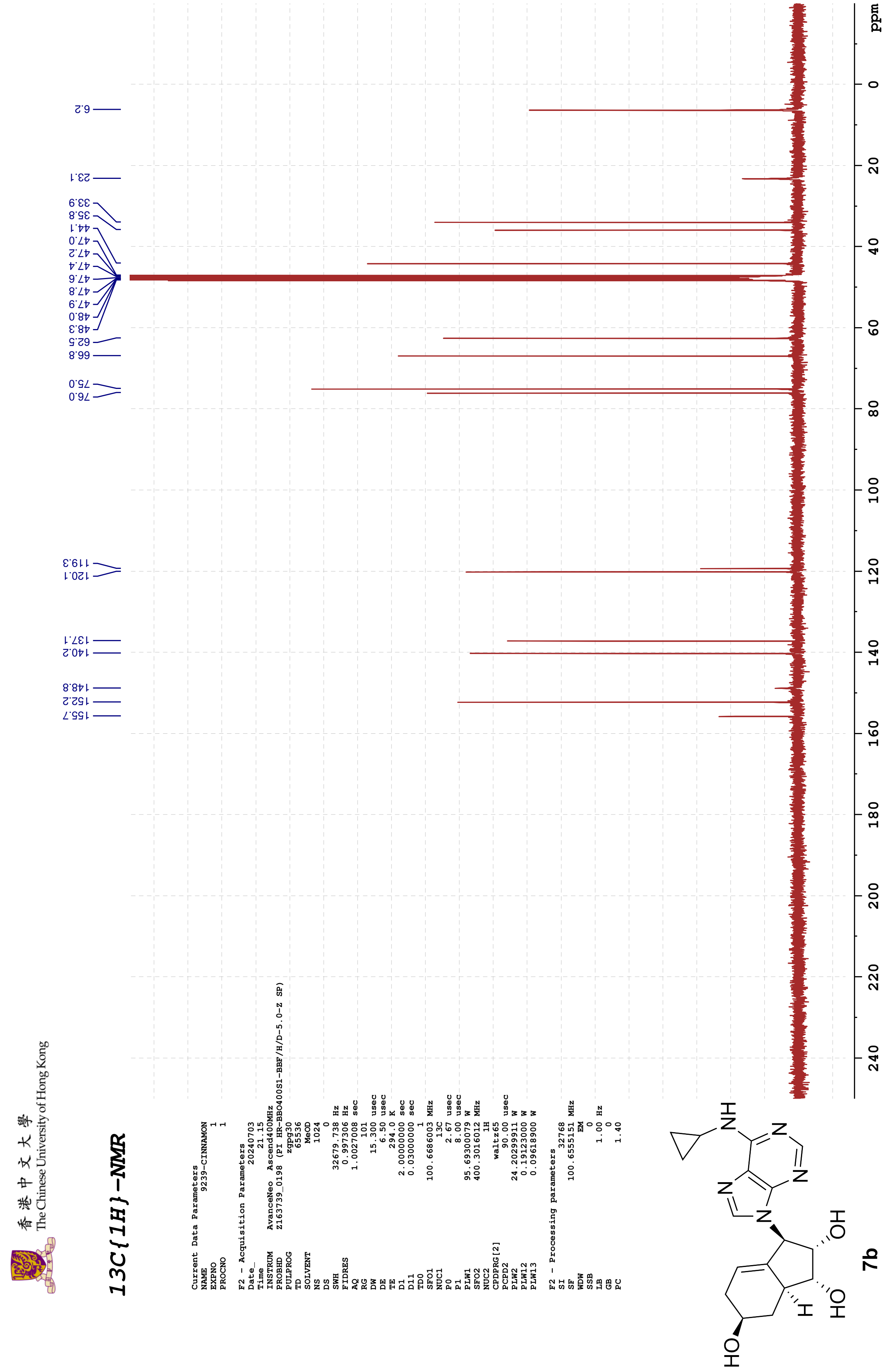

NMR-Spectra for Compound 7c

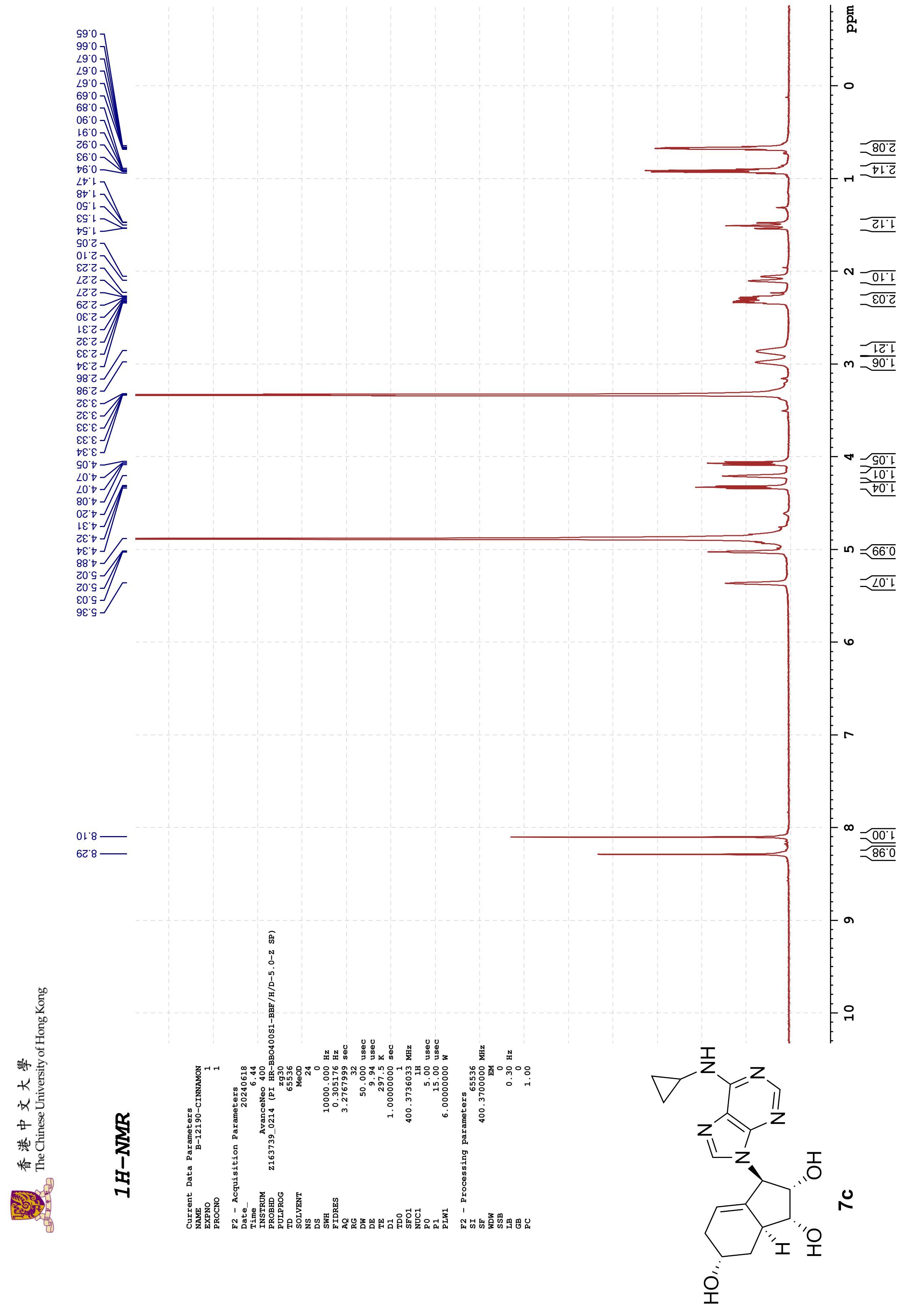

7c

NMR-Spectra for Compound 7c

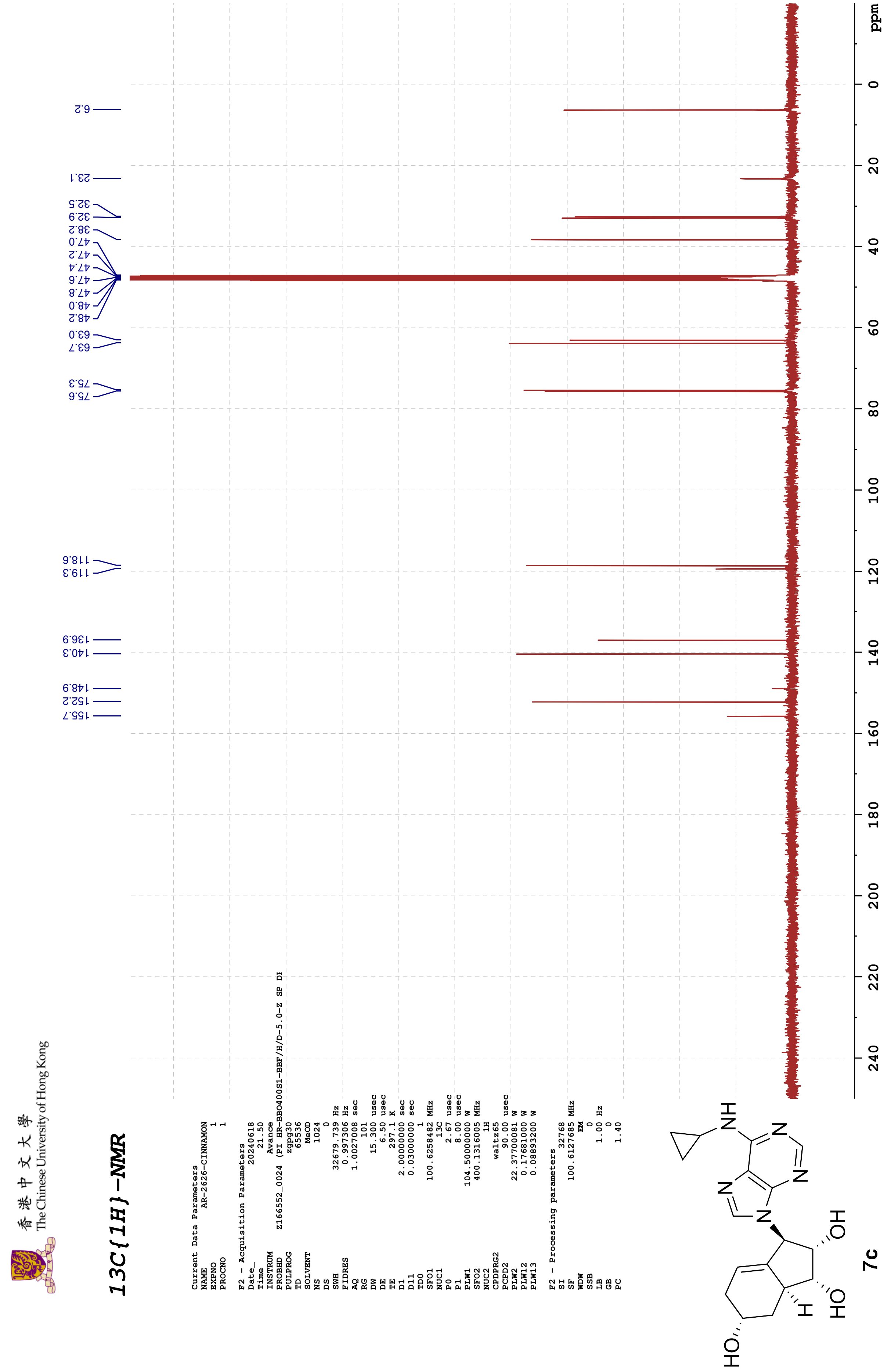

7c

NMR-Spectra for Compound 8a

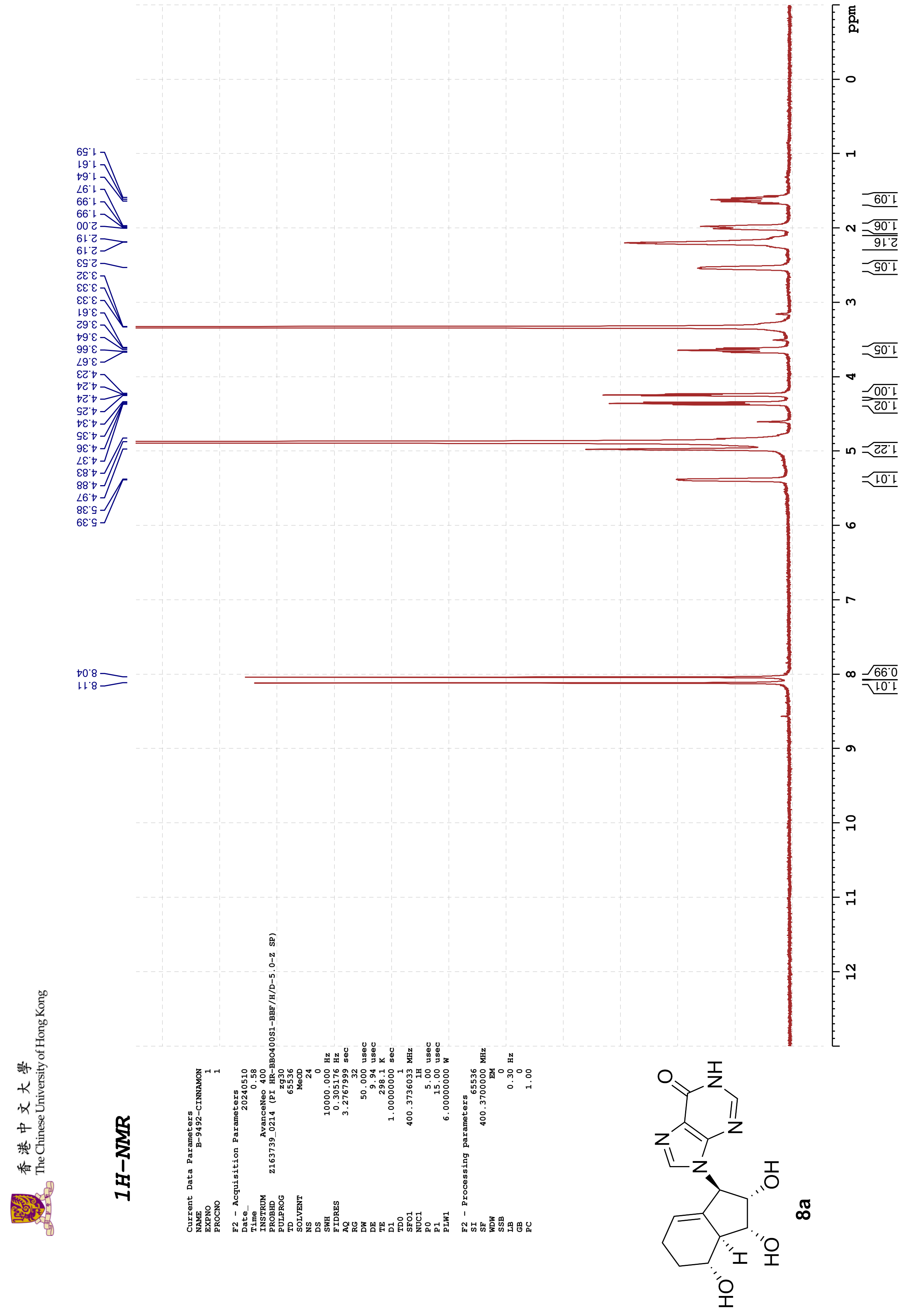

NMR-Spectra for Compound 8a

香港中文大學 The Chinese University of Hong Kong

13C{1H}-NMR

```
Current Data Parameters
NAME              B-9569-CINNAMON
EXPNO                           1
PROCNO                          1

F2 - Acquisition Parameters
Date_                    20240511
Time                        11.17
INSTRUM            AvanceNeo 400
PROBHD      Z163739_0214 (PI HR-BBO400S1-BBF/H/D-5.0-Z SP)
PULPROG                   zgpg30
TD                         65536
SOLVENT                     MeOD
NS                          1024
DS                             0
SWH                    32679.738 Hz
FIDRES                  0.997306 Hz
AQ                     1.0027008 sec
RG                           101
DW                        15.300 usec
DE                          6.50 usec
TE                         299.1 K
D1                    2.00000000 sec
D11                   0.03000000 sec
TD0                            1
SFO1                 100.6862041 MHz
NUC1                         13C
P0                          2.63 usec
P1                          7.90 usec
PLW1                100.30000305 W
SFO2                 400.3716015 MHz
NUC2                          1H
CPDPRG[2]                waltz65
PCPD2                      90.00 usec
PLW2                 24.89999962 W
PLW12                 0.18703000 W
PLW13                 0.09407300 W

F2 - Processing parameters
SI                         32768
SF                   100.6731166 MHz
WDW                           EM
SSB                            0
LB                          2.00 Hz
GB                             0
PC                          1.40
```

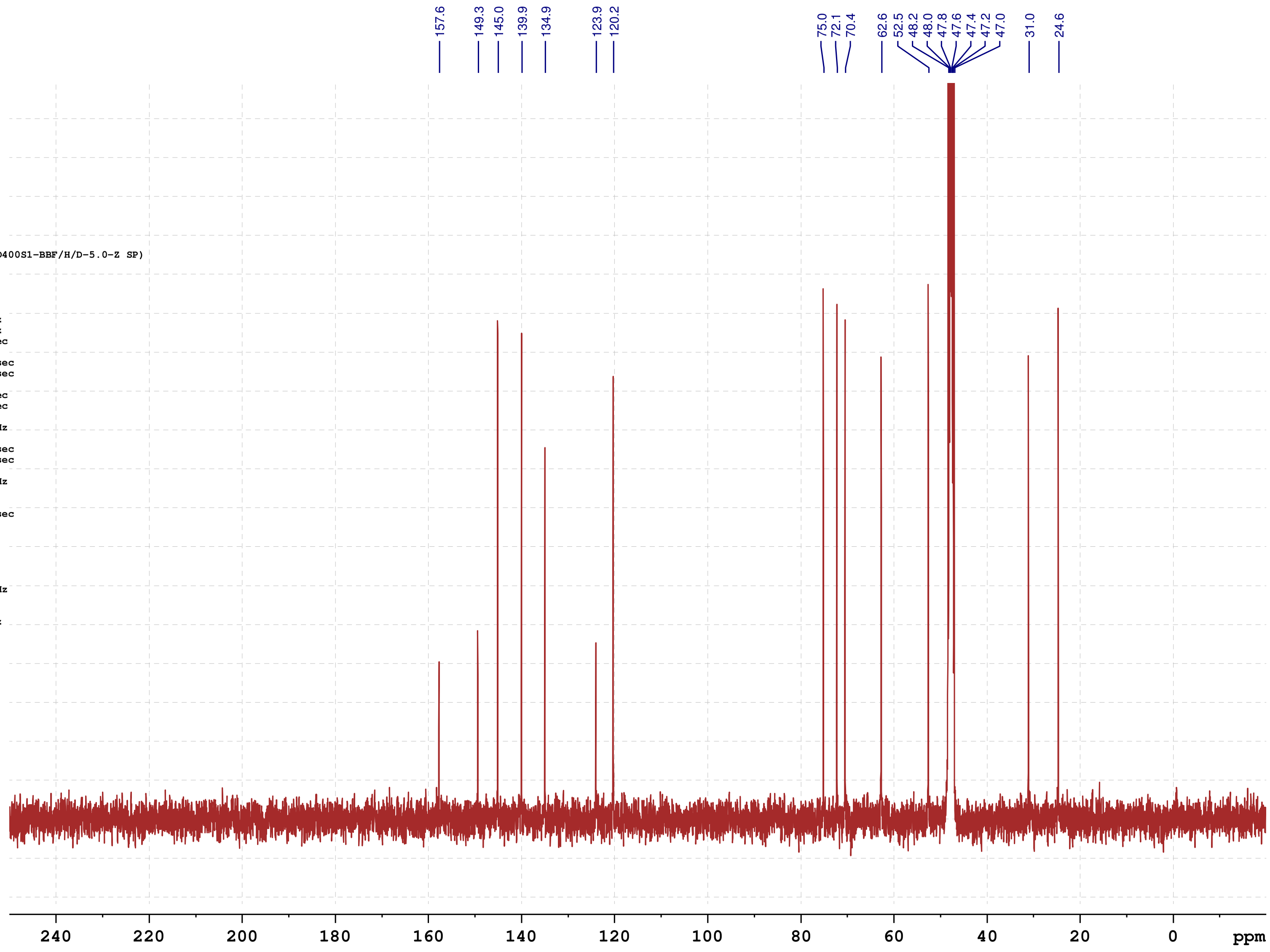

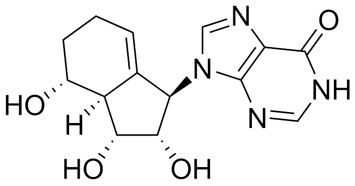

8a

NMR-Spectra for Compound 8b

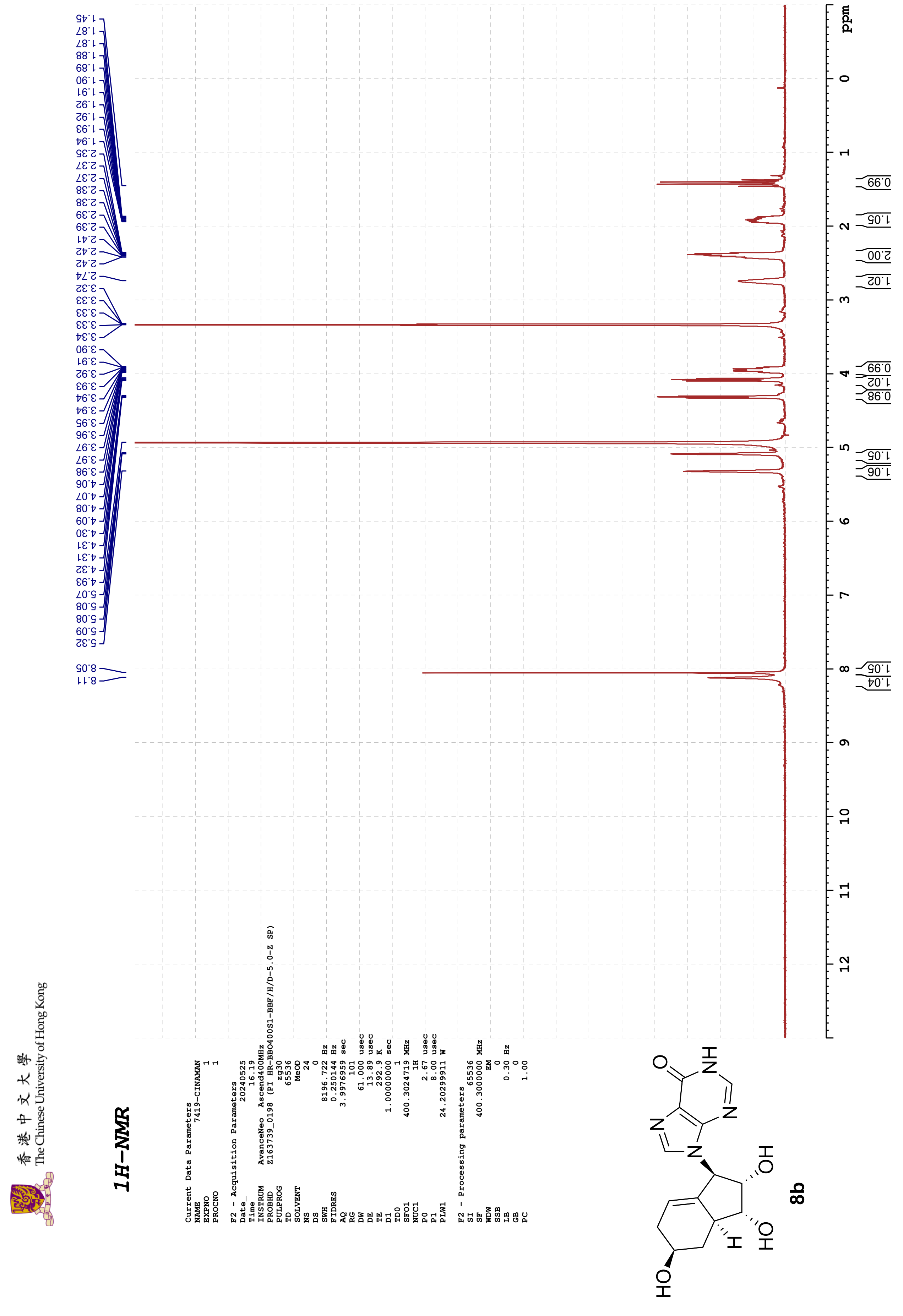

NMR-Spectra for Compound 8b

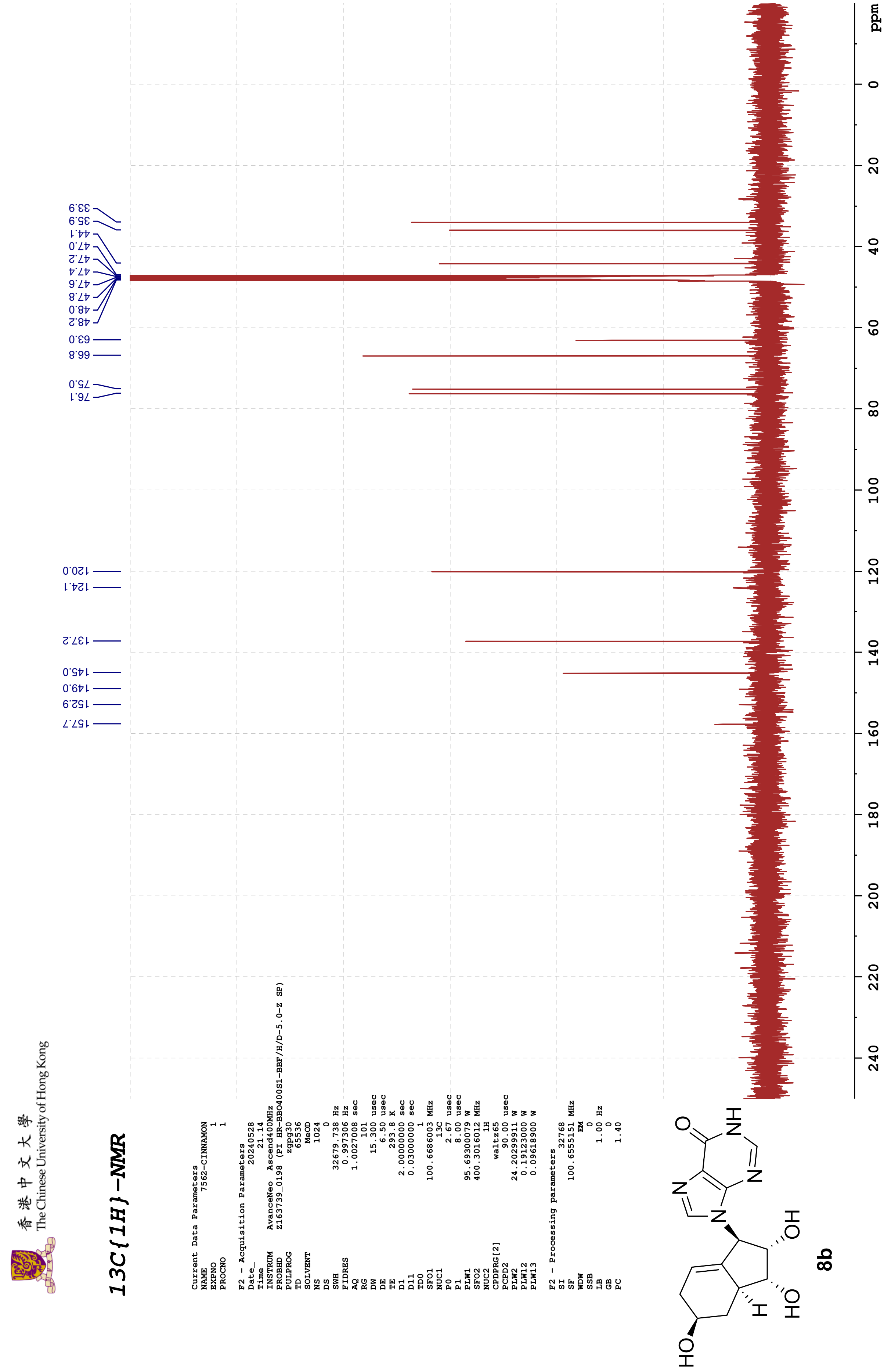

NMR-Spectra for Compound 8c

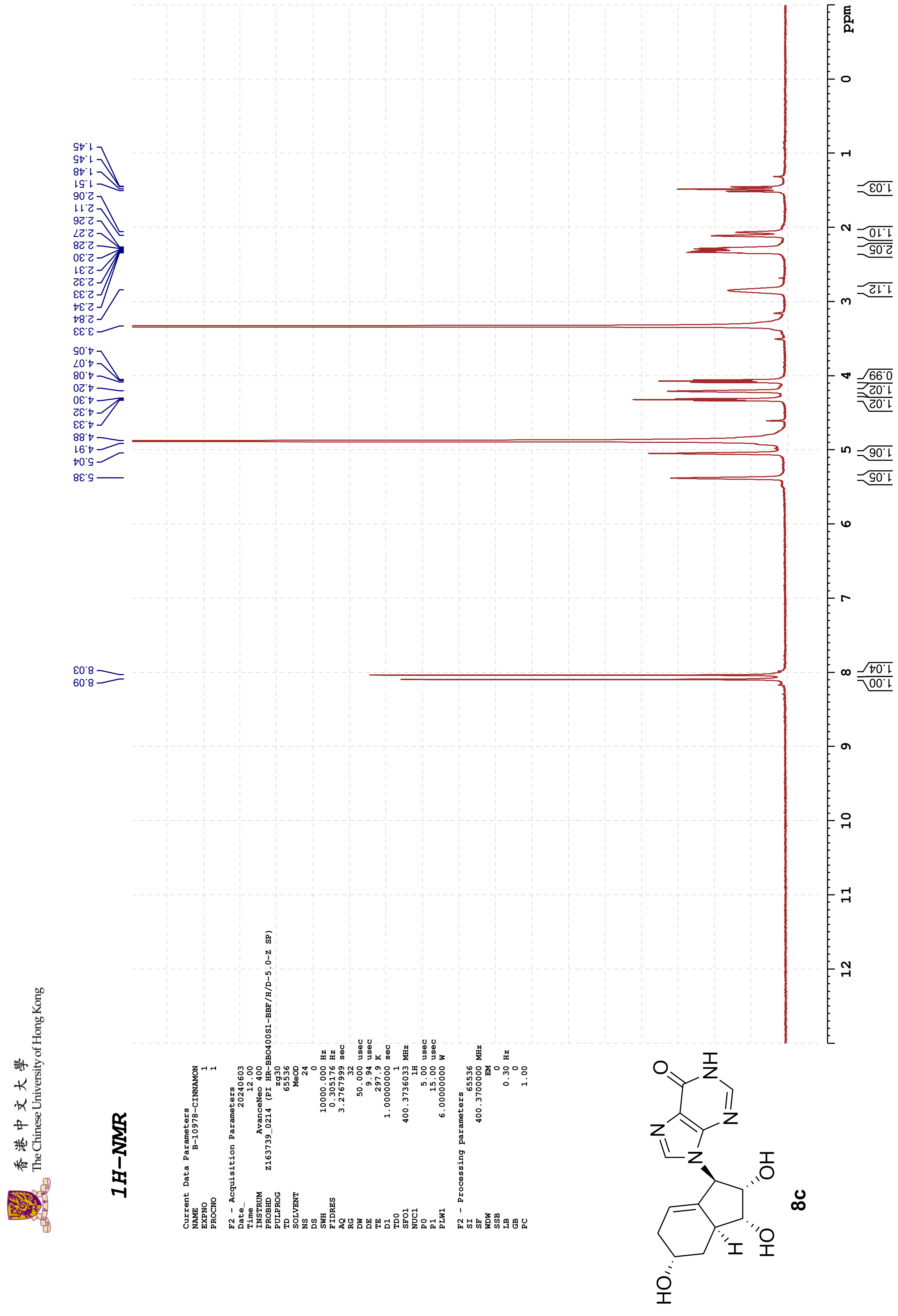

NMR-Spectra for Compound 8c

香港中文大學
The Chinese University of Hong Kong

13C{1H}-NMR

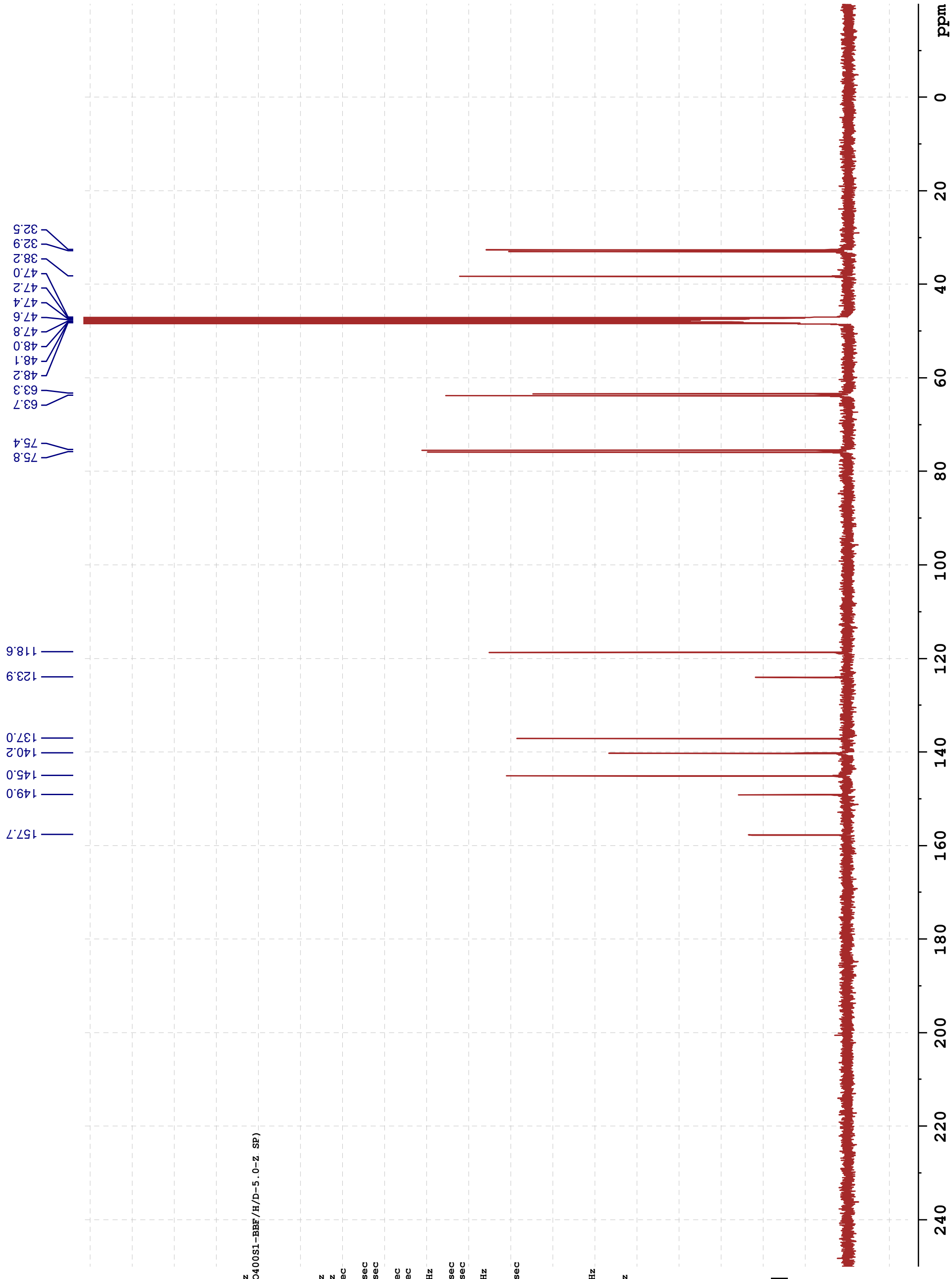


```
Current Data Parameters
NAME            7809-CINNAMON
EXPNO                       1
PROCNO                      1

F2 - Acquisition Parameters
Date_                20240604
Time                    22.46
INSTRUM    AvanceNeo  Ascend400MHz
PROBHD     Z163739_0198 (PI HR-BBO400S1-BBF/H/D-5.0-Z SP)
PULPROG                zgpg30
TD                      65536
SOLVENT                  MeOD
NS                       2500
DS                          0
SWH                 32679.738 Hz
FIDRES               0.997306 Hz
AQ                  1.0027008 sec
RG                        101
DW                     15.300 usec
DE                       6.50 usec
TE                      294.0 K
D1                 2.00000000 sec
D11                0.03000000 sec
TD0                         1
SFO1              100.6686003 MHz
NUC1                      13C
P0                       2.67 usec
P1                       8.00 usec
PLW1              95.69300079 W
SFO2              400.3016012 MHz
NUC2                       1H
CPDPRG[2]             waltz65
PCPD2                   90.00 usec
PLW2              24.20299911 W
PLW12              0.19123000 W
PLW13              0.09618900 W

F2 - Processing parameters
SI                      32768
SF                100.6555151 MHz
WDW                        EM
SSB                         0
LB                       1.00 Hz
GB                          0
PC                       1.40
```

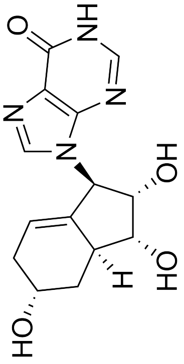

8c

NMR-Spectra for Compound 8e

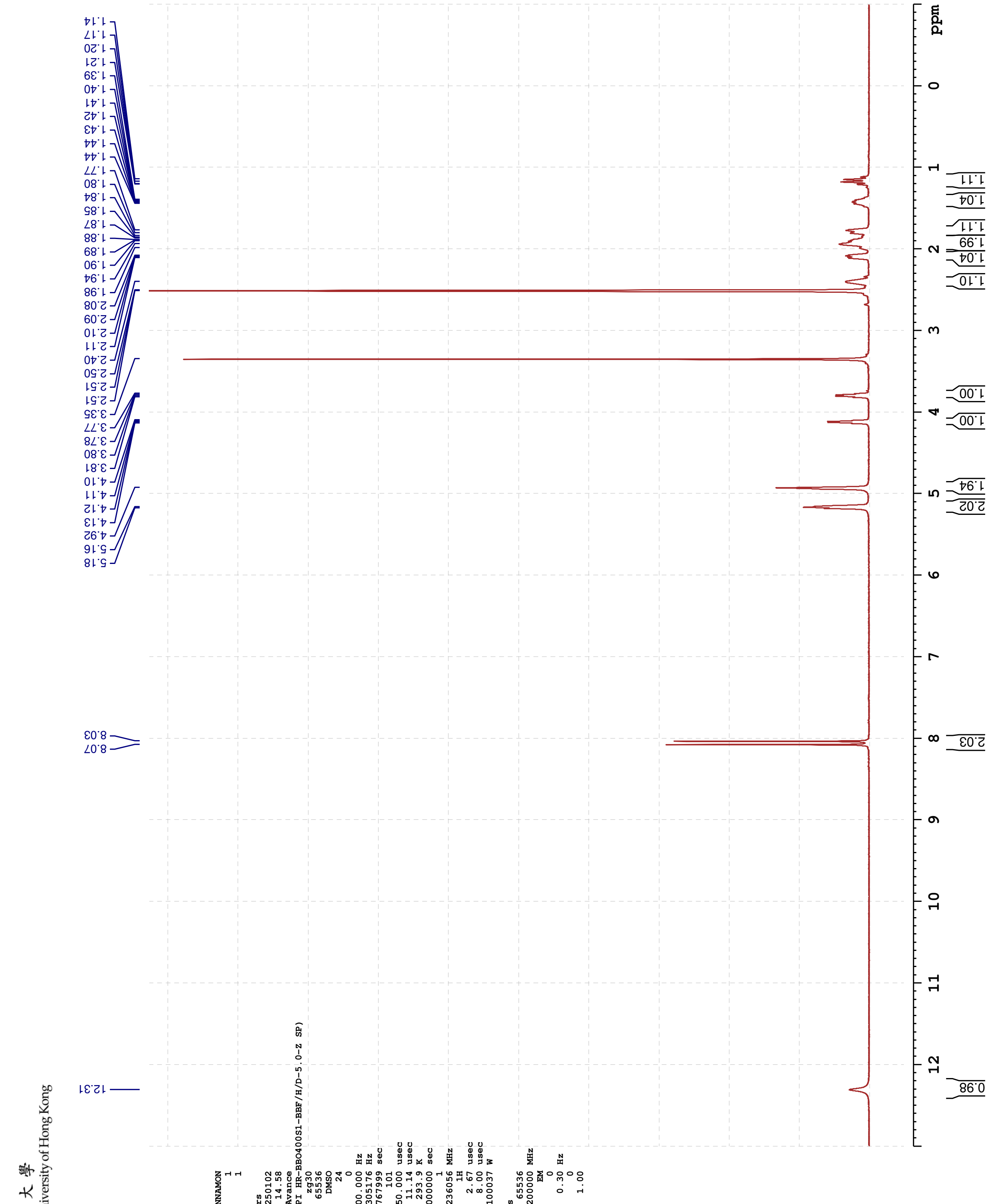

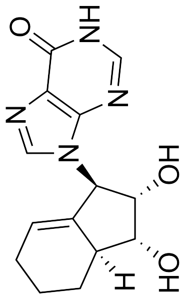

8e

NMR-Spectra for Compound 8e

香港中文大學
The Chinese University of Hong Kong

13C{1H}-NMR

```
Current Data Parameters
NAME          C-579-CINNAMONCMR
EXPNO                         1
PROCNO                        1

F2 - Acquisition Parameters
Date_                  20250106
Time                      13.30
INSTRUM                  Avance
PROBHD      Z163739_1043 (PI HR-BBO400S1-BBF/H/D-5.0-Z SP)
PULPROG                 zgpg30
TD                        65536
SOLVENT                    DMSO
NS                         1024
DS                            0
SWH                   32679.739 Hz
FIDRES                 0.997306 Hz
AQ                    1.0027008 sec
RG                         12.7
DW                       15.300 usec
DE                         6.50 usec
TE                        295.0 K
D1                   2.00000000 sec
D11                  0.03000000 sec
TD0                           1
SFO1                100.7490748 MHz
NUC1                        13C
P0                         2.67 usec
P1                         8.00 usec
PLW1                96.23899841 W
SFO2                400.6216025 MHz
NUC2                         1H
CPDPRG2                 waltz65
PCPD2                     90.00 usec
PLW2                21.54100037 W
PLW12                0.17020001 W
PLW13                0.08560800 W

F2 - Processing parameters
SI                        32768
SF                  100.7359791 MHz
WDW                          EM
SSB                           0
LB                         1.00 Hz
GB                            0
PC                         1.40
```

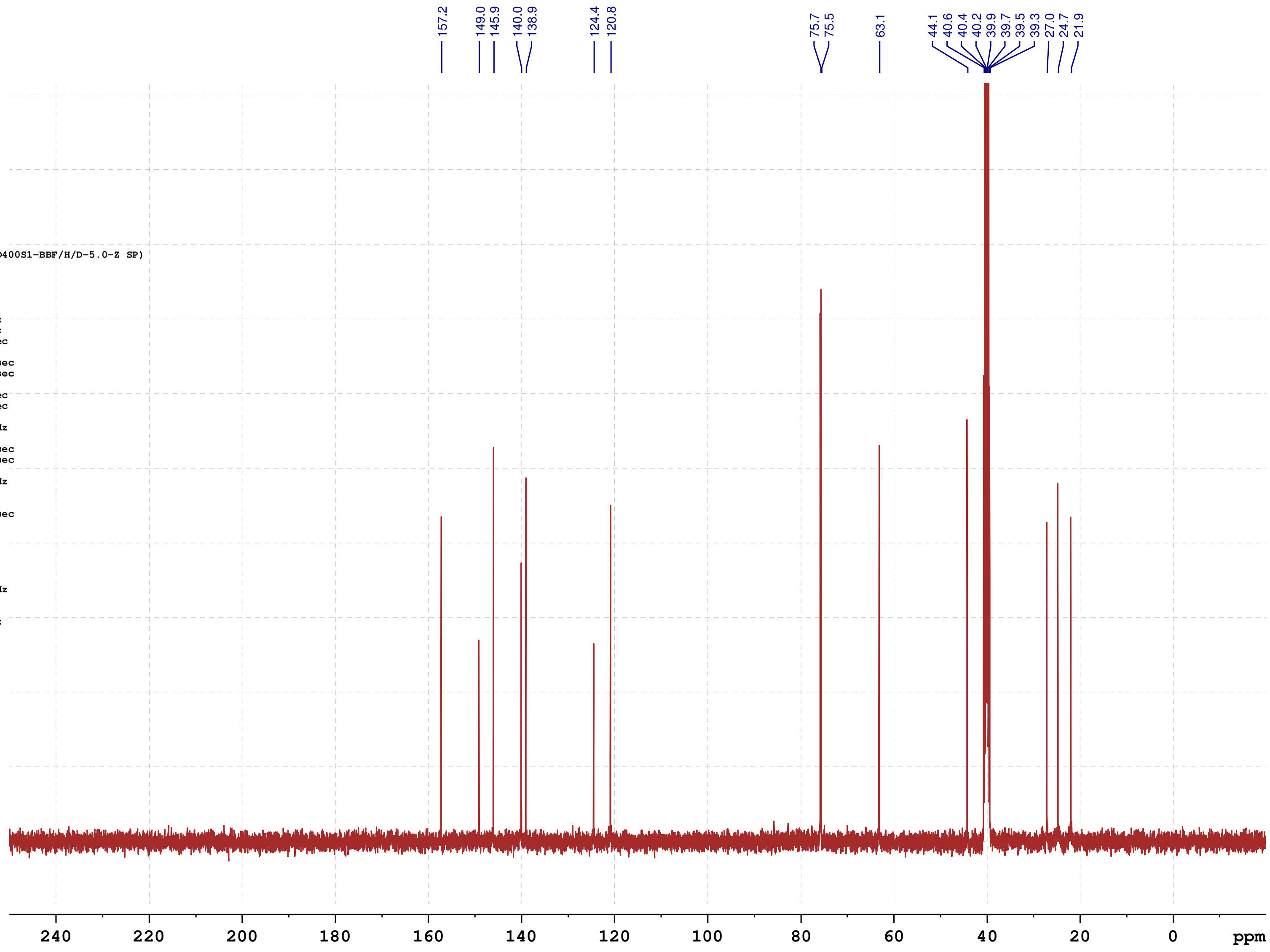

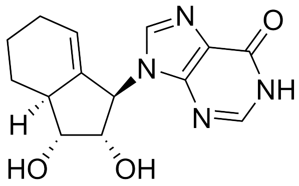

8e

NMR-Spectra for Compound 9a

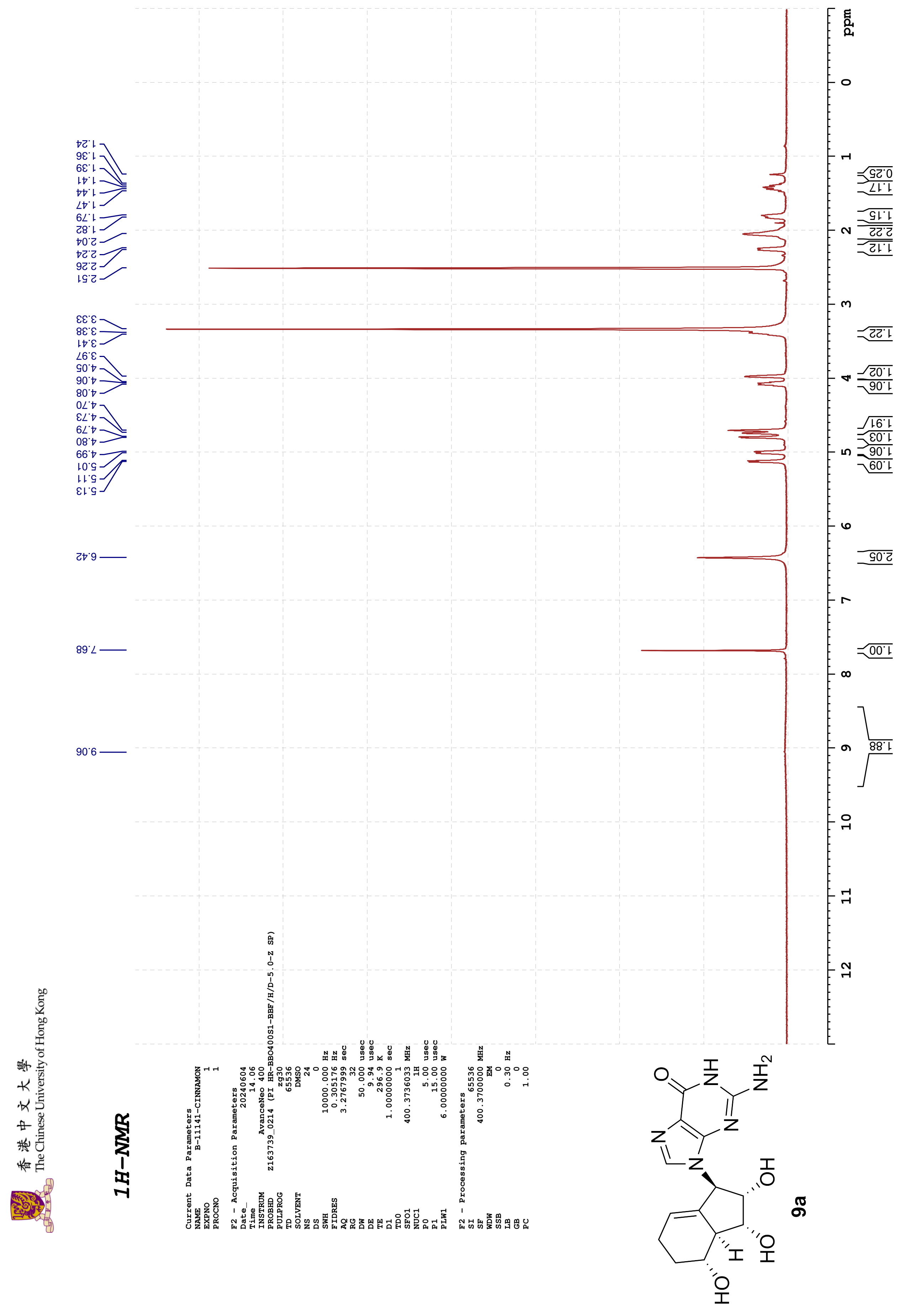

NMR-Spectra for Compound 9a

香港中文大學 The Chinese University of Hong Kong

13C{1H}-NMR

```
Current Data Parameters
NAME              7875-CINAMAN
EXPNO                        1
PROCNO                       1

F2 - Acquisition Parameters
Date_                 20240605
Time                     19.26
INSTRUM     AvanceNeo  Ascend400MHz
PROBHD      Z163739_0198 (PI HR-BBO400S1-BBF/H/D-5.0-Z SP)
PULPROG                zgpg30
TD                      65536
SOLVENT                  DMSO
NS                       1024
DS                          0
SWH                 32679.738 Hz
FIDRES               0.997306 Hz
AQ                  1.0027008 sec
RG                        101
DW                     15.300 usec
DE                       6.50 usec
TE                      293.4 K
D1                 2.00000000 sec
D11                0.03000000 sec
TD0                         1
SFO1              100.6686003 MHz
NUC1                      13C
P0                       2.67 usec
P1                       8.00 usec
PLW1              95.69300079 W
SFO2              400.3016012 MHz
NUC2                       1H
CPDPRG[2]             waltz65
PCPD2                   90.00 usec
PLW2              24.20299911 W
PLW12              0.19123000 W
PLW13              0.09618900 W

F2 - Processing parameters
SI                      32768
SF                100.6555151 MHz
WDW                        EM
SSB                         0
LB                       1.00 Hz
GB                          0
PC                       1.40
```

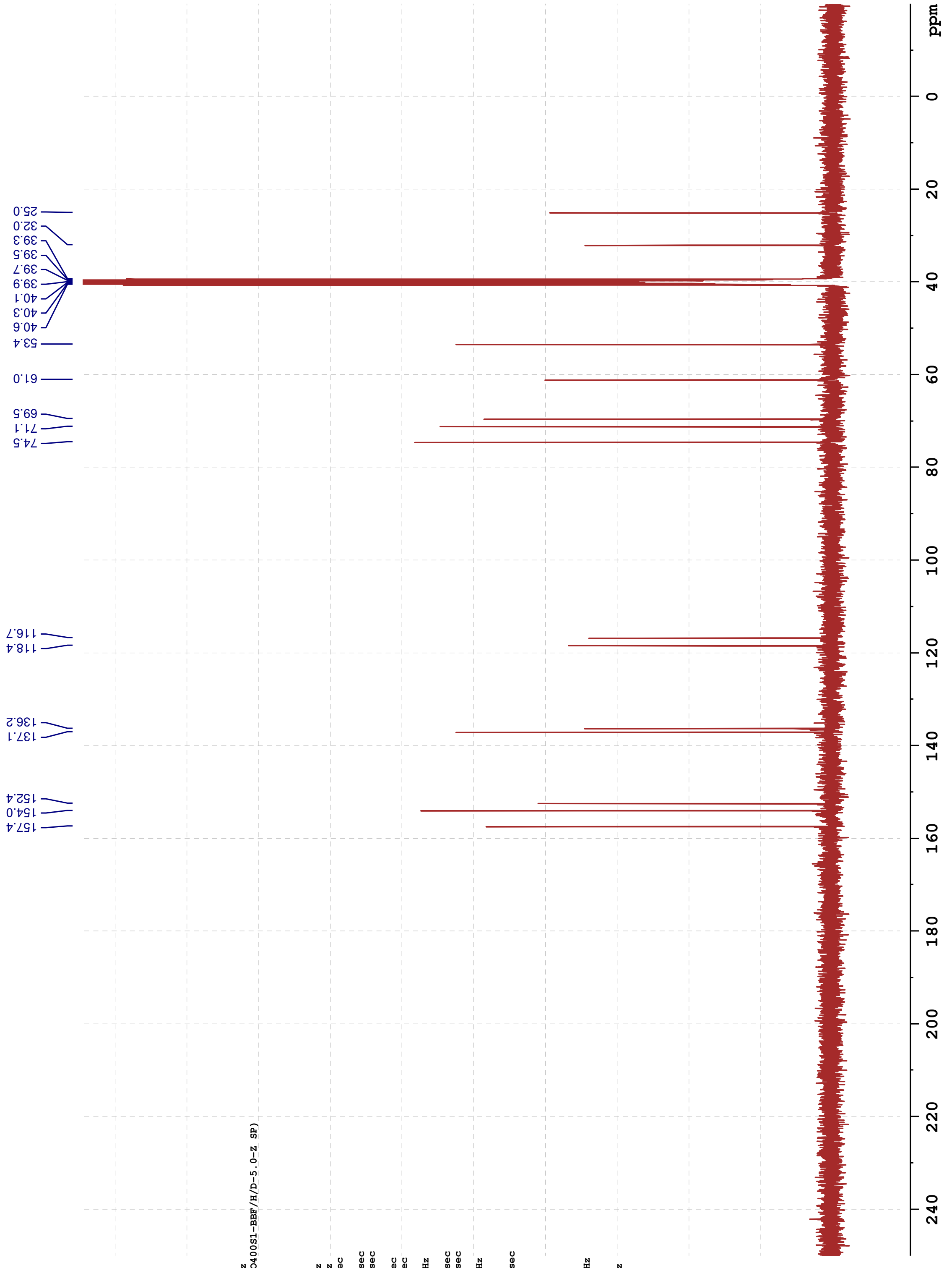

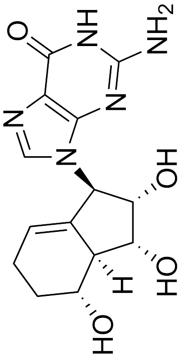

9a

NMR-Spectra for Compound 9b

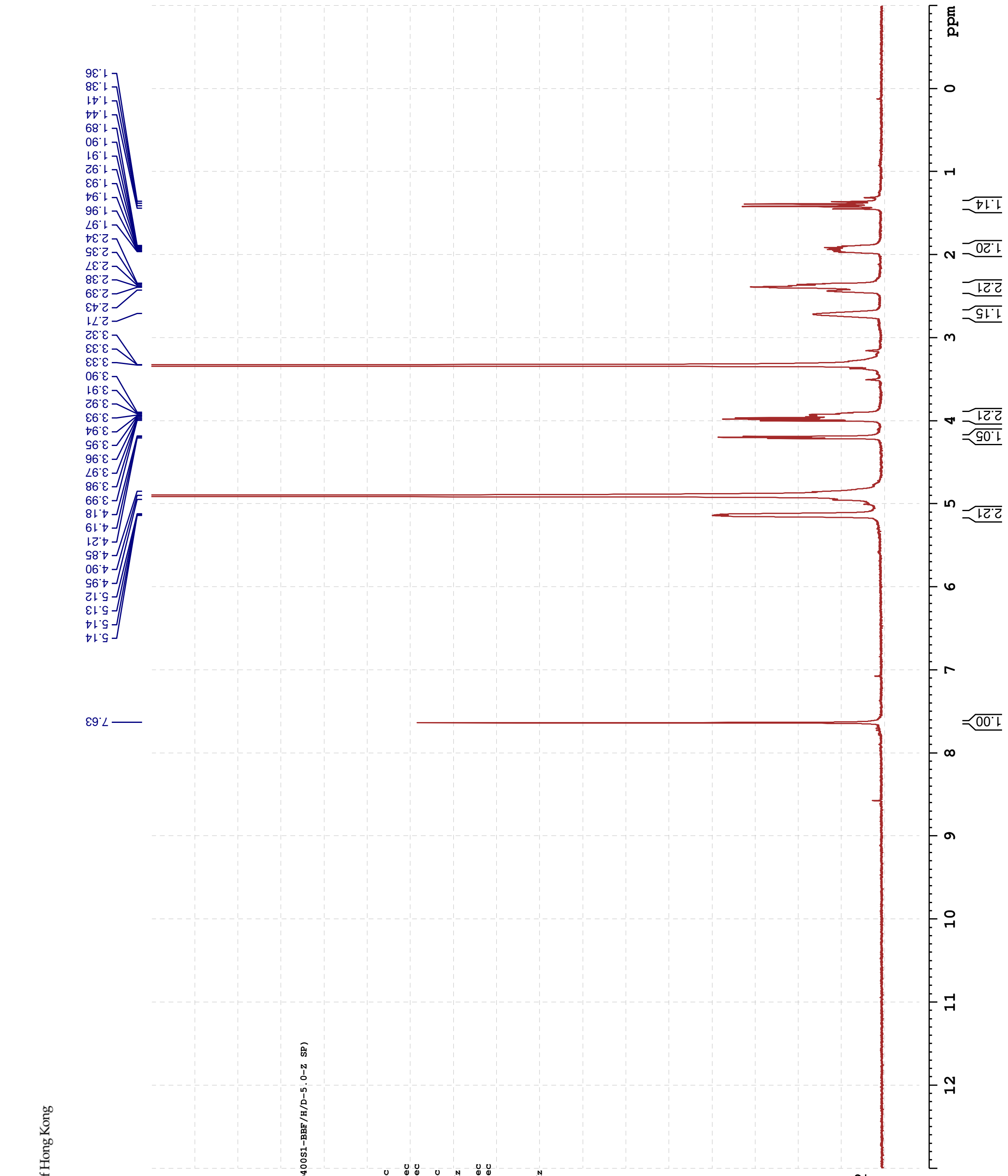

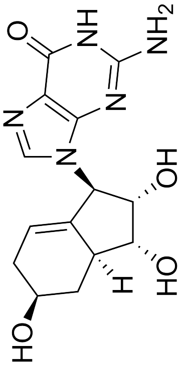

9b

NMR-Spectra for Compound 9b

The Chinese University of Hong Kong 香港中文大學

13C{1H}-NMR

```
Current Data Parameters
NAME              7952-CINNAMON
EXPNO                         1
PROCNO                        1

F2 - Acquisition Parameters
Date_                  20240607
Time                       3.33
INSTRUM    AvanceNeo  Ascend400MHz
PROBHD     Z163739_0198 (PI HR-BBO400S1-BBF/H/D-5.0-Z SP)
PULPROG                  zgpg30
TD                        65536
SOLVENT                    MeOD
NS                         5000
DS                            0
SWH                   32679.738 Hz
FIDRES                 0.997306 Hz
AQ                    1.0027008 sec
RG                          101
DW                       15.300 usec
DE                         6.50 usec
TE                        293.5 K
D1                   2.00000000 sec
D11                  0.03000000 sec
TD0                           1
SFO1                100.6686003 MHz
NUC1                        13C
P0                         2.67 usec
P1                         8.00 usec
PLW1                95.69300079 W
SFO2                400.3016012 MHz
NUC2                         1H
CPDPRG[2]               waltz65
PCPD2                     90.00 usec
PLW2                24.20299911 W
PLW12                0.19123000 W
PLW13                0.09618900 W

F2 - Processing parameters
SI                        32768
SF                  100.6555151 MHz
WDW                          EM
SSB                           0
LB                         2.00 Hz
GB                            0
PC                         1.40
```

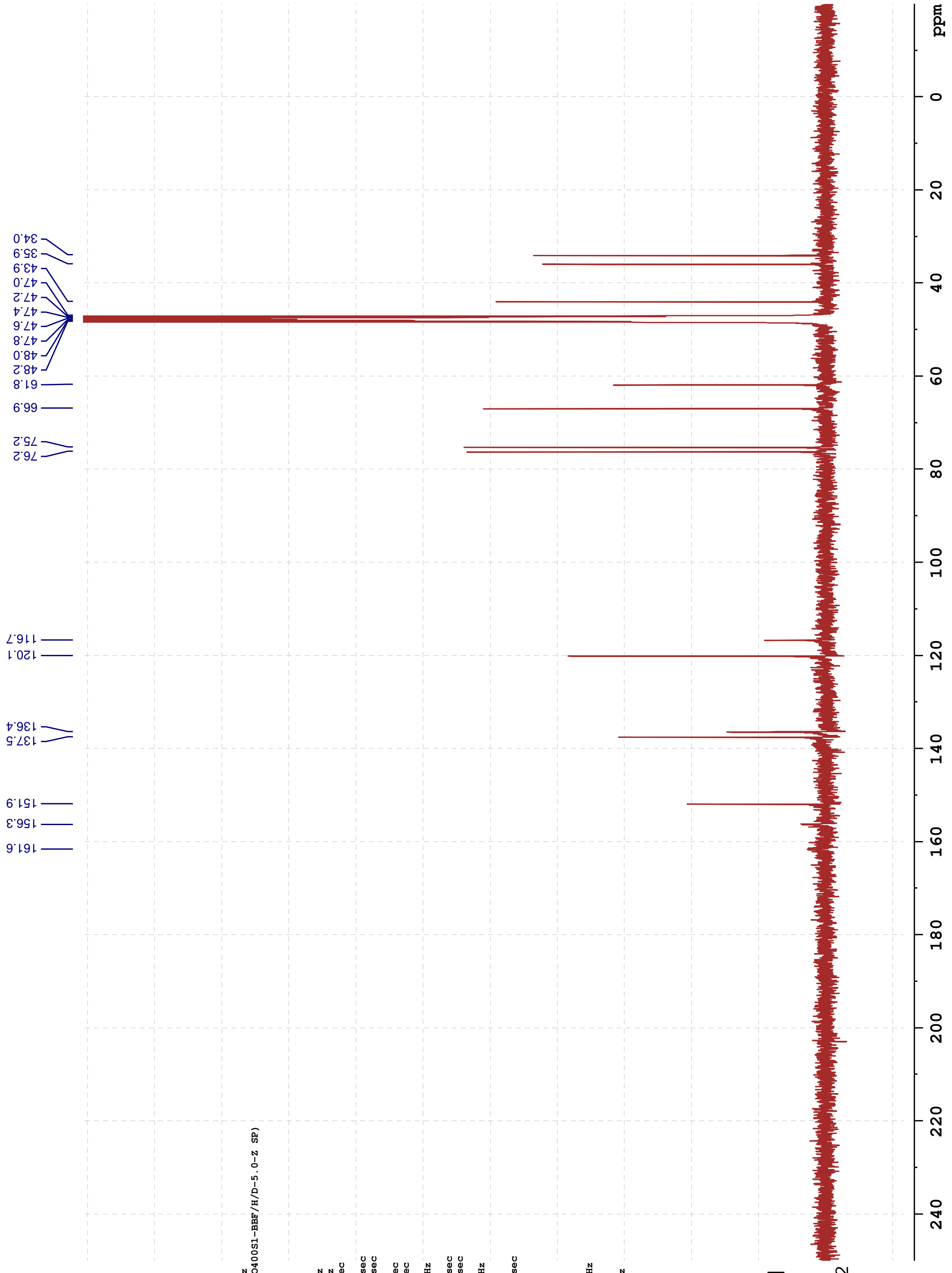

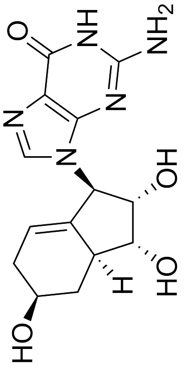

9b

NMR-Spectra for Compound 9c

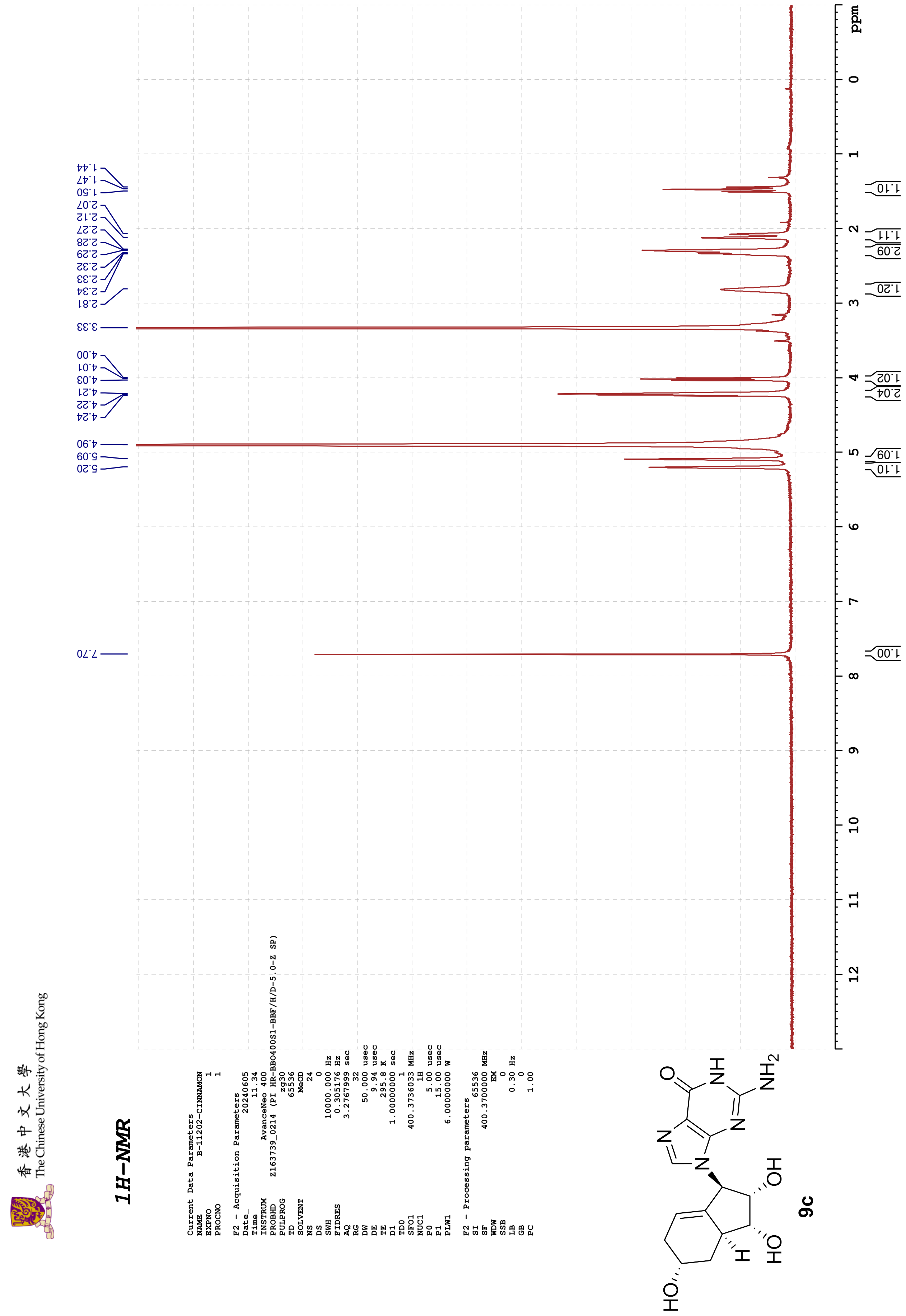

NMR-Spectra for Compound 9c

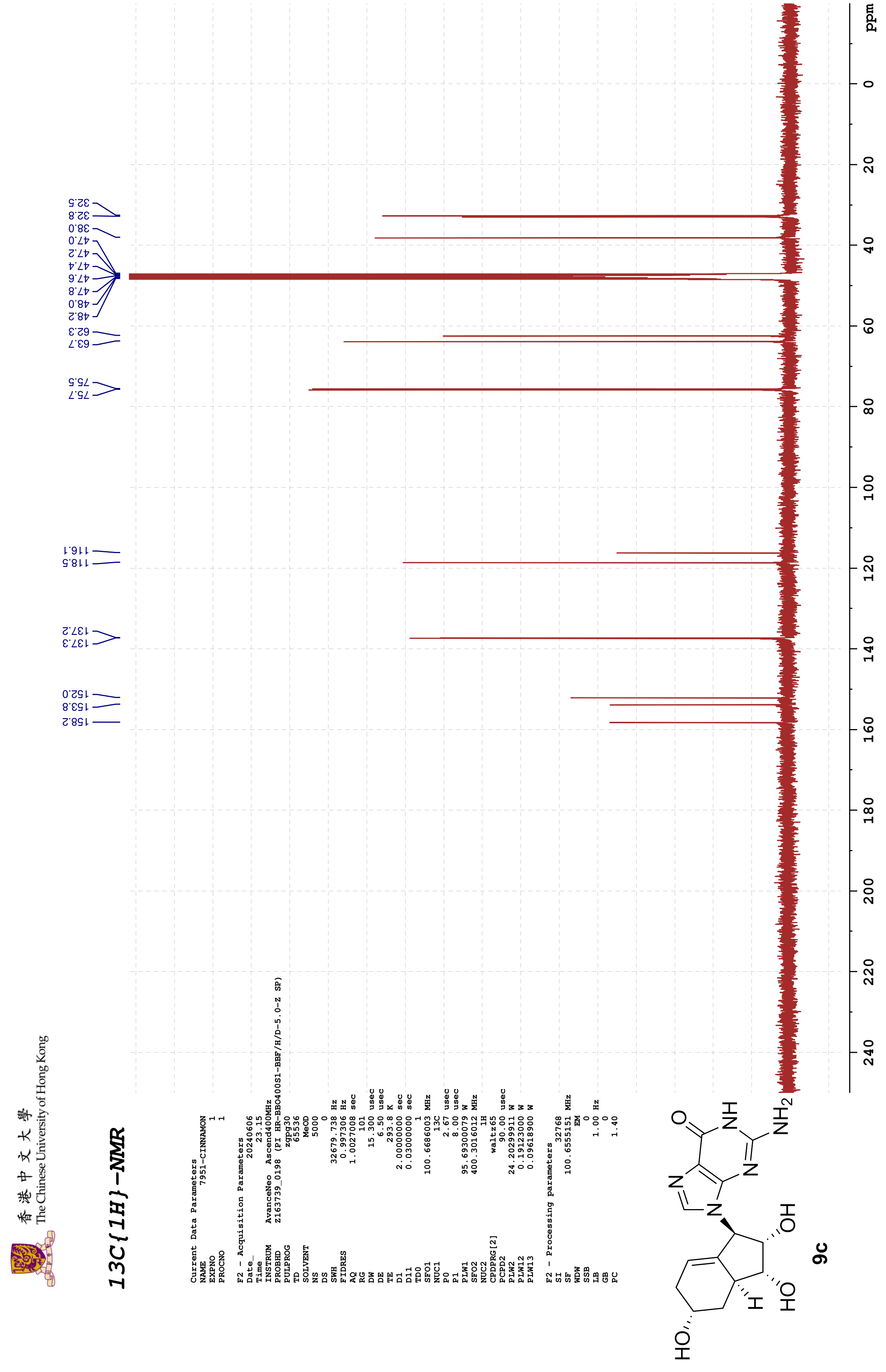

NMR-Spectra for Compound 10a

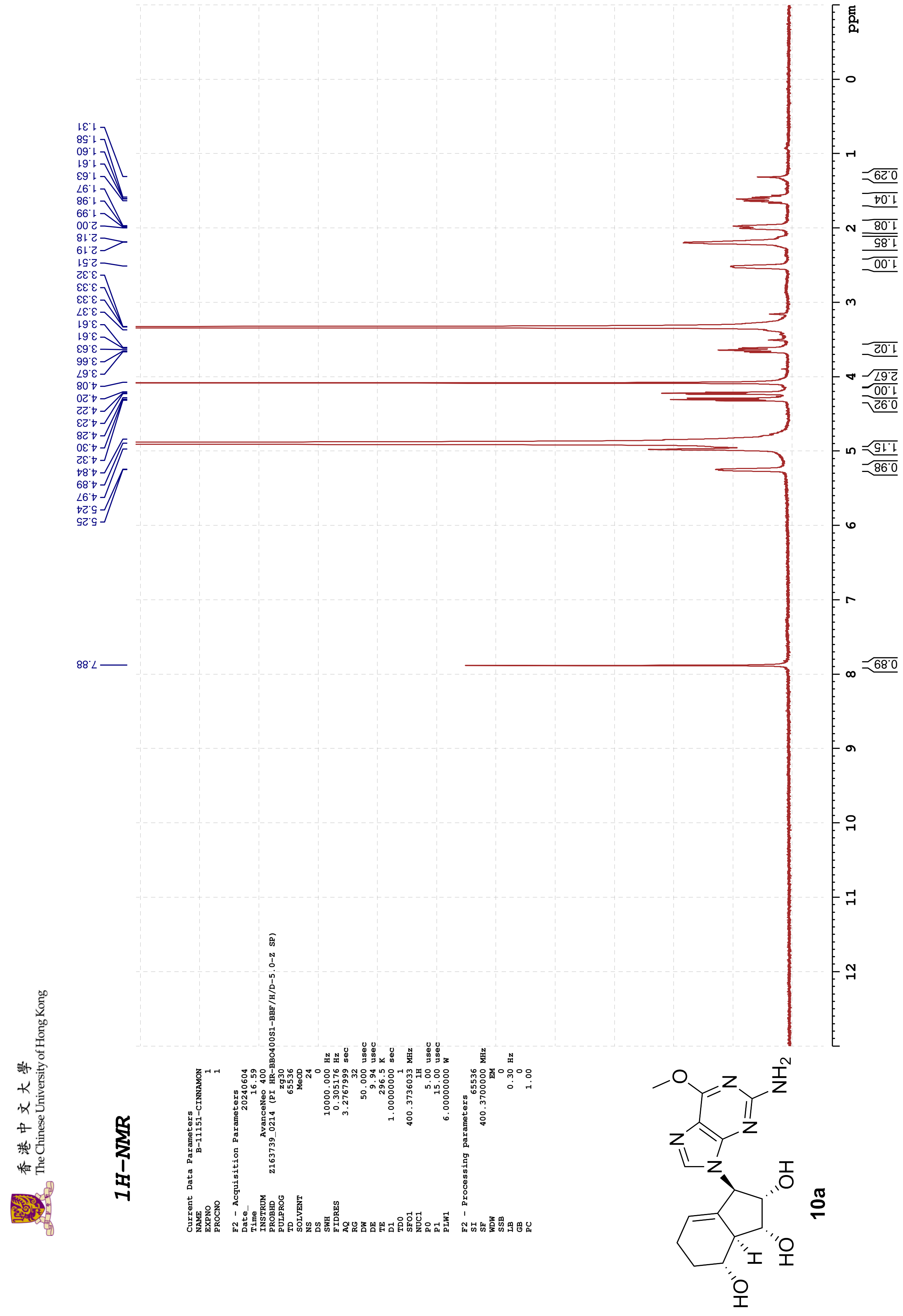

NMR-Spectra for Compound 10a

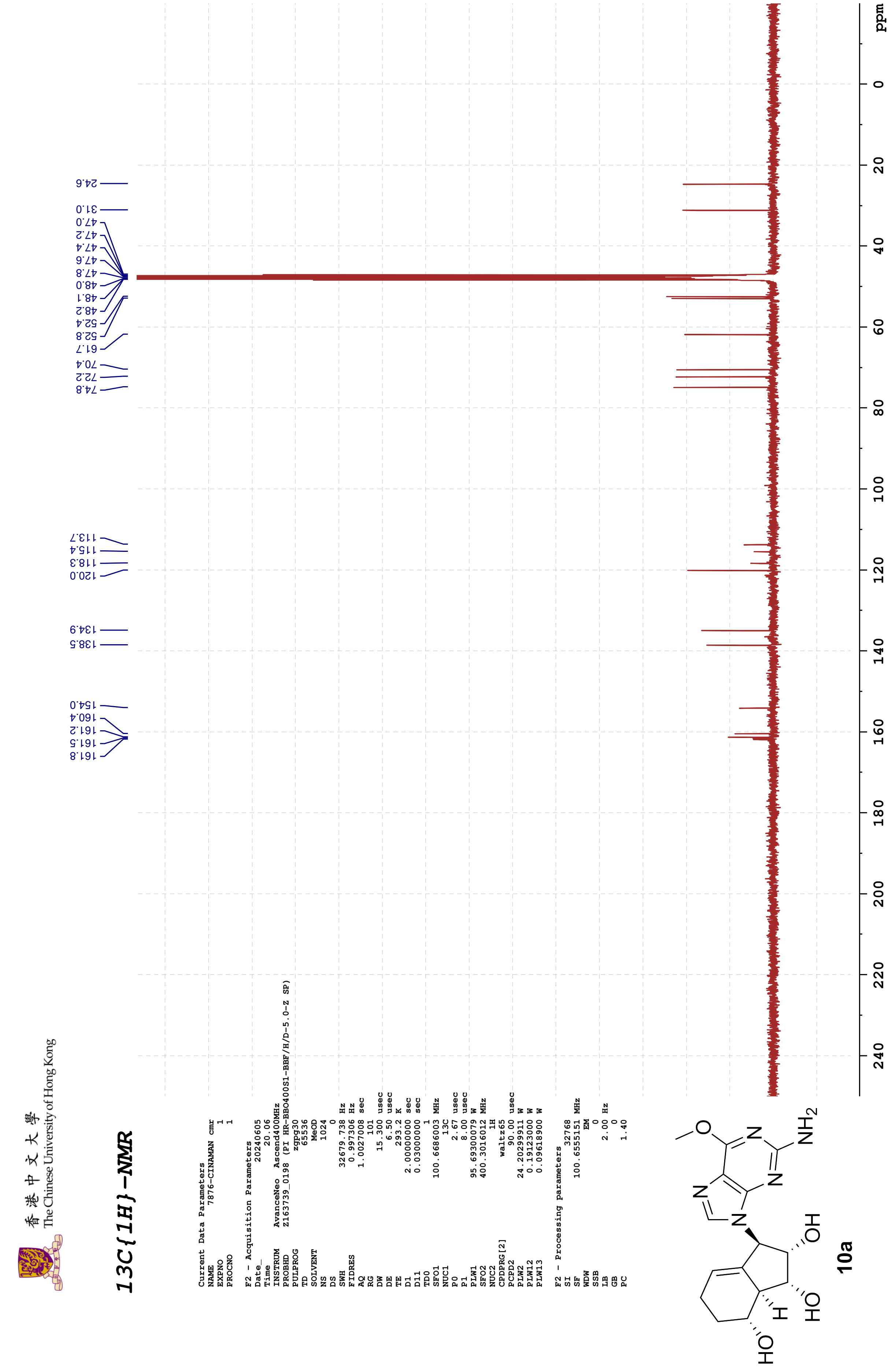

10a

NMR-Spectra for Compound 10b

香港中文大學 The Chinese University of Hong Kong

1H-NMR

```
Current Data Parameters
NAME            B-13851-CINNAMON
EXPNO                          1
PROCNO                         1

F2 - Acquisition Parameters
Date_                   20240709
Time                       14.41
INSTRUM           AvanceNeo 400
PROBHD     Z163739_0214 (PI HR-BBO400S1-BBF/H/D-5.0-Z SP)
PULPROG                     zg30
TD                         65536
SOLVENT                     MeOD
NS                            24
DS                             0
SWH                    10000.000 Hz
FIDRES                  0.305176 Hz
AQ                     3.2767999 sec
RG                            32
DW                        50.000 usec
DE                          9.94 usec
TE                         295.7 K
D1                    1.00000000 sec
TD0                            1
SFO1                 400.3736033 MHz
NUC1                          1H
P0                          5.00 usec
P1                         15.00 usec
PLW1                  6.00000000 W

F2 - Processing parameters
SI                         65536
SF                   400.3700000 MHz
WDW                           EM
SSB                            0
LB                          0.30 Hz
GB                             0
PC                          1.00
```

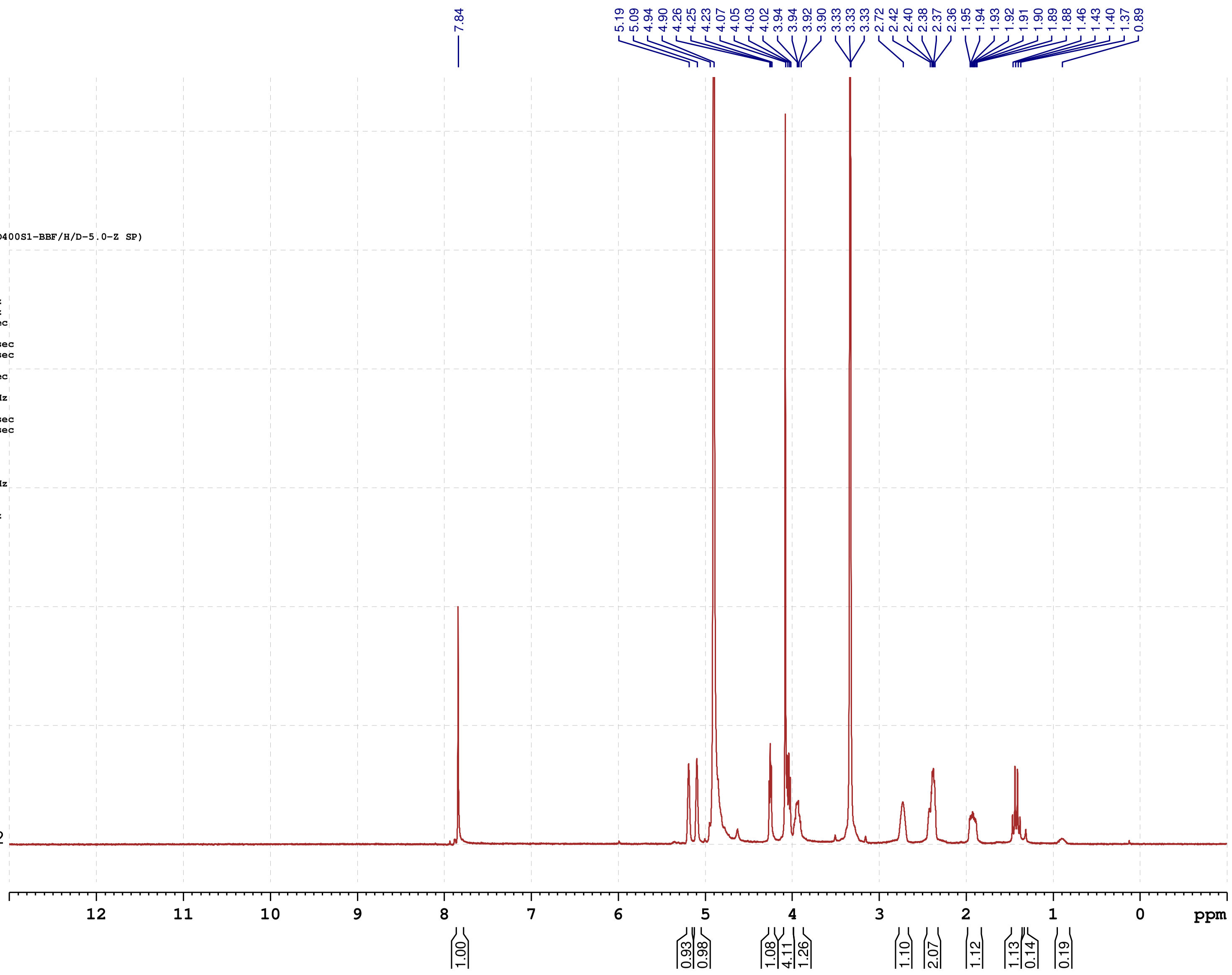

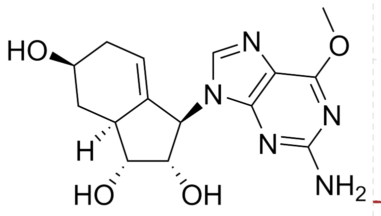

10b

NMR-Spectra for Compound 10b

香港中文大學
The Chinese University of Hong Kong

13C{1H}-NMR

```
Current Data Parameters
NAME            B-13946-CINNAMON
EXPNO                          1
PROCNO                         1

F2 - Acquisition Parameters
Date_                   20240710
Time                       11.32
INSTRUM             AvanceNeo 400
PROBHD      Z163739_0214 (PI HR-BBO400S1-BBF/H/D-5.0-Z SP)
PULPROG                  zgpg30
TD                        65536
SOLVENT                    MeOD
NS                         1024
DS                            0
SWH                   32679.738 Hz
FIDRES                 0.997306 Hz
AQ                    1.0027008 sec
RG                          101
DW                       15.300 usec
DE                         6.50 usec
TE                        296.8 K
D1                   2.00000000 sec
D11                  0.03000000 sec
TD0                           1
SFO1                100.6862041 MHz
NUC1                        13C
P0                         2.63 usec
P1                         7.90 usec
PLW1               100.30000305 W
SFO2                400.3716015 MHz
NUC2                         1H
CPDPRG[2]               waltz65
PCPD2                     90.00 usec
PLW2                24.89999962 W
PLW12                0.18703000 W
PLW13                0.09407300 W

F2 - Processing parameters
SI                        32768
SF                  100.6731166 MHz
WDW                          EM
SSB                           0
LB                         1.00 Hz
GB                            0
PC                         1.40
```

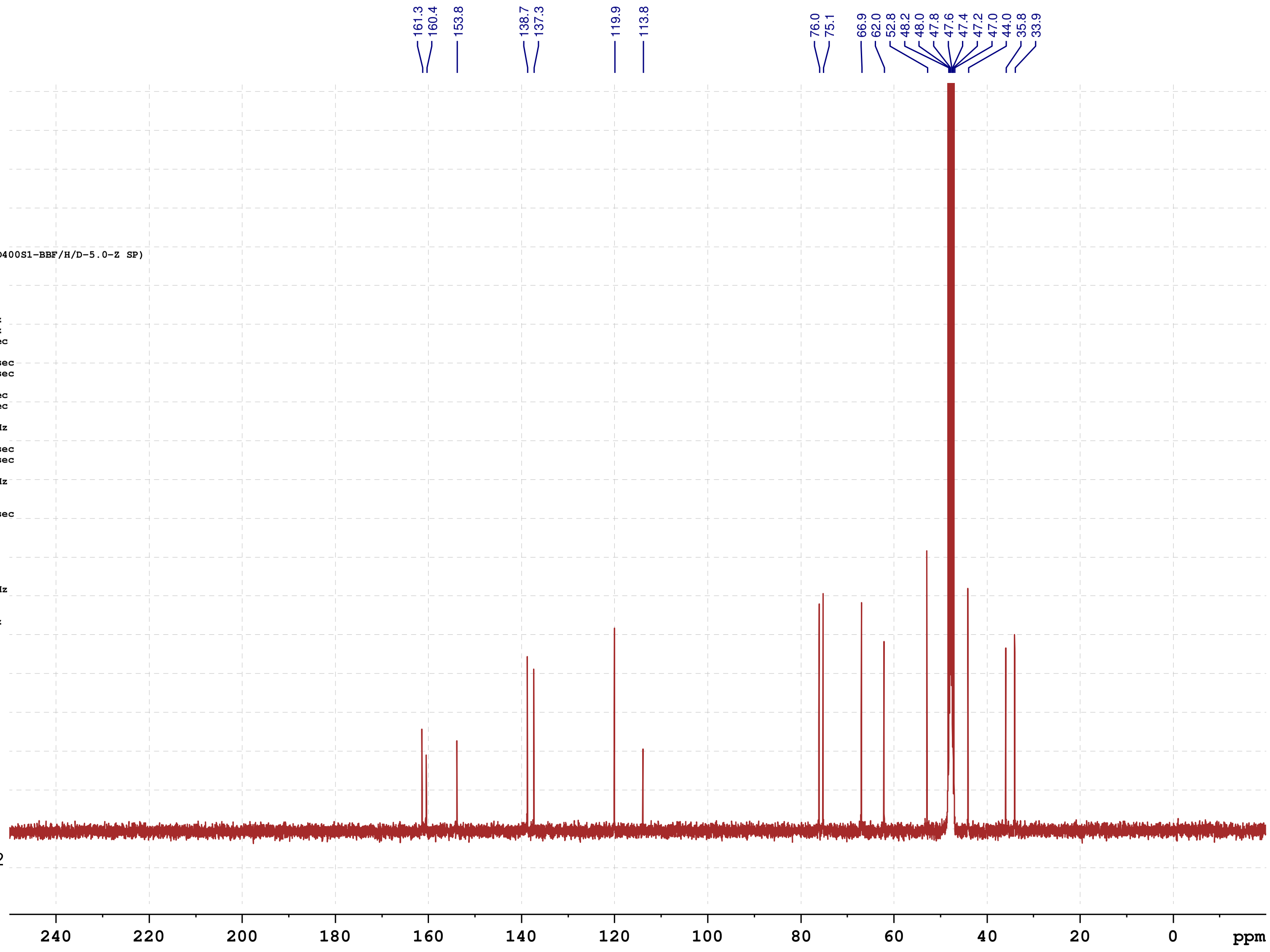

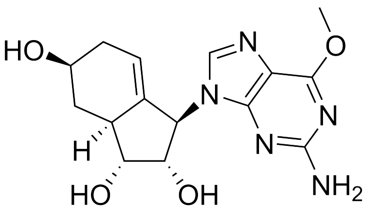

10b

NMR-Spectra for Compound 10c

香港中文大學
The Chinese University of Hong Kong

1H–NMR

```
Current Data Parameters
NAME          B-13579-CINNAMON
EXPNO                    1
PROCNO                   1

F2 - Acquisition Parameters
Date_             20240704
Time                 17.41
INSTRUM       AvanceNeo 400
PROBHD     Z163739_0214 (PI HR-BBO400S1-BBF/H/D-5.0-Z SP)
PULPROG               zg30
TD                   65536
SOLVENT               MeOD
NS                      24
DS                       0
SWH             10000.000 Hz
FIDRES           0.305176 Hz
AQ              3.2767999 sec
RG                      32
DW                  50.000 usec
DE                    9.94 usec
TE                   298.2 K
D1              1.00000000 sec
TD0                      1
SFO1           400.3736033 MHz
NUC1                    1H
P0                    5.00 usec
P1                   15.00 usec
PLW1            6.00000000 W

F2 - Processing parameters
SI                   65536
SF             400.3700000 MHz
WDW                     EM
SSB                      0
LB                    0.30 Hz
GB                       0
PC                    1.00
```

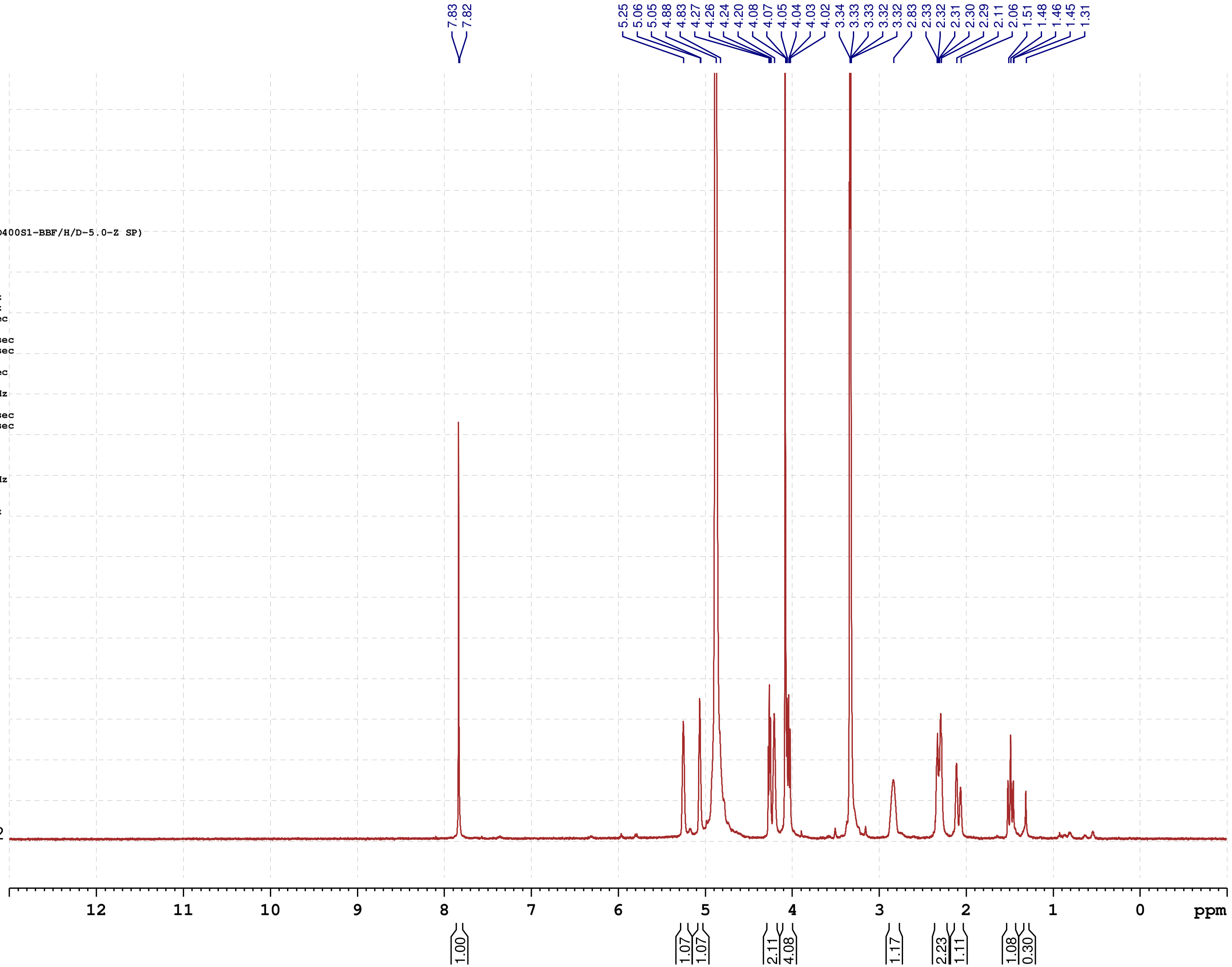

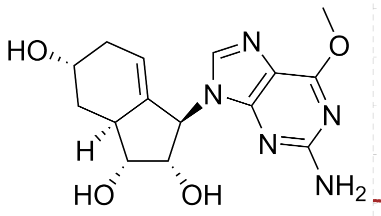

10c

NMR-Spectra for Compound 10c

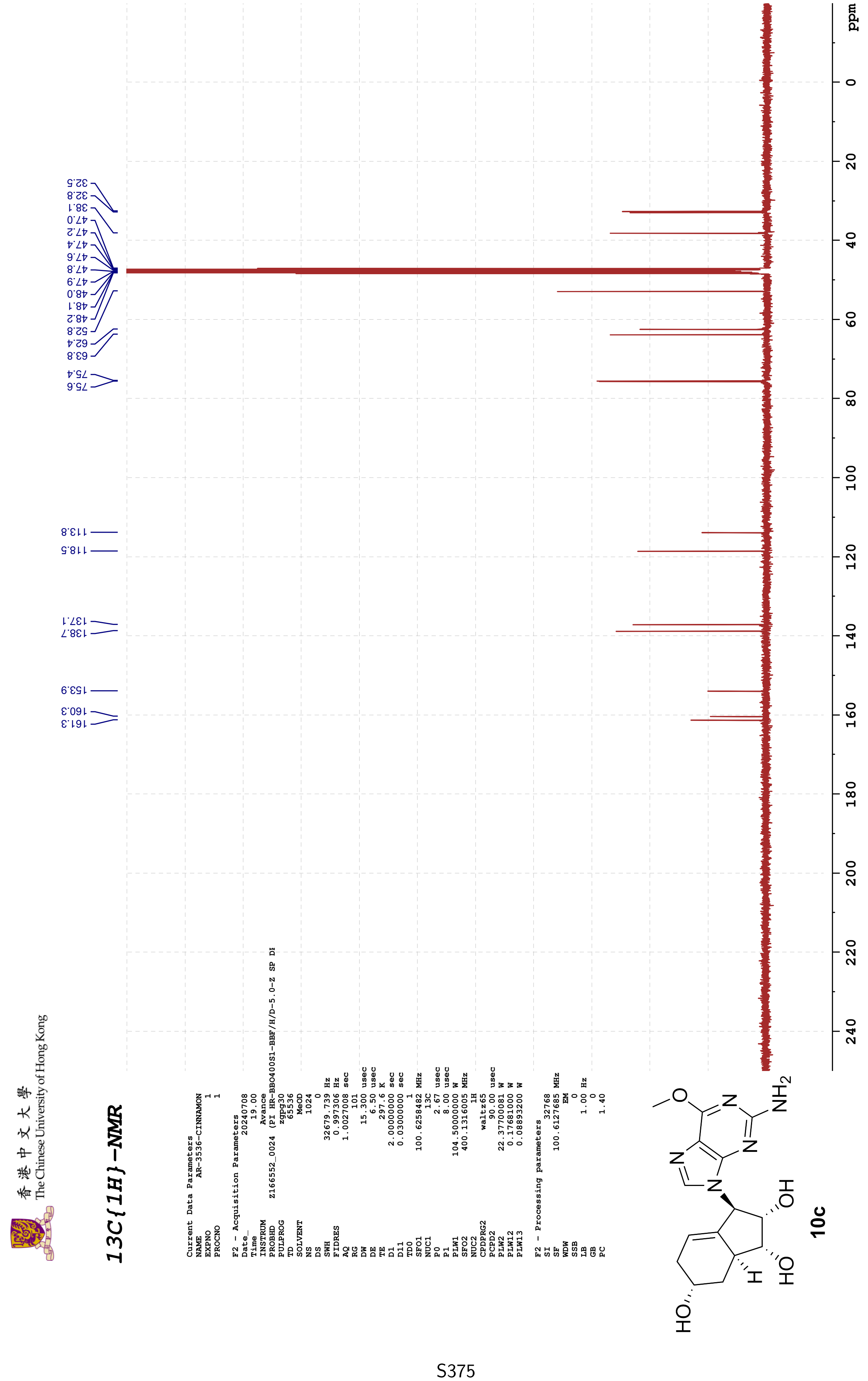

NMR-Spectra for Compound 11a

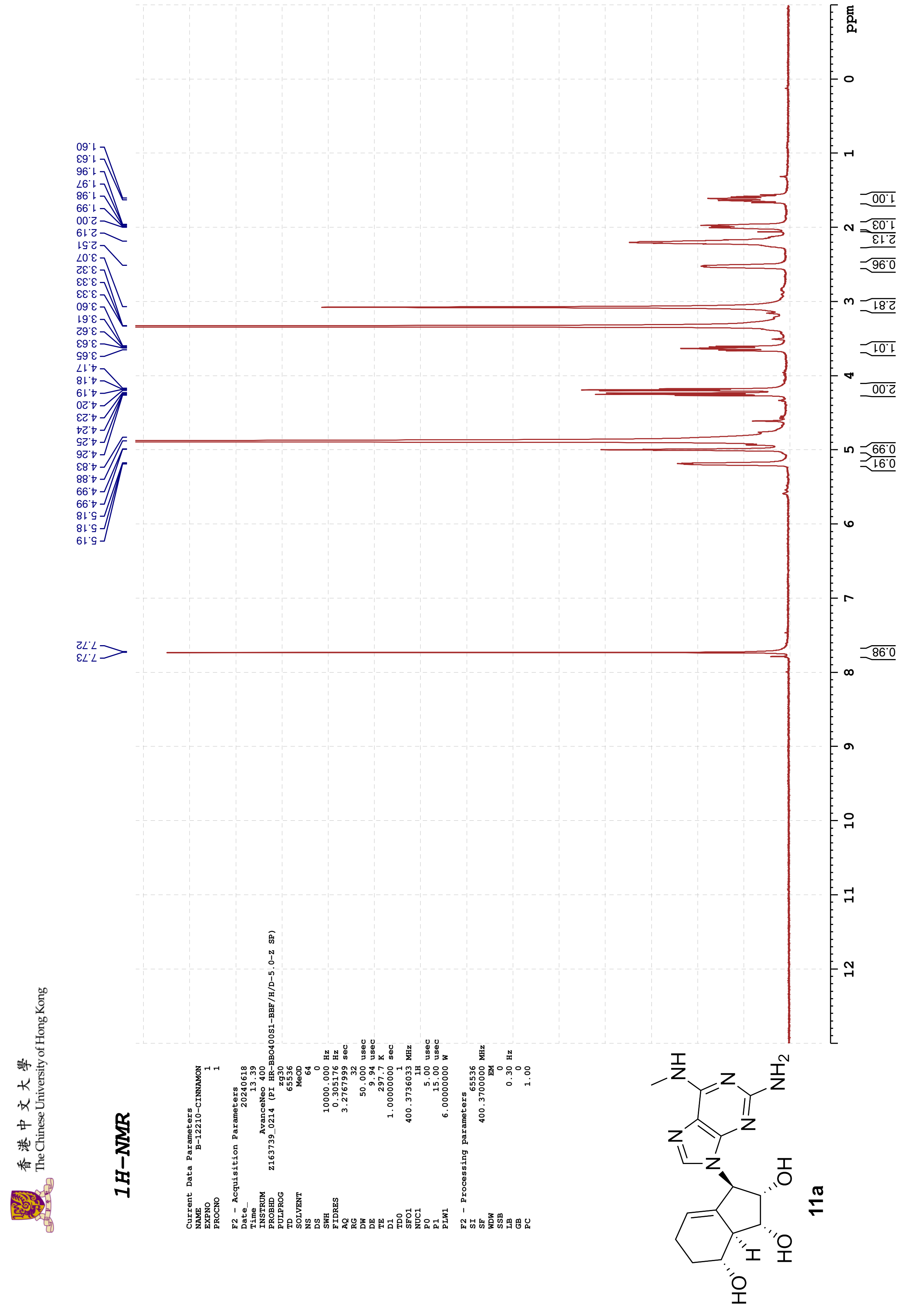

NMR-Spectra for Compound 11a

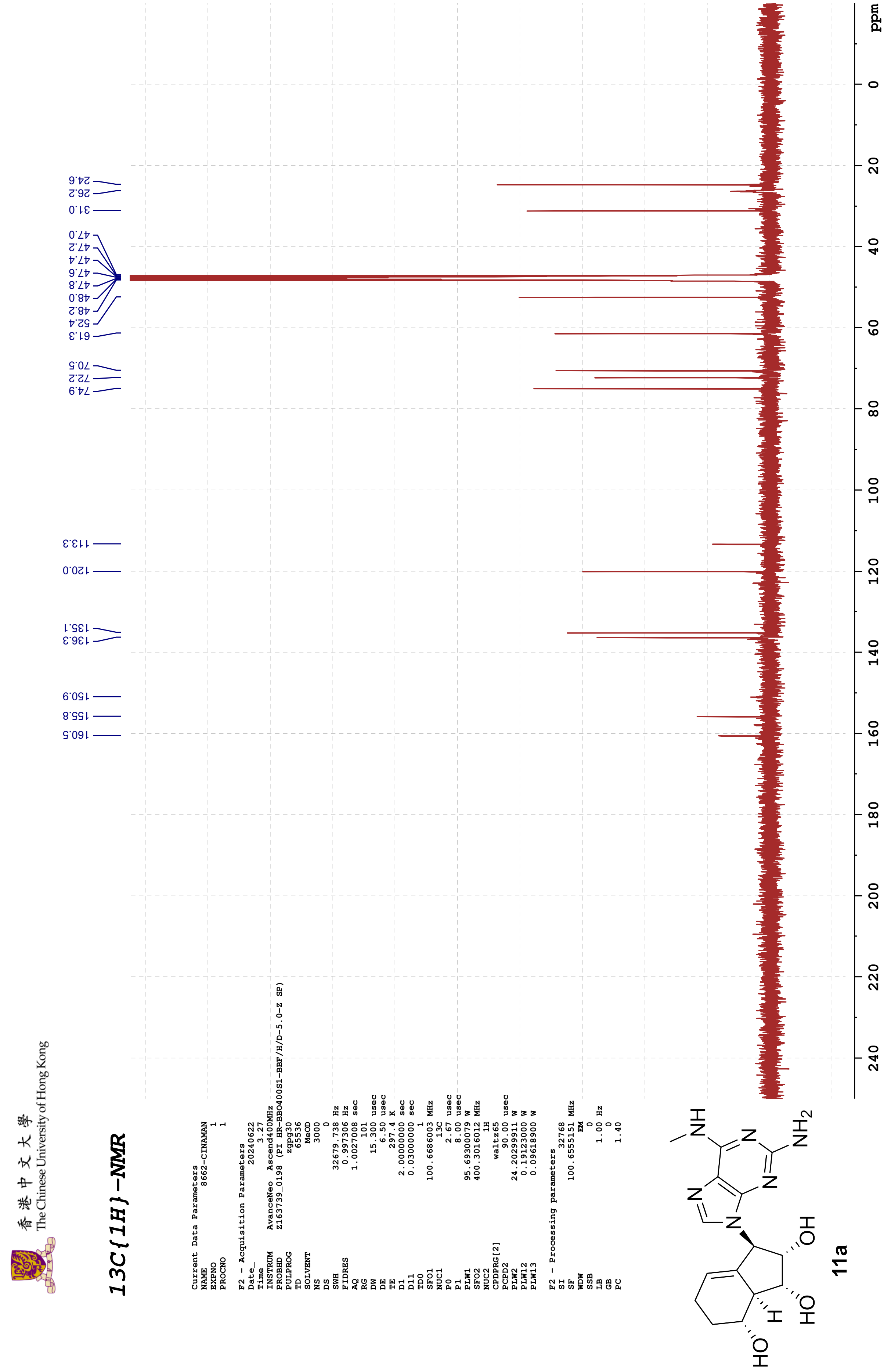

NMR-Spectra for Compound 12a

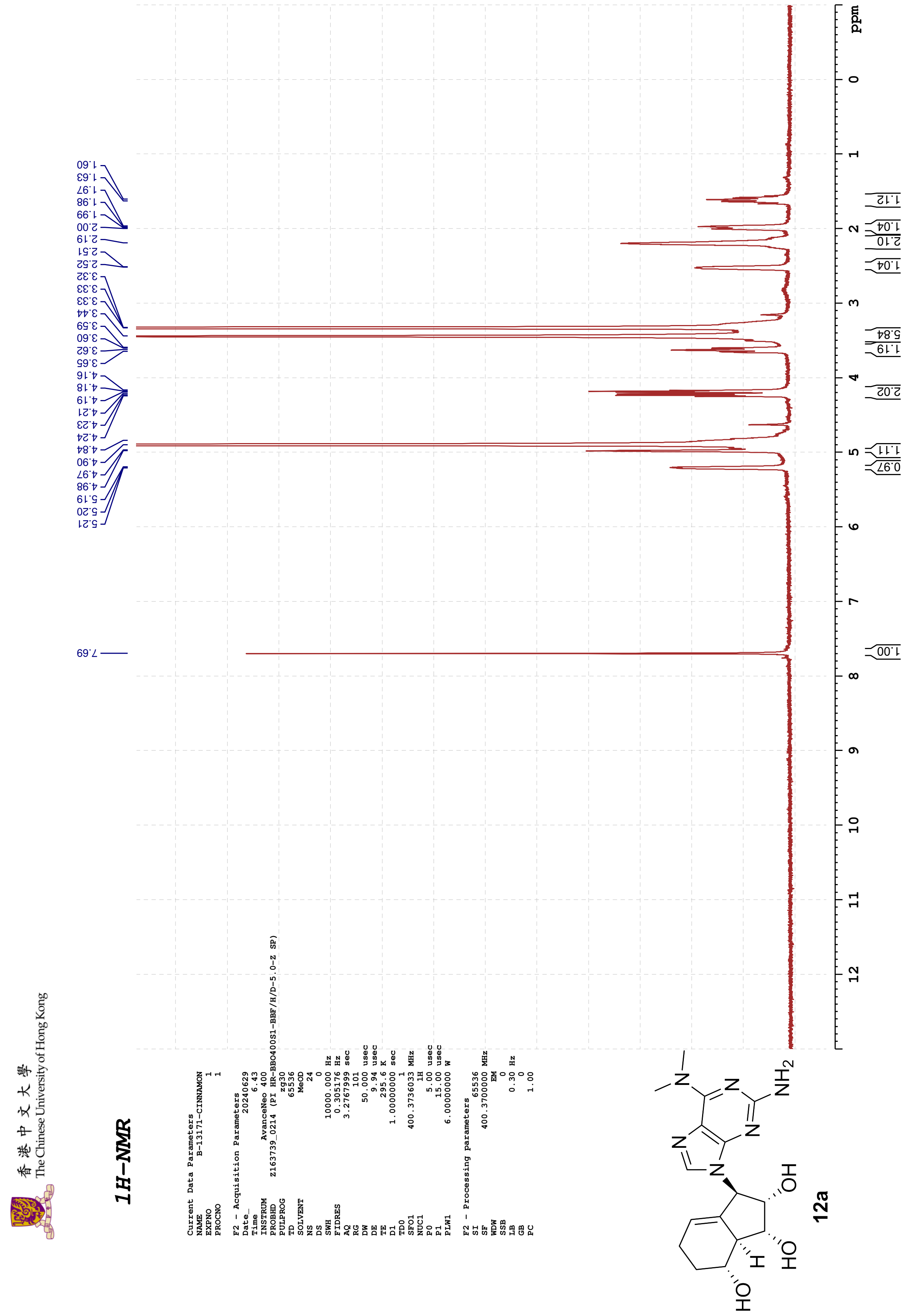

NMR-Spectra for Compound 12a

The Chinese University of Hong Kong 香港中文大學

13C{1H}-NMR

```
Current Data Parameters
NAME                9240-CINNAMON
EXPNO                           1
PROCNO                          1

F2 - Acquisition Parameters
Date_                    20240703
Time                        23.51
INSTRUM     AvanceNeo  Ascend400MHz
PROBHD      Z163739_0198 (PI HR-BBO400S1-BBF/H/D-5.0-Z SP)
PULPROG                   zgpg30
TD                         65536
SOLVENT                     MeOD
NS                          3000
DS                             0
SWH                    32679.738 Hz
FIDRES                  0.997306 Hz
AQ                     1.0027008 sec
RG                           101
DW                        15.300 usec
DE                          6.50 usec
TE                         293.5 K
D1                    2.00000000 sec
D11                   0.03000000 sec
TD0                            1
SFO1                 100.6686003 MHz
NUC1                         13C
P0                          2.67 usec
P1                          8.00 usec
PLW1                 95.69300079 W
SFO2                 400.3016012 MHz
NUC2                          1H
CPDPRG[2]                waltz65
PCPD2                      90.00 usec
PLW2                 24.20299911 W
PLW12                 0.19123000 W
PLW13                 0.09618900 W

F2 - Processing parameters
SI                         32768
SF                   100.6555151 MHz
WDW                           EM
SSB                            0
LB                          1.00 Hz
GB                             0
PC                          1.40
```

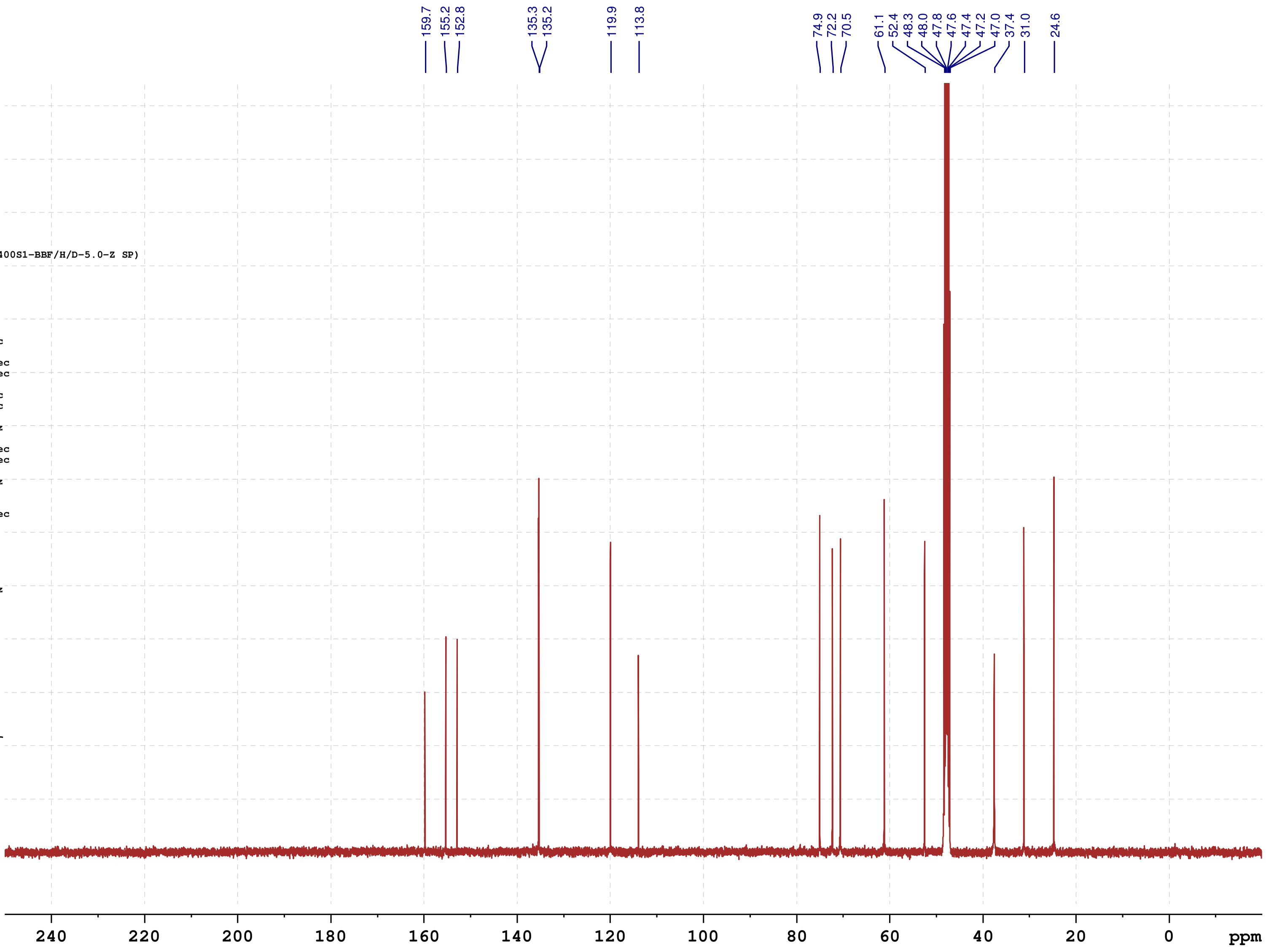

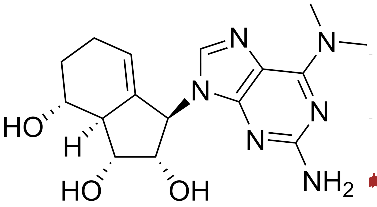

12a

NMR-Spectra for Compound 13a

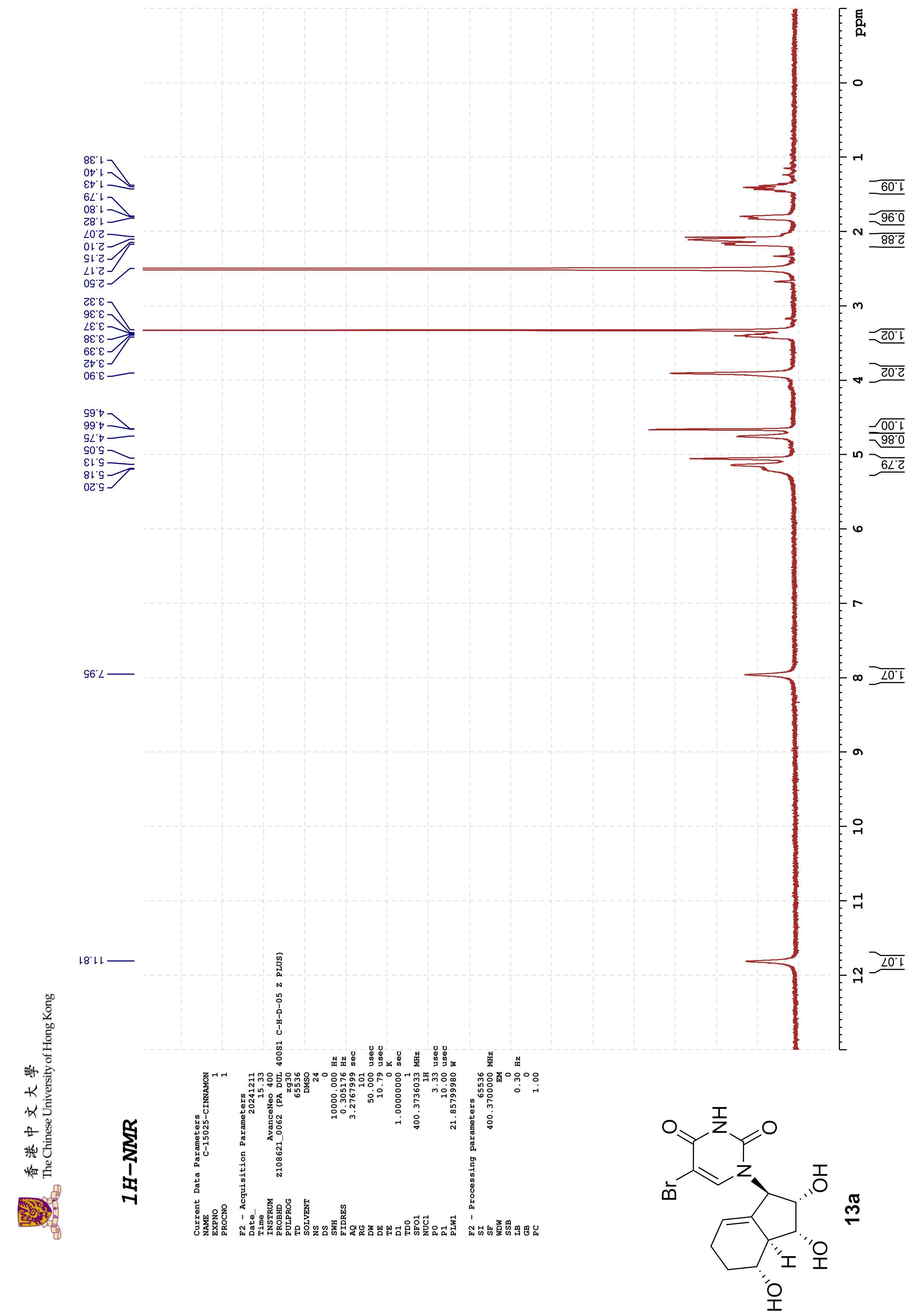

NMR-Spectra for Compound 14a

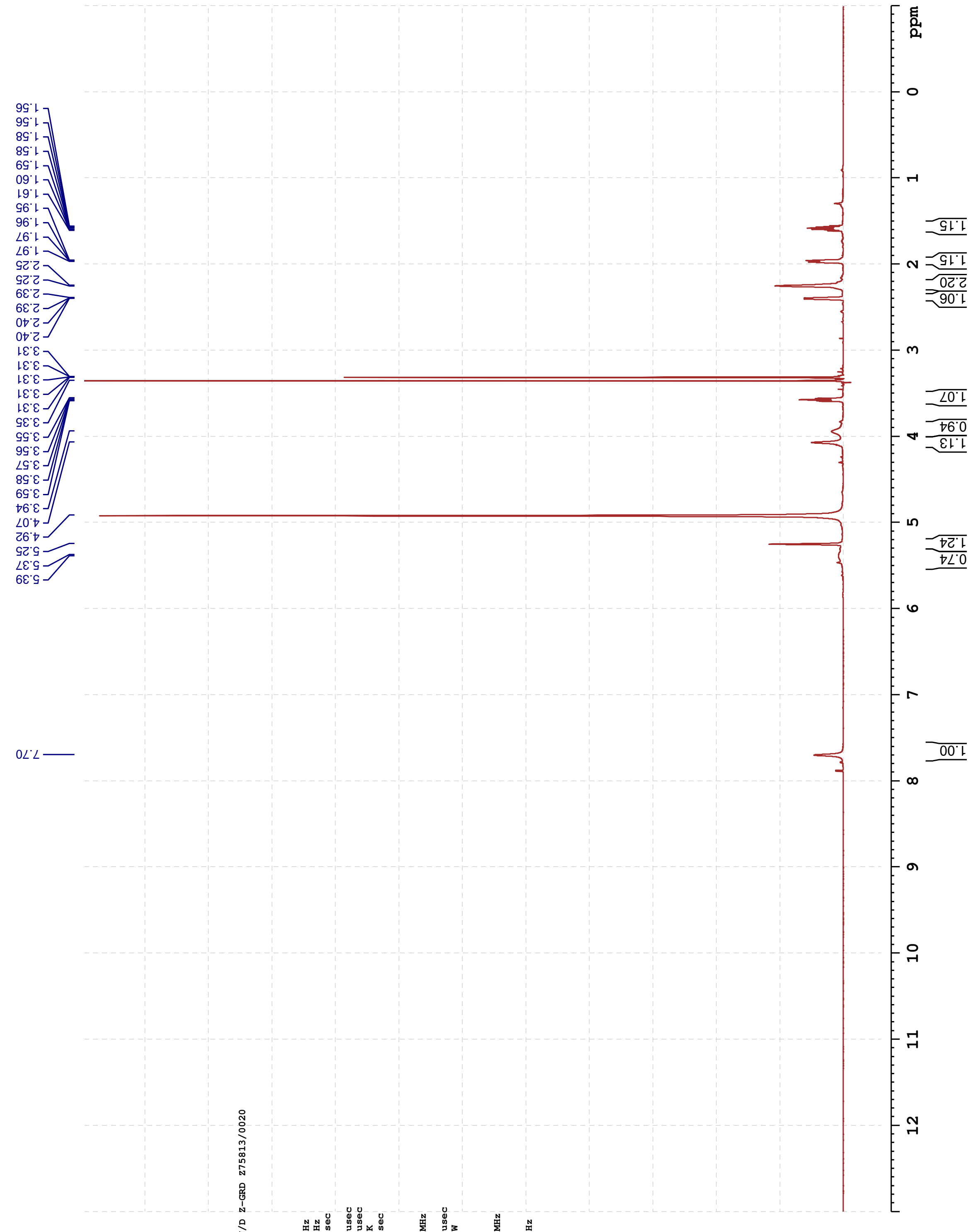

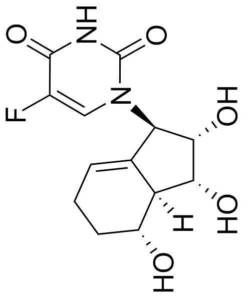

14a

NMR-Spectra for Compound 14a

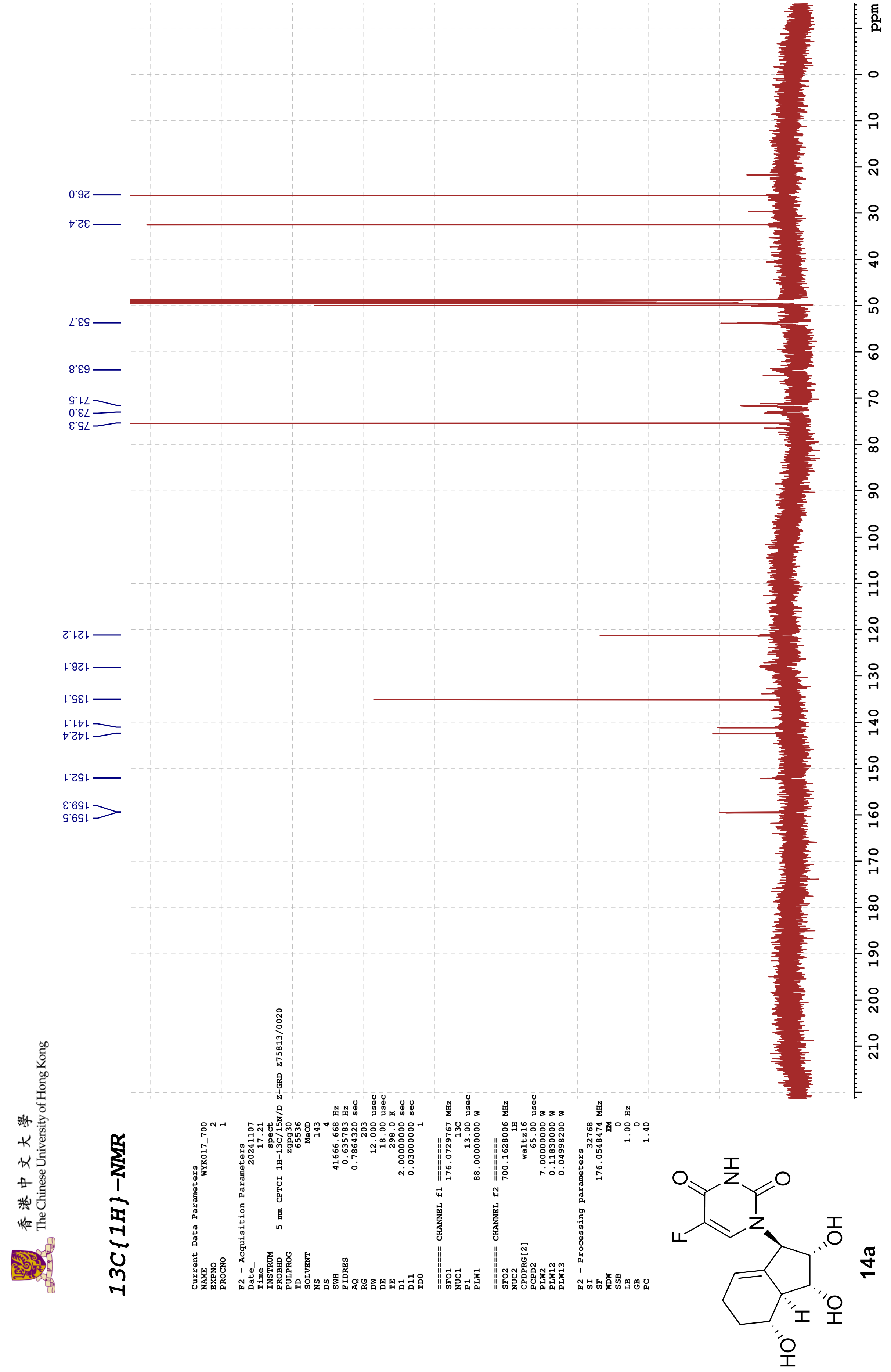

NMR-Spectra for Compound 14a

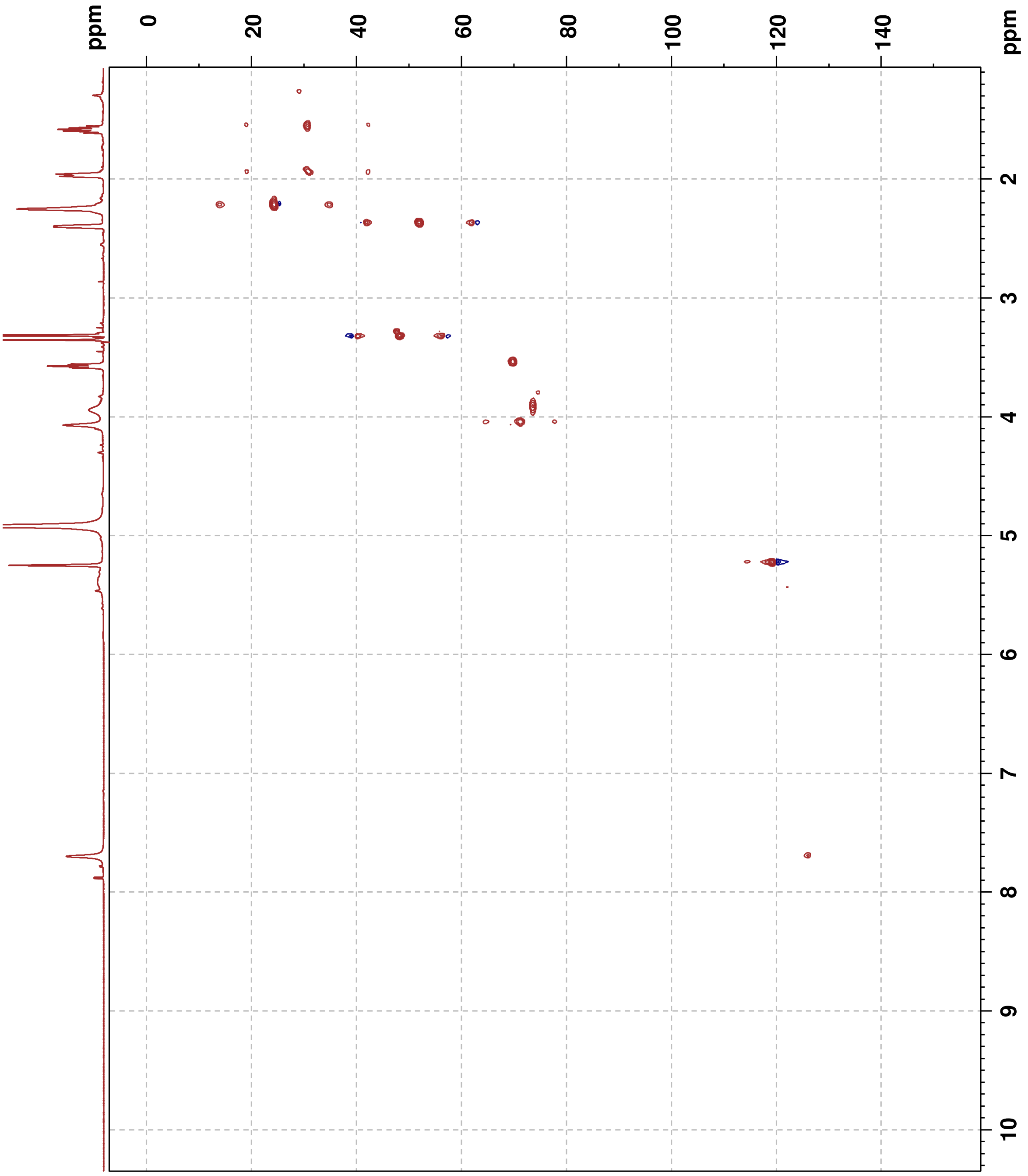

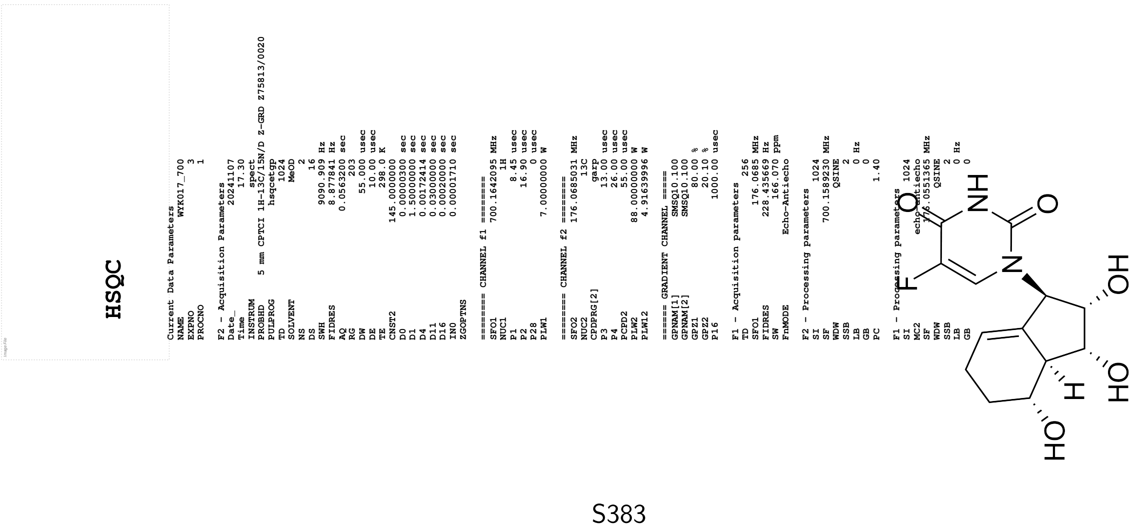

14a

NMR-Spectra for Compound 14a

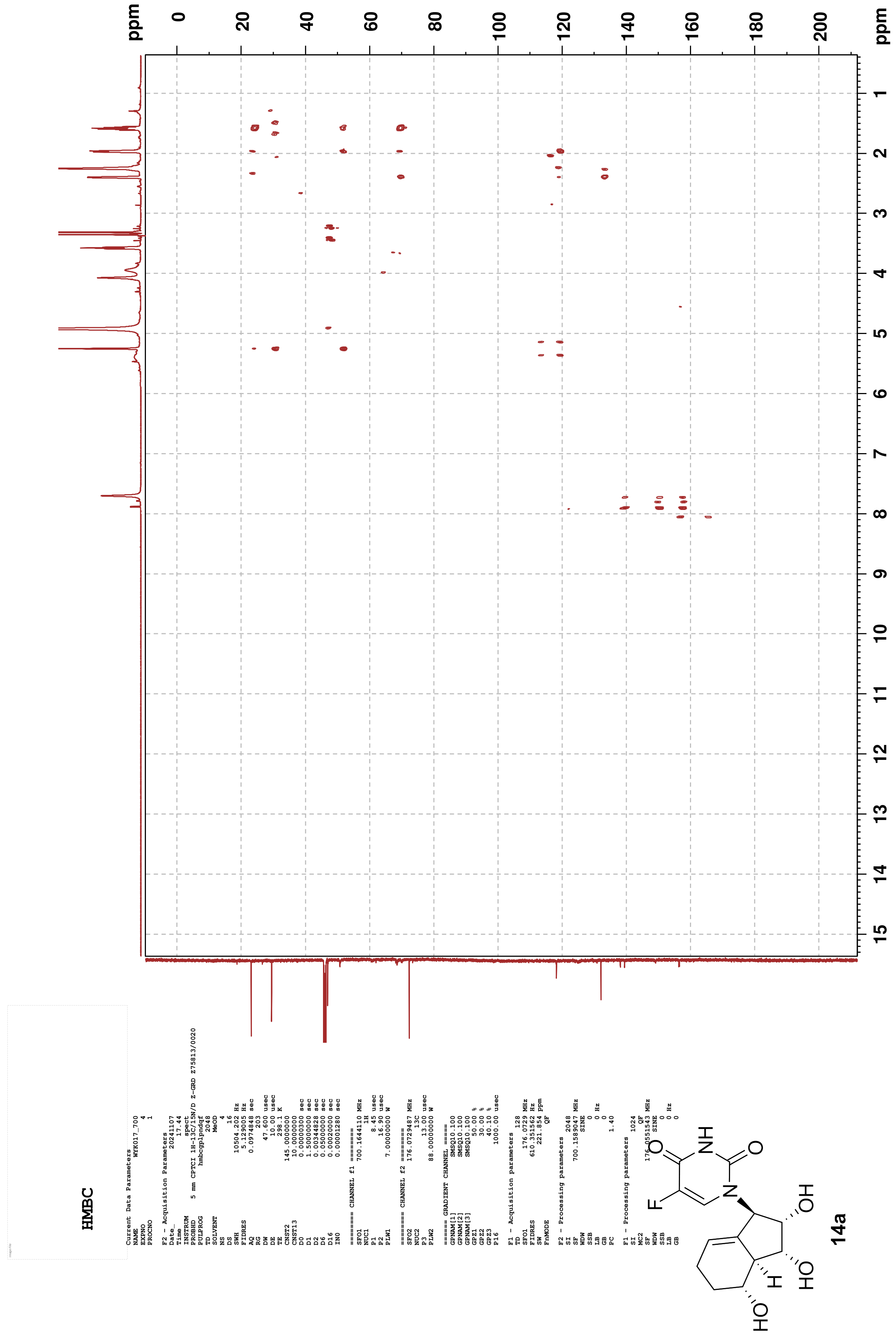

NMR-Spectra for Compound 15a

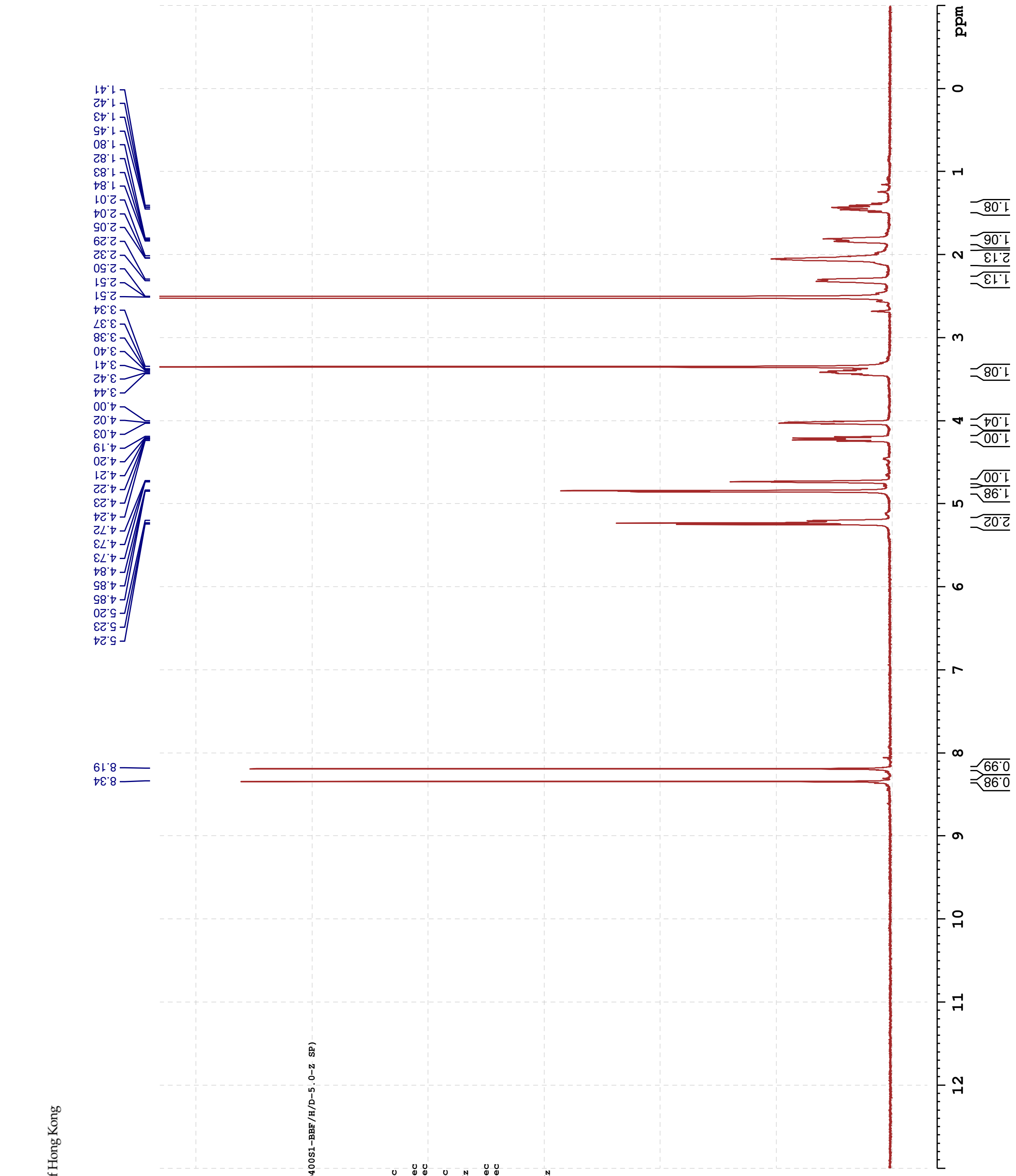

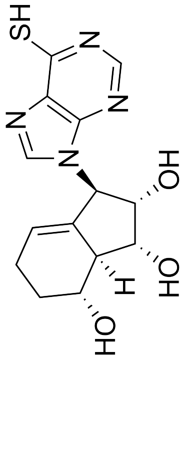

15a

NMR-Spectra for Compound 15a

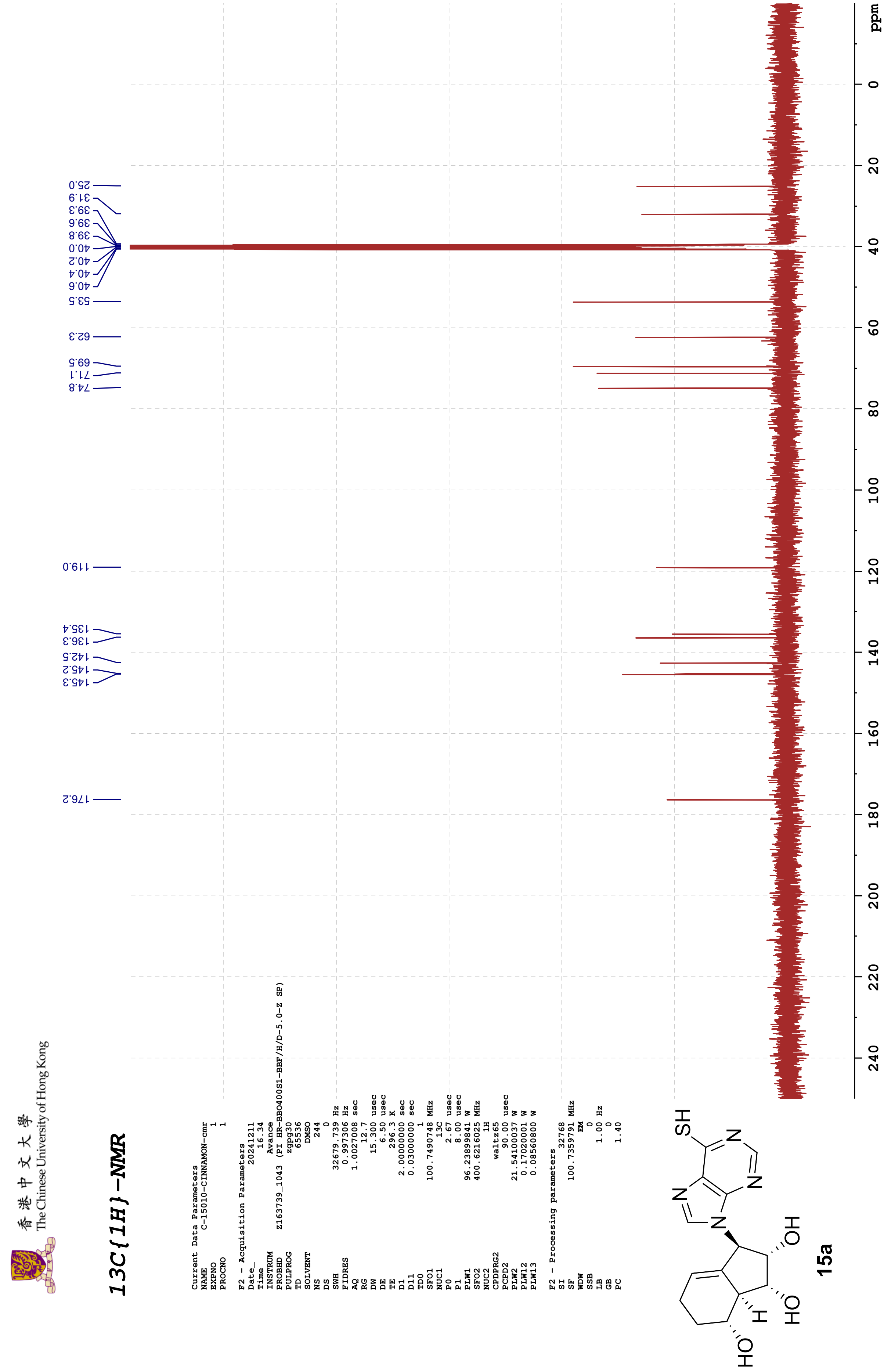

15a

NMR-Spectra for Compound 15b

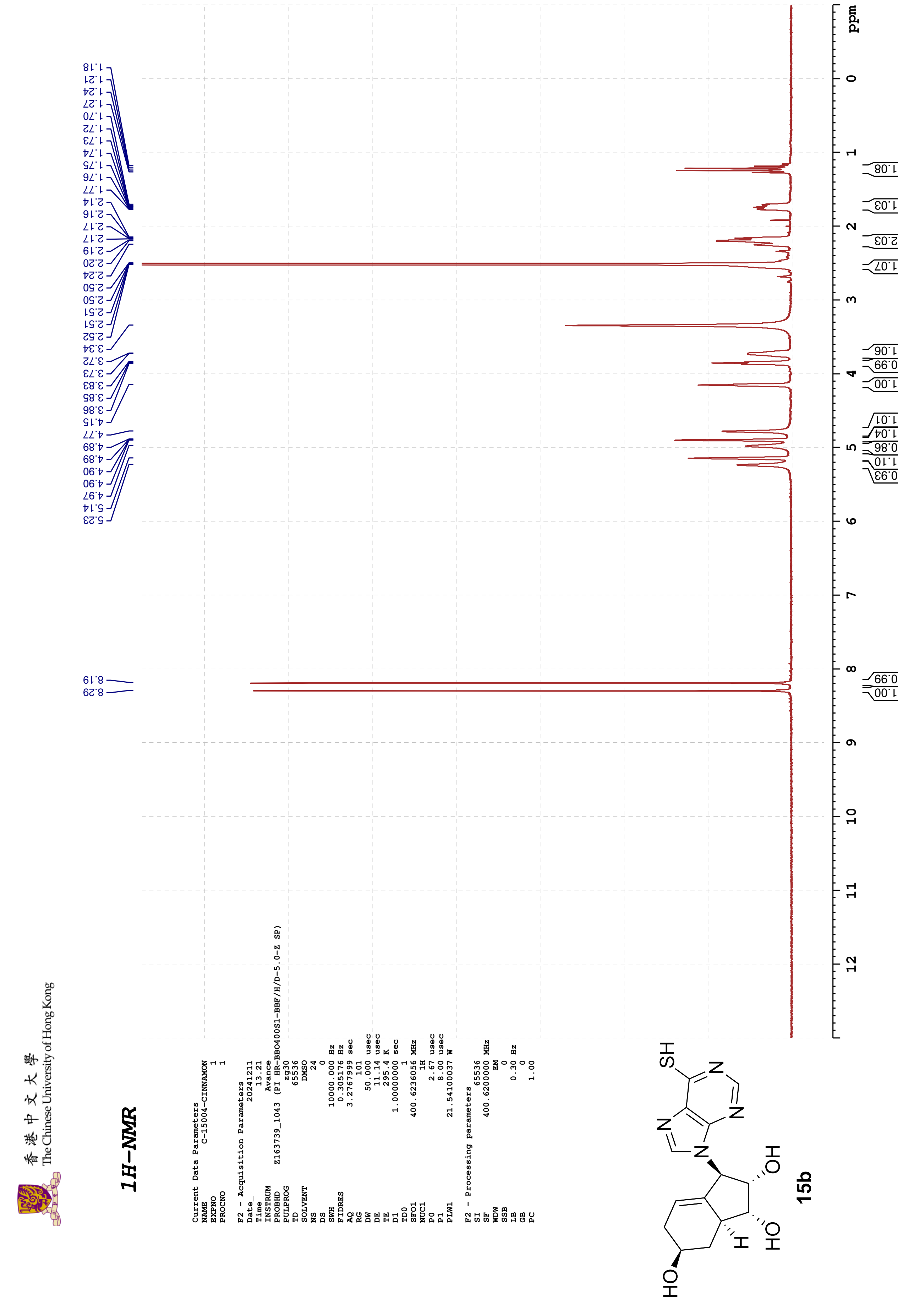

NMR-Spectra for Compound 15b

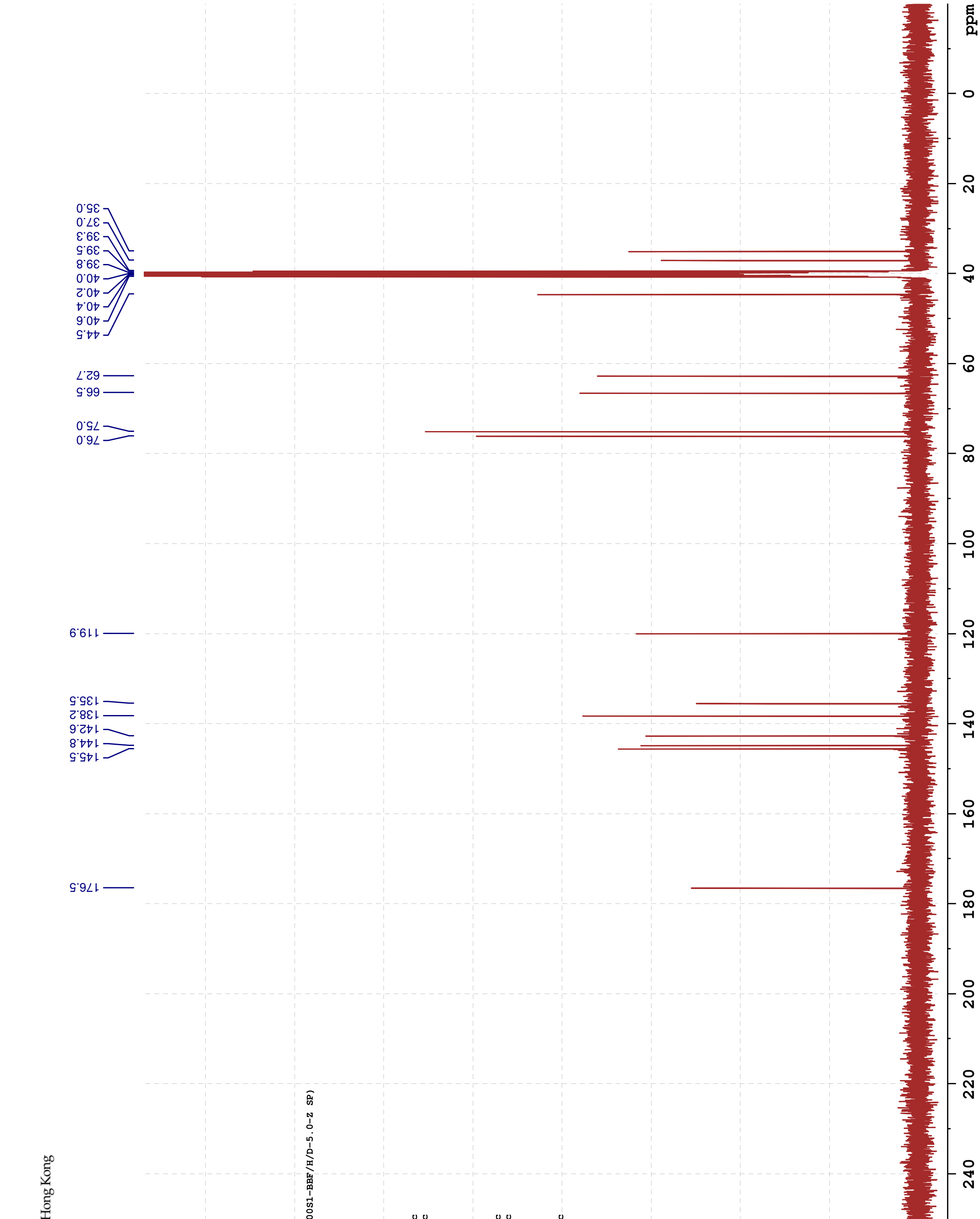

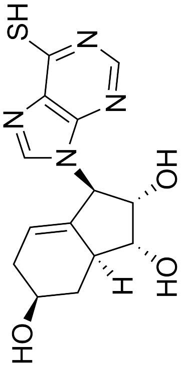

15b

NMR-Spectra for Compound 15c

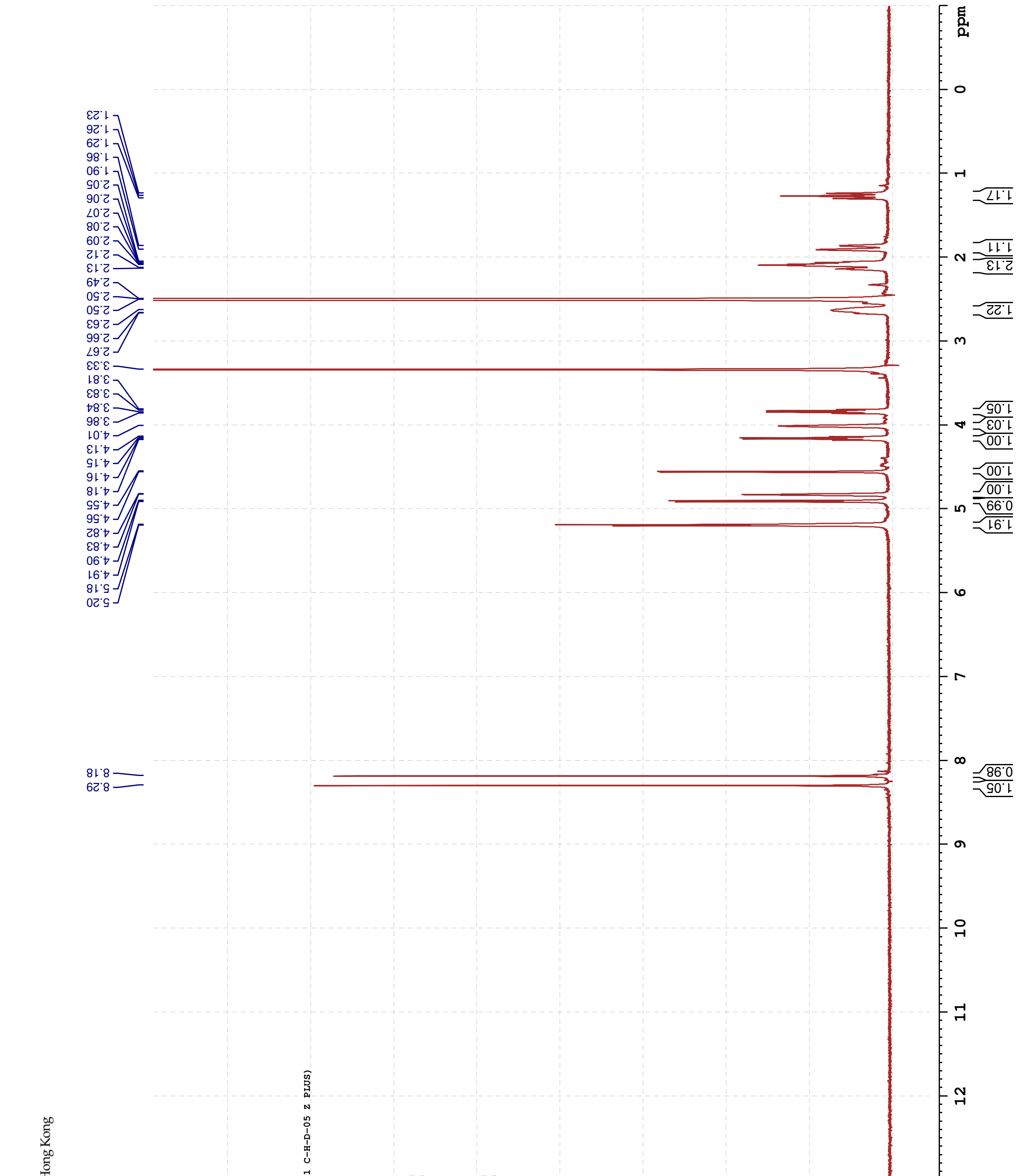

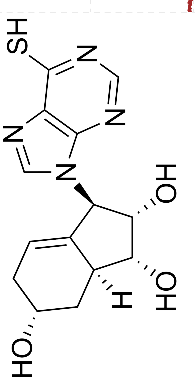

15c

NMR-Spectra for Compound 15c

香港中文大學 The Chinese University of Hong Kong

13C{1H}-NMR

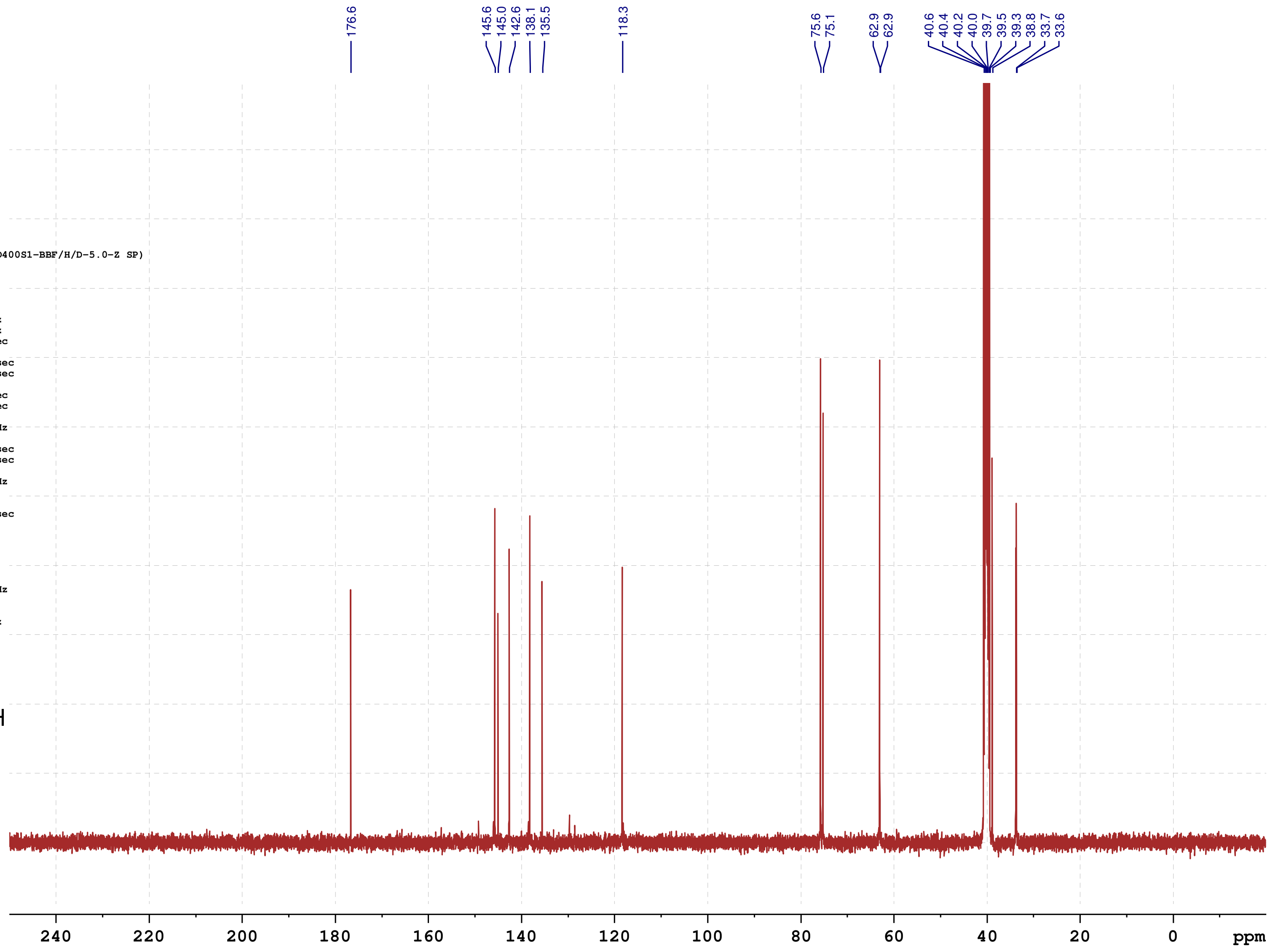


```
Current Data Parameters
NAME       C-17645-CINNAMON-cmr
EXPNO                     1
PROCNO                    1

F2 - Acquisition Parameters
Date_              20241230
Time                   3.54
INSTRUM              Avance
PROBHD     Z163739_1043 (PI HR-BBO400S1-BBF/H/D-5.0-Z SP)
PULPROG             zgpg30
TD                    65536
SOLVENT                DMSO
NS                     1024
DS                        0
SWH               32679.739 Hz
FIDRES             0.997306 Hz
AQ                1.0027008 sec
RG                     12.7
DW                   15.300 usec
DE                     6.50 usec
TE                    294.8 K
D1               2.00000000 sec
D11              0.03000000 sec
TD0                       1
SFO1            100.7490748 MHz
NUC1                    13C
P0                     2.67 usec
P1                     8.00 usec
PLW1            96.23899841 W
SFO2            400.6216025 MHz
NUC2                     1H
CPDPRG2             waltz65
PCPD2                 90.00 usec
PLW2            21.54100037 W
PLW12            0.17020001 W
PLW13            0.08560800 W

F2 - Processing parameters
SI                    32768
SF              100.7359791 MHz
WDW                      EM
SSB                       0
LB                     1.00 Hz
GB                        0
PC                     1.40
```

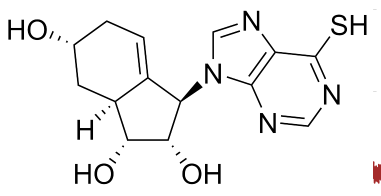

15c

NMR-Spectra for Compound 16a

香港中文大學
The Chinese University of Hong Kong

1H-NMR

```
Current Data Parameters
NAME              SCC726_700
EXPNO                      1
PROCNO                     1

F2 - Acquisition Parameters
Date_               20241017
Time                   11.46
INSTRUM                spect
PROBHD      5 mm CPTCI 1H-13C/15N/D Z-GRD Z75813/0020
PULPROG                 zg30
TD                     65536
SOLVENT                 MeOD
NS                        16
DS                         2
SWH              14097.744 Hz
FIDRES            0.215115 Hz
AQ               2.3243434 sec
RG                      12.7
DW                    35.467 usec
DE                     10.00 usec
TE                     298.0 K
D1                1.00000000 sec
TD0                        1

======== CHANNEL f1 ========
SFO1           700.1643238 MHz
NUC1                    1H
P1                    8.45 usec
PLW1            7.00000000 W

F2 - Processing parameters
SI                     65536
SF             700.1588971 MHz
WDW                       EM
SSB                        0
LB                      0.30 Hz
GB                         0
PC                      1.00
```

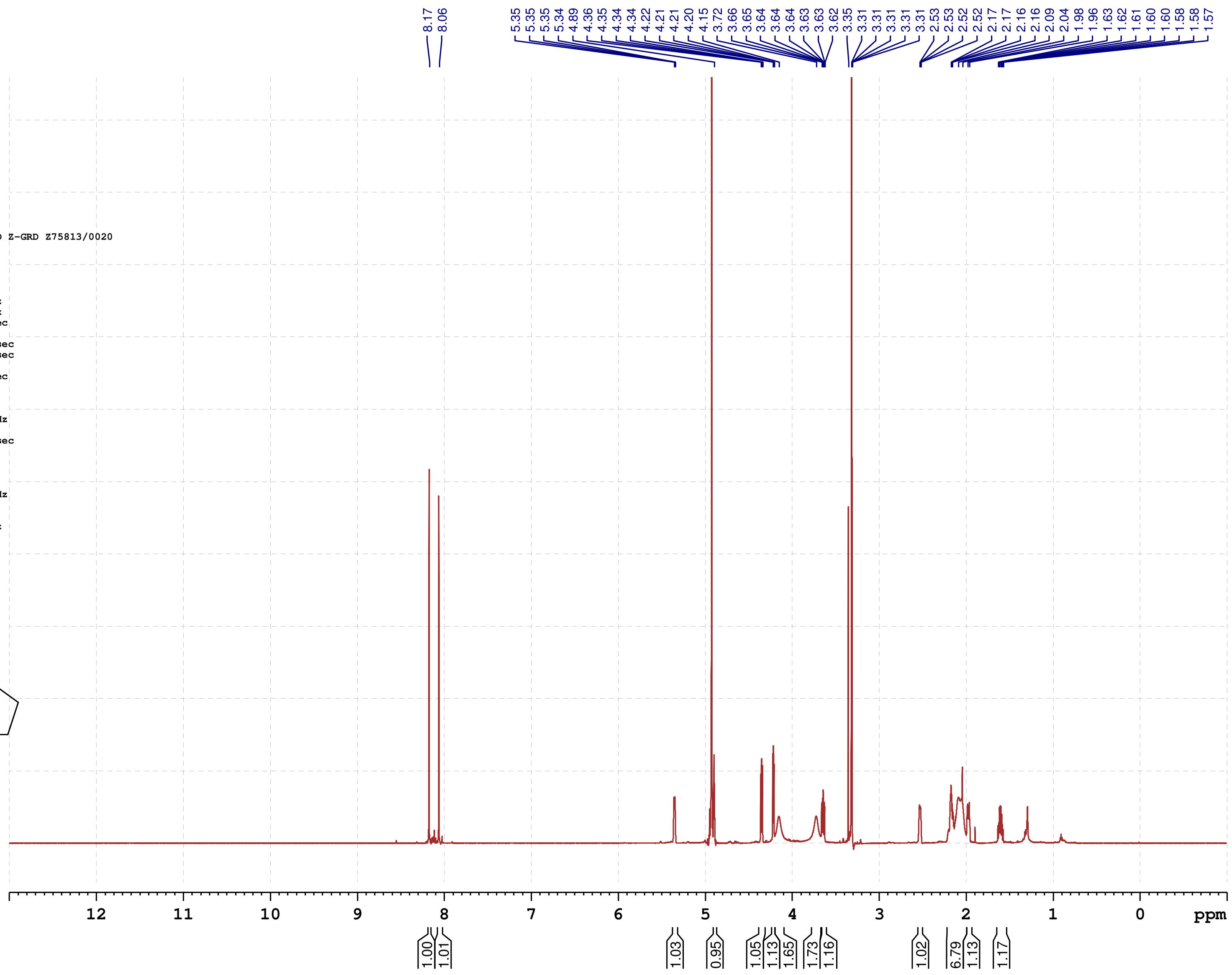

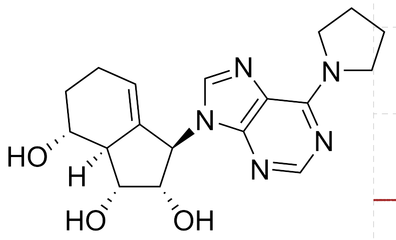

16a

NMR-Spectra for Compound 16a

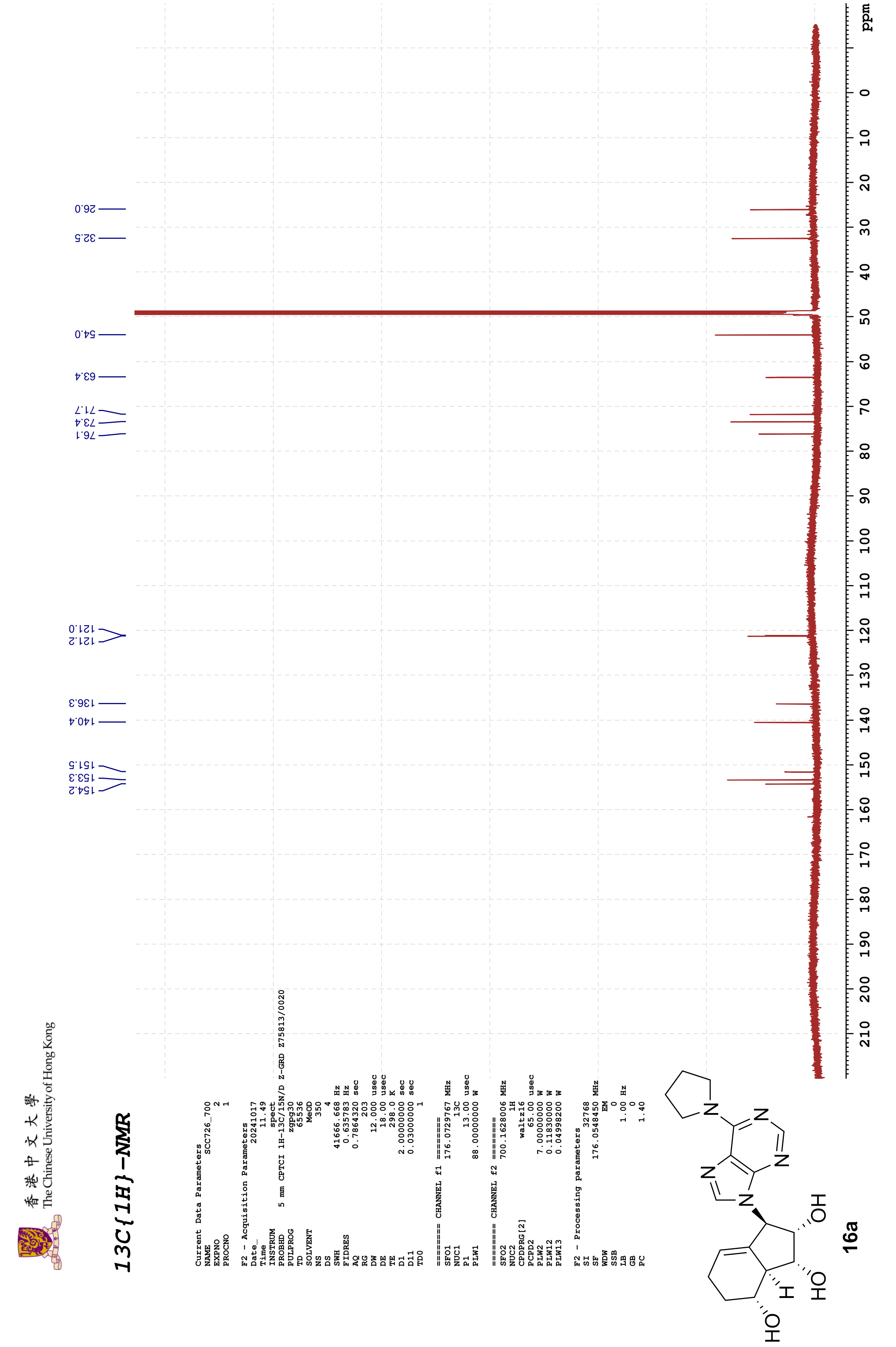

NMR-Spectra for Compound 17a

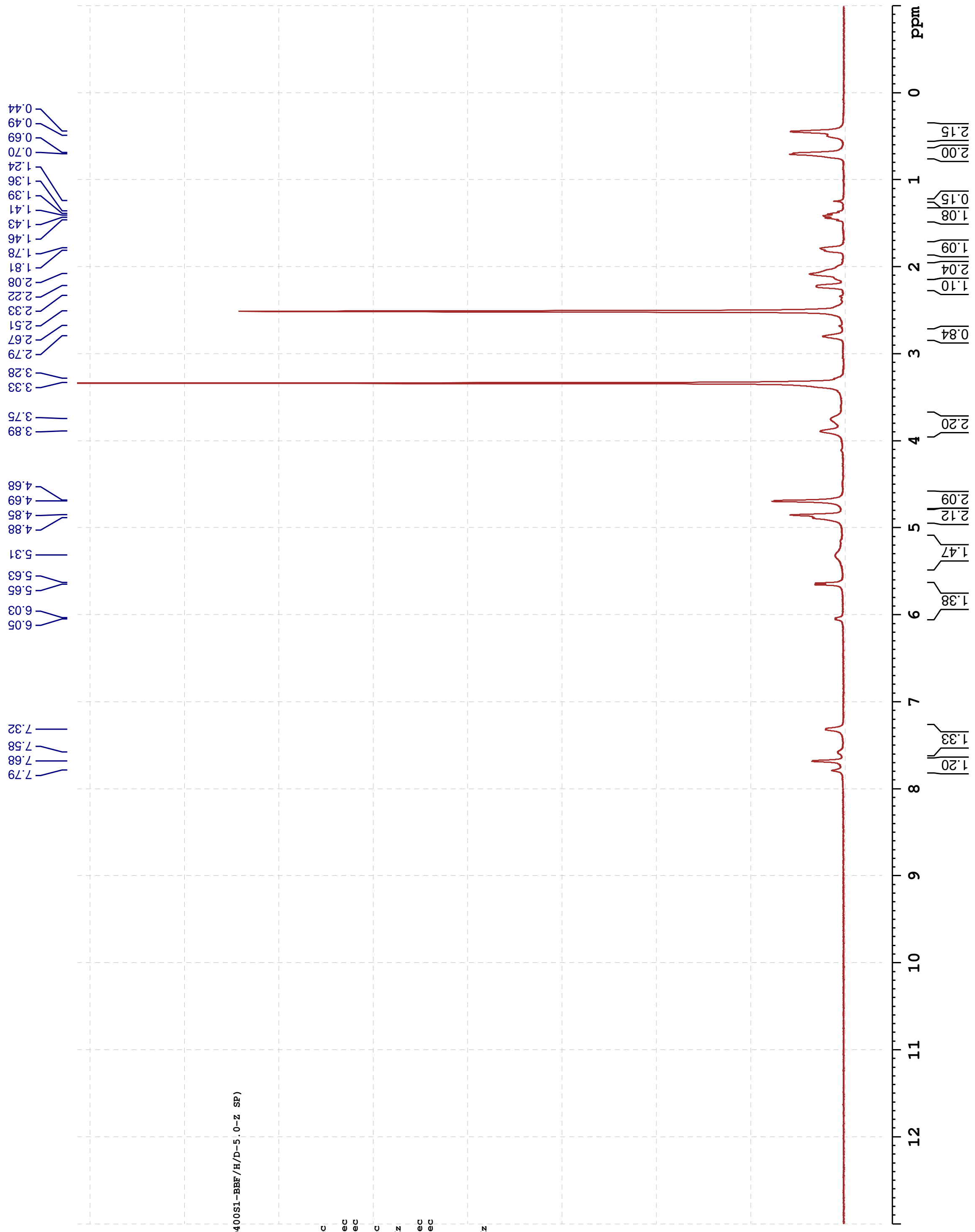

香港中文大學 The Chinese University of Hong Kong

1H-NMR

```
Current Data Parameters
NAME          B-11148-CINNAMON
EXPNO                    1
PROCNO                   1

F2 - Acquisition Parameters
Date_             20240604
Time                 14.50
INSTRUM        AvanceNeo 400
PROBHD     Z163739_0214 (PI HR-BBO400S1-BBF/H/D-5.0-Z SP)
PULPROG               zg30
TD                   65536
SOLVENT               DMSO
NS                      24
DS                       0
SWH              10000.000 Hz
FIDRES            0.305176 Hz
AQ               3.2767999 sec
RG                      32
DW                  50.000 usec
DE                    9.94 usec
TE                   296.8 K
D1              1.00000000 sec
TD0                      1
SFO1           400.3736033 MHz
NUC1                    1H
P0                    5.00 usec
P1                   15.00 usec
PLW1            6.00000000 W

F2 - Processing parameters
SI                   65536
SF             400.3700000 MHz
WDW                     EM
SSB                      0
LB                    0.30 Hz
GB                       0
PC                    1.00
```

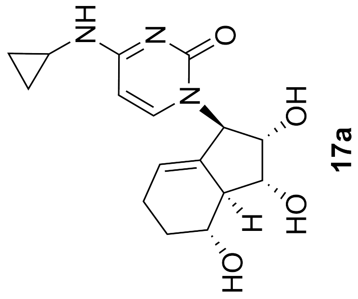

17a

NMR-Spectra for Compound 17a

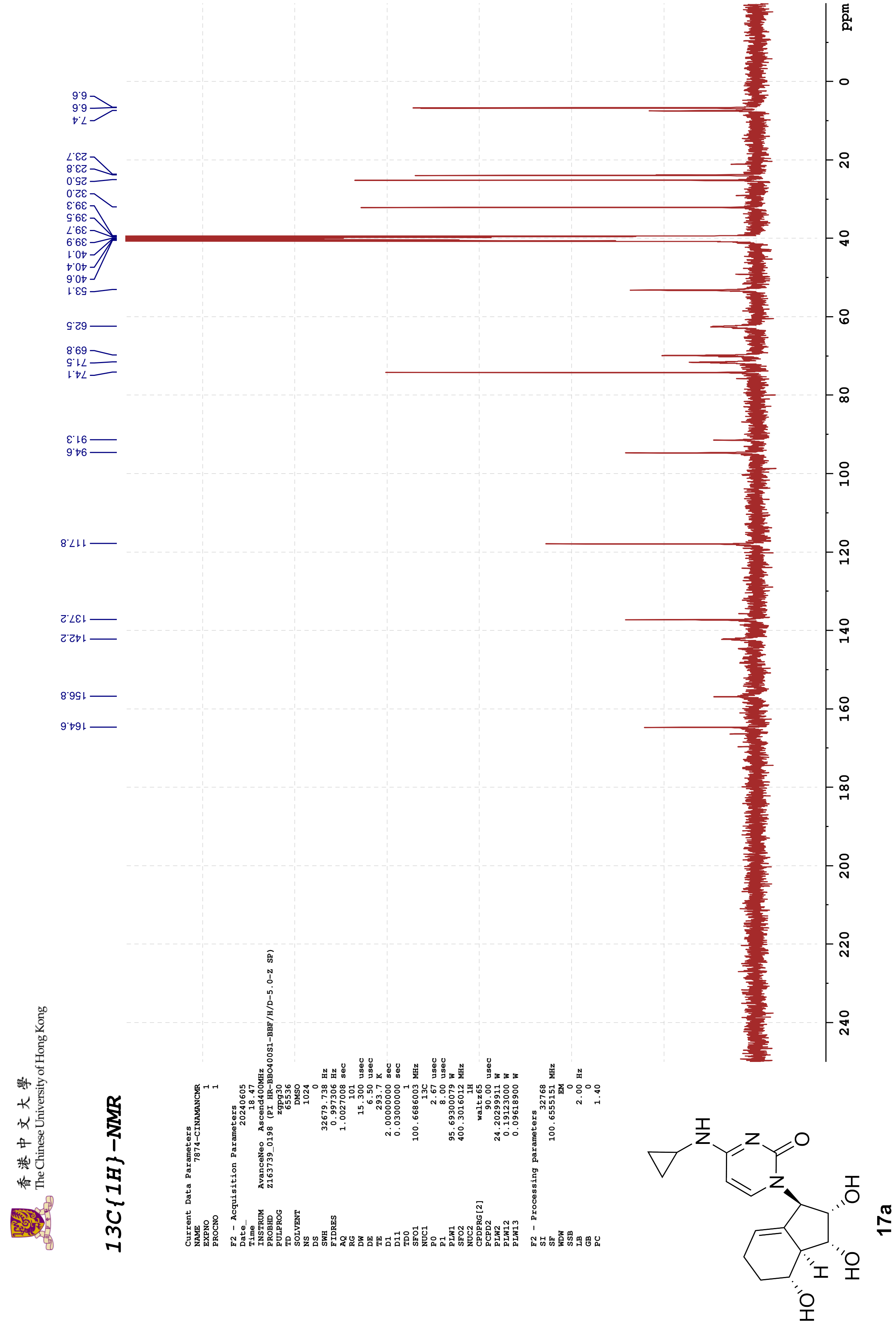

NMR-Spectra for Compound 17b

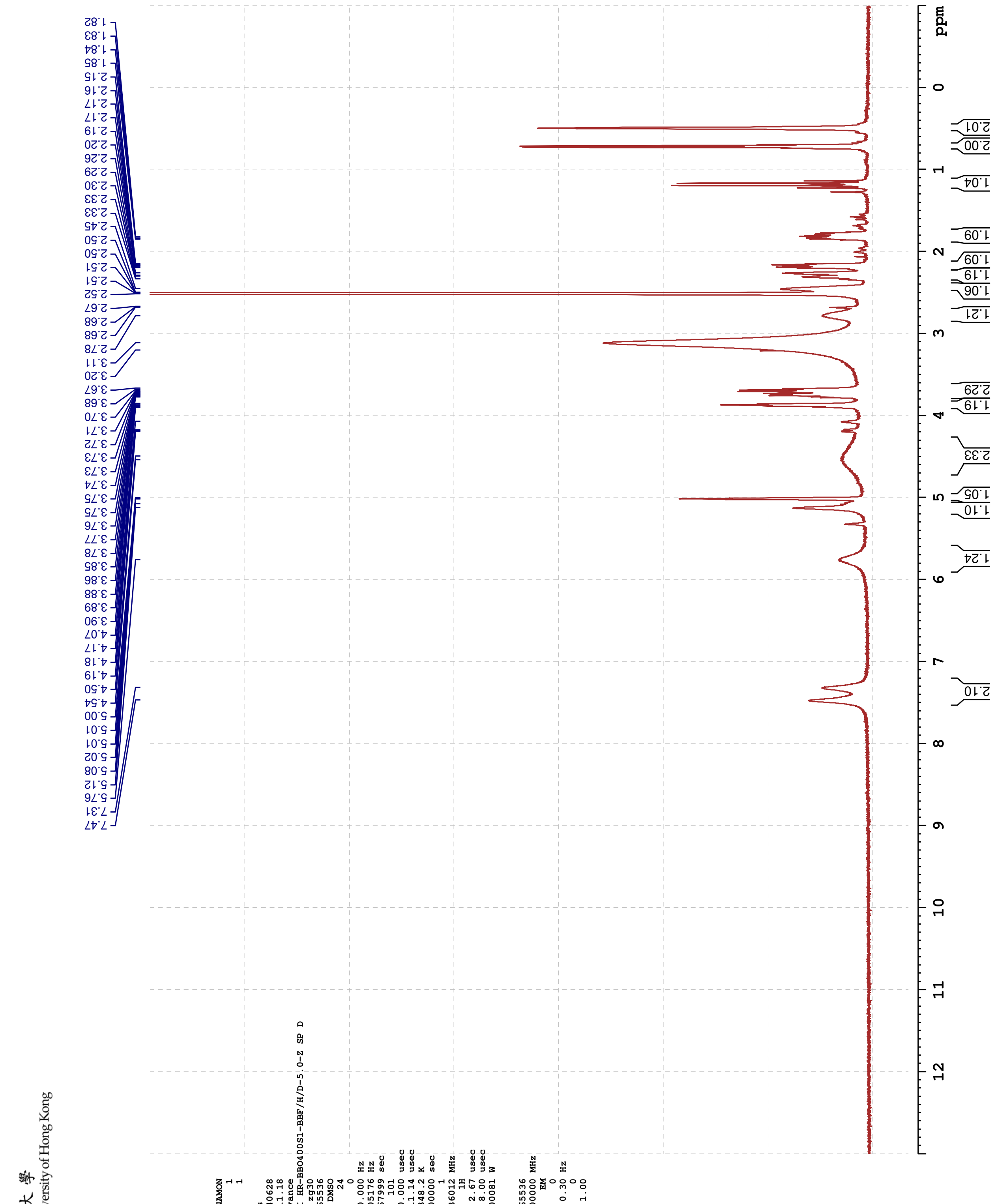

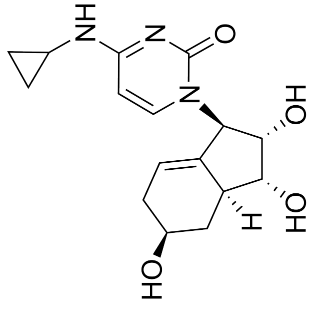

17b

NMR-Spectra for Compound 17b

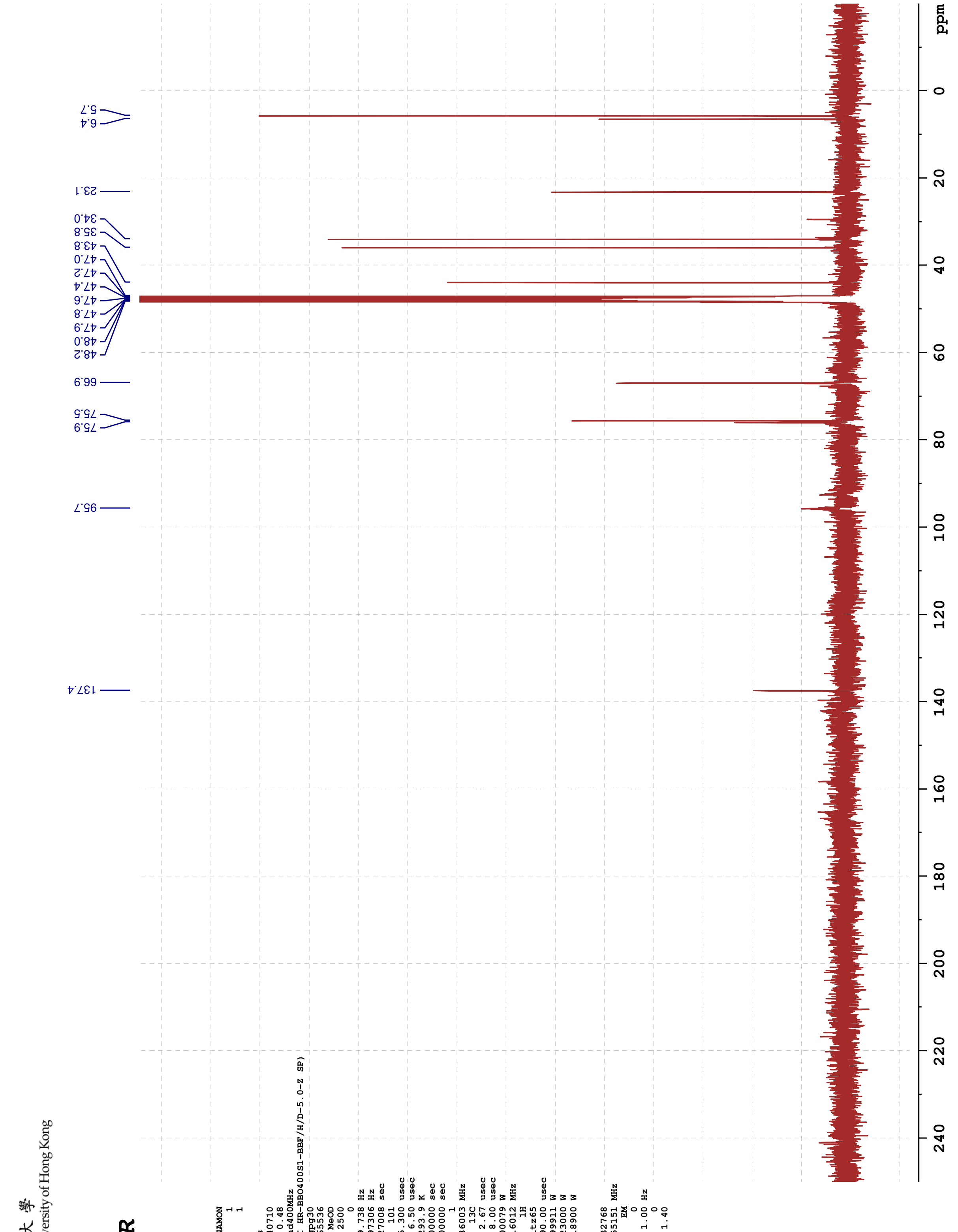

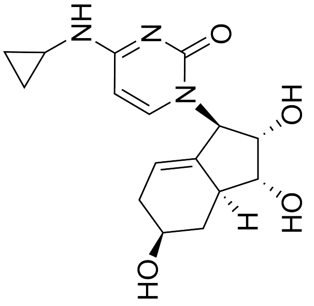

17b

NMR-Spectra for Compound 17c

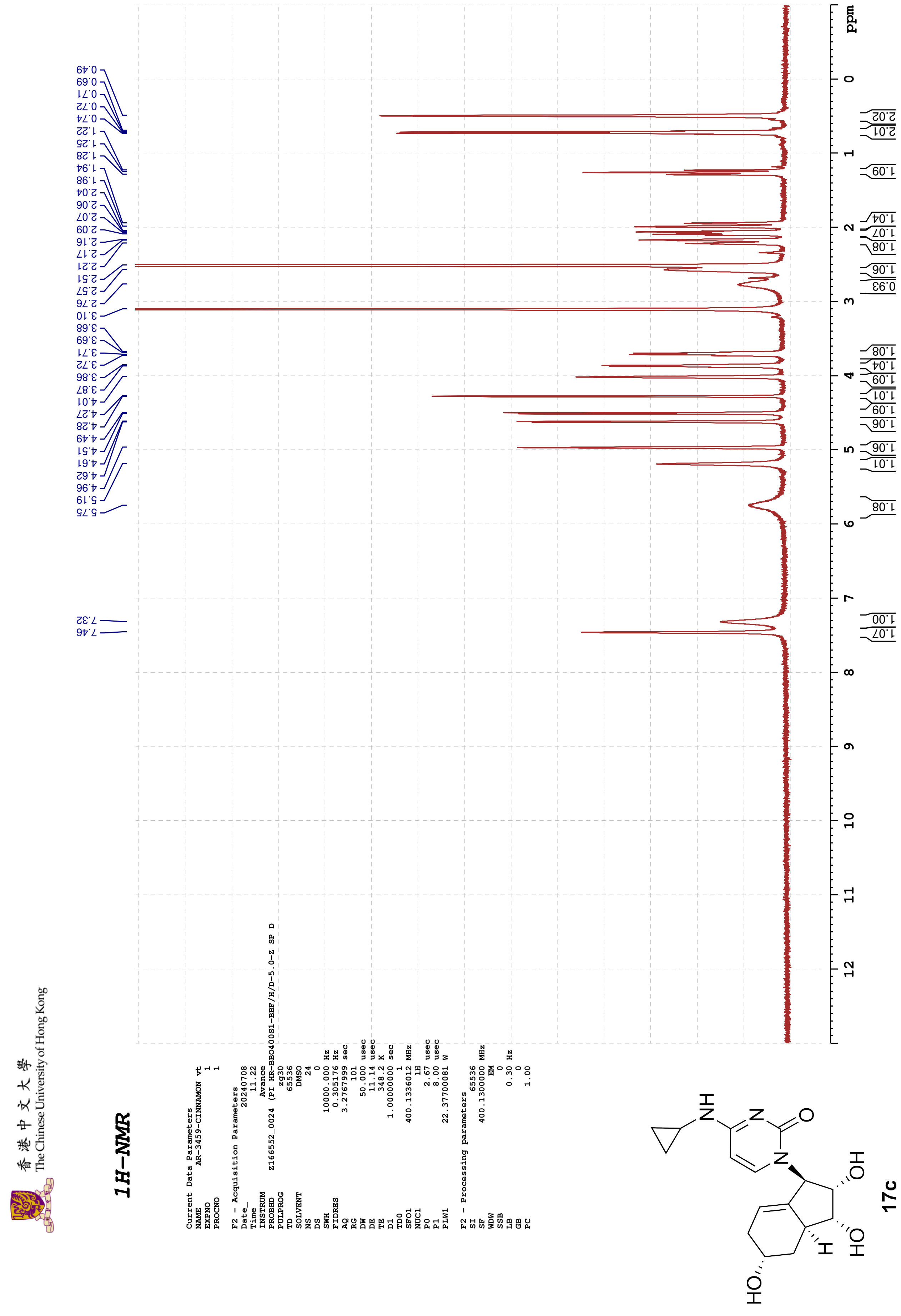

NMR-Spectra for Compound 17c

香港中文大學 The Chinese University of Hong Kong

13C{1H}-NMR

```
Current Data Parameters
NAME              9534-CINAMON
EXPNO                        1
PROCNO                       1

F2 - Acquisition Parameters
Date_                 20240709
Time                     22.35
INSTRUM    AvanceNeo  Ascend400MHz
PROBHD     Z163739_0198 (PI HR-BBO400S1-BBF/H/D-5.0-Z SP)
PULPROG                zgpg30
TD                       65536
SOLVENT                   MeOD
NS                        2500
DS                           0
SWH                  32679.738 Hz
FIDRES                0.997306 Hz
AQ                   1.0027008 sec
RG                         101
DW                      15.300 usec
DE                        6.50 usec
TE                       294.1 K
D1                  2.00000000 sec
D11                 0.03000000 sec
TD0                          1
SFO1               100.6686003 MHz
NUC1                       13C
P0                        2.67 usec
P1                        8.00 usec
PLW1               95.69300079 W
SFO2               400.3016012 MHz
NUC2                        1H
CPDPRG[2]              waltz65
PCPD2                    90.00 usec
PLW2               24.20299911 W
PLW12               0.19123000 W
PLW13               0.09618900 W

F2 - Processing parameters
SI                       32768
SF                 100.6555151 MHz
WDW                         EM
SSB                          0
LB                        1.00 Hz
GB                           0
PC                        1.40
```

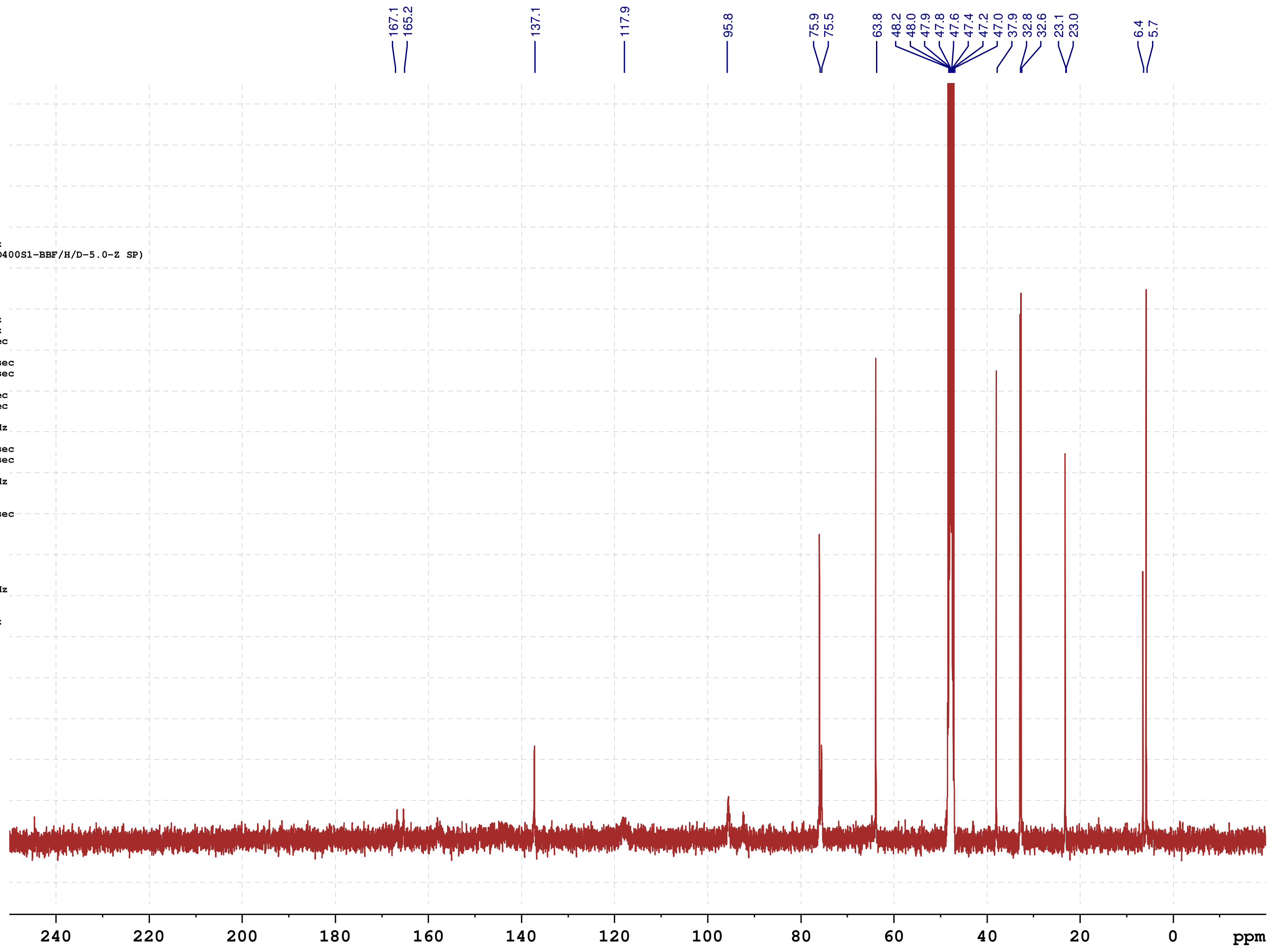

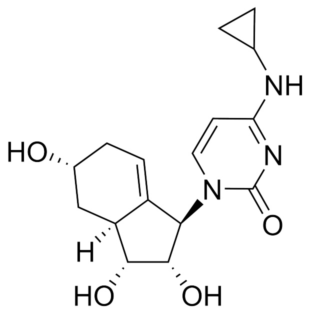

17c

NMR-Spectra for Compound 18d

香港中文大學 The Chinese University of Hong Kong

1H–NMR

```
Current Data Parameters
NAME          C-15573-CINNAMON
EXPNO                        1
PROCNO                       1

F2 - Acquisition Parameters
Date_                 20241214
Time                     16.10
INSTRUM                 Avance
PROBHD      Z163739_1043 (PI HR-BBO400S1-BBF/H/D-5.0-Z SP)
PULPROG                   zg30
TD                       65536
SOLVENT                   DMSO
NS                          24
DS                           0
SWH                  10000.000 Hz
FIDRES                0.305176 Hz
AQ                   3.2767999 sec
RG                         101
DW                      50.000 usec
DE                       11.14 usec
TE                       295.6 K
D1                  1.00000000 sec
TD0                          1
SFO1               400.6236056 MHz
NUC1                        1H
P0                        2.67 usec
P1                        8.00 usec
PLW1               21.54100037 W

F2 - Processing parameters
SI                       65536
SF                 400.6200000 MHz
WDW                         EM
SSB                          0
LB                        0.30 Hz
GB                           0
PC                        1.00
```

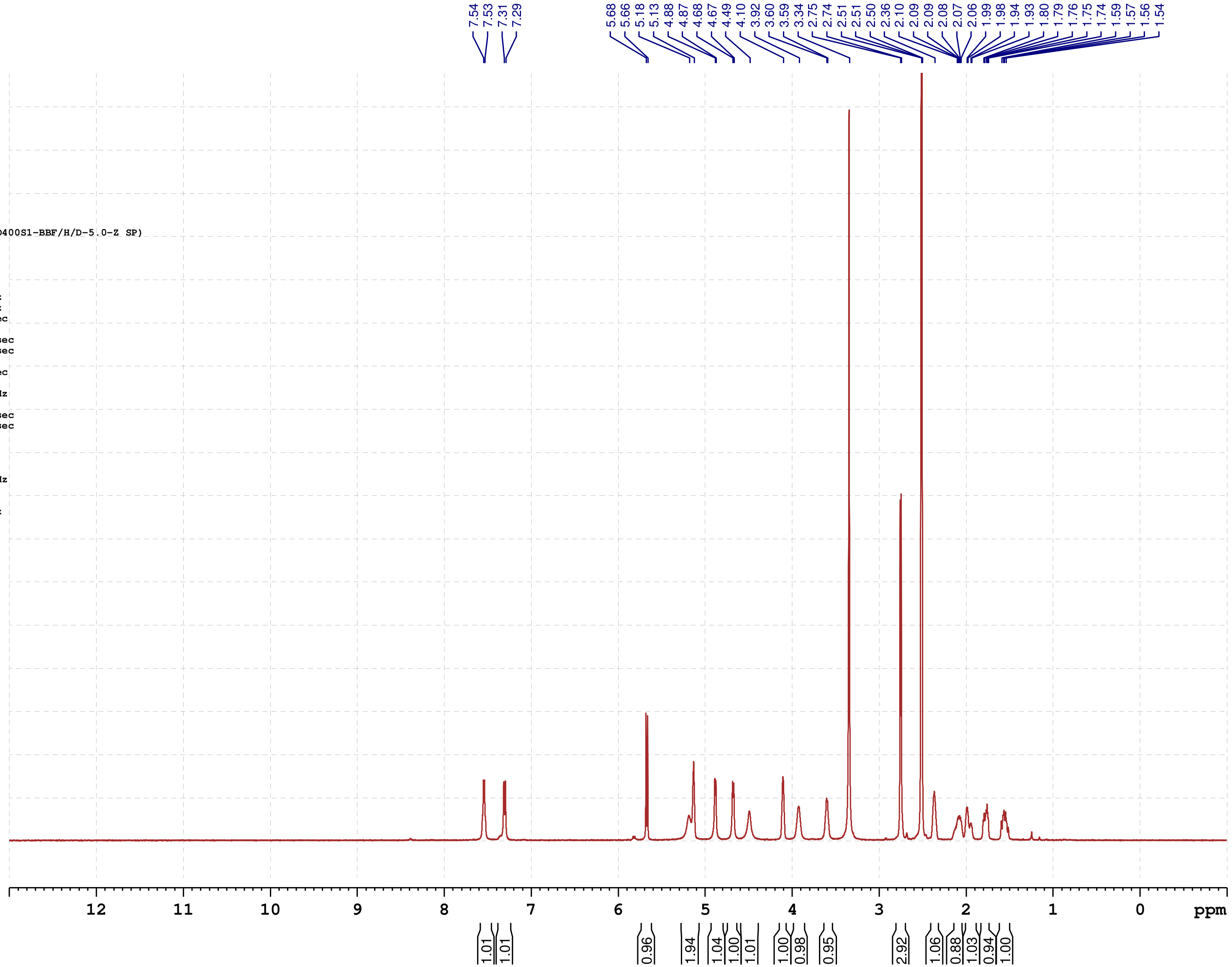

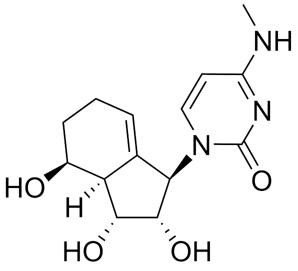

18d

NMR-Spectra for Compound 18d

The Chinese University of Hong Kong

13C{1H}-NMR

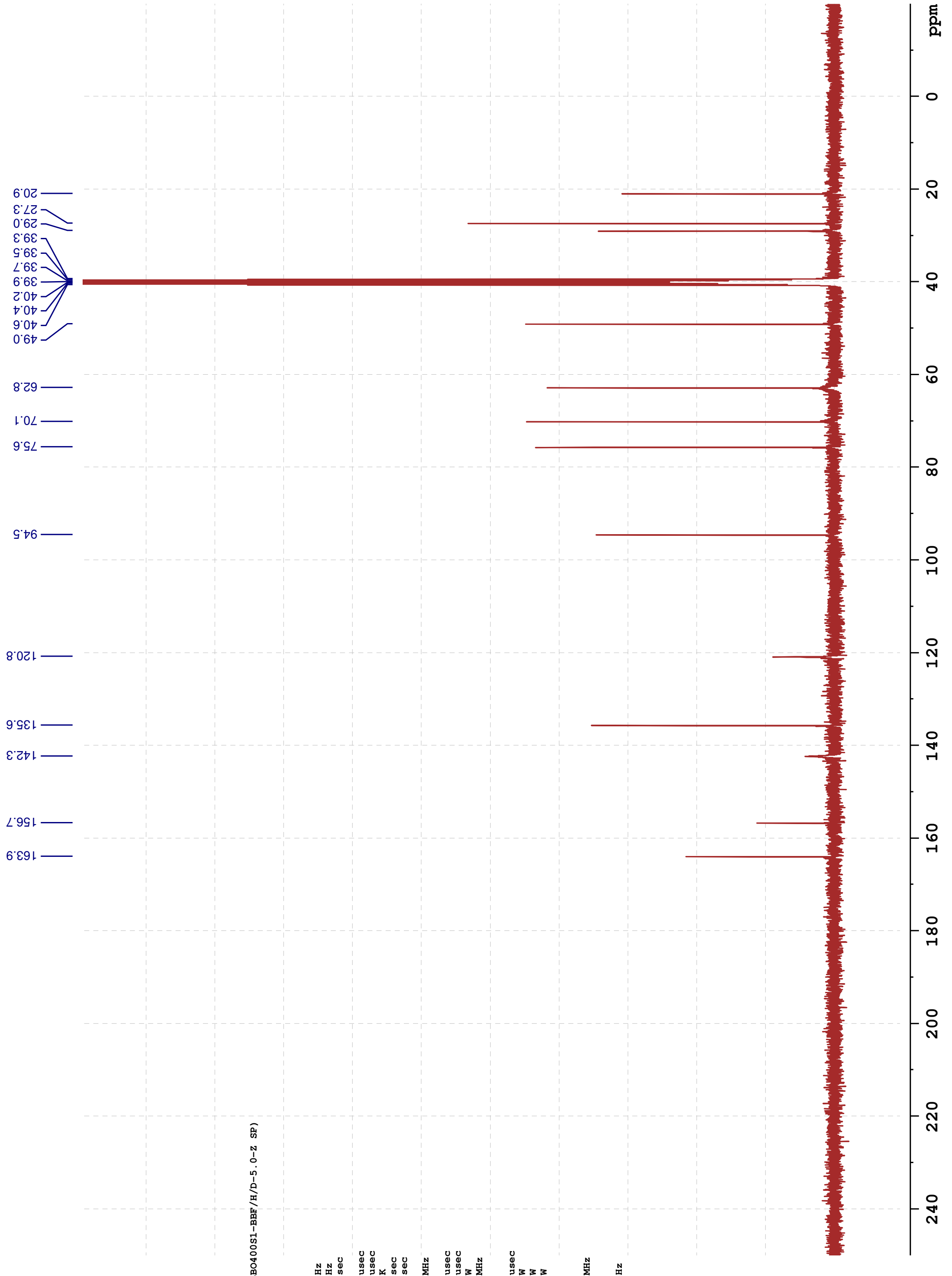

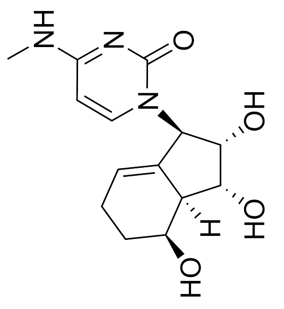

18d

NMR-Spectra for Compound 18e

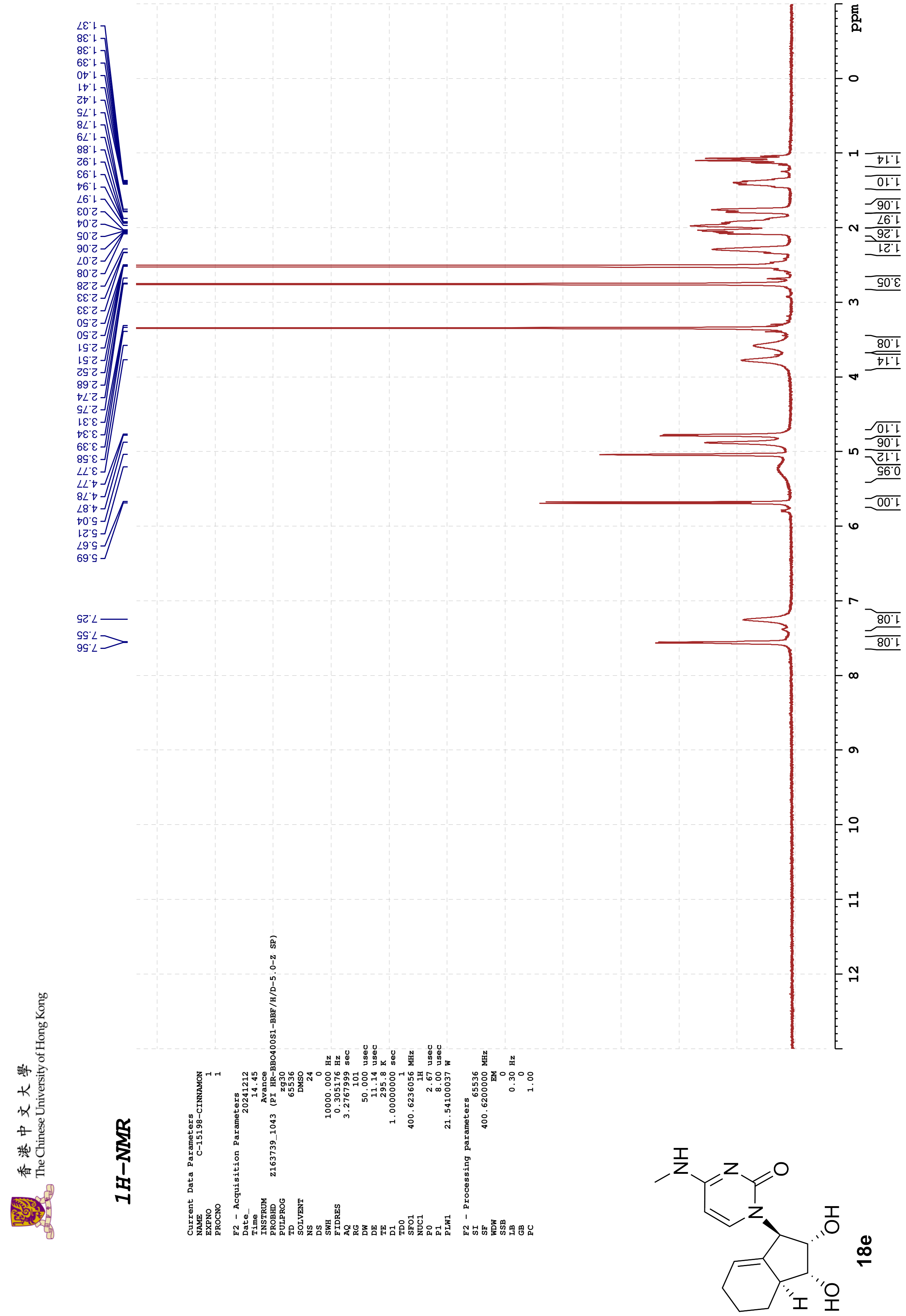

18e

NMR-Spectra for Compound 18e

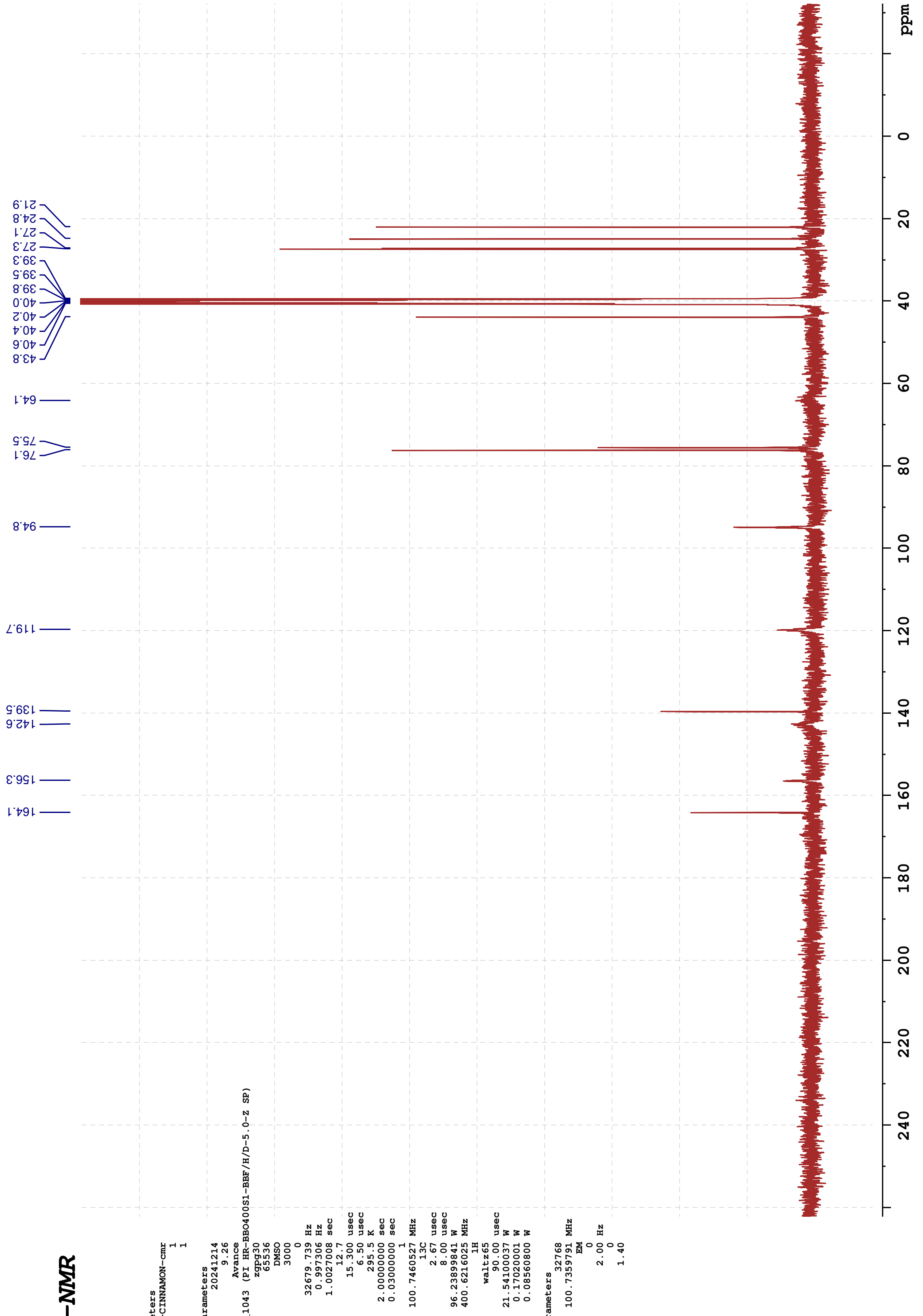

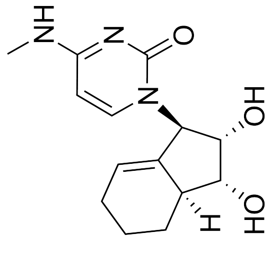

18e

NMR-Spectra for Compound 18f

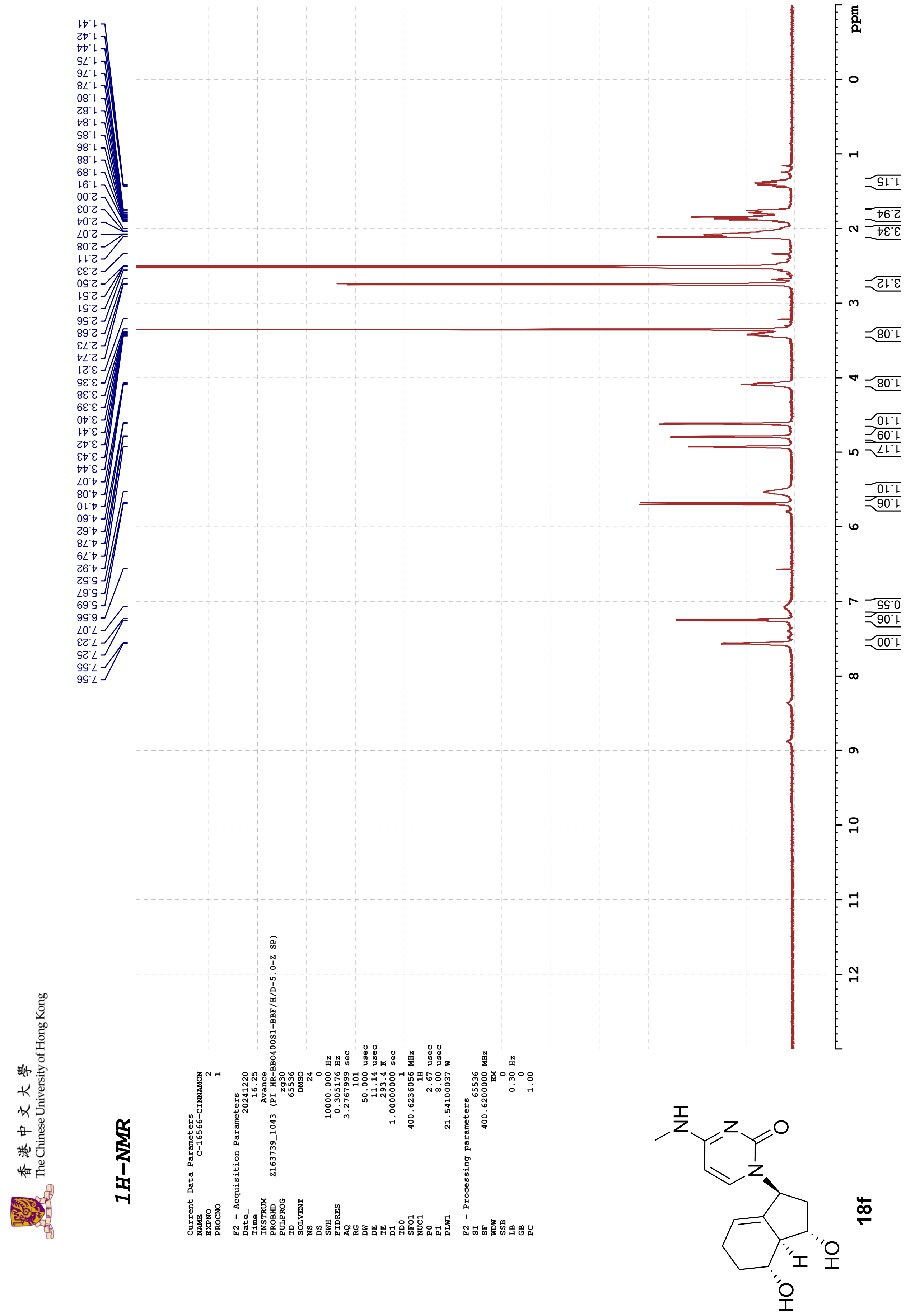

18f

NMR-Spectra for Compound 18f

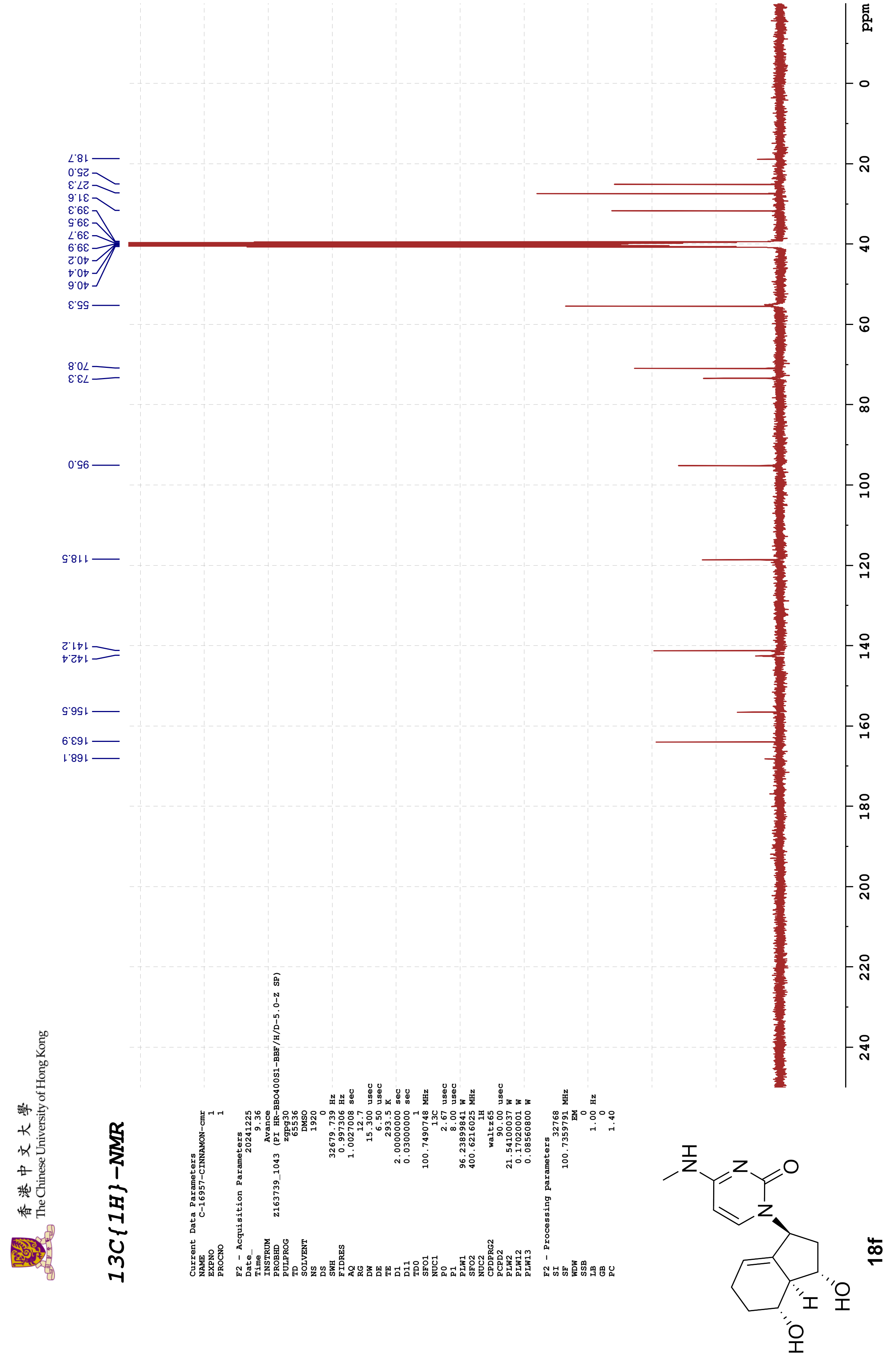

18f

NMR-Spectra for Compound 19a

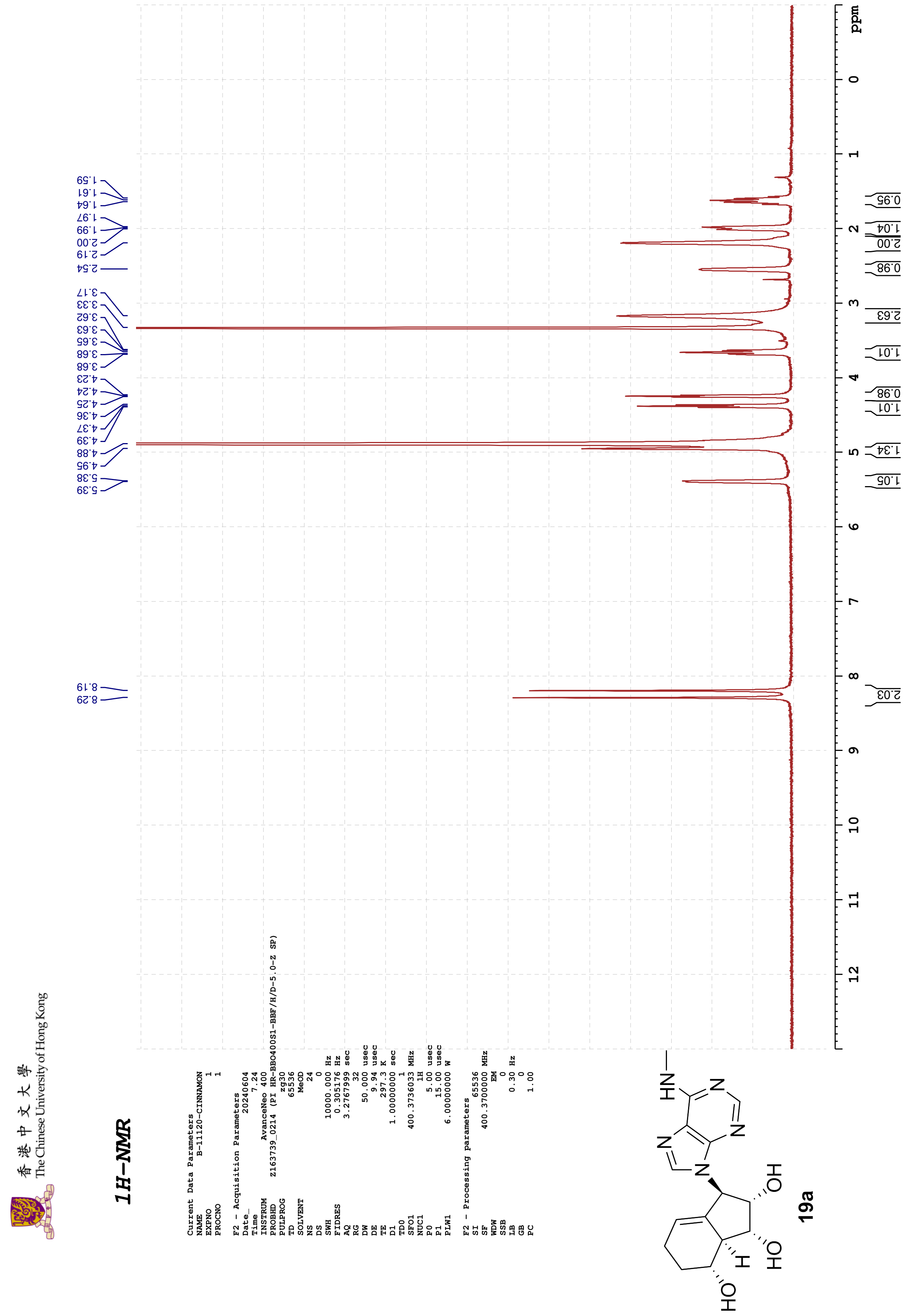

NMR-Spectra for Compound 19a

The Chinese University of Hong Kong
香港中文大學

13C{1H}-NMR

```
Current Data Parameters
NAME              7808-CINNAMON
EXPNO                         1
PROCNO                        1

F2 - Acquisition Parameters
Date_                  20240605
Time                      11.52
INSTRUM    AvanceNeo  Ascend400MHz
PROBHD     Z163739_0198 (PI HR-BBO400S1-BBF/H/D-5.0-Z SP)
PULPROG                 zgpg30
TD                        65536
SOLVENT                    MeOD
NS                         1024
DS                            0
SWH                   23809.523 Hz
FIDRES                 0.726609 Hz
AQ                    1.3762560 sec
RG                          101
DW                       21.000 usec
DE                         6.50 usec
TE                        293.6 K
D1                   2.00000000 sec
D11                  0.03000000 sec
TD0                           1
SFO1                100.6655806 MHz
NUC1                        13C
P0                         2.67 usec
P1                         8.00 usec
PLW1                95.69300079 W
SFO2                400.3016012 MHz
NUC2                         1H
CPDPRG[2]               waltz65
PCPD2                     90.00 usec
PLW2                24.20299911 W
PLW12                0.19123000 W
PLW13                0.09618900 W

F2 - Processing parameters
SI                        32768
SF                  100.6555151 MHz
WDW                          EM
SSB                           0
LB                         1.00 Hz
GB                            0
PC                         1.40
```

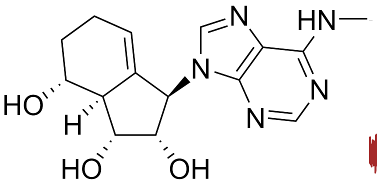

19a

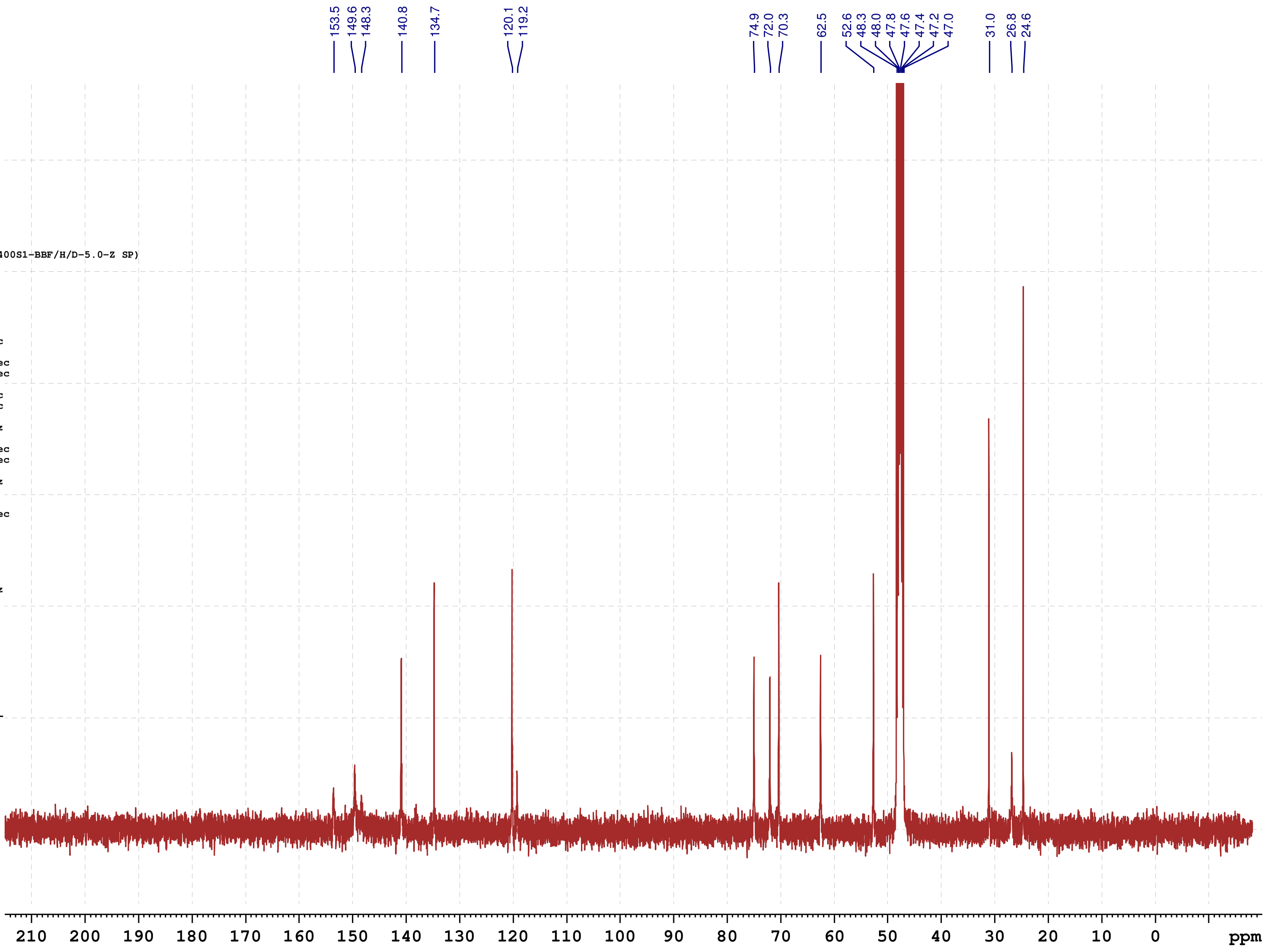

NMR-Spectra for Compound 19b

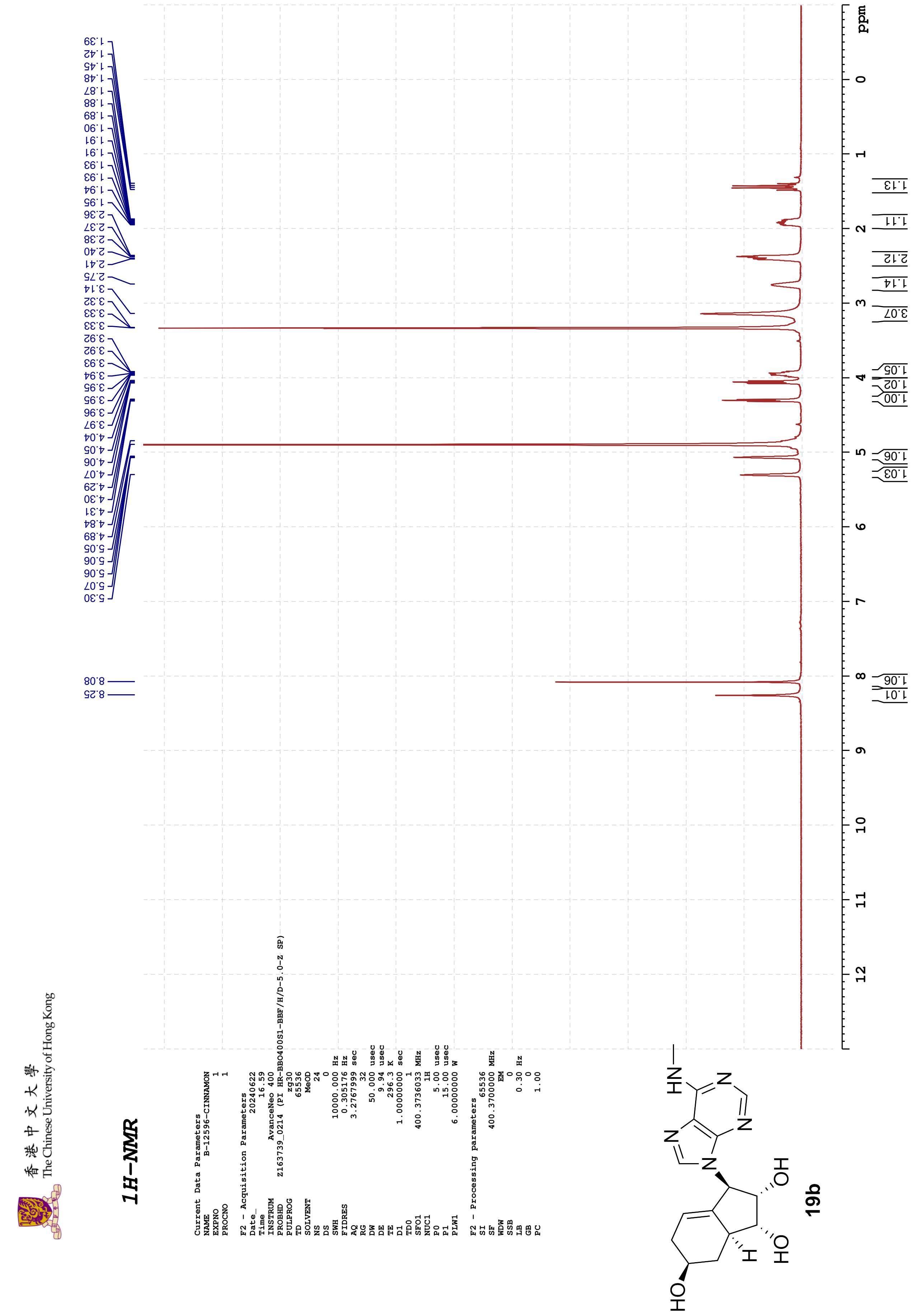

NMR-Spectra for Compound 19b

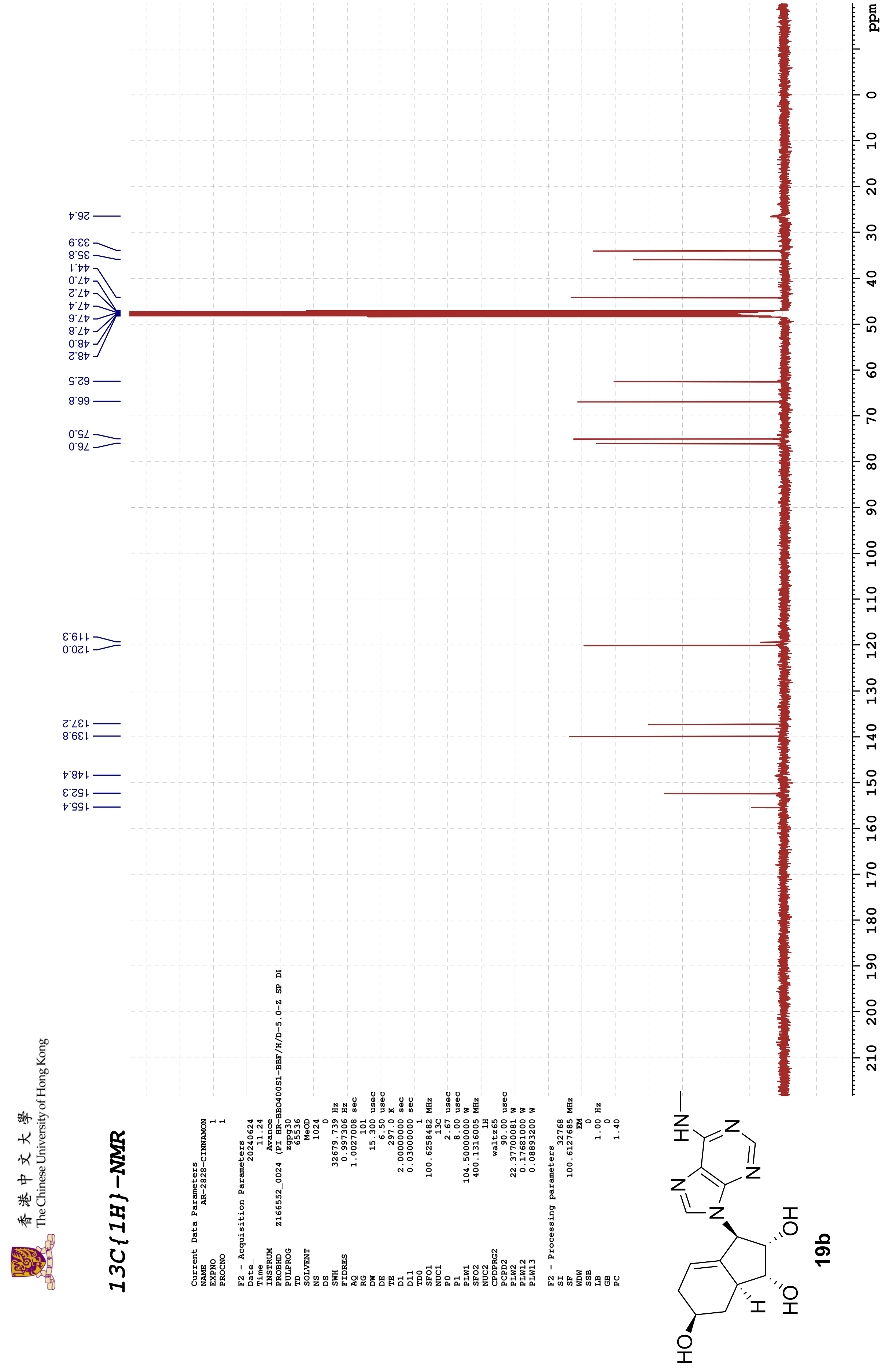

NMR-Spectra for Compound 19c

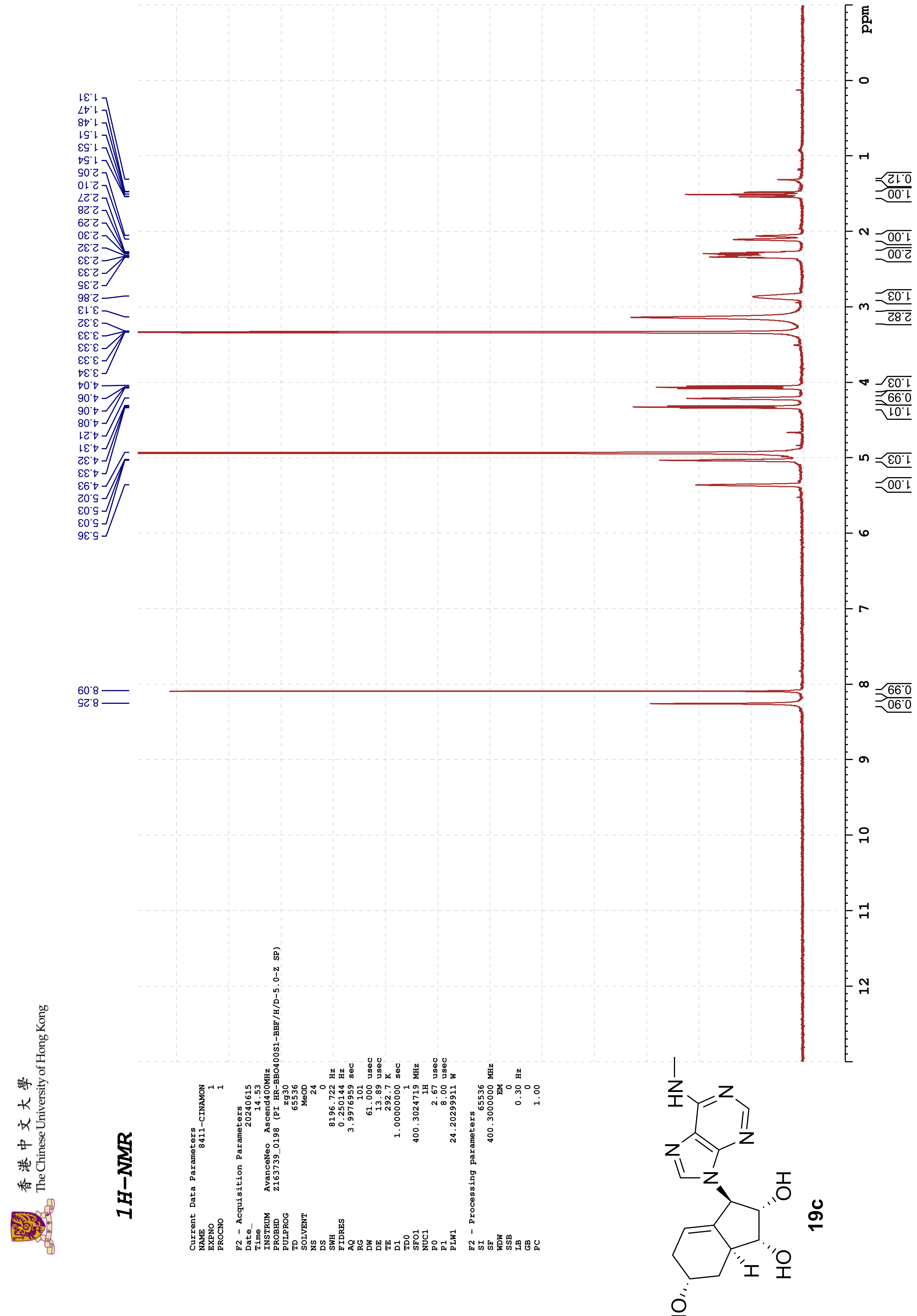

NMR-Spectra for Compound 19c

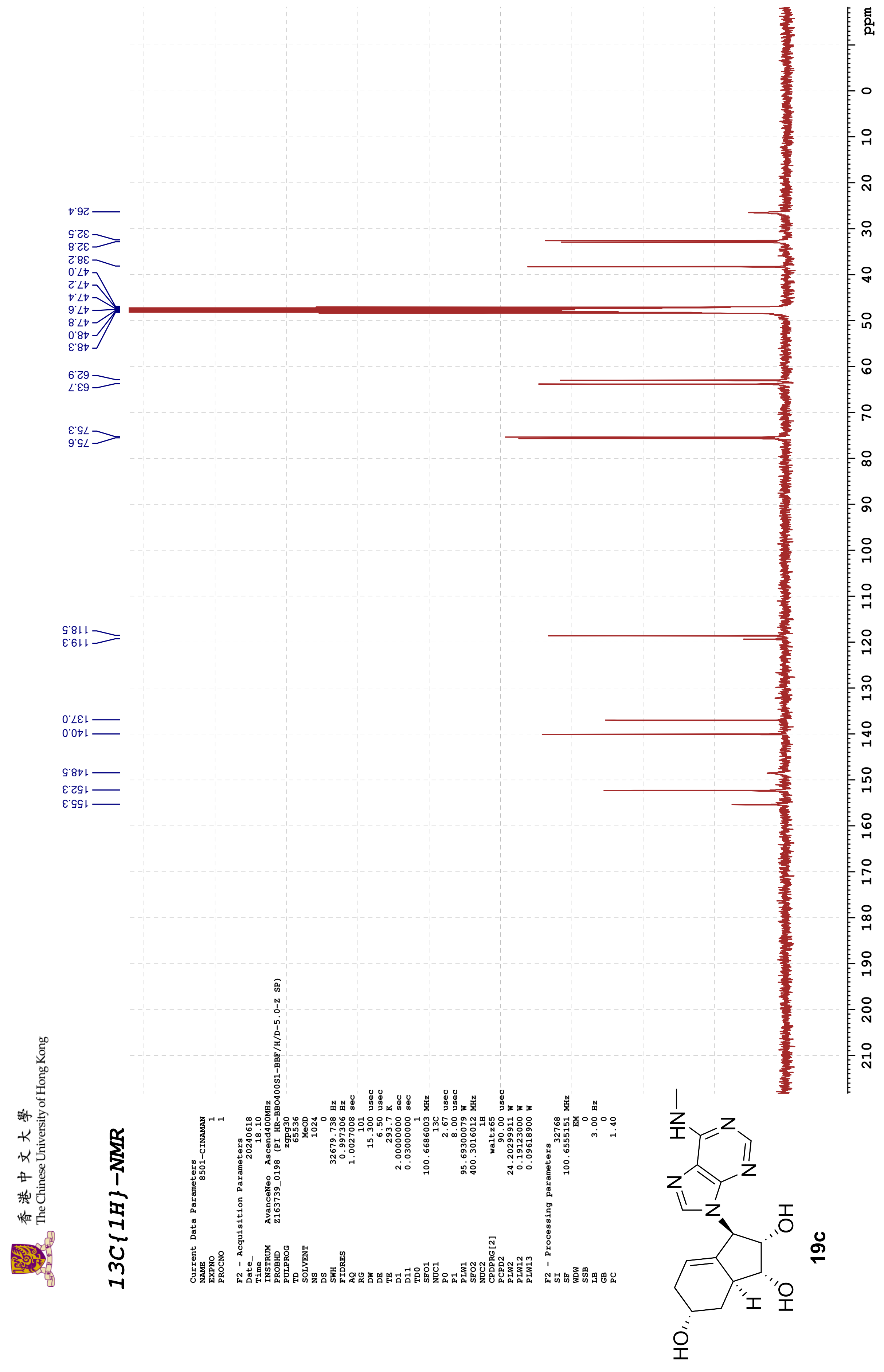

19c

NMR-Spectra for Compound 20a

香港中文大學
The Chinese University of Hong Kong

1H-NMR

```
Current Data Parameters
NAME          B-10726-CINNAMON
EXPNO                        1
PROCNO                       1

F2 - Acquisition Parameters
Date_                 20240528
Time                     16.31
INSTRUM         AvanceNeo 400
PROBHD     Z163739_0214 (PI HR-BBO400S1-BBF/H/D-5.0-Z SP)
PULPROG                   zg30
TD                       65536
SOLVENT                   MeOD
NS                          24
DS                           0
SWH              10000.000 Hz
FIDRES            0.305176 Hz
AQ               3.2767999 sec
RG                          32
DW                  50.000 usec
DE                    9.94 usec
TE                     297.2 K
D1              1.00000000 sec
TD0                          1
SFO1          400.3736033 MHz
NUC1                        1H
P0                    5.00 usec
P1                   15.00 usec
PLW1            6.00000000 W

F2 - Processing parameters
SI                       65536
SF            400.3700000 MHz
WDW                         EM
SSB                          0
LB                     0.30 Hz
GB                           0
PC                        1.00
```

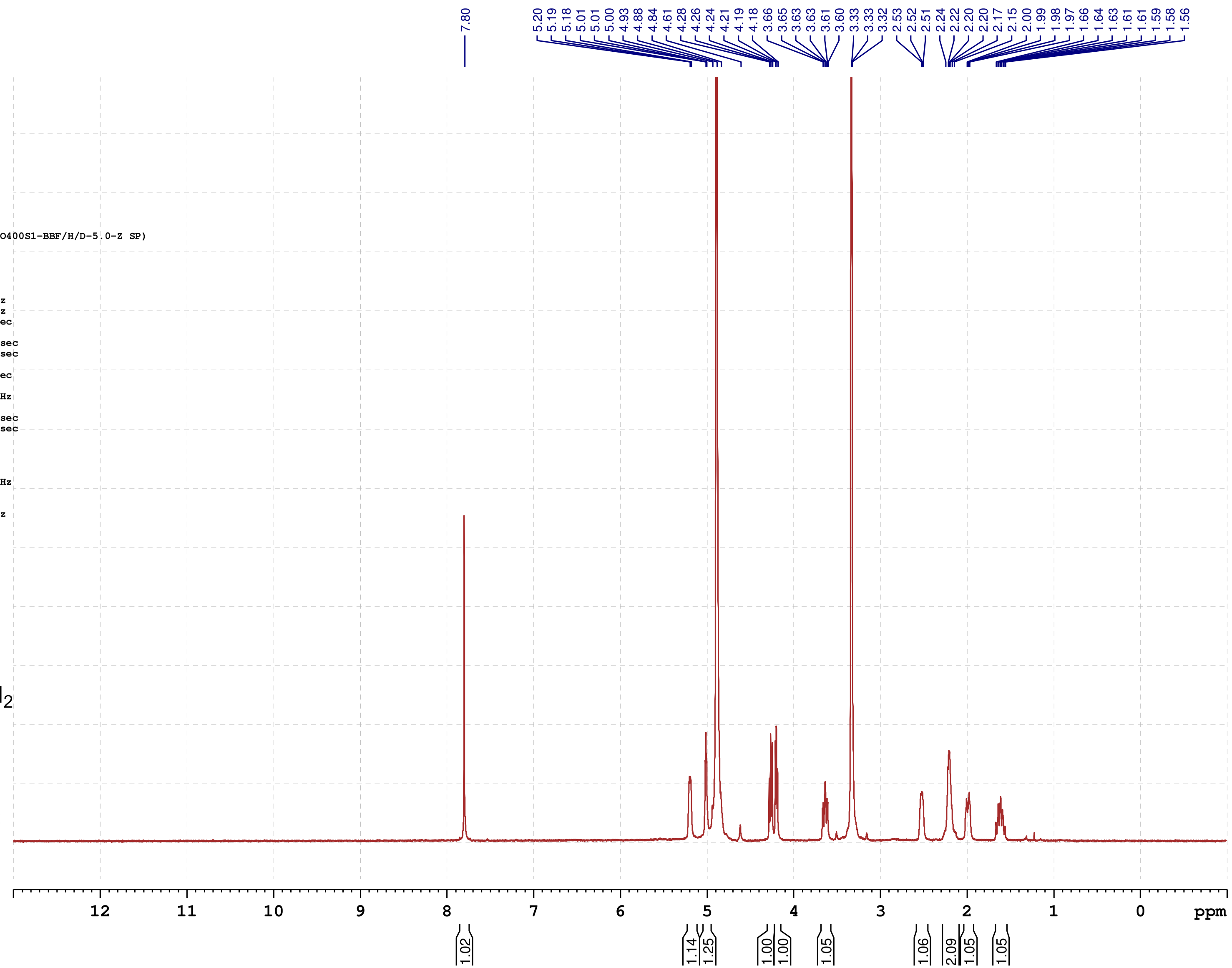

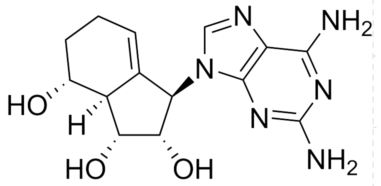

20a

NMR-Spectra for Compound 20a

香港中文大學
The Chinese University of Hong Kong

13C{1H}-NMR

```
Current Data Parameters
NAME              7707-CINNAMON
EXPNO                         1
PROCNO                        1

F2 - Acquisition Parameters
Date_                  20240530
Time                      21.43
INSTRUM    AvanceNeo  Ascend400MHz
PROBHD     Z163739_0198 (PI HR-BBO400S1-BBF/H/D-5.0-Z SP)
PULPROG                 zgpg30
TD                        65536
SOLVENT                    MeOD
NS                         1024
DS                            0
SWH                   32679.738 Hz
FIDRES                 0.997306 Hz
AQ                    1.0027008 sec
RG                          101
DW                       15.300 usec
DE                         6.50 usec
TE                        293.4 K
D1                   2.00000000 sec
D11                  0.03000000 sec
TD0                           1
SFO1                100.6686003 MHz
NUC1                        13C
P0                         2.67 usec
P1                         8.00 usec
PLW1                95.69300079 W
SFO2                400.3016012 MHz
NUC2                         1H
CPDPRG[2]               waltz65
PCPD2                     90.00 usec
PLW2                24.20299911 W
PLW12                0.19123000 W
PLW13                0.09618900 W

F2 - Processing parameters
SI                        32768
SF                  100.6555151 MHz
WDW                          EM
SSB                           0
LB                         1.00 Hz
GB                            0
PC                         1.40
```

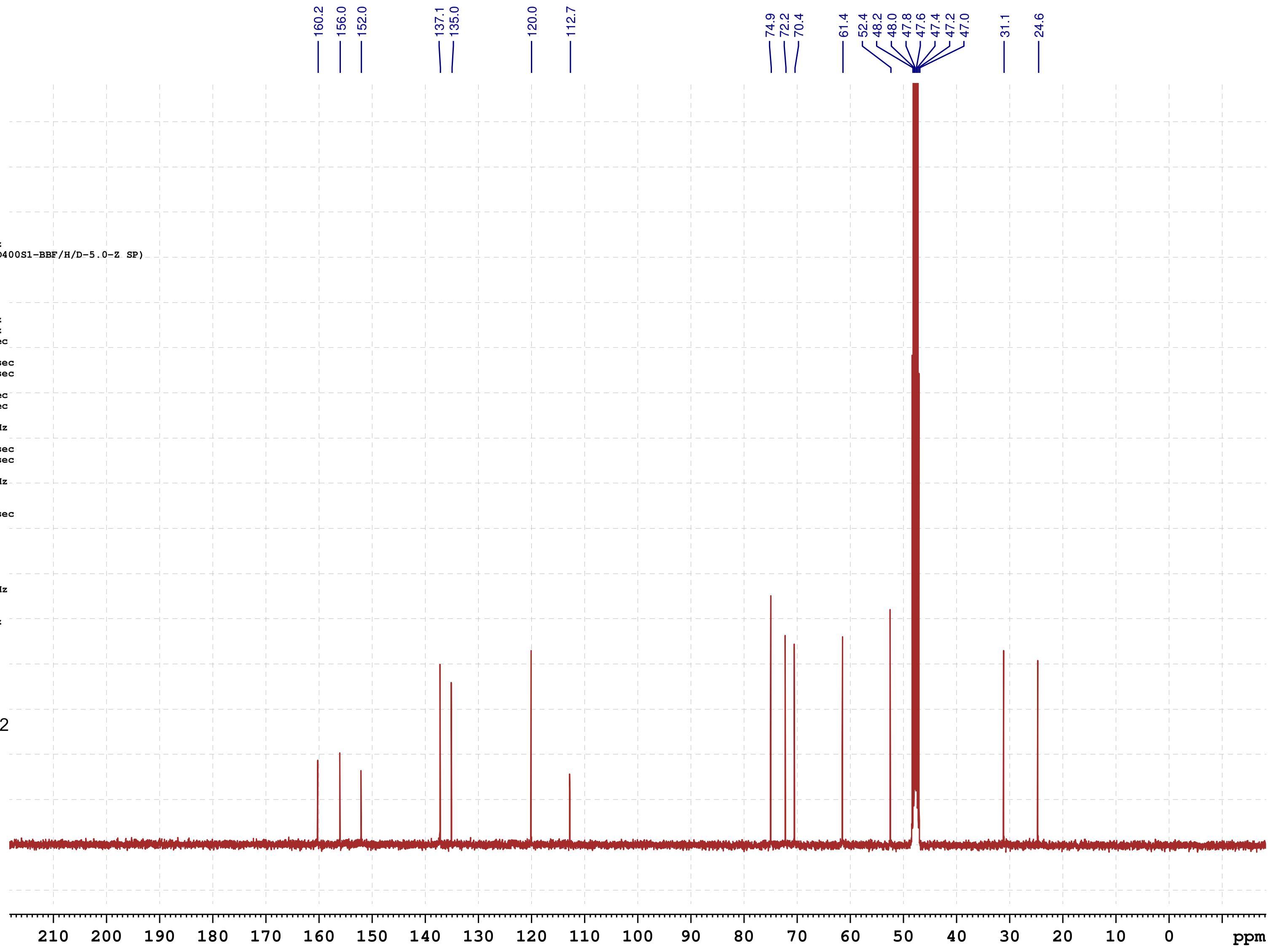

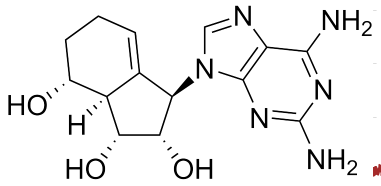

20a

NMR-Spectra for Compound 20b

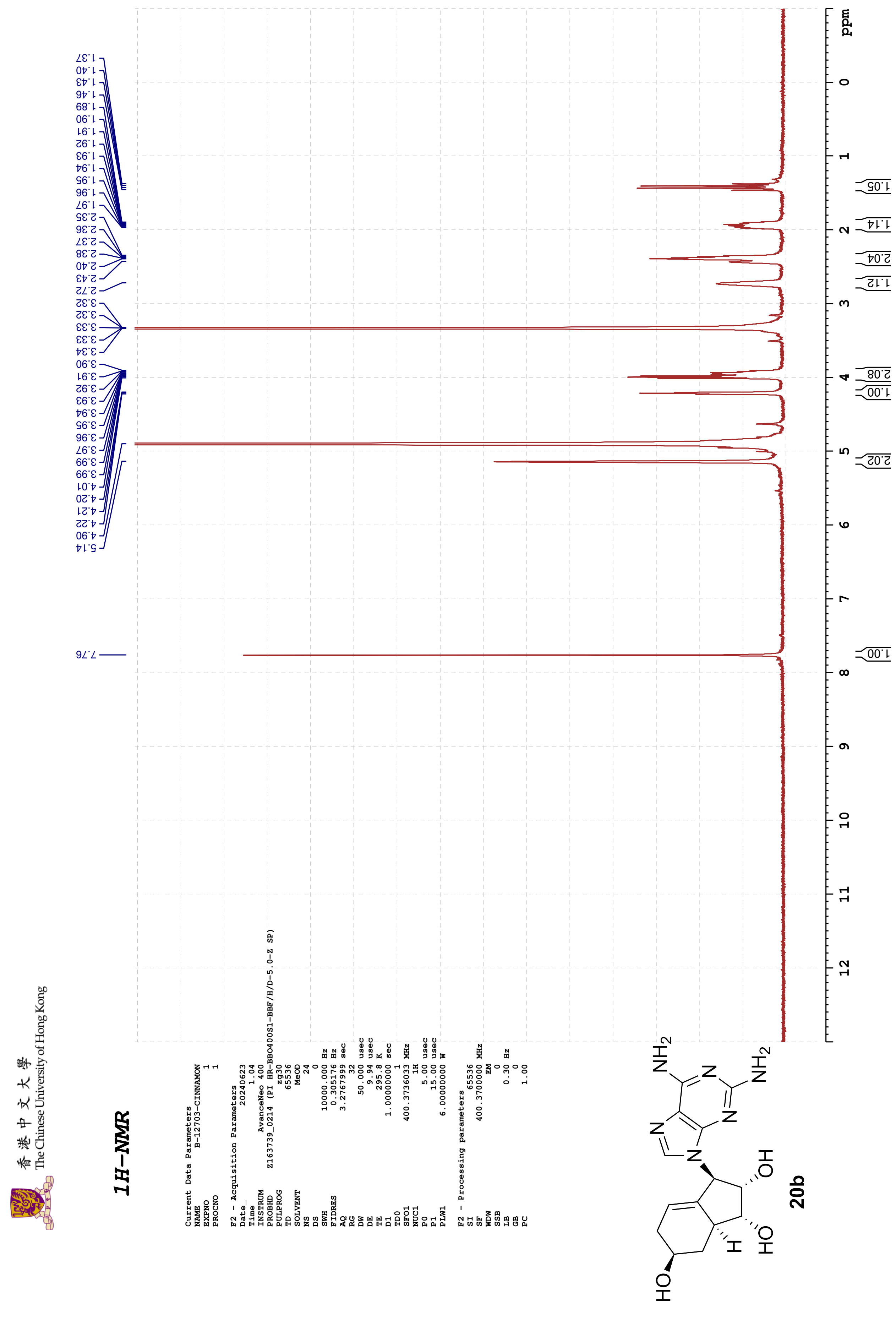

NMR-Spectra for Compound 20b

香港中文大學 The Chinese University of Hong Kong

13C{1H}-NMR

Current Data Parameters
NAME 8946-CINAMON
EXPNO 1
PROCNO 1

F2 - Acquisition Parameters
Date_ 20240627
Time 6.45
INSTRUM AvanceNeo Ascend400MHz
PROBHD Z163739_0198 (PI HR-BBO400S1-BBF/H/D-5.0-Z SP)
PULPROG zgpg30
TD 65536
SOLVENT MeOD
NS 3000
DS 0
SWH 32679.738 Hz
FIDRES 0.997306 Hz
AQ 1.0027008 sec
RG 101
DW 15.300 usec
DE 6.50 usec
TE 293.6 K
D1 2.00000000 sec
D11 0.03000000 sec
TD0 1
SFO1 100.6686003 MHz
NUC1 13C
P0 2.67 usec
P1 8.00 usec
PLW1 95.69300079 W
SFO2 400.3016012 MHz
NUC2 1H
CPDPRG[2] waltz65
PCPD2 90.00 usec
PLW2 24.20299911 W
PLW12 0.19123000 W
PLW13 0.09618900 W

F2 - Processing parameters
SI 32768
SF 100.6555151 MHz
WDW EM
SSB 0
LB 1.00 Hz
GB 0
PC 1.40

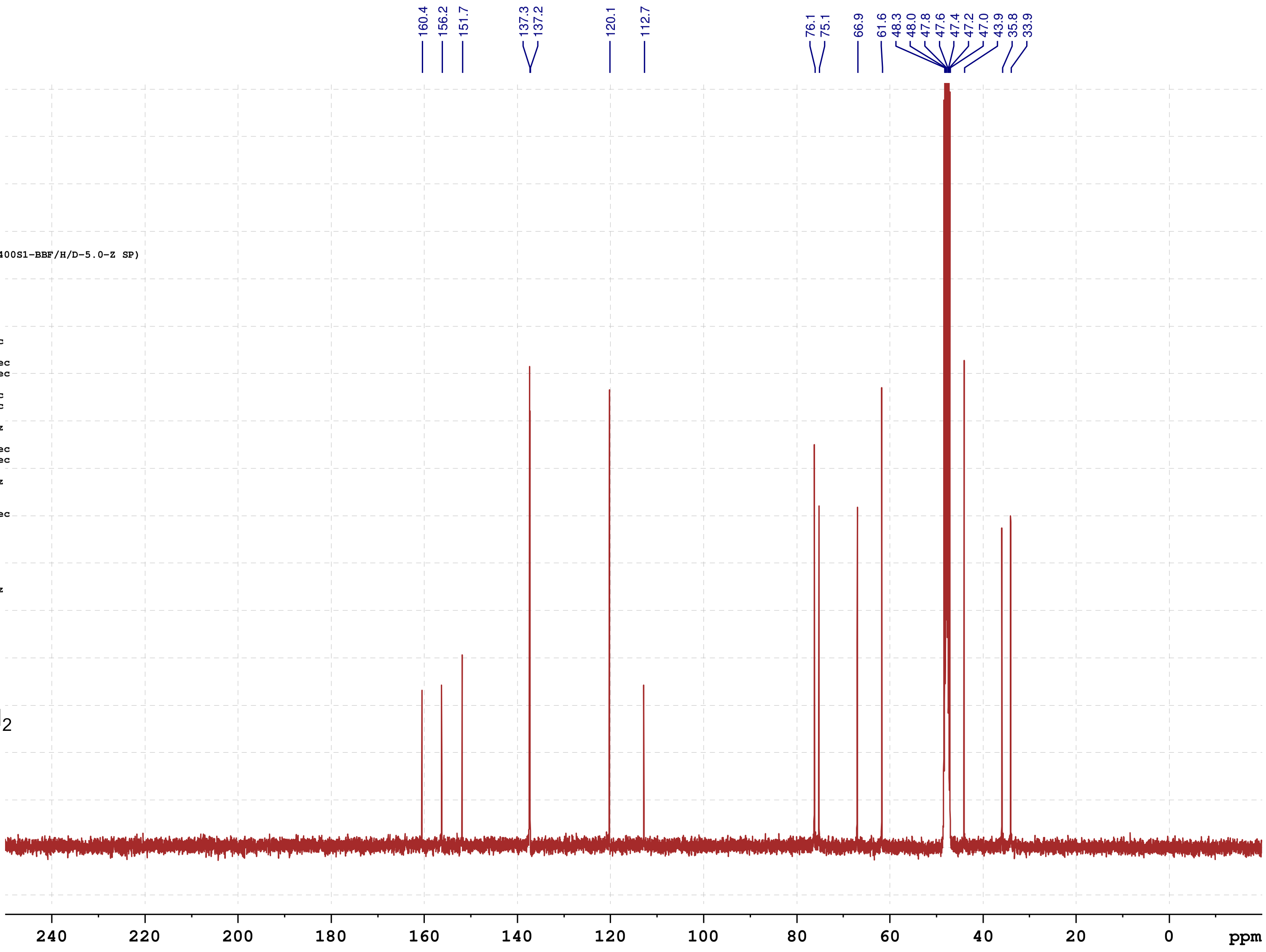

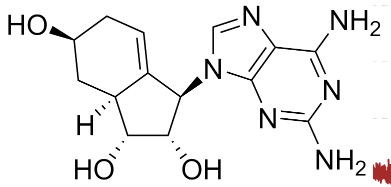

20b

NMR-Spectra for Compound 20c

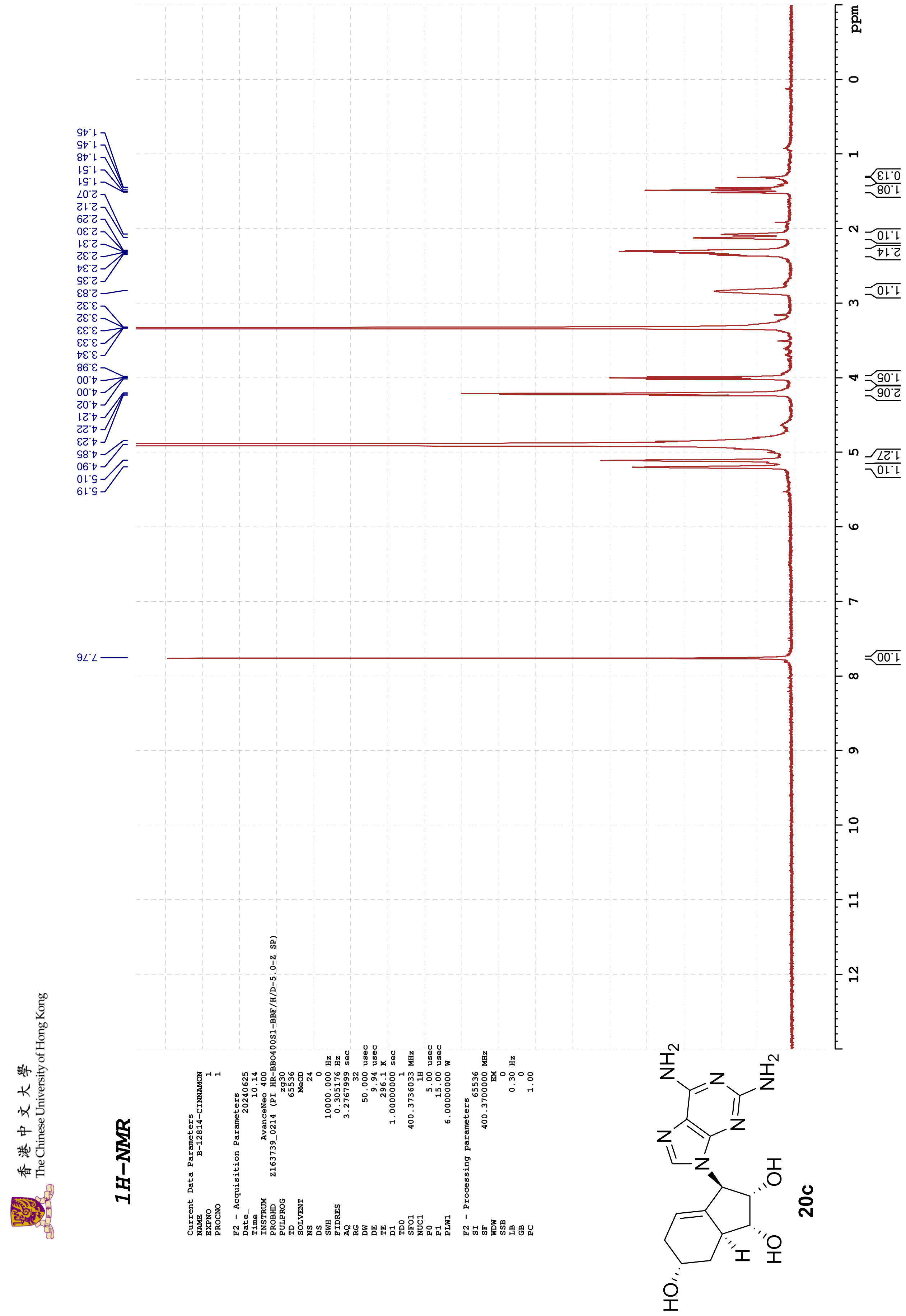

NMR-Spectra for Compound 20c

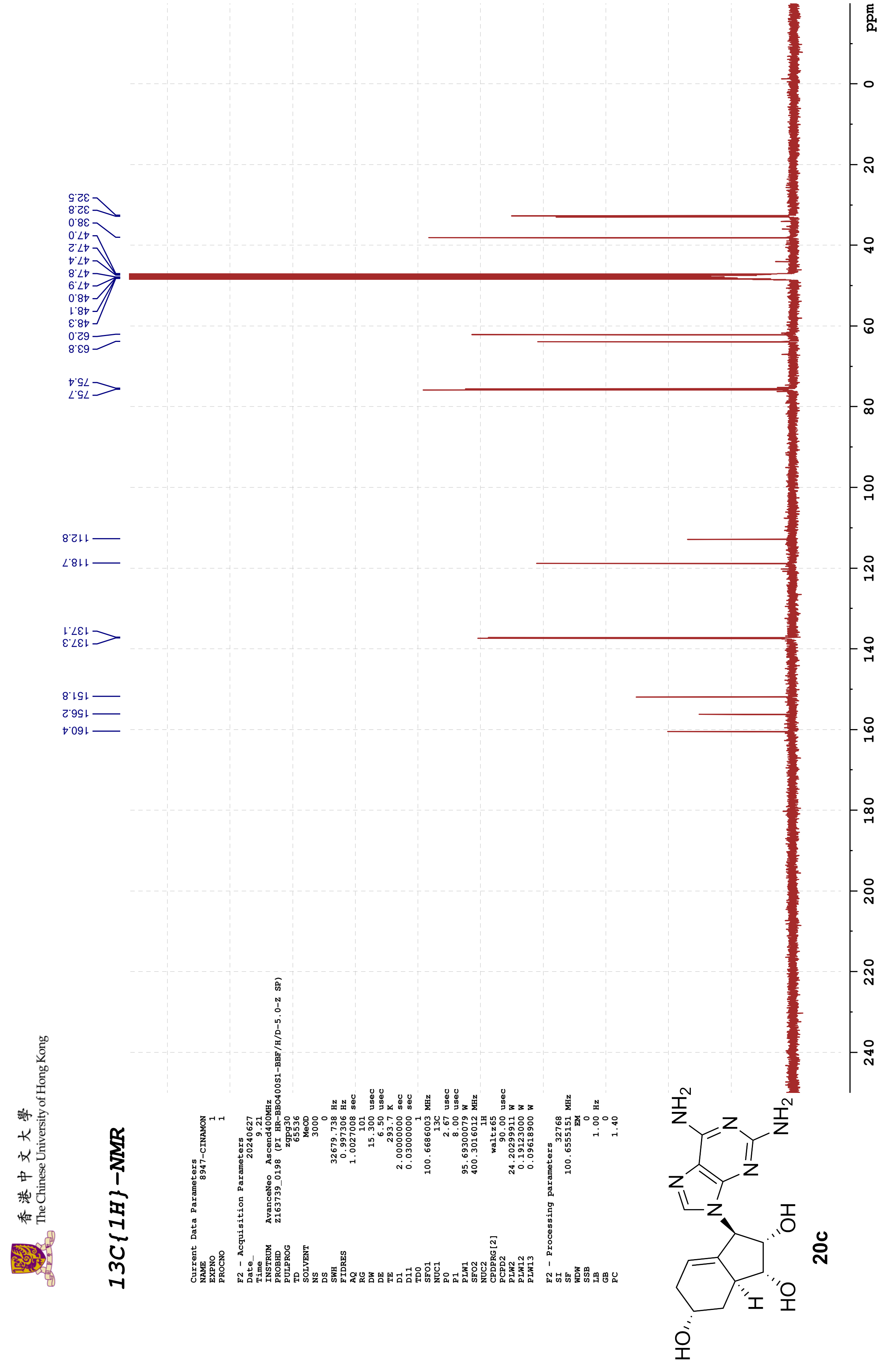

NMR-Spectra for Compound 21a

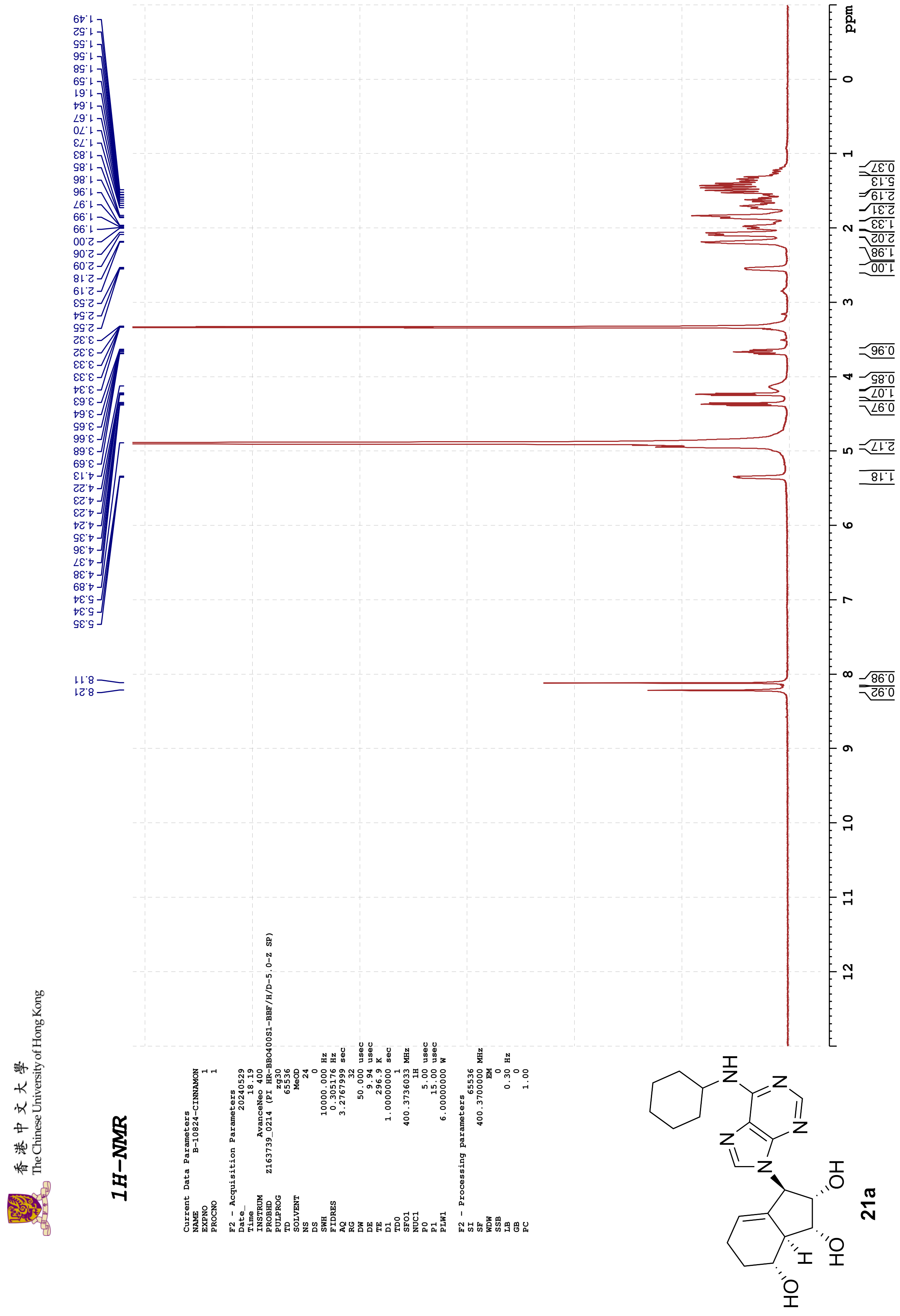

NMR-Spectra for Compound 21a

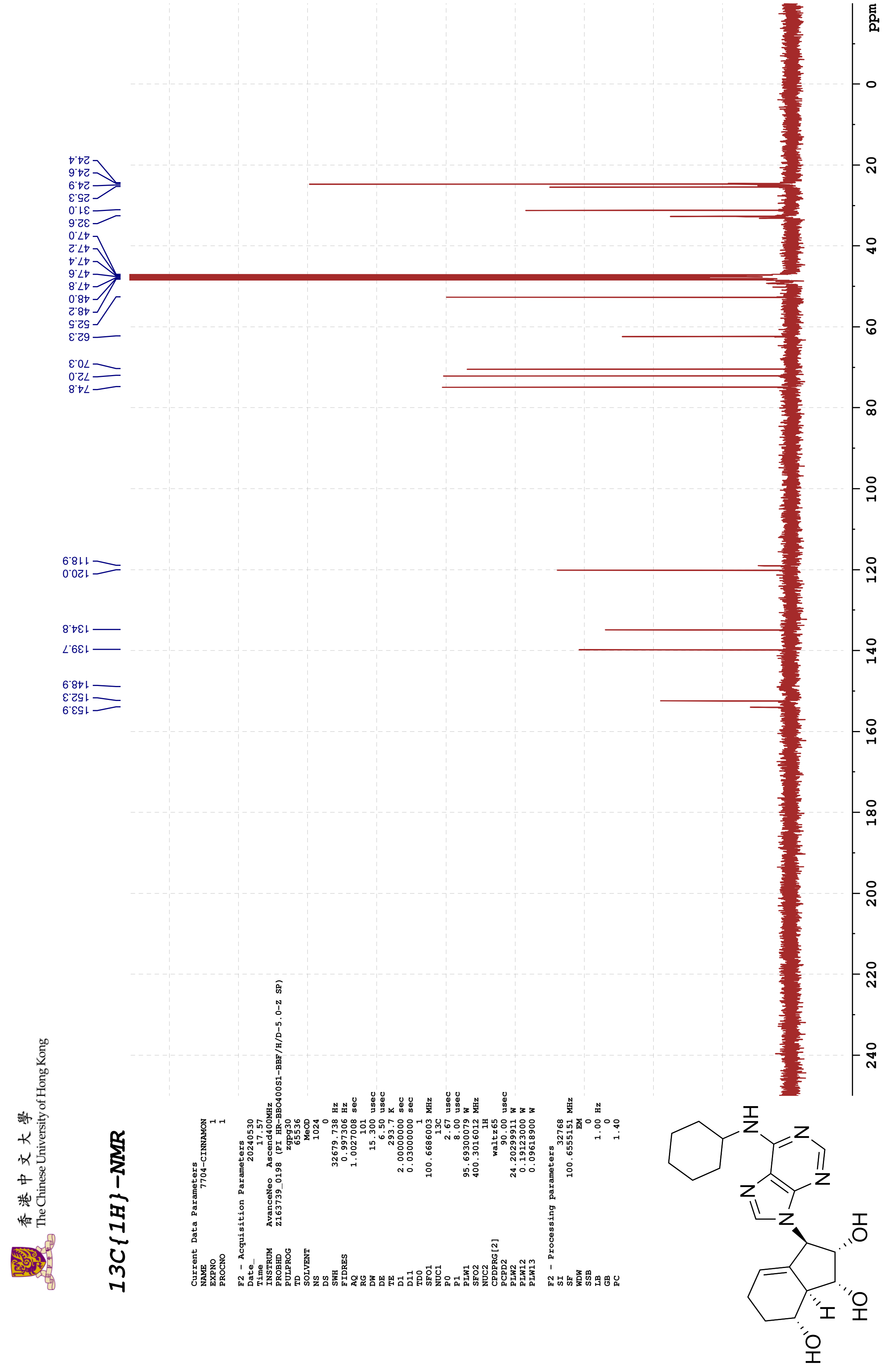

21a

NMR-Spectra for Compound 22a

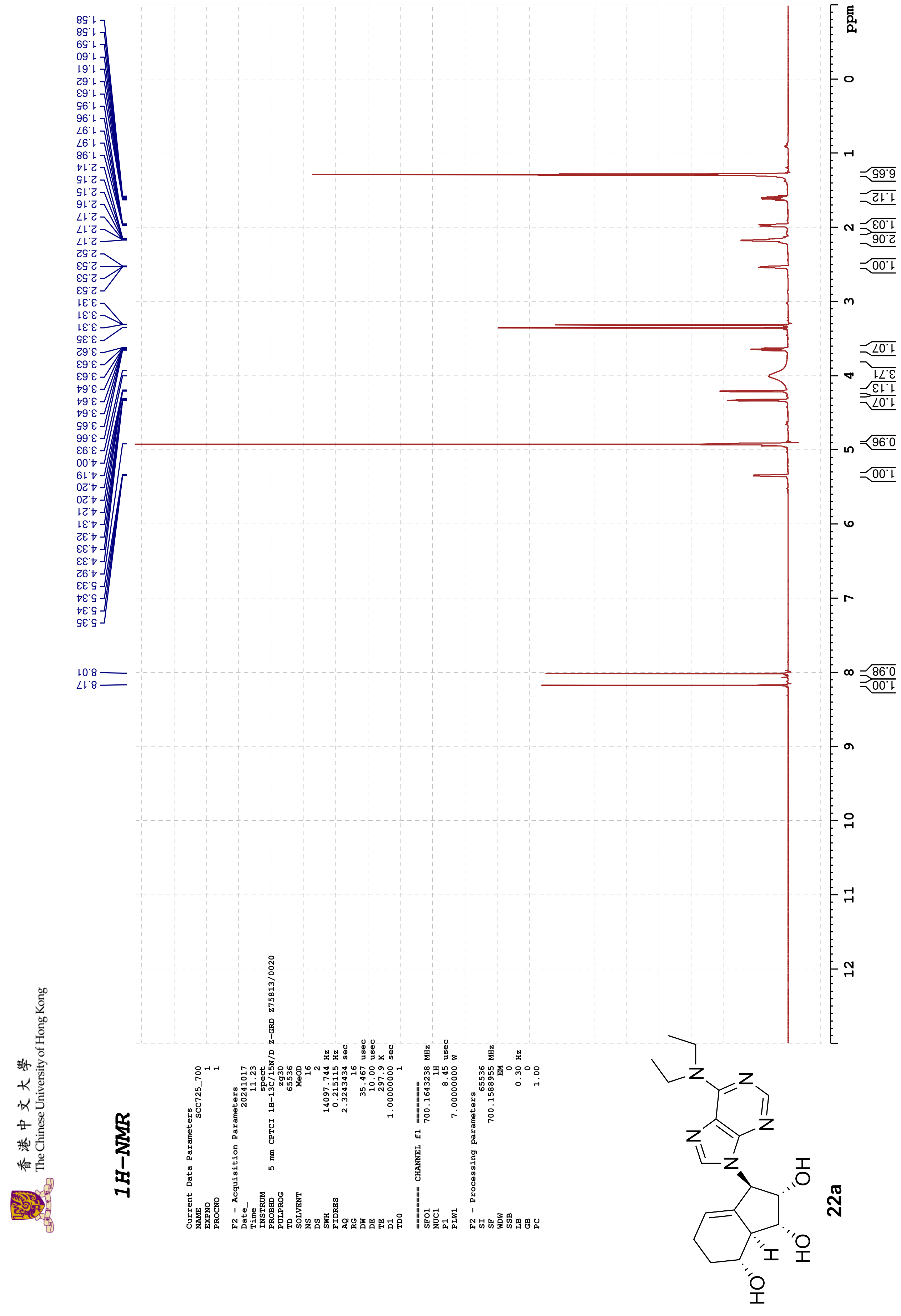

NMR-Spectra for Compound 22a

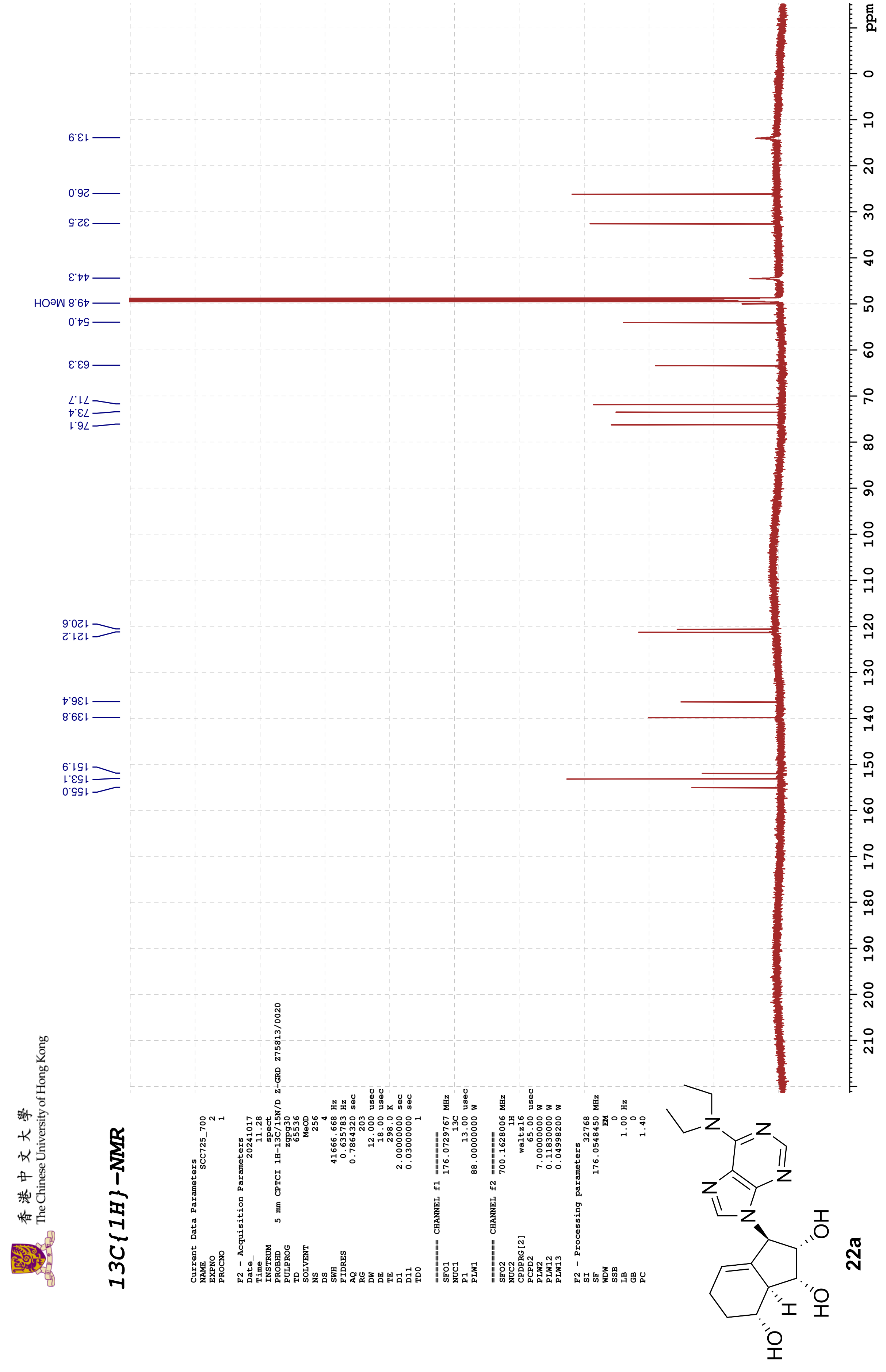

NMR-Spectra for Compound 23a

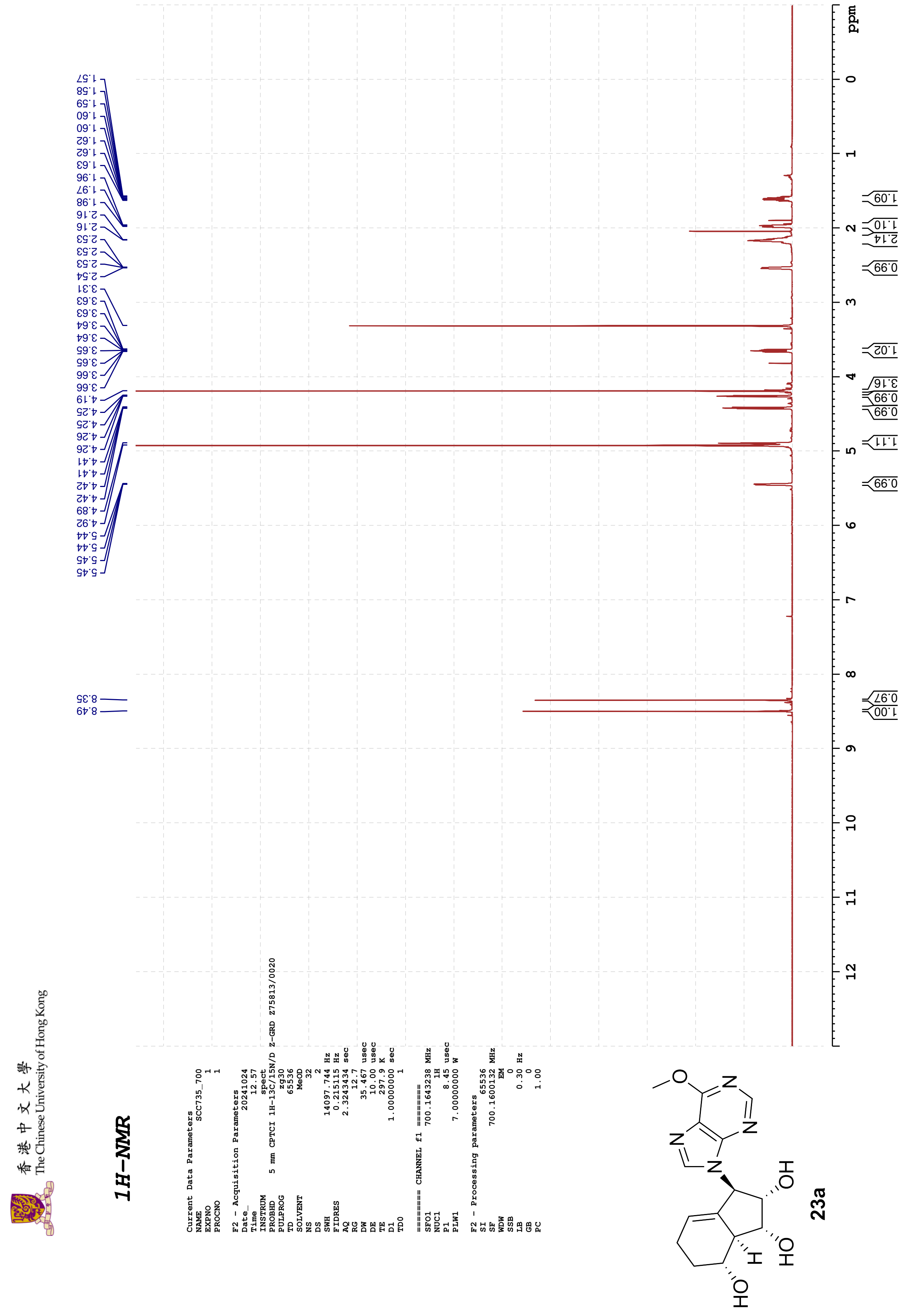

NMR-Spectra for Compound 23a

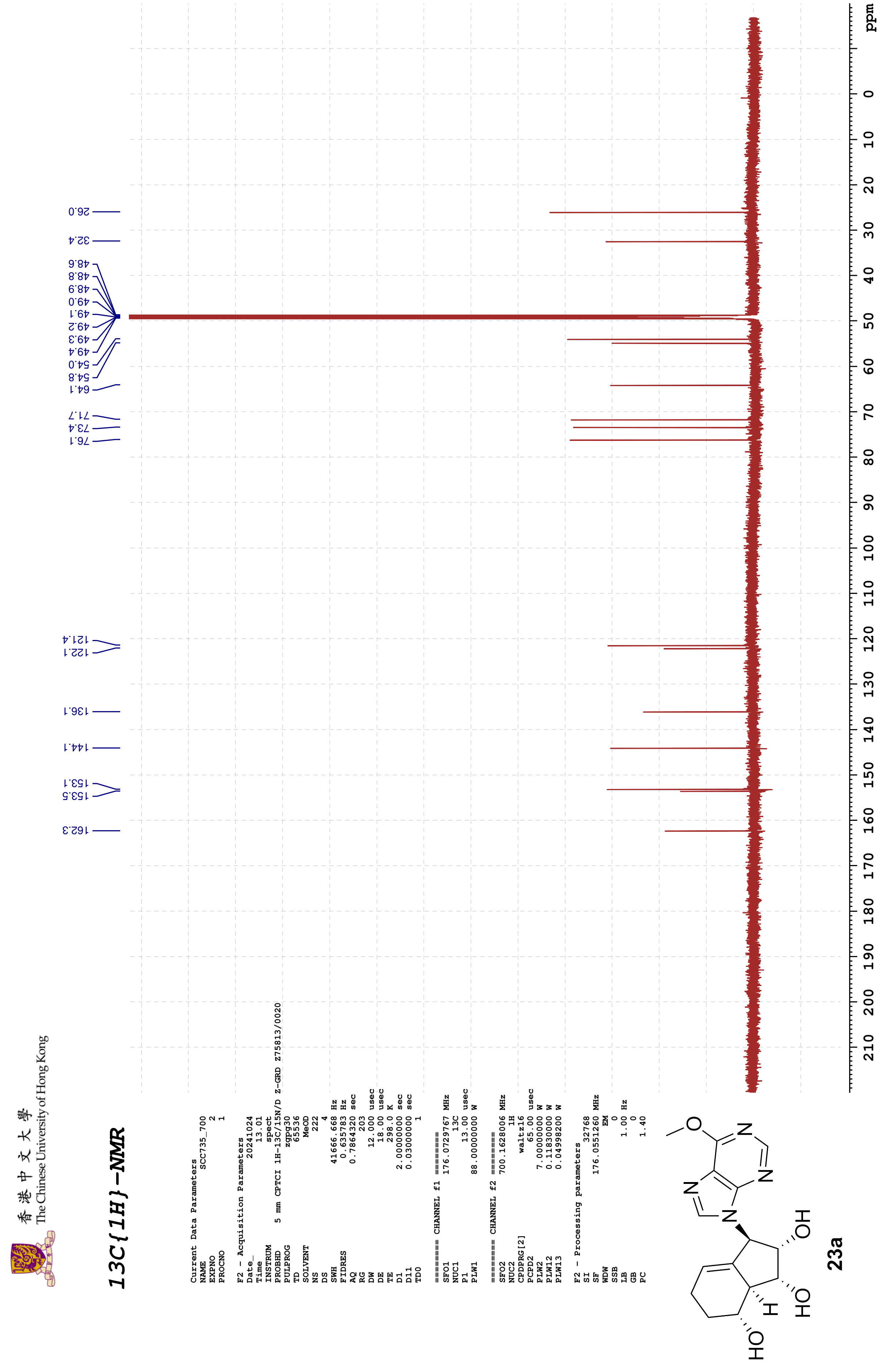

NMR-Spectra for Compound 23d

The Chinese University of Hong Kong

1H-NMR

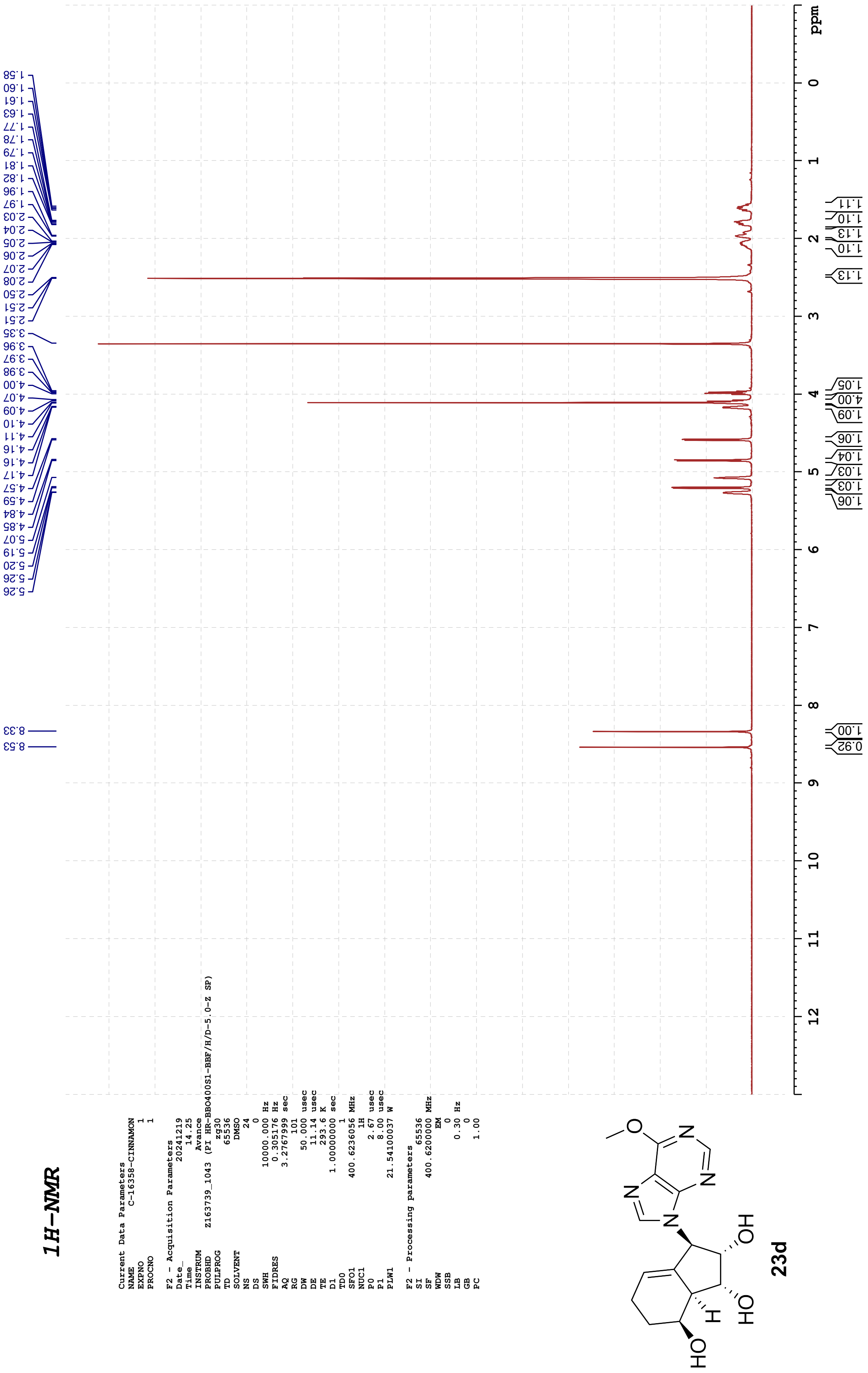

23d

NMR-Spectra for Compound 23d

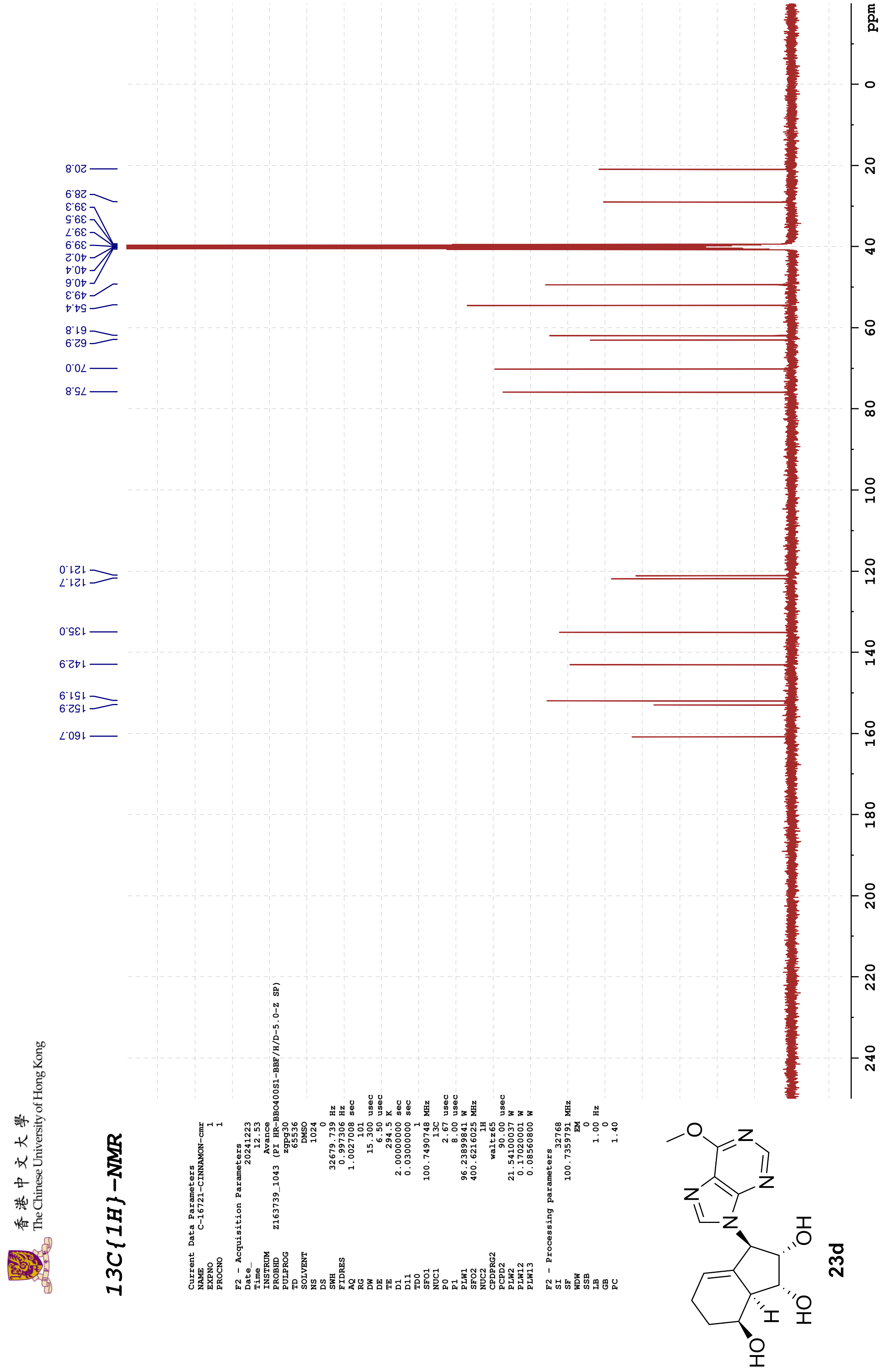

23d

NMR-Spectra for Compound 23e

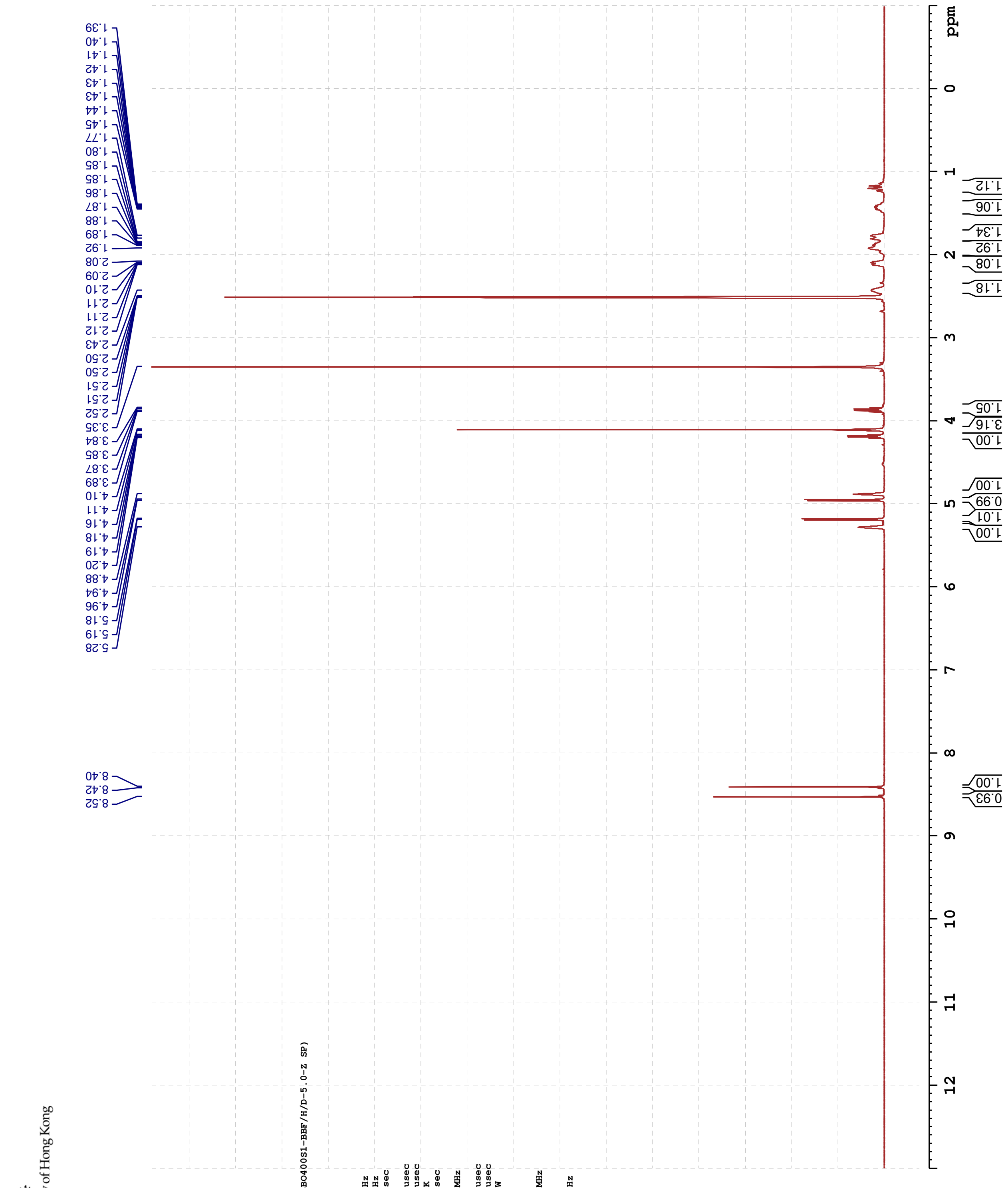

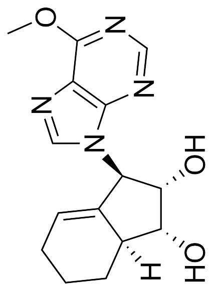

23e

NMR-Spectra for Compound 23e

香港中文大學 The Chinese University of Hong Kong

13C{1H}-NMR

```
Current Data Parameters
NAME          C-13499-CINNAMON
EXPNO                        1
PROCNO                       1

F2 - Acquisition Parameters
Date_                 20241130
Time                      9.11
INSTRUM                 Avance
PROBHD     Z163739_1043 (PI HR-BBO400S1-BBF/H/D-5.0-Z SP)
PULPROG                zgpg30
TD                       65536
SOLVENT                   DMSO
NS                        1024
DS                           0
SWH                 32679.739 Hz
FIDRES               0.997306 Hz
AQ                  1.0027008 sec
RG                        12.7
DW                      15.300 usec
DE                        6.50 usec
TE                       294.2 K
D1                  2.00000000 sec
D11                 0.03000000 sec
TD0                          1
SFO1               100.7490748 MHz
NUC1                       13C
P0                        2.67 usec
P1                        8.00 usec
PLW1               96.23899841 W
SFO2               400.6216025 MHz
NUC2                        1H
CPDPRG2                waltz65
PCPD2                    90.00 usec
PLW2               21.54100037 W
PLW12               0.17020001 W
PLW13               0.08560800 W

F2 - Processing parameters
SI                       32768
SF                 100.7359791 MHz
WDW                         EM
SSB                          0
LB                        1.00 Hz
GB                           0
PC                        1.40
```

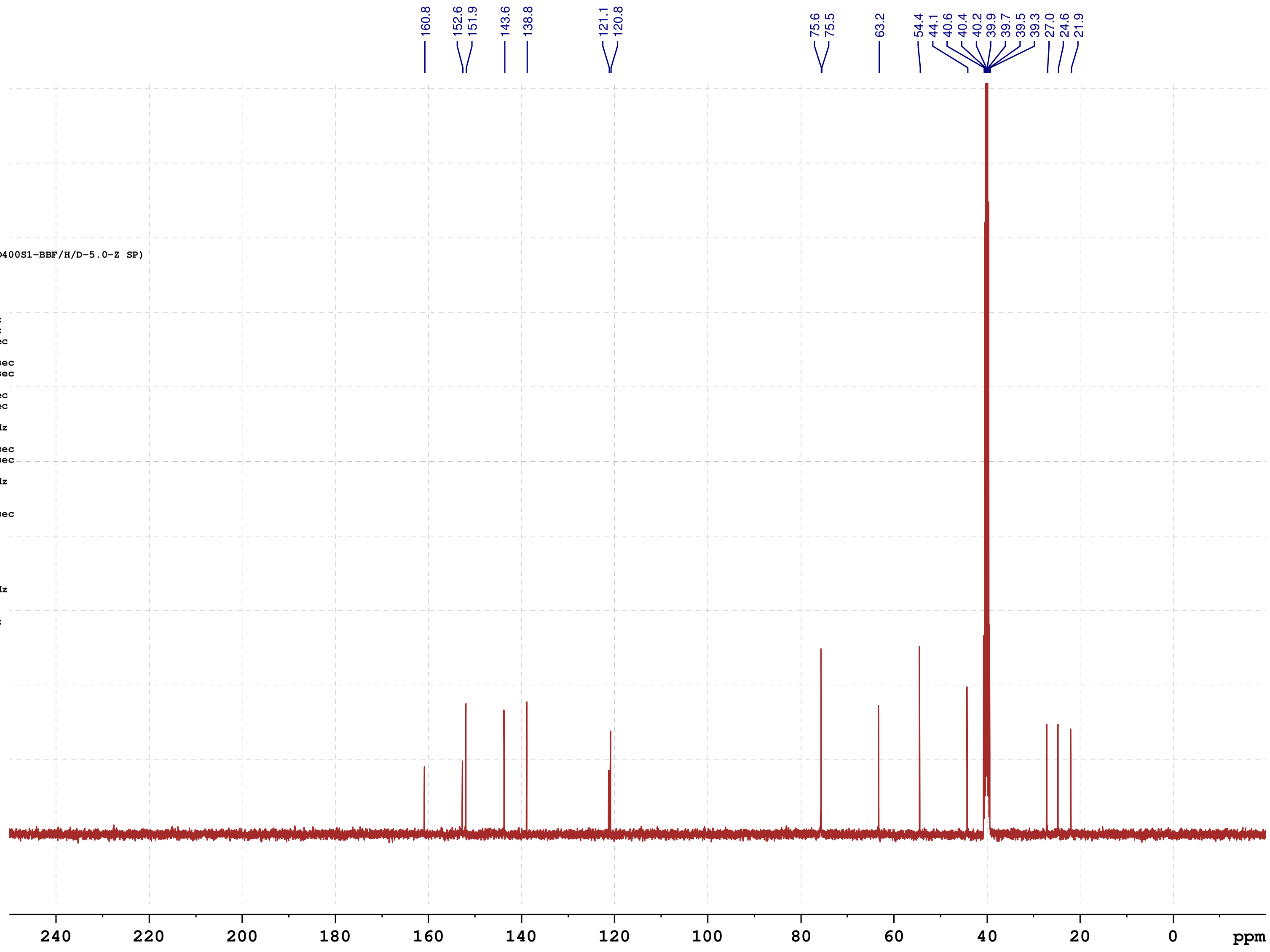

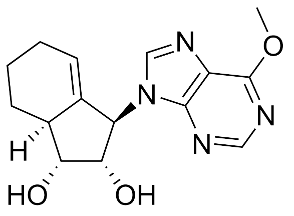

23e

NMR Spectra For Key Intermediates

NMR-Spectra for Compound 28

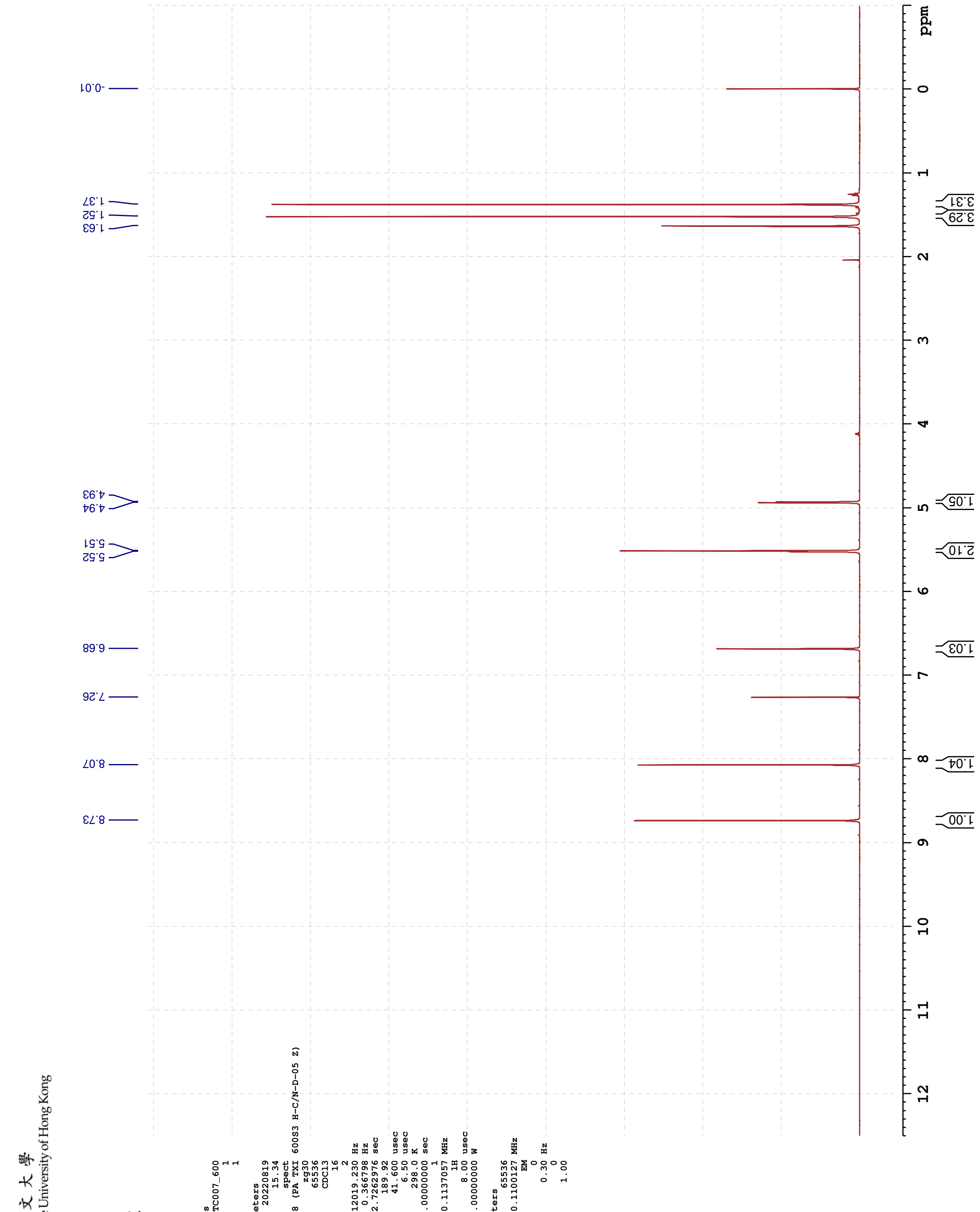

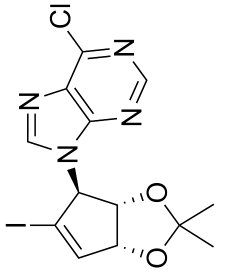

28

NMR-Spectra for Compound 28

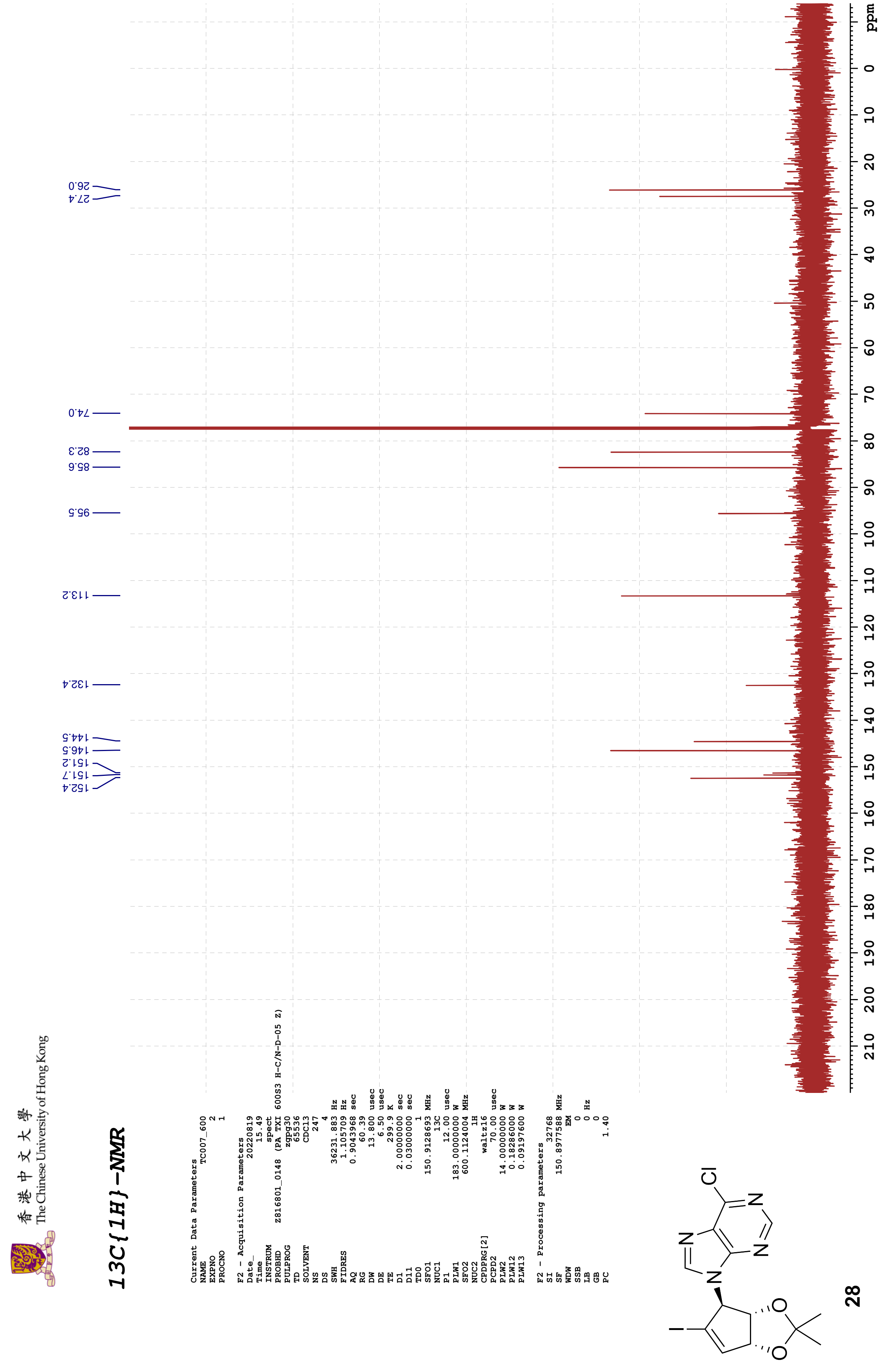

NMR-Spectra for Compound 29

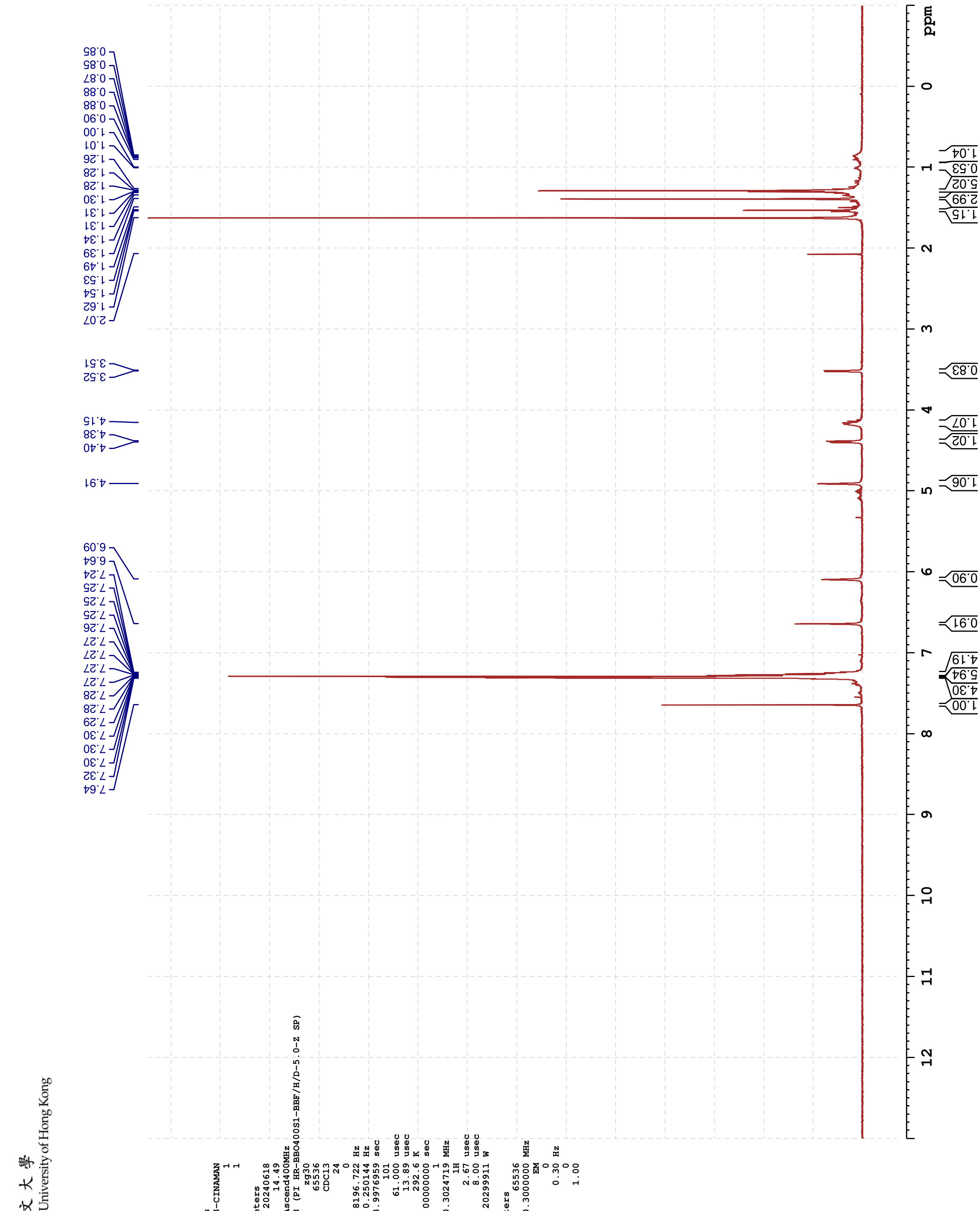

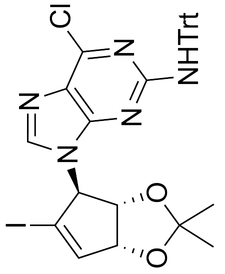

29

NMR-Spectra for Compound 29

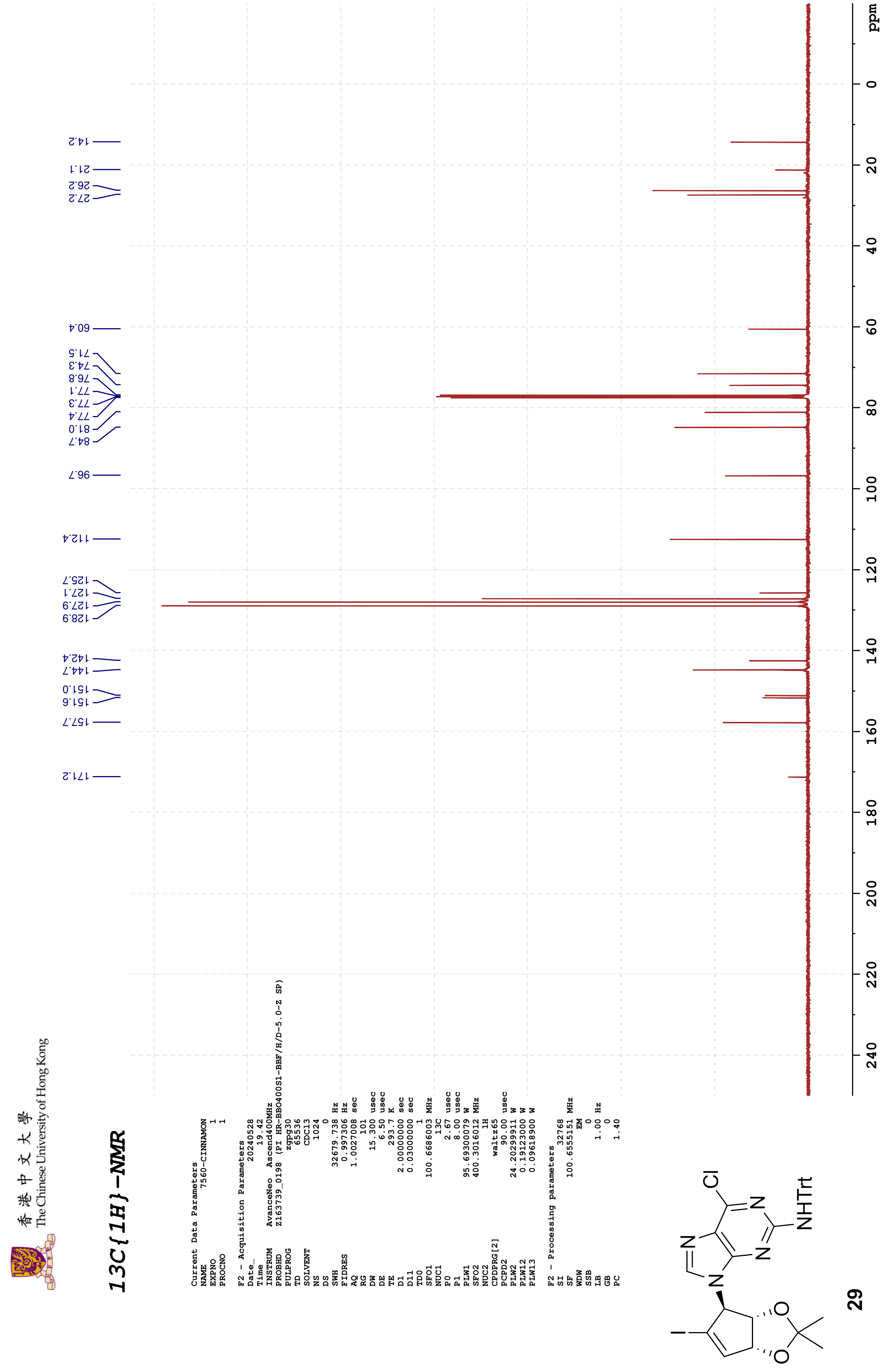

NMR-Spectra for Compound 30

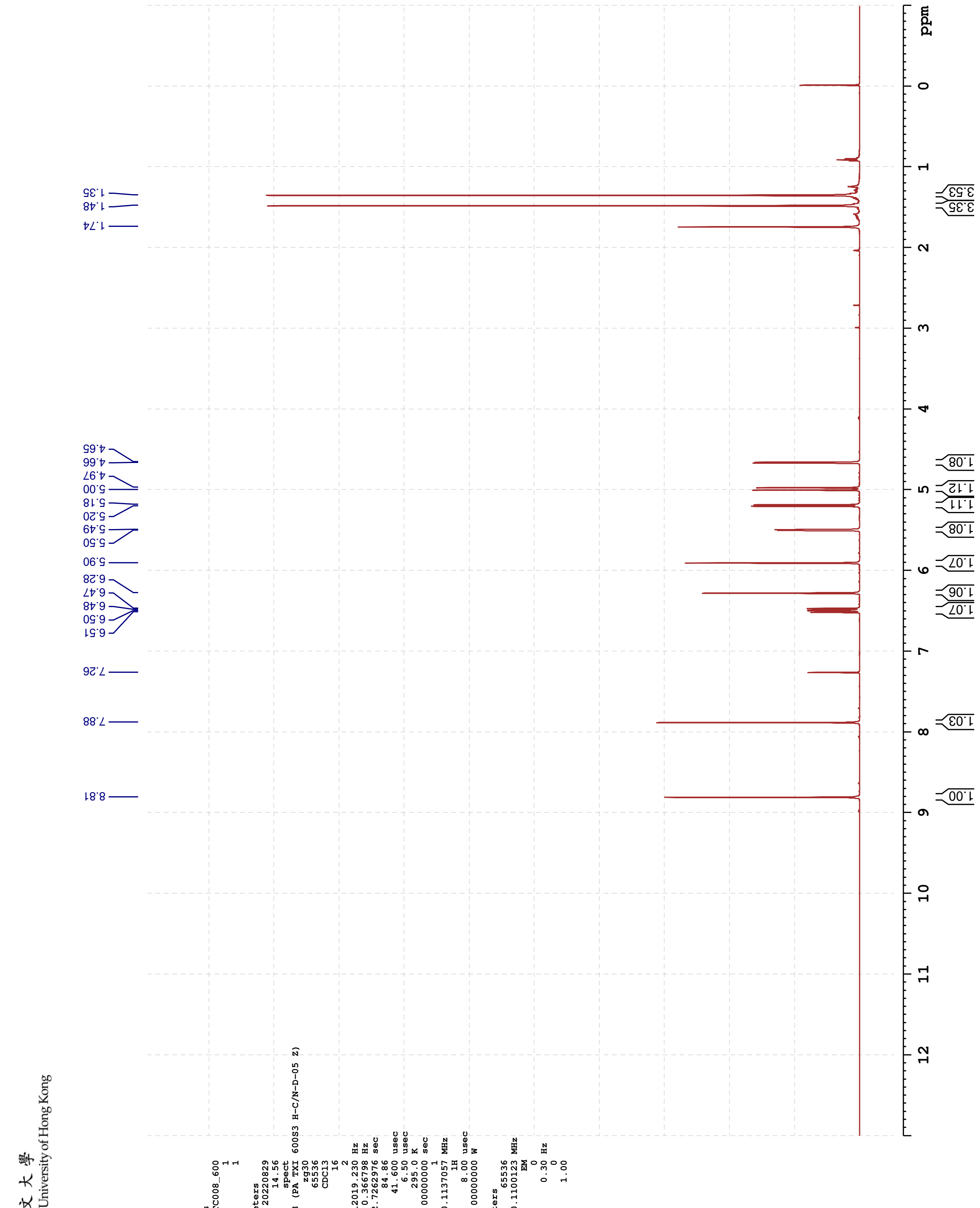

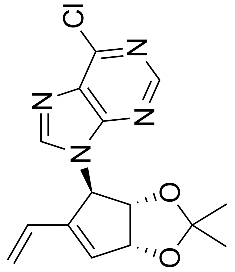

30

NMR-Spectra for Compound 30

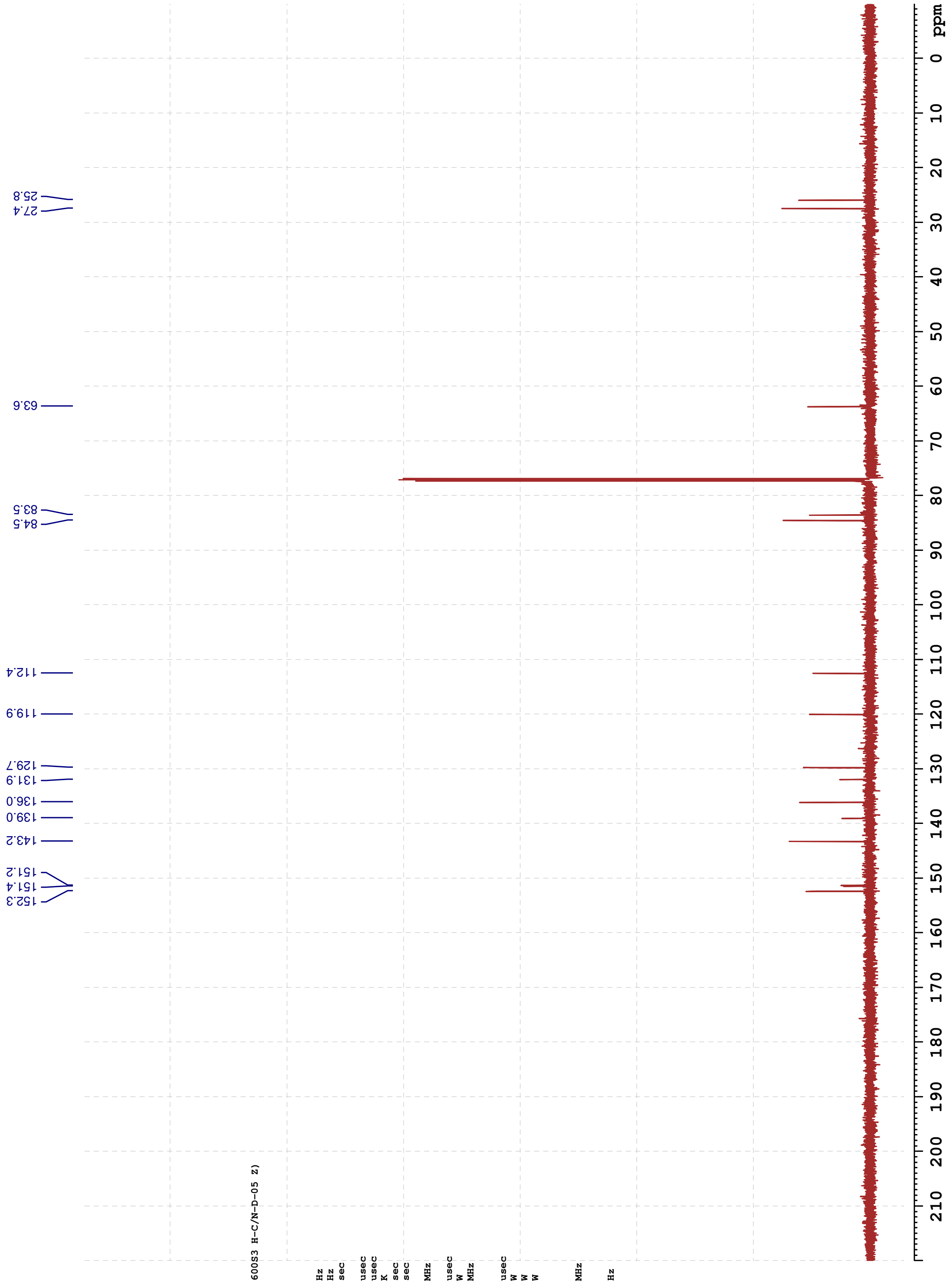

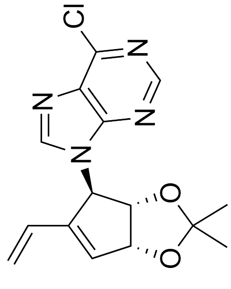

30

NMR-Spectra for Compound 31

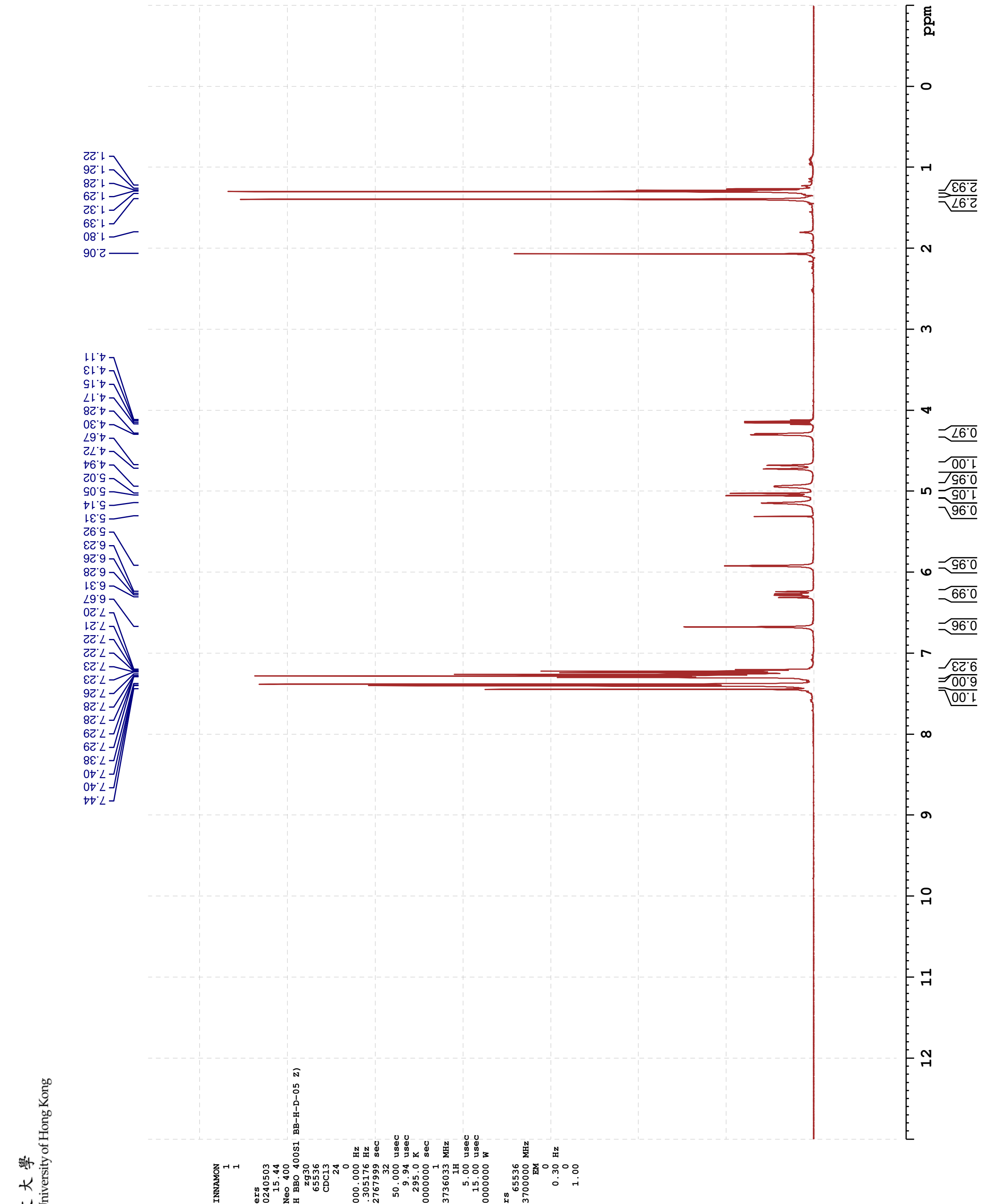

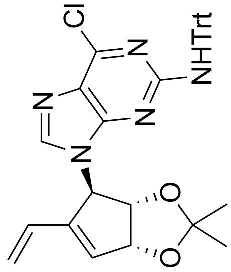

31

NMR-Spectra for Compound 31

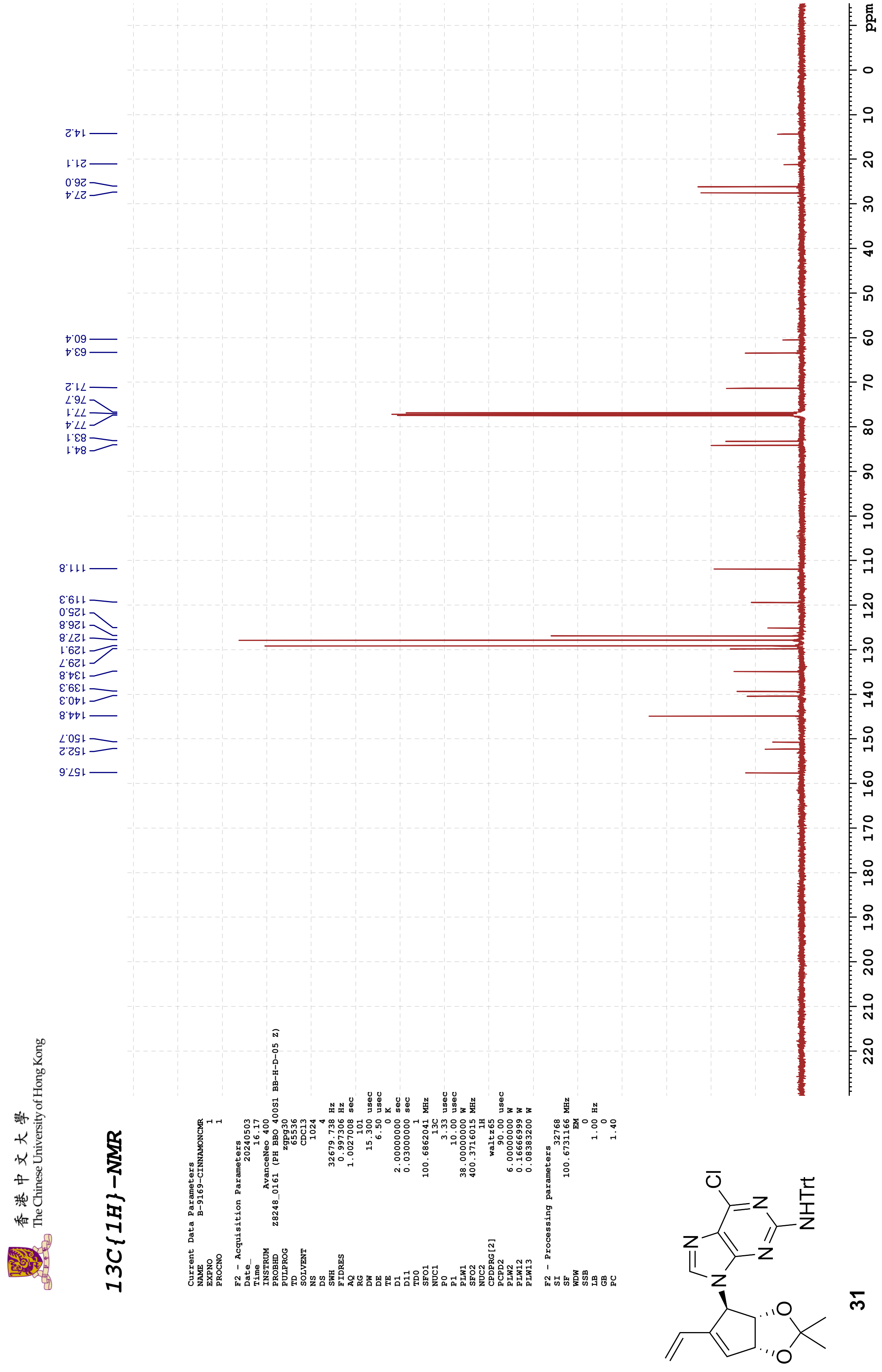

NMR-Spectra for Compound 32a

香港中文大學
The Chinese University of Hong Kong

1H-NMR

```
Current Data Parameters
NAME          B-13604-CINNAMON
EXPNO                        1
PROCNO                       1

F2 - Acquisition Parameters
Date_                 20240705
Time                      0.19
INSTRUM           AvanceNeo 400
PROBHD     Z163739_0214 (PI HR-BBO400S1-BBF/H/D-5.0-Z SP)
PULPROG                   zg30
TD                       65536
SOLVENT                  CDCl3
NS                          16
DS                           0
SWH                  10000.000 Hz
FIDRES                0.305176 Hz
AQ                   3.2767999 sec
RG                          32
DW                      50.000 usec
DE                        9.94 usec
TE                       296.3 K
D1                  1.00000000 sec
TD0                          1
SFO1               400.3736033 MHz
NUC1                        1H
P0                        5.00 usec
P1                       15.00 usec
PLW1                6.00000000 W

F2 - Processing parameters
SI                       65536
SF                 400.3700000 MHz
WDW                         EM
SSB                          0
LB                        0.30 Hz
GB                           0
PC                        1.00
```

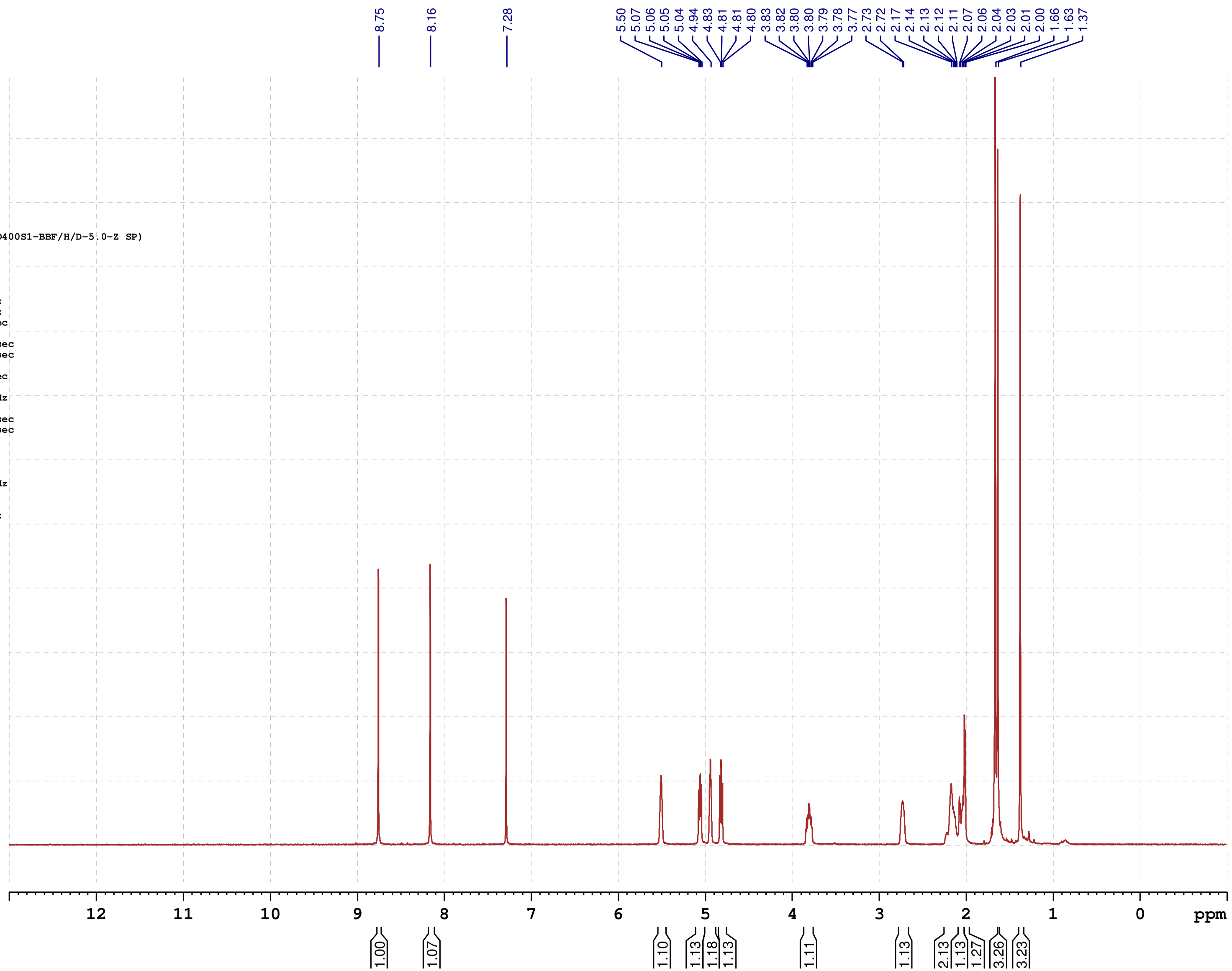

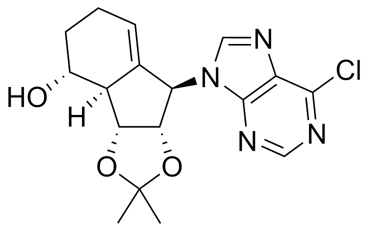

32a

NMR-Spectra for Compound 32a

香港中文大學 The Chinese University of Hong Kong

13C{1H}-NMR

```
Current Data Parameters
NAME              9462-CINNAMON
EXPNO                         1
PROCNO                        1

F2 - Acquisition Parameters
Date_                  20240705
Time                      21.50
INSTRUM     AvanceNeo  Ascend400MHz
PROBHD      Z163739_0198 (PI HR-BBO400S1-BBF/H/D-5.0-Z SP)
PULPROG                 zgpg30
TD                        65536
SOLVENT                   CDCl3
NS                         2000
DS                            0
SWH                  32679.738 Hz
FIDRES                0.997306 Hz
AQ                   1.0027008 sec
RG                          101
DW                       15.300 usec
DE                         6.50 usec
TE                        294.0 K
D1                   2.00000000 sec
D11                  0.03000000 sec
TD0                           1
SFO1                100.6686003 MHz
NUC1                        13C
P0                         2.67 usec
P1                         8.00 usec
PLW1                95.69300079 W
SFO2                400.3016012 MHz
NUC2                         1H
CPDPRG[2]               waltz65
PCPD2                     90.00 usec
PLW2                24.20299911 W
PLW12                0.19123000 W
PLW13                0.09618900 W

F2 - Processing parameters
SI                        32768
SF                  100.6555151 MHz
WDW                          EM
SSB                           0
LB                         1.00 Hz
GB                            0
PC                         1.40
```

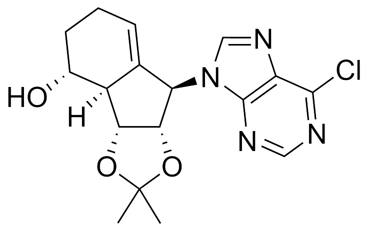

32a

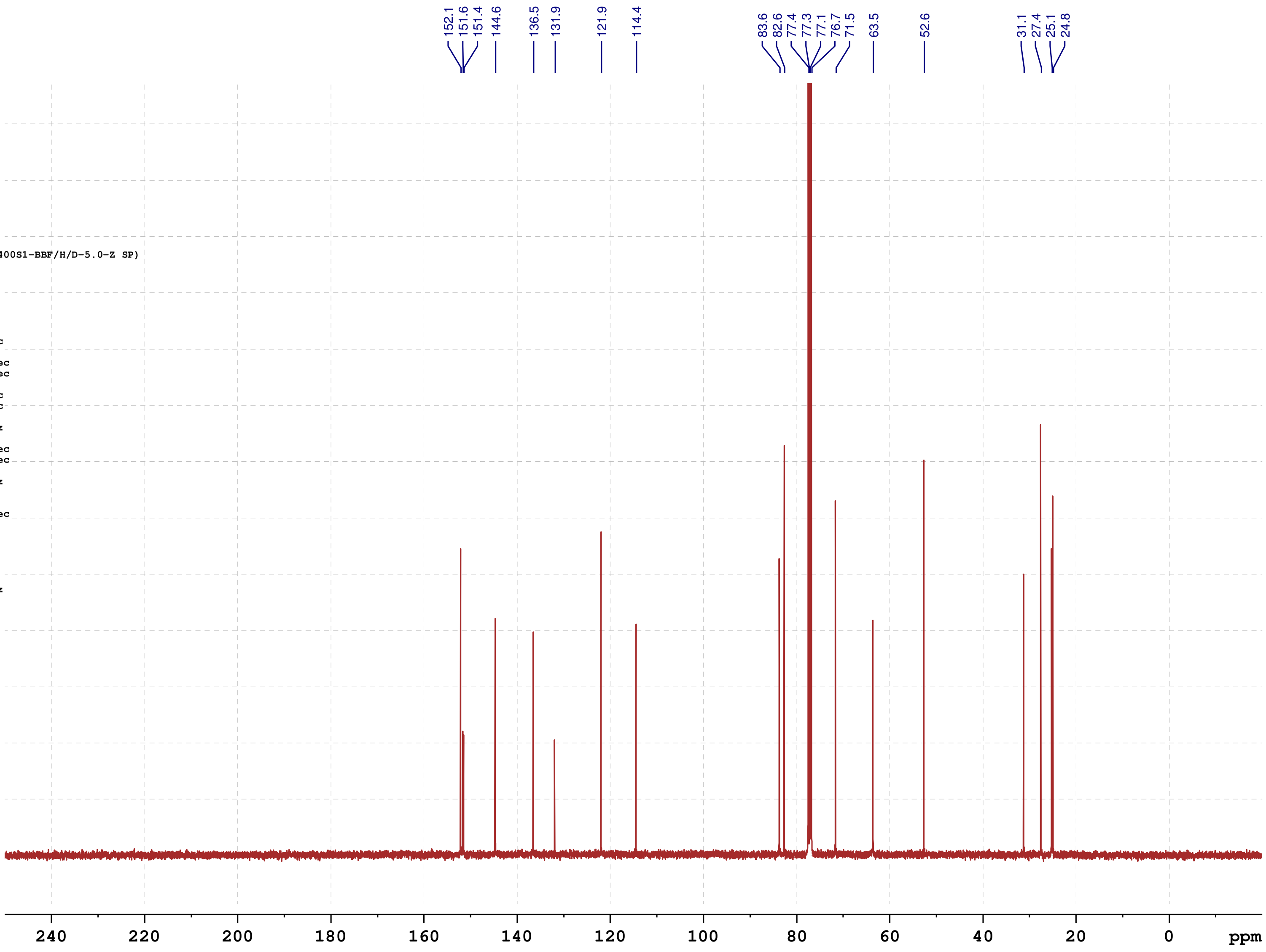

NMR-Spectra for Compound Bz32b

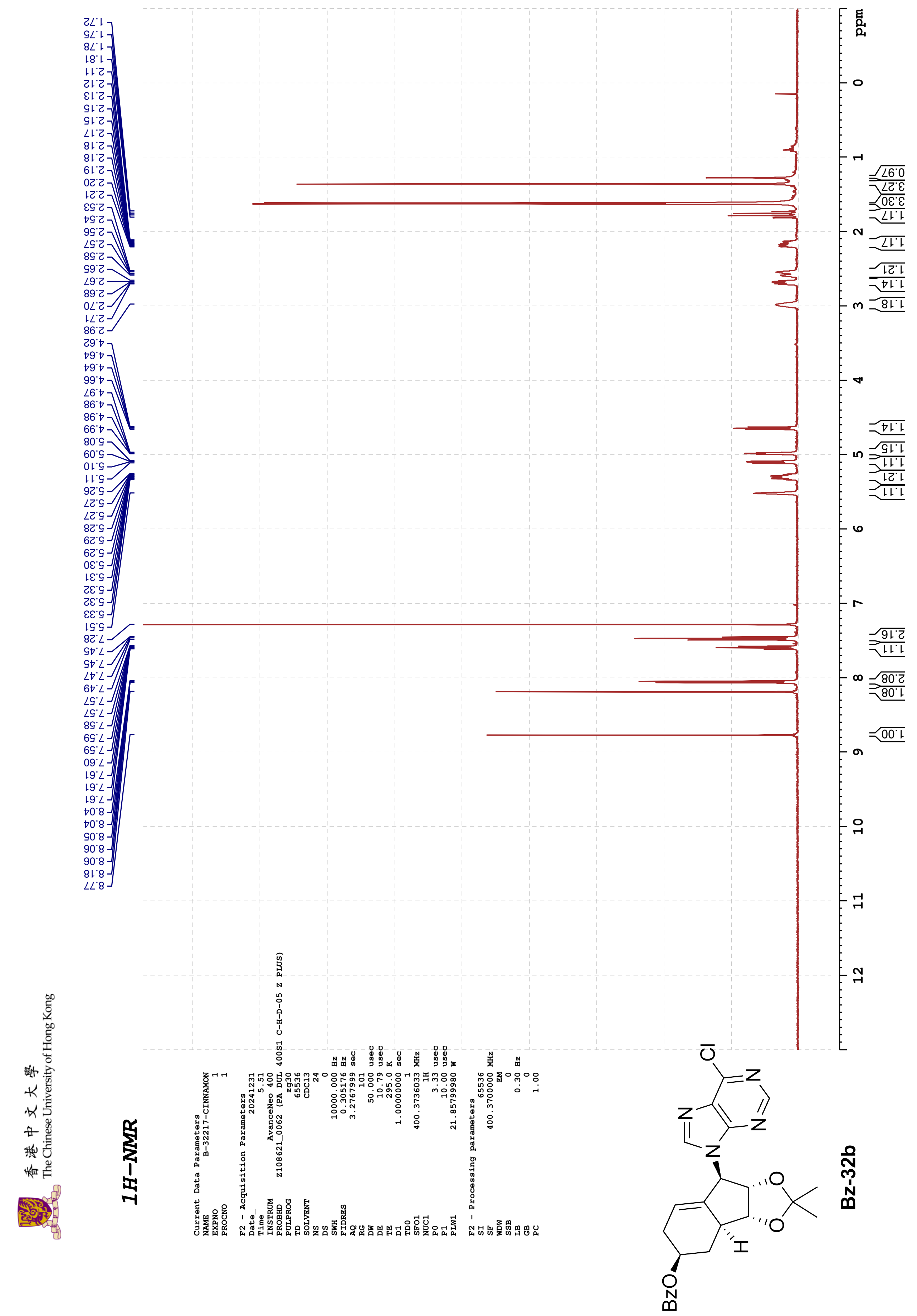

NMR-Spectra for Compound Bz32b

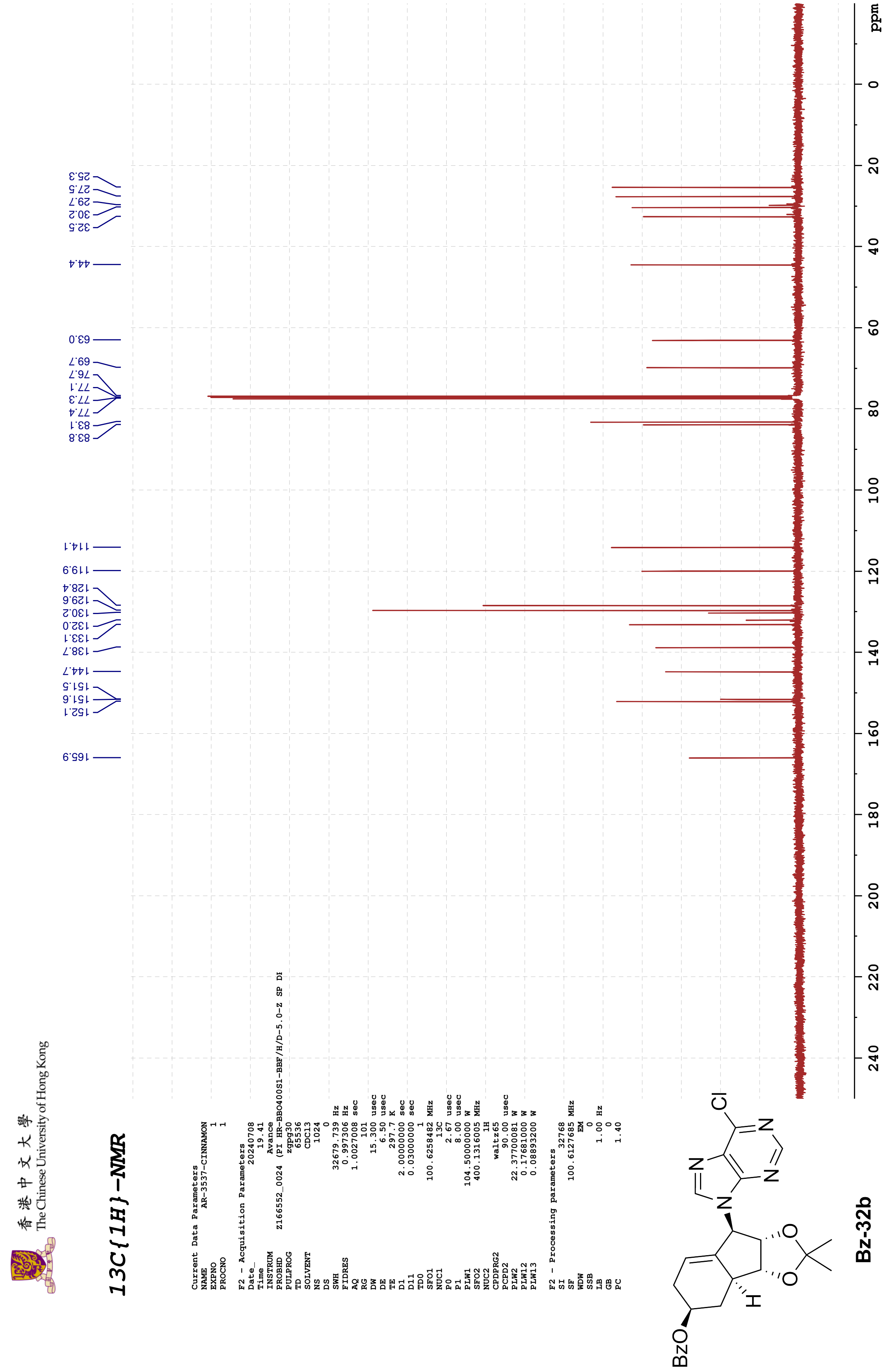

Bz-32b

NMR-Spectra for Compound Bz32c

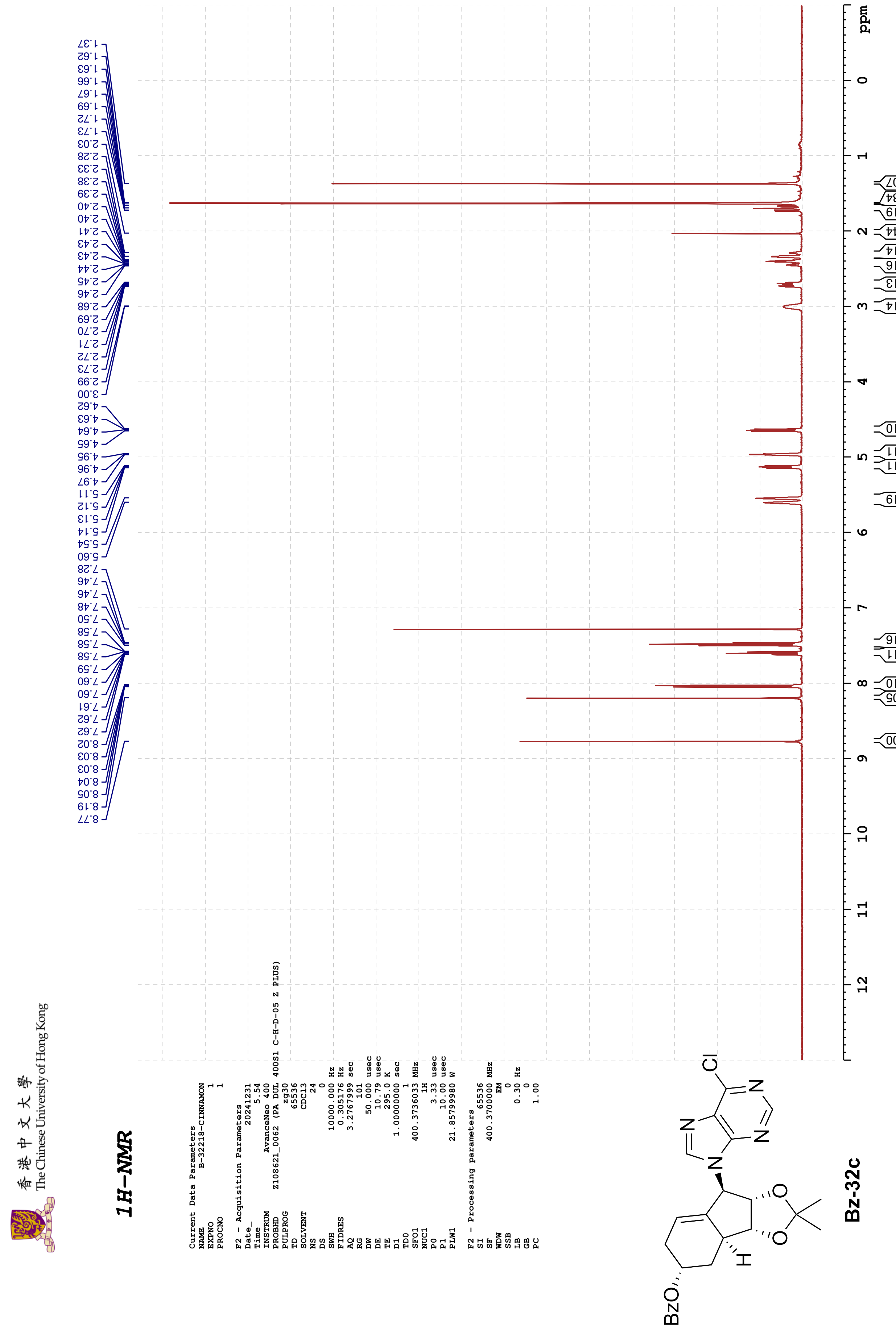

NMR-Spectra for Compound Bz32c

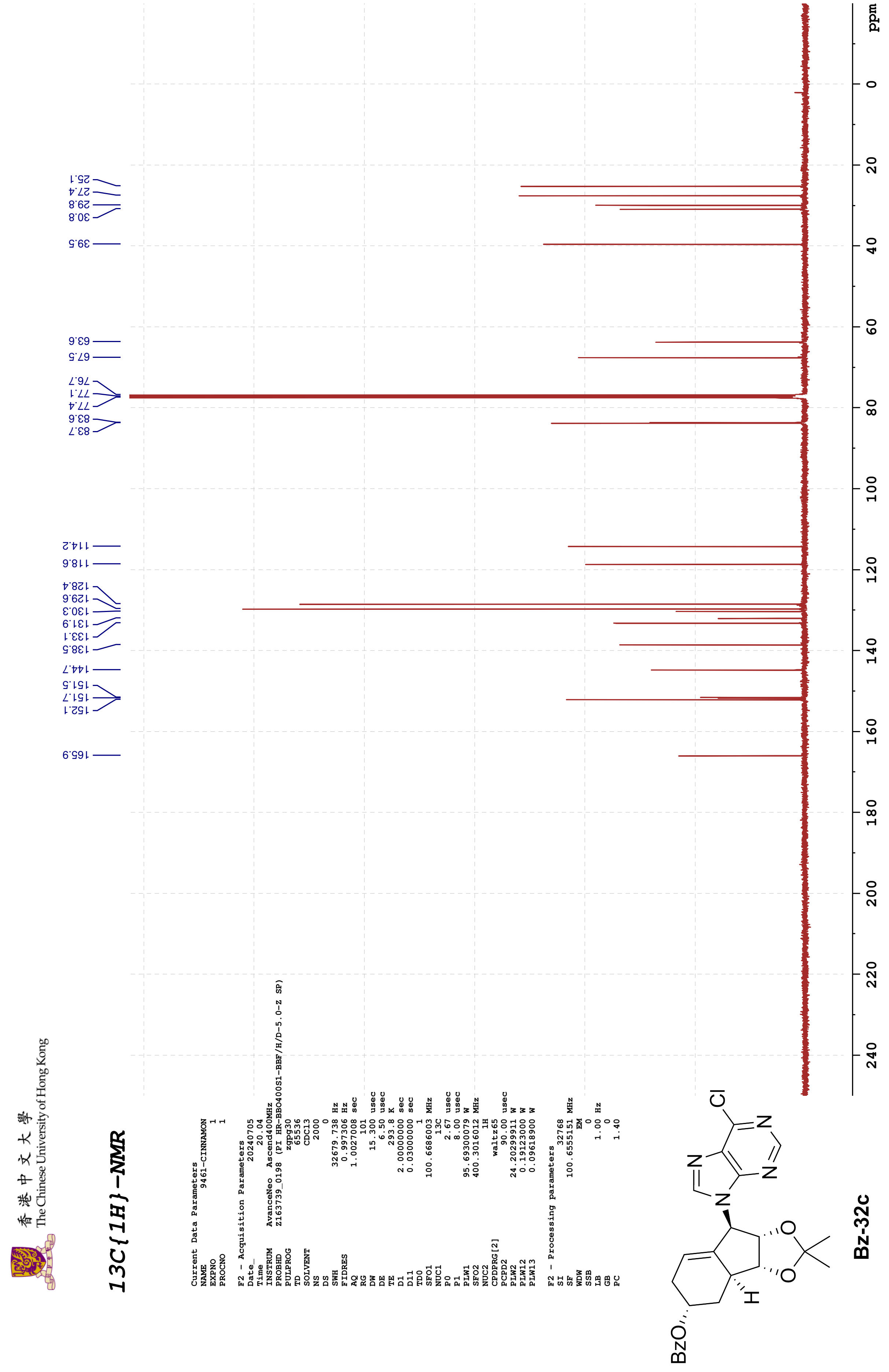

NMR-Spectra for Compound 33a

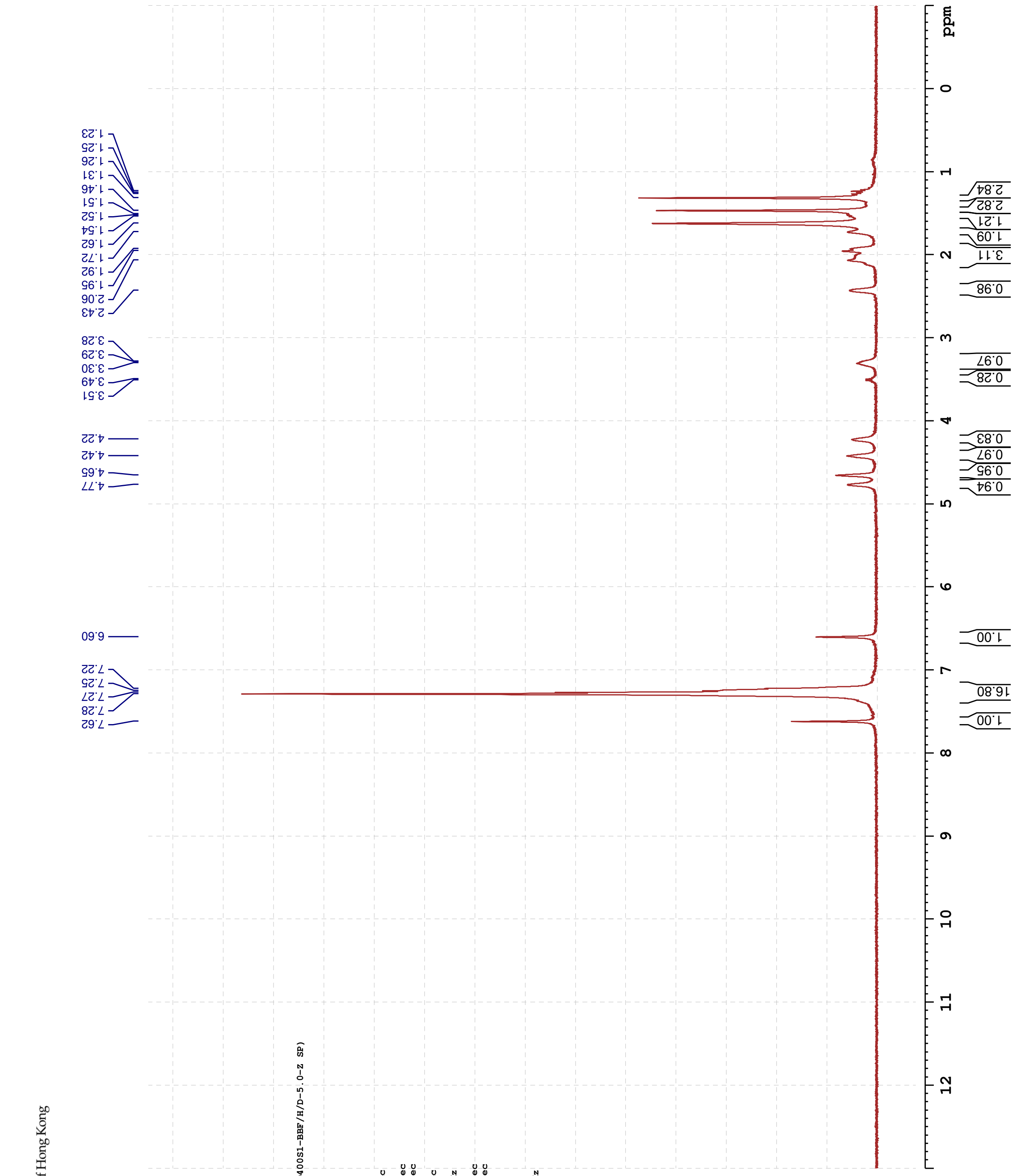

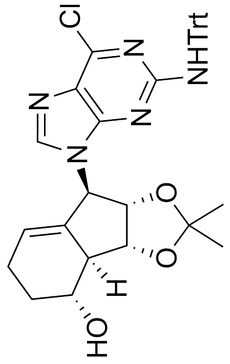

33a

NMR-Spectra for Compound 33a

香港中文大學 The Chinese University of Hong Kong

13C{1H}-NMR

```
Current Data Parameters
NAME          AR-1167-CINNAMONCMR
EXPNO                           1
PROCNO                          1

F2 - Acquisition Parameters
Date_                    20240515
Time                         0.44
INSTRUM                    Avance
PROBHD     Z166552_0024 (PI HR-BBO400S1-BBF/H/D-5.0-Z SP DI
PULPROG                   zgpg30
TD                          65536
SOLVENT                     CDCl3
NS                           2500
DS                              0
SWH                     32679.739 Hz
FIDRES                   0.997306 Hz
AQ                      1.0027008 sec
RG                            101
DW                         15.300 usec
DE                           6.50 usec
TE                          296.8 K
D1                     2.00000000 sec
D11                    0.03000000 sec
TD0                             1
SFO1                  100.6258482 MHz
NUC1                          13C
P0                           2.67 usec
P1                           8.00 usec
PLW1                 104.50000000 W
SFO2                  400.1316005 MHz
NUC2                           1H
CPDPRG2                   waltz65
PCPD2                       90.00 usec
PLW2                  22.37700081 W
PLW12                  0.17681000 W
PLW13                  0.08893200 W

F2 - Processing parameters
SI                          32768
SF                    100.6127685 MHz
WDW                            EM
SSB                             0
LB                           1.00 Hz
GB                              0
PC                           1.40
```

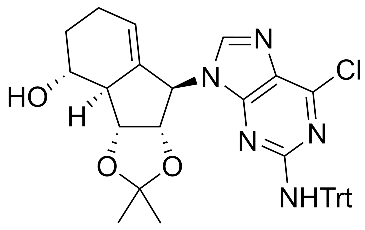

33a

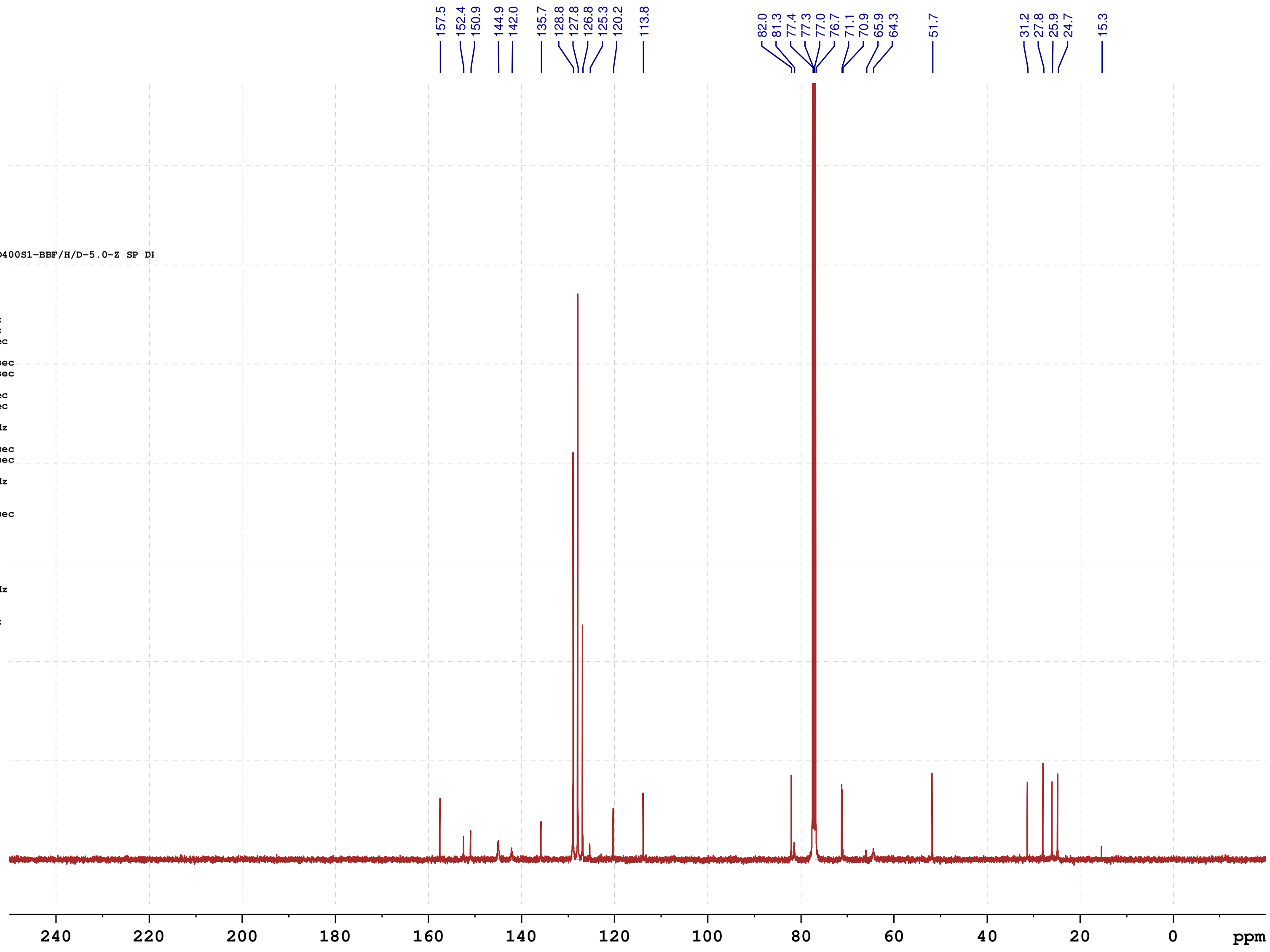

NMR-Spectra for Compound Bz33b

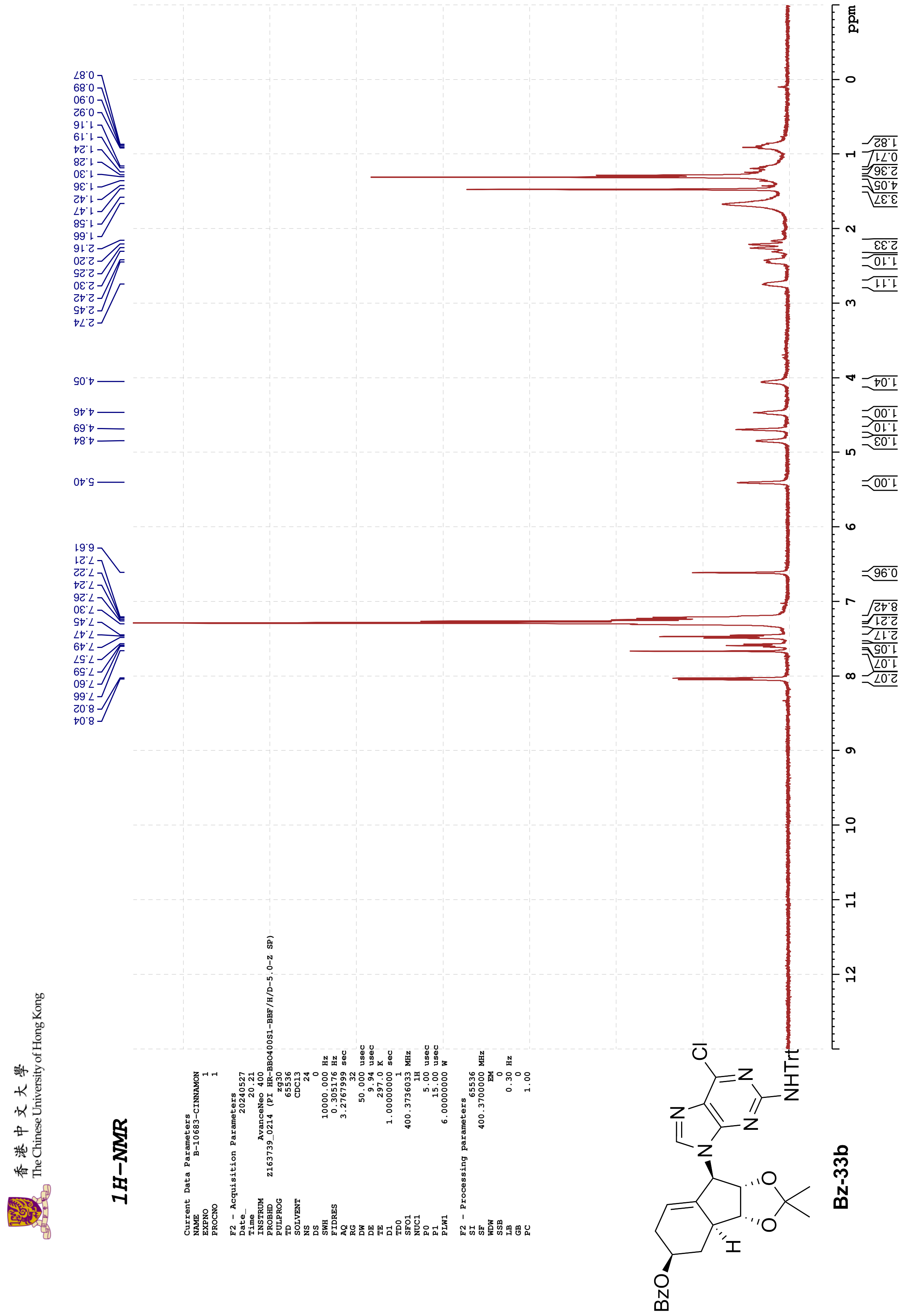

NMR-Spectra for Compound Bz33b

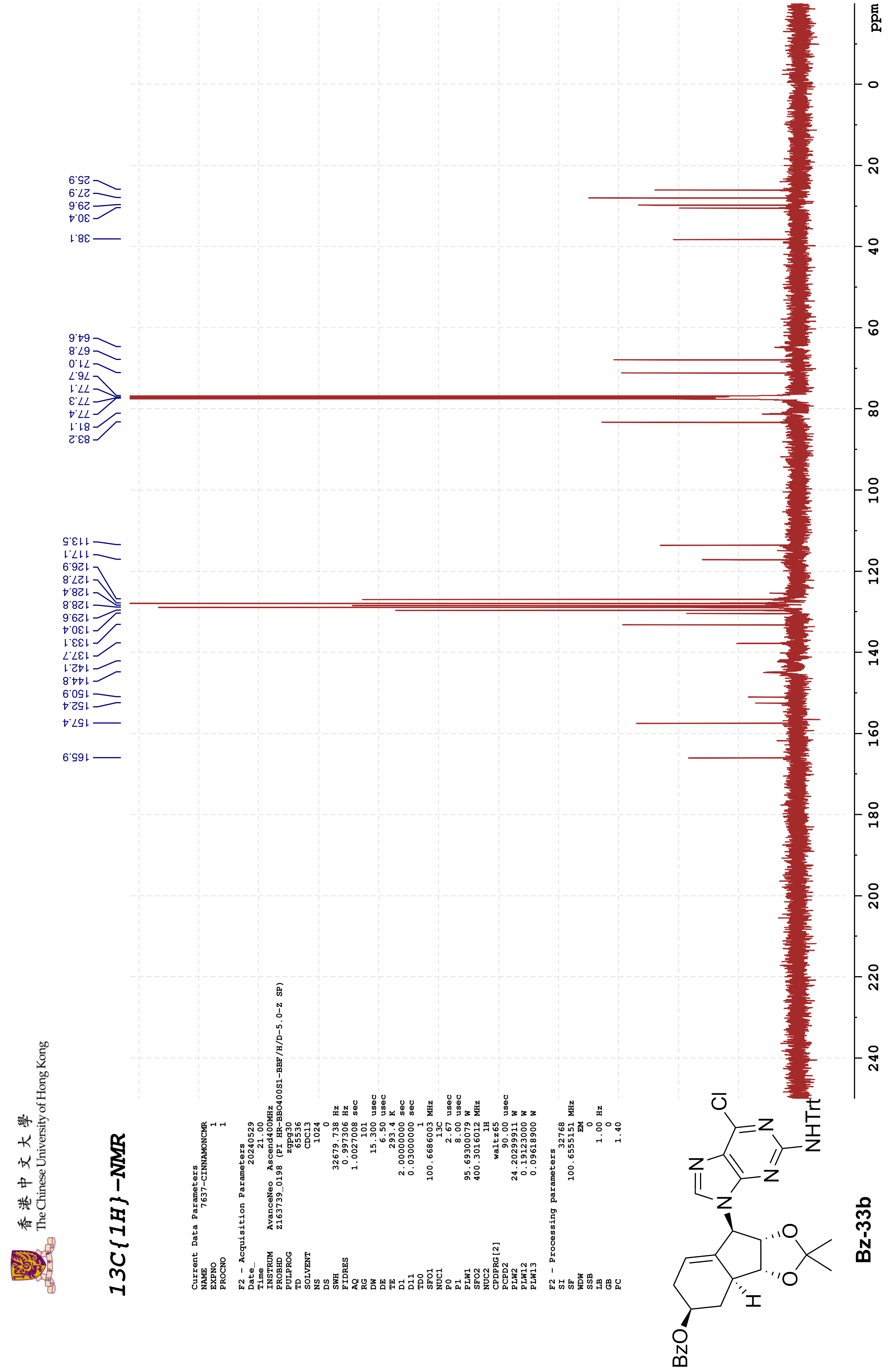

NMR-Spectra for Compound Bz33c

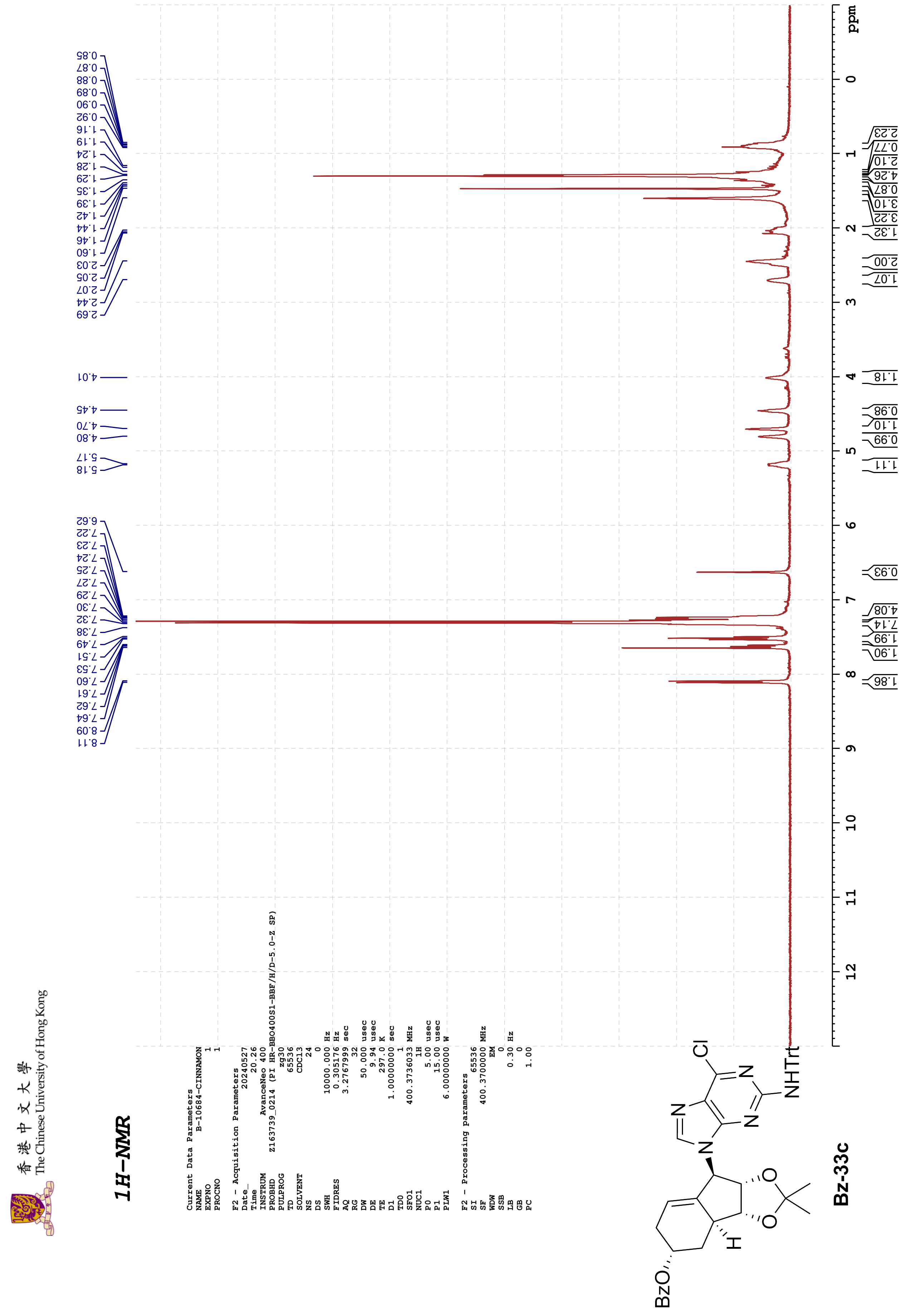

NMR-Spectra for Compound Bz33c

香港中文大學
The Chinese University of Hong Kong

13C{1H}-NMR

```
Current Data Parameters
NAME          B-10718-CINNAMONCMR
EXPNO                          1
PROCNO                         1

F2 - Acquisition Parameters
Date_                   20240528
Time                       12.05
INSTRUM           AvanceNeo 400
PROBHD      Z163739_0214 (PI HR-BBO400S1-BBF/H/D-5.0-Z SP)
PULPROG                  zgpg30
TD                        65536
SOLVENT                   CDCl3
NS                         2000
DS                            0
SWH                   32679.738 Hz
FIDRES                 0.997306 Hz
AQ                    1.0027008 sec
RG                          101
DW                       15.300 usec
DE                         6.50 usec
TE                        297.3 K
D1                   2.00000000 sec
D11                  0.03000000 sec
TD0                           1
SFO1                100.6862041 MHz
NUC1                        13C
P0                         2.63 usec
P1                         7.90 usec
PLW1               100.30000305 W
SFO2                400.3716015 MHz
NUC2                         1H
CPDPRG[2]               waltz65
PCPD2                     90.00 usec
PLW2                24.89999962 W
PLW12                0.18703000 W
PLW13                0.09407300 W

F2 - Processing parameters
SI                        32768
SF                  100.6731166 MHz
WDW                          EM
SSB                           0
LB                         3.00 Hz
GB                            0
PC                         1.40
```

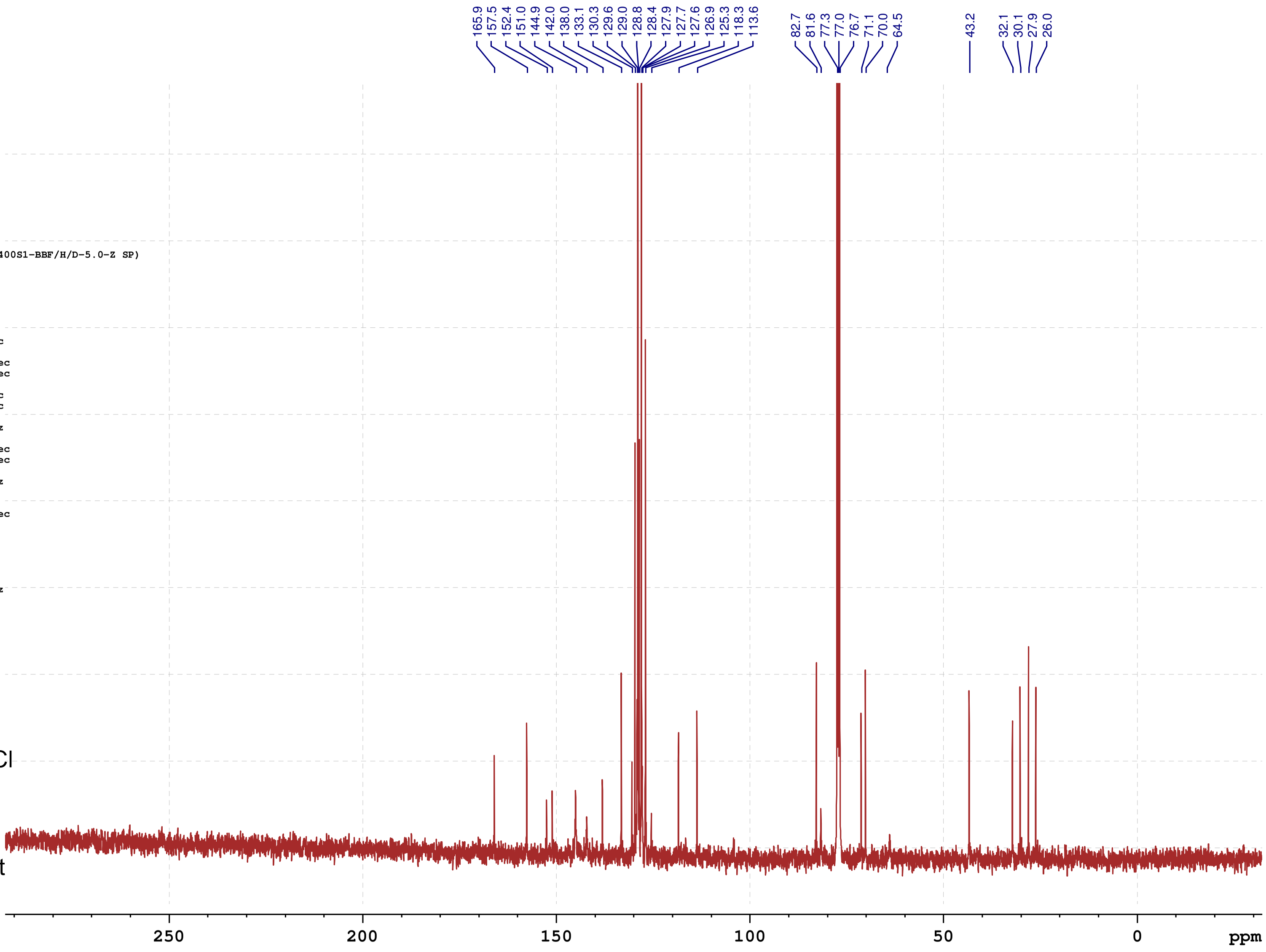

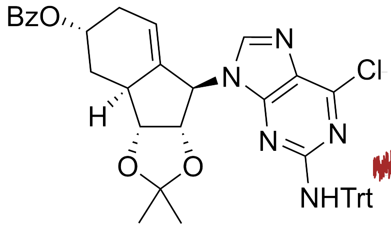

Bz-33c

NMR-Spectra for Compound TBS34a

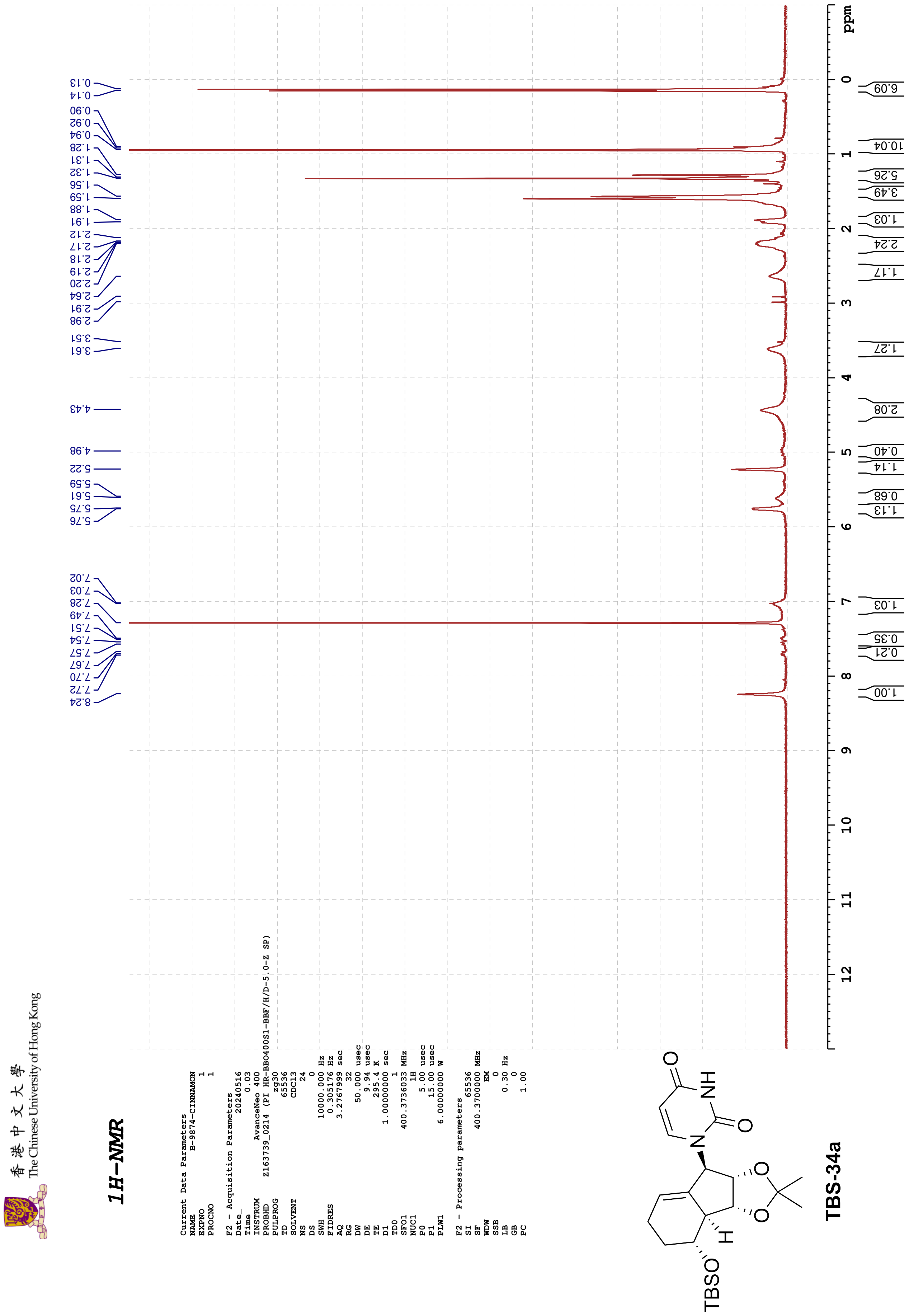

NMR-Spectra for Compound Bz34b

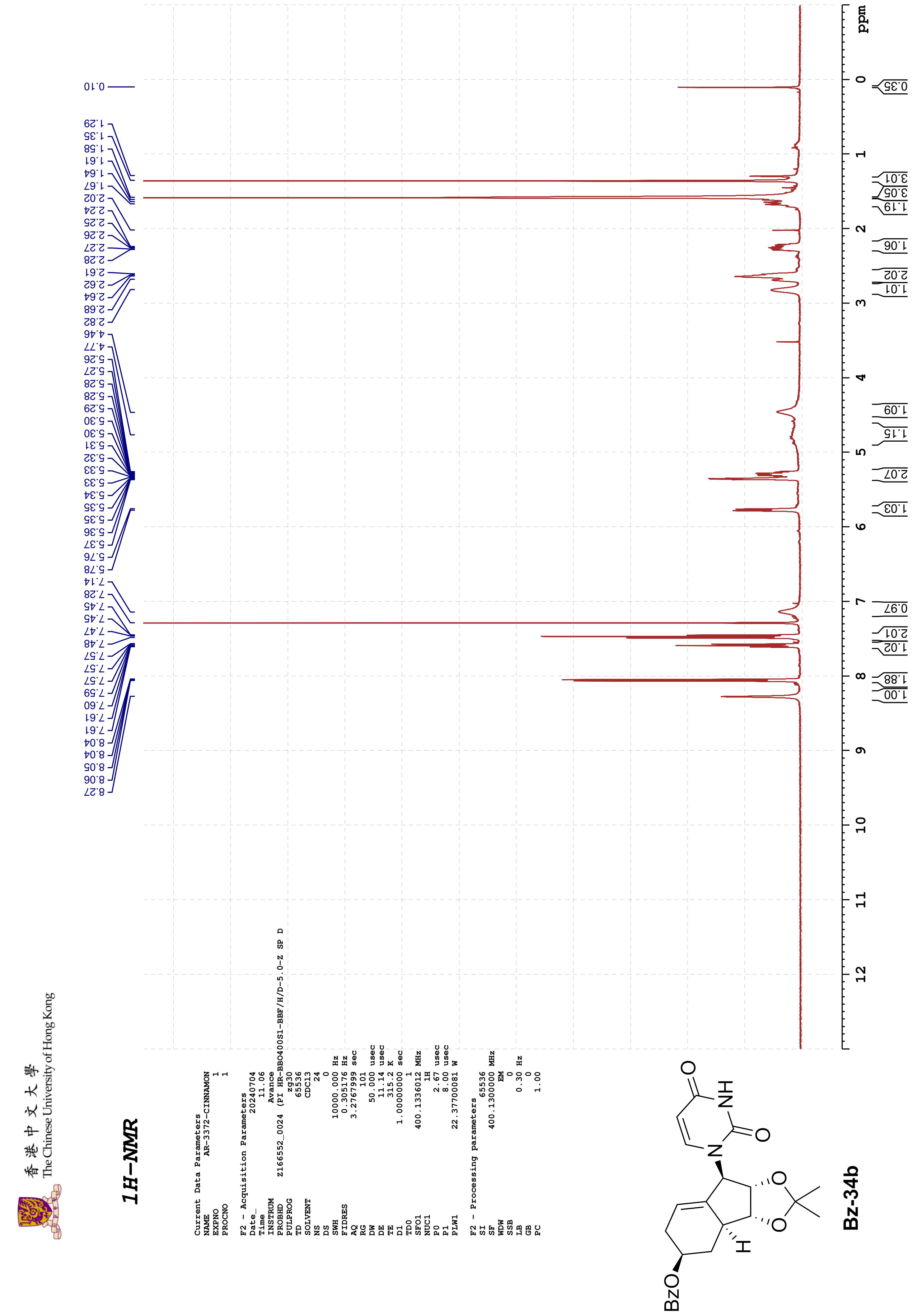

NMR-Spectra for Compound Bz34b

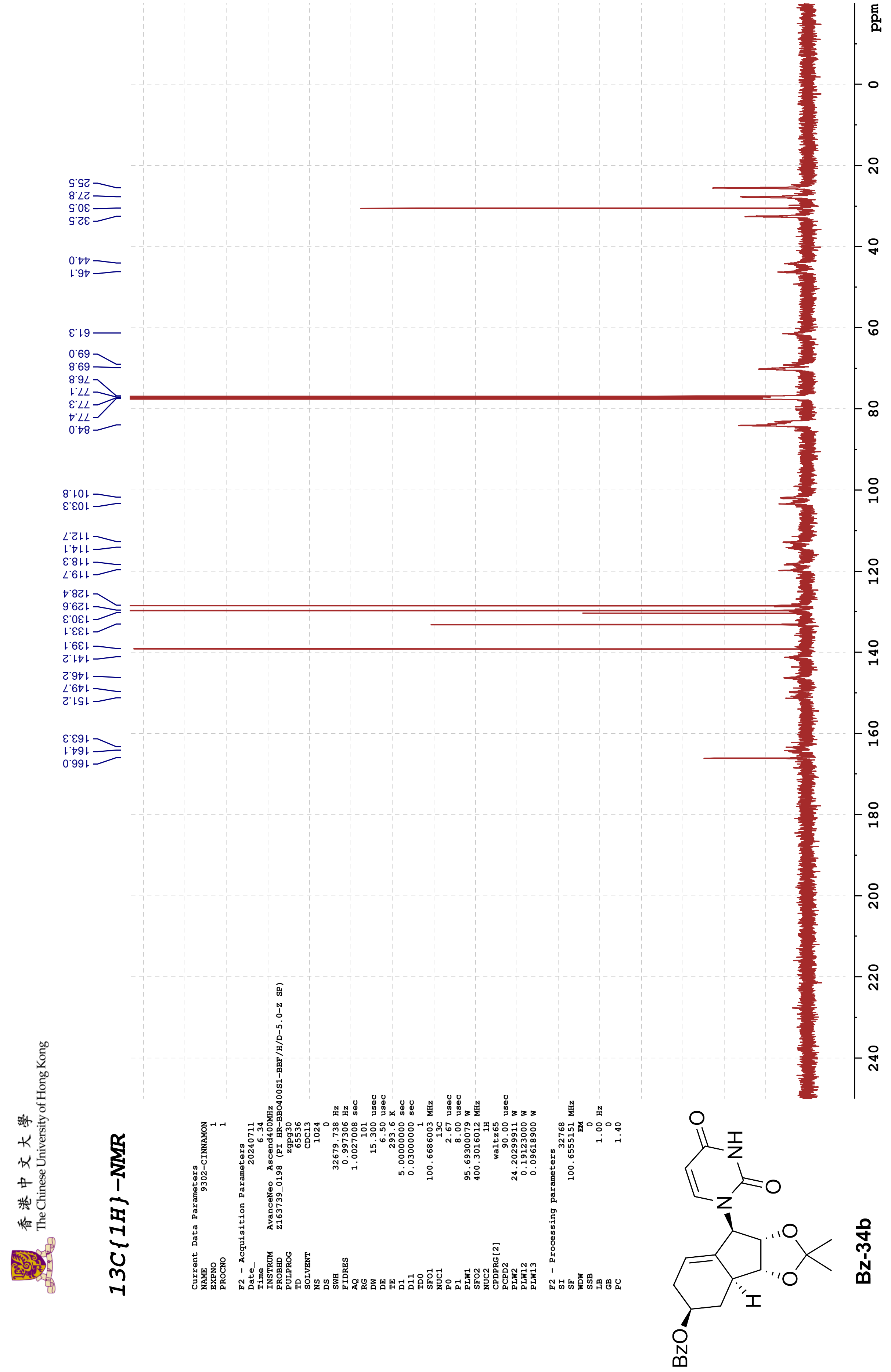

NMR-Spectra for Compound Bz34b

The Chinese University of Hong Kong 香港中文大學

13C{1H}-NMR

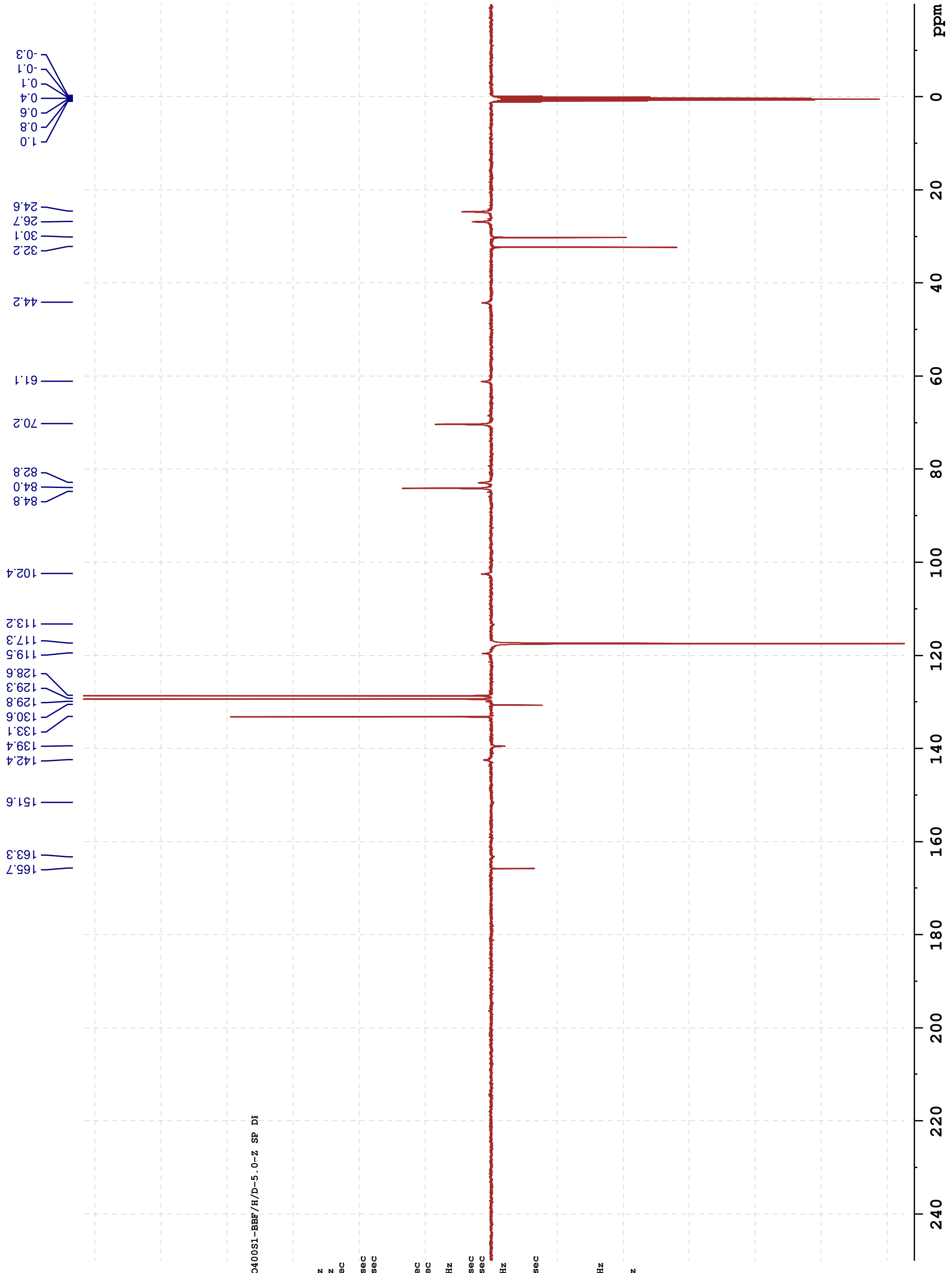


```
Current Data Parameters
NAME        AR-1658-CINNAMONAPT
EXPNO                     1
PROCNO                    1

F2 - Acquisition Parameters
Date_              20240526
Time                   0.49
INSTRUM              Avance
PROBHD     Z166552_0024 (PI HR-BBO400S1-BBF/H/D-5.0-Z SP DI
PULPROG                jmod
TD                    65536
SOLVENT               CD3CN
NS                     2500
DS                        0
SWH               32679.739 Hz
FIDRES             0.997306 Hz
AQ                1.0027008 sec
RG                      101
DW                   15.300 usec
DE                     6.50 usec
TE                    296.6 K
CNST2           145.0000000
CNST11            1.0000000
D1               2.00000000 sec
D20              0.00689655 sec
TD0                       1
SFO1            100.6258482 MHz
NUC1                    13C
P1                     8.00 usec
P2                    16.00 usec
PLW1           104.50000000 W
SFO2            400.1316005 MHz
NUC2                     1H
CPDPRG2             waltz65
PCPD2                 90.00 usec
PLW2            22.37700081 W
PLW12            0.17681000 W

F2 - Processing parameters
SI                    32768
SF              100.6127685 MHz
WDW                      EM
SSB                       0
LB                     2.00 Hz
GB                        0
PC                     1.40
```

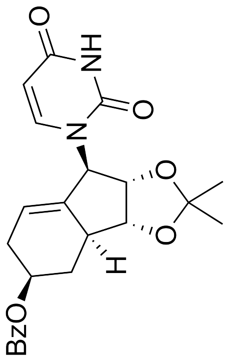

Bz-34b

NMR-Spectra for Compound Bz34c

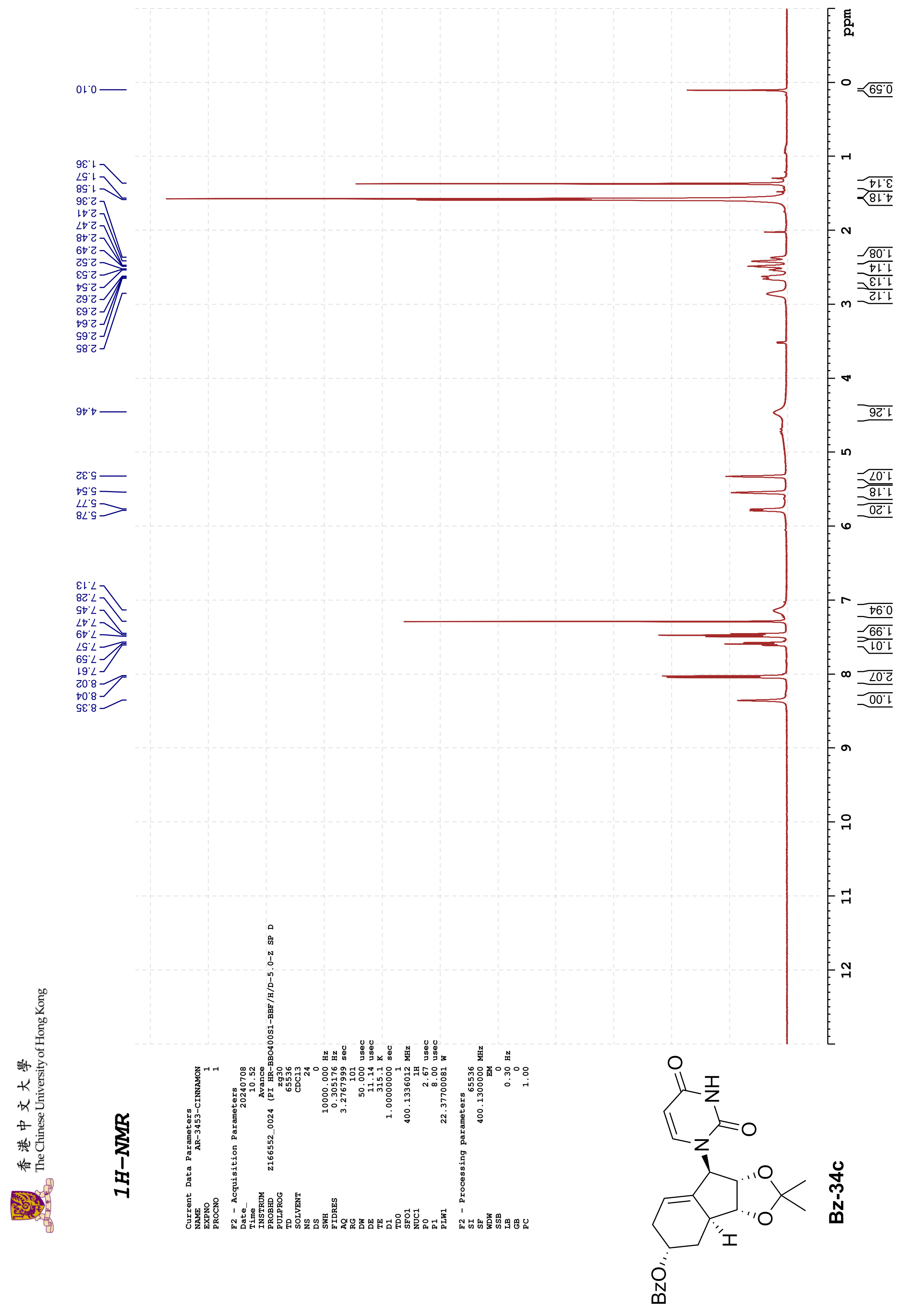

NMR-Spectra for Compound Bz34c

香港中文大學 The Chinese University of Hong Kong

13C{1H}-NMR

```
Current Data Parameters
NAME              9464-CINNAMON
EXPNO                         1
PROCNO                        1

F2 - Acquisition Parameters
Date_                  20240706
Time                       1.23
INSTRUM    AvanceNeo  Ascend400MHz
PROBHD     Z163739_0198 (PI HR-BBO400S1-BBF/H/D-5.0-Z SP)
PULPROG                 zgpg30
TD                       65536
SOLVENT                  CDCl3
NS                        2000
DS                           0
SWH                  32679.738 Hz
FIDRES                0.997306 Hz
AQ                   1.0027008 sec
RG                         101
DW                      15.300 usec
DE                        6.50 usec
TE                       293.7 K
D1                  2.00000000 sec
D11                 0.03000000 sec
TD0                          1
SFO1               100.6686003 MHz
NUC1                       13C
P0                        2.67 usec
P1                        8.00 usec
PLW1               95.69300079 W
SFO2               400.3016012 MHz
NUC2                        1H
CPDPRG[2]              waltz65
PCPD2                    90.00 usec
PLW2               24.20299911 W
PLW12               0.19123000 W
PLW13               0.09618900 W

F2 - Processing parameters
SI                       32768
SF                 100.6555151 MHz
WDW                         EM
SSB                          0
LB                        1.00 Hz
GB                           0
PC                        1.40
```

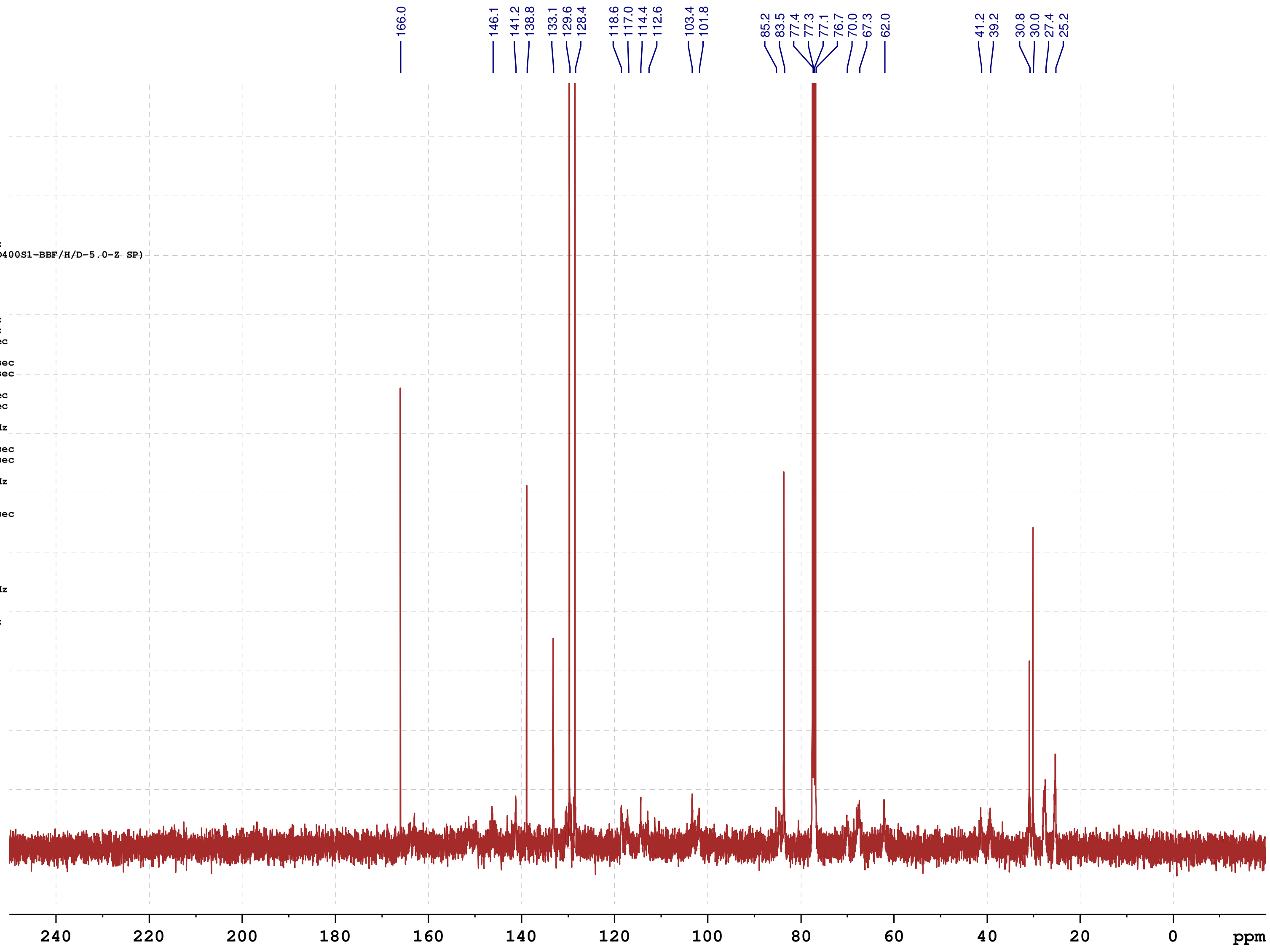

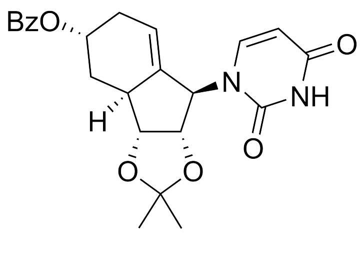

Bz-34c

NMR-Spectra for Compound Bz34c

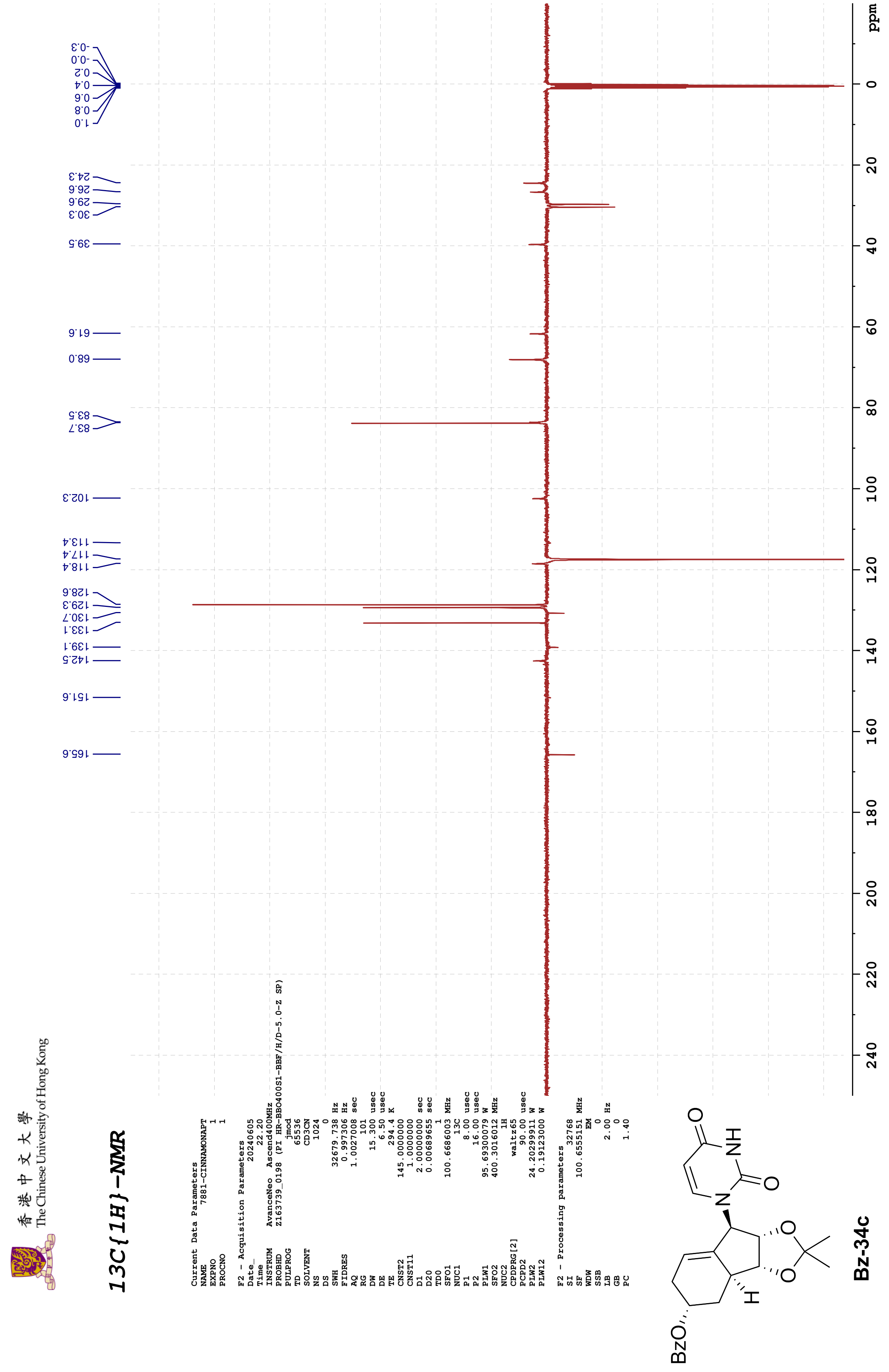

NMR-Spectra for Compound 36

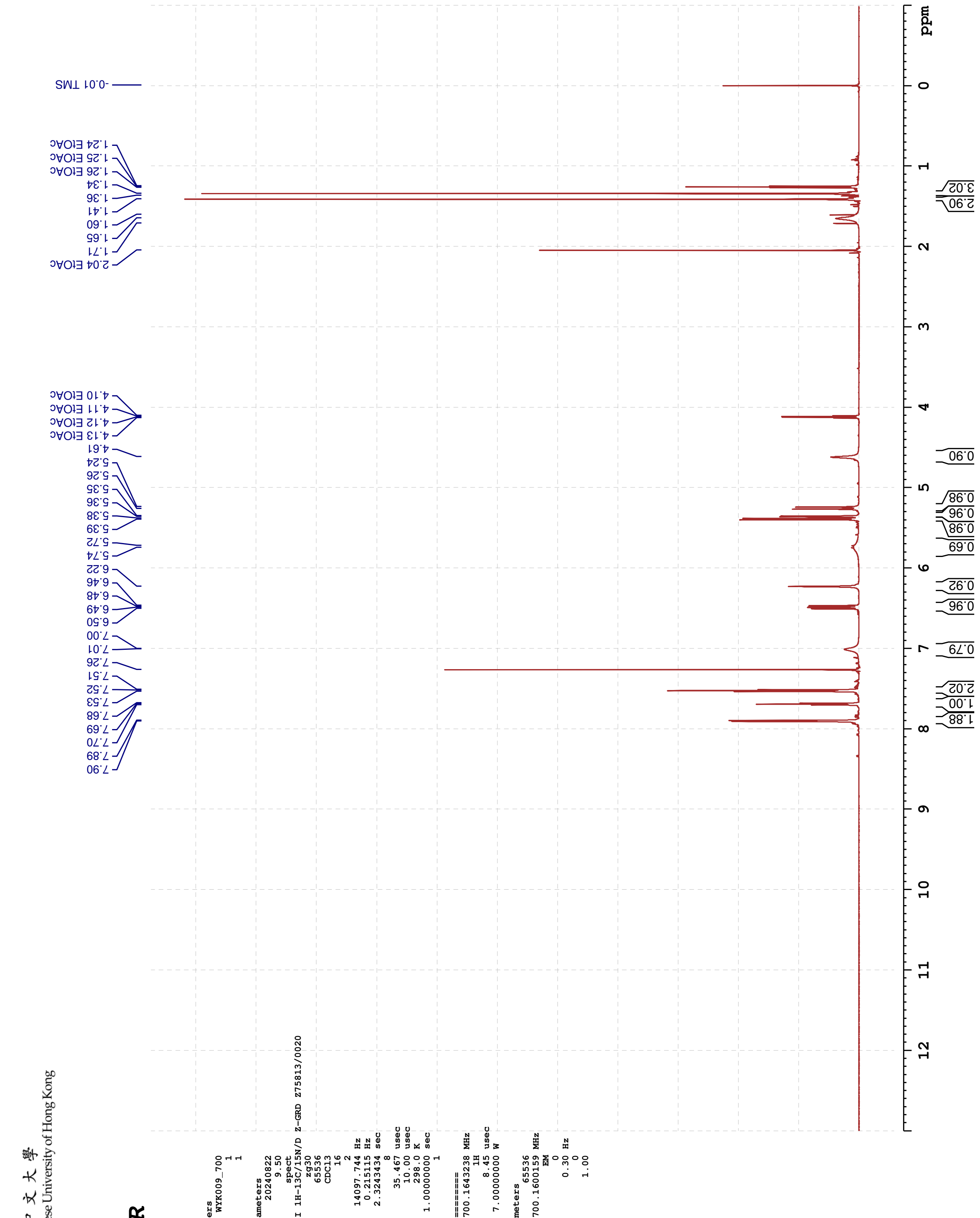

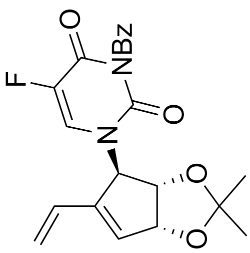

36

NMR-Spectra for Compound 36

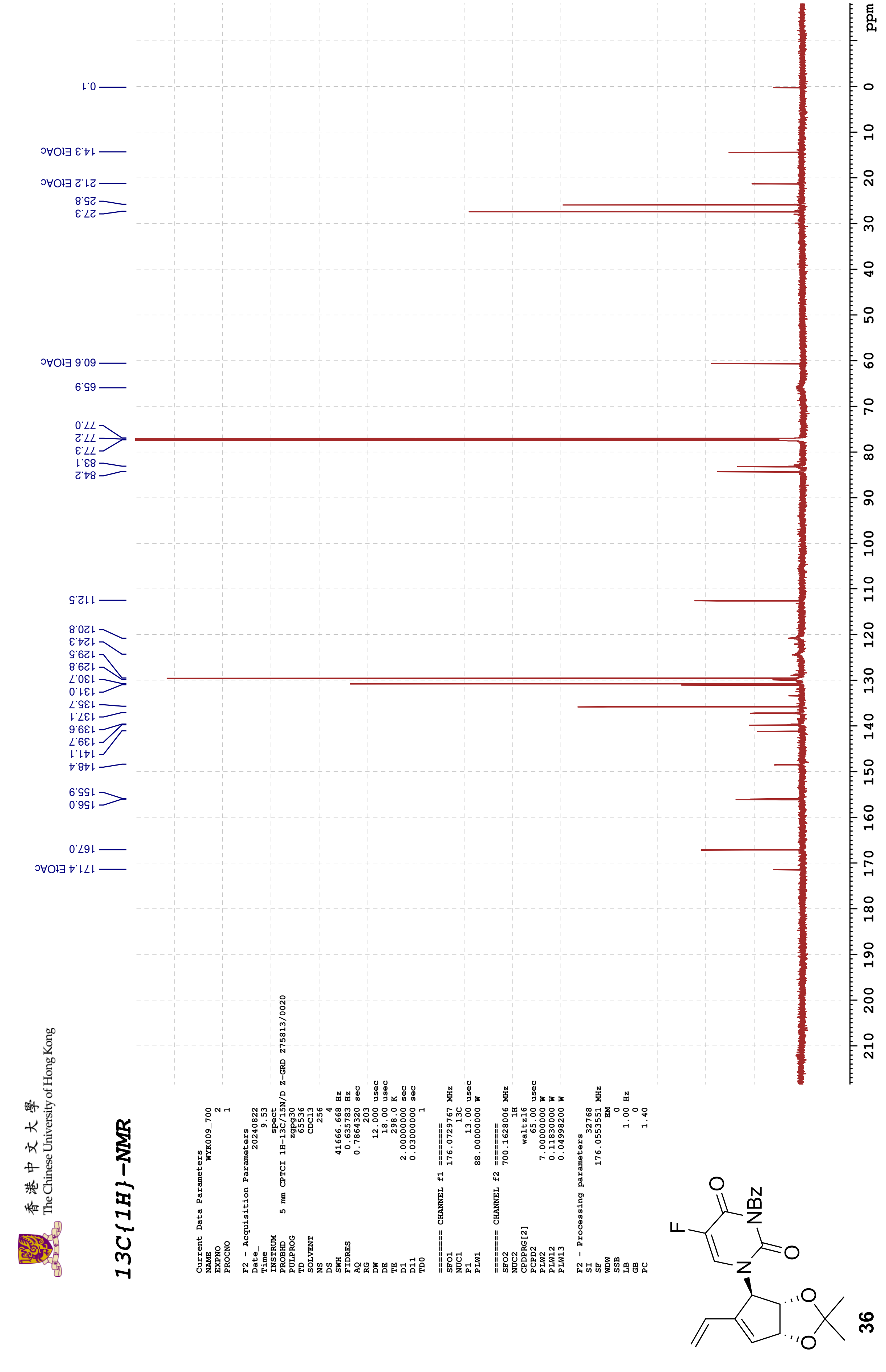

NMR-Spectra for Compound 37

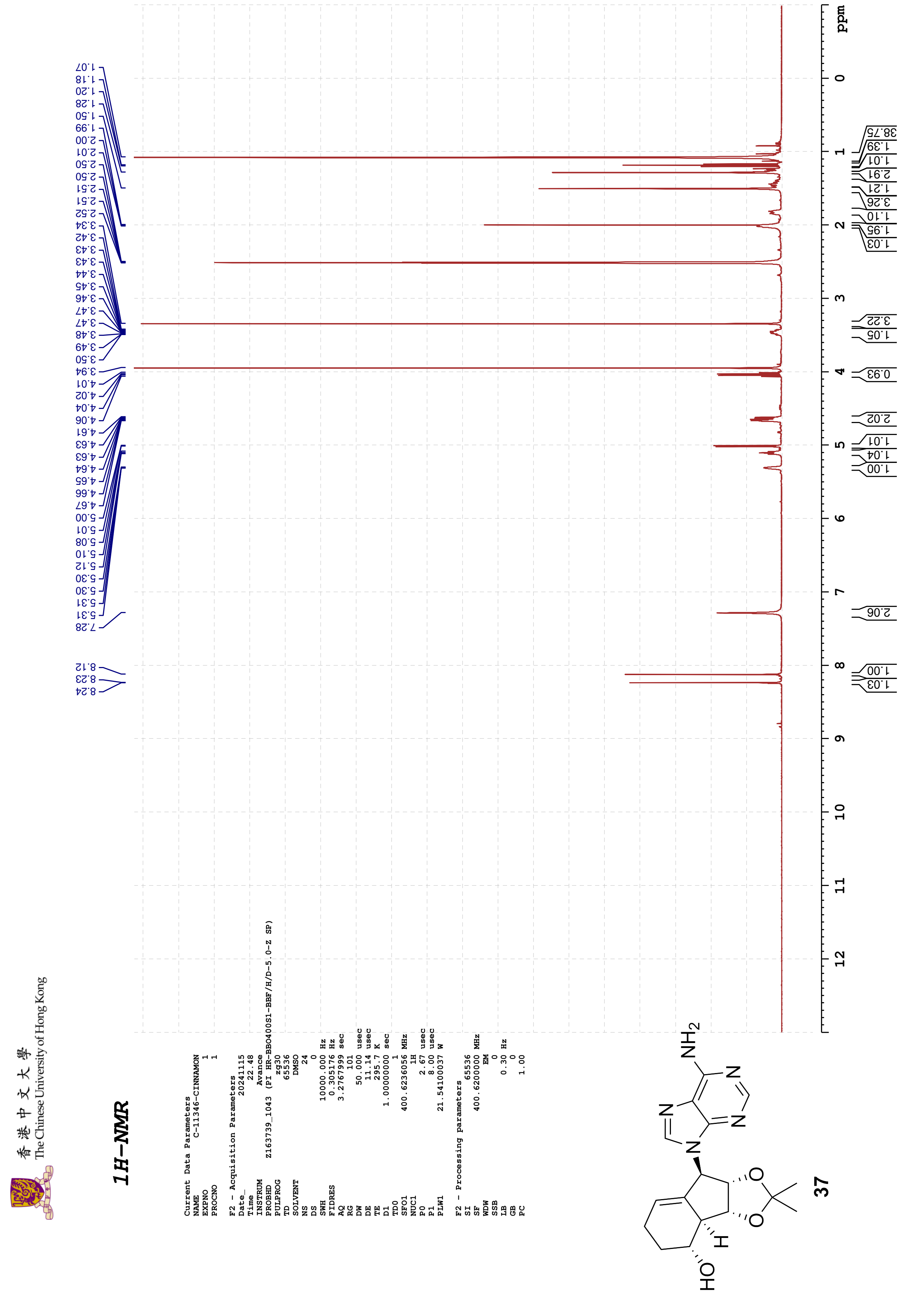

37

NMR-Spectra for Compound 37

香港中文大學
The Chinese University of Hong Kong

13C{1H}-NMR

```
Current Data Parameters
NAME        C-8504-CINNAMON13C
EXPNO                        1
PROCNO                       1

F2 - Acquisition Parameters
Date_                 20241019
Time                     11.00
INSTRUM                 Avance
PROBHD     Z163739_0989 (PI HR-BBO400S1-BBF/H/D-5.0-Z SP)
PULPROG                zgpg30
TD                      65536
SOLVENT                  DMSO
NS                       1200
DS                          0
SWH                 32679.739 Hz
FIDRES               0.997306 Hz
AQ                  1.0027008 sec
RG                       12.7
DW                     15.300 usec
DE                       6.50 usec
TE                      295.0 K
D1                 2.00000000 sec
D11                0.03000000 sec
TD0                         1
SFO1              100.7490748 MHz
NUC1                      13C
P0                       2.67 usec
P1                       8.00 usec
PLW1              94.87300110 W
SFO2              400.6216025 MHz
NUC2                       1H
CPDPRG2               waltz65
PCPD2                   90.00 usec
PLW2              21.67200089 W
PLW12              0.17124000 W
PLW13              0.08613100 W

F2 - Processing parameters
SI                      32768
SF                100.7359791 MHz
WDW                        EM
SSB                         0
LB                       1.00 Hz
GB                          0
PC                       1.40
```

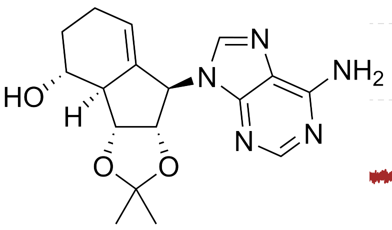

37

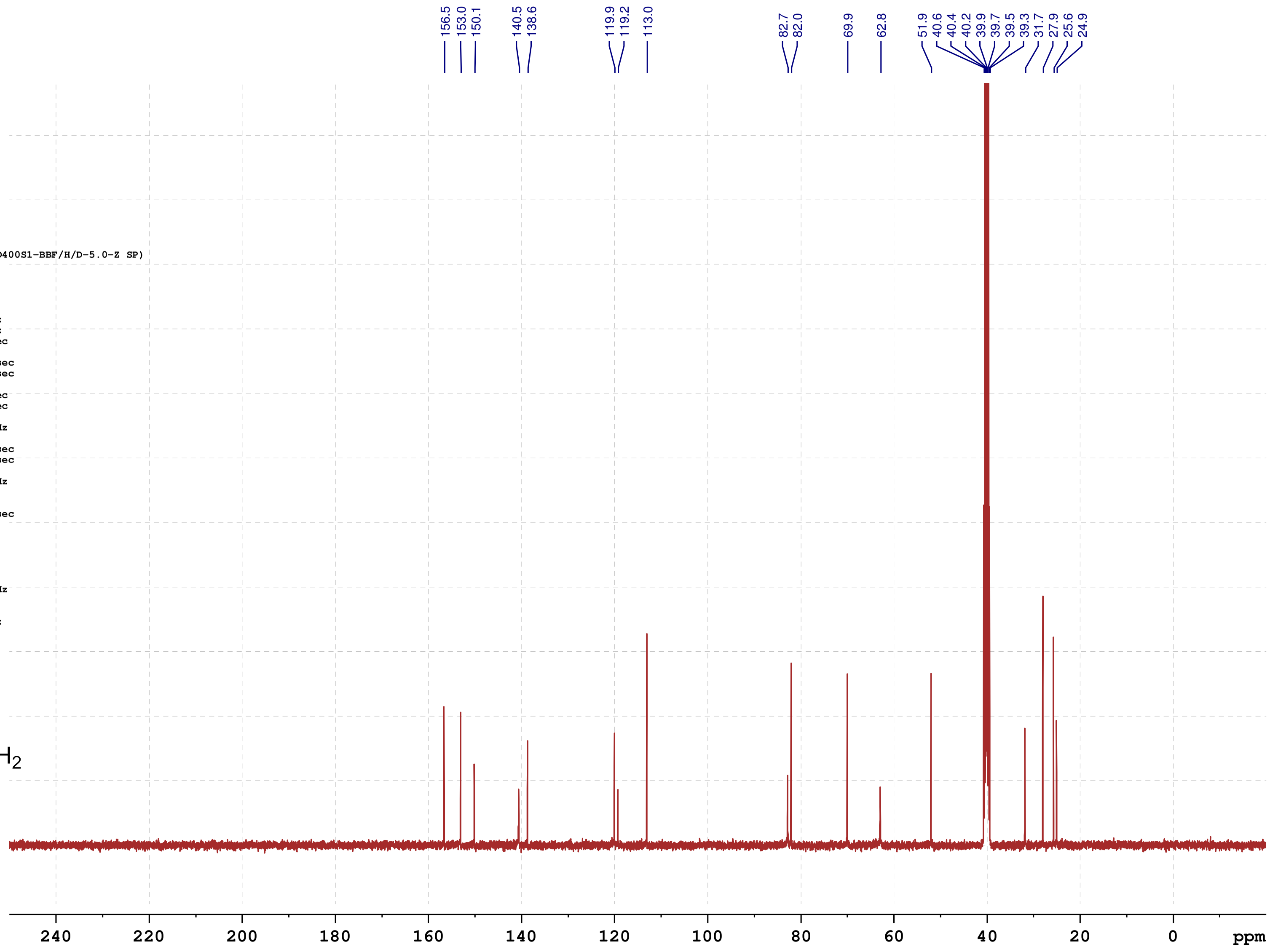

NMR-Spectra for Compound 47

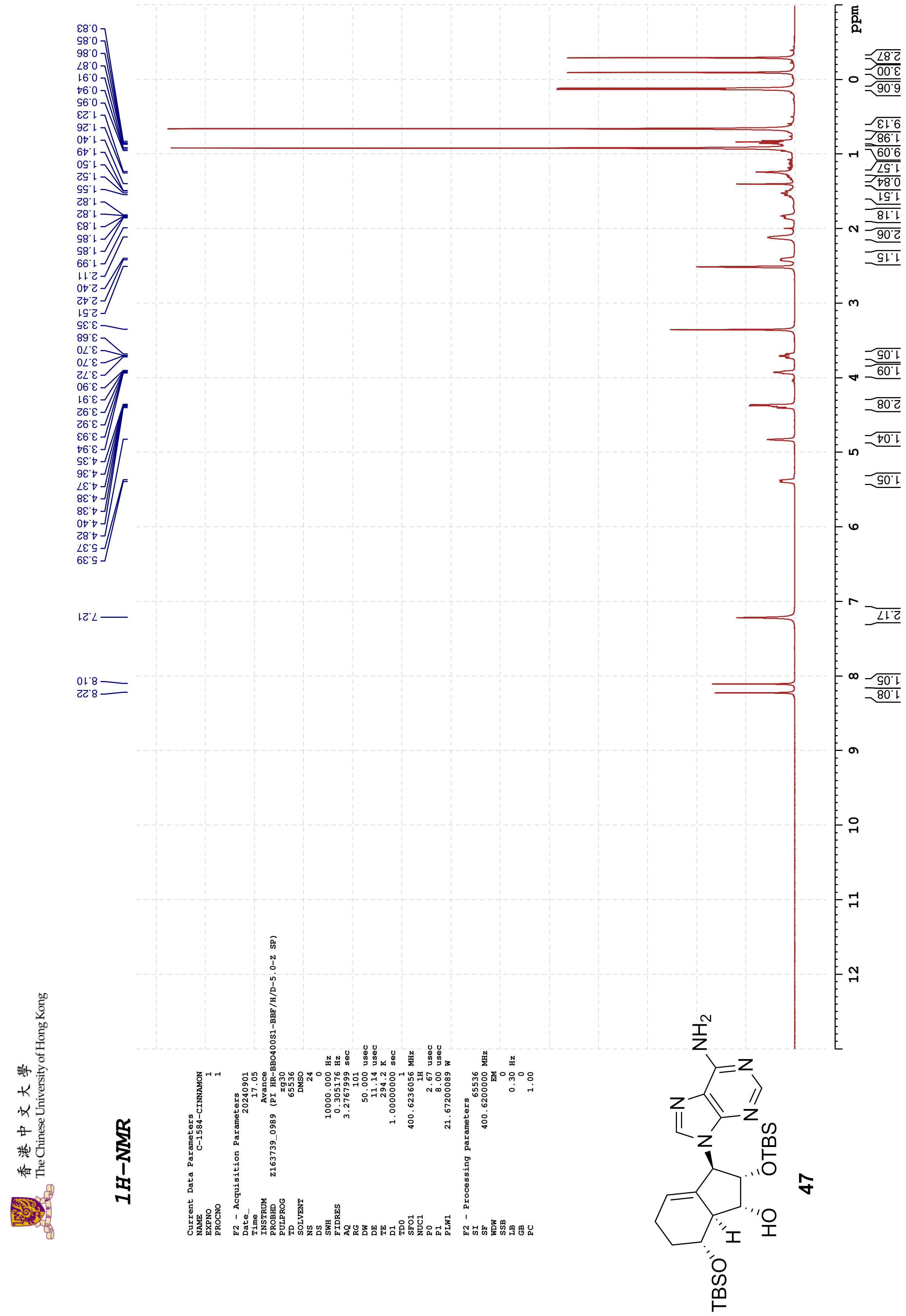

NMR-Spectra for Compound 47

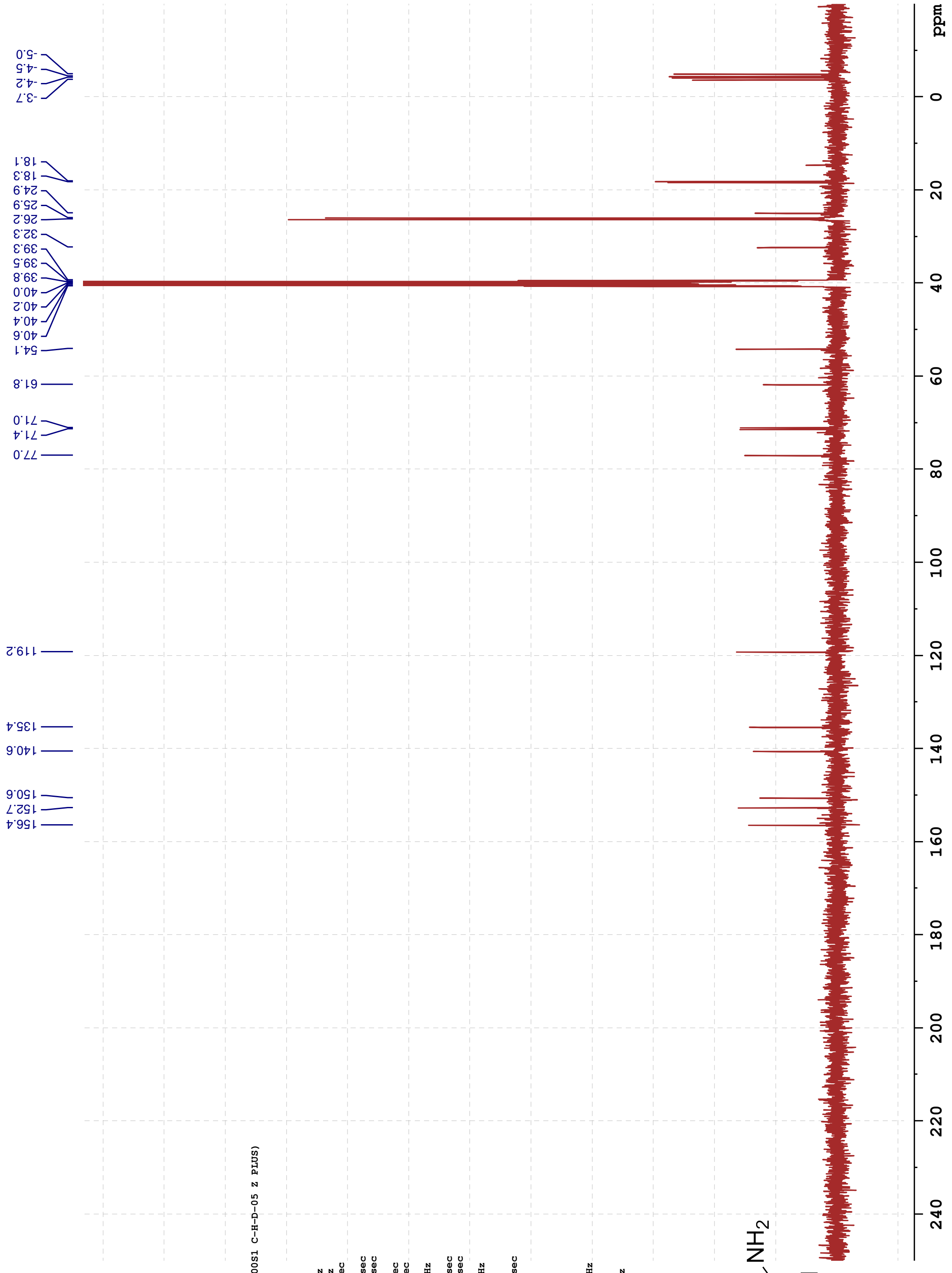

香港中文大學 The Chinese University of Hong Kong

13C{1H}-NMR

Current Data Parameters
NAME B-24835-CINNAMONCMR
EXPNO 1
PROCNO 1

F2 - Acquisition Parameters
Date_ 20241022
Time 4.28
INSTRUM AvanceNeo 400
PROBHD Z108621_0062 (PA DUL 400S1 C-H-D-05 Z PLUS)
PULPROG zgpg30
TD 65536
SOLVENT DMSO
NS 24
DS 0
SWH 32679.738 Hz
FIDRES 0.997306 Hz
AQ 1.0027008 sec
RG 5
DW 15.300 usec
DE 6.50 usec
TE 295.0 K
D1 2.00000000 sec
D11 0.03000000 sec
TD0 1
SFO1 100.6862041 MHz
NUC1 13C
P0 3.33 usec
P1 10.00 usec
PLW1 46.61299896 W
SFO2 400.3716015 MHz
NUC2 1H
CPDPRG[2] waltz65
PCPD2 90.00 usec
PLW2 21.85799980 W
PLW12 0.26984999 W
PLW13 0.13573000 W

F2 - Processing parameters
SI 32768
SF 100.6731166 MHz
WDW EM
SSB 0
LB 2.00 Hz
GB 0
PC 1.40

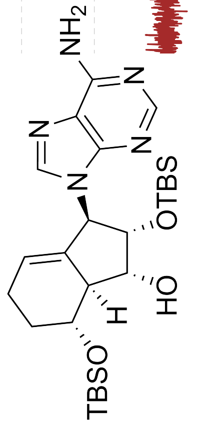

47

NMR-Spectra for Compound 47

香港中文大學 The Chinese University of Hong Kong

13C{1H}-NMR APT

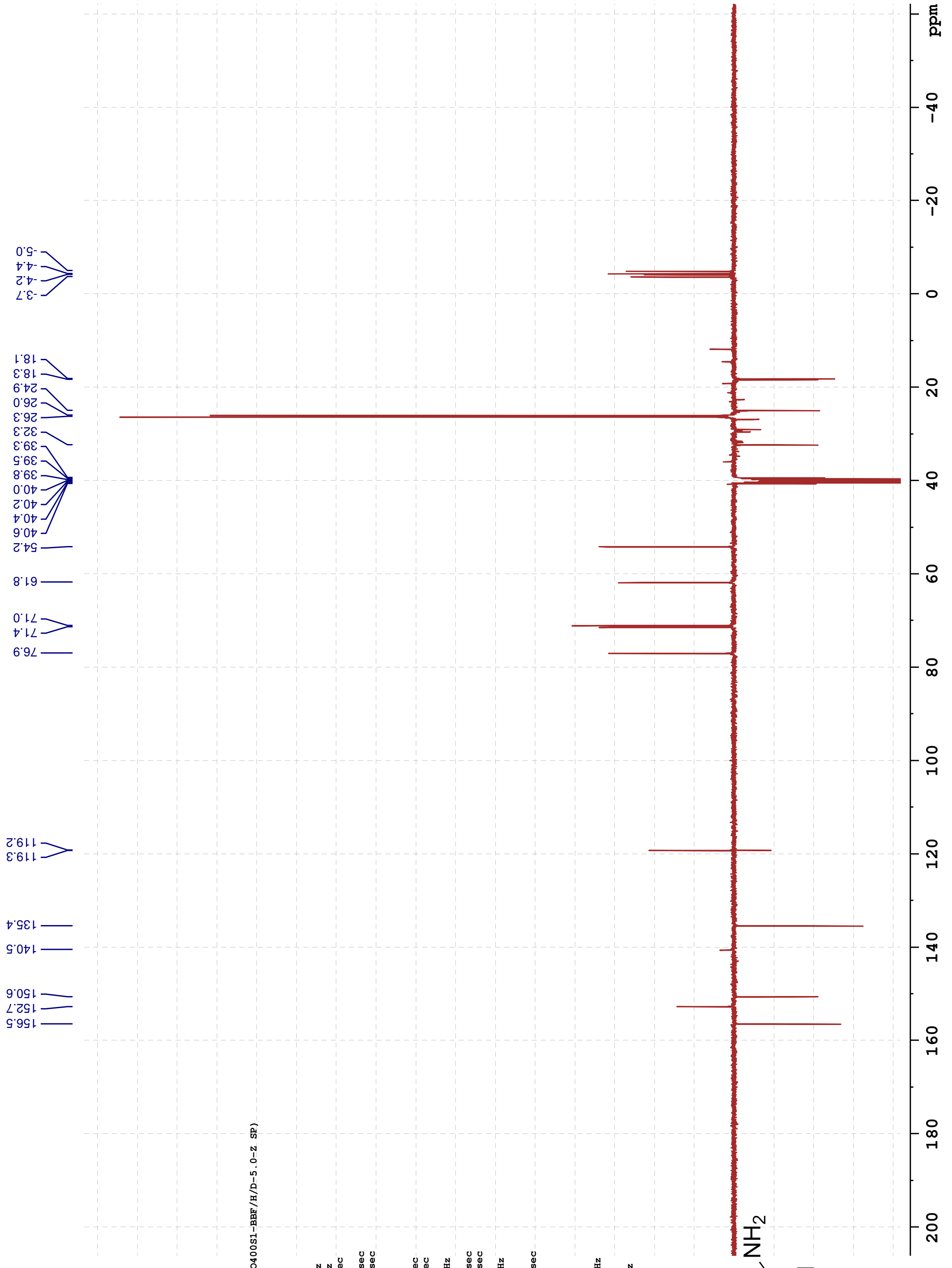


```
Current Data Parameters
NAME          C-1584-CINNAMONapt
EXPNO                    1
PROCNO                   1

F2 - Acquisition Parameters
Date_             20240901
Time                 19.14
INSTRUM             Avance
PROBHD      Z163739_0989 (PI HR-BBO400S1-BBF/H/D-5.0-Z SP)
PULPROG               jmod
TD                   65536
SOLVENT               DMSO
NS                    2500
DS                       0
SWH              32679.739 Hz
FIDRES            0.997306 Hz
AQ               1.0027008 sec
RG                    20.2
DW                  15.300 usec
DE                    6.50 usec
TE                   295.5 K
CNST2          145.0000000
CNST11           1.0000000
D1              2.00000000 sec
D20             0.00689655 sec
TD0                      1
SFO1           100.7460527 MHz
NUC1                   13C
P1                    8.00 usec
P2                   16.00 usec
PLW1           94.87300110 W
SFO2           400.6216025 MHz
NUC2                    1H
CPDPRG2            waltz65
PCPD2                90.00 usec
PLW2           21.67200089 W
PLW12           0.17124000 W

F2 - Processing parameters
SI                   32768
SF             100.7359791 MHz
WDW                     EM
SSB                      0
LB                    1.00 Hz
GB                       0
PC                    1.40
```

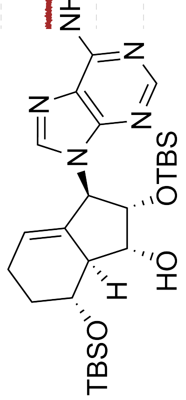

47

NMR-Spectra for Compound 51

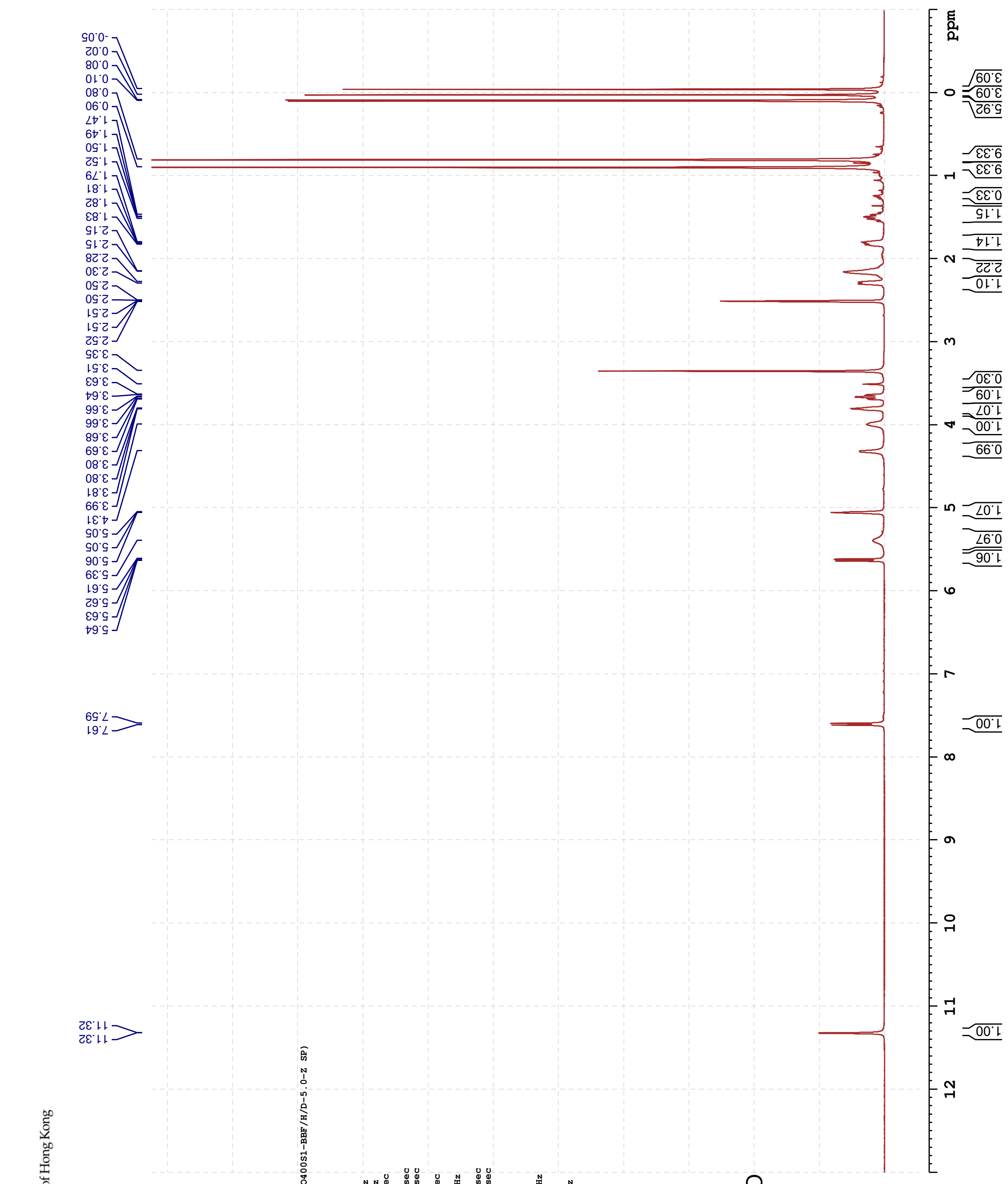

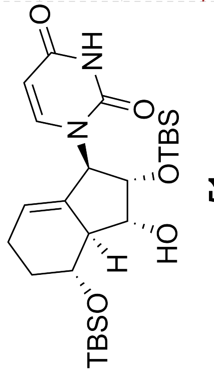

51

NMR-Spectra for Compound 51

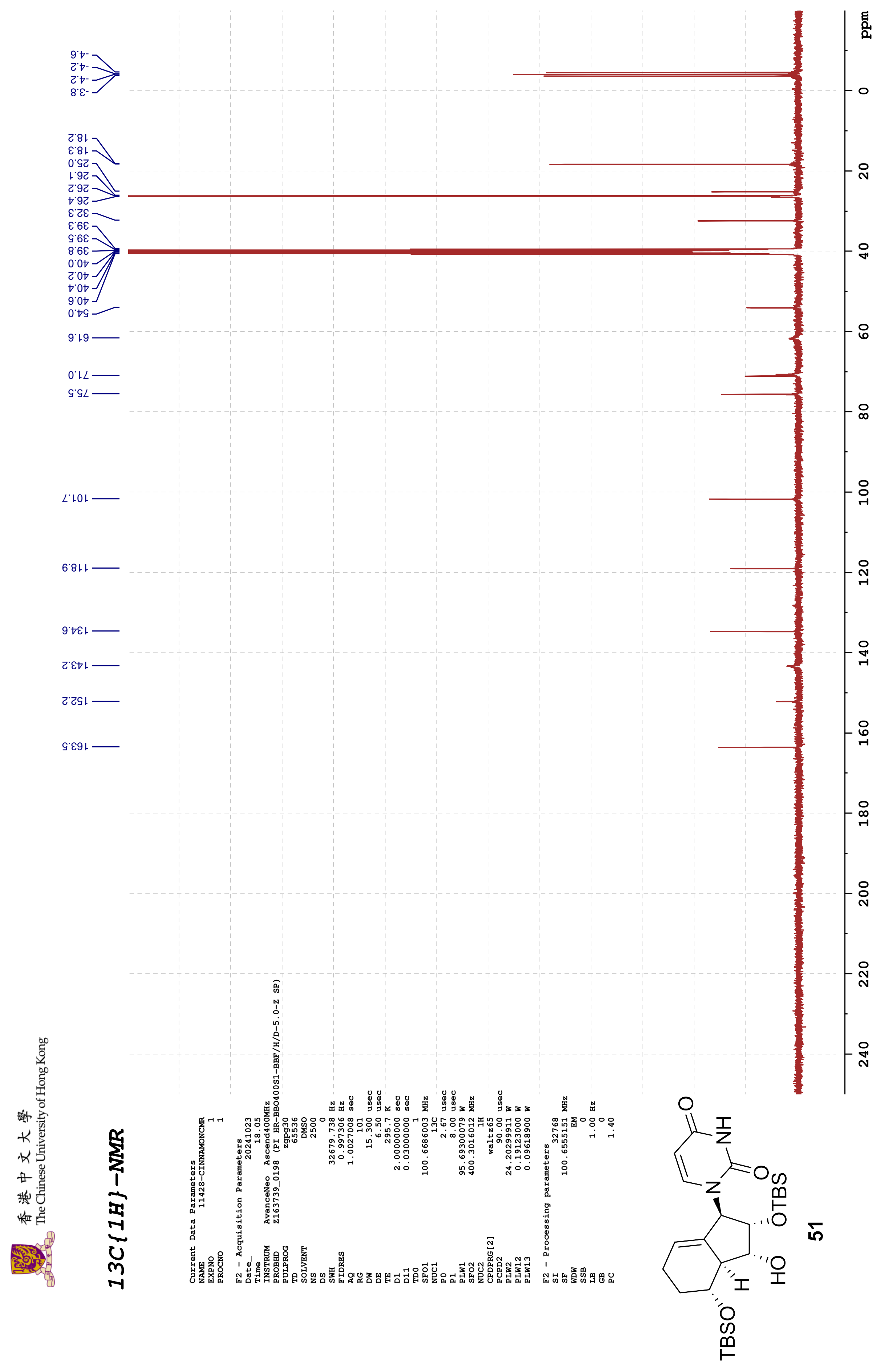

NMR-Spectra for Compound 52

香港中文大學 The Chinese University of Hong Kong

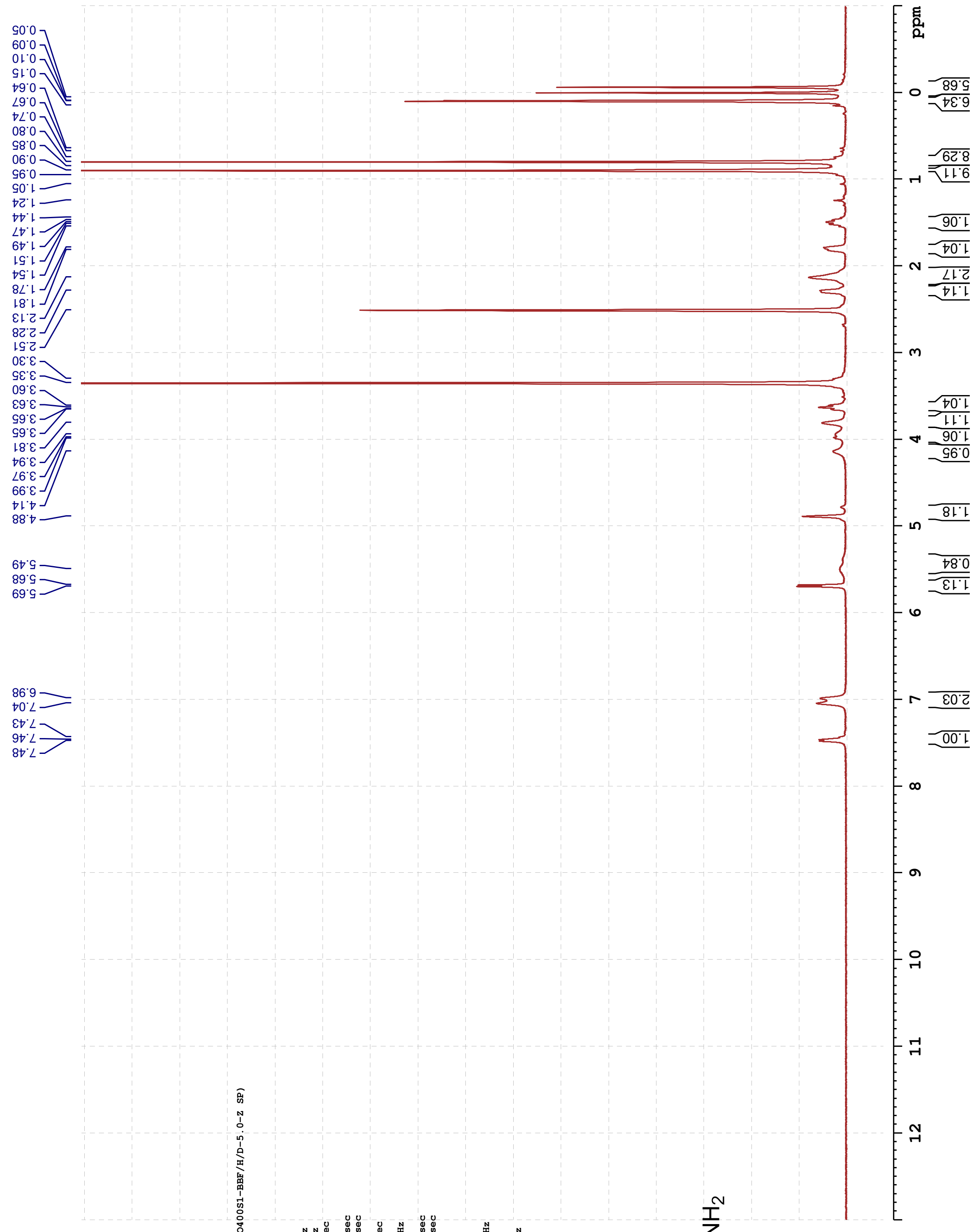

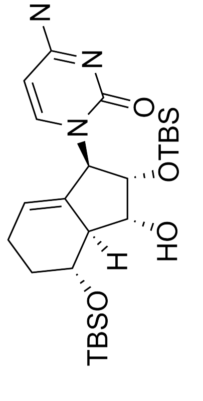

52

NMR-Spectra for Compound 64

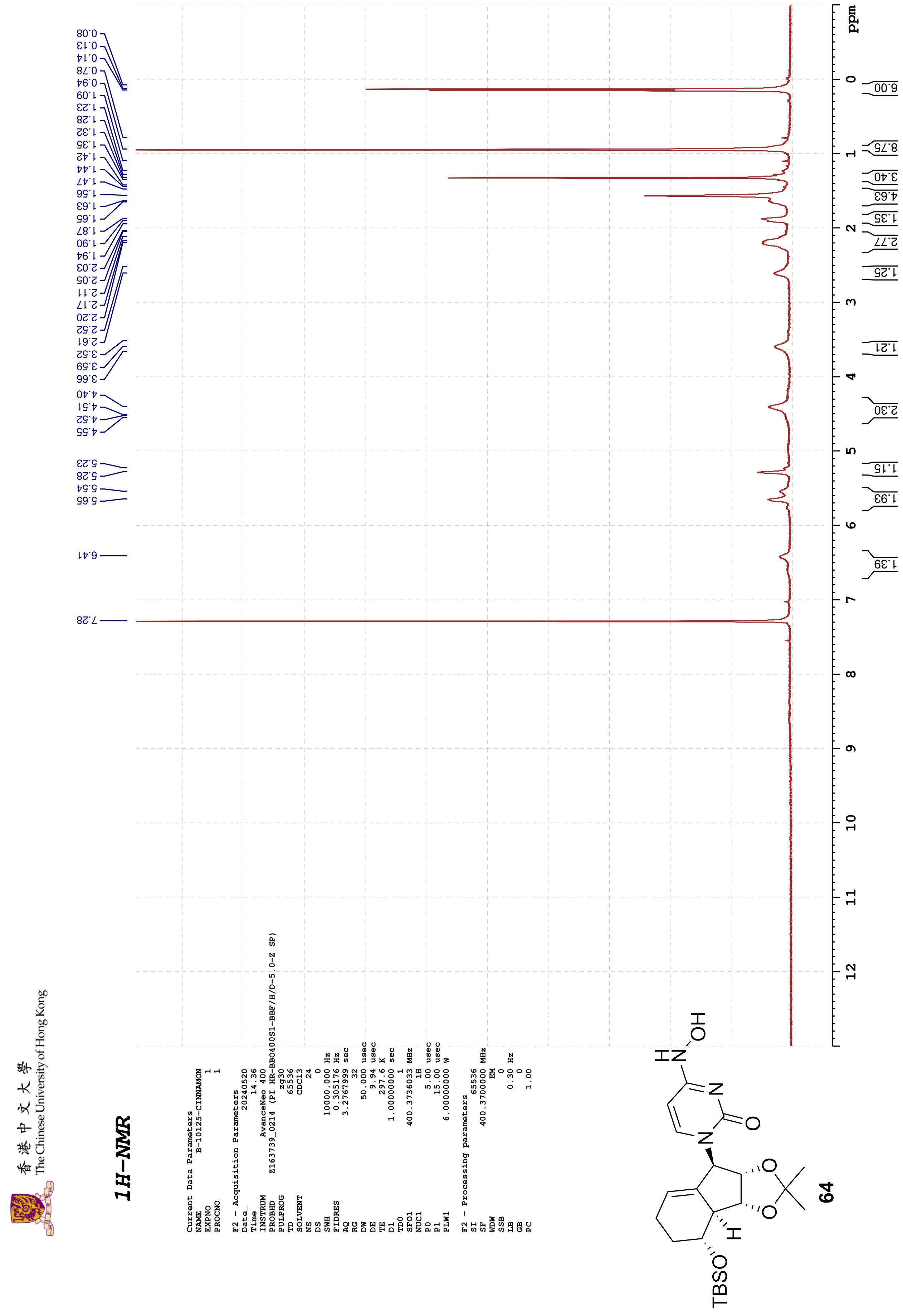

NMR-Spectra for Compound 66

The Chinese University of Hong Kong 香港中文大學

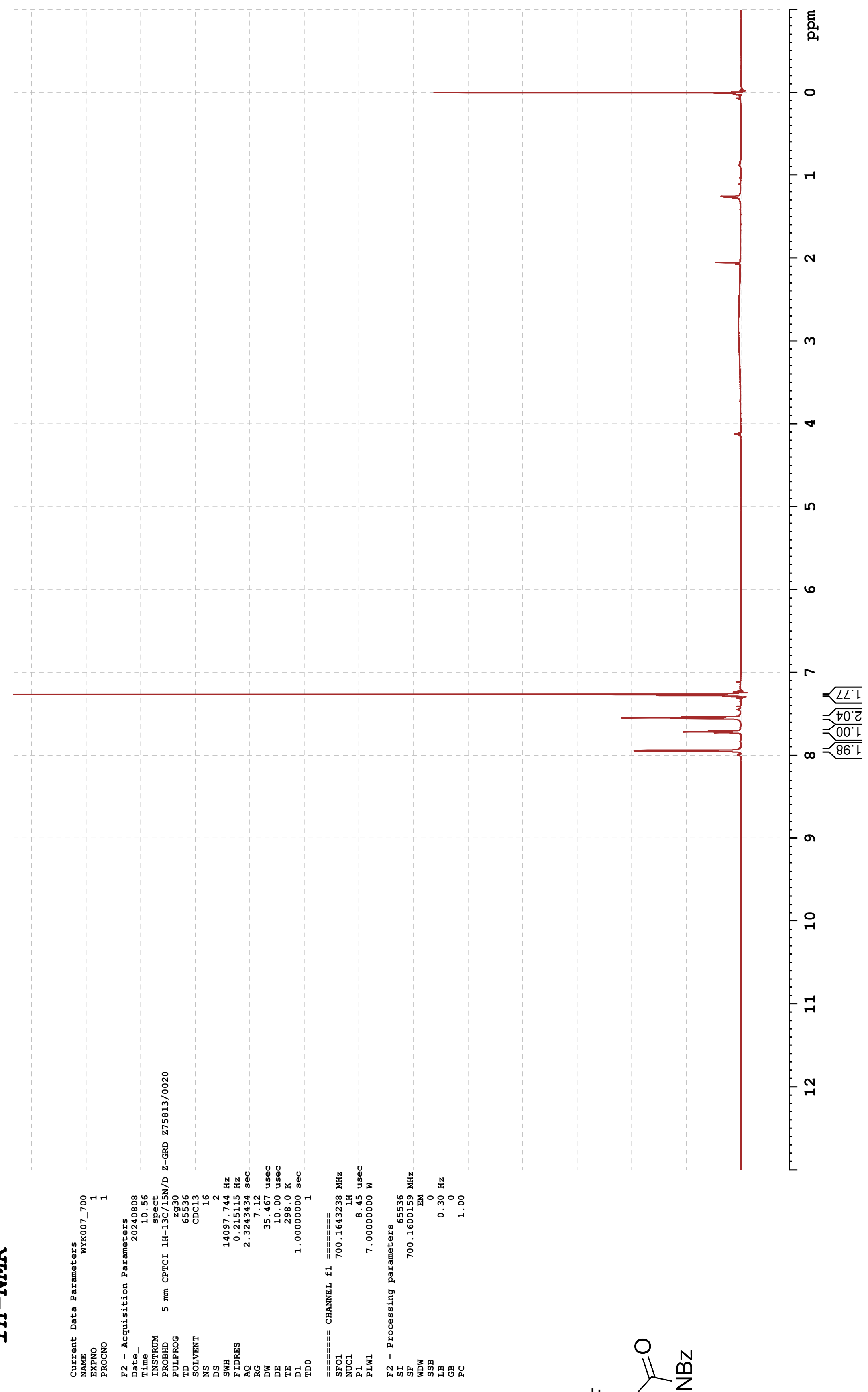

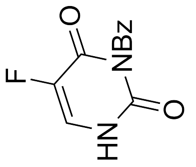

66

NMR-Spectra for Compound 66

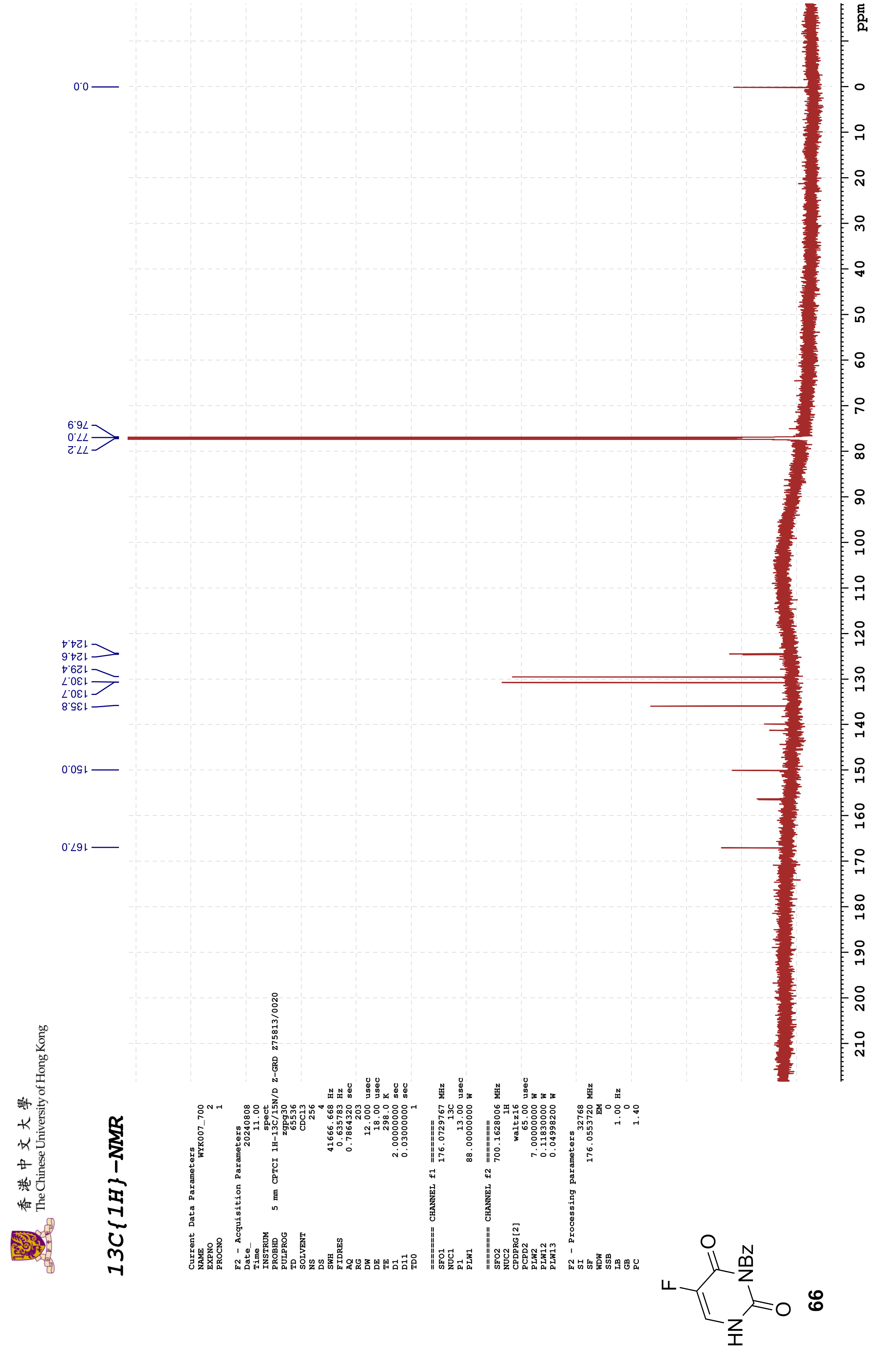

66

NMR-Spectra for Compound 72

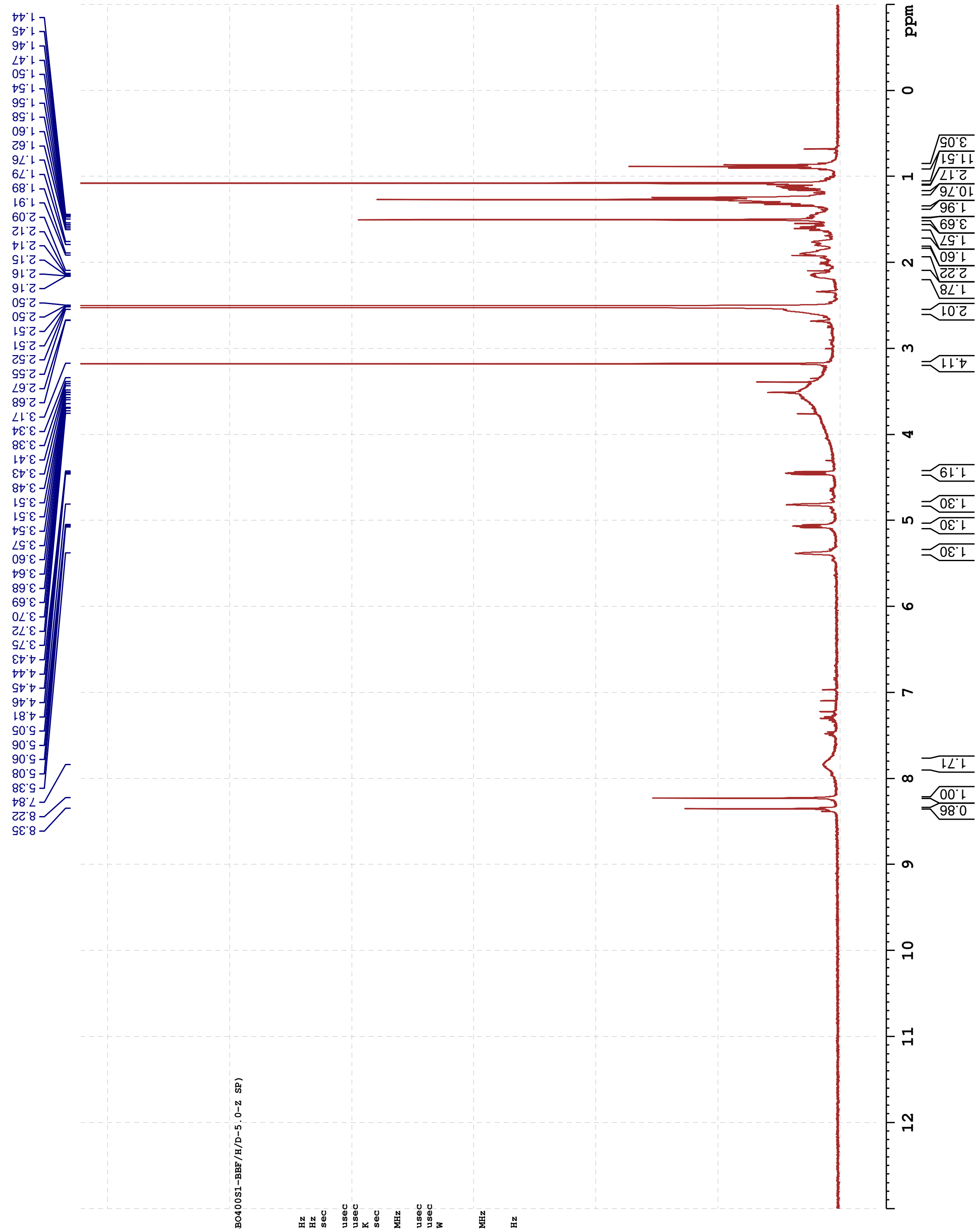

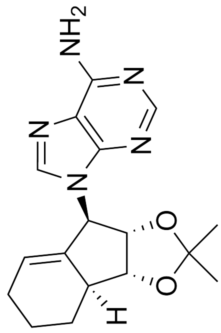

72

NMR-Spectra for Compound 73

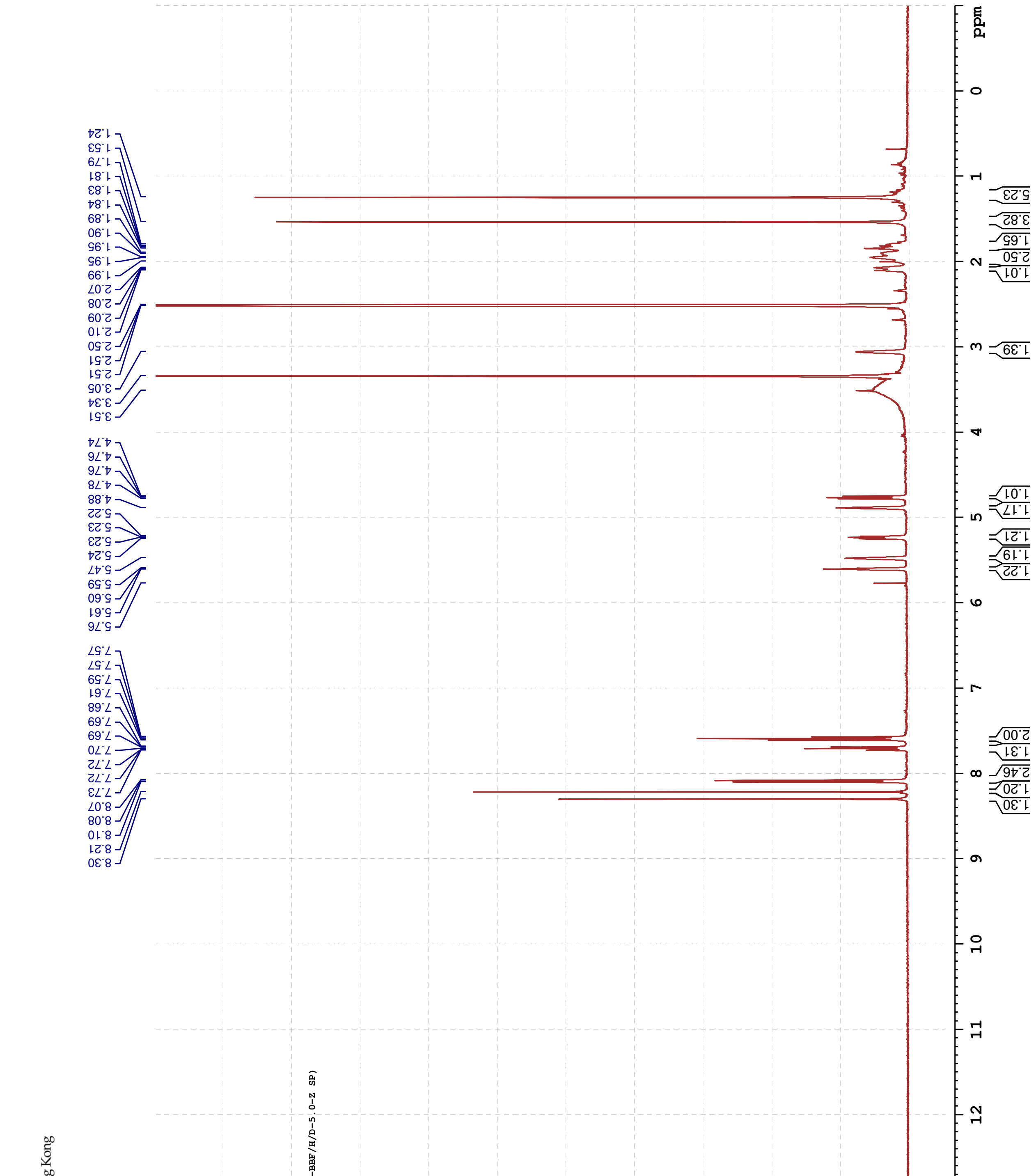

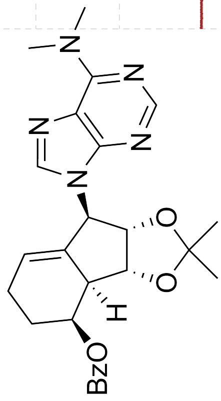

73

NMR-Spectra for Compound 73

The Chinese University of Hong Kong

13C{1H}-NMR

```
Current Data Parameters
NAME         C-9888-CINNAMON_cmr
EXPNO                         1
PROCNO                        1

F2 - Acquisition Parameters
Date_                  20241107
Time                      10.04
INSTRUM                  Avance
PROBHD      Z163739_1043 (PI HR-BBO400S1-BBF/H/D-5.0-Z SP)
PULPROG                 zgpg30
TD                        65536
SOLVENT                    DMSO
NS                         3000
DS                            4
SWH                   32679.739 Hz
FIDRES                 0.997306 Hz
AQ                    1.0027008 sec
RG                         12.7
DW                       15.300 usec
DE                         6.50 usec
TE                        296.4 K
D1                   2.00000000 sec
D11                  0.03000000 sec
TD0                           1
SFO1                100.7490748 MHz
NUC1                        13C
P0                         2.67 usec
P1                         8.00 usec
PLW1                96.23899841 W
SFO2                400.6216025 MHz
NUC2                         1H
CPDPRG2                 waltz65
PCPD2                     90.00 usec
PLW2                21.54100037 W
PLW12                0.17020001 W
PLW13                0.08560800 W

F2 - Processing parameters
SI                        32768
SF                  100.7359791 MHz
WDW                          EM
SSB                           0
LB                         1.00 Hz
GB                            0
PC                         1.40
```

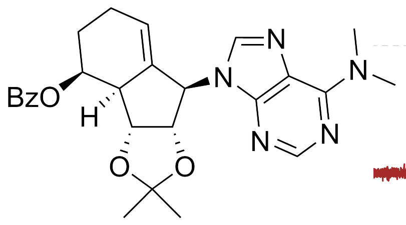

73

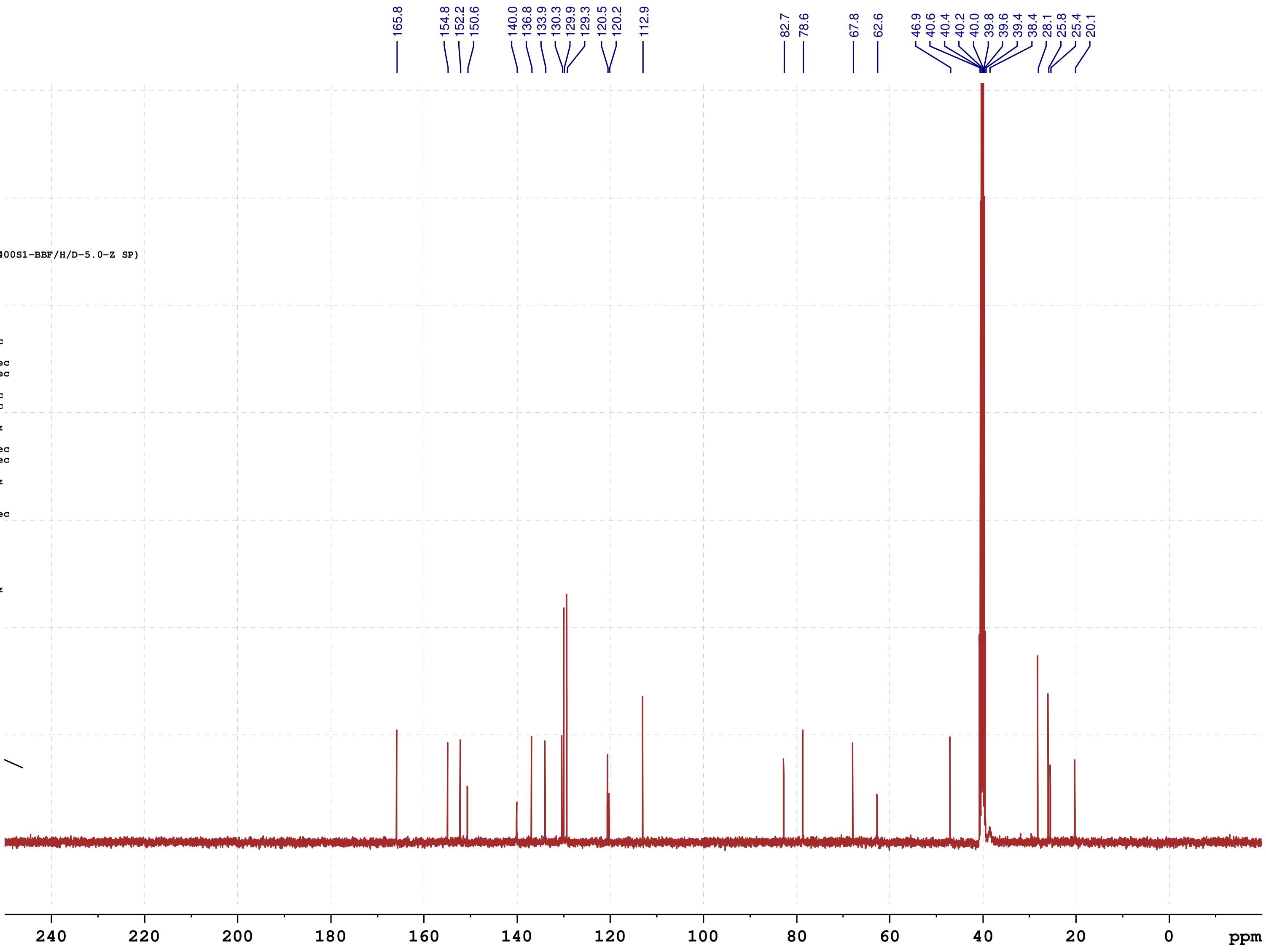

NMR-Spectra for Compound 75

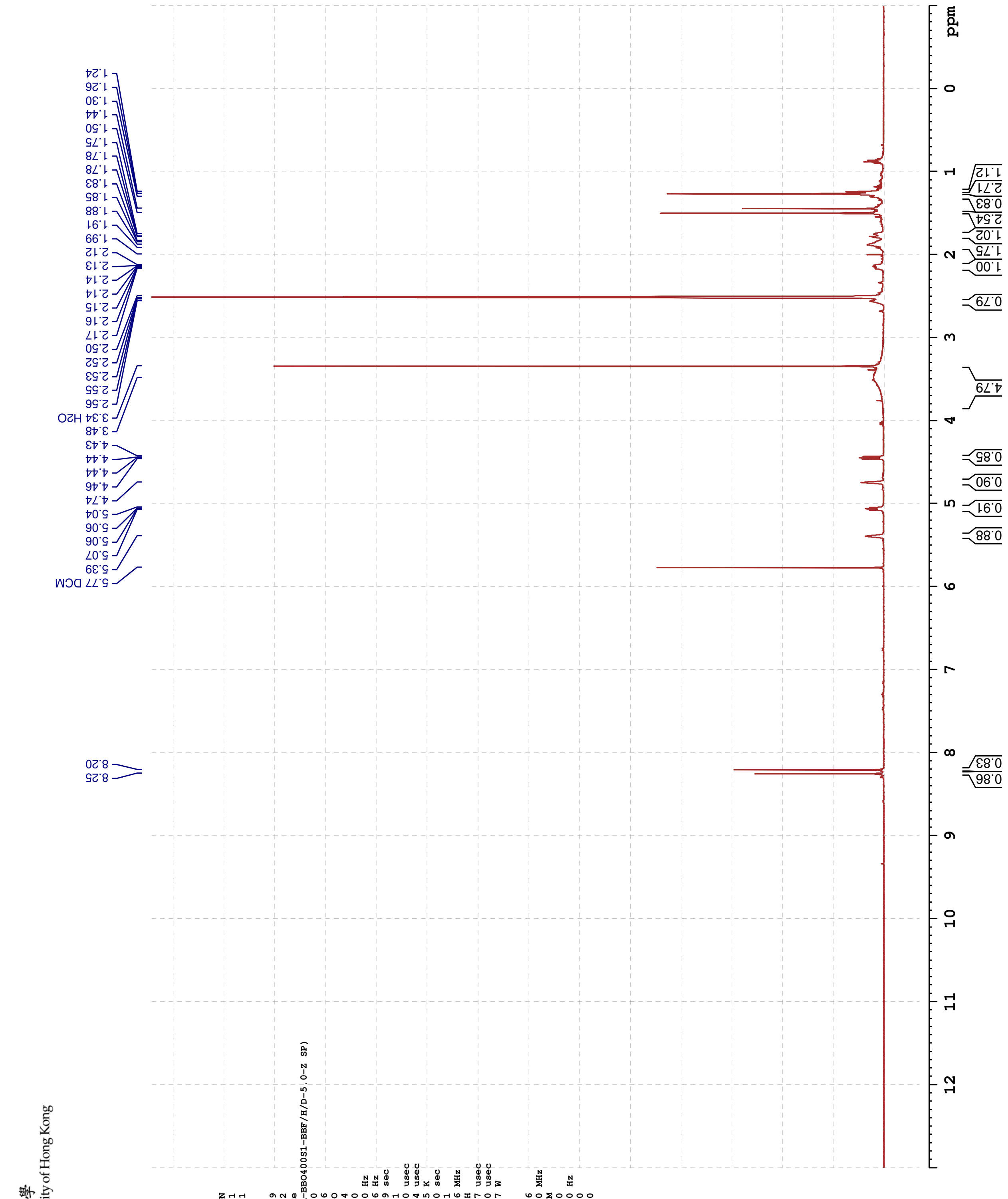

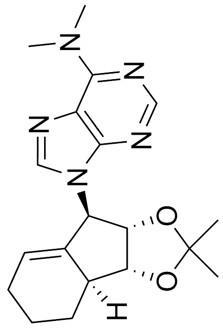

75

NMR-Spectra for Compound 76

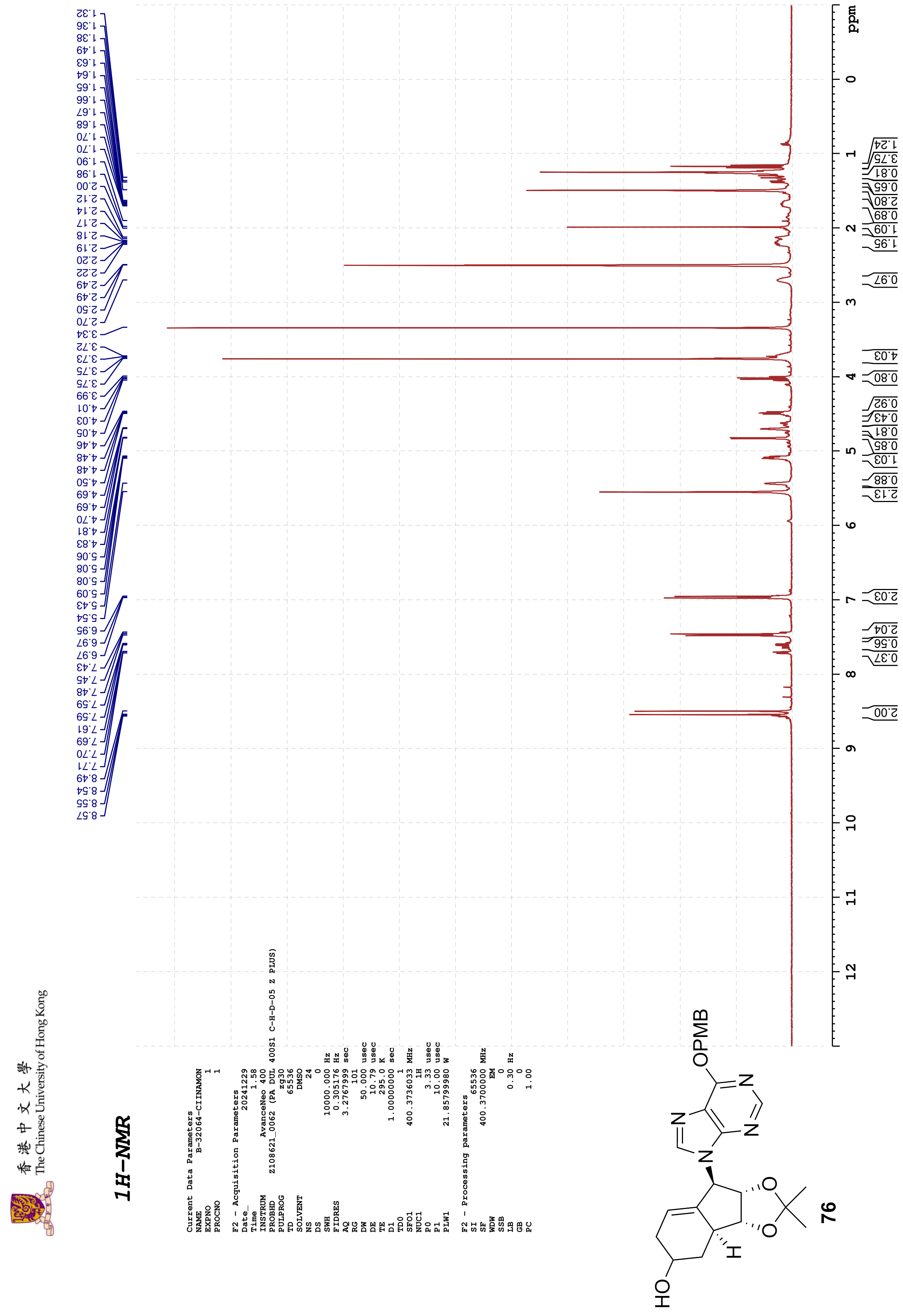

NMR-Spectra for Compound 79

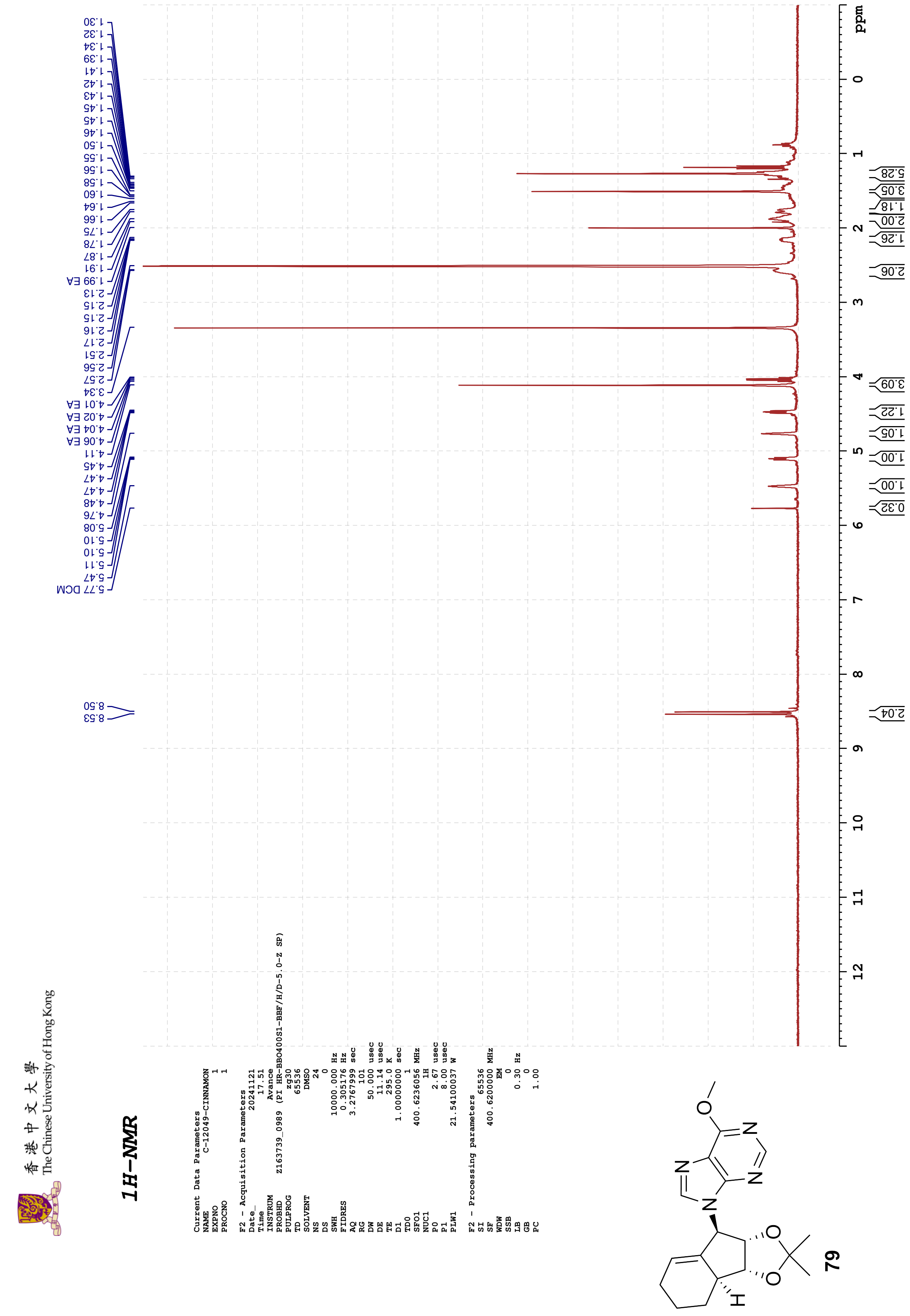

NMR-Spectra for Compound 80

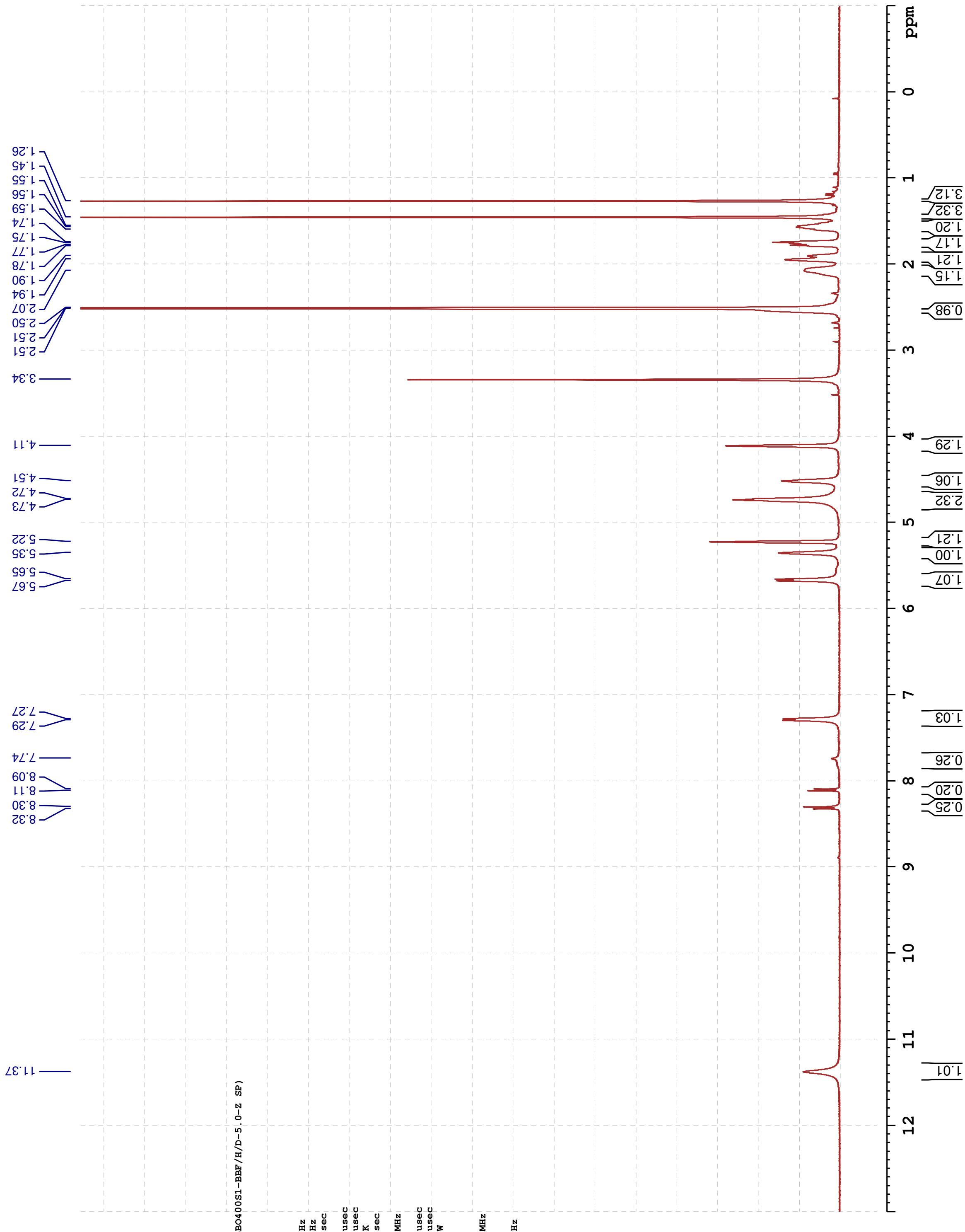

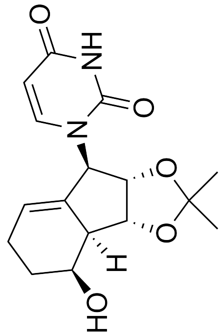

80

NMR-Spectra for Compound 86

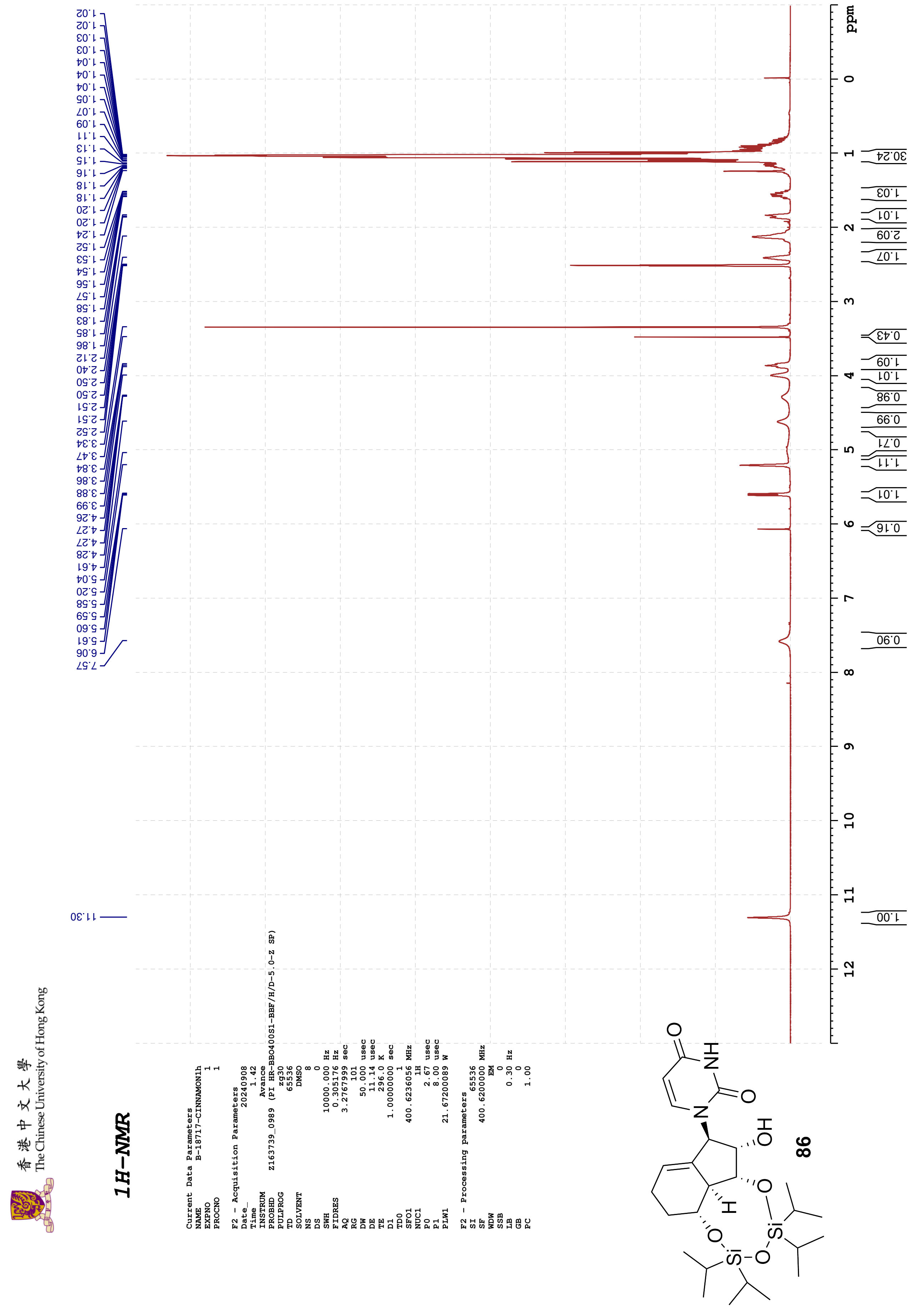

NMR-Spectra for Compound 86

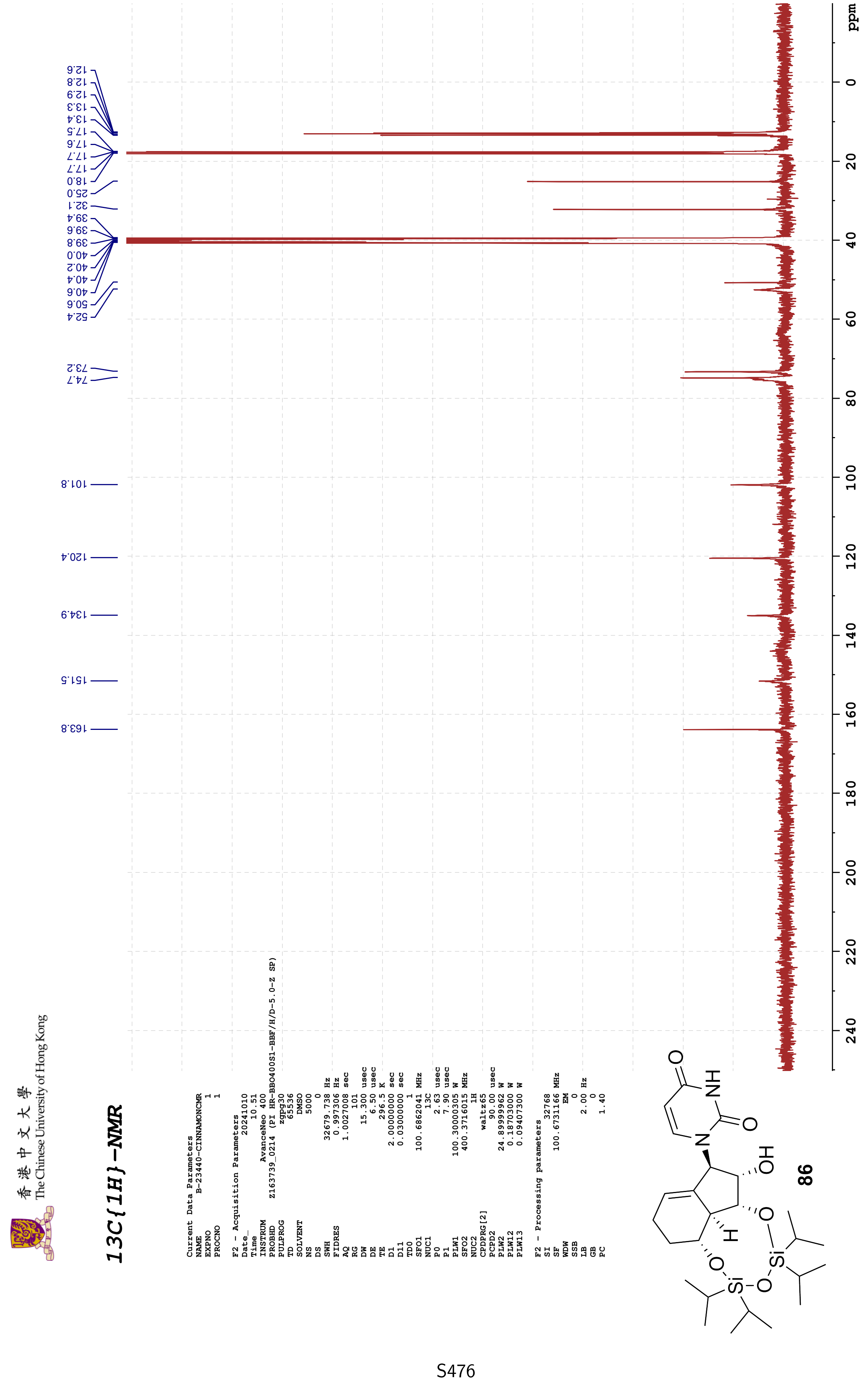

Supplement: Supplementary file 1 [file jm5c02584_si_001.pdf]
